# Mapping the Crystallographic Landscape of Antivitamin Ionic Liquids: Structural Blueprints for Novel Architectures

**DOI:** 10.1021/acs.cgd.5c00378

**Published:** 2025-05-23

**Authors:** Clare McNeill, Marija Scheuren, Joseph Cooper, Sophia Bellia, Muhammadiqboli Musozoda, Janayah N. Tolbert, Matthias Zeller, Arsalan Mirjafari, Patrick C. Hillesheim

**Affiliations:** † Department of Chemistry and Physics, 53713 Ave Maria University, Ave Maria, Florida 34142, United States; ‡ Department of Chemistry, 14828 State University of New York at Oswego, Oswego, New York 13126, United States; § Department of Chemistry, Purdue University, West Lafayette, Indiana 47907, United States; ∥ Department of Chemistry, Illinois State University, Normal, Illinois 61761, United States

## Abstract

This work presents the first in-depth crystallographic study of antivitamin-derived ionic liquids. Seven new amprolium salts incorporating hallmark ionic-liquid anions such as bis­(trifluoromethanesulfonyl)­imide (NTf_2_^–^), bis­(pentafluoroethanesulfonyl)­imide (BETI^–^), tetrafluoroborate (BF_4_^–^), and hexafluorophosphate (PF_6_^–^) were synthesized and crystallized, and their structures and interactions were elucidated through crystallographic and computational analyses. The well-documented biological functions of amprolium can help simplify future applications of these compounds as well as open the pathway for the development of novel cations for ionic liquid development. Despite their dicationic nature and bearing multiple H-bonding donors and acceptors, these compounds exhibited unexpectedly low melting points and displayed challenging crystallization conditions. The analysis identified key structural features explaining this behavior: (i) two points of conformational disorder in the pyrimidine ring and propyl moiety; (ii) three distinct cation conformations affecting aromatic components; and (iii) novel high-energy conformations of anions, reported here for the first time. Hydrogen interactions dominated intermolecular forces (85% of total interactions), with H-bonding to oxygen and fluorine being most prevalent. These insights advance our understanding of how to engineer functional materials from natural sources for potential applications in sustainability and medicine. The combined experimental-computational approach validates these design principles, providing a foundation for more targeted development of similar compounds with tailored properties.

## Graphical abstract

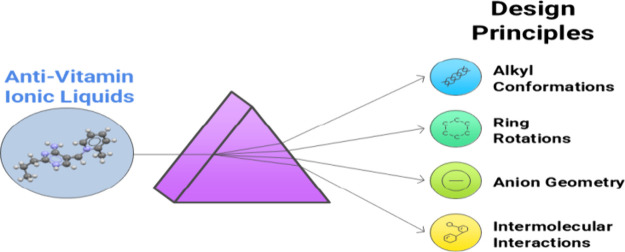

## 1 Introduction

Ionic liquids (ILs) represent a multifunctional class of organic materials that have attracted significant attention due to their unique physicochemical properties–e.g., negligible vapor pressure, powerful solvation capabilities, and tunable characteristics^1^–with applications extending across diverse sectors to address major societal challenges in energy^2,3^ and healthcare.^4,5^ However, many contemporary IL designs merely iterate on concepts established in the 1990s,^6^ primarily focusing on imidazolium, phosphonium, and quaternary ammonium cations. This persistent lack of fundamental innovation suggests the field has reached a pivotal juncture in developing novel molecular architectures, directly limiting our capacity to overcome persistent challenges such as high viscosity, prohibitive costs, and inadequate biocompatibility, or to enable transformative new functions. To advance the field, researchers must incorporate innovative design criteria into IL development, specifically through bioinspired principles, multifunctional integration, and precise structure–property relationships established via X-ray crystallography, decisively moving beyond the conventional cations that have dominated the literature for more than two decades

Nature consistently serves as inspiration for the design of novel molecules and materials–a principle that extends to the field of ILs. For example, amino acids have been used in the development of IL systems, typically as the anions.^7^ These amine-bearing anions can then be applied for various tasks such as direct air capture of CO_2_.^8^ One particular benefit of ILs based on these amino acid derivatives is that they eschew the more environmentally problematic fluorinated anionic systems (e.g., BF_4_^–^ or PF_6_^–^) which dominate the current literature.^9^ Amino acid–based ILs would be more biocompatible and biodegradable than perfluorinated anionic systems, potentially addressing concerns about environmental impact once an IL has reached the end of its lifecycle.^10^

Some of the less studied naturally occurring, bioinspired ILs are those of vitamin B1 (VB1) or thiamine. Containing a benzylated thiazolium ring, VB1 follows the general structure of the prototypical bis-alkylated heterocyclic ILs.^11^ Given its importance for biological systems, VB1 has been studied extensively throughout the years, with a crystal structure being reported back in 1933 by Bernal and Crowfoot.^12^ The structure of VB1 was further examined, revealing several binding pockets linked to distinct geometries of the thiazolium and pyridinium rings.^13,14^ Being a natural catalyst, the reactivity of VB1 was leveraged as a coupling agent,^15^ showcasing the applications’ potential of this naturally occurring IL. However, despite the prominence of this naturally occurring organic salt, few published studies exist wherein the fundamental structural and thermal characteristics of this compound when paired with the expected anions of the IL field, e.g., NTf_2_^–^, PF_6_^–^, BF_4_^–^, OTf^–^.^16,17^

Amprolium hydrochloride (**Amp-Cl**) belongs to a class of compounds known as antivitamins.^18^ As the name suggests, antivitamins act as antagonists to vitamins, interfering with their biological functions. A recent study by Ruetz et al. reported the development and crystallographic analysis of a vitamin B12 antivitamin, providing valuable insights into compound stability and opening new pathways for potential medical applications.^19^ **Amp-Cl** specifically acts as a vitamin B1 antivitamin, being structurally similar to the thiazolium cation of VB1 (Figure 1). Within biological systems, **Amp-Cl** inhibits thiamine dependent enzymes while limiting thiamine uptake, allowing it to be used in the treatment of parasites in animals.^20^ This cationic nature of **Amp-Cl** can be leveraged to create a diverse library of compounds through systematic variation of the associated anions, resulting in distinct physicochemical properties guided by established principles of ionic liquid chemistry.

**Figure 1:**
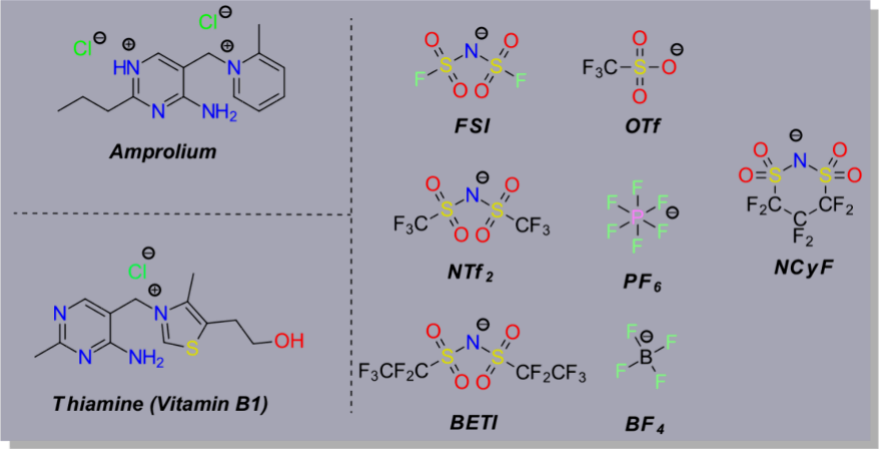
Chemical structures of thiamine, amprolium, and the counteranions used in this study. The amprolium-based ionic liquids were synthesized by pairing the amprolium cation with the seven different anions.

Crystallinity has not been adequately leveraged as a favorable property in IL research. Single crystal X-ray crystallography (SCXRD) provides unprecedented insights into IL structures at atomic resolution in the solid state, offering critical information that computational studies cannot deliver. In fact, X-ray diffraction reveals essential details about the noncovalent interactions (NCIs) such as cation–anion interactions, H-bonding networks, and packing arrangements, enabling direct correlations between molecular architecture and macroscopic properties such as melting points even for new structures with no known analogs.^21^ The structural insights derived from X-ray analyses have proven instrumental in the rational design of new ILs with tailored properties. By systematically relating crystallographic data to functional characteristics, we can implement empirical strategies for cation design rather than relying on theoretical approaches. This transition toward property-driven development represents a paradigm shift in IL research, positioning SCXRD as an indispensable tool for advancing the field.

Building on our group’s longstanding interest in designing novel, bioconscious ILs,^22^ we synthesized seven ILs containing the amprolium cation through anion metathesis reactions.^23^ Although a long-term objective is to replace perfluorinated anions with biocompatible alternatives, a rigorous structural benchmark is first required. Perfluorinated anions are retained within this study because of their importance in IL research in addition to having an established set of thermophysical data, providing a consistent reference frame for evaluating the new cation architecture. Insights gained from analysis of these recognized anions will guide subsequent studies with ‘greener’ anions (e.g., amino acids), allowing structure–will allow property trends to be transferred rather than inferred.

Rigorous analysis of the crystal structures of these compounds using SCXRD is supplemented with computational approaches, allowing deep insight into both the crystalline nature of these compounds as well as their molecular structures. These data provide a rationale behind the distinct crystallinity of the compounds beyond simple cursory explanations. Further, despite the commercial importance of amprolium chloride, this manuscript is the first report involving this cation in the formation of ILs. Moreover, there is a lack of structural data on the amprolium cation with only one^24^ currently deposited structure in the Cambridge Structural Database (CSD).^25^

## 2 Materials and Methods

### 2.1 Synthesis

A 0.50 g (1.59 mmol) sample of amprolium·HCl was dissolved in a minimal amount of water (ca. 3 mL) and stirred in a sealed glass container. To this mixture was added 0.55 g (1.91 mmol) sample of lithium bis­(trifluoromethanesulfonyl)­imide dissolved in a minimal amount of water (ca. 1 mL). Upon addition of the lithium sample, an immediate white precipitant formed. The mixture was stirred for an additional 2 h, filtered, and washed with DI water. The cleaned powder was dried under vacuum at 55 °C overnight prior to analysis.

The remaining samples were prepared using the same procedures. NMR characterization and crystallization procedures are provided in the SI.

### 2.2 Single Crystal Diffraction

Crystallization details are provided in the Supporting Information. Single crystals were coated with Parabar 10312 or Fomblin oil and transferred to the goniometer head of either a Bruker D8 Quest Eco diffractometer or Bruker Quest diffractometer with either Mo Kα wavelength (λ = 0.71073 Å) or Cu Kα wavelength (λ = 1.54178 Å) and a Photon II area detector with kappa geometry, a I-μ-S microsource X-ray tube, laterally graded multilayer (Goebel) mirror for monochromatization. For all compounds, data were collected, reflections were indexed and processed, and the files scaled and corrected for absorption using APEX3 and/or APEX4,^26^ SAINT and SADABS.^27^

For all compounds, the space groups were assigned using XPREP within the SHELXTL suite of programs,^28,29^ the structures were solved by direct or dual methods using ShelXS or ShelXT^30^ and refined by full matrix least-squares against *F* ^2^ with all reflections using Shelxl2018 ^31^ using the graphical interfaces Shelxle^32^ and/or Olex2.^33^ Unless otherwise specified, H atoms were positioned geometrically and constrained to ride on their parent atoms. C–H bond distances were constrained to 0.95 Å for aromatic and alkene C–H moieties, and to 0.99 and 0.98 Å for aliphatic CH_2_ and CH_3_ moieties, respectively. Methyl H atoms were allowed to rotate, but not to tip, to best fit the experimental electron density. *U*_iso_(H) values were set to a multiple of *U*_eq_(C) with 1.5 for CH_3_ and 1.2 for C–H and CH_2_ units, respectively.

Complete crystallographic data, in CIF format, have been deposited with the Cambridge Crystallographic Data Centre. CCDC 2433015–2433021 contains the supplementary crystallographic data for this paper. These data can be obtained free of charge via the CCDC’s and FIZ Karlsruhe’s joint Access Service at https://www.ccdc.cam.ac.uk/structures/

### 2.3 Computational Methods

Hirshfeld surfaces and fingerprint plots were calculated and produced using CrystalExplorer21.^34^ Images and analysis of the structures were accomplished using Mercury.^35^

The molecular structure of the dication from Amp-BF_4_ was loaded into Spartan’24 (Wave function, 2024) and the structure was optimized using ωB97X-D fuctional^36^ with a 6–311+G** basis set. A search for the lowest energy conformations was done using Spartan’24. The 10 lowest energy conformations were optimized using the same functional and basis set.

### 2.4 Thermal Characterization

Compounds were dried overnight in a vacuum oven at 55 °C prior to any analysis.

Melting points and decomposition temperatures were measured on a PerkinElmer 6000 simultaneous thermal analyzer (STA). A sample purge of nitrogen was used in all studies with a flow rate of 40 mL/min. Samples were heated at a constant rate of 5 °C/min from 35 to 650 °C. After the samples reached 650 °C, the heating rate was increased to 50 °C/min and the sample heated to 1000 °C. The purge gas was changed to air and held at 1000 °C for 10 min. This final step is simply to clean the pans and remove any carbonaceous residue and is not used for any analysis but is visible in the complete data that are presented in the Supporting Information. Platinum pans were used for all studies.

Thermal decomposition data are shown in the Supporting Information. Onset temperatures (*T*_onset_) are reported for the first decomposition step. The derivative thermogravimetric curves (DTG) were obtained from the experimental STA data. Decomposition temperatures (*T*_dec._) were obtained using the maximum thermal decomposition rate for the first major peak of the DTG curve.

### 2.5 Torsion and Plane Angles for the Cations

To attempt to standardize the analysis of the torsion angles of the two aromatic rings, we followed the steps set forth in previous studies involving torsion angles and conformations of alkyl chains in ILs.^37^ For the measurement we oriented the molecule so that we were looking down the C—N bond between the benzyl carbon and the pyridine nitrogen. The methyl group on the pyridine ring was oriented at the top (12 o’clock) position as the 0° starting point. Positive torsion angles were arbitrarily assigned to clockwise rotations until 180°. Negative torsion angles were assigned for counterclockwise rotation. Figure 2 is provided to help visualization. For the propyl chains, torsion angles are listed as absolute values. The Supporting Information contains all measured angles. Plane angles were calculated as the angle between the planes formed by the two rings using Mercury.

**Figure 2:**
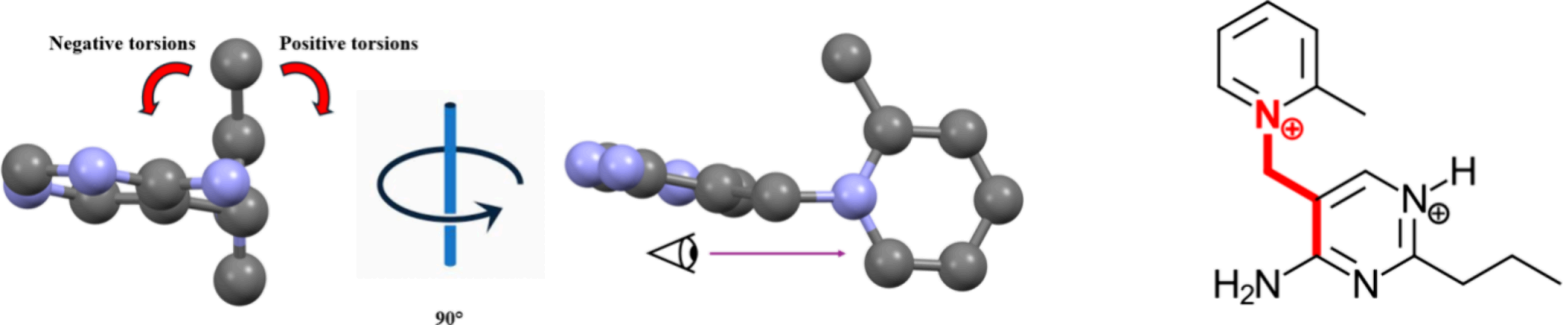
Schematic of how torsion angles for the pyridine and pyrimidinium rings were measured. Hydrogen atoms and the propyl chain are removed for clarity. The four atoms defining the torsion angle are highlighted in red.

## 3 Discussion

### 3.1 Crystal Structure Discussion

Seven amprolium-based IL salts were synthesized and crystallized. The molecular structures taken from the asymmetric units of the crystals are shown in Figure 3 (only the most prevalent moieties are shown where disorder is present). As discussed in the introduction, only one previously reported amprolium crystal structure exists in the Cambridge Crystallographic Database, that of the chloride derivative. As such, this structure was also examined within this manuscript to provide as complete a picture as possible. The original report on the **Amp-Cl** structure contains the relevant geometric and crystallographic data and a detailed discussion on the structure.^24^

**Figure 3:**
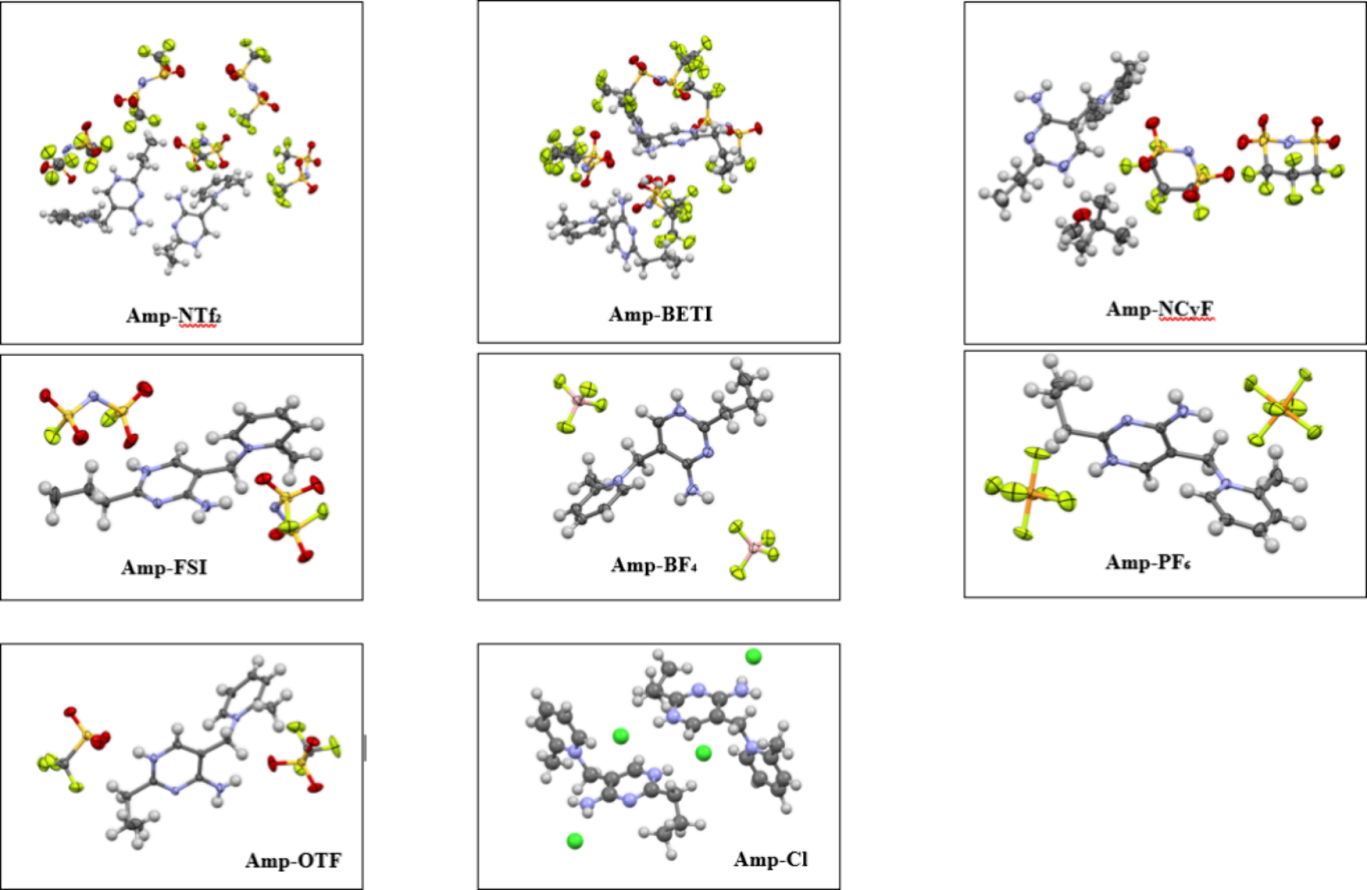
Asymmetric units of the amprolium crystals shown with 50% probability ellipsoids. Disorder is omitted for clarity. The chloride structure was previously reported^24^ and is shown to help with discussion.

Several general considerations and observations should be addressed with respect to the crystal structures. First, except for **Amp-Cl**, all compounds exhibit some degree of disorder in either the cations or the anions, complicating the analysis to some extent, particularly when focusing on interatomic distances. Where possible, disorder was retained during the calculation of Hirshfeld surfaces. Second, the compounds **Amp-NTf**_**2**_, **Amp-BETI**, and **Amp-Cl** contain two dications in the asymmetric unit, which, for simplicity, are referred to as 1 and 2 (e.g., **Amp-NTf**_**2**_ **-1**).

Third, **Amp-BETI** and **Amp-NCyF** contain solvent in the asymmetric unit. In the case of **Amp-NCyF** an ether moiety is found hydrogen bonding to the protonated nitrogen atom of the pyridinium ring. Our previous work on related VB1-based compounds has indicated that the NCyF anion appears to favor the formation of solvate species, though the exact reason is unknown.^17^ The current results with **Amp-NCyF** are consistent with our previous observations. Finally, regarding the crystallographic characteristics, three compounds crystallize in the P $\overset{-}{1}$ space group (**Amp-NTf**_**2**_ **, Amp-FSI, Amp-Cl**), four in a monoclinic space group (**Amp-BETI**, **Amp-NCyF**, **Amp-BF**_**4**_ **, Amp-OTF**), and one in an orthorhombic space group (**Amp-PF**_**6**_).

### 3.2 Cation Structure

#### 3.2.1 Cation Torsion and Plane Angles

Understanding the structure and conformations of the amprolium cations is important not only for the development of future pyridinium-based ILs, but also with respect to rationalizing the formation of noncovalent interactions. Several key details on cation structure should be addressed.

There are three conformations of the cation rings (pyridinium and pyrimidinium) observed within the crystals. These conformations, distinguished by torsion or plane angles of the aromatic moieties, mirror the possible *F*, *S*, or *V* conformations found within thiamine.^38^ Broadly the conformations correspond to a rotation about the N—C bond connecting the pyridine and benzyl carbon. These rotations are readily observed when examining the amine moiety on the pyrimidinium moiety, particularly when looking at the disordered cation in **Amp-OTF** or **Amp-NCyF** (Figure 4).

**Figure 4:**
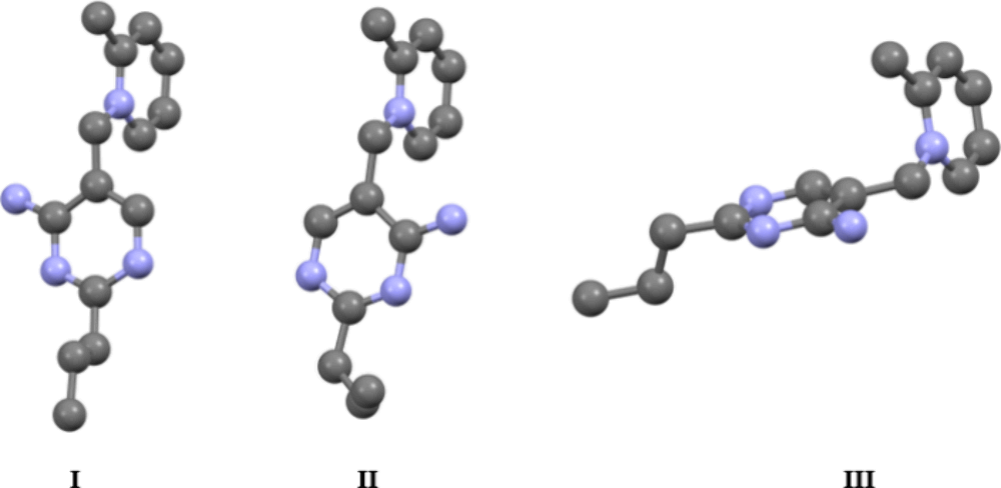
Depiction of the three distinct conformations of the amprolium cation observed in the crystals. The conformations are distinguished by the torsion angle of the bond joining the pyridine and pyrimidine rings. Conformations I and II are the most common and are observed as rotational disorder within several structures (e.g., **Amp-OTF**). Hydrogen atoms omitted for clarity.

Several key details emerge from the analysis of the torsion angles and plane angles of the pyridinium and pyrimidinium rings in the cation (see Table S1). First, the plane angles do not show much variance, with a range of 19° across all the cations measured (angles of 70–89°). Second, while there are three conformations involving rotation of the rings, two of the conformations are mirrors, showing torsion angles ranges of ca. 80–90°. Finally, the conformation with the torsion angles of 170–180° appear less favored when compared with the those of torsion angles of ±80–90°. This observation contrasts with the theoretical studies wherein the lowest energy conformer was found with a torsion angle of −171° (see Section 3.4). The presence of strong hydrogen bonding, which features prominently within these crystals, along with other noncovalent interactions are likely responsible for favoring specific conformations within the crystal structure.

#### 3.2.2 Cation Alkyl Chains

One structural change between amprolium and VB1 is the substitution of a methyl group on the pyrimidine ring in VB1 with a propyl group in amprolium. For the present study, this modification adds additional complexity to the cation structure, impacting crystallinity. The propyl group can adopt multiple conformations, leading to disorder within the crystals. These energetically accessible propyl conformations contribute, in part, to the difficulty of crystallizing these compounds, similar to bis-alkylated ILs.^39^

The torsion angles of the propyl chains were measured from the structures. Generally, there appear to be three conformations which are observed. First, there is a staggered conformation with torsion angles near 180°. Two gauche conformations exist, ranging, approximately, between 50–70° and another from 80 to 90°. This follows the observations from the computational studies wherein the lowest energy set of conformers display torsion angles that are gauche and staggard.

### 3.3 Anion Structures

#### 3.3.1 NCyF Anion Structure

Our initial report of ILs bearing NCyF anions involved the VB1 cation,^16^ resulting in compounds that displayed high thermal stabilities and crystallinity.^40^ A follow up study revealed that structures containing the NCyF^–^ anion displayed a unique propensity for the formation of solvates in the crystalline state, a phenomenon not observed in the other compounds in the series.^17^ The study of the NCyF anion was expanded upon by the Welton group, providing valuable structural and electronic insight into the behavior of this unique anion.^41^ Despite the unique benefits of this anion with respect to ILs, it still remains underutilized within the world of ILs, perhaps due to the significant cost when compared to other perfluorinated anions. Moreover, only 22 crystals containing the anion have been reported in the Cambridge Structural Database (CSD), making structural data rather sparse, particularly when contrasted to NTf_2_^–^ bearing crystals.

Notably, within our previously reported structure (database code: ILOCUK) a twist-boat conformation of the anion was observed as part of a disordered anion moiety. This preliminary observation points toward the conformational flexibility of these anions, an essential component in the formation of low melting ILs. Surprisingly, compound **Amp-NCyF** displays a boat conformation of the ring, which was calculated to be significantly higher in energy than the ground state chair conformation (Figure 5).^41^ As mentioned, the Welton group has reported a comprehensive set of studies on these perfluorinated cyclical anions, demonstrating that they undergo ring inversions analogously to cyclohexane derivatives, albeit with different energies. The reported structures in the database predominately show the chair conformations in agreement with the reported theoretical studies which show the chair conformation as the lowest energy conformation. Thus, **Amp-NCyF** is the first reported compound with a higher energy boat conformation.

**Figure 5:**
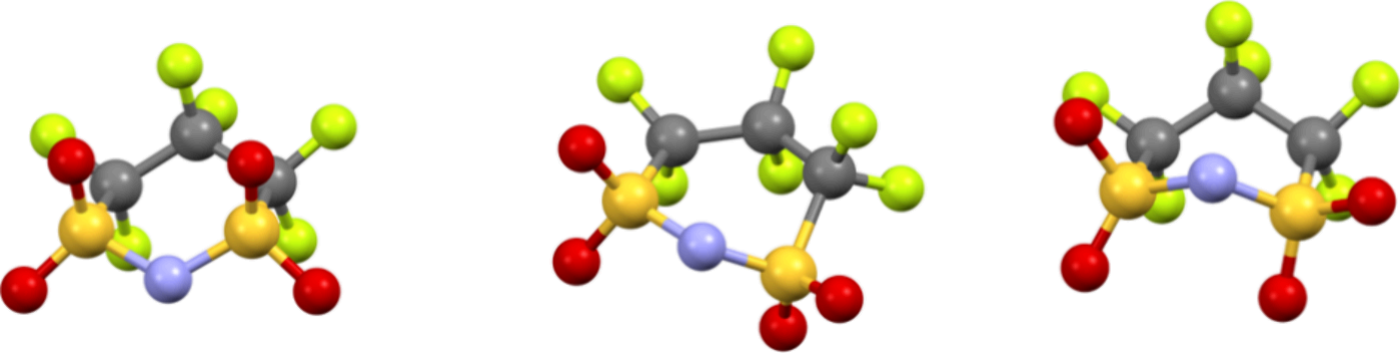
Depictions of the chair, skew-boat, and boat conformations of the NCyF^–^ anion. The chair and boat conformations are observed in **Amp-NCyF** while the skew-boat (middle) was previously reported in a VB1-based compound.^16^

#### 3.3.2 BETI Anion Structure

As with the case of the cyclical perfluorinated anions, the Welton group provided an extensive study on the conformations of the NTf_2_^–^ anion and several related derivatives.^42^ Within their work they noted three distinct transitional conformations of the NTf_2_^–^ anion which they label as TS1, TS2, TS3. These transitional conformations are intermediate geometries which exist along the conformational pathway as the SO_2_CF_3_ moiety rotates between the *cis* and *trans* conformations. Within **Amp-BETI** there appear to exist several of these intermediate transitional conformations, distinct from any of the previously reported structures (Figure 6). An in-depth computational study would certainly be warranted to better understand the impact of not only BETI but also more complex perfluoro anions with longer alkyl chains such as the bis­(nonafluorobutylsulfonyl)­imide anion^43^ or tris­(perfluoroalkyl)­trifluorophosphate anion.^44^

**Figure 6:**
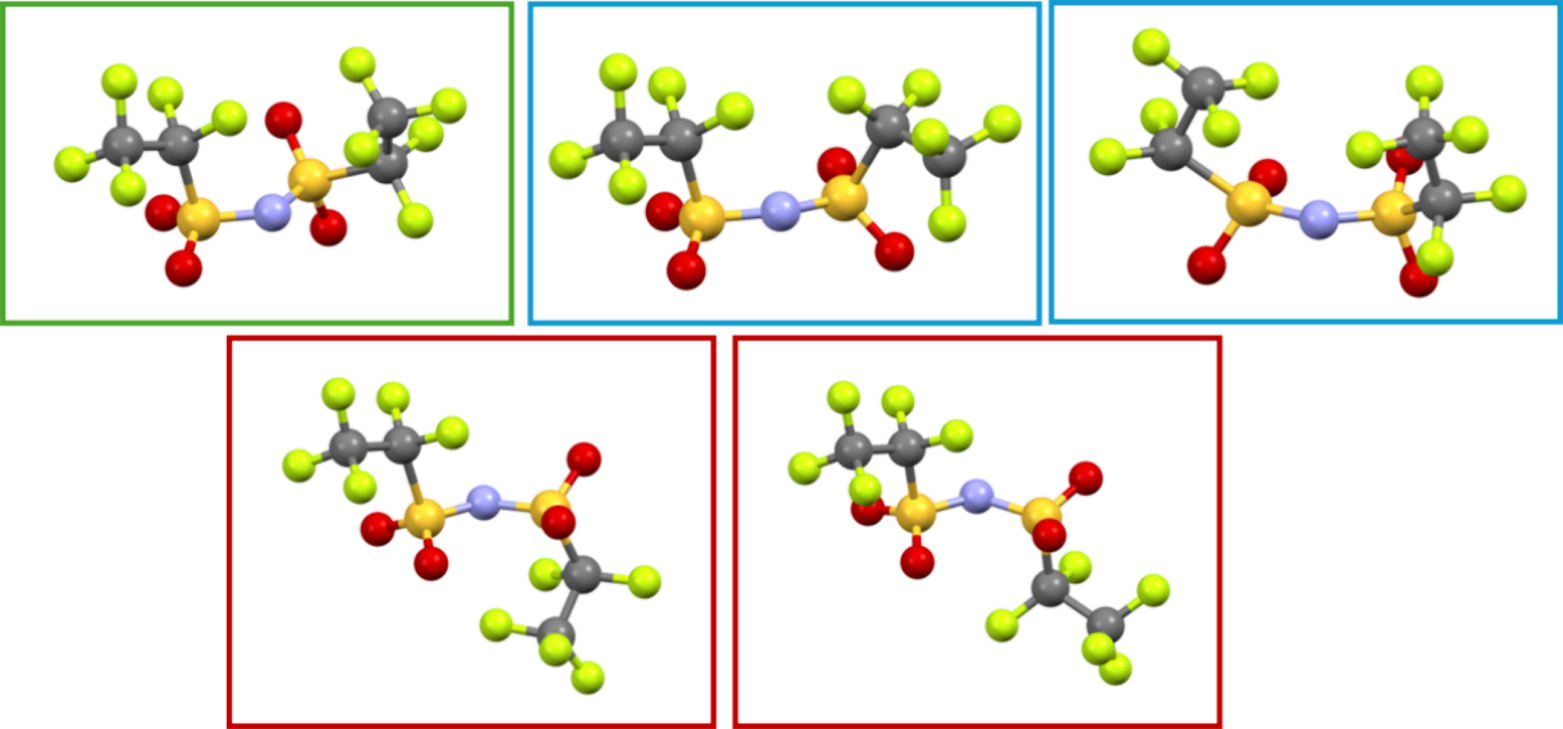
Representation of the multiple conformations of the BETI anion found in **Amp-BETI**. Multiple *cis* (blue) and *trans* (red) conformations are found, differing in the torsion angles of the fluorinated ethyl chains. A transitional geometry is also observed (green).

### 3.4 Theoretical Studies

To better understand the observations of the multiple conformations in the solid state, a search was conducted to identify any energetically accessible conformations of the cation. In total, nine structures were found all of which are within 5 kJ/mol of energy from the lowest energy conformer (Figure 7). We chose a 5 kJ/mol cutoff due to previous studies on interactions and synthons within crystalline systems based on approximate room temperature thermal energy, the temperature at which the crystals were grown.^45^

**Figure 7:**
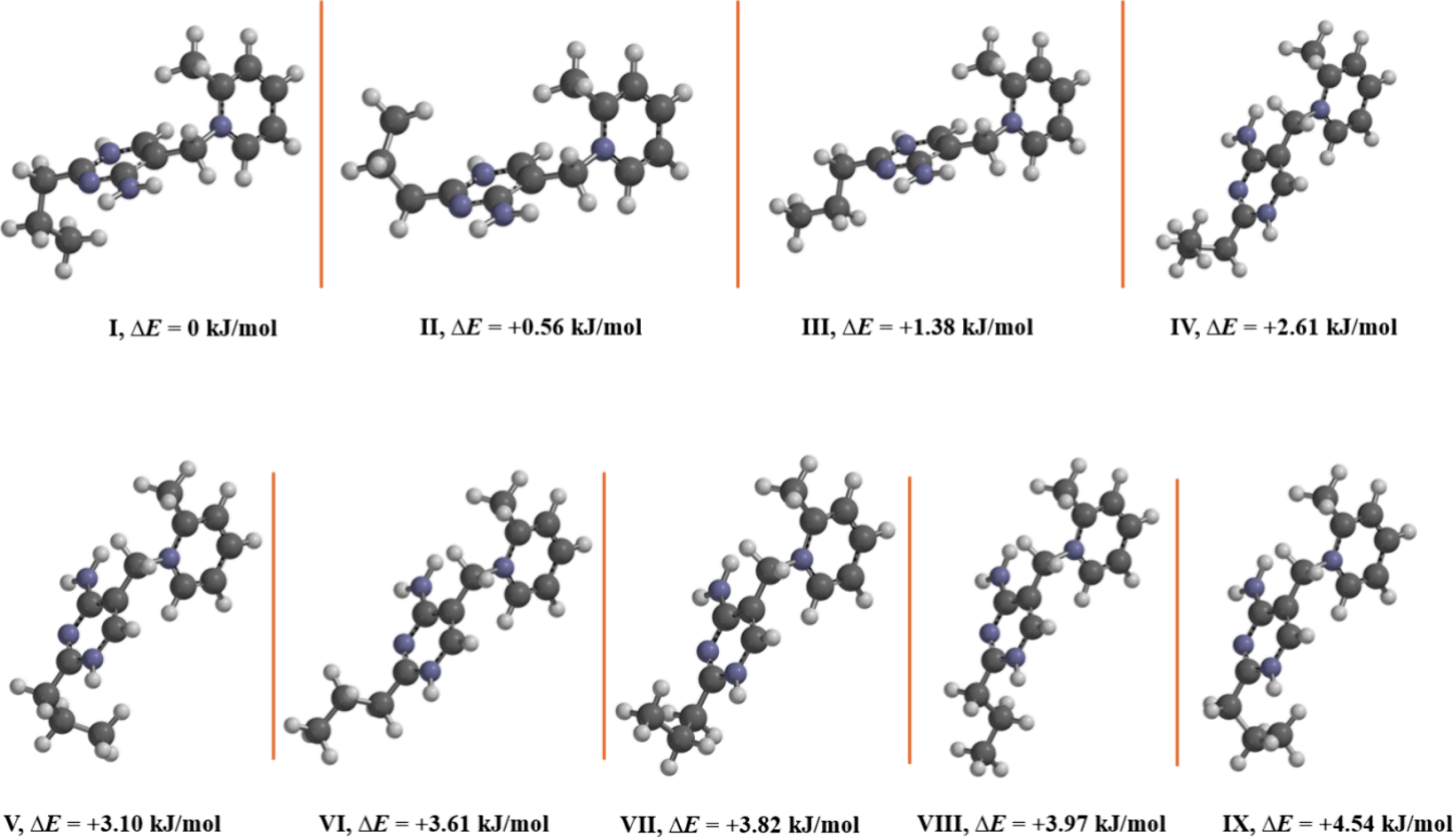
Nine lowest energy conformers of the amprolium dication. Energies are shown relative to the lowest (**I**).

Examining the nine theoretical structures reveals key insights into both the challenges encountered with growing the crystals as well as the observed geometries and disorder within the crystals. For example, one of the key differences in the molecules is the conformation of the propyl chain. All of the nine structures display either the gauche (torsion angles of ca. ±60°) or the staggard (ca. 180°) propyl chain conformation. Of the nine structures examined, seven display a gauche conformation including the two lowest energy cations. This helps rationalize our observations from the crystals wherein the gauche conformations are more common than the staggard.

As discussed in Section 3.2.1, three conformations of the rings in the cations are observed, distinguished by torsion angles. For the nine theoretical molecules, two conformations are also observed. Structures I–III have a N—C—C—C torsion angle of ca. 177°, comparable to that observed in **Amp-NTf**_**2**_ and **Amp-BETI**. The remaining compounds (IV–IX) display a torsion angle of ca. 72° which is similar to that observed in **Amp-FSI**. The other remaining crystal structures have slightly larger torsions in the ±80–90° range, placing them slightly outside the range of the calculated structures. However, the calculations are based on just a single dication in solution and do not account for interactions in the crystalline state which could help stabilize slightly larger torsion angles.

With respect to the electronic structure, the HOMO, LUMO, and the ESP are shown in Figure 8. The LUMO is a predominantly π character orbital set existing on the pyridinium ring while the HOMO is similar, but instead on the pyrimidinium ring. Despite the location of these orbitals, interactions with the π system of the rings (e.g., H···C|C···H and C···C) are less prevalent in these structures. This is likely due to the presence of multiple hydrogen bonding donors and acceptors, leading to the formation of more energetically favorable H-interactions rather than less energetically favored π interactions.

**Figure 8:**
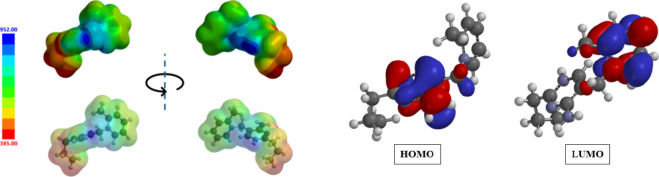
Calculated electrostatic potential of the amprolium cation mapped on an isosurface. The alkyl group shows the most negative region (red) on the cation while the hydrogens are the most positive (dark blue). The highest occupied and lowest unoccupied molecular orbitals on the amprolium dication are shown. Both orbitals are of predominantly π-character residing on the two aromatic rings.

With respect to the ESP, distinct regions of positive and negative potential are seen over the entire cation. Similar to what has been shown in bis-alkylated IL systems, the propyl chain is predominantly surrounded by negative potential. The pyrimidine nitrogen moiety, however, has the most negative potential. The benzylic hydrogens along with the protonated pyridinium N—H moieties are the most positive regions, as expected. As has been demonstrated, these positive and negative regions of the ESP are strongly correlated with the formation of interactions in the crystalline state.^46^

### 3.5 Thermal Properties

Thermal data for the compounds is shown in Figure 9 and is summarized in Table 1. Overall, the compounds follow the general trends of ILs with the perfluoroalkyl anion species exhibiting the highest thermal stabilities, both with respect to onset (*T*_onset_) and decomposition temperatures (*T*_dec_). As noted in our previous studies,^40^ the NCyF anion imparts higher thermal stability than NTf_2_ or BETI containing ILs. **Amp-NCyF** displays the highest *T*_onset_ (318 °C) and *T*_dec_ (362 °C) of all the compounds examined. Broadly examining the eight compounds shows a distinction between the perfluoroalkyl compounds (NTf_2_^–^, BETI, NCyF, FSI, OTf^–^) and the “spherical” anions (BF_4_^–^, PF_6_^–^, Cl^–^). The perfluoroalkyl species display a simpler decomposition profile, with a large single step wherein most of the mass is lost. The spherical anion species, however, display a more complex decomposition mechanism.

**Table 1:** Summarized Thermophysical Data for the Amprolium Compounds

	*T*_m_ (°C)	*T*_onset_ (°C)	*T*_dec_ (°C)
**Amp-NTf_2_**	98.02	296.39	350.08
**Amp-BETI**	112.11	302.59	349.11
**Amp-NCyF**	153.19	318.01	362.37
**Amp-FSI**	112.93	200.93	240.95
**Amp-BF_4_**		184.16	191.72
**Amp-PF_6_**		180.19	192.96
**Amp-OTF**		276.71	348.31
**Amp-Cl**		221.41	246.74

**Figure 9:**
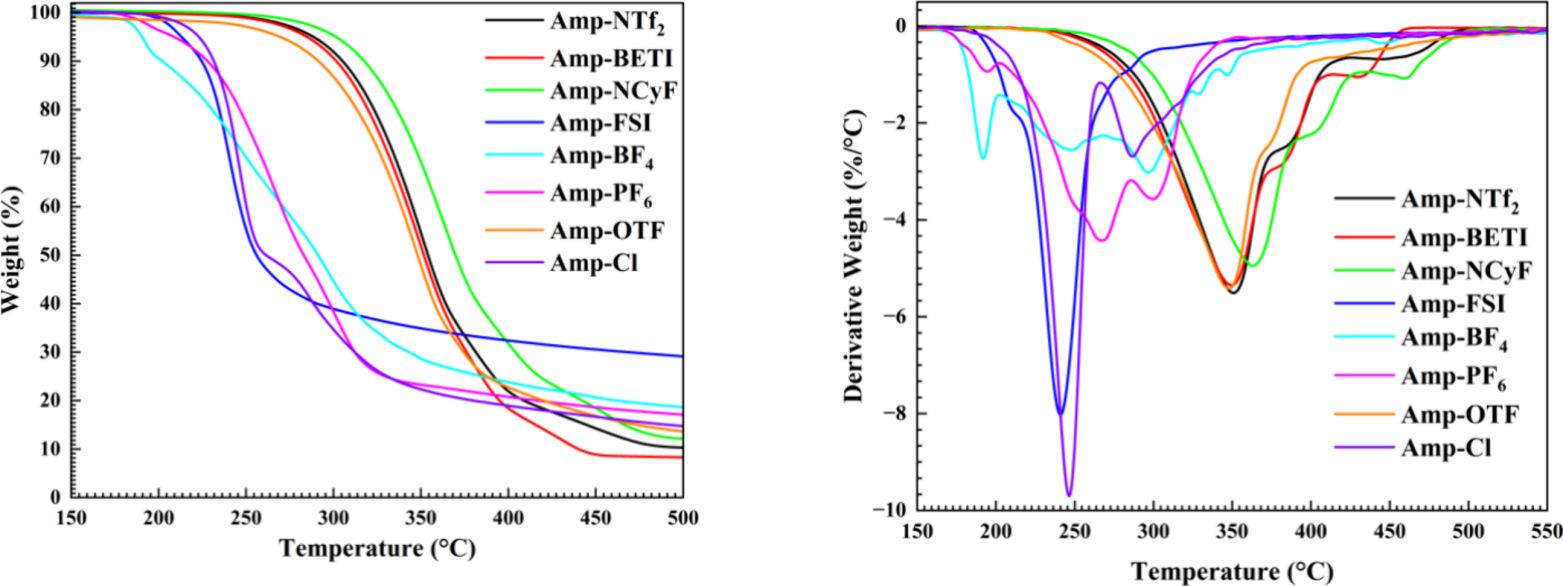
(left) Thermal decomposition traces of the amprolium compounds. (right) First derivative of the thermal decomposition traces providing insight into different decomposition steps in each curve. A complex decomposition pathway for the compounds is noted as seen by the presence of multiple peaks.

Examining the first derivative of the weight loss curve reveals that BF_4_^–^, PF_6_^–^, and Cl^–^ all have multiple, distinct decomposition steps. Both BF_4_^–^ and PF_6_^–^ display three steps while Cl^–^ shows two. It is likely that the three steps in BF_4_^–^ and PF_6_^–^ correspond to decomposition of the anion followed by decomposition of the cation. It has been shown that these particular anions decompose in the presence of acidic hydrogens.^47,48^ Given the similarities of the temperatures for each step for both the PF_6_^–^ and BF_4_^–^ compounds, it is further likely that the mechanism for each decomposition is similar. **Amp-BF**_**4**_ **, −PF**_**6**_, **–OTf**, and **–Cl** all show an endothermic event accompanying the *T*_onset_. The subsequent decomposition steps for these compounds are also accompanied by endotherms pointing toward a more complex decomposition pathway when contrasted with the perfluoroalkyl anions.

We examined the stability of both **Amp-NCyF** and **Amp-PF**_**6**_ under air to contrast stability under ambient conditions. Markedly, the decomposition for **Amp-PF**_**6**_ under air is nearly identical to that under nitrogen. One exception, however, is the presence of an exotherm at approximately 400 °C indicating the potential for oxidative degradation of the molecule. This exothermic event is also present in **Amp-NCyF** pointing toward this step being degradation of the cation, rather than the anion, given the similarity of the temperature at which it occurs.

The perfluoroalkyl containing compounds all display clear melting points, with **Amp-NTf**_**2**_ having the lowest of the grouping (ca. 98 °C) while **Amp-NCyF** has the highest observed melting point for the compounds at 153 °C. This follows on the trends observed from our previous work wherein the NCyF anion imparts high melting points. With a melting point below 100 °C, **Amp-NTf**_**2**_ falls within the thermally defined domain of ILs. The remaining compounds do not show any measurable phase transitions.

### 3.6 Noncovalent Interactions

Figure 10 presents the complete interaction fingerprints for the compounds. Note that the fingerprints for the cations in **Amp-NCyF** are omitted due to severe disorder and the presence of solvent. In the cases of **Amp-NTf**_**2**_, **Amp-BETI**, and **Amp-Cl**, two cations are present in the asymmetric unit, resulting in two distinct fingerprints. An overlay of these fingerprints, shown in Figure 11, illustrates variations in cation interactions. For example, although the two cations in both **Amp-BETI** and **Amp-Cl** share similar classical hydrogen bonding “spikes,″ differences in other hydrogen interaction regions, primarily H···H interactions, help visualize the crystallographically distinct nature of the cations.

**Figure 10:**
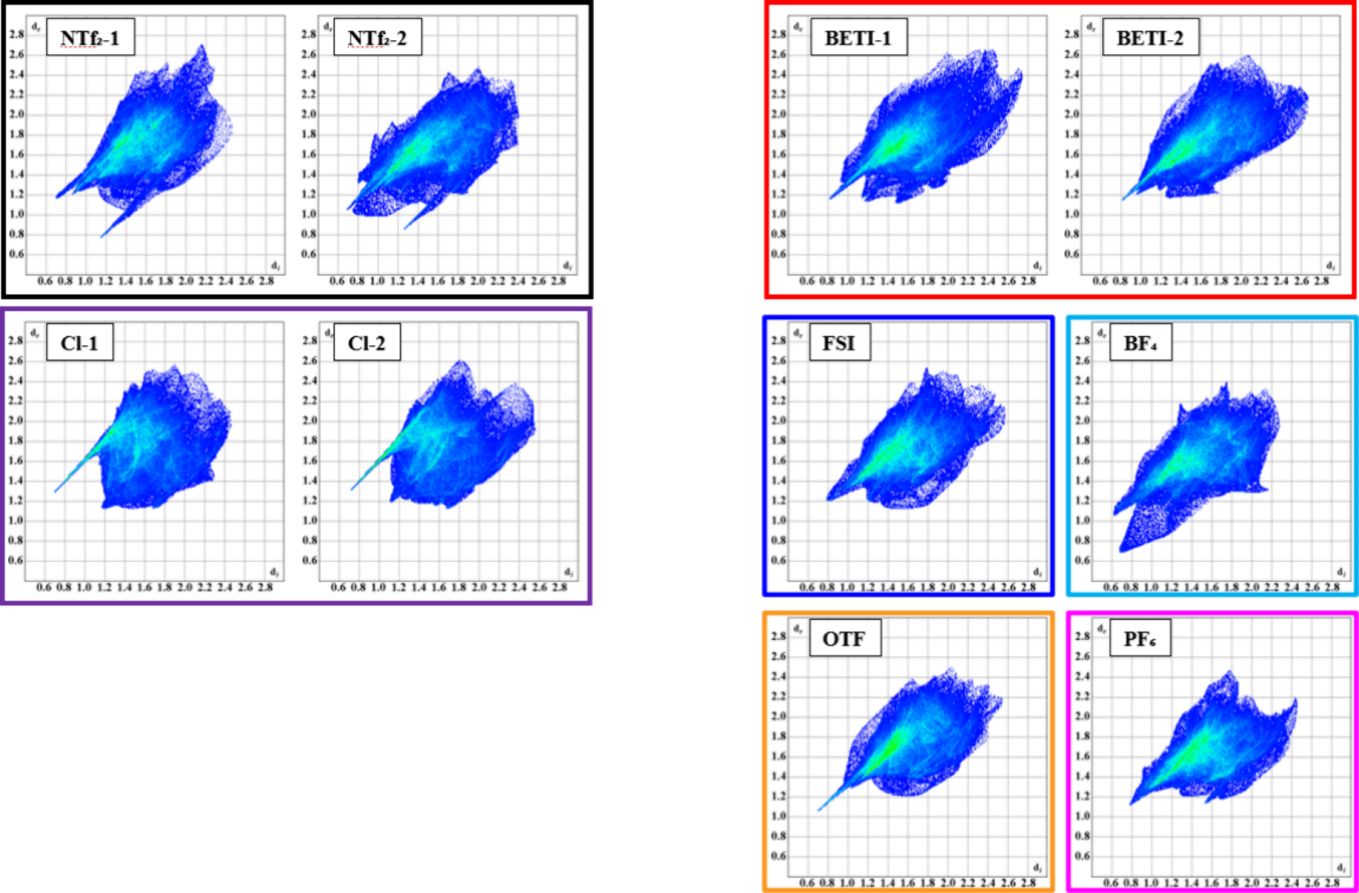
Complete interaction fingerprint for the ten independent cations within the crystal structures. Colors are used to distinguish the compounds by anions. Compounds **Amp-NTf**_**2**_, **Amp-BETI**, and **Amp-Cl** have two cations in the asymmetric unit. The interaction fingerprints help show common interaction motifs, such as multiple hydrogen bonds manifesting as sharp spikes in each image.

**Figure 11:**
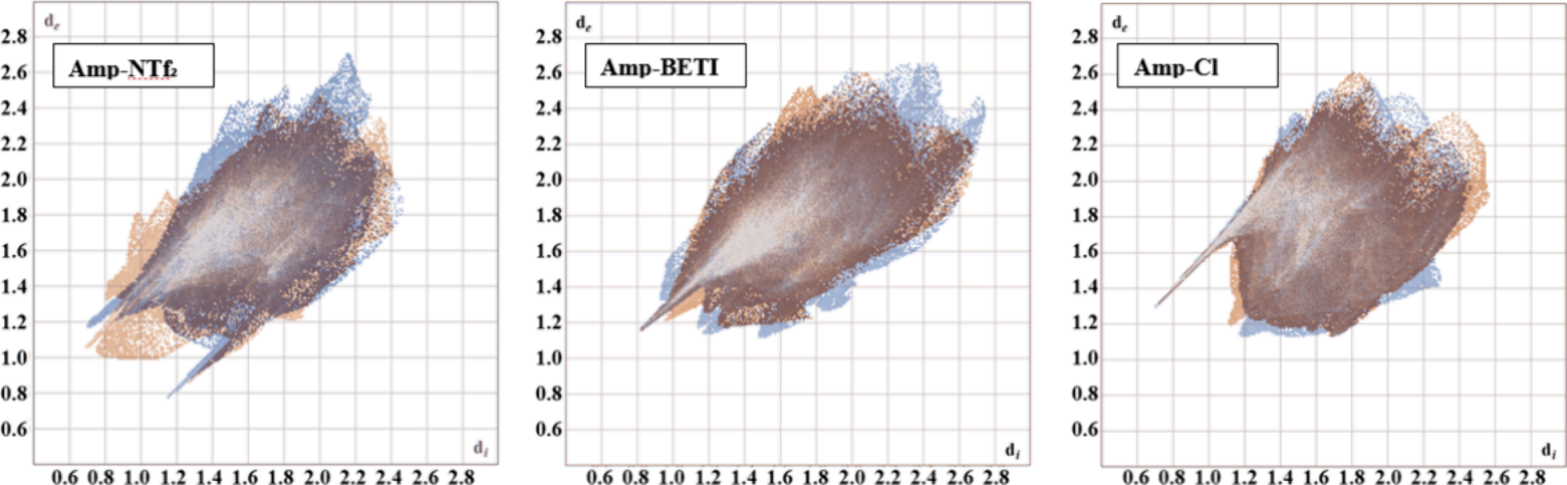
Overlapped fingerprints of **Amp-NTf**_**2**_, **Amp-BETI**, and **Amp-Cl** cations. The coloring (orange and blue) is used to distinguish the two independent cations. The independent colors of orange and blue show where unique interactions are formed with the cations while the darker color are the overlapped, similar interactions.

All fingerprints display a prominent spike corresponding to hydrogen bonding. Numerous hydrogen bonding interactions occur between anions and cations due to the various donor and acceptor groups. Overall, the fingerprints are similar in area and range since they derive from the same cation structure, with only minor variations in the propyl chain and plane angles that affect volume and surface area (see Supporting Information). Additionally, disorder in the cation increases the calculated surface area because disordered units were treated as a single part. Finally, the diffuse interaction regions in the top right quadrant of the fingerprints suggest inefficient crystal packing, a common feature of ionic liquids that contributes to their lower melting points.^49^

The green regions in the center of the fingerprint correspond to predominantly nondirectional H-based interactions (e.g., C—H···H). Given the organic nature of these cations this is logical as the hydrogen-based interactions are typically the largest percentage of interactions within organic crystals.^50^ The graphed interaction percentages are shown in Figure 12. As expected, the H interactions comprise the largest percentage for all the compounds studied, with an average of 84.7% of interactions involving a hydrogen atom. There is little variation in this overall percentage, with the entire set of crystals showing a range of only 1.6% for the H···All interactions.

**Figure 12:**
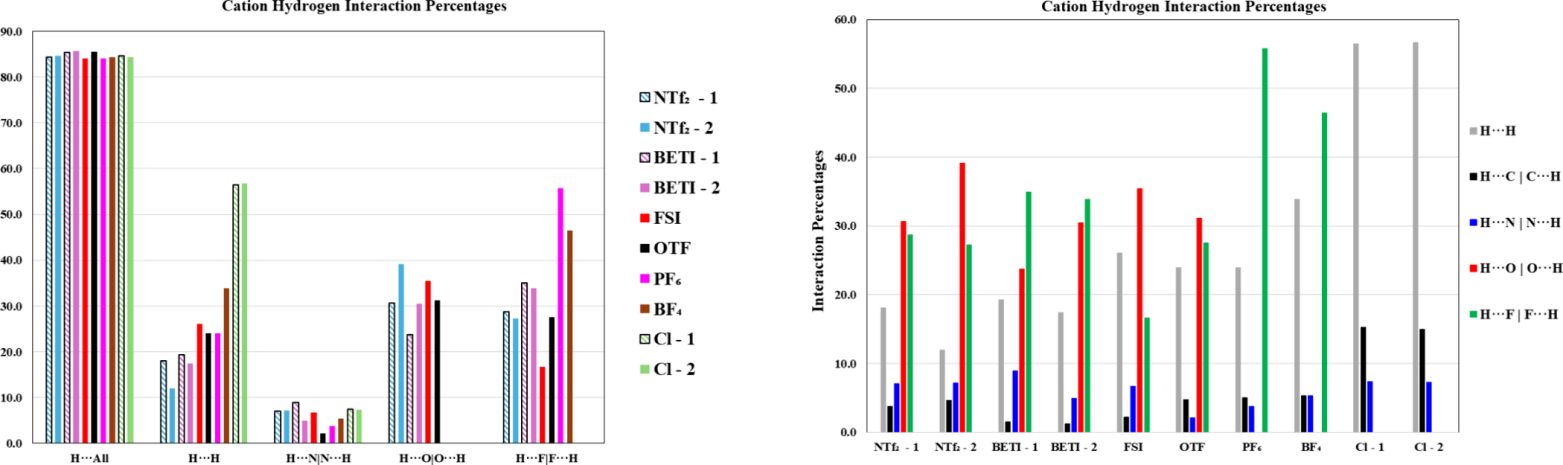
Two representations of the hydrogen interaction percentages in the amprolium crystals. (Left) Interaction totals grouped by interaction type; (Right) Interactions grouped by cation from each crystal structure.

Within the hydrogen interactions, the largest contributions arise from classical hydrogen bonding (e.g., H···N|N···H, H···O|O···H, H···F|F···H). Among these, H···F|F···H interactions have the highest overall mean percentage at 34.0%. However, this mean is skewed by **Amp-PF**_**6**_ and **Amp-BF**_**4**_ **,** which lack oxygen atoms and thus cannot form H···O|O···H interactions. Excluding these two compounds reduces the mean for H···F|F···H interactions to 28.2%, which is lower than the H···O|O···H percentage of 31.8%. This adjusted percentage aligns with previous studies indicating that oxygen moieties on anions, due to their increased electron density, contribute more significantly to interactions.^42^

The formation of interactions with π systems in ILs is of importance to the physicochemical properties. With respect to Hirshfeld surface analysis, these interactions correspond to interactions with C (e.g., C···All, H···C|C···H, C···C, etc.). These interactions comprise ca. 10% of the interactions on average. Most of these interactions arise from cation–anion interactions with F···C|C···F and O···C|C···O interactions accounting for 3.4 and 4.6% respectively. Given the dicationic nature of these compounds, a larger percentage of cation–anion interactions is expected due to charge balance. Moreover, both aromatic rings contain cationic ammonium moieties, facilitating the formation of these interactions with the π systems.

The H···C|C···H interactions represent cation–cation interactions, making these particularly impactful with respect to thermophysical properties.^51^ Notably, **Amp-Cl** exhibits the highest proportion of these interactions. This may be attributed to the unique monatomic nature of the chloride anion compared to the polyatomic anions examined. Chloride ions predominantly engage in hydrogen bonding, a process that requires strict geometric alignment to be effective. These directional hydrogen bonds enforce specific cation arrangements, thereby preventing the formation of, for example, stacking interactions. Consequently, the system favors the formation of cation–cation alkyl C—H···π interactions. In essence, because chloride ions are monatomic, they can only form hydrogen bonds with specific geometric parameters rather than less energetically favorable alternatives that would necessitate different geometric configurations of the cations.

Continuing from the **Amp-Cl** discussion, anion geometry appears to have a pronounced impact on the percentage of H···C|C···H interactions. For example, contrasting **Amp-NTf**_**2**_ and **Amp-BETI** reveals a marked decrease from when the larger BETI anion is used. An increase in F···C|C···F interactions with the BETI anion points toward an increase in potential cation–anion interactions due to the addition of the perfluoroethyl chains in BETI when compared with NTf_2_. Additionally, the two spherical anions, BF_4_ and PF_6_, form nearly identical percentages of interactions, helping to emphasize the importance of shape with respect to IL properties. This is further reflected in the similarity of the positions of the anions wherein H···F hydrogen bonding between the cation and anions are at comparable geometries, reflected in similarities of the H···F|F···H fingerprints (Figure 13).

**Figure 13:**
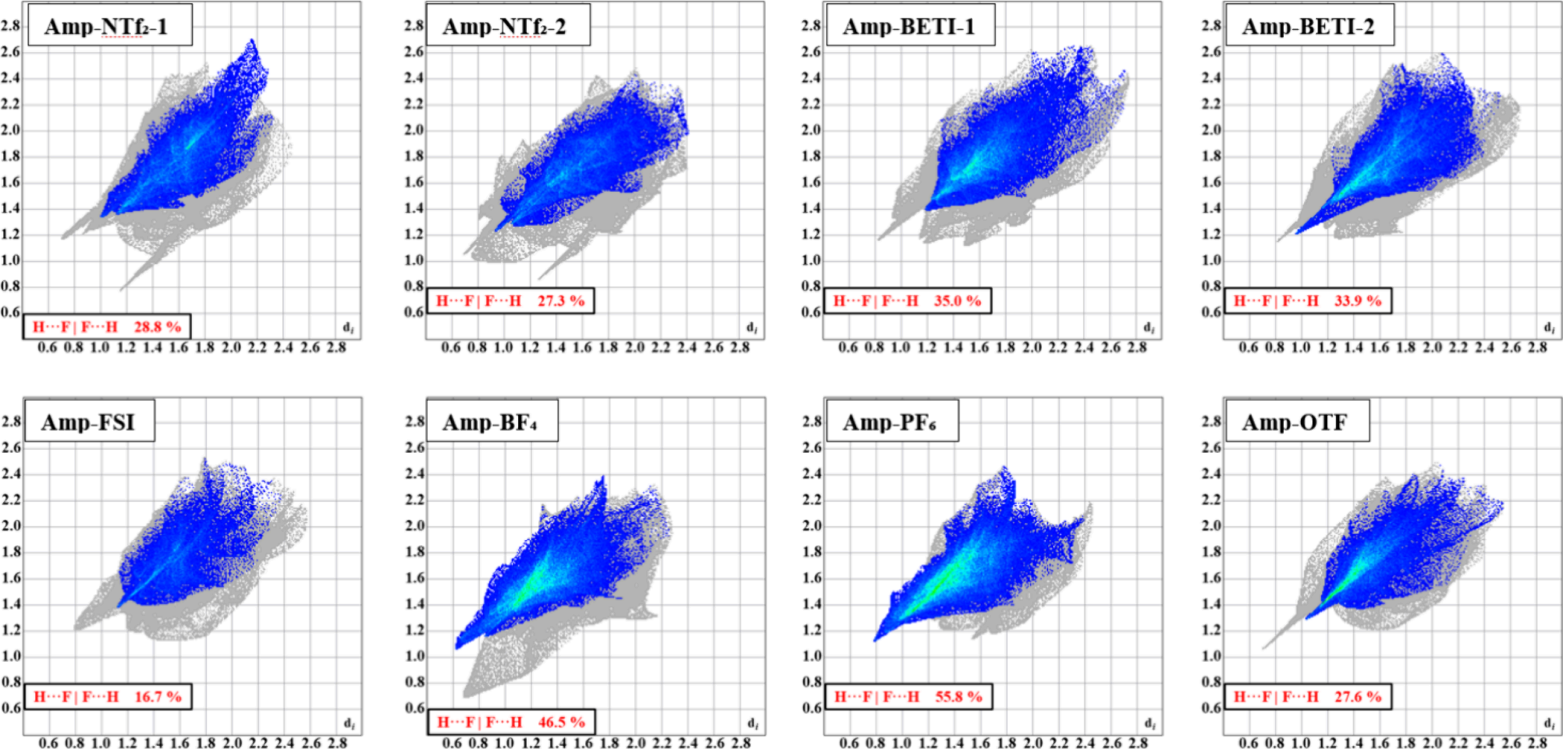
Depiction of the H···F|F···H interaction fingerprints for the crystals. The distinct shapes point toward the unique set of interactions based on the geometries and composition of the anions bearing the fluorine atoms.

One important observation is the low percentage of any π-π stacking interactions, represented by the C···C and N···C|C···N interactions. **Amp-BF**_**4**_ shows the largest percentage of the compounds (1.6% C···C), corresponding to a parallel offset stacking interaction formed between symmetry adjacent pyridinium rings. Of note, the higher percentage of N···C|C···N interactions in Amp-FSI is the result of close interactions between the imide nitrogen on the anion and the pyridinium ring. Thus, despite being comprised of two aromatic moieties, amprolium cations do not appear to favor the formation of π stacking type interactions.

## 4 Conclusions

The amprolium cation presents an interesting challenge with respect to the formation of ILs. The dicationic nature of the molecule along with numerous hydrogen bonding donors and acceptors seemingly points toward the formation of highly crystalline, high melting salts. Despite these characteristics, crystals proved challenging to grow while also displaying lower than anticipated melting points. A part of these challenges is attributed to the use of perfluoralkyl anions designed to frustrate crystalline growth. However, several key structural aspects of the cation, such as multiple conformations of the propyl chain in addition to multiple energetically accessible ring conformations, also helps lower the melting points to near the thermally defined limits of 100 °C for ILs. Two points of conformational disorder are noted in the cation: the pyrimidine ring and the propyl moiety. The combination of both of these rotations likely rationalizes the difficulty in crystallization for these compounds. Rotation of the pyrimidine ring is accompanied by a change in location of hydrogen bond donors and acceptors as the amine moieties (donors) and pyrimidine nitrogen (acceptors) undergo a significant change in location. These hydrogen bonds are a major driving force in the formation of the solid state.

- Three conformations of the cation exist with respect to the aromatic moieties. Multiple accessible conformations of the ancillary chains of ILs are a vital component of the thermal properties of this class of compounds. Further, this conformational flexibility perhaps sheds light onto the biological behavior of amprolium compounds as mimics of thiamine which also displays multiple conformations of its rings.
- Higher energy conformations of both the NCyF and BETI anion are observed within the crystal structures. A transition state geometry, between *cisoid* and *transoid* is found in **Amp-BETI**. Furthermore, a boat conformation of the NCyF anion is observed, providing the first crystallographic structure for both geometries for the anions. We speculate that due to the extensive hydrogen bonding within the lattice, higher energy conformations could be more readily stabilized.
- Hydrogen interactions dominate the intermolecular forces, with nearly 85% of the total interactions involving hydrogen atoms in some manner. Perhaps unsurprisingly, hydrogen bonding (H···O|O···H and H···F|F···H) interactions are the two highest average percentages of interactions, with H···H being third. Given the dicationic nature of the amprolium moiety, two anions are required for charge balance. The fluorinated sulfonimide based anions (NTf_2_^–^, BETI, NCyF^–^, FSI^–^), thus form extensive hydrogen bonds with the cation.

Our analysis of the amprolium compounds has revealed several key insights into the design and properties of these compounds. These principles, derived from the study herein, are useful heuristic concepts which can be more broadly applied to structurally ‘simpler’ pyridinium-based ILs to develop novel alkylated benzyl derivatives.

## Supplementary Material



## Notes

Crystallographic torsion and plane angles; complete interaction percentages of the crystals; molecular descriptor and fragment patches data; crystallographic experimental data; crystallization conditions; nuclear magnetic spectra for the compounds (^1^H, ^13^C, ^19^F, and ^31^P); simultaneous thermal analysis (STA) traces; and computational output files (PDF)

## Abbreviations

NTf_2_: bis­(trifluoromethane)­sulfonimide
BETI: bis­(pentafluoroethanesulfonyl)­imide
NCyF: 1,1,2,2,3,3-Hexafluoropropane-1,3-disulfonimide
FSI: bis­(fluorosulfonyl)­imide
OTF: trifluoromethanesulfonate, triflate
Amp: Amprolium
VB1: Vitamin B1 or thiamine HCl
HOMO: highest occupied molecular orbital
LUMO: lowest unoccupied molecular orbital
ESP: electrostatic potential
IL: ionic liquid
