# Supplementary material for: Mapping the Crystallographic Landscape of Antivitamin Ionic Liquids: Structural Blueprints for Novel Architectures
Source: Cryst Growth Des. 2025 May 23;25(11):3968–79. doi: 10.1021/acs.cgd.5c00378 (PMC12142575; doi:10.1021/acs.cgd.5c00378)
Supplement: Supplementary file 1 [file cg5c00378_si_001.pdf]

# Supporting Information

**Mapping the Crystallographic Landscape of Anti-Vitamin Ionic Liquids: Structural Blueprints for Novel Architectures**

Clare McNeill,[a] Marija Scheuren,[a] Joseph Cooper,[a] Sophia Bellia,[a] Muhammadiqboli Musozoda,[b] Janayah N. Tolbert,[b] Matthias Zeller,[c] Arsalan Mirjafari,[b,*] Patrick C. Hillesheim[*a,d]

[a] Department of Chemistry and Physics, Ave Maria University, Ave Maria, Florida, 34142, United States

[b] Department of Chemistry, State University of New York at Oswego, Oswego, New York 13126, United States

[c] Department of Chemistry, Purdue University, West Lafayette, Indiana, 47907, United States

[d] Department of Chemistry, Illinois State University, Normal, Illinois 61761, United States

## Table of Contents

Torsion Angles……………..…………………………………………………………………………............................S3

Interaction Percentages.……..…………………………………………………………………...............................S4

Molecular Descriptors from Hirshfeld Analysis….…..………………………………………….................................S5

Crystallographic Tables …..…..………………………………………..........................................................................S6

Crystallization Conditions and Characterization of Compounds……………………………………………………….S8

NMR Spectra of the Products………………………...……………………………………………..............................S10

STA Thermograms of the Products…………...……………………………………………………............................S24

Computational Output….……………………………………………………………………………..........................S29

| | ϕ | Plane Angle | Propyl Chain Torsion | Notes |
|---|---|---|---|---|
| $NTf_2$ | | | | |
| A | -168.13 | 83.54 | 176.96 | Chain Disorder |
| A' | | | 57.00 | Chain Disorder |
| B | -90.65 | 82.27 | 69.22 | |
| | | | | |
| BETI | | | | |
| 1 | -176.13 | 81.71 | 71.92 | |
| 2 | -177.02 | 70.40 | 69.98 | |
| | | | | |
| NCyF | | | | |
| A | -91.29 | 88.95 | 176.43 | Disordered Part A |
| B | -86.85 | 88.00 | 66.40 | Disordered Part B |
| C | 93.90 | 84.82 | 53.58 | Disordered Part C |
| | | | | |
| FSI | -74.73 | 71.65 | 175.55 | |
| | | | | |
| $BF_4$ | | | | |
| A | 82.99 | 78.32 | 62.23 | Chain Disorder |
| A' | | | 172.14 | Chain Disorder |
| | | | | |
| $PF_6$ | 83.62 | 79.09 | 62.48 | |
| | | | | |
| OTF | | | | |
| A | 91.71 | 84.29 | 60.49 | |
| B | -107.37 | 83.77 | 79.23 | |
| | | | | |
| Cl | | | | |
| 1 | -89.12 | 85.70 | 80.64 | |
| 2 | 92.47 | 88.78 | 81.79 | |

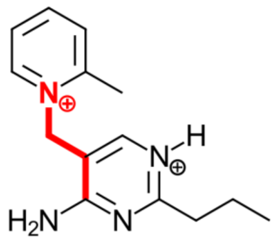


**Table S1.** Complete numerical data of the N—C—C—C torsion angle (ϕ) shown in the image. Plane angle and propyl chain torsion angles are also shown.

| | $NTf_2$ - 1 | $NTf_2$ - 2 | BETI - 1 | BETI - 2 | FSI | OTF | $PF_6$ | $BF_4$ | Cl - 1 | Cl - 2 |
|---|---|---|---|---|---|---|---|---|---|---|
| | | | | | | | | | | |
| **H···All** | 84.3 | 84.7 | 85.4 | 85.6 | 84.1 | 85.5 | 84.0 | 84.3 | 84.6 | 84.4 |
| **H···H** | 18.1 | 12.0 | 19.3 | 17.5 | 26.1 | 24.0 | 24.0 | 33.9 | 56.5 | 56.7 |
| **H···N\|N···H** | 7.1 | 7.2 | 9.0 | 5.0 | 6.7 | 2.2 | 3.8 | 5.4 | 7.4 | 7.3 |
| **H···O\|O···H** | 30.7 | 39.2 | 23.8 | 30.5 | 35.5 | 31.2 | - | - | - | - |
| **H···F\|F···H** | 28.8 | 27.3 | 35.0 | 33.9 | 16.7 | 27.6 | 55.8 | 46.5 | - | - |
| | | | | | | | | | | |
| **C·All** | 10.1 | 9.3 | 9.4 | 9.4 | 10.0 | 9.7 | 10.7 | 10.4 | 10.0 | 10.2 |
| **C·C** | 0.0 | 0.0 | 0.0 | 0.0 | 0.0 | 0.6 | 0.0 | 1.6 | 0.0 | 0.0 |
| **H·C\|C·H** | 3.8 | 4.7 | 1.6 | 1.3 | 2.3 | 4.8 | 5.1 | 5.4 | 15.3 | 15.0 |
| **N·C\|C·N** | 0.9 | 0.2 | 0.0 | 0.0 | 1.4 | 0.1 | 0.0 | 0.0 | 0.0 | 0.0 |
| **O·C\|C·O** | 4.6 | 5.1 | 4.8 | 4.9 | 5.1 | 3.3 | - | - | - | - |
| **F·C\|C·F** | 2.5 | 1.3 | 2.9 | 3.6 | 1.9 | 2.7 | 7.5 | 5.1 | - | - |
| | | | | | | | | | | |
| **N·All** | 5.6 | 6.0 | 5.2 | 5.0 | 5.9 | 4.7 | 5.3 | 5.3 | 5.4 | 5.4 |
| **N·N** | 0.4 | 0.4 | 0.0 | 0.0 | 0.7 | 0.4 | 0.0 | 0.2 | 0.0 | 0.0 |
| **O·N\|N·O** | 2.7 | 1.7 | 1.6 | 1.7 | 2.2 | 2.2 | - | - | - | - |
| **F·N\|N·F** | 0.3 | 1.0 | 2.0 | 1.6 | 1.7 | 0.9 | 3.1 | 2.0 | 1.1 | 1.3 |
| | | | | | | | | | These are N-Cl | |

Table S2. Numerical interaction percentages derived from the Hirshfeld surface analysis of the cations.

| | NTf2-1 | NTf2-2 | BETI 1 | BETI 2 | FSI | OTF | PF6 | BF4 | Cl 1 | Cl 2 |
|---|---|---|---|---|---|---|---|---|---|---|
| 1 | 4.8 | 15.6 | 9.2 | 13.5 | 22.7 | 10.8 | 10.8 | 26.3 | 38.3 | 11.4 |
| 2 | 17.8 | 1 | 19.4 | 19.7 | 13.9 | 16.4 | 9.2 | 12.7 | 4.9 | 37.7 |
| 3 | 8.8 | 0.9 | 44.0 | 12.3 | 28.1 | 33.2 | 11.5 | 23.1 | 2.6 | 17.2 |
| 4 | 17 | 13.9 | 13.6 | 12.4 | 8.4 | 11.2 | 24.9 | 2.8 | 7.0 | 3.7 |
| 5 | 20.5 | 3.8 | 8.1 | 36.3 | 9.7 | 5.4 | 16.9 | 17.3 | 1.0 | 6.7 |
| 6 | 34.6 | 1.7 | 0.7 | 5.5 | 6.4 | 5.6 | 27.1 | 21.7 | 26.0 | 20.7 |
| 7 | 37 | 24.6 | 34.3 | 13.9 | 9.8 | 4.0 | 10.1 | 4.7 | 9.9 | 1.9 |
| 8 | 11 | 46.5 | 2.3 | 10.7 | 32.3 | 32.0 | 17.4 | 5.8 | 4.9 | 12.0 |
| 9 | 19.7 | 35.7 | 42.0 | 49.1 | 18.8 | 23.7 | 0.6 | 9.8 | 13.6 | 9.8 |
| 10 | 13.1 | 14.5 | 11.3 | 34.5 | 12.4 | 10.7 | 10.4 | 6.8 | 7.4 | 11.2 |
| 11 | 35.3 | 11.4 | 22.7 | 21.9 | 2.4 | 10.2 | 8.1 | 11.9 | 15.3 | 21.6 |
| 12 | 16.4 | 10.5 | 4.5 | 14.9 | 32.1 | 13.5 | 7.8 | 5.9 | 20.6 | 6.1 |
| 13 | 1.8 | 22.5 | 11.4 | 20.8 | 18.7 | 16.8 | 9.4 | 34.2 | 17.2 | 11.1 |
| 14 | 30.4 | 30.6 | 43.5 | 8.0 | 20.6 | 14.2 | 12.5 | 12.9 | 2.6 | 40.4 |
| 15 | 30.9 | 12.2 | 5.8 | 42.8 | 14.6 | 26.0 | 27.1 | 8.8 | 19.5 | 4.6 |
| 16 | 13.8 | 21.9 | 20.1 | 0.8 | 37.5 | 0.1 | 15.8 | 26.1 | 8.2 | 3.1 |
| 17 | | 36.1 | 12.6 | 0.0 | 10.6 | 14.0 | 13.4 | 11.3 | 40.3 | 6.2 |
| 18 | | 1.2 | 15.1 | 5.9 | 10.2 | 19.1 | 27.0 | 0.3 | 2.6 | 1.1 |
| 19 | | 10.4 | | 0.7 | 3.9 | 10.5 | 0.8 | 1.7 | 21.8 | 26.1 |
| 20 | | | | | 2.1 | 19.8 | 15.6 | 6.0 | 11.8 | 19.0 |
| 21 | | | | | | 13.0 | 11.3 | 16.2 | 11.1 | 15.1 |
| 22 | | | | | | 5.1 | 15.2 | 19.8 | 2.0 | 7.5 |
| 23 | | | | | | | | 14.2 | 7.1 | 0.9 |
| 24 | | | | | | | | 3.0 | 10.2 | 10.1 |
| 25 | | | | | | | | 0.9 | | |

| | NTf2-1 | NTf2-2 | BETI 1 | BETI 2 | FSI | OTF | PF6 | BF4 | Cl 1 | Cl 2 |
|---|---|---|---|---|---|---|---|---|---|---|
| High | 37.0 | 46.5 | 44.0 | 49.1 | 37.5 | 33.2 | 27.1 | 34.2 | 40.3 | 40.4 |
| 2nd | 35.3 | 36.1 | 43.5 | 42.8 | 32.3 | 32.0 | 27.1 | 26.3 | 38.3 | 37.7 |
| 3rd | 34.6 | 35.7 | 42.0 | 36.3 | 32.1 | 26.0 | 27.0 | 26.1 | 26.0 | 26.1 |
| Fragment Count | 16 | 19 | 18 | 19 | 20 | 22 | 22 | 25 | 24 | 24 |
| Volume | 320.62 | 332.38 | 342.48 | 344.71 | 323.56 | 340.09 | 312.99 | 304.99 | 325.72 | 326.35 |
| Area | 313.19 | 315.17 | 320.67 | 323.78 | 315.5 | 315.51 | 303.28 | 304.35 | 306.19 | 305.43 |
| Globularity | 0.723 | 0.736 | 0.738 | 0.734 | 0.722 | 0.747 | 0.735 | 0.720 | 0.748 | 0.751 |
| Asphericity | 0.266 | 0.202 | 0.205 | 0.208 | 0.309 | 0.243 | 0.279 | 0.324 | 0.277 | 0.251 |

Table S3. Tabulated surface information from the analysis of the cations. Fragment patch surface area information is shown (colored grids).

| | Amp-$NTf_2$ | Amp-BETI | Amp-NCyF | Amp-FSI |
|---|---|---|---|---|
| **Crystal Data** | | | | |
| Chemical formula | $C_{14}H_{20}N_4{\cdot}2(C_2F_6NO_4S_2)$ | $4(C_4F_{10}NO_4S_2){\cdot}2(C_{14}H_{20}N_4){\cdot}H_2O$ | $C_{14}H_{20}N_4{\cdot}2(C_3F_6NO_4S_2){\cdot}C_5H_{12}O$ | $C_{14}H_{20}N_4{\cdot}2(F_2NO_4S_2)$ |
| $M_r$ | 804.64 | 2027.37 | 916.80 | 604.60 |
| Crystal system, space group | Triclinic, $P\bar{1}$ | Monoclinic, $P2_1$ | Monoclinic, $P2_1/c$ | Triclinic, $P\bar{1}$ |
| Temperature (K) | 150 | 150 | 150 | 150 |
| $a$, $b$, $c$ (Å) | 9.1248 (3), 12.8151 (3), 27.6177 (8) | 10.007 (3), 27.311 (17), 13.689 (6) | 8.7446 (9), 34.286 (7), 12.3052 (19) | 8.1667 (4), 12.1071 (6), 13.6674 (6) |
| α, β, γ (°) | 88.3243 (14), 82.0274 (15), 78.8368 (15) | 96.479 (11) | 97.948 (6) | 114.559 (2), 100.003 (2), 94.965 (2) |
| $V$ (Å$^3$) | 3137.75 (16) | 3718 (3) | 3653.9 (10) | 1191.28 (10) |
| $Z$ | 4 | 2 | 4 | 2 |
| Radiation type | Cu $K\alpha$ | Mo $K\alpha$ | Mo $K\alpha$ | Mo $K\alpha$ |
| μ (mm$^{-1}$) | 3.95 | 0.41 | 0.38 | 0.48 |
| Crystal size (mm) | 0.34×0.26×0.11 | 0.39 × 0.19 × 0.09 | 0.22 × 0.18 × 0.09 | 0.48 × 0.42 × 0.12 |
| **Data collection** | | | | |
| Diffractometer | Bruker AXS D8 Quest | | | |
| Absorption correction | Multi-scan *SADABS* 2016/2 | Multi-scan TWINABS 2012/1 | Multi-scan *SADABS* 2016/2 | Multi-scan *SADABS* 2016/2 |
| $T_{min}$,$T_{max}$ | 0.532,0.754 | 0.641, 0.746 | 0.691, 0.746 | 0.647, 0.747 |
| No. of measured, independent and observed [$I > 2\sigma(I)$] reflections | 48377, 13419, 11438 | 42765, 24328, 21162 | 36463, 9081, 7002 | 31991, 8929, 6671 |
| $R_{int}$ | 0.070 | 0.029 | 0.038 | 0.044 |
| $(\sin \theta/\lambda)_{max}$ (Å$^{-1}$) | 0.640 | 0.716 | 0.671 | 0.770 |
| **Refinement** | | | | |
| $R[F^2 > 2\sigma(F^2)]$, $wR(F^2)$, $S$ | 0.051,0.138,1.05 | 0.047, 0.127, 1.04 | 0.057, 0.158, 1.04 | 0.039, 0.108, 1.04 |
| No. of reflections | 13419 | 24328 | 9081 | 8929 |
| No. of parameters | 1304 | 1721 | 1333 | 382 |
| No. of restraints | 2583 | 4096 | 4301 | 168 |
| H-atom treatment | Constrained | Mixed | Constrained | Mixed |
| $\Delta\rho_{max}$, $\Delta\rho_{min}$(e Å$^{-3}$) | 0.78,−0.38 | 0.39, −0.49 | 0.35, −0.36 | 0.73, −0.52 |
| Absolute structure | - | Flack x determined using 6494 quotients [(I+)-(I-)]/[(I+)+(I-)] | - | - |
| Absolute structure parameter | - | 0.359 (19) | - | - |

| | Amp-BF4 | Amp-PF6 | Amp-OTF |
|---|---|---|---|
| **Crystal Data** | | | |
| Chemical formula | $C_{14}H_{20}N_4 \cdot 2(BF_4)$ | $C_{14}H_{20}N_4 \cdot 2(F_6P)$ | $2(CF_3O_3S) \cdot C_{14}H_{20}N_4$ |
| $M_r$ | 417.96 | 534.28 | 542.48 |
| Crystal system, space group | Monoclinic, $P2_1/n$ | Orthorhombic, $Pna2_1$ | Monoclinic, $P2_1/n$ |
| Temperature (K) | 150 | 150 | 150 |
| *a*, *b*, *c* (Å) | 11.9644 (12), 7.9063 (7), 20.2351 (16) | 24.7107 (10), 8.0988 (3), 10.4983 (4) | 13.5768 (5), 8.1075 (3), 20.6587 (7) |
| α, β, γ (°) | 99.734 (4) | 90 | 96.350 (1) |
| $V$ (Å$^3$) | 1886.6 (3) | 2100.99 (14) | 2260.03 (14) |
| $Z$ | 4 | 4 | 4 |
| Radiation type | Mo $K\alpha$ | Mo $K\alpha$ | Mo $K\alpha$ |
| μ (mm$^{-1}$) | 0.14 | 0.32 | 0.33 |
| Crystal size (mm) | 0.38 × 0.36 × 0.15 | 0.28 × 0.25 × 0.18 | 0.37 × 0.19 × 0.11 |
| **Data collection** | | | |
| Diffractometer | Bruker AXS D8 Quest | | Bruker AXS D8 Quest Eco |
| Absorption correction | Multi-scan, *SADABS* 2016/2 | | |
| $T_{min}, T_{max}$ | 0.686, 0.746 | 0.685, 0.747 | 0.707, 0.746 |
| No. of measured, independent and observed [$I > 2\sigma(I)$] reflections | 60528, 5757, 4805 | 37769, 7988, 7101 | 45954, 6949, 5999 |
| $R_{int}$ | 0.046 | 0.039 | 0.026 |
| $(\sin \theta/\lambda)_{max}$ (Å$^{-1}$) | 0.715 | 0.770 | 0.716 |
| Refinement | | | |
| $R[F^2 > 2\sigma(F^2)]$, $wR(F^2)$, $S$ | 0.049, 0.151, 1.08 | 0.035, 0.092, 1.03 | 0.042, 0.108, 1.07 |
| No. of reflections | 5757 | 7988 | 6949 |
| No. of parameters | 516 | 419 | 483 |
| No. of restraints | 1282 | 505 | 652 |
| H-atom treatment | Mixed | Constrained | Mixed |
| $\Delta\rho_{max}$, $\Delta\rho_{min}$ (e Å$^{-3}$) | 0.35, −0.24 | 0.33, −0.25 | 0.52, −0.49 |
| Absolute structure | - | Flack x determined using 3005 quotients [(I+)-(I-)]/[(I+)+(I-)] | - |
| Absolute structure parameter | - | 0.02 (2) | - |

**Amp-$NTf_2$**

Crystallized from the melt upon standing at room temperature. Yield: 83%

$^{1}$H NMR (500 MHz, DMSO) $\delta$ 8.66 (dd, $J$ = 6.3, 1.4 Hz, 1H), 8.55 (td, $J$ = 7.8, 1.4 Hz, 1H), 8.21 (s, 1H), 8.17 (dd, $J$ = 8.1, 1.6 Hz, 1H), 7.94 (ddd, $J$ = 7.9, 6.3, 1.7 Hz, 1H), 5.55 (s, 2H), 2.89 (t, $J$ = 7.5 Hz, 2H), 2.80 (q, $J$ = 7.4 Hz, 2H), 2.73 (t, $J$ = 7.4 Hz, 3H); $^{13}$C NMR (126 MHz, DMSO) $\delta$ 147.3, 146.2, 143.5, 130.5, 126.1, 121.3, 118.7, 105.5, 53.8, 40.0, 36.7, 20.5 (d, $J$ = 10.9 Hz), 13.8; $^{19}$F NMR (282 MHz, DMSO) $\delta$ 78.71.

**Amp-BETI**

The compound was mixed in hot water until it melted. Upon cooling, crystals formed in the water which were suitable for diffraction. Yield: 90%

$^{1}$H NMR (500 MHz, DMSO) $\delta$ 8.66 (dd, $J$ = 6.4, 1.4 Hz, 1H), 8.55 (dd, $J$ = 7.8, 1.4 Hz, 1H), 8.21 (s, 1H), 8.17 (dd, $J$ = 8.0, 1.6 Hz, 1H), 7.95 (m, 1H), 5.55 (s, 2H), 2.88 (s, 3H), 2.79 (t, $J$ = 7.5 Hz, 2H), 1.81 (q, $J$ = 7.4 Hz, 2H), 1.00 (t, $J$ = 7.4 Hz, 3H); $^{13}$C NMR (126 MHz, DMSO) $\delta$ 165.5, 163.9, 156.6, 147.5, 146.2, 143.5, 130.4, 126.1, 121.7, 121.4, 119.7, 119.4, 119.1, 117.4, 117.1, 116.8, 114.8, 114.1, 113.8, 112.1, 111.7, 111.4, 111.1, 109.4, 109.1, 105.4, 53.9, 36.7, 20.6, 20.5, 13.8; $^{19}$F NMR (282 MHz, DMSO) $\delta$ 78.55, 117.39.

**Amp-NCyF**

Slow diffusion of methyl tertbutyl ether into a saturated ethyl acetate solution of the compound. Yield: 85%

$^{1}$H NMR (500 MHz, DMSO) $\delta$ 8.65 (dd, $J$ = 6.4, 1.4 Hz, 1H), 8.56 (td, $J$ = 7.8, 1.4 Hz, 1H), 8.21 (s, 1H), 8.17 (m, 1H), 7.94 (ddd, $J$ = 7.7, 6.2, 1.6 Hz, 1H), 5.55 (s, 2H), 2.88 (s, 3H), 2.79 (t, $J$ = 7.5 Hz, 2H), 1.81 (m, 2H), 1.01 (t, $J$ = 7.4 Hz, 3H); $^{13}$C NMR (126 MHz, DMSO) $\delta$ 165.6, 163.9, 156.6, 146.2, 143.5, 130.5, 126.1, 115.3, 112.9, 112.7, 112.0, 110.5, 109.8, 109.6, 105.4, 53.9, 36.8, 20.6, 20.5, 13.9; $^{19}$F NMR (282 MHz, DMSO) $\delta$ 119.55, 125.82.

**Amp-FSI**

The compound was mixed in hot water until it melted. Upon cooling, crystals formed. Yield: 81%

$^{1}$H NMR (500 MHz, DMSO) $\delta$ 8.66 (dd, $J$ = 6.4, 1.4 Hz, 1H), 8.55 (td, $J$ = 7.8, 1.4 Hz, 1H), 8.21 (s, 1H), 8.16 (m, 1H), 7.94 (ddd, $J$ = 7.7, 6.2, 1.6 Hz, 1H), 5.55 (s, 2H), 2.88 (s, 3H), 2.80 (m, 2H), 1.81 (m, 2H), 1.01 (t, $J$ = 7.4 Hz, 3H); $^{13}$C NMR (126 MHz, DMSO) $\delta$ 165.4, 163.9, 163.8, 156.6, 147.3, 146.2, 143.5, 130.5, 126.1, 105.5, 53.8, 36.6, 20.6, 20.5, 13.8; $^{19}$F NMR (282 MHz, DMSO) $\delta$ 53.21.

**Amp-$BF_4$**

Slow diffusion of diethyl ether into a saturated acetonitrile solution of the compound. Yield: 70%

$^{1}$H NMR (500 MHz, DMSO) $\delta$ 8.64 (dd, $J$ = 6.4, 1.4 Hz, 1H), 8.55 (td, $J$ = 7.8, 1.4 Hz, 1H), 8.21 (s, 1H), 8.16 (m, 1H), 7.94 (ddd, $J$ = 7.8, 6.2, 1.6 Hz, 1H), 5.55 (s, 2H), 2.88 (s, 3H), 2.79 (d, $J$ = 7.7 Hz, 2H), 1.81 (q, $J$ = 7.4 Hz, 2H), 1.00 (t, $J$ = 7.4 Hz, 3H); $^{13}$C NMR (126 MHz, DMSO) $\delta$ 165.5, 163.9, 156.6, 147.5, 146.2, 143.5, 130.4, 126.1, 105.4, 53.8, 36.7, 20.6, 20.5, 13.9; $^{19}$F NMR (282 MHz, DMSO) $\delta$ 148.27.

**Amp-$PF_6$**

Slow diffusion of diethyl ether into a saturated methanol solution of the compound. Yield: 80%

$^1$H NMR (500 MHz, DMSO) $\delta$ 8.65 (dd, $J$ = 6.5, 1.4 Hz, 1H), 8.56 (td, $J$ = 7.8, 1.4 Hz, 1H), 8.21 (s, 1H), 8.17 (m, 1H), 7.94 (ddd, $J$ = 7.7, 6.2, 1.6 Hz, 1H), 5.55 (s, 2H), 2.88 (s, 3H), 2.80 (m, 2H), 1.81 (h, $J$ = 7.4 Hz, 2H), 1.01 (t, $J$ = 7.4 Hz, 3H); $^{13}$C NMR (126 MHz, DMSO) $\delta$ 165.5, 163.9, 156.6, 147.4, 146.2, 143.5, 130.5, 126.1, 105.4, 53.8, 36.7, 20.6, 20.5, 13.9; $^{19}$F NMR (282 MHz, DMSO) $\delta$ 68.88, 71.40.

**Amp-OTF**

Slow diffusion of chloroform into a saturated isopropyl alcohol solution of the compound. Yield: 74 %

$^1$H NMR (500 MHz, DMSO) $\delta$ 8.65 (d, $J$ = 5.5 Hz, 1H), 8.56 (td, $J$ = 7.8, 1.4 Hz, 1H), 8.18 (m, 2H), 7.94 (m, 1H), 5.54 (s, 2H), 2.88 (s, 3H), 2.79 (dd, $J$ = 7.9, 7.1 Hz, 2H), 1.80 (m, 2H), 1.00 (t, $J$ = 7.4 Hz, 3H); $^{13}$C NMR (126 MHz, DMSO) $\delta$ 165.6, 163.9, 156.6, 146.2, 143.5, 130.5, 126.1, 125.0, 122.4, 119.9, 105.4, 53.9, 36.7, 20.6, 20.5, 13.9; $^{19}$F NMR (282 MHz, DMSO) $\delta$ 77.75.

**$^{1}H$, $^{13}C$ and $^{19}F$ NMR spectra of products**

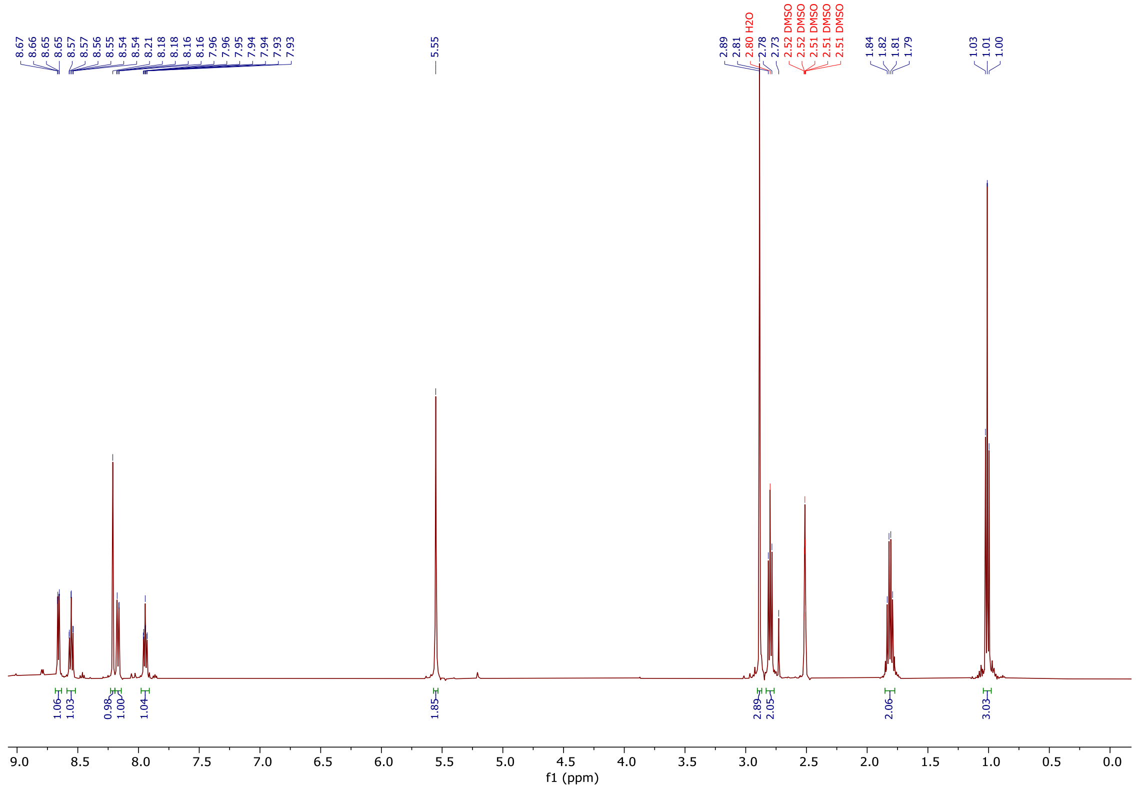

Figure S1. 1H NMR spectra of Amp-NTf₂

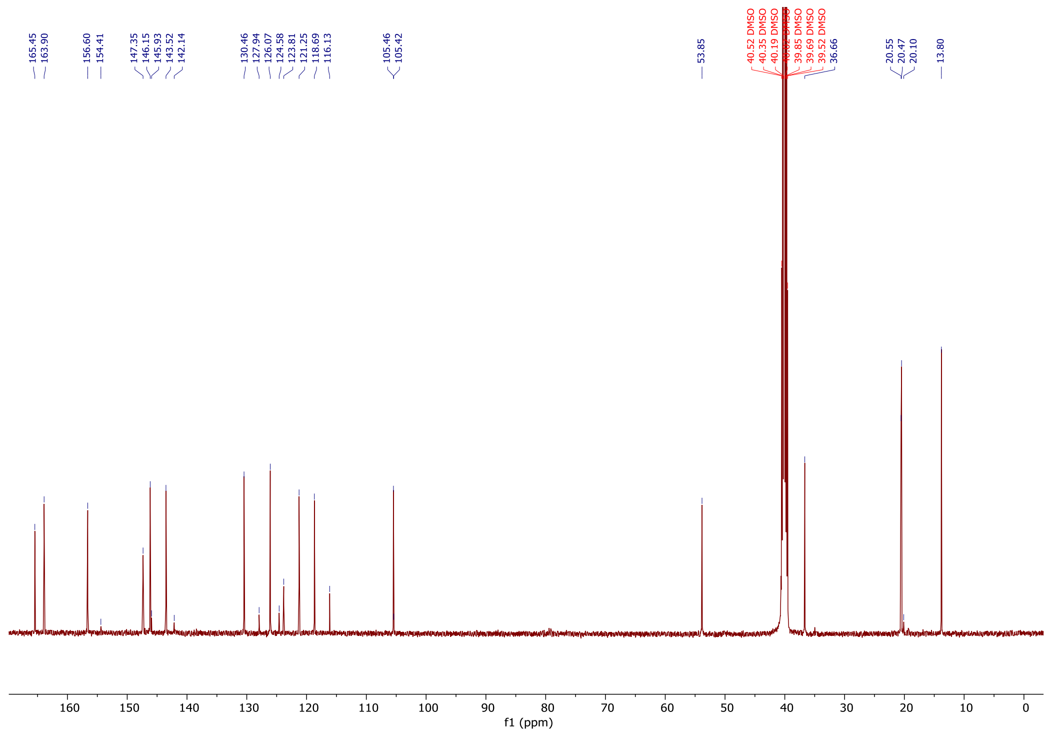

Figure S2. 13C NMR spectra of Amp-NTf₂

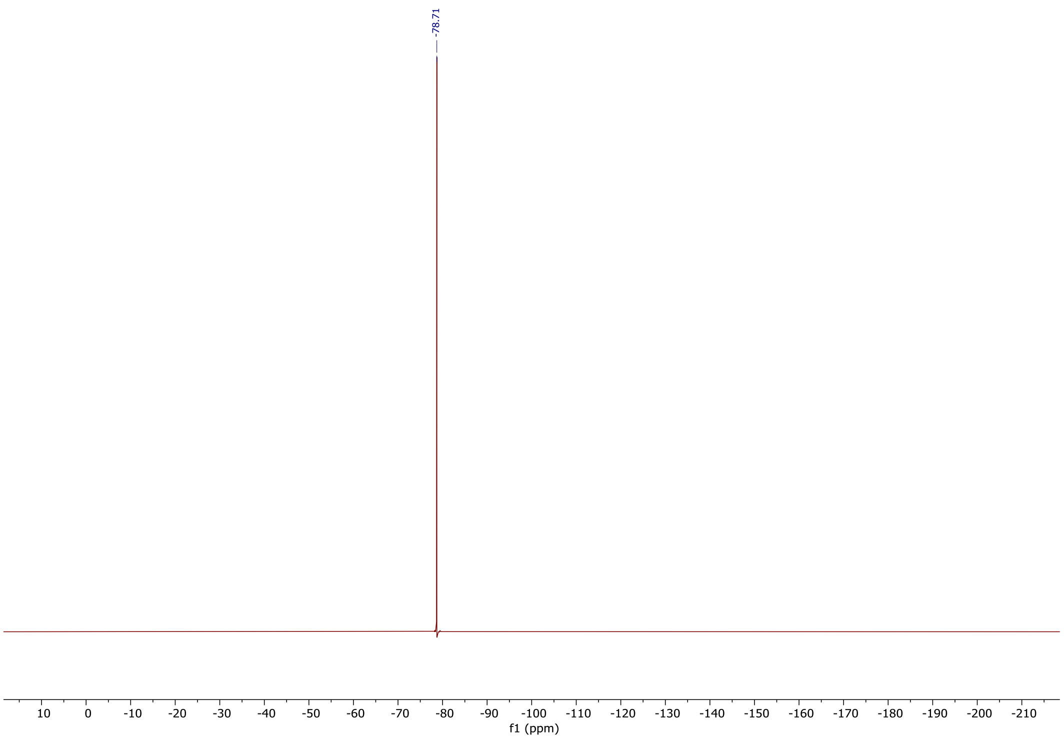


Figure S3. 19F NMR spectra of Amp-$NTf_2$

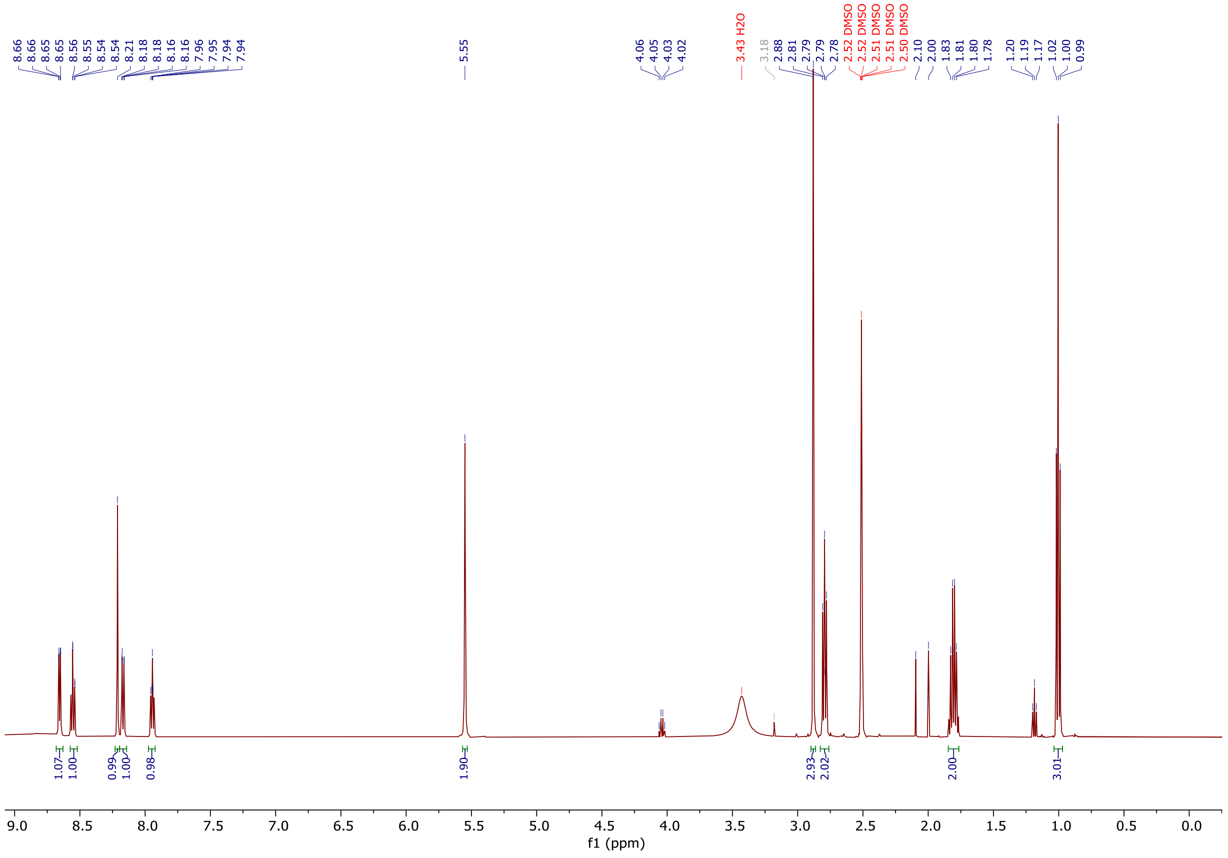


Figure S4. 1H NMR spectra of Amp-BETI

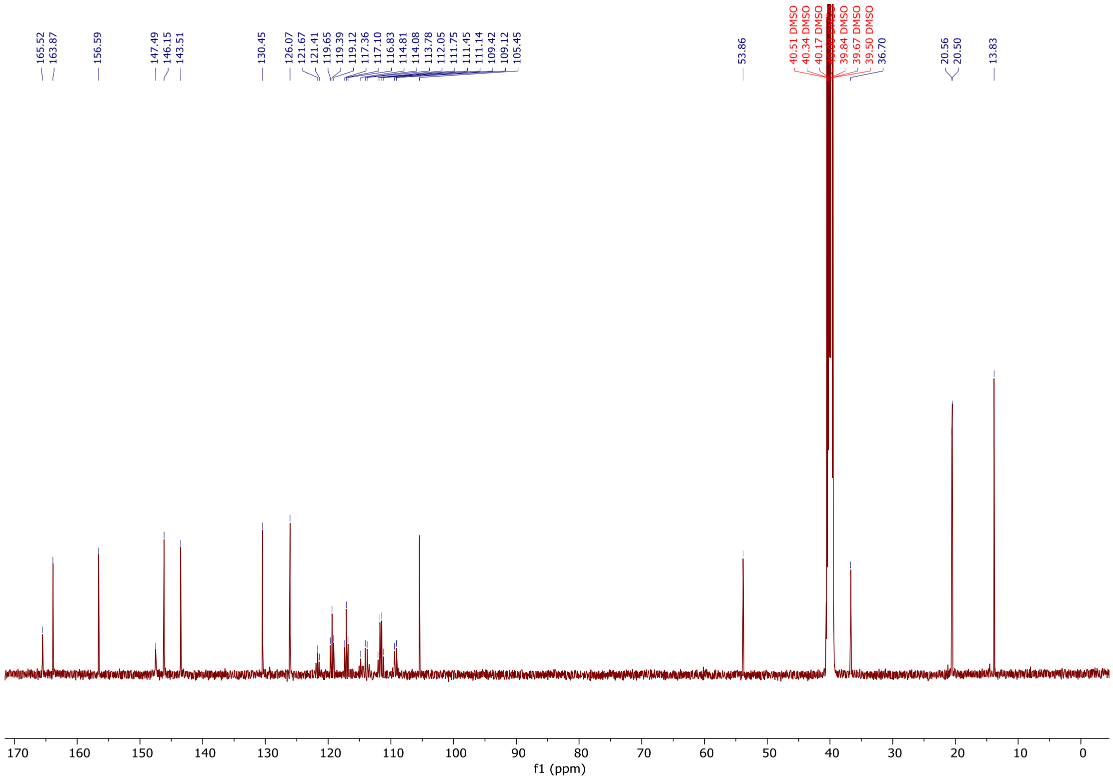


Figure S5. 13C NMR spectra of Amp-BETI

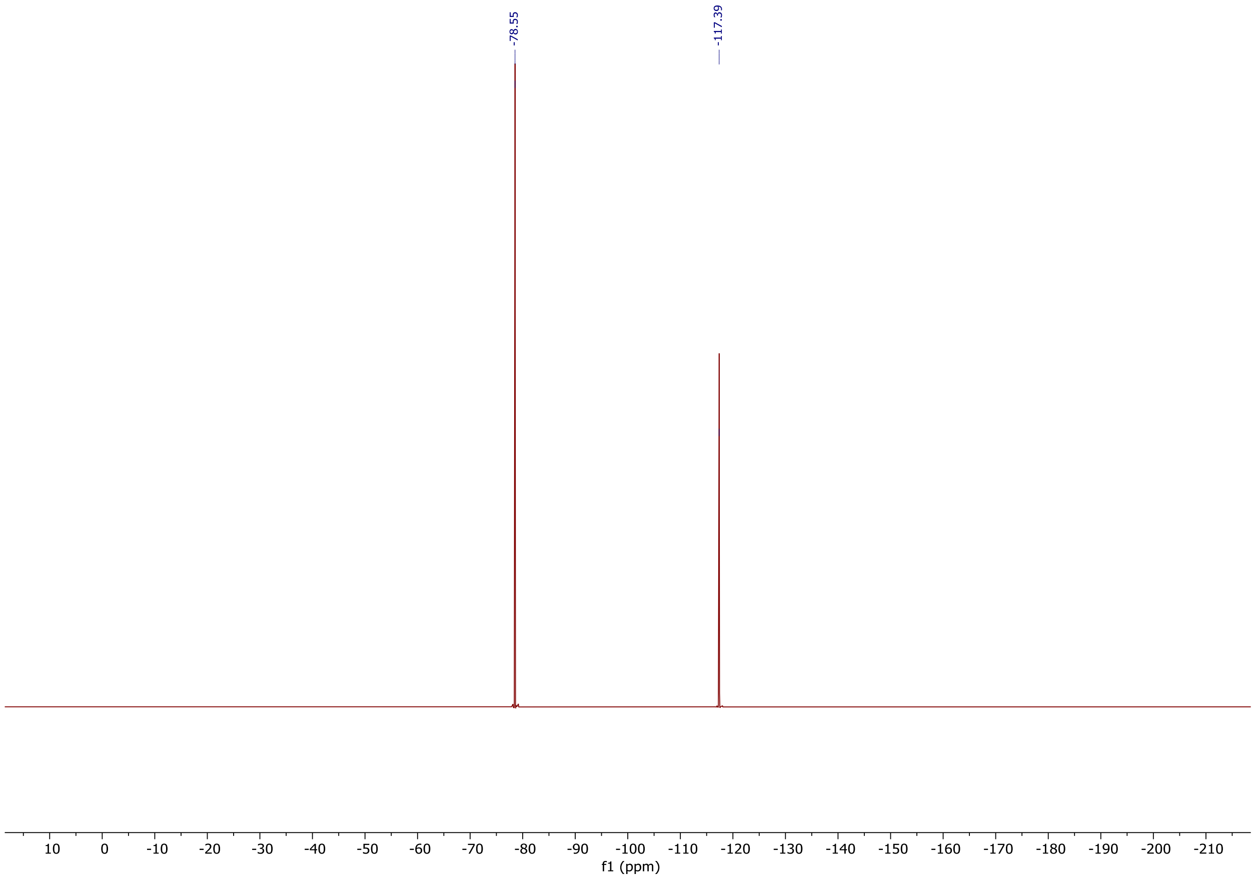


Figure S6. 19F NMR spectra of Amp-BETI

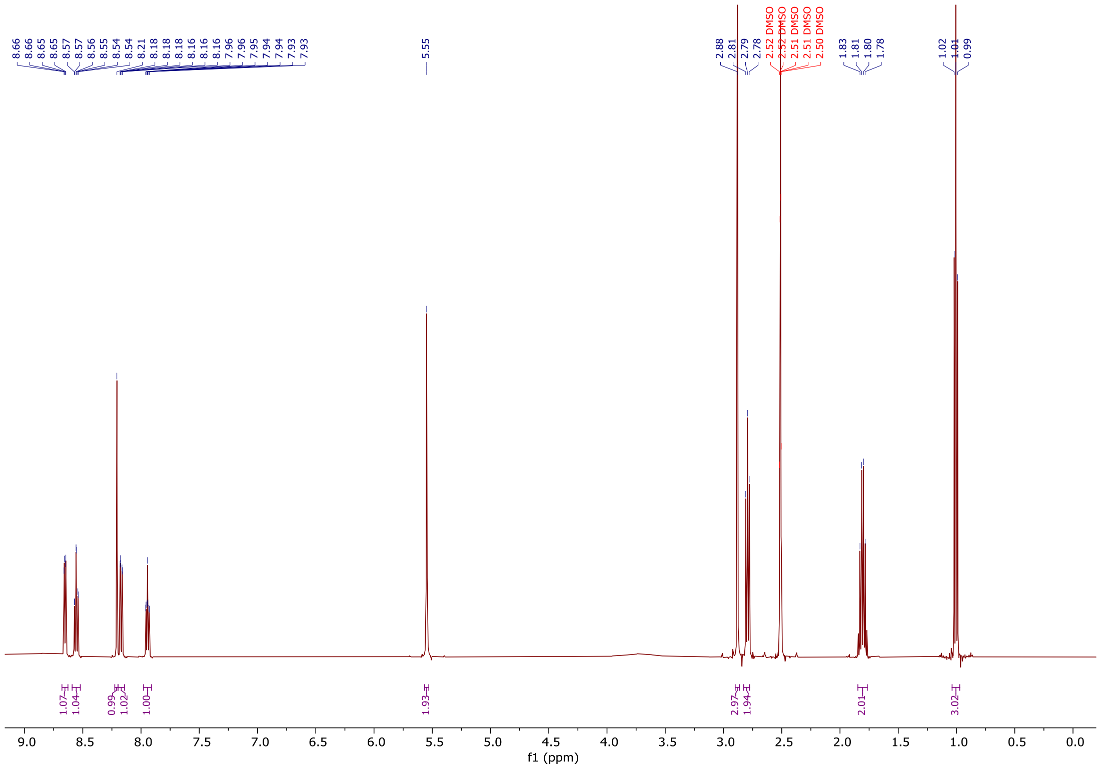

Figure S7. 1H NMR spectra of Amp-NCyF

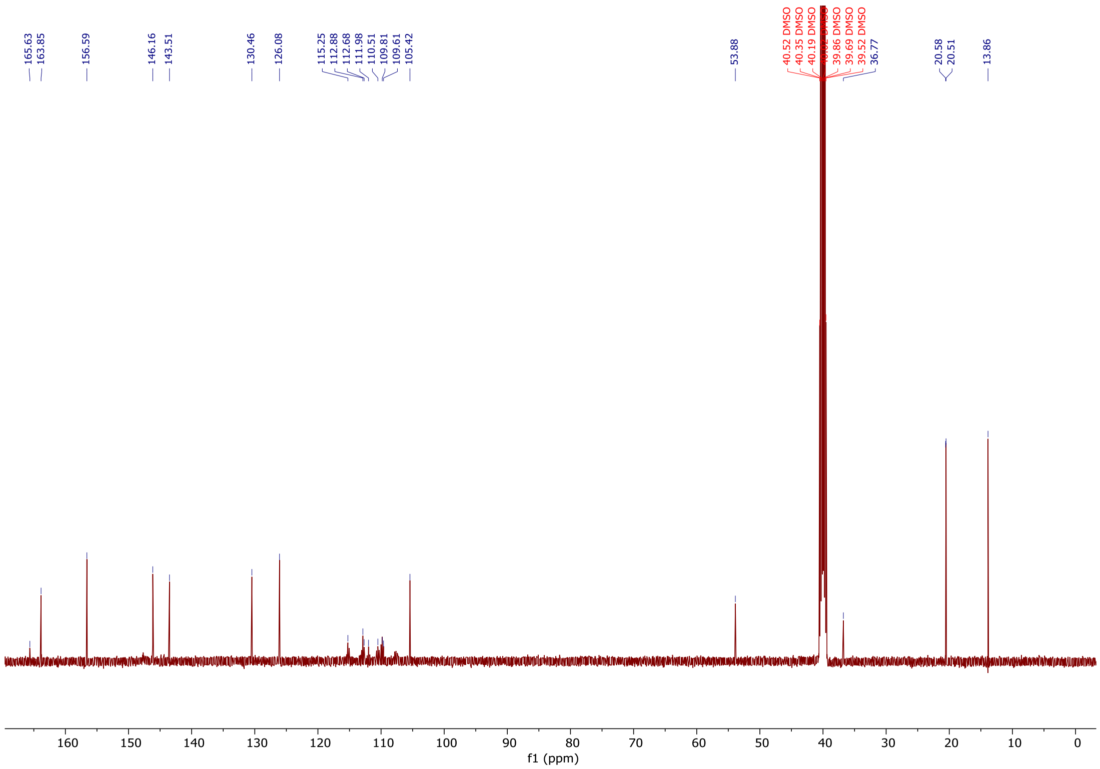

Figure S8. 13C NMR spectra of Amp-NCyF

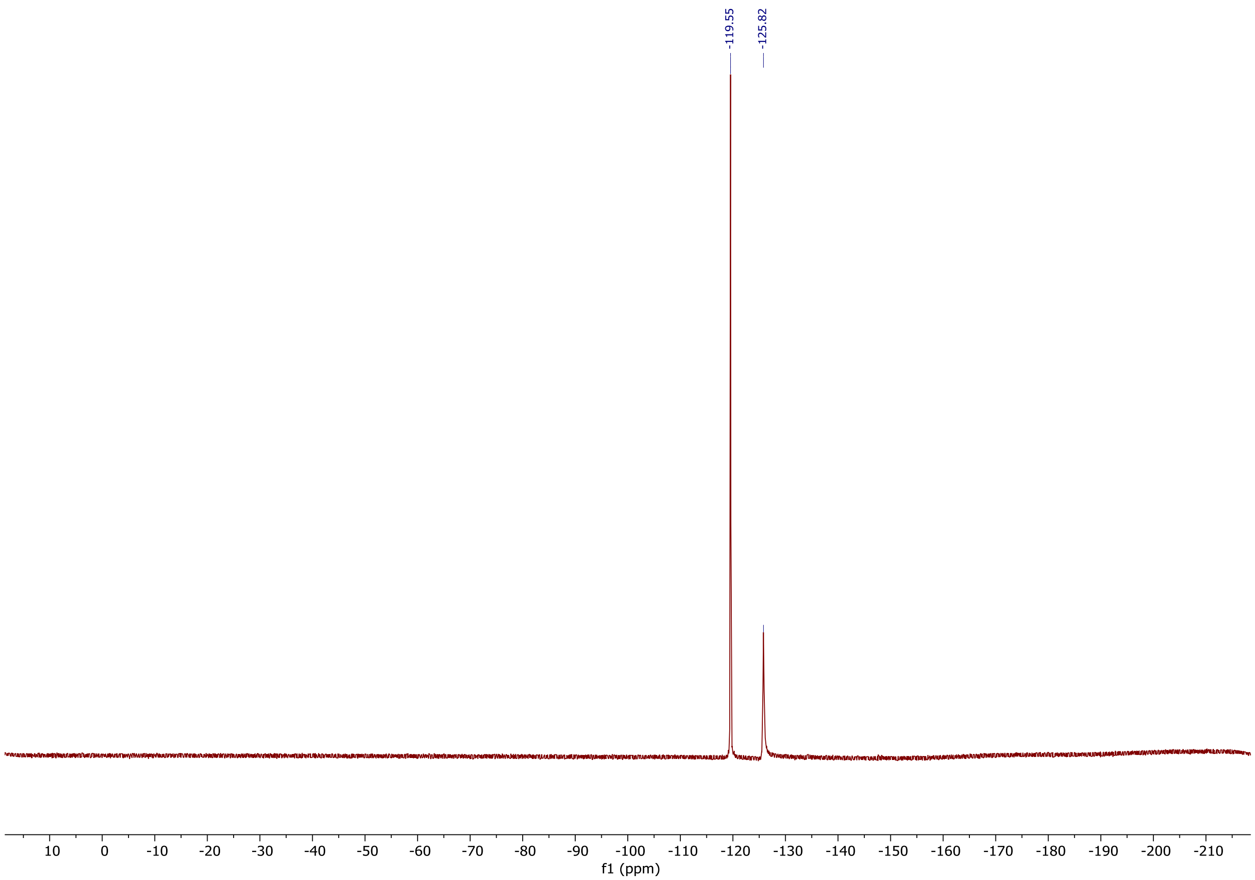


Figure S9. 19F NMR spectra of Amp-NCyF

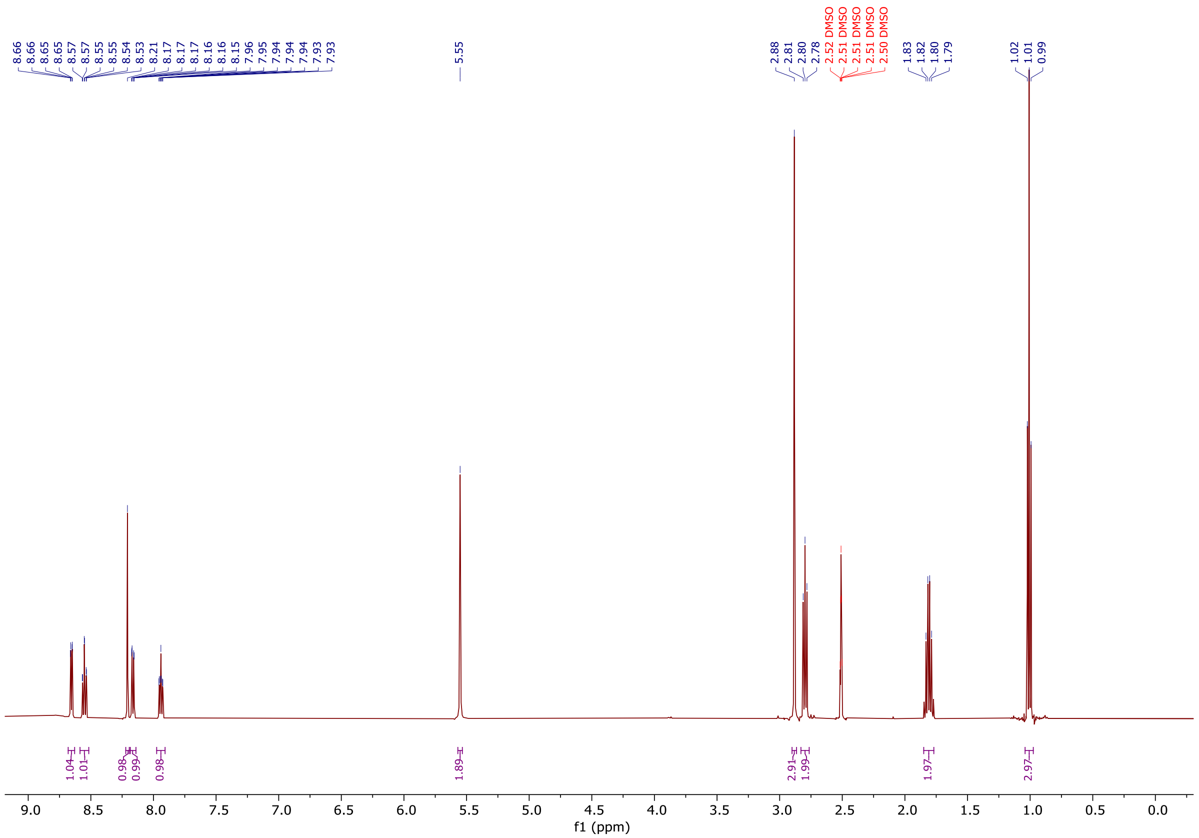

Figure S10. 1H NMR spectra of Amp-FSI

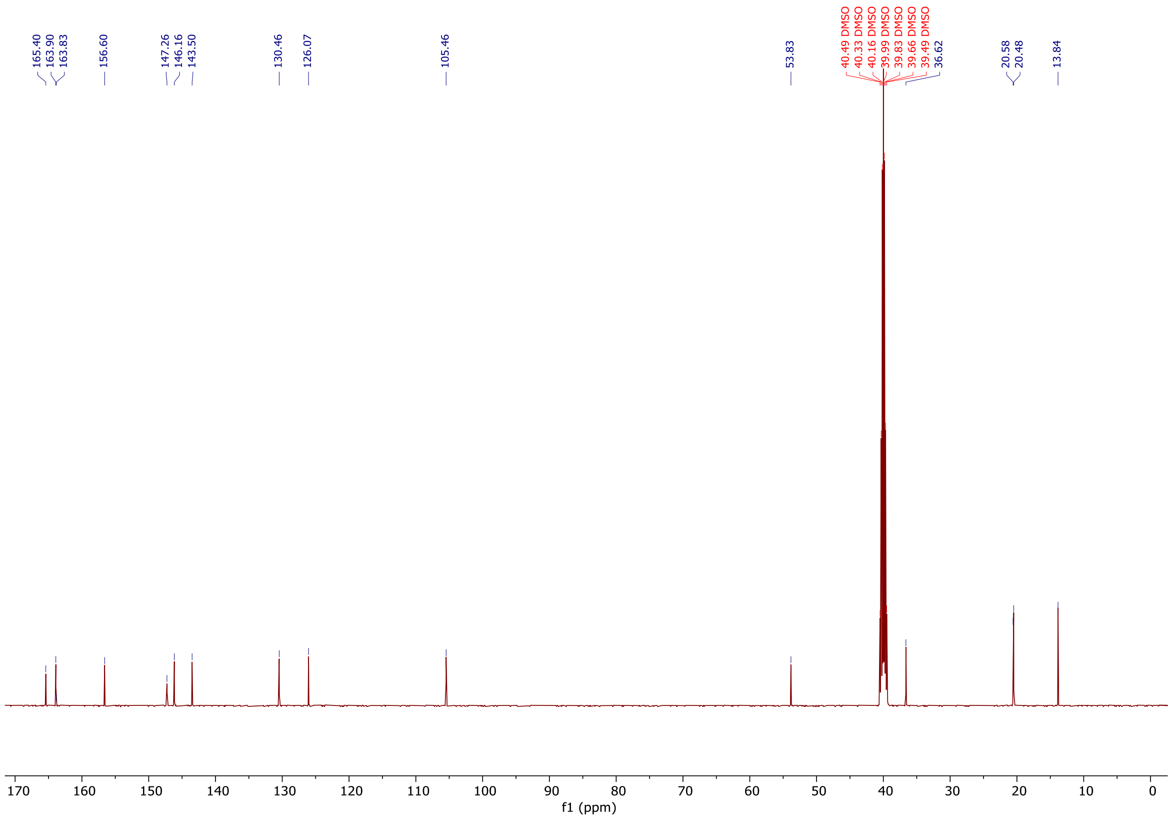

Figure S11. 13C NMR spectra of Amp-FSI

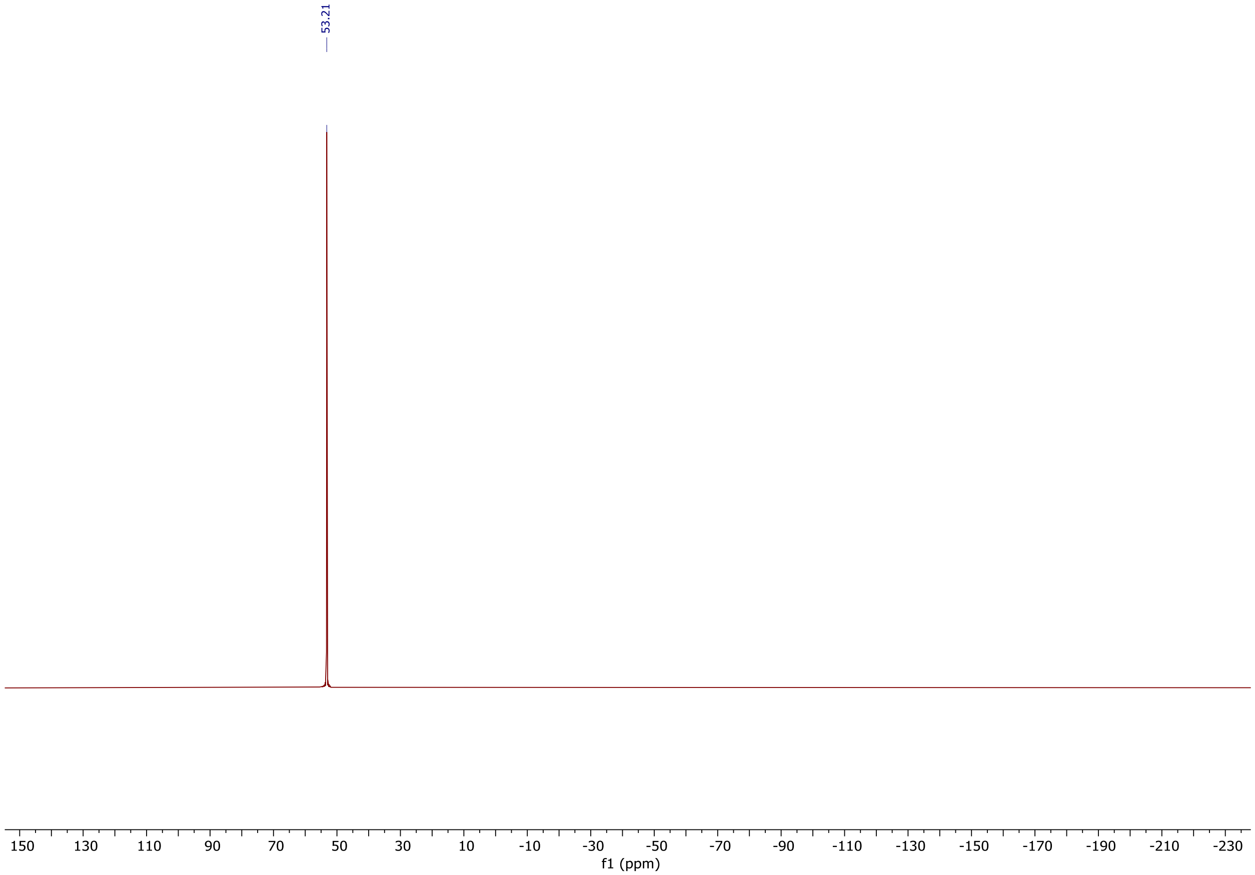


Figure S12. 19F NMR spectra of Amp-FSI

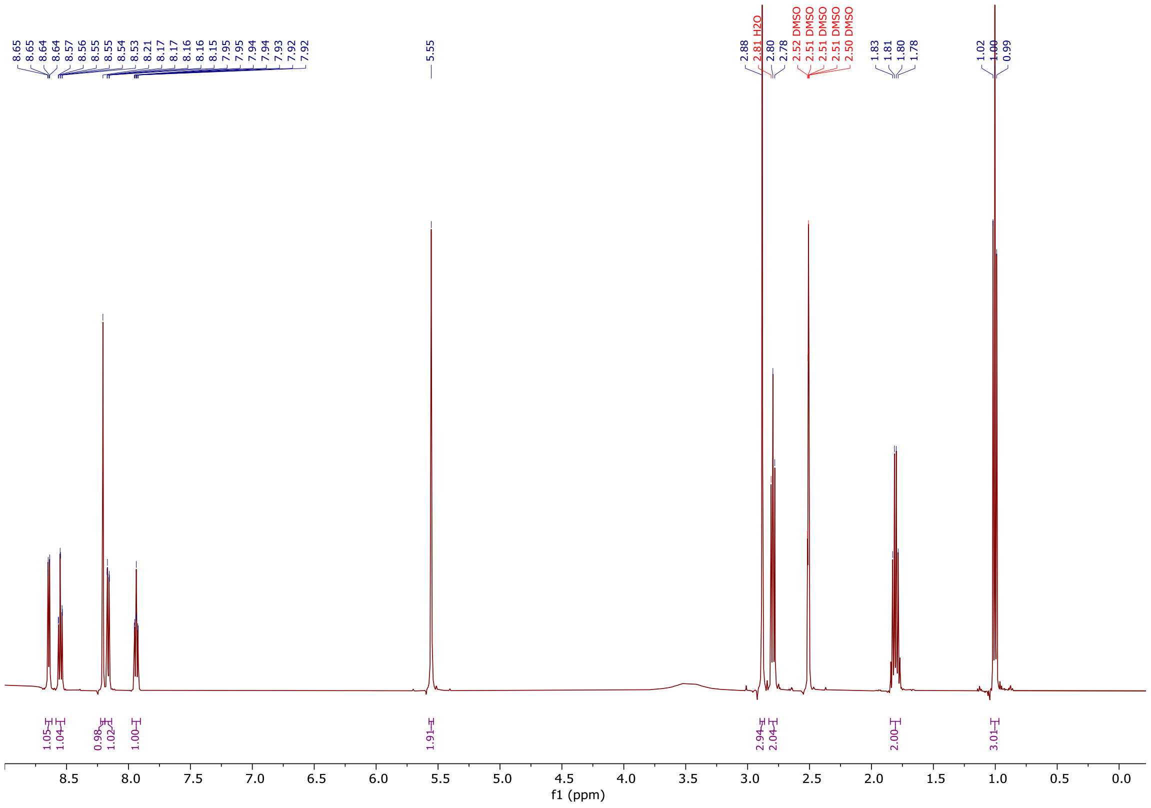


Figure S13. 1H NMR spectra of Amp-BF$_4$

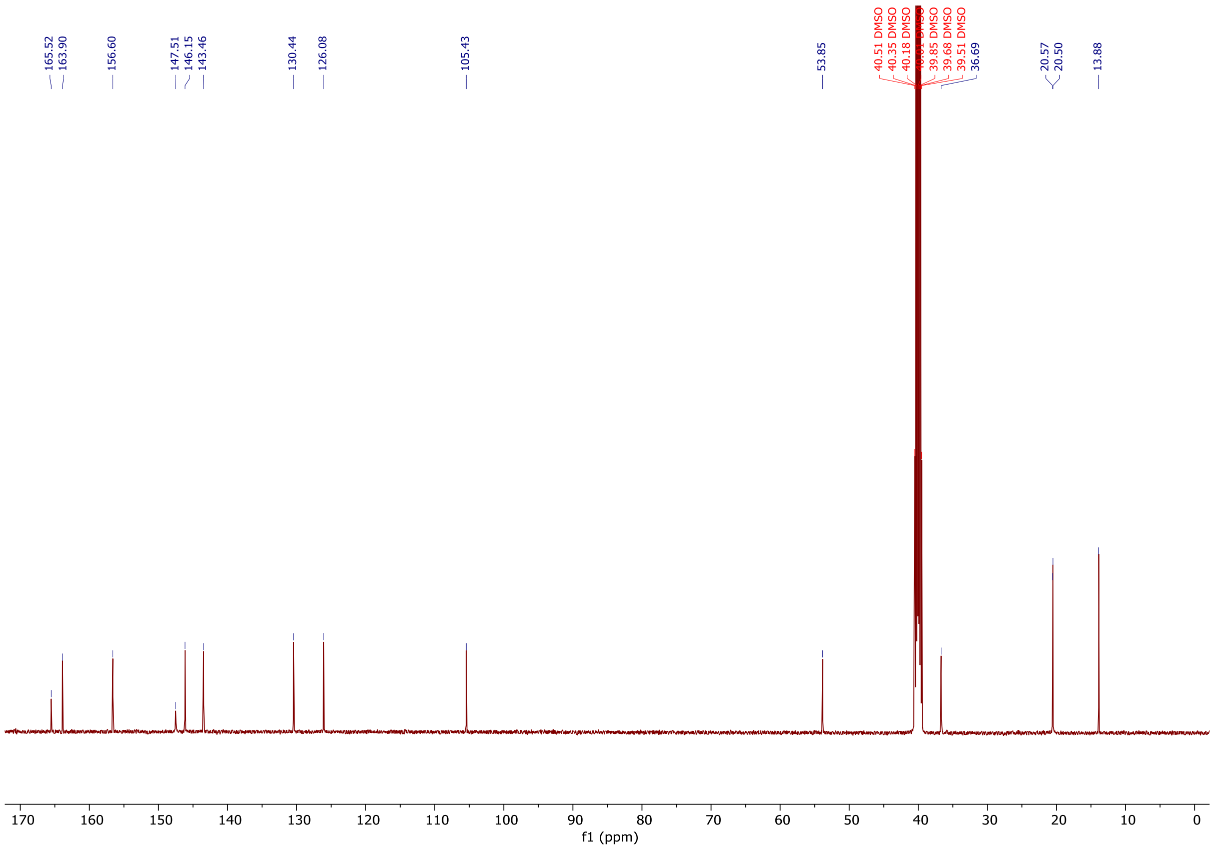


Figure S14. 13C NMR spectra of Amp- BF$_4$

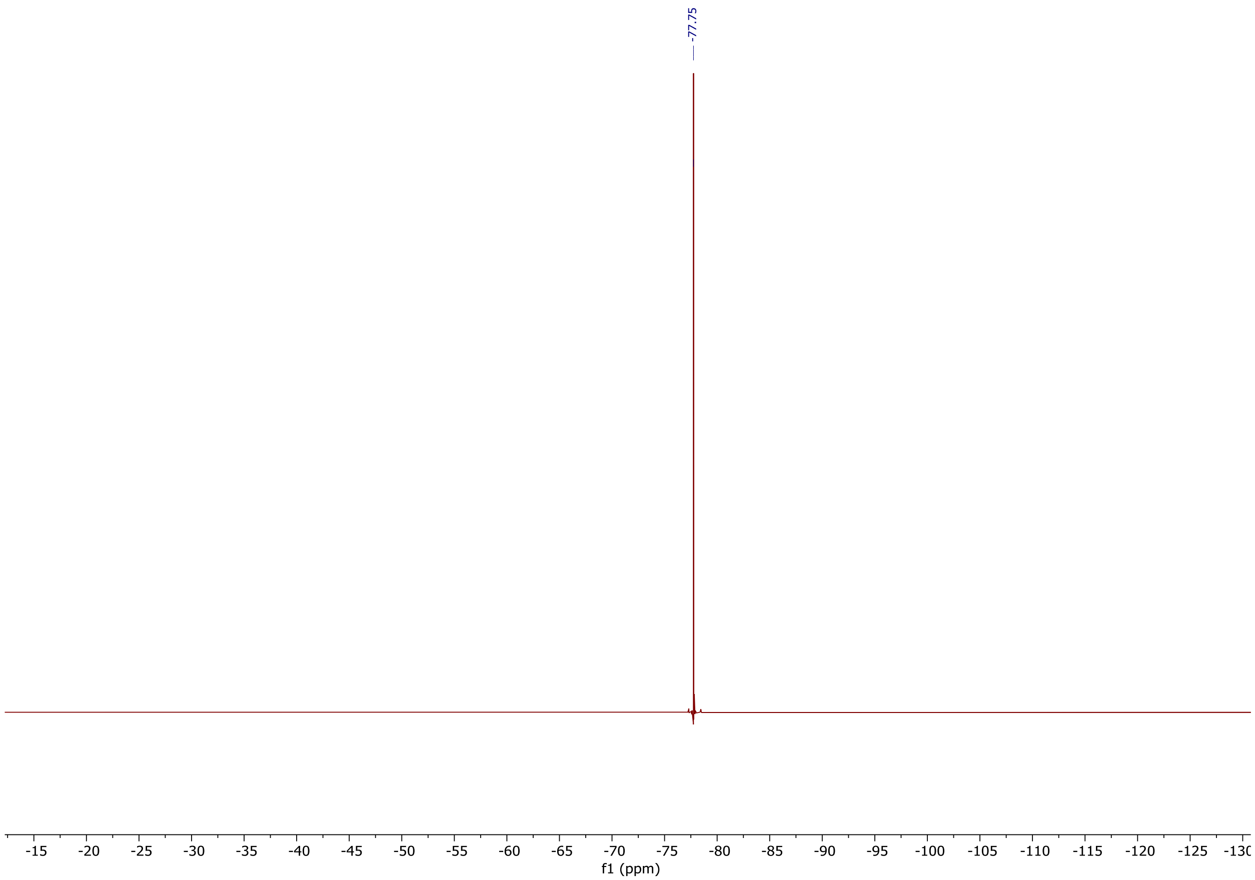


Figure S15. 19F NMR spectra of Amp- $BF_4$

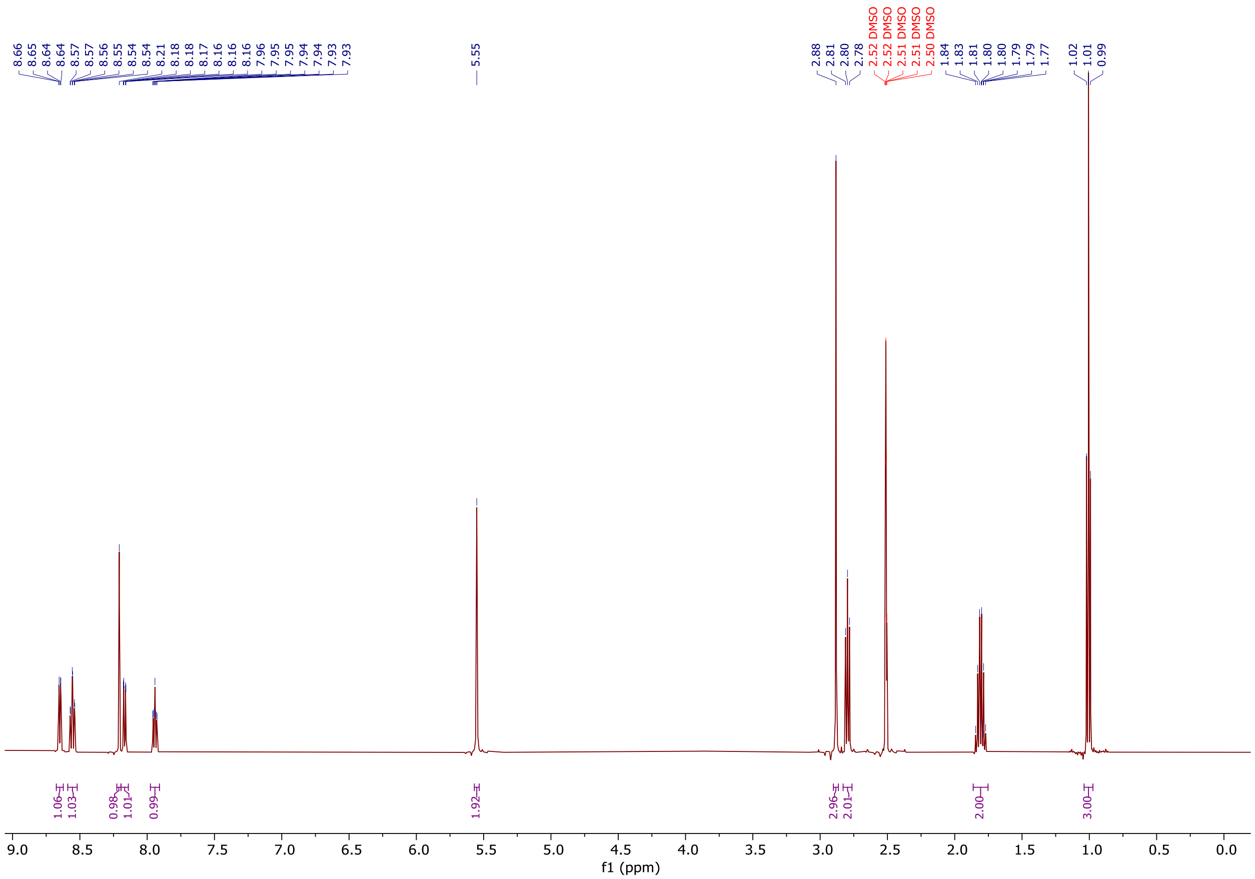


Figure S16. 1H NMR spectra of Amp- PF 6

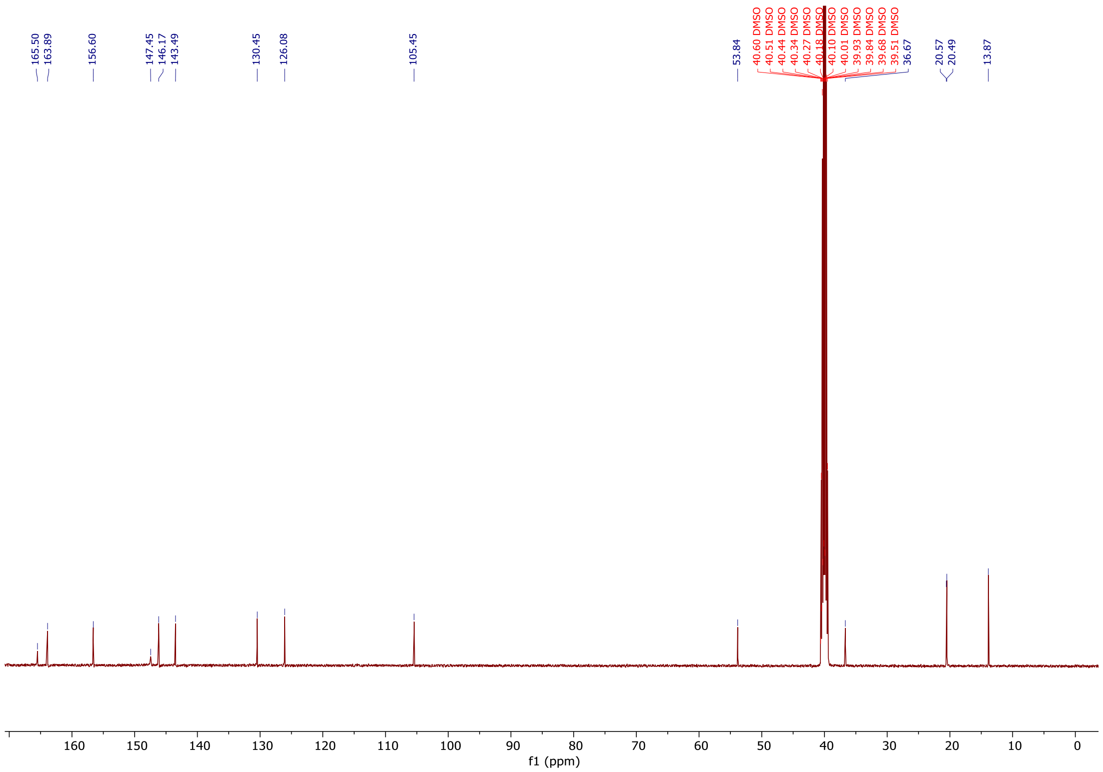


Figure S17. 13C NMR spectra of Amp- PF 6

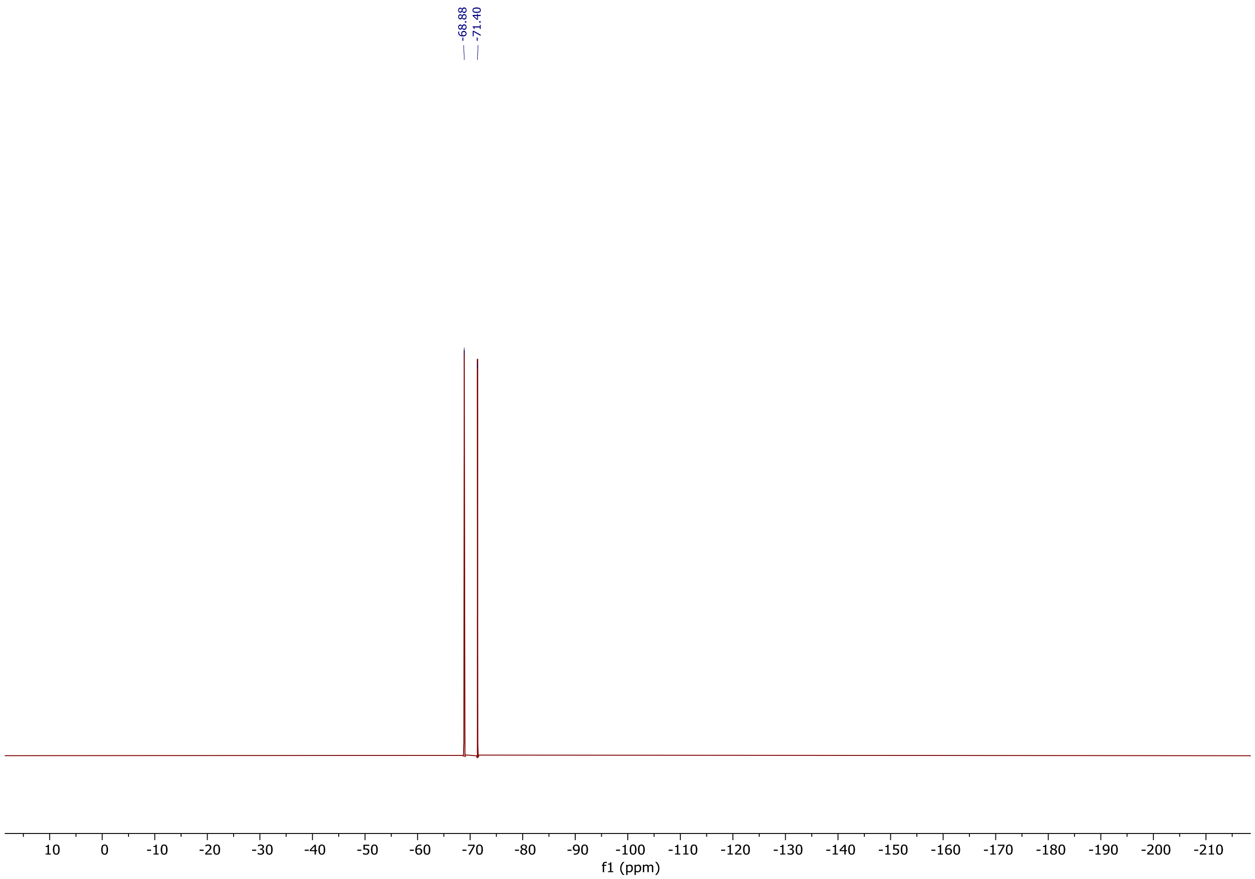


Figure S18. 19F NMR spectra of Amp- $PF_6$

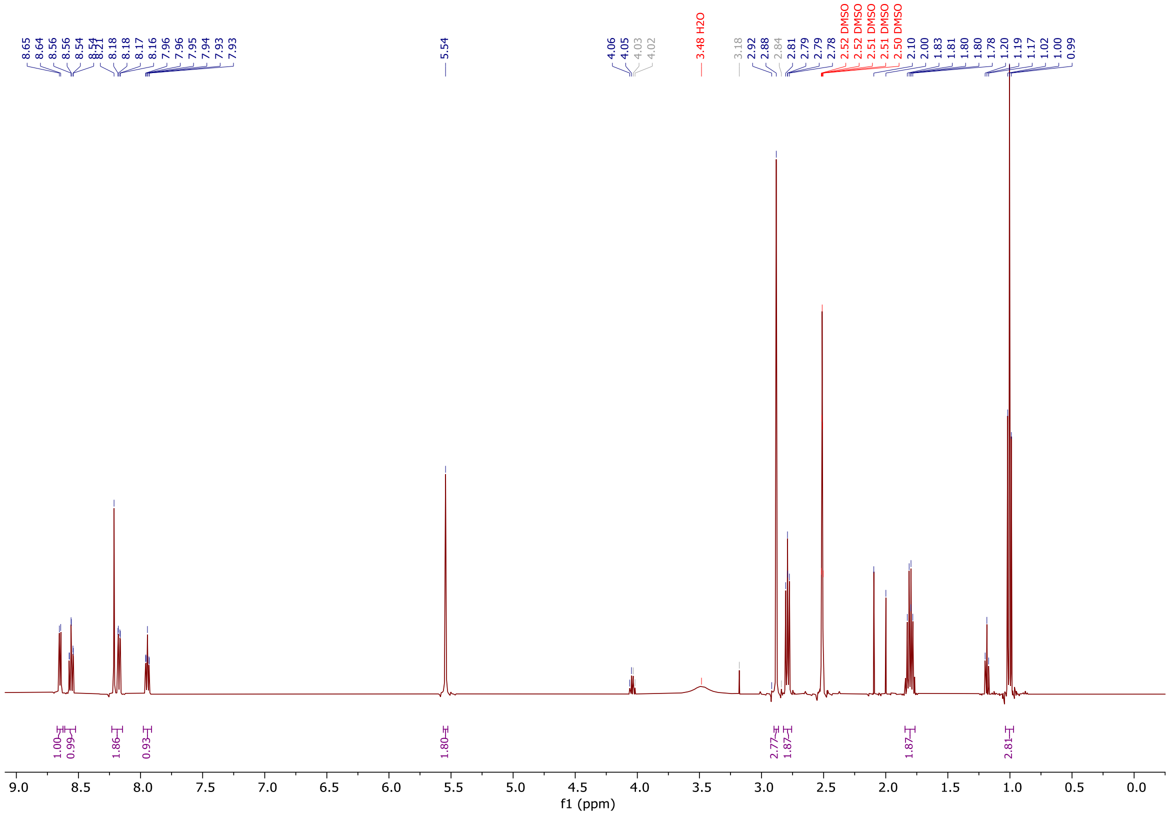


Figure S19. 1H NMR spectra of Amp- OTF

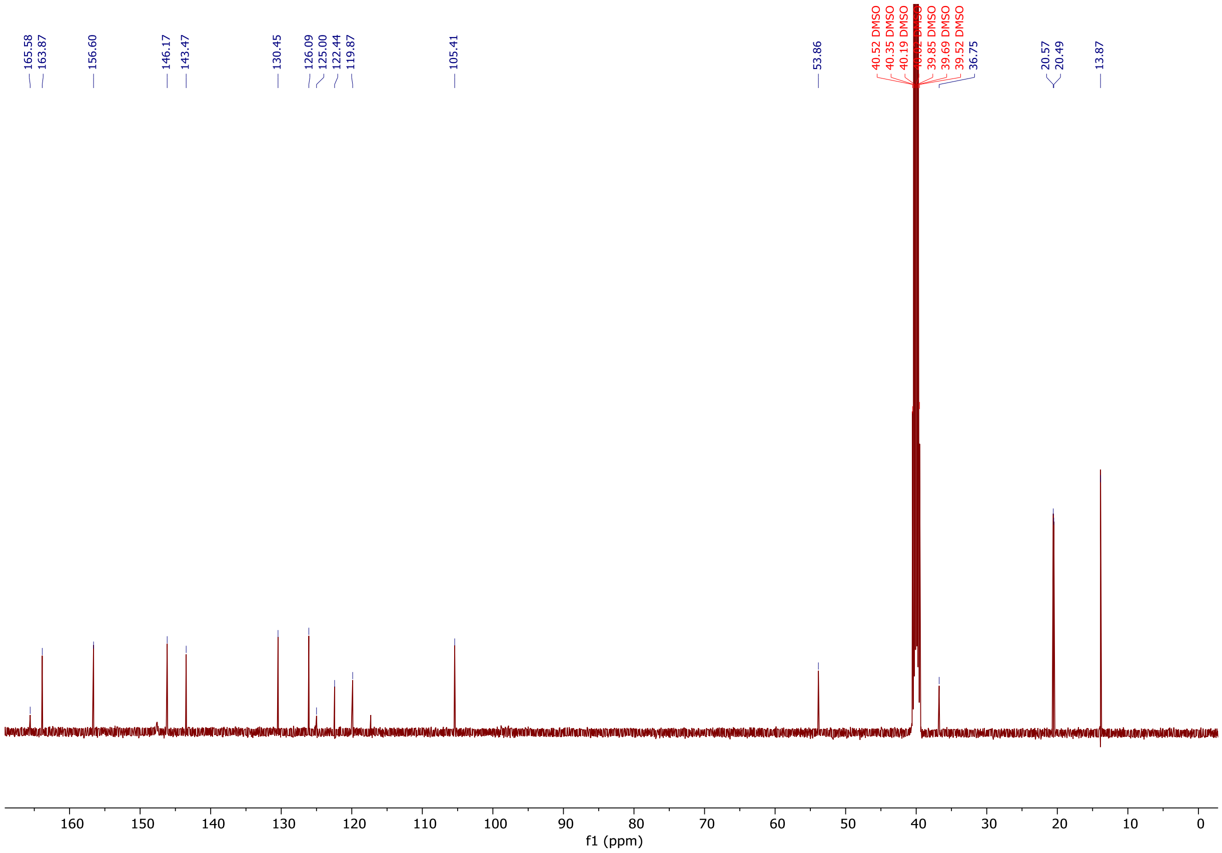


Figure S20. 13C NMR spectra of Amp- OTF

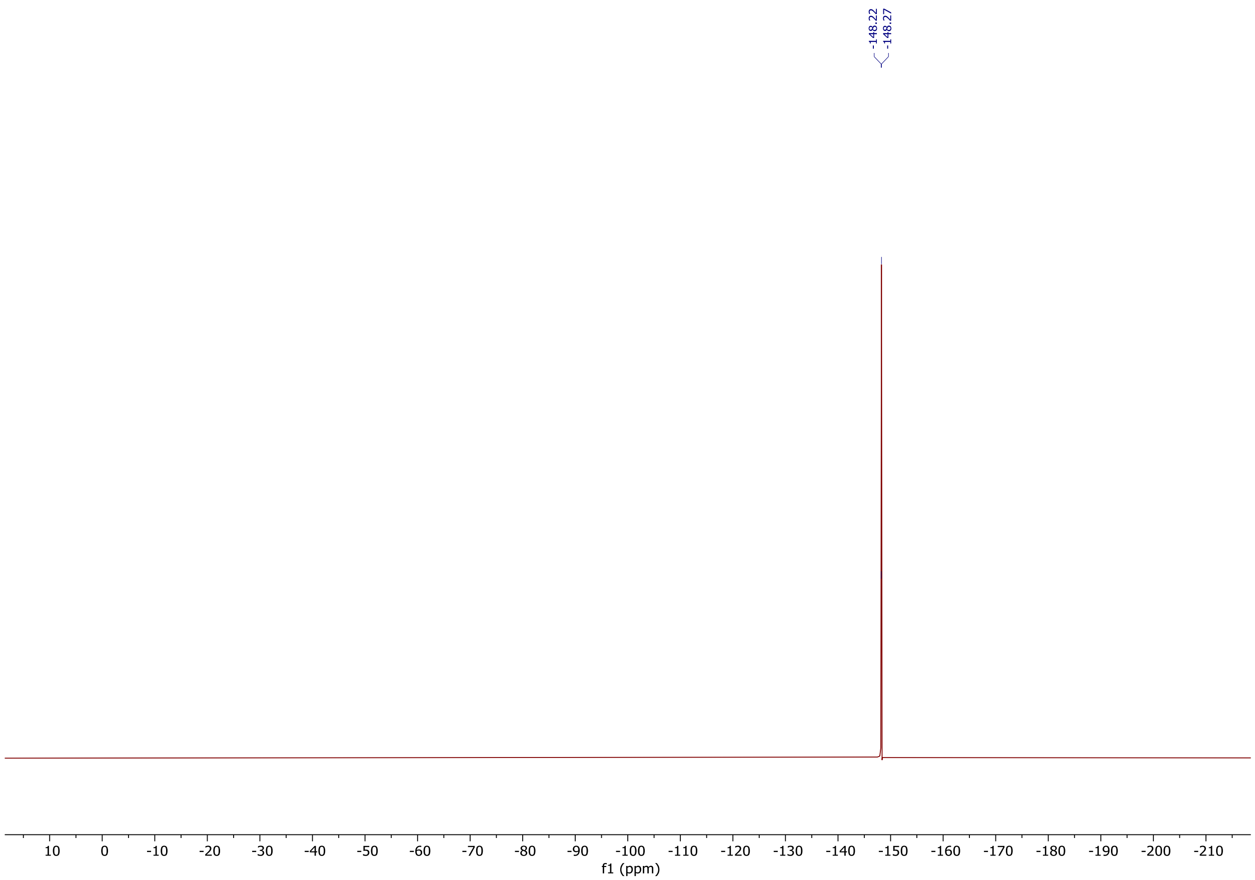


Figure S21. 19F NMR spectra of Amp- OTF

**Simultaneous thermal analysis traces for the amprolium compounds. Thermal decomposition traces shown in red and heat flow shown in blue.**

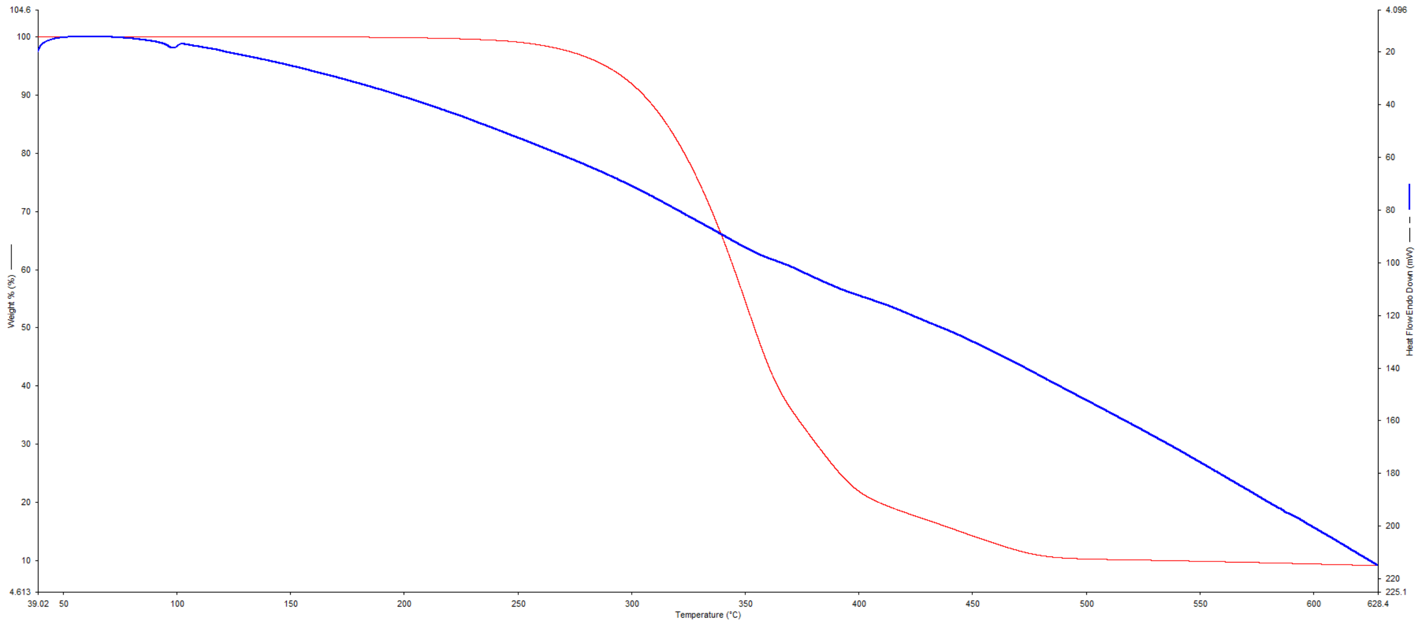


Figure S22. STA trace for Amp-NTf2 under nitrogen.

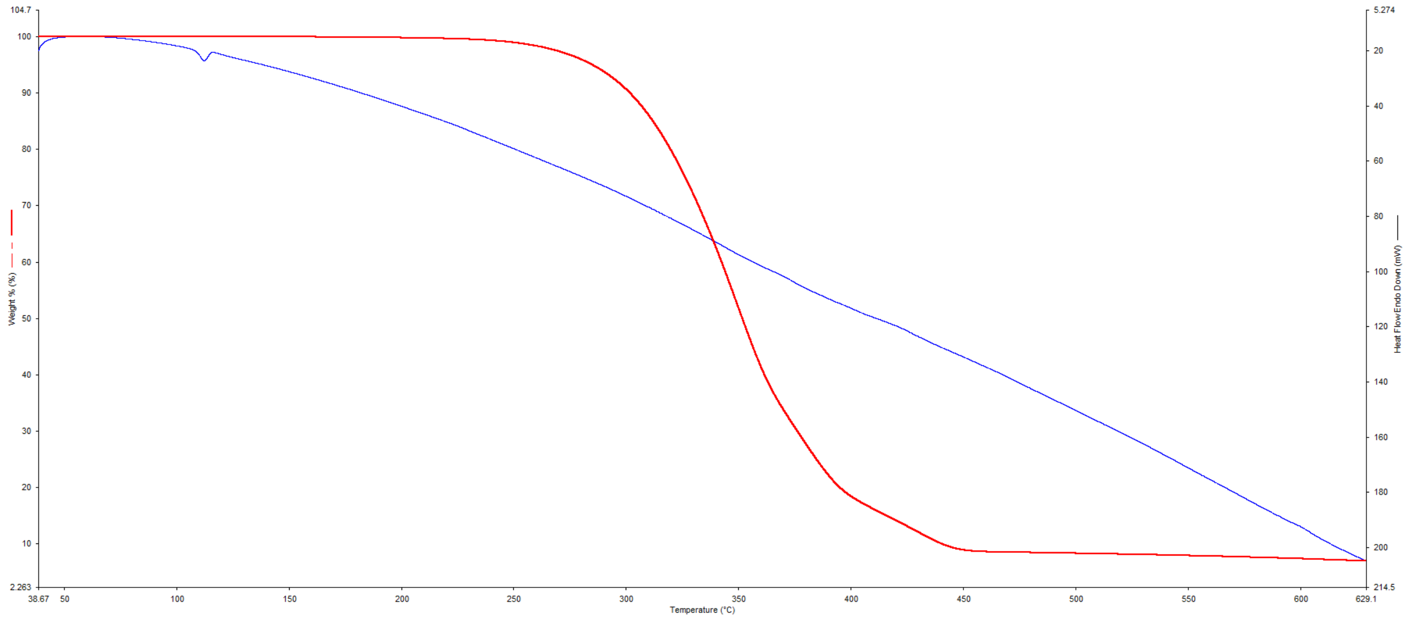


Figure S23. STA trace for Amp-BETI under nitrogen.

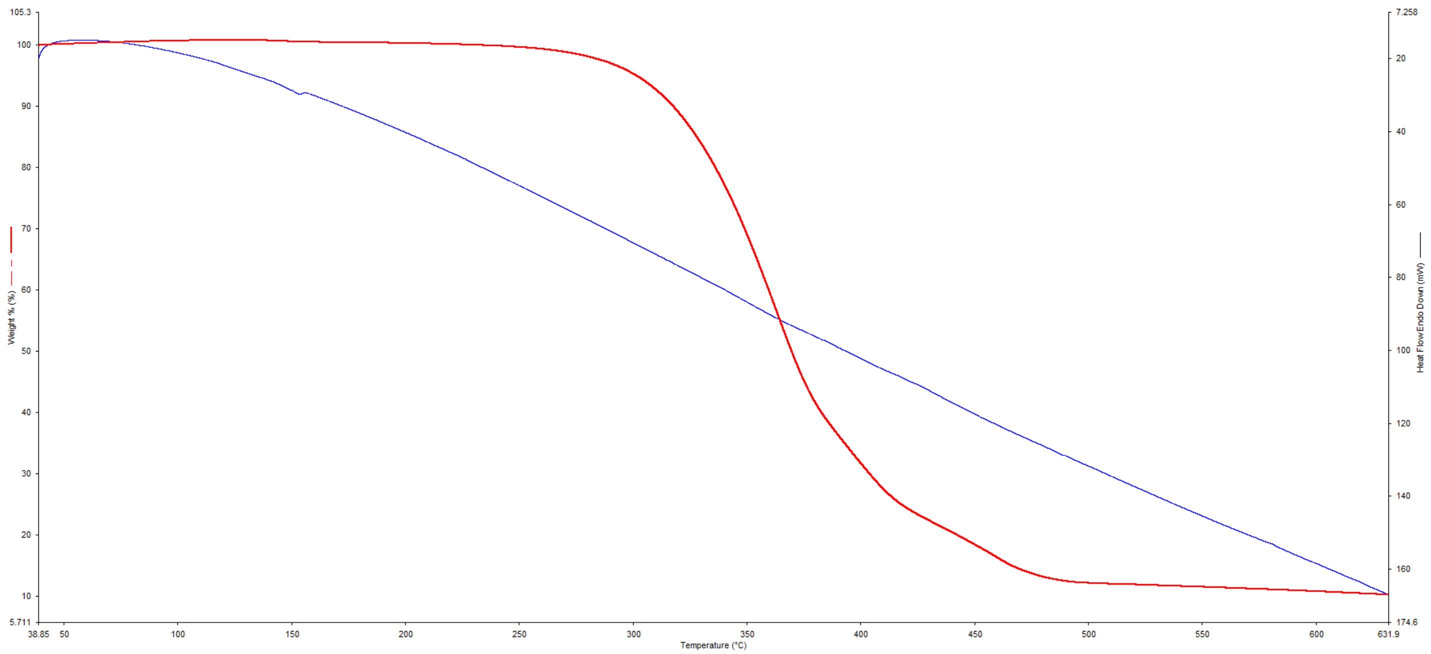


Figure S24. STA trace for Amp-NCyF under nitrogen.

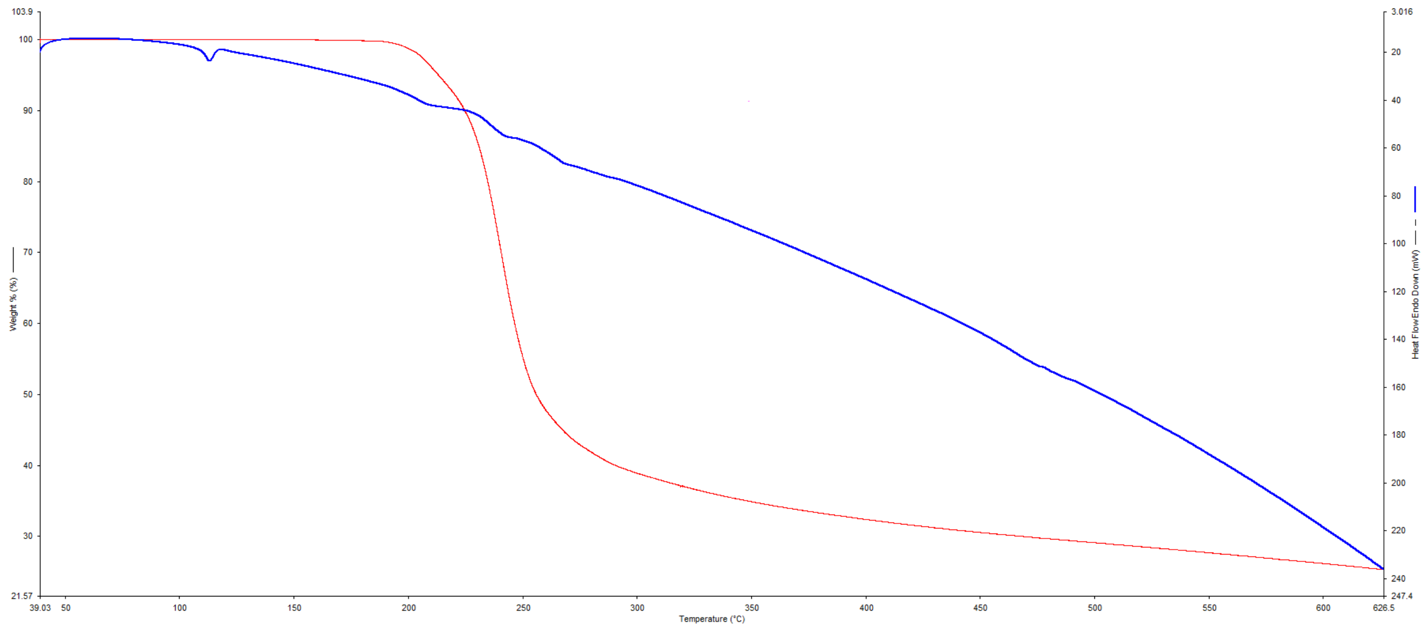


Figure S25. STA trace for Amp-FSI under nitrogen.

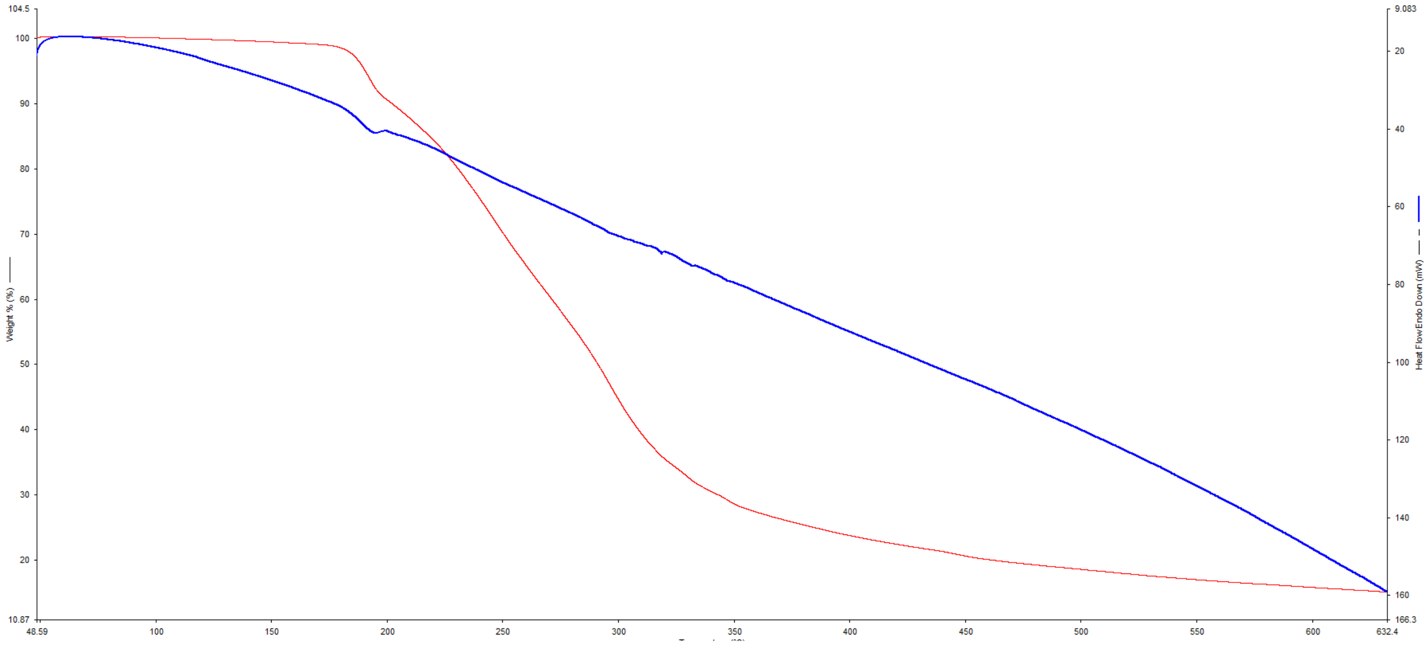


Figure S26. STA trace for Amp-BF4 under nitrogen.

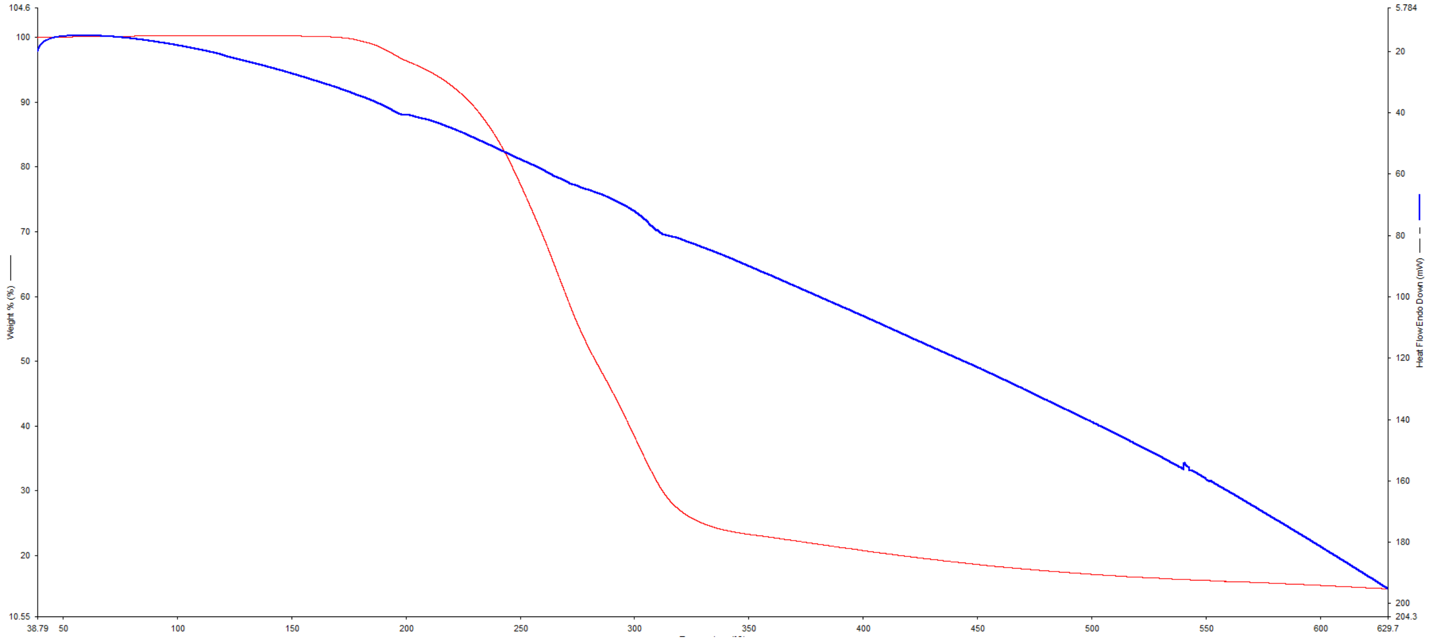


Figure S27. STA trace for Amp-PF6 under nitrogen.

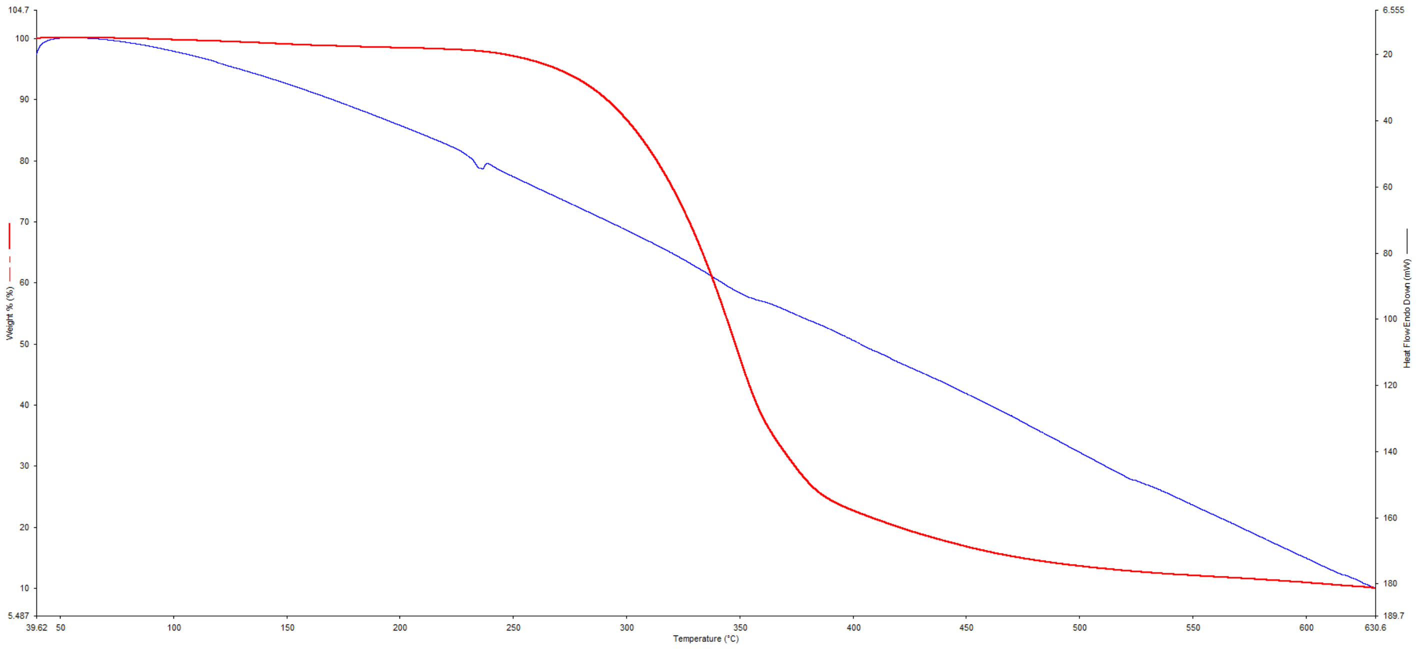


Figure S28. STA trace for Amp-OTF under nitrogen.

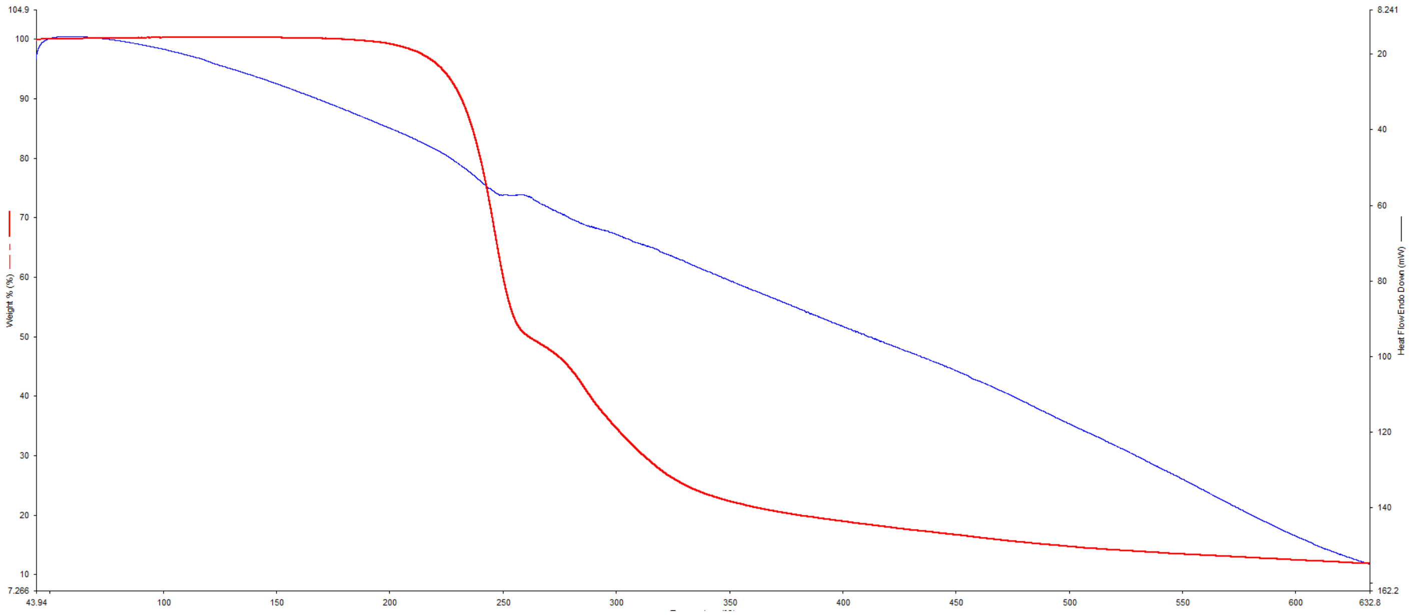


Figure S29. STA trace Amp-Cl under nitrogen.

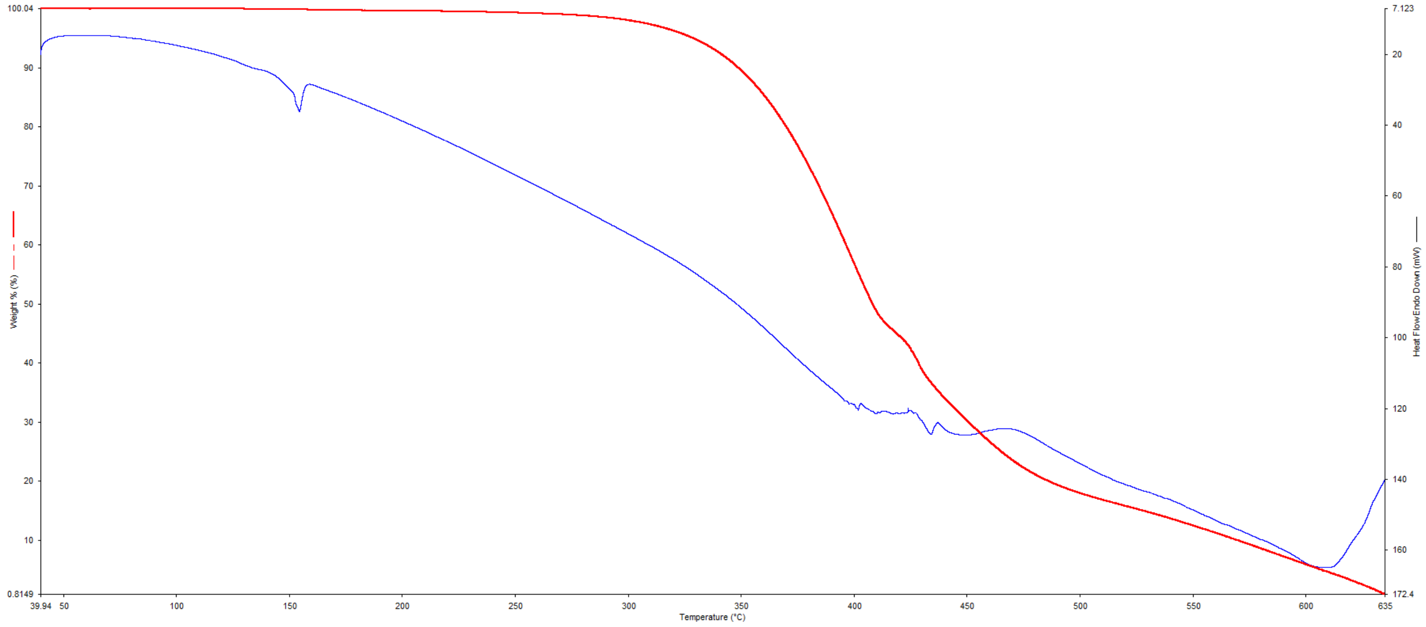


Figure S30. STA trace of Amp-NCyF under air.

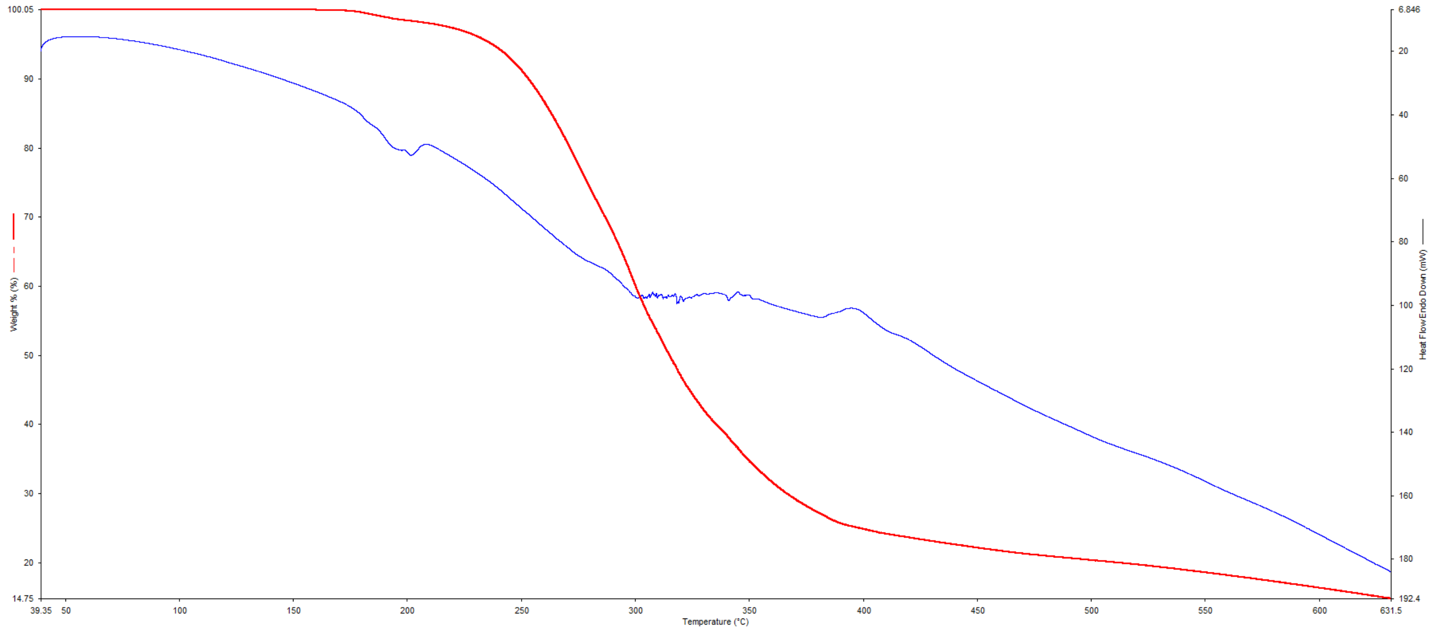


Figure S31. STA trace of Amp-$PF_6$ under air.

```
              SPARTAN'24
        build 1.1.0 (Mar  6 2024)

Wavefunction Developers:
    B.J. Deppmeier, A.J. Driessen, W.J. Hehre, T.S. Hehre,
    J.A. Johnson, W.S. Ohlinger, P.E. Klunzinger

Please cite Spartan as:
    Spartan'24
    Wavefunction Inc.
    Irvine CA


 Q-Chem, Inc., Pleasanton, CA (2023)
Q-Chem Developers:
    Yihan Shao, Zhengting Gan, E. Epifanovsky, A. T. B. Gilbert, M. Wormit,
    J. Kussmann, A. W. Lange, A. Behn, Jia Deng, Xintian Feng, D. Ghosh,
    M. Goldey, P. R. Horn, L. D. Jacobson, I. Kaliman, T. Kus, A. Landau, Jie Liu,
    E. I. Proynov, R. M. Richard, R. P. Steele, E. J. Sundstrom,
    H. L. Woodcock III, P. M. Zimmerman, D. Zuev, B. Alam, B. Albrecht,
    E. Alguire, S. A. Baeppler, D. Barton, Z. Benda, Y. A. Bernard,
    E. J. Berquist, K. B. Bravaya, H. Burton, K. Carter-Fenk, D. Casanova,
    Chun-Min Chang, Yunqing Chen, A. Chien, K. D. Closser, M. P. Coons,
    S. Coriani, S. Dasgupta, A. L. Dempwolff, M. Diedenhofen, Hainam Do,
    R. G. Edgar, Po-Tung Fang, S. Faraji, S. Fatehi, Qingguo Feng, J. Fosso-Tande,
    J. Gayvert, Qinghui Ge, A. Ghysels, G. Gidofalvi, J. Gomes, J. Gonthier,
    A. Gunina, D. Hait, M. W. D. Hanson-Heine, P. H. P. Harbach, A. W. Hauser,
    M. F. Herbst, J. E. Herr, E. G. Hohenstein, Z. C. Holden, Kerwin Hui,
    B. C. Huynh, T.-C. Jagau, Hyunjun Ji, B. Kaduk, K. Khistyaev, Jaehoon Kim,
    P. Klunzinger, K. Koh, D. Kosenkov, L. Koulias, T. Kowalczyk, C. M. Krauter,
    A. Kunitsa, Ka Un Lao, A. Laurent, K. V. Lawler, Joonho Lee, D. Lefrancois,
    S. Lehtola, D. S. Levine, Yi-Pei Li, You-Sheng Lin, Fenglai Liu, Kuan-Yu Liu,
    E. Livshits, M. Loipersberger, A. Luenser, P. Manohar, E. Mansoor,
    S. F. Manzer, Shan-Ping Mao, Yuezhi Mao, N. Mardirossian, A. V. Marenich,
    T. Markovich, L. A. Martinez-Martinez, S. A. Maurer, N. J. Mayhall,
    S. C. McKenzie, J.-M. Mewes, P. Morgante, A. F. Morrison, J. W. Mullinax,
    K. Nanda, T. S. Nguyen-Beck, R. Olivares-Amaya, J. A. Parkhill, S. K. Paul,
    Zheng Pei, T. M. Perrine, F. Plasser, P. Pokhilko, S. Prager, A. Prociuk,
    E. Ramos, B. Rana, D. R. Rehn, F. Rob, M. Scheurer, M. Schneider, N. Sergueev,
    S. M. Sharada, S. Sharma, D. W. Small, T. Stauch, C. J. Stein, T. Stein,
    Yu-Chuan Su, S. P. Veccham, A. J. W. Thom, A. Tkatchenko, T. Tsuchimochi,
    N. M. Tubman, L. Vogt, M. L. Vidal, O. Vydrov, M. A. Watson, J. Wenzel,
    M. de Wergifosse, T. A. Wesolowski, A. White, J. Witte, A. Yamada, Jun Yang,
    K. Yao, S. Yeganeh, S. R. Yost, Zhi-Qiang You, A. Zech, Igor Ying Zhang,
    Xing Zhang, Yan Zhao, Ying Zhu, B. R. Brooks, G. K. L. Chan, C. J. Cramer,
    M. S. Gordon, W. J. Hehre, A. Klamt, M. W. Schmidt, C. D. Sherrill,
    D. G. Truhlar, A. Aspuru-Guzik, R. Baer, A. T. Bell, N. A. Besley,
    Jeng-Da Chai, A. E. DePrince, III, R. A. DiStasio Jr., A. Dreuw,
    B. D. Dunietz, T. R. Furlani, Chao-Ping Hsu, Yousung Jung, Jing Kong,
    D. S. Lambrecht, WanZhen Liang, C. Ochsenfeld, V. A. Rassolov,
    L. V. Slipchenko, J. E. Subotnik, T. Van Voorhis, J. M. Herbert, A. I. Krylov,
    P. M. W. Gill, M. Head-Gordon,


Contributors to earlier versions of Q-Chem not listed above:
    R. D. Adamson, B. Austin, J. Baker, G. J. O. Beran, K. Brandhorst,
    S. T. Brown, E. F. C. Byrd, A. K. Chakraborty, C.-L. Cheng, Siu Hung Chien,
    D. M. Chipman, D. L. Crittenden, H. Dachsel, R. J. Doerksen, A. D. Dutoi,
    L. Fusti-Molnar, W. A. Goddard III, A. Golubeva-Zadorozhnaya, S. R. Gwaltney,
    G. Hawkins, A. Heyden, S. Hirata, G. Kedziora, F. J. Keil, C. Kelley,
    Jihan Kim, R. A. King, R. Z. Khaliullin, P. P. Korambath, W. Kurlancheek,
    A. M. Lee, M. S. Lee, S. V. Levchenko, Ching Yeh Lin, D. Liotard,
    R. C. Lochan, I. Lotan, P. E. Maslen, N. Nair, D. P. O'Neill, D. Neuhauser,
    E. Neuscamman, C. M. Oana, R. Olson, B. Peters, R. Peverati, P. A. Pieniazek,
    Y. M. Rhee, J. Ritchie, M. A. Rohrdanz, E. Rosta, N. J. Russ,
    H. F. Schaefer III, N. E. Schultz, N. Shenvi, A. C. Simmonett, A. Sodt,
    D. Stuck, K. S. Thanthiriwatte, V. Vanovschi, Tao Wang, A. Warshel,
    C. F. Williams, Q. Wu, X. Xu, W. Zhang,
```

```
 Please cite Q-Chem as follows :
     E. Epifanovsky et al., J. Chem. Phys. 155, 084801 (2021)
     https://doi.org/10.1063/5.0055522


 Parts of Q-Chem use Armadillo 8.300.2 (tropical Shenanigans).
 http://arma.sourceforge.net/


 Please cite Spooky-Net as:
     Unke, O. T., Chmiela, S., Gastegger, M., Schutt, K. T., Sauceda,
     H. E., & Muller, K. R.  (2021).
     Spookynet: Learning force fields with electronic degrees of freedom
     and nonlocal effects. Nat. Commun. 12(1), 2021, 1-14.

 Wavefunction Inc.            Sales:   sales@wavefun.com
 Irvine CA                    Support: support@wavefun.com
                              Web:     www.wavefun.com

Copyright © 1995 - 2024
----------------------------------------------------

Wavefunction Version of Q-Chem

 Parts of Q-Chem use Armadillo 8.300.2 (Tropical Shenanigans).
 http://arma.sourceforge.net/

 Q-Chem begins on Thu Nov 14 11:34:13 2024


     Scratch files written to
C:/Users/hille/AppData/Local/Temp/WF94FFB076D874ED4C//scratch///
Processing default memory
  ... MEM_TOTAL 4076 MB (default) [16 cores]
Processing $rem in C:/Program
Files/Wavefunction/Spartan24v110/P4e//../auxdir/config/preferences:
   (site specific preferences)
  ... THRESH             9
  ... SMALL_PROD_XCMAT   9
  ... BASIS_LIN_DEP_THRESH     5
  ... SCF_ALGORITHM     DIIS_GDM
  ... MAXSCF            250
  ... MAXDIIS          45
  ... THRESHDIIS         -1  (i.e. don't switch on delta-E)
  ... ECP_FIT          TRUE (Convert deprecated ECP files)
  ... GUI              GUI_SPARTAN
  ... TERSE_OUTPUT     TRUE !turn on spartan printing
  ... SCF_CONVERGENCE  7
  ... CCMAN2 FALSE  (qc4.3)
  ... SYMMETRY   FALSE  ! turn of symmetry for spartan16
  ... SYM_IGNORE TRUE   ! ..use FORCESYMMETRY to override
  ... GEOM_OPT_TOL_GRADIENT       700  ! loosen tolernaces for organic geometries
  ... GEOM_OPT_TOL_DISPLACEMENT  1400  ! was 1200 = .0012
  ... GEOM_OPT_TOL_ENERGY        2000  ! was  100 = .000 001
  ... GEN_SCFMAN  FALSE
Processing $rem in input file
  ... JOBTYPE    SP
  ... SCF_CONVERGENCE    7 (sp default for single point energy)
  ... METHOD            WB97X-D
  ... xc_grid           75000302  (75,302)
  ... BASIS             6-311+G**
  ... THRESH             12  #diffuse default
  ... MAXSCF            350  #diffuse default
  ... SOLVENT_METHOD    PCM DIMETHYLFORMAMIDE
  ... SET_ITER          100
  ... VARTHRESH         2    (default DFT)
  ... INCDFT            TRUE (default DFT)
  ... GUI                GUI_SPARTAN
  ... TERSE_OUTPUT       TRUE
  ... SCF_GUESS          READ
```

```
NAlpha2: 130
NElect   130
Mult     1
Symmetry turned off for PCM/SM12/SMD calculation

Checking the input file for inconsistencies...  ...done.
 Using dielectric of 37.2200 for DIMETHYLFORMAMIDE [pq]
 Using Optical Dielectric of 2.0478

--------------------------------------------------------------
User input:
--------------------------------------------------------------
$comment
Amprolium Dication
$end
$molecule
 2 1
    7     2.5870480602    0.16936255576   -0.31788854416
    6      2.973724942    -0.8410514669    -1.1422433564
    6     3.9235445668    -1.7373112064   -0.67441697881
    6     4.4613600912    -1.6032027061    0.59387354981
    6      4.044237047   -0.55593434296     1.4088169969
    6     3.1045677304    0.31669752483    0.92507910499
    6     2.3876490312   -0.95265363383    -2.5105867047
    6     1.5647800959     1.1369540177   -0.74556221919
    7    -1.4342283548   -0.86360484412    0.33313520016
    6    -2.4494382583  -0.086278070342  -0.091053101593
    7    -2.2249251103     1.0501206816   -0.70012377641
    6   -0.95514341296     1.4544836209   -0.90939005678
    6    0.16020620115    0.65640087348   -0.48795744575
    6   -0.14017858712   -0.50460086521    0.14259803859
    6    -3.8546524046   -0.51853574362    0.17820997665
    6    -4.5122964742    0.35059006006     1.2633991313
    6    -3.8159177384    0.24656084345     2.6170406646
    7   -0.79739280609     2.6183042341    -1.5208356793
    1       4.23376729    -2.5431121104    -1.3262172908
    1     5.2018046897    -2.3105650457    0.94723967392
    1     4.4386807697   -0.41238521403     2.4051066427
    1     2.7286270091     1.1518471303     1.5006066603
    1     2.7285480559    -1.8779954754    -2.9700172084
    1      2.715684391   -0.11615891255    -3.1346222486
    1     1.2957864217   -0.95556241554    -2.4878813478
    1     1.7104927132      1.347135616    -1.8057562668
    1     1.7497332199     2.0673286527   -0.20562405893
    1    -1.6358800915    -1.7353408677    0.80834000246
    1    0.59636856781     -1.202376047    0.51933607135
    1     -4.408470885   -0.42703876813   -0.75851854298
    1    -3.8670774344    -1.5688985768    0.47934826047
    1    -5.5525454594   0.027500867356     1.3513722624
    1    -4.5290899683     1.3901650851    0.92374605448
    1    -3.7790996466   -0.79232826195     2.9588750631
    1    -4.3474999247     0.8323559728     3.3700978863
    1    -2.7885338468    0.62186882751     2.5709103754
    1    0.10299276997       3.02633633    -1.7153834755
    1    -1.6172329952     3.1409217338    -1.7930532601
$end
$rem
JOBTYPE    SP
SCF_CONVERGENCE    7 (sp default for single point energy)
METHOD          WB97X-D
xc_grid         75000302  (75,302)
BASIS           6-311+G**
THRESH           12  #diffuse default
MAXSCF          350  #diffuse default
SOLVENT_METHOD  PCM DIMETHYLFORMAMIDE
SET_ITER        100
VARTHRESH       2    (default DFT)
INCDFT          TRUE (default DFT)
GUI              GUI_SPARTAN
TERSE_OUTPUT     TRUE
```

```
SCF_GUESS        READ
$end
$pcm
theory   CPCM
$end
$solvent
DIELECTRIC      DIMETHYLFORMAMIDE
$end
--------------------------------------------------------------
 ----------------------------------------------------------------
             Standard Nuclear Orientation (Angstroms)
    I     Atom           X                Y                Z
 ----------------------------------------------------------------
    1      N       2.5870480602     0.1693625558    -0.3178885442
    2      C       2.9737249420    -0.8410514669    -1.1422433564
    3      C       3.9235445668    -1.7373112064    -0.6744169788
    4      C       4.4613600912    -1.6032027061     0.5938735498
    5      C       4.0442370470    -0.5559343430     1.4088169969
    6      C       3.1045677304     0.3166975248     0.9250791050
    7      C       2.3876490312    -0.9526536338    -2.5105867047
    8      C       1.5647800959     1.1369540177    -0.7455622192
    9      N      -1.4342283548    -0.8636048441     0.3331352002
   10      C      -2.4494382583    -0.0862780703    -0.0910531016
   11      N      -2.2249251103     1.0501206816    -0.7001237764
   12      C      -0.9551434130     1.4544836209    -0.9093900568
   13      C       0.1602062012     0.6564008735    -0.4879574458
   14      C      -0.1401785871    -0.5046008652     0.1425980386
   15      C      -3.8546524046    -0.5185357436     0.1782099766
   16      C      -4.5122964742     0.3505900601     1.2633991313
   17      C      -3.8159177384     0.2465608435     2.6170406646
   18      N      -0.7973928061     2.6183042341    -1.5208356793
   19      H       4.2337672900    -2.5431121104    -1.3262172908
   20      H       5.2018046897    -2.3105650457     0.9472396739
   21      H       4.4386807697    -0.4123852140     2.4051066427
   22      H       2.7286270091     1.1518471303     1.5006066603
   23      H       2.7285480559    -1.8779954754    -2.9700172084
   24      H       2.7156843910    -0.1161589126    -3.1346222486
   25      H       1.2957864217    -0.9555624155    -2.4878813478
   26      H       1.7104927132     1.3471356160    -1.8057562668
   27      H       1.7497332199     2.0673286527    -0.2056240589
   28      H      -1.6358800915    -1.7353408677     0.8083400025
   29      H       0.5963685678    -1.2023760470     0.5193360714
   30      H      -4.4084708850    -0.4270387681    -0.7585185430
   31      H      -3.8670774344    -1.5688985768     0.4793482605
   32      H      -5.5525454594     0.0275008674     1.3513722624
   33      H      -4.5290899683     1.3901650851     0.9237460545
   34      H      -3.7790996466    -0.7923282619     2.9588750631
   35      H      -4.3474999247     0.8323559728     3.3700978863
   36      H      -2.7885338468     0.6218688275     2.5709103754
   37      H       0.1029927700     3.0263363300    -1.7153834755
   38      H      -1.6172329952     3.1409217338    -1.7930532601
 ----------------------------------------------------------------
 Nuclear Repulsion Energy =        1315.49143196 hartrees
 There are       65 alpha and       65 beta electrons
 Requested basis set is 6-311+G(d,p)
 There are 188 shells and 516 basis functions

 Total QAlloc Memory Limit   4076 MB
 Mega-Array Size       188 MB
 MEM_STATIC part       192 MB

 ..                                                                  (5.2.P)

--------------------------------------------------
-  Entering fldman on Thu Nov 14 11:34:13 2024  -
--------------------------------------------------

 Using dielectric of 37.2200 for DIMETHYLFORMAMIDE
 Using Optical Dielectric of 2.0478
 Discretize the solute cavity surface with Lebedev spheres
```

```
        Using 110 Lebedev grid points for each H atom
        Using 194 Lebedev grid points for other atoms
        Atomic van der Waals radii will be scaled by 1.20
 Remove points where switching function is < 1.0e-08
 Keep 2356 surface tesserae and discard 3336 interior tesserae
 Molecular Surface Area = 304.963 Angst**2
 A cutoff of  1.0D-12 yielded  12841 shell pairs
 There are    101830 function pairs (    108523 Cartesian)
 Smallest overlap matrix eigenvalue = 2.32E-06
 Linear dependence detected in AO basis
 Tighter screening thresholds may be required for diffuse basis sets
 Use S2THRESH > 12 and THRESH = 14 in case of SCF convergence issues
 Number of orthogonalized atomic orbitals = 510
 Maximum deviation from orthogonality = 2.005E-11

 Scale SEOQF with 1.000000e-02/1.000000e-01/1.000000e-01

 Standard Electronic Orientation quadrupole field applied
 Nucleus-field energy     =    -0.0000000067 hartrees

--------------------------------------------------
-  Entering gesman on Thu Nov 14 11:34:13 2024  -
--------------------------------------------------

 Guess MOs from SCF MO coefficient file
 Reading MOs from coefficient file
 Size of previous SCF MO coefficient file: 527340 (expected); 533544 (actual).

 Warning: Inconsistent size for SCF MO coefficient file.  Please check basis and PURECART
setting.

 Reading MOs from coefficient file

--------------------------------------------------
-  Entering scfman on Thu Nov 14 11:34:13 2024  -
--------------------------------------------------

 Long-range K will be added via erf
 Coulomb attenuation parameter = 0.2 bohr**(-1)
 A restricted hybrid HF-DFT SCF calculation will be
 performed using Pulay DIIS + Geometric Direct Minimization
 Polarizable Continuum solvation model will be applied
 Exchange:     0.2220 Hartree-Fock + 1.0000 wB97X-D + LR-HF
 Correlation:  1.0000 wB97X-D
 Using Euler-Maclaurin-Lebedev (75,302) quadrature formula
 Dispersion:   Grimme D
 SCF converges when RMS gradient is below 1.0E-07
 Exchange:     0.2220 Hartree-Fock + 1.0000 wB97X-D + LR-HF
 Correlation:  1.0000 wB97X-D
 Using Euler-Maclaurin-Lebedev (75,302) quadrature formula
 Dispersion:   Grimme D
 Using Q-Chem read-in guess as SCF_GUESS READ specified.

 using 6 threads for integral computing
 -------------------------------------------------------
 OpenMP Integral computing Module
 Release: version 1.0, May 2013, Q-Chem Inc. Pittsburgh
 -------------------------------------------------------
 using 6 threads for integral computing
 -------------------------------------------------------
 OpenMP Integral computing Module
 Release: version 1.0, May 2013, Q-Chem Inc. Pittsburgh
 -------------------------------------------------------
 ---------------------------------------
  Cycle       Energy         DIIS Error
 ---------------------------------------
    1    -763.9688736101      3.60E-04
    2    -764.2191001288      4.30E-04
    3    -764.2273602452      1.75E-04
    4    -764.2285224523      1.26E-04
```

```
    5    -764.2293640887      8.16E-05
    6    -764.2298369721      4.25E-05
    7    -764.2300040841      1.14E-05
    8    -764.2300176971      1.89E-06
    9    -764.2300179838      4.96E-07
   10    -764.2300179989      1.56E-07
   11    -764.2300180003      5.60E-08 Convergence criterion met
 ---------------------------------------

************** Final PCM Free Energy Summary **************
 G_electrostatic  =      -0.26826676 hartree =    -168.33993232 kcal/mol
 G_cavitation     =       0.00000000 hartree =       0.00000000 kcal/mol
 G_dispersion     =       0.00000000 hartree =       0.00000000 kcal/mol
 G_repulsion      =       0.00000000 hartree =       0.00000000 kcal/mol
 --------------------------------------------------
 Non-electrostatic Free Energy =       0.00000000 hartree =       0.00000000 kcal/mol
 Total                         =      -0.26826676 hartree =    -168.33993232 kcal/mol
 --------------------------------------------------
 SCF Energy (H0 + V/2)                      =    -764.23001800
 Solute Internal Energy (H0)                =    -763.96175124
 Total Free Energy (H0 + V/2 + non-elec)    =    -764.23001800 hartree
                                            = -479561.57431772 kcal/mol
***********************************************************

 SCF time:  CPU 243.45 s  wall 121.99 s
 SCF   energy in the final basis set = -764.23001800
 Total energy in the final basis set = -764.23001800

-------------------------------------------------
-  Entering anlman on Thu Nov 14 11:36:15 2024  -
-------------------------------------------------



 --------------------------------------------------------------

                   Orbital Energies (a.u.)
 --------------------------------------------------------------

 Alpha MOs
 -- Occupied --
-14.5762 -14.5438 -14.4947 -14.4731 -10.4339 -10.4251 -10.4029 -10.3946
-10.3874 -10.3825 -10.3595 -10.3512 -10.3376 -10.3368 -10.3118 -10.3109
-10.2948 -10.2759  -1.1747  -1.1675  -1.1057  -1.0361  -0.9911  -0.9605
 -0.9521  -0.9172  -0.8729  -0.8479  -0.8261  -0.8124  -0.7969  -0.7839
 -0.7511  -0.7171  -0.7112  -0.6863  -0.6671  -0.6606  -0.6473  -0.6193
 -0.6101  -0.6068  -0.5994  -0.5855  -0.5676  -0.5596  -0.5583  -0.5517
 -0.5389  -0.5343  -0.5151  -0.5138  -0.5114  -0.5033  -0.4948  -0.4933
 -0.4818  -0.4501  -0.4398  -0.4285  -0.4267  -0.4093  -0.4056  -0.3853
 -0.3645
 -- Virtual --
 -0.0336  -0.0120   0.0003   0.0101   0.0492   0.0601   0.0639   0.0656
  0.0764   0.0808   0.0850   0.0888   0.0945   0.0960   0.1024   0.1054
  0.1104   0.1134   0.1194   0.1236   0.1251   0.1292   0.1346   0.1368
  0.1415   0.1452   0.1515   0.1539   0.1564   0.1604   0.1648   0.1665
  0.1705   0.1708   0.1730   0.1765   0.1795   0.1881   0.1915   0.1955
  0.1998   0.2022   0.2052   0.2103   0.2134   0.2141   0.2167   0.2239
  0.2265   0.2314   0.2337   0.2359   0.2393   0.2427   0.2466   0.2473
  0.2518   0.2543   0.2575   0.2593   0.2648   0.2663   0.2705   0.2731
  0.2792   0.2803   0.2835   0.2910   0.2935   0.2976   0.3005   0.3030
  0.3050   0.3103   0.3122   0.3127   0.3199   0.3226   0.3249   0.3294
  0.3339   0.3375   0.3447   0.3495   0.3511   0.3560   0.3580   0.3642
  0.3660   0.3687   0.3766   0.3811   0.3859   0.3866   0.3936   0.3958
  0.3984   0.4042   0.4078   0.4146   0.4202   0.4282   0.4326   0.4419
  0.4525   0.4741   0.4758   0.4976   0.5028   0.5129   0.5224   0.5307
  0.5391   0.5506   0.5566   0.5689   0.5706   0.5783   0.5859   0.5875
  0.6034   0.6094   0.6114   0.6141   0.6251   0.6343   0.6405   0.6474
  0.6491   0.6678   0.6741   0.6791   0.6819   0.6903   0.6991   0.7077
  0.7101   0.7130   0.7245   0.7301   0.7349   0.7411   0.7428   0.7438
  0.7507   0.7562   0.7603   0.7671   0.7707   0.7751   0.7832   0.7835
  0.7895   0.7963   0.8012   0.8067   0.8123   0.8166   0.8198   0.8264
```

| | | | | | | | |
|---|---|---|---|---|---|---|---|
| 0.8300 | 0.8366 | 0.8466 | 0.8532 | 0.8552 | 0.8715 | 0.8775 | 0.8850 |
| 0.8945 | 0.8954 | 0.9046 | 0.9121 | 0.9268 | 0.9274 | 0.9355 | 0.9394 |
| 0.9495 | 0.9631 | 0.9786 | 0.9840 | 0.9906 | 0.9948 | 1.0056 | 1.0160 |
| 1.0295 | 1.0341 | 1.0385 | 1.0597 | 1.0605 | 1.0679 | 1.0897 | 1.0940 |
| 1.1009 | 1.1131 | 1.1332 | 1.1393 | 1.1505 | 1.1601 | 1.1641 | 1.1870 |
| 1.2051 | 1.2107 | 1.2269 | 1.2325 | 1.2382 | 1.2401 | 1.2597 | 1.2668 |
| 1.2966 | 1.3013 | 1.3106 | 1.3383 | 1.3549 | 1.3888 | 1.3936 | 1.4218 |
| 1.4370 | 1.4461 | 1.4591 | 1.4679 | 1.4871 | 1.5066 | 1.5242 | 1.5322 |
| 1.5462 | 1.5564 | 1.5646 | 1.5734 | 1.5745 | 1.5996 | 1.6028 | 1.6120 |
| 1.6203 | 1.6315 | 1.6348 | 1.6372 | 1.6412 | 1.6448 | 1.6490 | 1.6559 |
| 1.6628 | 1.6711 | 1.6800 | 1.6867 | 1.6897 | 1.7000 | 1.7085 | 1.7160 |
| 1.7282 | 1.7476 | 1.7528 | 1.7559 | 1.7784 | 1.7837 | 1.7864 | 1.7894 |
| 1.7938 | 1.8008 | 1.8025 | 1.8114 | 1.8197 | 1.8317 | 1.8395 | 1.8474 |
| 1.8570 | 1.8615 | 1.8693 | 1.8830 | 1.8873 | 1.8939 | 1.9095 | 1.9192 |
| 1.9342 | 1.9544 | 1.9628 | 1.9668 | 1.9811 | 1.9989 | 1.9989 | 2.0119 |
| 2.0182 | 2.0231 | 2.0316 | 2.0530 | 2.0767 | 2.0811 | 2.1060 | 2.1122 |
| 2.1247 | 2.1341 | 2.1474 | 2.1687 | 2.1990 | 2.2006 | 2.2228 | 2.2347 |
| 2.2506 | 2.2606 | 2.2682 | 2.2787 | 2.2834 | 2.3023 | 2.3143 | 2.3326 |
| 2.3468 | 2.3547 | 2.3633 | 2.3884 | 2.3996 | 2.4216 | 2.4351 | 2.4490 |
| 2.4588 | 2.4798 | 2.4952 | 2.5004 | 2.5097 | 2.5159 | 2.5249 | 2.5266 |
| 2.5498 | 2.5522 | 2.5631 | 2.5689 | 2.5936 | 2.6042 | 2.6135 | 2.6236 |
| 2.6438 | 2.6524 | 2.6629 | 2.6766 | 2.6875 | 2.7036 | 2.7181 | 2.7223 |
| 2.7320 | 2.7372 | 2.7443 | 2.7531 | 2.7658 | 2.7709 | 2.7760 | 2.7828 |
| 2.8009 | 2.8050 | 2.8157 | 2.8188 | 2.8270 | 2.8317 | 2.8442 | 2.8501 |
| 2.8670 | 2.8794 | 2.8858 | 2.8954 | 2.9109 | 2.9151 | 2.9418 | 2.9519 |
| 2.9638 | 2.9674 | 2.9706 | 2.9884 | 3.0027 | 3.0186 | 3.0249 | 3.0264 |
| 3.0353 | 3.0490 | 3.0668 | 3.0803 | 3.0908 | 3.1157 | 3.1251 | 3.1300 |
| 3.1394 | 3.1641 | 3.1993 | 3.2318 | 3.2891 | 3.3126 | 3.3594 | 3.3740 |
| 3.4261 | 3.4656 | 3.4801 | 3.5091 | 3.5372 | 3.5797 | 3.6188 | 3.6514 |
| 3.6676 | 3.7315 | 3.7762 | 3.7851 | 3.8123 | 3.8484 | 3.8702 | 3.8855 |
| 3.8989 | 3.9117 | 3.9299 | 3.9325 | 3.9603 | 3.9716 | 3.9925 | 3.9992 |
| 4.0165 | 4.0608 | 4.1027 | 4.1377 | 4.1657 | 4.2127 | 4.2482 | 4.3290 |
| 4.3397 | 4.4114 | 4.5261 | 4.5579 | 4.8439 | 4.9198 | 4.9285 | 5.0499 |
| 5.1736 | 5.2813 | 5.4462 | 23.7803 | 23.8374 | 23.9592 | 23.9770 | 23.9928 |
| 24.0181 | 24.0286 | 24.0670 | 24.0711 | 24.1096 | 24.1362 | 24.2034 | 24.2452 |
| 24.2730 | 35.6641 | 35.7121 | 35.7747 | 35.8654 | | | |

---

Ground-State Mulliken Net Atomic Charges

| Atom | Charge (a.u.) |
|---|---|
| 1 N | 0.346321 |
| 2 C | 0.271810 |
| 3 C | -0.126647 |
| 4 C | -0.212325 |
| 5 C | -0.279727 |
| 6 C | -0.061674 |
| 7 C | -0.889220 |
| 8 C | -0.205607 |
| 9 N | -0.113831 |
| 10 C | -0.232553 |
| 11 N | -0.069396 |
| 12 C | 0.287559 |
| 13 C | 0.020701 |
| 14 C | 0.002125 |
| 15 C | -0.329361 |
| 16 C | -0.248783 |
| 17 C | -0.569341 |
| 18 N | -0.540745 |
| 19 H | 0.222905 |
| 20 H | 0.230397 |
| 21 H | 0.228383 |
| 22 H | 0.250173 |
| 23 H | 0.217943 |
| 24 H | 0.257069 |
| 25 H | 0.226402 |
| 26 H | 0.264583 |
| 27 H | 0.278443 |
| 28 H | 0.420764 |

```
    29 H                    0.247660
    30 H                    0.237601
    31 H                    0.215513
    32 H                    0.188732
    33 H                    0.190958
    34 H                    0.173035
    35 H                    0.176427
    36 H                    0.169130
    37 H                    0.365646
    38 H                    0.388933
 ----------------------------------------
 Sum of atomic charges =     2.000000

-----------------------------------------------------------------
                   Cartesian Multipole Moments
-----------------------------------------------------------------
   Charge (ESU x 10^10)
                 9.6064
   Dipole Moment (Debye)
        X       9.4292      Y      -0.4690      Z      -2.1245
      Tot       9.6770
   Quadrupole Moments (Debye-Ang)
       XX     -28.8694     XY      -6.5971     YY     -68.3269
       XZ      -3.5393     YZ      -9.4954     ZZ     -81.4116
   Traceless Quadrupole Moments (Debye-Ang)
      QXX      91.9996    QYY     -26.3728    QZZ     -65.6268
      QXY     -19.7912    QXZ     -10.6180    QYZ     -28.4862
   Octopole Moments (Debye-Ang^2)
      XXX      73.1132    XXY     -85.9068    XYY      10.1410
      YYY      27.1887    XXZ      43.4201    XYZ      14.2472
      YYZ     -28.5105    XZZ      23.2186    YZZ       6.4118
      ZZZ       0.0041
   Traceless Octopole Moments (Debye-Ang^2)
      XXX     138.4426    YYY     878.5880    ZZZ    -134.1619
      XXY   -1131.6835    XXZ     606.5601    XYY    -167.3033
      XYZ     213.7084    XZZ      28.8607    YYZ    -472.3982
      YZZ     253.0955
   Hexadecapole Moments (Debye-Ang^3)
     XXXX   -4318.0109   XXXY      92.1859   XXYY    -833.8830
     XYYY     298.7091   YYYY    -664.4452   XXXZ     426.5378
     XXYZ      12.2338   XYYZ     110.2145   YYYZ     -56.6281
     XXZZ    -900.9951   XYZZ      88.7421   YYZZ    -248.6067
     XZZZ     327.9079   YZZZ      46.5008   ZZZZ   -1052.6001
   Traceless Hexadecapole Moments (Debye-Ang^3)
     XXXX    1350.6382   XXXY  -11904.1535   XXXZ    5876.7611
     XXYY    -566.4345   XXYZ    1260.4676   XXZZ    -784.2037
     XYYY    9780.7894   XYYZ   -1397.3765   XYZZ    2123.3641
     XZZZ   -4479.3845   YYYY   -2560.8365   YYYZ   -6018.2083
     YYZZ    3127.2710   YZZZ    4757.7407   ZZZZ   -2343.0673
-----------------------------------------------------------------
Total job time:  122.57s(wall), 243.62s(cpu)
Thu Nov 14 11:36:15 2024
```

```
Wavefunction Version of Q-Chem

 Parts of Q-Chem use Armadillo 8.300.2 (Tropical Shenanigans).
 http://arma.sourceforge.net/

 Q-Chem begins on Sun Oct 13 20:49:25 2024


     Scratch files written to
C:/Users/hille/AppData/Local/Temp/WFB0CB2A5C99266D7A/Conformer.15//scratch///
Processing default memory
  ... MEM_TOTAL 4076 MB (default) [16 cores]
Processing $rem in C:/Program
Files/Wavefunction/Spartan24v110/P4e//../auxdir/config/preferences:
   (site specific preferences)
  ... THRESH             9
  ... SMALL_PROD_XCMAT   9
  ... BASIS_LIN_DEP_THRESH     5
  ... SCF_ALGORITHM     DIIS_GDM
  ... MAXSCF            250
  ... MAXDIIS          45
  ... THRESHDIIS         -1  (i.e. don't switch on delta-E)
  ... ECP_FIT          TRUE (Convert deprecated ECP files)
  ... GUI              GUI_SPARTAN
  ... TERSE_OUTPUT     TRUE !turn on spartan printing
  ... SCF_CONVERGENCE  7
  ... CCMAN2 FALSE  (qc4.3)
  ... SYMMETRY   FALSE  ! turn of symmetry for spartan16
  ... SYM_IGNORE TRUE   ! ..use FORCESYMMETRY to override
  ... GEOM_OPT_TOL_GRADIENT       700  ! loosen tolernaces for organic geometries
  ... GEOM_OPT_TOL_DISPLACEMENT  1400  ! was 1200 = .0012
  ... GEOM_OPT_TOL_ENERGY        2000  ! was  100 = .000 001
  ... GEN_SCFMAN  FALSE
Processing $rem in input file
  ... JOBTYPE    OPT
  ... TIDY_SYM   TRUE
  ... METHOD            WB97X-D
  ... xc_grid           75000302  (75,302)
  ... BASIS             6-311+G**
  ... THRESH             12  #diffuse default
  ... MAXSCF            350  #diffuse default
  ... GEOM_OPT_TOL_ENERGY       22850
  ... GEOM_OPT_TOL_GRADIENT       800
  ... VARTHRESH         2    (default DFT)
  ... INCDFT            TRUE (default DFT)
  ... GEOM_OPT_HESSIAN      READ (main opt)
  ... EXTERNAL_HESSIAN      1
  ... GUI                GUI_SPARTAN
  ... TERSE_OUTPUT       TRUE
NAlpha2: 130
NElect   130
Mult     1

Checking the input file for inconsistencies...  ...done.

--------------------------------------------------------------
User input:
--------------------------------------------------------------
$comment
Molecule14
$end
$molecule
 2 1
    7     2.7035452826       0.14486072   -0.14369403984
    6     2.9088045296   -0.62622709281    -1.2591136013
    6      3.883021251    -1.6381561184    -1.2195728909
    6     4.6643180607    -1.8250505475  -0.089020133198
    6     4.4794808131   -0.99413390467     1.0000785244
    6     3.5026839078  -0.010780619718    0.94708837248
```

```
    6     2.1138218138   -0.44122212683    -2.5230481484
    6     1.6749033564     1.2296645461   -0.11076577628
    7    -1.4122500931     -0.545850597     1.1548726651
    6    -2.2891312989   -0.24163884938    0.18767989611
    7     -1.956482948    0.53747252729   -0.86666703978
    6   -0.70910467101     1.0170858783   -0.92871487445
    6    0.26145674352    0.71120364819   0.017339248282
    6   -0.14990150228  -0.089583177871     1.0873169261
    6    -3.6980567478   -0.75684674462     0.2899745633
    6    -4.6706011636    0.28637752066    0.84211098215
    6      -4.42592026    0.61821780812     2.3077871333
    7   -0.44164104231     1.8103126816    -2.0303159096
    1      4.053713047    -2.2770145277      -2.08733105
    1     5.4312787865    -2.5997499378  -0.071103284836
    1     5.1078220778    -1.1054603807     1.8826940929
    1     3.3600357467    0.66367261716     1.7884178406
    1      2.576095895     -0.973902924    -3.3613983657
    1     2.0711647415    0.61193605423    -2.8099528625
    1     1.1043984298   -0.83971178034    -2.3939883112
    1     1.8466623433     1.8472208285   -0.99729162906
    1     1.8851536696     1.8800647263    0.74717213157
    1    -1.7252384012    -1.1257814121     1.9360799454
    1    0.49597158598   -0.38856014942     1.9063113232
    1    -4.0256835932    -1.0564012649   -0.71284354088
    1      -3.71873914    -1.6612764269    0.90912999189
    1     -5.691033392   -0.10253493242       0.74219305
    1    -4.6208636617     1.2076740969    0.24945169857
    1    -4.4616748809   -0.28440160959     2.9260263635
    1    -5.1953708977     1.3074364134     2.6702076073
    1    -3.4537703155     1.0988225262     2.4533385163
    1   -0.19913091666     2.7765894915    -1.8182702555
    1    -1.2797371559     1.8356730403    -2.6221791591
$end
$rem
JOBTYPE    OPT
TIDY_SYM   TRUE
METHOD          WB97X-D
xc_grid         75000302  (75,302)
BASIS           6-311+G**
THRESH           12  #diffuse default
MAXSCF          350  #diffuse default
GEOM_OPT_TOL_ENERGY     22850
GEOM_OPT_TOL_GRADIENT     800
VARTHRESH       2    (default DFT)
INCDFT          TRUE (default DFT)
GEOM_OPT_HESSIAN      READ (main opt)
EXTERNAL_HESSIAN      1
GUI              GUI_SPARTAN
TERSE_OUTPUT     TRUE
$end
$opt
$end
--------------------------------------------------------------
```

Standard Nuclear Orientation (Angstroms)

| I | Atom | X | Y | Z |
|---|---|---|---|---|
| 1 | N | 2.7035452826 | 0.1448607200 | -0.1436940398 |
| 2 | C | 2.9088045296 | -0.6262270928 | -1.2591136013 |
| 3 | C | 3.8830212510 | -1.6381561184 | -1.2195728909 |
| 4 | C | 4.6643180607 | -1.8250505475 | -0.0890201332 |
| 5 | C | 4.4794808131 | -0.9941339047 | 1.0000785244 |
| 6 | C | 3.5026839078 | -0.0107806197 | 0.9470883725 |
| 7 | C | 2.1138218138 | -0.4412221268 | -2.5230481484 |
| 8 | C | 1.6749033564 | 1.2296645461 | -0.1107657763 |
| 9 | N | -1.4122500931 | -0.5458505970 | 1.1548726651 |
| 10 | C | -2.2891312989 | -0.2416388494 | 0.1876798961 |
| 11 | N | -1.9564829480 | 0.5374725273 | -0.8666670398 |
| 12 | C | -0.7091046710 | 1.0170858783 | -0.9287148745 |
| 13 | C | 0.2614567435 | 0.7112036482 | 0.0173392483 |

```
   14      C      -0.1499015023   -0.0895831779    1.0873169261
   15      C      -3.6980567478   -0.7568467446    0.2899745633
   16      C      -4.6706011636    0.2863775207    0.8421109822
   17      C      -4.4259202600    0.6182178081    2.3077871333
   18      N      -0.4416410423    1.8103126816   -2.0303159096
   19      H       4.0537130470   -2.2770145277   -2.0873310500
   20      H       5.4312787865   -2.5997499378   -0.0711032848
   21      H       5.1078220778   -1.1054603807    1.8826940929
   22      H       3.3600357467    0.6636726172    1.7884178406
   23      H       2.5760958950   -0.9739029240   -3.3613983657
   24      H       2.0711647415    0.6119360542   -2.8099528625
   25      H       1.1043984298   -0.8397117803   -2.3939883112
   26      H       1.8466623433    1.8472208285   -0.9972916291
   27      H       1.8851536696    1.8800647263    0.7471721316
   28      H      -1.7252384012   -1.1257814121    1.9360799454
   29      H       0.4959715860   -0.3885601494    1.9063113232
   30      H      -4.0256835932   -1.0564012649   -0.7128435409
   31      H      -3.7187391400   -1.6612764269    0.9091299919
   32      H      -5.6910333920   -0.1025349324    0.7421930500
   33      H      -4.6208636617    1.2076740969    0.2494516986
   34      H      -4.4616748809   -0.2844016096    2.9260263635
   35      H      -5.1953708977    1.3074364134    2.6702076073
   36      H      -3.4537703155    1.0988225262    2.4533385163
   37      H      -0.1991309167    2.7765894915   -1.8182702555
   38      H      -1.2797371559    1.8356730403   -2.6221791591
 ----------------------------------------------------------------
 Nuclear Repulsion Energy =        1307.88989677 hartrees
 There are       65 alpha and       65 beta electrons
 Requested basis set is 6-311+G(d,p)
 There are 188 shells and 516 basis functions

 Total QAlloc Memory Limit   4076 MB
 Mega-Array Size       188 MB
 MEM_STATIC part       192 MB

 ..                                                                      (5.2.P)

--------------------------------------------------
-  Entering fldman on Sun Oct 13 20:49:25 2024  -
--------------------------------------------------

 A cutoff of  1.0D-12 yielded  12750 shell pairs
 There are    101439 function pairs (    108143 Cartesian)
 Smallest overlap matrix eigenvalue = 2.14E-06
 Linear dependence detected in AO basis
 Tighter screening thresholds may be required for diffuse basis sets
 Use S2THRESH > 12 and THRESH = 14 in case of SCF convergence issues
 Number of orthogonalized atomic orbitals = 510
 Maximum deviation from orthogonality = 9.514E-12

 Scale SEOQF with 1.000000e-02/1.000000e-01/1.000000e-01

 Standard Electronic Orientation quadrupole field applied
 Nucleus-field energy     =    -0.0000000113 hartrees

--------------------------------------------------
-  Entering gesman on Sun Oct 13 20:49:25 2024  -
--------------------------------------------------

 Guess from superposition of atomic densities
 Warning:  Energy on first SCF cycle will be non-variational
 SAD guess density has 132.000000 electrons

--------------------------------------------------
-  Entering scfman on Sun Oct 13 20:49:25 2024  -
--------------------------------------------------

 Long-range K will be added via erf
 Coulomb attenuation parameter = 0.2 bohr**(-1)
 A restricted hybrid HF-DFT SCF calculation will be
```

```
 performed using Pulay DIIS + Geometric Direct Minimization
 Exchange:     0.2220 Hartree-Fock + 1.0000 wB97X-D + LR-HF
 Correlation:  1.0000 wB97X-D
 Using Euler-Maclaurin-Lebedev (75,302) quadrature formula
 Dispersion:   Grimme D
 SCF converges when RMS gradient is below 1.0E-07
 Exchange:     0.2220 Hartree-Fock + 1.0000 wB97X-D + LR-HF
 Correlation:  1.0000 wB97X-D
 Using Euler-Maclaurin-Lebedev (75,302) quadrature formula
 Dispersion:   Grimme D
 ---------------------------------------
  Cycle       Energy         DIIS Error
 ---------------------------------------
    1    -770.2947054854      2.95E-02
    2    -763.7487599488      2.40E-03
    3    -763.7516271085      2.61E-03
    4    -763.9445745699      3.76E-04
    5    -763.9480447323      1.86E-04
    6    -763.9490334137      3.40E-05
    7    -763.9490678165      1.34E-05
    8    -763.9490734881      3.15E-06
    9    -763.9490740253      1.36E-06
   10    -763.9490741145      4.69E-07
   11    -763.9490741307      2.06E-07
   12    -763.9490741341      8.78E-08 Convergence criterion met
 ---------------------------------------
 SCF time:  CPU 387.22 s  wall 628.71 s
 SCF   energy in the final basis set = -763.94907413
 Total energy in the final basis set = -763.94907413

-------------------------------------------------
-  Entering anlman on Sun Oct 13 20:59:54 2024  -
-------------------------------------------------



 --------------------------------------------------------------

                   Orbital Energies (a.u.)
 --------------------------------------------------------------

 Alpha MOs
 -- Occupied --
-14.8179 -14.8129 -14.7219 -14.7136 -10.6740 -10.6695 -10.6629 -10.6394
-10.6358 -10.6274 -10.6141 -10.5791 -10.5709 -10.5708 -10.5441 -10.5336
-10.4968 -10.4716  -1.4287  -1.3993  -1.3437  -1.2656  -1.2281  -1.2002
 -1.1796  -1.1464  -1.1042  -1.0771  -1.0633  -1.0505  -1.0288  -1.0105
 -0.9852  -0.9552  -0.9447  -0.9067  -0.9015  -0.8855  -0.8758  -0.8660
 -0.8469  -0.8418  -0.8378  -0.8191  -0.8069  -0.7922  -0.7851  -0.7772
 -0.7738  -0.7540  -0.7498  -0.7446  -0.7356  -0.7259  -0.7237  -0.7061
 -0.7037  -0.6698  -0.6570  -0.6493  -0.6373  -0.6302  -0.6282  -0.6190
 -0.6145
 -- Virtual --
 -0.2785  -0.2683  -0.2551  -0.2272  -0.1780  -0.1632  -0.1528  -0.1460
 -0.1307  -0.1262  -0.1184  -0.1141  -0.1120  -0.1047  -0.0988  -0.0920
 -0.0883  -0.0865  -0.0829  -0.0813  -0.0768  -0.0719  -0.0703  -0.0659
 -0.0614  -0.0601  -0.0560  -0.0519  -0.0507  -0.0476  -0.0413  -0.0358
 -0.0333  -0.0278  -0.0261  -0.0232  -0.0202  -0.0177  -0.0138  -0.0099
 -0.0037  -0.0020  -0.0003   0.0029   0.0065   0.0100   0.0117   0.0167
  0.0183   0.0228   0.0257   0.0269   0.0292   0.0343   0.0362   0.0398
  0.0437   0.0456   0.0482   0.0490   0.0529   0.0562   0.0611   0.0639
  0.0666   0.0697   0.0744   0.0770   0.0799   0.0844   0.0866   0.0889
  0.0916   0.0964   0.1006   0.1047   0.1052   0.1081   0.1141   0.1173
  0.1202   0.1227   0.1268   0.1299   0.1333   0.1347   0.1415   0.1455
  0.1473   0.1531   0.1575   0.1620   0.1654   0.1717   0.1734   0.1799
  0.1808   0.1830   0.1849   0.1928   0.1978   0.2041   0.2137   0.2179
  0.2247   0.2393   0.2497   0.2697   0.2727   0.2940   0.3095   0.3114
  0.3208   0.3253   0.3354   0.3389   0.3475   0.3511   0.3585   0.3635
  0.3758   0.3772   0.3852   0.3903   0.3963   0.4041   0.4129   0.4205
  0.4240   0.4350   0.4407   0.4476   0.4522   0.4621   0.4692   0.4726
  0.4831   0.4858   0.4946   0.4957   0.5045   0.5085   0.5130   0.5178
```

| | | | | | | | |
|---|---|---|---|---|---|---|---|
| 0.5256 | 0.5313 | 0.5378 | 0.5433 | 0.5470 | 0.5531 | 0.5568 | 0.5588 |
| 0.5648 | 0.5677 | 0.5701 | 0.5732 | 0.5812 | 0.5864 | 0.5932 | 0.5985 |
| 0.6050 | 0.6131 | 0.6161 | 0.6251 | 0.6377 | 0.6440 | 0.6498 | 0.6535 |
| 0.6682 | 0.6697 | 0.6729 | 0.6787 | 0.6921 | 0.6956 | 0.6967 | 0.7044 |
| 0.7149 | 0.7296 | 0.7335 | 0.7418 | 0.7558 | 0.7666 | 0.7750 | 0.7839 |
| 0.7917 | 0.7964 | 0.7990 | 0.8166 | 0.8181 | 0.8317 | 0.8389 | 0.8501 |
| 0.8541 | 0.8649 | 0.8764 | 0.8791 | 0.8926 | 0.9057 | 0.9136 | 0.9252 |
| 0.9283 | 0.9441 | 0.9570 | 0.9777 | 0.9894 | 1.0110 | 1.0132 | 1.0289 |
| 1.0495 | 1.0614 | 1.0719 | 1.1066 | 1.1130 | 1.1398 | 1.1645 | 1.1833 |
| 1.2106 | 1.2211 | 1.2308 | 1.2522 | 1.2557 | 1.2832 | 1.2945 | 1.3147 |
| 1.3257 | 1.3310 | 1.3376 | 1.3382 | 1.3589 | 1.3625 | 1.3690 | 1.3719 |
| 1.3856 | 1.3985 | 1.4034 | 1.4108 | 1.4218 | 1.4274 | 1.4337 | 1.4372 |
| 1.4399 | 1.4422 | 1.4481 | 1.4561 | 1.4670 | 1.4716 | 1.4777 | 1.4912 |
| 1.5028 | 1.5158 | 1.5168 | 1.5260 | 1.5313 | 1.5393 | 1.5453 | 1.5590 |
| 1.5606 | 1.5653 | 1.5690 | 1.5785 | 1.5825 | 1.5896 | 1.6025 | 1.6082 |
| 1.6180 | 1.6332 | 1.6401 | 1.6523 | 1.6577 | 1.6714 | 1.6729 | 1.6766 |
| 1.6921 | 1.7039 | 1.7112 | 1.7266 | 1.7305 | 1.7478 | 1.7552 | 1.7646 |
| 1.7786 | 1.7894 | 1.8008 | 1.8224 | 1.8266 | 1.8453 | 1.8496 | 1.8726 |
| 1.8853 | 1.8925 | 1.8963 | 1.9091 | 1.9325 | 1.9502 | 1.9667 | 1.9879 |
| 1.9925 | 2.0031 | 2.0211 | 2.0304 | 2.0359 | 2.0558 | 2.0696 | 2.0766 |
| 2.0970 | 2.1098 | 2.1277 | 2.1414 | 2.1536 | 2.1645 | 2.2031 | 2.2102 |
| 2.2222 | 2.2323 | 2.2370 | 2.2473 | 2.2622 | 2.2831 | 2.2879 | 2.2934 |
| 2.3174 | 2.3253 | 2.3423 | 2.3598 | 2.3657 | 2.3697 | 2.3786 | 2.3959 |
| 2.4093 | 2.4158 | 2.4394 | 2.4483 | 2.4516 | 2.4588 | 2.4740 | 2.4940 |
| 2.4985 | 2.5079 | 2.5238 | 2.5296 | 2.5390 | 2.5447 | 2.5482 | 2.5561 |
| 2.5666 | 2.5726 | 2.5764 | 2.5848 | 2.5949 | 2.5984 | 2.6072 | 2.6160 |
| 2.6204 | 2.6292 | 2.6428 | 2.6585 | 2.6680 | 2.6785 | 2.6869 | 2.7012 |
| 2.7054 | 2.7157 | 2.7178 | 2.7248 | 2.7390 | 2.7473 | 2.7567 | 2.7770 |
| 2.7852 | 2.7948 | 2.8153 | 2.8200 | 2.8331 | 2.8493 | 2.8643 | 2.8779 |
| 2.8883 | 2.9144 | 2.9297 | 2.9945 | 3.0133 | 3.0662 | 3.0921 | 3.1387 |
| 3.1455 | 3.1708 | 3.2256 | 3.2592 | 3.2769 | 3.3011 | 3.3347 | 3.3770 |
| 3.3999 | 3.4657 | 3.5000 | 3.5217 | 3.5385 | 3.5728 | 3.6012 | 3.6674 |
| 3.6802 | 3.6875 | 3.7029 | 3.7110 | 3.7291 | 3.7434 | 3.7550 | 3.7602 |
| 3.8210 | 3.8564 | 3.8941 | 3.9003 | 3.9436 | 4.0256 | 4.0339 | 4.0860 |
| 4.1163 | 4.2058 | 4.2968 | 4.3156 | 4.5581 | 4.6408 | 4.6820 | 4.7045 |
| 4.8730 | 5.0173 | 5.1457 | 23.5163 | 23.5590 | 23.6847 | 23.7153 | 23.7321 |
| 23.7927 | 23.7990 | 23.8264 | 23.8461 | 23.8729 | 23.9080 | 23.9207 | 23.9283 |
| 24.0251 | 35.4095 | 35.4389 | 35.4844 | 35.5779 | | | |

Ground-State Mulliken Net Atomic Charges

| Atom | | Charge (a.u.) |
|---|---|---|
| 1 | N | 0.444207 |
| 2 | C | 0.056908 |
| 3 | C | -0.035930 |
| 4 | C | -0.207464 |
| 5 | C | -0.296691 |
| 6 | C | -0.072816 |
| 7 | C | -0.718218 |
| 8 | C | -0.202056 |
| 9 | N | -0.126540 |
| 10 | C | -0.209813 |
| 11 | N | 0.069812 |
| 12 | C | 0.049318 |
| 13 | C | -0.033757 |
| 14 | C | 0.100243 |
| 15 | C | -0.342423 |
| 16 | C | -0.263860 |
| 17 | C | -0.517562 |
| 18 | N | -0.463443 |
| 19 | H | 0.224520 |
| 20 | H | 0.234068 |
| 21 | H | 0.232018 |
| 22 | H | 0.221068 |
| 23 | H | 0.232572 |
| 24 | H | 0.218738 |
| 25 | H | 0.231562 |
| 26 | H | 0.225073 |

```
    27 H                     0.248917
    28 H                     0.404980
    29 H                     0.218422
    30 H                     0.252004
    31 H                     0.209308
    32 H                     0.223695
    33 H                     0.203983
    34 H                     0.177024
    35 H                     0.217987
    36 H                     0.134151
    37 H                     0.322658
    38 H                     0.337337
  ----------------------------------------
  Sum of atomic charges =     2.000000

 -----------------------------------------------------------------
                    Cartesian Multipole Moments
 -----------------------------------------------------------------
    Charge (ESU x 10^10)
                 9.6064
    Dipole Moment (Debye)
         X       7.1092      Y      -0.9580      Z       1.2420
       Tot       7.2802
    Quadrupole Moments (Debye-Ang)
        XX      -3.5094     XY     -13.4491     YY     -78.0588
        XZ      -8.8417     YZ      -5.3580     ZZ     -73.2688
    Traceless Quadrupole Moments (Debye-Ang)
       QXX     144.3088    QYY     -79.3393    QZZ     -64.9694
       QXY     -40.3473    QXZ     -26.5250    QYZ     -16.0741
    Octopole Moments (Debye-Ang^2)
       XXX      49.1223    XXY     -98.7579    XYY      10.6500
       YYY      22.8117    XXZ      54.1256    XYZ      21.7006
       YYZ     -16.5460    XZZ      -2.3900    YZZ      -0.9435
       ZZZ       3.3417
    Traceless Octopole Moments (Debye-Ang^2)
       XXX     220.3939    YYY    1034.1827    ZZZ    -318.1656
       XXY   -1250.6991    XXZ     689.1199    XYY     -12.3968
       XYZ     325.5097    XZZ    -207.9971    YYZ    -370.9542
       YZZ     216.5163
    Hexadecapole Moments (Debye-Ang^3)
      XXXX   -3767.8933   XXXY    -125.1725   XXYY    -807.1558
      XYYY     289.9777   YYYY    -589.8733   XXXZ     347.4004
      XXYZ      56.2222   XYYZ     108.1552   YYYZ     -20.7673
      XXZZ    -908.5237   XYZZ      69.7209   YYZZ    -236.6238
      XZZZ     367.4114   YZZZ       5.3502   ZZZZ   -1012.0915
    Traceless Hexadecapole Moments (Debye-Ang^3)
      XXXX   14422.5703   XXXY  -23696.7846   XXXZ    -556.4767
      XXYY   -5816.3642   XXYZ    5296.5079   XXZZ   -8606.2061
      XYYY   19893.9809   XYYZ    -988.2095   XYZZ    3802.8037
      XZZZ    1544.6863   YYYY    1621.8780   YYYZ   -4001.0357
      YYZZ    4194.4862   YZZZ   -1295.4722   ZZZZ    4411.7199
 -----------------------------------------------------------------

------------------------------------------------
-  Entering drvman on Sun Oct 13 20:59:54 2024  -
------------------------------------------------

 Calculating analytic gradient of the SCF energy
 Gradient of SCF Energy
           1           2           3           4           5           6
    1  -0.0072284  -0.0057836   0.0024178  -0.0011125   0.0047415  -0.0086340
    2   0.0011741   0.0144593  -0.0064668   0.0048261  -0.0108799   0.0100344
    3   0.0010477   0.0082733  -0.0038140   0.0078264  -0.0087788  -0.0053176
           7           8           9          10          11          12
    1  -0.0020827  -0.0091471  -0.0045010   0.0226612   0.0030706   0.0215516
    2  -0.0045939  -0.0002613  -0.0021806  -0.0122281   0.0266684  -0.0168463
    3  -0.0042571  -0.0045869  -0.0004847   0.0384412  -0.0350104   0.0274921
          13          14          15          16          17          18
    1  -0.0097347  -0.0181539  -0.0050061   0.0070082   0.0003651   0.0184408
    2   0.0201053  -0.0144902   0.0062565  -0.0083661  -0.0002936  -0.0008071
```

```
   3  -0.0300550   0.0119204   0.0074907  -0.0062971  -0.0005296  -0.0073140
          19          20          21          22          23          24
   1   0.0044599   0.0034972   0.0047098   0.0026896  -0.0001165   0.0046047
   2  -0.0063067  -0.0027506  -0.0038103   0.0007234   0.0000165   0.0005670
   3  -0.0022879  -0.0000627   0.0019982   0.0046955  -0.0065549  -0.0002060
          25          26          27          28          29          30
   1   0.0017488   0.0072368  -0.0041284  -0.0027539  -0.0019510  -0.0041634
   2  -0.0028975   0.0036161   0.0046015  -0.0042013  -0.0011865  -0.0020477
   3   0.0012765  -0.0010940   0.0023684   0.0035595   0.0021302  -0.0012809
          31          32          33          34          35          36
   1  -0.0003159  -0.0041503   0.0001740   0.0008212  -0.0031242  -0.0007309
   2  -0.0013148   0.0008606   0.0036004  -0.0007143   0.0016835  -0.0018354
   3  -0.0010904   0.0031397   0.0018863  -0.0010690  -0.0005590  -0.0019109
          37          38
   1  -0.0094827  -0.0078977
   2   0.0046115   0.0006744
   3   0.0011103  -0.0020955
 Max gradient component =       3.844E-02
 RMS gradient           =       9.423E-03
 Gradient time:  CPU 152.98 s  wall 248.36 s

-------------------------------------------------
-  Entering optman on Sun Oct 13 21:04:02 2024  -
-------------------------------------------------

 Geometry Optimization Parameters
   NAtoms,    NIC,     NZ,  NCons,   NDum,   NFix, NCnnct, MaxDiis
       38     272       0       0       0       0       0       0

 Cartesian Hessian read from HESS file


** GEOMETRY OPTIMIZATION IN DELOCALIZED INTERNAL COORDINATES **
   Searching for a Minimum

   Optimization Cycle:   1

                    Coordinates (Angstroms)
     ATOM                X               Y               Z
      1  N         2.7035452826    0.1448607200   -0.1436940398
      2  C         2.9088045296   -0.6262270928   -1.2591136013
      3  C         3.8830212510   -1.6381561184   -1.2195728909
      4  C         4.6643180607   -1.8250505475   -0.0890201332
      5  C         4.4794808131   -0.9941339047    1.0000785244
      6  C         3.5026839078   -0.0107806197    0.9470883725
      7  C         2.1138218138   -0.4412221268   -2.5230481484
      8  C         1.6749033564    1.2296645461   -0.1107657763
      9  N        -1.4122500931   -0.5458505970    1.1548726651
     10  C        -2.2891312989   -0.2416388494    0.1876798961
     11  N        -1.9564829480    0.5374725273   -0.8666670398
     12  C        -0.7091046710    1.0170858783   -0.9287148745
     13  C         0.2614567435    0.7112036482    0.0173392483
     14  C        -0.1499015023   -0.0895831779    1.0873169261
     15  C        -3.6980567478   -0.7568467446    0.2899745633
     16  C        -4.6706011636    0.2863775207    0.8421109822
     17  C        -4.4259202600    0.6182178081    2.3077871333
     18  N        -0.4416410423    1.8103126816   -2.0303159096
     19  H         4.0537130470   -2.2770145277   -2.0873310500
     20  H         5.4312787865   -2.5997499378   -0.0711032848
     21  H         5.1078220778   -1.1054603807    1.8826940929
     22  H         3.3600357467    0.6636726172    1.7884178406
     23  H         2.5760958950   -0.9739029240   -3.3613983657
     24  H         2.0711647415    0.6119360542   -2.8099528625
     25  H         1.1043984298   -0.8397117803   -2.3939883112
     26  H         1.8466623433    1.8472208285   -0.9972916291
     27  H         1.8851536696    1.8800647263    0.7471721316
     28  H        -1.7252384012   -1.1257814121    1.9360799454
     29  H         0.4959715860   -0.3885601494    1.9063113232
     30  H        -4.0256835932   -1.0564012649   -0.7128435409
     31  H        -3.7187391400   -1.6612764269    0.9091299919
```

```
    32  H        -5.6910333920   -0.1025349324    0.7421930500
    33  H        -4.6208636617    1.2076740969    0.2494516986
    34  H        -4.4616748809   -0.2844016096    2.9260263635
    35  H        -5.1953708977    1.3074364134    2.6702076073
    36  H        -3.4537703155    1.0988225262    2.4533385163
    37  H        -0.1991309167    2.7765894915   -1.8182702555
    38  H        -1.2797371559    1.8356730403   -2.6221791591
  Point Group: c1    Number of degrees of freedom:   108


  Energy is   -763.949074134


 Attempting to generate delocalized internal coordinates

 Transforming Cartesian Hessian to Internal Coordinates
 Hessian Transformation does not Include Derivative of B-matrix
  internal optimization (0)

108 Hessian modes will be used to form the next step
  Hessian Eigenvalues:
     0.002207    0.002692    0.004091    0.005683    0.009924    0.012119
     0.015878    0.018909    0.019152    0.019758    0.020476    0.022141
     0.022524    0.022821    0.023920    0.024666    0.025718    0.026591
     0.028227    0.028325    0.030007    0.030445    0.035340    0.036509
     0.038707    0.041430    0.042789    0.043667    0.044354    0.046118
     0.050432    0.053786    0.054889    0.055169    0.063320    0.078792
     0.085481    0.093289    0.121394    0.121703    0.127088    0.130695
     0.131637    0.132980    0.134764    0.139098    0.143070    0.147632
     0.147678    0.148146    0.149497    0.151109    0.152373    0.153014
     0.153426    0.154216    0.176913    0.206074    0.206949    0.216528
     0.219168    0.229718    0.236919    0.240347    0.246385    0.256319
     0.264318    0.269324    0.280902    0.296160    0.299689    0.300128
     0.300610    0.301171    0.301680    0.301813    0.304072    0.304398
     0.305210    0.305353    0.307499    0.308147    0.311671    0.315819
     0.318289    0.329648    0.332028    0.334268    0.336985    0.341354
     0.344524    0.348862    0.349736    0.355591    0.362337    0.380365
     0.393465    0.394672    0.405667    0.412762    0.416140    0.420559
     0.430716    0.441553    0.448385    0.453524    0.559029    0.779535

 Minimum search - taking simple RFO step
 Searching for Lamda that Minimizes Along All modes
 Value Taken    Lamda =  -0.02364348
 Calculated Step too Large.  Step scaled by  0.709101 !!
 Step Taken.  Stepsize is  0.300000  0.019049

                             Maximum     Tolerance    Cnvgd?
        Gradient            0.023701      0.000800      NO
        Displacement        0.099413      0.001400      NO
        Energy change      *********      0.000228      NO


 New Cartesian Coordinates Obtained by Inverse Iteration

 Displacement from previous Coordinates is:  0.529730
 ----------------------------------------------------------------
             Standard Nuclear Orientation (Angstroms)
    I     Atom           X                Y                Z
 ----------------------------------------------------------------
    1      N       2.7111955414     0.1325029177    -0.1359778678
    2      C       2.9000656388    -0.6418215471    -1.2453842380
    3      C       3.8719176817    -1.6328327484    -1.2049860235
    4      C       4.6599846848    -1.8085101770    -0.0772882132
    5      C       4.4779409150    -0.9758623391     1.0205361518
    6      C       3.5052042066    -0.0144189269     0.9580607661
    7      C       2.0725797173    -0.4467793050    -2.4786138258
    8      C       1.6843496723     1.2128416581    -0.1064691721
    9      N      -1.4052560504    -0.5408037917     1.1032810886
   10      C      -2.3068590141    -0.2403772002     0.1416189860
   11      N      -1.9874688494     0.5391668365    -0.8679672715
```

```
  12      C      -0.7428687134     1.0364755936    -0.9317304758
  13      C       0.2645195197     0.7036568302     0.0129410332
  14      C      -0.1353468591    -0.0889314135     1.0491154624
  15      C      -3.6989176640    -0.7663860440     0.2621475447
  16      C      -4.6640690475     0.2805670291     0.8478182073
  17      C      -4.3816321135     0.6144298029     2.3092484453
  18      N      -0.4682584267     1.8268225352    -2.0202876800
  19      H       4.0255803043    -2.2625395971    -2.0744178009
  20      H       5.4260595121    -2.5780005386    -0.0601291269
  21      H       5.0967528706    -1.0689073676     1.9049714696
  22      H       3.3421640084     0.6685601610     1.7830937172
  23      H       2.5214591744    -0.9893384474    -3.3105045993
  24      H       2.0065546054     0.6018264705    -2.7751295537
  25      H       1.0608033910    -0.8363482783    -2.3356686983
  26      H       1.8222239469     1.8229738650    -0.9974328192
  27      H       1.9152295997     1.8572680920     0.7446894029
  28      H      -1.7092841638    -1.1235021191     1.8796982807
  29      H       0.5119195554    -0.4029842522     1.8600546327
  30      H      -4.0330239582    -1.0569521503    -0.7371548297
  31      H      -3.7054763447    -1.6693499624     0.8823760860
  32      H      -5.6765456059    -0.1203570278     0.7563406446
  33      H      -4.6300237092     1.1918558094     0.2428749642
  34      H      -4.4020951777    -0.2853285182     2.9324725319
  35      H      -5.1383743138     1.2998405040     2.6951134301
  36      H      -3.4088807328     1.1029029029     2.4387349576
  37      H      -0.0959225001     2.7538659657    -1.8450124898
  38      H      -1.2862013015     1.9047747780    -2.6210331177
 ----------------------------------------------------------------
 Nuclear Repulsion Energy =        1311.89409759 hartrees
 There are       65 alpha and       65 beta electrons

--------------------------------------------------
-  Entering fldman on Sun Oct 13 21:04:02 2024  -
--------------------------------------------------

 Applying Cartesian multipole field
    Component          Value
    ---------          -----
     (2,0,0)        1.00000E-12
     (0,2,0)        2.00000E-11
     (0,0,2)       -3.00000E-11
 Nucleus-field energy     =    -0.0000000109 hartrees

--------------------------------------------------
-  Entering gesman on Sun Oct 13 21:04:02 2024  -
--------------------------------------------------

 Requested basis set is 6-311+G(d,p)
 There are 188 shells and 516 basis functions
 A cutoff of  1.0D-12 yielded  12788 shell pairs
 There are    101687 function pairs (    108423 Cartesian)
 Smallest overlap matrix eigenvalue = 2.10E-06
 Linear dependence detected in AO basis
 Tighter screening thresholds may be required for diffuse basis sets
 Use S2THRESH > 12 and THRESH = 14 in case of SCF convergence issues
 Number of orthogonalized atomic orbitals = 510
 Maximum deviation from orthogonality = 1.648E-11
 Guess MOs from SCF MO coefficient file
 Reading MOs from coefficient file
 Reading MOs from coefficient file

--------------------------------------------------
-  Entering scfman on Sun Oct 13 21:04:03 2024  -
--------------------------------------------------

 Long-range K will be added via erf
 Coulomb attenuation parameter = 0.2 bohr**(-1)
 A restricted hybrid HF-DFT SCF calculation will be
 performed using Pulay DIIS + Geometric Direct Minimization
 Exchange:     0.2220 Hartree-Fock + 1.0000 wB97X-D + LR-HF
```

```
 Correlation:  1.0000 wB97X-D
 Using Euler-Maclaurin-Lebedev (75,302) quadrature formula
 Dispersion:   Grimme D
 SCF converges when RMS gradient is below 1.0E-07
 Geometry optimization detected.  Setting ReadMinima to 0
 Setting SaveMinima to 0
 ---------------------------------------
  Cycle       Energy         DIIS Error
 ---------------------------------------
    1    -764.0579212940      8.40E-04
    2    -763.9590287511      1.68E-04
    3    -763.9594435302      1.55E-04
    4    -763.9600926137      2.47E-05
    5    -763.9601140628      1.22E-05
    6    -763.9601204098      4.38E-06
    7    -763.9601214715      1.76E-06
    8    -763.9601216505      5.12E-07
    9    -763.9601216748      2.50E-07
   10    -763.9601216779      8.74E-08 Convergence criterion met
 ---------------------------------------
 SCF time:  CPU 352.48 s  wall 580.01 s
 SCF   energy in the final basis set = -763.96012168
 Total energy in the final basis set = -763.96012168

-------------------------------------------------
-  Entering anlman on Sun Oct 13 21:13:43 2024  -
-------------------------------------------------



 --------------------------------------------------------------

                   Orbital Energies (a.u.)
 --------------------------------------------------------------


 Alpha MOs
 -- Occupied --
-14.8166 -14.8028 -14.7213 -14.7172 -10.6760 -10.6685 -10.6550 -10.6382
-10.6350 -10.6256 -10.6104 -10.5821 -10.5706 -10.5699 -10.5456 -10.5308
-10.4967 -10.4714  -1.4245  -1.4024  -1.3431  -1.2743  -1.2304  -1.2017
 -1.1856  -1.1447  -1.1034  -1.0810  -1.0591  -1.0525  -1.0315  -1.0083
 -0.9888  -0.9538  -0.9467  -0.9091  -0.9029  -0.8885  -0.8791  -0.8661
 -0.8489  -0.8438  -0.8386  -0.8206  -0.8095  -0.7949  -0.7871  -0.7824
 -0.7736  -0.7535  -0.7510  -0.7470  -0.7374  -0.7276  -0.7227  -0.7125
 -0.7031  -0.6725  -0.6577  -0.6476  -0.6380  -0.6306  -0.6276  -0.6195
 -0.6180
 -- Virtual --
 -0.2723  -0.2691  -0.2519  -0.2255  -0.1758  -0.1634  -0.1517  -0.1455
 -0.1315  -0.1250  -0.1184  -0.1143  -0.1113  -0.1042  -0.0989  -0.0926
 -0.0876  -0.0863  -0.0822  -0.0809  -0.0758  -0.0710  -0.0700  -0.0651
 -0.0620  -0.0594  -0.0557  -0.0520  -0.0501  -0.0463  -0.0400  -0.0342
 -0.0324  -0.0267  -0.0256  -0.0228  -0.0202  -0.0169  -0.0138  -0.0090
 -0.0033  -0.0029   0.0010   0.0033   0.0074   0.0116   0.0129   0.0177
  0.0185   0.0232   0.0261   0.0271   0.0307   0.0356   0.0365   0.0412
  0.0432   0.0466   0.0500   0.0515   0.0533   0.0570   0.0619   0.0668
  0.0674   0.0713   0.0760   0.0779   0.0813   0.0832   0.0873   0.0883
  0.0927   0.0956   0.1011   0.1034   0.1070   0.1127   0.1149   0.1201
  0.1217   0.1227   0.1289   0.1299   0.1346   0.1376   0.1420   0.1461
  0.1487   0.1535   0.1581   0.1648   0.1660   0.1734   0.1747   0.1805
  0.1818   0.1839   0.1865   0.1950   0.1988   0.2046   0.2139   0.2182
  0.2234   0.2384   0.2511   0.2679   0.2723   0.2902   0.3098   0.3138
  0.3233   0.3260   0.3349   0.3425   0.3473   0.3509   0.3629   0.3646
  0.3745   0.3810   0.3865   0.3917   0.3976   0.4038   0.4165   0.4216
  0.4242   0.4374   0.4424   0.4472   0.4528   0.4645   0.4721   0.4756
  0.4824   0.4896   0.4959   0.4990   0.5085   0.5116   0.5143   0.5186
  0.5304   0.5326   0.5373   0.5417   0.5475   0.5533   0.5569   0.5601
  0.5670   0.5680   0.5716   0.5751   0.5819   0.5880   0.5948   0.6011
  0.6079   0.6147   0.6167   0.6284   0.6351   0.6440   0.6531   0.6540
  0.6674   0.6678   0.6740   0.6782   0.6925   0.6957   0.6992   0.7105
  0.7163   0.7285   0.7359   0.7409   0.7565   0.7688   0.7782   0.7828
  0.7911   0.7987   0.8029   0.8195   0.8232   0.8353   0.8379   0.8511
```

| | | | | | | | |
|---|---|---|---|---|---|---|---|
| 0.8602 | 0.8649 | 0.8749 | 0.8831 | 0.9000 | 0.9076 | 0.9161 | 0.9286 |
| 0.9376 | 0.9453 | 0.9587 | 0.9788 | 0.9915 | 1.0112 | 1.0145 | 1.0338 |
| 1.0505 | 1.0614 | 1.0770 | 1.1064 | 1.1144 | 1.1423 | 1.1636 | 1.1767 |
| 1.2073 | 1.2207 | 1.2327 | 1.2492 | 1.2567 | 1.2813 | 1.3078 | 1.3198 |
| 1.3267 | 1.3285 | 1.3356 | 1.3426 | 1.3579 | 1.3665 | 1.3683 | 1.3711 |
| 1.3895 | 1.3966 | 1.4045 | 1.4140 | 1.4193 | 1.4289 | 1.4365 | 1.4408 |
| 1.4417 | 1.4444 | 1.4543 | 1.4579 | 1.4681 | 1.4771 | 1.4907 | 1.4947 |
| 1.5053 | 1.5181 | 1.5217 | 1.5256 | 1.5342 | 1.5407 | 1.5488 | 1.5609 |
| 1.5621 | 1.5656 | 1.5717 | 1.5825 | 1.5868 | 1.5895 | 1.6070 | 1.6089 |
| 1.6216 | 1.6382 | 1.6432 | 1.6573 | 1.6632 | 1.6722 | 1.6743 | 1.6898 |
| 1.6952 | 1.7052 | 1.7104 | 1.7329 | 1.7382 | 1.7517 | 1.7575 | 1.7693 |
| 1.7820 | 1.7905 | 1.8030 | 1.8216 | 1.8364 | 1.8535 | 1.8617 | 1.8834 |
| 1.8893 | 1.8979 | 1.9014 | 1.9138 | 1.9379 | 1.9538 | 1.9641 | 1.9830 |
| 1.9962 | 2.0026 | 2.0253 | 2.0338 | 2.0419 | 2.0614 | 2.0678 | 2.0809 |
| 2.1028 | 2.1166 | 2.1304 | 2.1496 | 2.1559 | 2.1737 | 2.2082 | 2.2172 |
| 2.2297 | 2.2323 | 2.2429 | 2.2528 | 2.2679 | 2.2901 | 2.2919 | 2.3016 |
| 2.3147 | 2.3308 | 2.3476 | 2.3636 | 2.3650 | 2.3769 | 2.3859 | 2.3965 |
| 2.4097 | 2.4141 | 2.4393 | 2.4499 | 2.4543 | 2.4614 | 2.4697 | 2.4907 |
| 2.5033 | 2.5119 | 2.5260 | 2.5338 | 2.5442 | 2.5456 | 2.5510 | 2.5640 |
| 2.5653 | 2.5757 | 2.5797 | 2.5884 | 2.5938 | 2.6039 | 2.6100 | 2.6184 |
| 2.6326 | 2.6372 | 2.6485 | 2.6648 | 2.6711 | 2.6776 | 2.6926 | 2.7020 |
| 2.7092 | 2.7163 | 2.7239 | 2.7338 | 2.7408 | 2.7557 | 2.7678 | 2.7793 |
| 2.7854 | 2.8003 | 2.8204 | 2.8225 | 2.8430 | 2.8575 | 2.8657 | 2.8851 |
| 2.8928 | 2.9251 | 2.9324 | 2.9981 | 3.0259 | 3.0730 | 3.1192 | 3.1363 |
| 3.1534 | 3.1852 | 3.2416 | 3.2523 | 3.2909 | 3.3169 | 3.3552 | 3.3831 |
| 3.4159 | 3.4747 | 3.5117 | 3.5323 | 3.5495 | 3.5889 | 3.6151 | 3.6533 |
| 3.6900 | 3.7035 | 3.7101 | 3.7149 | 3.7338 | 3.7490 | 3.7593 | 3.7675 |
| 3.8284 | 3.8665 | 3.8907 | 3.9041 | 3.9370 | 4.0064 | 4.0345 | 4.0922 |
| 4.1005 | 4.2034 | 4.3007 | 4.3225 | 4.5761 | 4.6498 | 4.6890 | 4.7139 |
| 4.8858 | 5.0319 | 5.1715 | 23.5203 | 23.5605 | 23.7169 | 23.7191 | 23.7378 |
| 23.7755 | 23.8001 | 23.8357 | 23.8536 | 23.8924 | 23.9106 | 23.9636 | 23.9710 |
| 24.0372 | 35.4185 | 35.4456 | 35.4985 | 35.5932 | | | |

Ground-State Mulliken Net Atomic Charges

| Atom | Charge (a.u.) |
|---|---|
| 1 N | 0.441365 |
| 2 C | 0.081934 |
| 3 C | -0.031622 |
| 4 C | -0.221154 |
| 5 C | -0.290367 |
| 6 C | -0.073838 |
| 7 C | -0.726896 |
| 8 C | -0.200732 |
| 9 N | -0.163587 |
| 10 C | -0.177878 |
| 11 N | 0.067432 |
| 12 C | 0.069010 |
| 13 C | -0.045737 |
| 14 C | 0.112206 |
| 15 C | -0.340229 |
| 16 C | -0.265125 |
| 17 C | -0.520508 |
| 18 N | -0.467078 |
| 19 H | 0.222036 |
| 20 H | 0.235137 |
| 21 H | 0.231219 |
| 22 H | 0.217318 |
| 23 H | 0.232025 |
| 24 H | 0.218452 |
| 25 H | 0.235789 |
| 26 H | 0.211952 |
| 27 H | 0.247577 |
| 28 H | 0.400303 |
| 29 H | 0.215162 |
| 30 H | 0.250473 |
| 31 H | 0.209637 |
| 32 H | 0.223674 |

```
    33 H                    0.203598
    34 H                    0.178022
    35 H                    0.218198
    36 H                    0.135848
    37 H                    0.320746
    38 H                    0.345638
  ----------------------------------------
  Sum of atomic charges =     2.000000

 -----------------------------------------------------------------
                    Cartesian Multipole Moments
 -----------------------------------------------------------------
    Charge (ESU x 10^10)
                 9.6064
    Dipole Moment (Debye)
         X       7.3455      Y      -0.7284      Z       0.7847
       Tot       7.4231
    Quadrupole Moments (Debye-Ang)
        XX      -3.8539     XY     -13.2571     YY     -77.8366
        XZ      -7.9829     YZ      -5.9627     ZZ     -73.1195
    Traceless Quadrupole Moments (Debye-Ang)
       QXX     143.2482    QYY     -78.6997    QZZ     -64.5486
       QXY     -39.7713    QXZ     -23.9486    QYZ     -17.8880
    Octopole Moments (Debye-Ang^2)
       XXX      53.0733    XXY     -97.9844    XYY      11.0782
       YYY      23.6117    XXZ      55.5044    XYZ      20.4856
       YYZ     -18.5061    XZZ      -2.3382    YZZ       2.3743
       ZZZ       2.0106
    Traceless Octopole Moments (Debye-Ang^2)
       XXX     239.7795    YYY    1002.1604    ZZZ    -320.9210
       XXY   -1253.7704    XXZ     715.5394    XYY     -19.2673
       XYZ     307.2844    XZZ    -220.5121    YYZ    -394.6184
       YZZ     251.6100
    Hexadecapole Moments (Debye-Ang^3)
      XXXX   -3755.7307   XXXY    -118.1300   XXYY    -808.5560
      XYYY     291.3301   YYYY    -589.1745   XXXZ     345.1107
      XXYZ      55.3099   XYYZ     102.8562   YYYZ     -24.4066
      XXZZ    -902.7099   XYZZ      68.1474   YYZZ    -227.8154
      XZZZ     354.9079   YZZZ       0.3446   ZZZZ    -994.2529
    Traceless Hexadecapole Moments (Debye-Ang^3)
      XXXX   14722.0845   XXXY  -23264.2920   XXXZ     107.2561
      XXYY   -6162.2081   XXYZ    5343.9884   XXZZ   -8559.8764
      XYYY   19729.0254   XYYZ   -1243.2169   XYZZ    3535.2666
      XZZZ    1135.9607   YYYY    1479.9235   YYYZ   -3953.3390
      YYZZ    4682.2846   YZZZ   -1390.6494   ZZZZ    3877.5918
 -----------------------------------------------------------------

------------------------------------------------
-  Entering drvman on Sun Oct 13 21:13:43 2024  -
------------------------------------------------

 Calculating analytic gradient of the SCF energy
 Gradient of SCF Energy
           1           2           3           4           5           6
    1  -0.0018415   0.0023288  -0.0034597  -0.0011634  -0.0015552   0.0012582
    2  -0.0017869   0.0026199  -0.0000770   0.0025318   0.0007566  -0.0005329
    3   0.0006137   0.0037176  -0.0040234   0.0031141  -0.0006536  -0.0031783
           7           8           9          10          11          12
    1  -0.0004247  -0.0035842   0.0001730   0.0121947  -0.0088652   0.0030239
    2  -0.0018239   0.0002999   0.0015474   0.0014033   0.0038187  -0.0101374
    3  -0.0015205  -0.0020359  -0.0031042   0.0066929  -0.0052449   0.0171645
          13          14          15          16          17          18
    1  -0.0030871  -0.0025461  -0.0013005   0.0021998  -0.0009511   0.0153164
    2   0.0008817  -0.0002924   0.0041424  -0.0041931   0.0005654   0.0004762
    3  -0.0022636  -0.0019903   0.0016426  -0.0035148   0.0006143  -0.0104543
          19          20          21          22          23          24
    1   0.0014659   0.0012460   0.0013684   0.0017300   0.0001267   0.0014518
    2  -0.0020038  -0.0007560  -0.0013486  -0.0004456   0.0004785   0.0002569
    3  -0.0003716   0.0001440   0.0001677   0.0015910  -0.0015998  -0.0006380
          25          26          27          28          29          30
```

```
    1   0.0004763   0.0016658  -0.0014686  -0.0014257  -0.0009912  -0.0016510
    2  -0.0010490   0.0013764   0.0015484  -0.0020286  -0.0001324  -0.0013796
    3   0.0001129   0.0005305   0.0008414   0.0013608   0.0009263   0.0000443
           31          32          33          34          35          36
    1   0.0000267  -0.0013227   0.0001815   0.0004685  -0.0008980   0.0000598
    2  -0.0006412   0.0006807   0.0015331  -0.0003504   0.0003904  -0.0005960
    3  -0.0004224   0.0011761   0.0012745  -0.0004768  -0.0002643  -0.0005863
           37          38
    1  -0.0082598  -0.0019664
    2   0.0038217   0.0004455
    3   0.0005287   0.0000850
 Max gradient component =       1.716E-02
 RMS gradient           =       3.538E-03
 Gradient time:  CPU 146.47 s  wall 240.21 s

-------------------------------------------------
-  Entering optman on Sun Oct 13 21:17:43 2024  -
-------------------------------------------------

 Geometry Optimization Parameters
   NAtoms,    NIC,     NZ,  NCons,   NDum,   NFix, NCnnct, MaxDiis
       38     272       0       0       0       0       0       0

 Cartesian Hessian Update
 Hessian updated using BFGS update


** GEOMETRY OPTIMIZATION IN DELOCALIZED INTERNAL COORDINATES **
   Searching for a Minimum

   Optimization Cycle:   2

                      Coordinates (Angstroms)
     ATOM                X               Y               Z
      1  N         2.7111955414    0.1325029177   -0.1359778678
      2  C         2.9000656388   -0.6418215471   -1.2453842380
      3  C         3.8719176817   -1.6328327484   -1.2049860235
      4  C         4.6599846848   -1.8085101770   -0.0772882132
      5  C         4.4779409150   -0.9758623391    1.0205361518
      6  C         3.5052042066   -0.0144189269    0.9580607661
      7  C         2.0725797173   -0.4467793050   -2.4786138258
      8  C         1.6843496723    1.2128416581   -0.1064691721
      9  N        -1.4052560504   -0.5408037917    1.1032810886
     10  C        -2.3068590141   -0.2403772002    0.1416189860
     11  N        -1.9874688494    0.5391668365   -0.8679672715
     12  C        -0.7428687134    1.0364755936   -0.9317304758
     13  C         0.2645195197    0.7036568302    0.0129410332
     14  C        -0.1353468591   -0.0889314135    1.0491154624
     15  C        -3.6989176640   -0.7663860440    0.2621475447
     16  C        -4.6640690475    0.2805670291    0.8478182073
     17  C        -4.3816321135    0.6144298029    2.3092484453
     18  N        -0.4682584267    1.8268225352   -2.0202876800
     19  H         4.0255803043   -2.2625395971   -2.0744178009
     20  H         5.4260595121   -2.5780005386   -0.0601291269
     21  H         5.0967528706   -1.0689073676    1.9049714696
     22  H         3.3421640084    0.6685601610    1.7830937172
     23  H         2.5214591744   -0.9893384474   -3.3105045993
     24  H         2.0065546054    0.6018264705   -2.7751295537
     25  H         1.0608033910   -0.8363482783   -2.3356686983
     26  H         1.8222239469    1.8229738650   -0.9974328192
     27  H         1.9152295997    1.8572680920    0.7446894029
     28  H        -1.7092841638   -1.1235021191    1.8796982807
     29  H         0.5119195554   -0.4029842522    1.8600546327
     30  H        -4.0330239582   -1.0569521503   -0.7371548297
     31  H        -3.7054763447   -1.6693499624    0.8823760860
     32  H        -5.6765456059   -0.1203570278    0.7563406446
     33  H        -4.6300237092    1.1918558094    0.2428749642
     34  H        -4.4020951777   -0.2853285182    2.9324725319
     35  H        -5.1383743138    1.2998405040    2.6951134301
     36  H        -3.4088807328    1.1029029029    2.4387349576
```

```
   37  H        -0.0959225001    2.7538659657   -1.8450124898
   38  H        -1.2862013015    1.9047747780   -2.6210331177
  Point Group: c1    Number of degrees of freedom:   108


  Energy is   -763.960121678

 Hessian updated using BFGS update
  internal optimization (0)

108 Hessian modes will be used to form the next step
  Hessian Eigenvalues:
     0.002208    0.002692    0.004090    0.005687    0.009940    0.011501
     0.015875    0.018915    0.019164    0.019757    0.020473    0.022142
     0.022569    0.022820    0.023920    0.024639    0.025712    0.026593
     0.028224    0.028325    0.029888    0.030437    0.035089    0.036429
     0.038725    0.041337    0.042796    0.043655    0.044362    0.046117
     0.050406    0.053207    0.054032    0.055095    0.063329    0.078452
     0.085453    0.093275    0.121389    0.121707    0.127018    0.130581
     0.130782    0.132978    0.133972    0.138335    0.143108    0.145941
     0.147679    0.148155    0.149338    0.150543    0.152293    0.152926
     0.153128    0.156495    0.180428    0.205964    0.206867    0.216644
     0.221688    0.229787    0.235251    0.238995    0.247626    0.259139
     0.267299    0.269084    0.279861    0.296834    0.299422    0.300147
     0.300610    0.301200    0.301659    0.301823    0.304069    0.304366
     0.305210    0.305424    0.307539    0.308136    0.312070    0.315142
     0.323578    0.328888    0.332138    0.334262    0.337001    0.341477
     0.344628    0.349462    0.354252    0.361026    0.379992    0.388868
     0.392750    0.405064    0.407009    0.414632    0.420125    0.423516
     0.431185    0.441651    0.450734    0.484347    0.569589    0.776680

 Minimum search - taking simple RFO step
 Searching for Lamda that Minimizes Along All modes
 Value Taken    Lamda =  -0.00852618
 Calculated Step too Large.  Step scaled by  0.549751 !!
 Step Taken.  Stepsize is  0.300000  0.015349

                           Maximum     Tolerance    Cnvgd?
        Gradient          0.010766      0.000800      NO
        Displacement      0.090355      0.001400      NO
        Energy change    -0.011048      0.000228      NO


 New Cartesian Coordinates Obtained by Inverse Iteration

 Displacement from previous Coordinates is:  0.459835
 ----------------------------------------------------------------
             Standard Nuclear Orientation (Angstroms)
    I     Atom           X                Y                Z
 ----------------------------------------------------------------
    1      N       2.7081290120     0.1340072416    -0.1331001938
    2      C       2.8858484459    -0.6521397773    -1.2338207170
    3      C       3.8651745621    -1.6355838534    -1.1875619275
    4      C       4.6595347140    -1.8034028904    -0.0651845805
    5      C       4.4744718278    -0.9640817057     1.0300952670
    6      C       3.4986033314    -0.0049437590     0.9639527623
    7      C       2.0378546302    -0.4660755455    -2.4510959126
    8      C       1.6875525111     1.2142751881    -0.1130672180
    9      N      -1.3991916928    -0.5514470827     1.0816767764
   10      C      -2.3102127778    -0.2451487021     0.1247528719
   11      N      -1.9875739031     0.5545844731    -0.8660029596
   12      C      -0.7541770891     1.0728683491    -0.9348675941
   13      C       0.2634378250     0.7112710533    -0.0010236026
   14      C      -0.1281044345    -0.0966703525     1.0268177102
   15      C      -3.6954367259    -0.7772171409     0.2454073244
   16      C      -4.6556341089     0.2715219335     0.8482957759
   17      C      -4.3531710087     0.6058150659     2.3055944739
   18      N      -0.5003885052     1.8689927222    -2.0031083502
   19      H       4.0085556397    -2.2650157529    -2.0567082757
   20      H       5.4253632706    -2.5702276646    -0.0464818668
```

```
   21      H       5.0886524865    -1.0439869788     1.9174775308
   22      H       3.3264948398     0.6858229596     1.7789384100
   23      H       2.4707349289    -1.0244502869    -3.2788959196
   24      H       1.9628962140     0.5780469071    -2.7594363030
   25      H       1.0255747615    -0.8466057296    -2.2875915504
   26      H       1.8199810136     1.8141881604    -1.0105382666
   27      H       1.9249596067     1.8596958946     0.7330522134
   28      H      -1.6941595307    -1.1409426692     1.8529757958
   29      H       0.5299159201    -0.4235405839     1.8237308014
   30      H      -4.0291136515    -1.0534195955    -0.7566066490
   31      H      -3.6993138704    -1.6828051581     0.8607118026
   32      H      -5.6647493878    -0.1356652260     0.7618623100
   33      H      -4.6299933629     1.1769776175     0.2355740496
   34      H      -4.3672306196    -0.2917686030     2.9320892672
   35      H      -5.1033557262     1.2906941115     2.7025676489
   36      H      -3.3805295831     1.0977179619     2.4259885045
   37      H       0.0079330126     2.7341730129    -1.8805981245
   38      H      -1.3193325754     2.0044864057    -2.5858712848
 ----------------------------------------------------------------
 Nuclear Repulsion Energy =        1313.40511308 hartrees
 There are       65 alpha and       65 beta electrons

--------------------------------------------------
-  Entering fldman on Sun Oct 13 21:17:43 2024  -
--------------------------------------------------

 Applying Cartesian multipole field
    Component          Value
    ---------          -----
     (2,0,0)        1.00000E-12
     (0,2,0)        2.00000E-11
     (0,0,2)       -3.00000E-11
 Nucleus-field energy     =    -0.0000000104 hartrees

--------------------------------------------------
-  Entering gesman on Sun Oct 13 21:17:43 2024  -
--------------------------------------------------

 Requested basis set is 6-311+G(d,p)
 There are 188 shells and 516 basis functions
 A cutoff of  1.0D-12 yielded  12801 shell pairs
 There are    101740 function pairs (    108476 Cartesian)
 Smallest overlap matrix eigenvalue = 2.12E-06
 Linear dependence detected in AO basis
 Tighter screening thresholds may be required for diffuse basis sets
 Use S2THRESH > 12 and THRESH = 14 in case of SCF convergence issues
 Number of orthogonalized atomic orbitals = 510
 Maximum deviation from orthogonality = 8.038E-12
 Guess MOs from SCF MO coefficient file
 Reading MOs from coefficient file
 Reading MOs from coefficient file

--------------------------------------------------
-  Entering scfman on Sun Oct 13 21:17:43 2024  -
--------------------------------------------------

 Long-range K will be added via erf
 Coulomb attenuation parameter = 0.2 bohr**(-1)
 A restricted hybrid HF-DFT SCF calculation will be
 performed using Pulay DIIS + Geometric Direct Minimization
 Exchange:     0.2220 Hartree-Fock + 1.0000 wB97X-D + LR-HF
 Correlation:  1.0000 wB97X-D
 Using Euler-Maclaurin-Lebedev (75,302) quadrature formula
 Dispersion:   Grimme D
 SCF converges when RMS gradient is below 1.0E-07
 Geometry optimization detected.  Setting ReadMinima to 0
 Setting SaveMinima to 0
 ---------------------------------------
  Cycle       Energy         DIIS Error
 ---------------------------------------
```

```
    1    -763.9843947239      4.66E-04
    2    -763.9633267177      1.14E-04
    3    -763.9635099390      1.11E-04
    4    -763.9638694504      1.60E-05
    5    -763.9638811072      8.11E-06
    6    -763.9638836927      3.23E-06
    7    -763.9638842030      1.05E-06
    8    -763.9638842542      4.35E-07
    9    -763.9638842650      1.32E-07
   10    -763.9638842660      6.18E-08 Convergence criterion met
 ---------------------------------------
 SCF time:  CPU 303.23 s  wall 484.36 s
 SCF   energy in the final basis set = -763.96388427
 Total energy in the final basis set = -763.96388427

--------------------------------------------------
-  Entering anlman on Sun Oct 13 21:25:48 2024  -
--------------------------------------------------



 --------------------------------------------------------------


                   Orbital Energies (a.u.)
 --------------------------------------------------------------


 Alpha MOs
 -- Occupied --
-14.8166 -14.7982 -14.7266 -14.7132 -10.6761 -10.6668 -10.6523 -10.6388
-10.6348 -10.6259 -10.6086 -10.5831 -10.5717 -10.5704 -10.5475 -10.5297
-10.4964 -10.4710  -1.4208  -1.4040  -1.3448  -1.2751  -1.2305  -1.2010
 -1.1872  -1.1442  -1.1026  -1.0828  -1.0569  -1.0531  -1.0327  -1.0080
 -0.9896  -0.9530  -0.9467  -0.9113  -0.9042  -0.8913  -0.8804  -0.8657
 -0.8515  -0.8443  -0.8372  -0.8217  -0.8105  -0.7961  -0.7870  -0.7842
 -0.7732  -0.7536  -0.7521  -0.7489  -0.7410  -0.7286  -0.7218  -0.7157
 -0.7026  -0.6713  -0.6582  -0.6454  -0.6371  -0.6299  -0.6262  -0.6202
 -0.6157
 -- Virtual --
 -0.2695  -0.2679  -0.2499  -0.2262  -0.1748  -0.1636  -0.1508  -0.1450
 -0.1325  -0.1243  -0.1182  -0.1142  -0.1111  -0.1040  -0.0990  -0.0933
 -0.0876  -0.0863  -0.0821  -0.0807  -0.0754  -0.0705  -0.0696  -0.0648
 -0.0628  -0.0592  -0.0555  -0.0524  -0.0496  -0.0456  -0.0394  -0.0343
 -0.0320  -0.0263  -0.0256  -0.0228  -0.0207  -0.0161  -0.0134  -0.0092
 -0.0044  -0.0026   0.0013   0.0039   0.0075   0.0117   0.0136   0.0180
  0.0183   0.0233   0.0261   0.0273   0.0308   0.0362   0.0367   0.0414
  0.0436   0.0471   0.0510   0.0520   0.0532   0.0575   0.0624   0.0671
  0.0686   0.0718   0.0767   0.0783   0.0811   0.0828   0.0871   0.0876
  0.0925   0.0953   0.1011   0.1029   0.1077   0.1128   0.1151   0.1208
  0.1218   0.1233   0.1301   0.1307   0.1356   0.1391   0.1418   0.1468
  0.1496   0.1539   0.1579   0.1647   0.1658   0.1741   0.1755   0.1806
  0.1821   0.1857   0.1903   0.1960   0.2000   0.2058   0.2137   0.2181
  0.2220   0.2382   0.2516   0.2680   0.2717   0.2881   0.3097   0.3137
  0.3237   0.3270   0.3333   0.3421   0.3463   0.3506   0.3636   0.3666
  0.3750   0.3824   0.3871   0.3923   0.3986   0.4048   0.4176   0.4217
  0.4269   0.4382   0.4433   0.4459   0.4535   0.4644   0.4707   0.4788
  0.4828   0.4915   0.4962   0.5005   0.5087   0.5139   0.5162   0.5211
  0.5307   0.5324   0.5354   0.5421   0.5466   0.5526   0.5548   0.5611
  0.5658   0.5676   0.5725   0.5768   0.5828   0.5879   0.5957   0.6013
  0.6090   0.6152   0.6156   0.6296   0.6345   0.6436   0.6542   0.6549
  0.6664   0.6673   0.6751   0.6777   0.6916   0.6954   0.7021   0.7110
  0.7174   0.7274   0.7366   0.7388   0.7563   0.7680   0.7772   0.7852
  0.7895   0.7996   0.8072   0.8203   0.8281   0.8379   0.8398   0.8508
  0.8629   0.8659   0.8754   0.8832   0.9020   0.9057   0.9178   0.9282
  0.9419   0.9469   0.9616   0.9788   0.9909   1.0103   1.0143   1.0357
  1.0529   1.0620   1.0800   1.1036   1.1171   1.1479   1.1631   1.1740
  1.2051   1.2239   1.2373   1.2467   1.2558   1.2787   1.3079   1.3197
  1.3254   1.3283   1.3370   1.3410   1.3574   1.3682   1.3694   1.3733
  1.3915   1.3966   1.4046   1.4140   1.4169   1.4279   1.4340   1.4383
  1.4422   1.4441   1.4568   1.4606   1.4690   1.4808   1.4931   1.4955
  1.5047   1.5185   1.5229   1.5249   1.5347   1.5423   1.5511   1.5597
  1.5604   1.5660   1.5732   1.5844   1.5865   1.5927   1.6085   1.6105
```

| | | | | | | | |
|---|---|---|---|---|---|---|---|
| 1.6210 | 1.6411 | 1.6442 | 1.6557 | 1.6659 | 1.6715 | 1.6733 | 1.6914 |
| 1.7015 | 1.7043 | 1.7124 | 1.7365 | 1.7401 | 1.7501 | 1.7565 | 1.7715 |
| 1.7828 | 1.7904 | 1.8026 | 1.8187 | 1.8383 | 1.8531 | 1.8681 | 1.8861 |
| 1.8904 | 1.8998 | 1.9101 | 1.9161 | 1.9355 | 1.9544 | 1.9550 | 1.9807 |
| 1.9927 | 2.0047 | 2.0257 | 2.0342 | 2.0430 | 2.0606 | 2.0659 | 2.0818 |
| 2.1012 | 2.1204 | 2.1323 | 2.1530 | 2.1567 | 2.1773 | 2.2091 | 2.2185 |
| 2.2289 | 2.2327 | 2.2459 | 2.2540 | 2.2640 | 2.2882 | 2.2925 | 2.3000 |
| 2.3141 | 2.3307 | 2.3473 | 2.3626 | 2.3643 | 2.3811 | 2.3918 | 2.3960 |
| 2.4087 | 2.4131 | 2.4374 | 2.4488 | 2.4511 | 2.4634 | 2.4680 | 2.4838 |
| 2.5046 | 2.5161 | 2.5254 | 2.5371 | 2.5425 | 2.5470 | 2.5511 | 2.5632 |
| 2.5671 | 2.5776 | 2.5824 | 2.5909 | 2.5934 | 2.6031 | 2.6103 | 2.6220 |
| 2.6347 | 2.6428 | 2.6533 | 2.6623 | 2.6692 | 2.6771 | 2.6880 | 2.7034 |
| 2.7131 | 2.7170 | 2.7276 | 2.7315 | 2.7394 | 2.7637 | 2.7712 | 2.7810 |
| 2.7848 | 2.8033 | 2.8165 | 2.8283 | 2.8439 | 2.8614 | 2.8656 | 2.8848 |
| 2.8939 | 2.9225 | 2.9390 | 2.9970 | 3.0315 | 3.0716 | 3.1372 | 3.1486 |
| 3.1553 | 3.1914 | 3.2450 | 3.2503 | 3.2951 | 3.3179 | 3.3599 | 3.3908 |
| 3.4181 | 3.4769 | 3.5158 | 3.5348 | 3.5530 | 3.5922 | 3.6211 | 3.6473 |
| 3.6937 | 3.7042 | 3.7157 | 3.7191 | 3.7363 | 3.7515 | 3.7618 | 3.7694 |
| 3.8326 | 3.8672 | 3.8705 | 3.9057 | 3.9351 | 3.9965 | 4.0327 | 4.0923 |
| 4.0934 | 4.2032 | 4.2984 | 4.3128 | 4.5865 | 4.6560 | 4.6954 | 4.7225 |
| 4.8974 | 5.0349 | 5.1849 | 23.5219 | 23.5680 | 23.7163 | 23.7219 | 23.7396 |
| 23.7736 | 23.7985 | 23.8355 | 23.8567 | 23.8928 | 23.9122 | 23.9642 | 23.9698 |
| 24.0354 | 35.4198 | 35.4490 | 35.5050 | 35.6023 | | | |

Ground-State Mulliken Net Atomic Charges

| Atom | Charge (a.u.) |
|---|---|
| 1 N | 0.445295 |
| 2 C | 0.076949 |
| 3 C | -0.030555 |
| 4 C | -0.227608 |
| 5 C | -0.292075 |
| 6 C | -0.068789 |
| 7 C | -0.721187 |
| 8 C | -0.195402 |
| 9 N | -0.174680 |
| 10 C | -0.169707 |
| 11 N | 0.055711 |
| 12 C | 0.101741 |
| 13 C | -0.067168 |
| 14 C | 0.113736 |
| 15 C | -0.337380 |
| 16 C | -0.268311 |
| 17 C | -0.521535 |
| 18 N | -0.463467 |
| 19 H | 0.220936 |
| 20 H | 0.235527 |
| 21 H | 0.231485 |
| 22 H | 0.216939 |
| 23 H | 0.232698 |
| 24 H | 0.215641 |
| 25 H | 0.240065 |
| 26 H | 0.199701 |
| 27 H | 0.247156 |
| 28 H | 0.398951 |
| 29 H | 0.213834 |
| 30 H | 0.250128 |
| 31 H | 0.210328 |
| 32 H | 0.223645 |
| 33 H | 0.204091 |
| 34 H | 0.178441 |
| 35 H | 0.218087 |
| 36 H | 0.137177 |
| 37 H | 0.317400 |
| 38 H | 0.352201 |

Sum of atomic charges = 2.000000

```
 -----------------------------------------------------------------
                    Cartesian Multipole Moments
 -----------------------------------------------------------------
    Charge (ESU x 10^10)
                 9.6064
    Dipole Moment (Debye)
         X       7.5195      Y      -0.5927      Z       0.5005
       Tot       7.5595
    Quadrupole Moments (Debye-Ang)
        XX      -4.2302     XY     -12.9748     YY     -77.3639
        XZ      -7.6628     YZ      -6.6401     ZZ     -73.0473
    Traceless Quadrupole Moments (Debye-Ang)
       QXX     141.9508    QYY     -77.4502    QZZ     -64.5006
       QXY     -38.9245    QXZ     -22.9884    QYZ     -19.9204
    Octopole Moments (Debye-Ang^2)
       XXX      54.5041    XXY     -97.7465    XYY      11.7221
       YYY      23.4686    XXZ      55.9718    XYZ      20.1140
       YYZ     -20.5099    XZZ      -2.1750    YZZ       4.4294
       ZZZ       1.1674
    Traceless Octopole Moments (Debye-Ang^2)
       XXX     241.1005    YYY     980.6662    ZZZ    -312.1522
       XXY   -1256.6525    XXZ     729.6889    XYY     -16.3214
       XYZ     301.7096    XZZ    -224.7790    YYZ    -417.5367
       YZZ     275.9863
    Hexadecapole Moments (Debye-Ang^3)
      XXXX   -3743.0571   XXXY    -115.8091   XXYY    -805.7331
      XYYY     290.9283   YYYY    -598.6079   XXXZ     344.1549
      XXYZ      53.7129   XYYZ      98.9819   YYYZ     -26.7282
      XXZZ    -898.5790   XYZZ      67.8067   YYZZ    -220.8348
      XZZZ     345.0069   YZZZ      -1.6293   ZZZZ    -982.4074
    Traceless Hexadecapole Moments (Debye-Ang^3)
      XXXX   14672.9404   XXXY  -23091.6227   XXXZ     669.7967
      XXYY   -6036.8997   XXYZ    5250.0859   XXZZ   -8636.0407
      XYYY   19615.8052   XYYZ   -1429.0553   XYZZ    3475.8175
      XZZZ     759.2585   YYYY     842.6986   YYYZ   -3975.7752
      YYZZ    5194.2010   YZZZ   -1274.3107   ZZZZ    3441.8397
 -----------------------------------------------------------------

------------------------------------------------
-  Entering drvman on Sun Oct 13 21:25:48 2024  -
------------------------------------------------

 Calculating analytic gradient of the SCF energy
 Gradient of SCF Energy
            1           2           3           4           5           6
    1  -0.0008663   0.0010994  -0.0012713   0.0001897  -0.0006673   0.0003934
    2  -0.0012525   0.0010498  -0.0000791  -0.0000996   0.0004532   0.0004023
    3   0.0002648   0.0006472  -0.0009303   0.0005414  -0.0000316  -0.0008783
            7           8           9          10          11          12
    1   0.0001483  -0.0003987   0.0004367   0.0028714  -0.0011989  -0.0015989
    2  -0.0002609   0.0002908   0.0014218  -0.0016430   0.0029878  -0.0015505
    3  -0.0005841  -0.0009960  -0.0021633   0.0038399  -0.0021136   0.0087711
           13          14          15          16          17          18
    1  -0.0031810  -0.0000500  -0.0004523   0.0005849  -0.0008404   0.0115114
    2  -0.0001544  -0.0014183   0.0018959  -0.0020101   0.0003894  -0.0024436
    3  -0.0038377   0.0017058   0.0004747  -0.0014614   0.0005406  -0.0073281
           19          20          21          22          23          24
    1   0.0004038   0.0001589   0.0003670   0.0008015   0.0002296  -0.0000596
    2  -0.0004654   0.0000060  -0.0004223  -0.0004440   0.0002658   0.0000820
    3   0.0001118   0.0000863  -0.0001750   0.0004182   0.0000240  -0.0002235
           25          26          27          28          29          30
    1   0.0002383  -0.0004313  -0.0005506  -0.0006288  -0.0004486  -0.0004157
    2  -0.0003728   0.0004365   0.0000416  -0.0006545   0.0000167  -0.0007559
    3  -0.0003667   0.0004115   0.0003563   0.0000535   0.0006449   0.0003489
           31          32          33          34          35          36
    1  -0.0000450  -0.0002782   0.0001352   0.0002338  -0.0001356   0.0001531
    2  -0.0002503   0.0004941   0.0006471  -0.0001229   0.0000228  -0.0001073
    3  -0.0000442   0.0001774   0.0006924  -0.0001740  -0.0001205  -0.0000706
           37          38
    1  -0.0064694   0.0000315
```

```
    2   0.0033545   0.0002492
    3   0.0002194   0.0011686
 Max gradient component =       1.151E-02
 RMS gradient           =       1.956E-03
 Gradient time:  CPU 133.05 s  wall 213.84 s

-------------------------------------------------
-  Entering optman on Sun Oct 13 21:29:21 2024  -
-------------------------------------------------

 Geometry Optimization Parameters
   NAtoms,    NIC,     NZ,  NCons,   NDum,   NFix, NCnnct, MaxDiis
       38     272       0       0       0       0       0       0

 Cartesian Hessian Update
 Hessian updated using BFGS update


** GEOMETRY OPTIMIZATION IN DELOCALIZED INTERNAL COORDINATES **
   Searching for a Minimum

   Optimization Cycle:   3

                       Coordinates (Angstroms)
     ATOM                X               Y               Z
      1  N         2.7081290120    0.1340072416   -0.1331001938
      2  C         2.8858484459   -0.6521397773   -1.2338207170
      3  C         3.8651745621   -1.6355838534   -1.1875619275
      4  C         4.6595347140   -1.8034028904   -0.0651845805
      5  C         4.4744718278   -0.9640817057    1.0300952670
      6  C         3.4986033314   -0.0049437590    0.9639527623
      7  C         2.0378546302   -0.4660755455   -2.4510959126
      8  C         1.6875525111    1.2142751881   -0.1130672180
      9  N        -1.3991916928   -0.5514470827    1.0816767764
     10  C        -2.3102127778   -0.2451487021    0.1247528719
     11  N        -1.9875739031    0.5545844731   -0.8660029596
     12  C        -0.7541770891    1.0728683491   -0.9348675941
     13  C         0.2634378250    0.7112710533   -0.0010236026
     14  C        -0.1281044345   -0.0966703525    1.0268177102
     15  C        -3.6954367259   -0.7772171409    0.2454073244
     16  C        -4.6556341089    0.2715219335    0.8482957759
     17  C        -4.3531710087    0.6058150659    2.3055944739
     18  N        -0.5003885052    1.8689927222   -2.0031083502
     19  H         4.0085556397   -2.2650157529   -2.0567082757
     20  H         5.4253632706   -2.5702276646   -0.0464818668
     21  H         5.0886524865   -1.0439869788    1.9174775308
     22  H         3.3264948398    0.6858229596    1.7789384100
     23  H         2.4707349289   -1.0244502869   -3.2788959196
     24  H         1.9628962140    0.5780469071   -2.7594363030
     25  H         1.0255747615   -0.8466057296   -2.2875915504
     26  H         1.8199810136    1.8141881604   -1.0105382666
     27  H         1.9249596067    1.8596958946    0.7330522134
     28  H        -1.6941595307   -1.1409426692    1.8529757958
     29  H         0.5299159201   -0.4235405839    1.8237308014
     30  H        -4.0291136515   -1.0534195955   -0.7566066490
     31  H        -3.6993138704   -1.6828051581    0.8607118026
     32  H        -5.6647493878   -0.1356652260    0.7618623100
     33  H        -4.6299933629    1.1769776175    0.2355740496
     34  H        -4.3672306196   -0.2917686030    2.9320892672
     35  H        -5.1033557262    1.2906941115    2.7025676489
     36  H        -3.3805295831    1.0977179619    2.4259885045
     37  H         0.0079330126    2.7341730129   -1.8805981245
     38  H        -1.3193325754    2.0044864057   -2.5858712848
   Point Group: c1    Number of degrees of freedom:   108


   Energy is   -763.963884266

 Hessian updated using BFGS update
  internal optimization (0)
```

```
108 Hessian modes will be used to form the next step
  Hessian Eigenvalues:
     0.002193    0.002692    0.004085    0.005699    0.009845    0.010489
     0.015919    0.018915    0.019188    0.019813    0.020413    0.022230
     0.022652    0.022819    0.023917    0.024492    0.025725    0.026598
     0.028131    0.028309    0.028745    0.030430    0.033057    0.036312
     0.038754    0.041311    0.042846    0.043722    0.044361    0.046254
     0.050078    0.052789    0.053963    0.055093    0.063500    0.079157
     0.085552    0.093989    0.121639    0.122210    0.127088    0.130711
     0.131639    0.132959    0.136273    0.139163    0.142892    0.144610
     0.147679    0.148244    0.149468    0.152210    0.152648    0.152955
     0.153988    0.159821    0.192428    0.206073    0.206724    0.216584
     0.221153    0.229862    0.237992    0.247390    0.248923    0.260618
     0.267913    0.270657    0.280170    0.297595    0.300101    0.300131
     0.300667    0.301217    0.301706    0.302598    0.304110    0.304808
     0.305313    0.305434    0.307529    0.308162    0.312314    0.315958
     0.323472    0.331680    0.332436    0.334326    0.336998    0.341518
     0.344628    0.352084    0.353967    0.360753    0.380960    0.388593
     0.391995    0.401601    0.409691    0.414528    0.418749    0.421120
     0.433091    0.442121    0.450826    0.484405    0.570506    0.780359

 Minimum search - taking simple RFO step
 Searching for Lamda that Minimizes Along All modes
 Value Taken    Lamda =  -0.00537689
 Calculated Step too Large.  Step scaled by  0.542004 !!
 Step Taken.  Stepsize is  0.300000  0.013707

                             Maximum     Tolerance    Cnvgd?
         Gradient           0.004169      0.000800      NO
         Displacement       0.085689      0.001400      NO
         Energy change     -0.003763      0.000228      NO


 New Cartesian Coordinates Obtained by Inverse Iteration

 Displacement from previous Coordinates is:  0.504120
 ----------------------------------------------------------------
             Standard Nuclear Orientation (Angstroms)
    I     Atom           X                Y                Z
 ----------------------------------------------------------------
    1      N       2.7082761896     0.1382848727    -0.1315732801
    2      C       2.8725778040    -0.6665977292    -1.2197617847
    3      C       3.8536650022    -1.6466647433    -1.1664173227
    4      C       4.6544470532    -1.7992372331    -0.0468054487
    5      C       4.4738884676    -0.9476899461     1.0410671920
    6      C       3.4991366281     0.0117039121     0.9661829310
    7      C       2.0067830989    -0.4941030595    -2.4251787520
    8      C       1.6920745183     1.2198452856    -0.1240293545
    9      N      -1.3914685844    -0.5620054484     1.0546770639
   10      C      -2.3108055302    -0.2420297028     0.1061788987
   11      N      -1.9933119120     0.5739378175    -0.8654575792
   12      C      -0.7635086310     1.1029132168    -0.9356458106
   13      C       0.2674697011     0.7191988108    -0.0163138518
   14      C      -0.1199179135    -0.1047823855     0.9948994016
   15      C      -3.6910669163    -0.7822009805     0.2251377241
   16      C      -4.6491378194     0.2608067940     0.8480614018
   17      C      -4.3254416008     0.5927873718     2.3006024936
   18      N      -0.5406384775     1.9271522527    -1.9714076951
   19      H       3.9881466859    -2.2851743113    -2.0296729956
   20      H       5.4196366275    -2.5662183681    -0.0234804416
   21      H       5.0878147451    -1.0151635242     1.9292079175
   22      H       3.3249479792     0.7155781336     1.7692190359
   23      H       2.4101106060    -1.0824923382    -3.2468870385
   24      H       1.9472948398     0.5442260505    -2.7557635369
   25      H       0.9894168682    -0.8465022330    -2.2321645384
   26      H       1.8307240305     1.8078659015    -1.0287260544
   27      H       1.9323815135     1.8717259090     0.7155447634
   28      H      -1.6766534189    -1.1622998422     1.8198658388
   29      H       0.5473739087    -0.4485731137     1.7767758650
```

```
   30      H      -4.0286771161    -1.0417961159    -0.7797744956
   31      H      -3.6885849059    -1.6944309771     0.8296532190
   32      H      -5.6552371650    -0.1553435990     0.7725173461
   33      H      -4.6374097747     1.1642745253     0.2325822093
   34      H      -4.3266238219    -0.3051255132     2.9267440250
   35      H      -5.0725871452     1.2727816626     2.7112398330
   36      H      -3.3547052033     1.0909851146     2.4086200627
   37      H       0.0822772500     2.7169979032    -1.8979924280
   38      H      -1.3626675815     2.1173656300    -2.5317248141
 ----------------------------------------------------------------
 Nuclear Repulsion Energy =        1314.84213941 hartrees
 There are       65 alpha and       65 beta electrons

--------------------------------------------------
-  Entering fldman on Sun Oct 13 21:29:22 2024  -
--------------------------------------------------

 Applying Cartesian multipole field
    Component          Value
    ---------          -----
     (2,0,0)        1.00000E-12
     (0,2,0)        2.00000E-11
     (0,0,2)       -3.00000E-11
 Nucleus-field energy     =    -0.0000000098 hartrees

--------------------------------------------------
-  Entering gesman on Sun Oct 13 21:29:22 2024  -
--------------------------------------------------

 Requested basis set is 6-311+G(d,p)
 There are 188 shells and 516 basis functions
 A cutoff of  1.0D-12 yielded  12821 shell pairs
 There are    101899 function pairs (    108655 Cartesian)
 Smallest overlap matrix eigenvalue = 2.12E-06
 Linear dependence detected in AO basis
 Tighter screening thresholds may be required for diffuse basis sets
 Use S2THRESH > 12 and THRESH = 14 in case of SCF convergence issues
 Number of orthogonalized atomic orbitals = 510
 Maximum deviation from orthogonality = 1.687E-11
 Guess MOs from SCF MO coefficient file
 Reading MOs from coefficient file
 Reading MOs from coefficient file

--------------------------------------------------
-  Entering scfman on Sun Oct 13 21:29:22 2024  -
--------------------------------------------------

 Long-range K will be added via erf
 Coulomb attenuation parameter = 0.2 bohr**(-1)
 A restricted hybrid HF-DFT SCF calculation will be
 performed using Pulay DIIS + Geometric Direct Minimization
 Exchange:     0.2220 Hartree-Fock + 1.0000 wB97X-D + LR-HF
 Correlation:  1.0000 wB97X-D
 Using Euler-Maclaurin-Lebedev (75,302) quadrature formula
 Dispersion:   Grimme D
 SCF converges when RMS gradient is below 1.0E-07
 Geometry optimization detected.  Setting ReadMinima to 0
 Setting SaveMinima to 0
 ---------------------------------------
  Cycle       Energy         DIIS Error
 ---------------------------------------
    1    -763.9770198580      5.12E-04
    2    -763.9658226547      9.08E-05
    3    -763.9660607121      7.92E-05
    4    -763.9662504488      2.01E-05
    5    -763.9662674747      6.80E-06
    6    -763.9662693970      2.89E-06
    7    -763.9662697900      9.37E-07
    8    -763.9662698357      3.84E-07
    9    -763.9662698441      1.15E-07
```

```
   10    -763.9662698450      5.35E-08 Convergence criterion met
 ---------------------------------------
 SCF time:  CPU 286.17 s  wall 455.01 s
 SCF   energy in the final basis set = -763.96626984
 Total energy in the final basis set = -763.96626984

--------------------------------------------------
-  Entering anlman on Sun Oct 13 21:36:57 2024  -
--------------------------------------------------



 --------------------------------------------------------------

                   Orbital Energies (a.u.)
 --------------------------------------------------------------

 Alpha MOs
 -- Occupied --
-14.8171 -14.7941 -14.7313 -14.7100 -10.6764 -10.6642 -10.6497 -10.6395
-10.6356 -10.6266 -10.6071 -10.5841 -10.5727 -10.5713 -10.5496 -10.5281
-10.4956 -10.4703  -1.4185  -1.4054  -1.3465  -1.2759  -1.2311  -1.2008
 -1.1887  -1.1435  -1.1022  -1.0844  -1.0556  -1.0531  -1.0339  -1.0074
 -0.9904  -0.9536  -0.9466  -0.9135  -0.9053  -0.8924  -0.8816  -0.8651
 -0.8536  -0.8453  -0.8361  -0.8227  -0.8108  -0.7972  -0.7874  -0.7852
 -0.7727  -0.7546  -0.7538  -0.7500  -0.7437  -0.7296  -0.7211  -0.7174
 -0.7017  -0.6707  -0.6589  -0.6446  -0.6360  -0.6288  -0.6248  -0.6213
 -0.6134
 -- Virtual --
 -0.2700  -0.2641  -0.2471  -0.2268  -0.1743  -0.1642  -0.1504  -0.1449
 -0.1333  -0.1239  -0.1182  -0.1142  -0.1111  -0.1042  -0.0995  -0.0941
 -0.0879  -0.0862  -0.0823  -0.0805  -0.0748  -0.0703  -0.0694  -0.0649
 -0.0633  -0.0588  -0.0554  -0.0527  -0.0490  -0.0451  -0.0393  -0.0345
 -0.0321  -0.0263  -0.0262  -0.0231  -0.0211  -0.0152  -0.0130  -0.0097
 -0.0054  -0.0026   0.0016   0.0045   0.0073   0.0116   0.0137   0.0174
  0.0185   0.0232   0.0257   0.0276   0.0312   0.0363   0.0376   0.0412
  0.0440   0.0470   0.0517   0.0528   0.0533   0.0581   0.0629   0.0668
  0.0702   0.0721   0.0767   0.0784   0.0810   0.0826   0.0857   0.0881
  0.0921   0.0962   0.1006   0.1047   0.1082   0.1118   0.1163   0.1209
  0.1222   0.1239   0.1307   0.1317   0.1368   0.1404   0.1418   0.1477
  0.1507   0.1539   0.1580   0.1639   0.1661   0.1749   0.1767   0.1797
  0.1835   0.1887   0.1939   0.1970   0.2018   0.2071   0.2140   0.2179
  0.2217   0.2380   0.2526   0.2689   0.2708   0.2842   0.3098   0.3140
  0.3245   0.3285   0.3317   0.3425   0.3457   0.3504   0.3640   0.3702
  0.3763   0.3836   0.3868   0.3924   0.4004   0.4057   0.4187   0.4203
  0.4289   0.4379   0.4449   0.4451   0.4547   0.4637   0.4688   0.4788
  0.4823   0.4932   0.4975   0.5016   0.5092   0.5136   0.5192   0.5266
  0.5303   0.5327   0.5338   0.5432   0.5453   0.5515   0.5539   0.5607
  0.5643   0.5712   0.5737   0.5794   0.5822   0.5933   0.5962   0.6020
  0.6101   0.6143   0.6172   0.6309   0.6339   0.6453   0.6546   0.6564
  0.6650   0.6683   0.6756   0.6784   0.6895   0.6970   0.7040   0.7099
  0.7191   0.7251   0.7368   0.7373   0.7549   0.7664   0.7755   0.7866
  0.7890   0.8003   0.8102   0.8224   0.8328   0.8384   0.8439   0.8501
  0.8634   0.8684   0.8770   0.8826   0.9025   0.9067   0.9190   0.9290
  0.9451   0.9481   0.9664   0.9785   0.9909   1.0107   1.0165   1.0370
  1.0563   1.0630   1.0824   1.0996   1.1176   1.1510   1.1613   1.1740
  1.2039   1.2261   1.2384   1.2445   1.2541   1.2765   1.3066   1.3177
  1.3234   1.3281   1.3375   1.3422   1.3586   1.3680   1.3707   1.3769
  1.3916   1.3986   1.4034   1.4087   1.4182   1.4279   1.4298   1.4377
  1.4433   1.4460   1.4580   1.4622   1.4698   1.4831   1.4941   1.4981
  1.5045   1.5194   1.5224   1.5257   1.5354   1.5438   1.5527   1.5588
  1.5592   1.5669   1.5748   1.5852   1.5867   1.5953   1.6101   1.6125
  1.6198   1.6436   1.6442   1.6537   1.6656   1.6717   1.6739   1.6882
  1.6978   1.7063   1.7154   1.7367   1.7410   1.7493   1.7553   1.7730
  1.7832   1.7905   1.8044   1.8170   1.8399   1.8505   1.8668   1.8892
  1.8916   1.9003   1.9143   1.9233   1.9341   1.9540   1.9602   1.9850
  1.9909   2.0072   2.0256   2.0351   2.0427   2.0540   2.0715   2.0836
  2.0988   2.1235   2.1337   2.1564   2.1585   2.1799   2.2123   2.2196
  2.2290   2.2334   2.2483   2.2539   2.2597   2.2865   2.2909   2.2982
  2.3135   2.3303   2.3451   2.3611   2.3640   2.3845   2.3923   2.3958
  2.4082   2.4149   2.4356   2.4467   2.4486   2.4652   2.4667   2.4844
```

| | | | | | | | |
|---|---|---|---|---|---|---|---|
| 2.5049 | 2.5203 | 2.5247 | 2.5389 | 2.5406 | 2.5473 | 2.5503 | 2.5618 |
| 2.5691 | 2.5790 | 2.5866 | 2.5894 | 2.5947 | 2.6025 | 2.6126 | 2.6244 |
| 2.6371 | 2.6459 | 2.6566 | 2.6581 | 2.6701 | 2.6779 | 2.6842 | 2.7050 |
| 2.7165 | 2.7208 | 2.7279 | 2.7377 | 2.7411 | 2.7689 | 2.7727 | 2.7830 |
| 2.7843 | 2.8067 | 2.8131 | 2.8371 | 2.8441 | 2.8643 | 2.8666 | 2.8840 |
| 2.8951 | 2.9232 | 2.9475 | 2.9970 | 3.0363 | 3.0694 | 3.1414 | 3.1557 |
| 3.1651 | 3.2116 | 3.2482 | 3.2508 | 3.2966 | 3.3187 | 3.3632 | 3.3994 |
| 3.4193 | 3.4804 | 3.5213 | 3.5362 | 3.5553 | 3.5937 | 3.6217 | 3.6447 |
| 3.6948 | 3.7056 | 3.7193 | 3.7213 | 3.7387 | 3.7533 | 3.7638 | 3.7672 |
| 3.8354 | 3.8368 | 3.8673 | 3.9045 | 3.9379 | 3.9902 | 4.0315 | 4.0917 |
| 4.0930 | 4.2038 | 4.2972 | 4.3114 | 4.5907 | 4.6622 | 4.6994 | 4.7294 |
| 4.9091 | 5.0360 | 5.1898 | 23.5216 | 23.5732 | 23.7155 | 23.7287 | 23.7396 |
| 23.7734 | 23.7997 | 23.8352 | 23.8588 | 23.8926 | 23.9137 | 23.9672 | 23.9800 |
| 24.0347 | 35.4200 | 35.4534 | 35.5126 | 35.6128 | | | |

Ground-State Mulliken Net Atomic Charges

| Atom | Charge (a.u.) |
|---|---|
| 1 N | 0.440460 |
| 2 C | 0.083339 |
| 3 C | -0.029191 |
| 4 C | -0.232113 |
| 5 C | -0.289829 |
| 6 C | -0.067468 |
| 7 C | -0.723076 |
| 8 C | -0.186674 |
| 9 N | -0.183788 |
| 10 C | -0.168341 |
| 11 N | 0.048051 |
| 12 C | 0.127727 |
| 13 C | -0.085024 |
| 14 C | 0.113514 |
| 15 C | -0.332810 |
| 16 C | -0.273187 |
| 17 C | -0.520656 |
| 18 N | -0.460657 |
| 19 H | 0.220505 |
| 20 H | 0.236031 |
| 21 H | 0.231887 |
| 22 H | 0.217063 |
| 23 H | 0.233985 |
| 24 H | 0.214739 |
| 25 H | 0.242825 |
| 26 H | 0.189882 |
| 27 H | 0.247205 |
| 28 H | 0.398358 |
| 29 H | 0.212024 |
| 30 H | 0.249649 |
| 31 H | 0.210084 |
| 32 H | 0.223411 |
| 33 H | 0.204539 |
| 34 H | 0.178291 |
| 35 H | 0.217717 |
| 36 H | 0.138200 |
| 37 H | 0.315205 |
| 38 H | 0.358125 |

Sum of atomic charges = 2.000000

Cartesian Multipole Moments

Charge (ESU x 10^10)
9.6064
Dipole Moment (Debye)
X 7.7343 Y -0.4738 Z 0.2348
Tot 7.7523
Quadrupole Moments (Debye-Ang)

```
         XX      -4.6080     XY     -12.8066     YY     -76.7994
         XZ      -7.2160     YZ      -7.1213     ZZ     -73.2642
    Traceless Quadrupole Moments (Debye-Ang)
       QXX     140.8476    QYY     -75.7266    QZZ     -65.1210
       QXY     -38.4199    QXZ     -21.6480    QYZ     -21.3640
    Octopole Moments (Debye-Ang^2)
       XXX      56.8625    XXY     -96.8800    XYY      12.3301
       YYY      22.9728    XXZ      56.5151    XYZ      20.0718
       YYZ     -22.2659    XZZ      -1.7086    YZZ       5.7220
       ZZZ       0.5745
    Traceless Octopole Moments (Debye-Ang^2)
       XXX     245.5816    YYY     958.2591    ZZZ    -304.7953
       XXY   -1248.6451    XXZ     743.2554    XYY     -17.4999
       XYZ     301.0769    XZZ    -228.0816    YYZ    -438.4602
       YZZ     290.3860
    Hexadecapole Moments (Debye-Ang^3)
      XXXX   -3736.2524   XXXY    -111.4454   XXYY    -802.8139
      XYYY     288.6316   YYYY    -610.1593   XXXZ     345.3386
      XXYZ      51.2345   XYYZ      95.5359   YYYZ     -28.5215
      XXZZ    -894.7472   XYZZ      67.6383   YYZZ    -214.6601
      XZZZ     333.7393   YZZZ      -1.7944   ZZZZ    -969.2304
    Traceless Hexadecapole Moments (Debye-Ang^3)
      XXXX   14475.9594   XXXY  -22718.8707   XXXZ    1402.9325
      XXYY   -5794.0141   XXYZ    5053.9299   XXZZ   -8681.9453
      XYYY   19289.2209   XYYZ   -1587.9375   XYZZ    3429.6498
      XZZZ     185.0050   YYYY     159.5118   YYYZ   -3971.8419
      YYZZ    5634.5023   YZZZ   -1082.0880   ZZZZ    3047.4430
 -----------------------------------------------------------------

-------------------------------------------------
-  Entering drvman on Sun Oct 13 21:36:57 2024  -
-------------------------------------------------

 Calculating analytic gradient of the SCF energy
 Gradient of SCF Energy
           1           2           3           4           5           6
    1  -0.0001796   0.0006465  -0.0005719   0.0000225  -0.0004431   0.0004931
    2  -0.0006409   0.0000445   0.0000613  -0.0003650   0.0005629  -0.0000473
    3  -0.0002619  -0.0002074  -0.0000052  -0.0004317   0.0004935  -0.0000264
           7           8           9          10          11          12
    1   0.0004733   0.0000902   0.0000403   0.0001700   0.0003009  -0.0027998
    2   0.0003278   0.0001218   0.0010973  -0.0000969   0.0004309   0.0015306
    3  -0.0002100  -0.0000109  -0.0013792  -0.0001220   0.0011287   0.0038058
          13          14          15          16          17          18
    1  -0.0011278   0.0009670   0.0003277  -0.0001055  -0.0003703   0.0074367
    2  -0.0019491   0.0007334   0.0005000  -0.0002935  -0.0000329  -0.0046025
    3  -0.0010068  -0.0003026  -0.0001862  -0.0001291   0.0002201  -0.0040844
          19          20          21          22          23          24
    1  -0.0000805  -0.0000579  -0.0000708   0.0001844   0.0001989  -0.0004691
    2   0.0001834   0.0000434   0.0000506  -0.0001771   0.0000571  -0.0000001
    3   0.0000985  -0.0000120  -0.0001428  -0.0000424   0.0002105   0.0002561
          25          26          27          28          29          30
    1  -0.0000300  -0.0007362   0.0000407  -0.0002450  -0.0001295  -0.0001206
    2  -0.0000337  -0.0000157  -0.0004926  -0.0001574   0.0000307  -0.0003806
    3  -0.0001525   0.0001220   0.0001945  -0.0002025   0.0002607   0.0001994
          31          32          33          34          35          36
    1   0.0001286   0.0000384   0.0001475   0.0000827   0.0000561   0.0000489
    2   0.0000074   0.0002288   0.0001646   0.0000165  -0.0000411   0.0000589
    3   0.0000784  -0.0002611   0.0002700  -0.0000129  -0.0000367   0.0000831
          37          38
    1  -0.0046615   0.0003049
    2   0.0028082   0.0002661
    3   0.0004924   0.0013128
 Max gradient component =       7.437E-03
 RMS gradient           =       1.213E-03
 Gradient time:  CPU 121.06 s  wall 185.45 s

-------------------------------------------------
-  Entering optman on Sun Oct 13 21:40:02 2024  -
-------------------------------------------------
```

```
 Geometry Optimization Parameters
   NAtoms,    NIC,     NZ,  NCons,   NDum,   NFix, NCnnct, MaxDiis
       38     272       0       0       0       0       0       0

 Cartesian Hessian Update
 Hessian updated using BFGS update


** GEOMETRY OPTIMIZATION IN DELOCALIZED INTERNAL COORDINATES **
   Searching for a Minimum

   Optimization Cycle:   4

                     Coordinates (Angstroms)
     ATOM                X               Y               Z
      1  N         2.7082761896    0.1382848727   -0.1315732801
      2  C         2.8725778040   -0.6665977292   -1.2197617847
      3  C         3.8536650022   -1.6466647433   -1.1664173227
      4  C         4.6544470532   -1.7992372331   -0.0468054487
      5  C         4.4738884676   -0.9476899461    1.0410671920
      6  C         3.4991366281    0.0117039121    0.9661829310
      7  C         2.0067830989   -0.4941030595   -2.4251787520
      8  C         1.6920745183    1.2198452856   -0.1240293545
      9  N        -1.3914685844   -0.5620054484    1.0546770639
     10  C        -2.3108055302   -0.2420297028    0.1061788987
     11  N        -1.9933119120    0.5739378175   -0.8654575792
     12  C        -0.7635086310    1.1029132168   -0.9356458106
     13  C         0.2674697011    0.7191988108   -0.0163138518
     14  C        -0.1199179135   -0.1047823855    0.9948994016
     15  C        -3.6910669163   -0.7822009805    0.2251377241
     16  C        -4.6491378194    0.2608067940    0.8480614018
     17  C        -4.3254416008    0.5927873718    2.3006024936
     18  N        -0.5406384775    1.9271522527   -1.9714076951
     19  H         3.9881466859   -2.2851743113   -2.0296729956
     20  H         5.4196366275   -2.5662183681   -0.0234804416
     21  H         5.0878147451   -1.0151635242    1.9292079175
     22  H         3.3249479792    0.7155781336    1.7692190359
     23  H         2.4101106060   -1.0824923382   -3.2468870385
     24  H         1.9472948398    0.5442260505   -2.7557635369
     25  H         0.9894168682   -0.8465022330   -2.2321645384
     26  H         1.8307240305    1.8078659015   -1.0287260544
     27  H         1.9323815135    1.8717259090    0.7155447634
     28  H        -1.6766534189   -1.1622998422    1.8198658388
     29  H         0.5473739087   -0.4485731137    1.7767758650
     30  H        -4.0286771161   -1.0417961159   -0.7797744956
     31  H        -3.6885849059   -1.6944309771    0.8296532190
     32  H        -5.6552371650   -0.1553435990    0.7725173461
     33  H        -4.6374097747    1.1642745253    0.2325822093
     34  H        -4.3266238219   -0.3051255132    2.9267440250
     35  H        -5.0725871452    1.2727816626    2.7112398330
     36  H        -3.3547052033    1.0909851146    2.4086200627
     37  H         0.0822772500    2.7169979032   -1.8979924280
     38  H        -1.3626675815    2.1173656300   -2.5317248141
   Point Group: c1    Number of degrees of freedom:   108


   Energy is   -763.966269845

 Hessian updated using BFGS update
  internal optimization (0)

108 Hessian modes will be used to form the next step
  Hessian Eigenvalues:
     0.002224    0.002693    0.004092    0.005598    0.009323    0.010173
     0.015936    0.018915    0.019201    0.019799    0.020470    0.022280
     0.022686    0.022816    0.023936    0.024385    0.025698    0.026599
     0.027358    0.028263    0.028387    0.030427    0.032249    0.036318
     0.038753    0.041369    0.042883    0.043738    0.044363    0.046200
     0.049660    0.052209    0.054110    0.055093    0.063609    0.079754
```

```
    0.085576    0.093977    0.121638    0.122208    0.127121    0.130760
    0.131742    0.132977    0.136530    0.140371    0.142396    0.143846
    0.147684    0.148244    0.149732    0.152257    0.152639    0.152965
    0.154102    0.160917    0.192631    0.206167    0.206883    0.216777
    0.224413    0.229834    0.238388    0.247357    0.249026    0.261019
    0.268471    0.271334    0.282037    0.297826    0.300130    0.300419
    0.300693    0.301230    0.301709    0.302609    0.304110    0.304867
    0.305320    0.305462    0.307550    0.308157    0.312325    0.316519
    0.323400    0.331953    0.332751    0.334323    0.337038    0.341551
    0.344628    0.352133    0.354584    0.361934    0.380892    0.388600
    0.395378    0.406786    0.411814    0.417936    0.420237    0.428320
    0.433398    0.445304    0.452851    0.492792    0.572264    0.787007

 Minimum search - taking simple RFO step
 Searching for Lamda that Minimizes Along All modes
 Value Taken    Lamda =  -0.00311860
 Calculated Step too Large.  Step scaled by  0.606595
 Step Taken.  Stepsize is  0.300000

                           Maximum     Tolerance    Cnvgd?
         Gradient          0.002259     0.000800      NO
         Displacement      0.079822     0.001400      NO
         Energy change    -0.002386     0.000228      NO


 New Cartesian Coordinates Obtained by Inverse Iteration

 Displacement from previous Coordinates is:  0.514096
 ----------------------------------------------------------------
             Standard Nuclear Orientation (Angstroms)
    I     Atom           X                Y                Z
 ----------------------------------------------------------------
    1      N       2.7073285222     0.1428565043    -0.1312790213
    2      C       2.8617941565    -0.6817187332    -1.2060184812
    3      C       3.8429611971    -1.6608945560    -1.1421549382
    4      C       4.6490816934    -1.7958867387    -0.0237875695
    5      C       4.4734270945    -0.9288703563     1.0524672692
    6      C       3.4998671334     0.0310920817     0.9664243640
    7      C       1.9850517180    -0.5244779413    -2.4056947240
    8      C       1.6943602801     1.2262259111    -0.1394896871
    9      N      -1.3832648968    -0.5795342710     1.0253762848
   10      C      -2.3124146867    -0.2376891428     0.0908154675
   11      N      -2.0035894182     0.6004326366    -0.8633601817
   12      C      -0.7746920504     1.1370659252    -0.9353307764
   13      C       0.2685588525     0.7272789578    -0.0342893220
   14      C      -0.1119954373    -0.1207444665     0.9603953140
   15      C      -3.6904172526    -0.7839097398     0.2088411595
   16      C      -4.6471651344     0.2490861219     0.8517726567
   17      C      -4.3013814235     0.5810409254     2.2989079491
   18      N      -0.5769818251     1.9979990152    -1.9351145438
   19      H       3.9716925605    -2.3132624830    -1.9958659820
   20      H       5.4135608555    -2.5631852130     0.0075929943
   21      H       5.0887852577    -0.9842981051     1.9404440625
   22      H       3.3267693427     0.7483298692     1.7578998107
   23      H       2.3648124211    -1.1425340459    -3.2166975442
   24      H       1.9463062402     0.5062916137    -2.7635924741
   25      H       0.9626459360    -0.8485238957    -2.1918172926
   26      H       1.8429481124     1.8027269465    -1.0504199257
   27      H       1.9332843058     1.8874736385     0.6931045275
   28      H      -1.6585899956    -1.1951883017     1.7812215868
   29      H       0.5655232772    -0.4836374404     1.7245155053
   30      H      -4.0324087904    -1.0269741345    -0.7985860735
   31      H      -3.6824942685    -1.7046393531     0.7998761206
   32      H      -5.6502123929    -0.1768394328     0.7918110927
   33      H      -4.6531858937     1.1523414839     0.2361432215
   34      H      -4.2862718194    -0.3176951917     2.9237577427
   35      H      -5.0463966786     1.2547574762     2.7235479814
   36      H      -3.3328705240     1.0863193973     2.3917338654
   37      H       0.1439600205     2.7007577580    -1.9182434332
   38      H      -1.3983864894     2.2384272801    -2.4749070062
```

```
 --------------------------------------------------------------
 Nuclear Repulsion Energy =        1315.18944580 hartrees
 There are       65 alpha and       65 beta electrons

--------------------------------------------------
-  Entering fldman on Sun Oct 13 21:40:02 2024  -
--------------------------------------------------

 Applying Cartesian multipole field
    Component          Value
    ---------          -----
     (2,0,0)        1.00000E-12
     (0,2,0)        2.00000E-11
     (0,0,2)       -3.00000E-11
 Nucleus-field energy     =    -0.0000000091 hartrees

--------------------------------------------------
-  Entering gesman on Sun Oct 13 21:40:02 2024  -
--------------------------------------------------

 Requested basis set is 6-311+G(d,p)
 There are 188 shells and 516 basis functions
 A cutoff of  1.0D-12 yielded  12833 shell pairs
 There are    101970 function pairs (    108724 Cartesian)
 Smallest overlap matrix eigenvalue = 2.14E-06
 Linear dependence detected in AO basis
 Tighter screening thresholds may be required for diffuse basis sets
 Use S2THRESH > 12 and THRESH = 14 in case of SCF convergence issues
 Number of orthogonalized atomic orbitals = 510
 Maximum deviation from orthogonality = 4.305E-11
 Guess MOs from SCF MO coefficient file
 Reading MOs from coefficient file
 Reading MOs from coefficient file

--------------------------------------------------
-  Entering scfman on Sun Oct 13 21:40:03 2024  -
--------------------------------------------------

 Long-range K will be added via erf
 Coulomb attenuation parameter = 0.2 bohr**(-1)
 A restricted hybrid HF-DFT SCF calculation will be
 performed using Pulay DIIS + Geometric Direct Minimization
 Exchange:     0.2220 Hartree-Fock + 1.0000 wB97X-D + LR-HF
 Correlation:  1.0000 wB97X-D
 Using Euler-Maclaurin-Lebedev (75,302) quadrature formula
 Dispersion:   Grimme D
 SCF converges when RMS gradient is below 1.0E-07
 Geometry optimization detected.  Setting ReadMinima to 0
 Setting SaveMinima to 0
 ---------------------------------------
  Cycle       Energy         DIIS Error
 ---------------------------------------
    1    -763.9629820862      5.28E-04
    2    -763.9673205158      7.81E-05
    3    -763.9676100526      5.46E-05
    4    -763.9677011767      2.29E-05
    5    -763.9677210816      5.52E-06
    6    -763.9677224253      2.44E-06
    7    -763.9677226847      7.86E-07
    8    -763.9677227163      3.04E-07
    9    -763.9677227212      9.17E-08 Convergence criterion met
 ---------------------------------------
 SCF time:  CPU 256.64 s  wall 412.44 s
 SCF   energy in the final basis set = -763.96772272
 Total energy in the final basis set = -763.96772272

--------------------------------------------------
-  Entering anlman on Sun Oct 13 21:46:55 2024  -
--------------------------------------------------
```

```
 --------------------------------------------------------------

                   Orbital Energies (a.u.)
 --------------------------------------------------------------

 Alpha MOs
 -- Occupied --
-14.8177 -14.7915 -14.7347 -14.7081 -10.6768 -10.6627 -10.6483 -10.6403
-10.6366 -10.6272 -10.6066 -10.5849 -10.5734 -10.5722 -10.5513 -10.5269
-10.4945 -10.4695  -1.4163  -1.4062  -1.3473  -1.2756  -1.2313  -1.2002
 -1.1897  -1.1428  -1.1017  -1.0855  -1.0547  -1.0526  -1.0347  -1.0067
 -0.9910  -0.9540  -0.9461  -0.9151  -0.9061  -0.8925  -0.8824  -0.8644
 -0.8553  -0.8460  -0.8349  -0.8234  -0.8108  -0.7980  -0.7880  -0.7859
 -0.7721  -0.7560  -0.7549  -0.7502  -0.7451  -0.7304  -0.7208  -0.7181
 -0.7006  -0.6697  -0.6594  -0.6437  -0.6346  -0.6277  -0.6240  -0.6220
 -0.6113
 -- Virtual --
 -0.2706  -0.2613  -0.2450  -0.2274  -0.1741  -0.1648  -0.1501  -0.1448
 -0.1340  -0.1237  -0.1181  -0.1141  -0.1113  -0.1044  -0.1000  -0.0949
 -0.0886  -0.0860  -0.0824  -0.0802  -0.0745  -0.0704  -0.0693  -0.0653
 -0.0636  -0.0585  -0.0553  -0.0528  -0.0485  -0.0452  -0.0395  -0.0345
 -0.0322  -0.0276  -0.0263  -0.0233  -0.0211  -0.0144  -0.0127  -0.0100
 -0.0059  -0.0026   0.0017   0.0047   0.0070   0.0115   0.0131   0.0168
  0.0189   0.0230   0.0251   0.0278   0.0319   0.0363   0.0386   0.0409
  0.0443   0.0468   0.0518   0.0532   0.0542   0.0584   0.0628   0.0661
  0.0716   0.0727   0.0762   0.0780   0.0808   0.0824   0.0857   0.0886
  0.0915   0.0973   0.1002   0.1072   0.1085   0.1107   0.1168   0.1206
  0.1220   0.1257   0.1306   0.1327   0.1386   0.1406   0.1422   0.1483
  0.1521   0.1537   0.1581   0.1634   0.1661   0.1748   0.1769   0.1797
  0.1843   0.1911   0.1949   0.1982   0.2031   0.2099   0.2141   0.2173
  0.2230   0.2368   0.2537   0.2688   0.2709   0.2811   0.3099   0.3147
  0.3251   0.3292   0.3309   0.3427   0.3458   0.3503   0.3641   0.3721
  0.3778   0.3842   0.3870   0.3926   0.4023   0.4065   0.4188   0.4200
  0.4302   0.4370   0.4448   0.4464   0.4557   0.4629   0.4680   0.4783
  0.4804   0.4932   0.4981   0.5014   0.5101   0.5133   0.5201   0.5289
  0.5300   0.5326   0.5345   0.5440   0.5447   0.5499   0.5541   0.5593
  0.5629   0.5739   0.5745   0.5809   0.5840   0.5958   0.6018   0.6082
  0.6105   0.6124   0.6197   0.6316   0.6336   0.6482   0.6546   0.6582
  0.6636   0.6698   0.6749   0.6806   0.6881   0.6986   0.7036   0.7102
  0.7208   0.7224   0.7347   0.7374   0.7537   0.7659   0.7742   0.7852
  0.7907   0.8002   0.8120   0.8244   0.8375   0.8388   0.8484   0.8497
  0.8624   0.8709   0.8791   0.8828   0.9020   0.9079   0.9213   0.9301
  0.9467   0.9493   0.9709   0.9780   0.9914   1.0111   1.0193   1.0372
  1.0581   1.0626   1.0847   1.0927   1.1179   1.1525   1.1594   1.1758
  1.2027   1.2251   1.2344   1.2398   1.2556   1.2747   1.3049   1.3139
  1.3218   1.3276   1.3361   1.3440   1.3600   1.3678   1.3717   1.3802
  1.3875   1.3977   1.4033   1.4060   1.4195   1.4273   1.4289   1.4370
  1.4441   1.4484   1.4572   1.4621   1.4706   1.4834   1.4953   1.4981
  1.5034   1.5199   1.5206   1.5262   1.5362   1.5448   1.5538   1.5578
  1.5590   1.5677   1.5757   1.5858   1.5871   1.5964   1.6103   1.6136
  1.6190   1.6402   1.6448   1.6507   1.6635   1.6712   1.6749   1.6825
  1.6949   1.7065   1.7144   1.7323   1.7391   1.7505   1.7561   1.7733
  1.7829   1.7904   1.8080   1.8156   1.8401   1.8466   1.8658   1.8898
  1.8925   1.9000   1.9140   1.9285   1.9409   1.9540   1.9698   1.9874
  1.9927   2.0089   2.0259   2.0358   2.0416   2.0475   2.0754   2.0863
  2.0967   2.1239   2.1348   2.1586   2.1604   2.1813   2.2156   2.2202
  2.2294   2.2335   2.2497   2.2536   2.2550   2.2834   2.2877   2.2958
  2.3124   2.3286   2.3419   2.3587   2.3639   2.3869   2.3872   2.3963
  2.4068   2.4159   2.4351   2.4432   2.4467   2.4632   2.4680   2.4867
  2.5050   2.5219   2.5249   2.5338   2.5407   2.5471   2.5496   2.5611
  2.5704   2.5787   2.5868   2.5907   2.5948   2.6024   2.6124   2.6227
  2.6373   2.6461   2.6582   2.6633   2.6708   2.6777   2.6839   2.7045
  2.7176   2.7242   2.7289   2.7385   2.7499   2.7717   2.7727   2.7815
  2.7854   2.8098   2.8105   2.8430   2.8485   2.8617   2.8698   2.8829
  2.8953   2.9234   2.9555   2.9965   3.0387   3.0672   3.1411   3.1572
  3.1679   3.2273   3.2492   3.2582   3.2980   3.3187   3.3633   3.4052
  3.4192   3.4806   3.5236   3.5366   3.5564   3.5939   3.6202   3.6436
  3.6944   3.7074   3.7185   3.7257   3.7405   3.7535   3.7616   3.7666
  3.7999   3.8389   3.8666   3.9032   3.9386   3.9865   4.0299   4.0902
```

```
 4.0923   4.2051   4.2958   4.3096   4.5930   4.6653   4.6987   4.7342
 4.9174   5.0358   5.1918  23.5209  23.5767  23.7136  23.7295  23.7383
23.7724  23.8003  23.8346  23.8595  23.8920  23.9149  23.9660  23.9781
24.0337  35.4190  35.4566  35.5171  35.6172
--------------------------------------------------------------

         Ground-State Mulliken Net Atomic Charges

     Atom                 Charge (a.u.)
  ----------------------------------------
      1 N                     0.433420
      2 C                     0.097235
      3 C                    -0.029860
      4 C                    -0.233561
      5 C                    -0.287169
      6 C                    -0.065651
      7 C                    -0.731023
      8 C                    -0.177238
      9 N                    -0.184748
     10 C                    -0.180006
     11 N                     0.042060
     12 C                     0.161392
     13 C                    -0.103010
     14 C                     0.108727
     15 C                    -0.326049
     16 C                    -0.279227
     17 C                    -0.518303
     18 N                    -0.466481
     19 H                     0.220464
     20 H                     0.236365
     21 H                     0.232270
     22 H                     0.217662
     23 H                     0.234970
     24 H                     0.214387
     25 H                     0.244766
     26 H                     0.182081
     27 H                     0.247393
     28 H                     0.397571
     29 H                     0.210965
     30 H                     0.249599
     31 H                     0.209071
     32 H                     0.223128
     33 H                     0.205327
     34 H                     0.177634
     35 H                     0.217292
     36 H                     0.139048
     37 H                     0.315069
     38 H                     0.364431
  ----------------------------------------
  Sum of atomic charges =     2.000000

-----------------------------------------------------------------
                    Cartesian Multipole Moments
-----------------------------------------------------------------
    Charge (ESU x 10^10)
                9.6064
    Dipole Moment (Debye)
         X       7.9289      Y      -0.4156      Z       0.0079
       Tot       7.9398
    Quadrupole Moments (Debye-Ang)
        XX      -4.9007     XY     -12.6493     YY     -76.2147
        XZ      -6.8337     YZ      -7.5806     ZZ     -73.5533
    Traceless Quadrupole Moments (Debye-Ang)
       QXX     139.9666    QYY     -73.9755    QZZ     -65.9911
       QXY     -37.9480    QXZ     -20.5010    QYZ     -22.7419
    Octopole Moments (Debye-Ang^2)
       XXX      59.2091    XXY     -96.0926    XYY      12.7998
       YYY      22.0604    XXZ      57.3100    XYZ      20.0305
       YYZ     -23.8388    XZZ      -1.0229    YZZ       6.7469
       ZZZ      -0.2163
```

```
    Traceless Octopole Moments (Debye-Ang^2)
       XXX     249.2623    YYY     936.4737    ZZZ    -302.5386
       XXY   -1239.5332    XXZ     759.8854    XYY     -20.9609
       XYZ     300.4582    XZZ    -228.3015    YYZ    -457.3468
       YZZ     303.0595
    Hexadecapole Moments (Debye-Ang^3)
      XXXX   -3732.6148   XXXY    -106.3136   XXYY    -800.6920
      XYYY     285.8068   YYYY    -624.7561   XXXZ     347.3005
      XXYZ      48.7987   XYYZ      93.1324   YYYZ     -29.9236
      XXZZ    -892.2600   XYZZ      68.0606   YYZZ    -209.5339
      XZZZ     323.9298   YZZZ      -1.0726   ZZZZ    -957.6895
    Traceless Hexadecapole Moments (Debye-Ang^3)
      XXXX   14296.1698   XXXY  -22302.8474   XXXZ    2070.2279
      XXYY   -5524.5229   XXYZ    4855.7407   XXZZ   -8771.6469
      XYYY   18869.7930   XYYZ   -1686.5357   XYZZ    3433.0544
      XZZZ    -383.6923   YYYY    -531.3028   YYYZ   -3946.3616
      YYZZ    6055.8257   YZZZ    -909.3791   ZZZZ    2715.8212
 -----------------------------------------------------------------

--------------------------------------------------
-  Entering drvman on Sun Oct 13 21:46:55 2024  -
--------------------------------------------------

 Calculating analytic gradient of the SCF energy
 Gradient of SCF Energy
            1           2           3           4           5           6
    1   0.0002109   0.0002858  -0.0001430   0.0000037  -0.0002743   0.0004607
    2  -0.0002806  -0.0002172   0.0000300  -0.0002959   0.0004249  -0.0003746
    3  -0.0004566  -0.0004191   0.0003135  -0.0004576   0.0004380   0.0002065
            7           8           9          10          11          12
    1   0.0002434   0.0003834   0.0001118  -0.0014507   0.0008342  -0.0027769
    2   0.0004613   0.0001789   0.0003788  -0.0006123  -0.0002999   0.0034989
    3   0.0001753   0.0002553  -0.0000839  -0.0008392   0.0007935   0.0007672
           13          14          15          16          17          18
    1   0.0005474   0.0005075   0.0003511  -0.0003567  -0.0001214   0.0042126
    2  -0.0011221  -0.0000069  -0.0001689   0.0001849  -0.0000953  -0.0040661
    3  -0.0006955   0.0008286  -0.0003688   0.0003994   0.0000727  -0.0031027
           19          20          21          22          23          24
    1  -0.0001715  -0.0001280  -0.0001676  -0.0000852   0.0000686  -0.0005270
    2   0.0002967   0.0000638   0.0001419  -0.0000096   0.0000811   0.0000402
    3   0.0000388  -0.0000389  -0.0000828  -0.0001878   0.0002092   0.0002286
           25          26          27          28          29          30
    1  -0.0000807  -0.0006018   0.0002251   0.0000482  -0.0000070   0.0000452
    2   0.0000221  -0.0001015  -0.0005481   0.0001115   0.0000231  -0.0001044
    3  -0.0001341  -0.0000223   0.0001269  -0.0001760   0.0000395   0.0001669
           31          32          33          34          35          36
    1   0.0001136   0.0001010   0.0001513  -0.0000055   0.0000993  -0.0000033
    2   0.0000982   0.0001158  -0.0000398   0.0000351  -0.0000461   0.0001116
    3   0.0001256  -0.0003251   0.0000501   0.0000128   0.0000150   0.0001205
           37          38
    1  -0.0024731   0.0003693
    2   0.0016424   0.0004479
    3   0.0005770   0.0014295
 Max gradient component =       4.213E-03
 RMS gradient           =       8.812E-04
 Gradient time:  CPU 119.56 s  wall 196.18 s

--------------------------------------------------
-  Entering optman on Sun Oct 13 21:50:11 2024  -
--------------------------------------------------

 Geometry Optimization Parameters
   NAtoms,    NIC,     NZ,  NCons,   NDum,   NFix, NCnnct, MaxDiis
       38     272       0       0       0       0       0       0

 Cartesian Hessian Update
 Hessian updated using BFGS update


** GEOMETRY OPTIMIZATION IN DELOCALIZED INTERNAL COORDINATES **
```

```
   Searching for a Minimum

   Optimization Cycle:   5

                       Coordinates (Angstroms)
     ATOM                X                Y                Z
      1  N         2.7073285222    0.1428565043   -0.1312790213
      2  C         2.8617941565   -0.6817187332   -1.2060184812
      3  C         3.8429611971   -1.6608945560   -1.1421549382
      4  C         4.6490816934   -1.7958867387   -0.0237875695
      5  C         4.4734270945   -0.9288703563    1.0524672692
      6  C         3.4998671334    0.0310920817    0.9664243640
      7  C         1.9850517180   -0.5244779413   -2.4056947240
      8  C         1.6943602801    1.2262259111   -0.1394896871
      9  N        -1.3832648968   -0.5795342710    1.0253762848
     10  C        -2.3124146867   -0.2376891428    0.0908154675
     11  N        -2.0035894182    0.6004326366   -0.8633601817
     12  C        -0.7746920504    1.1370659252   -0.9353307764
     13  C         0.2685588525    0.7272789578   -0.0342893220
     14  C        -0.1119954373   -0.1207444665    0.9603953140
     15  C        -3.6904172526   -0.7839097398    0.2088411595
     16  C        -4.6471651344    0.2490861219    0.8517726567
     17  C        -4.3013814235    0.5810409254    2.2989079491
     18  N        -0.5769818251    1.9979990152   -1.9351145438
     19  H         3.9716925605   -2.3132624830   -1.9958659820
     20  H         5.4135608555   -2.5631852130    0.0075929943
     21  H         5.0887852577   -0.9842981051    1.9404440625
     22  H         3.3267693427    0.7483298692    1.7578998107
     23  H         2.3648124211   -1.1425340459   -3.2166975442
     24  H         1.9463062402    0.5062916137   -2.7635924741
     25  H         0.9626459360   -0.8485238957   -2.1918172926
     26  H         1.8429481124    1.8027269465   -1.0504199257
     27  H         1.9332843058    1.8874736385    0.6931045275
     28  H        -1.6585899956   -1.1951883017    1.7812215868
     29  H         0.5655232772   -0.4836374404    1.7245155053
     30  H        -4.0324087904   -1.0269741345   -0.7985860735
     31  H        -3.6824942685   -1.7046393531    0.7998761206
     32  H        -5.6502123929   -0.1768394328    0.7918110927
     33  H        -4.6531858937    1.1523414839    0.2361432215
     34  H        -4.2862718194   -0.3176951917    2.9237577427
     35  H        -5.0463966786    1.2547574762    2.7235479814
     36  H        -3.3328705240    1.0863193973    2.3917338654
     37  H         0.1439600205    2.7007577580   -1.9182434332
     38  H        -1.3983864894    2.2384272801   -2.4749070062
   Point Group: c1    Number of degrees of freedom:   108


   Energy is   -763.967722721

 Hessian updated using BFGS update
  internal optimization (0)

108 Hessian modes will be used to form the next step
  Hessian Eigenvalues:
     0.002210    0.002693    0.004102    0.005395    0.009777    0.010401
     0.016240    0.018915    0.019189    0.019801    0.020491    0.022249
     0.022720    0.022817    0.023949    0.024051    0.025590    0.026344
     0.026613    0.028257    0.028370    0.030426    0.031829    0.036311
     0.038756    0.041453    0.042886    0.043745    0.044363    0.046142
     0.049225    0.051917    0.054109    0.055104    0.063716    0.079907
     0.085588    0.093978    0.121664    0.122207    0.127124    0.130760
     0.131768    0.132982    0.136421    0.140427    0.142311    0.143586
     0.147686    0.148281    0.149747    0.152294    0.152683    0.152993
     0.154129    0.160910    0.192683    0.206253    0.206905    0.216793
     0.224538    0.229811    0.238435    0.247491    0.249848    0.260919
     0.268648    0.271629    0.282324    0.298005    0.300137    0.300480
     0.300705    0.301232    0.301709    0.302685    0.304110    0.304908
     0.305330    0.305478    0.307569    0.308190    0.312336    0.316782
     0.323323    0.331949    0.333099    0.334458    0.337045    0.341640
     0.344997    0.352279    0.354605    0.361963    0.380813    0.389253
```

```
    0.395844    0.406327    0.412543    0.418034    0.420040    0.430182
    0.438926    0.445297    0.453036    0.492384    0.572227    0.788599

 Minimum search - taking simple RFO step
 Searching for Lamda that Minimizes Along All modes
 Value Taken    Lamda =  -0.00140118
 Calculated Step too Large.  Step scaled by  0.792091
 Step Taken.  Stepsize is  0.300000

                        Maximum     Tolerance    Cnvgd?
        Gradient        0.002386      0.000800      NO
        Displacement    0.093759      0.001400      NO
        Energy change  -0.001453      0.000228      NO


 New Cartesian Coordinates Obtained by Inverse Iteration

 Displacement from previous Coordinates is:  0.598081
 ----------------------------------------------------------------
            Standard Nuclear Orientation (Angstroms)
    I     Atom           X                Y                Z
 ----------------------------------------------------------------
    1      N       2.7046077552     0.1450470502    -0.1331915310
    2      C       2.8589721489    -0.6997353033    -1.1926813645
    3      C       3.8422711938    -1.6757807994    -1.1116537318
    4      C       4.6482109552    -1.7896823102     0.0093519258
    5      C       4.4699636823    -0.9049043029     1.0703158901
    6      C       3.4946961773     0.0533449153     0.9676343399
    7      C       1.9824783266    -0.5590807217    -2.3951995511
    8      C       1.6912390742     1.2262617027    -0.1651330781
    9      N      -1.3755478931    -0.6102411231     0.9820101604
   10      C      -2.3187720549    -0.2312870503     0.0750757717
   11      N      -2.0220848268     0.6340437194    -0.8552498850
   12      C      -0.7908741307     1.1680787095    -0.9321323890
   13      C       0.2661286589     0.7251955564    -0.0605100096
   14      C      -0.1037272362    -0.1540360081     0.9082942747
   15      C      -3.6965951270    -0.7784811431     0.1952838694
   16      C      -4.6468885064     0.2445568588     0.8624153117
   17      C      -4.2720630750     0.5752770850     2.3023898452
   18      N      -0.6226241237     2.0806329169    -1.8809416040
   19      H       3.9724183584    -2.3433004153    -1.9534895744
   20      H       5.4138889930    -2.5550274695     0.0543973558
   21      H       5.0836815979    -0.9453245270     1.9603810977
   22      H       3.3183366366     0.7836533507     1.7464381301
   23      H       2.3364497556    -1.2188948418    -3.1847111467
   24      H       1.9812978447     0.4597259434    -2.7896764552
   25      H       0.9502969501    -0.8378878116    -2.1667727973
   26      H       1.8496573665     1.7861563597    -1.0850286606
   27      H       1.9244180081     1.9037057103     0.6562112174
   28      H      -1.6395281888    -1.2488273880     1.7223084375
   29      H       0.5855811333    -0.5444040815     1.6479001672
   30      H      -4.0467073302    -1.0024852559    -0.8138813437
   31      H      -3.6851333064    -1.7095129594     0.7697279174
   32      H      -5.6473206666    -0.1900164911     0.8237111045
   33      H      -4.6737025099     1.1486663195     0.2487349620
   34      H      -4.2394856369    -0.3245724732     2.9250994309
   35      H      -5.0114022335     1.2441663372     2.7442529455
   36      H      -3.3042596915     1.0849182170     2.3754008159
   37      H       0.1643317037     2.7056575878    -1.9206367037
   38      H      -1.4422097830     2.3543941367    -2.4064451455
 ----------------------------------------------------------------
 Nuclear Repulsion Energy =        1315.34461526 hartrees
 There are       65 alpha and       65 beta electrons

 --------------------------------------------------
 -  Entering fldman on Sun Oct 13 21:50:11 2024  -
 --------------------------------------------------

 Applying Cartesian multipole field
    Component          Value
```

```
    ---------          -----
     (2,0,0)        1.00000E-12
     (0,2,0)        2.00000E-11
     (0,0,2)       -3.00000E-11
 Nucleus-field energy     =    -0.0000000083 hartrees

--------------------------------------------------
-  Entering gesman on Sun Oct 13 21:50:11 2024  -
--------------------------------------------------

 Requested basis set is 6-311+G(d,p)
 There are 188 shells and 516 basis functions
 A cutoff of  1.0D-12 yielded  12845 shell pairs
 There are    102079 function pairs (    108836 Cartesian)
 Smallest overlap matrix eigenvalue = 2.15E-06
 Linear dependence detected in AO basis
 Tighter screening thresholds may be required for diffuse basis sets
 Use S2THRESH > 12 and THRESH = 14 in case of SCF convergence issues
 Number of orthogonalized atomic orbitals = 510
 Maximum deviation from orthogonality = 1.187E-11
 Guess MOs from SCF MO coefficient file
 Reading MOs from coefficient file
 Reading MOs from coefficient file

--------------------------------------------------
-  Entering scfman on Sun Oct 13 21:50:12 2024  -
--------------------------------------------------

 Long-range K will be added via erf
 Coulomb attenuation parameter = 0.2 bohr**(-1)
 A restricted hybrid HF-DFT SCF calculation will be
 performed using Pulay DIIS + Geometric Direct Minimization
 Exchange:     0.2220 Hartree-Fock + 1.0000 wB97X-D + LR-HF
 Correlation:  1.0000 wB97X-D
 Using Euler-Maclaurin-Lebedev (75,302) quadrature formula
 Dispersion:   Grimme D
 SCF converges when RMS gradient is below 1.0E-07
 Geometry optimization detected.  Setting ReadMinima to 0
 Setting SaveMinima to 0
 ---------------------------------------
  Cycle       Energy         DIIS Error
 ---------------------------------------
    1    -763.9695668914      6.55E-04
    2    -763.9680225660      8.20E-05
    3    -763.9684199302      5.29E-05
    4    -763.9685003367      2.79E-05
    5    -763.9685299309      4.78E-06
    6    -763.9685311120      2.09E-06
    7    -763.9685313090      6.57E-07
    8    -763.9685313365      2.78E-07
    9    -763.9685313402      9.54E-08 Convergence criterion met
 ---------------------------------------
 SCF time:  CPU 263.66 s  wall 412.30 s
 SCF   energy in the final basis set = -763.96853134
 Total energy in the final basis set = -763.96853134

--------------------------------------------------
-  Entering anlman on Sun Oct 13 21:57:04 2024  -
--------------------------------------------------



 --------------------------------------------------------------

                   Orbital Energies (a.u.)
 --------------------------------------------------------------

 Alpha MOs
 -- Occupied --
-14.8184 -14.7895 -14.7366 -14.7063 -10.6766 -10.6611 -10.6469 -10.6411
-10.6373 -10.6281 -10.6061 -10.5855 -10.5741 -10.5730 -10.5526 -10.5258
```

```
-10.4933 -10.4687  -1.4153  -1.4069  -1.3480  -1.2755  -1.2317  -1.2002
 -1.1904  -1.1423  -1.1016  -1.0862  -1.0544  -1.0521  -1.0354  -1.0061
 -0.9914  -0.9547  -0.9459  -0.9165  -0.9067  -0.8923  -0.8832  -0.8638
 -0.8565  -0.8467  -0.8343  -0.8240  -0.8108  -0.7985  -0.7885  -0.7865
 -0.7717  -0.7571  -0.7554  -0.7505  -0.7456  -0.7311  -0.7210  -0.7181
 -0.6994  -0.6690  -0.6599  -0.6430  -0.6334  -0.6265  -0.6237  -0.6221
 -0.6097
 -- Virtual --
 -0.2713  -0.2593  -0.2428  -0.2279  -0.1741  -0.1652  -0.1500  -0.1448
 -0.1343  -0.1234  -0.1181  -0.1141  -0.1115  -0.1045  -0.1005  -0.0956
 -0.0892  -0.0856  -0.0826  -0.0798  -0.0744  -0.0704  -0.0693  -0.0657
 -0.0638  -0.0583  -0.0553  -0.0527  -0.0482  -0.0459  -0.0400  -0.0345
 -0.0320  -0.0291  -0.0267  -0.0232  -0.0205  -0.0143  -0.0123  -0.0094
 -0.0062  -0.0028   0.0018   0.0044   0.0067   0.0115   0.0119   0.0165
  0.0192   0.0230   0.0250   0.0282   0.0326   0.0367   0.0394   0.0411
  0.0447   0.0469   0.0519   0.0528   0.0558   0.0590   0.0622   0.0648
  0.0717   0.0729   0.0770   0.0778   0.0804   0.0824   0.0874   0.0894
  0.0912   0.0982   0.1004   0.1079   0.1103   0.1105   0.1170   0.1203
  0.1211   0.1286   0.1303   0.1347   0.1395   0.1416   0.1433   0.1489
  0.1529   0.1548   0.1582   0.1632   0.1661   0.1732   0.1772   0.1812
  0.1846   0.1923   0.1952   0.1997   0.2038   0.2121   0.2159   0.2180
  0.2297   0.2353   0.2552   0.2668   0.2731   0.2802   0.3106   0.3157
  0.3259   0.3291   0.3328   0.3440   0.3460   0.3507   0.3646   0.3735
  0.3788   0.3846   0.3889   0.3931   0.4040   0.4069   0.4177   0.4224
  0.4308   0.4368   0.4446   0.4461   0.4567   0.4643   0.4683   0.4770
  0.4793   0.4931   0.4989   0.5004   0.5116   0.5138   0.5206   0.5273
  0.5292   0.5325   0.5358   0.5431   0.5470   0.5475   0.5560   0.5580
  0.5619   0.5741   0.5756   0.5796   0.5885   0.5952   0.6040   0.6094
  0.6096   0.6197   0.6281   0.6316   0.6349   0.6528   0.6548   0.6603
  0.6622   0.6701   0.6741   0.6827   0.6881   0.6983   0.7007   0.7133
  0.7226   0.7272   0.7331   0.7413   0.7539   0.7665   0.7751   0.7834
  0.7919   0.7985   0.8117   0.8262   0.8391   0.8406   0.8499   0.8572
  0.8622   0.8742   0.8820   0.8851   0.9013   0.9103   0.9261   0.9318
  0.9470   0.9560   0.9754   0.9785   0.9935   1.0120   1.0228   1.0358
  1.0574   1.0602   1.0857   1.0880   1.1192   1.1495   1.1588   1.1775
  1.2022   1.2157   1.2286   1.2343   1.2577   1.2757   1.3029   1.3105
  1.3189   1.3269   1.3350   1.3446   1.3599   1.3680   1.3735   1.3738
  1.3856   1.3949   1.4031   1.4085   1.4195   1.4264   1.4325   1.4359
  1.4450   1.4512   1.4570   1.4614   1.4703   1.4836   1.4961   1.4993
  1.5035   1.5174   1.5224   1.5277   1.5379   1.5448   1.5540   1.5576
  1.5599   1.5687   1.5768   1.5827   1.5905   1.5987   1.6084   1.6127
  1.6200   1.6361   1.6447   1.6477   1.6610   1.6699   1.6771   1.6777
  1.6944   1.7065   1.7101   1.7307   1.7371   1.7499   1.7580   1.7741
  1.7832   1.7904   1.8109   1.8153   1.8398   1.8465   1.8662   1.8894
  1.8918   1.9014   1.9153   1.9312   1.9537   1.9572   1.9787   1.9884
  1.9985   2.0106   2.0271   2.0345   2.0402   2.0481   2.0774   2.0879
  2.0991   2.1235   2.1353   2.1602   2.1628   2.1836   2.2195   2.2218
  2.2299   2.2344   2.2523   2.2530   2.2544   2.2770   2.2865   2.2928
  2.3115   2.3263   2.3384   2.3547   2.3641   2.3791   2.3907   2.3972
  2.4059   2.4171   2.4366   2.4416   2.4475   2.4622   2.4694   2.4917
  2.5055   2.5168   2.5258   2.5301   2.5406   2.5469   2.5487   2.5620
  2.5720   2.5775   2.5855   2.5926   2.5947   2.6018   2.6119   2.6221
  2.6387   2.6510   2.6592   2.6684   2.6729   2.6775   2.6890   2.7043
  2.7179   2.7227   2.7321   2.7483   2.7568   2.7733   2.7759   2.7788
  2.7929   2.8095   2.8142   2.8443   2.8525   2.8670   2.8741   2.8829
  2.8972   2.9267   2.9642   2.9993   3.0422   3.0655   3.1406   3.1620
  3.1703   3.2325   3.2503   3.2692   3.3020   3.3185   3.3656   3.4102
  3.4212   3.4821   3.5266   3.5372   3.5577   3.5945   3.6180   3.6422
  3.6929   3.7097   3.7153   3.7305   3.7422   3.7534   3.7569   3.7677
  3.7730   3.8407   3.8658   3.9023   3.9406   3.9862   4.0291   4.0897
  4.0920   4.2070   4.2946   4.3106   4.5941   4.6688   4.6984   4.7409
  4.9249   5.0352   5.1959  23.5203  23.5798  23.7139  23.7323  23.7381
 23.7762  23.8018  23.8337  23.8601  23.8910  23.9163  23.9636  23.9840
 24.0317  35.4175  35.4601  35.5230  35.6244
 --------------------------------------------------------------

          Ground-State Mulliken Net Atomic Charges

     Atom                 Charge (a.u.)
  ----------------------------------------
```

```
     1 N                      0.422953
     2 C                      0.121027
     3 C                     -0.030576
     4 C                     -0.231889
     5 C                     -0.288059
     6 C                     -0.055709
     7 C                     -0.751479
     8 C                     -0.164279
     9 N                     -0.181906
    10 C                     -0.208750
    11 N                      0.038390
    12 C                      0.201904
    13 C                     -0.119870
    14 C                      0.101201
    15 C                     -0.318742
    16 C                     -0.284740
    17 C                     -0.514936
    18 N                     -0.480402
    19 H                      0.220564
    20 H                      0.236602
    21 H                      0.232712
    22 H                      0.218588
    23 H                      0.235364
    24 H                      0.217232
    25 H                      0.244562
    26 H                      0.176615
    27 H                      0.248156
    28 H                      0.396200
    29 H                      0.209936
    30 H                      0.249975
    31 H                      0.207132
    32 H                      0.222670
    33 H                      0.206307
    34 H                      0.176874
    35 H                      0.216699
    36 H                      0.139869
    37 H                      0.317284
    38 H                      0.372523
  ----------------------------------------
  Sum of atomic charges =     2.000000

-----------------------------------------------------------------
                   Cartesian Multipole Moments
-----------------------------------------------------------------
    Charge (ESU x 10^10)
                 9.6064
    Dipole Moment (Debye)
         X       8.0927      Y      -0.4106      Z      -0.2205
       Tot       8.1061
    Quadrupole Moments (Debye-Ang)
        XX      -5.1082     XY     -12.6189     YY     -75.5411
        XZ      -6.4019     YZ      -7.9615     ZZ     -74.0132
    Traceless Quadrupole Moments (Debye-Ang)
       QXX     139.3378    QYY     -71.9608    QZZ     -67.3770
       QXY     -37.8567    QXZ     -19.2058    QYZ     -23.8845
    Octopole Moments (Debye-Ang^2)
       XXX      62.1593    XXY     -95.4277    XYY      12.8346
       YYY      21.5633    XXZ      57.9951    XYZ      20.0043
       YYZ     -24.9834    XZZ      -0.1640    YZZ       7.3980
       ZZZ      -1.1858
    Traceless Octopole Moments (Debye-Ang^2)
       XXX     258.9204    YYY     921.6466    ZZZ    -304.2209
       XXY   -1232.0163    XXZ     774.4490    XYY     -31.9710
       XYZ     300.0648    XZZ    -226.9493    YYZ    -470.2282
       YZZ     310.3697
    Hexadecapole Moments (Debye-Ang^3)
      XXXX   -3734.8301   XXXY     -99.7713   XXYY    -800.2437
      XYYY     284.8343   YYYY    -638.8538   XXXZ     349.4945
      XXYZ      46.2831   XYYZ      91.6341   YYYZ     -32.0138
      XXZZ    -889.7419   XYZZ      69.1278   YYZZ    -205.6150
```

```
      XZZZ     315.1867   YZZZ       1.2083   ZZZZ    -945.9222
    Traceless Hexadecapole Moments (Debye-Ang^3)
      XXXX   14078.9859   XXXY  -21914.5717   XXXZ    2662.7353
      XXYY   -5315.0885   XXYZ    4630.6884   XXZZ   -8763.8974
      XYYY   18469.0184   XYYZ   -1723.1502   XYZZ    3445.5533
      XZZZ    -939.5851   YYYY   -1052.7917   YYYZ   -4048.5693
      YYZZ    6367.8802   YZZZ    -582.1190   ZZZZ    2396.0172
 -----------------------------------------------------------------

-------------------------------------------------
-  Entering drvman on Sun Oct 13 21:57:04 2024  -
-------------------------------------------------

 Calculating analytic gradient of the SCF energy
 Gradient of SCF Energy
            1           2           3           4           5           6
    1   0.0003823  -0.0002062   0.0003997   0.0001084   0.0001894  -0.0002127
    2   0.0000551  -0.0002452  -0.0000485  -0.0004955  -0.0001680   0.0002255
    3  -0.0004763  -0.0003270   0.0005982  -0.0005425   0.0001222   0.0003573
            7           8           9          10          11          12
    1   0.0000492   0.0001740   0.0001868  -0.0012581   0.0007585  -0.0009039
    2   0.0004884   0.0001628   0.0000058   0.0007628  -0.0009336   0.0028866
    3   0.0001453   0.0005257   0.0003972  -0.0016322   0.0012071  -0.0019726
           13          14          15          16          17          18
    1   0.0007387   0.0001903   0.0003931  -0.0002908   0.0000569   0.0005803
    2  -0.0010917   0.0007858  -0.0004983   0.0004310  -0.0000732  -0.0038419
    3   0.0007174  -0.0004056  -0.0004623   0.0005613  -0.0000302  -0.0003495
           19          20          21          22          23          24
    1  -0.0001277  -0.0001725  -0.0001510  -0.0001825  -0.0001054  -0.0004918
    2   0.0002559   0.0000771   0.0001163   0.0000606   0.0000075   0.0001317
    3   0.0000062  -0.0000709  -0.0000253  -0.0002313   0.0001297   0.0002333
           25          26          27          28          29          30
    1  -0.0000766  -0.0000504   0.0003081   0.0001674   0.0000385   0.0001251
    2   0.0000766  -0.0001939  -0.0004515   0.0002261   0.0000299   0.0000786
    3  -0.0000240   0.0001861   0.0000251  -0.0001186  -0.0000930   0.0000958
           31          32          33          34          35          36
    1   0.0000558   0.0001354   0.0000620  -0.0000637   0.0000842  -0.0000587
    2   0.0000845  -0.0000097  -0.0001228   0.0000165  -0.0000569   0.0001127
    3   0.0001080  -0.0002740  -0.0000978   0.0000494   0.0000234   0.0001271
           37          38
    1  -0.0013068   0.0004745
    2   0.0005024   0.0006506
    3   0.0004593   0.0010576
 Max gradient component =       3.842E-03
 RMS gradient           =       6.507E-04
 Gradient time:  CPU 123.75 s  wall 194.17 s

-------------------------------------------------
-  Entering optman on Sun Oct 13 22:00:18 2024  -
-------------------------------------------------

 Geometry Optimization Parameters
   NAtoms,    NIC,     NZ,  NCons,   NDum,   NFix, NCnnct, MaxDiis
       38     272       0       0       0       0       0       0

 Cartesian Hessian Update
 Hessian updated using BFGS update


** GEOMETRY OPTIMIZATION IN DELOCALIZED INTERNAL COORDINATES **
   Searching for a Minimum

   Optimization Cycle:   6

                       Coordinates (Angstroms)
     ATOM                X               Y               Z
      1  N         2.7046077552    0.1450470502   -0.1331915310
      2  C         2.8589721489   -0.6997353033   -1.1926813645
      3  C         3.8422711938   -1.6757807994   -1.1116537318
      4  C         4.6482109552   -1.7896823102    0.0093519258
```

```
     5  C          4.4699636823   -0.9049043029    1.0703158901
     6  C          3.4946961773    0.0533449153    0.9676343399
     7  C          1.9824783266   -0.5590807217   -2.3951995511
     8  C          1.6912390742    1.2262617027   -0.1651330781
     9  N         -1.3755478931   -0.6102411231    0.9820101604
    10  C         -2.3187720549   -0.2312870503    0.0750757717
    11  N         -2.0220848268    0.6340437194   -0.8552498850
    12  C         -0.7908741307    1.1680787095   -0.9321323890
    13  C          0.2661286589    0.7251955564   -0.0605100096
    14  C         -0.1037272362   -0.1540360081    0.9082942747
    15  C         -3.6965951270   -0.7784811431    0.1952838694
    16  C         -4.6468885064    0.2445568588    0.8624153117
    17  C         -4.2720630750    0.5752770850    2.3023898452
    18  N         -0.6226241237    2.0806329169   -1.8809416040
    19  H          3.9724183584   -2.3433004153   -1.9534895744
    20  H          5.4138889930   -2.5550274695    0.0543973558
    21  H          5.0836815979   -0.9453245270    1.9603810977
    22  H          3.3183366366    0.7836533507    1.7464381301
    23  H          2.3364497556   -1.2188948418   -3.1847111467
    24  H          1.9812978447    0.4597259434   -2.7896764552
    25  H          0.9502969501   -0.8378878116   -2.1667727973
    26  H          1.8496573665    1.7861563597   -1.0850286606
    27  H          1.9244180081    1.9037057103    0.6562112174
    28  H         -1.6395281888   -1.2488273880    1.7223084375
    29  H          0.5855811333   -0.5444040815    1.6479001672
    30  H         -4.0467073302   -1.0024852559   -0.8138813437
    31  H         -3.6851333064   -1.7095129594    0.7697279174
    32  H         -5.6473206666   -0.1900164911    0.8237111045
    33  H         -4.6737025099    1.1486663195    0.2487349620
    34  H         -4.2394856369   -0.3245724732    2.9250994309
    35  H         -5.0114022335    1.2441663372    2.7442529455
    36  H         -3.3042596915    1.0849182170    2.3754008159
    37  H          0.1643317037    2.7056575878   -1.9206367037
    38  H         -1.4422097830    2.3543941367   -2.4064451455
   Point Group: c1    Number of degrees of freedom:   108


   Energy is   -763.968531340

 Hessian updated using BFGS update
  internal optimization (0)

108 Hessian modes will be used to form the next step
  Hessian Eigenvalues:
     0.002115    0.002687    0.004123    0.004503    0.009746    0.010036
     0.016780    0.018953    0.019218    0.019786    0.020747    0.022401
     0.022689    0.022834    0.023922    0.024011    0.025555    0.026246
     0.026620    0.028252    0.028400    0.030425    0.031581    0.036312
     0.038752    0.041454    0.042888    0.043745    0.044364    0.046052
     0.048656    0.051751    0.054109    0.055135    0.063903    0.079910
     0.085589    0.094162    0.121659    0.122189    0.127124    0.130762
     0.131845    0.132986    0.136240    0.140641    0.143023    0.144333
     0.147686    0.148410    0.149728    0.152321    0.152738    0.153002
     0.154514    0.160662    0.192754    0.206275    0.206912    0.216824
     0.225779    0.229824    0.238375    0.247488    0.250495    0.260798
     0.268608    0.271583    0.282180    0.298020    0.300132    0.300433
     0.300682    0.301231    0.301709    0.302651    0.304110    0.304902
     0.305313    0.305548    0.307567    0.308188    0.312315    0.316636
     0.323218    0.331976    0.332934    0.334474    0.337051    0.341678
     0.344866    0.352943    0.355153    0.362673    0.381012    0.389345
     0.396430    0.406784    0.412581    0.417949    0.420122    0.435282
     0.441696    0.448450    0.455427    0.491938    0.573114    0.784560

 Minimum search - taking simple RFO step
 Searching for Lamda that Minimizes Along All modes
 Value Taken    Lamda =  -0.00066415
 Calculated Step too Large.  Step scaled by  0.885234
 Step Taken.  Stepsize is  0.300000

                             Maximum     Tolerance    Cnvgd?
```

```
        Gradient            0.003149      0.000800      NO
        Displacement        0.113231      0.001400      NO
        Energy change      -0.000809      0.000228      NO


 New Cartesian Coordinates Obtained by Inverse Iteration

 Displacement from previous Coordinates is:  0.699659
 ----------------------------------------------------------------
             Standard Nuclear Orientation (Angstroms)
    I     Atom           X                Y                Z
 ----------------------------------------------------------------
    1      N       2.6984191549     0.1448155041    -0.1406469588
    2      C       2.8668619300    -0.7167399082    -1.1850770187
    3      C       3.8520206999    -1.6874256156    -1.0787182143
    4      C       4.6470952918    -1.7778127832     0.0530637496
    5      C       4.4556674983    -0.8768517880     1.0973224906
    6      C       3.4777534573     0.0749366701     0.9692013496
    7      C       2.0067190022    -0.5921037438    -2.4017507501
    8      C       1.6813493522     1.2200153358    -0.2042602846
    9      N      -1.3707356202    -0.6551900773     0.9297579656
   10      C      -2.3312287980    -0.2279281630     0.0633995326
   11      N      -2.0497690681     0.6747796228    -0.8370787319
   12      C      -0.8146133651     1.2021810292    -0.9203212526
   13      C       0.2578597259     0.7152839901    -0.0917156917
   14      C      -0.0974595559    -0.2040787166     0.8461058308
   15      C      -3.7120619691    -0.7686892856     0.1914375715
   16      C      -4.6491032024     0.2482754105     0.8834157152
   17      C      -4.2419016091     0.5736140578     2.3157006766
   18      N      -0.6622320623     2.1631735591    -1.8230334675
   19      H       3.9945618528    -2.3694920339    -1.9070576129
   20      H       5.4159580789    -2.5390283673     0.1185474668
   21      H       5.0612874506    -0.9015365775     1.9936130999
   22      H       3.2901968646     0.8164648435     1.7350161612
   23      H       2.3394293208    -1.2975281807    -3.1607195555
   24      H       2.0577931336     0.4096409397    -2.8366017632
   25      H       0.9611692876    -0.8147528213    -2.1743112058
   26      H       1.8438342278     1.7557229535    -1.1383988729
   27      H       1.9069050267     1.9219313095     0.5990749980
   28      H      -1.6227010506    -1.3239529865     1.6474280701
   29      H       0.6052861265    -0.6295944845     1.5529066702
   30      H      -4.0746405777    -0.9750387825    -0.8174194661
   31      H      -3.6997390751    -1.7091524518     0.7502845275
   32      H      -5.6496808215    -0.1878233630     0.8672675227
   33      H      -4.6915678573     1.1553896922     0.2751067512
   34      H      -4.1983015843    -0.3282631271     2.9348028739
   35      H      -4.9693162611     1.2437024208     2.7754203260
   36      H      -3.2704941333     1.0788468281     2.3677466742
   37      H       0.1677804118     2.7234760710    -1.9144470796
   38      H      -1.4824012836     2.4607330195    -2.3350620975
 ----------------------------------------------------------------
 Nuclear Repulsion Energy =        1314.62841174 hartrees
 There are       65 alpha and       65 beta electrons

--------------------------------------------------
-  Entering fldman on Sun Oct 13 22:00:18 2024  -
--------------------------------------------------

 Applying Cartesian multipole field
    Component          Value
    ---------          -----
     (2,0,0)        1.00000E-12
     (0,2,0)        2.00000E-11
     (0,0,2)       -3.00000E-11
 Nucleus-field energy     =    -0.0000000077 hartrees

--------------------------------------------------
-  Entering gesman on Sun Oct 13 22:00:18 2024  -
--------------------------------------------------
```

```
 Requested basis set is 6-311+G(d,p)
 There are 188 shells and 516 basis functions
 A cutoff of  1.0D-12 yielded  12834 shell pairs
 There are    101979 function pairs (    108729 Cartesian)
 Smallest overlap matrix eigenvalue = 2.16E-06
 Linear dependence detected in AO basis
 Tighter screening thresholds may be required for diffuse basis sets
 Use S2THRESH > 12 and THRESH = 14 in case of SCF convergence issues
 Number of orthogonalized atomic orbitals = 510
 Maximum deviation from orthogonality = 3.770E-11
 Guess MOs from SCF MO coefficient file
 Reading MOs from coefficient file
 Reading MOs from coefficient file

--------------------------------------------------
-  Entering scfman on Sun Oct 13 22:00:18 2024  -
--------------------------------------------------

 Long-range K will be added via erf
 Coulomb attenuation parameter = 0.2 bohr**(-1)
 A restricted hybrid HF-DFT SCF calculation will be
 performed using Pulay DIIS + Geometric Direct Minimization
 Exchange:     0.2220 Hartree-Fock + 1.0000 wB97X-D + LR-HF
 Correlation:  1.0000 wB97X-D
 Using Euler-Maclaurin-Lebedev (75,302) quadrature formula
 Dispersion:   Grimme D
 SCF converges when RMS gradient is below 1.0E-07
 Geometry optimization detected.  Setting ReadMinima to 0
 Setting SaveMinima to 0
 ---------------------------------------
  Cycle       Energy         DIIS Error
 ---------------------------------------
    1    -763.9651677389      7.26E-04
    2    -763.9683643780      8.76E-05
    3    -763.9688670048      5.48E-05
    4    -763.9689509793      2.94E-05
    5    -763.9689835378      6.14E-06
    6    -763.9689852122      2.25E-06
    7    -763.9689854549      7.40E-07
    8    -763.9689854870      3.49E-07
    9    -763.9689854928      8.55E-08 Convergence criterion met
 ---------------------------------------
 SCF time:  CPU 243.13 s  wall 389.00 s
 SCF   energy in the final basis set = -763.96898549
 Total energy in the final basis set = -763.96898549

--------------------------------------------------
-  Entering anlman on Sun Oct 13 22:06:48 2024  -
--------------------------------------------------



 --------------------------------------------------------------

                   Orbital Energies (a.u.)
 --------------------------------------------------------------

 Alpha MOs
 -- Occupied --
-14.8192 -14.7889 -14.7372 -14.7058 -10.6766 -10.6605 -10.6464 -10.6417
-10.6379 -10.6287 -10.6063 -10.5862 -10.5746 -10.5737 -10.5531 -10.5252
-10.4923 -10.4678  -1.4146  -1.4074  -1.3472  -1.2747  -1.2320  -1.2000
 -1.1910  -1.1419  -1.1015  -1.0866  -1.0541  -1.0515  -1.0359  -1.0055
 -0.9917  -0.9546  -0.9457  -0.9164  -0.9069  -0.8917  -0.8839  -0.8633
 -0.8570  -0.8471  -0.8340  -0.8244  -0.8104  -0.7989  -0.7888  -0.7872
 -0.7716  -0.7576  -0.7550  -0.7509  -0.7454  -0.7317  -0.7211  -0.7179
 -0.6983  -0.6682  -0.6603  -0.6425  -0.6322  -0.6254  -0.6243  -0.6220
 -0.6086
 -- Virtual --
 -0.2720  -0.2586  -0.2416  -0.2282  -0.1743  -0.1655  -0.1501  -0.1448
 -0.1346  -0.1227  -0.1180  -0.1140  -0.1117  -0.1044  -0.1008  -0.0963
```

| | | | | | | | |
|---|---|---|---|---|---|---|---|
| -0.0898 | -0.0854 | -0.0828 | -0.0794 | -0.0747 | -0.0704 | -0.0695 | -0.0662 |
| -0.0638 | -0.0584 | -0.0552 | -0.0528 | -0.0480 | -0.0469 | -0.0412 | -0.0349 |
| -0.0311 | -0.0300 | -0.0273 | -0.0232 | -0.0195 | -0.0148 | -0.0116 | -0.0086 |
| -0.0059 | -0.0031 | 0.0017 | 0.0038 | 0.0063 | 0.0106 | 0.0117 | 0.0162 |
| 0.0193 | 0.0232 | 0.0251 | 0.0286 | 0.0331 | 0.0369 | 0.0395 | 0.0419 |
| 0.0449 | 0.0470 | 0.0518 | 0.0523 | 0.0572 | 0.0595 | 0.0614 | 0.0629 |
| 0.0709 | 0.0719 | 0.0773 | 0.0783 | 0.0802 | 0.0818 | 0.0889 | 0.0910 |
| 0.0925 | 0.0987 | 0.1009 | 0.1068 | 0.1106 | 0.1122 | 0.1165 | 0.1195 |
| 0.1207 | 0.1298 | 0.1306 | 0.1363 | 0.1384 | 0.1422 | 0.1468 | 0.1495 |
| 0.1526 | 0.1555 | 0.1590 | 0.1634 | 0.1657 | 0.1718 | 0.1779 | 0.1822 |
| 0.1840 | 0.1916 | 0.1943 | 0.2003 | 0.2031 | 0.2104 | 0.2170 | 0.2198 |
| 0.2344 | 0.2408 | 0.2553 | 0.2656 | 0.2751 | 0.2830 | 0.3114 | 0.3166 |
| 0.3262 | 0.3292 | 0.3358 | 0.3458 | 0.3462 | 0.3511 | 0.3642 | 0.3731 |
| 0.3794 | 0.3847 | 0.3909 | 0.3935 | 0.4038 | 0.4062 | 0.4172 | 0.4229 |
| 0.4320 | 0.4364 | 0.4438 | 0.4449 | 0.4570 | 0.4658 | 0.4694 | 0.4735 |
| 0.4817 | 0.4923 | 0.4960 | 0.5014 | 0.5114 | 0.5148 | 0.5209 | 0.5251 |
| 0.5274 | 0.5311 | 0.5372 | 0.5416 | 0.5461 | 0.5494 | 0.5566 | 0.5598 |
| 0.5614 | 0.5729 | 0.5760 | 0.5787 | 0.5919 | 0.5942 | 0.6051 | 0.6066 |
| 0.6080 | 0.6217 | 0.6305 | 0.6317 | 0.6384 | 0.6532 | 0.6558 | 0.6581 |
| 0.6634 | 0.6694 | 0.6746 | 0.6815 | 0.6901 | 0.6966 | 0.7013 | 0.7133 |
| 0.7242 | 0.7300 | 0.7327 | 0.7495 | 0.7552 | 0.7669 | 0.7793 | 0.7854 |
| 0.7931 | 0.7953 | 0.8088 | 0.8263 | 0.8379 | 0.8408 | 0.8546 | 0.8618 |
| 0.8678 | 0.8801 | 0.8846 | 0.8908 | 0.9000 | 0.9133 | 0.9296 | 0.9355 |
| 0.9487 | 0.9665 | 0.9769 | 0.9818 | 0.9977 | 1.0138 | 1.0244 | 1.0332 |
| 1.0453 | 1.0597 | 1.0828 | 1.0898 | 1.1231 | 1.1436 | 1.1581 | 1.1776 |
| 1.2025 | 1.2037 | 1.2241 | 1.2295 | 1.2580 | 1.2783 | 1.3001 | 1.3094 |
| 1.3134 | 1.3250 | 1.3344 | 1.3450 | 1.3563 | 1.3664 | 1.3683 | 1.3755 |
| 1.3856 | 1.3913 | 1.4061 | 1.4090 | 1.4187 | 1.4265 | 1.4339 | 1.4369 |
| 1.4449 | 1.4529 | 1.4572 | 1.4595 | 1.4676 | 1.4830 | 1.4933 | 1.5003 |
| 1.5050 | 1.5156 | 1.5237 | 1.5274 | 1.5375 | 1.5440 | 1.5547 | 1.5565 |
| 1.5615 | 1.5698 | 1.5760 | 1.5771 | 1.5931 | 1.6010 | 1.6069 | 1.6103 |
| 1.6217 | 1.6347 | 1.6424 | 1.6467 | 1.6607 | 1.6692 | 1.6745 | 1.6805 |
| 1.6943 | 1.7029 | 1.7096 | 1.7295 | 1.7345 | 1.7491 | 1.7588 | 1.7755 |
| 1.7838 | 1.7904 | 1.8096 | 1.8158 | 1.8396 | 1.8463 | 1.8661 | 1.8861 |
| 1.8904 | 1.9029 | 1.9160 | 1.9313 | 1.9540 | 1.9673 | 1.9840 | 1.9923 |
| 1.9999 | 2.0120 | 2.0254 | 2.0319 | 2.0383 | 2.0527 | 2.0778 | 2.0860 |
| 2.1049 | 2.1211 | 2.1356 | 2.1606 | 2.1644 | 2.1854 | 2.2194 | 2.2244 |
| 2.2307 | 2.2355 | 2.2516 | 2.2541 | 2.2567 | 2.2694 | 2.2863 | 2.2897 |
| 2.3109 | 2.3231 | 2.3361 | 2.3496 | 2.3641 | 2.3730 | 2.3936 | 2.3980 |
| 2.4047 | 2.4165 | 2.4378 | 2.4421 | 2.4485 | 2.4618 | 2.4700 | 2.4943 |
| 2.5046 | 2.5109 | 2.5260 | 2.5282 | 2.5406 | 2.5462 | 2.5470 | 2.5636 |
| 2.5736 | 2.5763 | 2.5851 | 2.5930 | 2.5941 | 2.6021 | 2.6114 | 2.6222 |
| 2.6385 | 2.6497 | 2.6616 | 2.6684 | 2.6739 | 2.6785 | 2.6923 | 2.7038 |
| 2.7175 | 2.7221 | 2.7309 | 2.7516 | 2.7587 | 2.7732 | 2.7780 | 2.7796 |
| 2.8024 | 2.8098 | 2.8181 | 2.8454 | 2.8515 | 2.8750 | 2.8759 | 2.8843 |
| 2.8993 | 2.9283 | 2.9696 | 3.0014 | 3.0445 | 3.0644 | 3.1359 | 3.1643 |
| 3.1707 | 3.2321 | 3.2520 | 3.2705 | 3.3057 | 3.3196 | 3.3659 | 3.4110 |
| 3.4235 | 3.4800 | 3.5272 | 3.5352 | 3.5579 | 3.5952 | 3.6155 | 3.6411 |
| 3.6907 | 3.7098 | 3.7131 | 3.7333 | 3.7417 | 3.7503 | 3.7530 | 3.7629 |
| 3.7681 | 3.8406 | 3.8653 | 3.9006 | 3.9399 | 3.9851 | 4.0286 | 4.0875 |
| 4.0919 | 4.2085 | 4.2934 | 4.3116 | 4.5944 | 4.6692 | 4.6949 | 4.7468 |
| 4.9265 | 5.0345 | 5.1975 | 23.5197 | 23.5808 | 23.7139 | 23.7311 | 23.7367 |
| 23.7768 | 23.8018 | 23.8333 | 23.8600 | 23.8911 | 23.9175 | 23.9634 | 23.9805 |
| 24.0314 | 35.4157 | 35.4613 | 35.5238 | 35.6241 | | | |

--------------------------------------------------------------

Ground-State Mulliken Net Atomic Charges

| Atom | Charge (a.u.) |
|---|---|
| 1 N | 0.410005 |
| 2 C | 0.137756 |
| 3 C | -0.027493 |
| 4 C | -0.227424 |
| 5 C | -0.292872 |
| 6 C | -0.035911 |
| 7 C | -0.773970 |
| 8 C | -0.147778 |
| 9 N | -0.171810 |
| 10 C | -0.252002 |

```
   11 N                    0.040751
   12 C                    0.228875
   13 C                   -0.125069
   14 C                    0.089451
   15 C                   -0.313539
   16 C                   -0.289867
   17 C                   -0.510533
   18 N                   -0.491937
   19 H                    0.220953
   20 H                    0.236849
   21 H                    0.233025
   22 H                    0.219618
   23 H                    0.235014
   24 H                    0.223574
   25 H                    0.242690
   26 H                    0.172282
   27 H                    0.249914
   28 H                    0.393891
   29 H                    0.209670
   30 H                    0.251143
   31 H                    0.204064
   32 H                    0.222216
   33 H                    0.207415
   34 H                    0.176321
   35 H                    0.215982
   36 H                    0.140482
   37 H                    0.318627
   38 H                    0.379637
 ----------------------------------------
 Sum of atomic charges =     2.000000

-----------------------------------------------------------------
                  Cartesian Multipole Moments
-----------------------------------------------------------------
   Charge (ESU x 10^10)
                 9.6064
   Dipole Moment (Debye)
        X       8.2043      Y      -0.4868      Z      -0.4132
      Tot       8.2291
   Quadrupole Moments (Debye-Ang)
       XX      -5.1968     XY     -12.5842     YY     -74.9024
       XZ      -6.1396     YZ      -8.2968     ZZ     -74.5285
   Traceless Quadrupole Moments (Debye-Ang)
      QXX     139.0373    QYY     -70.0795    QZZ     -68.9578
      QXY     -37.7527    QXZ     -18.4189    QYZ     -24.8905
   Octopole Moments (Debye-Ang^2)
      XXX      64.3371    XXY     -94.9973    XYY      12.5464
      YYY      21.0632    XXZ      58.4641    XYZ      19.6960
      YYZ     -25.5554    XZZ       1.3242    YZZ       7.6203
      ZZZ      -2.3907
   Traceless Octopole Moments (Debye-Ang^2)
      XXX     261.1867    YYY     912.7717    ZZZ    -310.5228
      XXY   -1226.0181    XXZ     785.4078    XYY     -46.4270
      XYZ     295.4402    XZZ    -214.7597    YYZ    -474.8850
      YZZ     313.2465
   Hexadecapole Moments (Debye-Ang^3)
     XXXX   -3743.7173   XXXY     -91.8321   XXYY    -801.9575
     XYYY     285.8787   YYYY    -652.7935   XXXZ     352.6370
     XXYZ      43.8605   XYYZ      91.6656   YYYZ     -34.0059
     XXZZ    -886.4896   XYZZ      70.3832   YYZZ    -203.9052
     XZZZ     310.0766   YZZZ       5.1553   ZZZZ    -938.9190
   Traceless Hexadecapole Moments (Debye-Ang^3)
     XXXX   13723.2733   XXXY  -21541.7079   XXXZ    3079.8240
     XXYY   -5203.6325   XXYZ    4382.5302   XXZZ   -8519.6408
     XYYY   18117.9201   XYYZ   -1690.8012   XYZZ    3423.7878
     XZZZ   -1389.0228   YYYY   -1345.4717   YYYZ   -4239.0763
     YYZZ    6549.1042   YZZZ    -143.4538   ZZZZ    1970.5366
-----------------------------------------------------------------

-------------------------------------------------
```

```
-  Entering drvman on Sun Oct 13 22:06:48 2024  -
--------------------------------------------------

 Calculating analytic gradient of the SCF energy
 Gradient of SCF Energy
           1           2           3           4           5           6
    1   0.0003885   0.0001030  -0.0001164  -0.0001888  -0.0000748   0.0002066
    2  -0.0000390  -0.0003869   0.0001765   0.0001389   0.0001835  -0.0004141
    3  -0.0003205   0.0001593  -0.0000851   0.0000390   0.0001127  -0.0000724
           7           8           9          10          11          12
    1  -0.0001425  -0.0001138   0.0003316  -0.0007747   0.0000006  -0.0001174
    2   0.0002938   0.0001581  -0.0004419  -0.0000208  -0.0008143   0.0013607
    3   0.0000868   0.0003855   0.0007798  -0.0008015   0.0000321  -0.0008254
          13          14          15          16          17          18
    1   0.0009602  -0.0005439   0.0000578  -0.0001245   0.0001312   0.0000719
    2   0.0001865  -0.0000442  -0.0003414   0.0001634   0.0000051  -0.0006835
    3  -0.0000973   0.0003295  -0.0001306   0.0002897  -0.0000896  -0.0006791
          19          20          21          22          23          24
    1  -0.0000137   0.0000024  -0.0000603  -0.0000615  -0.0001708  -0.0004441
    2   0.0000764  -0.0000588   0.0000055   0.0000635  -0.0000411   0.0003314
    3  -0.0000655  -0.0000566  -0.0000003  -0.0000747  -0.0000879   0.0002336
          25          26          27          28          29          30
    1   0.0000706   0.0004089   0.0002034   0.0001133   0.0000435   0.0001242
    2  -0.0001061   0.0000315  -0.0002767   0.0001221   0.0000706   0.0001543
    3  -0.0000713   0.0002154   0.0000416   0.0000789  -0.0000722   0.0000385
          31          32          33          34          35          36
    1  -0.0000142   0.0000196  -0.0000204  -0.0000770  -0.0000203  -0.0000776
    2   0.0000625  -0.0000352  -0.0000439  -0.0000155  -0.0000170   0.0000469
    3   0.0000827  -0.0000632  -0.0000859   0.0000309   0.0000157   0.0000804
          37          38
    1  -0.0000869   0.0000063
    2  -0.0003538   0.0005031
    3   0.0000650   0.0005822
 Max gradient component =       1.361E-03
 RMS gradient           =       3.069E-04
 Gradient time:  CPU 116.81 s  wall 183.20 s

--------------------------------------------------
-  Entering optman on Sun Oct 13 22:09:51 2024  -
--------------------------------------------------

 Geometry Optimization Parameters
   NAtoms,    NIC,     NZ,  NCons,   NDum,   NFix, NCnnct, MaxDiis
       38     272       0       0       0       0       0       0

 Cartesian Hessian Update
 Hessian updated using BFGS update


** GEOMETRY OPTIMIZATION IN DELOCALIZED INTERNAL COORDINATES **
   Searching for a Minimum

   Optimization Cycle:   7

                        Coordinates (Angstroms)
     ATOM                X               Y               Z
      1  N         2.6984191549    0.1448155041   -0.1406469588
      2  C         2.8668619300   -0.7167399082   -1.1850770187
      3  C         3.8520206999   -1.6874256156   -1.0787182143
      4  C         4.6470952918   -1.7778127832    0.0530637496
      5  C         4.4556674983   -0.8768517880    1.0973224906
      6  C         3.4777534573    0.0749366701    0.9692013496
      7  C         2.0067190022   -0.5921037438   -2.4017507501
      8  C         1.6813493522    1.2200153358   -0.2042602846
      9  N        -1.3707356202   -0.6551900773    0.9297579656
     10  C        -2.3312287980   -0.2279281630    0.0633995326
     11  N        -2.0497690681    0.6747796228   -0.8370787319
     12  C        -0.8146133651    1.2021810292   -0.9203212526
     13  C         0.2578597259    0.7152839901   -0.0917156917
     14  C        -0.0974595559   -0.2040787166    0.8461058308
```

```
    15  C        -3.7120619691   -0.7686892856    0.1914375715
    16  C        -4.6491032024    0.2482754105    0.8834157152
    17  C        -4.2419016091    0.5736140578    2.3157006766
    18  N        -0.6622320623    2.1631735591   -1.8230334675
    19  H         3.9945618528   -2.3694920339   -1.9070576129
    20  H         5.4159580789   -2.5390283673    0.1185474668
    21  H         5.0612874506   -0.9015365775    1.9936130999
    22  H         3.2901968646    0.8164648435    1.7350161612
    23  H         2.3394293208   -1.2975281807   -3.1607195555
    24  H         2.0577931336    0.4096409397   -2.8366017632
    25  H         0.9611692876   -0.8147528213   -2.1743112058
    26  H         1.8438342278    1.7557229535   -1.1383988729
    27  H         1.9069050267    1.9219313095    0.5990749980
    28  H        -1.6227010506   -1.3239529865    1.6474280701
    29  H         0.6052861265   -0.6295944845    1.5529066702
    30  H        -4.0746405777   -0.9750387825   -0.8174194661
    31  H        -3.6997390751   -1.7091524518    0.7502845275
    32  H        -5.6496808215   -0.1878233630    0.8672675227
    33  H        -4.6915678573    1.1553896922    0.2751067512
    34  H        -4.1983015843   -0.3282631271    2.9348028739
    35  H        -4.9693162611    1.2437024208    2.7754203260
    36  H        -3.2704941333    1.0788468281    2.3677466742
    37  H         0.1677804118    2.7234760710   -1.9144470796
    38  H        -1.4824012836    2.4607330195   -2.3350620975
   Point Group: c1    Number of degrees of freedom:   108


   Energy is   -763.968985493

 Hessian updated using BFGS update
  internal optimization (0)

108 Hessian modes will be used to form the next step
  Hessian Eigenvalues:
     0.001147    0.002681    0.003737    0.004216    0.009686    0.010718
     0.018406    0.019079    0.019382    0.019980    0.021023    0.022584
     0.022663    0.023127    0.023794    0.024019    0.025428    0.026105
     0.026759    0.028248    0.028405    0.030468    0.031335    0.036310
     0.038749    0.041453    0.042881    0.043745    0.044365    0.045751
     0.048344    0.051562    0.054120    0.055207    0.063585    0.079690
     0.085577    0.094218    0.121648    0.122173    0.127123    0.130771
     0.131834    0.132990    0.136992    0.141377    0.143098    0.144298
     0.147687    0.148301    0.149682    0.152317    0.152807    0.152987
     0.156636    0.161149    0.193016    0.206281    0.206946    0.216792
     0.225796    0.229661    0.240420    0.247460    0.251731    0.260583
     0.268535    0.271591    0.282030    0.298340    0.300121    0.300485
     0.300709    0.301239    0.301722    0.302789    0.304110    0.304974
     0.305337    0.305536    0.307567    0.308247    0.312294    0.317041
     0.322981    0.332064    0.332759    0.334950    0.337072    0.341605
     0.344476    0.353451    0.354840    0.363243    0.381015    0.389684
     0.398614    0.406686    0.414364    0.418123    0.419871    0.433841
     0.437567    0.449198    0.468736    0.503209    0.571558    0.763740

 Minimum search - taking simple RFO step
 Searching for Lamda that Minimizes Along All modes
 Value Taken    Lamda =  -0.00075221
 Calculated Step too Large.  Step scaled by  0.537951
 Step Taken.  Stepsize is  0.300000

                             Maximum     Tolerance    Cnvgd?
        Gradient            0.003148      0.000800      NO
        Displacement        0.143802      0.001400      NO
        Energy change      -0.000454      0.000228      NO


 New Cartesian Coordinates Obtained by Inverse Iteration

 Displacement from previous Coordinates is:  0.742687
 -----------------------------------------------------------------
             Standard Nuclear Orientation (Angstroms)
```

```
    I     Atom           X                Y                Z
 ----------------------------------------------------------------
    1      N       2.6915215275     0.1441227697    -0.1516272970
    2      C       2.8801227791    -0.7322651122    -1.1807058749
    3      C       3.8678412567    -1.6969480163    -1.0448153826
    4      C       4.6466178689    -1.7656291109     0.0999237591
    5      C       4.4353801589    -0.8495919845     1.1268892110
    6      C       3.4543309626     0.0957119974     0.9706700578
    7      C       2.0438540415    -0.6213765961    -2.4153805430
    8      C       1.6693153142     1.2100281990    -0.2498634437
    9      N      -1.3693213532    -0.6973350086     0.8751676650
   10      C      -2.3457141757    -0.2225526727     0.0532018318
   11      N      -2.0772490375     0.7139957237    -0.8162353531
   12      C      -0.8378732171     1.2294050983    -0.9093487068
   13      C       0.2481817499     0.7004675329    -0.1254460033
   14      C      -0.0934889515    -0.2546142878     0.7804989919
   15      C      -3.7294817622    -0.7550668782     0.1905731537
   16      C      -4.6494185356     0.2577689799     0.9090001431
   17      C      -4.2122607235     0.5689128347     2.3355397893
   18      N      -0.6986130439     2.2324026549    -1.7660522556
   19      H       4.0268076173    -2.3910697817    -1.8601835783
   20      H       5.4184048152    -2.5215440845     0.1886069677
   21      H       5.0282930757    -0.8582598716     2.0320648702
   22      H       3.2508988576     0.8468280584     1.7230602979
   23      H       2.3446557284    -1.3815721028    -3.1335540867
   24      H       2.1653776109     0.3534506309    -2.8966013858
   25      H       0.9834494679    -0.7676033381    -2.1964677292
   26      H       1.8300191806     1.7158811522    -1.2011721076
   27      H       1.8877849702     1.9396687816     0.5308625616
   28      H      -1.6109997352    -1.3948389444     1.5686119357
   29      H       0.6205783888    -0.7154865580     1.4528063917
   30      H      -4.1063713627    -0.9433469759    -0.8168658249
   31      H      -3.7173413713    -1.7047180408     0.7336974588
   32      H      -5.6517948088    -0.1745896393     0.9095631132
   33      H      -4.7004776241     1.1707086586     0.3102652780
   34      H      -4.1628218809    -0.3379946662     2.9467247681
   35      H      -4.9253521301     1.2410131354     2.8143544662
   36      H      -3.2357182369     1.0656998660     2.3712290188
   37      H       0.1509883541     2.7554641282    -1.8918829651
   38      H      -1.5201257759     2.5448734689    -2.2671091932
 ----------------------------------------------------------------
 Nuclear Repulsion Energy =        1314.00158305 hartrees
 There are       65 alpha and       65 beta electrons

--------------------------------------------------
-  Entering fldman on Sun Oct 13 22:09:51 2024  -
--------------------------------------------------

 Applying Cartesian multipole field
    Component          Value
    ---------          -----
     (2,0,0)        1.00000E-12
     (0,2,0)        2.00000E-11
     (0,0,2)       -3.00000E-11
 Nucleus-field energy     =    -0.0000000072 hartrees

--------------------------------------------------
-  Entering gesman on Sun Oct 13 22:09:51 2024  -
--------------------------------------------------

 Requested basis set is 6-311+G(d,p)
 There are 188 shells and 516 basis functions
 A cutoff of  1.0D-12 yielded  12837 shell pairs
 There are    101903 function pairs (    108629 Cartesian)
 Smallest overlap matrix eigenvalue = 2.16E-06
 Linear dependence detected in AO basis
 Tighter screening thresholds may be required for diffuse basis sets
 Use S2THRESH > 12 and THRESH = 14 in case of SCF convergence issues
 Number of orthogonalized atomic orbitals = 510
 Maximum deviation from orthogonality = 3.233E-11
```

```
 Guess MOs from SCF MO coefficient file
 Reading MOs from coefficient file
 Reading MOs from coefficient file

-----------------------------------------------
-  Entering scfman on Sun Oct 13 22:09:51 2024  -
-----------------------------------------------

 Long-range K will be added via erf
 Coulomb attenuation parameter = 0.2 bohr**(-1)
 A restricted hybrid HF-DFT SCF calculation will be
 performed using Pulay DIIS + Geometric Direct Minimization
 Exchange:     0.2220 Hartree-Fock + 1.0000 wB97X-D + LR-HF
 Correlation:  1.0000 wB97X-D
 Using Euler-Maclaurin-Lebedev (75,302) quadrature formula
 Dispersion:   Grimme D
 SCF converges when RMS gradient is below 1.0E-07
 Geometry optimization detected.  Setting ReadMinima to 0
 Setting SaveMinima to 0
 ---------------------------------------
  Cycle       Energy         DIIS Error
 ---------------------------------------
    1    -763.9717191517      7.27E-04
    2    -763.9687078020      8.72E-05
    3    -763.9692066687      5.76E-05
    4    -763.9692998701      2.92E-05
    5    -763.9693330005      5.11E-06
    6    -763.9693342520      2.41E-06
    7    -763.9693344991      6.91E-07
    8    -763.9693345327      2.92E-07
    9    -763.9693345370      9.72E-08 Convergence criterion met
 ---------------------------------------
 SCF time:  CPU 241.08 s  wall 385.86 s
 SCF   energy in the final basis set = -763.96933454
 Total energy in the final basis set = -763.96933454

-----------------------------------------------
-  Entering anlman on Sun Oct 13 22:16:17 2024  -
-----------------------------------------------



 --------------------------------------------------------------


                   Orbital Energies (a.u.)
 --------------------------------------------------------------


 Alpha MOs
 -- Occupied --
-14.8197 -14.7884 -14.7373 -14.7052 -10.6762 -10.6600 -10.6460 -10.6421
-10.6382 -10.6293 -10.6064 -10.5867 -10.5751 -10.5742 -10.5533 -10.5248
-10.4914 -10.4671  -1.4144  -1.4078  -1.3471  -1.2744  -1.2322  -1.2000
 -1.1914  -1.1418  -1.1016  -1.0868  -1.0540  -1.0511  -1.0362  -1.0051
 -0.9919  -0.9545  -0.9456  -0.9164  -0.9068  -0.8914  -0.8846  -0.8629
 -0.8571  -0.8473  -0.8343  -0.8248  -0.8101  -0.7992  -0.7889  -0.7878
 -0.7716  -0.7578  -0.7544  -0.7514  -0.7451  -0.7320  -0.7213  -0.7176
 -0.6975  -0.6676  -0.6606  -0.6421  -0.6314  -0.6247  -0.6245  -0.6218
 -0.6082
 -- Virtual --
 -0.2726  -0.2581  -0.2404  -0.2284  -0.1743  -0.1656  -0.1502  -0.1448
 -0.1347  -0.1217  -0.1179  -0.1139  -0.1118  -0.1043  -0.1011  -0.0967
 -0.0901  -0.0852  -0.0829  -0.0790  -0.0749  -0.0705  -0.0697  -0.0669
 -0.0636  -0.0586  -0.0550  -0.0529  -0.0480  -0.0477  -0.0429  -0.0348
 -0.0306  -0.0296  -0.0277  -0.0235  -0.0185  -0.0153  -0.0110  -0.0079
 -0.0054  -0.0035   0.0015   0.0036   0.0053   0.0101   0.0120   0.0164
  0.0190   0.0232   0.0256   0.0293   0.0334   0.0369   0.0394   0.0427
  0.0449   0.0469   0.0514   0.0525   0.0581   0.0591   0.0610   0.0620
  0.0698   0.0716   0.0773   0.0785   0.0797   0.0809   0.0900   0.0913
  0.0971   0.0985   0.1017   0.1055   0.1107   0.1127   0.1160   0.1184
  0.1222   0.1295   0.1312   0.1366   0.1382   0.1426   0.1486   0.1505
  0.1519   0.1554   0.1598   0.1636   0.1657   0.1717   0.1789   0.1823
```

| | | | | | | | |
|---|---|---|---|---|---|---|---|
| 0.1828 | 0.1901 | 0.1943 | 0.2004 | 0.2020 | 0.2094 | 0.2178 | 0.2211 |
| 0.2352 | 0.2476 | 0.2543 | 0.2670 | 0.2777 | 0.2888 | 0.3122 | 0.3173 |
| 0.3262 | 0.3296 | 0.3385 | 0.3459 | 0.3484 | 0.3518 | 0.3635 | 0.3718 |
| 0.3804 | 0.3847 | 0.3918 | 0.3935 | 0.4019 | 0.4052 | 0.4172 | 0.4216 |
| 0.4315 | 0.4361 | 0.4427 | 0.4447 | 0.4570 | 0.4661 | 0.4688 | 0.4722 |
| 0.4844 | 0.4919 | 0.4922 | 0.5035 | 0.5100 | 0.5155 | 0.5201 | 0.5223 |
| 0.5274 | 0.5283 | 0.5370 | 0.5421 | 0.5461 | 0.5508 | 0.5549 | 0.5607 |
| 0.5642 | 0.5715 | 0.5756 | 0.5785 | 0.5917 | 0.5955 | 0.6027 | 0.6049 |
| 0.6098 | 0.6231 | 0.6287 | 0.6321 | 0.6408 | 0.6476 | 0.6559 | 0.6591 |
| 0.6650 | 0.6703 | 0.6766 | 0.6819 | 0.6911 | 0.6959 | 0.7007 | 0.7121 |
| 0.7278 | 0.7299 | 0.7327 | 0.7511 | 0.7588 | 0.7663 | 0.7775 | 0.7892 |
| 0.7924 | 0.7953 | 0.8057 | 0.8256 | 0.8345 | 0.8414 | 0.8583 | 0.8639 |
| 0.8711 | 0.8827 | 0.8883 | 0.8983 | 0.9016 | 0.9170 | 0.9294 | 0.9389 |
| 0.9519 | 0.9711 | 0.9809 | 0.9850 | 1.0041 | 1.0169 | 1.0226 | 1.0303 |
| 1.0421 | 1.0597 | 1.0841 | 1.0922 | 1.1274 | 1.1403 | 1.1565 | 1.1771 |
| 1.1970 | 1.2042 | 1.2223 | 1.2266 | 1.2585 | 1.2824 | 1.2984 | 1.3058 |
| 1.3114 | 1.3221 | 1.3340 | 1.3453 | 1.3528 | 1.3631 | 1.3687 | 1.3767 |
| 1.3842 | 1.3891 | 1.4071 | 1.4080 | 1.4196 | 1.4267 | 1.4335 | 1.4390 |
| 1.4444 | 1.4522 | 1.4555 | 1.4591 | 1.4664 | 1.4820 | 1.4917 | 1.4999 |
| 1.5075 | 1.5126 | 1.5239 | 1.5264 | 1.5365 | 1.5439 | 1.5545 | 1.5564 |
| 1.5625 | 1.5697 | 1.5717 | 1.5772 | 1.5951 | 1.6019 | 1.6075 | 1.6080 |
| 1.6220 | 1.6336 | 1.6416 | 1.6496 | 1.6625 | 1.6694 | 1.6731 | 1.6844 |
| 1.6949 | 1.6998 | 1.7117 | 1.7291 | 1.7318 | 1.7498 | 1.7586 | 1.7771 |
| 1.7837 | 1.7911 | 1.8078 | 1.8171 | 1.8386 | 1.8452 | 1.8655 | 1.8819 |
| 1.8903 | 1.9043 | 1.9157 | 1.9312 | 1.9549 | 1.9691 | 1.9855 | 1.9990 |
| 2.0025 | 2.0138 | 2.0244 | 2.0319 | 2.0364 | 2.0561 | 2.0792 | 2.0846 |
| 2.1107 | 2.1189 | 2.1356 | 2.1613 | 2.1663 | 2.1869 | 2.2182 | 2.2254 |
| 2.2317 | 2.2369 | 2.2511 | 2.2556 | 2.2584 | 2.2652 | 2.2874 | 2.2881 |
| 2.3117 | 2.3201 | 2.3345 | 2.3469 | 2.3634 | 2.3693 | 2.3956 | 2.3999 |
| 2.4040 | 2.4168 | 2.4402 | 2.4430 | 2.4504 | 2.4623 | 2.4707 | 2.4940 |
| 2.5036 | 2.5116 | 2.5263 | 2.5275 | 2.5409 | 2.5446 | 2.5466 | 2.5652 |
| 2.5729 | 2.5779 | 2.5861 | 2.5927 | 2.5939 | 2.6045 | 2.6112 | 2.6216 |
| 2.6383 | 2.6436 | 2.6635 | 2.6706 | 2.6738 | 2.6801 | 2.6924 | 2.7057 |
| 2.7174 | 2.7229 | 2.7301 | 2.7490 | 2.7556 | 2.7730 | 2.7805 | 2.7835 |
| 2.8067 | 2.8116 | 2.8210 | 2.8450 | 2.8546 | 2.8760 | 2.8813 | 2.8865 |
| 2.9021 | 2.9261 | 2.9669 | 3.0034 | 3.0476 | 3.0621 | 3.1294 | 3.1638 |
| 3.1681 | 3.2310 | 3.2539 | 3.2682 | 3.3072 | 3.3210 | 3.3675 | 3.4106 |
| 3.4259 | 3.4785 | 3.5280 | 3.5336 | 3.5585 | 3.5963 | 3.6137 | 3.6405 |
| 3.6885 | 3.7067 | 3.7148 | 3.7348 | 3.7404 | 3.7473 | 3.7527 | 3.7610 |
| 3.7678 | 3.8408 | 3.8647 | 3.9001 | 3.9403 | 3.9857 | 4.0289 | 4.0851 |
| 4.0921 | 4.2097 | 4.2922 | 4.3138 | 4.5945 | 4.6698 | 4.6926 | 4.7540 |
| 4.9277 | 5.0334 | 5.1995 | 23.5202 | 23.5823 | 23.7154 | 23.7309 | 23.7366 |
| 23.7786 | 23.8021 | 23.8327 | 23.8603 | 23.8907 | 23.9186 | 23.9616 | 23.9810 |
| 24.0300 | 35.4139 | 35.4622 | 35.5250 | 35.6257 | | | |

Ground-State Mulliken Net Atomic Charges

| Atom | Charge (a.u.) |
|---|---|
| 1 N | 0.396999 |
| 2 C | 0.140886 |
| 3 C | -0.022894 |
| 4 C | -0.220332 |
| 5 C | -0.301706 |
| 6 C | -0.008713 |
| 7 C | -0.792045 |
| 8 C | -0.136636 |
| 9 N | -0.163082 |
| 10 C | -0.287461 |
| 11 N | 0.043436 |
| 12 C | 0.239277 |
| 13 C | -0.121239 |
| 14 C | 0.076992 |
| 15 C | -0.312499 |
| 16 C | -0.291570 |
| 17 C | -0.507253 |
| 18 N | -0.491956 |
| 19 H | 0.221407 |
| 20 H | 0.237004 |

```
    21 H                    0.233346
    22 H                    0.220461
    23 H                    0.233935
    24 H                    0.233106
    25 H                    0.238860
    26 H                    0.171499
    27 H                    0.251827
    28 H                    0.391403
    29 H                    0.209565
    30 H                    0.252357
    31 H                    0.201414
    32 H                    0.221698
    33 H                    0.208110
    34 H                    0.176096
    35 H                    0.215328
    36 H                    0.141062
    37 H                    0.318020
    38 H                    0.383298
  ----------------------------------------
  Sum of atomic charges =     2.000000

 -----------------------------------------------------------------
                    Cartesian Multipole Moments
 -----------------------------------------------------------------
    Charge (ESU x 10^10)
                 9.6064
    Dipole Moment (Debye)
         X       8.2640      Y      -0.5547      Z      -0.5926
       Tot       8.3038
    Quadrupole Moments (Debye-Ang)
        XX      -5.2894     XY     -12.6322     YY     -74.2536
        XZ      -5.9374     YZ      -8.5340     ZZ     -75.0300
    Traceless Quadrupole Moments (Debye-Ang)
       QXX     138.7049    QYY     -68.1879    QZZ     -70.5170
       QXY     -37.8966    QXZ     -17.8122    QYZ     -25.6020
    Octopole Moments (Debye-Ang^2)
       XXX      65.8092    XXY     -94.7507    XYY      12.1556
       YYY      21.0388    XXZ      58.6635    XYZ      19.2772
       YYZ     -25.8359    XZZ       2.7555    YZZ       7.6915
       ZZZ      -3.3157
    Traceless Octopole Moments (Debye-Ang^2)
       XXX     260.6547    YYY     909.7660    ZZZ    -315.3429
       XXY   -1223.1998    XXZ     791.4165    XYY     -59.8267
       XYZ     289.1574    XZZ    -200.8280    YYZ    -476.0736
       YZZ     313.4338
    Hexadecapole Moments (Debye-Ang^3)
      XXXX   -3756.4649   XXXY     -84.0242   XXYY    -804.0722
      XYYY     287.6461   YYYY    -661.8214   XXXZ     355.9139
      XXYZ      41.3024   XYYZ      92.7975   YYYZ     -36.3172
      XXZZ    -882.7536   XYZZ      71.6425   YYZZ    -204.0177
      XZZZ     307.1747   YZZZ       9.6074   ZZZZ    -935.2227
    Traceless Hexadecapole Moments (Debye-Ang^3)
      XXXX   13250.5845   XXXY  -21209.4446   XXXZ    3356.0866
      XXYY   -5135.1344   XXYZ    4126.9761   XXZZ   -8115.4501
      XYYY   17815.9433   XYYZ   -1594.5538   XYZZ    3393.5013
      XZZZ   -1761.5328   YYYY   -1415.9963   YYYZ   -4442.6400
      YYZZ    6551.1307   YZZZ     315.6639   ZZZZ    1564.3194
 -----------------------------------------------------------------

-------------------------------------------------
-  Entering drvman on Sun Oct 13 22:16:17 2024  -
-------------------------------------------------

 Calculating analytic gradient of the SCF energy
 Gradient of SCF Energy
           1           2           3           4           5           6
    1   0.0002482  -0.0001209   0.0000331  -0.0000196   0.0001002  -0.0001515
    2  -0.0000294  -0.0002383   0.0000640   0.0000163  -0.0000906   0.0000368
    3  -0.0002108   0.0001935  -0.0001263   0.0002254  -0.0000805  -0.0001860
           7           8           9          10          11          12
```

```
    1  -0.0001926  -0.0001093   0.0004384  -0.0002619  -0.0002644   0.0005597
    2   0.0002939   0.0002591  -0.0005246   0.0002676  -0.0004039   0.0002638
    3  -0.0000730   0.0003175   0.0006774  -0.0003555  -0.0001867  -0.0013881
           13          14          15          16          17          18
    1   0.0005331  -0.0006537  -0.0000776   0.0000406   0.0000838  -0.0006677
    2   0.0002117  -0.0000623  -0.0002797   0.0000833   0.0000447   0.0002531
    3  -0.0003187   0.0003161  -0.0000538   0.0000658  -0.0000833   0.0006292
           19          20          21          22          23          24
    1   0.0000951   0.0000108   0.0000176  -0.0000071  -0.0002126  -0.0003661
    2  -0.0000013  -0.0000438  -0.0000614   0.0000355  -0.0000255   0.0002787
    3  -0.0000645  -0.0000544   0.0000226  -0.0000232  -0.0000853   0.0001390
           25          26          27          28          29          30
    1   0.0000182   0.0006113   0.0001868   0.0000695   0.0000430   0.0000874
    2  -0.0000199   0.0001029  -0.0002303   0.0000550   0.0000879   0.0001477
    3   0.0002050   0.0004106   0.0000394   0.0001370  -0.0000469   0.0000161
           31          32          33          34          35          36
    1  -0.0000407   0.0000107  -0.0000621  -0.0000704  -0.0000571  -0.0000523
    2   0.0000204  -0.0000640  -0.0000107  -0.0000199  -0.0000057   0.0000063
    3   0.0000432   0.0000253  -0.0000532   0.0000260  -0.0000035   0.0000289
           37          38
    1   0.0002017  -0.0000020
    2  -0.0006680   0.0002505
    3  -0.0002089   0.0000845
 Max gradient component =       1.388E-03
 RMS gradient           =       2.670E-04
 Gradient time:  CPU 111.66 s  wall 183.54 s

-------------------------------------------------
-  Entering optman on Sun Oct 13 22:19:21 2024  -
-------------------------------------------------

 Geometry Optimization Parameters
   NAtoms,    NIC,     NZ,  NCons,   NDum,   NFix, NCnnct, MaxDiis
       38     272       0       0       0       0       0       0

 Cartesian Hessian Update
 Hessian updated using BFGS update


** GEOMETRY OPTIMIZATION IN DELOCALIZED INTERNAL COORDINATES **
   Searching for a Minimum

   Optimization Cycle:   8

                      Coordinates (Angstroms)
     ATOM                X               Y               Z
      1  N        2.6915215275    0.1441227697   -0.1516272970
      2  C        2.8801227791   -0.7322651122   -1.1807058749
      3  C        3.8678412567   -1.6969480163   -1.0448153826
      4  C        4.6466178689   -1.7656291109    0.0999237591
      5  C        4.4353801589   -0.8495919845    1.1268892110
      6  C        3.4543309626    0.0957119974    0.9706700578
      7  C        2.0438540415   -0.6213765961   -2.4153805430
      8  C        1.6693153142    1.2100281990   -0.2498634437
      9  N       -1.3693213532   -0.6973350086    0.8751676650
     10  C       -2.3457141757   -0.2225526727    0.0532018318
     11  N       -2.0772490375    0.7139957237   -0.8162353531
     12  C       -0.8378732171    1.2294050983   -0.9093487068
     13  C        0.2481817499    0.7004675329   -0.1254460033
     14  C       -0.0934889515   -0.2546142878    0.7804989919
     15  C       -3.7294817622   -0.7550668782    0.1905731537
     16  C       -4.6494185356    0.2577689799    0.9090001431
     17  C       -4.2122607235    0.5689128347    2.3355397893
     18  N       -0.6986130439    2.2324026549   -1.7660522556
     19  H        4.0268076173   -2.3910697817   -1.8601835783
     20  H        5.4184048152   -2.5215440845    0.1886069677
     21  H        5.0282930757   -0.8582598716    2.0320648702
     22  H        3.2508988576    0.8468280584    1.7230602979
     23  H        2.3446557284   -1.3815721028   -3.1335540867
     24  H        2.1653776109    0.3534506309   -2.8966013858
```

```
    25  H          0.9834494679   -0.7676033381   -2.1964677292
    26  H          1.8300191806    1.7158811522   -1.2011721076
    27  H          1.8877849702    1.9396687816    0.5308625616
    28  H         -1.6109997352   -1.3948389444    1.5686119357
    29  H          0.6205783888   -0.7154865580    1.4528063917
    30  H         -4.1063713627   -0.9433469759   -0.8168658249
    31  H         -3.7173413713   -1.7047180408    0.7336974588
    32  H         -5.6517948088   -0.1745896393    0.9095631132
    33  H         -4.7004776241    1.1707086586    0.3102652780
    34  H         -4.1628218809   -0.3379946662    2.9467247681
    35  H         -4.9253521301    1.2410131354    2.8143544662
    36  H         -3.2357182369    1.0656998660    2.3712290188
    37  H          0.1509883541    2.7554641282   -1.8918829651
    38  H         -1.5201257759    2.5448734689   -2.2671091932
   Point Group: c1    Number of degrees of freedom:   108


   Energy is   -763.969334537

 Hessian updated using BFGS update
  internal optimization (0)

108 Hessian modes will be used to form the next step
  Hessian Eigenvalues:
     0.000855    0.002679    0.003392    0.004221    0.009429    0.011301
     0.018364    0.019059    0.019343    0.020002    0.020839    0.022481
     0.022740    0.023035    0.023923    0.024812    0.025757    0.026377
     0.027698    0.028245    0.029655    0.030528    0.031319    0.036340
     0.038777    0.041505    0.042870    0.043705    0.044350    0.045524
     0.048713    0.051479    0.054125    0.055173    0.063510    0.079606
     0.085563    0.093960    0.121689    0.122309    0.127126    0.130812
     0.131828    0.132984    0.136972    0.141376    0.142890    0.144397
     0.147687    0.148287    0.150323    0.152459    0.152804    0.153070
     0.157489    0.161261    0.193005    0.206341    0.206962    0.216785
     0.225552    0.229463    0.240359    0.247663    0.251909    0.260698
     0.268591    0.272157    0.281911    0.298561    0.300105    0.300572
     0.300865    0.301252    0.301702    0.302804    0.304110    0.304992
     0.305345    0.305727    0.307571    0.308560    0.312200    0.317464
     0.322608    0.332021    0.332771    0.334766    0.337112    0.340954
     0.343395    0.352554    0.355962    0.363346    0.381404    0.389348
     0.398532    0.407128    0.413219    0.418324    0.419843    0.430795
     0.438798    0.449165    0.469460    0.503948    0.569625    0.748182

 Minimum search - taking simple RFO step
 Searching for Lamda that Minimizes Along All modes
 Value Taken    Lamda =  -0.00082925
 Calculated Step too Large.  Step scaled by  0.480443
 Step Taken.  Stepsize is  0.300000

                             Maximum     Tolerance    Cnvgd?
         Gradient            0.003653     0.000800      NO
         Displacement        0.118363     0.001400      NO
         Energy change      -0.000349     0.000228      NO


 New Cartesian Coordinates Obtained by Inverse Iteration

 Displacement from previous Coordinates is:  0.868837
 ----------------------------------------------------------------
             Standard Nuclear Orientation (Angstroms)
    I     Atom           X                Y                Z
 ----------------------------------------------------------------
    1      N       2.6820893013     0.1449762751    -0.1686473529
    2      C       2.8985635751    -0.7468047570    -1.1793793684
    3      C       3.8837464442    -1.7076139303    -1.0035257446
    4      C       4.6369742888    -1.7529826868     0.1593297585
    5      C       4.4009300692    -0.8193204726     1.1646151160
    6      C       3.4200108159     0.1194929807     0.9710839180
    7      C       2.1005747043    -0.6503507084    -2.4404874796
    8      C       1.6532652155     1.1977490863    -0.3080897595
```

```
   9      N      -1.3709750073    -0.7424025479     0.8117531489
  10      C      -2.3631516796    -0.2175118270     0.0423563825
  11      N      -2.1075178525     0.7550042864    -0.7911663940
  12      C      -0.8639633431     1.2566422819    -0.8963328354
  13      C       0.2356621833     0.6826393716    -0.1655573292
  14      C      -0.0920750130    -0.3098767769     0.7036145449
  15      C      -3.7505706218    -0.7379780191     0.1938957326
  16      C      -4.6468481476     0.2713005883     0.9446342535
  17      C      -4.1746041353     0.5609626974     2.3644177681
  18      N      -0.7342462462     2.2992709656    -1.7068751921
  19      H       4.0634211486    -2.4157545197    -1.8025141424
  20      H       5.4089819233    -2.5045789585     0.2788245074
  21      H       4.9744924975    -0.8105793438     2.0822555260
  22      H       3.1958591940     0.8814016010     1.7066901962
  23      H       2.3845922031    -1.4529649014    -3.1182576166
  24      H       2.2901643294     0.2960424318    -2.9565983509
  25      H       1.0278869195    -0.7310652473    -2.2511768028
  26      H       1.8081186284     1.6660514539    -1.2799190383
  27      H       1.8637634326     1.9605711034     0.4431819141
  28      H      -1.6025329418    -1.4704135308     1.4767479856
  29      H       0.6327808687    -0.8098330394     1.3350103494
  30      H      -4.1465900971    -0.9048911416    -0.8103297787
  31      H      -3.7394222120    -1.6978344105     0.7188429515
  32      H      -5.6526230263    -0.1528577895     0.9628377471
  33      H      -4.7036218309     1.1925482038     0.3593705745
  34      H      -4.1201530810    -0.3534625997     2.9636926619
  35      H      -4.8692766794     1.2350800978     2.8669116115
  36      H      -3.1918807556     1.0465216723     2.3822379810
  37      H       0.1276393664     2.7942340891    -1.8620166809
  38      H      -1.5594644384     2.6285880216    -2.1914307630
 ----------------------------------------------------------------
 Nuclear Repulsion Energy =        1313.28194137 hartrees
 There are       65 alpha and       65 beta electrons

-------------------------------------------------
-  Entering fldman on Sun Oct 13 22:19:21 2024  -
-------------------------------------------------

 Applying Cartesian multipole field
    Component          Value
    ---------          -----
     (2,0,0)        1.00000E-12
     (0,2,0)        2.00000E-11
     (0,0,2)       -3.00000E-11
 Nucleus-field energy     =    -0.0000000068 hartrees

-------------------------------------------------
-  Entering gesman on Sun Oct 13 22:19:21 2024  -
-------------------------------------------------

 Requested basis set is 6-311+G(d,p)
 There are 188 shells and 516 basis functions
 A cutoff of  1.0D-12 yielded  12826 shell pairs
 There are    101828 function pairs (    108549 Cartesian)
 Smallest overlap matrix eigenvalue = 2.18E-06
 Linear dependence detected in AO basis
 Tighter screening thresholds may be required for diffuse basis sets
 Use S2THRESH > 12 and THRESH = 14 in case of SCF convergence issues
 Number of orthogonalized atomic orbitals = 510
 Maximum deviation from orthogonality = 1.083E-11
 Guess MOs from SCF MO coefficient file
 Reading MOs from coefficient file
 Reading MOs from coefficient file

-------------------------------------------------
-  Entering scfman on Sun Oct 13 22:19:21 2024  -
-------------------------------------------------

 Long-range K will be added via erf
 Coulomb attenuation parameter = 0.2 bohr**(-1)
```

```
 A restricted hybrid HF-DFT SCF calculation will be
 performed using Pulay DIIS + Geometric Direct Minimization
 Exchange:     0.2220 Hartree-Fock + 1.0000 wB97X-D + LR-HF
 Correlation:  1.0000 wB97X-D
 Using Euler-Maclaurin-Lebedev (75,302) quadrature formula
 Dispersion:   Grimme D
 SCF converges when RMS gradient is below 1.0E-07
 Geometry optimization detected.  Setting ReadMinima to 0
 Setting SaveMinima to 0
 ---------------------------------------
  Cycle       Energy         DIIS Error
 ---------------------------------------
    1    -763.9709556441      8.20E-04
    2    -763.9689337347      9.67E-05
    3    -763.9695411410      6.46E-05
    4    -763.9696572256      3.16E-05
    5    -763.9696961234      6.01E-06
    6    -763.9696978162      2.80E-06
    7    -763.9696981474      7.98E-07
    8    -763.9696981927      3.45E-07
    9    -763.9696981987      1.10E-07
   10    -763.9696981997      5.18E-08 Convergence criterion met
 ---------------------------------------
 SCF time:  CPU 267.05 s  wall 427.36 s
 SCF   energy in the final basis set = -763.96969820
 Total energy in the final basis set = -763.96969820

--------------------------------------------------
-  Entering anlman on Sun Oct 13 22:26:28 2024  -
--------------------------------------------------


 --------------------------------------------------------------

                   Orbital Energies (a.u.)
 --------------------------------------------------------------

 Alpha MOs
 -- Occupied --
-14.8203 -14.7881 -14.7371 -14.7048 -10.6758 -10.6596 -10.6457 -10.6426
-10.6384 -10.6299 -10.6066 -10.5872 -10.5756 -10.5746 -10.5532 -10.5245
-10.4906 -10.4665  -1.4145  -1.4082  -1.3467  -1.2740  -1.2325  -1.2001
 -1.1917  -1.1417  -1.1017  -1.0870  -1.0540  -1.0507  -1.0366  -1.0048
 -0.9921  -0.9543  -0.9454  -0.9160  -0.9066  -0.8910  -0.8853  -0.8624
 -0.8570  -0.8473  -0.8348  -0.8251  -0.8097  -0.7995  -0.7895  -0.7880
 -0.7715  -0.7576  -0.7536  -0.7519  -0.7447  -0.7324  -0.7213  -0.7174
 -0.6968  -0.6671  -0.6610  -0.6418  -0.6307  -0.6250  -0.6239  -0.6216
 -0.6080
 -- Virtual --
 -0.2732  -0.2578  -0.2395  -0.2286  -0.1744  -0.1657  -0.1504  -0.1448
 -0.1349  -0.1202  -0.1179  -0.1139  -0.1118  -0.1043  -0.1014  -0.0969
 -0.0905  -0.0852  -0.0830  -0.0787  -0.0752  -0.0707  -0.0699  -0.0679
 -0.0632  -0.0589  -0.0551  -0.0530  -0.0488  -0.0478  -0.0445  -0.0345
 -0.0311  -0.0285  -0.0272  -0.0239  -0.0180  -0.0154  -0.0105  -0.0070
 -0.0049  -0.0039   0.0012   0.0035   0.0045   0.0099   0.0123   0.0165
  0.0186   0.0229   0.0261   0.0299   0.0334   0.0363   0.0393   0.0433
  0.0447   0.0461   0.0508   0.0537   0.0569   0.0588   0.0611   0.0622
  0.0689   0.0716   0.0768   0.0779   0.0790   0.0805   0.0911   0.0916
  0.0971   0.1021   0.1026   0.1045   0.1106   0.1122   0.1159   0.1182
  0.1237   0.1292   0.1302   0.1357   0.1386   0.1432   0.1487   0.1496
  0.1534   0.1560   0.1591   0.1631   0.1671   0.1725   0.1796   0.1809
  0.1823   0.1880   0.1948   0.1980   0.2008   0.2103   0.2185   0.2215
  0.2365   0.2474   0.2549   0.2700   0.2822   0.2945   0.3129   0.3180
  0.3260   0.3310   0.3406   0.3447   0.3511   0.3526   0.3627   0.3687
  0.3809   0.3849   0.3912   0.3929   0.3990   0.4035   0.4171   0.4207
  0.4280   0.4338   0.4413   0.4462   0.4573   0.4652   0.4667   0.4733
  0.4850   0.4901   0.4923   0.5043   0.5097   0.5152   0.5173   0.5201
  0.5258   0.5286   0.5358   0.5443   0.5463   0.5511   0.5527   0.5616
  0.5652   0.5705   0.5751   0.5788   0.5900   0.5967   0.6004   0.6034
  0.6123   0.6238   0.6265   0.6314   0.6401   0.6434   0.6562   0.6601
```

| | | | | | | | |
|---|---|---|---|---|---|---|---|
| 0.6658 | 0.6705 | 0.6778 | 0.6839 | 0.6913 | 0.6958 | 0.6988 | 0.7091 |
| 0.7280 | 0.7334 | 0.7353 | 0.7508 | 0.7623 | 0.7643 | 0.7761 | 0.7846 |
| 0.7933 | 0.7973 | 0.8043 | 0.8236 | 0.8303 | 0.8412 | 0.8589 | 0.8645 |
| 0.8730 | 0.8819 | 0.8918 | 0.8983 | 0.9084 | 0.9193 | 0.9293 | 0.9411 |
| 0.9557 | 0.9703 | 0.9805 | 0.9850 | 1.0018 | 1.0214 | 1.0303 | 1.0318 |
| 1.0437 | 1.0605 | 1.0871 | 1.0954 | 1.1274 | 1.1434 | 1.1541 | 1.1786 |
| 1.1940 | 1.2063 | 1.2223 | 1.2270 | 1.2585 | 1.2847 | 1.2976 | 1.3013 |
| 1.3087 | 1.3182 | 1.3337 | 1.3456 | 1.3504 | 1.3609 | 1.3694 | 1.3757 |
| 1.3837 | 1.3888 | 1.4063 | 1.4068 | 1.4204 | 1.4271 | 1.4343 | 1.4385 |
| 1.4440 | 1.4472 | 1.4531 | 1.4595 | 1.4665 | 1.4803 | 1.4882 | 1.4994 |
| 1.5060 | 1.5116 | 1.5230 | 1.5269 | 1.5363 | 1.5428 | 1.5526 | 1.5578 |
| 1.5603 | 1.5660 | 1.5737 | 1.5762 | 1.5973 | 1.6021 | 1.6060 | 1.6093 |
| 1.6200 | 1.6321 | 1.6427 | 1.6529 | 1.6645 | 1.6706 | 1.6726 | 1.6866 |
| 1.6954 | 1.6978 | 1.7145 | 1.7282 | 1.7310 | 1.7510 | 1.7579 | 1.7784 |
| 1.7821 | 1.7925 | 1.8059 | 1.8179 | 1.8377 | 1.8425 | 1.8636 | 1.8806 |
| 1.8903 | 1.9054 | 1.9137 | 1.9313 | 1.9569 | 1.9675 | 1.9866 | 2.0002 |
| 2.0084 | 2.0157 | 2.0264 | 2.0316 | 2.0368 | 2.0578 | 2.0828 | 2.0836 |
| 2.1134 | 2.1175 | 2.1353 | 2.1614 | 2.1678 | 2.1876 | 2.2163 | 2.2253 |
| 2.2326 | 2.2379 | 2.2502 | 2.2570 | 2.2573 | 2.2659 | 2.2876 | 2.2892 |
| 2.3125 | 2.3167 | 2.3333 | 2.3452 | 2.3619 | 2.3680 | 2.3969 | 2.4013 |
| 2.4045 | 2.4174 | 2.4415 | 2.4446 | 2.4532 | 2.4618 | 2.4713 | 2.4891 |
| 2.5071 | 2.5125 | 2.5250 | 2.5287 | 2.5409 | 2.5427 | 2.5469 | 2.5654 |
| 2.5729 | 2.5793 | 2.5875 | 2.5908 | 2.5946 | 2.6088 | 2.6111 | 2.6180 |
| 2.6344 | 2.6401 | 2.6629 | 2.6729 | 2.6756 | 2.6821 | 2.6918 | 2.7079 |
| 2.7180 | 2.7241 | 2.7305 | 2.7458 | 2.7492 | 2.7734 | 2.7812 | 2.7865 |
| 2.8062 | 2.8130 | 2.8211 | 2.8444 | 2.8584 | 2.8770 | 2.8840 | 2.8884 |
| 2.9045 | 2.9159 | 2.9555 | 3.0041 | 3.0507 | 3.0591 | 3.1205 | 3.1552 |
| 3.1686 | 3.2297 | 3.2557 | 3.2645 | 3.3059 | 3.3230 | 3.3690 | 3.4094 |
| 3.4263 | 3.4777 | 3.5289 | 3.5326 | 3.5590 | 3.5961 | 3.6129 | 3.6413 |
| 3.6866 | 3.7039 | 3.7165 | 3.7356 | 3.7387 | 3.7458 | 3.7515 | 3.7601 |
| 3.7677 | 3.8413 | 3.8642 | 3.8994 | 3.9405 | 3.9857 | 4.0296 | 4.0820 |
| 4.0921 | 4.2107 | 4.2910 | 4.3164 | 4.5941 | 4.6702 | 4.6899 | 4.7622 |
| 4.9276 | 5.0318 | 5.2006 | 23.5214 | 23.5834 | 23.7171 | 23.7304 | 23.7366 |
| 23.7789 | 23.8022 | 23.8318 | 23.8606 | 23.8907 | 23.9194 | 23.9608 | 23.9818 |
| 24.0292 | 35.4121 | 35.4623 | 35.5252 | 35.6262 | | | |

Ground-State Mulliken Net Atomic Charges

| Atom | | Charge (a.u.) |
|---|---|---|
| 1 | N | 0.381172 |
| 2 | C | 0.134715 |
| 3 | C | -0.021002 |
| 4 | C | -0.210230 |
| 5 | C | -0.310776 |
| 6 | C | 0.021456 |
| 7 | C | -0.802477 |
| 8 | C | -0.133262 |
| 9 | N | -0.154191 |
| 10 | C | -0.316141 |
| 11 | N | 0.046008 |
| 12 | C | 0.239392 |
| 13 | C | -0.102985 |
| 14 | C | 0.060190 |
| 15 | C | -0.313682 |
| 16 | C | -0.291855 |
| 17 | C | -0.504231 |
| 18 | N | -0.483923 |
| 19 | H | 0.221924 |
| 20 | H | 0.237162 |
| 21 | H | 0.233614 |
| 22 | H | 0.221315 |
| 23 | H | 0.232625 |
| 24 | H | 0.241326 |
| 25 | H | 0.233784 |
| 26 | H | 0.173935 |
| 27 | H | 0.253895 |
| 28 | H | 0.388687 |
| 29 | H | 0.209540 |

```
    30 H                      0.253627
    31 H                      0.198818
    32 H                      0.221195
    33 H                      0.208653
    34 H                      0.175960
    35 H                      0.214701
    36 H                      0.141637
    37 H                      0.315946
    38 H                      0.383480
 ----------------------------------------
 Sum of atomic charges =     2.000000

 -----------------------------------------------------------------
                    Cartesian Multipole Moments
 -----------------------------------------------------------------
    Charge (ESU x 10^10)
                 9.6064
    Dipole Moment (Debye)
         X       8.2840      Y      -0.6328      Z      -0.7750
       Tot       8.3442
    Quadrupole Moments (Debye-Ang)
        XX      -5.5317     XY     -12.7159     YY     -73.5079
        XZ      -5.8208     YZ      -8.7265     ZZ     -75.5346
    Traceless Quadrupole Moments (Debye-Ang)
       QXX     137.9790    QYY     -65.9495    QZZ     -72.0295
       QXY     -38.1478    QXZ     -17.4623    QYZ     -26.1796
    Octopole Moments (Debye-Ang^2)
       XXX      65.7771    XXY     -94.3633    XYY      11.7248
       YYY      21.1310    XXZ      58.7191    XYZ      18.6261
       YYZ     -25.8824    XZZ       4.6952    YZZ       7.6233
       ZZZ      -4.1063
    Traceless Octopole Moments (Debye-Ang^2)
       XXX     246.8826    YYY     907.4458    ZZZ    -320.1681
       XXY   -1218.6221    XXZ     794.5960    XYY     -70.7197
       XYZ     279.3917    XZZ    -176.1629    YYZ    -474.4279
       YZZ     311.1763
    Hexadecapole Moments (Debye-Ang^3)
      XXXX   -3774.0864   XXXY     -73.9220   XXYY    -805.4144
      XYYY     288.9515   YYYY    -669.2315   XXXZ     359.8052
      XXYZ      38.0230   XYYZ      95.4024   YYYZ     -39.1378
      XXZZ    -876.3398   XYZZ      72.4517   YYZZ    -206.3716
      XZZZ     305.1115   YZZZ      15.3630   ZZZZ    -935.6068
    Traceless Hexadecapole Moments (Debye-Ang^3)
      XXXX   12349.9977   XXXY  -20698.4648   XXXZ    3565.1862
      XXYY   -4981.1729   XXYZ    3784.1375   XXZZ   -7368.8248
      XYYY   17403.2574   XYYZ   -1387.5344   XYZZ    3295.2074
      XZZZ   -2177.6519   YYYY   -1374.3206   YYYZ   -4734.3053
      YYZZ    6355.4935   YZZZ     950.1678   ZZZZ    1013.3313
 -----------------------------------------------------------------

------------------------------------------------
-  Entering drvman on Sun Oct 13 22:26:28 2024  -
------------------------------------------------

 Calculating analytic gradient of the SCF energy
 Gradient of SCF Energy
           1           2           3           4           5           6
    1  -0.0000846   0.0000580  -0.0000155  -0.0001075   0.0001007  -0.0000432
    2   0.0000823  -0.0001696  -0.0000941   0.0002426  -0.0002155   0.0001954
    3   0.0000185   0.0000570   0.0000001   0.0003232  -0.0002458  -0.0002879
           7           8           9          10          11          12
    1  -0.0004468  -0.0000775   0.0003155   0.0004251  -0.0005518   0.0009096
    2  -0.0000509   0.0002272  -0.0004143   0.0001102  -0.0000452  -0.0009976
    3  -0.0000529   0.0000864   0.0004593   0.0000010  -0.0003236  -0.0009264
          13          14          15          16          17          18
    1   0.0000562  -0.0006755  -0.0001920   0.0001731   0.0000060  -0.0007780
    2   0.0000454   0.0001202  -0.0000791  -0.0000667   0.0000617   0.0013022
    3  -0.0004240   0.0002799   0.0001030  -0.0001321  -0.0000621   0.0013267
          19          20          21          22          23          24
    1   0.0001463   0.0000718   0.0000707   0.0000824  -0.0000329  -0.0002244
```

```
    2  -0.0001040  -0.0000509  -0.0001275   0.0000230   0.0001155   0.0002817
    3  -0.0000635  -0.0000276   0.0000466   0.0000678  -0.0001674   0.0000549
            25          26          27          28          29          30
    1   0.0000510   0.0006289   0.0001157   0.0000074  -0.0000154   0.0000189
    2   0.0000494   0.0002977  -0.0001326  -0.0000083   0.0000687   0.0001235
    3   0.0001575   0.0004840   0.0000304   0.0001739   0.0000085  -0.0000119
            31          32          33          34          35          36
    1  -0.0000431  -0.0000316  -0.0000678  -0.0000451  -0.0000976  -0.0000328
    2  -0.0000262  -0.0000512   0.0000329  -0.0000085   0.0000279  -0.0000280
    3  -0.0000084   0.0000959  -0.0000165   0.0000009  -0.0000312  -0.0000358
            37          38
    1   0.0004860  -0.0001603
    2  -0.0007119  -0.0000255
    3  -0.0005276  -0.0004307
 Max gradient component =       1.327E-03
 RMS gradient           =       3.214E-04
 Gradient time:  CPU 119.70 s  wall 183.61 s

-------------------------------------------------
-  Entering optman on Sun Oct 13 22:29:32 2024  -
-------------------------------------------------

 Geometry Optimization Parameters
   NAtoms,    NIC,     NZ,  NCons,   NDum,   NFix, NCnnct, MaxDiis
       38     272       0       0       0       0       0       0

 Cartesian Hessian Update
 Hessian updated using BFGS update


** GEOMETRY OPTIMIZATION IN DELOCALIZED INTERNAL COORDINATES **
   Searching for a Minimum

   Optimization Cycle:   9

                       Coordinates (Angstroms)
     ATOM                X               Y               Z
      1  N         2.6820893013    0.1449762751   -0.1686473529
      2  C         2.8985635751   -0.7468047570   -1.1793793684
      3  C         3.8837464442   -1.7076139303   -1.0035257446
      4  C         4.6369742888   -1.7529826868    0.1593297585
      5  C         4.4009300692   -0.8193204726    1.1646151160
      6  C         3.4200108159    0.1194929807    0.9710839180
      7  C         2.1005747043   -0.6503507084   -2.4404874796
      8  C         1.6532652155    1.1977490863   -0.3080897595
      9  N        -1.3709750073   -0.7424025479    0.8117531489
     10  C        -2.3631516796   -0.2175118270    0.0423563825
     11  N        -2.1075178525    0.7550042864   -0.7911663940
     12  C        -0.8639633431    1.2566422819   -0.8963328354
     13  C         0.2356621833    0.6826393716   -0.1655573292
     14  C        -0.0920750130   -0.3098767769    0.7036145449
     15  C        -3.7505706218   -0.7379780191    0.1938957326
     16  C        -4.6468481476    0.2713005883    0.9446342535
     17  C        -4.1746041353    0.5609626974    2.3644177681
     18  N        -0.7342462462    2.2992709656   -1.7068751921
     19  H         4.0634211486   -2.4157545197   -1.8025141424
     20  H         5.4089819233   -2.5045789585    0.2788245074
     21  H         4.9744924975   -0.8105793438    2.0822555260
     22  H         3.1958591940    0.8814016010    1.7066901962
     23  H         2.3845922031   -1.4529649014   -3.1182576166
     24  H         2.2901643294    0.2960424318   -2.9565983509
     25  H         1.0278869195   -0.7310652473   -2.2511768028
     26  H         1.8081186284    1.6660514539   -1.2799190383
     27  H         1.8637634326    1.9605711034    0.4431819141
     28  H        -1.6025329418   -1.4704135308    1.4767479856
     29  H         0.6327808687   -0.8098330394    1.3350103494
     30  H        -4.1465900971   -0.9048911416   -0.8103297787
     31  H        -3.7394222120   -1.6978344105    0.7188429515
     32  H        -5.6526230263   -0.1528577895    0.9628377471
     33  H        -4.7036218309    1.1925482038    0.3593705745
```

```
    34  H        -4.1201530810   -0.3534625997    2.9636926619
    35  H        -4.8692766794    1.2350800978    2.8669116115
    36  H        -3.1918807556    1.0465216723    2.3822379810
    37  H         0.1276393664    2.7942340891   -1.8620166809
    38  H        -1.5594644384    2.6285880216   -2.1914307630
  Point Group: c1    Number of degrees of freedom:   108


  Energy is   -763.969698200

 Hessian updated using BFGS update
  internal optimization (0)

108 Hessian modes will be used to form the next step
  Hessian Eigenvalues:
     0.000444    0.002689    0.003439    0.004243    0.009282    0.011785
     0.017410    0.018958    0.019261    0.019920    0.020532    0.022572
     0.022811    0.022849    0.023978    0.024757    0.025749    0.026714
     0.028205    0.028418    0.029602    0.030441    0.031435    0.036346
     0.038803    0.041475    0.042880    0.043679    0.044344    0.045595
     0.050943    0.054012    0.054567    0.057614    0.060789    0.079821
     0.085574    0.093509    0.121689    0.122308    0.127133    0.130844
     0.131931    0.132976    0.136900    0.141407    0.143049    0.144507
     0.147569    0.147687    0.150130    0.152531    0.153041    0.153490
     0.157493    0.161616    0.193241    0.206360    0.207049    0.217036
     0.225976    0.229262    0.239858    0.247657    0.252143    0.261032
     0.268774    0.273261    0.281798    0.298331    0.300109    0.300591
     0.301030    0.301283    0.301709    0.302820    0.304111    0.304985
     0.305217    0.305395    0.307607    0.308599    0.312147    0.318172
     0.321679    0.332135    0.333600    0.334798    0.337135    0.339743
     0.342924    0.351796    0.356624    0.363451    0.381375    0.390349
     0.398573    0.407744    0.415822    0.418260    0.421551    0.437904
     0.438727    0.449297    0.473322    0.506234    0.568548    0.726188

 Minimum search - taking simple RFO step
 Searching for Lamda that Minimizes Along All modes
 Value Taken    Lamda =  -0.00098704
 Calculated Step too Large.  Step scaled by  0.397017
 Step Taken.  Stepsize is  0.300000

                             Maximum     Tolerance    Cnvgd?
        Gradient            0.003307      0.000800      NO
        Displacement        0.121064      0.001400      NO
        Energy change      -0.000364      0.000228      NO


 New Cartesian Coordinates Obtained by Inverse Iteration

 Displacement from previous Coordinates is:  0.903688
 ----------------------------------------------------------------
             Standard Nuclear Orientation (Angstroms)
    I     Atom           X                Y                Z
 ----------------------------------------------------------------
    1      N       2.6714680226     0.1453426673    -0.1874860476
    2      C       2.9175259124    -0.7616634863    -1.1776790084
    3      C       3.9013480561    -1.7153792848    -0.9609928105
    4      C       4.6268869828    -1.7360319059     0.2201330824
    5      C       4.3627537890    -0.7857680771     1.2028361361
    6      C       3.3814436620     0.1438637031     0.9702895007
    7      C       2.1584549243    -0.6839607702    -2.4637888794
    8      C       1.6365737744     1.1836972002    -0.3701051190
    9      N      -1.3764088216    -0.7837375901     0.7436758895
   10      C      -2.3816641446    -0.2097733401     0.0298111275
   11      N      -2.1356815533     0.7969632711    -0.7659660173
   12      C      -0.8881065250     1.2837371851    -0.8839728307
   13      C       0.2223304150     0.6652691906    -0.2083450026
   14      C      -0.0941084369    -0.3630814755     0.6217922283
   15      C      -3.7724101165    -0.7175127039     0.1964348201
   16      C      -4.6422472776     0.2861334403     0.9835678530
   17      C      -4.1338956795     0.5476891263     2.3962997990
```

```
   18      N      -0.7628819745     2.3615152817    -1.6495589020
   19      H       4.1036721134    -2.4366494563    -1.7426518064
   20      H       5.3995090399    -2.4812965520     0.3715073862
   21      H       4.9148954977    -0.7580011377     2.1331692160
   22      H       3.1338432263     0.9158271518     1.6877506888
   23      H       2.4222849829    -1.5278212837    -3.0983770616
   24      H       2.4140762806     0.2285339317    -3.0123503887
   25      H       1.0778303777    -0.7018111292    -2.3071696605
   26      H       1.7850295775     1.6100407937    -1.3626316850
   27      H       1.8393538916     1.9796265494     0.3488667429
   28      H      -1.6001174259    -1.5411080542     1.3778751461
   29      H       0.6393021924    -0.9020902551     1.2094824453
   30      H      -4.1890475628    -0.8600632438    -0.8035056086
   31      H      -3.7627102420    -1.6884825742     0.7006253237
   32      H      -5.6515657029    -0.1285582881     1.0181622386
   33      H      -4.7030685662     1.2177931114     0.4155486515
   34      H      -4.0742663883    -0.3767457470     2.9793308510
   35      H      -4.8090476983     1.2209460070     2.9258063337
   36      H      -3.1456875978     1.0224740179     2.3973785972
   37      H       0.1061878589     2.8384027073    -1.8225666345
   38      H      -1.5918548638     2.7116810192    -2.1131965943
 ----------------------------------------------------------------
 Nuclear Repulsion Energy =        1312.64956817 hartrees
 There are       65 alpha and       65 beta electrons

--------------------------------------------------
-  Entering fldman on Sun Oct 13 22:29:32 2024  -
--------------------------------------------------

 Applying Cartesian multipole field
    Component          Value
    ---------          -----
     (2,0,0)        1.00000E-12
     (0,2,0)        2.00000E-11
     (0,0,2)       -3.00000E-11
 Nucleus-field energy     =    -0.0000000065 hartrees

--------------------------------------------------
-  Entering gesman on Sun Oct 13 22:29:32 2024  -
--------------------------------------------------

 Requested basis set is 6-311+G(d,p)
 There are 188 shells and 516 basis functions
 A cutoff of  1.0D-12 yielded  12814 shell pairs
 There are    101747 function pairs (    108458 Cartesian)
 Smallest overlap matrix eigenvalue = 2.19E-06
 Linear dependence detected in AO basis
 Tighter screening thresholds may be required for diffuse basis sets
 Use S2THRESH > 12 and THRESH = 14 in case of SCF convergence issues
 Number of orthogonalized atomic orbitals = 510
 Maximum deviation from orthogonality = 8.743E-12
 Guess MOs from SCF MO coefficient file
 Reading MOs from coefficient file
 Reading MOs from coefficient file

--------------------------------------------------
-  Entering scfman on Sun Oct 13 22:29:32 2024  -
--------------------------------------------------

 Long-range K will be added via erf
 Coulomb attenuation parameter = 0.2 bohr**(-1)
 A restricted hybrid HF-DFT SCF calculation will be
 performed using Pulay DIIS + Geometric Direct Minimization
 Exchange:     0.2220 Hartree-Fock + 1.0000 wB97X-D + LR-HF
 Correlation:  1.0000 wB97X-D
 Using Euler-Maclaurin-Lebedev (75,302) quadrature formula
 Dispersion:   Grimme D
 SCF converges when RMS gradient is below 1.0E-07
 Geometry optimization detected.  Setting ReadMinima to 0
 Setting SaveMinima to 0
```

```
 ---------------------------------------
  Cycle       Energy         DIIS Error
 ---------------------------------------
    1    -763.9672935015      8.34E-04
    2    -763.9692769866      9.85E-05
    3    -763.9699026326      6.56E-05
    4    -763.9700203165      3.20E-05
    5    -763.9700595745      6.18E-06
    6    -763.9700614152      2.76E-06
    7    -763.9700617419      8.25E-07
    8    -763.9700617899      3.62E-07
    9    -763.9700617963      1.18E-07
   10    -763.9700617975      5.09E-08 Convergence criterion met
 ---------------------------------------
 SCF time:  CPU 256.00 s  wall 438.16 s
 SCF   energy in the final basis set = -763.97006180
 Total energy in the final basis set = -763.97006180

--------------------------------------------------
-  Entering anlman on Sun Oct 13 22:36:50 2024  -
--------------------------------------------------



 ---------------------------------------------------------------

                   Orbital Energies (a.u.)
 ---------------------------------------------------------------

 Alpha MOs
 -- Occupied --
-14.8207 -14.7879 -14.7369 -14.7045 -10.6755 -10.6593 -10.6455 -10.6430
-10.6386 -10.6303 -10.6067 -10.5876 -10.5760 -10.5750 -10.5531 -10.5243
-10.4900 -10.4661  -1.4145  -1.4085  -1.3462  -1.2738  -1.2327  -1.2001
 -1.1920  -1.1418  -1.1018  -1.0871  -1.0541  -1.0503  -1.0369  -1.0045
 -0.9922  -0.9541  -0.9453  -0.9156  -0.9063  -0.8907  -0.8860  -0.8621
 -0.8566  -0.8474  -0.8355  -0.8253  -0.8092  -0.8000  -0.7904  -0.7879
 -0.7715  -0.7572  -0.7530  -0.7523  -0.7444  -0.7327  -0.7213  -0.7171
 -0.6963  -0.6666  -0.6613  -0.6416  -0.6302  -0.6253  -0.6233  -0.6215
 -0.6079
 -- Virtual --
 -0.2737  -0.2575  -0.2388  -0.2286  -0.1746  -0.1657  -0.1506  -0.1449
 -0.1351  -0.1186  -0.1178  -0.1140  -0.1116  -0.1044  -0.1018  -0.0969
 -0.0908  -0.0854  -0.0832  -0.0789  -0.0755  -0.0710  -0.0701  -0.0689
 -0.0628  -0.0591  -0.0553  -0.0532  -0.0496  -0.0478  -0.0454  -0.0343
 -0.0314  -0.0282  -0.0260  -0.0244  -0.0182  -0.0148  -0.0104  -0.0061
 -0.0045  -0.0042   0.0010   0.0027   0.0044   0.0100   0.0125   0.0167
  0.0186   0.0225   0.0264   0.0298   0.0339   0.0352   0.0394   0.0435
  0.0441   0.0451   0.0505   0.0542   0.0563   0.0584   0.0615   0.0628
  0.0681   0.0718   0.0753   0.0773   0.0787   0.0810   0.0906   0.0927
  0.0957   0.1017   0.1048   0.1061   0.1104   0.1113   0.1161   0.1188
  0.1242   0.1284   0.1296   0.1348   0.1385   0.1439   0.1477   0.1493
  0.1550   0.1560   0.1587   0.1629   0.1683   0.1735   0.1779   0.1809
  0.1830   0.1855   0.1945   0.1960   0.1999   0.2122   0.2182   0.2222
  0.2375   0.2444   0.2549   0.2728   0.2875   0.2980   0.3133   0.3178
  0.3257   0.3332   0.3415   0.3427   0.3525   0.3547   0.3628   0.3647
  0.3796   0.3849   0.3900   0.3912   0.3966   0.4012   0.4149   0.4212
  0.4218   0.4323   0.4391   0.4487   0.4578   0.4639   0.4667   0.4743
  0.4843   0.4892   0.4934   0.5040   0.5112   0.5143   0.5153   0.5193
  0.5243   0.5293   0.5360   0.5444   0.5475   0.5502   0.5517   0.5624
  0.5651   0.5690   0.5753   0.5794   0.5884   0.5969   0.6005   0.6012
  0.6139   0.6241   0.6245   0.6293   0.6377   0.6443   0.6561   0.6605
  0.6658   0.6702   0.6771   0.6856   0.6901   0.6961   0.6972   0.7058
  0.7266   0.7361   0.7412   0.7503   0.7614   0.7644   0.7759   0.7797
  0.7954   0.7985   0.8044   0.8211   0.8265   0.8410   0.8562   0.8645
  0.8739   0.8806   0.8942   0.8979   0.9120   0.9170   0.9321   0.9436
  0.9594   0.9686   0.9772   0.9885   0.9985   1.0227   1.0301   1.0400
  1.0469   1.0600   1.0909   1.0989   1.1261   1.1468   1.1541   1.1818
  1.1940   1.2069   1.2244   1.2295   1.2572   1.2846   1.2957   1.2988
  1.3064   1.3109   1.3339   1.3437   1.3503   1.3600   1.3711   1.3745
  1.3822   1.3897   1.4041   1.4064   1.4176   1.4274   1.4348   1.4371
```

| | | | | | | | |
|---|---|---|---|---|---|---|---|
| 1.4410 | 1.4451 | 1.4504 | 1.4589 | 1.4667 | 1.4759 | 1.4855 | 1.4971 |
| 1.5054 | 1.5153 | 1.5224 | 1.5277 | 1.5384 | 1.5407 | 1.5509 | 1.5564 |
| 1.5589 | 1.5648 | 1.5752 | 1.5764 | 1.5989 | 1.6006 | 1.6055 | 1.6132 |
| 1.6169 | 1.6304 | 1.6443 | 1.6555 | 1.6648 | 1.6720 | 1.6739 | 1.6859 |
| 1.6958 | 1.6981 | 1.7169 | 1.7275 | 1.7318 | 1.7520 | 1.7577 | 1.7793 |
| 1.7806 | 1.7938 | 1.8044 | 1.8178 | 1.8378 | 1.8394 | 1.8625 | 1.8816 |
| 1.8898 | 1.9057 | 1.9111 | 1.9322 | 1.9588 | 1.9667 | 1.9877 | 2.0019 |
| 2.0109 | 2.0181 | 2.0283 | 2.0315 | 2.0409 | 2.0589 | 2.0834 | 2.0873 |
| 2.1137 | 2.1175 | 2.1351 | 2.1617 | 2.1695 | 2.1880 | 2.2142 | 2.2254 |
| 2.2336 | 2.2386 | 2.2492 | 2.2570 | 2.2596 | 2.2671 | 2.2877 | 2.2907 |
| 2.3128 | 2.3129 | 2.3327 | 2.3439 | 2.3622 | 2.3679 | 2.3973 | 2.4028 |
| 2.4055 | 2.4176 | 2.4420 | 2.4447 | 2.4570 | 2.4617 | 2.4720 | 2.4839 |
| 2.5095 | 2.5127 | 2.5240 | 2.5294 | 2.5404 | 2.5415 | 2.5471 | 2.5639 |
| 2.5742 | 2.5779 | 2.5878 | 2.5899 | 2.5950 | 2.6067 | 2.6113 | 2.6182 |
| 2.6303 | 2.6409 | 2.6613 | 2.6725 | 2.6789 | 2.6855 | 2.6914 | 2.7096 |
| 2.7189 | 2.7256 | 2.7334 | 2.7428 | 2.7457 | 2.7740 | 2.7809 | 2.7879 |
| 2.8033 | 2.8135 | 2.8195 | 2.8456 | 2.8614 | 2.8749 | 2.8861 | 2.8884 |
| 2.9021 | 2.9072 | 2.9475 | 3.0046 | 3.0545 | 3.0561 | 3.1120 | 3.1493 |
| 3.1697 | 3.2289 | 3.2567 | 3.2625 | 3.3034 | 3.3240 | 3.3701 | 3.4083 |
| 3.4253 | 3.4775 | 3.5295 | 3.5329 | 3.5596 | 3.5947 | 3.6149 | 3.6433 |
| 3.6849 | 3.7013 | 3.7180 | 3.7359 | 3.7376 | 3.7446 | 3.7503 | 3.7578 |
| 3.7684 | 3.8416 | 3.8637 | 3.8990 | 3.9408 | 3.9853 | 4.0302 | 4.0785 |
| 4.0925 | 4.2113 | 4.2902 | 4.3185 | 4.5939 | 4.6703 | 4.6870 | 4.7707 |
| 4.9271 | 5.0305 | 5.2018 | 23.5238 | 23.5844 | 23.7187 | 23.7296 | 23.7368 |
| 23.7778 | 23.8020 | 23.8309 | 23.8608 | 23.8912 | 23.9199 | 23.9602 | 23.9821 |
| 24.0283 | 35.4110 | 35.4623 | 35.5252 | 35.6265 | | | |

Ground-State Mulliken Net Atomic Charges

| Atom | Charge (a.u.) |
|---|---|
| 1 N | 0.365900 |
| 2 C | 0.129442 |
| 3 C | -0.025398 |
| 4 C | -0.202190 |
| 5 C | -0.314386 |
| 6 C | 0.041163 |
| 7 C | -0.804708 |
| 8 C | -0.140943 |
| 9 N | -0.146187 |
| 10 C | -0.336344 |
| 11 N | 0.047404 |
| 12 C | 0.237974 |
| 13 C | -0.078604 |
| 14 C | 0.043513 |
| 15 C | -0.315620 |
| 16 C | -0.291033 |
| 17 C | -0.501689 |
| 18 N | -0.473736 |
| 19 H | 0.222443 |
| 20 H | 0.237357 |
| 21 H | 0.233822 |
| 22 H | 0.221994 |
| 23 H | 0.231062 |
| 24 H | 0.248731 |
| 25 H | 0.228023 |
| 26 H | 0.181644 |
| 27 H | 0.255473 |
| 28 H | 0.386017 |
| 29 H | 0.209532 |
| 30 H | 0.254833 |
| 31 H | 0.196646 |
| 32 H | 0.220754 |
| 33 H | 0.209102 |
| 34 H | 0.175808 |
| 35 H | 0.214182 |
| 36 H | 0.142074 |
| 37 H | 0.314356 |
| 38 H | 0.381588 |

```
 ----------------------------------------
 Sum of atomic charges =     2.000000

 -----------------------------------------------------------------
                    Cartesian Multipole Moments
 -----------------------------------------------------------------
    Charge (ESU x 10^10)
                 9.6064
    Dipole Moment (Debye)
         X       8.2758      Y      -0.6923      Z      -0.9456
       Tot       8.3584
    Quadrupole Moments (Debye-Ang)
        XX      -5.7704     XY     -12.7959     YY     -72.7068
        XZ      -5.7524     YZ      -8.7944     ZZ     -76.0974
    Traceless Quadrupole Moments (Debye-Ang)
       QXX     137.2635    QYY     -63.5459    QZZ     -73.7177
       QXY     -38.3878    QXZ     -17.2571    QYZ     -26.3833
    Octopole Moments (Debye-Ang^2)
       XXX      65.4544    XXY     -93.9816    XYY      11.1119
       YYY      21.5924    XXZ      58.7038    XYZ      17.9502
       YYZ     -25.9271    XZZ       6.6649    YZZ       7.3863
       ZZZ      -4.2490
    Traceless Octopole Moments (Debye-Ang^2)
       XXX     232.7345    YYY     908.9125    ZZZ    -320.4842
       XXY   -1214.7155    XXZ     794.9740    XYY     -83.0148
       XYZ     269.2534    XZZ    -149.7197    YYZ    -474.4898
       YZZ     305.8030
    Hexadecapole Moments (Debye-Ang^3)
      XXXX   -3788.6176   XXXY     -63.7936   XXYY    -807.1355
      XYYY     289.9840   YYYY    -673.4315   XXXZ     363.0230
      XXYZ      34.8412   XYYZ      98.2896   YYYZ     -42.7585
      XXZZ    -869.3005   XYZZ      73.0515   YYZZ    -210.0706
      XZZZ     303.3156   YZZZ      21.3678   ZZZZ    -937.7420
    Traceless Hexadecapole Moments (Debye-Ang^3)
      XXXX   11494.7341   XXXY  -20164.2164   XXXZ    3709.1518
      XXYY   -4932.2682   XXYZ    3462.0893   XXZZ   -6562.4659
      XYYY   16982.4340   XYYZ   -1149.0168   XYZZ    3181.7824
      XZZZ   -2560.1350   YYYY   -1108.1640   YYYZ   -5078.3668
      YYZZ    6040.4323   YZZZ    1616.2775   ZZZZ     522.0337
 -----------------------------------------------------------------


--------------------------------------------------
-  Entering drvman on Sun Oct 13 22:36:50 2024  -
--------------------------------------------------


 Calculating analytic gradient of the SCF energy
 Gradient of SCF Energy
           1           2           3           4           5           6
    1  -0.0001461   0.0000534  -0.0002110  -0.0000628   0.0001378   0.0000178
    2   0.0001168  -0.0000770  -0.0001222   0.0002609  -0.0002399   0.0003167
    3   0.0001559   0.0001156  -0.0001501   0.0003659  -0.0002492  -0.0003710
           7           8           9          10          11          12
    1  -0.0003737   0.0000178   0.0001968   0.0007388  -0.0006449   0.0010029
    2  -0.0000255   0.0004562  -0.0001835  -0.0001244   0.0003426  -0.0020338
    3  -0.0000023   0.0000614   0.0002365   0.0004374  -0.0004335  -0.0005319
          13          14          15          16          17          18
    1  -0.0002803  -0.0005768  -0.0002543   0.0002946  -0.0000702  -0.0006771
    2  -0.0001948   0.0001706   0.0000272  -0.0001729   0.0000767   0.0021629
    3  -0.0007557   0.0004555   0.0001347  -0.0002258  -0.0000207   0.0016921
          19          20          21          22          23          24
    1   0.0001797   0.0000959   0.0001133   0.0001292   0.0000199  -0.0001122
    2  -0.0001481  -0.0000547  -0.0001414   0.0000065   0.0001300   0.0001946
    3  -0.0000360   0.0000202   0.0000455   0.0001344  -0.0001663   0.0000085
          25          26          27          28          29          30
    1   0.0000206   0.0005504   0.0000050  -0.0000305  -0.0001346  -0.0000519
    2   0.0000496   0.0001647  -0.0000644  -0.0000820  -0.0000264   0.0000821
    3   0.0001099   0.0003279   0.0000583   0.0001775   0.0000534  -0.0000111
          31          32          33          34          35          36
    1  -0.0000448  -0.0000537  -0.0000459  -0.0000229  -0.0001167  -0.0000199
    2  -0.0000707  -0.0000355   0.0000490   0.0000140   0.0000517  -0.0000275
```

```
    3  -0.0000461   0.0001205   0.0000035  -0.0000155  -0.0000666  -0.0000800
          37          38
    1   0.0006388  -0.0002824
    2  -0.0005890  -0.0002591
    3  -0.0006958  -0.0008568
 Max gradient component =       2.163E-03
 RMS gradient           =       4.250E-04
 Gradient time:  CPU 114.50 s  wall 193.20 s

-------------------------------------------------
-  Entering optman on Sun Oct 13 22:40:04 2024  -
-------------------------------------------------

 Geometry Optimization Parameters
   NAtoms,    NIC,     NZ,  NCons,   NDum,   NFix, NCnnct, MaxDiis
       38     272       0       0       0       0       0       0

 Cartesian Hessian Update
 Hessian updated using BFGS update


** GEOMETRY OPTIMIZATION IN DELOCALIZED INTERNAL COORDINATES **
   Searching for a Minimum

   Optimization Cycle:  10

                    Coordinates (Angstroms)
     ATOM              X              Y              Z
      1  N        2.6714680226    0.1453426673   -0.1874860476
      2  C        2.9175259124   -0.7616634863   -1.1776790084
      3  C        3.9013480561   -1.7153792848   -0.9609928105
      4  C        4.6268869828   -1.7360319059    0.2201330824
      5  C        4.3627537890   -0.7857680771    1.2028361361
      6  C        3.3814436620    0.1438637031    0.9702895007
      7  C        2.1584549243   -0.6839607702   -2.4637888794
      8  C        1.6365737744    1.1836972002   -0.3701051190
      9  N       -1.3764088216   -0.7837375901    0.7436758895
     10  C       -2.3816641446   -0.2097733401    0.0298111275
     11  N       -2.1356815533    0.7969632711   -0.7659660173
     12  C       -0.8881065250    1.2837371851   -0.8839728307
     13  C        0.2223304150    0.6652691906   -0.2083450026
     14  C       -0.0941084369   -0.3630814755    0.6217922283
     15  C       -3.7724101165   -0.7175127039    0.1964348201
     16  C       -4.6422472776    0.2861334403    0.9835678530
     17  C       -4.1338956795    0.5476891263    2.3962997990
     18  N       -0.7628819745    2.3615152817   -1.6495589020
     19  H        4.1036721134   -2.4366494563   -1.7426518064
     20  H        5.3995090399   -2.4812965520    0.3715073862
     21  H        4.9148954977   -0.7580011377    2.1331692160
     22  H        3.1338432263    0.9158271518    1.6877506888
     23  H        2.4222849829   -1.5278212837   -3.0983770616
     24  H        2.4140762806    0.2285339317   -3.0123503887
     25  H        1.0778303777   -0.7018111292   -2.3071696605
     26  H        1.7850295775    1.6100407937   -1.3626316850
     27  H        1.8393538916    1.9796265494    0.3488667429
     28  H       -1.6001174259   -1.5411080542    1.3778751461
     29  H        0.6393021924   -0.9020902551    1.2094824453
     30  H       -4.1890475628   -0.8600632438   -0.8035056086
     31  H       -3.7627102420   -1.6884825742    0.7006253237
     32  H       -5.6515657029   -0.1285582881    1.0181622386
     33  H       -4.7030685662    1.2177931114    0.4155486515
     34  H       -4.0742663883   -0.3767457470    2.9793308510
     35  H       -4.8090476983    1.2209460070    2.9258063337
     36  H       -3.1456875978    1.0224740179    2.3973785972
     37  H        0.1061878589    2.8384027073   -1.8225666345
     38  H       -1.5918548638    2.7116810192   -2.1131965943
   Point Group: c1    Number of degrees of freedom:   108


   Energy is   -763.970061798
```

Hessian updated using BFGS update
internal optimization (0)

108 Hessian modes will be used to form the next step
Hessian Eigenvalues:

| | | | | | |
|---|---|---|---|---|---|
| 0.000684 | 0.002689 | 0.003269 | 0.004242 | 0.008862 | 0.011950 |
| 0.015208 | 0.018945 | 0.019242 | 0.019923 | 0.020381 | 0.022484 |
| 0.022804 | 0.022886 | 0.023999 | 0.024832 | 0.025709 | 0.026784 |
| 0.028212 | 0.028847 | 0.029389 | 0.030429 | 0.031446 | 0.036343 |
| 0.038791 | 0.041378 | 0.042875 | 0.043632 | 0.044351 | 0.045440 |
| 0.050823 | 0.054033 | 0.054523 | 0.057162 | 0.059672 | 0.079912 |
| 0.085585 | 0.093288 | 0.121689 | 0.122341 | 0.127136 | 0.130817 |
| 0.131952 | 0.132993 | 0.136869 | 0.141422 | 0.143662 | 0.144886 |
| 0.146753 | 0.147688 | 0.150011 | 0.152523 | 0.153032 | 0.153659 |
| 0.157497 | 0.161566 | 0.193137 | 0.206400 | 0.207275 | 0.217243 |
| 0.226411 | 0.229011 | 0.239429 | 0.247951 | 0.252229 | 0.261362 |
| 0.268857 | 0.273053 | 0.281652 | 0.296914 | 0.300094 | 0.300568 |
| 0.300822 | 0.301237 | 0.301721 | 0.302676 | 0.304109 | 0.304330 |
| 0.305048 | 0.305365 | 0.307596 | 0.308695 | 0.312094 | 0.317294 |
| 0.320722 | 0.332138 | 0.333496 | 0.335088 | 0.337139 | 0.338556 |
| 0.342767 | 0.351094 | 0.356395 | 0.363470 | 0.381528 | 0.390432 |
| 0.398530 | 0.407511 | 0.417403 | 0.418296 | 0.421798 | 0.437753 |
| 0.442423 | 0.449682 | 0.479237 | 0.511375 | 0.567703 | 0.706601 |

Minimum search - taking simple RFO step
Searching for Lamda that Minimizes Along All modes
Value Taken    Lamda =  -0.00088513
Calculated Step too Large.  Step scaled by  0.456786
Step Taken.  Stepsize is  0.300000

| | Maximum | Tolerance | Cnvgd? |
|---|---|---|---|
| Gradient | 0.003463 | 0.000800 | NO |
| Displacement | 0.122780 | 0.001400 | NO |
| Energy change | -0.000364 | 0.000228 | NO |

New Cartesian Coordinates Obtained by Inverse Iteration

Displacement from previous Coordinates is:  1.011020

Standard Nuclear Orientation (Angstroms)

| I | Atom | X | Y | Z |
|---|---|---|---|---|
| 1 | N | 2.6577120948 | 0.1458798530 | -0.2104998035 |
| 2 | C | 2.9383932450 | -0.7772609237 | -1.1759917515 |
| 3 | C | 3.9205591454 | -1.7210670348 | -0.9121916234 |
| 4 | C | 4.6127268554 | -1.7135086687 | 0.2888150457 |
| 5 | C | 4.3145965434 | -0.7462538905 | 1.2451380649 |
| 6 | C | 3.3335801710 | 0.1711016398 | 0.9676057573 |
| 7 | C | 2.2225213144 | -0.7256351087 | -2.4877235995 |
| 8 | C | 1.6171674064 | 1.1672404014 | -0.4405798422 |
| 9 | N | -1.3867177568 | -0.8237102834 | 0.6643567867 |
| 10 | C | -2.4025439766 | -0.1985958973 | 0.0128564468 |
| 11 | N | -2.1635314520 | 0.8424388010 | -0.7403452625 |
| 12 | C | -0.9119847741 | 1.3121540183 | -0.8729413528 |
| 13 | C | 0.2065819790 | 0.6479431298 | -0.2574316878 |
| 14 | C | -0.1007580920 | -0.4167961687 | 0.5277912599 |
| 15 | C | -3.7967553510 | -0.6905769335 | 0.1982499710 |
| 16 | C | -4.6332641784 | 0.3042312455 | 1.0305064313 |
| 17 | C | -4.0831010612 | 0.5257869575 | 2.4344702181 |
| 18 | N | -0.7868402245 | 2.4212511079 | -1.5937943996 |
| 19 | H | 4.1489044079 | -2.4563016433 | -1.6733725366 |
| 20 | H | 5.3856458444 | -2.4501268406 | 0.4764849212 |
| 21 | H | 4.8402647108 | -0.6965073893 | 2.1897707544 |
| 22 | H | 3.0581443206 | 0.9532731873 | 1.6635954156 |
| 23 | H | 2.4748925941 | -1.6042674505 | -3.0784869965 |
| 24 | H | 2.5365671821 | 0.1523299738 | -3.0623018802 |
| 25 | H | 1.1373684041 | -0.6927757113 | -2.3707020110 |
| 26 | H | 1.7587212807 | 1.5495909733 | -1.4525241854 |

```
  27      H       1.8116838611     1.9963402083     0.2431196586
  28      H      -1.6048180225    -1.6106344716     1.2635049531
  29      H       0.6396254223    -0.9961003321     1.0662872274
  30      H      -4.2382237272    -0.8023856576    -0.7952387041
  31      H      -3.7895682637    -1.6747234552     0.6763059643
  32      H      -5.6468471846    -0.0980673994     1.0824181045
  33      H      -4.6961429308     1.2499083946     0.4865226864
  34      H      -4.0192353317    -0.4126932362     2.9939699857
  35      H      -4.7338333806     1.1953414457     2.9981174794
  36      H      -3.0887350971     0.9874103489     2.4180867653
  37      H       0.0869627136     2.8867878391    -1.7756087681
  38      H      -1.6197186914     2.7989789716    -2.0282394928
 ----------------------------------------------------------------
 Nuclear Repulsion Energy =        1312.17002815 hartrees
 There are       65 alpha and       65 beta electrons

--------------------------------------------------
-  Entering fldman on Sun Oct 13 22:40:04 2024  -
--------------------------------------------------

 Applying Cartesian multipole field
    Component          Value
    ---------          -----
     (2,0,0)        1.00000E-12
     (0,2,0)        2.00000E-11
     (0,0,2)       -3.00000E-11
 Nucleus-field energy     =    -0.0000000063 hartrees

--------------------------------------------------
-  Entering gesman on Sun Oct 13 22:40:04 2024  -
--------------------------------------------------

 Requested basis set is 6-311+G(d,p)
 There are 188 shells and 516 basis functions
 A cutoff of  1.0D-12 yielded  12816 shell pairs
 There are    101736 function pairs (    108442 Cartesian)
 Smallest overlap matrix eigenvalue = 2.20E-06
 Linear dependence detected in AO basis
 Tighter screening thresholds may be required for diffuse basis sets
 Use S2THRESH > 12 and THRESH = 14 in case of SCF convergence issues
 Number of orthogonalized atomic orbitals = 510
 Maximum deviation from orthogonality = 1.829E-11
 Guess MOs from SCF MO coefficient file
 Reading MOs from coefficient file
 Reading MOs from coefficient file

--------------------------------------------------
-  Entering scfman on Sun Oct 13 22:40:04 2024  -
--------------------------------------------------

 Long-range K will be added via erf
 Coulomb attenuation parameter = 0.2 bohr**(-1)
 A restricted hybrid HF-DFT SCF calculation will be
 performed using Pulay DIIS + Geometric Direct Minimization
 Exchange:     0.2220 Hartree-Fock + 1.0000 wB97X-D + LR-HF
 Correlation:  1.0000 wB97X-D
 Using Euler-Maclaurin-Lebedev (75,302) quadrature formula
 Dispersion:   Grimme D
 SCF converges when RMS gradient is below 1.0E-07
 Geometry optimization detected.  Setting ReadMinima to 0
 Setting SaveMinima to 0
 ---------------------------------------
  Cycle       Energy         DIIS Error
 ---------------------------------------
    1    -763.9635881531      9.15E-04
    2    -763.9694665812      1.09E-04
    3    -763.9701957270      7.33E-05
    4    -763.9703420680      3.44E-05
    5    -763.9703869368      7.34E-06
    6    -763.9703895162      2.98E-06
```

```
    7    -763.9703899119      9.29E-07
    8    -763.9703899725      4.15E-07
    9    -763.9703899813      1.36E-07
   10    -763.9703899828      5.85E-08 Convergence criterion met
 ---------------------------------------
 SCF time:  CPU 238.91 s  wall 430.41 s
 SCF   energy in the final basis set = -763.97038998
 Total energy in the final basis set = -763.97038998

-------------------------------------------------
-  Entering anlman on Sun Oct 13 22:47:14 2024  -
-------------------------------------------------



 --------------------------------------------------------------

                   Orbital Energies (a.u.)
 --------------------------------------------------------------

 Alpha MOs
 -- Occupied --
-14.8210 -14.7877 -14.7367 -14.7042 -10.6751 -10.6591 -10.6453 -10.6434
-10.6388 -10.6307 -10.6068 -10.5881 -10.5764 -10.5753 -10.5529 -10.5242
-10.4895 -10.4658  -1.4147  -1.4088  -1.3459  -1.2736  -1.2329  -1.2002
 -1.1924  -1.1419  -1.1020  -1.0872  -1.0543  -1.0500  -1.0371  -1.0043
 -0.9922  -0.9540  -0.9452  -0.9153  -0.9059  -0.8906  -0.8867  -0.8619
 -0.8561  -0.8474  -0.8363  -0.8255  -0.8087  -0.8007  -0.7913  -0.7877
 -0.7715  -0.7567  -0.7530  -0.7523  -0.7440  -0.7329  -0.7213  -0.7170
 -0.6959  -0.6662  -0.6617  -0.6414  -0.6297  -0.6256  -0.6229  -0.6215
 -0.6079
 -- Virtual --
 -0.2741  -0.2571  -0.2381  -0.2286  -0.1748  -0.1657  -0.1509  -0.1449
 -0.1355  -0.1180  -0.1166  -0.1140  -0.1112  -0.1046  -0.1020  -0.0966
 -0.0912  -0.0856  -0.0833  -0.0796  -0.0757  -0.0712  -0.0704  -0.0694
 -0.0625  -0.0594  -0.0554  -0.0536  -0.0501  -0.0478  -0.0456  -0.0344
 -0.0313  -0.0280  -0.0253  -0.0244  -0.0189  -0.0140  -0.0106  -0.0050
 -0.0048  -0.0038   0.0008   0.0019   0.0047   0.0102   0.0126   0.0164
  0.0191   0.0221   0.0267   0.0288   0.0337   0.0351   0.0393   0.0424
  0.0435   0.0449   0.0506   0.0525   0.0574   0.0588   0.0619   0.0634
  0.0675   0.0717   0.0736   0.0768   0.0788   0.0826   0.0894   0.0930
  0.0951   0.1003   0.1056   0.1077   0.1101   0.1118   0.1160   0.1195
  0.1236   0.1272   0.1303   0.1341   0.1378   0.1442   0.1473   0.1500
  0.1545   0.1565   0.1600   0.1629   0.1687   0.1739   0.1761   0.1815
  0.1827   0.1844   0.1918   0.1960   0.1992   0.2136   0.2173   0.2242
  0.2376   0.2424   0.2545   0.2755   0.2923   0.2975   0.3137   0.3165
  0.3248   0.3355   0.3398   0.3426   0.3515   0.3572   0.3616   0.3651
  0.3781   0.3848   0.3862   0.3895   0.3955   0.3996   0.4111   0.4194
  0.4221   0.4312   0.4370   0.4503   0.4594   0.4630   0.4677   0.4762
  0.4834   0.4881   0.4942   0.5041   0.5132   0.5139   0.5142   0.5195
  0.5242   0.5295   0.5379   0.5415   0.5477   0.5508   0.5522   0.5621
  0.5653   0.5663   0.5760   0.5806   0.5872   0.5954   0.6000   0.6021
  0.6137   0.6225   0.6240   0.6288   0.6365   0.6457   0.6549   0.6603
  0.6650   0.6695   0.6767   0.6839   0.6899   0.6964   0.6971   0.7031
  0.7259   0.7393   0.7445   0.7496   0.7610   0.7654   0.7747   0.7778
  0.7969   0.7985   0.8066   0.8183   0.8240   0.8408   0.8520   0.8633
  0.8732   0.8811   0.8964   0.8976   0.9128   0.9149   0.9358   0.9490
  0.9611   0.9672   0.9783   0.9912   0.9956   1.0238   1.0259   1.0438
  1.0482   1.0593   1.0941   1.1015   1.1247   1.1445   1.1598   1.1856
  1.1947   1.2061   1.2248   1.2338   1.2548   1.2801   1.2922   1.2950
  1.3042   1.3100   1.3342   1.3407   1.3519   1.3605   1.3722   1.3749
  1.3814   1.3907   1.4016   1.4061   1.4137   1.4277   1.4323   1.4357
  1.4372   1.4451   1.4480   1.4592   1.4667   1.4717   1.4848   1.4956
  1.5074   1.5184   1.5211   1.5293   1.5383   1.5414   1.5493   1.5540
  1.5588   1.5652   1.5760   1.5784   1.5972   1.6005   1.6057   1.6147
  1.6188   1.6282   1.6459   1.6573   1.6648   1.6737   1.6757   1.6826
  1.6981   1.6991   1.7179   1.7278   1.7339   1.7519   1.7586   1.7795
  1.7806   1.7948   1.8035   1.8168   1.8367   1.8389   1.8627   1.8835
  1.8892   1.9055   1.9088   1.9346   1.9607   1.9677   1.9890   2.0040
  2.0116   2.0208   2.0272   2.0356   2.0449   2.0600   2.0847   2.0911
  2.1131   2.1184   2.1353   2.1612   2.1708   2.1886   2.2123   2.2264
```

| | | | | | | | |
|---|---|---|---|---|---|---|---|
| 2.2347 | 2.2391 | 2.2489 | 2.2576 | 2.2629 | 2.2680 | 2.2876 | 2.2912 |
| 2.3094 | 2.3126 | 2.3335 | 2.3420 | 2.3658 | 2.3681 | 2.3970 | 2.4042 |
| 2.4063 | 2.4175 | 2.4417 | 2.4439 | 2.4590 | 2.4641 | 2.4726 | 2.4800 |
| 2.5086 | 2.5135 | 2.5220 | 2.5297 | 2.5391 | 2.5409 | 2.5471 | 2.5618 |
| 2.5733 | 2.5763 | 2.5866 | 2.5902 | 2.5959 | 2.6018 | 2.6124 | 2.6226 |
| 2.6286 | 2.6423 | 2.6594 | 2.6720 | 2.6806 | 2.6893 | 2.6919 | 2.7116 |
| 2.7199 | 2.7256 | 2.7374 | 2.7413 | 2.7499 | 2.7744 | 2.7810 | 2.7876 |
| 2.7992 | 2.8129 | 2.8190 | 2.8483 | 2.8589 | 2.8673 | 2.8846 | 2.8916 |
| 2.9008 | 2.9091 | 2.9462 | 3.0047 | 3.0524 | 3.0591 | 3.1069 | 3.1472 |
| 3.1715 | 3.2290 | 3.2572 | 3.2630 | 3.3005 | 3.3248 | 3.3712 | 3.4079 |
| 3.4247 | 3.4779 | 3.5302 | 3.5339 | 3.5610 | 3.5926 | 3.6175 | 3.6458 |
| 3.6831 | 3.6988 | 3.7190 | 3.7353 | 3.7368 | 3.7433 | 3.7495 | 3.7553 |
| 3.7693 | 3.8416 | 3.8633 | 3.8987 | 3.9414 | 3.9848 | 4.0313 | 4.0757 |
| 4.0933 | 4.2116 | 4.2893 | 4.3209 | 4.5940 | 4.6704 | 4.6848 | 4.7799 |
| 4.9268 | 5.0293 | 5.2041 | 23.5269 | 23.5854 | 23.7207 | 23.7290 | 23.7374 |
| 23.7764 | 23.8019 | 23.8297 | 23.8610 | 23.8917 | 23.9203 | 23.9598 | 23.9836 |
| 24.0277 | 35.4107 | 35.4626 | 35.5257 | 35.6278 | | | |

--------------------------------------------------------------

Ground-State Mulliken Net Atomic Charges

| Atom | Charge (a.u.) |
|---|---|
| 1 N | 0.350421 |
| 2 C | 0.130417 |
| 3 C | -0.036804 |
| 4 C | -0.196552 |
| 5 C | -0.309572 |
| 6 C | 0.042929 |
| 7 C | -0.802672 |
| 8 C | -0.151369 |
| 9 N | -0.139256 |
| 10 C | -0.351260 |
| 11 N | 0.047874 |
| 12 C | 0.240205 |
| 13 C | -0.051506 |
| 14 C | 0.029563 |
| 15 C | -0.317454 |
| 16 C | -0.289599 |
| 17 C | -0.499501 |
| 18 N | -0.467216 |
| 19 H | 0.222817 |
| 20 H | 0.237542 |
| 21 H | 0.233982 |
| 22 H | 0.222537 |
| 23 H | 0.229623 |
| 24 H | 0.254746 |
| 25 H | 0.222107 |
| 26 H | 0.193152 |
| 27 H | 0.256002 |
| 28 H | 0.383393 |
| 29 H | 0.209460 |
| 30 H | 0.255917 |
| 31 H | 0.194795 |
| 32 H | 0.220425 |
| 33 H | 0.209449 |
| 34 H | 0.175649 |
| 35 H | 0.213737 |
| 36 H | 0.142145 |
| 37 H | 0.314481 |
| 38 H | 0.379390 |

----------------------------------------
Sum of atomic charges = 2.000000

-----------------------------------------------------------------
Cartesian Multipole Moments
-----------------------------------------------------------------

Charge (ESU x 10^10)
9.6064
Dipole Moment (Debye)

```
         X        8.2350      Y      -0.7300      Z      -1.1219
       Tot        8.3431
    Quadrupole Moments (Debye-Ang)
        XX      -6.1151     XY     -12.8805     YY     -71.8065
        XZ      -5.7291     YZ      -8.7392     ZZ     -76.7092
    Traceless Quadrupole Moments (Debye-Ang)
       QXX     136.2854    QYY     -60.7888    QZZ     -75.4967
       QXY     -38.6415    QXZ     -17.1874    QYZ     -26.2177
    Octopole Moments (Debye-Ang^2)
       XXX      64.5286    XXY     -93.5303    XYY      10.3327
       YYY      22.4687    XXZ      58.6247    XYZ      17.1744
       YYZ     -25.9517    XZZ       8.8754    YZZ       6.9892
       ZZZ      -3.6569
    Traceless Octopole Moments (Debye-Ang^2)
       XXX     214.2982    YYY     913.6816    ZZZ    -315.9984
       XXY   -1210.7370    XXZ     792.3227    XYY     -96.2191
       XYZ     257.6160    XZZ    -118.0790    YYZ    -476.3244
       YZZ     297.0554
    Hexadecapole Moments (Debye-Ang^3)
      XXXX   -3800.3427   XXXY     -52.8017   XXYY    -808.5812
      XYYY     290.5019   YYYY    -674.3460   XXXZ     365.4340
      XXYZ      31.5422   XYYZ     101.5088   YYYZ     -47.1691
      XXZZ    -860.5871   XYZZ      73.2533   YYZZ    -215.5964
      XZZZ     301.1856   YZZZ      28.0139   ZZZZ    -942.7653
    Traceless Hexadecapole Moments (Debye-Ang^3)
      XXXX   10537.1490   XXXY  -19537.0870   XXXZ    3804.7916
      XXYY   -4941.4533   XXYZ    3126.3320   XXZZ   -5595.6957
      XYYY   16509.7912   XYYZ    -863.5020   XYZZ    3027.2958
      XZZZ   -2941.2897   YYYY    -622.0583   YYYZ   -5509.5453
      YYZZ    5563.5116   YZZZ    2383.2133   ZZZZ      32.1841
 -----------------------------------------------------------------

 -------------------------------------------------
 -  Entering drvman on Sun Oct 13 22:47:14 2024  -
 -------------------------------------------------

 Calculating analytic gradient of the SCF energy
 Gradient of SCF Energy
           1           2           3           4           5           6
    1  -0.0003341   0.0000289  -0.0002908  -0.0000484   0.0002001   0.0001698
    2   0.0001715   0.0000972  -0.0002075   0.0002964  -0.0003281   0.0003661
    3   0.0003358  -0.0000864  -0.0000995   0.0004013  -0.0002923  -0.0002848
           7           8           9          10          11          12
    1  -0.0004115   0.0000795   0.0000039   0.0010175  -0.0004677   0.0009129
    2  -0.0000745   0.0004016   0.0002155  -0.0003209   0.0005825  -0.0027659
    3   0.0000876  -0.0002000  -0.0001093   0.0006680  -0.0003869  -0.0000192
          13          14          15          16          17          18
    1  -0.0006175  -0.0004215  -0.0002397   0.0002855  -0.0000887  -0.0004903
    2  -0.0005783   0.0003583   0.0001659  -0.0001917   0.0000423   0.0024894
    3  -0.0007464   0.0004640   0.0001941  -0.0002589   0.0000159   0.0018059
          19          20          21          22          23          24
    1   0.0001515   0.0000974   0.0001504   0.0001339   0.0001895   0.0000779
    2  -0.0001686  -0.0000376  -0.0001397  -0.0000061   0.0001104   0.0000651
    3  -0.0000085   0.0000476   0.0000322   0.0001711  -0.0001268  -0.0000207
          25          26          27          28          29          30
    1  -0.0000321   0.0003000  -0.0001363  -0.0000748  -0.0001093  -0.0001228
    2   0.0002098   0.0001493   0.0000287  -0.0001476  -0.0001293   0.0000192
    3   0.0000633   0.0001122   0.0001163   0.0001347   0.0000586  -0.0000255
          31          32          33          34          35          36
    1  -0.0000172  -0.0000698  -0.0000077  -0.0000050  -0.0001065  -0.0000142
    2  -0.0000935  -0.0000067   0.0000499   0.0000477   0.0000668  -0.0000226
    3  -0.0000859   0.0001223   0.0000168  -0.0000124  -0.0000975  -0.0001085
          37          38
    1   0.0006869  -0.0003798
    2  -0.0003231  -0.0003919
    3  -0.0007336  -0.0011445
 Max gradient component =       2.766E-03
 RMS gradient           =       4.912E-04
 Gradient time:  CPU 105.73 s  wall 188.68 s
```

```
--------------------------------------------------
-  Entering optman on Sun Oct 13 22:50:23 2024  -
--------------------------------------------------

 Geometry Optimization Parameters
   NAtoms,    NIC,     NZ,  NCons,   NDum,   NFix, NCnnct, MaxDiis
       38     272       0       0       0       0       0       0

 Cartesian Hessian Update
 Hessian updated using BFGS update


** GEOMETRY OPTIMIZATION IN DELOCALIZED INTERNAL COORDINATES **
   Searching for a Minimum

   Optimization Cycle:  11

                      Coordinates (Angstroms)
     ATOM                X               Y               Z
      1  N         2.6577120948    0.1458798530   -0.2104998035
      2  C         2.9383932450   -0.7772609237   -1.1759917515
      3  C         3.9205591454   -1.7210670348   -0.9121916234
      4  C         4.6127268554   -1.7135086687    0.2888150457
      5  C         4.3145965434   -0.7462538905    1.2451380649
      6  C         3.3335801710    0.1711016398    0.9676057573
      7  C         2.2225213144   -0.7256351087   -2.4877235995
      8  C         1.6171674064    1.1672404014   -0.4405798422
      9  N        -1.3867177568   -0.8237102834    0.6643567867
     10  C        -2.4025439766   -0.1985958973    0.0128564468
     11  N        -2.1635314520    0.8424388010   -0.7403452625
     12  C        -0.9119847741    1.3121540183   -0.8729413528
     13  C         0.2065819790    0.6479431298   -0.2574316878
     14  C        -0.1007580920   -0.4167961687    0.5277912599
     15  C        -3.7967553510   -0.6905769335    0.1982499710
     16  C        -4.6332641784    0.3042312455    1.0305064313
     17  C        -4.0831010612    0.5257869575    2.4344702181
     18  N        -0.7868402245    2.4212511079   -1.5937943996
     19  H         4.1489044079   -2.4563016433   -1.6733725366
     20  H         5.3856458444   -2.4501268406    0.4764849212
     21  H         4.8402647108   -0.6965073893    2.1897707544
     22  H         3.0581443206    0.9532731873    1.6635954156
     23  H         2.4748925941   -1.6042674505   -3.0784869965
     24  H         2.5365671821    0.1523299738   -3.0623018802
     25  H         1.1373684041   -0.6927757113   -2.3707020110
     26  H         1.7587212807    1.5495909733   -1.4525241854
     27  H         1.8116838611    1.9963402083    0.2431196586
     28  H        -1.6048180225   -1.6106344716    1.2635049531
     29  H         0.6396254223   -0.9961003321    1.0662872274
     30  H        -4.2382237272   -0.8023856576   -0.7952387041
     31  H        -3.7895682637   -1.6747234552    0.6763059643
     32  H        -5.6468471846   -0.0980673994    1.0824181045
     33  H        -4.6961429308    1.2499083946    0.4865226864
     34  H        -4.0192353317   -0.4126932362    2.9939699857
     35  H        -4.7338333806    1.1953414457    2.9981174794
     36  H        -3.0887350971    0.9874103489    2.4180867653
     37  H         0.0869627136    2.8867878391   -1.7756087681
     38  H        -1.6197186914    2.7989789716   -2.0282394928
   Point Group: c1    Number of degrees of freedom:   108


   Energy is   -763.970389983

 Hessian updated using BFGS update
  internal optimization (0)

108 Hessian modes will be used to form the next step
  Hessian Eigenvalues:
     0.001334    0.002688    0.003080    0.004238    0.008248    0.012046
     0.013849    0.019035    0.019245    0.019947    0.020322    0.022544
     0.022798    0.023100    0.023996    0.024896    0.025679    0.026899
```

| | | | | | |
|---|---|---|---|---|---|
| 0.028192 | 0.028898 | 0.029072 | 0.030463 | 0.031457 | 0.036346 |
| 0.038776 | 0.041186 | 0.042871 | 0.043602 | 0.044345 | 0.045296 |
| 0.050419 | 0.052932 | 0.054146 | 0.056027 | 0.060992 | 0.079944 |
| 0.085591 | 0.093501 | 0.121686 | 0.122342 | 0.127133 | 0.130770 |
| 0.131942 | 0.132996 | 0.136869 | 0.141370 | 0.143696 | 0.145312 |
| 0.146232 | 0.147701 | 0.149999 | 0.152520 | 0.153027 | 0.153961 |
| 0.157493 | 0.161201 | 0.193039 | 0.206402 | 0.207273 | 0.217163 |
| 0.226516 | 0.228761 | 0.239599 | 0.248048 | 0.252347 | 0.261586 |
| 0.268872 | 0.271866 | 0.281518 | 0.295038 | 0.300088 | 0.300446 |
| 0.300668 | 0.301215 | 0.301727 | 0.302517 | 0.304026 | 0.304116 |
| 0.305048 | 0.305368 | 0.307589 | 0.308641 | 0.311955 | 0.315585 |
| 0.320184 | 0.332138 | 0.333357 | 0.335151 | 0.337125 | 0.338091 |
| 0.342700 | 0.350716 | 0.356058 | 0.363503 | 0.381696 | 0.390673 |
| 0.398915 | 0.407336 | 0.417533 | 0.418334 | 0.421539 | 0.437589 |
| 0.445966 | 0.450686 | 0.481030 | 0.504476 | 0.567415 | 0.691314 |

Minimum search - taking simple RFO step
Searching for Lamda that Minimizes Along All modes
Value Taken    Lamda =  -0.00052642
Calculated Step too Large.  Step scaled by  0.718814
Step Taken.  Stepsize is  0.300000

| | Maximum | Tolerance | Cnvgd? |
|---|---|---|---|
| Gradient | 0.003132 | 0.000800 | NO |
| Displacement | 0.132798 | 0.001400 | NO |
| Energy change | -0.000328 | 0.000228 | NO |

New Cartesian Coordinates Obtained by Inverse Iteration

Displacement from previous Coordinates is:  1.117500

Standard Nuclear Orientation (Angstroms)

| I | Atom | X | Y | Z |
|---|---|---|---|---|
| 1 | N | 2.6412737846 | 0.1445740519 | -0.2378706378 |
| 2 | C | 2.9618574008 | -0.7951517517 | -1.1738176323 |
| 3 | C | 3.9458502628 | -1.7218016572 | -0.8591308596 |
| 4 | C | 4.5995423347 | -1.6811854311 | 0.3622272079 |
| 5 | C | 4.2582928078 | -0.6991716775 | 1.2894468944 |
| 6 | C | 3.2764118076 | 0.1998618829 | 0.9619667100 |
| 7 | C | 2.2878269496 | -0.7832875643 | -2.5083012331 |
| 8 | C | 1.5966467098 | 1.1473896727 | -0.5190632568 |
| 9 | N | -1.4038234954 | -0.8579551977 | 0.5747072311 |
| 10 | C | -2.4261955450 | -0.1796297793 | -0.0081990560 |
| 11 | N | -2.1897118551 | 0.8953162957 | -0.7136810475 |
| 12 | C | -0.9336623456 | 1.3444525364 | -0.8631060632 |
| 13 | C | 0.1891118246 | 0.6326707977 | -0.3121366242 |
| 14 | C | -0.1135814532 | -0.4679568292 | 0.4225699418 |
| 15 | C | -3.8236332070 | -0.6538509943 | 0.1975745654 |
| 16 | C | -4.6205380249 | 0.3253787830 | 1.0847453119 |
| 17 | C | -4.0250522867 | 0.4915783848 | 2.4780667943 |
| 18 | N | -0.8021337851 | 2.4798504105 | -1.5413072297 |
| 19 | H | 4.2049234067 | -2.4704947794 | -1.5968588816 |
| 20 | H | 5.3744875466 | -2.4042479160 | 0.5895336703 |
| 21 | H | 4.7517911694 | -0.6239111761 | 2.2495530721 |
| 22 | H | 2.9674777192 | 0.9910189076 | 1.6333076902 |
| 23 | H | 2.5482773425 | -1.6861269055 | -3.0578812417 |
| 24 | H | 2.6352302454 | 0.0679344067 | -3.1034573220 |
| 25 | H | 1.1998766680 | -0.7310397347 | -2.4304839174 |
| 26 | H | 1.7313043381 | 1.4808105586 | -1.5496447925 |
| 27 | H | 1.7851353869 | 2.0101842009 | 0.1241917993 |
| 28 | H | -1.6200115712 | -1.6734966727 | 1.1348211959 |
| 29 | H | 0.6302096439 | -1.0883395585 | 0.9077240704 |
| 30 | H | -4.2927308952 | -0.7263701983 | -0.7870878676 |
| 31 | H | -3.8208416389 | -1.6537496091 | 0.6418690613 |
| 32 | H | -5.6386421229 | -0.0626676476 | 1.1531786378 |
| 33 | H | -4.6831825227 | 1.2894589859 | 0.5742481431 |
| 34 | H | -3.9566226843 | -0.4663161136 | 3.0029406087 |
| 35 | H | -4.6479835720 | 1.1501001662 | 3.0845093234 |

```
  36      H      -3.0250227786     0.9401750502     2.4456469203
  37      H       0.0755123999     2.9412974339    -1.7160947585
  38      H      -1.6376699651     2.8946986683    -1.9347064282
 ----------------------------------------------------------------
 Nuclear Repulsion Energy =        1311.69021194 hartrees
 There are       65 alpha and       65 beta electrons

--------------------------------------------------
-  Entering fldman on Sun Oct 13 22:50:23 2024  -
--------------------------------------------------

 Applying Cartesian multipole field
    Component          Value
    ---------          -----
     (2,0,0)        1.00000E-12
     (0,2,0)        2.00000E-11
     (0,0,2)       -3.00000E-11
 Nucleus-field energy     =    -0.0000000061 hartrees

--------------------------------------------------
-  Entering gesman on Sun Oct 13 22:50:23 2024  -
--------------------------------------------------

 Requested basis set is 6-311+G(d,p)
 There are 188 shells and 516 basis functions
 A cutoff of  1.0D-12 yielded  12806 shell pairs
 There are    101636 function pairs (    108325 Cartesian)
 Smallest overlap matrix eigenvalue = 2.23E-06
 Linear dependence detected in AO basis
 Tighter screening thresholds may be required for diffuse basis sets
 Use S2THRESH > 12 and THRESH = 14 in case of SCF convergence issues
 Number of orthogonalized atomic orbitals = 510
 Maximum deviation from orthogonality = 1.477E-11
 Guess MOs from SCF MO coefficient file
 Reading MOs from coefficient file
 Reading MOs from coefficient file

--------------------------------------------------
-  Entering scfman on Sun Oct 13 22:50:23 2024  -
--------------------------------------------------

 Long-range K will be added via erf
 Coulomb attenuation parameter = 0.2 bohr**(-1)
 A restricted hybrid HF-DFT SCF calculation will be
 performed using Pulay DIIS + Geometric Direct Minimization
 Exchange:     0.2220 Hartree-Fock + 1.0000 wB97X-D + LR-HF
 Correlation:  1.0000 wB97X-D
 Using Euler-Maclaurin-Lebedev (75,302) quadrature formula
 Dispersion:   Grimme D
 SCF converges when RMS gradient is below 1.0E-07
 Geometry optimization detected.  Setting ReadMinima to 0
 Setting SaveMinima to 0
 ---------------------------------------
  Cycle       Energy         DIIS Error
 ---------------------------------------
    1    -763.9616698117      9.97E-04
    2    -763.9696255924      1.21E-04
    3    -763.9704542075      8.51E-05
    4    -763.9706534643      3.67E-05
    5    -763.9707049788      8.52E-06
    6    -763.9707084531      3.27E-06
    7    -763.9707089375      1.05E-06
    8    -763.9707090133      4.74E-07
    9    -763.9707090254      1.52E-07
   10    -763.9707090272      6.45E-08 Convergence criterion met
 ---------------------------------------
 SCF time:  CPU 244.45 s  wall 427.86 s
 SCF   energy in the final basis set = -763.97070903
 Total energy in the final basis set = -763.97070903
```

```
---------------------------------------------
-  Entering anlman on Sun Oct 13 22:57:31 2024  -
---------------------------------------------



 --------------------------------------------------------------

                  Orbital Energies (a.u.)
 --------------------------------------------------------------

 Alpha MOs
 -- Occupied --
-14.8212 -14.7875 -14.7366 -14.7040 -10.6747 -10.6589 -10.6451 -10.6438
-10.6389 -10.6308 -10.6067 -10.5884 -10.5766 -10.5755 -10.5526 -10.5241
-10.4892 -10.4656  -1.4148  -1.4090  -1.3457  -1.2735  -1.2332  -1.2003
 -1.1926  -1.1421  -1.1021  -1.0873  -1.0544  -1.0498  -1.0373  -1.0042
 -0.9924  -0.9539  -0.9451  -0.9151  -0.9054  -0.8905  -0.8873  -0.8619
 -0.8555  -0.8475  -0.8373  -0.8256  -0.8081  -0.8016  -0.7919  -0.7877
 -0.7715  -0.7562  -0.7535  -0.7519  -0.7437  -0.7331  -0.7212  -0.7168
 -0.6956  -0.6657  -0.6621  -0.6413  -0.6294  -0.6258  -0.6228  -0.6215
 -0.6079
 -- Virtual --
 -0.2744  -0.2566  -0.2376  -0.2285  -0.1750  -0.1656  -0.1512  -0.1451
 -0.1360  -0.1180  -0.1156  -0.1135  -0.1106  -0.1049  -0.1020  -0.0962
 -0.0916  -0.0857  -0.0835  -0.0805  -0.0758  -0.0712  -0.0704  -0.0694
 -0.0624  -0.0595  -0.0554  -0.0543  -0.0503  -0.0479  -0.0455  -0.0350
 -0.0310  -0.0279  -0.0258  -0.0231  -0.0198  -0.0132  -0.0109  -0.0054
 -0.0041  -0.0027   0.0002   0.0016   0.0045   0.0107   0.0128   0.0157
  0.0194   0.0220   0.0265   0.0284   0.0325   0.0359   0.0387   0.0410
  0.0433   0.0448   0.0499   0.0510   0.0576   0.0606   0.0622   0.0641
  0.0673   0.0711   0.0724   0.0763   0.0793   0.0848   0.0883   0.0922
  0.0966   0.0992   0.1051   0.1083   0.1098   0.1136   0.1151   0.1189
  0.1228   0.1270   0.1304   0.1339   0.1368   0.1431   0.1470   0.1510
  0.1549   0.1580   0.1623   0.1632   0.1684   0.1730   0.1752   0.1804
  0.1830   0.1840   0.1902   0.1923   0.2004   0.2130   0.2174   0.2269
  0.2370   0.2426   0.2549   0.2781   0.2925   0.2961   0.3144   0.3149
  0.3230   0.3364   0.3399   0.3428   0.3494   0.3581   0.3616   0.3690
  0.3780   0.3800   0.3849   0.3890   0.3946   0.3994   0.4102   0.4191
  0.4224   0.4309   0.4353   0.4505   0.4621   0.4622   0.4685   0.4791
  0.4824   0.4872   0.4949   0.5046   0.5118   0.5137   0.5164   0.5210
  0.5254   0.5298   0.5372   0.5410   0.5479   0.5522   0.5529   0.5580
  0.5646   0.5670   0.5764   0.5820   0.5859   0.5920   0.6014   0.6039
  0.6116   0.6215   0.6229   0.6306   0.6356   0.6465   0.6527   0.6595
  0.6631   0.6689   0.6771   0.6810   0.6899   0.6962   0.6993   0.7012
  0.7252   0.7429   0.7442   0.7500   0.7637   0.7663   0.7721   0.7790
  0.7969   0.7994   0.8084   0.8174   0.8238   0.8391   0.8497   0.8610
  0.8728   0.8833   0.8960   0.9002   0.9109   0.9177   0.9390   0.9527
  0.9630   0.9670   0.9839   0.9906   0.9952   1.0201   1.0252   1.0395
  1.0486   1.0612   1.0963   1.1023   1.1231   1.1421   1.1656   1.1891
  1.1946   1.2040   1.2242   1.2353   1.2523   1.2728   1.2879   1.2949
  1.3056   1.3122   1.3338   1.3380   1.3533   1.3616   1.3693   1.3792
  1.3816   1.3918   1.3998   1.4055   1.4114   1.4240   1.4289   1.4329
  1.4372   1.4452   1.4480   1.4606   1.4665   1.4702   1.4840   1.4950
  1.5098   1.5181   1.5200   1.5314   1.5379   1.5426   1.5477   1.5549
  1.5590   1.5665   1.5771   1.5801   1.5939   1.5997   1.6065   1.6144
  1.6213   1.6274   1.6470   1.6584   1.6655   1.6757   1.6758   1.6810
  1.6991   1.7031   1.7174   1.7292   1.7362   1.7515   1.7599   1.7796
  1.7813   1.7946   1.8030   1.8143   1.8352   1.8398   1.8641   1.8852
  1.8900   1.9053   1.9072   1.9384   1.9641   1.9688   1.9906   2.0064
  2.0117   2.0217   2.0308   2.0395   2.0467   2.0612   2.0876   2.0947
  2.1124   2.1192   2.1357   2.1588   2.1717   2.1897   2.2114   2.2287
  2.2360   2.2398   2.2501   2.2585   2.2656   2.2690   2.2861   2.2908
  2.3062   2.3116   2.3355   2.3397   2.3672   2.3712   2.3961   2.4058
  2.4068   2.4183   2.4393   2.4457   2.4593   2.4666   2.4735   2.4784
  2.5065   2.5126   2.5193   2.5304   2.5386   2.5396   2.5473   2.5604
  2.5716   2.5771   2.5860   2.5895   2.5974   2.6003   2.6128   2.6238
  2.6295   2.6429   2.6573   2.6711   2.6819   2.6888   2.6955   2.7137
  2.7193   2.7271   2.7389   2.7415   2.7625   2.7738   2.7832   2.7872
  2.7977   2.8124   2.8207   2.8484   2.8512   2.8695   2.8834   2.8950
  2.8992   2.9114   2.9487   3.0044   3.0483   3.0629   3.1066   3.1469
```

```
 3.1742   3.2303   3.2583   3.2645   3.2977   3.3255   3.3716   3.4086
 3.4252   3.4788   3.5314   3.5344   3.5631   3.5915   3.6189   3.6472
 3.6808   3.6964   3.7194   3.7341   3.7366   3.7424   3.7490   3.7539
 3.7696   3.8408   3.8631   3.8986   3.9422   3.9842   4.0326   4.0738
 4.0948   4.2116   4.2883   4.3232   4.5949   4.6707   4.6833   4.7890
 4.9269   5.0289   5.2071  23.5306  23.5868  23.7232  23.7286  23.7385
23.7752  23.8017  23.8283  23.8612  23.8925  23.9203  23.9602  23.9852
24.0276  35.4113  35.4632  35.5270  35.6295
--------------------------------------------------------------

         Ground-State Mulliken Net Atomic Charges

    Atom                 Charge (a.u.)
 ----------------------------------------
     1 N                     0.337149
     2 C                     0.137709
     3 C                    -0.050897
     4 C                    -0.195950
     5 C                    -0.290723
     6 C                     0.016873
     7 C                    -0.800447
     8 C                    -0.162649
     9 N                    -0.134125
    10 C                    -0.358557
    11 N                     0.047322
    12 C                     0.251636
    13 C                    -0.022390
    14 C                     0.019586
    15 C                    -0.318964
    16 C                    -0.287525
    17 C                    -0.497907
    18 N                    -0.470275
    19 H                     0.222901
    20 H                     0.237721
    21 H                     0.234001
    22 H                     0.222886
    23 H                     0.228661
    24 H                     0.258256
    25 H                     0.216492
    26 H                     0.206990
    27 H                     0.254986
    28 H                     0.381054
    29 H                     0.209147
    30 H                     0.256858
    31 H                     0.193413
    32 H                     0.220176
    33 H                     0.209699
    34 H                     0.175304
    35 H                     0.213437
    36 H                     0.141990
    37 H                     0.317531
    38 H                     0.378631
 ----------------------------------------
 Sum of atomic charges =     2.000000

-----------------------------------------------------------------
                   Cartesian Multipole Moments
-----------------------------------------------------------------
   Charge (ESU x 10^10)
                9.6064
   Dipole Moment (Debye)
        X       8.1648      Y      -0.7314      Z      -1.2914
      Tot       8.2986
   Quadrupole Moments (Debye-Ang)
       XX      -6.4924     XY     -12.9684     YY     -70.8084
       XZ      -5.7662     YZ      -8.4718     ZZ     -77.4035
   Traceless Quadrupole Moments (Debye-Ang)
      QXX     135.2271    QYY     -57.7210    QZZ     -77.5061
      QXY     -38.9051    QXZ     -17.2985    QYZ     -25.4155
   Octopole Moments (Debye-Ang^2)
```

```
      XXX      63.4425    XXY     -93.2441    XYY       9.3335
      YYY      24.0677    XXZ      58.5216    XYZ      16.3989
      YYZ     -26.0470    XZZ      11.1682    YZZ       6.1348
      ZZZ      -1.9812
   Traceless Octopole Moments (Debye-Ang^2)
      XXX     196.1389    YYY     928.3900    ZZZ    -304.1581
      XXY   -1209.5366    XXZ     786.3435    XYY    -111.8298
      XYZ     245.9831    XZZ     -84.3091    YYZ    -482.1853
      YZZ     281.1467
   Hexadecapole Moments (Debye-Ang^3)
     XXXX   -3807.1972   XXXY     -42.1138   XXYY    -810.8370
     XYYY     291.3647   YYYY    -670.9593   XXXZ     366.9254
     XXYZ      28.8851   XYYZ     104.5712   YYYZ     -52.4713
     XXZZ    -851.5702   XYZZ      73.1734   YYZZ    -223.2037
     XZZZ     299.0738   YZZZ      35.2058   ZZZZ    -950.6706
   Traceless Hexadecapole Moments (Debye-Ang^3)
     XXXX    9708.2484   XXXY  -18931.0391   XXXZ    3851.4951
     XXYY   -5118.9677   XXYZ    2861.7275   XXZZ   -4589.2807
     XYYY   16084.1979   XYYZ    -578.5752   XYZZ    2846.8412
     XZZZ   -3272.9199   YYYY     198.8325   YYYZ   -6023.1088
     YYZZ    4920.1352   YZZZ    3161.3813   ZZZZ    -330.8546
 -----------------------------------------------------------------

--------------------------------------------------
-  Entering drvman on Sun Oct 13 22:57:31 2024  -
--------------------------------------------------

 Calculating analytic gradient of the SCF energy
 Gradient of SCF Energy
           1           2           3           4           5           6
    1  -0.0002592  -0.0001234  -0.0003389  -0.0000382   0.0001533   0.0003296
    2   0.0000497   0.0005154  -0.0002217   0.0001168  -0.0002667   0.0002047
    3   0.0004708  -0.0000954  -0.0001864   0.0000626  -0.0002253  -0.0000706
           7           8           9          10          11          12
    1  -0.0003861   0.0000953  -0.0002097   0.0009959  -0.0001537   0.0006282
    2  -0.0001301   0.0005594   0.0005804  -0.0004839   0.0007662  -0.0030217
    3   0.0002314  -0.0001474  -0.0003119   0.0008252  -0.0002609   0.0003911
          13          14          15          16          17          18
    1  -0.0006719  -0.0000720  -0.0001054   0.0001202  -0.0000614  -0.0002175
    2  -0.0007048   0.0001998   0.0002933  -0.0001088  -0.0000724   0.0022582
    3  -0.0008210   0.0003810   0.0002379  -0.0002816   0.0000405   0.0013410
          19          20          21          22          23          24
    1   0.0000491   0.0000418   0.0001345   0.0000928   0.0003512   0.0002153
    2  -0.0001388  -0.0000250  -0.0000721  -0.0000322   0.0000584  -0.0001495
    3   0.0000224   0.0000389   0.0000086   0.0001662  -0.0001296   0.0000027
          25          26          27          28          29          30
    1  -0.0001681  -0.0000084  -0.0002887  -0.0001183  -0.0000599  -0.0001647
    2   0.0003786  -0.0000301   0.0001367  -0.0001396  -0.0001300  -0.0001000
    3   0.0000827  -0.0001177   0.0001295   0.0000471   0.0000348  -0.0000529
          31          32          33          34          35          36
    1  -0.0000086  -0.0000647   0.0000259   0.0000313  -0.0000505   0.0000052
    2  -0.0000993   0.0000197   0.0000386   0.0000736   0.0000770  -0.0000121
    3  -0.0001282   0.0000950   0.0000259  -0.0000202  -0.0000587  -0.0001243
          37          38
    1   0.0006914  -0.0003919
    2   0.0000426  -0.0004303
    3  -0.0004520  -0.0011511
 Max gradient component =       3.022E-03
 RMS gradient           =       4.827E-04
 Gradient time:  CPU 97.94 s  wall 187.12 s

--------------------------------------------------
-  Entering optman on Sun Oct 13 23:00:39 2024  -
--------------------------------------------------

 Geometry Optimization Parameters
   NAtoms,    NIC,     NZ,  NCons,   NDum,   NFix, NCnnct, MaxDiis
      38     272       0       0       0       0       0       0

 Cartesian Hessian Update
```

```
 Hessian updated using BFGS update


** GEOMETRY OPTIMIZATION IN DELOCALIZED INTERNAL COORDINATES **
   Searching for a Minimum

   Optimization Cycle:  12

                    Coordinates (Angstroms)
     ATOM                X               Y               Z
      1  N         2.6412737846    0.1445740519   -0.2378706378
      2  C         2.9618574008   -0.7951517517   -1.1738176323
      3  C         3.9458502628   -1.7218016572   -0.8591308596
      4  C         4.5995423347   -1.6811854311    0.3622272079
      5  C         4.2582928078   -0.6991716775    1.2894468944
      6  C         3.2764118076    0.1998618829    0.9619667100
      7  C         2.2878269496   -0.7832875643   -2.5083012331
      8  C         1.5966467098    1.1473896727   -0.5190632568
      9  N        -1.4038234954   -0.8579551977    0.5747072311
     10  C        -2.4261955450   -0.1796297793   -0.0081990560
     11  N        -2.1897118551    0.8953162957   -0.7136810475
     12  C        -0.9336623456    1.3444525364   -0.8631060632
     13  C         0.1891118246    0.6326707977   -0.3121366242
     14  C        -0.1135814532   -0.4679568292    0.4225699418
     15  C        -3.8236332070   -0.6538509943    0.1975745654
     16  C        -4.6205380249    0.3253787830    1.0847453119
     17  C        -4.0250522867    0.4915783848    2.4780667943
     18  N        -0.8021337851    2.4798504105   -1.5413072297
     19  H         4.2049234067   -2.4704947794   -1.5968588816
     20  H         5.3744875466   -2.4042479160    0.5895336703
     21  H         4.7517911694   -0.6239111761    2.2495530721
     22  H         2.9674777192    0.9910189076    1.6333076902
     23  H         2.5482773425   -1.6861269055   -3.0578812417
     24  H         2.6352302454    0.0679344067   -3.1034573220
     25  H         1.1998766680   -0.7310397347   -2.4304839174
     26  H         1.7313043381    1.4808105586   -1.5496447925
     27  H         1.7851353869    2.0101842009    0.1241917993
     28  H        -1.6200115712   -1.6734966727    1.1348211959
     29  H         0.6302096439   -1.0883395585    0.9077240704
     30  H        -4.2927308952   -0.7263701983   -0.7870878676
     31  H        -3.8208416389   -1.6537496091    0.6418690613
     32  H        -5.6386421229   -0.0626676476    1.1531786378
     33  H        -4.6831825227    1.2894589859    0.5742481431
     34  H        -3.9566226843   -0.4663161136    3.0029406087
     35  H        -4.6479835720    1.1501001662    3.0845093234
     36  H        -3.0250227786    0.9401750502    2.4456469203
     37  H         0.0755123999    2.9412974339   -1.7160947585
     38  H        -1.6376699651    2.8946986683   -1.9347064282
   Point Group: c1    Number of degrees of freedom:   108


   Energy is   -763.970709027

 Hessian updated using BFGS update
  internal optimization (0)

108 Hessian modes will be used to form the next step
  Hessian Eigenvalues:
     0.000860    0.002687    0.002798    0.004237    0.007292    0.012020
     0.014261    0.019055    0.019274    0.019955    0.020273    0.022572
     0.022797    0.023304    0.023989    0.025029    0.025713    0.027000
     0.028030    0.028415    0.029027    0.030465    0.031446    0.036391
     0.038750    0.040853    0.042863    0.043590    0.044424    0.045047
     0.049089    0.051913    0.054338    0.056131    0.060260    0.079902
     0.085596    0.093955    0.121689    0.122368    0.127136    0.130676
     0.131923    0.133018    0.136871    0.141301    0.143680    0.145272
     0.146113    0.147702    0.150309    0.152548    0.153020    0.153766
     0.157490    0.160887    0.193089    0.206470    0.207016    0.217049
     0.226661    0.229476    0.239942    0.249246    0.252425    0.261912
     0.268832    0.270024    0.281523    0.293120    0.300065    0.300251
```

```
    0.300638    0.301204    0.301726    0.302391    0.303883    0.304115
    0.305047    0.305365    0.307580    0.308815    0.311699    0.314051
    0.320058    0.332189    0.333209    0.335125    0.337102    0.338119
    0.342651    0.351846    0.355299    0.363758    0.385539    0.391185
    0.400434    0.407085    0.417189    0.418335    0.420782    0.437214
    0.447502    0.452088    0.478015    0.494451    0.567270    0.682330

 Minimum search - taking simple RFO step
 Searching for Lamda that Minimizes Along All modes
 Value Taken    Lamda =  -0.00068622
 Calculated Step too Large.  Step scaled by  0.534089
 Step Taken.  Stepsize is  0.300000

                         Maximum     Tolerance    Cnvgd?
        Gradient        0.003534      0.000800      NO
        Displacement    0.134514      0.001400      NO
        Energy change  -0.000319      0.000228      NO


 New Cartesian Coordinates Obtained by Inverse Iteration

 Displacement from previous Coordinates is:  1.228604
 ----------------------------------------------------------------
             Standard Nuclear Orientation (Angstroms)
    I     Atom           X                Y                Z
 ----------------------------------------------------------------
    1      N       2.6226639038     0.1395203385    -0.2708460336
    2      C       2.9904142277    -0.8154094770    -1.1728916556
    3      C       3.9793918082    -1.7168720109    -0.8025132925
    4      C       4.5878974537    -1.6388530018     0.4400511709
    5      C       4.1938631455    -0.6454161497     1.3341426578
    6      C       3.2103276602     0.2286135377     0.9512731119
    7      C       2.3608675267    -0.8510490130    -2.5284997137
    8      C       1.5747015049     1.1206644291    -0.6055609893
    9      N      -1.4302373272    -0.8862392402     0.4784325033
   10      C      -2.4537739137    -0.1535449339    -0.0310104842
   11      N      -2.2141059308     0.9526050941    -0.6854037219
   12      C      -0.9529267822     1.3773396995    -0.8549299484
   13      C       0.1693009544     0.6158233464    -0.3727154383
   14      C      -0.1353075481    -0.5169484954     0.3087524295
   15      C      -3.8546244290    -0.6062257994     0.1976177232
   16      C      -4.6037607331     0.3510592505     1.1476157583
   17      C      -3.9638542866     0.4454157387     2.5280680576
   18      N      -0.8104283891     2.5349007756    -1.4905198539
   19      H       4.2762580446    -2.4767889978    -1.5139947419
   20      H       5.3666722273    -2.3426351203     0.7103041072
   21      H       4.6487569320    -0.5418143219     2.3104435598
   22      H       2.8623235070     1.0268697342     1.5945327034
   23      H       2.6503392735    -1.7667197875    -3.0409897348
   24      H       2.7185210065    -0.0141921541    -3.1375500279
   25      H       1.2703176413    -0.8101451558    -2.4899047473
   26      H       1.7037889675     1.4053918439    -1.6517307903
   27      H       1.7590721707     2.0152604540    -0.0051650686
   28      H      -1.6488628877    -1.7278084236     0.9973221092
   29      H       0.6071224624    -1.1790366538     0.7372034382
   30      H      -4.3536916744    -0.6291926782    -0.7747250528
   31      H      -3.8603268463    -1.6235244486     0.6004375551
   32      H      -5.6277765774    -0.0181286488     1.2301287831
   33      H      -4.6596830849     1.3370568696     0.6801919054
   34      H      -3.8982427938    -0.5357970709     3.0083588263
   35      H      -4.5546928713     1.0880862634     3.1817952453
   36      H      -2.9563813435     0.8761430538     2.4837433074
   37      H       0.0721838684     2.9879017635    -1.6610356668
   38      H      -1.6461068673     2.9936893902    -1.8304279911
 ----------------------------------------------------------------
 Nuclear Repulsion Energy =        1310.94416374 hartrees
 There are       65 alpha and       65 beta electrons

 --------------------------------------------------
 -  Entering fldman on Sun Oct 13 23:00:39 2024  -
```

```
--------------------------------------------------

 Applying Cartesian multipole field
    Component          Value
    ---------          -----
     (2,0,0)        1.00000E-12
     (0,2,0)        2.00000E-11
     (0,0,2)       -3.00000E-11
 Nucleus-field energy     =    -0.0000000062 hartrees

--------------------------------------------------
-  Entering gesman on Sun Oct 13 23:00:39 2024  -
--------------------------------------------------

 Requested basis set is 6-311+G(d,p)
 There are 188 shells and 516 basis functions
 A cutoff of  1.0D-12 yielded  12786 shell pairs
 There are    101482 function pairs (    108159 Cartesian)
 Smallest overlap matrix eigenvalue = 2.26E-06
 Linear dependence detected in AO basis
 Tighter screening thresholds may be required for diffuse basis sets
 Use S2THRESH > 12 and THRESH = 14 in case of SCF convergence issues
 Number of orthogonalized atomic orbitals = 510
 Maximum deviation from orthogonality = 1.405E-11
 Guess MOs from SCF MO coefficient file
 Reading MOs from coefficient file
 Reading MOs from coefficient file

--------------------------------------------------
-  Entering scfman on Sun Oct 13 23:00:39 2024  -
--------------------------------------------------

 Long-range K will be added via erf
 Coulomb attenuation parameter = 0.2 bohr**(-1)
 A restricted hybrid HF-DFT SCF calculation will be
 performed using Pulay DIIS + Geometric Direct Minimization
 Exchange:     0.2220 Hartree-Fock + 1.0000 wB97X-D + LR-HF
 Correlation:  1.0000 wB97X-D
 Using Euler-Maclaurin-Lebedev (75,302) quadrature formula
 Dispersion:   Grimme D
 SCF converges when RMS gradient is below 1.0E-07
 Geometry optimization detected.  Setting ReadMinima to 0
 Setting SaveMinima to 0
 ---------------------------------------
  Cycle       Energy         DIIS Error
 ---------------------------------------
    1    -763.9645858612      1.09E-03
    2    -763.9697381087      1.35E-04
    3    -763.9706781709      1.01E-04
    4    -763.9709685825      3.91E-05
    5    -763.9710286247      9.55E-06
    6    -763.9710328918      3.76E-06
    7    -763.9710335100      1.18E-06
    8    -763.9710336014      5.34E-07
    9    -763.9710336173      1.54E-07
   10    -763.9710336192      6.81E-08 Convergence criterion met
 ---------------------------------------
 SCF time:  CPU 244.53 s  wall 430.24 s
 SCF   energy in the final basis set = -763.97103362
 Total energy in the final basis set = -763.97103362

--------------------------------------------------
-  Entering anlman on Sun Oct 13 23:07:49 2024  -
--------------------------------------------------


 --------------------------------------------------------------

                   Orbital Energies (a.u.)
 --------------------------------------------------------------
```

```
 Alpha MOs
 -- Occupied --
-14.8214 -14.7871 -14.7365 -14.7036 -10.6743 -10.6587 -10.6448 -10.6441
-10.6390 -10.6307 -10.6066 -10.5886 -10.5767 -10.5756 -10.5524 -10.5239
-10.4890 -10.4656  -1.4148  -1.4091  -1.3458  -1.2736  -1.2334  -1.2003
 -1.1927  -1.1423  -1.1022  -1.0872  -1.0546  -1.0496  -1.0374  -1.0041
 -0.9925  -0.9539  -0.9450  -0.9152  -0.9050  -0.8905  -0.8878  -0.8619
 -0.8549  -0.8477  -0.8381  -0.8256  -0.8073  -0.8025  -0.7923  -0.7876
 -0.7715  -0.7558  -0.7538  -0.7515  -0.7434  -0.7332  -0.7211  -0.7167
 -0.6954  -0.6652  -0.6626  -0.6411  -0.6292  -0.6259  -0.6227  -0.6215
 -0.6079
 -- Virtual --
 -0.2746  -0.2558  -0.2371  -0.2284  -0.1752  -0.1657  -0.1513  -0.1453
 -0.1364  -0.1179  -0.1155  -0.1127  -0.1101  -0.1053  -0.1012  -0.0959
 -0.0920  -0.0856  -0.0838  -0.0809  -0.0759  -0.0711  -0.0700  -0.0693
 -0.0626  -0.0595  -0.0560  -0.0543  -0.0503  -0.0482  -0.0450  -0.0357
 -0.0310  -0.0284  -0.0258  -0.0223  -0.0201  -0.0129  -0.0116  -0.0060
 -0.0036  -0.0012  -0.0004   0.0014   0.0041   0.0110   0.0129   0.0157
  0.0189   0.0214   0.0262   0.0292   0.0323   0.0356   0.0383   0.0395
  0.0434   0.0449   0.0486   0.0510   0.0578   0.0617   0.0625   0.0644
  0.0678   0.0701   0.0722   0.0762   0.0799   0.0862   0.0879   0.0914
  0.0972   0.1000   0.1040   0.1079   0.1099   0.1137   0.1155   0.1171
  0.1215   0.1276   0.1304   0.1341   0.1358   0.1412   0.1471   0.1504
  0.1586   0.1590   0.1630   0.1650   0.1676   0.1716   0.1743   0.1787
  0.1823   0.1845   0.1864   0.1896   0.2021   0.2115   0.2185   0.2293
  0.2376   0.2435   0.2556   0.2804   0.2861   0.2970   0.3128   0.3158
  0.3208   0.3365   0.3412   0.3423   0.3470   0.3578   0.3640   0.3733
  0.3745   0.3798   0.3848   0.3894   0.3933   0.4002   0.4118   0.4195
  0.4228   0.4315   0.4356   0.4498   0.4608   0.4653   0.4690   0.4778
  0.4852   0.4883   0.4956   0.5053   0.5101   0.5135   0.5180   0.5229
  0.5262   0.5294   0.5351   0.5442   0.5491   0.5496   0.5536   0.5547
  0.5635   0.5679   0.5758   0.5826   0.5848   0.5901   0.6026   0.6052
  0.6093   0.6191   0.6247   0.6330   0.6351   0.6462   0.6512   0.6559
  0.6632   0.6687   0.6769   0.6791   0.6897   0.6967   0.7002   0.7024
  0.7245   0.7428   0.7456   0.7500   0.7661   0.7666   0.7702   0.7825
  0.7975   0.8000   0.8081   0.8204   0.8244   0.8349   0.8501   0.8580
  0.8733   0.8843   0.8938   0.9043   0.9100   0.9198   0.9424   0.9509
  0.9663   0.9688   0.9871   0.9919   0.9978   1.0141   1.0267   1.0299
  1.0504   1.0651   1.0975   1.1021   1.1227   1.1404   1.1694   1.1922
  1.1942   1.2013   1.2238   1.2331   1.2503   1.2679   1.2860   1.2968
  1.3077   1.3141   1.3324   1.3363   1.3551   1.3625   1.3663   1.3804
  1.3829   1.3956   1.3986   1.4037   1.4112   1.4174   1.4280   1.4328
  1.4366   1.4456   1.4489   1.4621   1.4664   1.4708   1.4832   1.4936
  1.5104   1.5152   1.5206   1.5323   1.5380   1.5431   1.5465   1.5570
  1.5608   1.5676   1.5777   1.5812   1.5909   1.5995   1.6071   1.6146
  1.6202   1.6295   1.6474   1.6590   1.6666   1.6747   1.6776   1.6810
  1.6992   1.7088   1.7154   1.7308   1.7382   1.7522   1.7614   1.7791
  1.7818   1.7916   1.8030   1.8124   1.8346   1.8411   1.8654   1.8863
  1.8917   1.9042   1.9075   1.9427   1.9682   1.9711   1.9923   2.0073
  2.0116   2.0212   2.0357   2.0399   2.0480   2.0642   2.0893   2.1010
  2.1119   2.1193   2.1353   2.1556   2.1728   2.1904   2.2116   2.2309
  2.2379   2.2406   2.2522   2.2588   2.2671   2.2709   2.2836   2.2907
  2.3037   2.3104   2.3350   2.3406   2.3672   2.3748   2.3950   2.4069
  2.4075   2.4196   2.4385   2.4484   2.4592   2.4674   2.4753   2.4795
  2.5027   2.5111   2.5186   2.5307   2.5371   2.5394   2.5483   2.5597
  2.5711   2.5779   2.5860   2.5884   2.5988   2.5997   2.6130   2.6227
  2.6323   2.6426   2.6551   2.6704   2.6829   2.6874   2.6977   2.7132
  2.7171   2.7303   2.7381   2.7420   2.7699   2.7732   2.7829   2.7914
  2.8011   2.8126   2.8242   2.8438   2.8527   2.8727   2.8825   2.8921
  2.8982   2.9145   2.9519   3.0037   3.0443   3.0652   3.1096   3.1475
  3.1777   3.2326   3.2602   3.2666   3.2954   3.3264   3.3721   3.4100
  3.4270   3.4806   3.5328   3.5346   3.5654   3.5914   3.6185   3.6467
  3.6786   3.6949   3.7196   3.7326   3.7371   3.7421   3.7489   3.7537
  3.7690   3.8388   3.8634   3.8985   3.9428   3.9834   4.0339   4.0733
  4.0965   4.2118   4.2870   4.3252   4.5964   4.6711   4.6829   4.7970
  4.9279   5.0294   5.2099  23.5334  23.5887  23.7249  23.7288  23.7404
 23.7747  23.8020  23.8270  23.8616  23.8932  23.9202  23.9605  23.9878
 24.0277  35.4123  35.4642  35.5292  35.6317
 --------------------------------------------------------------
```

Ground-State Mulliken Net Atomic Charges

| Atom | Charge (a.u.) |
|---|---|
| 1 N | 0.329973 |
| 2 C | 0.165148 |
| 3 C | -0.068776 |
| 4 C | -0.200479 |
| 5 C | -0.259428 |
| 6 C | -0.035039 |
| 7 C | -0.811303 |
| 8 C | -0.176377 |
| 9 N | -0.131460 |
| 10 C | -0.356514 |
| 11 N | 0.046207 |
| 12 C | 0.260549 |
| 13 C | 0.012297 |
| 14 C | 0.015574 |
| 15 C | -0.320752 |
| 16 C | -0.284163 |
| 17 C | -0.497659 |
| 18 N | -0.475518 |
| 19 H | 0.222713 |
| 20 H | 0.237926 |
| 21 H | 0.233815 |
| 22 H | 0.223310 |
| 23 H | 0.228591 |
| 24 H | 0.259783 |
| 25 H | 0.211995 |
| 26 H | 0.220993 |
| 27 H | 0.251638 |
| 28 H | 0.379126 |
| 29 H | 0.208883 |
| 30 H | 0.257504 |
| 31 H | 0.192480 |
| 32 H | 0.219924 |
| 33 H | 0.209649 |
| 34 H | 0.175030 |
| 35 H | 0.213189 |
| 36 H | 0.141490 |
| 37 H | 0.321202 |
| 38 H | 0.378477 |

Sum of atomic charges = 2.000000

Cartesian Multipole Moments

Charge (ESU x 10^10)

9.6064

Dipole Moment (Debye)

| | | | | | |
|---|---|---|---|---|---|
| X | 8.0727 | Y | -0.7128 | Z | -1.4702 |
| Tot | 8.2364 | | | | |

Quadrupole Moments (Debye-Ang)

| | | | | | |
|---|---|---|---|---|---|
| XX | -6.8087 | XY | -13.0648 | YY | -69.9063 |
| XZ | -5.9336 | YZ | -8.0531 | ZZ | -77.9992 |

Traceless Quadrupole Moments (Debye-Ang)

| | | | | | |
|---|---|---|---|---|---|
| QXX | 134.2881 | QYY | -55.0046 | QZZ | -79.2835 |
| QXY | -39.1943 | QXZ | -17.8009 | QYZ | -24.1592 |

Octopole Moments (Debye-Ang^2)

| | | | | | |
|---|---|---|---|---|---|
| XXX | 62.5203 | XXY | -93.1538 | XYY | 8.1310 |
| YYY | 25.8842 | XXZ | 58.3467 | XYZ | 15.5751 |
| YYZ | -26.1468 | XZZ | 13.6260 | YZZ | 5.0446 |
| ZZZ | 0.3732 | | | | |

Traceless Octopole Moments (Debye-Ang^2)

| | | | | | |
|---|---|---|---|---|---|
| XXX | 179.3081 | YYY | 948.2880 | ZZZ | -287.5599 |
| XXY | -1210.6326 | XXZ | 777.4814 | XYY | -130.8665 |
| XYZ | 233.6262 | XZZ | -48.4416 | YYZ | -489.9215 |
| YZZ | 262.3446 | | | | |

```
    Hexadecapole Moments (Debye-Ang^3)
      XXXX   -3811.3269   XXXY     -32.6164   XXYY    -814.2956
      XYYY     293.1125   YYYY    -664.7308   XXXZ     368.3075
      XXYZ      26.9625   XYYZ     107.4825   YYYZ     -57.7277
      XXZZ    -843.2763   XYZZ      73.2466   YYZZ    -232.5336
      XZZZ     298.2205   YZZZ      41.7447   ZZZZ    -962.2675
    Traceless Hexadecapole Moments (Debye-Ang^3)
      XXXX    9044.7423   XXXY  -18443.1434   XXXZ    3841.8204
      XXYY   -5449.7625   XXYZ    2677.5368   XXZZ   -3594.9797
      XYYY   15758.3895   XYYZ    -324.4966   XYZZ    2684.7539
      XZZZ   -3517.3238   YYYY    1276.8400   YYYZ   -6521.9725
      YYZZ    4172.9225   YZZZ    3844.4357   ZZZZ    -577.9428
 -----------------------------------------------------------------

--------------------------------------------------
-  Entering drvman on Sun Oct 13 23:07:49 2024  -
--------------------------------------------------

 Calculating analytic gradient of the SCF energy
 Gradient of SCF Energy
            1           2           3           4           5           6
    1  -0.0001459  -0.0004653  -0.0001322   0.0001524   0.0000348   0.0004004
    2   0.0000898   0.0008508  -0.0002966  -0.0000500  -0.0001412  -0.0000671
    3   0.0004000  -0.0002502  -0.0001437   0.0000405  -0.0001146  -0.0000154
            7           8           9          10          11          12
    1  -0.0003955   0.0000786  -0.0003819   0.0008574   0.0000701   0.0004323
    2  -0.0001802   0.0004644   0.0007595  -0.0004454   0.0006261  -0.0020633
    3   0.0003224  -0.0001507  -0.0002702   0.0005635  -0.0000676   0.0003174
           13          14          15          16          17          18
    1  -0.0004094   0.0002255   0.0000714  -0.0000638  -0.0000494   0.0000954
    2  -0.0010211   0.0002684   0.0003845  -0.0000848  -0.0000852   0.0010993
    3  -0.0004122   0.0002022   0.0003469  -0.0003767   0.0000271   0.0010068
           19          20          21          22          23          24
    1  -0.0000776  -0.0000196   0.0000667   0.0000888   0.0003774   0.0002038
    2  -0.0000864  -0.0000164  -0.0000104  -0.0000587   0.0000530  -0.0002345
    3   0.0000034   0.0000061  -0.0000300   0.0001831  -0.0000817   0.0000374
           25          26          27          28          29          30
    1  -0.0001109  -0.0003838  -0.0003509  -0.0001590  -0.0001636  -0.0001315
    2   0.0003469   0.0001068   0.0002328  -0.0000732  -0.0001121  -0.0001785
    3  -0.0001313  -0.0002495   0.0001240  -0.0000713  -0.0000233  -0.0000693
           31          32          33          34          35          36
    1   0.0000247  -0.0000511   0.0000409   0.0000489  -0.0000048   0.0000148
    2  -0.0000632   0.0000294   0.0000256   0.0000529   0.0000697  -0.0000020
    3  -0.0001435   0.0000680   0.0000594  -0.0000313   0.0000333  -0.0001245
           37          38
    1   0.0004012  -0.0001892
    2   0.0001933  -0.0003827
    3  -0.0001052  -0.0008791
 Max gradient component =       2.063E-03
 RMS gradient           =       3.690E-04
 Gradient time:  CPU 104.89 s  wall 186.75 s

--------------------------------------------------
-  Entering optman on Sun Oct 13 23:10:56 2024  -
--------------------------------------------------

 Geometry Optimization Parameters
   NAtoms,    NIC,     NZ,  NCons,   NDum,   NFix, NCnnct, MaxDiis
       38     272       0       0       0       0       0       0

 Cartesian Hessian Update
 Hessian updated using BFGS update


** GEOMETRY OPTIMIZATION IN DELOCALIZED INTERNAL COORDINATES **
   Searching for a Minimum

   Optimization Cycle:  13

                    Coordinates (Angstroms)
```

| ATOM | | X | Y | Z |
|---|---|---|---|---|
| 1 | N | 2.6226639038 | 0.1395203385 | -0.2708460336 |
| 2 | C | 2.9904142277 | -0.8154094770 | -1.1728916556 |
| 3 | C | 3.9793918082 | -1.7168720109 | -0.8025132925 |
| 4 | C | 4.5878974537 | -1.6388530018 | 0.4400511709 |
| 5 | C | 4.1938631455 | -0.6454161497 | 1.3341426578 |
| 6 | C | 3.2103276602 | 0.2286135377 | 0.9512731119 |
| 7 | C | 2.3608675267 | -0.8510490130 | -2.5284997137 |
| 8 | C | 1.5747015049 | 1.1206644291 | -0.6055609893 |
| 9 | N | -1.4302373272 | -0.8862392402 | 0.4784325033 |
| 10 | C | -2.4537739137 | -0.1535449339 | -0.0310104842 |
| 11 | N | -2.2141059308 | 0.9526050941 | -0.6854037219 |
| 12 | C | -0.9529267822 | 1.3773396995 | -0.8549299484 |
| 13 | C | 0.1693009544 | 0.6158233464 | -0.3727154383 |
| 14 | C | -0.1353075481 | -0.5169484954 | 0.3087524295 |
| 15 | C | -3.8546244290 | -0.6062257994 | 0.1976177232 |
| 16 | C | -4.6037607331 | 0.3510592505 | 1.1476157583 |
| 17 | C | -3.9638542866 | 0.4454157387 | 2.5280680576 |
| 18 | N | -0.8104283891 | 2.5349007756 | -1.4905198539 |
| 19 | H | 4.2762580446 | -2.4767889978 | -1.5139947419 |
| 20 | H | 5.3666722273 | -2.3426351203 | 0.7103041072 |
| 21 | H | 4.6487569320 | -0.5418143219 | 2.3104435598 |
| 22 | H | 2.8623235070 | 1.0268697342 | 1.5945327034 |
| 23 | H | 2.6503392735 | -1.7667197875 | -3.0409897348 |
| 24 | H | 2.7185210065 | -0.0141921541 | -3.1375500279 |
| 25 | H | 1.2703176413 | -0.8101451558 | -2.4899047473 |
| 26 | H | 1.7037889675 | 1.4053918439 | -1.6517307903 |
| 27 | H | 1.7590721707 | 2.0152604540 | -0.0051650686 |
| 28 | H | -1.6488628877 | -1.7278084236 | 0.9973221092 |
| 29 | H | 0.6071224624 | -1.1790366538 | 0.7372034382 |
| 30 | H | -4.3536916744 | -0.6291926782 | -0.7747250528 |
| 31 | H | -3.8603268463 | -1.6235244486 | 0.6004375551 |
| 32 | H | -5.6277765774 | -0.0181286488 | 1.2301287831 |

***Abridged***Abridged***Abridged***

| | | | | | | | |
|---|---|---|---|---|---|---|---|
| 0.0436 | 0.0454 | 0.0490 | 0.0518 | 0.0602 | 0.0621 | 0.0635 | 0.0645 |
| 0.0697 | 0.0717 | 0.0732 | 0.0775 | 0.0817 | 0.0880 | 0.0894 | 0.0936 |
| 0.0963 | 0.1019 | 0.1053 | 0.1078 | 0.1097 | 0.1131 | 0.1165 | 0.1193 |
| 0.1225 | 0.1293 | 0.1329 | 0.1346 | 0.1371 | 0.1405 | 0.1479 | 0.1511 |
| 0.1564 | 0.1607 | 0.1620 | 0.1649 | 0.1703 | 0.1745 | 0.1762 | 0.1787 |
| 0.1813 | 0.1824 | 0.1892 | 0.1916 | 0.2058 | 0.2103 | 0.2190 | 0.2279 |
| 0.2351 | 0.2435 | 0.2472 | 0.2618 | 0.2693 | 0.2761 | 0.2826 | 0.2996 |
| 0.3035 | 0.3097 | 0.3125 | 0.3259 | 0.3298 | 0.3350 | 0.3410 | 0.3448 |
| 0.3503 | 0.3519 | 0.3604 | 0.3651 | 0.3704 | 0.3749 | 0.3801 | 0.3852 |
| 0.3940 | 0.3971 | 0.4003 | 0.4055 | 0.4151 | 0.4225 | 0.4295 | 0.4321 |
| 0.4371 | 0.4422 | 0.4454 | 0.4482 | 0.4519 | 0.4602 | 0.4654 | 0.4658 |
| 0.4683 | 0.4717 | 0.4760 | 0.4765 | 0.4774 | 0.4879 | 0.4936 | 0.4963 |
| 0.4974 | 0.4993 | 0.5059 | 0.5081 | 0.5144 | 0.5174 | 0.5220 | 0.5272 |
| 0.5295 | 0.5338 | 0.5377 | 0.5421 | 0.5440 | 0.5484 | 0.5521 | 0.5572 |
| 0.5581 | 0.5600 | 0.5694 | 0.5752 | 0.5769 | 0.5851 | 0.5890 | 0.5979 |
| 0.5989 | 0.6019 | 0.6080 | 0.6113 | 0.6202 | 0.6230 | 0.6328 | 0.6380 |
| 0.6445 | 0.6466 | 0.6483 | 0.6551 | 0.6608 | 0.6675 | 0.6705 | 0.6788 |
| 0.6806 | 0.6906 | 0.7033 | 0.7153 | 0.7181 | 0.7220 | 0.7294 | 0.7394 |
| 0.7401 | 0.7445 | 0.7598 | 0.7657 | 0.7723 | 0.7838 | 0.7931 | 0.7942 |
| 0.8051 | 0.8181 | 0.8222 | 0.8281 | 0.8331 | 0.8408 | 0.8417 | 0.8464 |
| 0.8621 | 0.8738 | 0.8764 | 0.8803 | 0.8907 | 0.9021 | 0.9118 | 0.9198 |
| 0.9231 | 0.9257 | 0.9313 | 0.9388 | 0.9445 | 0.9452 | 0.9521 | 0.9644 |
| 0.9667 | 0.9789 | 0.9845 | 0.9912 | 0.9976 | 1.0095 | 1.0181 | 1.0223 |
| 1.0276 | 1.0386 | 1.0454 | 1.0549 | 1.0607 | 1.0631 | 1.0747 | 1.0773 |
| 1.0808 | 1.0828 | 1.0902 | 1.0943 | 1.1021 | 1.1044 | 1.1184 | 1.1270 |
| 1.1314 | 1.1338 | 1.1497 | 1.1506 | 1.1556 | 1.1579 | 1.1643 | 1.1682 |
| 1.1763 | 1.1833 | 1.1861 | 1.1957 | 1.1968 | 1.2067 | 1.2112 | 1.2164 |
| 1.2241 | 1.2286 | 1.2388 | 1.2468 | 1.2513 | 1.2579 | 1.2615 | 1.2660 |
| 1.2729 | 1.2799 | 1.2865 | 1.2873 | 1.2981 | 1.3034 | 1.3110 | 1.3181 |
| 1.3200 | 1.3228 | 1.3344 | 1.3390 | 1.3437 | 1.3517 | 1.3551 | 1.3600 |
| 1.3710 | 1.3767 | 1.3850 | 1.3906 | 1.3975 | 1.4088 | 1.4142 | 1.4232 |
| 1.4279 | 1.4350 | 1.4384 | 1.4500 | 1.4556 | 1.4651 | 1.4776 | 1.4823 |
| 1.4917 | 1.4995 | 1.5026 | 1.5142 | 1.5294 | 1.5378 | 1.5425 | 1.5496 |
| 1.5541 | 1.5565 | 1.5686 | 1.5717 | 1.5900 | 1.6084 | 1.6175 | 1.6356 |

| | | | | | | | |
|---|---|---|---|---|---|---|---|
| 1.6426 | 1.6553 | 1.6754 | 1.6947 | 1.6996 | 1.7317 | 1.7501 | 1.7557 |
| 1.7895 | 1.8011 | 1.8199 | 1.8391 | 1.8517 | 1.8644 | 1.8716 | 1.9160 |
| 1.9415 | 1.9683 | 1.9814 | 2.0164 | 2.0391 | 2.1019 | 2.1290 | 2.1805 |
| 2.2033 | 2.2635 | 2.3245 | 2.3480 | 2.3698 | 2.3960 | 2.4081 | 2.4476 |
| 2.4585 | 2.4626 | 2.4662 | 2.4764 | 2.4825 | 2.4921 | 2.5068 | 2.5226 |
| 2.5249 | 2.5323 | 2.5495 | 2.5546 | 2.5574 | 2.5647 | 2.5758 | 2.5867 |
| 2.5907 | 2.6039 | 2.6077 | 2.6220 | 2.6248 | 2.6359 | 2.6411 | 2.6494 |
| 2.6612 | 2.6645 | 2.6705 | 2.6806 | 2.6872 | 2.6982 | 2.7069 | 2.7133 |
| 2.7152 | 2.7215 | 2.7285 | 2.7347 | 2.7354 | 2.7456 | 2.7513 | 2.7524 |
| 2.7678 | 2.7719 | 2.7843 | 2.8008 | 2.8063 | 2.8168 | 2.8235 | 2.8269 |
| 2.8361 | 2.8446 | 2.8493 | 2.8617 | 2.8645 | 2.8711 | 2.8741 | 2.8794 |
| 2.8905 | 2.8952 | 2.9051 | 2.9077 | 2.9152 | 2.9265 | 2.9370 | 2.9383 |
| 2.9513 | 2.9565 | 2.9633 | 2.9668 | 2.9765 | 2.9907 | 2.9926 | 3.0046 |
| 3.0110 | 3.0197 | 3.0218 | 3.0325 | 3.0408 | 3.0421 | 3.0527 | 3.0564 |
| 3.0587 | 3.0745 | 3.0800 | 3.0851 | 3.0980 | 3.1064 | 3.1119 | 3.1221 |
| 3.1283 | 3.1355 | 3.1390 | 3.1459 | 3.1497 | 3.1561 | 3.1662 | 3.1750 |
| 3.1808 | 3.1965 | 3.2016 | 3.2019 | 3.2067 | 3.2132 | 3.2298 | 3.2353 |
| 3.2380 | 3.2426 | 3.2497 | 3.2569 | 3.2624 | 3.2640 | 3.2722 | 3.2744 |
| 3.2780 | 3.2921 | 3.2958 | 3.2984 | 3.3039 | 3.3121 | 3.3165 | 3.3229 |
| 3.3300 | 3.3424 | 3.3467 | 3.3568 | 3.3696 | 3.3766 | 3.3887 | 3.3968 |
| 3.4052 | 3.4123 | 3.4191 | 3.4282 | 3.4356 | 3.4446 | 3.4504 | 3.4532 |
| 3.4707 | 3.4779 | 3.4893 | 3.4930 | 3.5151 | 3.5247 | 3.5264 | 3.5344 |
| 3.5401 | 3.5471 | 3.5555 | 3.5596 | 3.5639 | 3.5804 | 3.5831 | 3.5901 |
| 3.5922 | 3.6082 | 3.6182 | 3.6250 | 3.6341 | 3.6485 | 3.6490 | 3.6522 |
| 3.6558 | 3.6699 | 3.6801 | 3.6886 | 3.6951 | 3.7150 | 3.7199 | 3.7270 |
| 3.7321 | 3.7390 | 3.7537 | 3.7616 | 3.7709 | 3.7766 | 3.7837 | 3.7919 |
| 3.8021 | 3.8082 | 3.8283 | 3.8346 | 3.8405 | 3.8479 | 3.8530 | 3.8665 |
| 3.8731 | 3.8746 | 3.8886 | 3.9065 | 3.9166 | 3.9288 | 3.9340 | 3.9373 |
| 3.9452 | 3.9540 | 3.9667 | 3.9722 | 3.9771 | 3.9831 | 3.9985 | 4.0039 |
| 4.0087 | 4.0193 | 4.0388 | 4.0477 | 4.0522 | 4.0553 | 4.0910 | 4.0980 |
| 4.1078 | 4.1115 | 4.1182 | 4.1277 | 4.1334 | 4.1367 | 4.1452 | 4.1576 |
| 4.1693 | 4.1751 | 4.1765 | 4.1847 | 4.1969 | 4.1983 | 4.2177 | 4.2208 |
| 4.2298 | 4.2373 | 4.2442 | 4.2603 | 4.2802 | 4.2928 | 4.3116 | 4.3120 |
| 4.3189 | 4.3209 | 4.3269 | 4.3332 | 4.3465 | 4.3578 | 4.3700 | 4.3779 |
| 4.3854 | 4.3891 | 4.4010 | 4.4119 | 4.4191 | 4.4302 | 4.4350 | 4.4459 |
| 4.4549 | 4.4628 | 4.4723 | 4.4849 | 4.4915 | 4.4967 | 4.5152 | 4.5234 |
| 4.5252 | 4.5464 | 4.5613 | 4.5674 | 4.5792 | 4.5874 | 4.6069 | 4.6183 |
| 4.6341 | 4.6431 | 4.6609 | 4.7056 | 4.7123 | 4.7224 | 4.7337 | 4.7555 |
| 4.7702 | 4.8094 | 4.8289 | 4.8472 | 4.8629 | 4.8957 | 4.9043 | 4.9113 |
| 4.9203 | 4.9389 | 4.9481 | 4.9641 | 4.9833 | 4.9916 | 4.9989 | 5.0275 |
| 5.0521 | 5.0673 | 5.0698 | 5.0908 | 5.1057 | 5.1211 | 5.1241 | 5.1902 |
| 5.2035 | 5.2266 | 5.2455 | 5.2590 | 5.2669 | 5.2775 | 5.3051 | 5.3484 |
| 5.3675 | 5.4029 | 5.4186 | 5.4398 | 5.4549 | 5.5138 | 5.5273 | 5.5405 |
| 5.5598 | 5.5741 | 5.5871 | 5.5924 | 5.6205 | 5.6510 | 5.6581 | 5.6714 |
| 5.6917 | 5.7080 | 5.7225 | 5.7404 | 5.7572 | 5.7827 | 5.8022 | 5.8365 |
| 5.8781 | 5.9385 | 5.9544 | 5.9864 | 6.0105 | 6.0812 | 6.0920 | 6.1308 |
| 6.1858 | 6.2606 | 6.2993 | 6.3440 | 6.5334 | 6.5765 | 6.8096 | 23.9745 |
| 24.1381 | 24.1637 | 24.2426 | 24.2581 | 24.2812 | 24.3080 | 24.3456 | 24.4252 |
| 24.6122 | 24.6179 | 24.6291 | 24.8042 | 24.9173 | 35.7632 | 35.7725 | 35.7870 |
| 35.9456 | | | | | | | |

---

Ground-State Mulliken Net Atomic Charges

| Atom | Charge (a.u.) |
|---|---|
| 1 N | 0.359333 |
| 2 C | 0.204567 |
| 3 C | -0.237519 |
| 4 C | -0.157424 |
| 5 C | -0.252209 |
| 6 C | -0.004152 |
| 7 C | -0.460911 |
| 8 C | -0.080539 |
| 9 N | -0.199856 |
| 10 C | 0.045380 |
| 11 N | -0.298833 |
| 12 C | 0.490907 |
| 13 C | -0.037894 |
| 14 C | 0.028765 |

```
    15 C                      -0.259728
    16 C                      -0.092839
    17 C                      -0.419739
    18 N                      -0.379594
    19 H                       0.199301
    20 H                       0.215745
    21 H                       0.204844
    22 H                       0.208346
    23 H                       0.167957
    24 H                       0.188810
    25 H                       0.164592
    26 H                       0.185972
    27 H                       0.185734
    28 H                       0.284196
    29 H                       0.185237
    30 H                       0.180467
    31 H                       0.141297
    32 H                       0.150197
    33 H                       0.144760
    34 H                       0.131504
    35 H                       0.160051
    36 H                       0.100429
    37 H                       0.243750
    38 H                       0.309097
 ----------------------------------------
 Sum of atomic charges =     2.000000

-----------------------------------------------------------------
                    Cartesian Multipole Moments
-----------------------------------------------------------------
   Charge (ESU x 10^10)
                 9.6064
   Dipole Moment (Debye)
         X       7.7693      Y      -0.4812      Z      -1.7661
       Tot       7.9820
   Quadrupole Moments (Debye-Ang)
        XX      -7.1975     XY     -13.6541     YY     -69.2287
        XZ      -5.9219     YZ      -7.8335     ZZ     -78.1421
   Traceless Quadrupole Moments (Debye-Ang)
       QXX     132.9758    QYY     -53.1179    QZZ     -79.8580
       QXY     -40.9622    QXZ     -17.7656    QYZ     -23.5005
   Octopole Moments (Debye-Ang^2)
       XXX      60.7738    XXY     -92.7510    XYY       6.9618
       YYY      28.1820    XXZ      59.2496    XYZ      14.4530
       YYZ     -27.5769    XZZ      15.9120    YZZ       4.5615
       ZZZ       4.9273
   Traceless Octopole Moments (Debye-Ang^2)
       XXX     158.7784    YYY     962.7974    ZZZ    -255.4900
       XXY   -1211.2425    XXZ     778.9439    XYY    -146.5163
       XYZ     216.7947    XZZ     -12.2621    YYZ    -523.4539
       YZZ     248.4450
   Hexadecapole Moments (Debye-Ang^3)
      XXXX   -3797.1074   XXXY     -21.6126   XXYY    -818.4300
      XYYY     292.2547   YYYY    -657.5171   XXXZ     365.5224
      XXYZ      24.2613   XYYZ     111.7473   YYYZ     -57.0686
      XXZZ    -832.5931   XYZZ      72.3947   YYZZ    -246.4851
      XZZZ     291.5842   YZZZ      53.5056   ZZZZ   -1000.2732
   Traceless Hexadecapole Moments (Debye-Ang^3)
      XXXX    8386.2414   XXXY  -17705.9829   XXXZ    3781.4279
      XXYY   -6126.4505   XXYZ    2244.5467   XXZZ   -2259.7909
      XYYY   15250.0922   XYYZ     200.6558   XYZZ    2455.8907
      XZZZ   -3982.0837   YYYY    2730.3712   YYYZ   -6900.8734
      YYZZ    3396.0793   YZZZ    4656.3268   ZZZZ   -1136.2884
-----------------------------------------------------------------
Total job time:  547.92s(wall), 391.19s(cpu)
Mon Oct 14 03:46:23 2024
```

```
Wavefunction Version of Q-Chem

 Parts of Q-Chem use Armadillo 8.300.2 (Tropical Shenanigans).
 http://arma.sourceforge.net/

 Q-Chem begins on Sun Oct 13 19:18:00 2024


     Scratch files written to
C:/Users/hille/AppData/Local/Temp/WFB0CB2A5C99266D7A/Conformer.13//scratch///
Processing default memory
  ... MEM_TOTAL 4076 MB (default) [16 cores]
Processing $rem in C:/Program
Files/Wavefunction/Spartan24v110/P4e//../auxdir/config/preferences:
   (site specific preferences)
  ... THRESH             9
  ... SMALL_PROD_XCMAT   9
  ... BASIS_LIN_DEP_THRESH     5
  ... SCF_ALGORITHM     DIIS_GDM
  ... MAXSCF            250
  ... MAXDIIS          45
  ... THRESHDIIS         -1  (i.e. don't switch on delta-E)
  ... ECP_FIT          TRUE (Convert deprecated ECP files)
  ... GUI              GUI_SPARTAN
  ... TERSE_OUTPUT     TRUE !turn on spartan printing
  ... SCF_CONVERGENCE  7
  ... CCMAN2 FALSE  (qc4.3)
  ... SYMMETRY   FALSE  ! turn of symmetry for spartan16
  ... SYM_IGNORE TRUE   ! ..use FORCESYMMETRY to override
  ... GEOM_OPT_TOL_GRADIENT       700  ! loosen tolernaces for organic geometries
  ... GEOM_OPT_TOL_DISPLACEMENT  1400  ! was 1200 = .0012
  ... GEOM_OPT_TOL_ENERGY        2000  ! was  100 = .000 001
  ... GEN_SCFMAN  FALSE
Processing $rem in input file
  ... JOBTYPE    OPT
  ... TIDY_SYM   TRUE
  ... METHOD            WB97X-D
  ... xc_grid           75000302  (75,302)
  ... BASIS             6-311+G**
  ... THRESH             12  #diffuse default
  ... MAXSCF            350  #diffuse default
  ... GEOM_OPT_TOL_ENERGY       22850
  ... GEOM_OPT_TOL_GRADIENT       800
  ... VARTHRESH         2    (default DFT)
  ... INCDFT            TRUE (default DFT)
  ... GEOM_OPT_HESSIAN      READ (main opt)
  ... EXTERNAL_HESSIAN      1
  ... GUI                GUI_SPARTAN
  ... TERSE_OUTPUT       TRUE
NAlpha2: 130
NElect   130
Mult     1

Checking the input file for inconsistencies...  ...done.

--------------------------------------------------------------
User input:
--------------------------------------------------------------
$comment
Molecule12
$end
$molecule
 2 1
    7     2.5357343105    0.42627173452    0.49234361313
    6     2.7552168359   -0.37645618948   -0.59776766008
    6     3.5865236612    -1.5011109222   -0.46096753931
    6     4.2198931443    -1.7711741596    0.74294832873
    6     4.0308553048   -0.91178246192     1.8089924653
    6     3.1947160477    0.18464664963     1.6585438241
```

```
    6      2.1214738731    -0.1081659636    -1.9357405713
    6      1.6518089742     1.6304065258    0.42250748115
    7     -1.7440024116    0.25556958622     1.3510802297
    6     -2.4733846518    0.66367480806    0.30305365276
    7     -1.9390356681     1.3839421849   -0.70934270811
    6   -0.64297508079     1.7078295592   -0.63831142639
    6    0.17943793573     1.2917875362    0.40144940174
    6   -0.43523519877    0.55369121251      1.417675841
    6     -3.927342862    0.28682263694    0.23348654759
    6    -4.1819711864   -0.91921097647   -0.67196330176
    6    -3.6157068093    -2.2180821941   -0.11465671439
    7   -0.16716612866     2.4531093714    -1.7024433611
    1      3.763214085    -2.1657089717    -1.3079449636
    1     4.8785014968    -2.6348884488    0.83753589203
    1     4.5478298186    -1.0905618015     2.7507989748
    1     3.0527754973     0.8807656384     2.4821714393
    1      2.597593609   -0.70183529656    -2.7238676771
    1     2.2379032197    0.93932962899    -2.2228817305
    1     1.0628673493    -0.3787177051    -1.9113388623
    1     1.9896167534     2.2134794666   -0.43931744724
    1      1.851181278     2.2576366997     1.3000468149
    1    -2.2022995729   -0.28634391296     2.0865029361
    1   0.081683864602    0.17996141459     2.2954063581
    1     -4.309506444   0.092109810119     1.2423415173
    1    -4.4856037902     1.1484366845   -0.15206081155
    1    -3.7655814149   -0.74249666942    -1.6709235915
    1    -5.2650493392    -1.0393581152   -0.79447923325
    1    -2.5232497246    -2.1886293802  -0.060154831742
    1    -3.8940069112    -3.0567658465    -0.7608613988
    1    -4.0068805674    -2.4188180877    0.88779002884
    1    0.17011486372     3.3835967405    -1.4618237291
    1   -0.92994416126     2.5770392143    -2.3778277873
$end
$rem
JOBTYPE    OPT
TIDY_SYM   TRUE
METHOD           WB97X-D
xc_grid          75000302  (75,302)
BASIS            6-311+G**
THRESH            12  #diffuse default
MAXSCF           350  #diffuse default
GEOM_OPT_TOL_ENERGY     22850
GEOM_OPT_TOL_GRADIENT     800
VARTHRESH        2    (default DFT)
INCDFT           TRUE (default DFT)
GEOM_OPT_HESSIAN      READ (main opt)
EXTERNAL_HESSIAN      1
GUI               GUI_SPARTAN
TERSE_OUTPUT      TRUE
$end
$opt
$end
--------------------------------------------------------------
 ----------------------------------------------------------------
             Standard Nuclear Orientation (Angstroms)
    I     Atom           X                Y                Z
 ----------------------------------------------------------------
    1      N       2.5357343105     0.4262717345     0.4923436131
    2      C       2.7552168359    -0.3764561895    -0.5977676601
    3      C       3.5865236612    -1.5011109222    -0.4609675393
    4      C       4.2198931443    -1.7711741596     0.7429483287
    5      C       4.0308553048    -0.9117824619     1.8089924653
    6      C       3.1947160477     0.1846466496     1.6585438241
    7      C       2.1214738731    -0.1081659636    -1.9357405713
    8      C       1.6518089742     1.6304065258     0.4225074811
    9      N      -1.7440024116     0.2555695862     1.3510802297
   10      C      -2.4733846518     0.6636748081     0.3030536528
   11      N      -1.9390356681     1.3839421849    -0.7093427081
   12      C      -0.6429750808     1.7078295592    -0.6383114264
   13      C       0.1794379357     1.2917875362     0.4014494017
```

```
  14      C      -0.4352351988     0.5536912125     1.4176758410
  15      C      -3.9273428620     0.2868226369     0.2334865476
  16      C      -4.1819711864    -0.9192109765    -0.6719633018
  17      C      -3.6157068093    -2.2180821941    -0.1146567144
  18      N      -0.1671661287     2.4531093714    -1.7024433611
  19      H       3.7632140850    -2.1657089717    -1.3079449636
  20      H       4.8785014968    -2.6348884488     0.8375358920
  21      H       4.5478298186    -1.0905618015     2.7507989748
  22      H       3.0527754973     0.8807656384     2.4821714393
  23      H       2.5975936090    -0.7018352966    -2.7238676771
  24      H       2.2379032197     0.9393296290    -2.2228817305
  25      H       1.0628673493    -0.3787177051    -1.9113388623
  26      H       1.9896167534     2.2134794666    -0.4393174472
  27      H       1.8511812780     2.2576366997     1.3000468149
  28      H      -2.2022995729    -0.2863439130     2.0865029361
  29      H       0.0816838646     0.1799614146     2.2954063581
  30      H      -4.3095064440     0.0921098101     1.2423415173
  31      H      -4.4856037902     1.1484366845    -0.1520608116
  32      H      -3.7655814149    -0.7424966694    -1.6709235915
  33      H      -5.2650493392    -1.0393581152    -0.7944792333
  34      H      -2.5232497246    -2.1886293802    -0.0601548317
  35      H      -3.8940069112    -3.0567658465    -0.7608613988
  36      H      -4.0068805674    -2.4188180877     0.8877900288
  37      H       0.1701148637     3.3835967405    -1.4618237291
  38      H      -0.9299441613     2.5770392143    -2.3778277873
 ----------------------------------------------------------------
 Nuclear Repulsion Energy =        1316.31526893 hartrees
 There are       65 alpha and       65 beta electrons
 Requested basis set is 6-311+G(d,p)
 There are 188 shells and 516 basis functions

 Total QAlloc Memory Limit   4076 MB
 Mega-Array Size       188 MB
 MEM_STATIC part       192 MB

 ..                                                                         (5.2.P)

------------------------------------------------
-  Entering fldman on Sun Oct 13 19:18:00 2024  -
------------------------------------------------

 A cutoff of  1.0D-12 yielded  13018 shell pairs
 There are    103010 function pairs (    109780 Cartesian)
 Smallest overlap matrix eigenvalue = 2.17E-06
 Linear dependence detected in AO basis
 Tighter screening thresholds may be required for diffuse basis sets
 Use S2THRESH > 12 and THRESH = 14 in case of SCF convergence issues
 Number of orthogonalized atomic orbitals = 510
 Maximum deviation from orthogonality = 8.688E-12

 Scale SEOQF with 1.000000e-02/1.000000e-01/1.000000e-01

 Standard Electronic Orientation quadrupole field applied
 Nucleus-field energy     =     0.0000000018 hartrees

------------------------------------------------
-  Entering gesman on Sun Oct 13 19:18:01 2024  -
------------------------------------------------

 Guess from superposition of atomic densities
 Warning:  Energy on first SCF cycle will be non-variational
 SAD guess density has 132.000000 electrons

------------------------------------------------
-  Entering scfman on Sun Oct 13 19:18:01 2024  -
------------------------------------------------

 Long-range K will be added via erf
 Coulomb attenuation parameter = 0.2 bohr**(-1)
 A restricted hybrid HF-DFT SCF calculation will be
```

```
 performed using Pulay DIIS + Geometric Direct Minimization
 Exchange:     0.2220 Hartree-Fock + 1.0000 wB97X-D + LR-HF
 Correlation:  1.0000 wB97X-D
 Using Euler-Maclaurin-Lebedev (75,302) quadrature formula
 Dispersion:   Grimme D
 SCF converges when RMS gradient is below 1.0E-07
 Exchange:     0.2220 Hartree-Fock + 1.0000 wB97X-D + LR-HF
 Correlation:  1.0000 wB97X-D
 Using Euler-Maclaurin-Lebedev (75,302) quadrature formula
 Dispersion:   Grimme D
 ---------------------------------------
  Cycle       Energy         DIIS Error
 ---------------------------------------
    1    -770.2949568597      2.95E-02
    2    -763.7489672348      2.40E-03
    3    -763.7516260136      2.61E-03
    4    -763.9445108952      3.75E-04
    5    -763.9479670246      1.86E-04
    6    -763.9489559343      3.40E-05
    7    -763.9489903912      1.34E-05
    8    -763.9489959922      3.16E-06
    9    -763.9489965251      1.34E-06
   10    -763.9489966115      4.67E-07
   11    -763.9489966271      2.04E-07
   12    -763.9489966304      8.65E-08 Convergence criterion met
 ---------------------------------------
 SCF time:  CPU 421.19 s  wall 695.55 s
 SCF   energy in the final basis set = -763.94899663
 Total energy in the final basis set = -763.94899663

--------------------------------------------------
-  Entering anlman on Sun Oct 13 19:29:36 2024  -
--------------------------------------------------


 --------------------------------------------------------------

                   Orbital Energies (a.u.)
 --------------------------------------------------------------

 Alpha MOs
 -- Occupied --
-14.8184 -14.8132 -14.7221 -14.7138 -10.6744 -10.6697 -10.6630 -10.6400
-10.6359 -10.6278 -10.6144 -10.5797 -10.5714 -10.5713 -10.5449 -10.5342
-10.4996 -10.4762  -1.4289  -1.3997  -1.3439  -1.2658  -1.2284  -1.2006
 -1.1802  -1.1472  -1.1054  -1.0777  -1.0638  -1.0516  -1.0293  -1.0121
 -0.9857  -0.9559  -0.9451  -0.9078  -0.9023  -0.8882  -0.8763  -0.8634
 -0.8502  -0.8448  -0.8329  -0.8194  -0.8071  -0.7947  -0.7855  -0.7787
 -0.7748  -0.7548  -0.7505  -0.7452  -0.7340  -0.7287  -0.7261  -0.7068
 -0.7061  -0.6695  -0.6588  -0.6509  -0.6403  -0.6324  -0.6296  -0.6205
 -0.6151
 -- Virtual --
 -0.2786  -0.2689  -0.2553  -0.2277  -0.1781  -0.1630  -0.1534  -0.1476
 -0.1316  -0.1241  -0.1181  -0.1162  -0.1102  -0.1058  -0.1020  -0.0939
 -0.0911  -0.0858  -0.0842  -0.0805  -0.0756  -0.0721  -0.0704  -0.0651
 -0.0633  -0.0626  -0.0572  -0.0527  -0.0520  -0.0447  -0.0412  -0.0404
 -0.0331  -0.0282  -0.0259  -0.0232  -0.0216  -0.0186  -0.0142  -0.0109
 -0.0051  -0.0042  -0.0030   0.0028   0.0083   0.0093   0.0117   0.0124
  0.0165   0.0182   0.0219   0.0288   0.0317   0.0359   0.0373   0.0383
  0.0404   0.0449   0.0465   0.0509   0.0529   0.0575   0.0598   0.0629
  0.0642   0.0687   0.0712   0.0718   0.0770   0.0835   0.0852   0.0883
  0.0919   0.0966   0.1008   0.1031   0.1074   0.1103   0.1131   0.1178
  0.1192   0.1232   0.1244   0.1270   0.1322   0.1372   0.1387   0.1433
  0.1498   0.1535   0.1582   0.1601   0.1640   0.1657   0.1708   0.1731
  0.1748   0.1814   0.1884   0.1908   0.1967   0.2075   0.2129   0.2192
  0.2258   0.2348   0.2461   0.2676   0.2728   0.2897   0.2949   0.3090
  0.3174   0.3244   0.3362   0.3400   0.3452   0.3489   0.3546   0.3644
  0.3757   0.3805   0.3836   0.3883   0.3926   0.4073   0.4157   0.4231
  0.4335   0.4385   0.4416   0.4462   0.4576   0.4629   0.4661   0.4705
  0.4766   0.4878   0.4892   0.4989   0.5017   0.5103   0.5106   0.5163
```

| | | | | | | | |
|---|---|---|---|---|---|---|---|
| 0.5261 | 0.5322 | 0.5358 | 0.5434 | 0.5452 | 0.5494 | 0.5527 | 0.5594 |
| 0.5641 | 0.5678 | 0.5725 | 0.5764 | 0.5765 | 0.5824 | 0.5886 | 0.5968 |
| 0.5986 | 0.6124 | 0.6185 | 0.6283 | 0.6347 | 0.6409 | 0.6510 | 0.6581 |
| 0.6679 | 0.6698 | 0.6753 | 0.6816 | 0.6873 | 0.6916 | 0.7007 | 0.7087 |
| 0.7111 | 0.7277 | 0.7317 | 0.7445 | 0.7561 | 0.7646 | 0.7717 | 0.7814 |
| 0.7880 | 0.8002 | 0.8058 | 0.8102 | 0.8232 | 0.8274 | 0.8372 | 0.8410 |
| 0.8556 | 0.8639 | 0.8757 | 0.8823 | 0.8988 | 0.9036 | 0.9132 | 0.9201 |
| 0.9327 | 0.9432 | 0.9560 | 0.9822 | 0.9892 | 1.0095 | 1.0187 | 1.0390 |
| 1.0489 | 1.0592 | 1.0748 | 1.1019 | 1.1116 | 1.1374 | 1.1716 | 1.1817 |
| 1.2136 | 1.2161 | 1.2315 | 1.2522 | 1.2582 | 1.2838 | 1.2911 | 1.3142 |
| 1.3269 | 1.3300 | 1.3347 | 1.3391 | 1.3543 | 1.3626 | 1.3690 | 1.3774 |
| 1.3843 | 1.3961 | 1.4025 | 1.4117 | 1.4220 | 1.4275 | 1.4324 | 1.4333 |
| 1.4389 | 1.4445 | 1.4477 | 1.4576 | 1.4621 | 1.4739 | 1.4770 | 1.4880 |
| 1.5011 | 1.5051 | 1.5142 | 1.5198 | 1.5285 | 1.5368 | 1.5449 | 1.5584 |
| 1.5603 | 1.5635 | 1.5677 | 1.5779 | 1.5817 | 1.5897 | 1.5993 | 1.6090 |
| 1.6160 | 1.6293 | 1.6415 | 1.6508 | 1.6641 | 1.6667 | 1.6744 | 1.6821 |
| 1.6922 | 1.7013 | 1.7084 | 1.7306 | 1.7374 | 1.7464 | 1.7539 | 1.7596 |
| 1.7798 | 1.7886 | 1.7972 | 1.8216 | 1.8277 | 1.8461 | 1.8504 | 1.8693 |
| 1.8900 | 1.8908 | 1.9032 | 1.9059 | 1.9280 | 1.9456 | 1.9660 | 1.9833 |
| 1.9905 | 1.9994 | 2.0178 | 2.0339 | 2.0343 | 2.0523 | 2.0700 | 2.0765 |
| 2.0960 | 2.1169 | 2.1222 | 2.1394 | 2.1576 | 2.1656 | 2.2008 | 2.2058 |
| 2.2225 | 2.2263 | 2.2442 | 2.2461 | 2.2547 | 2.2867 | 2.2887 | 2.2944 |
| 2.3176 | 2.3269 | 2.3467 | 2.3535 | 2.3626 | 2.3689 | 2.3872 | 2.3896 |
| 2.4119 | 2.4160 | 2.4187 | 2.4411 | 2.4455 | 2.4555 | 2.4836 | 2.4983 |
| 2.5031 | 2.5067 | 2.5209 | 2.5303 | 2.5370 | 2.5433 | 2.5492 | 2.5532 |
| 2.5674 | 2.5703 | 2.5777 | 2.5819 | 2.5890 | 2.6004 | 2.6100 | 2.6136 |
| 2.6221 | 2.6337 | 2.6520 | 2.6572 | 2.6626 | 2.6798 | 2.6864 | 2.6954 |
| 2.7056 | 2.7161 | 2.7207 | 2.7211 | 2.7369 | 2.7432 | 2.7582 | 2.7740 |
| 2.7911 | 2.7982 | 2.8089 | 2.8195 | 2.8319 | 2.8519 | 2.8537 | 2.8846 |
| 2.8905 | 2.9132 | 2.9371 | 2.9833 | 3.0163 | 3.0663 | 3.0918 | 3.1397 |
| 3.1486 | 3.1695 | 3.2248 | 3.2572 | 3.2766 | 3.3011 | 3.3354 | 3.3710 |
| 3.3976 | 3.4689 | 3.4994 | 3.5211 | 3.5380 | 3.5726 | 3.6003 | 3.6672 |
| 3.6737 | 3.6875 | 3.6997 | 3.7118 | 3.7272 | 3.7418 | 3.7516 | 3.7631 |
| 3.8214 | 3.8559 | 3.8963 | 3.8995 | 3.9427 | 4.0265 | 4.0317 | 4.0854 |
| 4.1162 | 4.2031 | 4.2968 | 4.3158 | 4.5578 | 4.6413 | 4.6830 | 4.7047 |
| 4.8724 | 5.0166 | 5.1447 | 23.5161 | 23.5635 | 23.6869 | 23.7147 | 23.7330 |
| 23.7942 | 23.7968 | 23.8261 | 23.8449 | 23.8727 | 23.9050 | 23.9202 | 23.9276 |
| 24.0245 | 35.4089 | 35.4392 | 35.4850 | 35.5780 | | | |

Ground-State Mulliken Net Atomic Charges

| Atom | Charge (a.u.) |
|---|---|
| 1 N | 0.442552 |
| 2 C | 0.056921 |
| 3 C | -0.036939 |
| 4 C | -0.208153 |
| 5 C | -0.297687 |
| 6 C | -0.072362 |
| 7 C | -0.718615 |
| 8 C | -0.203302 |
| 9 N | -0.109461 |
| 10 C | -0.220308 |
| 11 N | 0.059105 |
| 12 C | 0.059917 |
| 13 C | -0.036392 |
| 14 C | 0.094332 |
| 15 C | -0.333638 |
| 16 C | -0.251800 |
| 17 C | -0.526030 |
| 18 N | -0.454005 |
| 19 H | 0.224275 |
| 20 H | 0.234091 |
| 21 H | 0.232203 |
| 22 H | 0.221721 |
| 23 H | 0.232244 |
| 24 H | 0.218853 |
| 25 H | 0.234836 |
| 26 H | 0.225851 |

```
    27 H                    0.247911
    28 H                    0.395484
    29 H                    0.220132
    30 H                    0.208975
    31 H                    0.255531
    32 H                    0.202231
    33 H                    0.225093
    34 H                    0.125838
    35 H                    0.216326
    36 H                    0.179390
    37 H                    0.319216
    38 H                    0.335665
 ----------------------------------------
 Sum of atomic charges =     2.000000

 -----------------------------------------------------------------
                  Cartesian Multipole Moments
 -----------------------------------------------------------------
   Charge (ESU x 10^10)
               9.6064
   Dipole Moment (Debye)
        X       5.6419      Y       2.3572      Z       4.3608
      Tot       7.5102
   Quadrupole Moments (Debye-Ang)
       XX     -13.7971     XY      -8.2775     YY     -72.0890
       XZ       5.0515     YZ      -4.2152     ZZ     -72.2939
   Traceless Quadrupole Moments (Debye-Ang)
      QXX     116.7888    QYY     -58.0870    QZZ     -58.7018
      QXY     -24.8324    QXZ      15.1544    QYZ     -12.6457
   Octopole Moments (Debye-Ang^2)
      XXX       3.1257    XXY    -103.9603    XYY      17.4008
      YYY      -1.4793    XXZ      50.2850    XYZ      -0.1019
      YYZ     -31.5216    XZZ      13.1233    YZZ      -7.0242
      ZZZ       1.7433
   Traceless Octopole Moments (Debye-Ang^2)
      XXX    -255.9625    YYY     989.9847    ZZZ    -158.4112
      XXY   -1222.0128    XXZ     692.7549    XYY     160.0621
      XYZ      -1.5286    XZZ      95.9004    YYZ    -534.3437
      YZZ     232.0281
   Hexadecapole Moments (Debye-Ang^3)
     XXXX   -3581.4842   XXXY    -179.3434   XXYY    -772.0435
     XYYY      59.4581   YYYY    -944.2976   XXXZ      37.8575
     XXYZ       2.6604   XYYZ     -21.0888   YYYZ      -0.6956
     XXZZ    -736.6253   XYZZ       3.4787   YYZZ    -277.1191
     XZZZ    -133.2015   YZZZ      46.1201   ZZZZ    -727.8009
   Traceless Hexadecapole Moments (Debye-Ang^3)
     XXXX    2631.4994   XXXY  -13592.7583   XXXZ    9214.5137
     XXYY   -1285.8423   XXYZ    -440.1307   XXZZ   -1345.6571
     XYYY   11481.3970   XYYZ    -467.8318   XYZZ    2111.3613
     XZZZ   -8746.6819   YYYY     833.7429   YYYZ   -2231.4555
     YYZZ     452.0994   YZZZ    2671.5861   ZZZZ     893.5577
 -----------------------------------------------------------------

--------------------------------------------------
-  Entering drvman on Sun Oct 13 19:29:36 2024  -
--------------------------------------------------

 Calculating analytic gradient of the SCF energy
 Gradient of SCF Energy
           1           2           3           4           5           6
    1  -0.0069828  -0.0047955   0.0020014  -0.0012817   0.0042071  -0.0067364
    2   0.0021100   0.0151268  -0.0067147   0.0049360  -0.0114923   0.0109659
    3   0.0002435   0.0074992  -0.0034023   0.0075820  -0.0081117  -0.0062327
           7           8           9          10          11          12
    1  -0.0022034  -0.0086847  -0.0043523   0.0164342   0.0103680   0.0155635
    2  -0.0042559   0.0006667  -0.0021086  -0.0144302   0.0260165  -0.0183241
    3  -0.0043679  -0.0056860  -0.0011348   0.0405541  -0.0342048   0.0302839
          13          14          15          16          17          18
    1  -0.0040529  -0.0211312  -0.0000385   0.0007189   0.0004843   0.0190348
    2   0.0210575  -0.0112080  -0.0104927   0.0104264   0.0009673  -0.0029619
```

```
   3  -0.0310010   0.0104936  -0.0031053   0.0057955   0.0002329  -0.0056195
         19          20          21          22          23          24
   1   0.0038361   0.0030859   0.0039773   0.0022761   0.0005585   0.0046388
   2  -0.0068283  -0.0031504  -0.0043237   0.0004226  -0.0000474   0.0000375
   3  -0.0018253   0.0003307   0.0024217   0.0049449  -0.0065216   0.0002461
         25          26          27          28          29          30
   1   0.0013366   0.0078168  -0.0038259  -0.0037120  -0.0022690  -0.0010359
   2  -0.0032285   0.0025286   0.0050590  -0.0034566  -0.0009985   0.0010500
   3   0.0013262  -0.0002859   0.0019818   0.0034707   0.0018922   0.0003949
         31          32          33          34          35          36
   1  -0.0046510   0.0021922  -0.0028144  -0.0019352  -0.0018020   0.0001356
   2   0.0005802  -0.0028956  -0.0043880   0.0018766  -0.0011678   0.0014450
   3  -0.0008089  -0.0017473  -0.0006279   0.0000759  -0.0027561   0.0004252
         37          38
   1  -0.0088545  -0.0075069
   2   0.0056653   0.0015353
   3   0.0001674  -0.0029238
 Max gradient component =       4.055E-02
 RMS gradient           =       9.414E-03
 Gradient time:  CPU 158.13 s  wall 259.66 s

-------------------------------------------------
-  Entering optman on Sun Oct 13 19:33:56 2024  -
-------------------------------------------------

 Geometry Optimization Parameters
   NAtoms,    NIC,     NZ,  NCons,   NDum,   NFix, NCnnct, MaxDiis
       38     272       0       0       0       0       0       0

 Cartesian Hessian read from HESS file


** GEOMETRY OPTIMIZATION IN DELOCALIZED INTERNAL COORDINATES **
   Searching for a Minimum

   Optimization Cycle:   1

                     Coordinates (Angstroms)
     ATOM                X               Y               Z
      1  N         2.5357343105    0.4262717345    0.4923436131
      2  C         2.7552168359   -0.3764561895   -0.5977676601
      3  C         3.5865236612   -1.5011109222   -0.4609675393
      4  C         4.2198931443   -1.7711741596    0.7429483287
      5  C         4.0308553048   -0.9117824619    1.8089924653
      6  C         3.1947160477    0.1846466496    1.6585438241
      7  C         2.1214738731   -0.1081659636   -1.9357405713
      8  C         1.6518089742    1.6304065258    0.4225074811
      9  N        -1.7440024116    0.2555695862    1.3510802297
     10  C        -2.4733846518    0.6636748081    0.3030536528
     11  N        -1.9390356681    1.3839421849   -0.7093427081
     12  C        -0.6429750808    1.7078295592   -0.6383114264
     13  C         0.1794379357    1.2917875362    0.4014494017
     14  C        -0.4352351988    0.5536912125    1.4176758410
     15  C        -3.9273428620    0.2868226369    0.2334865476
     16  C        -4.1819711864   -0.9192109765   -0.6719633018
     17  C        -3.6157068093   -2.2180821941   -0.1146567144
     18  N        -0.1671661287    2.4531093714   -1.7024433611
     19  H         3.7632140850   -2.1657089717   -1.3079449636
     20  H         4.8785014968   -2.6348884488    0.8375358920
     21  H         4.5478298186   -1.0905618015    2.7507989748
     22  H         3.0527754973    0.8807656384    2.4821714393
     23  H         2.5975936090   -0.7018352966   -2.7238676771
     24  H         2.2379032197    0.9393296290   -2.2228817305
     25  H         1.0628673493   -0.3787177051   -1.9113388623
     26  H         1.9896167534    2.2134794666   -0.4393174472
     27  H         1.8511812780    2.2576366997    1.3000468149
     28  H        -2.2022995729   -0.2863439130    2.0865029361
     29  H         0.0816838646    0.1799614146    2.2954063581
     30  H        -4.3095064440    0.0921098101    1.2423415173
     31  H        -4.4856037902    1.1484366845   -0.1520608115
```

```
    32  H        -3.7655814149   -0.7424966694   -1.6709235915
    33  H        -5.2650493392   -1.0393581152   -0.7944792332
    34  H        -2.5232497246   -2.1886293802   -0.0601548317
    35  H        -3.8940069112   -3.0567658465   -0.7608613988
    36  H        -4.0068805674   -2.4188180877    0.8877900288
    37  H         0.1701148637    3.3835967405   -1.4618237291
    38  H        -0.9299441613    2.5770392143   -2.3778277873
  Point Group: c1    Number of degrees of freedom:   108


  Energy is   -763.948996630


 Attempting to generate delocalized internal coordinates

 Transforming Cartesian Hessian to Internal Coordinates
 Hessian Transformation does not Include Derivative of B-matrix
  internal optimization (0)

108 Hessian modes will be used to form the next step
  Hessian Eigenvalues:
     0.002204    0.002585    0.003953    0.005610    0.009309    0.012115
     0.014838    0.018764    0.019272    0.019857    0.021052    0.022378
     0.022521    0.022892    0.023989    0.024478    0.025688    0.026656
     0.028213    0.028449    0.029634    0.030844    0.035715    0.037068
     0.038746    0.041880    0.043212    0.043963    0.045302    0.047623
     0.053552    0.053867    0.055301    0.055426    0.062466    0.078774
     0.085418    0.093260    0.121398    0.121732    0.127105    0.130667
     0.131627    0.132976    0.134697    0.139091    0.143077    0.147513
     0.147680    0.148221    0.149549    0.151112    0.152514    0.153055
     0.153780    0.154547    0.176931    0.206054    0.209732    0.216785
     0.219607    0.230564    0.236769    0.240851    0.246503    0.256655
     0.262704    0.268989    0.281374    0.297242    0.299764    0.300128
     0.300606    0.301190    0.301665    0.301817    0.304080    0.304383
     0.305153    0.305406    0.307497    0.308017    0.311591    0.315222
     0.319564    0.329669    0.332030    0.334383    0.336974    0.341661
     0.342143    0.349269    0.349856    0.362019    0.364020    0.379800
     0.393458    0.395186    0.405952    0.415987    0.420288    0.421668
     0.439840    0.441205    0.450478    0.462399    0.562375    0.782226

 Minimum search - taking simple RFO step
 Searching for Lamda that Minimizes Along All modes
 Value Taken    Lamda =  -0.02350483
 Calculated Step too Large.  Step scaled by  0.718117 !!
 Step Taken.  Stepsize is  0.300000  0.018744

                             Maximum     Tolerance    Cnvgd?
        Gradient            0.022038      0.000800      NO
        Displacement        0.087843      0.001400      NO
        Energy change      *********      0.000228      NO


 New Cartesian Coordinates Obtained by Inverse Iteration

 Displacement from previous Coordinates is:  0.526491
 ----------------------------------------------------------------
             Standard Nuclear Orientation (Angstroms)
    I     Atom           X                Y                Z
 ----------------------------------------------------------------
    1      N       2.5443203912     0.4063442636     0.4974570984
    2      C       2.7445110658    -0.3987435963    -0.5875772157
    3      C       3.5781158128    -1.5005145103    -0.4512131726
    4      C       4.2237824692    -1.7573149107     0.7491996584
    5      C       4.0381286536    -0.8955563127     1.8238168825
    6      C       3.2021009060     0.1766489715     1.6651473853
    7      C       2.0721537328    -0.1181532736    -1.8962759380
    8      C       1.6613044154     1.6047917141     0.4244283128
    9      N      -1.7294029768     0.2651152857     1.3144996616
   10      C      -2.4892926082     0.6782379212     0.2754856195
   11      N      -1.9742013219     1.3936522709    -0.6993025252
```

```
   12      C      -0.6752381982     1.7264293419    -0.6396304598
   13      C       0.1842433785     1.2770793757     0.3991166146
   14      C      -0.4122216455     0.5478479280     1.3853696902
   15      C      -3.9316133440     0.2948953771     0.2341634332
   16      C      -4.1792421198    -0.9217559221    -0.6753067959
   17      C      -3.5785298905    -2.2148010768    -0.1321669804
   18      N      -0.1971021944     2.4649024853    -1.6931872648
   19      H       3.7381145724    -2.1545058017    -1.3012331146
   20      H       4.8841998736    -2.6143114274     0.8421274343
   21      H       4.5502787198    -1.0527940367     2.7654832219
   22      H       3.0434331644     0.8845075106     2.4697641162
   23      H       2.5302707307    -0.7211931337    -2.6799715155
   24      H       2.1648588433     0.9271103815    -2.1972584116
   25      H       1.0107649714    -0.3783302398    -1.8536714779
   26      H       1.9615108716     2.1839188985    -0.4471461038
   27      H       1.8839101511     2.2250636965     1.2954457650
   28      H      -2.1764014170    -0.2803040022     2.0474783515
   29      H       0.1081090736     0.1561056239     2.2519114741
   30      H      -4.2925994009     0.0842126599     1.2466813892
   31      H      -4.4947837121     1.1548046648    -0.1369232027
   32      H      -3.7892286749    -0.7189992655    -1.6774722686
   33      H      -5.2608000586    -1.0399881638    -0.7766154229
   34      H      -2.4834410947    -2.1752445200    -0.0956500596
   35      H      -3.8490316037    -3.0575816551    -0.7708001078
   36      H      -3.9494267818    -2.4312938322     0.8747604561
   37      H       0.2738737626     3.3375514990    -1.4801036792
   38      H      -0.9354285174     2.6421658107    -2.3708308479
 ----------------------------------------------------------------
 Nuclear Repulsion Energy =        1320.09124106 hartrees
 There are       65 alpha and       65 beta electrons

------------------------------------------------
-  Entering fldman on Sun Oct 13 19:33:56 2024  -
------------------------------------------------

 Applying Cartesian multipole field
    Component          Value
    ---------          -----
     (2,0,0)        1.00000E-12
     (0,2,0)        2.00000E-11
     (0,0,2)       -3.00000E-11
 Nucleus-field energy     =     0.0000000021 hartrees

------------------------------------------------
-  Entering gesman on Sun Oct 13 19:33:56 2024  -
------------------------------------------------

 Requested basis set is 6-311+G(d,p)
 There are 188 shells and 516 basis functions
 A cutoff of  1.0D-12 yielded  13038 shell pairs
 There are    103162 function pairs (    109952 Cartesian)
 Smallest overlap matrix eigenvalue = 2.10E-06
 Linear dependence detected in AO basis
 Tighter screening thresholds may be required for diffuse basis sets
 Use S2THRESH > 12 and THRESH = 14 in case of SCF convergence issues
 Number of orthogonalized atomic orbitals = 510
 Maximum deviation from orthogonality = 3.496E-11
 Guess MOs from SCF MO coefficient file
 Reading MOs from coefficient file
 Reading MOs from coefficient file

------------------------------------------------
-  Entering scfman on Sun Oct 13 19:33:56 2024  -
------------------------------------------------

 Long-range K will be added via erf
 Coulomb attenuation parameter = 0.2 bohr**(-1)
 A restricted hybrid HF-DFT SCF calculation will be
 performed using Pulay DIIS + Geometric Direct Minimization
 Exchange:     0.2220 Hartree-Fock + 1.0000 wB97X-D + LR-HF
```

```
 Correlation:  1.0000 wB97X-D
 Using Euler-Maclaurin-Lebedev (75,302) quadrature formula
 Dispersion:   Grimme D
 SCF converges when RMS gradient is below 1.0E-07
 Geometry optimization detected.  Setting ReadMinima to 0
 Setting SaveMinima to 0
 ---------------------------------------
  Cycle       Energy         DIIS Error
 ---------------------------------------
    1    -764.0556909962      8.62E-04
    2    -763.9589019260      1.66E-04
    3    -763.9593376915      1.52E-04
    4    -763.9599653679      2.47E-05
    5    -763.9599874611      1.24E-05
    6    -763.9599939773      4.55E-06
    7    -763.9599950709      1.76E-06
    8    -763.9599952468      5.19E-07
    9    -763.9599952713      2.51E-07
   10    -763.9599952745      9.01E-08 Convergence criterion met
 ---------------------------------------
 SCF time:  CPU 369.89 s  wall 616.83 s
 SCF   energy in the final basis set = -763.95999527
 Total energy in the final basis set = -763.95999527

--------------------------------------------------
-  Entering anlman on Sun Oct 13 19:44:13 2024  -
--------------------------------------------------



 --------------------------------------------------------------

                   Orbital Energies (a.u.)
 --------------------------------------------------------------

 Alpha MOs
 -- Occupied --
-14.8171 -14.8029 -14.7216 -14.7173 -10.6763 -10.6687 -10.6550 -10.6388
-10.6352 -10.6260 -10.6106 -10.5828 -10.5711 -10.5705 -10.5465 -10.5313
-10.4995 -10.4763  -1.4247  -1.4030  -1.3433  -1.2747  -1.2308  -1.2021
 -1.1863  -1.1455  -1.1047  -1.0817  -1.0595  -1.0537  -1.0321  -1.0099
 -0.9894  -0.9544  -0.9472  -0.9102  -0.9036  -0.8914  -0.8796  -0.8643
 -0.8494  -0.8482  -0.8351  -0.8211  -0.8090  -0.7967  -0.7894  -0.7806
 -0.7776  -0.7543  -0.7517  -0.7476  -0.7360  -0.7286  -0.7261  -0.7134
 -0.7058  -0.6728  -0.6591  -0.6493  -0.6413  -0.6330  -0.6289  -0.6202
 -0.6192
 -- Virtual --
 -0.2723  -0.2697  -0.2521  -0.2260  -0.1759  -0.1632  -0.1521  -0.1472
 -0.1325  -0.1229  -0.1179  -0.1166  -0.1097  -0.1051  -0.1014  -0.0947
 -0.0912  -0.0848  -0.0845  -0.0797  -0.0747  -0.0718  -0.0698  -0.0647
 -0.0631  -0.0619  -0.0567  -0.0534  -0.0506  -0.0444  -0.0404  -0.0390
 -0.0316  -0.0274  -0.0247  -0.0241  -0.0209  -0.0182  -0.0134  -0.0096
 -0.0063  -0.0035  -0.0021   0.0050   0.0092   0.0108   0.0119   0.0138
  0.0173   0.0178   0.0216   0.0305   0.0317   0.0374   0.0383   0.0387
  0.0412   0.0448   0.0465   0.0518   0.0537   0.0599   0.0613   0.0637
  0.0653   0.0698   0.0727   0.0734   0.0766   0.0837   0.0853   0.0881
  0.0946   0.0966   0.1016   0.1047   0.1087   0.1103   0.1151   0.1182
  0.1208   0.1234   0.1251   0.1298   0.1325   0.1386   0.1412   0.1447
  0.1518   0.1542   0.1582   0.1620   0.1653   0.1685   0.1702   0.1742
  0.1773   0.1823   0.1897   0.1908   0.1980   0.2083   0.2123   0.2189
  0.2269   0.2350   0.2469   0.2654   0.2721   0.2871   0.2934   0.3088
  0.3200   0.3274   0.3360   0.3377   0.3486   0.3510   0.3544   0.3673
  0.3782   0.3808   0.3860   0.3918   0.3937   0.4068   0.4192   0.4240
  0.4332   0.4388   0.4418   0.4463   0.4590   0.4656   0.4697   0.4739
  0.4770   0.4915   0.4936   0.5000   0.5047   0.5126   0.5132   0.5200
  0.5302   0.5333   0.5367   0.5428   0.5466   0.5498   0.5553   0.5594
  0.5636   0.5663   0.5727   0.5766   0.5790   0.5856   0.5892   0.5990
  0.6006   0.6126   0.6193   0.6281   0.6359   0.6414   0.6534   0.6585
  0.6672   0.6708   0.6766   0.6803   0.6904   0.6938   0.7004   0.7096
  0.7142   0.7261   0.7337   0.7425   0.7593   0.7679   0.7741   0.7827
  0.7909   0.8016   0.8052   0.8154   0.8245   0.8327   0.8359   0.8474
```

| | | | | | | | |
|---|---|---|---|---|---|---|---|
| 0.8575 | 0.8646 | 0.8765 | 0.8864 | 0.9015 | 0.9068 | 0.9162 | 0.9268 |
| 0.9363 | 0.9411 | 0.9594 | 0.9826 | 0.9940 | 1.0106 | 1.0201 | 1.0429 |
| 1.0530 | 1.0616 | 1.0778 | 1.1039 | 1.1115 | 1.1388 | 1.1676 | 1.1791 |
| 1.2079 | 1.2163 | 1.2328 | 1.2519 | 1.2566 | 1.2843 | 1.3038 | 1.3180 |
| 1.3268 | 1.3298 | 1.3341 | 1.3440 | 1.3538 | 1.3617 | 1.3721 | 1.3773 |
| 1.3876 | 1.3967 | 1.4036 | 1.4136 | 1.4196 | 1.4302 | 1.4332 | 1.4353 |
| 1.4401 | 1.4450 | 1.4527 | 1.4628 | 1.4671 | 1.4770 | 1.4852 | 1.4919 |
| 1.5025 | 1.5099 | 1.5172 | 1.5194 | 1.5315 | 1.5400 | 1.5471 | 1.5610 |
| 1.5618 | 1.5650 | 1.5694 | 1.5819 | 1.5863 | 1.5880 | 1.6060 | 1.6113 |
| 1.6187 | 1.6303 | 1.6486 | 1.6589 | 1.6657 | 1.6700 | 1.6779 | 1.6895 |
| 1.6961 | 1.7048 | 1.7085 | 1.7363 | 1.7432 | 1.7513 | 1.7578 | 1.7626 |
| 1.7820 | 1.7903 | 1.7979 | 1.8236 | 1.8370 | 1.8555 | 1.8615 | 1.8830 |
| 1.8908 | 1.8977 | 1.9032 | 1.9155 | 1.9338 | 1.9493 | 1.9638 | 1.9798 |
| 1.9958 | 2.0000 | 2.0215 | 2.0379 | 2.0401 | 2.0586 | 2.0660 | 2.0822 |
| 2.1093 | 2.1124 | 2.1233 | 2.1442 | 2.1608 | 2.1782 | 2.2055 | 2.2130 |
| 2.2270 | 2.2299 | 2.2476 | 2.2519 | 2.2620 | 2.2902 | 2.2977 | 2.3016 |
| 2.3165 | 2.3333 | 2.3514 | 2.3561 | 2.3654 | 2.3758 | 2.3894 | 2.3924 |
| 2.4133 | 2.4172 | 2.4193 | 2.4393 | 2.4494 | 2.4580 | 2.4786 | 2.4992 |
| 2.5046 | 2.5108 | 2.5247 | 2.5340 | 2.5413 | 2.5448 | 2.5531 | 2.5577 |
| 2.5678 | 2.5713 | 2.5795 | 2.5849 | 2.5918 | 2.6057 | 2.6123 | 2.6172 |
| 2.6306 | 2.6397 | 2.6587 | 2.6616 | 2.6686 | 2.6773 | 2.6944 | 2.6990 |
| 2.7061 | 2.7166 | 2.7206 | 2.7343 | 2.7453 | 2.7497 | 2.7698 | 2.7771 |
| 2.7925 | 2.8026 | 2.8077 | 2.8231 | 2.8445 | 2.8542 | 2.8604 | 2.8883 |
| 2.8980 | 2.9254 | 2.9415 | 2.9887 | 3.0273 | 3.0729 | 3.1203 | 3.1365 |
| 3.1571 | 3.1846 | 3.2403 | 3.2524 | 3.2910 | 3.3170 | 3.3551 | 3.3787 |
| 3.4149 | 3.4771 | 3.5120 | 3.5315 | 3.5489 | 3.5885 | 3.6153 | 3.6528 |
| 3.6824 | 3.6995 | 3.7105 | 3.7140 | 3.7341 | 3.7464 | 3.7564 | 3.7705 |
| 3.8287 | 3.8661 | 3.8929 | 3.9037 | 3.9371 | 4.0066 | 4.0334 | 4.0918 |
| 4.1007 | 4.2006 | 4.3007 | 4.3229 | 4.5758 | 4.6505 | 4.6901 | 4.7150 |
| 4.8858 | 5.0316 | 5.1711 | 23.5201 | 23.5651 | 23.7163 | 23.7210 | 23.7380 |
| 23.7768 | 23.7990 | 23.8355 | 23.8535 | 23.8923 | 23.9079 | 23.9640 | 23.9716 |
| 24.0369 | 35.4181 | 35.4458 | 35.4997 | 35.5942 | | | |

Ground-State Mulliken Net Atomic Charges

| Atom | Charge (a.u.) |
|---|---|
| 1 N | 0.438653 |
| 2 C | 0.085191 |
| 3 C | -0.033308 |
| 4 C | -0.219897 |
| 5 C | -0.289633 |
| 6 C | -0.075584 |
| 7 C | -0.729620 |
| 8 C | -0.202571 |
| 9 N | -0.147963 |
| 10 C | -0.193535 |
| 11 N | 0.055558 |
| 12 C | 0.084456 |
| 13 C | -0.042542 |
| 14 C | 0.106495 |
| 15 C | -0.333336 |
| 16 C | -0.245807 |
| 17 C | -0.531665 |
| 18 N | -0.461500 |
| 19 H | 0.221761 |
| 20 H | 0.235187 |
| 21 H | 0.231413 |
| 22 H | 0.217939 |
| 23 H | 0.231868 |
| 24 H | 0.218446 |
| 25 H | 0.238553 |
| 26 H | 0.211745 |
| 27 H | 0.246461 |
| 28 H | 0.390164 |
| 29 H | 0.216810 |
| 30 H | 0.210019 |
| 31 H | 0.254212 |
| 32 H | 0.201290 |

```
    33 H                      0.224862
    34 H                      0.126403
    35 H                      0.216450
    36 H                      0.180659
    37 H                      0.317290
    38 H                      0.345076
  ----------------------------------------
  Sum of atomic charges =     2.000000

 -----------------------------------------------------------------
                   Cartesian Multipole Moments
 -----------------------------------------------------------------
    Charge (ESU x 10^10)
                 9.6064
    Dipole Moment (Debye)
         X       5.9905      Y       2.5181      Z       3.9381
       Tot       7.5984
    Quadrupole Moments (Debye-Ang)
        XX     -14.0838     XY      -8.0358     YY     -71.6433
        XZ       5.8486     YZ      -5.1914     ZZ     -72.4476
    Traceless Quadrupole Moments (Debye-Ang)
       QXX     115.9233    QYY     -56.7553    QZZ     -59.1680
       QXY     -24.1073    QXZ      17.5458    QYZ     -15.5743
    Octopole Moments (Debye-Ang^2)
       XXX       7.1649    XXY    -102.5232    XYY      17.9517
       YYY      -0.9154    XXZ      51.6493    XYZ      -0.6504
       YYZ     -34.0700    XZZ      15.1412    YZZ      -4.9563
       ZZZ      -0.2079
    Traceless Octopole Moments (Debye-Ang^2)
       XXX    -254.8467    YYY     961.8238    ZZZ    -159.4613
       XXY   -1212.6638    XXZ     722.6251    XYY     148.5023
       XYZ      -9.7557    XZZ     106.3444    YYZ    -563.1638
       YZZ     250.8400
    Hexadecapole Moments (Debye-Ang^3)
      XXXX   -3566.1373   XXXY    -175.0810   XXYY    -772.2364
      XYYY      71.8354   YYYY    -943.2129   XXXZ      36.5750
      XXYZ       0.9737   XYYZ     -28.3441   YYYZ      -7.8259
      XXZZ    -734.5543   XYZZ       6.4718   YYZZ    -268.2911
      XZZZ    -138.2012   YZZZ      40.3337   ZZZZ    -717.5175
    Traceless Hexadecapole Moments (Debye-Ang^3)
      XXXX    3125.8268   XXXY  -14028.6863   XXXZ    9689.0381
      XXYY   -1565.8912   XXYZ    -394.9761   XXZZ   -1559.9356
      XYYY   11897.5369   XYYZ   -1026.5740   XYZZ    2131.1494
      XZZZ   -8662.4641   YYYY     505.9983   YYYZ   -2313.3737
      YYZZ    1059.8929   YZZZ    2708.3498   ZZZZ     500.0427
 -----------------------------------------------------------------


-------------------------------------------------
-  Entering drvman on Sun Oct 13 19:44:13 2024  -
-------------------------------------------------

 Calculating analytic gradient of the SCF energy
 Gradient of SCF Energy
           1           2           3           4           5           6
    1  -0.0019475   0.0023433  -0.0031186  -0.0011481  -0.0014840   0.0016167
    2  -0.0015337   0.0021742   0.0003972   0.0026764   0.0010814  -0.0008947
    3   0.0003772   0.0038666  -0.0043015   0.0028961  -0.0006463  -0.0030343
           7           8           9          10          11          12
    1  -0.0004350  -0.0032037   0.0008485   0.0112894  -0.0078574  -0.0005093
    2  -0.0017299   0.0004508   0.0012644   0.0000206   0.0045750  -0.0097395
    3  -0.0015619  -0.0024818  -0.0033391   0.0073431  -0.0057223   0.0175613
          13          14          15          16          17          18
    1  -0.0025979  -0.0022604   0.0012597  -0.0006949  -0.0001765   0.0161384
    2   0.0009911   0.0008533  -0.0029678   0.0051059  -0.0008695  -0.0014174
    3  -0.0021945  -0.0020664  -0.0025526   0.0021312  -0.0003875  -0.0089429
          19          20          21          22          23          24
    1   0.0012046   0.0010820   0.0011502   0.0014793   0.0003878   0.0014267
    2  -0.0020910  -0.0008693  -0.0014340  -0.0006494   0.0004727   0.0001191
    3  -0.0001923   0.0002914   0.0002764   0.0017157  -0.0015026  -0.0004471
          25          26          27          28          29          30
```

```
    1   0.0002703   0.0016888  -0.0013150  -0.0018664  -0.0010724  -0.0004763
    2  -0.0010676   0.0010962   0.0016286  -0.0015298  -0.0000249   0.0004913
    3   0.0002101   0.0007114   0.0007089   0.0013151   0.0007925   0.0002525
           31          32          33          34          35          36
    1  -0.0020871   0.0010566  -0.0006643  -0.0003737  -0.0005635   0.0000914
    2   0.0000106  -0.0015168  -0.0016592   0.0006446  -0.0001775   0.0006380
    3   0.0004263  -0.0004985  -0.0003328   0.0001387  -0.0007209   0.0003120
           37          38
    1  -0.0076842  -0.0017975
    2   0.0048336   0.0006470
    3  -0.0003225  -0.0000787
 Max gradient component =       1.756E-02
 RMS gradient           =       3.486E-03
 Gradient time:  CPU 158.95 s  wall 267.27 s

-------------------------------------------------
-  Entering optman on Sun Oct 13 19:48:40 2024  -
-------------------------------------------------

 Geometry Optimization Parameters
   NAtoms,    NIC,     NZ,  NCons,   NDum,   NFix, NCnnct, MaxDiis
       38     272       0       0       0       0       0       0

 Cartesian Hessian Update
 Hessian updated using BFGS update


** GEOMETRY OPTIMIZATION IN DELOCALIZED INTERNAL COORDINATES **
   Searching for a Minimum

   Optimization Cycle:   2

                    Coordinates (Angstroms)
     ATOM                X               Y               Z
      1  N         2.5443203912    0.4063442636    0.4974570984
      2  C         2.7445110658   -0.3987435963   -0.5875772157
      3  C         3.5781158128   -1.5005145103   -0.4512131726
      4  C         4.2237824692   -1.7573149107    0.7491996584
      5  C         4.0381286536   -0.8955563127    1.8238168825
      6  C         3.2021009060    0.1766489715    1.6651473853
      7  C         2.0721537328   -0.1181532736   -1.8962759380
      8  C         1.6613044154    1.6047917141    0.4244283128
      9  N        -1.7294029768    0.2651152857    1.3144996616
     10  C        -2.4892926082    0.6782379212    0.2754856195
     11  N        -1.9742013219    1.3936522709   -0.6993025252
     12  C        -0.6752381982    1.7264293419   -0.6396304598
     13  C         0.1842433785    1.2770793757    0.3991166146
     14  C        -0.4122216455    0.5478479280    1.3853696902
     15  C        -3.9316133440    0.2948953771    0.2341634332
     16  C        -4.1792421198   -0.9217559221   -0.6753067959
     17  C        -3.5785298905   -2.2148010768   -0.1321669804
     18  N        -0.1971021944    2.4649024853   -1.6931872648
     19  H         3.7381145724   -2.1545058017   -1.3012331146
     20  H         4.8841998736   -2.6143114274    0.8421274343
     21  H         4.5502787198   -1.0527940367    2.7654832219
     22  H         3.0434331644    0.8845075106    2.4697641162
     23  H         2.5302707307   -0.7211931337   -2.6799715155
     24  H         2.1648588433    0.9271103815   -2.1972584116
     25  H         1.0107649714   -0.3783302398   -1.8536714779
     26  H         1.9615108716    2.1839188985   -0.4471461038
     27  H         1.8839101511    2.2250636965    1.2954457650
     28  H        -2.1764014170   -0.2803040022    2.0474783515
     29  H         0.1081090736    0.1561056239    2.2519114741
     30  H        -4.2925994009    0.0842126599    1.2466813892
     31  H        -4.4947837121    1.1548046648   -0.1369232027
     32  H        -3.7892286749   -0.7189992655   -1.6774722686
     33  H        -5.2608000586   -1.0399881638   -0.7766154229
     34  H        -2.4834410947   -2.1752445200   -0.0956500596
     35  H        -3.8490316037   -3.0575816551   -0.7708001078
     36  H        -3.9494267818   -2.4312938322    0.8747604561
```

```
    37  H         0.2738737626    3.3375514990   -1.4801036792
    38  H        -0.9354285174    2.6421658107   -2.3708308479
  Point Group: c1    Number of degrees of freedom:   108


  Energy is   -763.959995275

 Hessian updated using BFGS update
  internal optimization (0)

108 Hessian modes will be used to form the next step
  Hessian Eigenvalues:
     0.002206    0.002584    0.003954    0.005609    0.009327    0.011494
     0.014820    0.018744    0.019289    0.019858    0.021048    0.022374
     0.022570    0.022893    0.023986    0.024468    0.025683    0.026658
     0.028210    0.028451    0.029586    0.030687    0.035468    0.036956
     0.038758    0.041888    0.043210    0.043943    0.045282    0.047569
     0.052898    0.053602    0.054851    0.055343    0.062432    0.078453
     0.085375    0.093244    0.121355    0.121753    0.127046    0.129828
     0.131394    0.132965    0.134047    0.138342    0.143145    0.145778
     0.147685    0.148224    0.149357    0.150592    0.152468    0.152944
     0.153855    0.156534    0.180656    0.205906    0.209661    0.217073
     0.222077    0.230429    0.235068    0.239262    0.247662    0.258003
     0.267117    0.270037    0.280480    0.297577    0.299466    0.300189
     0.300607    0.301199    0.301590    0.301848    0.304074    0.304395
     0.305175    0.305427    0.307517    0.308073    0.312077    0.317120
     0.321954    0.329006    0.332151    0.334373    0.336981    0.341642
     0.342156    0.349050    0.360851    0.362710    0.379389    0.390778
     0.392636    0.405084    0.413636    0.414615    0.420264    0.425724
     0.440300    0.441941    0.461019    0.484538    0.573140    0.779948

 Minimum search - taking simple RFO step
 Searching for Lamda that Minimizes Along All modes
 Value Taken    Lamda =  -0.00831181
 Calculated Step too Large.  Step scaled by  0.552857 !!
 Step Taken.  Stepsize is  0.300000  0.014739

                          Maximum     Tolerance    Cnvgd?
        Gradient          0.008343     0.000800      NO
        Displacement      0.085526     0.001400      NO
        Energy change    -0.010999     0.000228      NO


 New Cartesian Coordinates Obtained by Inverse Iteration

 Displacement from previous Coordinates is:  0.470780
 ----------------------------------------------------------------
            Standard Nuclear Orientation (Angstroms)
    I     Atom           X                Y                Z
 ----------------------------------------------------------------
    1      N       2.5413181479     0.4034274526     0.4968614042
    2      C       2.7281118977    -0.4133953411    -0.5795820511
    3      C       3.5713082437    -1.5071470032    -0.4359516678
    4      C       4.2262868564    -1.7543084965     0.7594832229
    5      C       4.0383808728    -0.8843896835     1.8301799836
    6      C       3.1976764858     0.1845886373     1.6669313516
    7      C       2.0317306837    -0.1416843262    -1.8744397941
    8      C       1.6649288020     1.6003793487     0.4138425096
    9      N      -1.7229641999     0.2580981239     1.3063543394
   10      C      -2.4961987483     0.6824369397     0.2762934615
   11      N      -1.9786630003     1.4125276007    -0.6845968900
   12      C      -0.6851661898     1.7603902369    -0.6398435427
   13      C       0.1848270493     1.2798755101     0.3857465374
   14      C      -0.4021178858     0.5360789649     1.3676002326
   15      C      -3.9329321225     0.2929109310     0.2396775795
   16      C      -4.1691359040    -0.9240420066    -0.6796886750
   17      C      -3.5482835022    -2.2130570481    -0.1498582357
   18      N      -0.2311564596     2.5029483192    -1.6793772775
   19      H       3.7215042351    -2.1605888387    -1.2859405397
   20      H       4.8881538672    -2.6074092760     0.8544055277
```

```
  21      H       4.5482492205    -1.0261354257     2.7741075121
  22      H       3.0319134422     0.9020643938     2.4598379716
  23      H       2.4706397881    -0.7604084065    -2.6547314851
  24      H       2.1166990418     0.8992698659    -2.1906300273
  25      H       0.9689405051    -0.3919563769    -1.8102831110
  26      H       1.9570410239     2.1680030182    -0.4668026789
  27      H       1.8966852820     2.2232629782     1.2782187491
  28      H      -2.1588603504    -0.2957714909     2.0359984375
  29      H       0.1317122259     0.1281455490     2.2181205372
  30      H      -4.2880795265     0.0693806266     1.2508379115
  31      H      -4.4961529944     1.1528838221    -0.1267987336
  32      H      -3.7913434357    -0.6994141965    -1.6810291260
  33      H      -5.2497284983    -1.0458006723    -0.7740870019
  34      H      -2.4529537337    -2.1637664370    -0.1186878394
  35      H      -3.8094812300    -3.0549772664    -0.7919958132
  36      H      -3.9118351053    -2.4457944172     0.8560406415
  37      H       0.3765125907     3.2931082098    -1.5112129375
  38      H      -0.9675673754     2.7402661806    -2.3350004829
 ----------------------------------------------------------------
 Nuclear Repulsion Energy =        1321.30395372 hartrees
 There are       65 alpha and       65 beta electrons

--------------------------------------------------
-  Entering fldman on Sun Oct 13 19:48:41 2024  -
--------------------------------------------------

 Applying Cartesian multipole field
    Component          Value
    ---------          -----
     (2,0,0)        1.00000E-12
     (0,2,0)        2.00000E-11
     (0,0,2)       -3.00000E-11
 Nucleus-field energy     =     0.0000000024 hartrees

--------------------------------------------------
-  Entering gesman on Sun Oct 13 19:48:41 2024  -
--------------------------------------------------

 Requested basis set is 6-311+G(d,p)
 There are 188 shells and 516 basis functions
 A cutoff of  1.0D-12 yielded  13053 shell pairs
 There are    103232 function pairs (    110023 Cartesian)
 Smallest overlap matrix eigenvalue = 2.09E-06
 Linear dependence detected in AO basis
 Tighter screening thresholds may be required for diffuse basis sets
 Use S2THRESH > 12 and THRESH = 14 in case of SCF convergence issues
 Number of orthogonalized atomic orbitals = 510
 Maximum deviation from orthogonality = 3.688E-11
 Guess MOs from SCF MO coefficient file
 Reading MOs from coefficient file
 Reading MOs from coefficient file

--------------------------------------------------
-  Entering scfman on Sun Oct 13 19:48:41 2024  -
--------------------------------------------------

 Long-range K will be added via erf
 Coulomb attenuation parameter = 0.2 bohr**(-1)
 A restricted hybrid HF-DFT SCF calculation will be
 performed using Pulay DIIS + Geometric Direct Minimization
 Exchange:     0.2220 Hartree-Fock + 1.0000 wB97X-D + LR-HF
 Correlation:  1.0000 wB97X-D
 Using Euler-Maclaurin-Lebedev (75,302) quadrature formula
 Dispersion:   Grimme D
 SCF converges when RMS gradient is below 1.0E-07
 Geometry optimization detected.  Setting ReadMinima to 0
 Setting SaveMinima to 0
 ---------------------------------------
  Cycle       Energy         DIIS Error
 ---------------------------------------
```

```
    1    -763.9834524394      4.77E-04
    2    -763.9631415043      1.13E-04
    3    -763.9633382167      1.10E-04
    4    -763.9636865581      1.65E-05
    5    -763.9636989411      7.72E-06
    6    -763.9637013300      3.35E-06
    7    -763.9637018532      1.03E-06
    8    -763.9637019027      4.46E-07
    9    -763.9637019134      1.31E-07
   10    -763.9637019145      6.25E-08 Convergence criterion met
 ---------------------------------------
 SCF time:  CPU 369.05 s  wall 623.13 s
 SCF   energy in the final basis set = -763.96370191
 Total energy in the final basis set = -763.96370191

-------------------------------------------------
-  Entering anlman on Sun Oct 13 19:59:04 2024  -
-------------------------------------------------


 --------------------------------------------------------------

                   Orbital Energies (a.u.)
 --------------------------------------------------------------

 Alpha MOs
 -- Occupied --
-14.8172 -14.7984 -14.7270 -14.7133 -10.6765 -10.6670 -10.6524 -10.6394
-10.6351 -10.6264 -10.6089 -10.5838 -10.5722 -10.5711 -10.5483 -10.5301
-10.4993 -10.4762  -1.4210  -1.4046  -1.3450  -1.2753  -1.2309  -1.2014
 -1.1879  -1.1450  -1.1039  -1.0835  -1.0573  -1.0545  -1.0333  -1.0097
 -0.9902  -0.9535  -0.9474  -0.9125  -0.9047  -0.8942  -0.8809  -0.8644
 -0.8516  -0.8475  -0.8355  -0.8223  -0.8093  -0.7978  -0.7902  -0.7813
 -0.7777  -0.7541  -0.7528  -0.7493  -0.7402  -0.7293  -0.7250  -0.7166
 -0.7054  -0.6722  -0.6594  -0.6473  -0.6410  -0.6328  -0.6270  -0.6210
 -0.6165
 -- Virtual --
 -0.2701  -0.2680  -0.2501  -0.2268  -0.1749  -0.1635  -0.1511  -0.1467
 -0.1338  -0.1224  -0.1176  -0.1164  -0.1094  -0.1050  -0.1009  -0.0956
 -0.0915  -0.0847  -0.0845  -0.0794  -0.0744  -0.0709  -0.0696  -0.0650
 -0.0636  -0.0612  -0.0567  -0.0539  -0.0498  -0.0446  -0.0404  -0.0387
 -0.0318  -0.0268  -0.0250  -0.0232  -0.0208  -0.0179  -0.0129  -0.0090
 -0.0067  -0.0030  -0.0018   0.0051   0.0092   0.0110   0.0121   0.0139
  0.0174   0.0184   0.0212   0.0306   0.0316   0.0376   0.0385   0.0391
  0.0417   0.0446   0.0466   0.0525   0.0538   0.0595   0.0617   0.0635
  0.0673   0.0691   0.0730   0.0736   0.0764   0.0837   0.0851   0.0884
  0.0958   0.0962   0.1012   0.1056   0.1091   0.1102   0.1163   0.1181
  0.1201   0.1232   0.1260   0.1304   0.1320   0.1389   0.1430   0.1454
  0.1532   0.1543   0.1583   0.1610   0.1672   0.1683   0.1717   0.1735
  0.1783   0.1842   0.1897   0.1931   0.1991   0.2099   0.2117   0.2197
  0.2255   0.2354   0.2473   0.2650   0.2718   0.2856   0.2936   0.3074
  0.3210   0.3278   0.3337   0.3375   0.3485   0.3512   0.3539   0.3695
  0.3799   0.3813   0.3879   0.3918   0.3966   0.4069   0.4206   0.4263
  0.4317   0.4395   0.4409   0.4463   0.4592   0.4666   0.4714   0.4754
  0.4782   0.4917   0.4955   0.5018   0.5077   0.5117   0.5149   0.5236
  0.5297   0.5333   0.5366   0.5416   0.5470   0.5504   0.5560   0.5588
  0.5629   0.5642   0.5741   0.5782   0.5807   0.5863   0.5890   0.5985
  0.6001   0.6114   0.6182   0.6285   0.6368   0.6408   0.6545   0.6592
  0.6653   0.6710   0.6773   0.6798   0.6892   0.6970   0.6996   0.7102
  0.7148   0.7262   0.7334   0.7399   0.7581   0.7686   0.7770   0.7836
  0.7923   0.7974   0.8091   0.8194   0.8253   0.8352   0.8363   0.8502
  0.8596   0.8649   0.8772   0.8872   0.9005   0.9086   0.9170   0.9314
  0.9347   0.9433   0.9589   0.9812   0.9951   1.0113   1.0210   1.0446
  1.0557   1.0641   1.0788   1.1027   1.1136   1.1436   1.1651   1.1807
  1.2034   1.2203   1.2368   1.2480   1.2555   1.2837   1.3043   1.3173
  1.3247   1.3285   1.3359   1.3433   1.3545   1.3633   1.3734   1.3791
  1.3883   1.3971   1.4055   1.4123   1.4157   1.4282   1.4315   1.4365
  1.4374   1.4449   1.4544   1.4653   1.4719   1.4777   1.4879   1.4921
  1.5023   1.5114   1.5156   1.5186   1.5336   1.5407   1.5485   1.5596
  1.5614   1.5645   1.5711   1.5838   1.5846   1.5927   1.6072   1.6131
```

| | | | | | | | |
|---|---|---|---|---|---|---|---|
| 1.6182 | 1.6306 | 1.6489 | 1.6620 | 1.6648 | 1.6701 | 1.6766 | 1.6905 |
| 1.7005 | 1.7023 | 1.7179 | 1.7366 | 1.7453 | 1.7492 | 1.7571 | 1.7647 |
| 1.7820 | 1.7913 | 1.7968 | 1.8217 | 1.8383 | 1.8546 | 1.8688 | 1.8852 |
| 1.8967 | 1.9009 | 1.9028 | 1.9180 | 1.9364 | 1.9495 | 1.9534 | 1.9800 |
| 1.9944 | 2.0005 | 2.0218 | 2.0383 | 2.0411 | 2.0582 | 2.0614 | 2.0817 |
| 2.1057 | 2.1178 | 2.1237 | 2.1472 | 2.1607 | 2.1834 | 2.2060 | 2.2140 |
| 2.2262 | 2.2296 | 2.2498 | 2.2528 | 2.2599 | 2.2876 | 2.2953 | 2.3034 |
| 2.3161 | 2.3334 | 2.3500 | 2.3562 | 2.3654 | 2.3815 | 2.3892 | 2.3957 |
| 2.4110 | 2.4164 | 2.4222 | 2.4357 | 2.4500 | 2.4567 | 2.4729 | 2.4934 |
| 2.5064 | 2.5159 | 2.5250 | 2.5367 | 2.5421 | 2.5435 | 2.5516 | 2.5592 |
| 2.5682 | 2.5747 | 2.5798 | 2.5860 | 2.5941 | 2.6038 | 2.6112 | 2.6213 |
| 2.6341 | 2.6429 | 2.6587 | 2.6621 | 2.6678 | 2.6758 | 2.6891 | 2.7040 |
| 2.7079 | 2.7170 | 2.7236 | 2.7349 | 2.7449 | 2.7583 | 2.7727 | 2.7788 |
| 2.7908 | 2.8032 | 2.8065 | 2.8237 | 2.8460 | 2.8553 | 2.8641 | 2.8870 |
| 2.8997 | 2.9246 | 2.9443 | 2.9898 | 3.0313 | 3.0713 | 3.1366 | 3.1485 |
| 3.1599 | 3.1912 | 3.2427 | 3.2520 | 3.2951 | 3.3178 | 3.3601 | 3.3876 |
| 3.4173 | 3.4784 | 3.5158 | 3.5337 | 3.5521 | 3.5916 | 3.6214 | 3.6469 |
| 3.6869 | 3.6998 | 3.7130 | 3.7191 | 3.7375 | 3.7483 | 3.7594 | 3.7725 |
| 3.8319 | 3.8670 | 3.8715 | 3.9050 | 3.9359 | 3.9966 | 4.0330 | 4.0916 |
| 4.0943 | 4.2002 | 4.2987 | 4.3124 | 4.5864 | 4.6566 | 4.6961 | 4.7241 |
| 4.8976 | 5.0346 | 5.1847 | 23.5217 | 23.5719 | 23.7160 | 23.7237 | 23.7394 |
| 23.7757 | 23.7975 | 23.8353 | 23.8570 | 23.8926 | 23.9096 | 23.9643 | 23.9696 |
| 24.0350 | 35.4192 | 35.4491 | 35.5062 | 35.6035 | | | |

Ground-State Mulliken Net Atomic Charges

| Atom | Charge (a.u.) |
|---|---|
| 1 N | 0.441486 |
| 2 C | 0.082302 |
| 3 C | -0.033448 |
| 4 C | -0.224691 |
| 5 C | -0.290035 |
| 6 C | -0.073149 |
| 7 C | -0.724766 |
| 8 C | -0.196824 |
| 9 N | -0.159353 |
| 10 C | -0.186450 |
| 11 N | 0.041856 |
| 12 C | 0.123174 |
| 13 C | -0.062042 |
| 14 C | 0.107992 |
| 15 C | -0.331385 |
| 16 C | -0.241965 |
| 17 C | -0.536744 |
| 18 N | -0.462587 |
| 19 H | 0.220681 |
| 20 H | 0.235586 |
| 21 H | 0.231697 |
| 22 H | 0.217655 |
| 23 H | 0.232559 |
| 24 H | 0.215620 |
| 25 H | 0.241803 |
| 26 H | 0.198696 |
| 27 H | 0.246003 |
| 28 H | 0.388262 |
| 29 H | 0.215454 |
| 30 H | 0.211340 |
| 31 H | 0.253577 |
| 32 H | 0.201172 |
| 33 H | 0.224725 |
| 34 H | 0.126648 |
| 35 H | 0.216342 |
| 36 H | 0.181435 |
| 37 H | 0.314577 |
| 38 H | 0.352796 |

Sum of atomic charges = 2.000000

```
 -----------------------------------------------------------------
                    Cartesian Multipole Moments
 -----------------------------------------------------------------
    Charge (ESU x 10^10)
                 9.6064
    Dipole Moment (Debye)
         X       6.2202      Y       2.5977      Z       3.6799
       Tot       7.7798
    Quadrupole Moments (Debye-Ang)
        XX     -14.3690     XY      -7.6633     YY     -71.1757
        XZ       6.0404     YZ      -6.0162     ZZ     -72.5181
    Traceless Quadrupole Moments (Debye-Ang)
       QXX     114.9559    QYY     -55.4643    QZZ     -59.4915
       QXY     -22.9900    QXZ      18.1212    QYZ     -18.0485
    Octopole Moments (Debye-Ang^2)
       XXX       8.7826    XXY    -101.7627    XYY      19.0772
       YYY      -1.0501    XXZ      52.2143    XYZ      -0.9173
       YYZ     -36.7082    XZZ      15.9944    YZZ      -3.8005
       ZZZ      -1.5390
    Traceless Octopole Moments (Debye-Ang^2)
       XXX    -262.9489    YYY     943.7680    ZZZ    -148.7889
       XXY   -1206.6000    XXZ     741.3135    XYY     154.5958
       XYZ     -13.7592    XZZ     108.3531    YYZ    -592.5246
       YZZ     262.8319
    Hexadecapole Moments (Debye-Ang^3)
      XXXX   -3549.9432   XXXY    -172.0681   XXYY    -769.2321
      XYYY      78.0824   YYYY    -955.2476   XXXZ      35.4271
      XXYZ      -1.4364   XYYZ     -31.8500   YYYZ     -14.9964
      XXZZ    -730.7776   XYZZ       9.1611   YYZZ    -262.3693
      XZZZ    -142.8054   YZZZ      37.1721   ZZZZ    -712.7147
    Traceless Hexadecapole Moments (Debye-Ang^3)
      XXXX    3067.7523   XXXY  -14250.0423   XXXZ    9985.1199
      XXYY   -1445.3326   XXYZ    -459.3329   XXZZ   -1622.4197
      XYYY   12015.7544   XYYZ   -1255.8251   XYZZ    2234.2879
      XZZZ   -8729.2948   YYYY    -168.5604   YYYZ   -2500.1438
      YYZZ    1613.8929   YZZZ    2959.4767   ZZZZ       8.5268
 -----------------------------------------------------------------

--------------------------------------------------
-  Entering drvman on Sun Oct 13 19:59:04 2024  -
--------------------------------------------------

 Calculating analytic gradient of the SCF energy
 Gradient of SCF Energy
            1           2           3           4           5           6
    1  -0.0009321   0.0011877  -0.0012199   0.0001487  -0.0006625   0.0005924
    2  -0.0011336   0.0008526   0.0000919  -0.0001145   0.0005974   0.0002589
    3   0.0001742   0.0007294  -0.0010225   0.0005202  -0.0000203  -0.0008191
            7           8           9          10          11          12
    1   0.0001681  -0.0001860   0.0008779   0.0020460  -0.0006821  -0.0028307
    2  -0.0002759   0.0001142   0.0010938  -0.0018220   0.0026432  -0.0007861
    3  -0.0005216  -0.0011243  -0.0022982   0.0039985  -0.0021758   0.0084799
           13          14          15          16          17          18
    1  -0.0027886  -0.0003098   0.0006780  -0.0006852  -0.0002831   0.0115403
    2   0.0001793  -0.0009376  -0.0008319   0.0021840  -0.0007518  -0.0038765
    3  -0.0038524   0.0018441  -0.0009597   0.0008095  -0.0002811  -0.0061694
           19          20          21          22          23          24
    1   0.0003222   0.0001199   0.0003198   0.0006753   0.0002854  -0.0000053
    2  -0.0004512   0.0000071  -0.0004059  -0.0005375   0.0002700   0.0000184
    3   0.0001693   0.0001195  -0.0001452   0.0004665   0.0000854  -0.0002071
           25          26          27          28          29          30
    1   0.0002288  -0.0004434  -0.0005272  -0.0007580  -0.0004936  -0.0002963
    2  -0.0004257   0.0005389   0.0000335  -0.0003444   0.0000469   0.0000903
    3  -0.0004484   0.0004715   0.0003165   0.0000393   0.0005641   0.0000634
           31          32          33          34          35          36
    1  -0.0007096   0.0004914   0.0000139   0.0000296  -0.0001211   0.0000601
    2  -0.0000721  -0.0007097  -0.0004358   0.0001463   0.0000739   0.0002370
    3   0.0005733  -0.0000852  -0.0002665   0.0001015  -0.0000632   0.0001694
           37          38
    1  -0.0059116   0.0000608
```

```
   2   0.0042025   0.0002321
   3  -0.0004116   0.0011759
 Max gradient component =       1.154E-02
 RMS gradient           =       1.914E-03
 Gradient time:  CPU 160.91 s  wall 268.63 s

-------------------------------------------------
-  Entering optman on Sun Oct 13 20:03:33 2024  -
-------------------------------------------------

 Geometry Optimization Parameters
   NAtoms,    NIC,     NZ,  NCons,   NDum,   NFix, NCnnct, MaxDiis
       38     272       0       0       0       0       0       0

 Cartesian Hessian Update
 Hessian updated using BFGS update


** GEOMETRY OPTIMIZATION IN DELOCALIZED INTERNAL COORDINATES **
   Searching for a Minimum

   Optimization Cycle:   3

                       Coordinates (Angstroms)
     ATOM                X               Y                Z
      1  N         2.5413181479    0.4034274526    0.4968614042
      2  C         2.7281118977   -0.4133953411   -0.5795820511
      3  C         3.5713082437   -1.5071470032   -0.4359516678
      4  C         4.2262868564   -1.7543084965    0.7594832229
      5  C         4.0383808728   -0.8843896835    1.8301799836
      6  C         3.1976764858    0.1845886373    1.6669313516
      7  C         2.0317306837   -0.1416843262   -1.8744397941
      8  C         1.6649288020    1.6003793487    0.4138425096
      9  N        -1.7229641999    0.2580981239    1.3063543394
     10  C        -2.4961987483    0.6824369397    0.2762934615
     11  N        -1.9786630003    1.4125276007   -0.6845968900
     12  C        -0.6851661898    1.7603902369   -0.6398435427
     13  C         0.1848270493    1.2798755101    0.3857465374
     14  C        -0.4021178858    0.5360789649    1.3676002326
     15  C        -3.9329321225    0.2929109310    0.2396775795
     16  C        -4.1691359040   -0.9240420066   -0.6796886750
     17  C        -3.5482835022   -2.2130570481   -0.1498582357
     18  N        -0.2311564596    2.5029483192   -1.6793772775
     19  H         3.7215042351   -2.1605888387   -1.2859405397
     20  H         4.8881538672   -2.6074092760    0.8544055277
     21  H         4.5482492205   -1.0261354257    2.7741075121
     22  H         3.0319134422    0.9020643938    2.4598379716
     23  H         2.4706397881   -0.7604084065   -2.6547314851
     24  H         2.1166990418    0.8992698659   -2.1906300273
     25  H         0.9689405051   -0.3919563769   -1.8102831110
     26  H         1.9570410239    2.1680030182   -0.4668026789
     27  H         1.8966852820    2.2232629782    1.2782187491
     28  H        -2.1588603504   -0.2957714909    2.0359984375
     29  H         0.1317122259    0.1281455490    2.2181205372
     30  H        -4.2880795265    0.0693806266    1.2508379115
     31  H        -4.4961529944    1.1528838221   -0.1267987336
     32  H        -3.7913434357   -0.6994141965   -1.6810291260
     33  H        -5.2497284983   -1.0458006723   -0.7740870019
     34  H        -2.4529537337   -2.1637664370   -0.1186878394
     35  H        -3.8094812300   -3.0549772664   -0.7919958132
     36  H        -3.9118351053   -2.4457944172    0.8560406415
     37  H         0.3765125907    3.2931082098   -1.5112129375
     38  H        -0.9675673754    2.7402661806   -2.3350004829
   Point Group: c1    Number of degrees of freedom:   108


   Energy is   -763.963701914

 Hessian updated using BFGS update
  internal optimization (0)
```

108 Hessian modes will be used to form the next step
Hessian Eigenvalues:

| | | | | | |
|---|---|---|---|---|---|
| 0.002189 | 0.002585 | 0.003953 | 0.005615 | 0.009364 | 0.010181 |
| 0.014874 | 0.018735 | 0.019316 | 0.019861 | 0.020982 | 0.022367 |
| 0.022649 | 0.022909 | 0.023974 | 0.024412 | 0.025700 | 0.026659 |
| 0.028119 | 0.028453 | 0.028549 | 0.029992 | 0.034126 | 0.036640 |
| 0.038779 | 0.042109 | 0.043211 | 0.043903 | 0.045241 | 0.046966 |
| 0.052389 | 0.053696 | 0.054772 | 0.055363 | 0.062482 | 0.078953 |
| 0.085441 | 0.094067 | 0.121663 | 0.122191 | 0.127096 | 0.131175 |
| 0.131685 | 0.132953 | 0.136257 | 0.139268 | 0.142896 | 0.144592 |
| 0.147686 | 0.148278 | 0.149521 | 0.152379 | 0.152868 | 0.153239 |
| 0.154128 | 0.159771 | 0.193183 | 0.206093 | 0.209935 | 0.217016 |
| 0.221221 | 0.230538 | 0.237894 | 0.247083 | 0.250016 | 0.261020 |
| 0.267048 | 0.272079 | 0.280423 | 0.298082 | 0.300178 | 0.300265 |
| 0.300609 | 0.301213 | 0.301720 | 0.302677 | 0.304120 | 0.304795 |
| 0.305256 | 0.305558 | 0.307499 | 0.308078 | 0.312506 | 0.317297 |
| 0.321807 | 0.331746 | 0.332474 | 0.334407 | 0.336982 | 0.341789 |
| 0.342128 | 0.351512 | 0.360197 | 0.362910 | 0.380784 | 0.390557 |
| 0.391497 | 0.403279 | 0.412035 | 0.416997 | 0.421271 | 0.422135 |
| 0.441528 | 0.442880 | 0.461869 | 0.484641 | 0.574694 | 0.780055 |

Minimum search - taking simple RFO step
Searching for Lamda that Minimizes Along All modes
Value Taken    Lamda =  -0.00536547
Calculated Step too Large.  Step scaled by  0.539109 !!
Step Taken.  Stepsize is  0.300000  0.012735

| | Maximum | Tolerance | Cnvgd? |
|---|---|---|---|
| Gradient | 0.004180 | 0.000800 | NO |
| Displacement | 0.081903 | 0.001400 | NO |
| Energy change | -0.003707 | 0.000228 | NO |

New Cartesian Coordinates Obtained by Inverse Iteration

Displacement from previous Coordinates is:  0.538846

Standard Nuclear Orientation (Angstroms)

| I | Atom | X | Y | Z |
|---|---|---|---|---|
| 1 | N | 2.5415204146 | 0.4039255172 | 0.4960820264 |
| 2 | C | 2.7091111442 | -0.4323049338 | -0.5675381079 |
| 3 | C | 3.5553395132 | -1.5216578150 | -0.4165977343 |
| 4 | C | 4.2223728741 | -1.7509890119 | 0.7753741026 |
| 5 | C | 4.0431911493 | -0.8672309620 | 1.8375326625 |
| 6 | C | 3.2031215720 | 0.2008347726 | 1.6655463737 |
| 7 | C | 1.9873472446 | -0.1757363089 | -1.8503659798 |
| 8 | C | 1.6701709939 | 1.6006969074 | 0.3999837323 |
| 9 | N | -1.7133561242 | 0.2485778364 | 1.2953257140 |
| 10 | C | -2.4981718524 | 0.6886336800 | 0.2769307275 |
| 11 | N | -1.9881585842 | 1.4328968739 | -0.6696233281 |
| 12 | C | -0.6948779426 | 1.7860558183 | -0.6355333843 |
| 13 | C | 0.1902700685 | 1.2823781672 | 0.3736811086 |
| 14 | C | -0.3900874814 | 0.5246244251 | 1.3433319777 |
| 15 | C | -3.9307592696 | 0.2894238870 | 0.2432198165 |
| 16 | C | -4.1577757441 | -0.9241479592 | -0.6873327833 |
| 17 | C | -3.5127226089 | -2.2084821504 | -0.1773730023 |
| 18 | N | -0.2734850735 | 2.5551127742 | -1.6512285112 |
| 19 | H | 3.6932180994 | -2.1850413528 | -1.2603033690 |
| 20 | H | 4.8843316317 | -2.6031956680 | 0.8750078686 |
| 21 | H | 4.5567555357 | -0.9942731201 | 2.7811574323 |
| 22 | H | 3.0396889356 | 0.9331822261 | 2.4450543175 |
| 23 | H | 2.3895372542 | -0.8229400971 | -2.6271514476 |
| 24 | H | 2.0867265289 | 0.8564146096 | -2.1907895436 |
| 25 | H | 0.9207233248 | -0.3971367596 | -1.7513526546 |
| 26 | H | 1.9646251464 | 2.1521239185 | -0.4905658446 |
| 27 | H | 1.9087705052 | 2.2329832652 | 1.2549033076 |
| 28 | H | -2.1373073893 | -0.3177597799 | 2.0209673099 |
| 29 | H | 0.1553623473 | 0.0987855938 | 2.1774874320 |

```
  30      H      -4.2778773396     0.0529031855     1.2537640622
  31      H      -4.4997160439     1.1478620998    -0.1167191204
  32      H      -3.7958983562    -0.6779207150    -1.6891596912
  33      H      -5.2378404774    -1.0565220648    -0.7706410537
  34      H      -2.4179272274    -2.1453790823    -0.1570717677
  35      H      -3.7676027671    -3.0479435927    -0.8251361034
  36      H      -3.8630652590    -2.4589037533     0.8288729876
  37      H       0.4451158031     3.2520783736    -1.5287241282
  38      H      -1.0106705463     2.8480711955    -2.2810154042
 ----------------------------------------------------------------
 Nuclear Repulsion Energy =        1322.76266440 hartrees
 There are       65 alpha and       65 beta electrons

--------------------------------------------------
-  Entering fldman on Sun Oct 13 20:03:33 2024  -
--------------------------------------------------

 Applying Cartesian multipole field
    Component          Value
    ---------          -----
     (2,0,0)        1.00000E-12
     (0,2,0)        2.00000E-11
     (0,0,2)       -3.00000E-11
 Nucleus-field energy     =     0.0000000029 hartrees

--------------------------------------------------
-  Entering gesman on Sun Oct 13 20:03:33 2024  -
--------------------------------------------------

 Requested basis set is 6-311+G(d,p)
 There are 188 shells and 516 basis functions
 A cutoff of  1.0D-12 yielded  13075 shell pairs
 There are    103395 function pairs (    110212 Cartesian)
 Smallest overlap matrix eigenvalue = 2.08E-06
 Linear dependence detected in AO basis
 Tighter screening thresholds may be required for diffuse basis sets
 Use S2THRESH > 12 and THRESH = 14 in case of SCF convergence issues
 Number of orthogonalized atomic orbitals = 510
 Maximum deviation from orthogonality = 4.280E-11
 Guess MOs from SCF MO coefficient file
 Reading MOs from coefficient file
 Reading MOs from coefficient file

--------------------------------------------------
-  Entering scfman on Sun Oct 13 20:03:33 2024  -
--------------------------------------------------

 Long-range K will be added via erf
 Coulomb attenuation parameter = 0.2 bohr**(-1)
 A restricted hybrid HF-DFT SCF calculation will be
 performed using Pulay DIIS + Geometric Direct Minimization
 Exchange:     0.2220 Hartree-Fock + 1.0000 wB97X-D + LR-HF
 Correlation:  1.0000 wB97X-D
 Using Euler-Maclaurin-Lebedev (75,302) quadrature formula
 Dispersion:   Grimme D
 SCF converges when RMS gradient is below 1.0E-07
 Geometry optimization detected.  Setting ReadMinima to 0
 Setting SaveMinima to 0
 ---------------------------------------
  Cycle       Energy         DIIS Error
 ---------------------------------------
    1    -763.9751350819      5.26E-04
    2    -763.9655948262      9.18E-05
    3    -763.9658670174      7.72E-05
    4    -763.9660446295      2.14E-05
    5    -763.9660634306      6.27E-06
    6    -763.9660651924      3.10E-06
    7    -763.9660656084      9.50E-07
    8    -763.9660656566      3.96E-07
    9    -763.9660656654      1.14E-07
```

```
   10    -763.9660656663      5.65E-08 Convergence criterion met
 ---------------------------------------
 SCF time:  CPU 366.48 s  wall 609.27 s
 SCF   energy in the final basis set = -763.96606567
 Total energy in the final basis set = -763.96606567

--------------------------------------------------
-  Entering anlman on Sun Oct 13 20:13:42 2024  -
--------------------------------------------------



 --------------------------------------------------------------

                    Orbital Energies (a.u.)
 --------------------------------------------------------------

 Alpha MOs
 -- Occupied --
-14.8176 -14.7943 -14.7315 -14.7101 -10.6767 -10.6644 -10.6498 -10.6401
-10.6360 -10.6271 -10.6075 -10.5848 -10.5732 -10.5720 -10.5505 -10.5285
-10.4987 -10.4760  -1.4187  -1.4061  -1.3468  -1.2761  -1.2316  -1.2012
 -1.1894  -1.1443  -1.1036  -1.0851  -1.0558  -1.0548  -1.0345  -1.0092
 -0.9910  -0.9541  -0.9474  -0.9147  -0.9058  -0.8953  -0.8820  -0.8642
 -0.8534  -0.8475  -0.8361  -0.8233  -0.8092  -0.7989  -0.7908  -0.7812
 -0.7784  -0.7545  -0.7543  -0.7506  -0.7435  -0.7303  -0.7241  -0.7185
 -0.7048  -0.6719  -0.6600  -0.6466  -0.6400  -0.6323  -0.6257  -0.6221
 -0.6141
 -- Virtual --
 -0.2706  -0.2642  -0.2473  -0.2274  -0.1744  -0.1641  -0.1505  -0.1465
 -0.1348  -0.1223  -0.1174  -0.1164  -0.1093  -0.1053  -0.1008  -0.0965
 -0.0917  -0.0853  -0.0842  -0.0794  -0.0742  -0.0702  -0.0698  -0.0656
 -0.0639  -0.0605  -0.0568  -0.0546  -0.0496  -0.0452  -0.0407  -0.0386
 -0.0321  -0.0266  -0.0255  -0.0222  -0.0200  -0.0174  -0.0124  -0.0085
 -0.0075  -0.0026  -0.0014   0.0047   0.0085   0.0105   0.0120   0.0139
  0.0171   0.0194   0.0210   0.0294   0.0324   0.0375   0.0388   0.0397
  0.0419   0.0446   0.0469   0.0530   0.0541   0.0591   0.0622   0.0633
  0.0680   0.0698   0.0733   0.0738   0.0761   0.0838   0.0846   0.0883
  0.0958   0.0969   0.1006   0.1070   0.1093   0.1106   0.1172   0.1179
  0.1191   0.1235   0.1268   0.1311   0.1324   0.1390   0.1443   0.1459
  0.1532   0.1556   0.1584   0.1601   0.1676   0.1691   0.1727   0.1747
  0.1797   0.1867   0.1893   0.1954   0.2008   0.2112   0.2126   0.2203
  0.2246   0.2361   0.2487   0.2649   0.2718   0.2823   0.2941   0.3069
  0.3222   0.3284   0.3322   0.3380   0.3484   0.3517   0.3539   0.3720
  0.3809   0.3818   0.3903   0.3916   0.3993   0.4071   0.4211   0.4259
  0.4315   0.4401   0.4408   0.4465   0.4593   0.4664   0.4711   0.4749
  0.4795   0.4908   0.4967   0.5031   0.5100   0.5119   0.5179   0.5275
  0.5299   0.5335   0.5377   0.5404   0.5476   0.5510   0.5554   0.5601
  0.5617   0.5656   0.5760   0.5801   0.5853   0.5884   0.5893   0.5982
  0.6000   0.6104   0.6184   0.6284   0.6368   0.6422   0.6552   0.6596
  0.6642   0.6714   0.6774   0.6797   0.6888   0.6960   0.7040   0.7104
  0.7157   0.7268   0.7314   0.7394   0.7562   0.7672   0.7775   0.7847
  0.7926   0.7953   0.8124   0.8236   0.8259   0.8348   0.8393   0.8522
  0.8611   0.8643   0.8777   0.8884   0.9013   0.9115   0.9183   0.9339
  0.9343   0.9480   0.9585   0.9802   0.9976   1.0140   1.0222   1.0462
  1.0587   1.0656   1.0800   1.1001   1.1136   1.1458   1.1628   1.1835
  1.2005   1.2230   1.2390   1.2431   1.2542   1.2815   1.3038   1.3161
  1.3210   1.3281   1.3355   1.3444   1.3554   1.3670   1.3742   1.3827
  1.3880   1.3977   1.4064   1.4079   1.4144   1.4266   1.4295   1.4366
  1.4371   1.4459   1.4565   1.4669   1.4742   1.4800   1.4891   1.4937
  1.5024   1.5124   1.5162   1.5188   1.5349   1.5409   1.5500   1.5590
  1.5600   1.5651   1.5732   1.5838   1.5857   1.5953   1.6080   1.6144
  1.6179   1.6298   1.6488   1.6572   1.6689   1.6710   1.6754   1.6857
  1.6973   1.7040   1.7228   1.7373   1.7457   1.7477   1.7560   1.7652
  1.7822   1.7920   1.7980   1.8201   1.8393   1.8528   1.8683   1.8866
  1.9003   1.9006   1.9080   1.9175   1.9451   1.9488   1.9555   1.9848
  1.9926   2.0032   2.0207   2.0367   2.0432   2.0521   2.0661   2.0811
  2.1020   2.1233   2.1243   2.1506   2.1615   2.1876   2.2087   2.2146
  2.2264   2.2293   2.2512   2.2522   2.2570   2.2852   2.2925   2.3037
  2.3153   2.3325   2.3480   2.3558   2.3641   2.3867   2.3891   2.3961
  2.4071   2.4156   2.4285   2.4337   2.4502   2.4551   2.4686   2.4940
```

```
 2.5069   2.5213   2.5238   2.5376   2.5428   2.5439   2.5477   2.5607
 2.5687   2.5779   2.5802   2.5866   2.5955   2.6018   2.6119   2.6239
 2.6383   2.6452   2.6562   2.6602   2.6722   2.6767   2.6864   2.7067
 2.7138   2.7171   2.7293   2.7379   2.7461   2.7634   2.7742   2.7795
 2.7881   2.8020   2.8104   2.8292   2.8466   2.8588   2.8688   2.8860
 2.8988   2.9261   2.9499   2.9904   3.0352   3.0692   3.1404   3.1583
 3.1649   3.2128   3.2444   3.2546   3.2969   3.3187   3.3635   3.3974
 3.4185   3.4816   3.5211   3.5350   3.5544   3.5934   3.6223   3.6440
 3.6894   3.6999   3.7159   3.7204   3.7401   3.7495   3.7618   3.7708
 3.8334   3.8379   3.8667   3.9037   3.9377   3.9899   4.0332   4.0915
 4.0938   4.2006   4.2974   4.3105   4.5908   4.6626   4.6997   4.7316
 4.9092   5.0356   5.1897  23.5214  23.5763  23.7153  23.7301  23.7392
23.7759  23.7991  23.8348  23.8590  23.8923  23.9109  23.9666  23.9797
24.0341  35.4193  35.4536  35.5138  35.6141
--------------------------------------------------------------

         Ground-State Mulliken Net Atomic Charges

    Atom                 Charge (a.u.)
 ----------------------------------------
     1 N                     0.436641
     2 C                     0.088254
     3 C                    -0.031639
     4 C                    -0.228280
     5 C                    -0.286955
     6 C                    -0.072514
     7 C                    -0.724934
     8 C                    -0.189213
     9 N                    -0.167165
    10 C                    -0.183875
    11 N                     0.033472
    12 C                     0.151564
    13 C                    -0.082104
    14 C                     0.108771
    15 C                    -0.329355
    16 C                    -0.240682
    17 C                    -0.540281
    18 N                    -0.462202
    19 H                     0.220258
    20 H                     0.236097
    21 H                     0.232126
    22 H                     0.217836
    23 H                     0.233952
    24 H                     0.214120
    25 H                     0.243795
    26 H                     0.188912
    27 H                     0.246166
    28 H                     0.386973
    29 H                     0.213637
    30 H                     0.211546
    31 H                     0.252496
    32 H                     0.201110
    33 H                     0.224557
    34 H                     0.126481
    35 H                     0.216130
    36 H                     0.181767
    37 H                     0.313216
    38 H                     0.359318
 ----------------------------------------
 Sum of atomic charges =     2.000000

-----------------------------------------------------------------
                   Cartesian Multipole Moments
-----------------------------------------------------------------
   Charge (ESU x 10^10)
                9.6064
   Dipole Moment (Debye)
        X       6.4691       Y       2.6615      Z       3.4596
      Tot       7.8039
   Quadrupole Moments (Debye-Ang)
```

```
       XX     -14.7691     XY      -7.4002     YY     -70.6624
       XZ       6.4388     YZ      -6.5988     ZZ     -72.7786
    Traceless Quadrupole Moments (Debye-Ang)
      QXX     113.9029    QYY     -53.7772    QZZ     -60.1257
      QXY     -22.2007    QXZ      19.3165    QYZ     -19.7964
    Octopole Moments (Debye-Ang^2)
      XXX      11.4526    XXY    -100.4266    XYY      19.7511
      YYY      -1.1841    XXZ      53.1293    XYZ      -0.7682
      YYZ     -38.8628    XZZ      16.9037    YZZ      -3.2478
      ZZZ      -2.7115
    Traceless Octopole Moments (Debye-Ang^2)
      XXX    -261.1771    YYY     925.9653    ZZZ    -144.6675
      XXY   -1191.8243    XXZ     762.2743    XYY     151.9440
      XYZ     -11.5231    XZZ     109.2331    YYZ    -617.6068
      YZZ     265.8590
    Hexadecapole Moments (Debye-Ang^3)
     XXXX   -3535.3806   XXXY    -165.1912   XXYY    -765.6784
     XYYY      80.5529   YYYY    -968.6826   XXXZ      35.7259
     XXYZ      -4.1014   XYYZ     -34.7123   YYYZ     -22.0134
     XXZZ    -725.1741   XYZZ      11.9992   YYZZ    -257.4303
     XZZZ    -150.2186   YZZZ      34.4486   ZZZZ    -706.5640
    Traceless Hexadecapole Moments (Debye-Ang^3)
     XXXX    2781.2854   XXXY  -14076.3191   XXXZ   10465.4470
     XXYY   -1247.4484   XXYZ    -548.9275   XXZZ   -1533.8371
     XYYY   11726.8169   XYYZ   -1406.7146   XYZZ    2349.5022
     XZZZ   -9058.7324   YYYY    -815.1909   YYYZ   -2666.2405
     YYZZ    2062.6392   YZZZ    3215.1680   ZZZZ    -528.8021
 -----------------------------------------------------------------

--------------------------------------------------
-  Entering drvman on Sun Oct 13 20:13:42 2024  -
--------------------------------------------------

 Calculating analytic gradient of the SCF energy
 Gradient of SCF Energy
           1           2           3           4           5           6
    1  -0.0001877   0.0006959  -0.0005748   0.0000573  -0.0004289   0.0004738
    2  -0.0006097  -0.0000391   0.0001074  -0.0003571   0.0006072  -0.0000615
    3  -0.0002901  -0.0001410  -0.0000296  -0.0004584   0.0004758   0.0000322
           7           8           9          10          11          12
    1   0.0004985   0.0001513   0.0003060   0.0001268   0.0003289  -0.0030138
    2   0.0002852  -0.0000088   0.0008083  -0.0000764   0.0001150   0.0022548
    3  -0.0001009   0.0000663  -0.0015114  -0.0000246   0.0010479   0.0033893
          13          14          15          16          17          18
    1  -0.0012689   0.0010866   0.0005353  -0.0004063  -0.0002261   0.0069502
    2  -0.0017502   0.0008381   0.0001467   0.0002655  -0.0002622  -0.0056003
    3  -0.0009523  -0.0001761  -0.0002155  -0.0000728   0.0000427  -0.0031825
          19          20          21          22          23          24
    1  -0.0000647  -0.0000637  -0.0000530   0.0001465   0.0001910  -0.0005363
    2   0.0002263   0.0000542   0.0000966  -0.0001910   0.0000564   0.0000436
    3   0.0001002   0.0000000  -0.0001368  -0.0000413   0.0002399   0.0001836
          25          26          27          28          29          30
    1   0.0000641  -0.0008285  -0.0000138  -0.0002885  -0.0001360   0.0000903
    2  -0.0000928   0.0001090  -0.0005574   0.0000373   0.0000140  -0.0000568
    3  -0.0002147  -0.0000997   0.0002240  -0.0001845   0.0002013  -0.0000122
          31          32          33          34          35          36
    1  -0.0001756   0.0002000   0.0001010   0.0000487   0.0000151   0.0000298
    2   0.0000085  -0.0002039   0.0000889  -0.0000294   0.0000598   0.0000468
    3   0.0003534   0.0000880  -0.0002043   0.0000526   0.0001008   0.0000418
          37          38
    1  -0.0041200   0.0002896
    2   0.0033853   0.0002416
    3   0.0001024   0.0013062
 Max gradient component =       6.950E-03
 RMS gradient           =       1.198E-03
 Gradient time:  CPU 163.17 s  wall 276.73 s

--------------------------------------------------
-  Entering optman on Sun Oct 13 20:18:19 2024  -
--------------------------------------------------
```

```
 Geometry Optimization Parameters
   NAtoms,    NIC,     NZ,  NCons,   NDum,   NFix, NCnnct, MaxDiis
       38     272       0       0       0       0       0       0

 Cartesian Hessian Update
 Hessian updated using BFGS update


** GEOMETRY OPTIMIZATION IN DELOCALIZED INTERNAL COORDINATES **
   Searching for a Minimum

   Optimization Cycle:   4

                     Coordinates (Angstroms)
     ATOM                X               Y               Z
      1  N         2.5415204146    0.4039255172    0.4960820264
      2  C         2.7091111442   -0.4323049338   -0.5675381079
      3  C         3.5553395132   -1.5216578150   -0.4165977343
      4  C         4.2223728741   -1.7509890119    0.7753741026
      5  C         4.0431911493   -0.8672309620    1.8375326625
      6  C         3.2031215720    0.2008347726    1.6655463737
      7  C         1.9873472446   -0.1757363089   -1.8503659798
      8  C         1.6701709939    1.6006969074    0.3999837323
      9  N        -1.7133561242    0.2485778364    1.2953257140
     10  C        -2.4981718524    0.6886336800    0.2769307275
     11  N        -1.9881585842    1.4328968739   -0.6696233281
     12  C        -0.6948779426    1.7860558183   -0.6355333843
     13  C         0.1902700685    1.2823781672    0.3736811086
     14  C        -0.3900874814    0.5246244251    1.3433319777
     15  C        -3.9307592696    0.2894238870    0.2432198165
     16  C        -4.1577757441   -0.9241479592   -0.6873327833
     17  C        -3.5127226089   -2.2084821504   -0.1773730023
     18  N        -0.2734850735    2.5551127742   -1.6512285112
     19  H         3.6932180994   -2.1850413528   -1.2603033690
     20  H         4.8843316317   -2.6031956680    0.8750078686
     21  H         4.5567555357   -0.9942731201    2.7811574323
     22  H         3.0396889356    0.9331822261    2.4450543175
     23  H         2.3895372542   -0.8229400971   -2.6271514476
     24  H         2.0867265289    0.8564146096   -2.1907895436
     25  H         0.9207233248   -0.3971367596   -1.7513526546
     26  H         1.9646251464    2.1521239185   -0.4905658446
     27  H         1.9087705052    2.2329832652    1.2549033076
     28  H        -2.1373073893   -0.3177597799    2.0209673099
     29  H         0.1553623473    0.0987855938    2.1774874320
     30  H        -4.2778773396    0.0529031855    1.2537640622
     31  H        -4.4997160439    1.1478620998   -0.1167191204
     32  H        -3.7958983562   -0.6779207150   -1.6891596912
     33  H        -5.2378404774   -1.0565220648   -0.7706410537
     34  H        -2.4179272274   -2.1453790823   -0.1570717677
     35  H        -3.7676027671   -3.0479435927   -0.8251361034
     36  H        -3.8630652590   -2.4589037533    0.8288729876
     37  H         0.4451158031    3.2520783736   -1.5287241282
     38  H        -1.0106705463    2.8480711955   -2.2810154042
   Point Group: c1    Number of degrees of freedom:   108


   Energy is   -763.966065666

 Hessian updated using BFGS update
  internal optimization (0)

108 Hessian modes will be used to form the next step
  Hessian Eigenvalues:
     0.002215    0.002584    0.003951    0.005649    0.009219    0.009486
     0.015034    0.018765    0.019316    0.019862    0.021091    0.022390
     0.022658    0.022914    0.024018    0.024418    0.025694    0.026662
     0.027008    0.028243    0.028459    0.029887    0.033567    0.036548
     0.038779    0.042162    0.043211    0.043879    0.045203    0.046507
     0.051855    0.053832    0.054700    0.055362    0.062787    0.079363
```

| | | | | | |
|---|---|---|---|---|---|
| 0.085493 | 0.094046 | 0.121666 | 0.122198 | 0.127112 | 0.131139 |
| 0.131847 | 0.132968 | 0.136430 | 0.140546 | 0.142476 | 0.143883 |
| 0.147686 | 0.148291 | 0.149737 | 0.152400 | 0.152862 | 0.153264 |
| 0.154240 | 0.160749 | 0.193438 | 0.206128 | 0.210052 | 0.216990 |
| 0.224471 | 0.230788 | 0.238179 | 0.247176 | 0.250046 | 0.261249 |
| 0.267632 | 0.272569 | 0.282204 | 0.298369 | 0.300192 | 0.300595 |
| 0.300636 | 0.301237 | 0.301761 | 0.302716 | 0.304120 | 0.304898 |
| 0.305264 | 0.305643 | 0.307496 | 0.308086 | 0.312530 | 0.317925 |
| 0.321669 | 0.331996 | 0.332790 | 0.334402 | 0.337039 | 0.341805 |
| 0.342186 | 0.351498 | 0.361599 | 0.362856 | 0.380735 | 0.390810 |
| 0.396355 | 0.406633 | 0.416696 | 0.420097 | 0.421007 | 0.426466 |
| 0.442830 | 0.447047 | 0.462008 | 0.494215 | 0.574638 | 0.787735 |

Minimum search - taking simple RFO step
Searching for Lamda that Minimizes Along All modes
Value Taken Lamda = -0.00305773
Calculated Step too Large. Step scaled by 0.613579
Step Taken. Stepsize is 0.300000

| | Maximum | Tolerance | Cnvgd? |
|---|---|---|---|
| Gradient | 0.002191 | 0.000800 | NO |
| Displacement | 0.077111 | 0.001400 | NO |
| Energy change | -0.002364 | 0.000228 | NO |

New Cartesian Coordinates Obtained by Inverse Iteration

Displacement from previous Coordinates is: 0.563599

Standard Nuclear Orientation (Angstroms)

| I | Atom | X | Y | Z |
|---|---|---|---|---|
| 1 | N | 2.5406675949 | 0.4066396324 | 0.4938568748 |
| 2 | C | 2.6912947294 | -0.4515556828 | -0.5546593870 |
| 3 | C | 3.5348910844 | -1.5409427452 | -0.3912401017 |
| 4 | C | 4.2110672984 | -1.7508604267 | 0.7994973616 |
| 5 | C | 4.0437377817 | -0.8484474407 | 1.8476625483 |
| 6 | C | 3.2077969819 | 0.2209299972 | 1.6626577485 |
| 7 | C | 1.9554083185 | -0.2098586815 | -1.8324065004 |
| 8 | C | 1.6743344124 | 1.6045067386 | 0.3801777327 |
| 9 | N | -1.7004933652 | 0.2328427924 | 1.2863857717 |
| 10 | C | -2.4991807592 | 0.6914395405 | 0.2839067577 |
| 11 | N | -2.0017911065 | 1.4553161286 | -0.6523088350 |
| 12 | C | -0.7071077653 | 1.8136430522 | -0.6314218963 |
| 13 | C | 0.1936752509 | 1.2874519890 | 0.3584934881 |
| 14 | C | -0.3765682856 | 0.5096490210 | 1.3194281219 |
| 15 | C | -3.9292437804 | 0.2826675011 | 0.2568685360 |
| 16 | C | -4.1526503076 | -0.9209257495 | -0.6887649114 |
| 17 | C | -3.4762276262 | -2.2023669439 | -0.2141789439 |
| 18 | N | -0.3151114020 | 2.6162514159 | -1.6218572624 |
| 19 | H | 3.6618562481 | -2.2202685246 | -1.2239162262 |
| 20 | H | 4.8701977077 | -2.6040058227 | 0.9086724341 |
| 21 | H | 4.5617232300 | -0.9617418103 | 2.7906077126 |
| 22 | H | 3.0510182092 | 0.9691940902 | 2.4284319490 |
| 23 | H | 2.3223735080 | -0.8904241393 | -2.5979495844 |
| 24 | H | 2.0846627454 | 0.8092522053 | -2.2027574488 |
| 25 | H | 0.8840149316 | -0.3923687626 | -1.7094974613 |
| 26 | H | 1.9751215095 | 2.1396950703 | -0.5182428286 |
| 27 | H | 1.9162468469 | 2.2497453501 | 1.2244499320 |
| 28 | H | -2.1111016177 | -0.3492748365 | 2.0065057714 |
| 29 | H | 0.1823764643 | 0.0675717770 | 2.1358898118 |
| 30 | H | -4.2656297045 | 0.0296032839 | 1.2669242370 |
| 31 | H | -4.5068114546 | 1.1408197750 | -0.0890344931 |
| 32 | H | -3.8158459653 | -0.6495477883 | -1.6927992460 |
| 33 | H | -5.2319700105 | -1.0692516275 | -0.7525984458 |
| 34 | H | -2.3824151688 | -2.1222873281 | -0.2153168856 |
| 35 | H | -3.7306708758 | -3.0351989349 | -0.8706743423 |
| 36 | H | -3.8019776011 | -2.4760505138 | 0.7942943588 |
| 37 | H | 0.4935534994 | 3.2124800309 | -1.5574383241 |
| 38 | H | -1.0512215566 | 2.9556783676 | -2.2276480237 |

```
 ------------------------------------------------------------------
 Nuclear Repulsion Energy =        1323.34779267 hartrees
 There are       65 alpha and       65 beta electrons

--------------------------------------------------
-  Entering fldman on Sun Oct 13 20:18:19 2024  -
--------------------------------------------------

 Applying Cartesian multipole field
    Component          Value
    ---------          -----
     (2,0,0)        1.00000E-12
     (0,2,0)        2.00000E-11
     (0,0,2)       -3.00000E-11
 Nucleus-field energy     =     0.0000000034 hartrees

--------------------------------------------------
-  Entering gesman on Sun Oct 13 20:18:19 2024  -
--------------------------------------------------

 Requested basis set is 6-311+G(d,p)
 There are 188 shells and 516 basis functions
 A cutoff of  1.0D-12 yielded  13093 shell pairs
 There are    103492 function pairs (    110319 Cartesian)
 Smallest overlap matrix eigenvalue = 2.09E-06
 Linear dependence detected in AO basis
 Tighter screening thresholds may be required for diffuse basis sets
 Use S2THRESH > 12 and THRESH = 14 in case of SCF convergence issues
 Number of orthogonalized atomic orbitals = 510
 Maximum deviation from orthogonality = 8.414E-12
 Guess MOs from SCF MO coefficient file
 Reading MOs from coefficient file
 Reading MOs from coefficient file

--------------------------------------------------
-  Entering scfman on Sun Oct 13 20:18:19 2024  -
--------------------------------------------------

 Long-range K will be added via erf
 Coulomb attenuation parameter = 0.2 bohr**(-1)
 A restricted hybrid HF-DFT SCF calculation will be
 performed using Pulay DIIS + Geometric Direct Minimization
 Exchange:     0.2220 Hartree-Fock + 1.0000 wB97X-D + LR-HF
 Correlation:  1.0000 wB97X-D
 Using Euler-Maclaurin-Lebedev (75,302) quadrature formula
 Dispersion:   Grimme D
 SCF converges when RMS gradient is below 1.0E-07
 Geometry optimization detected.  Setting ReadMinima to 0
 Setting SaveMinima to 0
 ---------------------------------------
  Cycle       Energy         DIIS Error
 ---------------------------------------
    1    -763.9605837045      5.42E-04
    2    -763.9670873484      8.02E-05
    3    -763.9674190166      5.03E-05
    4    -763.9674951654      2.40E-05
    5    -763.9675159426      5.00E-06
    6    -763.9675172145      2.46E-06
    7    -763.9675174645      8.33E-07
    8    -763.9675174994      3.24E-07
    9    -763.9675175051      9.21E-08 Convergence criterion met
 ---------------------------------------
 SCF time:  CPU 329.86 s  wall 544.42 s
 SCF   energy in the final basis set = -763.96751751
 Total energy in the final basis set = -763.96751751

--------------------------------------------------
-  Entering anlman on Sun Oct 13 20:27:24 2024  -
--------------------------------------------------
```

```
 --------------------------------------------------------------

                 Orbital Energies (a.u.)
 --------------------------------------------------------------

 Alpha MOs
 -- Occupied --
-14.8183 -14.7917 -14.7348 -14.7081 -10.6770 -10.6629 -10.6484 -10.6409
-10.6369 -10.6278 -10.6070 -10.5856 -10.5740 -10.5729 -10.5522 -10.5274
-10.4981 -10.4759  -1.4165  -1.4069  -1.3475  -1.2757  -1.2317  -1.2007
 -1.1904  -1.1437  -1.1032  -1.0863  -1.0551  -1.0543  -1.0353  -1.0088
 -0.9915  -0.9544  -0.9471  -0.9164  -0.9066  -0.8954  -0.8829  -0.8640
 -0.8548  -0.8474  -0.8363  -0.8240  -0.8092  -0.7997  -0.7912  -0.7810
 -0.7783  -0.7556  -0.7555  -0.7510  -0.7454  -0.7311  -0.7234  -0.7197
 -0.7042  -0.6713  -0.6606  -0.6461  -0.6389  -0.6317  -0.6247  -0.6231
 -0.6117
 -- Virtual --
 -0.2712  -0.2616  -0.2452  -0.2280  -0.1742  -0.1647  -0.1502  -0.1463
 -0.1355  -0.1222  -0.1172  -0.1164  -0.1093  -0.1056  -0.1011  -0.0973
 -0.0918  -0.0858  -0.0842  -0.0793  -0.0739  -0.0700  -0.0699  -0.0662
 -0.0640  -0.0598  -0.0570  -0.0551  -0.0500  -0.0461  -0.0411  -0.0386
 -0.0323  -0.0267  -0.0259  -0.0224  -0.0188  -0.0163  -0.0123  -0.0084
 -0.0074  -0.0023  -0.0012   0.0042   0.0074   0.0099   0.0116   0.0140
  0.0169   0.0202   0.0213   0.0282   0.0332   0.0372   0.0390   0.0403
  0.0420   0.0444   0.0472   0.0532   0.0543   0.0591   0.0627   0.0629
  0.0671   0.0708   0.0736   0.0737   0.0769   0.0834   0.0849   0.0879
  0.0950   0.0976   0.1006   0.1079   0.1089   0.1116   0.1168   0.1178
  0.1196   0.1239   0.1268   0.1315   0.1334   0.1393   0.1442   0.1460
  0.1532   0.1569   0.1585   0.1600   0.1671   0.1705   0.1721   0.1769
  0.1809   0.1875   0.1903   0.1955   0.2030   0.2109   0.2131   0.2221
  0.2257   0.2361   0.2497   0.2657   0.2727   0.2789   0.2951   0.3070
  0.3231   0.3290   0.3310   0.3382   0.3482   0.3520   0.3542   0.3732
  0.3814   0.3829   0.3901   0.3930   0.4016   0.4077   0.4213   0.4242
  0.4328   0.4405   0.4423   0.4468   0.4593   0.4658   0.4699   0.4745
  0.4797   0.4886   0.4971   0.5032   0.5104   0.5128   0.5201   0.5279
  0.5299   0.5333   0.5403   0.5410   0.5481   0.5506   0.5533   0.5600
  0.5625   0.5687   0.5776   0.5820   0.5857   0.5892   0.5977   0.5999
  0.6043   0.6095   0.6203   0.6276   0.6360   0.6446   0.6550   0.6607
  0.6640   0.6710   0.6771   0.6800   0.6889   0.6946   0.7073   0.7096
  0.7177   0.7258   0.7310   0.7402   0.7550   0.7658   0.7774   0.7842
  0.7923   0.7946   0.8156   0.8253   0.8283   0.8347   0.8416   0.8530
  0.8614   0.8655   0.8785   0.8896   0.9020   0.9147   0.9198   0.9335
  0.9366   0.9526   0.9591   0.9795   0.9994   1.0166   1.0229   1.0463
  1.0600   1.0659   1.0815   1.0942   1.1135   1.1473   1.1600   1.1849
  1.1987   1.2236   1.2345   1.2385   1.2557   1.2795   1.3023   1.3137
  1.3181   1.3272   1.3344   1.3450   1.3556   1.3699   1.3746   1.3831
  1.3873   1.3961   1.4045   1.4073   1.4173   1.4246   1.4283   1.4366
  1.4373   1.4467   1.4577   1.4686   1.4729   1.4815   1.4895   1.4955
  1.5026   1.5126   1.5161   1.5195   1.5360   1.5399   1.5515   1.5583
  1.5591   1.5656   1.5752   1.5843   1.5872   1.5956   1.6074   1.6134
  1.6188   1.6266   1.6472   1.6523   1.6695   1.6706   1.6733   1.6802
  1.6957   1.7040   1.7195   1.7371   1.7418   1.7490   1.7558   1.7653
  1.7821   1.7918   1.8016   1.8195   1.8395   1.8496   1.8667   1.8882
  1.9002   1.9018   1.9110   1.9180   1.9490   1.9520   1.9657   1.9889
  1.9936   2.0053   2.0196   2.0342   2.0453   2.0476   2.0691   2.0809
  2.0981   2.1246   2.1270   2.1537   2.1624   2.1899   2.2109   2.2156
  2.2267   2.2289   2.2499   2.2516   2.2549   2.2813   2.2898   2.3009
  2.3139   2.3306   2.3433   2.3556   2.3624   2.3881   2.3889   2.3927
  2.4056   2.4141   2.4313   2.4350   2.4480   2.4546   2.4659   2.4940
  2.5066   2.5216   2.5264   2.5355   2.5405   2.5440   2.5458   2.5618
  2.5697   2.5777   2.5797   2.5885   2.5953   2.6007   2.6125   2.6194
  2.6406   2.6459   2.6588   2.6643   2.6745   2.6764   2.6857   2.7075
  2.7163   2.7214   2.7313   2.7430   2.7465   2.7667   2.7735   2.7808
  2.7857   2.8018   2.8147   2.8341   2.8461   2.8652   2.8721   2.8844
  2.8958   2.9266   2.9565   2.9913   3.0369   3.0670   3.1404   3.1611
  3.1650   3.2282   3.2459   3.2618   3.2988   3.3187   3.3643   3.4040
  3.4185   3.4818   3.5231   3.5353   3.5559   3.5940   3.6205   3.6426
  3.6899   3.6998   3.7185   3.7206   3.7415   3.7496   3.7627   3.7686
  3.7998   3.8360   3.8660   3.9024   3.9387   3.9854   4.0318   4.0911
```

```
  4.0925   4.2017   4.2956   4.3083   4.5931   4.6656   4.6992   4.7365
  4.9177   5.0355   5.1916  23.5207  23.5790  23.7139  23.7310  23.7379
 23.7751  23.8000  23.8343  23.8592  23.8916  23.9116  23.9655  23.9772
 24.0330  35.4184  35.4569  35.5183  35.6187
--------------------------------------------------------------

         Ground-State Mulliken Net Atomic Charges

    Atom                 Charge (a.u.)
  ----------------------------------------
     1 N                     0.428309
     2 C                     0.105272
     3 C                    -0.031076
     4 C                    -0.228862
     5 C                    -0.283916
     6 C                    -0.071811
     7 C                    -0.735509
     8 C                    -0.179218
     9 N                    -0.167415
    10 C                    -0.189064
    11 N                     0.026622
    12 C                     0.184546
    13 C                    -0.106268
    14 C                     0.105686
    15 C                    -0.326365
    16 C                    -0.242140
    17 C                    -0.542134
    18 N                    -0.468772
    19 H                     0.220187
    20 H                     0.236438
    21 H                     0.232525
    22 H                     0.218532
    23 H                     0.234990
    24 H                     0.214309
    25 H                     0.245385
    26 H                     0.181450
    27 H                     0.246340
    28 H                     0.385675
    29 H                     0.212531
    30 H                     0.211792
    31 H                     0.251960
    32 H                     0.201784
    33 H                     0.224436
    34 H                     0.126293
    35 H                     0.216072
    36 H                     0.181564
    37 H                     0.314283
    38 H                     0.365570
  ----------------------------------------
  Sum of atomic charges =     2.000000

-----------------------------------------------------------------
                    Cartesian Multipole Moments
-----------------------------------------------------------------
    Charge (ESU x 10^10)
                9.6064
    Dipole Moment (Debye)
         X       6.6731      Y       2.6773      Z       3.2841
       Tot       7.9046
    Quadrupole Moments (Debye-Ang)
        XX     -15.2064     XY      -7.1682     YY     -70.2118
        XZ       6.7793     YZ      -7.1721     ZZ     -72.9786
    Traceless Quadrupole Moments (Debye-Ang)
       QXX     112.7777    QYY     -52.2385    QZZ     -60.5392
       QXY     -21.5045    QXZ      20.3379    QYZ     -21.5163
    Octopole Moments (Debye-Ang^2)
       XXX      13.4056    XXY     -99.0182    XYY      20.3176
       YYY      -1.7975    XXZ      54.2264    XYZ      -0.7402
       YYZ     -40.8349    XZZ      17.9336    YZZ      -2.8679
       ZZZ      -4.1314
```

```
    Traceless Octopole Moments (Debye-Ang^2)
       XXX    -263.8268    YYY     906.1899    ZZZ    -145.3118
       XXY   -1174.2223    XXZ     785.6159    XYY     149.7932
       XYZ     -11.1036    XZZ     114.0336    YYZ    -640.3041
       YZZ     268.0324
    Hexadecapole Moments (Debye-Ang^3)
      XXXX   -3523.5809   XXXY    -155.8425   XXYY    -762.4322
      XYYY      80.3150   YYYY    -984.9962   XXXZ      35.7554
      XXYZ      -6.7069   XYYZ     -36.2897   YYYZ     -28.4204
      XXZZ    -719.4711   XYZZ      15.0343   YYZZ    -253.3132
      XZZZ    -157.0151   YZZZ      32.0306   ZZZZ    -701.6381
    Traceless Hexadecapole Moments (Debye-Ang^3)
      XXXX    2391.7461   XXXY  -13641.2714   XXXZ   10844.0367
      XXYY   -1003.9384   XXYZ    -657.3129   XXZZ   -1387.8077
      XYYY   11155.2703   XYYZ   -1447.1727   XYZZ    2486.0011
      XZZZ   -9396.8640   YYYY   -1483.6870   YYYZ   -2843.4155
      YYZZ    2487.6254   YZZZ    3500.7284   ZZZZ   -1099.8177
 -----------------------------------------------------------------

--------------------------------------------------
-  Entering drvman on Sun Oct 13 20:27:24 2024  -
--------------------------------------------------

 Calculating analytic gradient of the SCF energy
 Gradient of SCF Energy
           1           2           3           4           5           6
    1   0.0002614   0.0002789  -0.0001857   0.0000089  -0.0002685   0.0004356
    2  -0.0003162  -0.0003036   0.0000284  -0.0002778   0.0004795  -0.0004049
    3  -0.0003879  -0.0003915   0.0003165  -0.0004457   0.0004119   0.0002801
           7           8           9          10          11          12
    1   0.0004035   0.0003708   0.0001123  -0.0013824   0.0007335  -0.0021965
    2   0.0004266   0.0001091   0.0002025  -0.0003946  -0.0005293   0.0039209
    3   0.0001206   0.0002202  -0.0002477  -0.0008561   0.0007820   0.0002561
          13          14          15          16          17          18
    1   0.0004806   0.0004349   0.0001031  -0.0001183  -0.0002023   0.0037979
    2  -0.0011346  -0.0000091   0.0004915  -0.0004109  -0.0001277  -0.0046171
    3  -0.0005410   0.0009079   0.0001483  -0.0002523   0.0001623  -0.0025008
          19          20          21          22          23          24
    1  -0.0001268  -0.0001084  -0.0001509  -0.0000795   0.0000423  -0.0006801
    2   0.0003361   0.0000833   0.0001814   0.0000111   0.0000444   0.0002553
    3   0.0000239  -0.0000364  -0.0000834  -0.0002127   0.0002242   0.0002389
          25          26          27          28          29          30
    1  -0.0000352  -0.0006306   0.0001632   0.0000484  -0.0000037   0.0001501
    2  -0.0000321  -0.0000843  -0.0005858   0.0001960  -0.0000123  -0.0001190
    3  -0.0001139  -0.0000349   0.0001582  -0.0001377  -0.0000193  -0.0000411
          31          32          33          34          35          36
    1   0.0000478   0.0000719   0.0001047   0.0000519   0.0000811  -0.0000118
    2  -0.0000074   0.0000163   0.0002794  -0.0001133   0.0000091   0.0000214
    3   0.0002007   0.0001078  -0.0001285   0.0000180   0.0001306  -0.0000259
          37          38
    1  -0.0023417   0.0003394
    2   0.0019700   0.0004178
    3   0.0003503   0.0013983
 Max gradient component =       4.617E-03
 RMS gradient           =       8.701E-04
 Gradient time:  CPU 163.27 s  wall 273.47 s

--------------------------------------------------
-  Entering optman on Sun Oct 13 20:31:57 2024  -
--------------------------------------------------

 Geometry Optimization Parameters
   NAtoms,    NIC,     NZ,  NCons,   NDum,   NFix, NCnnct, MaxDiis
       38     272       0       0       0       0       0       0

 Cartesian Hessian Update
 Hessian updated using BFGS update


** GEOMETRY OPTIMIZATION IN DELOCALIZED INTERNAL COORDINATES **
```

Searching for a Minimum

Optimization Cycle: 5

Coordinates (Angstroms)

| ATOM | | X | Y | Z |
|---|---|---|---|---|
| 1 | N | 2.5406675949 | 0.4066396324 | 0.4938568748 |
| 2 | C | 2.6912947294 | -0.4515556828 | -0.5546593870 |
| 3 | C | 3.5348910844 | -1.5409427452 | -0.3912401017 |
| 4 | C | 4.2110672984 | -1.7508604267 | 0.7994973616 |
| 5 | C | 4.0437377817 | -0.8484474407 | 1.8476625483 |
| 6 | C | 3.2077969819 | 0.2209299972 | 1.6626577485 |
| 7 | C | 1.9554083185 | -0.2098586815 | -1.8324065004 |
| 8 | C | 1.6743344124 | 1.6045067386 | 0.3801777327 |
| 9 | N | -1.7004933652 | 0.2328427924 | 1.2863857717 |
| 10 | C | -2.4991807592 | 0.6914395405 | 0.2839067577 |
| 11 | N | -2.0017911065 | 1.4553161286 | -0.6523088350 |
| 12 | C | -0.7071077653 | 1.8136430522 | -0.6314218963 |
| 13 | C | 0.1936752509 | 1.2874519890 | 0.3584934881 |
| 14 | C | -0.3765682856 | 0.5096490210 | 1.3194281219 |
| 15 | C | -3.9292437804 | 0.2826675011 | 0.2568685360 |
| 16 | C | -4.1526503076 | -0.9209257495 | -0.6887649114 |
| 17 | C | -3.4762276262 | -2.2023669439 | -0.2141789439 |
| 18 | N | -0.3151114020 | 2.6162514159 | -1.6218572624 |
| 19 | H | 3.6618562481 | -2.2202685246 | -1.2239162262 |
| 20 | H | 4.8701977077 | -2.6040058227 | 0.9086724341 |
| 21 | H | 4.5617232300 | -0.9617418103 | 2.7906077126 |
| 22 | H | 3.0510182092 | 0.9691940902 | 2.4284319490 |
| 23 | H | 2.3223735080 | -0.8904241393 | -2.5979495844 |
| 24 | H | 2.0846627454 | 0.8092522053 | -2.2027574488 |
| 25 | H | 0.8840149316 | -0.3923687626 | -1.7094974613 |
| 26 | H | 1.9751215095 | 2.1396950703 | -0.5182428286 |
| 27 | H | 1.9162468469 | 2.2497453501 | 1.2244499320 |
| 28 | H | -2.1111016177 | -0.3492748365 | 2.0065057714 |
| 29 | H | 0.1823764643 | 0.0675717770 | 2.1358898118 |
| 30 | H | -4.2656297045 | 0.0296032839 | 1.2669242370 |
| 31 | H | -4.5068114546 | 1.1408197750 | -0.0890344931 |
| 32 | H | -3.8158459653 | -0.6495477883 | -1.6927992460 |
| 33 | H | -5.2319700105 | -1.0692516275 | -0.7525984458 |
| 34 | H | -2.3824151688 | -2.1222873281 | -0.2153168856 |
| 35 | H | -3.7306708758 | -3.0351989349 | -0.8706743423 |
| 36 | H | -3.8019776011 | -2.4760505138 | 0.7942943588 |
| 37 | H | 0.4935534994 | 3.2124800309 | -1.5574383241 |
| 38 | H | -1.0512215566 | 2.9556783676 | -2.2276480237 |

Point Group: c1 Number of degrees of freedom: 108

Energy is -763.967517505

Hessian updated using BFGS update
internal optimization (0)

108 Hessian modes will be used to form the next step
Hessian Eigenvalues:

| | | | | | |
|---|---|---|---|---|---|
| 0.002159 | 0.002584 | 0.003918 | 0.005625 | 0.008769 | 0.009607 |
| 0.015720 | 0.018714 | 0.019335 | 0.019906 | 0.021144 | 0.022413 |
| 0.022644 | 0.022907 | 0.024046 | 0.024280 | 0.025407 | 0.025951 |
| 0.026673 | 0.028235 | 0.028481 | 0.029848 | 0.033233 | 0.036522 |
| 0.038779 | 0.042163 | 0.043210 | 0.043853 | 0.045141 | 0.046169 |
| 0.051481 | 0.053842 | 0.054668 | 0.055373 | 0.063017 | 0.079504 |
| 0.085504 | 0.094057 | 0.121667 | 0.122193 | 0.127112 | 0.131058 |
| 0.131892 | 0.132977 | 0.136261 | 0.140594 | 0.142467 | 0.143610 |
| 0.147691 | 0.148374 | 0.149765 | 0.152489 | 0.152939 | 0.153264 |
| 0.154279 | 0.160762 | 0.193497 | 0.206239 | 0.209987 | 0.217008 |
| 0.224717 | 0.230795 | 0.238138 | 0.247413 | 0.250467 | 0.261017 |
| 0.267678 | 0.272795 | 0.282437 | 0.298453 | 0.300192 | 0.300605 |
| 0.300676 | 0.301240 | 0.301766 | 0.302767 | 0.304125 | 0.304910 |
| 0.305269 | 0.305648 | 0.307530 | 0.308117 | 0.312518 | 0.318261 |
| 0.321535 | 0.331990 | 0.333099 | 0.334549 | 0.337048 | 0.342025 |
| 0.342215 | 0.351897 | 0.361809 | 0.363155 | 0.380636 | 0.391033 |

```
    0.396840    0.406137    0.417465    0.419963    0.421403    0.431854
    0.443936    0.446711    0.464766    0.493832    0.574641    0.792062

 Minimum search - taking simple RFO step
 Searching for Lamda that Minimizes Along All modes
 Value Taken    Lamda =  -0.00158424
 Calculated Step too Large.  Step scaled by  0.709409
 Step Taken.  Stepsize is  0.300000

                            Maximum     Tolerance    Cnvgd?
         Gradient           0.002633      0.000800      NO
         Displacement       0.114263      0.001400      NO
         Energy change     -0.001452      0.000228      NO


 New Cartesian Coordinates Obtained by Inverse Iteration

 Displacement from previous Coordinates is:  0.667007
 ----------------------------------------------------------------
             Standard Nuclear Orientation (Angstroms)
    I     Atom           X                 Y                 Z
 ----------------------------------------------------------------
    1      N       2.5384634098     0.4076423858     0.4875162209
    2      C       2.6817790807    -0.4746239853    -0.5423358914
    3      C       3.5226500903    -1.5630982977    -0.3578505527
    4      C       4.2007793202    -1.7499447547     0.8357561604
    5      C       4.0383543779    -0.8252169172     1.8647106702
    6      C       3.2055798600     0.2443981242     1.6592464282
    7      C       1.9465419777    -0.2502746644    -1.8240453991
    8      C       1.6747584047     1.6034957942     0.3474885802
    9      N      -1.6848993595     0.2094032983     1.2757345929
   10      C      -2.5021521858     0.6955870615     0.3002882057
   11      N      -2.0228386042     1.4816009257    -0.6240486738
   12      C      -0.7254521097     1.8362348015    -0.6224856689
   13      C       0.1941701586     1.2850217282     0.3383370687
   14      C      -0.3605092858     0.4847727307     1.2875641178
   15      C      -3.9301046183     0.2779938154     0.2848528245
   16      C      -4.1574247933    -0.9080466551    -0.6820464491
   17      C      -3.4379952656    -2.1854529595    -0.2635210884
   18      N      -0.3647663865     2.6803201893    -1.5813511706
   19      H       3.6456328367    -2.2609132241    -1.1758826407
   20      H       4.8572512737    -2.6027943671     0.9615020566
   21      H       4.5566395765    -0.9215741005     2.8095115293
   22      H       3.0512338026     1.0090502627     2.4093260382
   23      H       2.2699667487    -0.9794686518    -2.5640975922
   24      H       2.1314637202     0.7447256607    -2.2358214254
   25      H       0.8681961276    -0.3685281787    -1.6877591869
   26      H       1.9781348028     2.1150920139    -0.5641117981
   27      H       1.9201926836     2.2702876101     1.1740488806
   28      H      -2.0800082793    -0.3933130971     1.9871574838
   29      H       0.2139560175     0.0227138305     2.0818573781
   30      H      -4.2501859942     0.0023563236     1.2942940778
   31      H      -4.5184959448     1.1380179947    -0.0370705166
   32      H      -3.8597453620    -0.6036568472    -1.6889973928
   33      H      -5.2348440549    -1.0790597745    -0.7147803044
   34      H      -2.3468334852    -2.0818887729    -0.3003276480
   35      H      -3.6979090759    -3.0071102489    -0.9318883833
   36      H      -3.7226386068    -2.4916261162     0.7481974499
   37      H       0.5021373064     3.1902578804    -1.5793846617
   38      H      -1.1010781646     3.0476191817    -2.1695833195
 ----------------------------------------------------------------
 Nuclear Repulsion Energy =        1323.88609755 hartrees
 There are       65 alpha and       65 beta electrons

--------------------------------------------------
-  Entering fldman on Sun Oct 13 20:31:57 2024  -
--------------------------------------------------

 Applying Cartesian multipole field
    Component           Value
```

```
    ---------          -----
     (2,0,0)        1.00000E-12
     (0,2,0)        2.00000E-11
     (0,0,2)       -3.00000E-11
 Nucleus-field energy     =     0.0000000039 hartrees

--------------------------------------------------
-  Entering gesman on Sun Oct 13 20:31:57 2024  -
--------------------------------------------------

 Requested basis set is 6-311+G(d,p)
 There are 188 shells and 516 basis functions
 A cutoff of  1.0D-12 yielded  13112 shell pairs
 There are    103594 function pairs (    110433 Cartesian)
 Smallest overlap matrix eigenvalue = 2.10E-06
 Linear dependence detected in AO basis
 Tighter screening thresholds may be required for diffuse basis sets
 Use S2THRESH > 12 and THRESH = 14 in case of SCF convergence issues
 Number of orthogonalized atomic orbitals = 510
 Maximum deviation from orthogonality = 1.965E-11
 Guess MOs from SCF MO coefficient file
 Reading MOs from coefficient file
 Reading MOs from coefficient file

--------------------------------------------------
-  Entering scfman on Sun Oct 13 20:31:58 2024  -
--------------------------------------------------

 Long-range K will be added via erf
 Coulomb attenuation parameter = 0.2 bohr**(-1)
 A restricted hybrid HF-DFT SCF calculation will be
 performed using Pulay DIIS + Geometric Direct Minimization
 Exchange:     0.2220 Hartree-Fock + 1.0000 wB97X-D + LR-HF
 Correlation:  1.0000 wB97X-D
 Using Euler-Maclaurin-Lebedev (75,302) quadrature formula
 Dispersion:   Grimme D
 SCF converges when RMS gradient is below 1.0E-07
 Geometry optimization detected.  Setting ReadMinima to 0
 Setting SaveMinima to 0
 ---------------------------------------
  Cycle       Energy         DIIS Error
 ---------------------------------------
    1    -763.9641206333      6.36E-04
    2    -763.9678163226      8.29E-05
    3    -763.9682481375      4.30E-05
    4    -763.9683041334      2.60E-05
    5    -763.9683283864      6.05E-06
    6    -763.9683299312      2.49E-06
    7    -763.9683301965      6.88E-07
    8    -763.9683302256      3.59E-07
    9    -763.9683302316      8.64E-08 Convergence criterion met
 ---------------------------------------
 SCF time:  CPU 324.92 s  wall 535.16 s
 SCF   energy in the final basis set = -763.96833023
 Total energy in the final basis set = -763.96833023

--------------------------------------------------
-  Entering anlman on Sun Oct 13 20:40:53 2024  -
--------------------------------------------------



 --------------------------------------------------------------

                   Orbital Energies (a.u.)
 --------------------------------------------------------------

 Alpha MOs
 -- Occupied --
-14.8190 -14.7897 -14.7367 -14.7066 -10.6769 -10.6613 -10.6471 -10.6417
-10.6376 -10.6286 -10.6064 -10.5862 -10.5747 -10.5737 -10.5534 -10.5264
```

```
-10.4976 -10.4760  -1.4156  -1.4075  -1.3481  -1.2755  -1.2321  -1.2006
 -1.1911  -1.1433  -1.1031  -1.0870  -1.0550  -1.0539  -1.0359  -1.0086
 -0.9919  -0.9550  -0.9471  -0.9177  -0.9071  -0.8954  -0.8837  -0.8637
 -0.8558  -0.8474  -0.8367  -0.8246  -0.8092  -0.8004  -0.7913  -0.7815
 -0.7775  -0.7569  -0.7561  -0.7514  -0.7461  -0.7318  -0.7230  -0.7206
 -0.7037  -0.6709  -0.6613  -0.6460  -0.6380  -0.6312  -0.6243  -0.6237
 -0.6100
 -- Virtual --
 -0.2719  -0.2596  -0.2431  -0.2285  -0.1742  -0.1652  -0.1500  -0.1461
 -0.1359  -0.1220  -0.1170  -0.1164  -0.1093  -0.1061  -0.1013  -0.0979
 -0.0919  -0.0861  -0.0843  -0.0792  -0.0737  -0.0704  -0.0701  -0.0669
 -0.0639  -0.0592  -0.0572  -0.0553  -0.0506  -0.0474  -0.0413  -0.0384
 -0.0325  -0.0271  -0.0260  -0.0229  -0.0182  -0.0154  -0.0123  -0.0078
 -0.0068  -0.0020  -0.0011   0.0035   0.0066   0.0092   0.0110   0.0139
  0.0167   0.0204   0.0223   0.0283   0.0338   0.0373   0.0392   0.0406
  0.0423   0.0441   0.0477   0.0529   0.0551   0.0596   0.0625   0.0640
  0.0666   0.0708   0.0733   0.0737   0.0792   0.0826   0.0858   0.0882
  0.0936   0.0986   0.1009   0.1069   0.1091   0.1123   0.1161   0.1183
  0.1210   0.1247   0.1265   0.1324   0.1343   0.1394   0.1436   0.1456
  0.1524   0.1580   0.1598   0.1617   0.1667   0.1712   0.1720   0.1787
  0.1813   0.1884   0.1917   0.1954   0.2054   0.2097   0.2117   0.2251
  0.2332   0.2362   0.2511   0.2664   0.2753   0.2765   0.2964   0.3072
  0.3242   0.3298   0.3311   0.3380   0.3480   0.3535   0.3548   0.3740
  0.3820   0.3843   0.3892   0.3947   0.4042   0.4084   0.4206   0.4249
  0.4320   0.4404   0.4464   0.4479   0.4593   0.4654   0.4683   0.4750
  0.4802   0.4865   0.4972   0.5031   0.5108   0.5138   0.5216   0.5268
  0.5288   0.5323   0.5416   0.5422   0.5477   0.5504   0.5515   0.5593
  0.5641   0.5716   0.5789   0.5839   0.5859   0.5916   0.5985   0.6006
  0.6052   0.6182   0.6260   0.6274   0.6347   0.6461   0.6548   0.6633
  0.6651   0.6701   0.6784   0.6802   0.6910   0.6955   0.7075   0.7100
  0.7209   0.7217   0.7341   0.7414   0.7573   0.7643   0.7773   0.7846
  0.7909   0.7939   0.8171   0.8257   0.8320   0.8393   0.8430   0.8539
  0.8634   0.8669   0.8801   0.8914   0.9043   0.9201   0.9212   0.9340
  0.9408   0.9569   0.9649   0.9792   1.0010   1.0208   1.0227   1.0455
  1.0591   1.0639   1.0804   1.0881   1.1152   1.1477   1.1558   1.1839
  1.1979   1.2188   1.2289   1.2340   1.2577   1.2795   1.3004   1.3113
  1.3153   1.3263   1.3338   1.3450   1.3547   1.3703   1.3748   1.3750
  1.3877   1.3929   1.4053   1.4076   1.4221   1.4239   1.4274   1.4366
  1.4378   1.4479   1.4594   1.4707   1.4713   1.4814   1.4903   1.4987
  1.5038   1.5130   1.5173   1.5213   1.5370   1.5387   1.5503   1.5571
  1.5604   1.5665   1.5780   1.5831   1.5897   1.5948   1.6061   1.6107
  1.6210   1.6246   1.6435   1.6498   1.6638   1.6698   1.6729   1.6804
  1.6956   1.7046   1.7131   1.7337   1.7395   1.7493   1.7569   1.7658
  1.7823   1.7912   1.8056   1.8207   1.8392   1.8490   1.8665   1.8867
  1.9011   1.9036   1.9130   1.9231   1.9502   1.9596   1.9752   1.9914
  1.9989   2.0067   2.0205   2.0320   2.0402   2.0546   2.0681   2.0825
  2.0964   2.1256   2.1302   2.1569   2.1640   2.1917   2.2122   2.2189
  2.2271   2.2306   2.2478   2.2519   2.2548   2.2758   2.2882   2.2961
  2.3127   2.3278   2.3370   2.3556   2.3602   2.3817   2.3886   2.3937
  2.4053   2.4143   2.4338   2.4380   2.4477   2.4562   2.4665   2.4934
  2.5056   2.5187   2.5272   2.5314   2.5398   2.5436   2.5453   2.5627
  2.5717   2.5765   2.5795   2.5904   2.5942   2.6009   2.6123   2.6164
  2.6423   2.6458   2.6612   2.6715   2.6751   2.6770   2.6890   2.7099
  2.7153   2.7287   2.7339   2.7461   2.7509   2.7702   2.7748   2.7815
  2.7918   2.8036   2.8192   2.8375   2.8468   2.8737   2.8764   2.8838
  2.8951   2.9291   2.9642   2.9928   3.0396   3.0653   3.1401   3.1635
  3.1677   3.2327   3.2466   3.2713   3.3029   3.3181   3.3674   3.4093
  3.4197   3.4835   3.5254   3.5351   3.5583   3.5951   3.6175   3.6417
  3.6890   3.6994   3.7164   3.7255   3.7416   3.7502   3.7615   3.7684
  3.7724   3.8361   3.8652   3.9014   3.9410   3.9839   4.0304   4.0913
  4.0919   4.2030   4.2940   4.3087   4.5942   4.6687   4.6990   4.7425
  4.9250   5.0349   5.1949  23.5201  23.5810  23.7145  23.7334  23.7378
 23.7782  23.8019  23.8337  23.8586  23.8907  23.9120  23.9630  23.9828
 24.0311  35.4170  35.4603  35.5237  35.6249
 --------------------------------------------------------------

          Ground-State Mulliken Net Atomic Charges

     Atom                 Charge (a.u.)
  ----------------------------------------
```

```
      1  N                        0.415928
      2  C                        0.137450
      3  C                       -0.031062
      4  C                       -0.226875
      5  C                       -0.284151
      6  C                       -0.066408
      7  C                       -0.762224
      8  C                       -0.164627
      9  N                       -0.165523
     10  C                       -0.199095
     11  N                        0.020537
     12  C                        0.217234
     13  C                       -0.132868
     14  C                        0.100472
     15  C                       -0.322010
     16  C                       -0.246386
     17  C                       -0.542079
     18  N                       -0.480149
     19  H                        0.220277
     20  H                        0.236668
     21  H                        0.232956
     22  H                        0.219659
     23  H                        0.235167
     24  H                        0.219127
     25  H                        0.244701
     26  H                        0.175953
     27  H                        0.247281
     28  H                        0.384206
     29  H                        0.211182
     30  H                        0.211998
     31  H                        0.251573
     32  H                        0.203145
     33  H                        0.224581
     34  H                        0.126247
     35  H                        0.216230
     36  H                        0.180838
     37  H                        0.317099
     38  H                        0.372947
  ----------------------------------------
  Sum of atomic charges =     2.000000

 -----------------------------------------------------------------
                    Cartesian Multipole Moments
 -----------------------------------------------------------------
    Charge (ESU x 10^10)
                 9.6064
    Dipole Moment (Debye)
         X       6.8301      Y       2.6457      Z       3.1265
       Tot       7.9640
    Quadrupole Moments (Debye-Ang)
        XX     -15.5904     XY      -7.0949     YY     -69.8392
        XZ       7.1088     YZ      -7.7097     ZZ     -73.1768
    Traceless Quadrupole Moments (Debye-Ang)
       QXX     111.8352    QYY     -50.9112    QZZ     -60.9239
       QXY     -21.2847    QXZ      21.3263    QYZ     -23.1290
    Octopole Moments (Debye-Ang^2)
       XXX      15.0777    XXY     -97.8129    XYY      20.5871
       YYY      -2.2564    XXZ      55.1063    XYZ      -0.7250
       YYZ     -42.3635    XZZ      19.0676    YZZ      -2.8894
       ZZZ      -6.1280
    Traceless Octopole Moments (Debye-Ang^2)
       XXX    -266.4259    YYY     892.7826    ZZZ    -151.4537
       XXY   -1158.3175    XXZ     806.7504    XYY     144.6097
       XYZ     -10.8746    XZZ     121.8162    YYZ    -655.2967
       YZZ     265.5349
    Hexadecapole Moments (Debye-Ang^3)
      XXXX   -3518.5526   XXXY    -143.5755   XXYY    -760.7085
      XYYY      81.2857   YYYY    -997.0868   XXXZ      35.4434
      XXYZ      -9.5364   XYYZ     -36.6069   YYYZ     -33.9598
      XXZZ    -714.2982   XYZZ      18.7297   YYZZ    -249.7786
```

```
      XZZZ    -161.6066   YZZZ      29.7252   ZZZZ    -696.8347
    Traceless Hexadecapole Moments (Debye-Ang^3)
      XXXX    2013.9138   XXXY  -13115.2250   XXXZ   11046.2069
      XXYY    -843.5305   XXYZ    -783.8748   XXZZ   -1170.3833
      XYYY   10495.2044   XYYZ   -1402.1681   XYZZ    2620.0206
      XZZZ   -9644.0387   YYYY   -1970.8621   YYYZ   -2913.4533
      YYZZ    2814.3926   YZZZ    3697.3281   ZZZZ   -1644.0093
 -----------------------------------------------------------------

-------------------------------------------------
-  Entering drvman on Sun Oct 13 20:40:53 2024  -
-------------------------------------------------

 Calculating analytic gradient of the SCF energy
 Gradient of SCF Energy
           1           2           3           4           5           6
    1   0.0004067  -0.0002519   0.0003273   0.0000602   0.0001507  -0.0001441
    2   0.0000085  -0.0002064  -0.0001514  -0.0004753  -0.0001703   0.0002585
    3  -0.0003815  -0.0003636   0.0006667  -0.0004552   0.0000873   0.0003456
           7           8           9          10          11          12
    1   0.0001077   0.0001634   0.0001782  -0.0010046   0.0005386  -0.0005013
    2   0.0005378   0.0000395  -0.0001343   0.0009403  -0.0010510   0.0030991
    3   0.0001492   0.0004241   0.0002453  -0.0014531   0.0011392  -0.0018434
          13          14          15          16          17          18
    1   0.0006303   0.0002643   0.0000461  -0.0000565  -0.0001769   0.0002611
    2  -0.0012340   0.0007428   0.0003980  -0.0006692  -0.0000088  -0.0037317
    3   0.0007251  -0.0003458   0.0000617  -0.0002086   0.0002041  -0.0005028
          19          20          21          22          23          24
    1  -0.0000610  -0.0001337  -0.0001366  -0.0001642  -0.0000975  -0.0005132
    2   0.0002608   0.0000954   0.0001314   0.0000900  -0.0000087   0.0002581
    3  -0.0000117  -0.0000749  -0.0000202  -0.0002379   0.0000875   0.0002853
          25          26          27          28          29          30
    1  -0.0000615  -0.0001410   0.0002458   0.0001786   0.0000455   0.0001818
    2   0.0000141  -0.0000718  -0.0004989   0.0002189   0.0000056  -0.0001293
    3  -0.0000467   0.0001571   0.0000833  -0.0000813  -0.0001311  -0.0000587
          31          32          33          34          35          36
    1   0.0001575  -0.0000153   0.0000748   0.0000537   0.0000995  -0.0000041
    2  -0.0000194   0.0001356   0.0002807  -0.0001946  -0.0000050   0.0000057
    3   0.0000410   0.0000843  -0.0000961   0.0000039   0.0001078  -0.0000366
          37          38
    1  -0.0011598   0.0004513
    2   0.0006560   0.0005833
    3   0.0003533   0.0010975
 Max gradient component =       3.732E-03
 RMS gradient           =       6.327E-04
 Gradient time:  CPU 152.70 s  wall 247.90 s

-------------------------------------------------
-  Entering optman on Sun Oct 13 20:45:01 2024  -
-------------------------------------------------

 Geometry Optimization Parameters
   NAtoms,    NIC,     NZ,  NCons,   NDum,   NFix, NCnnct, MaxDiis
       38     272       0       0       0       0       0       0

 Cartesian Hessian Update
 Hessian updated using BFGS update


** GEOMETRY OPTIMIZATION IN DELOCALIZED INTERNAL COORDINATES **
   Searching for a Minimum

   Optimization Cycle:   6

                     Coordinates (Angstroms)
     ATOM                X               Y               Z
      1  N          2.5384634098    0.4076423858    0.4875162209
      2  C          2.6817790807   -0.4746239853   -0.5423358914
      3  C          3.5226500903   -1.5630982977   -0.3578505527
      4  C          4.2007793202   -1.7499447547    0.8357561604
```

| | | | | |
|---|---|---|---|---|
| 5 | C | 4.0383543779 | -0.8252169172 | 1.8647106702 |
| 6 | C | 3.2055798600 | 0.2443981242 | 1.6592464282 |
| 7 | C | 1.9465419777 | -0.2502746644 | -1.8240453991 |
| 8 | C | 1.6747584047 | 1.6034957942 | 0.3474885802 |
| 9 | N | -1.6848993595 | 0.2094032983 | 1.2757345929 |
| 10 | C | -2.5021521858 | 0.6955870615 | 0.3002882057 |
| 11 | N | -2.0228386042 | 1.4816009257 | -0.6240486738 |
| 12 | C | -0.7254521097 | 1.8362348015 | -0.6224856689 |
| 13 | C | 0.1941701586 | 1.2850217282 | 0.3383370687 |
| 14 | C | -0.3605092858 | 0.4847727307 | 1.2875641178 |
| 15 | C | -3.9301046183 | 0.2779938154 | 0.2848528245 |
| 16 | C | -4.1574247933 | -0.9080466551 | -0.6820464491 |
| 17 | C | -3.4379952656 | -2.1854529595 | -0.2635210884 |
| 18 | N | -0.3647663865 | 2.6803201893 | -1.5813511706 |
| 19 | H | 3.6456328367 | -2.2609132241 | -1.1758826407 |
| 20 | H | 4.8572512737 | -2.6027943671 | 0.9615020566 |
| 21 | H | 4.5566395765 | -0.9215741005 | 2.8095115293 |
| 22 | H | 3.0512338026 | 1.0090502627 | 2.4093260382 |
| 23 | H | 2.2699667487 | -0.9794686518 | -2.5640975922 |
| 24 | H | 2.1314637202 | 0.7447256607 | -2.2358214254 |
| 25 | H | 0.8681961276 | -0.3685281787 | -1.6877591869 |
| 26 | H | 1.9781348028 | 2.1150920139 | -0.5641117981 |
| 27 | H | 1.9201926836 | 2.2702876101 | 1.1740488806 |
| 28 | H | -2.0800082793 | -0.3933130971 | 1.9871574838 |
| 29 | H | 0.2139560175 | 0.0227138305 | 2.0818573781 |
| 30 | H | -4.2501859942 | 0.0023563236 | 1.2942940778 |
| 31 | H | -4.5184959448 | 1.1380179947 | -0.0370705166 |
| 32 | H | -3.8597453620 | -0.6036568472 | -1.6889973928 |
| 33 | H | -5.2348440549 | -1.0790597745 | -0.7147803044 |
| 34 | H | -2.3468334852 | -2.0818887729 | -0.3003276480 |
| 35 | H | -3.6979090759 | -3.0071102489 | -0.9318883833 |
| 36 | H | -3.7226386068 | -2.4916261162 | 0.7481974499 |
| 37 | H | 0.5021373064 | 3.1902578804 | -1.5793846617 |
| 38 | H | -1.1010781646 | 3.0476191817 | -2.1695833195 |

Point Group: c1    Number of degrees of freedom:   108

Energy is   -763.968330232

Hessian updated using BFGS update
internal optimization (0)

108 Hessian modes will be used to form the next step
Hessian Eigenvalues:

| | | | | | |
|---|---|---|---|---|---|
| 0.002086 | 0.002586 | 0.003833 | 0.005422 | 0.007452 | 0.010260 |
| 0.016844 | 0.018753 | 0.019337 | 0.019907 | 0.021553 | 0.022495 |
| 0.022610 | 0.022877 | 0.024113 | 0.024223 | 0.025027 | 0.025827 |
| 0.026678 | 0.028232 | 0.028497 | 0.029826 | 0.033008 | 0.036481 |
| 0.038782 | 0.042139 | 0.043209 | 0.043820 | 0.045039 | 0.045870 |
| 0.051195 | 0.053845 | 0.054660 | 0.055374 | 0.063033 | 0.079525 |
| 0.085504 | 0.094173 | 0.121665 | 0.122198 | 0.127119 | 0.130960 |
| 0.131980 | 0.132984 | 0.136122 | 0.140623 | 0.143096 | 0.144364 |
| 0.147692 | 0.148398 | 0.149785 | 0.152480 | 0.152954 | 0.153546 |
| 0.154482 | 0.160591 | 0.193557 | 0.206275 | 0.210058 | 0.217001 |
| 0.225680 | 0.230774 | 0.238045 | 0.247429 | 0.250845 | 0.260687 |
| 0.267682 | 0.272762 | 0.282317 | 0.298408 | 0.300191 | 0.300556 |
| 0.300618 | 0.301231 | 0.301754 | 0.302722 | 0.304128 | 0.304895 |
| 0.305266 | 0.305641 | 0.307531 | 0.308122 | 0.312489 | 0.318137 |
| 0.321409 | 0.332008 | 0.332962 | 0.334585 | 0.337052 | 0.342049 |
| 0.342200 | 0.353113 | 0.362126 | 0.363558 | 0.381096 | 0.391016 |
| 0.397286 | 0.406695 | 0.417691 | 0.420202 | 0.421641 | 0.439689 |
| 0.443571 | 0.447591 | 0.465161 | 0.493214 | 0.575372 | 0.788323 |

Minimum search - taking simple RFO step
Searching for Lamda that Minimizes Along All modes
Value Taken    Lamda =  -0.00076462
Calculated Step too Large.  Step scaled by  0.824434
Step Taken.  Stepsize is  0.300000

Maximum     Tolerance    Cnvgd?

```
        Gradient           0.002922      0.000800      NO
        Displacement       0.131358      0.001400      NO
        Energy change     -0.000813      0.000228      NO


 New Cartesian Coordinates Obtained by Inverse Iteration

 Displacement from previous Coordinates is:  0.797980
 ----------------------------------------------------------------
             Standard Nuclear Orientation (Angstroms)
    I     Atom           X                Y                Z
 ----------------------------------------------------------------
    1      N       2.5372221719     0.4079582530     0.4753369115
    2      C       2.6857734265    -0.4985864395    -0.5330726213
    3      C       3.5234358334    -1.5829962777    -0.3185771915
    4      C       4.1941062165    -1.7408926673     0.8842058779
    5      C       4.0271071771    -0.7926820333     1.8902812019
    6      C       3.1968718182     0.2720686008     1.6545079510
    7      C       1.9638527861    -0.2975193526    -1.8267290475
    8      C       1.6728289451     1.5978494322     0.2999590724
    9      N      -1.6661652722     0.1726557677     1.2643148625
   10      C      -2.5054399029     0.6925409365     0.3253534134
   11      N      -2.0484172458     1.5089213334    -0.5848471848
   12      C      -0.7484865651     1.8588225646    -0.6058539344
   13      C       0.1925879051     1.2761747522     0.3152597553
   14      C      -0.3414091503     0.4461361349     1.2520564346
   15      C      -3.9334430035     0.2727243884     0.3291711786
   16      C      -4.1767653288    -0.8850671761    -0.6668698543
   17      C      -3.4138761153    -2.1599339806    -0.3242818583
   18      N      -0.4110887785     2.7436556027    -1.5352996368
   19      H       3.6511196551    -2.3015177137    -1.1179648003
   20      H       4.8490875800    -2.5913521189     1.0342784255
   21      H       4.5397015041    -0.8688150692     2.8400754904
   22      H       3.0376819206     1.0536229407     2.3862276098
   23      H       2.2454265018    -1.0784939632    -2.5302310280
   24      H       2.2123660408     0.6637077958    -2.2847475609
   25      H       0.8804740370    -0.3433650960    -1.6905503117
   26      H       1.9705405610     2.0743420172    -0.6327522001
   27      H       1.9238107418     2.2965052459     1.0985304950
   28      H      -2.0445934380    -0.4549148367     1.9632927362
   29      H       0.2509260723    -0.0404719573     2.0179935134
   30      H      -4.2312063486    -0.0322923968     1.3370875368
   31      H      -4.5316930364     1.1390673845     0.0440634455
   32      H      -3.9271048207    -0.5412306110    -1.6742203232
   33      H      -5.2508119519    -1.0795856584    -0.6610442626
   34      H      -2.3278614081    -2.0303582740    -0.4034944017
   35      H      -3.6858535742    -2.9635712149    -1.0096285634
   36      H      -3.6484247702    -2.5070661466     0.6872923988
   37      H       0.4892817337     3.1881186131    -1.5900704413
   38      H      -1.1515619178     3.1258412203    -2.1090530885
 ----------------------------------------------------------------
 Nuclear Repulsion Energy =        1323.66702223 hartrees
 There are       65 alpha and       65 beta electrons

------------------------------------------------
-  Entering fldman on Sun Oct 13 20:45:01 2024  -
------------------------------------------------

 Applying Cartesian multipole field
    Component          Value
    ---------          -----
      (2,0,0)       1.00000E-12
      (0,2,0)       2.00000E-11
      (0,0,2)      -3.00000E-11
 Nucleus-field energy     =     0.0000000043 hartrees

------------------------------------------------
-  Entering gesman on Sun Oct 13 20:45:01 2024  -
------------------------------------------------
```

```
 Requested basis set is 6-311+G(d,p)
 There are 188 shells and 516 basis functions
 A cutoff of  1.0D-12 yielded  13107 shell pairs
 There are    103527 function pairs (    110336 Cartesian)
 Smallest overlap matrix eigenvalue = 2.13E-06
 Linear dependence detected in AO basis
 Tighter screening thresholds may be required for diffuse basis sets
 Use S2THRESH > 12 and THRESH = 14 in case of SCF convergence issues
 Number of orthogonalized atomic orbitals = 510
 Maximum deviation from orthogonality = 1.927E-11
 Guess MOs from SCF MO coefficient file
 Reading MOs from coefficient file
 Reading MOs from coefficient file

--------------------------------------------------
-  Entering scfman on Sun Oct 13 20:45:01 2024  -
--------------------------------------------------

 Long-range K will be added via erf
 Coulomb attenuation parameter = 0.2 bohr**(-1)
 A restricted hybrid HF-DFT SCF calculation will be
 performed using Pulay DIIS + Geometric Direct Minimization
 Exchange:     0.2220 Hartree-Fock + 1.0000 wB97X-D + LR-HF
 Correlation:  1.0000 wB97X-D
 Using Euler-Maclaurin-Lebedev (75,302) quadrature formula
 Dispersion:   Grimme D
 SCF converges when RMS gradient is below 1.0E-07
 Geometry optimization detected.  Setting ReadMinima to 0
 Setting SaveMinima to 0
 ---------------------------------------
  Cycle       Energy         DIIS Error
 ---------------------------------------
    1    -763.9582211150      7.28E-04
    2    -763.9681914433      9.05E-05
    3    -763.9687343167      4.52E-05
    4    -763.9688006174      2.47E-05
    5    -763.9688232271      8.04E-06
    6    -763.9688254866      2.59E-06
    7    -763.9688257837      6.70E-07
    8    -763.9688258125      3.40E-07
    9    -763.9688258181      8.02E-08 Convergence criterion met
 ---------------------------------------
 SCF time:  CPU 317.38 s  wall 528.04 s
 SCF   energy in the final basis set = -763.96882582
 Total energy in the final basis set = -763.96882582

--------------------------------------------------
-  Entering anlman on Sun Oct 13 20:53:49 2024  -
--------------------------------------------------


 --------------------------------------------------------------

                   Orbital Energies (a.u.)
 --------------------------------------------------------------

 Alpha MOs
 -- Occupied --
-14.8195 -14.7889 -14.7373 -14.7060 -10.6768 -10.6606 -10.6466 -10.6422
-10.6380 -10.6292 -10.6065 -10.5868 -10.5752 -10.5743 -10.5539 -10.5259
-10.4973 -10.4764  -1.4149  -1.4079  -1.3475  -1.2748  -1.2323  -1.2004
 -1.1917  -1.1431  -1.1031  -1.0874  -1.0546  -1.0537  -1.0364  -1.0083
 -0.9922  -0.9549  -0.9471  -0.9178  -0.9073  -0.8950  -0.8844  -0.8634
 -0.8562  -0.8474  -0.8368  -0.8249  -0.8090  -0.8009  -0.7914  -0.7823
 -0.7766  -0.7576  -0.7557  -0.7519  -0.7462  -0.7323  -0.7229  -0.7209
 -0.7034  -0.6704  -0.6618  -0.6460  -0.6373  -0.6310  -0.6246  -0.6241
 -0.6088
 -- Virtual --
 -0.2726  -0.2588  -0.2418  -0.2287  -0.1742  -0.1654  -0.1500  -0.1459
 -0.1361  -0.1216  -0.1169  -0.1162  -0.1092  -0.1065  -0.1014  -0.0984
```

| | | | | | | | |
|---|---|---|---|---|---|---|---|
| -0.0919 | -0.0862 | -0.0846 | -0.0790 | -0.0737 | -0.0709 | -0.0704 | -0.0675 |
| -0.0638 | -0.0588 | -0.0576 | -0.0550 | -0.0512 | -0.0488 | -0.0412 | -0.0380 |
| -0.0331 | -0.0273 | -0.0260 | -0.0236 | -0.0179 | -0.0152 | -0.0120 | -0.0075 |
| -0.0060 | -0.0018 | -0.0013 | 0.0026 | 0.0058 | 0.0088 | 0.0105 | 0.0139 |
| 0.0168 | 0.0202 | 0.0233 | 0.0290 | 0.0341 | 0.0373 | 0.0390 | 0.0404 |
| 0.0429 | 0.0437 | 0.0481 | 0.0522 | 0.0561 | 0.0599 | 0.0625 | 0.0645 |
| 0.0664 | 0.0711 | 0.0726 | 0.0735 | 0.0808 | 0.0824 | 0.0858 | 0.0897 |
| 0.0923 | 0.1000 | 0.1011 | 0.1051 | 0.1086 | 0.1119 | 0.1161 | 0.1186 |
| 0.1217 | 0.1248 | 0.1269 | 0.1336 | 0.1350 | 0.1389 | 0.1430 | 0.1453 |
| 0.1511 | 0.1593 | 0.1613 | 0.1640 | 0.1664 | 0.1705 | 0.1728 | 0.1790 |
| 0.1818 | 0.1902 | 0.1919 | 0.1944 | 0.2064 | 0.2071 | 0.2119 | 0.2267 |
| 0.2348 | 0.2436 | 0.2519 | 0.2662 | 0.2743 | 0.2809 | 0.2978 | 0.3073 |
| 0.3249 | 0.3299 | 0.3320 | 0.3375 | 0.3470 | 0.3549 | 0.3560 | 0.3733 |
| 0.3826 | 0.3848 | 0.3883 | 0.3957 | 0.4063 | 0.4082 | 0.4196 | 0.4264 |
| 0.4299 | 0.4389 | 0.4464 | 0.4532 | 0.4583 | 0.4651 | 0.4668 | 0.4768 |
| 0.4796 | 0.4851 | 0.4965 | 0.5026 | 0.5098 | 0.5136 | 0.5226 | 0.5251 |
| 0.5284 | 0.5305 | 0.5398 | 0.5436 | 0.5463 | 0.5494 | 0.5521 | 0.5589 |
| 0.5652 | 0.5730 | 0.5783 | 0.5846 | 0.5888 | 0.5939 | 0.5985 | 0.6009 |
| 0.6040 | 0.6203 | 0.6253 | 0.6296 | 0.6358 | 0.6448 | 0.6535 | 0.6610 |
| 0.6693 | 0.6703 | 0.6811 | 0.6820 | 0.6918 | 0.6989 | 0.7073 | 0.7098 |
| 0.7172 | 0.7247 | 0.7359 | 0.7420 | 0.7562 | 0.7684 | 0.7790 | 0.7861 |
| 0.7885 | 0.7956 | 0.8153 | 0.8235 | 0.8352 | 0.8420 | 0.8483 | 0.8602 |
| 0.8662 | 0.8699 | 0.8819 | 0.8941 | 0.9059 | 0.9220 | 0.9266 | 0.9360 |
| 0.9455 | 0.9605 | 0.9739 | 0.9825 | 1.0037 | 1.0209 | 1.0271 | 1.0436 |
| 1.0521 | 1.0603 | 1.0703 | 1.0879 | 1.1206 | 1.1469 | 1.1514 | 1.1804 |
| 1.1973 | 1.2123 | 1.2271 | 1.2290 | 1.2588 | 1.2806 | 1.2986 | 1.3081 |
| 1.3141 | 1.3246 | 1.3338 | 1.3454 | 1.3523 | 1.3632 | 1.3727 | 1.3758 |
| 1.3869 | 1.3898 | 1.4057 | 1.4089 | 1.4214 | 1.4266 | 1.4276 | 1.4359 |
| 1.4389 | 1.4493 | 1.4609 | 1.4687 | 1.4710 | 1.4793 | 1.4900 | 1.5000 |
| 1.5037 | 1.5128 | 1.5177 | 1.5255 | 1.5349 | 1.5389 | 1.5466 | 1.5562 |
| 1.5621 | 1.5676 | 1.5776 | 1.5802 | 1.5919 | 1.5940 | 1.6059 | 1.6077 |
| 1.6219 | 1.6254 | 1.6392 | 1.6495 | 1.6594 | 1.6693 | 1.6721 | 1.6835 |
| 1.6960 | 1.7053 | 1.7074 | 1.7304 | 1.7364 | 1.7491 | 1.7579 | 1.7660 |
| 1.7829 | 1.7908 | 1.8074 | 1.8219 | 1.8388 | 1.8493 | 1.8658 | 1.8835 |
| 1.9009 | 1.9041 | 1.9133 | 1.9257 | 1.9512 | 1.9665 | 1.9804 | 1.9956 |
| 2.0012 | 2.0063 | 2.0226 | 2.0295 | 2.0366 | 2.0608 | 2.0645 | 2.0809 |
| 2.0998 | 2.1258 | 2.1315 | 2.1585 | 2.1658 | 2.1923 | 2.2125 | 2.2212 |
| 2.2277 | 2.2340 | 2.2467 | 2.2522 | 2.2553 | 2.2689 | 2.2870 | 2.2910 |
| 2.3125 | 2.3233 | 2.3321 | 2.3548 | 2.3583 | 2.3740 | 2.3882 | 2.3952 |
| 2.4044 | 2.4160 | 2.4355 | 2.4400 | 2.4473 | 2.4588 | 2.4681 | 2.4892 |
| 2.5046 | 2.5159 | 2.5244 | 2.5292 | 2.5418 | 2.5432 | 2.5441 | 2.5630 |
| 2.5732 | 2.5767 | 2.5800 | 2.5910 | 2.5938 | 2.6018 | 2.6120 | 2.6153 |
| 2.6405 | 2.6431 | 2.6632 | 2.6730 | 2.6752 | 2.6772 | 2.6919 | 2.7118 |
| 2.7141 | 2.7304 | 2.7356 | 2.7431 | 2.7576 | 2.7700 | 2.7768 | 2.7825 |
| 2.8011 | 2.8070 | 2.8218 | 2.8379 | 2.8482 | 2.8763 | 2.8807 | 2.8864 |
| 2.8956 | 2.9291 | 2.9659 | 2.9955 | 3.0416 | 3.0638 | 3.1347 | 3.1618 |
| 3.1693 | 3.2326 | 3.2462 | 3.2714 | 3.3062 | 3.3191 | 3.3691 | 3.4102 |
| 3.4215 | 3.4820 | 3.5253 | 3.5333 | 3.5601 | 3.5962 | 3.6144 | 3.6412 |
| 3.6872 | 3.6985 | 3.7123 | 3.7302 | 3.7383 | 3.7513 | 3.7548 | 3.7626 |
| 3.7693 | 3.8351 | 3.8648 | 3.8999 | 3.9428 | 3.9826 | 4.0277 | 4.0897 |
| 4.0917 | 4.2038 | 4.2928 | 4.3088 | 4.5942 | 4.6694 | 4.6963 | 4.7476 |
| 4.9273 | 5.0343 | 5.1966 | 23.5196 | 23.5817 | 23.7153 | 23.7327 | 23.7369 |
| 23.7791 | 23.8024 | 23.8337 | 23.8571 | 23.8909 | 23.9120 | 23.9630 | 23.9797 |
| 24.0308 | 35.4155 | 35.4618 | 35.5249 | 35.6252 | | | |

Ground-State Mulliken Net Atomic Charges

| Atom | Charge (a.u.) |
|---|---|
| 1 N | 0.399603 |
| 2 C | 0.162739 |
| 3 C | -0.028644 |
| 4 C | -0.222267 |
| 5 C | -0.287856 |
| 6 C | -0.052582 |
| 7 C | -0.790193 |
| 8 C | -0.147239 |
| 9 N | -0.161180 |
| 10 C | -0.212243 |

```
    11 N                       0.017082
    12 C                       0.240008
    13 C                      -0.153446
    14 C                       0.089396
    15 C                      -0.316422
    16 C                      -0.253389
    17 C                      -0.539252
    18 N                      -0.490227
    19 H                       0.220622
    20 H                       0.236932
    21 H                       0.233259
    22 H                       0.220977
    23 H                       0.234667
    24 H                       0.227593
    25 H                       0.242561
    26 H                       0.171853
    27 H                       0.249143
    28 H                       0.382824
    29 H                       0.210370
    30 H                       0.212528
    31 H                       0.251821
    32 H                       0.205161
    33 H                       0.224834
    34 H                       0.126368
    35 H                       0.216579
    36 H                       0.179801
    37 H                       0.318814
    38 H                       0.379402
  ----------------------------------------
  Sum of atomic charges =      2.000000

 -----------------------------------------------------------------
                   Cartesian Multipole Moments
 -----------------------------------------------------------------
    Charge (ESU x 10^10)
                9.6064
    Dipole Moment (Debye)
         X       6.9412      Y       2.5539      Z       3.0157
       Tot       7.9874
    Quadrupole Moments (Debye-Ang)
        XX     -15.7423     XY      -7.0377     YY     -69.6116
        XZ       7.3414     YZ      -8.2046     ZZ     -73.3847
    Traceless Quadrupole Moments (Debye-Ang)
       QXX     111.5117    QYY     -50.0961    QZZ     -61.4156
       QXY     -21.1132    QXZ      22.0241    QYZ     -24.6138
    Octopole Moments (Debye-Ang^2)
       XXX      15.9797    XXY     -96.8817    XYY      20.5292
       YYY      -2.4654    XXZ      55.7706    XYZ      -0.7529
       YYZ     -43.4212    XZZ      20.3896    YZZ      -3.5139
       ZZZ      -8.4079
    Traceless Octopole Moments (Debye-Ang^2)
       XXX    -272.3902    YYY     888.7678    ZZZ    -161.5918
       XXY   -1144.6423    XXZ     824.7345    XYY     137.2419
       XYZ     -11.2938    XZZ     135.1483    YYZ    -663.1427
       YZZ     255.8745
    Hexadecapole Moments (Debye-Ang^3)
      XXXX   -3524.4621   XXXY    -130.8104   XXYY    -762.4077
      XYYY      82.5456   YYYY   -1004.5728   XXXZ      35.1115
      XXYZ     -12.1628   XYYZ     -35.9848   YYYZ     -38.5087
      XXZZ    -710.4757   XYZZ      22.0101   YYZZ    -247.2634
      XZZZ    -163.0910   YZZZ      27.7386   ZZZZ    -693.8293
    Traceless Hexadecapole Moments (Debye-Ang^3)
      XXXX    1724.1584   XXXY  -12553.6268   XXXZ   11065.1013
      XXYY    -868.4438   XXYZ    -922.0210   XXZZ    -855.7147
      XYYY    9848.7497   XYYZ   -1318.9422   XYZZ    2704.8770
      XZZZ   -9746.1590   YYYY   -2166.6086   YYYZ   -2978.1961
      YYZZ    3035.0523   YZZZ    3900.2171   ZZZZ   -2179.3377
 -----------------------------------------------------------------

 ------------------------------------------------
```

```
-  Entering drvman on Sun Oct 13 20:53:49 2024  -
-------------------------------------------------

 Calculating analytic gradient of the SCF energy
 Gradient of SCF Energy
           1           2           3           4           5           6
    1   0.0003659   0.0001258  -0.0001520  -0.0001444  -0.0000923   0.0001971
    2  -0.0001116  -0.0005018   0.0001890   0.0000886   0.0002650  -0.0003827
    3  -0.0002938   0.0001127  -0.0001312   0.0000283   0.0000999   0.0000465
           7           8           9          10          11          12
    1  -0.0001775  -0.0000024   0.0001641  -0.0007734  -0.0000398   0.0001313
    2   0.0002410   0.0001758  -0.0005191   0.0002590  -0.0006168   0.0014801
    3   0.0001063   0.0003614   0.0006670  -0.0007956   0.0002428  -0.0013783
          13          14          15          16          17          18
    1   0.0010411  -0.0004771  -0.0001362  -0.0000420   0.0000576  -0.0001025
    2  -0.0001475   0.0000660   0.0002073  -0.0004325   0.0001055  -0.0011051
    3  -0.0000353   0.0003347   0.0000915   0.0001660  -0.0000393  -0.0005038
          19          20          21          22          23          24
    1  -0.0000043  -0.0000145  -0.0000957  -0.0000910  -0.0000995  -0.0004017
    2   0.0000942  -0.0000458   0.0000208   0.0000617   0.0000513   0.0002828
    3  -0.0000405  -0.0000467  -0.0000003  -0.0001044  -0.0000749   0.0000830
          25          26          27          28          29          30
    1   0.0000167   0.0003509   0.0001577   0.0001530   0.0000482   0.0000617
    2  -0.0000044   0.0000831  -0.0003348   0.0000988   0.0000511  -0.0000707
    3   0.0001033   0.0004014   0.0000626   0.0000427  -0.0000964  -0.0000305
          31          32          33          34          35          36
    1   0.0000374  -0.0000122   0.0000119   0.0000506   0.0000437  -0.0000081
    2  -0.0000126   0.0001163   0.0001516  -0.0001229  -0.0000344   0.0000097
    3  -0.0000654  -0.0000156  -0.0000172   0.0000478   0.0000495   0.0000090
          37          38
    1  -0.0002521   0.0001036
    2  -0.0001746   0.0005185
    3   0.0000540   0.0005589
 Max gradient component =       1.480E-03
 RMS gradient           =       3.319E-04
 Gradient time:  CPU 153.02 s  wall 250.91 s

-------------------------------------------------
-  Entering optman on Sun Oct 13 20:58:00 2024  -
-------------------------------------------------

 Geometry Optimization Parameters
   NAtoms,    NIC,     NZ,  NCons,   NDum,   NFix, NCnnct, MaxDiis
       38     272       0       0       0       0       0       0

 Cartesian Hessian Update
 Hessian updated using BFGS update


** GEOMETRY OPTIMIZATION IN DELOCALIZED INTERNAL COORDINATES **
   Searching for a Minimum

   Optimization Cycle:   7

                    Coordinates (Angstroms)
     ATOM                X               Y               Z
      1  N         2.5372221719    0.4079582530    0.4753369115
      2  C         2.6857734265   -0.4985864395   -0.5330726213
      3  C         3.5234358334   -1.5829962777   -0.3185771915
      4  C         4.1941062165   -1.7408926673    0.8842058779
      5  C         4.0271071771   -0.7926820333    1.8902812019
      6  C         3.1968718182    0.2720686008    1.6545079510
      7  C         1.9638527861   -0.2975193526   -1.8267290475
      8  C         1.6728289451    1.5978494322    0.2999590724
      9  N        -1.6661652722    0.1726557677    1.2643148625
     10  C        -2.5054399029    0.6925409365    0.3253534134
     11  N        -2.0484172458    1.5089213334   -0.5848471848
     12  C        -0.7484865651    1.8588225646   -0.6058539344
     13  C         0.1925879051    1.2761747522    0.3152597553
     14  C        -0.3414091503    0.4461361349    1.2520564346
```

```
    15  C        -3.9334430035    0.2727243884    0.3291711786
    16  C        -4.1767653288   -0.8850671761   -0.6668698543
    17  C        -3.4138761153   -2.1599339806   -0.3242818583
    18  N        -0.4110887785    2.7436556027   -1.5352996368
    19  H         3.6511196551   -2.3015177137   -1.1179648003
    20  H         4.8490875800   -2.5913521189    1.0342784255
    21  H         4.5397015041   -0.8688150692    2.8400754904
    22  H         3.0376819206    1.0536229407    2.3862276098
    23  H         2.2454265018   -1.0784939632   -2.5302310280
    24  H         2.2123660408    0.6637077958   -2.2847475609
    25  H         0.8804740370   -0.3433650960   -1.6905503117
    26  H         1.9705405610    2.0743420172   -0.6327522001
    27  H         1.9238107418    2.2965052459    1.0985304950
    28  H        -2.0445934380   -0.4549148367    1.9632927362
    29  H         0.2509260723   -0.0404719573    2.0179935134
    30  H        -4.2312063486   -0.0322923968    1.3370875368
    31  H        -4.5316930364    1.1390673845    0.0440634455
    32  H        -3.9271048207   -0.5412306110   -1.6742203232
    33  H        -5.2508119519   -1.0795856584   -0.6610442626
    34  H        -2.3278614081   -2.0303582740   -0.4034944017
    35  H        -3.6858535742   -2.9635712149   -1.0096285634
    36  H        -3.6484247702   -2.5070661466    0.6872923988
    37  H         0.4892817337    3.1881186131   -1.5900704413
    38  H        -1.1515619178    3.1258412203   -2.1090530885
  Point Group: c1    Number of degrees of freedom:   108


  Energy is   -763.968825818

 Hessian updated using BFGS update
  internal optimization (0)

108 Hessian modes will be used to form the next step
  Hessian Eigenvalues:
     0.001399    0.002600    0.003750    0.004524    0.006977    0.010771
     0.018569    0.018880    0.019344    0.019937    0.021789    0.022489
     0.022703    0.023115    0.023867    0.024476    0.024727    0.025793
     0.026764    0.028230    0.028698    0.029823    0.032906    0.036471
     0.038791    0.042022    0.043216    0.043742    0.044741    0.045621
     0.051116    0.053847    0.054584    0.055372    0.062906    0.079424
     0.085503    0.094276    0.121679    0.122197    0.127123    0.130873
     0.131979    0.132981    0.136384    0.141021    0.143155    0.144403
     0.147693    0.148569    0.149856    0.152521    0.152984    0.153680
     0.156066    0.160476    0.193673    0.206276    0.210090    0.217149
     0.225376    0.230788    0.239411    0.247664    0.251029    0.260162
     0.267622    0.272783    0.282081    0.298791    0.300185    0.300559
     0.300638    0.301224    0.301742    0.302783    0.304125    0.304943
     0.305274    0.305628    0.307531    0.308142    0.312477    0.318661
     0.321273    0.332063    0.332809    0.335117    0.337063    0.341820
     0.342158    0.353780    0.361840    0.365398    0.381085    0.391043
     0.399810    0.406568    0.417783    0.420046    0.425054    0.433000
     0.443190    0.451734    0.465285    0.501725    0.579355    0.766845

 Minimum search - taking simple RFO step
 Searching for Lamda that Minimizes Along All modes
 Value Taken    Lamda =  -0.00074053
 Calculated Step too Large.  Step scaled by  0.598976
 Step Taken.  Stepsize is  0.300000

                             Maximum     Tolerance    Cnvgd?
        Gradient            0.002645      0.000800      NO
        Displacement        0.142812      0.001400      NO
        Energy change      -0.000496      0.000228      NO


 New Cartesian Coordinates Obtained by Inverse Iteration

 Displacement from previous Coordinates is:  0.849436
 ----------------------------------------------------------------
             Standard Nuclear Orientation (Angstroms)
```

```
    I     Atom           X                Y                Z
 ----------------------------------------------------------------
    1      N       2.5387162677     0.4094621303     0.4579043040
    2      C       2.7007046939    -0.5205252481    -0.5273260615
    3      C       3.5360866394    -1.5995162586    -0.2764136014
    4      C       4.1931708165    -1.7273204207     0.9374382306
    5      C       4.0143976722    -0.7546656226     1.9174993028
    6      C       3.1853186070     0.3039201105     1.6472001285
    7      C       2.0036854495    -0.3429713634    -1.8382957049
    8      C       1.6695091052     1.5876290873     0.2427176978
    9      N      -1.6453341918     0.1302051878     1.2507615272
   10      C      -2.5072902790     0.6866558883     0.3549054756
   11      N      -2.0734070302     1.5342718997    -0.5375847001
   12      C      -0.7722082836     1.8767139863    -0.5833932687
   13      C       0.1908573726     1.2603339698     0.2913595712
   14      C      -0.3201063034     0.3996308109     1.2121845091
   15      C      -3.9352437209     0.2664166794     0.3786094885
   16      C      -4.1999059774    -0.8559385684    -0.6516347595
   17      C      -3.4165398207    -2.1354666457    -0.3793663782
   18      N      -0.4575638865     2.8008101415    -1.4808538250
   19      H       3.6741693356    -2.3374792020    -1.0563367774
   20      H       4.8470972523    -2.5730884551     1.1159085575
   21      H       4.5170299416    -0.8076417424     2.8743122663
   22      H       3.0161257909     1.1021076074     2.3585860751
   23      H       2.2427631808    -1.1744779711    -2.4982284667
   24      H       2.3232449990     0.5759846554    -2.3387205854
   25      H       0.9183384815    -0.3102697150    -1.7179517863
   26      H       1.9520509929     2.0205697636    -0.7160007589
   27      H       1.9279333496     2.3238903054     1.0047511718
   28      H      -2.0072665662    -0.5233263837     1.9345390126
   29      H       0.2897643461    -0.1152517455     1.9450041773
   30      H      -4.2094348474    -0.0726271850     1.3823804022
   31      H      -4.5405168457     1.1411502648     0.1371200331
   32      H      -3.9831877247    -0.4743881800    -1.6528823814
   33      H      -5.2714825349    -1.0617248712    -0.6203715420
   34      H      -2.3348189066    -1.9913507672    -0.4862131028
   35      H      -3.7023080196    -2.9154873249    -1.0860345921
   36      H      -3.6174026939    -2.5224369233     0.6251000315
   37      H       0.4579852103     3.2046343002    -1.5807891357
   38      H      -1.2049318725     3.1915678053    -2.0398845347
 ----------------------------------------------------------------
 Nuclear Repulsion Energy =        1323.10802605 hartrees
 There are       65 alpha and       65 beta electrons

------------------------------------------------
-  Entering fldman on Sun Oct 13 20:58:00 2024  -
------------------------------------------------

 Applying Cartesian multipole field
    Component          Value
    ---------          -----
     (2,0,0)       1.00000E-12
     (0,2,0)       2.00000E-11
     (0,0,2)      -3.00000E-11
 Nucleus-field energy     =     0.0000000046 hartrees

------------------------------------------------
-  Entering gesman on Sun Oct 13 20:58:00 2024  -
------------------------------------------------

 Requested basis set is 6-311+G(d,p)
 There are 188 shells and 516 basis functions
 A cutoff of  1.0D-12 yielded  13099 shell pairs
 There are    103516 function pairs (    110324 Cartesian)
 Smallest overlap matrix eigenvalue = 2.17E-06
 Linear dependence detected in AO basis
 Tighter screening thresholds may be required for diffuse basis sets
 Use S2THRESH > 12 and THRESH = 14 in case of SCF convergence issues
 Number of orthogonalized atomic orbitals = 510
 Maximum deviation from orthogonality = 1.637E-11
```

```
 Guess MOs from SCF MO coefficient file
 Reading MOs from coefficient file
 Reading MOs from coefficient file

-------------------------------------------------
-  Entering scfman on Sun Oct 13 20:58:01 2024  -
-------------------------------------------------

 Long-range K will be added via erf
 Coulomb attenuation parameter = 0.2 bohr**(-1)
 A restricted hybrid HF-DFT SCF calculation will be
 performed using Pulay DIIS + Geometric Direct Minimization
 Exchange:     0.2220 Hartree-Fock + 1.0000 wB97X-D + LR-HF
 Correlation:  1.0000 wB97X-D
 Using Euler-Maclaurin-Lebedev (75,302) quadrature formula
 Dispersion:   Grimme D
 SCF converges when RMS gradient is below 1.0E-07
 Geometry optimization detected.  Setting ReadMinima to 0
 Setting SaveMinima to 0
 ---------------------------------------
  Cycle       Energy         DIIS Error
 ---------------------------------------
    1    -763.9645978603      7.77E-04
    2    -763.9685357047      9.19E-05
    3    -763.9691089433      4.75E-05
    4    -763.9691784619      2.75E-05
    5    -763.9692058943      7.89E-06
    6    -763.9692080984      2.66E-06
    7    -763.9692084124      6.82E-07
    8    -763.9692084434      3.38E-07
    9    -763.9692084488      8.13E-08 Convergence criterion met
 ---------------------------------------
 SCF time:  CPU 320.23 s  wall 528.24 s
 SCF   energy in the final basis set = -763.96920845
 Total energy in the final basis set = -763.96920845

-------------------------------------------------
-  Entering anlman on Sun Oct 13 21:06:49 2024  -
-------------------------------------------------



 --------------------------------------------------------------

                   Orbital Energies (a.u.)
 --------------------------------------------------------------

 Alpha MOs
 -- Occupied --
-14.8201 -14.7882 -14.7374 -14.7055 -10.6763 -10.6599 -10.6461 -10.6427
-10.6383 -10.6299 -10.6064 -10.5873 -10.5758 -10.5748 -10.5540 -10.5255
-10.4970 -10.4764  -1.4147  -1.4083  -1.3473  -1.2745  -1.2326  -1.2003
 -1.1920  -1.1430  -1.1032  -1.0876  -1.0544  -1.0537  -1.0368  -1.0081
 -0.9923  -0.9549  -0.9471  -0.9178  -0.9072  -0.8948  -0.8851  -0.8630
 -0.8565  -0.8474  -0.8373  -0.8253  -0.8087  -0.8014  -0.7915  -0.7833
 -0.7758  -0.7577  -0.7551  -0.7525  -0.7460  -0.7326  -0.7227  -0.7212
 -0.7032  -0.6700  -0.6624  -0.6458  -0.6369  -0.6308  -0.6249  -0.6241
 -0.6081
 -- Virtual --
 -0.2732  -0.2582  -0.2406  -0.2290  -0.1742  -0.1655  -0.1499  -0.1457
 -0.1362  -0.1208  -0.1168  -0.1160  -0.1089  -0.1069  -0.1014  -0.0987
 -0.0920  -0.0861  -0.0850  -0.0788  -0.0739  -0.0715  -0.0710  -0.0682
 -0.0637  -0.0589  -0.0579  -0.0545  -0.0514  -0.0502  -0.0409  -0.0377
 -0.0335  -0.0275  -0.0261  -0.0240  -0.0180  -0.0150  -0.0114  -0.0079
 -0.0053  -0.0016  -0.0013   0.0018   0.0050   0.0085   0.0109   0.0140
  0.0171   0.0195   0.0239   0.0302   0.0338   0.0370   0.0389   0.0405
  0.0431   0.0437   0.0482   0.0519   0.0577   0.0597   0.0627   0.0642
  0.0665   0.0712   0.0723   0.0739   0.0806   0.0832   0.0862   0.0894
  0.0934   0.1008   0.1012   0.1040   0.1078   0.1108   0.1166   0.1188
  0.1218   0.1232   0.1284   0.1345   0.1359   0.1387   0.1426   0.1454
  0.1498   0.1601   0.1625   0.1652   0.1660   0.1705   0.1731   0.1775
```

| | | | | | | | |
|---|---|---|---|---|---|---|---|
| 0.1834 | 0.1909 | 0.1921 | 0.1940 | 0.2034 | 0.2075 | 0.2141 | 0.2268 |
| 0.2349 | 0.2490 | 0.2536 | 0.2670 | 0.2743 | 0.2858 | 0.2993 | 0.3071 |
| 0.3252 | 0.3299 | 0.3331 | 0.3377 | 0.3457 | 0.3564 | 0.3571 | 0.3715 |
| 0.3829 | 0.3849 | 0.3888 | 0.3952 | 0.4073 | 0.4078 | 0.4188 | 0.4262 |
| 0.4280 | 0.4351 | 0.4472 | 0.4555 | 0.4594 | 0.4644 | 0.4661 | 0.4757 |
| 0.4799 | 0.4858 | 0.4959 | 0.5026 | 0.5080 | 0.5111 | 0.5224 | 0.5247 |
| 0.5277 | 0.5285 | 0.5365 | 0.5446 | 0.5457 | 0.5476 | 0.5527 | 0.5586 |
| 0.5657 | 0.5717 | 0.5775 | 0.5851 | 0.5916 | 0.5934 | 0.5979 | 0.6011 |
| 0.6051 | 0.6206 | 0.6254 | 0.6284 | 0.6378 | 0.6426 | 0.6514 | 0.6592 |
| 0.6694 | 0.6716 | 0.6837 | 0.6860 | 0.6920 | 0.7006 | 0.7071 | 0.7102 |
| 0.7133 | 0.7270 | 0.7352 | 0.7412 | 0.7556 | 0.7696 | 0.7826 | 0.7850 |
| 0.7895 | 0.7979 | 0.8113 | 0.8214 | 0.8339 | 0.8423 | 0.8497 | 0.8661 |
| 0.8680 | 0.8775 | 0.8827 | 0.8998 | 0.9086 | 0.9226 | 0.9293 | 0.9410 |
| 0.9493 | 0.9660 | 0.9735 | 0.9898 | 1.0125 | 1.0204 | 1.0323 | 1.0369 |
| 1.0429 | 1.0601 | 1.0663 | 1.0889 | 1.1268 | 1.1476 | 1.1489 | 1.1781 |
| 1.1990 | 1.2090 | 1.2242 | 1.2276 | 1.2593 | 1.2821 | 1.2981 | 1.3045 |
| 1.3133 | 1.3211 | 1.3339 | 1.3453 | 1.3502 | 1.3605 | 1.3712 | 1.3762 |
| 1.3852 | 1.3882 | 1.4035 | 1.4112 | 1.4215 | 1.4258 | 1.4290 | 1.4349 |
| 1.4392 | 1.4503 | 1.4623 | 1.4655 | 1.4679 | 1.4781 | 1.4893 | 1.4958 |
| 1.5057 | 1.5128 | 1.5182 | 1.5295 | 1.5335 | 1.5385 | 1.5440 | 1.5550 |
| 1.5630 | 1.5699 | 1.5729 | 1.5786 | 1.5930 | 1.5937 | 1.6050 | 1.6089 |
| 1.6202 | 1.6267 | 1.6351 | 1.6525 | 1.6593 | 1.6701 | 1.6721 | 1.6864 |
| 1.6971 | 1.7014 | 1.7094 | 1.7281 | 1.7332 | 1.7492 | 1.7579 | 1.7659 |
| 1.7824 | 1.7909 | 1.8087 | 1.8227 | 1.8377 | 1.8484 | 1.8641 | 1.8826 |
| 1.9003 | 1.9052 | 1.9120 | 1.9267 | 1.9525 | 1.9682 | 1.9833 | 1.9997 |
| 2.0033 | 2.0072 | 2.0237 | 2.0271 | 2.0374 | 2.0575 | 2.0675 | 2.0802 |
| 2.1052 | 2.1259 | 2.1328 | 2.1590 | 2.1678 | 2.1918 | 2.2123 | 2.2227 |
| 2.2285 | 2.2367 | 2.2470 | 2.2521 | 2.2566 | 2.2638 | 2.2849 | 2.2899 |
| 2.3126 | 2.3189 | 2.3285 | 2.3538 | 2.3577 | 2.3677 | 2.3880 | 2.3970 |
| 2.4036 | 2.4198 | 2.4369 | 2.4430 | 2.4476 | 2.4615 | 2.4690 | 2.4871 |
| 2.5032 | 2.5141 | 2.5233 | 2.5289 | 2.5421 | 2.5433 | 2.5453 | 2.5629 |
| 2.5729 | 2.5789 | 2.5821 | 2.5915 | 2.5944 | 2.6031 | 2.6119 | 2.6151 |
| 2.6321 | 2.6432 | 2.6651 | 2.6725 | 2.6753 | 2.6794 | 2.6927 | 2.7116 |
| 2.7162 | 2.7298 | 2.7363 | 2.7415 | 2.7567 | 2.7700 | 2.7792 | 2.7854 |
| 2.8064 | 2.8094 | 2.8224 | 2.8371 | 2.8508 | 2.8769 | 2.8831 | 2.8909 |
| 2.8971 | 2.9238 | 2.9570 | 2.9964 | 3.0446 | 3.0610 | 3.1260 | 3.1569 |
| 3.1680 | 3.2314 | 3.2461 | 3.2671 | 3.3069 | 3.3207 | 3.3720 | 3.4092 |
| 3.4227 | 3.4816 | 3.5260 | 3.5319 | 3.5618 | 3.5969 | 3.6132 | 3.6413 |
| 3.6869 | 3.6962 | 3.7084 | 3.7318 | 3.7361 | 3.7493 | 3.7540 | 3.7594 |
| 3.7705 | 3.8344 | 3.8642 | 3.8993 | 3.9443 | 3.9826 | 4.0272 | 4.0874 |
| 4.0915 | 4.2038 | 4.2916 | 4.3103 | 4.5943 | 4.6702 | 4.6945 | 4.7542 |
| 4.9289 | 5.0329 | 5.1985 | 23.5199 | 23.5829 | 23.7173 | 23.7326 | 23.7374 |
| 23.7808 | 23.8033 | 23.8330 | 23.8567 | 23.8901 | 23.9121 | 23.9607 | 23.9813 |
| 24.0291 | 35.4135 | 35.4627 | 35.5261 | 35.6267 | | | |

Ground-State Mulliken Net Atomic Charges

| Atom | | Charge (a.u.) |
|---|---|---|
| 1 | N | 0.381663 |
| 2 | C | 0.170316 |
| 3 | C | -0.026111 |
| 4 | C | -0.214645 |
| 5 | C | -0.296410 |
| 6 | C | -0.026883 |
| 7 | C | -0.808950 |
| 8 | C | -0.134977 |
| 9 | N | -0.158529 |
| 10 | C | -0.215942 |
| 11 | N | 0.012531 |
| 12 | C | 0.249143 |
| 13 | C | -0.163519 |
| 14 | C | 0.074754 |
| 15 | C | -0.312356 |
| 16 | C | -0.258305 |
| 17 | C | -0.536980 |
| 18 | N | -0.490341 |
| 19 | H | 0.221144 |
| 20 | H | 0.237104 |

```
     21 H                    0.233621
     22 H                    0.222377
     23 H                    0.233478
     24 H                    0.237673
     25 H                    0.238039
     26 H                    0.170428
     27 H                    0.251414
     28 H                    0.381470
     29 H                    0.209644
     30 H                    0.212727
     31 H                    0.251733
     32 H                    0.206094
     33 H                    0.224998
     34 H                    0.126328
     35 H                    0.216696
     36 H                    0.179358
     37 H                    0.318234
     38 H                    0.382980
  ----------------------------------------
  Sum of atomic charges =     2.000000

 -----------------------------------------------------------------
                    Cartesian Multipole Moments
 -----------------------------------------------------------------
    Charge (ESU x 10^10)
                 9.6064
    Dipole Moment (Debye)
         X       7.0383      Y       2.4618      Z       2.9115
       Tot       8.0046
    Quadrupole Moments (Debye-Ang)
        XX     -15.7221     XY      -7.0379     YY     -69.3499
        XZ       7.5156     YZ      -8.6356     ZZ     -73.6750
    Traceless Quadrupole Moments (Debye-Ang)
       QXX     111.5808    QYY     -49.3027    QZZ     -62.2780
       QXY     -21.1136    QXZ      22.5468    QYZ     -25.9068
    Octopole Moments (Debye-Ang^2)
       XXX      16.8104    XXY     -96.2513    XYY      20.0779
       YYY      -1.8576    XXZ      55.9374    XYZ      -0.7010
       YYZ     -44.0351    XZZ      21.6900    YZZ      -4.5374
       ZZZ     -10.7909
    Traceless Octopole Moments (Debye-Ang^2)
       XXX    -275.0488    YYY     895.9528    ZZZ    -171.8663
       XXY   -1135.8311    XXZ     835.7267    XYY     125.4343
       XYZ     -10.5143    XZZ     149.6145    YYZ    -663.8604
       YZZ     239.8783
    Hexadecapole Moments (Debye-Ang^3)
      XXXX   -3543.0905   XXXY    -120.1322   XXYY    -766.6978
      XYYY      82.5716   YYYY   -1006.1282   XXXZ      35.9186
      XXYZ     -14.6369   XYYZ     -34.3584   YYYZ     -42.7806
      XXZZ    -708.4051   XYZZ      24.4726   YYZZ    -246.0252
      XZZZ    -162.2022   YZZZ      26.7418   ZZZZ    -691.7884
    Traceless Hexadecapole Moments (Debye-Ang^3)
      XXXX    1463.5350   XXXY  -12024.9208   XXXZ   11000.3404
      XXYY    -997.3933   XXYZ   -1065.6094   XXZZ    -466.1417
      XYYY    9258.9778   XYYZ   -1198.0062   XYZZ    2765.9431
      XZZZ   -9802.3342   YYYY   -2096.2213   YYYZ   -3078.1746
      YYZZ    3093.6146   YZZZ    4143.7840   ZZZZ   -2627.4728
 -----------------------------------------------------------------

 --------------------------------------------------
 -  Entering drvman on Sun Oct 13 21:06:49 2024  -
 --------------------------------------------------

 Calculating analytic gradient of the SCF energy
 Gradient of SCF Energy
            1           2           3           4           5           6
    1   0.0000899  -0.0001809   0.0001864  -0.0000199   0.0001264  -0.0001714
    2   0.0000356  -0.0001682  -0.0001291  -0.0000218  -0.0002360   0.0002363
    3  -0.0001410   0.0000563   0.0001055   0.0002211  -0.0001919  -0.0001712
            7           8           9          10          11          12
```

```
    1  -0.0002350   0.0000113   0.0002279  -0.0000917  -0.0003145   0.0006633
    2   0.0001648   0.0002540  -0.0006070   0.0005303  -0.0002945   0.0000924
    3  -0.0000141   0.0002491   0.0005466  -0.0005441   0.0001595  -0.0015744
           13          14          15          16          17          18
    1   0.0004849  -0.0005404  -0.0000888  -0.0001058   0.0002137  -0.0008547
    2  -0.0001448   0.0003331  -0.0001271  -0.0000165   0.0001370   0.0000277
    3   0.0000945   0.0000740   0.0000400   0.0000241  -0.0000672   0.0007006
           19          20          21          22          23          24
    1   0.0000751   0.0000027  -0.0000233  -0.0000286  -0.0001464  -0.0003551
    2  -0.0000320  -0.0000239  -0.0000843   0.0000376   0.0000929   0.0003533
    3  -0.0000327  -0.0000537   0.0000443  -0.0000344  -0.0001018   0.0000682
           25          26          27          28          29          30
    1   0.0000689   0.0005606   0.0001263   0.0000993  -0.0000191   0.0000342
    2  -0.0000263   0.0001415  -0.0002595  -0.0000198   0.0000653   0.0000389
    3   0.0000700   0.0004447   0.0000414   0.0000443  -0.0000477   0.0000464
           31          32          33          34          35          36
    1  -0.0000269   0.0000214  -0.0000021   0.0000485   0.0000008  -0.0000290
    2  -0.0000030  -0.0000047  -0.0000230   0.0001048  -0.0000269  -0.0000254
    3  -0.0000884  -0.0000301   0.0000440   0.0001103   0.0000336   0.0000444
           37          38
    1   0.0001407   0.0000510
    2  -0.0006444   0.0002726
    3  -0.0002295   0.0000592
 Max gradient component =       1.574E-03
 RMS gradient           =       2.757E-04
 Gradient time:  CPU 161.56 s  wall 269.68 s

-------------------------------------------------
-  Entering optman on Sun Oct 13 21:11:19 2024  -
-------------------------------------------------

 Geometry Optimization Parameters
   NAtoms,    NIC,     NZ,  NCons,   NDum,   NFix, NCnnct, MaxDiis
       38     272       0       0       0       0       0       0

 Cartesian Hessian Update
 Hessian updated using BFGS update


** GEOMETRY OPTIMIZATION IN DELOCALIZED INTERNAL COORDINATES **
   Searching for a Minimum

   Optimization Cycle:   8

                    Coordinates (Angstroms)
     ATOM                X               Y               Z
      1  N         2.5387162677    0.4094621303    0.4579043040
      2  C         2.7007046939   -0.5205252481   -0.5273260615
      3  C         3.5360866394   -1.5995162586   -0.2764136014
      4  C         4.1931708165   -1.7273204207    0.9374382306
      5  C         4.0143976722   -0.7546656226    1.9174993028
      6  C         3.1853186070    0.3039201105    1.6472001285
      7  C         2.0036854495   -0.3429713634   -1.8382957049
      8  C         1.6695091052    1.5876290873    0.2427176978
      9  N        -1.6453341918    0.1302051878    1.2507615272
     10  C        -2.5072902790    0.6866558883    0.3549054756
     11  N        -2.0734070302    1.5342718997   -0.5375847001
     12  C        -0.7722082836    1.8767139863   -0.5833932687
     13  C         0.1908573726    1.2603339698    0.2913595712
     14  C        -0.3201063034    0.3996308109    1.2121845091
     15  C        -3.9352437209    0.2664166794    0.3786094885
     16  C        -4.1999059774   -0.8559385684   -0.6516347595
     17  C        -3.4165398207   -2.1354666457   -0.3793663782
     18  N        -0.4575638865    2.8008101415   -1.4808538250
     19  H         3.6741693356   -2.3374792020   -1.0563367774
     20  H         4.8470972523   -2.5730884551    1.1159085575
     21  H         4.5170299416   -0.8076417424    2.8743122663
     22  H         3.0161257909    1.1021076074    2.3585860751
     23  H         2.2427631808   -1.1744779711   -2.4982284667
     24  H         2.3232449990    0.5759846554   -2.3387205854
```

```
    25  H          0.9183384815   -0.3102697150   -1.7179517863
    26  H          1.9520509929    2.0205697636   -0.7160007589
    27  H          1.9279333496    2.3238903054    1.0047511718
    28  H         -2.0072665662   -0.5233263837    1.9345390126
    29  H          0.2897643461   -0.1152517455    1.9450041773
    30  H         -4.2094348474   -0.0726271850    1.3823804022
    31  H         -4.5405168457    1.1411502648    0.1371200331
    32  H         -3.9831877247   -0.4743881800   -1.6528823814
    33  H         -5.2714825349   -1.0617248712   -0.6203715420
    34  H         -2.3348189066   -1.9913507672   -0.4862131028
    35  H         -3.7023080196   -2.9154873249   -1.0860345921
    36  H         -3.6174026939   -2.5224369233    0.6251000315
    37  H          0.4579852103    3.2046343002   -1.5807891357
    38  H         -1.2049318725    3.1915678053   -2.0398845347
   Point Group: c1    Number of degrees of freedom:   108


   Energy is   -763.969208449

 Hessian updated using BFGS update
  internal optimization (0)

108 Hessian modes will be used to form the next step
  Hessian Eigenvalues:
     0.000806    0.002601    0.003700    0.004185    0.007224    0.011133
     0.018350    0.019245    0.019343    0.020056    0.021592    0.022428
     0.022693    0.023084    0.023953    0.024529    0.025768    0.026571
     0.027655    0.028261    0.028641    0.029873    0.032959    0.036685
     0.038798    0.041840    0.043244    0.043708    0.044589    0.045651
     0.052946    0.054014    0.054585    0.055389    0.062066    0.079715
     0.085503    0.094080    0.121679    0.122209    0.127132    0.131164
     0.131968    0.132973    0.136500    0.140996    0.143005    0.144542
     0.147693    0.147974    0.149961    0.152711    0.153002    0.153706
     0.156926    0.161711    0.194072    0.206286    0.210404    0.217275
     0.225199    0.232536    0.239342    0.247675    0.251855    0.259981
     0.267553    0.272599    0.281901    0.298853    0.300180    0.300580
     0.300704    0.301242    0.301848    0.302785    0.304163    0.304956
     0.305273    0.305572    0.307580    0.308126    0.312376    0.319491
     0.321415    0.332021    0.333167    0.335509    0.337084    0.341841
     0.343204    0.355297    0.365131    0.366119    0.382894    0.390982
     0.400020    0.407242    0.417609    0.419987    0.421794    0.431127
     0.443734    0.451841    0.471518    0.502598    0.575570    0.746860

 Minimum search - taking simple RFO step
 Searching for Lamda that Minimizes Along All modes
 Value Taken    Lamda =  -0.00084024
 Calculated Step too Large.  Step scaled by  0.469417
 Step Taken.  Stepsize is  0.300000

                             Maximum     Tolerance    Cnvgd?
         Gradient           0.003051      0.000800      NO
         Displacement       0.143681      0.001400      NO
         Energy change     -0.000383      0.000228      NO


 New Cartesian Coordinates Obtained by Inverse Iteration

 Displacement from previous Coordinates is:  0.892247
 ----------------------------------------------------------------
             Standard Nuclear Orientation (Angstroms)
    I     Atom           X                Y                Z
 ----------------------------------------------------------------
    1      N       2.5439165040     0.4129952927     0.4377360534
    2      C       2.7227635204    -0.5396537008    -0.5228438565
    3      C       3.5552023287    -1.6107357563    -0.2336914178
    4      C       4.1959255147    -1.7061083542     0.9920201941
    5      C       4.0029849321    -0.7095622719     1.9450607631
    6      C       3.1749270799     0.3396314328     1.6378107798
    7      C       2.0539727715    -0.3886254677    -1.8519209784
    8      C       1.6668844504     1.5756431041     0.1816984987
```

```
    9      N      -1.6223209160     0.0828489484     1.2329761552
   10      C      -2.5056036003     0.6746646278     0.3827519442
   11      N      -2.0937106878     1.5555158269    -0.4886928417
   12      C      -0.7926155173     1.8933858726    -0.5572369307
   13      C       0.1910975186     1.2421007462     0.2668277784
   14      C      -0.2969729630     0.3485677904     1.1684411626
   15      C      -3.9341001913     0.2576539511     0.4281166449
   16      C      -4.2285814423    -0.8252592378    -0.6354730347
   17      C      -3.4431289824    -2.1161211100    -0.4316255398
   18      N      -0.4966600322     2.8550173753    -1.4222530458
   19      H       3.7059725725    -2.3678831960    -0.9926983328
   20      H       4.8488867760    -2.5458310885     1.2007788794
   21      H       4.4938075899    -0.7378320421     2.9090372831
   22      H       2.9926780704     1.1540924022     2.3273451205
   23      H       2.2505176395    -1.2658508472    -2.4652373261
   24      H       2.4440895377     0.4809651867    -2.3904462784
   25      H       0.9715193305    -0.2790906383    -1.7559960390
   26      H       1.9300547876     1.9614267758    -0.8027617325
   27      H       1.9315243617     2.3500372834     0.9032849258
   28      H      -1.9688028834    -0.5958891226     1.9000532200
   29      H       0.3296006109    -0.1957582577     1.8648642286
   30      H      -4.1845553451    -0.1163791030     1.4256370425
   31      H      -4.5422623640     1.1421343060     0.2338064333
   32      H      -4.0370319687    -0.4080700882    -1.6275108793
   33      H      -5.2995301591    -1.0306054222    -0.5835868177
   34      H      -2.3646602417    -1.9709235968    -0.5655045997
   35      H      -3.7525309901    -2.8692970962    -1.1572403739
   36      H      -3.6157235763    -2.5383469025     0.5638649753
   37      H       0.4250499112     3.2356535186    -1.5542619410
   38      H      -1.2525839474     3.2554888591    -1.9631301170
 ----------------------------------------------------------------
 Nuclear Repulsion Energy =        1322.11616742 hartrees
 There are       65 alpha and       65 beta electrons

--------------------------------------------------
-  Entering fldman on Sun Oct 13 21:11:19 2024  -
--------------------------------------------------

 Applying Cartesian multipole field
    Component          Value
    ---------          -----
     (2,0,0)        1.00000E-12
     (0,2,0)        2.00000E-11
     (0,0,2)       -3.00000E-11
 Nucleus-field energy     =     0.0000000049 hartrees

--------------------------------------------------
-  Entering gesman on Sun Oct 13 21:11:19 2024  -
--------------------------------------------------

 Requested basis set is 6-311+G(d,p)
 There are 188 shells and 516 basis functions
 A cutoff of  1.0D-12 yielded  13077 shell pairs
 There are    103293 function pairs (    110073 Cartesian)
 Smallest overlap matrix eigenvalue = 2.20E-06
 Linear dependence detected in AO basis
 Tighter screening thresholds may be required for diffuse basis sets
 Use S2THRESH > 12 and THRESH = 14 in case of SCF convergence issues
 Number of orthogonalized atomic orbitals = 510
 Maximum deviation from orthogonality = 2.854E-11
 Guess MOs from SCF MO coefficient file
 Reading MOs from coefficient file
 Reading MOs from coefficient file

--------------------------------------------------
-  Entering scfman on Sun Oct 13 21:11:19 2024  -
--------------------------------------------------

 Long-range K will be added via erf
 Coulomb attenuation parameter = 0.2 bohr**(-1)
```

```
 A restricted hybrid HF-DFT SCF calculation will be
 performed using Pulay DIIS + Geometric Direct Minimization
 Exchange:     0.2220 Hartree-Fock + 1.0000 wB97X-D + LR-HF
 Correlation:  1.0000 wB97X-D
 Using Euler-Maclaurin-Lebedev (75,302) quadrature formula
 Dispersion:   Grimme D
 SCF converges when RMS gradient is below 1.0E-07
 Geometry optimization detected.  Setting ReadMinima to 0
 Setting SaveMinima to 0
 ---------------------------------------
  Cycle       Energy         DIIS Error
 ---------------------------------------
    1    -763.9615242009      7.99E-04
    2    -763.9688798389      9.21E-05
    3    -763.9694747620      4.55E-05
    4    -763.9695393629      2.72E-05
    5    -763.9695657648      7.90E-06
    6    -763.9695679313      2.63E-06
    7    -763.9695682350      6.82E-07
    8    -763.9695682664      3.30E-07
    9    -763.9695682715      8.94E-08 Convergence criterion met
 ---------------------------------------
 SCF time:  CPU 306.88 s  wall 503.99 s
 SCF   energy in the final basis set = -763.96956827
 Total energy in the final basis set = -763.96956827

--------------------------------------------------
-  Entering anlman on Sun Oct 13 21:19:43 2024  -
--------------------------------------------------



 --------------------------------------------------------------


                    Orbital Energies (a.u.)
 --------------------------------------------------------------


 Alpha MOs
 -- Occupied --
-14.8205 -14.7879 -14.7372 -14.7052 -10.6759 -10.6595 -10.6459 -10.6431
-10.6384 -10.6304 -10.6065 -10.5878 -10.5762 -10.5752 -10.5539 -10.5252
-10.4966 -10.4760  -1.4147  -1.4086  -1.3467  -1.2741  -1.2328  -1.2003
 -1.1924  -1.1429  -1.1032  -1.0877  -1.0542  -1.0536  -1.0371  -1.0078
 -0.9924  -0.9547  -0.9470  -0.9174  -0.9070  -0.8946  -0.8858  -0.8626
 -0.8564  -0.8473  -0.8377  -0.8255  -0.8082  -0.8019  -0.7917  -0.7843
 -0.7752  -0.7574  -0.7544  -0.7529  -0.7457  -0.7329  -0.7224  -0.7213
 -0.7030  -0.6695  -0.6629  -0.6453  -0.6366  -0.6305  -0.6253  -0.6238
 -0.6078
 -- Virtual --
 -0.2738  -0.2579  -0.2398  -0.2291  -0.1742  -0.1655  -0.1500  -0.1456
 -0.1364  -0.1199  -0.1168  -0.1156  -0.1086  -0.1073  -0.1013  -0.0987
 -0.0921  -0.0862  -0.0853  -0.0785  -0.0747  -0.0719  -0.0714  -0.0689
 -0.0637  -0.0594  -0.0578  -0.0539  -0.0514  -0.0508  -0.0403  -0.0380
 -0.0331  -0.0282  -0.0264  -0.0238  -0.0184  -0.0147  -0.0106  -0.0087
 -0.0047  -0.0018  -0.0008   0.0011   0.0045   0.0077   0.0122   0.0142
  0.0177   0.0188   0.0240   0.0312   0.0330   0.0362   0.0389   0.0410
  0.0431   0.0440   0.0480   0.0516   0.0586   0.0598   0.0626   0.0644
  0.0664   0.0704   0.0719   0.0755   0.0804   0.0831   0.0868   0.0885
  0.0955   0.0997   0.1018   0.1039   0.1069   0.1099   0.1172   0.1195
  0.1213   0.1219   0.1290   0.1348   0.1369   0.1393   0.1425   0.1456
  0.1486   0.1600   0.1629   0.1646   0.1666   0.1707   0.1732   0.1761
  0.1844   0.1900   0.1917   0.1951   0.2012   0.2074   0.2168   0.2265
  0.2349   0.2504   0.2540   0.2696   0.2752   0.2887   0.3014   0.3072
  0.3249   0.3302   0.3336   0.3385   0.3444   0.3578   0.3582   0.3685
  0.3825   0.3847   0.3904   0.3930   0.4053   0.4070   0.4177   0.4224
  0.4268   0.4324   0.4475   0.4549   0.4601   0.4638   0.4673   0.4724
  0.4826   0.4873   0.4950   0.5018   0.5053   0.5094   0.5221   0.5238
  0.5251   0.5278   0.5363   0.5445   0.5448   0.5466   0.5525   0.5587
  0.5653   0.5696   0.5763   0.5846   0.5889   0.5967   0.5970   0.6034
  0.6049   0.6186   0.6247   0.6290   0.6387   0.6400   0.6490   0.6587
  0.6683   0.6723   0.6863   0.6890   0.6911   0.6993   0.7063   0.7100
```

| | | | | | | | |
|---|---|---|---|---|---|---|---|
| 0.7116 | 0.7290 | 0.7338 | 0.7400 | 0.7567 | 0.7696 | 0.7813 | 0.7878 |
| 0.7926 | 0.7995 | 0.8063 | 0.8203 | 0.8297 | 0.8431 | 0.8489 | 0.8652 |
| 0.8697 | 0.8799 | 0.8843 | 0.9029 | 0.9149 | 0.9231 | 0.9275 | 0.9457 |
| 0.9501 | 0.9679 | 0.9715 | 0.9887 | 1.0163 | 1.0235 | 1.0323 | 1.0356 |
| 1.0441 | 1.0603 | 1.0664 | 1.0908 | 1.1297 | 1.1476 | 1.1522 | 1.1776 |
| 1.2034 | 1.2084 | 1.2215 | 1.2287 | 1.2591 | 1.2825 | 1.2965 | 1.3020 |
| 1.3106 | 1.3143 | 1.3345 | 1.3442 | 1.3507 | 1.3608 | 1.3699 | 1.3754 |
| 1.3835 | 1.3876 | 1.4018 | 1.4112 | 1.4211 | 1.4257 | 1.4291 | 1.4330 |
| 1.4392 | 1.4506 | 1.4580 | 1.4613 | 1.4667 | 1.4779 | 1.4882 | 1.4910 |
| 1.5079 | 1.5138 | 1.5189 | 1.5304 | 1.5348 | 1.5380 | 1.5433 | 1.5536 |
| 1.5631 | 1.5678 | 1.5725 | 1.5764 | 1.5933 | 1.5941 | 1.6037 | 1.6128 |
| 1.6169 | 1.6279 | 1.6320 | 1.6565 | 1.6606 | 1.6705 | 1.6729 | 1.6870 |
| 1.6982 | 1.6986 | 1.7126 | 1.7277 | 1.7307 | 1.7493 | 1.7574 | 1.7658 |
| 1.7808 | 1.7917 | 1.8093 | 1.8228 | 1.8375 | 1.8455 | 1.8628 | 1.8840 |
| 1.8991 | 1.9042 | 1.9111 | 1.9287 | 1.9539 | 1.9662 | 1.9849 | 2.0026 |
| 2.0045 | 2.0110 | 2.0210 | 2.0288 | 2.0405 | 2.0545 | 2.0687 | 2.0829 |
| 2.1093 | 2.1257 | 2.1334 | 2.1594 | 2.1694 | 2.1907 | 2.2117 | 2.2239 |
| 2.2294 | 2.2370 | 2.2471 | 2.2523 | 2.2583 | 2.2625 | 2.2827 | 2.2909 |
| 2.3118 | 2.3153 | 2.3256 | 2.3527 | 2.3578 | 2.3650 | 2.3881 | 2.3985 |
| 2.4032 | 2.4238 | 2.4371 | 2.4460 | 2.4464 | 2.4631 | 2.4688 | 2.4872 |
| 2.5009 | 2.5129 | 2.5225 | 2.5291 | 2.5405 | 2.5431 | 2.5483 | 2.5629 |
| 2.5718 | 2.5788 | 2.5854 | 2.5916 | 2.5952 | 2.6038 | 2.6091 | 2.6154 |
| 2.6239 | 2.6431 | 2.6661 | 2.6714 | 2.6753 | 2.6834 | 2.6924 | 2.7115 |
| 2.7192 | 2.7289 | 2.7348 | 2.7422 | 2.7513 | 2.7701 | 2.7805 | 2.7878 |
| 2.8064 | 2.8091 | 2.8199 | 2.8357 | 2.8551 | 2.8771 | 2.8845 | 2.8930 |
| 2.8993 | 2.9076 | 2.9477 | 2.9962 | 3.0485 | 3.0582 | 3.1148 | 3.1507 |
| 3.1682 | 3.2302 | 3.2465 | 3.2621 | 3.3053 | 3.3225 | 3.3742 | 3.4075 |
| 3.4219 | 3.4810 | 3.5267 | 3.5311 | 3.5629 | 3.5956 | 3.6151 | 3.6430 |
| 3.6870 | 3.6927 | 3.7060 | 3.7319 | 3.7358 | 3.7468 | 3.7518 | 3.7589 |
| 3.7713 | 3.8340 | 3.8638 | 3.8985 | 3.9448 | 3.9817 | 4.0272 | 4.0842 |
| 4.0915 | 4.2044 | 4.2909 | 4.3112 | 4.5939 | 4.6703 | 4.6920 | 4.7610 |
| 4.9287 | 5.0314 | 5.1995 | 23.5209 | 23.5836 | 23.7189 | 23.7317 | 23.7376 |
| 23.7806 | 23.8037 | 23.8323 | 23.8567 | 23.8904 | 23.9122 | 23.9605 | 23.9809 |
| 24.0284 | 35.4119 | 35.4630 | 35.5258 | 35.6263 | | | |

Ground-State Mulliken Net Atomic Charges

| Atom | Charge (a.u.) |
|---|---|
| 1 N | 0.363580 |
| 2 C | 0.166897 |
| 3 C | -0.024673 |
| 4 C | -0.206895 |
| 5 C | -0.304665 |
| 6 C | 0.001698 |
| 7 C | -0.815982 |
| 8 C | -0.136750 |
| 9 N | -0.156681 |
| 10 C | -0.215040 |
| 11 N | 0.008326 |
| 12 C | 0.251002 |
| 13 C | -0.161922 |
| 14 C | 0.058843 |
| 15 C | -0.311422 |
| 16 C | -0.260422 |
| 17 C | -0.535484 |
| 18 N | -0.483628 |
| 19 H | 0.221800 |
| 20 H | 0.237326 |
| 21 H | 0.233893 |
| 22 H | 0.223567 |
| 23 H | 0.231732 |
| 24 H | 0.247150 |
| 25 H | 0.232322 |
| 26 H | 0.174473 |
| 27 H | 0.253481 |
| 28 H | 0.380302 |
| 29 H | 0.209179 |
| 30 H | 0.212880 |

```
    31 H                      0.251631
    32 H                      0.206374
    33 H                      0.224904
    34 H                      0.126666
    35 H                      0.216659
    36 H                      0.178870
    37 H                      0.316640
    38 H                      0.383373
  ----------------------------------------
  Sum of atomic charges =     2.000000

 -----------------------------------------------------------------
                    Cartesian Multipole Moments
 -----------------------------------------------------------------
    Charge (ESU x 10^10)
                 9.6064
    Dipole Moment (Debye)
         X       7.1346      Y       2.3800      Z       2.8323
       Tot       8.0368
    Quadrupole Moments (Debye-Ang)
        XX     -15.5128     XY      -6.9908     YY     -68.9962
        XZ       7.6559     YZ      -8.9624     ZZ     -74.0952
    Traceless Quadrupole Moments (Debye-Ang)
       QXX     112.0659    QYY     -48.3843    QZZ     -63.6816
       QXY     -20.9725    QXZ      22.9676    QYZ     -26.8872
    Octopole Moments (Debye-Ang^2)
       XXX      17.4468    XXY     -95.6852    XYY      19.2097
       YYY      -0.3302    XXZ      55.8832    XYZ      -0.5850
       YYZ     -44.3519    XZZ      23.0463    YZZ      -5.8828
       ZZZ     -12.8045
    Traceless Octopole Moments (Debye-Ang^2)
       XXX    -275.6231    YYY     912.1313    ZZZ    -180.6089
       XXY   -1129.5836    XXZ     842.0678    XYY     109.0366
       XYZ      -8.7752    XZZ     166.5865    YYZ    -661.4590
       YZZ     217.4523
    Hexadecapole Moments (Debye-Ang^3)
      XXXX   -3569.5181   XXXY    -112.1177   XXYY    -773.4535
      XYYY      79.9927   YYYY   -1003.0963   XXXZ      37.5006
      XXYZ     -16.5539   XYYZ     -32.6187   YYYZ     -47.5976
      XXZZ    -707.5431   XYZZ      25.7371   YYZZ    -246.1053
      XZZZ    -160.6620   YZZZ      26.5262   ZZZZ    -690.2391
    Traceless Hexadecapole Moments (Debye-Ang^3)
      XXXX    1293.4000   XXXY  -11484.9027   XXXZ   10947.6671
      XXYY   -1266.2385   XXYZ   -1165.8830   XXZZ     -27.1615
      XYYY    8686.6900   XYYZ   -1088.2618   XYZZ    2798.2127
      XZZZ   -9859.4053   YYYY   -1739.6719   YYYZ   -3280.9339
      YYZZ    3005.9104   YZZZ    4446.8170   ZZZZ   -2978.7489
 -----------------------------------------------------------------

--------------------------------------------------
-  Entering drvman on Sun Oct 13 21:19:43 2024  -
--------------------------------------------------

 Calculating analytic gradient of the SCF energy
 Gradient of SCF Energy
            1           2           3           4           5           6
    1  -0.0000281   0.0000834  -0.0002140  -0.0000984   0.0000800   0.0000311
    2  -0.0000159  -0.0003177   0.0001259   0.0001895  -0.0001254   0.0000386
    3  -0.0000400   0.0002129  -0.0002131   0.0004045  -0.0001098  -0.0003668
            7           8           9          10          11          12
    1  -0.0002614   0.0000386   0.0002319   0.0002294  -0.0004674   0.0008369
    2   0.0001841   0.0005121  -0.0004196  -0.0000595   0.0001855  -0.0011902
    3  -0.0000139   0.0000972   0.0004904  -0.0001222  -0.0001098  -0.0010095
           13          14          15          16          17          18
    1  -0.0000252  -0.0006373  -0.0000338   0.0000679   0.0000981  -0.0006269
    2   0.0000867   0.0001939  -0.0003161   0.0003227   0.0000704   0.0013692
    3  -0.0002233   0.0002593   0.0000783  -0.0001563   0.0000855   0.0010729
           19          20          21          22          23          24
    1   0.0001137   0.0000598   0.0000075   0.0000528  -0.0001176  -0.0001761
    2  -0.0001072  -0.0000562  -0.0001331   0.0000006   0.0000746   0.0002814
```

```
    3  -0.0000223  -0.0000310   0.0000476   0.0000549  -0.0001561  -0.0000372
          25          26          27          28          29          30
    1   0.0000601   0.0006130   0.0000412   0.0000202  -0.0000987  -0.0000523
    2  -0.0000139   0.0000633  -0.0001841  -0.0000735   0.0000694   0.0001336
    3   0.0000360   0.0003776   0.0000340   0.0000366   0.0000696   0.0000758
          31          32          33          34          35          36
    1  -0.0001014   0.0000402  -0.0000142  -0.0000140   0.0000064  -0.0000018
    2   0.0000396  -0.0000834  -0.0001651   0.0001669  -0.0000383  -0.0000155
    3  -0.0000770  -0.0000136   0.0000636   0.0000815   0.0000039   0.0000277
          37          38
    1   0.0003988  -0.0001422
    2  -0.0008110   0.0000179
    3  -0.0005004  -0.0004073
 Max gradient component =       1.369E-03
 RMS gradient           =       3.161E-04
 Gradient time:  CPU 134.73 s  wall 213.71 s

-------------------------------------------------
-  Entering optman on Sun Oct 13 21:23:17 2024  -
-------------------------------------------------

 Geometry Optimization Parameters
   NAtoms,    NIC,     NZ,  NCons,   NDum,   NFix, NCnnct, MaxDiis
       38     272       0       0       0       0       0       0

 Cartesian Hessian Update
 Hessian updated using BFGS update


** GEOMETRY OPTIMIZATION IN DELOCALIZED INTERNAL COORDINATES **
   Searching for a Minimum

   Optimization Cycle:   9

                       Coordinates (Angstroms)
     ATOM                X               Y               Z
      1  N         2.5439165040    0.4129952927    0.4377360534
      2  C         2.7227635204   -0.5396537008   -0.5228438565
      3  C         3.5552023287   -1.6107357563   -0.2336914178
      4  C         4.1959255147   -1.7061083542    0.9920201941
      5  C         4.0029849321   -0.7095622719    1.9450607631
      6  C         3.1749270799    0.3396314328    1.6378107798
      7  C         2.0539727715   -0.3886254677   -1.8519209784
      8  C         1.6668844504    1.5756431041    0.1816984987
      9  N        -1.6223209160    0.0828489484    1.2329761552
     10  C        -2.5056036003    0.6746646278    0.3827519442
     11  N        -2.0937106878    1.5555158269   -0.4886928417
     12  C        -0.7926155173    1.8933858726   -0.5572369307
     13  C         0.1910975186    1.2421007462    0.2668277784
     14  C        -0.2969729630    0.3485677904    1.1684411626
     15  C        -3.9341001913    0.2576539511    0.4281166449
     16  C        -4.2285814423   -0.8252592378   -0.6354730347
     17  C        -3.4431289824   -2.1161211100   -0.4316255398
     18  N        -0.4966600322    2.8550173753   -1.4222530458
     19  H         3.7059725725   -2.3678831960   -0.9926983328
     20  H         4.8488867760   -2.5458310885    1.2007788794
     21  H         4.4938075899   -0.7378320421    2.9090372831
     22  H         2.9926780704    1.1540924022    2.3273451205
     23  H         2.2505176395   -1.2658508472   -2.4652373261
     24  H         2.4440895377    0.4809651867   -2.3904462784
     25  H         0.9715193305   -0.2790906383   -1.7559960390
     26  H         1.9300547876    1.9614267758   -0.8027617325
     27  H         1.9315243617    2.3500372834    0.9032849258
     28  H        -1.9688028834   -0.5958891226    1.9000532200
     29  H         0.3296006109   -0.1957582577    1.8648642286
     30  H        -4.1845553451   -0.1163791030    1.4256370425
     31  H        -4.5422623640    1.1421343060    0.2338064333
     32  H        -4.0370319687   -0.4080700882   -1.6275108793
     33  H        -5.2995301591   -1.0306054222   -0.5835868177
     34  H        -2.3646602417   -1.9709235968   -0.5655045997
```

```
   35  H         -3.7525309901   -2.8692970962   -1.1572403739
   36  H         -3.6157235763   -2.5383469025    0.5638649753
   37  H          0.4250499112    3.2356535186   -1.5542619410
   38  H         -1.2525839474    3.2554888591   -1.9631301170
 Point Group: c1    Number of degrees of freedom:   108


 Energy is   -763.969568272

Hessian updated using BFGS update
 internal optimization (0)

108 Hessian modes will be used to form the next step
 Hessian Eigenvalues:
    0.000655    0.002610    0.003279    0.004183    0.007270    0.011411
    0.017481    0.019147    0.019416    0.020035    0.021165    0.022611
    0.022722    0.023031    0.024092    0.024488    0.025756    0.026763
    0.027545    0.028259    0.028992    0.029868    0.033100    0.036742
    0.038863    0.041611    0.043221    0.043680    0.044792    0.045659
    0.053895    0.054059    0.055276    0.057611    0.060943    0.079987
    0.085507    0.093658    0.121726    0.122198    0.127134    0.131185
    0.132032    0.132960    0.136483    0.141279    0.143143    0.144347
    0.147284    0.147709    0.149938    0.152816    0.153150    0.154064
    0.158130    0.162272    0.193993    0.206302    0.210452    0.217295
    0.225918    0.232067    0.238957    0.247697    0.252113    0.260066
    0.267847    0.272786    0.281900    0.299035    0.300185    0.300629
    0.300884    0.301379    0.301877    0.303115    0.304162    0.305009
    0.305217    0.305603    0.307589    0.308120    0.312458    0.319559
    0.320832    0.332107    0.333362    0.335537    0.337088    0.341496
    0.343081    0.355247    0.363482    0.365993    0.382902    0.391156
    0.399784    0.407392    0.418077    0.420760    0.422056    0.435490
    0.444175    0.458787    0.470660    0.507874    0.571993    0.727243

Minimum search - taking simple RFO step
Searching for Lamda that Minimizes Along All modes
Value Taken    Lamda =  -0.00091472
Calculated Step too Large.  Step scaled by  0.436844
Step Taken.  Stepsize is  0.300000

                           Maximum     Tolerance    Cnvgd?
        Gradient           0.003077     0.000800      NO
        Displacement       0.124207     0.001400      NO
        Energy change     -0.000360     0.000228      NO


New Cartesian Coordinates Obtained by Inverse Iteration

Displacement from previous Coordinates is:  1.024169
----------------------------------------------------------------
             Standard Nuclear Orientation (Angstroms)
   I     Atom           X                Y                Z
----------------------------------------------------------------
    1      N       2.5518326569     0.4174712405     0.4136215562
    2      C       2.7511734580    -0.5607578333    -0.5167562380
    3      C       3.5831487688    -1.6196809429    -0.1834582749
    4      C       4.2066325432    -1.6749121743     1.0533219242
    5      C       3.9954475386    -0.6512234156     1.9734464281
    6      C       3.1657012099     0.3838378964     1.6244682956
    7      C       2.1100698602    -0.4473706184    -1.8631306394
    8      C       1.6632997959     1.5583756701     0.1121661283
    9      N      -1.5973786664     0.0257243004     1.2041580949
   10      C      -2.5023254762     0.6598737129     0.4103210022
   11      N      -2.1125643688     1.5776675882    -0.4332880317
   12      C      -0.8120795119     1.9095517979    -0.5262831860
   13      C       0.1922629099     1.2176474334     0.2368400730
   14      C      -0.2721207464     0.2857883213     1.1107946892
   15      C      -3.9315697661     0.2489056391     0.4812953784
   16      C      -4.2657125199    -0.7884315554    -0.6153140359
   17      C      -3.4841599404    -2.0915307207    -0.4899575566
   18      N      -0.5333595044     2.9110146353    -1.3521833232
```

```
  19      H        3.7482781521    -2.3983643120    -0.9173377735
  20      H        4.8600617748    -2.5048249343     1.2970091195
  21      H        4.4732501699    -0.6483891413     2.9444013068
  22      H        2.9667159698     1.2160571356     2.2878006736
  23      H        2.2795717867    -1.3612642164    -2.4293018071
  24      H        2.5542543834     0.3752092705    -2.4335672668
  25      H        1.0326987098    -0.2814104948    -1.7975513317
  26      H        1.9041681153     1.8930631083    -0.8969341690
  27      H        1.9309658878     2.3725249053     0.7882421018
  28      H       -1.9283357434    -0.6815102295     1.8492126427
  29      H        0.3712764337    -0.2943946099     1.7612726037
  30      H       -4.1549047691    -0.1639553932     1.4698866400
  31      H       -4.5393590327     1.1437489640     0.3412903180
  32      H       -4.1023417493    -0.3322922682    -1.5951842644
  33      H       -5.3359963428    -0.9896467715    -0.5385117405
  34      H       -2.4098183708    -1.9483083971    -0.6551030618
  35      H       -3.8243128722    -2.8126731630    -1.2341075494
  36      H       -3.6260792141    -2.5523176565     0.4932683431
  37      H        0.3902136441     3.2792796359    -1.5071940851
  38      H       -1.2986051743     3.3275175932    -1.8676529844
 ----------------------------------------------------------------
 Nuclear Repulsion Energy =        1320.96908386 hartrees
 There are       65 alpha and       65 beta electrons

-------------------------------------------------
-  Entering fldman on Sun Oct 13 21:23:17 2024  -
-------------------------------------------------

 Applying Cartesian multipole field
    Component          Value
    ---------          -----
     (2,0,0)        1.00000E-12
     (0,2,0)        2.00000E-11
     (0,0,2)       -3.00000E-11
 Nucleus-field energy     =     0.0000000053 hartrees

-------------------------------------------------
-  Entering gesman on Sun Oct 13 21:23:17 2024  -
-------------------------------------------------

 Requested basis set is 6-311+G(d,p)
 There are 188 shells and 516 basis functions
 A cutoff of  1.0D-12 yielded  13053 shell pairs
 There are    103119 function pairs (    109889 Cartesian)
 Smallest overlap matrix eigenvalue = 2.23E-06
 Linear dependence detected in AO basis
 Tighter screening thresholds may be required for diffuse basis sets
 Use S2THRESH > 12 and THRESH = 14 in case of SCF convergence issues
 Number of orthogonalized atomic orbitals = 510
 Maximum deviation from orthogonality = 2.376E-11
 Guess MOs from SCF MO coefficient file
 Reading MOs from coefficient file
 Reading MOs from coefficient file

-------------------------------------------------
-  Entering scfman on Sun Oct 13 21:23:17 2024  -
-------------------------------------------------

 Long-range K will be added via erf
 Coulomb attenuation parameter = 0.2 bohr**(-1)
 A restricted hybrid HF-DFT SCF calculation will be
 performed using Pulay DIIS + Geometric Direct Minimization
 Exchange:     0.2220 Hartree-Fock + 1.0000 wB97X-D + LR-HF
 Correlation:  1.0000 wB97X-D
 Using Euler-Maclaurin-Lebedev (75,302) quadrature formula
 Dispersion:   Grimme D
 SCF converges when RMS gradient is below 1.0E-07
 Geometry optimization detected.  Setting ReadMinima to 0
 Setting SaveMinima to 0
 ---------------------------------------
```

```
  Cycle       Energy         DIIS Error
 ---------------------------------------
    1    -763.9575326811      9.21E-04
    2    -763.9690240462      1.05E-04
    3    -763.9697786882      5.53E-05
    4    -763.9698720535      3.06E-05
    5    -763.9699056673      9.71E-06
    6    -763.9699089675      3.01E-06
    7    -763.9699093771      7.81E-07
    8    -763.9699094210      3.78E-07
    9    -763.9699094280      1.12E-07
   10    -763.9699094290      5.32E-08 Convergence criterion met
 ---------------------------------------
 SCF time:  CPU 304.83 s  wall 486.64 s
 SCF   energy in the final basis set = -763.96990943
 Total energy in the final basis set = -763.96990943

--------------------------------------------------
-  Entering anlman on Sun Oct 13 21:31:24 2024  -
--------------------------------------------------



 --------------------------------------------------------------

                    Orbital Energies (a.u.)
 --------------------------------------------------------------


 Alpha MOs
 -- Occupied --
-14.8208 -14.7876 -14.7370 -14.7049 -10.6755 -10.6591 -10.6456 -10.6435
-10.6385 -10.6308 -10.6065 -10.5882 -10.5767 -10.5756 -10.5538 -10.5249
-10.4962 -10.4756  -1.4148  -1.4089  -1.3463  -1.2738  -1.2330  -1.2003
 -1.1927  -1.1429  -1.1033  -1.0878  -1.0541  -1.0535  -1.0373  -1.0075
 -0.9925  -0.9545  -0.9470  -0.9170  -0.9066  -0.8943  -0.8866  -0.8622
 -0.8561  -0.8474  -0.8382  -0.8257  -0.8075  -0.8026  -0.7919  -0.7853
 -0.7746  -0.7569  -0.7537  -0.7533  -0.7454  -0.7332  -0.7222  -0.7213
 -0.7027  -0.6690  -0.6634  -0.6449  -0.6364  -0.6301  -0.6256  -0.6235
 -0.6076
 -- Virtual --
 -0.2742  -0.2576  -0.2389  -0.2292  -0.1742  -0.1655  -0.1502  -0.1456
 -0.1366  -0.1189  -0.1168  -0.1149  -0.1085  -0.1075  -0.1012  -0.0985
 -0.0922  -0.0867  -0.0852  -0.0785  -0.0762  -0.0719  -0.0717  -0.0695
 -0.0637  -0.0600  -0.0573  -0.0537  -0.0518  -0.0501  -0.0397  -0.0384
 -0.0323  -0.0295  -0.0265  -0.0234  -0.0188  -0.0146  -0.0100  -0.0094
 -0.0041  -0.0019  -0.0001   0.0005   0.0045   0.0067   0.0129   0.0150
  0.0181   0.0187   0.0241   0.0313   0.0321   0.0352   0.0389   0.0414
  0.0434   0.0443   0.0477   0.0513   0.0573   0.0610   0.0625   0.0649
  0.0662   0.0694   0.0716   0.0774   0.0805   0.0824   0.0863   0.0889
  0.0969   0.0985   0.1019   0.1036   0.1069   0.1093   0.1169   0.1199
  0.1212   0.1221   0.1285   0.1348   0.1383   0.1401   0.1425   0.1458
  0.1475   0.1582   0.1628   0.1638   0.1674   0.1711   0.1729   0.1764
  0.1851   0.1886   0.1903   0.1968   0.2004   0.2065   0.2200   0.2259
  0.2346   0.2500   0.2535   0.2731   0.2771   0.2897   0.3025   0.3089
  0.3239   0.3305   0.3340   0.3392   0.3443   0.3584   0.3598   0.3643
  0.3817   0.3844   0.3889   0.3922   0.4018   0.4045   0.4128   0.4211
  0.4261   0.4318   0.4438   0.4552   0.4573   0.4647   0.4700   0.4730
  0.4847   0.4890   0.4930   0.5007   0.5039   0.5115   0.5211   0.5219
  0.5243   0.5272   0.5386   0.5418   0.5447   0.5462   0.5530   0.5593
  0.5639   0.5689   0.5748   0.5817   0.5882   0.5946   0.5991   0.6038
  0.6073   0.6164   0.6251   0.6280   0.6358   0.6408   0.6477   0.6591
  0.6666   0.6721   0.6860   0.6890   0.6903   0.6976   0.7044   0.7095
  0.7136   0.7287   0.7367   0.7400   0.7562   0.7705   0.7792   0.7911
  0.7936   0.8003   0.8042   0.8199   0.8256   0.8429   0.8494   0.8624
  0.8699   0.8792   0.8866   0.9020   0.9178   0.9225   0.9287   0.9481
  0.9508   0.9657   0.9720   0.9878   1.0155   1.0216   1.0313   1.0401
  1.0482   1.0584   1.0683   1.0951   1.1291   1.1464   1.1572   1.1795
  1.2031   1.2123   1.2214   1.2300   1.2570   1.2811   1.2890   1.3011
  1.3082   1.3113   1.3347   1.3432   1.3523   1.3633   1.3697   1.3742
  1.3820   1.3879   1.4004   1.4093   1.4191   1.4263   1.4279   1.4327
  1.4393   1.4484   1.4509   1.4587   1.4666   1.4777   1.4879   1.4896
```

| | | | | | | | |
|---|---|---|---|---|---|---|---|
| 1.5107 | 1.5156 | 1.5199 | 1.5316 | 1.5333 | 1.5403 | 1.5436 | 1.5519 |
| 1.5607 | 1.5656 | 1.5736 | 1.5755 | 1.5927 | 1.5951 | 1.6039 | 1.6122 |
| 1.6196 | 1.6291 | 1.6296 | 1.6592 | 1.6609 | 1.6709 | 1.6745 | 1.6843 |
| 1.6977 | 1.7002 | 1.7152 | 1.7283 | 1.7312 | 1.7487 | 1.7580 | 1.7656 |
| 1.7795 | 1.7925 | 1.8097 | 1.8218 | 1.8380 | 1.8424 | 1.8632 | 1.8848 |
| 1.8970 | 1.9024 | 1.9126 | 1.9321 | 1.9561 | 1.9646 | 1.9862 | 2.0050 |
| 2.0061 | 2.0130 | 2.0191 | 2.0312 | 2.0432 | 2.0535 | 2.0697 | 2.0869 |
| 2.1119 | 2.1255 | 2.1340 | 2.1607 | 2.1697 | 2.1898 | 2.2110 | 2.2253 |
| 2.2304 | 2.2363 | 2.2464 | 2.2537 | 2.2609 | 2.2628 | 2.2806 | 2.2915 |
| 2.3092 | 2.3135 | 2.3244 | 2.3504 | 2.3581 | 2.3671 | 2.3885 | 2.3989 |
| 2.4037 | 2.4273 | 2.4362 | 2.4448 | 2.4486 | 2.4644 | 2.4697 | 2.4878 |
| 2.4980 | 2.5110 | 2.5227 | 2.5282 | 2.5385 | 2.5432 | 2.5510 | 2.5638 |
| 2.5691 | 2.5763 | 2.5875 | 2.5910 | 2.5956 | 2.6032 | 2.6055 | 2.6134 |
| 2.6254 | 2.6427 | 2.6658 | 2.6702 | 2.6756 | 2.6888 | 2.6932 | 2.7123 |
| 2.7212 | 2.7278 | 2.7353 | 2.7436 | 2.7478 | 2.7697 | 2.7808 | 2.7897 |
| 2.7998 | 2.8103 | 2.8177 | 2.8351 | 2.8603 | 2.8698 | 2.8823 | 2.8925 |
| 2.8985 | 2.9061 | 2.9453 | 2.9954 | 3.0521 | 3.0570 | 3.1062 | 3.1484 |
| 3.1698 | 3.2294 | 3.2468 | 3.2595 | 3.3023 | 3.3237 | 3.3763 | 3.4059 |
| 3.4207 | 3.4809 | 3.5271 | 3.5316 | 3.5641 | 3.5936 | 3.6178 | 3.6450 |
| 3.6871 | 3.6888 | 3.7051 | 3.7316 | 3.7359 | 3.7440 | 3.7486 | 3.7607 |
| 3.7720 | 3.8337 | 3.8631 | 3.8982 | 3.9451 | 3.9803 | 4.0273 | 4.0807 |
| 4.0918 | 4.2054 | 4.2900 | 4.3125 | 4.5936 | 4.6703 | 4.6895 | 4.7696 |
| 4.9283 | 5.0298 | 5.2008 | 23.5232 | 23.5839 | 23.7201 | 23.7307 | 23.7381 |
| 23.7792 | 23.8041 | 23.8309 | 23.8566 | 23.8904 | 23.9120 | 23.9595 | 23.9820 |
| 24.0273 | 35.4108 | 35.4632 | 35.5257 | 35.6266 | | | |

Ground-State Mulliken Net Atomic Charges

| Atom | Charge (a.u.) |
|---|---|
| 1 N | 0.343344 |
| 2 C | 0.157715 |
| 3 C | -0.027058 |
| 4 C | -0.198975 |
| 5 C | -0.313380 |
| 6 C | 0.028749 |
| 7 C | -0.812752 |
| 8 C | -0.148813 |
| 9 N | -0.155834 |
| 10 C | -0.214651 |
| 11 N | 0.005185 |
| 12 C | 0.252251 |
| 13 C | -0.149834 |
| 14 C | 0.044655 |
| 15 C | -0.312861 |
| 16 C | -0.261719 |
| 17 C | -0.534021 |
| 18 N | -0.473932 |
| 19 H | 0.222409 |
| 20 H | 0.237483 |
| 21 H | 0.234176 |
| 22 H | 0.224471 |
| 23 H | 0.229926 |
| 24 H | 0.253769 |
| 25 H | 0.227044 |
| 26 H | 0.184315 |
| 27 H | 0.254866 |
| 28 H | 0.379014 |
| 29 H | 0.208726 |
| 30 H | 0.213171 |
| 31 H | 0.251446 |
| 32 H | 0.206577 |
| 33 H | 0.224796 |
| 34 H | 0.127354 |
| 35 H | 0.216686 |
| 36 H | 0.178295 |
| 37 H | 0.315377 |
| 38 H | 0.382030 |

```
 Sum of atomic charges =     2.000000

 -----------------------------------------------------------------
                    Cartesian Multipole Moments
 -----------------------------------------------------------------
    Charge (ESU x 10^10)
                 9.6064
    Dipole Moment (Debye)
         X       7.2382      Y       2.3047      Z       2.7520
       Tot       8.0794
    Quadrupole Moments (Debye-Ang)
        XX     -15.0941     XY      -6.9392     YY     -68.4588
        XZ       7.8475     YZ      -9.1862     ZZ     -74.7864
    Traceless Quadrupole Moments (Debye-Ang)
       QXX     113.0570    QYY     -47.0370    QZZ     -66.0200
       QXY     -20.8175    QXZ      23.5425    QYZ     -27.5587
    Octopole Moments (Debye-Ang^2)
       XXX      18.3582    XXY     -95.1871    XYY      17.9268
       YYY       2.4448    XXZ      55.9641    XYZ      -0.3161
       YYZ     -44.3935    XZZ      24.5186    YZZ      -7.5849
       ZZZ     -14.6995
    Traceless Octopole Moments (Debye-Ang^2)
       XXX    -271.8604    YYY     939.6178    ZZZ    -192.3327
       XXY   -1126.8254    XXZ     848.8487    XYY      86.4917
       XYZ      -4.7417    XZZ     185.3687    YYZ    -656.5160
       YZZ     187.2075
    Hexadecapole Moments (Debye-Ang^3)
      XXXX   -3601.7640   XXXY    -105.1292   XXYY    -782.7540
      XYYY      76.5774   YYYY    -993.4948   XXXZ      40.4603
      XXYZ     -18.0521   XYYZ     -31.2677   YYYZ     -54.1607
      XXZZ    -707.7480   XYZZ      26.8546   YYZZ    -247.0131
      XZZZ    -159.3567   YZZZ      26.9140   ZZZZ    -687.6557
    Traceless Hexadecapole Moments (Debye-Ang^3)
      XXXX    1297.2147   XXXY  -10962.1943   XXXZ   11005.7160
      XXYY   -1730.0836   XXYZ   -1202.2762   XXZZ     432.8689
      XYYY    8117.0032   XYYZ   -1030.6508   XYZZ    2845.1911
      XZZZ   -9975.0652   YYYY   -1044.8865   YYYZ   -3607.2968
      YYZZ    2774.9701   YZZZ    4809.5731   ZZZZ   -3207.8389
 -----------------------------------------------------------------


-------------------------------------------------
-  Entering drvman on Sun Oct 13 21:31:24 2024  -
-------------------------------------------------

 Calculating analytic gradient of the SCF energy
 Gradient of SCF Energy
            1           2           3           4           5           6
    1  -0.0002176   0.0000905  -0.0001547  -0.0000986   0.0001501  -0.0000012
    2   0.0001080  -0.0001448  -0.0000576   0.0002113  -0.0003292   0.0003297
    3   0.0001702   0.0000079   0.0000353   0.0002671  -0.0001273  -0.0004249
            7           8           9          10          11          12
    1  -0.0004526   0.0000381   0.0002201   0.0005935  -0.0005544   0.0008203
    2   0.0001498   0.0005117  -0.0000517  -0.0002670   0.0004283  -0.0022908
    3   0.0000325  -0.0001777   0.0002843   0.0002224  -0.0001401  -0.0006026
           13          14          15          16          17          18
    1  -0.0004583  -0.0005574  -0.0000400   0.0002451  -0.0000605  -0.0004896
    2  -0.0000187   0.0002466  -0.0002984   0.0004104  -0.0000769   0.0022447
    3  -0.0002734   0.0002575  -0.0000554  -0.0000681   0.0000777   0.0014362
           19          20          21          22          23          24
    1   0.0001100   0.0000583   0.0000452   0.0001055   0.0001401  -0.0000170
    2  -0.0001689  -0.0000473  -0.0001631  -0.0000055   0.0001184   0.0001709
    3  -0.0000079   0.0000048   0.0000710   0.0001364  -0.0001499  -0.0000637
           25          26          27          28          29          30
    1  -0.0000019   0.0006467  -0.0000501  -0.0000543  -0.0001297  -0.0000960
    2   0.0002856  -0.0002128  -0.0000883  -0.0000865   0.0000229   0.0001579
    3   0.0000877   0.0001977   0.0000304   0.0000551   0.0000914   0.0000378
           31          32          33          34          35          36
    1  -0.0001089   0.0000326  -0.0000263  -0.0000312  -0.0000006   0.0000365
    2   0.0000337  -0.0000967  -0.0002194   0.0001616  -0.0000150   0.0000080
    3  -0.0000884   0.0000133   0.0000332   0.0000748  -0.0000011   0.0000260
```

```
          37          38
    1   0.0005329  -0.0002649
    2  -0.0007148  -0.0002461
    3  -0.0006468  -0.0008235
 Max gradient component =       2.291E-03
 RMS gradient           =       4.194E-04
 Gradient time:  CPU 130.63 s  wall 207.66 s

-------------------------------------------------
-  Entering optman on Sun Oct 13 21:34:52 2024  -
-------------------------------------------------

 Geometry Optimization Parameters
   NAtoms,    NIC,     NZ,  NCons,   NDum,   NFix, NCnnct, MaxDiis
       38     272       0       0       0       0       0       0

 Cartesian Hessian Update
 Hessian updated using BFGS update


** GEOMETRY OPTIMIZATION IN DELOCALIZED INTERNAL COORDINATES **
   Searching for a Minimum

   Optimization Cycle:  10

                    Coordinates (Angstroms)
     ATOM                X               Y               Z
      1  N         2.5518326569    0.4174712405    0.4136215562
      2  C         2.7511734580   -0.5607578333   -0.5167562380
      3  C         3.5831487688   -1.6196809429   -0.1834582749
      4  C         4.2066325432   -1.6749121743    1.0533219242
      5  C         3.9954475386   -0.6512234156    1.9734464281
      6  C         3.1657012099    0.3838378964    1.6244682956
      7  C         2.1100698602   -0.4473706184   -1.8631306394
      8  C         1.6632997959    1.5583756701    0.1121661283
      9  N        -1.5973786664    0.0257243004    1.2041580949
     10  C        -2.5023254762    0.6598737129    0.4103210022
     11  N        -2.1125643688    1.5776675882   -0.4332880317
     12  C        -0.8120795119    1.9095517979   -0.5262831860
     13  C         0.1922629099    1.2176474334    0.2368400730
     14  C        -0.2721207464    0.2857883213    1.1107946892
     15  C        -3.9315697661    0.2489056391    0.4812953784
     16  C        -4.2657125199   -0.7884315554   -0.6153140359
     17  C        -3.4841599404   -2.0915307207   -0.4899575566
     18  N        -0.5333595044    2.9110146353   -1.3521833232
     19  H         3.7482781521   -2.3983643120   -0.9173377735
     20  H         4.8600617748   -2.5048249343    1.2970091195
     21  H         4.4732501699   -0.6483891413    2.9444013068
     22  H         2.9667159698    1.2160571356    2.2878006736
     23  H         2.2795717867   -1.3612642164   -2.4293018071
     24  H         2.5542543834    0.3752092705   -2.4335672668
     25  H         1.0326987098   -0.2814104948   -1.7975513317
     26  H         1.9041681153    1.8930631083   -0.8969341690
     27  H         1.9309658878    2.3725249053    0.7882421018
     28  H        -1.9283357434   -0.6815102295    1.8492126427
     29  H         0.3712764337   -0.2943946099    1.7612726037
     30  H        -4.1549047691   -0.1639553932    1.4698866400
     31  H        -4.5393590327    1.1437489640    0.3412903180
     32  H        -4.1023417493   -0.3322922682   -1.5951842644
     33  H        -5.3359963428   -0.9896467715   -0.5385117405
     34  H        -2.4098183708   -1.9483083971   -0.6551030618
     35  H        -3.8243128722   -2.8126731630   -1.2341075494
     36  H        -3.6260792141   -2.5523176565    0.4932683431
     37  H         0.3902136441    3.2792796359   -1.5071940851
     38  H        -1.2986051743    3.3275175932   -1.8676529844
   Point Group: c1    Number of degrees of freedom:   108


   Energy is   -763.969909429
```

```
 Hessian updated using BFGS update
  internal optimization (0)

108 Hessian modes will be used to form the next step
  Hessian Eigenvalues:
     0.001011    0.002612    0.002967    0.004189    0.007298    0.011554
     0.016182    0.019188    0.019417    0.020021    0.020870    0.022651
     0.022734    0.023126    0.024182    0.024623    0.025735    0.026811
     0.027366    0.028248    0.029301    0.029861    0.033408    0.036780
     0.038865    0.041366    0.043200    0.043654    0.044800    0.045637
     0.053204    0.053924    0.055291    0.059180    0.061143    0.079961
     0.085516    0.093228    0.121715    0.122206    0.127146    0.131195
     0.132033    0.132957    0.136460    0.141294    0.143214    0.144189
     0.146962    0.147710    0.149940    0.152814    0.153167    0.154560
     0.157974    0.162253    0.193892    0.206385    0.210501    0.217438
     0.226551    0.231398    0.238498    0.247819    0.252114    0.259948
     0.268083    0.273364    0.282036    0.298578    0.300184    0.300643
     0.301053    0.301509    0.301874    0.303202    0.304163    0.304545
     0.305014    0.305552    0.307593    0.308441    0.311904    0.317325
     0.319628    0.332136    0.333384    0.335916    0.337124    0.341103
     0.343233    0.356650    0.361072    0.366800    0.382747    0.391227
     0.400162    0.407396    0.418300    0.420485    0.424298    0.434943
     0.444297    0.459020    0.469896    0.505249    0.569144    0.716750

 Minimum search - taking simple RFO step
 Searching for Lamda that Minimizes Along All modes
 Value Taken    Lamda =  -0.00073667
 Calculated Step too Large.  Step scaled by  0.542699
 Step Taken.  Stepsize is  0.300000

                            Maximum     Tolerance    Cnvgd?
        Gradient           0.003304      0.000800      NO
        Displacement       0.129495      0.001400      NO
        Energy change     -0.000341      0.000228      NO


 New Cartesian Coordinates Obtained by Inverse Iteration

 Displacement from previous Coordinates is:  1.165269
 ----------------------------------------------------------------
            Standard Nuclear Orientation (Angstroms)
    I     Atom           X                Y                Z
 ----------------------------------------------------------------
    1      N       2.5616740088     0.4213177941     0.3860892136
    2      C       2.7859594792    -0.5842049955    -0.5084777392
    3      C       3.6246241973    -1.6219747497    -0.1280996696
    4      C       4.2319916883    -1.6276209812     1.1178542757
    5      C       3.9960590020    -0.5763040028     2.0003395325
    6      C       3.1582635405     0.4353407188     1.6063672872
    7      C       2.1678822609    -0.5246256179    -1.8689275612
    8      C       1.6575997284     1.5345827516     0.0377227462
    9      N      -1.5736644104    -0.0413721388     1.1636689283
   10      C      -2.4982581739     0.6411330339     0.4367899886
   11      N      -2.1287096034     1.5998907045    -0.3704709699
   12      C      -0.8292450446     1.9243150227    -0.4880937521
   13      C       0.1932058727     1.1870245994     0.2044303980
   14      C      -0.2488572421     0.2121890809     1.0411384521
   15      C      -3.9284416121     0.2395654208     0.5367968153
   16      C      -4.3119000238    -0.7430134334    -0.5935595313
   17      C      -3.5420043176    -2.0585181192    -0.5558964610
   18      N      -0.5631907557     2.9640855807    -1.2713298575
   19      H       3.8081321020    -2.4233034876    -0.8326324665
   20      H       4.8911396520    -2.4411060991     1.3988184672
   21      H       4.4607502314    -0.5348543806     2.9767363462
   22      H       2.9371219775     1.2852374378     2.2397203943
   23      H       2.3285687086    -1.4688124488    -2.3863274564
   24      H       2.6447277190     0.2578013547    -2.4690413529
   25      H       1.0937614021    -0.3311744667    -1.8350582320
   26      H       1.8729364584     1.8182316724    -0.9932282806
   27      H       1.9247518243     2.3877514481     0.6652946461
```

```
  28      H      -1.8906348153    -0.7801093745     1.7798658092
  29      H       0.4094784638    -0.4082288035     1.6371440357
  30      H      -4.1222951167    -0.2172732512     1.5122578283
  31      H      -4.5312999722     1.1452249602     0.4600180755
  32      H      -4.1772095153    -0.2442825633    -1.5569801350
  33      H      -5.3811886509    -0.9367059098    -0.4886498944
  34      H      -2.4728093270    -1.9198754770    -0.7545785721
  35      H      -3.9183774723    -2.7414632228    -1.3184700112
  36      H      -3.6536672514    -2.5619850747     0.4102739239
  37      H       0.3604441262     3.3275633872    -1.4395969490
  38      H      -1.3373191386     3.4055536302    -1.7519082717
 ----------------------------------------------------------------
 Nuclear Repulsion Energy =        1319.57519550 hartrees
 There are       65 alpha and       65 beta electrons

-------------------------------------------------
-  Entering fldman on Sun Oct 13 21:34:52 2024  -
-------------------------------------------------

 Applying Cartesian multipole field
    Component          Value
    ---------          -----
     (2,0,0)        1.00000E-12
     (0,2,0)        2.00000E-11
     (0,0,2)       -3.00000E-11
 Nucleus-field energy     =     0.0000000056 hartrees

-------------------------------------------------
-  Entering gesman on Sun Oct 13 21:34:52 2024  -
-------------------------------------------------

 Requested basis set is 6-311+G(d,p)
 There are 188 shells and 516 basis functions
 A cutoff of  1.0D-12 yielded  13028 shell pairs
 There are    102897 function pairs (    109654 Cartesian)
 Smallest overlap matrix eigenvalue = 2.26E-06
 Linear dependence detected in AO basis
 Tighter screening thresholds may be required for diffuse basis sets
 Use S2THRESH > 12 and THRESH = 14 in case of SCF convergence issues
 Number of orthogonalized atomic orbitals = 510
 Maximum deviation from orthogonality = 1.134E-11
 Guess MOs from SCF MO coefficient file
 Reading MOs from coefficient file
 Reading MOs from coefficient file

-------------------------------------------------
-  Entering scfman on Sun Oct 13 21:34:52 2024  -
-------------------------------------------------

 Long-range K will be added via erf
 Coulomb attenuation parameter = 0.2 bohr**(-1)
 A restricted hybrid HF-DFT SCF calculation will be
 performed using Pulay DIIS + Geometric Direct Minimization
 Exchange:     0.2220 Hartree-Fock + 1.0000 wB97X-D + LR-HF
 Correlation:  1.0000 wB97X-D
 Using Euler-Maclaurin-Lebedev (75,302) quadrature formula
 Dispersion:   Grimme D
 SCF converges when RMS gradient is below 1.0E-07
 Geometry optimization detected.  Setting ReadMinima to 0
 Setting SaveMinima to 0
 ---------------------------------------
  Cycle       Energy         DIIS Error
 ---------------------------------------
    1    -763.9523536526      1.04E-03
    2    -763.9691431294      1.20E-04
    3    -763.9700742484      6.76E-05
    4    -763.9702109385      3.45E-05
    5    -763.9702539598      1.18E-05
    6    -763.9702589218      3.37E-06
    7    -763.9702594515      9.03E-07
```

```
    8    -763.9702595120      4.34E-07
    9    -763.9702595217      1.30E-07
   10    -763.9702595231      6.38E-08 Convergence criterion met
 ---------------------------------------
 SCF time:  CPU 286.47 s  wall 451.75 s
 SCF   energy in the final basis set = -763.97025952
 Total energy in the final basis set = -763.97025952

-------------------------------------------------
-  Entering anlman on Sun Oct 13 21:42:24 2024  -
-------------------------------------------------


 --------------------------------------------------------------

                   Orbital Energies (a.u.)
 --------------------------------------------------------------


 Alpha MOs
 -- Occupied --
-14.8212 -14.7873 -14.7368 -14.7047 -10.6750 -10.6588 -10.6453 -10.6439
-10.6386 -10.6311 -10.6065 -10.5886 -10.5770 -10.5758 -10.5535 -10.5246
-10.4958 -10.4751  -1.4149  -1.4091  -1.3458  -1.2736  -1.2333  -1.2004
 -1.1930  -1.1430  -1.1033  -1.0878  -1.0541  -1.0532  -1.0376  -1.0071
 -0.9925  -0.9543  -0.9469  -0.9165  -0.9061  -0.8940  -0.8873  -0.8619
 -0.8556  -0.8475  -0.8387  -0.8259  -0.8067  -0.8035  -0.7922  -0.7861
 -0.7742  -0.7562  -0.7536  -0.7530  -0.7451  -0.7334  -0.7220  -0.7210
 -0.7023  -0.6684  -0.6639  -0.6445  -0.6362  -0.6296  -0.6259  -0.6231
 -0.6074
 -- Virtual --
 -0.2746  -0.2571  -0.2382  -0.2291  -0.1744  -0.1655  -0.1504  -0.1456
 -0.1370  -0.1182  -0.1167  -0.1140  -0.1086  -0.1072  -0.1012  -0.0979
 -0.0925  -0.0872  -0.0851  -0.0787  -0.0778  -0.0722  -0.0713  -0.0697
 -0.0639  -0.0605  -0.0565  -0.0542  -0.0516  -0.0489  -0.0397  -0.0381
 -0.0316  -0.0309  -0.0266  -0.0230  -0.0193  -0.0150  -0.0104  -0.0091
 -0.0038  -0.0023   0.0005   0.0009   0.0051   0.0062   0.0126   0.0160
  0.0187   0.0197   0.0241   0.0289   0.0315   0.0347   0.0390   0.0418
  0.0440   0.0443   0.0470   0.0513   0.0558   0.0612   0.0628   0.0649
  0.0669   0.0690   0.0718   0.0778   0.0811   0.0817   0.0861   0.0897
  0.0965   0.0985   0.1010   0.1038   0.1080   0.1093   0.1156   0.1195
  0.1212   0.1238   0.1282   0.1344   0.1401   0.1403   0.1420   0.1451
  0.1480   0.1560   0.1626   0.1636   0.1681   0.1725   0.1726   0.1785
  0.1854   0.1876   0.1886   0.1965   0.2007   0.2044   0.2228   0.2249
  0.2348   0.2489   0.2530   0.2761   0.2797   0.2889   0.3024   0.3113
  0.3225   0.3309   0.3340   0.3396   0.3454   0.3572   0.3608   0.3627
  0.3815   0.3815   0.3858   0.3928   0.3982   0.4023   0.4089   0.4210
  0.4253   0.4316   0.4391   0.4539   0.4571   0.4658   0.4734   0.4759
  0.4863   0.4893   0.4918   0.5005   0.5058   0.5141   0.5192   0.5221
  0.5241   0.5277   0.5374   0.5422   0.5450   0.5463   0.5548   0.5597
  0.5624   0.5681   0.5734   0.5781   0.5883   0.5932   0.5987   0.6033
  0.6090   0.6172   0.6251   0.6259   0.6349   0.6421   0.6474   0.6600
  0.6653   0.6706   0.6802   0.6873   0.6905   0.6966   0.7030   0.7098
  0.7148   0.7270   0.7418   0.7441   0.7541   0.7705   0.7793   0.7891
  0.7929   0.8006   0.8085   0.8193   0.8239   0.8418   0.8518   0.8591
  0.8692   0.8791   0.8875   0.8996   0.9144   0.9199   0.9362   0.9483
  0.9547   0.9669   0.9700   0.9897   1.0133   1.0207   1.0293   1.0411
  1.0495   1.0572   1.0697   1.1005   1.1265   1.1438   1.1608   1.1838
  1.2010   1.2132   1.2218   1.2321   1.2545   1.2756   1.2853   1.2999
  1.3088   1.3134   1.3339   1.3425   1.3540   1.3650   1.3703   1.3741
  1.3821   1.3880   1.3986   1.4069   1.4167   1.4248   1.4284   1.4337
  1.4392   1.4417   1.4498   1.4566   1.4661   1.4768   1.4872   1.4907
  1.5123   1.5167   1.5215   1.5315   1.5338   1.5415   1.5456   1.5506
  1.5569   1.5652   1.5739   1.5771   1.5908   1.5965   1.6040   1.6113
  1.6239   1.6269   1.6308   1.6593   1.6621   1.6710   1.6765   1.6816
  1.6991   1.7019   1.7167   1.7289   1.7341   1.7481   1.7584   1.7666
  1.7797   1.7934   1.8097   1.8196   1.8378   1.8420   1.8643   1.8841
  1.8965   1.9019   1.9140   1.9362   1.9596   1.9645   1.9873   2.0055
  2.0092   2.0137   2.0193   2.0337   2.0474   2.0532   2.0713   2.0902
  2.1130   2.1249   2.1350   2.1631   2.1676   2.1894   2.2103   2.2268
  2.2316   2.2358   2.2457   2.2558   2.2627   2.2645   2.2789   2.2907
```

| | | | | | | | |
|---|---|---|---|---|---|---|---|
| 2.3069 | 2.3121 | 2.3250 | 2.3481 | 2.3582 | 2.3710 | 2.3887 | 2.3985 |
| 2.4048 | 2.4292 | 2.4342 | 2.4434 | 2.4500 | 2.4656 | 2.4722 | 2.4875 |
| 2.4949 | 2.5094 | 2.5215 | 2.5276 | 2.5369 | 2.5432 | 2.5525 | 2.5619 |
| 2.5675 | 2.5744 | 2.5868 | 2.5891 | 2.5975 | 2.6017 | 2.6050 | 2.6132 |
| 2.6286 | 2.6421 | 2.6640 | 2.6697 | 2.6760 | 2.6942 | 2.6960 | 2.7131 |
| 2.7222 | 2.7276 | 2.7396 | 2.7440 | 2.7533 | 2.7691 | 2.7801 | 2.7909 |
| 2.7940 | 2.8107 | 2.8168 | 2.8342 | 2.8567 | 2.8650 | 2.8836 | 2.8937 |
| 2.8978 | 2.9091 | 2.9465 | 2.9946 | 3.0509 | 3.0587 | 3.1021 | 3.1484 |
| 3.1725 | 3.2293 | 3.2470 | 3.2587 | 3.2993 | 3.3245 | 3.3774 | 3.4054 |
| 3.4203 | 3.4809 | 3.5263 | 3.5336 | 3.5656 | 3.5926 | 3.6194 | 3.6466 |
| 3.6844 | 3.6877 | 3.7048 | 3.7304 | 3.7362 | 3.7411 | 3.7469 | 3.7624 |
| 3.7728 | 3.8331 | 3.8627 | 3.8981 | 3.9454 | 3.9786 | 4.0277 | 4.0777 |
| 4.0929 | 4.2060 | 4.2890 | 4.3140 | 4.5937 | 4.6702 | 4.6873 | 4.7794 |
| 4.9280 | 5.0287 | 5.2027 | 23.5269 | 23.5840 | 23.7217 | 23.7297 | 23.7385 |
| 23.7774 | 23.8044 | 23.8294 | 23.8567 | 23.8909 | 23.9117 | 23.9595 | 23.9835 |
| 24.0270 | 35.4108 | 35.4636 | 35.5258 | 35.6272 | | | |

Ground-State Mulliken Net Atomic Charges

| Atom | Charge (a.u.) |
|---|---|
| 1 N | 0.323966 |
| 2 C | 0.148417 |
| 3 C | -0.034086 |
| 4 C | -0.194985 |
| 5 C | -0.312652 |
| 6 C | 0.036017 |
| 7 C | -0.803952 |
| 8 C | -0.161263 |
| 9 N | -0.155993 |
| 10 C | -0.216792 |
| 11 N | 0.003711 |
| 12 C | 0.258462 |
| 13 C | -0.130521 |
| 14 C | 0.035576 |
| 15 C | -0.316072 |
| 16 C | -0.260970 |
| 17 C | -0.533308 |
| 18 N | -0.467847 |
| 19 H | 0.222815 |
| 20 H | 0.237633 |
| 21 H | 0.234357 |
| 22 H | 0.224765 |
| 23 H | 0.228368 |
| 24 H | 0.258124 |
| 25 H | 0.221870 |
| 26 H | 0.198067 |
| 27 H | 0.254845 |
| 28 H | 0.377830 |
| 29 H | 0.208376 |
| 30 H | 0.213689 |
| 31 H | 0.251207 |
| 32 H | 0.206770 |
| 33 H | 0.224649 |
| 34 H | 0.128413 |
| 35 H | 0.216803 |
| 36 H | 0.177758 |
| 37 H | 0.315676 |
| 38 H | 0.380276 |
| Sum of atomic charges = | 2.000000 |

Cartesian Multipole Moments

Charge (ESU x 10^10)
9.6064

Dipole Moment (Debye)

| | | | | | |
|---|---|---|---|---|---|
| X | 7.3527 | Y | 2.2312 | Z | 2.6816 |

```
      Tot       8.1383
   Quadrupole Moments (Debye-Ang)
       XX     -14.3296     XY      -6.8592     YY     -67.8175
       XZ       8.0848     YZ      -9.1915     ZZ     -75.7496
   Traceless Quadrupole Moments (Debye-Ang)
      QXX     114.9079    QYY     -45.5558    QZZ     -69.3520
      QXY     -20.5777    QXZ      24.2543    QYZ     -27.5745
   Octopole Moments (Debye-Ang^2)
      XXX      20.2094    XXY     -94.8989    XYY      16.1819
      YYY       6.3604    XXZ      56.4246    XYZ       0.1587
      YYZ     -44.0637    XZZ      25.9982    YZZ      -9.5974
      ZZZ     -16.4021
   Traceless Octopole Moments (Debye-Ang^2)
      XXX    -258.3649    YYY     978.6293    ZZZ    -209.6606
      XXY   -1129.0753    XXZ     858.4929    XYY      55.5601
      XYZ       2.3798    XZZ     202.8048    YYZ    -648.8323
      YZZ     150.4460
   Hexadecapole Moments (Debye-Ang^3)
     XXXX   -3637.8428   XXXY    -100.5883   XXYY    -795.5486
     XYYY      72.7431   YYYY    -976.2336   XXXZ      45.9688
     XXYZ     -18.9190   XYYZ     -31.3711   YYYZ     -62.5806
     XXZZ    -709.8306   XYZZ      28.2396   YYZZ    -248.8959
     XZZZ    -158.6032   YZZZ      26.9640   ZZZZ    -683.7404
   Traceless Hexadecapole Moments (Debye-Ang^3)
     XXXX    1659.1796   XXXY  -10579.5195   XXXZ   11306.9693
     XXYY   -2493.2009   XXYZ   -1154.0043   XXZZ     834.0213
     XYYY    7620.2768   XYYZ   -1133.8799   XYZZ    2959.2427
     XZZZ  -10173.0894   YYYY      99.2005   YYYZ   -4073.4788
     YYZZ    2394.0004   YZZZ    5227.4831   ZZZZ   -3228.0217
 -----------------------------------------------------------------

 -------------------------------------------------
-  Entering drvman on Sun Oct 13 21:42:24 2024  -
 -------------------------------------------------

 Calculating analytic gradient of the SCF energy
 Gradient of SCF Energy
            1           2           3           4           5           6
    1  -0.0003582   0.0000681  -0.0004129  -0.0000766   0.0001294   0.0003085
    2   0.0000633   0.0002881  -0.0000397   0.0002909  -0.0003572   0.0000789
    3   0.0003767  -0.0000039  -0.0002024   0.0002335  -0.0001454  -0.0002415
            7           8           9          10          11          12
    1  -0.0006066   0.0001908   0.0001180   0.0008707  -0.0004386   0.0005328
    2   0.0000977   0.0003297   0.0003267  -0.0005925   0.0006405  -0.0030244
    3   0.0001319  -0.0005862  -0.0000170   0.0005119  -0.0002754   0.0003644
           13          14          15          16          17          18
    1  -0.0007219  -0.0004360  -0.0001189   0.0003572  -0.0001283  -0.0001831
    2  -0.0001333   0.0002686  -0.0000490   0.0002947  -0.0000936   0.0027499
    3  -0.0004845   0.0003321  -0.0001503   0.0001868  -0.0000656   0.0013420
           19          20          21          22          23          24
    1   0.0000846   0.0000411   0.0000726   0.0001036   0.0003010   0.0002463
    2  -0.0001994  -0.0000514  -0.0001464  -0.0000050   0.0000341  -0.0000685
    3   0.0000114   0.0000836   0.0000480   0.0001967  -0.0001775   0.0000050
           25          26          27          28          29          30
    1  -0.0000307   0.0003156  -0.0001935  -0.0001149  -0.0000543  -0.0000933
    2   0.0002974   0.0000626   0.0001022  -0.0000536  -0.0000764   0.0001089
    3  -0.0000499   0.0001550   0.0000416   0.0001227   0.0000145  -0.0000073
           31          32          33          34          35          36
    1  -0.0000834   0.0000278  -0.0000295  -0.0000428  -0.0000457   0.0000640
    2  -0.0000375  -0.0000860  -0.0002252   0.0001051   0.0000065  -0.0000357
    3  -0.0000842  -0.0000394  -0.0000015   0.0000605  -0.0000084   0.0000669
           37          38
    1   0.0007341  -0.0003971
    2  -0.0004498  -0.0004212
    3  -0.0006358  -0.0011087
 Max gradient component =       3.024E-03
 RMS gradient           =       4.955E-04
 Gradient time:  CPU 125.30 s  wall 194.61 s

 -------------------------------------------------
```

```
-  Entering optman on Sun Oct 13 21:45:38 2024  -
------------------------------------------------

 Geometry Optimization Parameters
   NAtoms,    NIC,     NZ,  NCons,   NDum,   NFix, NCnnct, MaxDiis
       38     272       0       0       0       0       0       0

 Cartesian Hessian Update
 Hessian updated using BFGS update


** GEOMETRY OPTIMIZATION IN DELOCALIZED INTERNAL COORDINATES **
   Searching for a Minimum

   Optimization Cycle:  11

                      Coordinates (Angstroms)
     ATOM                X               Y               Z
      1  N         2.5616740088    0.4213177941    0.3860892136
      2  C         2.7859594792   -0.5842049955   -0.5084777392
      3  C         3.6246241973   -1.6219747497   -0.1280996696
      4  C         4.2319916883   -1.6276209812    1.1178542757
      5  C         3.9960590020   -0.5763040028    2.0003395325
      6  C         3.1582635405    0.4353407188    1.6063672872
      7  C         2.1678822609   -0.5246256179   -1.8689275612
      8  C         1.6575997284    1.5345827516    0.0377227462
      9  N        -1.5736644104   -0.0413721388    1.1636689283
     10  C        -2.4982581739    0.6411330339    0.4367899886
     11  N        -2.1287096034    1.5998907045   -0.3704709699
     12  C        -0.8292450446    1.9243150227   -0.4880937521
     13  C         0.1932058727    1.1870245994    0.2044303980
     14  C        -0.2488572421    0.2121890809    1.0411384521
     15  C        -3.9284416121    0.2395654208    0.5367968153
     16  C        -4.3119000238   -0.7430134334   -0.5935595313
     17  C        -3.5420043176   -2.0585181192   -0.5558964610
     18  N        -0.5631907557    2.9640855807   -1.2713298575
     19  H         3.8081321020   -2.4233034876   -0.8326324665
     20  H         4.8911396520   -2.4411060991    1.3988184672
     21  H         4.4607502314   -0.5348543806    2.9767363462
     22  H         2.9371219775    1.2852374378    2.2397203943
     23  H         2.3285687086   -1.4688124488   -2.3863274564
     24  H         2.6447277190    0.2578013547   -2.4690413529
     25  H         1.0937614021   -0.3311744667   -1.8350582320
     26  H         1.8729364584    1.8182316724   -0.9932282806
     27  H         1.9247518243    2.3877514481    0.6652946461
     28  H        -1.8906348153   -0.7801093745    1.7798658092
     29  H         0.4094784638   -0.4082288035    1.6371440357
     30  H        -4.1222951167   -0.2172732512    1.5122578283
     31  H        -4.5312999722    1.1452249602    0.4600180755
     32  H        -4.1772095153   -0.2442825633   -1.5569801350
     33  H        -5.3811886509   -0.9367059098   -0.4886498944
     34  H        -2.4728093270   -1.9198754770   -0.7545785721
     35  H        -3.9183774723   -2.7414632228   -1.3184700112
     36  H        -3.6536672514   -2.5619850747    0.4102739239
     37  H         0.3604441262    3.3275633872   -1.4395969490
     38  H        -1.3373191386    3.4055536302   -1.7519082717
   Point Group: c1    Number of degrees of freedom:   108


   Energy is   -763.970259523

 Hessian updated using BFGS update
  internal optimization (0)

108 Hessian modes will be used to form the next step
  Hessian Eigenvalues:
     0.000782    0.002608    0.002743    0.004177    0.007236    0.011620
     0.015360    0.019259    0.019459    0.020020    0.020662    0.022643
     0.022736    0.023433    0.024159    0.024927    0.025731    0.026729
     0.027216    0.028244    0.029185    0.029857    0.033217    0.036785
```

| | | | | | |
|---|---|---|---|---|---|
| 0.038906 | 0.041241 | 0.043193 | 0.043676 | 0.044795 | 0.045790 |
| 0.051616 | 0.054067 | 0.055316 | 0.057701 | 0.063567 | 0.079885 |
| 0.085554 | 0.094217 | 0.121708 | 0.122203 | 0.127147 | 0.131208 |
| 0.132055 | 0.133031 | 0.136421 | 0.141417 | 0.143368 | 0.145014 |
| 0.147000 | 0.147737 | 0.150895 | 0.152807 | 0.153168 | 0.154380 |
| 0.157857 | 0.161577 | 0.193820 | 0.206374 | 0.210522 | 0.217457 |
| 0.226962 | 0.233448 | 0.238079 | 0.248625 | 0.252218 | 0.261595 |
| 0.268209 | 0.273378 | 0.282356 | 0.296045 | 0.300190 | 0.300647 |
| 0.300908 | 0.301295 | 0.301889 | 0.302747 | 0.303973 | 0.304190 |
| 0.305099 | 0.305518 | 0.307596 | 0.308479 | 0.311573 | 0.315243 |
| 0.319750 | 0.332143 | 0.333259 | 0.335931 | 0.337122 | 0.340685 |
| 0.343541 | 0.356691 | 0.363835 | 0.367169 | 0.384303 | 0.391909 |
| 0.400896 | 0.407286 | 0.418317 | 0.420184 | 0.424615 | 0.436879 |
| 0.446995 | 0.466677 | 0.475163 | 0.501111 | 0.562809 | 0.693830 |

Minimum search - taking simple RFO step
Searching for Lamda that Minimizes Along All modes
Value Taken    Lamda =  -0.00081259
Calculated Step too Large.  Step scaled by  0.487973
Step Taken.  Stepsize is  0.300000

| | Maximum | Tolerance | Cnvgd? |
|---|---|---|---|
| Gradient | 0.003192 | 0.000800 | NO |
| Displacement | 0.130029 | 0.001400 | NO |
| Energy change | -0.000350 | 0.000228 | NO |

New Cartesian Coordinates Obtained by Inverse Iteration

Displacement from previous Coordinates is:  1.257313

Standard Nuclear Orientation (Angstroms)

| I | Atom | X | Y | Z |
|---|---|---|---|---|
| 1 | N | 2.5720332225 | 0.4206003368 | 0.3575647298 |
| 2 | C | 2.8288107266 | -0.6097756715 | -0.4988786799 |
| 3 | C | 3.6859979276 | -1.6146280423 | -0.0731850294 |
| 4 | C | 4.2778143332 | -1.5636526423 | 1.1790229805 |
| 5 | C | 4.0055818490 | -0.4892976828 | 2.0231315306 |
| 6 | C | 3.1503042158 | 0.4882939343 | 1.5850673452 |
| 7 | C | 2.2260132293 | -0.6142156617 | -1.8673432670 |
| 8 | C | 1.6488669187 | 1.5005143651 | -0.0372705117 |
| 9 | N | -1.5565518242 | -0.1124803463 | 1.1179433551 |
| 10 | C | -2.4960318089 | 0.6193071342 | 0.4626916291 |
| 11 | N | -2.1419164649 | 1.6173804967 | -0.3035453698 |
| 12 | C | -0.8433506079 | 1.9313968822 | -0.4469723724 |
| 13 | C | 0.1923160475 | 1.1487111705 | 0.1723453148 |
| 14 | C | -0.2322942429 | 0.1333034442 | 0.9676354556 |
| 15 | C | -3.9271152305 | 0.2296326613 | 0.5919735480 |
| 16 | C | -4.3647768497 | -0.6881325541 | -0.5727626316 |
| 17 | C | -3.6152013428 | -2.0150286355 | -0.6263918176 |
| 18 | N | -0.5853766403 | 3.0053436889 | -1.1859885038 |
| 19 | H | 3.8951580126 | -2.4353014976 | -0.7475417832 |
| 20 | H | 4.9515163219 | -2.3517739654 | 1.4954672603 |
| 21 | H | 4.4561828217 | -0.4042318941 | 3.0032600843 |
| 22 | H | 2.8995544951 | 1.3524750712 | 2.1873186928 |
| 23 | H | 2.3933106156 | -1.5817358473 | -2.3375115938 |
| 24 | H | 2.7116142811 | 0.1390667344 | -2.4970697541 |
| 25 | H | 1.1516543584 | -0.4191207875 | -1.8571938035 |
| 26 | H | 1.8388883244 | 1.7294650890 | -1.0873868593 |
| 27 | H | 1.9141441590 | 2.3909462238 | 0.5382457163 |
| 28 | H | -1.8630746066 | -0.8820570595 | 1.7007898645 |
| 29 | H | 0.4363542099 | -0.5260563259 | 1.5074818616 |
| 30 | H | -4.0929398169 | -0.2753677105 | 1.5488352620 |
| 31 | H | -4.5195018828 | 1.1454159879 | 0.5848285372 |
| 32 | H | -4.2538195492 | -0.1439537738 | -1.5143298361 |
| 33 | H | -5.4329656513 | -0.8709730018 | -0.4411272107 |
| 34 | H | -2.5509781664 | -1.8829355890 | -0.8540020753 |
| 35 | H | -4.0274553314 | -2.6534916929 | -1.4087264934 |
| 36 | H | -3.7038358326 | -2.5643477097 | 0.3169058844 |

```
   37      H       0.3376301083     3.3677619306    -1.3607641014
   38      H      -1.3665603287     3.4789429408    -1.6225173579
 ----------------------------------------------------------------
 Nuclear Repulsion Energy =        1317.89541612 hartrees
 There are       65 alpha and       65 beta electrons

--------------------------------------------------
-  Entering fldman on Sun Oct 13 21:45:38 2024  -
--------------------------------------------------

 Applying Cartesian multipole field
    Component          Value
    ---------          -----
     (2,0,0)        1.00000E-12
     (0,2,0)        2.00000E-11
     (0,0,2)       -3.00000E-11
 Nucleus-field energy     =     0.0000000059 hartrees

--------------------------------------------------
-  Entering gesman on Sun Oct 13 21:45:38 2024  -
--------------------------------------------------

 Requested basis set is 6-311+G(d,p)
 There are 188 shells and 516 basis functions
 A cutoff of  1.0D-12 yielded  12987 shell pairs
 There are    102625 function pairs (    109350 Cartesian)
 Smallest overlap matrix eigenvalue = 2.29E-06
 Linear dependence detected in AO basis
 Tighter screening thresholds may be required for diffuse basis sets
 Use S2THRESH > 12 and THRESH = 14 in case of SCF convergence issues
 Number of orthogonalized atomic orbitals = 510
 Maximum deviation from orthogonality = 1.204E-11
 Guess MOs from SCF MO coefficient file
 Reading MOs from coefficient file
 Reading MOs from coefficient file

--------------------------------------------------
-  Entering scfman on Sun Oct 13 21:45:39 2024  -
--------------------------------------------------

 Long-range K will be added via erf
 Coulomb attenuation parameter = 0.2 bohr**(-1)
 A restricted hybrid HF-DFT SCF calculation will be
 performed using Pulay DIIS + Geometric Direct Minimization
 Exchange:     0.2220 Hartree-Fock + 1.0000 wB97X-D + LR-HF
 Correlation:  1.0000 wB97X-D
 Using Euler-Maclaurin-Lebedev (75,302) quadrature formula
 Dispersion:   Grimme D
 SCF converges when RMS gradient is below 1.0E-07
 Geometry optimization detected.  Setting ReadMinima to 0
 Setting SaveMinima to 0
 ---------------------------------------
  Cycle       Energy         DIIS Error
 ---------------------------------------
    1    -763.9524265667      1.09E-03
    2    -763.9693760388      1.30E-04
    3    -763.9703897204      7.91E-05
    4    -763.9705742041      3.73E-05
    5    -763.9706252575      1.28E-05
    6    -763.9706312643      3.65E-06
    7    -763.9706318788      1.00E-06
    8    -763.9706319501      4.96E-07
    9    -763.9706319632      1.37E-07
   10    -763.9706319648      6.27E-08 Convergence criterion met
 ---------------------------------------
 SCF time:  CPU 287.81 s  wall 467.76 s
 SCF   energy in the final basis set = -763.97063196
 Total energy in the final basis set = -763.97063196

--------------------------------------------------
```

```
-  Entering anlman on Sun Oct 13 21:53:26 2024  -
--------------------------------------------------



 --------------------------------------------------------------

                    Orbital Energies (a.u.)
 --------------------------------------------------------------

 Alpha MOs
 -- Occupied --
-14.8214 -14.7869 -14.7366 -14.7044 -10.6746 -10.6585 -10.6450 -10.6442
-10.6387 -10.6311 -10.6064 -10.5889 -10.5771 -10.5760 -10.5532 -10.5243
-10.4954 -10.4745  -1.4150  -1.4092  -1.3456  -1.2734  -1.2334  -1.2003
 -1.1931  -1.1430  -1.1033  -1.0878  -1.0541  -1.0529  -1.0377  -1.0067
 -0.9926  -0.9541  -0.9467  -0.9161  -0.9055  -0.8938  -0.8879  -0.8617
 -0.8549  -0.8477  -0.8391  -0.8258  -0.8060  -0.8044  -0.7924  -0.7866
 -0.7739  -0.7557  -0.7537  -0.7523  -0.7447  -0.7335  -0.7219  -0.7207
 -0.7018  -0.6676  -0.6643  -0.6441  -0.6359  -0.6292  -0.6261  -0.6227
 -0.6073
 -- Virtual --
 -0.2748  -0.2566  -0.2375  -0.2289  -0.1746  -0.1655  -0.1506  -0.1456
 -0.1374  -0.1179  -0.1164  -0.1134  -0.1086  -0.1066  -0.1008  -0.0973
 -0.0927  -0.0877  -0.0848  -0.0791  -0.0790  -0.0723  -0.0705  -0.0695
 -0.0642  -0.0607  -0.0561  -0.0544  -0.0513  -0.0475  -0.0402  -0.0374
 -0.0327  -0.0307  -0.0264  -0.0225  -0.0199  -0.0155  -0.0107  -0.0089
 -0.0040  -0.0033   0.0011   0.0021   0.0059   0.0064   0.0120   0.0166
  0.0195   0.0217   0.0233   0.0266   0.0310   0.0345   0.0396   0.0420
  0.0424   0.0442   0.0477   0.0517   0.0548   0.0607   0.0637   0.0646
  0.0675   0.0692   0.0724   0.0757   0.0810   0.0827   0.0863   0.0907
  0.0961   0.0979   0.1009   0.1045   0.1084   0.1103   0.1145   0.1193
  0.1207   0.1251   0.1289   0.1341   0.1394   0.1407   0.1420   0.1450
  0.1476   0.1570   0.1625   0.1636   0.1691   0.1728   0.1742   0.1810
  0.1841   0.1862   0.1883   0.1939   0.2003   0.2025   0.2224   0.2272
  0.2357   0.2486   0.2529   0.2770   0.2821   0.2872   0.3025   0.3126
  0.3216   0.3308   0.3340   0.3399   0.3463   0.3546   0.3613   0.3658
  0.3770   0.3815   0.3847   0.3937   0.3969   0.4021   0.4067   0.4212
  0.4240   0.4312   0.4381   0.4512   0.4585   0.4659   0.4756   0.4809
  0.4869   0.4890   0.4924   0.4997   0.5091   0.5150   0.5184   0.5226
  0.5242   0.5297   0.5343   0.5427   0.5468   0.5478   0.5561   0.5589
  0.5616   0.5658   0.5724   0.5767   0.5878   0.5923   0.5959   0.6039
  0.6074   0.6197   0.6240   0.6280   0.6347   0.6432   0.6478   0.6604
  0.6648   0.6676   0.6753   0.6848   0.6904   0.6959   0.7028   0.7087
  0.7164   0.7262   0.7446   0.7502   0.7537   0.7698   0.7804   0.7840
  0.7929   0.8029   0.8121   0.8192   0.8247   0.8408   0.8534   0.8579
  0.8680   0.8801   0.8875   0.8972   0.9121   0.9184   0.9397   0.9485
  0.9584   0.9676   0.9721   0.9925   1.0095   1.0213   1.0280   1.0395
  1.0485   1.0584   1.0705   1.1055   1.1234   1.1412   1.1630   1.1895
  1.1986   1.2104   1.2216   1.2317   1.2534   1.2683   1.2852   1.2996
  1.3113   1.3163   1.3326   1.3414   1.3558   1.3638   1.3698   1.3757
  1.3847   1.3899   1.3972   1.4048   1.4144   1.4232   1.4290   1.4339
  1.4379   1.4411   1.4490   1.4548   1.4663   1.4757   1.4866   1.4919
  1.5110   1.5164   1.5224   1.5323   1.5372   1.5421   1.5468   1.5507
  1.5553   1.5655   1.5745   1.5790   1.5879   1.5978   1.6026   1.6126
  1.6241   1.6264   1.6326   1.6593   1.6624   1.6709   1.6778   1.6820
  1.7001   1.7051   1.7171   1.7297   1.7371   1.7490   1.7578   1.7682
  1.7809   1.7937   1.8086   1.8167   1.8361   1.8431   1.8655   1.8839
  1.8976   1.9018   1.9152   1.9398   1.9645   1.9655   1.9883   2.0062
  2.0101   2.0144   2.0209   2.0359   2.0531   2.0556   2.0732   2.0949
  2.1133   2.1248   2.1358   2.1632   2.1654   2.1894   2.2100   2.2285
  2.2327   2.2362   2.2461   2.2580   2.2625   2.2680   2.2781   2.2880
  2.3048   2.3103   2.3264   2.3464   2.3583   2.3751   2.3886   2.3985
  2.4060   2.4302   2.4325   2.4436   2.4509   2.4671   2.4749   2.4876
  2.4921   2.5083   2.5188   2.5273   2.5365   2.5427   2.5529   2.5577
  2.5700   2.5738   2.5856   2.5871   2.5998   2.6006   2.6054   2.6138
  2.6299   2.6409   2.6614   2.6691   2.6760   2.6970   2.7011   2.7128
  2.7220   2.7294   2.7421   2.7453   2.7675   2.7680   2.7795   2.7906
  2.7939   2.8116   2.8166   2.8336   2.8487   2.8691   2.8842   2.8949
  2.8954   2.9120   2.9492   2.9945   3.0476   3.0606   3.1031   3.1494
  3.1763   3.2306   3.2474   3.2602   3.2970   3.3252   3.3773   3.4064
```

```
  3.4213   3.4812   3.5256   3.5353   3.5674   3.5925   3.6198   3.6471
  3.6809   3.6887   3.7048   3.7291   3.7358   3.7394   3.7465   3.7633
  3.7728   3.8321   3.8627   3.8981   3.9456   3.9771   4.0290   4.0752
  4.0946   4.2059   4.2879   4.3157   4.5945   4.6699   4.6856   4.7891
  4.9281   5.0284   5.2050  23.5309  23.5849  23.7235  23.7291  23.7393
 23.7759  23.8044  23.8281  23.8570  23.8918  23.9117  23.9599  23.9853
 24.0271  35.4116  35.4640  35.5267  35.6285
--------------------------------------------------------------

          Ground-State Mulliken Net Atomic Charges

     Atom                 Charge (a.u.)
  ----------------------------------------
      1 N                     0.312512
      2 C                     0.149098
      3 C                    -0.045023
      4 C                    -0.196865
      5 C                    -0.294817
      6 C                     0.010791
      7 C                    -0.799145
      8 C                    -0.171836
      9 N                    -0.157178
     10 C                    -0.220424
     11 N                     0.003816
     12 C                     0.271151
     13 C                    -0.106537
     14 C                     0.031028
     15 C                    -0.320066
     16 C                    -0.258388
     17 C                    -0.533464
     18 N                    -0.467741
     19 H                     0.222933
     20 H                     0.237794
     21 H                     0.234400
     22 H                     0.224749
     23 H                     0.227496
     24 H                     0.260643
     25 H                     0.216172
     26 H                     0.213544
     27 H                     0.252625
     28 H                     0.376989
     29 H                     0.208226
     30 H                     0.214031
     31 H                     0.250918
     32 H                     0.206831
     33 H                     0.224472
     34 H                     0.129462
     35 H                     0.216896
     36 H                     0.177582
     37 H                     0.318080
     38 H                     0.379247
  ----------------------------------------
  Sum of atomic charges =     2.000000

-----------------------------------------------------------------
                    Cartesian Multipole Moments
-----------------------------------------------------------------
    Charge (ESU x 10^10)
                 9.6064
    Dipole Moment (Debye)
         X       7.4731      Y       2.1614      Z       2.6230
       Tot       8.2097
    Quadrupole Moments (Debye-Ang)
        XX     -13.1314     XY      -6.8087     YY     -67.2676
        XZ       8.2871     YZ      -8.9464     ZZ     -76.8345
    Traceless Quadrupole Moments (Debye-Ang)
       QXX     117.8392    QYY     -44.5692    QZZ     -73.2700
       QXY     -20.4261    QXZ      24.8613    QYZ     -26.8392
    Octopole Moments (Debye-Ang^2)
       XXX      23.5980    XXY     -94.9979    XYY      14.0197
```

```
       YYY      10.8929    XXZ       57.2287    XYZ        0.8529
       YYZ     -43.3269    XZZ       27.2529    YZZ      -11.5683
       ZZZ     -17.8323
    Traceless Octopole Moments (Debye-Ang^2)
       XXX    -229.8654    YYY     1024.4538    ZZZ     -232.1103
       XXY   -1137.9489    XXZ      870.2225    XYY       15.6831
       XYZ      12.7929    XZZ      214.1823    YYZ     -638.1122
       YZZ     113.4951
    Hexadecapole Moments (Debye-Ang^3)
      XXXX   -3676.4806   XXXY     -100.2695   XXYY     -811.8660
      XYYY      69.6270   YYYY     -950.6499   XXXZ       54.1200
      XXYZ     -19.0668   XYYZ      -33.5174   YYYZ      -71.4625
      XXZZ    -715.0865   XYZZ       30.4321   YYZZ     -251.4027
      XZZZ    -158.3856   YZZZ       25.9458   ZZZZ     -679.0180
    Traceless Hexadecapole Moments (Debye-Ang^3)
      XXXX    2512.7828   XXXY   -10518.8313   XXXZ    11882.8342
      XXYY   -3574.2296   XXYZ    -1019.0750   XXZZ     1061.4468
      XYYY    7320.3039   XYYZ    -1452.5839   XYZZ     3198.5275
      XZZZ  -10430.2503   YYYY     1668.6995   YYYZ    -4554.7421
      YYZZ    1905.5301   YZZZ     5573.8171   ZZZZ    -2966.9769
 -----------------------------------------------------------------

 -------------------------------------------------
 -  Entering drvman on Sun Oct 13 21:53:26 2024  -
 -------------------------------------------------

 Calculating analytic gradient of the SCF energy
 Gradient of SCF Energy
            1           2           3           4           5           6
    1  -0.0002225  -0.0001167  -0.0004446  -0.0000991   0.0000576   0.0005003
    2   0.0001349   0.0004715  -0.0000891   0.0002067  -0.0002657  -0.0000579
    3   0.0005191  -0.0001101  -0.0003427  -0.0000313  -0.0000709  -0.0000310
            7           8           9          10          11          12
    1  -0.0005034   0.0002432  -0.0000500   0.0009021  -0.0002782   0.0003302
    2   0.0001025   0.0002813   0.0005729  -0.0006392   0.0007688  -0.0031695
    3   0.0001965  -0.0005328  -0.0002635   0.0006752  -0.0003287   0.0007222
           13          14          15          16          17          18
    1  -0.0006275  -0.0001418   0.0000343   0.0000080   0.0000533   0.0000862
    2  -0.0004273   0.0002976  -0.0000097   0.0001759   0.0000476   0.0025733
    3  -0.0005810   0.0003980  -0.0002738   0.0001904  -0.0000265   0.0013017
           19          20          21          22          23          24
    1   0.0000138  -0.0000119   0.0000765   0.0000602   0.0003331   0.0003179
    2  -0.0001288  -0.0000753  -0.0000848  -0.0000433  -0.0000279  -0.0001499
    3   0.0000133   0.0000793   0.0000122   0.0001895  -0.0001147   0.0000569
           25          26          27          28          29          30
    1  -0.0001134   0.0000782  -0.0003008  -0.0001634  -0.0001577  -0.0001043
    2   0.0004060  -0.0000423   0.0002981  -0.0000900  -0.0001300   0.0000594
    3  -0.0000672  -0.0001462   0.0000513   0.0001039   0.0000106   0.0000549
           31          32          33          34          35          36
    1  -0.0000521   0.0000328  -0.0000192  -0.0000076  -0.0000567   0.0000367
    2  -0.0000417  -0.0001070  -0.0001977   0.0000230  -0.0000091  -0.0000320
    3   0.0000016  -0.0000781  -0.0000314   0.0000473  -0.0000205   0.0000662
           37          38
    1   0.0006981  -0.0003916
    2  -0.0001444  -0.0004570
    3  -0.0004517  -0.0011880
 Max gradient component =       3.170E-03
 RMS gradient           =       5.009E-04
 Gradient time:  CPU 125.14 s  wall 195.59 s

 -------------------------------------------------
 -  Entering optman on Sun Oct 13 21:56:42 2024  -
 -------------------------------------------------


 Geometry Optimization Parameters
   NAtoms,    NIC,     NZ,  NCons,   NDum,   NFix, NCnnct, MaxDiis
      38     272       0       0       0       0       0       0

 Cartesian Hessian Update
 Hessian updated using BFGS update
```

```
** GEOMETRY OPTIMIZATION IN DELOCALIZED INTERNAL COORDINATES **
   Searching for a Minimum

   Optimization Cycle:  12

                    Coordinates (Angstroms)
     ATOM                X               Y               Z
      1  N          2.5720332225    0.4206003368    0.3575647298
      2  C          2.8288107266   -0.6097756715   -0.4988786799
      3  C          3.6859979276   -1.6146280423   -0.0731850294
      4  C          4.2778143332   -1.5636526423    1.1790229805
      5  C          4.0055818490   -0.4892976828    2.0231315306
      6  C          3.1503042158    0.4882939343    1.5850673452
      7  C          2.2260132293   -0.6142156617   -1.8673432670
      8  C          1.6488669187    1.5005143651   -0.0372705117
      9  N         -1.5565518242   -0.1124803463    1.1179433551
     10  C         -2.4960318089    0.6193071342    0.4626916291
     11  N         -2.1419164649    1.6173804967   -0.3035453698
     12  C         -0.8433506079    1.9313968822   -0.4469723724
     13  C          0.1923160475    1.1487111705    0.1723453148
     14  C         -0.2322942429    0.1333034442    0.9676354556
     15  C         -3.9271152305    0.2296326613    0.5919735480
     16  C         -4.3647768497   -0.6881325541   -0.5727626316
     17  C         -3.6152013428   -2.0150286355   -0.6263918176
     18  N         -0.5853766403    3.0053436889   -1.1859885038
     19  H          3.8951580126   -2.4353014976   -0.7475417832
     20  H          4.9515163219   -2.3517739654    1.4954672603
     21  H          4.4561828217   -0.4042318941    3.0032600843
     22  H          2.8995544951    1.3524750712    2.1873186928
     23  H          2.3933106156   -1.5817358473   -2.3375115938
     24  H          2.7116142811    0.1390667344   -2.4970697541
     25  H          1.1516543584   -0.4191207875   -1.8571938035
     26  H          1.8388883244    1.7294650890   -1.0873868593
     27  H          1.9141441590    2.3909462238    0.5382457163
     28  H         -1.8630746066   -0.8820570595    1.7007898645
     29  H          0.4363542099   -0.5260563259    1.5074818616
     30  H         -4.0929398169   -0.2753677105    1.5488352620
     31  H         -4.5195018828    1.1454159879    0.5848285372
     32  H         -4.2538195492   -0.1439537738   -1.5143298361
     33  H         -5.4329656513   -0.8709730018   -0.4411272107
     34  H         -2.5509781664   -1.8829355890   -0.8540020753
     35  H         -4.0274553314   -2.6534916929   -1.4087264934
     36  H         -3.7038358326   -2.5643477097    0.3169058844
     37  H          0.3376301083    3.3677619306   -1.3607641014
     38  H         -1.3665603287    3.4789429408   -1.6225173579
   Point Group: c1    Number of degrees of freedom:   108


   Energy is   -763.970631965

 Hessian updated using BFGS update
  internal optimization (0)

108 Hessian modes will be used to form the next step
  Hessian Eigenvalues:
     0.000404    0.002545    0.002627    0.004189    0.007155    0.011349
     0.015097    0.019302    0.019459    0.020076    0.020464    0.022631
     0.022733    0.023445    0.024096    0.025529    0.025976    0.026262
     0.027833    0.028243    0.029126    0.029856    0.033383    0.036764
     0.038906    0.040942    0.043201    0.043720    0.045051    0.045764
     0.048906    0.054144    0.055369    0.057732    0.063033    0.079833
     0.085531    0.094890    0.121815    0.122199    0.127158    0.131161
     0.132085    0.133043    0.136511    0.141352    0.143770    0.145196
     0.146497    0.147790    0.151184    0.152861    0.153394    0.153817
     0.157751    0.161228    0.193749    0.206422    0.212397    0.217546
     0.226711    0.233442    0.238346    0.249544    0.252838    0.262571
     0.269179    0.274882    0.281875    0.291388    0.300190    0.300462
     0.300730    0.301245    0.301889    0.302424    0.303939    0.304214
```

```
    0.305133    0.305474    0.307604    0.308540    0.311472    0.315254
    0.320110    0.332157    0.333162    0.336048    0.337100    0.340511
    0.343171    0.356197    0.363894    0.367416    0.389024    0.392104
    0.401194    0.406812    0.418325    0.419745    0.424615    0.437941
    0.448148    0.466829    0.477826    0.491639    0.556744    0.693554

 Minimum search - taking simple RFO step
 Searching for Lamda that Minimizes Along All modes
 Value Taken    Lamda =  -0.00117280
 Calculated Step too Large.  Step scaled by  0.395734
 Step Taken.  Stepsize is  0.300000

                              Maximum     Tolerance    Cnvgd?
         Gradient            0.003561      0.000800      NO
         Displacement        0.127375      0.001400      NO
         Energy change      -0.000372      0.000228      NO


 New Cartesian Coordinates Obtained by Inverse Iteration

 Displacement from previous Coordinates is:  1.442756
 ----------------------------------------------------------------
             Standard Nuclear Orientation (Angstroms)
    I     Atom           X                Y                Z
 ----------------------------------------------------------------
    1      N       2.5838540251     0.4161951190     0.3263255176
    2      C       2.8835142643    -0.6363871019    -0.4877130072
    3      C       3.7685614055    -1.5958496950    -0.0149679754
    4      C       4.3429308688    -1.4794185918     1.2407775725
    5      C       4.0222887425    -0.3858894329     2.0426651841
    6      C       3.1413123285     0.5453226843     1.5589297375
    7      C       2.2955126741    -0.7171637337    -1.8600799730
    8      C       1.6365584243     1.4551623530    -0.1149169638
    9      N      -1.5436931329    -0.1910167310     1.0688757523
   10      C      -2.4940350544     0.5892516217     0.4907178448
   11      N      -2.1519551646     1.6244888325    -0.2308896022
   12      C      -0.8550577165     1.9265432810    -0.4031420884
   13      C       0.1897688907     1.0997698055     0.1392511229
   14      C      -0.2205032709     0.0460066903     0.8894138143
   15      C      -3.9257857125     0.2152924611     0.6531349566
   16      C      -4.4283586046    -0.6184063300    -0.5480137256
   17      C      -3.7148706590    -1.9566538955    -0.7087932069
   18      N      -0.6022150894     3.0297171311    -1.0994256542
   19      H       4.0118170242    -2.4334047604    -0.6562944303
   20      H       5.0387584783    -2.2321640239     1.5932415731
   21      H       4.4561402608    -0.2505839577     3.0246500446
   22      H       2.8532071207     1.4206928268     2.1274323178
   23      H       2.4965242251    -1.6988755556    -2.2854900047
   24      H       2.7657960619     0.0217736933    -2.5175626224
   25      H       1.2158277433    -0.5535162356    -1.8695434096
   26      H       1.8007668272     1.6316122420    -1.1799831324
   27      H       1.8959255222     2.3797483931     0.4081698301
   28      H      -1.8423558808    -0.9893475109     1.6159160824
   29      H       0.4558988253    -0.6507740300     1.3692111208
   30      H      -4.0634010578    -0.3470992100     1.5820545118
   31      H      -4.4995441911     1.1398969669     0.7287230790
   32      H      -4.3378445590    -0.0205814310    -1.4586625850
   33      H      -5.4953484083    -0.7840015242    -0.3874158709
   34      H      -2.6562747481    -1.8357850342    -0.9667522406
   35      H      -4.1698368563    -2.5366312864    -1.5126390968
   36      H      -3.7847155607    -2.5599557653     0.2024719529
   37      H       0.3198662592     3.3922440020    -1.2786894374
   38      H      -1.3890343057     3.5397877337    -1.4809869881
 ----------------------------------------------------------------
 Nuclear Repulsion Energy =        1315.68170758 hartrees
 There are       65 alpha and       65 beta electrons

 ------------------------------------------------
 -  Entering fldman on Sun Oct 13 21:56:42 2024  -
 ------------------------------------------------
```

```
 Applying Cartesian multipole field
    Component          Value
    ---------          -----
     (2,0,0)        1.00000E-12
     (0,2,0)        2.00000E-11
     (0,0,2)       -3.00000E-11
 Nucleus-field energy     =     0.0000000060 hartrees

--------------------------------------------------
-  Entering gesman on Sun Oct 13 21:56:42 2024  -
--------------------------------------------------

 Requested basis set is 6-311+G(d,p)
 There are 188 shells and 516 basis functions
 A cutoff of  1.0D-12 yielded  12951 shell pairs
 There are    102361 function pairs (    109083 Cartesian)
 Smallest overlap matrix eigenvalue = 2.31E-06
 Linear dependence detected in AO basis
 Tighter screening thresholds may be required for diffuse basis sets
 Use S2THRESH > 12 and THRESH = 14 in case of SCF convergence issues
 Number of orthogonalized atomic orbitals = 510
 Maximum deviation from orthogonality = 1.286E-11
 Guess MOs from SCF MO coefficient file
 Reading MOs from coefficient file
 Reading MOs from coefficient file

--------------------------------------------------
-  Entering scfman on Sun Oct 13 21:56:42 2024  -
--------------------------------------------------

 Long-range K will be added via erf
 Coulomb attenuation parameter = 0.2 bohr**(-1)
 A restricted hybrid HF-DFT SCF calculation will be
 performed using Pulay DIIS + Geometric Direct Minimization
 Exchange:     0.2220 Hartree-Fock + 1.0000 wB97X-D + LR-HF
 Correlation:  1.0000 wB97X-D
 Using Euler-Maclaurin-Lebedev (75,302) quadrature formula
 Dispersion:   Grimme D
 SCF converges when RMS gradient is below 1.0E-07
 Geometry optimization detected.  Setting ReadMinima to 0
 Setting SaveMinima to 0
 ---------------------------------------
  Cycle       Energy         DIIS Error
 ---------------------------------------
    1    -763.9530210900      1.21E-03
    2    -763.9694440926      1.51E-04
    3    -763.9706464890      1.03E-04
    4    -763.9709618328      4.11E-05
    5    -763.9710251652      1.50E-05
    6    -763.9710333821      4.13E-06
    7    -763.9710341637      1.20E-06
    8    -763.9710342543      6.19E-07
    9    -763.9710342748      1.53E-07
   10    -763.9710342768      6.56E-08 Convergence criterion met
 ---------------------------------------
 SCF time:  CPU 288.61 s  wall 447.96 s
 SCF   energy in the final basis set = -763.97103428
 Total energy in the final basis set = -763.97103428

--------------------------------------------------
-  Entering anlman on Sun Oct 13 22:04:10 2024  -
--------------------------------------------------


 --------------------------------------------------------------

                   Orbital Energies (a.u.)
 --------------------------------------------------------------
```

```
 Alpha MOs
 -- Occupied --
-14.8215 -14.7866 -14.7365 -14.7041 -10.6741 -10.6583 -10.6448 -10.6444
-10.6389 -10.6309 -10.6062 -10.5889 -10.5770 -10.5759 -10.5529 -10.5239
-10.4948 -10.4735  -1.4149  -1.4091  -1.3455  -1.2734  -1.2335  -1.2003
 -1.1931  -1.1430  -1.1031  -1.0877  -1.0542  -1.0525  -1.0376  -1.0062
 -0.9926  -0.9539  -0.9463  -0.9158  -0.9049  -0.8934  -0.8883  -0.8617
 -0.8542  -0.8480  -0.8392  -0.8256  -0.8060  -0.8045  -0.7924  -0.7870
 -0.7737  -0.7553  -0.7537  -0.7517  -0.7443  -0.7335  -0.7217  -0.7201
 -0.7011  -0.6668  -0.6647  -0.6433  -0.6353  -0.6285  -0.6261  -0.6222
 -0.6072
 -- Virtual --
 -0.2749  -0.2559  -0.2370  -0.2286  -0.1748  -0.1655  -0.1508  -0.1456
 -0.1376  -0.1177  -0.1162  -0.1133  -0.1086  -0.1060  -0.1000  -0.0967
 -0.0927  -0.0881  -0.0842  -0.0798  -0.0790  -0.0722  -0.0697  -0.0688
 -0.0646  -0.0605  -0.0567  -0.0535  -0.0513  -0.0461  -0.0410  -0.0372
 -0.0339  -0.0306  -0.0260  -0.0219  -0.0207  -0.0158  -0.0111  -0.0084
 -0.0047  -0.0041   0.0016   0.0030   0.0067   0.0072   0.0120   0.0171
  0.0192   0.0211   0.0246   0.0256   0.0316   0.0341   0.0393   0.0403
  0.0424   0.0443   0.0495   0.0520   0.0549   0.0606   0.0640   0.0651
  0.0675   0.0690   0.0725   0.0731   0.0812   0.0840   0.0859   0.0918
  0.0959   0.0973   0.1007   0.1055   0.1088   0.1113   0.1144   0.1182
  0.1204   0.1265   0.1300   0.1333   0.1382   0.1409   0.1434   0.1456
  0.1464   0.1600   0.1625   0.1644   0.1699   0.1735   0.1746   0.1800
  0.1828   0.1844   0.1884   0.1909   0.2002   0.2020   0.2210   0.2303
  0.2369   0.2496   0.2530   0.2752   0.2834   0.2870   0.3036   0.3132
  0.3205   0.3296   0.3343   0.3402   0.3462   0.3525   0.3630   0.3688
  0.3761   0.3800   0.3863   0.3945   0.3964   0.4036   0.4054   0.4205
  0.4237   0.4318   0.4394   0.4492   0.4596   0.4652   0.4758   0.4846
  0.4872   0.4913   0.4940   0.4991   0.5113   0.5142   0.5185   0.5214
  0.5251   0.5322   0.5340   0.5411   0.5481   0.5497   0.5555   0.5583
  0.5613   0.5631   0.5728   0.5765   0.5862   0.5902   0.5947   0.6030
  0.6069   0.6200   0.6258   0.6323   0.6342   0.6446   0.6475   0.6575
  0.6644   0.6671   0.6738   0.6821   0.6907   0.6954   0.7027   0.7079
  0.7208   0.7260   0.7449   0.7498   0.7593   0.7702   0.7769   0.7835
  0.7934   0.8049   0.8137   0.8200   0.8256   0.8392   0.8521   0.8598
  0.8677   0.8813   0.8872   0.8961   0.9114   0.9178   0.9408   0.9493
  0.9582   0.9664   0.9794   0.9947   1.0068   1.0206   1.0277   1.0344
  1.0488   1.0615   1.0717   1.1095   1.1212   1.1389   1.1647   1.1950
  1.1963   1.2065   1.2221   1.2270   1.2537   1.2634   1.2858   1.3000
  1.3133   1.3179   1.3312   1.3396   1.3585   1.3613   1.3692   1.3762
  1.3855   1.3930   1.3994   1.4043   1.4119   1.4216   1.4296   1.4343
  1.4386   1.4427   1.4479   1.4539   1.4661   1.4754   1.4869   1.4920
  1.5072   1.5149   1.5229   1.5335   1.5397   1.5437   1.5471   1.5496
  1.5577   1.5653   1.5748   1.5811   1.5851   1.5991   1.6024   1.6136
  1.6227   1.6275   1.6335   1.6603   1.6618   1.6708   1.6779   1.6855
  1.7010   1.7087   1.7160   1.7306   1.7376   1.7526   1.7574   1.7700
  1.7819   1.7916   1.8069   1.8148   1.8348   1.8453   1.8662   1.8840
  1.8980   1.9033   1.9166   1.9433   1.9656   1.9712   1.9911   2.0069
  2.0094   2.0151   2.0219   2.0363   2.0537   2.0635   2.0743   2.1022
  2.1128   2.1253   2.1341   2.1585   2.1671   2.1896   2.2107   2.2305
  2.2327   2.2386   2.2482   2.2590   2.2630   2.2706   2.2788   2.2849
  2.3031   2.3083   2.3275   2.3459   2.3590   2.3772   2.3889   2.3988
  2.4074   2.4305   2.4329   2.4454   2.4507   2.4684   2.4753   2.4891
  2.4924   2.5048   2.5184   2.5261   2.5378   2.5421   2.5518   2.5576
  2.5717   2.5746   2.5844   2.5859   2.5998   2.6024   2.6046   2.6148
  2.6314   2.6392   2.6589   2.6680   2.6758   2.6970   2.7048   2.7112
  2.7203   2.7314   2.7421   2.7454   2.7682   2.7762   2.7801   2.7897
  2.7982   2.8126   2.8176   2.8337   2.8467   2.8738   2.8814   2.8898
  2.8966   2.9150   2.9517   2.9960   3.0437   3.0609   3.1077   3.1504
  3.1814   3.2329   3.2482   3.2633   3.2953   3.3262   3.3769   3.4083
  3.4235   3.4822   3.5256   3.5361   3.5692   3.5923   3.6188   3.6467
  3.6784   3.6902   3.7052   3.7285   3.7347   3.7394   3.7474   3.7638
  3.7718   3.8308   3.8632   3.8981   3.9456   3.9759   4.0307   4.0741
  4.0966   4.2062   4.2868   4.3178   4.5956   4.6698   4.6847   4.7972
  4.9287   5.0291   5.2078  23.5337  23.5869  23.7245  23.7291  23.7412
 23.7754  23.8047  23.8269  23.8580  23.8929  23.9123  23.9605  23.9875
 24.0276  35.4127  35.4646  35.5281  35.6301
 --------------------------------------------------------------
```

Ground-State Mulliken Net Atomic Charges

| Atom | | Charge (a.u.) |
|---|---|---|
| 1 | N | 0.310662 |
| 2 | C | 0.188041 |
| 3 | C | -0.063444 |
| 4 | C | -0.203801 |
| 5 | C | -0.259122 |
| 6 | C | -0.047580 |
| 7 | C | -0.820451 |
| 8 | C | -0.183834 |
| 9 | N | -0.158679 |
| 10 | C | -0.225790 |
| 11 | N | 0.005728 |
| 12 | C | 0.281519 |
| 13 | C | -0.070773 |
| 14 | C | 0.030004 |
| 15 | C | -0.324646 |
| 16 | C | -0.254806 |
| 17 | C | -0.532642 |
| 18 | N | -0.472113 |
| 19 | H | 0.222663 |
| 20 | H | 0.237951 |
| 21 | H | 0.234164 |
| 22 | H | 0.224730 |
| 23 | H | 0.227922 |
| 24 | H | 0.261404 |
| 25 | H | 0.211521 |
| 26 | H | 0.228551 |
| 27 | H | 0.247541 |
| 28 | H | 0.376807 |
| 29 | H | 0.208444 |
| 30 | H | 0.213477 |
| 31 | H | 0.250899 |
| 32 | H | 0.206720 |
| 33 | H | 0.223979 |
| 34 | H | 0.130402 |
| 35 | H | 0.216675 |
| 36 | H | 0.177490 |
| 37 | H | 0.321389 |
| 38 | H | 0.378999 |

Sum of atomic charges = 2.000000

Cartesian Multipole Moments

Charge (ESU x 10^10)

9.6064

Dipole Moment (Debye)

| | | | | | |
|---|---|---|---|---|---|
| X | 7.6122 | Y | 2.0843 | Z | 2.5836 |
| Tot | 8.3045 | | | | |

Quadrupole Moments (Debye-Ang)

| | | | | | |
|---|---|---|---|---|---|
| XX | -11.4535 | XY | -6.7637 | YY | -66.9167 |
| XZ | 8.4297 | YZ | -8.4579 | ZZ | -77.9573 |

Traceless Quadrupole Moments (Debye-Ang)

| | | | | | |
|---|---|---|---|---|---|
| QXX | 121.9671 | QYY | -44.4227 | QZZ | -77.5444 |
| QXY | -20.2911 | QXZ | 25.2890 | QYZ | -25.3737 |

Octopole Moments (Debye-Ang^2)

| | | | | | |
|---|---|---|---|---|---|
| XXX | 28.6849 | XXY | -95.2043 | XYY | 11.4508 |
| YYY | 15.4966 | XXZ | 58.4336 | XYZ | 1.8231 |
| YYZ | -42.0043 | XZZ | 28.2985 | YZZ | -13.2930 |
| ZZZ | -19.1488 | | | | |

Traceless Octopole Moments (Debye-Ang^2)

| | | | | | |
|---|---|---|---|---|---|
| XXX | -185.6342 | YYY | 1069.4554 | ZZZ | -262.7563 |
| XXY | -1149.0622 | XXZ | 884.6629 | XYY | -33.5405 |
| XYZ | 27.3471 | XZZ | 219.1747 | YYZ | -621.9066 |
| YZZ | 79.6067 | | | | |

Hexadecapole Moments (Debye-Ang^3)

```
      XXXX   -3722.6047   XXXY    -104.3122   XXYY    -832.9211
      XYYY      66.1283   YYYY    -916.7718   XXXZ      65.4635
      XXYZ     -18.2374   XYYZ     -38.0211   YYYZ     -80.0668
      XXZZ    -724.0981   XYZZ      33.1390   YYZZ    -253.9718
      XZZZ    -158.6643   YZZZ      23.2853   ZZZZ    -674.9367
    Traceless Hexadecapole Moments (Debye-Ang^3)
      XXXX    3866.0035   XXXY  -10725.7599   XXXZ   12778.6532
      XXYY   -5016.2744   XXYZ    -785.3664   XXZZ    1150.2709
      XYYY    7170.4909   XYYZ   -2023.8869   XYZZ    3555.2690
      XZZZ  -10754.7663   YYYY    3642.1308   YYYZ   -5018.3216
      YYZZ    1374.1436   YZZZ    5803.6880   ZZZZ   -2524.4144
 -----------------------------------------------------------------

-------------------------------------------------
-  Entering drvman on Sun Oct 13 22:04:10 2024  -
-------------------------------------------------

 Calculating analytic gradient of the SCF energy
 Gradient of SCF Energy
            1           2           3           4           5           6
    1  -0.0002004  -0.0003618  -0.0002483   0.0000015  -0.0001842   0.0005965
    2   0.0003179   0.0007754  -0.0003238   0.0001819  -0.0000357  -0.0003083
    3   0.0005657  -0.0003417  -0.0002752  -0.0000359  -0.0000481   0.0000005
            7           8           9          10          11          12
    1  -0.0004242   0.0001663  -0.0001643   0.0007537  -0.0000176   0.0001155
    2  -0.0000895   0.0000858   0.0009742  -0.0006400   0.0007075  -0.0028082
    3   0.0002357  -0.0007455  -0.0002817   0.0004662  -0.0002736   0.0009553
           13          14          15          16          17          18
    1  -0.0003625   0.0001502   0.0002571  -0.0003058   0.0001749   0.0004541
    2  -0.0008267   0.0003769  -0.0001618   0.0003364   0.0000281   0.0019219
    3  -0.0002916   0.0003588  -0.0003615   0.0001769  -0.0000025   0.0010533
           19          20          21          22          23          24
    1  -0.0000638  -0.0000676   0.0000717   0.0000724   0.0003933   0.0003228
    2  -0.0000731  -0.0000809  -0.0000157  -0.0000298  -0.0000665  -0.0002055
    3  -0.0000401   0.0000356  -0.0000324   0.0002616  -0.0000926   0.0001473
           25          26          27          28          29          30
    1  -0.0001178  -0.0002805  -0.0004253  -0.0002315  -0.0002038  -0.0001235
    2   0.0004287  -0.0000181   0.0004701  -0.0001258  -0.0001622   0.0000407
    3  -0.0001117  -0.0003921   0.0001781  -0.0000384   0.0000309   0.0001203
           31          32          33          34          35          36
    1  -0.0000188   0.0000282   0.0000030  -0.0000176   0.0000202  -0.0000284
    2   0.0000006  -0.0001587  -0.0001462  -0.0000788  -0.0000564   0.0000298
    3   0.0001005  -0.0001006  -0.0000416  -0.0000350   0.0000054   0.0000660
           37          38
    1   0.0005832  -0.0003170
    2   0.0001524  -0.0004169
    3  -0.0001508  -0.0010650
 Max gradient component =       2.808E-03
 RMS gradient           =       4.646E-04
 Gradient time:  CPU 112.39 s  wall 185.42 s

-------------------------------------------------
-  Entering optman on Sun Oct 13 22:07:16 2024  -
-------------------------------------------------

 Geometry Optimization Parameters
   NAtoms,    NIC,     NZ,  NCons,   NDum,   NFix, NCnnct, MaxDiis
       38     272       0       0       0       0       0       0

 Cartesian Hessian Update
 Hessian updated using BFGS update


** GEOMETRY OPTIMIZATION IN DELOCALIZED INTERNAL COORDINATES **
   Searching for a Minimum

   Optimization Cycle:  13

                        Coordinates (Angstroms)
     ATOM                X               Y               Z
```

| | | | | |
|---|---|---|---|---|
| 1 | N | 2.5838540251 | 0.4161951190 | 0.3263255176 |
| 2 | C | 2.8835142643 | -0.6363871019 | -0.4877130072 |
| 3 | C | 3.7685614055 | -1.5958496950 | -0.0149679754 |
| 4 | C | 4.3429308688 | -1.4794185918 | 1.2407775725 |
| 5 | C | 4.0222887425 | -0.3858894329 | 2.0426651841 |
| 6 | C | 3.1413123285 | 0.5453226843 | 1.5589297375 |
| 7 | C | 2.2955126741 | -0.7171637337 | -1.8600799730 |
| 8 | C | 1.6365584243 | 1.4551623530 | -0.1149169638 |
| 9 | N | -1.5436931329 | -0.1910167310 | 1.0688757523 |
| 10 | C | -2.4940350544 | 0.5892516217 | 0.4907178448 |
| 11 | N | -2.1519551646 | 1.6244888325 | -0.2308896022 |
| 12 | C | -0.8550577165 | 1.9265432810 | -0.4031420884 |
| 13 | C | 0.1897688907 | 1.0997698055 | 0.1392511229 |
| 14 | C | -0.2205032709 | 0.0460066903 | 0.8894138143 |
| 15 | C | -3.9257857125 | 0.2152924611 | 0.6531349566 |
| 16 | C | -4.4283586046 | -0.6184063300 | -0.5480137256 |
| 17 | C | -3.7148706590 | -1.9566538955 | -0.7087932069 |
| 18 | N | -0.6022150894 | 3.0297171311 | -1.0994256542 |
| 19 | H | 4.0118170242 | -2.4334047604 | -0.6562944303 |
| 20 | H | 5.0387584783 | -2.2321640239 | 1.5932415731 |
| 21 | H | 4.4561402608 | -0.2505839577 | 3.0246500446 |
| 22 | H | 2.8532071207 | 1.4206928268 | 2.1274323178 |
| 23 | H | 2.4965242251 | -1.6988755556 | -2.2854900047 |
| 24 | H | 2.7657960619 | 0.0217736933 | -2.5175626224 |
| 25 | H | 1.2158277433 | -0.5535162356 | -1.8695434096 |
| 26 | H | 1.8007668272 | 1.6316122420 | -1.1799831324 |
| 27 | H | 1.8959255222 | 2.3797483931 | 0.4081698301 |
| 28 | H | -1.8423558808 | -0.9893475109 | 1.6159160824 |
| 29 | H | 0.4558988253 | -0.6507740300 | 1.3692111208 |
| 30 | H | -4.0634010578 | -0.3470992100 | 1.5820545118 |
| 31 | H | -4.4995441911 | 1.1398969669 | 0.7287230790 |
| 32 | H | -4.3378445590 | -0.0205814310 | -1.4586625850 |

***Abridged***Abridged***Abridged***

| | | | | | | | |
|---|---|---|---|---|---|---|---|
| 0.0444 | 0.0452 | 0.0531 | 0.0553 | 0.0578 | 0.0613 | 0.0647 | 0.0668 |
| 0.0701 | 0.0709 | 0.0740 | 0.0781 | 0.0850 | 0.0856 | 0.0874 | 0.0934 |
| 0.0949 | 0.1008 | 0.1052 | 0.1100 | 0.1129 | 0.1138 | 0.1161 | 0.1206 |
| 0.1290 | 0.1307 | 0.1334 | 0.1357 | 0.1408 | 0.1445 | 0.1460 | 0.1488 |
| 0.1567 | 0.1600 | 0.1644 | 0.1665 | 0.1725 | 0.1770 | 0.1781 | 0.1815 |
| 0.1847 | 0.1907 | 0.1948 | 0.1987 | 0.2072 | 0.2121 | 0.2262 | 0.2330 |
| 0.2419 | 0.2489 | 0.2549 | 0.2659 | 0.2712 | 0.2768 | 0.2929 | 0.2987 |
| 0.3015 | 0.3166 | 0.3228 | 0.3263 | 0.3322 | 0.3365 | 0.3406 | 0.3487 |
| 0.3541 | 0.3611 | 0.3636 | 0.3652 | 0.3758 | 0.3769 | 0.3837 | 0.3875 |
| 0.3986 | 0.4011 | 0.4068 | 0.4142 | 0.4183 | 0.4226 | 0.4317 | 0.4362 |
| 0.4435 | 0.4450 | 0.4490 | 0.4515 | 0.4609 | 0.4651 | 0.4661 | 0.4682 |
| 0.4721 | 0.4753 | 0.4773 | 0.4804 | 0.4836 | 0.4870 | 0.4935 | 0.4965 |
| 0.4999 | 0.5036 | 0.5096 | 0.5106 | 0.5168 | 0.5193 | 0.5264 | 0.5287 |
| 0.5341 | 0.5348 | 0.5366 | 0.5429 | 0.5455 | 0.5495 | 0.5578 | 0.5609 |
| 0.5644 | 0.5693 | 0.5732 | 0.5772 | 0.5811 | 0.5854 | 0.5884 | 0.5944 |
| 0.6029 | 0.6079 | 0.6108 | 0.6166 | 0.6240 | 0.6265 | 0.6297 | 0.6327 |
| 0.6359 | 0.6453 | 0.6533 | 0.6574 | 0.6646 | 0.6693 | 0.6797 | 0.6842 |
| 0.6911 | 0.7095 | 0.7149 | 0.7153 | 0.7236 | 0.7263 | 0.7310 | 0.7491 |
| 0.7509 | 0.7553 | 0.7615 | 0.7733 | 0.7845 | 0.7904 | 0.7973 | 0.8035 |
| 0.8185 | 0.8232 | 0.8305 | 0.8350 | 0.8399 | 0.8439 | 0.8477 | 0.8526 |
| 0.8703 | 0.8710 | 0.8761 | 0.8838 | 0.9026 | 0.9051 | 0.9141 | 0.9213 |
| 0.9263 | 0.9333 | 0.9376 | 0.9434 | 0.9465 | 0.9502 | 0.9622 | 0.9760 |
| 0.9831 | 0.9839 | 0.9950 | 1.0022 | 1.0076 | 1.0099 | 1.0197 | 1.0285 |
| 1.0350 | 1.0441 | 1.0469 | 1.0563 | 1.0612 | 1.0685 | 1.0773 | 1.0809 |
| 1.0864 | 1.0912 | 1.0942 | 1.1062 | 1.1110 | 1.1145 | 1.1266 | 1.1295 |
| 1.1354 | 1.1426 | 1.1500 | 1.1572 | 1.1627 | 1.1668 | 1.1692 | 1.1747 |
| 1.1771 | 1.1817 | 1.1894 | 1.1943 | 1.2006 | 1.2109 | 1.2163 | 1.2243 |
| 1.2265 | 1.2373 | 1.2454 | 1.2483 | 1.2559 | 1.2649 | 1.2701 | 1.2754 |
| 1.2798 | 1.2830 | 1.2887 | 1.2933 | 1.2976 | 1.3075 | 1.3140 | 1.3190 |
| 1.3324 | 1.3340 | 1.3362 | 1.3429 | 1.3456 | 1.3537 | 1.3679 | 1.3684 |
| 1.3795 | 1.3852 | 1.3958 | 1.4019 | 1.4082 | 1.4174 | 1.4232 | 1.4249 |
| 1.4257 | 1.4390 | 1.4508 | 1.4553 | 1.4611 | 1.4721 | 1.4776 | 1.4852 |
| 1.4937 | 1.5006 | 1.5168 | 1.5177 | 1.5364 | 1.5425 | 1.5458 | 1.5523 |
| 1.5625 | 1.5671 | 1.5736 | 1.5923 | 1.6082 | 1.6184 | 1.6251 | 1.6444 |
| 1.6455 | 1.6728 | 1.6981 | 1.7004 | 1.7318 | 1.7489 | 1.7578 | 1.7900 |

| | | | | | | | |
|---|---|---|---|---|---|---|---|
| 1.8051 | 1.8113 | 1.8379 | 1.8505 | 1.8640 | 1.8735 | 1.9175 | 1.9398 |
| 1.9654 | 1.9776 | 2.0206 | 2.0401 | 2.0980 | 2.1279 | 2.1816 | 2.2009 |
| 2.2649 | 2.3338 | 2.3428 | 2.3711 | 2.3922 | 2.4075 | 2.4402 | 2.4564 |
| 2.4614 | 2.4639 | 2.4732 | 2.4831 | 2.4885 | 2.5084 | 2.5153 | 2.5262 |
| 2.5337 | 2.5449 | 2.5572 | 2.5583 | 2.5661 | 2.5777 | 2.5835 | 2.5895 |
| 2.6047 | 2.6075 | 2.6162 | 2.6185 | 2.6352 | 2.6447 | 2.6485 | 2.6544 |
| 2.6675 | 2.6733 | 2.6750 | 2.6864 | 2.6882 | 2.7030 | 2.7077 | 2.7174 |
| 2.7206 | 2.7238 | 2.7269 | 2.7335 | 2.7380 | 2.7468 | 2.7518 | 2.7632 |
| 2.7741 | 2.7747 | 2.8015 | 2.8068 | 2.8128 | 2.8178 | 2.8263 | 2.8410 |
| 2.8437 | 2.8492 | 2.8516 | 2.8643 | 2.8673 | 2.8832 | 2.8845 | 2.8894 |
| 2.8905 | 2.9019 | 2.9097 | 2.9147 | 2.9309 | 2.9369 | 2.9434 | 2.9462 |
| 2.9574 | 2.9623 | 2.9672 | 2.9746 | 2.9854 | 2.9945 | 3.0033 | 3.0120 |
| 3.0193 | 3.0222 | 3.0341 | 3.0369 | 3.0415 | 3.0502 | 3.0562 | 3.0616 |
| 3.0752 | 3.0772 | 3.0849 | 3.0977 | 3.1026 | 3.1135 | 3.1140 | 3.1229 |
| 3.1325 | 3.1364 | 3.1429 | 3.1529 | 3.1587 | 3.1654 | 3.1738 | 3.1788 |
| 3.1926 | 3.1989 | 3.2047 | 3.2078 | 3.2189 | 3.2262 | 3.2309 | 3.2359 |
| 3.2397 | 3.2483 | 3.2539 | 3.2584 | 3.2629 | 3.2719 | 3.2761 | 3.2784 |
| 3.2867 | 3.2895 | 3.2996 | 3.3010 | 3.3054 | 3.3170 | 3.3268 | 3.3344 |
| 3.3424 | 3.3463 | 3.3529 | 3.3649 | 3.3802 | 3.3879 | 3.3914 | 3.4011 |
| 3.4124 | 3.4169 | 3.4311 | 3.4347 | 3.4451 | 3.4470 | 3.4549 | 3.4686 |
| 3.4743 | 3.4892 | 3.4991 | 3.5150 | 3.5208 | 3.5234 | 3.5342 | 3.5410 |
| 3.5427 | 3.5567 | 3.5655 | 3.5679 | 3.5789 | 3.5825 | 3.5892 | 3.5971 |
| 3.6099 | 3.6234 | 3.6261 | 3.6366 | 3.6467 | 3.6506 | 3.6535 | 3.6598 |
| 3.6681 | 3.6806 | 3.6889 | 3.6980 | 3.7144 | 3.7200 | 3.7290 | 3.7332 |
| 3.7446 | 3.7552 | 3.7602 | 3.7665 | 3.7708 | 3.7845 | 3.7946 | 3.8020 |
| 3.8066 | 3.8235 | 3.8339 | 3.8376 | 3.8475 | 3.8536 | 3.8579 | 3.8731 |
| 3.8777 | 3.8804 | 3.9097 | 3.9180 | 3.9247 | 3.9337 | 3.9352 | 3.9404 |
| 3.9547 | 3.9567 | 3.9673 | 3.9722 | 3.9757 | 3.9932 | 4.0076 | 4.0128 |
| 4.0162 | 4.0335 | 4.0440 | 4.0516 | 4.0729 | 4.0883 | 4.0926 | 4.1026 |
| 4.1086 | 4.1182 | 4.1261 | 4.1345 | 4.1430 | 4.1483 | 4.1579 | 4.1642 |
| 4.1695 | 4.1730 | 4.1885 | 4.1920 | 4.2006 | 4.2173 | 4.2281 | 4.2334 |
| 4.2385 | 4.2466 | 4.2591 | 4.2799 | 4.2929 | 4.3017 | 4.3046 | 4.3103 |
| 4.3250 | 4.3289 | 4.3355 | 4.3385 | 4.3538 | 4.3712 | 4.3761 | 4.3865 |
| 4.3954 | 4.4002 | 4.4090 | 4.4187 | 4.4249 | 4.4369 | 4.4416 | 4.4542 |
| 4.4682 | 4.4730 | 4.4857 | 4.4918 | 4.4970 | 4.5142 | 4.5164 | 4.5226 |
| 4.5455 | 4.5551 | 4.5635 | 4.5845 | 4.5875 | 4.6076 | 4.6208 | 4.6317 |
| 4.6459 | 4.6602 | 4.6985 | 4.7093 | 4.7222 | 4.7386 | 4.7407 | 4.7649 |
| 4.8113 | 4.8183 | 4.8487 | 4.8652 | 4.8897 | 4.9069 | 4.9103 | 4.9215 |
| 4.9413 | 4.9472 | 4.9649 | 4.9890 | 4.9909 | 5.0006 | 5.0253 | 5.0509 |
| 5.0615 | 5.0695 | 5.0907 | 5.1103 | 5.1139 | 5.1243 | 5.1898 | 5.2056 |
| 5.2243 | 5.2379 | 5.2564 | 5.2761 | 5.2813 | 5.3035 | 5.3513 | 5.3691 |
| 5.4038 | 5.4162 | 5.4411 | 5.4577 | 5.5145 | 5.5252 | 5.5397 | 5.5621 |
| 5.5699 | 5.5906 | 5.5935 | 5.6196 | 5.6518 | 5.6568 | 5.6695 | 5.6970 |
| 5.7005 | 5.7228 | 5.7407 | 5.7581 | 5.7835 | 5.8069 | 5.8349 | 5.8798 |
| 5.9414 | 5.9549 | 5.9865 | 6.0050 | 6.0795 | 6.0883 | 6.1279 | 6.1818 |
| 6.2597 | 6.2998 | 6.3452 | 6.5330 | 6.5765 | 6.8060 | 23.9739 | 24.1382 |
| 24.1599 | 24.2427 | 24.2587 | 24.2831 | 24.3046 | 24.3465 | 24.4252 | 24.6078 |
| 24.6172 | 24.6266 | 24.8047 | 24.9171 | 35.7624 | 35.7713 | 35.7862 | 35.9453 |

Ground-State Mulliken Net Atomic Charges

| Atom | Charge (a.u.) |
|---|---|
| 1 N | 0.321827 |
| 2 C | 0.134204 |
| 3 C | -0.228927 |
| 4 C | -0.162629 |
| 5 C | -0.269001 |
| 6 C | 0.025447 |
| 7 C | -0.367291 |
| 8 C | -0.051347 |
| 9 N | -0.202520 |
| 10 C | 0.078176 |
| 11 N | -0.318225 |
| 12 C | 0.517765 |
| 13 C | -0.084129 |
| 14 C | 0.020036 |
| 15 C | -0.272461 |
| 16 C | -0.078519 |

```
    17 C                    -0.442871
    18 N                    -0.379124
    19 H                     0.199404
    20 H                     0.215070
    21 H                     0.204868
    22 H                     0.208504
    23 H                     0.162773
    24 H                     0.187733
    25 H                     0.164685
    26 H                     0.188391
    27 H                     0.183177
    28 H                     0.286256
    29 H                     0.186360
    30 H                     0.150663
    31 H                     0.176972
    32 H                     0.143908
    33 H                     0.150591
    34 H                     0.099302
    35 H                     0.163086
    36 H                     0.134819
    37 H                     0.244313
    38 H                     0.308717
 ----------------------------------------
 Sum of atomic charges =     2.000000

-----------------------------------------------------------------
                   Cartesian Multipole Moments
-----------------------------------------------------------------
   Charge (ESU x 10^10)
                9.6064
   Dipole Moment (Debye)
        X       7.6699      Y       2.0976      Z       2.5037
      Tot       8.3364
   Quadrupole Moments (Debye-Ang)
        XX      -9.2704     XY      -7.3908     YY     -66.7245
        XZ       8.8663     YZ      -8.0892     ZZ     -78.9929
   Traceless Quadrupole Moments (Debye-Ang)
      QXX     127.1765    QYY     -45.1857    QZZ     -81.9909
      QXY     -22.1725    QXZ      26.5989    QYZ     -24.2676
   Octopole Moments (Debye-Ang^2)
      XXX      36.9411    XXY     -94.7336    XYY       7.2199
      YYY      20.9098    XXZ      60.7636    XYZ       2.7406
      YYZ     -40.7278    XZZ      29.1164    YZZ     -13.5090
      ZZZ     -20.2136
   Traceless Octopole Moments (Debye-Ang^2)
      XXX    -105.3799    YYY    1099.6425    ZZZ    -301.6034
      XXY   -1159.0055    XXZ     911.9874    XYY    -111.5335
      XYZ      41.1090    XZZ     216.9134    YYZ    -610.3840
      YZZ      59.3629
   Hexadecapole Moments (Debye-Ang^3)
     XXXX   -3770.3311   XXXY    -112.4205   XXYY    -856.5126
     XYYY      61.3177   YYYY    -871.8261   XXXZ      77.1157
     XXYZ     -17.3745   XYYZ     -44.6547   YYYZ     -86.2575
     XXZZ    -739.0218   XYZZ      36.2867   YYZZ    -254.4722
     XZZZ    -162.6282   YZZZ      20.4944   ZZZZ    -680.5988
   Traceless Hexadecapole Moments (Debye-Ang^3)
     XXXX    5838.2054   XXXY  -11137.4213   XXXZ   13954.6720
     XXYY   -6771.9802   XXYZ    -569.8436   XXZZ     933.7748
     XYYY    7105.0806   XYYZ   -2736.2361   XYZZ    4032.3407
     XZZZ  -11218.4359   YYYY    5706.3158   YYYZ   -5293.5959
     YYZZ    1065.6644   YZZZ    5863.4394   ZZZZ   -1999.4392
-----------------------------------------------------------------
Total job time:  553.99s(wall), 395.05s(cpu)
Mon Oct 14 03:46:26 2024
```

```
Wavefunction Version of Q-Chem

 Parts of Q-Chem use Armadillo 8.300.2 (Tropical Shenanigans).
 http://arma.sourceforge.net/

 Q-Chem begins on Sun Oct 13 21:06:46 2024


     Scratch files written to
C:/Users/hille/AppData/Local/Temp/WFB0CB2A5C99266D7A/Conformer.16//scratch///
Processing default memory
  ... MEM_TOTAL 4076 MB (default) [16 cores]
Processing $rem in C:/Program
Files/Wavefunction/Spartan24v110/P4e//../auxdir/config/preferences:
   (site specific preferences)
  ... THRESH              9
  ... SMALL_PROD_XCMAT   9
  ... BASIS_LIN_DEP_THRESH     5
  ... SCF_ALGORITHM     DIIS_GDM
  ... MAXSCF            250
  ... MAXDIIS          45
  ... THRESHDIIS         -1  (i.e. don't switch on delta-E)
  ... ECP_FIT          TRUE (Convert deprecated ECP files)
  ... GUI              GUI_SPARTAN
  ... TERSE_OUTPUT     TRUE !turn on spartan printing
  ... SCF_CONVERGENCE  7
  ... CCMAN2 FALSE  (qc4.3)
  ... SYMMETRY   FALSE  ! turn of symmetry for spartan16
  ... SYM_IGNORE TRUE   ! ..use FORCESYMMETRY to override
  ... GEOM_OPT_TOL_GRADIENT       700  ! loosen tolernaces for organic geometries
  ... GEOM_OPT_TOL_DISPLACEMENT  1400  ! was 1200 = .0012
  ... GEOM_OPT_TOL_ENERGY        2000  ! was  100 = .000 001
  ... GEN_SCFMAN  FALSE
Processing $rem in input file
  ... JOBTYPE    OPT
  ... TIDY_SYM   TRUE
  ... METHOD            WB97X-D
  ... xc_grid           75000302  (75,302)
  ... BASIS             6-311+G**
  ... THRESH             12  #diffuse default
  ... MAXSCF            350  #diffuse default
  ... GEOM_OPT_TOL_ENERGY       22850
  ... GEOM_OPT_TOL_GRADIENT       800
  ... VARTHRESH         2    (default DFT)
  ... INCDFT            TRUE (default DFT)
  ... GEOM_OPT_HESSIAN      READ (main opt)
  ... EXTERNAL_HESSIAN      1
  ... GUI                GUI_SPARTAN
  ... TERSE_OUTPUT       TRUE
NAlpha2: 130
NElect   130
Mult     1

Checking the input file for inconsistencies...  ...done.

--------------------------------------------------------------
User input:
--------------------------------------------------------------
$comment
Molecule15
$end
$molecule
 2 1
    7     2.8661792801   0.086146062765   -0.10001503464
    6     3.0163434453   -0.81598375368    -1.1220152754
    6     3.9351108583    -1.8688456395   -0.97364925672
    6     4.7186458026     -1.969493067     0.1662829089
    6     4.5917787136    -1.0121000158     1.1552341006
    6      3.668739202   0.011036413499    0.99675806694
```

```
    6     2.2174613871   -0.73151245773    -2.3941813061
    6     1.8978732416     1.2224892427   -0.18433843859
    7    -1.2650398211   -0.22644017767     1.2926301266
    6    -2.134866448   0.012105905682    0.30101508739
    7    -1.7744688794    0.64941623662   -0.83586669794
    6   -0.50382847884     1.0498173696   -0.95909606222
    6    0.46019640125    0.80009594796    0.01001766909
    6   0.019117642033     0.1495488752     1.1666665989
    6    -3.5679746843   -0.40762006866    0.47319880556
    6    -4.3700710304    0.66878121578     1.2017331204
    6    -5.8222083566     0.2551412548     1.3788439333
    7   -0.20719513421     1.6977857349     -2.145049782
    1     4.0608269627    -2.6099425676     -1.764377201
    1     5.4429747881    -2.7780825204    0.26747158405
    1     5.2236995877    -1.0573736536     2.0411475788
    1     3.5726325002    0.78293060177      1.757029537
    1     2.6403275601    -1.3800002071    -3.1693749697
    1     2.2281859425    0.28326508894    -2.7983946882
    1     1.1896476359    -1.0568825509    -2.2149659045
    1      2.091936601     1.7256080999    -1.1361858419
    1     2.1531357801     1.9531260611    0.59302519362
    1    -1.6005414147   -0.69611204754     2.1361297705
    1    0.65757824381  -0.089338510337        2.0108173
    1     -4.005321394   -0.60364475698   -0.51299131879
    1    -3.6010321873    -1.3507253496     1.0312664811
    1    -4.3297385244     1.6118040245    0.64284123757
    1    -3.9293741528    0.86586691511     2.1867172237
    1    -6.3030020375   0.086600884245    0.40994866481
    1     -6.378509181     1.0390243635     1.9022612075
    1    -5.8988931367   -0.66561216784     1.9659226966
    1   0.089352431244     2.6671023975    -2.0452702591
    1    -1.0496791461     1.7020168158     -2.731186856
$end
$rem
JOBTYPE    OPT
TIDY_SYM   TRUE
METHOD          WB97X-D
xc_grid         75000302  (75,302)
BASIS           6-311+G**
THRESH           12  #diffuse default
MAXSCF          350  #diffuse default
GEOM_OPT_TOL_ENERGY     22850
GEOM_OPT_TOL_GRADIENT     800
VARTHRESH        2    (default DFT)
INCDFT          TRUE (default DFT)
GEOM_OPT_HESSIAN      READ (main opt)
EXTERNAL_HESSIAN      1
GUI              GUI_SPARTAN
TERSE_OUTPUT     TRUE
$end
$opt
$end
--------------------------------------------------------------
 --------------------------------------------------------------
             Standard Nuclear Orientation (Angstroms)
    I     Atom           X                Y                Z
 --------------------------------------------------------------
    1      N       2.8661792801     0.0861460628    -0.1000150346
    2      C       3.0163434453    -0.8159837537    -1.1220152754
    3      C       3.9351108583    -1.8688456395    -0.9736492567
    4      C       4.7186458026    -1.9694930670     0.1662829089
    5      C       4.5917787136    -1.0121000158     1.1552341006
    6      C       3.6687392020     0.0110364135     0.9967580669
    7      C       2.2174613871    -0.7315124577    -2.3941813061
    8      C       1.8978732416     1.2224892427    -0.1843384386
    9      N      -1.2650398211    -0.2264401777     1.2926301266
   10      C      -2.1348664480     0.0121059057     0.3010150874
   11      N      -1.7744688794     0.6494162366    -0.8358666979
   12      C      -0.5038284788     1.0498173696    -0.9590960622
   13      C       0.4601964012     0.8000959480     0.0100176691
```

```
   14      C         0.0191176420     0.1495488752     1.1666665989
   15      C        -3.5679746843    -0.4076200687     0.4731988056
   16      C        -4.3700710304     0.6687812158     1.2017331204
   17      C        -5.8222083566     0.2551412548     1.3788439333
   18      N        -0.2071951342     1.6977857349    -2.1450497820
   19      H         4.0608269627    -2.6099425676    -1.7643772010
   20      H         5.4429747881    -2.7780825204     0.2674715840
   21      H         5.2236995877    -1.0573736536     2.0411475788
   22      H         3.5726325002     0.7829306018     1.7570295370
   23      H         2.6403275601    -1.3800002071    -3.1693749697
   24      H         2.2281859425     0.2832650889    -2.7983946882
   25      H         1.1896476359    -1.0568825509    -2.2149659045
   26      H         2.0919366010     1.7256080999    -1.1361858419
   27      H         2.1531357801     1.9531260611     0.5930251936
   28      H        -1.6005414147    -0.6961120475     2.1361297705
   29      H         0.6575782438    -0.0893385103     2.0108173000
   30      H        -4.0053213940    -0.6036447570    -0.5129913188
   31      H        -3.6010321873    -1.3507253496     1.0312664811
   32      H        -4.3297385244     1.6118040245     0.6428412376
   33      H        -3.9293741528     0.8658669151     2.1867172237
   34      H        -6.3030020375     0.0866008842     0.4099486648
   35      H        -6.3785091810     1.0390243635     1.9022612075
   36      H        -5.8988931367    -0.6656121678     1.9659226966
   37      H         0.0893524312     2.6671023975    -2.0452702591
   38      H        -1.0496791461     1.7020168158    -2.7311868560
 ----------------------------------------------------------------
 Nuclear Repulsion Energy =        1295.54503186 hartrees
 There are       65 alpha and       65 beta electrons
 Requested basis set is 6-311+G(d,p)
 There are 188 shells and 516 basis functions

 Total QAlloc Memory Limit   4076 MB
 Mega-Array Size       188 MB
 MEM_STATIC part       192 MB

 ..                                                                   (5.2.P)

------------------------------------------------
-  Entering fldman on Sun Oct 13 21:06:46 2024  -
------------------------------------------------

 A cutoff of  1.0D-12 yielded  12514 shell pairs
 There are     99956 function pairs (    106539 Cartesian)
 Smallest overlap matrix eigenvalue = 1.51E-06
 Linear dependence detected in AO basis
 Tighter screening thresholds may be required for diffuse basis sets
 Use S2THRESH > 12 and THRESH = 14 in case of SCF convergence issues
 Number of orthogonalized atomic orbitals = 510
 Maximum deviation from orthogonality = 1.117E-11

 Scale SEOQF with 1.000000e-02/1.000000e-01/1.000000e-01

 Standard Electronic Orientation quadrupole field applied
 Nucleus-field energy     =    -0.0000000077 hartrees

------------------------------------------------
-  Entering gesman on Sun Oct 13 21:06:46 2024  -
------------------------------------------------

 Guess from superposition of atomic densities
 Warning:  Energy on first SCF cycle will be non-variational
 SAD guess density has 132.000000 electrons

------------------------------------------------
-  Entering scfman on Sun Oct 13 21:06:46 2024  -
------------------------------------------------

 Long-range K will be added via erf
 Coulomb attenuation parameter = 0.2 bohr**(-1)
 A restricted hybrid HF-DFT SCF calculation will be
```

```
 performed using Pulay DIIS + Geometric Direct Minimization
 Exchange:     0.2220 Hartree-Fock + 1.0000 wB97X-D + LR-HF
 Correlation:  1.0000 wB97X-D
 Using Euler-Maclaurin-Lebedev (75,302) quadrature formula
 Dispersion:   Grimme D
 SCF converges when RMS gradient is below 1.0E-07
 Exchange:     0.2220 Hartree-Fock + 1.0000 wB97X-D + LR-HF
 Correlation:  1.0000 wB97X-D
 Using Euler-Maclaurin-Lebedev (75,302) quadrature formula
 Dispersion:   Grimme D
 ---------------------------------------
  Cycle       Energy         DIIS Error
 ---------------------------------------
    1    -770.2987699198      2.95E-02
    2    -763.7469952311      2.41E-03
    3    -763.7468144423      2.64E-03
    4    -763.9443758810      3.66E-04
    5    -763.9476082589      1.89E-04
    6    -763.9486260119      3.29E-05
    7    -763.9486589650      1.38E-05
    8    -763.9486649740      3.03E-06
    9    -763.9486654930      1.45E-06
   10    -763.9486655904      4.57E-07
   11    -763.9486656070      2.19E-07
   12    -763.9486656107      8.67E-08 Convergence criterion met
 ---------------------------------------
 SCF time:  CPU 386.17 s  wall 635.15 s
 SCF   energy in the final basis set = -763.94866561
 Total energy in the final basis set = -763.94866561

--------------------------------------------------
-  Entering anlman on Sun Oct 13 21:17:21 2024  -
--------------------------------------------------



 --------------------------------------------------------------

                   Orbital Energies (a.u.)
 --------------------------------------------------------------


 Alpha MOs
 -- Occupied --
-14.8178 -14.8131 -14.7213 -14.7135 -10.6734 -10.6692 -10.6632 -10.6393
-10.6356 -10.6273 -10.6138 -10.5790 -10.5708 -10.5707 -10.5440 -10.5324
-10.4982 -10.4535  -1.4288  -1.3992  -1.3435  -1.2653  -1.2280  -1.2002
 -1.1795  -1.1462  -1.1033  -1.0769  -1.0630  -1.0492  -1.0286  -1.0055
 -0.9844  -0.9513  -0.9431  -0.9041  -0.9005  -0.8879  -0.8757  -0.8656
 -0.8471  -0.8412  -0.8341  -0.8189  -0.8059  -0.7921  -0.7823  -0.7747
 -0.7714  -0.7694  -0.7497  -0.7440  -0.7316  -0.7260  -0.7190  -0.7054
 -0.6836  -0.6695  -0.6562  -0.6475  -0.6362  -0.6230  -0.6206  -0.6148
 -0.6144
 -- Virtual --
 -0.2783  -0.2682  -0.2545  -0.2270  -0.1787  -0.1631  -0.1530  -0.1463
 -0.1299  -0.1259  -0.1181  -0.1150  -0.1117  -0.1037  -0.0994  -0.0914
 -0.0877  -0.0867  -0.0829  -0.0813  -0.0765  -0.0711  -0.0679  -0.0653
 -0.0611  -0.0589  -0.0573  -0.0510  -0.0485  -0.0447  -0.0369  -0.0349
 -0.0322  -0.0255  -0.0237  -0.0236  -0.0195  -0.0148  -0.0135  -0.0079
 -0.0044   0.0003   0.0009   0.0060   0.0099   0.0122   0.0138   0.0157
  0.0197   0.0210   0.0266   0.0283   0.0298   0.0345   0.0367   0.0389
  0.0401   0.0428   0.0511   0.0526   0.0543   0.0578   0.0585   0.0620
  0.0689   0.0718   0.0748   0.0791   0.0834   0.0861   0.0894   0.0919
  0.0934   0.0968   0.1015   0.1055   0.1091   0.1140   0.1158   0.1187
  0.1207   0.1250   0.1261   0.1300   0.1375   0.1392   0.1412   0.1489
  0.1528   0.1550   0.1607   0.1610   0.1644   0.1683   0.1717   0.1739
  0.1778   0.1809   0.1896   0.1909   0.1978   0.2033   0.2126   0.2143
  0.2232   0.2276   0.2538   0.2701   0.2729   0.2921   0.2958   0.3120
  0.3238   0.3244   0.3368   0.3431   0.3494   0.3519   0.3568   0.3693
  0.3709   0.3785   0.3849   0.3925   0.3959   0.4067   0.4112   0.4184
  0.4245   0.4345   0.4434   0.4493   0.4590   0.4675   0.4709   0.4738
  0.4836   0.4867   0.4942   0.4987   0.5042   0.5058   0.5139   0.5225
```

| | | | | | | | |
|---|---|---|---|---|---|---|---|
| 0.5256 | 0.5298 | 0.5383 | 0.5407 | 0.5507 | 0.5527 | 0.5536 | 0.5574 |
| 0.5631 | 0.5708 | 0.5722 | 0.5830 | 0.5848 | 0.5869 | 0.5919 | 0.5960 |
| 0.6079 | 0.6153 | 0.6244 | 0.6315 | 0.6372 | 0.6418 | 0.6533 | 0.6584 |
| 0.6684 | 0.6718 | 0.6778 | 0.6839 | 0.6870 | 0.6936 | 0.6952 | 0.7015 |
| 0.7141 | 0.7267 | 0.7320 | 0.7388 | 0.7602 | 0.7694 | 0.7735 | 0.7881 |
| 0.7917 | 0.7940 | 0.7984 | 0.8122 | 0.8239 | 0.8354 | 0.8416 | 0.8487 |
| 0.8631 | 0.8680 | 0.8744 | 0.8793 | 0.8873 | 0.8993 | 0.9102 | 0.9169 |
| 0.9271 | 0.9404 | 0.9545 | 0.9732 | 0.9862 | 0.9897 | 1.0049 | 1.0225 |
| 1.0463 | 1.0667 | 1.1060 | 1.1098 | 1.1191 | 1.1450 | 1.1612 | 1.1824 |
| 1.2100 | 1.2221 | 1.2332 | 1.2525 | 1.2663 | 1.2853 | 1.2925 | 1.3070 |
| 1.3154 | 1.3277 | 1.3315 | 1.3386 | 1.3567 | 1.3619 | 1.3655 | 1.3749 |
| 1.3799 | 1.3877 | 1.3985 | 1.4056 | 1.4112 | 1.4210 | 1.4288 | 1.4358 |
| 1.4395 | 1.4439 | 1.4557 | 1.4580 | 1.4640 | 1.4752 | 1.4854 | 1.4934 |
| 1.4994 | 1.5048 | 1.5139 | 1.5256 | 1.5309 | 1.5418 | 1.5442 | 1.5496 |
| 1.5594 | 1.5664 | 1.5674 | 1.5825 | 1.5858 | 1.5951 | 1.6090 | 1.6134 |
| 1.6249 | 1.6292 | 1.6365 | 1.6441 | 1.6551 | 1.6715 | 1.6747 | 1.6972 |
| 1.7021 | 1.7106 | 1.7220 | 1.7338 | 1.7429 | 1.7544 | 1.7565 | 1.7664 |
| 1.7773 | 1.7878 | 1.8065 | 1.8256 | 1.8404 | 1.8452 | 1.8662 | 1.8715 |
| 1.8860 | 1.8938 | 1.8996 | 1.9209 | 1.9529 | 1.9570 | 1.9631 | 1.9895 |
| 1.9981 | 2.0129 | 2.0212 | 2.0344 | 2.0399 | 2.0487 | 2.0696 | 2.0754 |
| 2.0815 | 2.1124 | 2.1172 | 2.1238 | 2.1554 | 2.1874 | 2.1892 | 2.2105 |
| 2.2219 | 2.2356 | 2.2471 | 2.2482 | 2.2676 | 2.2877 | 2.2913 | 2.3014 |
| 2.3178 | 2.3253 | 2.3436 | 2.3597 | 2.3662 | 2.3744 | 2.3818 | 2.4009 |
| 2.4154 | 2.4195 | 2.4341 | 2.4494 | 2.4625 | 2.4717 | 2.4768 | 2.4850 |
| 2.4956 | 2.5024 | 2.5121 | 2.5208 | 2.5321 | 2.5377 | 2.5386 | 2.5533 |
| 2.5662 | 2.5771 | 2.5832 | 2.5837 | 2.5912 | 2.5955 | 2.6127 | 2.6217 |
| 2.6246 | 2.6357 | 2.6524 | 2.6562 | 2.6654 | 2.6801 | 2.6899 | 2.6978 |
| 2.7059 | 2.7148 | 2.7162 | 2.7213 | 2.7244 | 2.7512 | 2.7576 | 2.7604 |
| 2.7812 | 2.7903 | 2.8114 | 2.8301 | 2.8407 | 2.8479 | 2.8554 | 2.8813 |
| 2.8872 | 2.9131 | 2.9284 | 2.9559 | 3.0126 | 3.0658 | 3.0931 | 3.1316 |
| 3.1411 | 3.1722 | 3.2251 | 3.2637 | 3.2770 | 3.3009 | 3.3342 | 3.3735 |
| 3.3992 | 3.4635 | 3.4987 | 3.5217 | 3.5398 | 3.5725 | 3.6011 | 3.6671 |
| 3.6778 | 3.6877 | 3.7019 | 3.7284 | 3.7309 | 3.7445 | 3.7537 | 3.7589 |
| 3.8128 | 3.8566 | 3.8953 | 3.9008 | 3.9495 | 4.0278 | 4.0496 | 4.0861 |
| 4.1164 | 4.1932 | 4.2967 | 4.3124 | 4.5575 | 4.6414 | 4.6837 | 4.7051 |
| 4.8767 | 5.0176 | 5.1448 | 23.5166 | 23.5639 | 23.6921 | 23.7153 | 23.7332 |
| 23.7922 | 23.8088 | 23.8210 | 23.8276 | 23.8732 | 23.9158 | 23.9205 | 23.9283 |
| 24.0252 | 35.4096 | 35.4413 | 35.4832 | 35.5771 | | | |

Ground-State Mulliken Net Atomic Charges

| Atom | Charge (a.u.) |
|---|---|
| 1 N | 0.442797 |
| 2 C | 0.052919 |
| 3 C | -0.040989 |
| 4 C | -0.209236 |
| 5 C | -0.293445 |
| 6 C | -0.068070 |
| 7 C | -0.712986 |
| 8 C | -0.214772 |
| 9 N | -0.074174 |
| 10 C | -0.393130 |
| 11 N | 0.092914 |
| 12 C | 0.067685 |
| 13 C | 0.013294 |
| 14 C | 0.148885 |
| 15 C | -0.374642 |
| 16 C | -0.095757 |
| 17 C | -0.601080 |
| 18 N | -0.459008 |
| 19 H | 0.224495 |
| 20 H | 0.234050 |
| 21 H | 0.231978 |
| 22 H | 0.220959 |
| 23 H | 0.232362 |
| 24 H | 0.218562 |
| 25 H | 0.232524 |
| 26 H | 0.225833 |

```
    27 H                     0.250146
    28 H                     0.402959
    29 H                     0.219031
    30 H                     0.244157
    31 H                     0.210097
    32 H                     0.201114
    33 H                     0.168499
    34 H                     0.178513
    35 H                     0.199268
    36 H                     0.167809
    37 H                     0.319835
    38 H                     0.336603
 ----------------------------------------
 Sum of atomic charges =     2.000000

 -----------------------------------------------------------------
                    Cartesian Multipole Moments
 -----------------------------------------------------------------
    Charge (ESU x 10^10)
                9.6064
    Dipole Moment (Debye)
         X       8.4409      Y      -0.4202      Z       1.9853
       Tot       8.6814
    Quadrupole Moments (Debye-Ang)
        XX      -0.5416     XY     -16.5032     YY     -78.2690
        XZ      -6.7894     YZ      -4.7449     ZZ     -71.9035
    Traceless Quadrupole Moments (Debye-Ang)
       QXX     149.0891    QYY     -84.0928    QZZ     -64.9964
       QXY     -49.5095    QXZ     -20.3683    QYZ     -14.2347
    Octopole Moments (Debye-Ang^2)
       XXX      42.5483    XXY     -95.7170    XYY      24.5458
       YYY       9.0625    XXZ      68.9428    XYZ      16.2614
       YYZ     -23.4885    XZZ      -8.1375    YZZ       1.5469
       ZZZ       1.1324
    Traceless Octopole Moments (Debye-Ang^2)
       XXX     107.6148    YYY     901.9058    ZZZ    -402.2941
       XXY   -1180.4328    XXZ     894.3817    XYY     191.3169
       XYZ     243.9212    XZZ    -298.9317    YYZ    -492.0876
       YZZ     278.5270
    Hexadecapole Moments (Debye-Ang^3)
      XXXX   -4225.7839   XXXY    -116.6860   XXYY    -882.7889
      XYYY     343.7978   YYYY    -618.7570   XXXZ     375.5634
      XXYZ      39.7571   XYYZ      89.5388   YYYZ     -10.4839
      XXZZ    -995.9016   XYZZ     103.3750   YYZZ    -220.3087
      XZZZ     338.5321   YZZZ      -0.5255   ZZZZ    -883.6981
    Traceless Hexadecapole Moments (Debye-Ang^3)
      XXXX   16359.2497   XXXY  -27123.9355   XXXZ    3270.6144
      XXYY   -5076.6104   XXYZ    3735.3991   XXZZ  -11282.6394
      XYYY   21226.8613   XYYZ   -2652.9436   XYZZ    5897.0742
      XZZZ    -617.6708   YYYY     661.2918   YYYZ   -2418.1060
      YYZZ    4415.3186   YZZZ   -1317.2931   ZZZZ    6867.3208
 -----------------------------------------------------------------

-------------------------------------------------
-  Entering drvman on Sun Oct 13 21:17:21 2024  -
-------------------------------------------------

 Calculating analytic gradient of the SCF energy
 Gradient of SCF Energy
           1           2           3           4           5           6
    1  -0.0071365  -0.0050180   0.0019801  -0.0007545   0.0041204  -0.0081117
    2   0.0016576   0.0156758  -0.0069297   0.0055906  -0.0121488   0.0097932
    3   0.0008971   0.0066450  -0.0030560   0.0071773  -0.0074893  -0.0063255
           7           8           9          10          11          12
    1  -0.0023736  -0.0092722  -0.0043161   0.0228784   0.0036691   0.0201500
    2  -0.0048204  -0.0000668  -0.0022273  -0.0080094   0.0225992  -0.0151682
    3  -0.0036535  -0.0044246  -0.0007871   0.0433770  -0.0373918   0.0289412
          13          14          15          16          17          18
    1  -0.0089782  -0.0184136  -0.0098799   0.0037993   0.0037327   0.0184843
    2   0.0169152  -0.0122305   0.0053715  -0.0097541   0.0015891  -0.0025337
```

```
   3  -0.0319362   0.0133581   0.0043009  -0.0049004  -0.0007649  -0.0074865
          19          20          21          22          23          24
   1   0.0040797   0.0032924   0.0044970   0.0027926  -0.0001455   0.0045877
   2  -0.0067019  -0.0029068  -0.0038201   0.0010953  -0.0006834   0.0002783
   3  -0.0015543   0.0002229   0.0023460   0.0044961  -0.0065312  -0.0003251
          25          26          27          28          29          30
   1   0.0016226   0.0075020  -0.0037879  -0.0034387  -0.0020011  -0.0040014
   2  -0.0028935   0.0029806   0.0050481  -0.0030839  -0.0007352  -0.0023645
   3   0.0015824  -0.0015289   0.0019038   0.0040949   0.0022864  -0.0020079
          31          32          33          34          35          36
   1   0.0004949  -0.0020408  -0.0007970  -0.0000609  -0.0010645   0.0010170
   2  -0.0006854   0.0022642   0.0000261  -0.0002456   0.0020515  -0.0009746
   3  -0.0009903   0.0002478   0.0003372  -0.0015488   0.0013593   0.0005950
          37          38
   1  -0.0092737  -0.0078342
   2   0.0051911   0.0008566
   3   0.0006512  -0.0021170
 Max gradient component =       4.338E-02
 RMS gradient           =       9.527E-03
 Gradient time:  CPU 132.34 s  wall 211.83 s

-------------------------------------------------
-  Entering optman on Sun Oct 13 21:20:53 2024  -
-------------------------------------------------

 Geometry Optimization Parameters
   NAtoms,    NIC,     NZ,  NCons,   NDum,   NFix, NCnnct, MaxDiis
       38     272       0       0       0       0       0       0

 Cartesian Hessian read from HESS file


** GEOMETRY OPTIMIZATION IN DELOCALIZED INTERNAL COORDINATES **
   Searching for a Minimum

   Optimization Cycle:   1

                     Coordinates (Angstroms)
     ATOM                X               Y               Z
      1  N         2.8661792801    0.0861460628   -0.1000150346
      2  C         3.0163434453   -0.8159837537   -1.1220152754
      3  C         3.9351108583   -1.8688456395   -0.9736492567
      4  C         4.7186458026   -1.9694930670    0.1662829089
      5  C         4.5917787136   -1.0121000158    1.1552341006
      6  C         3.6687392020    0.0110364135    0.9967580669
      7  C         2.2174613871   -0.7315124577   -2.3941813061
      8  C         1.8978732416    1.2224892427   -0.1843384386
      9  N        -1.2650398211   -0.2264401777    1.2926301266
     10  C        -2.1348664480    0.0121059057    0.3010150874
     11  N        -1.7744688794    0.6494162366   -0.8358666979
     12  C        -0.5038284788    1.0498173696   -0.9590960622
     13  C         0.4601964013    0.8000959480    0.0100176691
     14  C         0.0191176420    0.1495488752    1.1666665989
     15  C        -3.5679746843   -0.4076200687    0.4731988056
     16  C        -4.3700710304    0.6687812158    1.2017331204
     17  C        -5.8222083566    0.2551412548    1.3788439333
     18  N        -0.2071951342    1.6977857349   -2.1450497820
     19  H         4.0608269627   -2.6099425676   -1.7643772010
     20  H         5.4429747881   -2.7780825204    0.2674715840
     21  H         5.2236995877   -1.0573736536    2.0411475788
     22  H         3.5726325002    0.7829306018    1.7570295370
     23  H         2.6403275601   -1.3800002071   -3.1693749697
     24  H         2.2281859425    0.2832650889   -2.7983946882
     25  H         1.1896476359   -1.0568825509   -2.2149659045
     26  H         2.0919366010    1.7256080999   -1.1361858419
     27  H         2.1531357801    1.9531260611    0.5930251936
     28  H        -1.6005414147   -0.6961120475    2.1361297705
     29  H         0.6575782438   -0.0893385103    2.0108173000
     30  H        -4.0053213940   -0.6036447570   -0.5129913188
     31  H        -3.6010321873   -1.3507253496    1.0312664811
```

```
    32  H         -4.3297385244    1.6118040245    0.6428412376
    33  H         -3.9293741528    0.8658669151    2.1867172237
    34  H         -6.3030020375    0.0866008842    0.4099486648
    35  H         -6.3785091810    1.0390243635    1.9022612075
    36  H         -5.8988931367   -0.6656121678    1.9659226966
    37  H          0.0893524312    2.6671023975   -2.0452702591
    38  H         -1.0496791461    1.7020168158   -2.7311868560
  Point Group: c1    Number of degrees of freedom:   108


  Energy is   -763.948665611


 Attempting to generate delocalized internal coordinates

 Transforming Cartesian Hessian to Internal Coordinates
 Hessian Transformation does not Include Derivative of B-matrix
  internal optimization (0)

108 Hessian modes will be used to form the next step
  Hessian Eigenvalues:
     0.002199    0.002740    0.003725    0.004393    0.009698    0.012019
     0.014053    0.018465    0.019104    0.019724    0.020463    0.022059
     0.022491    0.022798    0.024023    0.024381    0.025673    0.026607
     0.028211    0.028306    0.029611    0.030386    0.034936    0.036131
     0.038372    0.041193    0.043298    0.043812    0.044293    0.047396
     0.051923    0.053781    0.054955    0.055197    0.063940    0.077190
     0.083830    0.093337    0.121393    0.122088    0.126855    0.128054
     0.131604    0.132972    0.134738    0.139097    0.143048    0.147504
     0.147629    0.148010    0.149490    0.151112    0.151660    0.152556
     0.152940    0.154078    0.176925    0.190910    0.206090    0.216071
     0.219539    0.226640    0.237416    0.241019    0.246287    0.257757
     0.266048    0.271814    0.281316    0.295015    0.297955    0.299783
     0.300141    0.300768    0.301136    0.301815    0.302587    0.304132
     0.304825    0.304981    0.305410    0.307525    0.308127    0.311778
     0.318006    0.328996    0.329697    0.332015    0.334402    0.337027
     0.342100    0.348064    0.349742    0.350597    0.362334    0.380150
     0.393465    0.394979    0.405777    0.415999    0.418624    0.420682
     0.434691    0.441520    0.448419    0.454591    0.562725    0.778696

 Minimum search - taking simple RFO step
 Searching for Lamda that Minimizes Along All modes
 Value Taken    Lamda =  -0.02339113
 Calculated Step too Large.  Step scaled by  0.708806 !!
 Step Taken.  Stepsize is  0.300000  0.018727

                             Maximum     Tolerance    Cnvgd?
        Gradient            0.022308      0.000800      NO
        Displacement        0.094512      0.001400      NO
        Energy change      *********      0.000228      NO


 New Cartesian Coordinates Obtained by Inverse Iteration

 Displacement from previous Coordinates is:  0.500351
 -----------------------------------------------------------------
             Standard Nuclear Orientation (Angstroms)
    I     Atom           X                Y                Z
 -----------------------------------------------------------------
    1      N       2.8792676841     0.0718083331    -0.0938718517
    2      C       3.0147476173    -0.8320039223    -1.1092310357
    3      C       3.9341131574    -1.8621933383    -0.9619927735
    4      C       4.7249366823    -1.9503399484     0.1740716885
    5      C       4.5988123115    -0.9906273746     1.1714459514
    6      C       3.6766926112     0.0075328424     1.0056121928
    7      C       2.1839586279    -0.7335719590    -2.3517162361
    8      C       1.9112156175     1.2018608094    -0.1814569174
    9      N      -1.2542038404    -0.2333097200     1.2352306069
   10      C      -2.1500721260     0.0082623706     0.2512615698
   11      N      -1.8011982876     0.6529023708    -0.8404331853
```

```
  12      C      -0.5314430710     1.0695631473    -0.9667944346
  13      C       0.4676799539     0.7877652222     0.0026141337
  14      C       0.0377445050     0.1380828810     1.1229387301
  15      C      -3.5676958670    -0.4107251439     0.4524281325
  16      C      -4.3646891230     0.6695566859     1.2071977418
  17      C      -5.8178798455     0.2506774863     1.3987399729
  18      N      -0.2282594977     1.7181536125    -2.1379946699
  19      H       4.0448244885    -2.5935262041    -1.7551299469
  20      H       5.4502900681    -2.7523645407     0.2742646246
  21      H       5.2219332834    -1.0158349077     2.0574413661
  22      H       3.5586560777     0.7868132950     1.7490106875
  23      H       2.5929213854    -1.3906275164    -3.1190873019
  24      H       2.1704575934     0.2768353664    -2.7650082512
  25      H       1.1545064816    -1.0483588199    -2.1592690150
  26      H       2.0705891341     1.7000165899    -1.1362355343
  27      H       2.1854977008     1.9246548255     0.5901360891
  28      H      -1.5789761895    -0.7102655050     2.0730655397
  29      H       0.6771080512    -0.1207436025     1.9592942284
  30      H      -4.0151846251    -0.5906990114    -0.5273959915
  31      H      -3.5983995346    -1.3549619529     1.0079811127
  32      H      -4.3218111973     1.6100595762     0.6482707017
  33      H      -3.9059747288     0.8589573392     2.1852395327
  34      H      -6.3098364003     0.0881107849     0.4362670663
  35      H      -6.3676964321     1.0293324126     1.9304774175
  36      H      -5.8916247490    -0.6721384000     1.9804989229
  37      H       0.1975297843     2.6362235419    -2.0699284364
  38      H      -1.0485373021     1.7751223736    -2.7379424278
 ----------------------------------------------------------------
 Nuclear Repulsion Energy =        1298.68766276 hartrees
 There are       65 alpha and       65 beta electrons

--------------------------------------------------
-  Entering fldman on Sun Oct 13 21:20:53 2024  -
--------------------------------------------------

 Applying Cartesian multipole field
    Component          Value
    ---------          -----
     (2,0,0)        1.00000E-12
     (0,2,0)        2.00000E-11
     (0,0,2)       -3.00000E-11
 Nucleus-field energy     =    -0.0000000074 hartrees

--------------------------------------------------
-  Entering gesman on Sun Oct 13 21:20:53 2024  -
--------------------------------------------------

 Requested basis set is 6-311+G(d,p)
 There are 188 shells and 516 basis functions
 A cutoff of  1.0D-12 yielded  12529 shell pairs
 There are    100104 function pairs (    106707 Cartesian)
 Smallest overlap matrix eigenvalue = 1.48E-06
 Linear dependence detected in AO basis
 Tighter screening thresholds may be required for diffuse basis sets
 Use S2THRESH > 12 and THRESH = 14 in case of SCF convergence issues
 Number of orthogonalized atomic orbitals = 510
 Maximum deviation from orthogonality = 9.887E-12
 Guess MOs from SCF MO coefficient file
 Reading MOs from coefficient file
 Reading MOs from coefficient file

--------------------------------------------------
-  Entering scfman on Sun Oct 13 21:20:54 2024  -
--------------------------------------------------

 Long-range K will be added via erf
 Coulomb attenuation parameter = 0.2 bohr**(-1)
 A restricted hybrid HF-DFT SCF calculation will be
 performed using Pulay DIIS + Geometric Direct Minimization
 Exchange:     0.2220 Hartree-Fock + 1.0000 wB97X-D + LR-HF
```

```
 Correlation:  1.0000 wB97X-D
 Using Euler-Maclaurin-Lebedev (75,302) quadrature formula
 Dispersion:   Grimme D
 SCF converges when RMS gradient is below 1.0E-07
 Geometry optimization detected.  Setting ReadMinima to 0
 Setting SaveMinima to 0
 ---------------------------------------
  Cycle       Energy         DIIS Error
 ---------------------------------------
    1    -764.0558327185      8.43E-04
    2    -763.9583949842      1.77E-04
    3    -763.9587952465      1.69E-04
    4    -763.9595663511      2.51E-05
    5    -763.9595887358      1.25E-05
    6    -763.9595954270      4.54E-06
    7    -763.9595965692      1.75E-06
    8    -763.9595967519      5.20E-07
    9    -763.9595967757      2.62E-07
   10    -763.9595967790      8.71E-08 Convergence criterion met
 ---------------------------------------
 SCF time:  CPU 297.83 s  wall 477.59 s
 SCF   energy in the final basis set = -763.95959678
 Total energy in the final basis set = -763.95959678

-------------------------------------------------
-  Entering anlman on Sun Oct 13 21:28:51 2024  -
-------------------------------------------------


 --------------------------------------------------------------

                   Orbital Energies (a.u.)
 --------------------------------------------------------------

 Alpha MOs
 -- Occupied --
-14.8165 -14.8029 -14.7214 -14.7169 -10.6758 -10.6682 -10.6553 -10.6382
-10.6348 -10.6255 -10.6100 -10.5821 -10.5706 -10.5698 -10.5456 -10.5295
-10.4983 -10.4524  -1.4245  -1.4024  -1.3431  -1.2741  -1.2304  -1.2016
 -1.1855  -1.1442  -1.1023  -1.0809  -1.0589  -1.0510  -1.0314  -1.0028
 -0.9884  -0.9511  -0.9441  -0.9059  -0.9018  -0.8909  -0.8791  -0.8657
 -0.8488  -0.8444  -0.8344  -0.8205  -0.8083  -0.7950  -0.7854  -0.7798
 -0.7708  -0.7673  -0.7510  -0.7465  -0.7344  -0.7277  -0.7183  -0.7113
 -0.6834  -0.6713  -0.6567  -0.6471  -0.6354  -0.6228  -0.6197  -0.6189
 -0.6142
 -- Virtual --
 -0.2725  -0.2690  -0.2513  -0.2254  -0.1765  -0.1634  -0.1519  -0.1459
 -0.1307  -0.1247  -0.1181  -0.1153  -0.1110  -0.1032  -0.0994  -0.0921
 -0.0876  -0.0858  -0.0820  -0.0805  -0.0761  -0.0703  -0.0676  -0.0644
 -0.0612  -0.0584  -0.0568  -0.0517  -0.0483  -0.0432  -0.0364  -0.0332
 -0.0304  -0.0248  -0.0237  -0.0231  -0.0181  -0.0151  -0.0124  -0.0071
 -0.0043   0.0007   0.0016   0.0074   0.0104   0.0129   0.0138   0.0178
  0.0202   0.0211   0.0271   0.0294   0.0310   0.0345   0.0378   0.0387
  0.0393   0.0438   0.0534   0.0549   0.0558   0.0587   0.0614   0.0629
  0.0717   0.0725   0.0758   0.0783   0.0823   0.0858   0.0904   0.0917
  0.0928   0.0975   0.1016   0.1081   0.1117   0.1146   0.1159   0.1183
  0.1216   0.1264   0.1278   0.1313   0.1382   0.1404   0.1407   0.1496
  0.1536   0.1565   0.1620   0.1652   0.1673   0.1700   0.1726   0.1755
  0.1806   0.1823   0.1884   0.1935   0.1977   0.2034   0.2134   0.2142
  0.2219   0.2274   0.2550   0.2676   0.2724   0.2878   0.2958   0.3136
  0.3251   0.3273   0.3374   0.3457   0.3489   0.3534   0.3598   0.3700
  0.3739   0.3789   0.3870   0.3925   0.3982   0.4067   0.4147   0.4187
  0.4243   0.4359   0.4435   0.4512   0.4607   0.4689   0.4723   0.4768
  0.4820   0.4919   0.4969   0.5010   0.5054   0.5103   0.5173   0.5238
  0.5297   0.5314   0.5374   0.5400   0.5503   0.5524   0.5549   0.5580
  0.5630   0.5719   0.5749   0.5828   0.5868   0.5894   0.5926   0.5973
  0.6098   0.6158   0.6257   0.6318   0.6359   0.6417   0.6556   0.6590
  0.6658   0.6700   0.6811   0.6833   0.6922   0.6931   0.6975   0.7009
  0.7146   0.7257   0.7333   0.7395   0.7635   0.7709   0.7776   0.7865
  0.7914   0.7930   0.8035   0.8146   0.8278   0.8369   0.8456   0.8496
```

| | | | | | | | |
|---|---|---|---|---|---|---|---|
| 0.8645 | 0.8705 | 0.8754 | 0.8783 | 0.8935 | 0.9046 | 0.9149 | 0.9196 |
| 0.9316 | 0.9407 | 0.9569 | 0.9725 | 0.9831 | 0.9929 | 1.0112 | 1.0243 |
| 1.0479 | 1.0710 | 1.1033 | 1.1114 | 1.1232 | 1.1485 | 1.1610 | 1.1746 |
| 1.2046 | 1.2203 | 1.2360 | 1.2551 | 1.2610 | 1.2825 | 1.3070 | 1.3094 |
| 1.3202 | 1.3282 | 1.3328 | 1.3433 | 1.3502 | 1.3633 | 1.3650 | 1.3731 |
| 1.3839 | 1.3900 | 1.3980 | 1.4095 | 1.4114 | 1.4197 | 1.4301 | 1.4375 |
| 1.4434 | 1.4444 | 1.4587 | 1.4656 | 1.4703 | 1.4814 | 1.4892 | 1.4977 |
| 1.5016 | 1.5067 | 1.5156 | 1.5221 | 1.5338 | 1.5431 | 1.5471 | 1.5524 |
| 1.5615 | 1.5648 | 1.5721 | 1.5850 | 1.5902 | 1.5949 | 1.6130 | 1.6158 |
| 1.6256 | 1.6293 | 1.6418 | 1.6476 | 1.6618 | 1.6730 | 1.6857 | 1.6979 |
| 1.7017 | 1.7216 | 1.7293 | 1.7408 | 1.7441 | 1.7556 | 1.7609 | 1.7658 |
| 1.7799 | 1.7884 | 1.8071 | 1.8330 | 1.8450 | 1.8602 | 1.8707 | 1.8817 |
| 1.8929 | 1.8975 | 1.9063 | 1.9277 | 1.9567 | 1.9576 | 1.9637 | 1.9816 |
| 1.9992 | 2.0118 | 2.0249 | 2.0400 | 2.0441 | 2.0508 | 2.0675 | 2.0806 |
| 2.0874 | 2.1048 | 2.1270 | 2.1339 | 2.1582 | 2.1906 | 2.1972 | 2.2177 |
| 2.2306 | 2.2447 | 2.2506 | 2.2530 | 2.2719 | 2.2923 | 2.2962 | 2.3087 |
| 2.3156 | 2.3312 | 2.3487 | 2.3633 | 2.3718 | 2.3834 | 2.3869 | 2.4033 |
| 2.4130 | 2.4260 | 2.4376 | 2.4528 | 2.4609 | 2.4677 | 2.4761 | 2.4837 |
| 2.4970 | 2.5042 | 2.5184 | 2.5229 | 2.5356 | 2.5394 | 2.5427 | 2.5551 |
| 2.5647 | 2.5784 | 2.5850 | 2.5893 | 2.5928 | 2.5994 | 2.6150 | 2.6206 |
| 2.6355 | 2.6421 | 2.6549 | 2.6616 | 2.6714 | 2.6792 | 2.6895 | 2.7031 |
| 2.7086 | 2.7156 | 2.7185 | 2.7276 | 2.7357 | 2.7561 | 2.7656 | 2.7697 |
| 2.7868 | 2.7907 | 2.8135 | 2.8406 | 2.8450 | 2.8495 | 2.8585 | 2.8875 |
| 2.8930 | 2.9240 | 2.9307 | 2.9528 | 3.0257 | 3.0728 | 3.1191 | 3.1317 |
| 3.1457 | 3.1860 | 3.2407 | 3.2570 | 3.2911 | 3.3165 | 3.3544 | 3.3787 |
| 3.4156 | 3.4723 | 3.5097 | 3.5322 | 3.5508 | 3.5888 | 3.6153 | 3.6530 |
| 3.6814 | 3.7077 | 3.7141 | 3.7327 | 3.7357 | 3.7492 | 3.7594 | 3.7671 |
| 3.8208 | 3.8666 | 3.8904 | 3.9037 | 3.9413 | 4.0073 | 4.0486 | 4.0924 |
| 4.1003 | 4.1856 | 4.3002 | 4.3169 | 4.5754 | 4.6509 | 4.6900 | 4.7149 |
| 4.8890 | 5.0323 | 5.1704 | 23.5205 | 23.5649 | 23.7171 | 23.7265 | 23.7386 |
| 23.7760 | 23.8036 | 23.8306 | 23.8369 | 23.8925 | 23.9207 | 23.9633 | 23.9703 |
| 24.0373 | 35.4185 | 35.4483 | 35.4971 | 35.5914 | | | |

Ground-State Mulliken Net Atomic Charges

| Atom | | Charge (a.u.) |
|---|---|---|
| 1 | N | 0.439179 |
| 2 | C | 0.079940 |
| 3 | C | -0.036748 |
| 4 | C | -0.223269 |
| 5 | C | -0.285594 |
| 6 | C | -0.071252 |
| 7 | C | -0.721946 |
| 8 | C | -0.211709 |
| 9 | N | -0.109621 |
| 10 | C | -0.365301 |
| 11 | N | 0.092446 |
| 12 | C | 0.081097 |
| 13 | C | 0.006761 |
| 14 | C | 0.161655 |
| 15 | C | -0.377733 |
| 16 | C | -0.088422 |
| 17 | C | -0.604482 |
| 18 | N | -0.462312 |
| 19 | H | 0.222075 |
| 20 | H | 0.235159 |
| 21 | H | 0.231205 |
| 22 | H | 0.217310 |
| 23 | H | 0.231863 |
| 24 | H | 0.217992 |
| 25 | H | 0.236426 |
| 26 | H | 0.212151 |
| 27 | H | 0.248672 |
| 28 | H | 0.397603 |
| 29 | H | 0.215868 |
| 30 | H | 0.242211 |
| 31 | H | 0.209814 |
| 32 | H | 0.199748 |

```
    33 H                    0.169672
    34 H                    0.178380
    35 H                    0.199099
    36 H                    0.168773
    37 H                    0.318029
    38 H                    0.345260
  ----------------------------------------
  Sum of atomic charges =     2.000000

 -----------------------------------------------------------------
                    Cartesian Multipole Moments
 -----------------------------------------------------------------
    Charge (ESU x 10^10)
                 9.6064
    Dipole Moment (Debye)
         X       8.7352      Y      -0.2746      Z       1.4694
       Tot       8.8622
    Quadrupole Moments (Debye-Ang)
        XX      -0.3051     XY     -16.2231     YY     -78.1644
        XZ      -6.1669     YZ      -5.5310     ZZ     -71.8744
    Traceless Quadrupole Moments (Debye-Ang)
       QXX     149.4286    QYY     -84.1493    QZZ     -65.2793
       QXY     -48.6692    QXZ     -18.5006    QYZ     -16.5930
    Octopole Moments (Debye-Ang^2)
       XXX      45.7936    XXY     -94.9893    XYY      24.4970
       YYY       9.5123    XXZ      71.0736    XYZ      15.2220
       YYZ     -24.7984    XZZ      -7.0914    YZZ       4.6273
       ZZZ      -1.8461
    Traceless Octopole Moments (Debye-Ang^2)
       XXX     118.1116    YYY     870.3321    ZZZ    -427.5530
       XXY   -1182.2912    XXZ     932.8167    XYY     177.8571
       XYZ     228.3306    XZZ    -295.9687    YYZ    -505.2637
       YZZ     311.9591
    Hexadecapole Moments (Debye-Ang^3)
      XXXX   -4209.1431   XXXY    -112.3602   XXYY    -887.8151
      XYYY     346.8116   YYYY    -619.2319   XXXZ     372.0875
      XXYZ      39.0302   XYYZ      85.0757   YYYZ     -11.2395
      XXZZ    -994.3699   XYZZ     104.0717   YYZZ    -212.0422
      XZZZ     333.6776   YZZZ      -6.3773   ZZZZ    -868.5662
    Traceless Hexadecapole Moments (Debye-Ang^3)
      XXXX   17290.9448   XXXY  -27031.3581   XXXZ    3481.3491
      XXYY   -5720.5139   XXYZ    3771.3084   XXZZ  -11570.4309
      XYYY   21181.6793   XYYZ   -2929.6593   XYZZ    5849.6788
      XZZZ    -551.6898   YYYY     730.1172   YYYZ   -2160.7260
      YYZZ    4990.3968   YZZZ   -1610.5824   ZZZZ    6580.0342
 -----------------------------------------------------------------

-------------------------------------------------
-  Entering drvman on Sun Oct 13 21:28:51 2024  -
-------------------------------------------------

 Calculating analytic gradient of the SCF energy
 Gradient of SCF Energy
           1           2           3           4           5           6
    1  -0.0018518   0.0024764  -0.0035302  -0.0010292  -0.0015330   0.0012367
    2  -0.0015814   0.0028527  -0.0003409   0.0028857   0.0007962  -0.0011106
    3   0.0007782   0.0033338  -0.0039646   0.0027766  -0.0006650  -0.0031302
           7           8           9          10          11          12
    1  -0.0005498  -0.0035652   0.0000483   0.0127898  -0.0086539   0.0022951
    2  -0.0018358   0.0002295   0.0010078   0.0011769   0.0041427  -0.0084116
    3  -0.0012650  -0.0020486  -0.0036908   0.0083754  -0.0055534   0.0181470
          13          14          15          16          17          18
    1  -0.0030694  -0.0023611  -0.0033380   0.0019278   0.0005960   0.0153120
    2   0.0007349  -0.0003012   0.0035611  -0.0039682   0.0008678  -0.0015523
    3  -0.0022251  -0.0020965  -0.0000936  -0.0021586   0.0001123  -0.0106404
          19          20          21          22          23          24
    1   0.0013304   0.0011865   0.0012972   0.0017398   0.0001521   0.0013200
    2  -0.0020865  -0.0008098  -0.0013723  -0.0003500   0.0003204   0.0001684
    3  -0.0001645   0.0002156   0.0003108   0.0015978  -0.0016302  -0.0006058
          25          26          27          28          29          30
```

```
    1   0.0005051   0.0017543  -0.0013727  -0.0017978  -0.0010051  -0.0013730
    2  -0.0011815   0.0013607   0.0016884  -0.0013839   0.0000670  -0.0011792
    3   0.0000977   0.0003934   0.0007129   0.0017267   0.0009642  -0.0001811
           31          32          33          34          35          36
    1   0.0002508  -0.0008936  -0.0004805   0.0000504  -0.0002178   0.0003321
    2  -0.0005440   0.0009414   0.0003101  -0.0001555   0.0004822  -0.0003557
    3  -0.0003368   0.0003586   0.0004091  -0.0004838   0.0003317   0.0001101
           37          38
    1  -0.0080538  -0.0019252
    2   0.0043550   0.0005714
    3   0.0001268   0.0000551
 Max gradient component =       1.815E-02
 RMS gradient           =       3.575E-03
 Gradient time:  CPU 126.81 s  wall 201.06 s

-------------------------------------------------
-  Entering optman on Sun Oct 13 21:32:12 2024  -
-------------------------------------------------

 Geometry Optimization Parameters
   NAtoms,    NIC,     NZ,  NCons,   NDum,   NFix, NCnnct, MaxDiis
       38     272       0       0       0       0       0       0

 Cartesian Hessian Update
 Hessian updated using BFGS update


** GEOMETRY OPTIMIZATION IN DELOCALIZED INTERNAL COORDINATES **
   Searching for a Minimum

   Optimization Cycle:   2

                    Coordinates (Angstroms)
     ATOM                X               Y               Z
      1  N         2.8792676841    0.0718083331   -0.0938718517
      2  C         3.0147476173   -0.8320039223   -1.1092310357
      3  C         3.9341131574   -1.8621933383   -0.9619927735
      4  C         4.7249366823   -1.9503399484    0.1740716885
      5  C         4.5988123115   -0.9906273746    1.1714459514
      6  C         3.6766926112    0.0075328424    1.0056121928
      7  C         2.1839586279   -0.7335719590   -2.3517162361
      8  C         1.9112156175    1.2018608094   -0.1814569174
      9  N        -1.2542038404   -0.2333097200    1.2352306069
     10  C        -2.1500721260    0.0082623706    0.2512615698
     11  N        -1.8011982876    0.6529023708   -0.8404331853
     12  C        -0.5314430710    1.0695631473   -0.9667944346
     13  C         0.4676799539    0.7877652222    0.0026141337
     14  C         0.0377445050    0.1380828810    1.1229387301
     15  C        -3.5676958670   -0.4107251439    0.4524281325
     16  C        -4.3646891230    0.6695566859    1.2071977418
     17  C        -5.8178798455    0.2506774863    1.3987399729
     18  N        -0.2282594977    1.7181536125   -2.1379946699
     19  H         4.0448244885   -2.5935262041   -1.7551299469
     20  H         5.4502900681   -2.7523645407    0.2742646246
     21  H         5.2219332834   -1.0158349077    2.0574413661
     22  H         3.5586560777    0.7868132950    1.7490106875
     23  H         2.5929213854   -1.3906275164   -3.1190873019
     24  H         2.1704575934    0.2768353664   -2.7650082512
     25  H         1.1545064816   -1.0483588199   -2.1592690150
     26  H         2.0705891341    1.7000165899   -1.1362355343
     27  H         2.1854977008    1.9246548255    0.5901360891
     28  H        -1.5789761895   -0.7102655050    2.0730655397
     29  H         0.6771080512   -0.1207436025    1.9592942284
     30  H        -4.0151846251   -0.5906990114   -0.5273959915
     31  H        -3.5983995346   -1.3549619529    1.0079811127
     32  H        -4.3218111973    1.6100595762    0.6482707017
     33  H        -3.9059747288    0.8589573392    2.1852395327
     34  H        -6.3098364003    0.0881107849    0.4362670663
     35  H        -6.3676964321    1.0293324126    1.9304774175
     36  H        -5.8916247490   -0.6721384000    1.9804989229
```

```
   37  H          0.1975297843    2.6362235419   -2.0699284364
   38  H         -1.0485373021    1.7751223736   -2.7379424278
 Point Group: c1    Number of degrees of freedom:   108


 Energy is   -763.959596779

Hessian updated using BFGS update
 internal optimization (0)

108 Hessian modes will be used to form the next step
 Hessian Eigenvalues:
    0.002199    0.002740    0.003725    0.004396    0.009715    0.011402
    0.014042    0.018491    0.019118    0.019732    0.020459    0.022088
    0.022528    0.022799    0.024016    0.024351    0.025673    0.026608
    0.028208    0.028308    0.029580    0.030379    0.034446    0.036081
    0.038374    0.041146    0.043298    0.043811    0.044297    0.047390
    0.051713    0.053474    0.054053    0.055102    0.063946    0.077059
    0.083835    0.093328    0.121422    0.122061    0.126854    0.127881
    0.130671    0.132969    0.133956    0.138300    0.143061    0.145820
    0.147509    0.148046    0.149349    0.150501    0.151665    0.152349
    0.152613    0.156567    0.180581    0.190886    0.205973    0.216132
    0.221660    0.226219    0.237252    0.239426    0.247523    0.261341
    0.269221    0.271778    0.280492    0.294632    0.298202    0.299462
    0.300148    0.300858    0.301147    0.301841    0.302517    0.304136
    0.304782    0.304985    0.305422    0.307525    0.308118    0.312093
    0.321622    0.328734    0.329124    0.332155    0.334399    0.337035
    0.342122    0.348139    0.349917    0.360966    0.379828    0.389561
    0.392775    0.405073    0.410296    0.414794    0.420211    0.425767
    0.434444    0.441538    0.452712    0.483716    0.572708    0.775926

Minimum search - taking simple RFO step
Searching for Lamda that Minimizes Along All modes
Value Taken    Lamda =  -0.00849276
Calculated Step too Large.  Step scaled by  0.547740 !!
Step Taken.  Stepsize is  0.300000  0.014553

                         Maximum     Tolerance    Cnvgd?
       Gradient          0.010159     0.000800      NO
       Displacement      0.092939     0.001400      NO
       Energy change    -0.010931     0.000228      NO


New Cartesian Coordinates Obtained by Inverse Iteration

Displacement from previous Coordinates is:  0.457757
-------------------------------------------------------------
          Standard Nuclear Orientation (Angstroms)
   I     Atom           X                Y                Z
-------------------------------------------------------------
   1      N       2.8794506218     0.0731062954    -0.0934308801
   2      C       3.0040762917    -0.8417413130    -1.0978324755
   3      C       3.9321439066    -1.8632624622    -0.9447833496
   4      C       4.7297959070    -1.9426112950     0.1850612216
   5      C       4.6000646976    -0.9756091016     1.1782587252
   6      C       3.6737744771     0.0191475706     1.0082739583
   7      C       2.1524634934    -0.7505754005    -2.3233924872
   8      C       1.9162553890     1.2003916468    -0.1914252249
   9      N      -1.2454027716    -0.2525571744     1.2115872168
  10      C      -2.1524813496     0.0008946625     0.2343582869
  11      N      -1.7997706979     0.6682482633    -0.8402405916
  12      C      -0.5395735175     1.1050133371    -0.9745659837
  13      C       0.4691349629     0.7905518360    -0.0147928043
  14      C       0.0478756011     0.1207579561     1.0973076228
  15      C      -3.5638024043    -0.4199589901     0.4419604351
  16      C      -4.3561757196     0.6616310804     1.2106285546
  17      C      -5.8111246243     0.2459849481     1.4022523022
  18      N      -0.2580148646     1.7654784148    -2.1247536383
  19      H       4.0334006023    -2.5943799183    -1.7369348240
  20      H       5.4558734563    -2.7409877741     0.2871940496
```

```
   21      H        5.2192389976    -0.9860924652     2.0657609662
   22      H        3.5460598043     0.8058513477     1.7403344355
   23      H        2.5410137382    -1.4266948164    -3.0824959456
   24      H        2.1345432879     0.2531108075    -2.7515748392
   25      H        1.1218192069    -1.0478750860    -2.1095730077
   26      H        2.0687931114     1.6872114334    -1.1520120569
   27      H        2.1964246253     1.9241529535     0.5743633980
   28      H       -1.5591675818    -0.7422893933     2.0429977599
   29      H        0.6984129181    -0.1567388985     1.9187574347
   30      H       -4.0125388919    -0.5832857961    -0.5386901238
   31      H       -3.5972361844    -1.3673976323     0.9905932565
   32      H       -4.3059756107     1.6028460030     0.6550820856
   33      H       -3.8902605429     0.8402879572     2.1868970404
   34      H       -6.3064798832     0.0926131723     0.4406682571
   35      H       -6.3564042686     1.0227849202     1.9397008566
   36      H       -5.8891808202    -0.6796289711     1.9780395052
   37      H        0.2996393493     2.6083411880    -2.1017832995
   38      H       -1.0766647130     1.8832806944    -2.7117958366
 ----------------------------------------------------------------
 Nuclear Repulsion Energy =        1299.73979911 hartrees
 There are       65 alpha and       65 beta electrons

--------------------------------------------------
-  Entering fldman on Sun Oct 13 21:32:12 2024  -
--------------------------------------------------

 Applying Cartesian multipole field
    Component          Value
    ---------          -----
     (2,0,0)        1.00000E-12
     (0,2,0)        2.00000E-11
     (0,0,2)       -3.00000E-11
 Nucleus-field energy     =    -0.0000000069 hartrees

--------------------------------------------------
-  Entering gesman on Sun Oct 13 21:32:12 2024  -
--------------------------------------------------

 Requested basis set is 6-311+G(d,p)
 There are 188 shells and 516 basis functions
 A cutoff of  1.0D-12 yielded  12548 shell pairs
 There are    100188 function pairs (    106793 Cartesian)
 Smallest overlap matrix eigenvalue = 1.49E-06
 Linear dependence detected in AO basis
 Tighter screening thresholds may be required for diffuse basis sets
 Use S2THRESH > 12 and THRESH = 14 in case of SCF convergence issues
 Number of orthogonalized atomic orbitals = 510
 Maximum deviation from orthogonality = 3.497E-11
 Guess MOs from SCF MO coefficient file
 Reading MOs from coefficient file
 Reading MOs from coefficient file

--------------------------------------------------
-  Entering scfman on Sun Oct 13 21:32:13 2024  -
--------------------------------------------------

 Long-range K will be added via erf
 Coulomb attenuation parameter = 0.2 bohr**(-1)
 A restricted hybrid HF-DFT SCF calculation will be
 performed using Pulay DIIS + Geometric Direct Minimization
 Exchange:     0.2220 Hartree-Fock + 1.0000 wB97X-D + LR-HF
 Correlation:  1.0000 wB97X-D
 Using Euler-Maclaurin-Lebedev (75,302) quadrature formula
 Dispersion:   Grimme D
 SCF converges when RMS gradient is below 1.0E-07
 Geometry optimization detected.  Setting ReadMinima to 0
 Setting SaveMinima to 0
 ---------------------------------------
  Cycle       Energy         DIIS Error
 ---------------------------------------
```

```
    1    -763.9851370542      4.84E-04
    2    -763.9627662321      1.16E-04
    3    -763.9629605083      1.13E-04
    4    -763.9633380312      1.67E-05
    5    -763.9633506552      8.57E-06
    6    -763.9633534905      3.27E-06
    7    -763.9633540149      1.06E-06
    8    -763.9633540668      4.31E-07
    9    -763.9633540774      1.33E-07
   10    -763.9633540784      6.13E-08 Convergence criterion met
 ---------------------------------------
 SCF time:  CPU 278.39 s  wall 434.96 s
 SCF   energy in the final basis set = -763.96335408
 Total energy in the final basis set = -763.96335408

--------------------------------------------------
-  Entering anlman on Sun Oct 13 21:39:28 2024  -
--------------------------------------------------



 --------------------------------------------------------------

                    Orbital Energies (a.u.)
 --------------------------------------------------------------

 Alpha MOs
 -- Occupied --
-14.8166 -14.7983 -14.7268 -14.7131 -10.6761 -10.6667 -10.6526 -10.6388
-10.6347 -10.6259 -10.6083 -10.5831 -10.5717 -10.5705 -10.5475 -10.5283
-10.4979 -10.4516  -1.4207  -1.4041  -1.3448  -1.2749  -1.2305  -1.2010
 -1.1872  -1.1435  -1.1015  -1.0827  -1.0568  -1.0516  -1.0327  -1.0020
 -0.9894  -0.9512  -0.9433  -0.9071  -0.9039  -0.8934  -0.8804  -0.8655
 -0.8512  -0.8449  -0.8334  -0.8217  -0.8092  -0.7962  -0.7860  -0.7811
 -0.7703  -0.7660  -0.7521  -0.7488  -0.7396  -0.7286  -0.7178  -0.7134
 -0.6831  -0.6699  -0.6572  -0.6445  -0.6342  -0.6216  -0.6201  -0.6177
 -0.6128
 -- Virtual --
 -0.2695  -0.2682  -0.2495  -0.2262  -0.1754  -0.1637  -0.1510  -0.1454
 -0.1318  -0.1239  -0.1179  -0.1152  -0.1108  -0.1032  -0.0993  -0.0930
 -0.0880  -0.0855  -0.0815  -0.0802  -0.0760  -0.0696  -0.0674  -0.0641
 -0.0616  -0.0581  -0.0569  -0.0520  -0.0483  -0.0424  -0.0366  -0.0324
 -0.0302  -0.0252  -0.0235  -0.0230  -0.0174  -0.0155  -0.0116  -0.0071
 -0.0053   0.0009   0.0024   0.0079   0.0098   0.0130   0.0137   0.0183
  0.0203   0.0213   0.0269   0.0292   0.0315   0.0355   0.0372   0.0393
  0.0400   0.0440   0.0538   0.0547   0.0569   0.0599   0.0624   0.0635
  0.0723   0.0734   0.0762   0.0780   0.0815   0.0858   0.0896   0.0913
  0.0935   0.0972   0.1013   0.1087   0.1128   0.1131   0.1159   0.1184
  0.1216   0.1277   0.1288   0.1312   0.1388   0.1401   0.1409   0.1502
  0.1541   0.1569   0.1619   0.1659   0.1685   0.1714   0.1726   0.1763
  0.1816   0.1868   0.1887   0.1950   0.1976   0.2047   0.2135   0.2141
  0.2207   0.2279   0.2556   0.2672   0.2719   0.2854   0.2959   0.3126
  0.3254   0.3277   0.3367   0.3466   0.3483   0.3537   0.3618   0.3701
  0.3755   0.3794   0.3877   0.3924   0.3998   0.4068   0.4167   0.4195
  0.4253   0.4364   0.4435   0.4518   0.4615   0.4676   0.4719   0.4805
  0.4813   0.4923   0.4979   0.5016   0.5063   0.5118   0.5212   0.5256
  0.5292   0.5312   0.5373   0.5382   0.5488   0.5521   0.5538   0.5591
  0.5632   0.5714   0.5766   0.5825   0.5878   0.5903   0.5928   0.5983
  0.6088   0.6156   0.6265   0.6313   0.6353   0.6419   0.6559   0.6600
  0.6639   0.6692   0.6803   0.6844   0.6899   0.6957   0.6982   0.7006
  0.7151   0.7246   0.7344   0.7390   0.7630   0.7697   0.7790   0.7867
  0.7895   0.7941   0.8072   0.8166   0.8315   0.8370   0.8475   0.8509
  0.8652   0.8709   0.8760   0.8790   0.8944   0.9071   0.9161   0.9211
  0.9312   0.9415   0.9591   0.9751   0.9807   0.9927   1.0151   1.0252
  1.0503   1.0745   1.1017   1.1126   1.1267   1.1507   1.1617   1.1713
  1.2008   1.2232   1.2415   1.2524   1.2582   1.2800   1.3065   1.3115
  1.3197   1.3273   1.3363   1.3419   1.3492   1.3634   1.3661   1.3732
  1.3857   1.3938   1.3980   1.4087   1.4125   1.4153   1.4282   1.4341
  1.4446   1.4450   1.4611   1.4677   1.4701   1.4825   1.4904   1.4977
  1.5030   1.5082   1.5167   1.5198   1.5348   1.5440   1.5479   1.5536
  1.5603   1.5630   1.5741   1.5833   1.5941   1.5978   1.6139   1.6167
```

| | | | | | | | |
|---|---|---|---|---|---|---|---|
| 1.6251 | 1.6284 | 1.6443 | 1.6476 | 1.6612 | 1.6713 | 1.6931 | 1.6978 |
| 1.7023 | 1.7295 | 1.7302 | 1.7427 | 1.7435 | 1.7549 | 1.7617 | 1.7661 |
| 1.7806 | 1.7876 | 1.8033 | 1.8330 | 1.8427 | 1.8654 | 1.8733 | 1.8866 |
| 1.8963 | 1.9026 | 1.9123 | 1.9295 | 1.9450 | 1.9585 | 1.9624 | 1.9798 |
| 1.9961 | 2.0102 | 2.0253 | 2.0408 | 2.0434 | 2.0516 | 2.0624 | 2.0827 |
| 2.0898 | 2.1019 | 2.1296 | 2.1387 | 2.1586 | 2.1922 | 2.2010 | 2.2191 |
| 2.2310 | 2.2486 | 2.2511 | 2.2543 | 2.2676 | 2.2897 | 2.2940 | 2.3062 |
| 2.3163 | 2.3320 | 2.3484 | 2.3629 | 2.3734 | 2.3889 | 2.3911 | 2.4038 |
| 2.4106 | 2.4293 | 2.4358 | 2.4517 | 2.4594 | 2.4620 | 2.4721 | 2.4824 |
| 2.4975 | 2.5058 | 2.5207 | 2.5227 | 2.5386 | 2.5406 | 2.5456 | 2.5543 |
| 2.5642 | 2.5793 | 2.5855 | 2.5899 | 2.5947 | 2.5989 | 2.6150 | 2.6209 |
| 2.6384 | 2.6467 | 2.6557 | 2.6620 | 2.6652 | 2.6769 | 2.6876 | 2.7060 |
| 2.7099 | 2.7172 | 2.7212 | 2.7310 | 2.7365 | 2.7587 | 2.7701 | 2.7727 |
| 2.7866 | 2.7926 | 2.8118 | 2.8417 | 2.8461 | 2.8504 | 2.8619 | 2.8861 |
| 2.8949 | 2.9231 | 2.9369 | 2.9483 | 3.0309 | 3.0714 | 3.1322 | 3.1467 |
| 3.1492 | 3.1921 | 3.2439 | 3.2552 | 3.2953 | 3.3175 | 3.3589 | 3.3868 |
| 3.4179 | 3.4744 | 3.5135 | 3.5344 | 3.5545 | 3.5925 | 3.6212 | 3.6467 |
| 3.6829 | 3.7134 | 3.7204 | 3.7347 | 3.7372 | 3.7519 | 3.7626 | 3.7696 |
| 3.8253 | 3.8672 | 3.8698 | 3.9053 | 3.9370 | 3.9962 | 4.0481 | 4.0922 |
| 4.0938 | 4.1827 | 4.2965 | 4.3080 | 4.5859 | 4.6568 | 4.6962 | 4.7242 |
| 4.9002 | 5.0353 | 5.1836 | 23.5220 | 23.5724 | 23.7169 | 23.7296 | 23.7399 |
| 23.7745 | 23.8013 | 23.8332 | 23.8379 | 23.8928 | 23.9227 | 23.9636 | 23.9693 |
| 24.0354 | 35.4198 | 35.4518 | 35.5035 | 35.6004 | | | |

Ground-State Mulliken Net Atomic Charges

| Atom | Charge (a.u.) |
|---|---|
| 1 N | 0.441452 |
| 2 C | 0.078099 |
| 3 C | -0.035856 |
| 4 C | -0.229850 |
| 5 C | -0.286047 |
| 6 C | -0.066465 |
| 7 C | -0.719992 |
| 8 C | -0.204716 |
| 9 N | -0.118867 |
| 10 C | -0.361539 |
| 11 N | 0.081618 |
| 12 C | 0.110969 |
| 13 C | -0.011208 |
| 14 C | 0.164264 |
| 15 C | -0.378330 |
| 16 C | -0.085059 |
| 17 C | -0.606059 |
| 18 N | -0.458974 |
| 19 H | 0.220961 |
| 20 H | 0.235572 |
| 21 H | 0.231509 |
| 22 H | 0.217021 |
| 23 H | 0.232605 |
| 24 H | 0.216162 |
| 25 H | 0.240408 |
| 26 H | 0.199424 |
| 27 H | 0.248170 |
| 28 H | 0.395564 |
| 29 H | 0.214641 |
| 30 H | 0.241607 |
| 31 H | 0.209718 |
| 32 H | 0.199353 |
| 33 H | 0.170267 |
| 34 H | 0.178249 |
| 35 H | 0.198918 |
| 36 H | 0.169263 |
| 37 H | 0.314961 |
| 38 H | 0.352189 |

Sum of atomic charges = 2.000000

```
 --------------------------------------------------------------------
                 Cartesian Multipole Moments
 --------------------------------------------------------------------
    Charge (ESU x 10^10)
                 9.6064
    Dipole Moment (Debye)
         X       8.9437      Y      -0.1967      Z       1.1514
       Tot       9.0196
    Quadrupole Moments (Debye-Ang)
        XX      -0.3682     XY     -15.8857     YY     -77.8140
        XZ      -6.0051     YZ      -6.3382     ZZ     -71.8201
    Traceless Quadrupole Moments (Debye-Ang)
       QXX     148.8976    QYY     -83.4396    QZZ     -65.4580
       QXY     -47.6572    QXZ     -18.0154    QYZ     -19.0145
    Octopole Moments (Debye-Ang^2)
       XXX      47.0980    XXY     -94.5252    XYY      24.8930
       YYY       8.9461    XXZ      71.8835    XYZ      14.8035
       YYZ     -26.3396    XZZ      -6.4682    YZZ       6.6602
       ZZZ      -3.6649
    Traceless Octopole Moments (Debye-Ang^2)
       XXX     116.7650    YYY     844.4614    ZZZ    -431.8845
       XXY   -1181.1212    XXZ     952.6158    XYY     176.8269
       XYZ     222.0525    XZZ    -293.5919    YYZ    -520.7313
       YZZ     336.6598
    Hexadecapole Moments (Debye-Ang^3)
      XXXX   -4196.1040   XXXY    -111.2603   XXYY    -886.8066
      XYYY     345.7922   YYYY    -629.3578   XXXZ     370.7388
      XXYZ      37.3293   XYYZ      81.8721   YYYZ     -10.5335
      XXZZ    -992.0054   XYZZ     104.7597   YYZZ    -205.3580
      XZZZ     327.2373   YZZZ      -9.0900   ZZZZ    -858.1979
    Traceless Hexadecapole Moments (Debye-Ang^3)
      XXXX   17483.5185   XXXY  -26950.4538   XXXZ    3834.4043
      XXYY   -5724.1129   XXYZ    3652.6380   XXZZ  -11759.4055
      XYYY   21040.0572   XYYZ   -3101.1523   XYZZ    5910.3966
      XZZZ    -733.2520   YYYY     186.4447   YYYZ   -1906.8283
      YYZZ    5537.6683   YZZZ   -1745.8096   ZZZZ    6221.7372
 --------------------------------------------------------------------

-------------------------------------------------
-  Entering drvman on Sun Oct 13 21:39:28 2024  -
-------------------------------------------------

 Calculating analytic gradient of the SCF energy
 Gradient of SCF Energy
            1           2           3           4           5           6
    1  -0.0008168   0.0011567  -0.0013322   0.0001740  -0.0006704   0.0003951
    2  -0.0011686   0.0010140  -0.0001124  -0.0000908   0.0005061   0.0002278
    3   0.0003209   0.0004817  -0.0008807   0.0005103  -0.0000404  -0.0009474
            7           8           9          10          11          12
    1   0.0002285  -0.0003769   0.0002993   0.0031659  -0.0010825  -0.0017007
    2  -0.0003425   0.0001623   0.0009879  -0.0016337   0.0029772  -0.0005640
    3  -0.0006578  -0.0010300  -0.0026288   0.0047528  -0.0023426   0.0088611
           13          14          15          16          17          18
    1  -0.0031881   0.0000078  -0.0011325   0.0009000  -0.0001187   0.0113534
    2  -0.0005127  -0.0010344   0.0016761  -0.0015925   0.0004184  -0.0039083
    3  -0.0037312   0.0017549  -0.0001727  -0.0008310   0.0002004  -0.0072128
           19          20          21          22          23          24
    1   0.0003622   0.0001502   0.0003456   0.0008000   0.0002499  -0.0000650
    2  -0.0004616  -0.0000092  -0.0004397  -0.0004363   0.0002887   0.0000094
    3   0.0001414   0.0000727  -0.0001249   0.0004539  -0.0000131  -0.0001690
           25          26          27          28          29          30
    1   0.0001452  -0.0004949  -0.0005418  -0.0008042  -0.0004494  -0.0001506
    2  -0.0003516   0.0006063   0.0000547  -0.0003320   0.0001081  -0.0004297
    3  -0.0002214   0.0004532   0.0003973   0.0002732   0.0006473   0.0002293
           31          32          33          34          35          36
    1  -0.0000491  -0.0003905  -0.0002489   0.0000392   0.0000141   0.0000839
    2  -0.0003231   0.0003887   0.0002547  -0.0000777   0.0000274  -0.0000944
    3  -0.0000753   0.0002688   0.0002500  -0.0000875   0.0000070  -0.0000237
           37          38
    1  -0.0063337   0.0000759
```

```
    2   0.0038155   0.0003918
    3  -0.0000371   0.0011512
 Max gradient component =       1.135E-02
 RMS gradient           =       1.980E-03
 Gradient time:  CPU 118.41 s  wall 192.85 s

--------------------------------------------------
-  Entering optman on Sun Oct 13 21:42:41 2024  -
--------------------------------------------------

 Geometry Optimization Parameters
   NAtoms,    NIC,     NZ,  NCons,   NDum,   NFix, NCnnct, MaxDiis
       38     272       0       0       0       0       0       0

 Cartesian Hessian Update
 Hessian updated using BFGS update


** GEOMETRY OPTIMIZATION IN DELOCALIZED INTERNAL COORDINATES **
   Searching for a Minimum

   Optimization Cycle:   3

                       Coordinates (Angstroms)
     ATOM                X               Y               Z
      1  N         2.8794506218    0.0731062954   -0.0934308801
      2  C         3.0040762917   -0.8417413130   -1.0978324755
      3  C         3.9321439066   -1.8632624622   -0.9447833496
      4  C         4.7297959070   -1.9426112950    0.1850612216
      5  C         4.6000646976   -0.9756091016    1.1782587252
      6  C         3.6737744771    0.0191475706    1.0082739583
      7  C         2.1524634934   -0.7505754005   -2.3233924872
      8  C         1.9162553890    1.2003916468   -0.1914252249
      9  N        -1.2454027716   -0.2525571744    1.2115872168
     10  C        -2.1524813496    0.0008946625    0.2343582869
     11  N        -1.7997706979    0.6682482633   -0.8402405916
     12  C        -0.5395735175    1.1050133371   -0.9745659837
     13  C         0.4691349629    0.7905518360   -0.0147928043
     14  C         0.0478756011    0.1207579561    1.0973076228
     15  C        -3.5638024043   -0.4199589901    0.4419604351
     16  C        -4.3561757196    0.6616310804    1.2106285546
     17  C        -5.8111246243    0.2459849481    1.4022523022
     18  N        -0.2580148646    1.7654784148   -2.1247536383
     19  H         4.0334006023   -2.5943799183   -1.7369348240
     20  H         5.4558734563   -2.7409877741    0.2871940496
     21  H         5.2192389976   -0.9860924652    2.0657609662
     22  H         3.5460598043    0.8058513477    1.7403344355
     23  H         2.5410137382   -1.4266948164   -3.0824959456
     24  H         2.1345432879    0.2531108075   -2.7515748392
     25  H         1.1218192069   -1.0478750860   -2.1095730077
     26  H         2.0687931114    1.6872114334   -1.1520120569
     27  H         2.1964246253    1.9241529535    0.5743633980
     28  H        -1.5591675818   -0.7422893933    2.0429977599
     29  H         0.6984129181   -0.1567388985    1.9187574347
     30  H        -4.0125388919   -0.5832857961   -0.5386901238
     31  H        -3.5972361844   -1.3673976323    0.9905932565
     32  H        -4.3059756107    1.6028460030    0.6550820856
     33  H        -3.8902605429    0.8402879572    2.1868970404
     34  H        -6.3064798832    0.0926131723    0.4406682571
     35  H        -6.3564042686    1.0227849202    1.9397008566
     36  H        -5.8891808202   -0.6796289711    1.9780395052
     37  H         0.2996393493    2.6083411880   -2.1017832995
     38  H        -1.0766647130    1.8832806944   -2.7117958366
   Point Group: c1    Number of degrees of freedom:   108


   Energy is   -763.963354078

 Hessian updated using BFGS update
  internal optimization (0)
```

```
108 Hessian modes will be used to form the next step
  Hessian Eigenvalues:
     0.002206    0.002740    0.003725    0.004405    0.009736    0.010045
     0.014053    0.018512    0.019124    0.019770    0.020434    0.022186
     0.022579    0.022807    0.023986    0.024276    0.025670    0.026609
     0.028145    0.028334    0.028846    0.030309    0.031800    0.036041
     0.038376    0.041233    0.043297    0.043814    0.044303    0.047754
     0.051043    0.053152    0.053988    0.055096    0.064176    0.077199
     0.083835    0.094133    0.121826    0.122279    0.126854    0.128228
     0.131641    0.132952    0.136122    0.139285    0.142825    0.144660
     0.147510    0.148137    0.149459    0.151664    0.152107    0.152503
     0.154100    0.159751    0.190635    0.192494    0.206088    0.216151
     0.221636    0.228633    0.238124    0.247288    0.247489    0.263058
     0.271746    0.273044    0.280422    0.295343    0.298693    0.300045
     0.300198    0.300877    0.301426    0.302445    0.303552    0.304137
     0.304915    0.305257    0.305673    0.307510    0.308101    0.312511
     0.321472    0.328683    0.331692    0.332488    0.334469    0.337033
     0.342181    0.348205    0.353045    0.360612    0.380894    0.389211
     0.391970    0.401477    0.410585    0.416849    0.420872    0.421840
     0.436515    0.441858    0.452853    0.483776    0.573608    0.779880

 Minimum search - taking simple RFO step
 Searching for Lamda that Minimizes Along All modes
 Value Taken    Lamda =  -0.00541340
  dLimit reduced  1.260000000000000E-002  0.300000000000000
  2.322067906726987E-002  3.867888179467625E-003  7.999999999999999E-004
           0
 Calculated Step part too Large.  Step scaled by  0.162786
 Step Taken.  Stepsize is  0.089912  0.023221

                             Maximum     Tolerance    Cnvgd?
        Gradient            0.003868      0.000800      NO
        Displacement        0.029851      0.001400      NO
        Energy change      -0.003757      0.000228      NO


 New Cartesian Coordinates Obtained by Inverse Iteration

 Displacement from previous Coordinates is:  0.145483
 -----------------------------------------------------------------
             Standard Nuclear Orientation (Angstroms)
    I     Atom           X                Y                Z
 -----------------------------------------------------------------
    1      N       2.8799293418     0.0739211456    -0.0935183864
    2      C       3.0010247445    -0.8455448168    -1.0937998386
    3      C       3.9304085134    -1.8652384970    -0.9389034735
    4      C       4.7301084860    -1.9399527573     0.1897050184
    5      C       4.6010355921    -0.9700658820     1.1805686650
    6      C       3.6744194898     0.0238805575     1.0081159242
    7      C       2.1439133757    -0.7582543210    -2.3154820863
    8      C       1.9177575894     1.2007859023    -0.1954574650
    9      N      -1.2428439064    -0.2581665899     1.2036677547
   10      C      -2.1527386451     0.0010376534     0.2292035469
   11      N      -1.8014089567     0.6738945002    -0.8402678607
   12      C      -0.5419356436     1.1133938019    -0.9757819670
   13      C       0.4706269957     0.7912389164    -0.0200064759
   14      C       0.0506346884     0.1152931878     1.0873827500
   15      C      -3.5628177982    -0.4214600069     0.4375782994
   16      C      -4.3536715990     0.6590847006     1.2109876827
   17      C      -5.8086361631     0.2438994002     1.4031820079
   18      N      -0.2694345072     1.7838059907    -2.1173866443
   19      H       4.0295161265    -2.5984857721    -1.7291346305
   20      H       5.4565636328    -2.7377246653     0.2931049667
   21      H       5.2201668101    -0.9767619526     2.0679977231
   22      H       3.5455736667     0.8138355148     1.7364025890
   23      H       2.5252226307    -1.4407465786    -3.0725165689
   24      H       2.1280649174     0.2431571299    -2.7491276078
   25      H       1.1129225576    -1.0491961212    -2.0946238929
   26      H       2.0719977250     1.6834591595    -1.1579787247
```

```
  27      H       2.1987806106     1.9265021562     0.5679269547
  28      H      -1.5532998229    -0.7528112316     2.0329979363
  29      H       0.7039847402    -0.1687370715     1.9043370349
  30      H      -4.0130063433    -0.5802694247    -0.5431452649
  31      H      -3.5956682638    -1.3704029737     0.9832656096
  32      H      -4.3025017685     1.6012431345     0.6573571501
  33      H      -3.8855285715     0.8340858826     2.1867412294
  34      H      -6.3054624446     0.0938252410     0.4418963068
  35      H      -6.3526175487     1.0196020644     1.9434889937
  36      H      -5.8869809595    -0.6831364257     1.9765365544
  37      H       0.3240856583     2.6011523609    -2.1056939160
  38      H      -1.0881849507     1.9198566872    -2.6996198944
 ----------------------------------------------------------------
 Nuclear Repulsion Energy =        1300.04067653 hartrees
 There are       65 alpha and       65 beta electrons

--------------------------------------------------
-  Entering fldman on Sun Oct 13 21:42:41 2024  -
--------------------------------------------------

 Applying Cartesian multipole field
    Component          Value
    ---------          -----
     (2,0,0)        1.00000E-12
     (0,2,0)        2.00000E-11
     (0,0,2)       -3.00000E-11
 Nucleus-field energy     =    -0.0000000068 hartrees

--------------------------------------------------
-  Entering gesman on Sun Oct 13 21:42:41 2024  -
--------------------------------------------------

 Requested basis set is 6-311+G(d,p)
 There are 188 shells and 516 basis functions
 A cutoff of  1.0D-12 yielded  12554 shell pairs
 There are    100238 function pairs (    106847 Cartesian)
 Smallest overlap matrix eigenvalue = 1.49E-06
 Linear dependence detected in AO basis
 Tighter screening thresholds may be required for diffuse basis sets
 Use S2THRESH > 12 and THRESH = 14 in case of SCF convergence issues
 Number of orthogonalized atomic orbitals = 510
 Maximum deviation from orthogonality = 1.215E-11
 Guess MOs from SCF MO coefficient file
 Reading MOs from coefficient file
 Reading MOs from coefficient file

--------------------------------------------------
-  Entering scfman on Sun Oct 13 21:42:41 2024  -
--------------------------------------------------

 Long-range K will be added via erf
 Coulomb attenuation parameter = 0.2 bohr**(-1)
 A restricted hybrid HF-DFT SCF calculation will be
 performed using Pulay DIIS + Geometric Direct Minimization
 Exchange:     0.2220 Hartree-Fock + 1.0000 wB97X-D + LR-HF
 Correlation:  1.0000 wB97X-D
 Using Euler-Maclaurin-Lebedev (75,302) quadrature formula
 Dispersion:   Grimme D
 SCF converges when RMS gradient is below 1.0E-07
 Geometry optimization detected.  Setting ReadMinima to 0
 Setting SaveMinima to 0
 ---------------------------------------
  Cycle       Energy         DIIS Error
 ---------------------------------------
    1    -763.9705620960      1.55E-04
    2    -763.9641351694      3.28E-05
    3    -763.9641532894      3.25E-05
    4    -763.9641848168      5.48E-06
    5    -763.9641862335      2.50E-06
    6    -763.9641864760      9.47E-07
```

```
    7    -763.9641865204      3.33E-07
    8    -763.9641865259      1.20E-07
    9    -763.9641865269      4.46E-08 Convergence criterion met
 ---------------------------------------
 SCF time:  CPU 241.64 s  wall 391.21 s
 SCF   energy in the final basis set = -763.96418653
 Total energy in the final basis set = -763.96418653

--------------------------------------------------
-  Entering anlman on Sun Oct 13 21:49:12 2024  -
--------------------------------------------------



 --------------------------------------------------------------

                   Orbital Energies (a.u.)
 --------------------------------------------------------------


 Alpha MOs
 -- Occupied --
-14.8168 -14.7970 -14.7283 -14.7121 -10.6762 -10.6659 -10.6518 -10.6390
-10.6349 -10.6261 -10.6078 -10.5834 -10.5720 -10.5707 -10.5481 -10.5278
-10.4976 -10.4514  -1.4200  -1.4045  -1.3453  -1.2752  -1.2307  -1.2009
 -1.1877  -1.1433  -1.1013  -1.0832  -1.0564  -1.0516  -1.0331  -1.0017
 -0.9897  -0.9515  -0.9432  -0.9075  -0.9044  -0.8936  -0.8808  -0.8654
 -0.8519  -0.8452  -0.8332  -0.8220  -0.8093  -0.7965  -0.7863  -0.7813
 -0.7700  -0.7656  -0.7526  -0.7494  -0.7409  -0.7290  -0.7180  -0.7135
 -0.6828  -0.6697  -0.6574  -0.6441  -0.6338  -0.6214  -0.6202  -0.6174
 -0.6123
 -- Virtual --
 -0.2696  -0.2670  -0.2486  -0.2264  -0.1751  -0.1639  -0.1509  -0.1454
 -0.1321  -0.1237  -0.1180  -0.1152  -0.1108  -0.1034  -0.0993  -0.0933
 -0.0881  -0.0854  -0.0813  -0.0801  -0.0760  -0.0695  -0.0674  -0.0641
 -0.0617  -0.0579  -0.0569  -0.0521  -0.0482  -0.0423  -0.0368  -0.0322
 -0.0304  -0.0253  -0.0234  -0.0230  -0.0172  -0.0157  -0.0114  -0.0071
 -0.0057   0.0009   0.0026   0.0079   0.0097   0.0130   0.0136   0.0182
  0.0205   0.0214   0.0268   0.0290   0.0316   0.0358   0.0370   0.0395
  0.0403   0.0441   0.0538   0.0545   0.0572   0.0600   0.0628   0.0638
  0.0722   0.0736   0.0762   0.0780   0.0813   0.0859   0.0895   0.0913
  0.0939   0.0971   0.1013   0.1088   0.1125   0.1135   0.1159   0.1186
  0.1215   0.1277   0.1296   0.1310   0.1390   0.1400   0.1411   0.1503
  0.1543   0.1569   0.1620   0.1660   0.1690   0.1713   0.1735   0.1766
  0.1821   0.1880   0.1888   0.1955   0.1977   0.2052   0.2136   0.2143
  0.2207   0.2282   0.2559   0.2672   0.2717   0.2843   0.2961   0.3123
  0.3256   0.3279   0.3366   0.3468   0.3482   0.3538   0.3626   0.3702
  0.3759   0.3796   0.3878   0.3924   0.4002   0.4067   0.4169   0.4199
  0.4256   0.4364   0.4437   0.4520   0.4615   0.4670   0.4718   0.4801
  0.4823   0.4923   0.4983   0.5020   0.5065   0.5123   0.5230   0.5259
  0.5290   0.5310   0.5372   0.5382   0.5488   0.5519   0.5534   0.5598
  0.5635   0.5713   0.5771   0.5823   0.5884   0.5906   0.5931   0.5987
  0.6085   0.6156   0.6267   0.6311   0.6354   0.6425   0.6562   0.6604
  0.6635   0.6691   0.6800   0.6848   0.6892   0.6964   0.6984   0.7010
  0.7154   0.7239   0.7345   0.7389   0.7618   0.7693   0.7791   0.7871
  0.7894   0.7944   0.8082   0.8173   0.8322   0.8372   0.8474   0.8518
  0.8650   0.8708   0.8761   0.8796   0.8944   0.9074   0.9169   0.9216
  0.9312   0.9421   0.9598   0.9762   0.9804   0.9926   1.0161   1.0260
  1.0511   1.0754   1.1010   1.1126   1.1272   1.1509   1.1621   1.1709
  1.2000   1.2238   1.2429   1.2514   1.2569   1.2791   1.3061   1.3119
  1.3193   1.3272   1.3372   1.3420   1.3493   1.3633   1.3665   1.3733
  1.3862   1.3953   1.3983   1.4079   1.4123   1.4144   1.4279   1.4327
  1.4453   1.4453   1.4615   1.4679   1.4699   1.4832   1.4909   1.4975
  1.5033   1.5088   1.5168   1.5195   1.5349   1.5444   1.5480   1.5538
  1.5600   1.5629   1.5745   1.5829   1.5946   1.5993   1.6138   1.6170
  1.6251   1.6284   1.6445   1.6476   1.6604   1.6712   1.6911   1.6981
  1.7041   1.7299   1.7312   1.7428   1.7434   1.7545   1.7617   1.7666
  1.7807   1.7876   1.8028   1.8328   1.8421   1.8658   1.8731   1.8884
  1.8969   1.9039   1.9144   1.9306   1.9421   1.9588   1.9635   1.9808
  1.9956   2.0103   2.0253   2.0406   2.0443   2.0513   2.0617   2.0832
  2.0904   2.1009   2.1304   2.1396   2.1589   2.1927   2.2022   2.2195
  2.2313   2.2493   2.2515   2.2544   2.2663   2.2890   2.2933   2.3054
```

| | | | | | | | |
|---|---|---|---|---|---|---|---|
| 2.3161 | 2.3319 | 2.3477 | 2.3625 | 2.3738 | 2.3896 | 2.3916 | 2.4048 |
| 2.4103 | 2.4302 | 2.4354 | 2.4510 | 2.4569 | 2.4626 | 2.4716 | 2.4826 |
| 2.4978 | 2.5059 | 2.5203 | 2.5235 | 2.5391 | 2.5411 | 2.5470 | 2.5539 |
| 2.5641 | 2.5793 | 2.5859 | 2.5896 | 2.5950 | 2.5986 | 2.6155 | 2.6218 |
| 2.6395 | 2.6478 | 2.6561 | 2.6580 | 2.6662 | 2.6772 | 2.6873 | 2.7065 |
| 2.7107 | 2.7171 | 2.7242 | 2.7325 | 2.7368 | 2.7590 | 2.7709 | 2.7731 |
| 2.7850 | 2.7945 | 2.8120 | 2.8418 | 2.8465 | 2.8504 | 2.8632 | 2.8857 |
| 2.8951 | 2.9231 | 2.9391 | 2.9473 | 3.0321 | 3.0709 | 3.1331 | 3.1482 |
| 3.1546 | 3.1967 | 3.2448 | 3.2554 | 3.2957 | 3.3178 | 3.3599 | 3.3895 |
| 3.4182 | 3.4755 | 3.5154 | 3.5346 | 3.5552 | 3.5928 | 3.6213 | 3.6460 |
| 3.6833 | 3.7144 | 3.7207 | 3.7352 | 3.7375 | 3.7526 | 3.7632 | 3.7691 |
| 3.8264 | 3.8592 | 3.8676 | 3.9050 | 3.9372 | 3.9933 | 4.0482 | 4.0920 |
| 4.0936 | 4.1825 | 4.2961 | 4.3076 | 4.5875 | 4.6587 | 4.6976 | 4.7264 |
| 4.9037 | 5.0356 | 5.1851 | 23.5218 | 23.5740 | 23.7166 | 23.7317 | 23.7397 |
| 23.7743 | 23.8016 | 23.8336 | 23.8383 | 23.8928 | 23.9231 | 23.9654 | 23.9713 |
| 24.0352 | 35.4198 | 35.4532 | 35.5057 | 35.6036 | | | |

Ground-State Mulliken Net Atomic Charges

| Atom | Charge (a.u.) |
|---|---|
| 1 N | 0.439747 |
| 2 C | 0.079215 |
| 3 C | -0.035000 |
| 4 C | -0.231385 |
| 5 C | -0.285207 |
| 6 C | -0.066249 |
| 7 C | -0.719785 |
| 8 C | -0.202270 |
| 9 N | -0.121499 |
| 10 C | -0.361554 |
| 11 N | 0.079360 |
| 12 C | 0.117994 |
| 13 C | -0.015383 |
| 14 C | 0.164707 |
| 15 C | -0.377531 |
| 16 C | -0.084734 |
| 17 C | -0.606490 |
| 18 N | -0.457936 |
| 19 H | 0.220853 |
| 20 H | 0.235727 |
| 21 H | 0.231635 |
| 22 H | 0.217073 |
| 23 H | 0.233012 |
| 24 H | 0.215672 |
| 25 H | 0.241228 |
| 26 H | 0.196360 |
| 27 H | 0.248185 |
| 28 H | 0.395171 |
| 29 H | 0.214111 |
| 30 H | 0.241388 |
| 31 H | 0.209400 |
| 32 H | 0.199241 |
| 33 H | 0.170347 |
| 34 H | 0.178158 |
| 35 H | 0.198803 |
| 36 H | 0.169309 |
| 37 H | 0.314134 |
| 38 H | 0.354192 |

Sum of atomic charges = 2.000000

Cartesian Multipole Moments

Charge (ESU x 10^10)
9.6064
Dipole Moment (Debye)
X 9.0196 Y -0.1762 Z 1.0612

```
       Tot        9.0835
    Quadrupole Moments (Debye-Ang)
        XX      -0.4112      XY     -15.8041      YY     -77.6785
        XZ      -5.9185      YZ      -6.5338      ZZ     -71.8741
    Traceless Quadrupole Moments (Debye-Ang)
       QXX     148.7301     QYY     -83.0717     QZZ     -65.6585
       QXY     -47.4124     QXZ     -17.7556     QYZ     -19.6015
    Octopole Moments (Debye-Ang^2)
       XXX      48.0420     XXY     -94.2514     XYY      25.0191
       YYY       8.7220     XXZ      72.0291     XYZ      14.7594
       YYZ     -26.7579     XZZ      -6.2352     YZZ       7.0763
       ZZZ      -4.0818
    Traceless Octopole Moments (Debye-Ang^2)
       XXX     119.1971     YYY     836.9083     ZZZ    -431.9319
       XXY   -1178.4115     XXZ     956.8681     XYY     174.8088
       XYZ     221.3903     XZZ    -294.0060     YYZ    -524.9362
       YZZ     341.5032
    Hexadecapole Moments (Debye-Ang^3)
      XXXX   -4194.7860    XXXY    -110.1596   XXYY    -886.5981
      XYYY     345.2257    YYYY    -632.8719   XXXZ     371.2978
      XXYZ      36.6721    XYYZ      81.0538   YYYZ     -10.2424
      XXZZ    -991.1698    XYZZ     104.8251   YYZZ    -203.5225
      XZZZ     324.8776    YZZZ      -9.4217   ZZZZ    -854.8445
    Traceless Hexadecapole Moments (Debye-Ang^3)
      XXXX   17471.5756    XXXY  -26861.8597   XXXZ    4010.9498
      XXYY   -5694.8579    XXYZ    3589.1229   XXZZ  -11776.7177
      XYYY   20953.5909    XYYZ   -3147.7870   XYZZ    5908.2689
      XZZZ    -863.1628    YYYY      12.0275   YYYZ   -1859.7884
      YYZZ    5682.8304    YZZZ   -1729.3346   ZZZZ    6093.8873
 -----------------------------------------------------------------

 --------------------------------------------------
-  Entering drvman on Sun Oct 13 21:49:12 2024  -
 --------------------------------------------------

 Calculating analytic gradient of the SCF energy
 Gradient of SCF Energy
            1           2           3           4           5           6
    1  -0.0006324   0.0010311  -0.0011191   0.0001234  -0.0005995   0.0004236
    2  -0.0010130   0.0007020  -0.0000596  -0.0001708   0.0005562   0.0001163
    3   0.0001554   0.0002591  -0.0006272   0.0002537   0.0001049  -0.0006823
            7           8           9          10          11          12
    1   0.0002576  -0.0002453   0.0002277   0.0023136  -0.0006735  -0.0020228
    2  -0.0001447   0.0001274   0.0009287  -0.0011831   0.0022772   0.0000695
    3  -0.0004742  -0.0007376  -0.0023518   0.0034055  -0.0012780   0.0074400
           13          14          15          16          17          18
    1  -0.0026055   0.0002959  -0.0007407   0.0006965  -0.0001210   0.0100064
    2  -0.0009114  -0.0005014   0.0012555  -0.0011262   0.0002934  -0.0042229
    3  -0.0027873   0.0011062  -0.0002097  -0.0005900   0.0001533  -0.0063007
           19          20          21          22          23          24
    1   0.0002255   0.0000860   0.0002230   0.0006175   0.0002313  -0.0001536
    2  -0.0002685   0.0000006  -0.0002943  -0.0003604   0.0002324   0.0000174
    3   0.0001205   0.0000461  -0.0001316   0.0003133   0.0000429  -0.0000454
           25          26          27          28          29          30
    1   0.0001543  -0.0005519  -0.0003704  -0.0006578  -0.0003502  -0.0000901
    2  -0.0003188   0.0004237  -0.0001170  -0.0002364   0.0000860  -0.0003236
    3  -0.0002758   0.0003264   0.0003662   0.0001717   0.0005297   0.0001907
           31          32          33          34          35          36
    1  -0.0000401  -0.0003054  -0.0001964   0.0000297   0.0000295   0.0000529
    2  -0.0002586   0.0002960   0.0002051  -0.0000585   0.0000091  -0.0000617
    3  -0.0000722   0.0002315   0.0001926  -0.0000497  -0.0000079  -0.0000264
           37          38
    1  -0.0057034   0.0001536
    2   0.0036819   0.0003523
    3   0.0001038   0.0011346
 Max gradient component =       1.001E-02
 RMS gradient           =       1.698E-03
 Gradient time:  CPU 125.48 s  wall 199.62 s

 --------------------------------------------------
```

```
-  Entering optman on Sun Oct 13 21:52:32 2024  -
------------------------------------------------

 Geometry Optimization Parameters
   NAtoms,    NIC,     NZ,  NCons,   NDum,   NFix, NCnnct, MaxDiis
       38     272       0       0       0       0       0       0

 Cartesian Hessian Update
 Hessian updated using BFGS update


** GEOMETRY OPTIMIZATION IN DELOCALIZED INTERNAL COORDINATES **
   Searching for a Minimum

   Optimization Cycle:   4

                      Coordinates (Angstroms)
     ATOM                X               Y               Z
      1  N         2.8799293418    0.0739211456   -0.0935183864
      2  C         3.0010247445   -0.8455448168   -1.0937998386
      3  C         3.9304085134   -1.8652384970   -0.9389034735
      4  C         4.7301084860   -1.9399527573    0.1897050184
      5  C         4.6010355921   -0.9700658820    1.1805686650
      6  C         3.6744194898    0.0238805575    1.0081159242
      7  C         2.1439133757   -0.7582543210   -2.3154820863
      8  C         1.9177575894    1.2007859023   -0.1954574650
      9  N        -1.2428439064   -0.2581665899    1.2036677547
     10  C        -2.1527386451    0.0010376534    0.2292035469
     11  N        -1.8014089567    0.6738945002   -0.8402678607
     12  C        -0.5419356436    1.1133938019   -0.9757819670
     13  C         0.4706269957    0.7912389164   -0.0200064759
     14  C         0.0506346884    0.1152931878    1.0873827500
     15  C        -3.5628177982   -0.4214600069    0.4375782994
     16  C        -4.3536715990    0.6590847006    1.2109876827
     17  C        -5.8086361631    0.2438994002    1.4031820079
     18  N        -0.2694345072    1.7838059907   -2.1173866443
     19  H         4.0295161265   -2.5984857721   -1.7291346305
     20  H         5.4565636328   -2.7377246653    0.2931049667
     21  H         5.2201668101   -0.9767619526    2.0679977231
     22  H         3.5455736667    0.8138355148    1.7364025890
     23  H         2.5252226307   -1.4407465786   -3.0725165689
     24  H         2.1280649174    0.2431571299   -2.7491276078
     25  H         1.1129225576   -1.0491961212   -2.0946238929
     26  H         2.0719977250    1.6834591595   -1.1579787247
     27  H         2.1987806106    1.9265021562    0.5679269547
     28  H        -1.5532998229   -0.7528112316    2.0329979363
     29  H         0.7039847402   -0.1687370715    1.9043370349
     30  H        -4.0130063433   -0.5802694247   -0.5431452649
     31  H        -3.5956682638   -1.3704029737    0.9832656096
     32  H        -4.3025017685    1.6012431345    0.6573571501
     33  H        -3.8855285715    0.8340858826    2.1867412294
     34  H        -6.3054624446    0.0938252410    0.4418963068
     35  H        -6.3526175487    1.0196020644    1.9434889937
     36  H        -5.8869809595   -0.6831364257    1.9765365544
     37  H         0.3240856583    2.6011523609   -2.1056939160
     38  H        -1.0881849507    1.9198566872   -2.6996198944
   Point Group: c1    Number of degrees of freedom:   108


   Energy is   -763.964186527

 Hessian updated using BFGS update
  internal optimization (0)

108 Hessian modes will be used to form the next step
  Hessian Eigenvalues:
     0.002191     0.002741     0.003726     0.004420     0.008969     0.010287
     0.014022     0.018514     0.019192     0.019761     0.020478     0.022199
     0.022581     0.022807     0.023955     0.024217     0.025719     0.026600
     0.027683     0.028279     0.028397     0.030249     0.031086     0.036045
```

```
   0.038383    0.041285    0.043300    0.043814    0.044303    0.047738
   0.050431    0.053014    0.054062    0.055102    0.064179    0.077387
   0.083835    0.094155    0.121820    0.122290    0.126855    0.128230
   0.131782    0.132969    0.136369    0.140198    0.142321    0.143899
   0.147520    0.148183    0.149789    0.151664    0.152080    0.152533
   0.154126    0.160462    0.190657    0.193145    0.206178    0.216179
   0.223551    0.228809    0.238340    0.247331    0.247472    0.263567
   0.271763    0.274729    0.281850    0.295413    0.298812    0.300145
   0.300391    0.300954    0.301433    0.302469    0.303636    0.304137
   0.304916    0.305487    0.305694    0.307495    0.308153    0.312613
   0.321676    0.328870    0.331958    0.332691    0.334485    0.337085
   0.342172    0.348422    0.353005    0.361883    0.380851    0.389419
   0.395404    0.406326    0.416530    0.419666    0.420140    0.424139
   0.437089    0.444303    0.453762    0.492464    0.575661    0.780337

Minimum search - taking simple RFO step
Searching for Lamda that Minimizes Along All modes
Value Taken    Lamda =  -0.00481009
Calculated Step too Large.  Step scaled by  0.536666
Step Taken.  Stepsize is  0.300000

                           Maximum     Tolerance    Cnvgd?
        Gradient           0.003056     0.000800      NO
        Displacement       0.103458     0.001400      NO
        Energy change     -0.000832     0.000228      NO


New Cartesian Coordinates Obtained by Inverse Iteration

Displacement from previous Coordinates is:  0.492497
----------------------------------------------------------------
            Standard Nuclear Orientation (Angstroms)
   I     Atom           X                Y                Z
----------------------------------------------------------------
   1      N       2.8817334474     0.0796064378    -0.0925198162
   2      C       2.9884327892    -0.8564273582    -1.0785068072
   3      C       3.9200840408    -1.8727229643    -0.9182042972
   4      C       4.7295137418    -1.9316464661     0.2044151237
   5      C       4.6073399784    -0.9503259286     1.1851851542
   6      C       3.6806154047     0.0426097757     1.0059728574
   7      C       2.1117626184    -0.7828437667    -2.2868868345
   8      C       1.9223346022     1.2056778856    -0.2061911153
   9      N      -1.2342177620    -0.2810514259     1.1766317879
  10      C      -2.1521523398    -0.0014989883     0.2109908269
  11      N      -1.8063088270     0.6919383399    -0.8436340231
  12      C      -0.5480853881     1.1404912087    -0.9818334839
  13      C       0.4747766856     0.7946909828    -0.0374152208
  14      C       0.0589814909     0.0947736944     1.0555086615
  15      C      -3.5601072353    -0.4269933073     0.4217310201
  16      C      -4.3444729544     0.6489200000     1.2111894298
  17      C      -5.7999512437     0.2373255843     1.4060842080
  18      N      -0.3015713115     1.8479927479    -2.0925966561
  19      H       4.0101728227    -2.6160943271    -1.6997438124
  20      H       5.4563967476    -2.7283032812     0.3118073737
  21      H       5.2299355538    -0.9457969992     2.0700492143
  22      H       3.5531191329     0.8436110864     1.7223426677
  23      H       2.4626827830    -1.4925181389    -3.0334056672
  24      H       2.1107788911     0.2081711607    -2.7450724359
  25      H       1.0785719343    -1.0430922036    -2.0387882187
  26      H       2.0853025532     1.6799576618    -1.1718134194
  27      H       2.2022308170     1.9366837029     0.5522830403
  28      H      -1.5348026768    -0.7929579005     1.9980796893
  29      H       0.7210517529    -0.2086731833     1.8582353748
  30      H      -4.0154598562    -0.5710246103    -0.5588638583
  31      H      -3.5919153291    -1.3814438776     0.9569077211
  32      H      -4.2901678222     1.5950307189     0.6650667692
  33      H      -3.8691916069     0.8114199481     2.1854354574
  34      H      -6.3020317053     0.0990574065     0.4458499753
  35      H      -6.3386338732     1.0095187976     1.9566296040
  36      H      -5.8796153357    -0.6948433154     1.9706927025
```

```
   37      H       0.3990973405     2.5722718794    -2.1224009117
   38      H      -1.1162298613     2.0385090232    -2.6632120809
 ----------------------------------------------------------------
 Nuclear Repulsion Energy =        1300.77603190 hartrees
 There are       65 alpha and       65 beta electrons

--------------------------------------------------
-  Entering fldman on Sun Oct 13 21:52:32 2024  -
--------------------------------------------------

 Applying Cartesian multipole field
    Component          Value
    ---------          -----
     (2,0,0)        1.00000E-12
     (0,2,0)        2.00000E-11
     (0,0,2)       -3.00000E-11
 Nucleus-field energy     =    -0.0000000062 hartrees

--------------------------------------------------
-  Entering gesman on Sun Oct 13 21:52:32 2024  -
--------------------------------------------------

 Requested basis set is 6-311+G(d,p)
 There are 188 shells and 516 basis functions
 A cutoff of  1.0D-12 yielded  12572 shell pairs
 There are    100340 function pairs (    106963 Cartesian)
 Smallest overlap matrix eigenvalue = 1.51E-06
 Linear dependence detected in AO basis
 Tighter screening thresholds may be required for diffuse basis sets
 Use S2THRESH > 12 and THRESH = 14 in case of SCF convergence issues
 Number of orthogonalized atomic orbitals = 510
 Maximum deviation from orthogonality = 1.994E-11
 Guess MOs from SCF MO coefficient file
 Reading MOs from coefficient file
 Reading MOs from coefficient file

--------------------------------------------------
-  Entering scfman on Sun Oct 13 21:52:32 2024  -
--------------------------------------------------

 Long-range K will be added via erf
 Coulomb attenuation parameter = 0.2 bohr**(-1)
 A restricted hybrid HF-DFT SCF calculation will be
 performed using Pulay DIIS + Geometric Direct Minimization
 Exchange:     0.2220 Hartree-Fock + 1.0000 wB97X-D + LR-HF
 Correlation:  1.0000 wB97X-D
 Using Euler-Maclaurin-Lebedev (75,302) quadrature formula
 Dispersion:   Grimme D
 SCF converges when RMS gradient is below 1.0E-07
 Geometry optimization detected.  Setting ReadMinima to 0
 Setting SaveMinima to 0
 ---------------------------------------
  Cycle       Energy         DIIS Error
 ---------------------------------------
    1    -763.9673829970      5.13E-04
    2    -763.9658434045      8.77E-05
    3    -763.9660941214      7.46E-05
    4    -763.9662639930      2.05E-05
    5    -763.9662814655      6.43E-06
    6    -763.9662831902      2.83E-06
    7    -763.9662835498      8.60E-07
    8    -763.9662835897      3.58E-07
    9    -763.9662835965      1.04E-07
   10    -763.9662835973      4.84E-08 Convergence criterion met
 ---------------------------------------
 SCF time:  CPU 277.70 s  wall 438.12 s
 SCF   energy in the final basis set = -763.96628360
 Total energy in the final basis set = -763.96628360

--------------------------------------------------
```

```
-  Entering anlman on Sun Oct 13 21:59:50 2024  -
------------------------------------------------


 --------------------------------------------------------------

                    Orbital Energies (a.u.)
 --------------------------------------------------------------

 Alpha MOs
 -- Occupied --
-14.8174 -14.7936 -14.7327 -14.7095 -10.6766 -10.6640 -10.6498 -10.6398
-10.6360 -10.6268 -10.6069 -10.5843 -10.5728 -10.5716 -10.5501 -10.5263
-10.4966 -10.4505  -1.4174  -1.4056  -1.3467  -1.2755  -1.2310  -1.2004
 -1.1889  -1.1425  -1.1008  -1.0845  -1.0552  -1.0513  -1.0340  -1.0008
 -0.9904  -0.9525  -0.9426  -0.9094  -0.9056  -0.8939  -0.8818  -0.8651
 -0.8537  -0.8460  -0.8322  -0.8229  -0.8096  -0.7975  -0.7873  -0.7819
 -0.7687  -0.7643  -0.7540  -0.7518  -0.7443  -0.7299  -0.7188  -0.7129
 -0.6819  -0.6688  -0.6578  -0.6431  -0.6325  -0.6219  -0.6191  -0.6162
 -0.6103
 -- Virtual --
 -0.2702  -0.2635  -0.2463  -0.2270  -0.1747  -0.1647  -0.1505  -0.1453
 -0.1330  -0.1234  -0.1180  -0.1152  -0.1109  -0.1038  -0.0996  -0.0943
 -0.0887  -0.0852  -0.0810  -0.0800  -0.0758  -0.0694  -0.0673  -0.0644
 -0.0620  -0.0576  -0.0567  -0.0525  -0.0479  -0.0422  -0.0376  -0.0318
 -0.0310  -0.0256  -0.0233  -0.0231  -0.0166  -0.0160  -0.0108  -0.0078
 -0.0063   0.0012   0.0031   0.0077   0.0094   0.0126   0.0132   0.0175
  0.0209   0.0219   0.0269   0.0281   0.0319   0.0359   0.0373   0.0398
  0.0412   0.0441   0.0537   0.0544   0.0581   0.0600   0.0638   0.0652
  0.0717   0.0729   0.0761   0.0780   0.0813   0.0860   0.0896   0.0916
  0.0951   0.0963   0.1015   0.1091   0.1108   0.1146   0.1158   0.1199
  0.1213   0.1276   0.1300   0.1324   0.1390   0.1399   0.1414   0.1506
  0.1548   0.1568   0.1619   0.1658   0.1701   0.1716   0.1762   0.1787
  0.1833   0.1888   0.1919   0.1957   0.1991   0.2066   0.2134   0.2154
  0.2204   0.2293   0.2565   0.2679   0.2709   0.2808   0.2967   0.3117
  0.3256   0.3284   0.3367   0.3475   0.3481   0.3542   0.3648   0.3706
  0.3766   0.3805   0.3873   0.3928   0.4017   0.4066   0.4177   0.4205
  0.4272   0.4364   0.4445   0.4525   0.4615   0.4650   0.4719   0.4788
  0.4828   0.4916   0.4998   0.5025   0.5072   0.5134   0.5261   0.5278
  0.5287   0.5305   0.5367   0.5394   0.5496   0.5508   0.5521   0.5625
  0.5653   0.5712   0.5798   0.5812   0.5902   0.5920   0.5969   0.6000
  0.6071   0.6166   0.6276   0.6300   0.6365   0.6449   0.6572   0.6616
  0.6626   0.6693   0.6783   0.6857   0.6873   0.6975   0.6989   0.7033
  0.7160   0.7207   0.7350   0.7389   0.7578   0.7679   0.7790   0.7876
  0.7898   0.7959   0.8116   0.8195   0.8346   0.8376   0.8461   0.8554
  0.8638   0.8704   0.8770   0.8827   0.8936   0.9081   0.9195   0.9234
  0.9310   0.9447   0.9621   0.9779   0.9800   0.9921   1.0196   1.0284
  1.0536   1.0783   1.0963   1.1122   1.1290   1.1502   1.1632   1.1710
  1.1979   1.2255   1.2419   1.2486   1.2550   1.2768   1.3042   1.3121
  1.3183   1.3267   1.3365   1.3441   1.3498   1.3639   1.3681   1.3736
  1.3871   1.3966   1.3993   1.4043   1.4110   1.4162   1.4265   1.4299
  1.4458   1.4483   1.4614   1.4674   1.4692   1.4850   1.4915   1.4972
  1.5045   1.5103   1.5160   1.5189   1.5354   1.5456   1.5482   1.5542
  1.5594   1.5629   1.5760   1.5830   1.5953   1.6032   1.6130   1.6175
  1.6252   1.6281   1.6420   1.6475   1.6571   1.6710   1.6826   1.6974
  1.7092   1.7263   1.7343   1.7420   1.7431   1.7532   1.7625   1.7675
  1.7806   1.7877   1.8032   1.8311   1.8403   1.8651   1.8723   1.8920
  1.8982   1.9059   1.9191   1.9362   1.9406   1.9598   1.9687   1.9857
  1.9953   2.0111   2.0253   2.0391   2.0457   2.0485   2.0620   2.0841
  2.0916   2.0997   2.1320   2.1417   2.1604   2.1946   2.2059   2.2200
  2.2319   2.2500   2.2525   2.2544   2.2613   2.2863   2.2896   2.3025
  2.3152   2.3318   2.3444   2.3613   2.3749   2.3895   2.3921   2.4046
  2.4123   2.4319   2.4344   2.4460   2.4512   2.4634   2.4712   2.4835
  2.4985   2.5063   2.5192   2.5249   2.5390   2.5424   2.5515   2.5538
  2.5643   2.5785   2.5850   2.5904   2.5957   2.5977   2.6165   2.6224
  2.6433   2.6461   2.6504   2.6598   2.6687   2.6788   2.6863   2.7070
  2.7128   2.7176   2.7338   2.7350   2.7402   2.7578   2.7715   2.7742
  2.7810   2.8013   2.8143   2.8418   2.8483   2.8515   2.8676   2.8840
  2.8949   2.9230   2.9410   2.9494   3.0348   3.0689   3.1344   3.1520
  3.1625   3.2169   3.2481   3.2574   3.2970   3.3181   3.3612   3.3974
```

```
  3.4185   3.4770   3.5195   3.5348   3.5567   3.5933   3.6205   3.6438
  3.6842   3.7167   3.7206   3.7364   3.7387   3.7547   3.7634   3.7674
  3.8216   3.8296   3.8669   3.9038   3.9373   3.9871   4.0483   4.0916
  4.0927   4.1829   4.2947   4.3060   4.5909   4.6630   4.6995   4.7322
  4.9138   5.0359   5.1880  23.5212  23.5789  23.7148  23.7348  23.7385
 23.7735  23.8026  23.8336  23.8395  23.8919  23.9241  23.9656  23.9731
 24.0342  35.4193  35.4573  35.5114  35.6109
 --------------------------------------------------------------

          Ground-State Mulliken Net Atomic Charges

     Atom                 Charge (a.u.)
  ----------------------------------------
      1 N                     0.434501
      2 C                     0.086503
      3 C                    -0.032885
      4 C                    -0.234492
      5 C                    -0.282387
      6 C                    -0.063340
      7 C                    -0.722795
      8 C                    -0.196933
      9 N                    -0.125654
     10 C                    -0.366916
     11 N                     0.072536
     12 C                     0.144534
     13 C                    -0.030536
     14 C                     0.163298
     15 C                    -0.374447
     16 C                    -0.084594
     17 C                    -0.607401
     18 N                    -0.459606
     19 H                     0.220647
     20 H                     0.236117
     21 H                     0.232061
     22 H                     0.217395
     23 H                     0.234286
     24 H                     0.215143
     25 H                     0.243649
     26 H                     0.188089
     27 H                     0.248186
     28 H                     0.394168
     29 H                     0.212768
     30 H                     0.240782
     31 H                     0.208078
     32 H                     0.198863
     33 H                     0.170421
     34 H                     0.177862
     35 H                     0.198476
     36 H                     0.169388
     37 H                     0.313270
     38 H                     0.360963
  ----------------------------------------
  Sum of atomic charges =     2.000000

 -----------------------------------------------------------------
                    Cartesian Multipole Moments
 -----------------------------------------------------------------
    Charge (ESU x 10^10)
                 9.6064
    Dipole Moment (Debye)
         X       9.2559      Y      -0.1323      Z       0.7873
       Tot       9.2903
    Quadrupole Moments (Debye-Ang)
        XX      -0.5734     XY     -15.4898     YY     -77.2508
        XZ      -5.6003     YZ      -7.1436     ZZ     -72.0766
    Traceless Quadrupole Moments (Debye-Ang)
       QXX     148.1807    QYY     -81.8515    QZZ     -66.3292
       QXY     -46.4695    QXZ     -16.8009    QYZ     -21.4309
    Octopole Moments (Debye-Ang^2)
       XXX      51.2700    XXY     -93.1508    XYY      25.3690
```

```
      YYY       7.6800    XXZ      72.8302    XYZ      14.6398
      YYZ     -27.9666    XZZ      -5.6451    YZZ       8.4495
      ZZZ      -5.4291
   Traceless Octopole Moments (Debye-Ang^2)
      XXX     130.1048    YYY     808.3919    ZZZ    -436.3467
      XXY   -1166.1986    XXZ     974.1499    XYY     167.5528
      XYZ     219.5963    XZZ    -297.6576    YYZ    -537.8032
      YZZ     357.8067
   Hexadecapole Moments (Debye-Ang^3)
     XXXX   -4188.2369   XXXY    -106.6031   XXYY    -885.6437
     XYYY     341.2838   YYYY    -645.5950   XXXZ     373.6780
     XXYZ      34.6074   XYYZ      78.4246   YYYZ      -8.7017
     XXZZ    -988.9938   XYZZ     105.0516   YYZZ    -197.6730
     XZZZ     316.4396   YZZZ     -10.3384   ZZZZ    -842.7396
   Traceless Hexadecapole Moments (Debye-Ang^3)
     XXXX   17503.0862   XXXY  -26481.2787   XXXZ    4651.7928
     XXYY   -5579.3750   XXYZ    3395.1954   XXZZ  -11923.7112
     XYYY   20546.8435   XYYZ   -3293.5555   XYZZ    5934.4352
     XZZZ   -1358.2374   YYYY    -576.1497   YYYZ   -1629.4389
     YYZZ    6155.5247   YZZZ   -1765.7565   ZZZZ    5768.1865
 -----------------------------------------------------------------

--------------------------------------------------
-  Entering drvman on Sun Oct 13 21:59:50 2024  -
--------------------------------------------------

 Calculating analytic gradient of the SCF energy
 Gradient of SCF Energy
            1           2           3           4           5           6
    1  -0.0001768   0.0005924  -0.0005285   0.0000417  -0.0003621   0.0003303
    2  -0.0005544   0.0002253  -0.0000574  -0.0002374   0.0004375  -0.0001503
    3  -0.0001465  -0.0001226  -0.0000343  -0.0001064   0.0002248  -0.0001573
            7           8           9          10          11          12
    1   0.0003371   0.0002483   0.0001743   0.0000929   0.0001544  -0.0019968
    2   0.0001296   0.0001776   0.0004986  -0.0010958   0.0011338   0.0020244
    3  -0.0002497  -0.0002149  -0.0008343   0.0010477  -0.0001812   0.0027044
           13          14          15          16          17          18
    1  -0.0006183   0.0002731  -0.0001367   0.0001954  -0.0001302   0.0059377
    2  -0.0009300  -0.0004007   0.0004496  -0.0003350   0.0001279  -0.0045793
    3  -0.0015899   0.0012050  -0.0001529  -0.0001133   0.0001033  -0.0039271
           19          20          21          22          23          24
    1   0.0000137  -0.0000356   0.0000277   0.0002009   0.0001318  -0.0003990
    2   0.0000227   0.0000320  -0.0000531  -0.0001386   0.0001568   0.0000864
    3   0.0000531  -0.0000023  -0.0000973   0.0000332   0.0000882   0.0000408
           25          26          27          28          29          30
    1  -0.0001250  -0.0006179   0.0000018  -0.0002045  -0.0001304   0.0000440
    2  -0.0001141   0.0002288  -0.0003089  -0.0000004   0.0000406  -0.0000611
    3  -0.0000610   0.0002119   0.0002518   0.0000055   0.0002202   0.0001290
           31          32          33          34          35          36
    1  -0.0000240  -0.0000943  -0.0000704   0.0000216   0.0000136   0.0000112
    2  -0.0000953   0.0000844   0.0000892  -0.0000111  -0.0000291   0.0000022
    3  -0.0000533   0.0001043   0.0000739   0.0000070  -0.0000246  -0.0000234
           37          38
    1  -0.0034805   0.0002873
    2   0.0028367   0.0003677
    3   0.0005933   0.0009950
 Max gradient component =       5.938E-03
 RMS gradient           =       1.036E-03
 Gradient time:  CPU 114.81 s  wall 181.47 s

--------------------------------------------------
-  Entering optman on Sun Oct 13 22:02:52 2024  -
--------------------------------------------------

 Geometry Optimization Parameters
   NAtoms,    NIC,     NZ,  NCons,   NDum,   NFix, NCnnct, MaxDiis
      38     272       0       0       0       0       0       0

 Cartesian Hessian Update
 Hessian updated using BFGS update
```

```
** GEOMETRY OPTIMIZATION IN DELOCALIZED INTERNAL COORDINATES **
   Searching for a Minimum

   Optimization Cycle:   5

                     Coordinates (Angstroms)
     ATOM                X               Y               Z
      1  N         2.8817334474    0.0796064378   -0.0925198162
      2  C         2.9884327892   -0.8564273582   -1.0785068072
      3  C         3.9200840408   -1.8727229643   -0.9182042972
      4  C         4.7295137418   -1.9316464661    0.2044151237
      5  C         4.6073399784   -0.9503259286    1.1851851542
      6  C         3.6806154047    0.0426097757    1.0059728574
      7  C         2.1117626184   -0.7828437667   -2.2868868345
      8  C         1.9223346022    1.2056778856   -0.2061911153
      9  N        -1.2342177620   -0.2810514259    1.1766317879
     10  C        -2.1521523398   -0.0014989883    0.2109908269
     11  N        -1.8063088270    0.6919383399   -0.8436340231
     12  C        -0.5480853881    1.1404912087   -0.9818334839
     13  C         0.4747766856    0.7946909828   -0.0374152208
     14  C         0.0589814909    0.0947736944    1.0555086615
     15  C        -3.5601072353   -0.4269933073    0.4217310201
     16  C        -4.3444729544    0.6489200000    1.2111894298
     17  C        -5.7999512437    0.2373255843    1.4060842080
     18  N        -0.3015713115    1.8479927479   -2.0925966561
     19  H         4.0101728227   -2.6160943271   -1.6997438124
     20  H         5.4563967476   -2.7283032812    0.3118073737
     21  H         5.2299355538   -0.9457969992    2.0700492143
     22  H         3.5531191329    0.8436110864    1.7223426677
     23  H         2.4626827830   -1.4925181389   -3.0334056672
     24  H         2.1107788911    0.2081711607   -2.7450724359
     25  H         1.0785719343   -1.0430922036   -2.0387882187
     26  H         2.0853025532    1.6799576618   -1.1718134194
     27  H         2.2022308170    1.9366837029    0.5522830403
     28  H        -1.5348026768   -0.7929579005    1.9980796893
     29  H         0.7210517529   -0.2086731833    1.8582353748
     30  H        -4.0154598562   -0.5710246103   -0.5588638583
     31  H        -3.5919153291   -1.3814438776    0.9569077211
     32  H        -4.2901678222    1.5950307189    0.6650667692
     33  H        -3.8691916069    0.8114199481    2.1854354574
     34  H        -6.3020317053    0.0990574065    0.4458499753
     35  H        -6.3386338732    1.0095187976    1.9566296040
     36  H        -5.8796153357   -0.6948433154    1.9706927025
     37  H         0.3990973405    2.5722718794   -2.1224009117
     38  H        -1.1162298613    2.0385090232   -2.6632120809
   Point Group: c1    Number of degrees of freedom:   108


   Energy is   -763.966283597

 Hessian updated using BFGS update
  internal optimization (0)

108 Hessian modes will be used to form the next step
  Hessian Eigenvalues:
     0.002208    0.002740    0.003724    0.004381    0.009318    0.010005
     0.014195    0.018522    0.019160    0.019789    0.020481    0.022252
     0.022589    0.022807    0.023913    0.024129    0.025674    0.026580
     0.026732    0.028253    0.028424    0.030212    0.030894    0.036043
     0.038383    0.041311    0.043299    0.043814    0.044309    0.047710
     0.049874    0.052826    0.054087    0.055103    0.064374    0.077475
     0.083835    0.094135    0.121815    0.122294    0.126855    0.128230
     0.131781    0.132969    0.136304    0.140380    0.142360    0.143700
     0.147522    0.148235    0.149801    0.151667    0.152214    0.152536
     0.154125    0.160802    0.190714    0.192823    0.206237    0.216247
     0.224309    0.228795    0.238323    0.247425    0.247635    0.263616
     0.271765    0.275223    0.282334    0.295449    0.298957    0.300149
     0.300479    0.301020    0.301450    0.302477    0.303802    0.304137
```

```
    0.304926    0.305528    0.305848    0.307636    0.308161    0.312704
    0.321692    0.329054    0.331972    0.333125    0.334554    0.337104
    0.342186    0.348972    0.352920    0.362011    0.380801    0.389578
    0.395407    0.405784    0.416969    0.419640    0.420283    0.429780
    0.439354    0.444466    0.453773    0.492780    0.575543    0.784851

 Minimum search - taking simple RFO step
 Searching for Lamda that Minimizes Along All modes
 Value Taken    Lamda =  -0.00246929
 Calculated Step too Large.  Step scaled by  0.658831
 Step Taken.  Stepsize is  0.300000

                           Maximum     Tolerance    Cnvgd?
        Gradient           0.001835     0.000800      NO
        Displacement       0.097267     0.001400      NO
        Energy change     -0.002097     0.000228      NO


 New Cartesian Coordinates Obtained by Inverse Iteration

 Displacement from previous Coordinates is:  0.495961
 ----------------------------------------------------------------
            Standard Nuclear Orientation (Angstroms)
    I     Atom           X                Y                Z
 ----------------------------------------------------------------
    1      N       2.8821952064     0.0822469980    -0.0935469193
    2      C       2.9819400894    -0.8700373288    -1.0646204711
    3      C       3.9156417067    -1.8826452652    -0.8942356746
    4      C       4.7296764530    -1.9247367658     0.2259248421
    5      C       4.6096485447    -0.9308503620     1.1941106914
    6      C       3.6822810443     0.0605545226     1.0040462338
    7      C       2.0977586974    -0.8089019533    -2.2682047393
    8      C       1.9241032472     1.2062973520    -0.2247487024
    9      N      -1.2252892887    -0.3073803322     1.1457845601
   10      C      -2.1537208074     0.0026274206     0.1973069253
   11      N      -1.8169110181     0.7199337735    -0.8404392441
   12      C      -0.5580872359     1.1711136005    -0.9830576217
   13      C       0.4765265665     0.7951122520    -0.0585866804
   14      C       0.0676843717     0.0675937321     1.0164644384
   15      C      -3.5597095398    -0.4266933933     0.4111371514
   16      C      -4.3387854549     0.6426096040     1.2156607595
   17      C      -5.7940035568     0.2315928316     1.4128215072
   18      N      -0.3381847629     1.9228036206    -2.0589871701
   19      H       4.0024913102    -2.6368154733    -1.6657268856
   20      H       5.4575172651    -2.7193339326     0.3412674921
   21      H       5.2331560397    -0.9146224516     2.0782211905
   22      H       3.5542168513     0.8720387703     1.7084889813
   23      H       2.4235051107    -1.5470782148    -2.9983089484
   24      H       2.1221009857     0.1697334610    -2.7525879386
   25      H       1.0601571502    -1.0334201999    -2.0056812698
   26      H       2.0970034848     1.6673887612    -1.1954695076
   27      H       2.2014304877     1.9473282645     0.5248930814
   28      H      -1.5152607765    -0.8414372593     1.9562526689
   29      H       0.7393857912    -0.2605928924     1.8011946338
   30      H      -4.0204575264    -0.5587061565    -0.5687455437
   31      H      -3.5892901512    -1.3866110247     0.9361543008
   32      H      -4.2840901552     1.5933300352     0.6778321020
   33      H      -3.8576768181     0.7938814614     2.1887260220
   34      H      -6.3007103543     0.1039455461     0.4535493377
   35      H      -6.3289390278     0.9991805622     1.9734106200
   36      H      -5.8732100442    -0.7059285762     1.9685271666
   37      H       0.4465130937     2.5470549839    -2.1450147715
   38      H      -1.1506069795     2.1594240284    -2.6138126177
 ----------------------------------------------------------------
 Nuclear Repulsion Energy =        1301.05353526 hartrees
 There are       65 alpha and       65 beta electrons

------------------------------------------------
-  Entering fldman on Sun Oct 13 22:02:52 2024  -
------------------------------------------------
```

```
 Applying Cartesian multipole field
    Component          Value
    ---------          -----
     (2,0,0)        1.00000E-12
     (0,2,0)        2.00000E-11
     (0,0,2)       -3.00000E-11
 Nucleus-field energy     =    -0.0000000055 hartrees

--------------------------------------------------
-  Entering gesman on Sun Oct 13 22:02:52 2024  -
--------------------------------------------------

 Requested basis set is 6-311+G(d,p)
 There are 188 shells and 516 basis functions
 A cutoff of  1.0D-12 yielded  12573 shell pairs
 There are    100340 function pairs (    106960 Cartesian)
 Smallest overlap matrix eigenvalue = 1.51E-06
 Linear dependence detected in AO basis
 Tighter screening thresholds may be required for diffuse basis sets
 Use S2THRESH > 12 and THRESH = 14 in case of SCF convergence issues
 Number of orthogonalized atomic orbitals = 510
 Maximum deviation from orthogonality = 2.515E-11
 Guess MOs from SCF MO coefficient file
 Reading MOs from coefficient file
 Reading MOs from coefficient file

--------------------------------------------------
-  Entering scfman on Sun Oct 13 22:02:52 2024  -
--------------------------------------------------

 Long-range K will be added via erf
 Coulomb attenuation parameter = 0.2 bohr**(-1)
 A restricted hybrid HF-DFT SCF calculation will be
 performed using Pulay DIIS + Geometric Direct Minimization
 Exchange:     0.2220 Hartree-Fock + 1.0000 wB97X-D + LR-HF
 Correlation:  1.0000 wB97X-D
 Using Euler-Maclaurin-Lebedev (75,302) quadrature formula
 Dispersion:   Grimme D
 SCF converges when RMS gradient is below 1.0E-07
 Geometry optimization detected.  Setting ReadMinima to 0
 Setting SaveMinima to 0
 ---------------------------------------
  Cycle       Energy         DIIS Error
 ---------------------------------------
    1    -763.9706636359      5.52E-04
    2    -763.9670746940      7.92E-05
    3    -763.9673760400      5.78E-05
    4    -763.9674753466      2.41E-05
    5    -763.9674982345      4.84E-06
    6    -763.9674994617      2.20E-06
    7    -763.9674996784      7.11E-07
    8    -763.9674997070      2.94E-07
    9    -763.9674997111      9.52E-08 Convergence criterion met
 ---------------------------------------
 SCF time:  CPU 236.02 s  wall 379.19 s
 SCF   energy in the final basis set = -763.96749971
 Total energy in the final basis set = -763.96749971

--------------------------------------------------
-  Entering anlman on Sun Oct 13 22:09:11 2024  -
--------------------------------------------------



 --------------------------------------------------------------

                   Orbital Energies (a.u.)
 --------------------------------------------------------------

 Alpha MOs
```

```
 -- Occupied --
-14.8181 -14.7909 -14.7357 -14.7074 -10.6766 -10.6620 -10.6481 -10.6406
-10.6369 -10.6276 -10.6061 -10.5851 -10.5736 -10.5725 -10.5518 -10.5250
-10.4954 -10.4496  -1.4159  -1.4065  -1.3478  -1.2756  -1.2314  -1.2002
 -1.1898  -1.1419  -1.1005  -1.0856  -1.0546  -1.0509  -1.0349  -1.0001
 -0.9911  -0.9535  -0.9422  -0.9112  -0.9064  -0.8936  -0.8827  -0.8645
 -0.8553  -0.8468  -0.8315  -0.8236  -0.8098  -0.7982  -0.7881  -0.7827
 -0.7676  -0.7632  -0.7551  -0.7538  -0.7467  -0.7307  -0.7197  -0.7120
 -0.6810  -0.6680  -0.6582  -0.6422  -0.6314  -0.6227  -0.6178  -0.6151
 -0.6086
 -- Virtual --
 -0.2708  -0.2608  -0.2438  -0.2276  -0.1745  -0.1653  -0.1502  -0.1452
 -0.1336  -0.1231  -0.1179  -0.1152  -0.1110  -0.1041  -0.1000  -0.0952
 -0.0893  -0.0850  -0.0807  -0.0798  -0.0757  -0.0694  -0.0674  -0.0648
 -0.0620  -0.0575  -0.0566  -0.0530  -0.0475  -0.0425  -0.0385  -0.0321
 -0.0310  -0.0254  -0.0234  -0.0233  -0.0164  -0.0156  -0.0108  -0.0081
 -0.0064   0.0015   0.0034   0.0071   0.0092   0.0122   0.0131   0.0168
  0.0212   0.0225   0.0273   0.0276   0.0323   0.0357   0.0381   0.0402
  0.0424   0.0440   0.0532   0.0551   0.0586   0.0599   0.0645   0.0672
  0.0706   0.0715   0.0764   0.0776   0.0824   0.0863   0.0898   0.0925
  0.0959   0.0971   0.1023   0.1091   0.1092   0.1151   0.1157   0.1207
  0.1228   0.1272   0.1287   0.1357   0.1386   0.1404   0.1419   0.1507
  0.1553   0.1567   0.1613   0.1661   0.1710   0.1725   0.1769   0.1820
  0.1850   0.1903   0.1948   0.1951   0.2017   0.2082   0.2133   0.2176
  0.2215   0.2305   0.2570   0.2676   0.2712   0.2785   0.2977   0.3121
  0.3254   0.3295   0.3371   0.3476   0.3487   0.3548   0.3674   0.3709
  0.3781   0.3815   0.3862   0.3940   0.4031   0.4065   0.4187   0.4220
  0.4291   0.4363   0.4453   0.4537   0.4617   0.4640   0.4721   0.4787
  0.4813   0.4909   0.5003   0.5027   0.5086   0.5149   0.5259   0.5273
  0.5291   0.5315   0.5373   0.5404   0.5494   0.5495   0.5523   0.5642
  0.5657   0.5704   0.5802   0.5830   0.5907   0.5934   0.6014   0.6047
  0.6089   0.6203   0.6275   0.6303   0.6391   0.6474   0.6587   0.6614
  0.6637   0.6718   0.6764   0.6852   0.6875   0.6952   0.7004   0.7096
  0.7169   0.7174   0.7348   0.7389   0.7552   0.7665   0.7799   0.7877
  0.7900   0.7973   0.8142   0.8221   0.8369   0.8388   0.8438   0.8587
  0.8627   0.8707   0.8784   0.8865   0.8931   0.9093   0.9227   0.9266
  0.9322   0.9479   0.9651   0.9779   0.9829   0.9938   1.0223   1.0306
  1.0549   1.0803   1.0871   1.1126   1.1308   1.1497   1.1644   1.1719
  1.1965   1.2232   1.2349   1.2424   1.2580   1.2754   1.3026   1.3112
  1.3175   1.3257   1.3347   1.3457   1.3506   1.3651   1.3698   1.3724
  1.3860   1.3896   1.3973   1.4081   1.4130   1.4206   1.4263   1.4282
  1.4459   1.4520   1.4598   1.4662   1.4700   1.4866   1.4920   1.4984
  1.5067   1.5108   1.5156   1.5193   1.5363   1.5470   1.5482   1.5544
  1.5599   1.5634   1.5773   1.5853   1.5943   1.6060   1.6115   1.6167
  1.6252   1.6256   1.6370   1.6453   1.6550   1.6691   1.6771   1.6982
  1.7102   1.7210   1.7349   1.7411   1.7433   1.7523   1.7645   1.7667
  1.7810   1.7879   1.8053   1.8315   1.8396   1.8654   1.8729   1.8928
  1.8997   1.9073   1.9208   1.9423   1.9496   1.9624   1.9753   1.9886
  1.9986   2.0136   2.0262   2.0365   2.0421   2.0503   2.0655   2.0856
  2.0916   2.1017   2.1331   2.1443   2.1615   2.1959   2.2104   2.2200
  2.2331   2.2498   2.2537   2.2539   2.2588   2.2817   2.2872   2.2993
  2.3136   2.3313   2.3403   2.3598   2.3760   2.3842   2.3932   2.4041
  2.4157   2.4310   2.4380   2.4413   2.4490   2.4627   2.4750   2.4847
  2.4998   2.5071   2.5187   2.5234   2.5353   2.5429   2.5500   2.5585
  2.5657   2.5770   2.5831   2.5927   2.5954   2.5982   2.6138   2.6200
  2.6453   2.6475   2.6575   2.6641   2.6708   2.6834   2.6866   2.7072
  2.7125   2.7213   2.7344   2.7397   2.7514   2.7561   2.7711   2.7757
  2.7824   2.8066   2.8149   2.8420   2.8500   2.8576   2.8721   2.8822
  2.8958   2.9254   2.9385   2.9586   3.0371   3.0670   3.1355   3.1567
  3.1648   3.2301   3.2526   3.2658   3.2998   3.3180   3.3635   3.4049
  3.4190   3.4796   3.5241   3.5351   3.5582   3.5938   3.6189   3.6428
  3.6846   3.7173   3.7203   3.7373   3.7399   3.7561   3.7613   3.7677
  3.7865   3.8315   3.8661   3.9027   3.9389   3.9850   4.0493   4.0916
  4.0921   4.1837   4.2941   4.3057   4.5929   4.6670   4.7000   4.7389
  4.9233   5.0356   5.1913  23.5206  23.5826  23.7145  23.7374  23.7392
 23.7755  23.8046  23.8333  23.8409  23.8912  23.9251  23.9643  23.9791
 24.0327  35.4181  35.4615  35.5180  35.6184
 --------------------------------------------------------------

          Ground-State Mulliken Net Atomic Charges
```

| Atom | Charge (a.u.) |
|---|---|
| 1 N | 0.427696 |
| 2 C | 0.105547 |
| 3 C | -0.032084 |
| 4 C | -0.235981 |
| 5 C | -0.280121 |
| 6 C | -0.058517 |
| 7 C | -0.736840 |
| 8 C | -0.187174 |
| 9 N | -0.126527 |
| 10 C | -0.378551 |
| 11 N | 0.063380 |
| 12 C | 0.177750 |
| 13 C | -0.046639 |
| 14 C | 0.158562 |
| 15 C | -0.368650 |
| 16 C | -0.085461 |
| 17 C | -0.608310 |
| 18 N | -0.467951 |
| 19 H | 0.220614 |
| 20 H | 0.236439 |
| 21 H | 0.232515 |
| 22 H | 0.218082 |
| 23 H | 0.235223 |
| 24 H | 0.215139 |
| 25 H | 0.244968 |
| 26 H | 0.180723 |
| 27 H | 0.248546 |
| 28 H | 0.392800 |
| 29 H | 0.211359 |
| 30 H | 0.240346 |
| 31 H | 0.205805 |
| 32 H | 0.198715 |
| 33 H | 0.170738 |
| 34 H | 0.177490 |
| 35 H | 0.198164 |
| 36 H | 0.169374 |
| 37 H | 0.314780 |
| 38 H | 0.368049 |

Sum of atomic charges = 2.000000

## Cartesian Multipole Moments

Charge (ESU x 10^10)

| | | | | | |
|---|---|---|---|---|---|
| | 9.6064 | | | | |

Dipole Moment (Debye)

| | | | | | |
|---|---|---|---|---|---|
| X | 9.4709 | Y | -0.1350 | Z | 0.5304 |
| Tot | 9.4867 | | | | |

Quadrupole Moments (Debye-Ang)

| | | | | | |
|---|---|---|---|---|---|
| XX | -0.6765 | XY | -15.3585 | YY | -76.8233 |
| XZ | -5.3335 | YZ | -7.7080 | ZZ | -72.3143 |

Traceless Quadrupole Moments (Debye-Ang)

| | | | | | |
|---|---|---|---|---|---|
| QXX | 147.7845 | QYY | -80.6557 | QZZ | -67.1288 |
| QXY | -46.0756 | QXZ | -16.0005 | QYZ | -23.1241 |

Octopole Moments (Debye-Ang^2)

| | | | | | |
|---|---|---|---|---|---|
| XXX | 54.6822 | XXY | -92.3482 | XYY | 25.4524 |
| YYY | 6.4364 | XXZ | 73.4050 | XYZ | 14.5202 |
| YYZ | -29.0233 | XZZ | -4.7234 | YZZ | 9.4358 |
| ZZZ | -6.9958 | | | | |

Traceless Octopole Moments (Debye-Ang^2)

| | | | | | |
|---|---|---|---|---|---|
| XXX | 141.5325 | YYY | 784.8297 | ZZZ | -441.4103 |
| XXY | -1155.7951 | XXZ | 988.9174 | XYY | 155.5525 |
| XYZ | 217.8026 | XZZ | -297.0850 | YYZ | -547.5071 |
| YZZ | 370.9654 | | | | |

Hexadecapole Moments (Debye-Ang^3)

| | | | | | |
|---|---|---|---|---|---|
| XXXX | -4189.5431 | XXXY | -101.7654 | XXYY | -885.6992 |

```
      XYYY     338.7448   YYYY    -660.4103   XXXZ     376.7043
      XXYZ      32.6235   XYYZ      77.4584   YYYZ      -7.2434
      XXZZ    -986.7690   XYZZ     105.8717   YYZZ    -192.7621
      XZZZ     309.4385   YZZZ      -9.8496   ZZZZ    -832.3932
    Traceless Hexadecapole Moments (Debye-Ang^3)
      XXXX   17363.7249   XXXY  -26113.6671   XXXZ    5191.8940
      XXYY   -5423.5953   XXYZ    3179.7647   XXZZ  -11940.1296
      XYYY   20139.9023   XYYZ   -3320.8874   XYZZ    5973.7648
      XZZZ   -1871.0066   YYYY   -1159.8986   YYYZ   -1497.6604
      YYZZ    6583.4939   YZZZ   -1682.1043   ZZZZ    5356.6357
 -----------------------------------------------------------------

--------------------------------------------------
-  Entering drvman on Sun Oct 13 22:09:11 2024  -
--------------------------------------------------

 Calculating analytic gradient of the SCF energy
 Gradient of SCF Energy
           1           2           3           4           5           6
    1   0.0002029   0.0000870  -0.0000369   0.0000667  -0.0000129  -0.0000323
    2  -0.0002341  -0.0000065  -0.0000926  -0.0003102   0.0000121   0.0001285
    3  -0.0003214  -0.0001785   0.0003037  -0.0002376   0.0001242   0.0000809
           7           8           9          10          11          12
    1   0.0001172   0.0000635   0.0001570  -0.0004487   0.0002595  -0.0010567
    2   0.0002566   0.0000567   0.0001580   0.0000832  -0.0003275   0.0028017
    3  -0.0000523   0.0001077  -0.0000493  -0.0007274   0.0006272  -0.0001038
          13          14          15          16          17          18
    1   0.0003261   0.0000737   0.0001879   0.0000365  -0.0000829   0.0023827
    2  -0.0009098   0.0003288  -0.0000734   0.0001021  -0.0000152  -0.0043707
    3  -0.0001067   0.0000005  -0.0001827   0.0001496   0.0000246  -0.0014813
          19          20          21          22          23          24
    1  -0.0000317  -0.0000899  -0.0000432  -0.0000271   0.0000430  -0.0005172
    2   0.0001081   0.0000347   0.0000215  -0.0000060   0.0000943   0.0002463
    3  -0.0000013  -0.0000394  -0.0000397  -0.0001012   0.0000417   0.0002126
          25          26          27          28          29          30
    1  -0.0000843  -0.0003147   0.0001889   0.0000377  -0.0000412   0.0000983
    2   0.0000214   0.0001065  -0.0003668   0.0000885   0.0000261   0.0000416
    3   0.0000558   0.0001274   0.0001461  -0.0000539   0.0000227   0.0000687
          31          32          33          34          35          36
    1  -0.0000345   0.0000169   0.0000041   0.0000011   0.0000234  -0.0000151
    2  -0.0000522  -0.0000097   0.0000291   0.0000071  -0.0000204   0.0000194
    3  -0.0000303   0.0000222   0.0000194   0.0000244  -0.0000319  -0.0000196
          37          38
    1  -0.0018668   0.0003617
    2   0.0014091   0.0006139
    3   0.0005922   0.0010070
 Max gradient component =       4.371E-03
 RMS gradient           =       6.439E-04
 Gradient time:  CPU 111.81 s  wall 178.52 s

--------------------------------------------------
-  Entering optman on Sun Oct 13 22:12:10 2024  -
--------------------------------------------------

 Geometry Optimization Parameters
   NAtoms,    NIC,     NZ,  NCons,   NDum,   NFix, NCnnct, MaxDiis
       38     272       0       0       0       0       0       0

 Cartesian Hessian Update
 Hessian updated using BFGS update


** GEOMETRY OPTIMIZATION IN DELOCALIZED INTERNAL COORDINATES **
   Searching for a Minimum

   Optimization Cycle:   6

                    Coordinates (Angstroms)
     ATOM                X               Y               Z
      1  N          2.8821952064    0.0822469980   -0.0935469193
```

```
     2  C         2.9819400894   -0.8700373288   -1.0646204711
     3  C         3.9156417067   -1.8826452652   -0.8942356746
     4  C         4.7296764530   -1.9247367658    0.2259248421
     5  C         4.6096485447   -0.9308503620    1.1941106914
     6  C         3.6822810443    0.0605545226    1.0040462338
     7  C         2.0977586974   -0.8089019533   -2.2682047393
     8  C         1.9241032472    1.2062973520   -0.2247487024
     9  N        -1.2252892887   -0.3073803322    1.1457845601
    10  C        -2.1537208074    0.0026274206    0.1973069253
    11  N        -1.8169110181    0.7199337735   -0.8404392441
    12  C        -0.5580872359    1.1711136005   -0.9830576217
    13  C         0.4765265665    0.7951122520   -0.0585866804
    14  C         0.0676843717    0.0675937321    1.0164644384
    15  C        -3.5597095398   -0.4266933933    0.4111371514
    16  C        -4.3387854549    0.6426096040    1.2156607595
    17  C        -5.7940035568    0.2315928316    1.4128215072
    18  N        -0.3381847629    1.9228036206   -2.0589871701
    19  H         4.0024913102   -2.6368154733   -1.6657268856
    20  H         5.4575172651   -2.7193339326    0.3412674921
    21  H         5.2331560397   -0.9146224516    2.0782211905
    22  H         3.5542168513    0.8720387703    1.7084889813
    23  H         2.4235051107   -1.5470782148   -2.9983089484
    24  H         2.1221009857    0.1697334610   -2.7525879386
    25  H         1.0601571502   -1.0334201999   -2.0056812698
    26  H         2.0970034848    1.6673887612   -1.1954695076
    27  H         2.2014304877    1.9473282645    0.5248930814
    28  H        -1.5152607765   -0.8414372593    1.9562526689
    29  H         0.7393857912   -0.2605928924    1.8011946338
    30  H        -4.0204575264   -0.5587061565   -0.5687455437
    31  H        -3.5892901512   -1.3866110247    0.9361543008
    32  H        -4.2840901552    1.5933300352    0.6778321020
    33  H        -3.8576768181    0.7938814614    2.1887260220
    34  H        -6.3007103543    0.1039455461    0.4535493377
    35  H        -6.3289390278    0.9991805622    1.9734106200
    36  H        -5.8732100442   -0.7059285762    1.9685271666
    37  H         0.4465130937    2.5470549839   -2.1450147715
    38  H        -1.1506069795    2.1594240284   -2.6138126177
   Point Group: c1    Number of degrees of freedom:   108


   Energy is   -763.967499711

 Hessian updated using BFGS update
  internal optimization (0)

108 Hessian modes will be used to form the next step
  Hessian Eigenvalues:
     0.002131    0.002737    0.003716    0.004217    0.009235    0.009919
     0.014799    0.018554    0.019132    0.019793    0.020486    0.022295
     0.022590    0.022816    0.023569    0.024132    0.025549    0.026062
     0.026640    0.028246    0.028455    0.030184    0.030801    0.036041
     0.038383    0.041330    0.043299    0.043814    0.044306    0.047662
     0.049363    0.052713    0.054105    0.055109    0.064599    0.077511
     0.083835    0.094232    0.121823    0.122294    0.126855    0.128230
     0.131804    0.132971    0.136214    0.140644    0.142564    0.143703
     0.147525    0.148260    0.149825    0.151667    0.152215    0.152541
     0.154326    0.160813    0.190700    0.193071    0.206237    0.216363
     0.225553    0.228789    0.238313    0.247422    0.247780    0.263628
     0.271771    0.275237    0.282574    0.295448    0.298946    0.300150
     0.300488    0.301029    0.301440    0.302488    0.303762    0.304137
     0.304924    0.305525    0.305826    0.307629    0.308174    0.312691
     0.321643    0.329184    0.332021    0.333106    0.334563    0.337105
     0.342199    0.348814    0.353734    0.362376    0.380959    0.389593
     0.395561    0.406002    0.417143    0.419891    0.420181    0.435187
     0.440416    0.447092    0.455213    0.494281    0.575578    0.790341

 Minimum search - taking simple RFO step
 Searching for Lamda that Minimizes Along All modes
 Value Taken    Lamda =  -0.00107314
 Calculated Step too Large.  Step scaled by  0.767789
```

```
 Step Taken.  Stepsize is  0.300000

                             Maximum     Tolerance    Cnvgd?
         Gradient           0.002602      0.000800      NO
         Displacement       0.125058      0.001400      NO
         Energy change     -0.001216      0.000228      NO


 New Cartesian Coordinates Obtained by Inverse Iteration

 Displacement from previous Coordinates is:  0.573640
 ----------------------------------------------------------------
             Standard Nuclear Orientation (Angstroms)
    I     Atom           X                Y                Z
 ----------------------------------------------------------------
    1      N       2.8805772277     0.0807698812    -0.0995309863
    2      C       2.9870738881    -0.8850479211    -1.0568658956
    3      C       3.9238053834    -1.8909312302    -0.8691178342
    4      C       4.7313576032    -1.9148785392     0.2566505593
    5      C       4.6020285965    -0.9100241378     1.2119941933
    6      C       3.6728778343     0.0757898266     1.0036338964
    7      C       2.1122566052    -0.8353219597    -2.2680192489
    8      C       1.9213768658     1.1995138161    -0.2555441190
    9      N      -1.2170472082    -0.3449386957     1.1107778104
   10      C      -2.1593866909     0.0060997498     0.1893451408
   11      N      -1.8345576365     0.7557814054    -0.8285748163
   12      C      -0.5737046577     1.2026479411    -0.9797457474
   13      C       0.4742823410     0.7871030345    -0.0847116701
   14      C       0.0765887247     0.0252130592     0.9704698374
   15      C      -3.5646344157    -0.4226303993     0.4100265139
   16      C      -4.3374912370     0.6423814412     1.2259583629
   17      C      -5.7923852580     0.2325433626     1.4275697288
   18      N      -0.3745685130     2.0062632498    -2.0171151856
   19      H       4.0183971122    -2.6545026014    -1.6303800741
   20      H       5.4618385238    -2.7050330836     0.3859677274
   21      H       5.2192868253    -0.8812364911     2.1001176989
   22      H       3.5362246905     0.8959107400     1.6964749851
   23      H       2.4042465702    -1.6191433557    -2.9640353538
   24      H       2.1922389659     0.1199206854    -2.7926349532
   25      H       1.0631876044    -0.9960644736    -2.0062911193
   26      H       2.0993844735     1.6380098515    -1.2360521607
   27      H       2.1952910024     1.9581966689     0.4776900146
   28      H      -1.4958395952    -0.9059019236     1.9065550845
   29      H       0.7596655095    -0.3343745355     1.7311790073
   30      H      -4.0317920679    -0.5466531611    -0.5680303813
   31      H      -3.5927881659    -1.3860317690     0.9283964495
   32      H      -4.2833240015     1.5963087262     0.6939261997
   33      H      -3.8504402875     0.7856112272     2.1971694521
   34      H      -6.3039618865     0.1123331409     0.4698889287
   35      H      -6.3235323248     0.9969361537     1.9960924437
   36      H      -5.8705959210    -0.7087054796     1.9771142714
   37      H       0.4601872720     2.5507294394    -2.1516706147
   38      H      -1.1861237525     2.2733563565    -2.5586781457
 ----------------------------------------------------------------
 Nuclear Repulsion Energy =        1300.41883994 hartrees
 There are       65 alpha and       65 beta electrons

--------------------------------------------------
-  Entering fldman on Sun Oct 13 22:12:10 2024  -
--------------------------------------------------

 Applying Cartesian multipole field
    Component          Value
    ---------          -----
     (2,0,0)       1.00000E-12
     (0,2,0)       2.00000E-11
     (0,0,2)      -3.00000E-11
 Nucleus-field energy     =    -0.0000000050 hartrees

--------------------------------------------------
```

```
-  Entering gesman on Sun Oct 13 22:12:10 2024  -
--------------------------------------------------

 Requested basis set is 6-311+G(d,p)
 There are 188 shells and 516 basis functions
 A cutoff of  1.0D-12 yielded  12566 shell pairs
 There are    100275 function pairs (    106892 Cartesian)
 Smallest overlap matrix eigenvalue = 1.49E-06
 Linear dependence detected in AO basis
 Tighter screening thresholds may be required for diffuse basis sets
 Use S2THRESH > 12 and THRESH = 14 in case of SCF convergence issues
 Number of orthogonalized atomic orbitals = 510
 Maximum deviation from orthogonality = 1.778E-11
 Guess MOs from SCF MO coefficient file
 Reading MOs from coefficient file
 Reading MOs from coefficient file

--------------------------------------------------
-  Entering scfman on Sun Oct 13 22:12:10 2024  -
--------------------------------------------------

 Long-range K will be added via erf
 Coulomb attenuation parameter = 0.2 bohr**(-1)
 A restricted hybrid HF-DFT SCF calculation will be
 performed using Pulay DIIS + Geometric Direct Minimization
 Exchange:     0.2220 Hartree-Fock + 1.0000 wB97X-D + LR-HF
 Correlation:  1.0000 wB97X-D
 Using Euler-Maclaurin-Lebedev (75,302) quadrature formula
 Dispersion:   Grimme D
 SCF converges when RMS gradient is below 1.0E-07
 Geometry optimization detected.  Setting ReadMinima to 0
 Setting SaveMinima to 0
 ---------------------------------------
  Cycle       Energy         DIIS Error
 ---------------------------------------
    1    -763.9705313518      6.17E-04
    2    -763.9676168659      7.88E-05
    3    -763.9679899451      5.35E-05
    4    -763.9680702692      2.58E-05
    5    -763.9680961988      4.29E-06
    6    -763.9680971785      1.98E-06
    7    -763.9680973479      5.88E-07
    8    -763.9680973701      2.62E-07
    9    -763.9680973733      8.20E-08 Convergence criterion met
 ---------------------------------------
 SCF time:  CPU 236.91 s  wall 377.26 s
 SCF   energy in the final basis set = -763.96809737
 Total energy in the final basis set = -763.96809737

--------------------------------------------------
-  Entering anlman on Sun Oct 13 22:18:27 2024  -
--------------------------------------------------



 --------------------------------------------------------------

                   Orbital Energies (a.u.)
 --------------------------------------------------------------

 Alpha MOs
 -- Occupied --
-14.8187 -14.7894 -14.7370 -14.7062 -10.6766 -10.6610 -10.6471 -10.6412
-10.6374 -10.6283 -10.6060 -10.5857 -10.5742 -10.5732 -10.5527 -10.5242
-10.4943 -10.4489  -1.4148  -1.4070  -1.3477  -1.2750  -1.2317  -1.2000
 -1.1906  -1.1414  -1.1003  -1.0862  -1.0542  -1.0504  -1.0355  -0.9995
 -0.9915  -0.9537  -0.9420  -0.9119  -0.9068  -0.8931  -0.8834  -0.8640
 -0.8561  -0.8473  -0.8309  -0.8241  -0.8097  -0.7987  -0.7886  -0.7834
 -0.7669  -0.7623  -0.7559  -0.7542  -0.7477  -0.7313  -0.7203  -0.7113
 -0.6803  -0.6672  -0.6583  -0.6415  -0.6305  -0.6235  -0.6168  -0.6142
 -0.6073
```

```
-- Virtual --
-0.2715  -0.2593  -0.2421  -0.2279  -0.1745  -0.1657  -0.1501  -0.1451
-0.1339  -0.1227  -0.1179  -0.1150  -0.1111  -0.1043  -0.1003  -0.0959
-0.0897  -0.0850  -0.0803  -0.0794  -0.0756  -0.0694  -0.0676  -0.0654
-0.0619  -0.0577  -0.0564  -0.0534  -0.0474  -0.0427  -0.0400  -0.0326
-0.0303  -0.0251  -0.0238  -0.0235  -0.0163  -0.0152  -0.0112  -0.0077
-0.0066   0.0015   0.0034   0.0064   0.0084   0.0123   0.0130   0.0165
 0.0214   0.0230   0.0272   0.0283   0.0327   0.0360   0.0381   0.0406
 0.0437   0.0438   0.0528   0.0552   0.0591   0.0600   0.0648   0.0673
 0.0707   0.0712   0.0768   0.0768   0.0843   0.0860   0.0901   0.0925
 0.0972   0.0990   0.1036   0.1074   0.1089   0.1141   0.1159   0.1204
 0.1249   0.1265   0.1277   0.1375   0.1388   0.1418   0.1427   0.1505
 0.1556   0.1572   0.1603   0.1661   0.1721   0.1733   0.1773   0.1819
 0.1864   0.1898   0.1950   0.1976   0.2033   0.2089   0.2134   0.2212
 0.2251   0.2316   0.2561   0.2672   0.2738   0.2787   0.2990   0.3125
 0.3257   0.3301   0.3381   0.3477   0.3488   0.3559   0.3692   0.3714
 0.3799   0.3818   0.3856   0.3953   0.4043   0.4059   0.4186   0.4254
 0.4298   0.4364   0.4452   0.4553   0.4611   0.4644   0.4720   0.4785
 0.4802   0.4903   0.4982   0.5042   0.5096   0.5164   0.5253   0.5265
 0.5298   0.5331   0.5374   0.5411   0.5464   0.5483   0.5546   0.5651
 0.5656   0.5687   0.5801   0.5843   0.5910   0.5938   0.6015   0.6036
 0.6137   0.6248   0.6262   0.6333   0.6429   0.6502   0.6591   0.6604
 0.6654   0.6734   0.6770   0.6843   0.6894   0.6932   0.7018   0.7146
 0.7158   0.7220   0.7349   0.7408   0.7547   0.7651   0.7815   0.7882
 0.7903   0.7992   0.8150   0.8246   0.8358   0.8406   0.8452   0.8613
 0.8626   0.8716   0.8811   0.8892   0.8946   0.9106   0.9246   0.9317
 0.9357   0.9524   0.9686   0.9791   0.9852   0.9995   1.0239   1.0326
 1.0544   1.0694   1.0834   1.1161   1.1316   1.1494   1.1648   1.1716
 1.1956   1.2145   1.2289   1.2361   1.2602   1.2775   1.3004   1.3104
 1.3175   1.3220   1.3334   1.3459   1.3512   1.3625   1.3682   1.3723
 1.3837   1.3867   1.3934   1.4098   1.4144   1.4233   1.4268   1.4291
 1.4465   1.4543   1.4590   1.4635   1.4711   1.4871   1.4920   1.4996
 1.5084   1.5110   1.5161   1.5197   1.5381   1.5442   1.5491   1.5544
 1.5611   1.5636   1.5793   1.5887   1.5910   1.6066   1.6098   1.6126
 1.6234   1.6260   1.6361   1.6424   1.6539   1.6628   1.6791   1.6996
 1.7072   1.7188   1.7328   1.7403   1.7432   1.7531   1.7650   1.7668
 1.7824   1.7876   1.8057   1.8328   1.8397   1.8661   1.8734   1.8895
 1.9011   1.9088   1.9210   1.9461   1.9560   1.9696   1.9798   1.9908
 2.0017   2.0148   2.0277   2.0321   2.0415   2.0523   2.0671   2.0855
 2.0907   2.1065   2.1327   2.1472   2.1623   2.1959   2.2148   2.2199
 2.2347   2.2496   2.2528   2.2554   2.2586   2.2753   2.2868   2.2951
 2.3119   2.3284   2.3369   2.3574   2.3762   2.3784   2.3935   2.4040
 2.4170   2.4301   2.4380   2.4421   2.4481   2.4622   2.4795   2.4857
 2.5013   2.5076   2.5140   2.5213   2.5310   2.5426   2.5497   2.5598
 2.5680   2.5754   2.5826   2.5927   2.5950   2.5986   2.6100   2.6206
 2.6442   2.6497   2.6627   2.6670   2.6723   2.6875   2.6906   2.7068
 2.7120   2.7239   2.7343   2.7388   2.7549   2.7624   2.7702   2.7783
 2.7904   2.8091   2.8149   2.8425   2.8513   2.8649   2.8753   2.8817
 2.8974   2.9294   2.9374   2.9660   3.0385   3.0657   3.1341   3.1591
 3.1670   3.2326   3.2540   3.2724   3.3041   3.3186   3.3646   3.4087
 3.4209   3.4796   3.5261   3.5345   3.5589   3.5956   3.6164   3.6419
 3.6845   3.7140   3.7211   3.7379   3.7407   3.7537   3.7596   3.7656
 3.7695   3.8310   3.8656   3.9014   3.9387   3.9839   4.0503   4.0902
 4.0922   4.1847   4.2932   4.3054   4.5938   4.6687   4.6984   4.7448
 4.9278   5.0354   5.1942  23.5200  23.5845  23.7147  23.7363  23.7399
23.7768  23.8061  23.8333  23.8416  23.8914  23.9260  23.9641  23.9792
24.0323  35.4169  35.4638  35.5213  35.6217
--------------------------------------------------------------

          Ground-State Mulliken Net Atomic Charges

     Atom                 Charge (a.u.)
  ----------------------------------------
      1 N                     0.416918
      2 C                     0.133055
      3 C                    -0.030612
      4 C                    -0.234660
      5 C                    -0.280983
      6 C                    -0.047724
      7 C                    -0.763104
```

```
    8  C                   -0.171909
    9  N                   -0.122798
   10  C                   -0.395524
   11  N                    0.054817
   12  C                    0.205262
   13  C                   -0.061204
   14  C                    0.150179
   15  C                   -0.361100
   16  C                   -0.087525
   17  C                   -0.609038
   18  N                   -0.478894
   19  H                    0.220719
   20  H                    0.236692
   21  H                    0.232836
   22  H                    0.218958
   23  H                    0.234969
   24  H                    0.221200
   25  H                    0.243582
   26  H                    0.175276
   27  H                    0.249796
   28  H                    0.390669
   29  H                    0.210457
   30  H                    0.240506
   31  H                    0.202375
   32  H                    0.198909
   33  H                    0.171156
   34  H                    0.177068
   35  H                    0.198004
   36  H                    0.169308
   37  H                    0.317097
   38  H                    0.375272
 ----------------------------------------
 Sum of atomic charges =     2.000000

-----------------------------------------------------------------
                    Cartesian Multipole Moments
-----------------------------------------------------------------
   Charge (ESU x 10^10)
                9.6064
   Dipole Moment (Debye)
        X       9.6209      Y      -0.1945      Z       0.3226
      Tot       9.6283
   Quadrupole Moments (Debye-Ang)
       XX      -0.6913     XY     -15.3643     YY     -76.3626
       XZ      -5.1852     YZ      -8.2252     ZZ     -72.5936
   Traceless Quadrupole Moments (Debye-Ang)
      QXX     147.5735    QYY     -79.4403    QZZ     -68.1332
      QXY     -46.0930    QXZ     -15.5555    QYZ     -24.6756
   Octopole Moments (Debye-Ang^2)
      XXX      57.0944    XXY     -91.9113    XYY      25.1933
      YYY       5.8859    XXZ      73.7666    XYZ      14.3224
      YYZ     -29.8105    XZZ      -3.4476    YZZ       9.7497
      ZZZ      -8.4799
   Traceless Octopole Moments (Debye-Ang^2)
      XXX     146.8543    YYY     774.7703    ZZZ    -446.4842
      XXY   -1149.8424    XXZ    1000.0699    XYY     141.3797
      XYZ     214.8364    XZZ    -288.2339    YYZ    -553.5856
      YZZ     375.0721
   Hexadecapole Moments (Debye-Ang^3)
     XXXX   -4200.8368   XXXY     -95.8859   XXYY    -888.1331
     XYYY     339.4023   YYYY    -674.4737   XXXZ     379.7018
     XXYZ      31.0440   XYYZ      78.3970   YYYZ      -6.3920
     XXZZ    -984.1783   XYZZ     106.9525   YYZZ    -189.5560
     XZZZ     306.6812   YZZZ      -7.2114   ZZZZ    -827.0816
   Traceless Hexadecapole Moments (Debye-Ang^3)
     XXXX   17060.3332   XXXY  -25839.1210   XXXZ    5453.5854
     XXYY   -5352.6957   XXYZ    2994.8405   XXZZ  -11707.6375
     XYYY   19866.1450   XYYZ   -3240.0154   XYZZ    5972.9760
     XZZZ   -2213.5700   YYYY   -1560.2266   YYYZ   -1465.5004
     YYZZ    6912.9223   YZZZ   -1529.3400   ZZZZ    4794.7151
```

```
 ------------------------------------------------------------------

------------------------------------------------
-  Entering drvman on Sun Oct 13 22:18:27 2024  -
------------------------------------------------

 Calculating analytic gradient of the SCF energy
 Gradient of SCF Energy
           1           2           3           4           5           6
    1   0.0003662  -0.0000812   0.0000482  -0.0001175  -0.0001071   0.0001856
    2  -0.0001035  -0.0003572   0.0000459  -0.0000393   0.0002376  -0.0002576
    3  -0.0004124   0.0000531   0.0001251  -0.0001119   0.0001114   0.0001080
           7           8           9          10          11          12
    1   0.0000469   0.0000117   0.0002443  -0.0010289   0.0000268  -0.0001402
    2   0.0004737   0.0002768  -0.0001658   0.0002262  -0.0006441   0.0020705
    3   0.0000203   0.0003537   0.0006907  -0.0010325   0.0004392  -0.0015682
          13          14          15          16          17          18
    1   0.0008821  -0.0001991   0.0001934  -0.0000341  -0.0000682   0.0004271
    2  -0.0002897   0.0000755  -0.0002797   0.0001524  -0.0000180  -0.0024848
    3   0.0000111   0.0003127  -0.0001219   0.0002233   0.0000129  -0.0006480
          19          20          21          22          23          24
    1  -0.0000206  -0.0000657  -0.0000852  -0.0000913  -0.0001742  -0.0004325
    2   0.0001416  -0.0000103   0.0000468   0.0000228   0.0000075   0.0001957
    3  -0.0000419  -0.0000364  -0.0000353  -0.0001300   0.0000034   0.0002658
          25          26          27          28          29          30
    1  -0.0000268   0.0001770   0.0002311   0.0001616   0.0000218   0.0001603
    2  -0.0000917  -0.0000314  -0.0003661   0.0001239   0.0000547   0.0000855
    3   0.0000222   0.0002438   0.0000721  -0.0000620  -0.0000609   0.0000546
          31          32          33          34          35          36
    1  -0.0000373   0.0000492   0.0000283   0.0000050  -0.0000023  -0.0000153
    2  -0.0000067  -0.0000387   0.0000010   0.0000096  -0.0000227   0.0000165
    3   0.0000173  -0.0000115  -0.0000004   0.0000192  -0.0000336  -0.0000229
          37          38
    1  -0.0008186   0.0002794
    2   0.0002859   0.0006568
    3   0.0003832   0.0007865
 Max gradient component =       2.485E-03
 RMS gradient           =       4.397E-04
 Gradient time:  CPU 112.03 s  wall 178.77 s

------------------------------------------------
-  Entering optman on Sun Oct 13 22:21:26 2024  -
------------------------------------------------

 Geometry Optimization Parameters
   NAtoms,    NIC,     NZ,  NCons,   NDum,   NFix, NCnnct, MaxDiis
       38     272       0       0       0       0       0       0

 Cartesian Hessian Update
 Hessian updated using BFGS update


** GEOMETRY OPTIMIZATION IN DELOCALIZED INTERNAL COORDINATES **
   Searching for a Minimum

   Optimization Cycle:   7

                      Coordinates (Angstroms)
     ATOM                X               Y               Z
      1  N         2.8805772277    0.0807698812   -0.0995309863
      2  C         2.9870738881   -0.8850479211   -1.0568658956
      3  C         3.9238053834   -1.8909312302   -0.8691178342
      4  C         4.7313576032   -1.9148785392    0.2566505593
      5  C         4.6020285965   -0.9100241378    1.2119941933
      6  C         3.6728778343    0.0757898266    1.0036338964
      7  C         2.1122566052   -0.8353219597   -2.2680192489
      8  C         1.9213768658    1.1995138161   -0.2555441190
      9  N        -1.2170472082   -0.3449386957    1.1107778104
     10  C        -2.1593866909    0.0060997498    0.1893451408
     11  N        -1.8345576365    0.7557814054   -0.8285748163
```

```
    12  C        -0.5737046577    1.2026479411   -0.9797457474
    13  C         0.4742823410    0.7871030345   -0.0847116701
    14  C         0.0765887247    0.0252130592    0.9704698374
    15  C        -3.5646344157   -0.4226303993    0.4100265139
    16  C        -4.3374912370    0.6423814412    1.2259583629
    17  C        -5.7923852580    0.2325433626    1.4275697288
    18  N        -0.3745685130    2.0062632498   -2.0171151856
    19  H         4.0183971122   -2.6545026014   -1.6303800741
    20  H         5.4618385238   -2.7050330836    0.3859677274
    21  H         5.2192868253   -0.8812364911    2.1001176989
    22  H         3.5362246905    0.8959107400    1.6964749851
    23  H         2.4042465702   -1.6191433557   -2.9640353538
    24  H         2.1922389659    0.1199206854   -2.7926349532
    25  H         1.0631876044   -0.9960644736   -2.0062911193
    26  H         2.0993844735    1.6380098515   -1.2360521607
    27  H         2.1952910024    1.9581966689    0.4776900146
    28  H        -1.4958395952   -0.9059019236    1.9065550845
    29  H         0.7596655095   -0.3343745355    1.7311790073
    30  H        -4.0317920679   -0.5466531611   -0.5680303813
    31  H        -3.5927881659   -1.3860317690    0.9283964495
    32  H        -4.2833240015    1.5963087262    0.6939261997
    33  H        -3.8504402875    0.7856112272    2.1971694521
    34  H        -6.3039618865    0.1123331409    0.4698889287
    35  H        -6.3235323248    0.9969361537    1.9960924437
    36  H        -5.8705959210   -0.7087054796    1.9771142714
    37  H         0.4601872720    2.5507294394   -2.1516706147
    38  H        -1.1861237525    2.2733563565   -2.5586781457
   Point Group: c1    Number of degrees of freedom:   108


   Energy is   -763.968097373

 Hessian updated using BFGS update
  internal optimization (0)

108 Hessian modes will be used to form the next step
  Hessian Eigenvalues:
     0.001807    0.002726    0.003599    0.003829    0.008515    0.010448
     0.016053    0.018652    0.019148    0.019841    0.020510    0.022585
     0.022626    0.022935    0.023198    0.024159    0.025401    0.025876
     0.026718    0.028243    0.028479    0.030160    0.030762    0.036038
     0.038382    0.041331    0.043303    0.043815    0.044324    0.047548
     0.048769    0.052601    0.054105    0.055268    0.064644    0.077527
     0.083837    0.094480    0.121824    0.122294    0.126855    0.128263
     0.131809    0.132979    0.136267    0.140616    0.143092    0.143979
     0.147526    0.148272    0.149826    0.151667    0.152322    0.152545
     0.154686    0.160711    0.190671    0.193038    0.206264    0.216348
     0.225168    0.228725    0.238380    0.247463    0.247829    0.263692
     0.271771    0.275257    0.282561    0.295524    0.299010    0.300150
     0.300477    0.301027    0.301424    0.302487    0.303718    0.304137
     0.304917    0.305536    0.305819    0.307632    0.308177    0.312685
     0.321584    0.329135    0.332025    0.333077    0.334673    0.337114
     0.342214    0.348990    0.353770    0.362308    0.380965    0.389838
     0.396195    0.405920    0.417435    0.419818    0.420970    0.435347
     0.442163    0.446371    0.454599    0.494283    0.578472    0.779469

 Minimum search - taking simple RFO step
 Searching for Lamda that Minimizes Along All modes
 Value Taken    Lamda =  -0.00062058
 Calculated Step too Large.  Step scaled by  0.733115
 Step Taken.  Stepsize is  0.300000

                             Maximum     Tolerance    Cnvgd?
         Gradient           0.002260      0.000800      NO
         Displacement       0.140618      0.001400      NO
         Energy change     -0.000598      0.000228      NO


 New Cartesian Coordinates Obtained by Inverse Iteration
```

```
 Displacement from previous Coordinates is:  0.638366
 ----------------------------------------------------------------
             Standard Nuclear Orientation (Angstroms)
    I     Atom           X                Y                Z
 ----------------------------------------------------------------
    1      N       2.8775799896     0.0783485550    -0.1099079424
    2      C       3.0014127167    -0.8975805008    -1.0553951995
    3      C       3.9403279108    -1.8969398723    -0.8443068631
    4      C       4.7338690486    -1.9040349654     0.2917758846
    5      C       4.5872379083    -0.8893143787     1.2338894792
    6      C       3.6553956318     0.0902696041     1.0033342886
    7      C       2.1479155244    -0.8581445798    -2.2823556726
    8      C       1.9136385438     1.1871484126    -0.2936506043
    9      N      -1.2109380846    -0.3893575692     1.0738887316
   10      C      -2.1672589589     0.0082401315     0.1868960044
   11      N      -1.8552882979     0.7931208367    -0.8075875710
   12      C      -0.5924774526     1.2309831616    -0.9677922017
   13      C       0.4686137665     0.7720749623    -0.1096562433
   14      C       0.0837117992    -0.0262434850     0.9216692406
   15      C      -3.5727698833    -0.4163454846     0.4156815821
   16      C      -4.3382527196     0.6481173851     1.2383805184
   17      C      -5.7927537162     0.2405930388     1.4469164646
   18      N      -0.4076534684     2.0806970775    -1.9688941681
   19      H       4.0487123820    -2.6688616971    -1.5953932728
   20      H       5.4669742333    -2.6886327616     0.4390360697
   21      H       5.1930494776    -0.8480243535     2.1295258799
   22      H       3.5048943733     0.9170279304     1.6855038901
   23      H       2.4055611132    -1.6886652927    -2.9365154205
   24      H       2.2960426586     0.0661285153    -2.8477993943
   25      H       1.0870205271    -0.9438263762    -2.0354330519
   26      H       2.0881235492     1.5976795056    -1.2872549900
   27      H       2.1843456174     1.9688256204     0.4167335223
   28      H      -1.4795551332    -0.9795599174     1.8518154145
   29      H       0.7773829902    -0.4216926530     1.6543774301
   30      H      -4.0466890724    -0.5360281723    -0.5600060593
   31      H      -3.6011459410    -1.3814193513     0.9307370784
   32      H      -4.2850252610     1.6031949124     0.7083921552
   33      H      -3.8451045403     0.7873735842     2.2069913808
   34      H      -6.3097872994     0.1238591623     0.4916730498
   35      H      -6.3193926661     1.0044096418     2.0204375613
   36      H      -5.8699031014    -0.7019855721     1.9944077354
   37      H       0.4524224650     2.5727937766    -2.1403497279
   38      H      -1.2202366305     2.3657711686    -2.4997649789
 ----------------------------------------------------------------
 Nuclear Repulsion Energy =        1299.58024003 hartrees
 There are       65 alpha and       65 beta electrons

------------------------------------------------
-  Entering fldman on Sun Oct 13 22:21:26 2024  -
------------------------------------------------

 Applying Cartesian multipole field
    Component           Value
    ---------           -----
     (2,0,0)        1.00000E-12
     (0,2,0)        2.00000E-11
     (0,0,2)       -3.00000E-11
 Nucleus-field energy     =    -0.0000000045 hartrees

------------------------------------------------
-  Entering gesman on Sun Oct 13 22:21:26 2024  -
------------------------------------------------

 Requested basis set is 6-311+G(d,p)
 There are 188 shells and 516 basis functions
 A cutoff of  1.0D-12 yielded  12567 shell pairs
 There are    100259 function pairs (    106852 Cartesian)
 Smallest overlap matrix eigenvalue = 1.47E-06
 Linear dependence detected in AO basis
 Tighter screening thresholds may be required for diffuse basis sets
```

```
 Use S2THRESH > 12 and THRESH = 14 in case of SCF convergence issues
 Number of orthogonalized atomic orbitals = 510
 Maximum deviation from orthogonality = 1.390E-11
 Guess MOs from SCF MO coefficient file
 Reading MOs from coefficient file
 Reading MOs from coefficient file

-------------------------------------------------
-  Entering scfman on Sun Oct 13 22:21:27 2024  -
-------------------------------------------------

 Long-range K will be added via erf
 Coulomb attenuation parameter = 0.2 bohr**(-1)
 A restricted hybrid HF-DFT SCF calculation will be
 performed using Pulay DIIS + Geometric Direct Minimization
 Exchange:     0.2220 Hartree-Fock + 1.0000 wB97X-D + LR-HF
 Correlation:  1.0000 wB97X-D
 Using Euler-Maclaurin-Lebedev (75,302) quadrature formula
 Dispersion:   Grimme D
 SCF converges when RMS gradient is below 1.0E-07
 Geometry optimization detected.  Setting ReadMinima to 0
 Setting SaveMinima to 0
 ---------------------------------------
  Cycle       Energy         DIIS Error
 ---------------------------------------
    1    -763.9744403369      6.48E-04
    2    -763.9679427346      8.11E-05
    3    -763.9683506357      5.78E-05
    4    -763.9684448954      2.53E-05
    5    -763.9684712227      4.61E-06
    6    -763.9684722313      2.34E-06
    7    -763.9684724508      6.41E-07
    8    -763.9684724781      2.69E-07
    9    -763.9684724817      8.74E-08 Convergence criterion met
 ---------------------------------------
 SCF time:  CPU 237.08 s  wall 378.32 s
 SCF   energy in the final basis set = -763.96847248
 Total energy in the final basis set = -763.96847248

-------------------------------------------------
-  Entering anlman on Sun Oct 13 22:27:45 2024  -
-------------------------------------------------



 --------------------------------------------------------------

                    Orbital Energies (a.u.)
 --------------------------------------------------------------

 Alpha MOs
 -- Occupied --
-14.8192 -14.7887 -14.7374 -14.7055 -10.6763 -10.6602 -10.6464 -10.6417
-10.6378 -10.6290 -10.6059 -10.5862 -10.5748 -10.5737 -10.5530 -10.5237
-10.4932 -10.4482  -1.4145  -1.4075  -1.3475  -1.2747  -1.2320  -1.1999
 -1.1910  -1.1412  -1.1003  -1.0865  -1.0540  -1.0501  -1.0359  -0.9990
 -0.9917  -0.9537  -0.9419  -0.9120  -0.9069  -0.8926  -0.8840  -0.8636
 -0.8566  -0.8475  -0.8311  -0.8244  -0.8094  -0.7989  -0.7887  -0.7843
 -0.7665  -0.7618  -0.7564  -0.7538  -0.7481  -0.7317  -0.7206  -0.7107
 -0.6798  -0.6667  -0.6584  -0.6410  -0.6300  -0.6239  -0.6160  -0.6134
 -0.6066
 -- Virtual --
 -0.2722  -0.2584  -0.2408  -0.2282  -0.1745  -0.1658  -0.1501  -0.1450
 -0.1341  -0.1221  -0.1178  -0.1148  -0.1111  -0.1043  -0.1006  -0.0964
 -0.0900  -0.0851  -0.0799  -0.0789  -0.0756  -0.0696  -0.0678  -0.0660
 -0.0619  -0.0580  -0.0561  -0.0539  -0.0480  -0.0429  -0.0415  -0.0327
 -0.0293  -0.0254  -0.0239  -0.0231  -0.0161  -0.0148  -0.0117  -0.0072
 -0.0067   0.0012   0.0032   0.0060   0.0075   0.0122   0.0132   0.0168
  0.0213   0.0235   0.0272   0.0295   0.0333   0.0366   0.0375   0.0409
  0.0436   0.0450   0.0527   0.0547   0.0592   0.0604   0.0641   0.0667
  0.0707   0.0726   0.0759   0.0773   0.0850   0.0862   0.0909   0.0924
```

| | | | | | | | |
|---|---|---|---|---|---|---|---|
| 0.0984 | 0.1010 | 0.1048 | 0.1060 | 0.1081 | 0.1124 | 0.1166 | 0.1200 |
| 0.1241 | 0.1266 | 0.1287 | 0.1373 | 0.1388 | 0.1434 | 0.1440 | 0.1501 |
| 0.1553 | 0.1581 | 0.1602 | 0.1663 | 0.1722 | 0.1748 | 0.1776 | 0.1808 |
| 0.1859 | 0.1889 | 0.1946 | 0.1998 | 0.2041 | 0.2080 | 0.2138 | 0.2236 |
| 0.2292 | 0.2347 | 0.2549 | 0.2685 | 0.2748 | 0.2824 | 0.3007 | 0.3127 |
| 0.3260 | 0.3305 | 0.3392 | 0.3477 | 0.3484 | 0.3570 | 0.3702 | 0.3727 |
| 0.3803 | 0.3820 | 0.3862 | 0.3955 | 0.4045 | 0.4056 | 0.4183 | 0.4262 |
| 0.4297 | 0.4372 | 0.4439 | 0.4546 | 0.4609 | 0.4658 | 0.4717 | 0.4752 |
| 0.4824 | 0.4885 | 0.4948 | 0.5064 | 0.5094 | 0.5170 | 0.5241 | 0.5258 |
| 0.5309 | 0.5348 | 0.5364 | 0.5399 | 0.5427 | 0.5495 | 0.5560 | 0.5636 |
| 0.5656 | 0.5700 | 0.5790 | 0.5842 | 0.5909 | 0.5945 | 0.5986 | 0.6050 |
| 0.6145 | 0.6253 | 0.6263 | 0.6351 | 0.6446 | 0.6517 | 0.6558 | 0.6604 |
| 0.6665 | 0.6759 | 0.6784 | 0.6853 | 0.6917 | 0.6936 | 0.7025 | 0.7134 |
| 0.7165 | 0.7270 | 0.7356 | 0.7442 | 0.7543 | 0.7653 | 0.7827 | 0.7872 |
| 0.7925 | 0.8022 | 0.8133 | 0.8268 | 0.8346 | 0.8380 | 0.8519 | 0.8618 |
| 0.8641 | 0.8726 | 0.8847 | 0.8895 | 0.8989 | 0.9112 | 0.9252 | 0.9362 |
| 0.9427 | 0.9561 | 0.9695 | 0.9860 | 0.9870 | 1.0084 | 1.0252 | 1.0347 |
| 1.0516 | 1.0548 | 1.0832 | 1.1221 | 1.1307 | 1.1497 | 1.1641 | 1.1706 |
| 1.1963 | 1.2054 | 1.2266 | 1.2301 | 1.2611 | 1.2808 | 1.2984 | 1.3110 |
| 1.3169 | 1.3177 | 1.3328 | 1.3447 | 1.3517 | 1.3565 | 1.3682 | 1.3750 |
| 1.3826 | 1.3852 | 1.3912 | 1.4078 | 1.4157 | 1.4242 | 1.4273 | 1.4311 |
| 1.4473 | 1.4546 | 1.4595 | 1.4601 | 1.4711 | 1.4876 | 1.4923 | 1.5008 |
| 1.5075 | 1.5105 | 1.5164 | 1.5214 | 1.5383 | 1.5407 | 1.5507 | 1.5537 |
| 1.5610 | 1.5648 | 1.5790 | 1.5850 | 1.5926 | 1.6043 | 1.6079 | 1.6133 |
| 1.6234 | 1.6256 | 1.6371 | 1.6415 | 1.6539 | 1.6597 | 1.6828 | 1.6994 |
| 1.7064 | 1.7179 | 1.7301 | 1.7396 | 1.7439 | 1.7545 | 1.7641 | 1.7684 |
| 1.7835 | 1.7883 | 1.8054 | 1.8331 | 1.8389 | 1.8665 | 1.8734 | 1.8848 |
| 1.9021 | 1.9101 | 1.9202 | 1.9481 | 1.9578 | 1.9765 | 1.9828 | 1.9945 |
| 2.0030 | 2.0149 | 2.0272 | 2.0296 | 2.0422 | 2.0549 | 2.0667 | 2.0842 |
| 2.0918 | 2.1116 | 2.1318 | 2.1497 | 2.1634 | 2.1947 | 2.2177 | 2.2190 |
| 2.2365 | 2.2496 | 2.2526 | 2.2576 | 2.2582 | 2.2708 | 2.2875 | 2.2917 |
| 2.3115 | 2.3241 | 2.3341 | 2.3556 | 2.3720 | 2.3788 | 2.3937 | 2.4036 |
| 2.4175 | 2.4302 | 2.4384 | 2.4451 | 2.4479 | 2.4621 | 2.4829 | 2.4872 |
| 2.5007 | 2.5063 | 2.5123 | 2.5229 | 2.5283 | 2.5421 | 2.5499 | 2.5606 |
| 2.5696 | 2.5750 | 2.5834 | 2.5924 | 2.5949 | 2.5990 | 2.6090 | 2.6211 |
| 2.6398 | 2.6496 | 2.6662 | 2.6673 | 2.6730 | 2.6895 | 2.6957 | 2.7058 |
| 2.7144 | 2.7251 | 2.7333 | 2.7353 | 2.7552 | 2.7642 | 2.7713 | 2.7803 |
| 2.8000 | 2.8080 | 2.8179 | 2.8432 | 2.8534 | 2.8693 | 2.8759 | 2.8846 |
| 2.8995 | 2.9294 | 2.9388 | 2.9666 | 3.0397 | 3.0637 | 3.1309 | 3.1591 |
| 3.1649 | 3.2320 | 3.2547 | 3.2727 | 3.3069 | 3.3194 | 3.3666 | 3.4096 |
| 3.4229 | 3.4793 | 3.5275 | 3.5332 | 3.5596 | 3.5971 | 3.6141 | 3.6406 |
| 3.6844 | 3.7091 | 3.7219 | 3.7383 | 3.7404 | 3.7464 | 3.7605 | 3.7621 |
| 3.7686 | 3.8299 | 3.8649 | 3.9007 | 3.9386 | 3.9840 | 4.0514 | 4.0883 |
| 4.0921 | 4.1856 | 4.2923 | 4.3068 | 4.5940 | 4.6698 | 4.6967 | 4.7512 |
| 4.9299 | 5.0344 | 5.1968 | 23.5200 | 23.5859 | 23.7162 | 23.7355 | 23.7412 |
| 23.7787 | 23.8076 | 23.8330 | 23.8418 | 23.8909 | 23.9267 | 23.9622 | 23.9821 |
| 24.0306 | 35.4152 | 35.4651 | 35.5234 | 35.6242 | | | |

Ground-State Mulliken Net Atomic Charges

| Atom | | Charge (a.u.) |
|---|---|---|
| 1 | N | 0.404079 |
| 2 | C | 0.150532 |
| 3 | C | -0.027629 |
| 4 | C | -0.228471 |
| 5 | C | -0.287291 |
| 6 | C | -0.026522 |
| 7 | C | -0.789677 |
| 8 | C | -0.155794 |
| 9 | N | -0.118944 |
| 10 | C | -0.404641 |
| 11 | N | 0.045910 |
| 12 | C | 0.218235 |
| 13 | C | -0.070531 |
| 14 | C | 0.137375 |
| 15 | C | -0.351983 |
| 16 | C | -0.090966 |
| 17 | C | -0.609813 |

```
    18 N                     -0.484385
    19 H                      0.221008
    20 H                      0.236831
    21 H                      0.233193
    22 H                      0.220047
    23 H                      0.234152
    24 H                      0.230638
    25 H                      0.240765
    26 H                      0.171766
    27 H                      0.251603
    28 H                      0.387948
    29 H                      0.209933
    30 H                      0.241133
    31 H                      0.198303
    32 H                      0.199373
    33 H                      0.171615
    34 H                      0.176626
    35 H                      0.197874
    36 H                      0.169243
    37 H                      0.317997
    38 H                      0.380469
 ----------------------------------------
 Sum of atomic charges =      2.000000

 -----------------------------------------------------------------
                    Cartesian Multipole Moments
 -----------------------------------------------------------------
    Charge (ESU x 10^10)
                 9.6064
    Dipole Moment (Debye)
         X       9.7206      Y      -0.2861      Z       0.1515
       Tot       9.7260
    Quadrupole Moments (Debye-Ang)
        XX      -0.6887     XY     -15.4607     YY     -75.8912
        XZ      -5.1379     YZ      -8.6425     ZZ     -72.9126
    Traceless Quadrupole Moments (Debye-Ang)
       QXX     147.4264    QYY     -78.1812    QZZ     -69.2452
       QXY     -46.3821    QXZ     -15.4138    QYZ     -25.9274
    Octopole Moments (Debye-Ang^2)
       XXX      58.7107    XXY     -91.7937    XYY      24.7923
       YYY       5.8100    XXZ      73.6895    XYZ      14.0555
       YYZ     -30.1826    XZZ      -1.9602    YZZ       9.5302
       ZZZ      -9.9419
    Traceless Octopole Moments (Debye-Ang^2)
       XXX     146.7754    YYY     775.2309    ZZZ    -451.2133
       XXY   -1147.5448    XXZ    1004.6476    XYY     127.2564
       XYZ     210.8318    XZZ    -274.0318    YYZ    -553.4342
       YZZ     372.3139
    Hexadecapole Moments (Debye-Ang^3)
      XXXX   -4220.0274   XXXY     -89.9778   XXYY    -891.5836
      XYYY     342.5662   YYYY    -685.8261   XXXZ     383.1716
      XXYZ      29.6985   XYYZ      80.6902   YYYZ      -6.6498
      XXZZ    -981.1118   XYZZ     108.2137   YYZZ    -188.1076
      XZZZ     307.3421   YZZZ      -3.5214   ZZZZ    -824.7558
    Traceless Hexadecapole Moments (Debye-Ang^3)
      XXXX   16572.2341   XXXY  -25683.7632   XXXZ    5528.8441
      XXYY   -5299.3206   XXYZ    2817.6114   XXZZ  -11272.9135
      XYYY   19733.3533   XYYZ   -3095.5908   XYZZ    5950.4099
      XZZZ   -2433.2533   YYYY   -1785.1194   YYYZ   -1600.4185
      YYZZ    7084.4400   YZZZ   -1217.1930   ZZZZ    4188.4735
 -----------------------------------------------------------------

--------------------------------------------------
-  Entering drvman on Sun Oct 13 22:27:45 2024  -
--------------------------------------------------

 Calculating analytic gradient of the SCF energy
 Gradient of SCF Energy
            1            2            3            4            5            6
    1   0.0001109   -0.0001396    0.0002551   -0.0000356    0.0001933   -0.0002109
```

```
    2   0.0000632  -0.0003694   0.0000943  -0.0001134  -0.0002628   0.0002871
    3  -0.0002917  -0.0000170   0.0003232  -0.0000244  -0.0000559   0.0000171
            7           8           9          10          11          12
    1  -0.0000611  -0.0000449   0.0003571  -0.0005624  -0.0001220   0.0002355
    2   0.0002616   0.0001650  -0.0002181   0.0007048  -0.0008450   0.0009685
    3  -0.0000454   0.0003455   0.0007192  -0.0011754   0.0004917  -0.0014245
           13          14          15          16          17          18
    1   0.0006621  -0.0004110   0.0001121  -0.0000104  -0.0000195  -0.0005023
    2  -0.0004173   0.0004775  -0.0002519   0.0001029  -0.0000277  -0.0011993
    3   0.0002509  -0.0002135  -0.0001181   0.0001901  -0.0000237   0.0002163
           19          20          21          22          23          24
    1   0.0000409  -0.0000436  -0.0000287  -0.0000743  -0.0002223  -0.0004244
    2   0.0000530  -0.0000097   0.0000009   0.0000529  -0.0000065   0.0002767
    3  -0.0000435  -0.0000342   0.0000267  -0.0000896  -0.0000172   0.0001354
           25          26          27          28          29          30
    1  -0.0000189   0.0004379   0.0001957   0.0001388   0.0000298   0.0001206
    2   0.0000486   0.0000871  -0.0002986   0.0000784   0.0000718   0.0000633
    3   0.0001731   0.0002454   0.0000395   0.0000081  -0.0000973   0.0000208
           31          32          33          34          35          36
    1  -0.0000070   0.0000227   0.0000269   0.0000056  -0.0000079  -0.0000096
    2  -0.0000010  -0.0000076  -0.0000074   0.0000049  -0.0000047  -0.0000004
    3   0.0000251  -0.0000108  -0.0000005  -0.0000018  -0.0000237  -0.0000200
           37          38
    1  -0.0001181   0.0001295
    2  -0.0002764   0.0004545
    3   0.0001072   0.0003928
 Max gradient component =       1.425E-03
 RMS gradient           =       3.258E-04
 Gradient time:  CPU 109.28 s  wall 181.91 s

-------------------------------------------------
-  Entering optman on Sun Oct 13 22:30:47 2024  -
-------------------------------------------------

 Geometry Optimization Parameters
   NAtoms,    NIC,     NZ,  NCons,   NDum,   NFix, NCnnct, MaxDiis
       38     272       0       0       0       0       0       0

 Cartesian Hessian Update
 Hessian updated using BFGS update


** GEOMETRY OPTIMIZATION IN DELOCALIZED INTERNAL COORDINATES **
   Searching for a Minimum

   Optimization Cycle:   8

                       Coordinates (Angstroms)
     ATOM                X               Y               Z
      1  N         2.8775799896    0.0783485550   -0.1099079424
      2  C         3.0014127167   -0.8975805008   -1.0553951995
      3  C         3.9403279108   -1.8969398723   -0.8443068631
      4  C         4.7338690486   -1.9040349654    0.2917758846
      5  C         4.5872379083   -0.8893143787    1.2338894792
      6  C         3.6553956318    0.0902696041    1.0033342886
      7  C         2.1479155244   -0.8581445798   -2.2823556726
      8  C         1.9136385438    1.1871484126   -0.2936506043
      9  N        -1.2109380846   -0.3893575692    1.0738887316
     10  C        -2.1672589589    0.0082401315    0.1868960044
     11  N        -1.8552882979    0.7931208367   -0.8075875710
     12  C        -0.5924774526    1.2309831616   -0.9677922017
     13  C         0.4686137665    0.7720749623   -0.1096562433
     14  C         0.0837117992   -0.0262434850    0.9216692406
     15  C        -3.5727698833   -0.4163454846    0.4156815821
     16  C        -4.3382527196    0.6481173851    1.2383805184
     17  C        -5.7927537162    0.2405930388    1.4469164646
     18  N        -0.4076534684    2.0806970775   -1.9688941681
     19  H         4.0487123820   -2.6688616971   -1.5953932728
     20  H         5.4669742333   -2.6886327616    0.4390360697
     21  H         5.1930494776   -0.8480243535    2.1295258799
```

```
    22  H         3.5048943733    0.9170279304    1.6855038901
    23  H         2.4055611132   -1.6886652927   -2.9365154205
    24  H         2.2960426586    0.0661285153   -2.8477993943
    25  H         1.0870205271   -0.9438263762   -2.0354330519
    26  H         2.0881235492    1.5976795056   -1.2872549900
    27  H         2.1843456174    1.9688256204    0.4167335223
    28  H        -1.4795551332   -0.9795599174    1.8518154145
    29  H         0.7773829902   -0.4216926530    1.6543774301
    30  H        -4.0466890724   -0.5360281723   -0.5600060593
    31  H        -3.6011459410   -1.3814193513    0.9307370784
    32  H        -4.2850252610    1.6031949124    0.7083921552
    33  H        -3.8451045403    0.7873735842    2.2069913808
    34  H        -6.3097872994    0.1238591623    0.4916730498
    35  H        -6.3193926661    1.0044096418    2.0204375613
    36  H        -5.8699031014   -0.7019855721    1.9944077354
    37  H         0.4524224650    2.5727937766   -2.1403497279
    38  H        -1.2202366305    2.3657711686   -2.4997649789
  Point Group: c1    Number of degrees of freedom:   108


  Energy is   -763.968472482

 Hessian updated using BFGS update
  internal optimization (0)

108 Hessian modes will be used to form the next step
  Hessian Eigenvalues:
     0.001137    0.002700    0.003130    0.003758    0.008103    0.010995
     0.017170    0.019029    0.019152    0.019866    0.020580    0.022579
     0.022654    0.022740    0.023828    0.024322    0.025497    0.025892
     0.027001    0.028240    0.028702    0.030147    0.030833    0.036034
     0.038382    0.041334    0.043300    0.043814    0.044300    0.047303
     0.048262    0.052432    0.054205    0.055185    0.064054    0.077507
     0.083837    0.094338    0.121824    0.122319    0.126855    0.128232
     0.131939    0.132988    0.136536    0.140599    0.143034    0.144178
     0.147528    0.148451    0.149983    0.151665    0.152259    0.152553
     0.155651    0.160863    0.190772    0.193760    0.206292    0.216311
     0.224375    0.228660    0.238635    0.247550    0.247913    0.263728
     0.271782    0.275209    0.282379    0.295480    0.299198    0.300150
     0.300505    0.301048    0.301485    0.302493    0.303892    0.304139
     0.304919    0.305467    0.306069    0.307636    0.308182    0.312615
     0.321528    0.329071    0.332070    0.333142    0.334664    0.337125
     0.342154    0.347616    0.356405    0.363329    0.381378    0.389839
     0.396980    0.406884    0.417733    0.420513    0.421048    0.430447
     0.439449    0.445819    0.454481    0.494192    0.576766    0.760569

 Minimum search - taking simple RFO step
 Searching for Lamda that Minimizes Along All modes
 Value Taken    Lamda =  -0.00066531
 Calculated Step too Large.  Step scaled by  0.556114
 Step Taken.  Stepsize is  0.300000

                          Maximum     Tolerance    Cnvgd?
        Gradient         0.002552      0.000800      NO
        Displacement     0.119804      0.001400      NO
        Energy change   -0.000375      0.000228      NO


 New Cartesian Coordinates Obtained by Inverse Iteration

 Displacement from previous Coordinates is:  0.732651
 ----------------------------------------------------------------
             Standard Nuclear Orientation (Angstroms)
    I     Atom           X                Y                Z
 ----------------------------------------------------------------
    1      N        2.8736874660     0.0762848193    -0.1248637827
    2      C        3.0212452824    -0.9087965109    -1.0576026267
    3      C        3.9584406552    -1.9025406482    -0.8163706095
    4      C        4.7306667925    -1.8914752775     0.3346436854
    5      C        4.5627660636    -0.8659045321     1.2613824464
```

```
    6      C       3.6304915300     0.1058093200     1.0023591690
    7      C       2.2013510365    -0.8793410027    -2.3077312147
    8      C       1.9024303526     1.1716420779    -0.3395002542
    9      N      -1.2057546628    -0.4426066995     1.0311866899
   10      C      -2.1765916456     0.0055963365     0.1861473335
   11      N      -1.8779481338     0.8299761235    -0.7810572463
   12      C      -0.6135052094     1.2577369712    -0.9523801623
   13      C       0.4606394030     0.7519870930    -0.1377774680
   14      C       0.0901392417    -0.0873191760     0.8658494321
   15      C      -3.5828885802    -0.4111547992     0.4265839622
   16      C      -4.3376699513     0.6572123649     1.2531369761
   17      C      -5.7921119473     0.2556495628     1.4729627157
   18      N      -0.4405726340     2.1532229404    -1.9152387139
   19      H       4.0846348121    -2.6832499890    -1.5555248808
   20      H       5.4646912067    -2.6706482592     0.5051908132
   21      H       5.1522582759    -0.8111796765     2.1671408686
   22      H       3.4619067885     0.9392991664     1.6721558480
   23      H       2.4373652392    -1.7458804121    -2.9223109689
   24      H       2.4163380661     0.0133158411    -2.9029290617
   25      H       1.1307418830    -0.9011998114    -2.0930580036
   26      H       2.0683156103     1.5484253507    -1.3480921437
   27      H       2.1691206453     1.9797190500     0.3428868198
   28      H      -1.4641782669    -1.0645011601     1.7876179718
   29      H       0.7943219111    -0.5220663290     1.5653950928
   30      H      -4.0651294215    -0.5285942811    -0.5456543529
   31      H      -3.6131625377    -1.3761846712     0.9414238261
   32      H      -4.2846355303     1.6112090030     0.7212813803
   33      H      -3.8365155999     0.7955390686     2.2176602575
   34      H      -6.3164663710     0.1390129622     0.5216415461
   35      H      -6.3118098939     1.0224470284     2.0488718993
   36      H      -5.8689558501    -0.6855940292     2.0228431173
   37      H       0.4327808828     2.6096874287    -2.1159562337
   38      H      -1.2564369086     2.4544647561    -2.4323141272
 ----------------------------------------------------------------
 Nuclear Repulsion Energy =        1298.58243522 hartrees
 There are       65 alpha and       65 beta electrons

------------------------------------------------
-  Entering fldman on Sun Oct 13 22:30:47 2024  -
------------------------------------------------

 Applying Cartesian multipole field
    Component          Value
    ---------          -----
     (2,0,0)       1.00000E-12
     (0,2,0)       2.00000E-11
     (0,0,2)      -3.00000E-11
 Nucleus-field energy     =    -0.0000000041 hartrees

------------------------------------------------
-  Entering gesman on Sun Oct 13 22:30:47 2024  -
------------------------------------------------

 Requested basis set is 6-311+G(d,p)
 There are 188 shells and 516 basis functions
 A cutoff of  1.0D-12 yielded  12552 shell pairs
 There are    100190 function pairs (    106785 Cartesian)
 Smallest overlap matrix eigenvalue = 1.46E-06
 Linear dependence detected in AO basis
 Tighter screening thresholds may be required for diffuse basis sets
 Use S2THRESH > 12 and THRESH = 14 in case of SCF convergence issues
 Number of orthogonalized atomic orbitals = 510
 Maximum deviation from orthogonality = 1.497E-11
 Guess MOs from SCF MO coefficient file
 Reading MOs from coefficient file
 Reading MOs from coefficient file

------------------------------------------------
-  Entering scfman on Sun Oct 13 22:30:47 2024  -
------------------------------------------------
```

```
 Long-range K will be added via erf
 Coulomb attenuation parameter = 0.2 bohr**(-1)
 A restricted hybrid HF-DFT SCF calculation will be
 performed using Pulay DIIS + Geometric Direct Minimization
 Exchange:     0.2220 Hartree-Fock + 1.0000 wB97X-D + LR-HF
 Correlation:  1.0000 wB97X-D
 Using Euler-Maclaurin-Lebedev (75,302) quadrature formula
 Dispersion:   Grimme D
 SCF converges when RMS gradient is below 1.0E-07
 Geometry optimization detected.  Setting ReadMinima to 0
 Setting SaveMinima to 0
 ---------------------------------------
  Cycle       Energy         DIIS Error
 ---------------------------------------
    1    -763.9739097989      7.21E-04
    2    -763.9681891900      8.61E-05
    3    -763.9686792442      5.85E-05
    4    -763.9687748719      2.80E-05
    5    -763.9688060200      5.21E-06
    6    -763.9688072931      2.47E-06
    7    -763.9688075475      7.19E-07
    8    -763.9688075824      3.05E-07
    9    -763.9688075870      9.66E-08 Convergence criterion met
 ---------------------------------------
 SCF time:  CPU 231.66 s  wall 389.78 s
 SCF   energy in the final basis set = -763.96880759
 Total energy in the final basis set = -763.96880759

-------------------------------------------------
-  Entering anlman on Sun Oct 13 22:37:17 2024  -
-------------------------------------------------



 --------------------------------------------------------------

                   Orbital Energies (a.u.)
 --------------------------------------------------------------


 Alpha MOs
 -- Occupied --
-14.8197 -14.7883 -14.7372 -14.7049 -10.6760 -10.6598 -10.6460 -10.6421
-10.6380 -10.6294 -10.6061 -10.5868 -10.5753 -10.5741 -10.5530 -10.5234
-10.4923 -10.4477  -1.4143  -1.4078  -1.3470  -1.2742  -1.2321  -1.1999
 -1.1914  -1.1410  -1.1003  -1.0867  -1.0539  -1.0497  -1.0362  -0.9986
 -0.9918  -0.9535  -0.9417  -0.9118  -0.9067  -0.8921  -0.8846  -0.8631
 -0.8568  -0.8475  -0.8314  -0.8247  -0.8091  -0.7991  -0.7887  -0.7852
 -0.7663  -0.7614  -0.7564  -0.7532  -0.7482  -0.7321  -0.7207  -0.7102
 -0.6795  -0.6663  -0.6582  -0.6404  -0.6297  -0.6244  -0.6154  -0.6127
 -0.6062
 -- Virtual --
 -0.2727  -0.2580  -0.2399  -0.2284  -0.1745  -0.1658  -0.1502  -0.1450
 -0.1342  -0.1211  -0.1178  -0.1145  -0.1112  -0.1043  -0.1008  -0.0967
 -0.0903  -0.0855  -0.0795  -0.0782  -0.0757  -0.0699  -0.0681  -0.0667
 -0.0619  -0.0584  -0.0558  -0.0543  -0.0490  -0.0440  -0.0417  -0.0325
 -0.0285  -0.0258  -0.0241  -0.0223  -0.0159  -0.0145  -0.0120  -0.0073
 -0.0063   0.0009   0.0029   0.0057   0.0068   0.0116   0.0136   0.0176
  0.0209   0.0238   0.0273   0.0308   0.0338   0.0361   0.0376   0.0412
  0.0434   0.0458   0.0528   0.0544   0.0586   0.0609   0.0628   0.0668
  0.0704   0.0738   0.0751   0.0776   0.0835   0.0877   0.0910   0.0939
  0.0982   0.1027   0.1038   0.1069   0.1071   0.1109   0.1178   0.1195
  0.1233   0.1263   0.1298   0.1365   0.1387   0.1440   0.1450   0.1497
  0.1551   0.1580   0.1613   0.1670   0.1715   0.1759   0.1774   0.1791
  0.1851   0.1887   0.1948   0.2002   0.2033   0.2082   0.2139   0.2248
  0.2298   0.2390   0.2556   0.2704   0.2753   0.2868   0.3026   0.3132
  0.3265   0.3309   0.3398   0.3471   0.3484   0.3576   0.3702   0.3737
  0.3790   0.3836   0.3871   0.3946   0.4036   0.4058   0.4185   0.4250
  0.4266   0.4384   0.4412   0.4528   0.4607   0.4671   0.4714   0.4720
  0.4846   0.4868   0.4916   0.5069   0.5095   0.5168   0.5224   0.5250
  0.5304   0.5328   0.5356   0.5383   0.5433   0.5518   0.5556   0.5615
```

| | | | | | | | |
|---|---|---|---|---|---|---|---|
| 0.5660 | 0.5719 | 0.5764 | 0.5838 | 0.5902 | 0.5947 | 0.5972 | 0.6064 |
| 0.6148 | 0.6243 | 0.6263 | 0.6349 | 0.6409 | 0.6513 | 0.6555 | 0.6610 |
| 0.6666 | 0.6790 | 0.6810 | 0.6860 | 0.6930 | 0.6942 | 0.7025 | 0.7119 |
| 0.7169 | 0.7292 | 0.7369 | 0.7431 | 0.7568 | 0.7661 | 0.7835 | 0.7854 |
| 0.7943 | 0.8041 | 0.8092 | 0.8276 | 0.8314 | 0.8418 | 0.8546 | 0.8605 |
| 0.8651 | 0.8726 | 0.8879 | 0.8890 | 0.9044 | 0.9119 | 0.9247 | 0.9389 |
| 0.9503 | 0.9579 | 0.9684 | 0.9877 | 0.9908 | 1.0192 | 1.0271 | 1.0361 |
| 1.0448 | 1.0535 | 1.0831 | 1.1267 | 1.1293 | 1.1512 | 1.1630 | 1.1700 |
| 1.1987 | 1.2013 | 1.2238 | 1.2277 | 1.2615 | 1.2829 | 1.2977 | 1.3096 |
| 1.3120 | 1.3176 | 1.3321 | 1.3420 | 1.3511 | 1.3551 | 1.3687 | 1.3766 |
| 1.3795 | 1.3865 | 1.3914 | 1.4054 | 1.4148 | 1.4230 | 1.4296 | 1.4331 |
| 1.4471 | 1.4532 | 1.4548 | 1.4613 | 1.4698 | 1.4875 | 1.4923 | 1.5020 |
| 1.5043 | 1.5085 | 1.5152 | 1.5255 | 1.5351 | 1.5407 | 1.5513 | 1.5532 |
| 1.5605 | 1.5659 | 1.5739 | 1.5814 | 1.5949 | 1.6029 | 1.6072 | 1.6151 |
| 1.6210 | 1.6261 | 1.6383 | 1.6408 | 1.6551 | 1.6605 | 1.6855 | 1.6974 |
| 1.7087 | 1.7167 | 1.7289 | 1.7397 | 1.7453 | 1.7552 | 1.7635 | 1.7693 |
| 1.7827 | 1.7902 | 1.8043 | 1.8312 | 1.8387 | 1.8641 | 1.8726 | 1.8830 |
| 1.9008 | 1.9109 | 1.9196 | 1.9482 | 1.9586 | 1.9787 | 1.9849 | 1.9985 |
| 2.0053 | 2.0162 | 2.0263 | 2.0294 | 2.0422 | 2.0573 | 2.0677 | 2.0825 |
| 2.0938 | 2.1150 | 2.1307 | 2.1513 | 2.1641 | 2.1930 | 2.2176 | 2.2190 |
| 2.2375 | 2.2490 | 2.2532 | 2.2575 | 2.2600 | 2.2691 | 2.2889 | 2.2898 |
| 2.3118 | 2.3193 | 2.3318 | 2.3546 | 2.3682 | 2.3799 | 2.3939 | 2.4032 |
| 2.4178 | 2.4309 | 2.4399 | 2.4476 | 2.4478 | 2.4614 | 2.4826 | 2.4899 |
| 2.4973 | 2.5082 | 2.5137 | 2.5245 | 2.5280 | 2.5415 | 2.5502 | 2.5618 |
| 2.5692 | 2.5759 | 2.5851 | 2.5922 | 2.5949 | 2.5992 | 2.6089 | 2.6212 |
| 2.6309 | 2.6489 | 2.6659 | 2.6690 | 2.6742 | 2.6898 | 2.7015 | 2.7055 |
| 2.7168 | 2.7253 | 2.7281 | 2.7345 | 2.7550 | 2.7611 | 2.7739 | 2.7819 |
| 2.8033 | 2.8074 | 2.8186 | 2.8443 | 2.8557 | 2.8715 | 2.8752 | 2.8882 |
| 2.9013 | 2.9210 | 2.9401 | 2.9587 | 3.0413 | 3.0609 | 3.1239 | 3.1539 |
| 3.1607 | 3.2306 | 3.2550 | 3.2693 | 3.3070 | 3.3216 | 3.3678 | 3.4091 |
| 3.4244 | 3.4781 | 3.5285 | 3.5322 | 3.5600 | 3.5975 | 3.6133 | 3.6410 |
| 3.6842 | 3.7042 | 3.7225 | 3.7380 | 3.7394 | 3.7428 | 3.7597 | 3.7625 |
| 3.7680 | 3.8287 | 3.8645 | 3.8998 | 3.9379 | 3.9839 | 4.0522 | 4.0853 |
| 4.0920 | 4.1867 | 4.2917 | 4.3082 | 4.5941 | 4.6700 | 4.6942 | 4.7578 |
| 4.9305 | 5.0333 | 5.1984 | 23.5207 | 23.5870 | 23.7177 | 23.7343 | 23.7412 |
| 23.7792 | 23.8085 | 23.8326 | 23.8414 | 23.8910 | 23.9273 | 23.9619 | 23.9814 |
| 24.0299 | 35.4136 | 35.4654 | 35.5240 | 35.6249 | | | |

Ground-State Mulliken Net Atomic Charges

| Atom | | Charge (a.u.) |
|---|---|---|
| 1 | N | 0.390359 |
| 2 | C | 0.153022 |
| 3 | C | -0.022952 |
| 4 | C | -0.219553 |
| 5 | C | -0.295536 |
| 6 | C | 0.001296 |
| 7 | C | -0.806606 |
| 8 | C | -0.146750 |
| 9 | N | -0.115352 |
| 10 | C | -0.402323 |
| 11 | N | 0.035321 |
| 12 | C | 0.222644 |
| 13 | C | -0.073013 |
| 14 | C | 0.117575 |
| 15 | C | -0.340575 |
| 16 | C | -0.096388 |
| 17 | C | -0.610614 |
| 18 | N | -0.483736 |
| 19 | H | 0.221441 |
| 20 | H | 0.237039 |
| 21 | H | 0.233433 |
| 22 | H | 0.221032 |
| 23 | H | 0.232910 |
| 24 | H | 0.239366 |
| 25 | H | 0.236612 |
| 26 | H | 0.171482 |
| 27 | H | 0.253597 |

```
    28 H                    0.384644
    29 H                    0.209973
    30 H                    0.242162
    31 H                    0.194004
    32 H                    0.199919
    33 H                    0.172064
    34 H                    0.176164
    35 H                    0.197760
    36 H                    0.169253
    37 H                    0.317642
    38 H                    0.382681
 ----------------------------------------
 Sum of atomic charges =     2.000000

-----------------------------------------------------------------
                   Cartesian Multipole Moments
-----------------------------------------------------------------
   Charge (ESU x 10^10)
                 9.6064
   Dipole Moment (Debye)
        X       9.7808      Y      -0.3975      Z      -0.0067
      Tot       9.7889
   Quadrupole Moments (Debye-Ang)
       XX      -0.7725     XY     -15.5890     YY     -75.3025
       XZ      -5.1616     YZ      -9.0353     ZZ     -73.2936
   Traceless Quadrupole Moments (Debye-Ang)
      QXX     147.0511    QYY     -76.5389    QZZ     -70.5122
      QXY     -46.7669    QXZ     -15.4848    QYZ     -27.1060
   Octopole Moments (Debye-Ang^2)
      XXX      59.1613    XXY     -91.5589    XYY      24.1829
      YYY       6.3164    XXZ      73.3459    XYZ      13.7289
      YYZ     -30.3242    XZZ      -0.0863    YZZ       9.0071
      ZZZ     -11.2424
   Traceless Octopole Moments (Debye-Ang^2)
      XXX     138.0975    YYY     780.8641    ZZZ    -454.6497
      XXY   -1144.6769    XXZ    1004.8499    XYY     112.9701
      XYZ     205.9337    XZZ    -251.0676    YYZ    -550.2002
      YZZ     363.8129
   Hexadecapole Moments (Debye-Ang^3)
     XXXX   -4245.0072   XXXY     -82.2262   XXYY    -895.2066
     XYYY     346.6880   YYYY    -695.6779   XXXZ     386.7946
     XXYZ      28.3036   XYYZ      84.2487   YYYZ      -8.4508
     XXZZ    -976.0219   XYZZ     109.0344   YYZZ    -187.9913
     XZZZ     309.9377   YZZZ       1.4842   ZZZZ    -825.4637
   Traceless Hexadecapole Moments (Debye-Ang^3)
     XXXX   15774.1655   XXXY  -25441.0803   XXXZ    5469.2879
     XXYY   -5223.7881   XXYZ    2659.0817   XXZZ  -10550.3775
     XYYY   19594.9111   XYYZ   -2868.6050   XYZZ    5846.1692
     XZZZ   -2600.6828   YYYY   -1908.6513   YYYZ   -1825.7072
     YYZZ    7132.4393   YZZZ    -833.3746   ZZZZ    3417.9381
-----------------------------------------------------------------

-------------------------------------------------
-  Entering drvman on Sun Oct 13 22:37:17 2024  -
-------------------------------------------------

 Calculating analytic gradient of the SCF energy
 Gradient of SCF Energy
           1           2           3           4           5           6
    1   0.0000877   0.0000777  -0.0000894  -0.0000584   0.0000516   0.0000298
    2  -0.0000229  -0.0002326   0.0000126   0.0001042  -0.0000657   0.0000306
    3  -0.0000476   0.0001770  -0.0000622   0.0001701  -0.0000671  -0.0001718
           7           8           9          10          11          12
    1  -0.0003030   0.0000017   0.0002642  -0.0003442  -0.0001404   0.0004853
    2   0.0001450   0.0003108  -0.0002728   0.0003216  -0.0004027   0.0000189
    3   0.0000444   0.0002378   0.0006802  -0.0005905   0.0000107  -0.0011787
          13          14          15          16          17          18
    1   0.0002872  -0.0004749  -0.0000340   0.0000274   0.0000053  -0.0006097
    2  -0.0001712   0.0003140  -0.0001839  -0.0000133  -0.0000028   0.0001961
    3  -0.0001702   0.0001922  -0.0000579   0.0001350  -0.0000421   0.0007358
```

```
          19           20           21           22           23           24
    1   0.0000545   0.0000316  -0.0000221   0.0000011  -0.0001061  -0.0003316
    2  -0.0000067  -0.0000327  -0.0000246   0.0000556   0.0000171   0.0002638
    3  -0.0000273  -0.0000104   0.0000390  -0.0000327  -0.0000603   0.0000868
          25           26           27           28           29           30
    1   0.0001664   0.0005119   0.0001245   0.0000882   0.0000088   0.0000983
    2  -0.0000490   0.0001365  -0.0002167   0.0000610   0.0000494   0.0000639
    3  -0.0000492   0.0003222   0.0000060   0.0000583  -0.0000645   0.0000206
          31           32           33           34           35           36
    1  -0.0000019  -0.0000143   0.0000130   0.0000108  -0.0000196   0.0000038
    2   0.0000054   0.0000286   0.0000013  -0.0000005   0.0000080  -0.0000096
    3   0.0000333  -0.0000074   0.0000073  -0.0000205  -0.0000178  -0.0000191
          37           38
    1   0.0001315  -0.0000124
    2  -0.0005866   0.0001499
    3  -0.0002438  -0.0000157
 Max gradient component =       1.179E-03
 RMS gradient           =       2.321E-04
 Gradient time:  CPU 110.30 s  wall 189.78 s

-------------------------------------------------
-  Entering optman on Sun Oct 13 22:40:27 2024  -
-------------------------------------------------

 Geometry Optimization Parameters
   NAtoms,    NIC,     NZ,  NCons,   NDum,   NFix, NCnnct, MaxDiis
       38     272       0       0       0       0       0       0

 Cartesian Hessian Update
 Hessian updated using BFGS update


** GEOMETRY OPTIMIZATION IN DELOCALIZED INTERNAL COORDINATES **
   Searching for a Minimum

   Optimization Cycle:   9

                      Coordinates (Angstroms)
     ATOM                X               Y               Z
      1  N         2.8736874660    0.0762848193   -0.1248637827
      2  C         3.0212452824   -0.9087965109   -1.0576026267
      3  C         3.9584406552   -1.9025406482   -0.8163706095
      4  C         4.7306667925   -1.8914752775    0.3346436854
      5  C         4.5627660636   -0.8659045321    1.2613824464
      6  C         3.6304915300    0.1058093200    1.0023591690
      7  C         2.2013510365   -0.8793410027   -2.3077312147
      8  C         1.9024303526    1.1716420779   -0.3395002542
      9  N        -1.2057546628   -0.4426066995    1.0311866899
     10  C        -2.1765916456    0.0055963365    0.1861473335
     11  N        -1.8779481338    0.8299761235   -0.7810572463
     12  C        -0.6135052094    1.2577369712   -0.9523801623
     13  C         0.4606394030    0.7519870930   -0.1377774680
     14  C         0.0901392417   -0.0873191760    0.8658494321
     15  C        -3.5828885802   -0.4111547992    0.4265839622
     16  C        -4.3376699513    0.6572123649    1.2531369761
     17  C        -5.7921119473    0.2556495628    1.4729627157
     18  N        -0.4405726340    2.1532229404   -1.9152387139
     19  H         4.0846348121   -2.6832499890   -1.5555248808
     20  H         5.4646912067   -2.6706482592    0.5051908132
     21  H         5.1522582759   -0.8111796765    2.1671408686
     22  H         3.4619067885    0.9392991664    1.6721558480
     23  H         2.4373652392   -1.7458804121   -2.9223109689
     24  H         2.4163380661    0.0133158411   -2.9029290617
     25  H         1.1307418830   -0.9011998114   -2.0930580036
     26  H         2.0683156103    1.5484253507   -1.3480921437
     27  H         2.1691206453    1.9797190500    0.3428868198
     28  H        -1.4641782669   -1.0645011601    1.7876179718
     29  H         0.7943219111   -0.5220663290    1.5653950928
     30  H        -4.0651294215   -0.5285942811   -0.5456543529
     31  H        -3.6131625377   -1.3761846712    0.9414238261
```

| | | | | |
|---|---|---|---|---|
| 32 | H | -4.2846355303 | 1.6112090030 | 0.7212813803 |
| 33 | H | -3.8365155999 | 0.7955390686 | 2.2176602575 |
| 34 | H | -6.3164663710 | 0.1390129622 | 0.5216415461 |
| 35 | H | -6.3118098939 | 1.0224470284 | 2.0488718993 |
| 36 | H | -5.8689558501 | -0.6855940292 | 2.0228431173 |
| 37 | H | 0.4327808828 | 2.6096874287 | -2.1159562337 |
| 38 | H | -1.2564369086 | 2.4544647561 | -2.4323141272 |

Point Group: c1    Number of degrees of freedom:   108

Energy is   -763.968807587

Hessian updated using BFGS update
internal optimization (0)

108 Hessian modes will be used to form the next step
Hessian Eigenvalues:

| | | | | | |
|---|---|---|---|---|---|
| 0.000434 | 0.002725 | 0.003234 | 0.003751 | 0.008236 | 0.011364 |
| 0.018087 | 0.018745 | 0.019240 | 0.019838 | 0.020728 | 0.022467 |
| 0.022661 | 0.022788 | 0.024058 | 0.024305 | 0.025796 | 0.026581 |
| 0.027463 | 0.028262 | 0.028828 | 0.030138 | 0.030833 | 0.036030 |
| 0.038400 | 0.041413 | 0.043303 | 0.043811 | 0.044254 | 0.047620 |
| 0.052256 | 0.053378 | 0.054307 | 0.055102 | 0.062329 | 0.077532 |
| 0.083839 | 0.093906 | 0.121964 | 0.122340 | 0.126856 | 0.128186 |
| 0.132007 | 0.132987 | 0.136586 | 0.140841 | 0.142843 | 0.144024 |
| 0.147529 | 0.147956 | 0.149947 | 0.151663 | 0.152538 | 0.152709 |
| 0.157900 | 0.160966 | 0.190772 | 0.193768 | 0.206307 | 0.216454 |
| 0.224960 | 0.228618 | 0.238842 | 0.247768 | 0.248487 | 0.263708 |
| 0.271795 | 0.275478 | 0.282902 | 0.295410 | 0.299250 | 0.300153 |
| 0.300561 | 0.301110 | 0.301514 | 0.302503 | 0.304082 | 0.304146 |
| 0.304915 | 0.305432 | 0.306541 | 0.308165 | 0.308648 | 0.312538 |
| 0.321882 | 0.329180 | 0.332188 | 0.333225 | 0.334662 | 0.337126 |
| 0.341946 | 0.346164 | 0.356261 | 0.365524 | 0.381417 | 0.389808 |
| 0.397659 | 0.406818 | 0.417743 | 0.420533 | 0.420806 | 0.429778 |
| 0.439185 | 0.446534 | 0.458182 | 0.500999 | 0.574578 | 0.745763 |

Minimum search - taking simple RFO step
Searching for Lamda that Minimizes Along All modes
Value Taken    Lamda =  -0.00083379
Calculated Step too Large.  Step scaled by  0.394011
Step Taken.  Stepsize is  0.300000

| | Maximum | Tolerance | Cnvgd? |
|---|---|---|---|
| Gradient | 0.001941 | 0.000800 | NO |
| Displacement | 0.129766 | 0.001400 | NO |
| Energy change | -0.000335 | 0.000228 | NO |

New Cartesian Coordinates Obtained by Inverse Iteration

Displacement from previous Coordinates is:  0.739650

Standard Nuclear Orientation (Angstroms)

| I | Atom | X | Y | Z |
|---|---|---|---|---|
| 1 | N | 2.8692177272 | 0.0735225767 | -0.1406543057 |
| 2 | C | 3.0411914968 | -0.9207707525 | -1.0594703750 |
| 3 | C | 3.9794714658 | -1.9059280542 | -0.7888227192 |
| 4 | C | 4.7305208967 | -1.8754977392 | 0.3757972313 |
| 5 | C | 4.5389977230 | -0.8400501489 | 1.2870047140 |
| 6 | C | 3.6044413489 | 0.1219013586 | 1.0001878970 |
| 7 | C | 2.2522470330 | -0.9044458075 | -2.3295442840 |
| 8 | C | 1.8905736772 | 1.1540593364 | -0.3863655511 |
| 9 | N | -1.2036577296 | -0.4938438455 | 0.9842889530 |
| 10 | C | -2.1863706393 | 0.0042514489 | 0.1833908929 |
| 11 | N | -1.8985446011 | 0.8668019088 | -0.7537995147 |
| 12 | C | -0.6324536607 | 1.2834384564 | -0.9352060911 |
| 13 | C | 0.4523266189 | 0.7314004000 | -0.1659655805 |
| 14 | C | 0.0936825894 | -0.1474339352 | 0.8068514645 |
| 15 | C | -3.5934691696 | -0.4044441082 | 0.4345567212 |

```
   16      C      -4.3366065670     0.6661691084     1.2678456642
   17      C      -5.7905234750     0.2700487384     1.5004405152
   18      N      -0.4665152253     2.2205508585    -1.8599520051
   19      H       4.1246254039    -2.6945718818    -1.5160155404
   20      H       5.4666961922    -2.6473148443     0.5691084174
   21      H       5.1120155668    -0.7706377320     2.2023204972
   22      H       3.4161117954     0.9611626068     1.6574992674
   23      H       2.4580947659    -1.8070692180    -2.9016543879
   24      H       2.5341783720    -0.0503917758    -2.9538294079
   25      H       1.1767409179    -0.8590394164    -2.1460017893
   26      H       2.0467786866     1.4954292181    -1.4093946440
   27      H       2.1532860302     1.9877519416     0.2667721762
   28      H      -1.4541028412    -1.1468119155     1.7169084255
   29      H       0.8059760587    -0.6214789184     1.4716771530
   30      H      -4.0844608703    -0.5167412707    -0.5342928674
   31      H      -3.6261093490    -1.3708762459     0.9464687678
   32      H      -4.2845044808     1.6199070964     0.7355751185
   33      H      -3.8262363718     0.8020353939     2.2277652328
   34      H      -6.3234573270     0.1542774699     0.5537383185
   35      H      -6.3025814609     1.0392186938     2.0800372236
   36      H      -5.8661629376    -0.6703186544     2.0520265626
   37      H       0.4139754447     2.6554977284    -2.0779225823
   38      H      -1.2853931052     2.5402419243    -2.3613695680
 ----------------------------------------------------------------
 Nuclear Repulsion Energy =        1297.74365281 hartrees
 There are       65 alpha and       65 beta electrons

--------------------------------------------------
-  Entering fldman on Sun Oct 13 22:40:27 2024  -
--------------------------------------------------

 Applying Cartesian multipole field
    Component          Value
    ---------          -----
     (2,0,0)        1.00000E-12
     (0,2,0)        2.00000E-11
     (0,0,2)       -3.00000E-11
 Nucleus-field energy     =    -0.0000000037 hartrees

--------------------------------------------------
-  Entering gesman on Sun Oct 13 22:40:27 2024  -
--------------------------------------------------

 Requested basis set is 6-311+G(d,p)
 There are 188 shells and 516 basis functions
 A cutoff of  1.0D-12 yielded  12534 shell pairs
 There are    100088 function pairs (    106673 Cartesian)
 Smallest overlap matrix eigenvalue = 1.44E-06
 Linear dependence detected in AO basis
 Tighter screening thresholds may be required for diffuse basis sets
 Use S2THRESH > 12 and THRESH = 14 in case of SCF convergence issues
 Number of orthogonalized atomic orbitals = 510
 Maximum deviation from orthogonality = 1.809E-11
 Guess MOs from SCF MO coefficient file
 Reading MOs from coefficient file
 Reading MOs from coefficient file

--------------------------------------------------
-  Entering scfman on Sun Oct 13 22:40:27 2024  -
--------------------------------------------------

 Long-range K will be added via erf
 Coulomb attenuation parameter = 0.2 bohr**(-1)
 A restricted hybrid HF-DFT SCF calculation will be
 performed using Pulay DIIS + Geometric Direct Minimization
 Exchange:     0.2220 Hartree-Fock + 1.0000 wB97X-D + LR-HF
 Correlation:  1.0000 wB97X-D
 Using Euler-Maclaurin-Lebedev (75,302) quadrature formula
 Dispersion:   Grimme D
 SCF converges when RMS gradient is below 1.0E-07
```

```
 Geometry optimization detected.  Setting ReadMinima to 0
 Setting SaveMinima to 0
 ---------------------------------------
  Cycle       Energy         DIIS Error
 ---------------------------------------
    1    -763.9712382224      7.15E-04
    2    -763.9684816151      8.54E-05
    3    -763.9689718374      5.70E-05
    4    -763.9690615428      2.78E-05
    5    -763.9690916650      5.23E-06
    6    -763.9690929591      2.48E-06
    7    -763.9690932159      7.05E-07
    8    -763.9690932515      3.15E-07
    9    -763.9690932562      9.95E-08 Convergence criterion met
 ---------------------------------------
 SCF time:  CPU 213.88 s  wall 379.59 s
 SCF   energy in the final basis set = -763.96909326
 Total energy in the final basis set = -763.96909326

-------------------------------------------------
-  Entering anlman on Sun Oct 13 22:46:47 2024  -
-------------------------------------------------



 --------------------------------------------------------------

                   Orbital Energies (a.u.)
 --------------------------------------------------------------

 Alpha MOs
 -- Occupied --
-14.8201 -14.7881 -14.7371 -14.7046 -10.6757 -10.6595 -10.6457 -10.6425
-10.6381 -10.6299 -10.6062 -10.5872 -10.5757 -10.5745 -10.5529 -10.5232
-10.4914 -10.4472  -1.4143  -1.4082  -1.3465  -1.2739  -1.2323  -1.1999
 -1.1917  -1.1409  -1.1004  -1.0868  -1.0538  -1.0494  -1.0365  -0.9983
 -0.9919  -0.9533  -0.9416  -0.9114  -0.9065  -0.8916  -0.8852  -0.8627
 -0.8567  -0.8476  -0.8319  -0.8249  -0.8087  -0.7992  -0.7890  -0.7859
 -0.7661  -0.7612  -0.7561  -0.7529  -0.7481  -0.7324  -0.7208  -0.7097
 -0.6793  -0.6659  -0.6581  -0.6399  -0.6295  -0.6246  -0.6149  -0.6121
 -0.6059
 -- Virtual --
 -0.2732  -0.2576  -0.2391  -0.2285  -0.1746  -0.1657  -0.1503  -0.1451
 -0.1344  -0.1199  -0.1178  -0.1142  -0.1112  -0.1043  -0.1011  -0.0967
 -0.0905  -0.0860  -0.0793  -0.0778  -0.0758  -0.0705  -0.0686  -0.0673
 -0.0620  -0.0588  -0.0554  -0.0545  -0.0499  -0.0451  -0.0411  -0.0319
 -0.0284  -0.0258  -0.0241  -0.0214  -0.0161  -0.0141  -0.0121  -0.0074
 -0.0058   0.0005   0.0027   0.0055   0.0061   0.0114   0.0139   0.0181
  0.0206   0.0239   0.0273   0.0321   0.0338   0.0355   0.0388   0.0415
  0.0433   0.0460   0.0530   0.0546   0.0575   0.0610   0.0621   0.0670
  0.0702   0.0738   0.0754   0.0778   0.0817   0.0890   0.0908   0.0958
  0.0972   0.1020   0.1029   0.1064   0.1086   0.1108   0.1186   0.1195
  0.1234   0.1263   0.1304   0.1359   0.1386   0.1442   0.1446   0.1498
  0.1551   0.1576   0.1620   0.1680   0.1705   0.1750   0.1770   0.1780
  0.1855   0.1883   0.1957   0.1993   0.2024   0.2106   0.2140   0.2249
  0.2300   0.2409   0.2572   0.2722   0.2762   0.2908   0.3044   0.3138
  0.3264   0.3315   0.3403   0.3457   0.3501   0.3577   0.3695   0.3723
  0.3789   0.3846   0.3876   0.3939   0.4009   0.4057   0.4169   0.4192
  0.4246   0.4394   0.4405   0.4509   0.4604   0.4656   0.4705   0.4734
  0.4840   0.4882   0.4892   0.5052   0.5108   0.5163   0.5205   0.5240
  0.5258   0.5336   0.5350   0.5396   0.5437   0.5540   0.5546   0.5608
  0.5660   0.5721   0.5744   0.5835   0.5888   0.5936   0.5990   0.6071
  0.6151   0.6222   0.6261   0.6342   0.6346   0.6520   0.6558   0.6626
  0.6663   0.6787   0.6838   0.6867   0.6932   0.6939   0.7020   0.7110
  0.7165   0.7316   0.7384   0.7406   0.7597   0.7659   0.7826   0.7849
  0.7960   0.8031   0.8063   0.8268   0.8287   0.8473   0.8524   0.8600
  0.8649   0.8716   0.8880   0.8906   0.9074   0.9142   0.9221   0.9411
  0.9547   0.9589   0.9670   0.9879   0.9897   1.0250   1.0294   1.0385
  1.0490   1.0528   1.0846   1.1254   1.1309   1.1536   1.1631   1.1695
  1.1980   1.2049   1.2218   1.2284   1.2612   1.2839   1.2972   1.3050
  1.3069   1.3170   1.3303   1.3381   1.3513   1.3557   1.3695   1.3761
```

| | | | | | | | |
|---|---|---|---|---|---|---|---|
| 1.3790 | 1.3866 | 1.3936 | 1.4033 | 1.4124 | 1.4211 | 1.4300 | 1.4346 |
| 1.4459 | 1.4491 | 1.4531 | 1.4613 | 1.4684 | 1.4852 | 1.4912 | 1.5009 |
| 1.5034 | 1.5077 | 1.5159 | 1.5287 | 1.5355 | 1.5403 | 1.5497 | 1.5543 |
| 1.5606 | 1.5655 | 1.5696 | 1.5790 | 1.5963 | 1.6019 | 1.6095 | 1.6166 |
| 1.6178 | 1.6265 | 1.6391 | 1.6398 | 1.6570 | 1.6633 | 1.6863 | 1.6957 |
| 1.7117 | 1.7166 | 1.7285 | 1.7412 | 1.7465 | 1.7552 | 1.7632 | 1.7699 |
| 1.7812 | 1.7923 | 1.8031 | 1.8286 | 1.8386 | 1.8612 | 1.8715 | 1.8839 |
| 1.8972 | 1.9107 | 1.9208 | 1.9474 | 1.9593 | 1.9777 | 1.9877 | 2.0017 |
| 2.0093 | 2.0165 | 2.0274 | 2.0305 | 2.0416 | 2.0584 | 2.0714 | 2.0824 |
| 2.0958 | 2.1171 | 2.1300 | 2.1524 | 2.1658 | 2.1916 | 2.2155 | 2.2190 |
| 2.2382 | 2.2482 | 2.2539 | 2.2580 | 2.2619 | 2.2696 | 2.2887 | 2.2907 |
| 2.3122 | 2.3147 | 2.3294 | 2.3551 | 2.3665 | 2.3808 | 2.3940 | 2.4032 |
| 2.4173 | 2.4323 | 2.4416 | 2.4474 | 2.4493 | 2.4615 | 2.4799 | 2.4913 |
| 2.4972 | 2.5106 | 2.5152 | 2.5246 | 2.5303 | 2.5409 | 2.5506 | 2.5634 |
| 2.5675 | 2.5761 | 2.5873 | 2.5917 | 2.5943 | 2.5984 | 2.6081 | 2.6199 |
| 2.6232 | 2.6487 | 2.6639 | 2.6694 | 2.6770 | 2.6892 | 2.7058 | 2.7085 |
| 2.7189 | 2.7218 | 2.7263 | 2.7343 | 2.7530 | 2.7593 | 2.7759 | 2.7819 |
| 2.8033 | 2.8074 | 2.8157 | 2.8454 | 2.8585 | 2.8731 | 2.8756 | 2.8900 |
| 2.9015 | 2.9058 | 2.9410 | 2.9530 | 3.0452 | 3.0582 | 3.1154 | 3.1476 |
| 3.1601 | 3.2292 | 3.2554 | 3.2665 | 3.3054 | 3.3232 | 3.3691 | 3.4081 |
| 3.4247 | 3.4775 | 3.5296 | 3.5318 | 3.5605 | 3.5962 | 3.6152 | 3.6429 |
| 3.6838 | 3.7008 | 3.7232 | 3.7364 | 3.7391 | 3.7414 | 3.7555 | 3.7644 |
| 3.7674 | 3.8280 | 3.8640 | 3.8993 | 3.9375 | 3.9834 | 4.0529 | 4.0822 |
| 4.0922 | 4.1878 | 4.2912 | 4.3099 | 4.5939 | 4.6701 | 4.6916 | 4.7649 |
| 4.9302 | 5.0319 | 5.1996 | 23.5220 | 23.5877 | 23.7191 | 23.7334 | 23.7416 |
| 23.7786 | 23.8091 | 23.8320 | 23.8411 | 23.8910 | 23.9278 | 23.9611 | 23.9821 |
| 24.0289 | 35.4122 | 35.4655 | 35.5242 | 35.6255 | | | |

---

Ground-State Mulliken Net Atomic Charges

| Atom | Charge (a.u.) |
|---|---|
| 1 N | 0.378315 |
| 2 C | 0.150810 |
| 3 C | -0.023012 |
| 4 C | -0.210139 |
| 5 C | -0.303547 |
| 6 C | 0.027924 |
| 7 C | -0.813953 |
| 8 C | -0.148939 |
| 9 N | -0.112881 |
| 10 C | -0.393100 |
| 11 N | 0.025437 |
| 12 C | 0.226286 |
| 13 C | -0.072352 |
| 14 C | 0.094596 |
| 15 C | -0.329418 |
| 16 C | -0.102314 |
| 17 C | -0.611252 |
| 18 N | -0.479321 |
| 19 H | 0.221989 |
| 20 H | 0.237182 |
| 21 H | 0.233656 |
| 22 H | 0.221880 |
| 23 H | 0.231507 |
| 24 H | 0.247732 |
| 25 H | 0.231623 |
| 26 H | 0.176077 |
| 27 H | 0.255147 |
| 28 H | 0.381405 |
| 29 H | 0.210156 |
| 30 H | 0.243278 |
| 31 H | 0.190400 |
| 32 H | 0.200434 |
| 33 H | 0.172387 |
| 34 H | 0.175740 |
| 35 H | 0.197645 |
| 36 H | 0.169292 |
| 37 H | 0.316753 |

```
    38 H                       0.382578
  ----------------------------------------
  Sum of atomic charges =     2.000000

 -----------------------------------------------------------------
                    Cartesian Multipole Moments
 -----------------------------------------------------------------
    Charge (ESU x 10^10)
                9.6064
    Dipole Moment (Debye)
         X       9.8208      Y      -0.4931      Z      -0.1513
       Tot       9.8343
    Quadrupole Moments (Debye-Ang)
        XX      -0.7970     XY     -15.7088     YY     -74.6273
        XZ      -5.2055     YZ      -9.3075     ZZ     -73.8250
    Traceless Quadrupole Moments (Debye-Ang)
       QXX     146.8583    QYY     -74.6326    QZZ     -72.2257
       QXY     -47.1263    QXZ     -15.6165    QYZ     -27.9224
    Octopole Moments (Debye-Ang^2)
       XXX      59.8053    XXY     -91.4133    XYY      23.4013
       YYY       7.2943    XXZ      72.9085    XYZ      13.4561
       YYZ     -30.4531    XZZ       1.6845    YZZ       8.3488
       ZZZ     -11.9671
    Traceless Octopole Moments (Debye-Ang^2)
       XXX     133.0593    YYY     791.3463    ZZZ    -453.9013
       XXY   -1143.8884    XXZ    1002.1628    XYY      96.3459
       XYZ     201.8418    XZZ    -229.4052    YYZ    -548.2615
       YZZ     352.5421
    Hexadecapole Moments (Debye-Ang^3)
      XXXX   -4266.5208   XXXY     -75.1442   XXYY    -899.5256
      XYYY     351.1534   YYYY    -703.2133   XXXZ     389.9656
      XXYZ      27.1137   XYYZ      87.7617   YYYZ     -11.9231
      XXZZ    -971.0293   XYZZ     109.8480   YYZZ    -188.6389
      XZZZ     312.9243   YZZZ       6.7507   ZZZZ    -826.1324
    Traceless Hexadecapole Moments (Debye-Ang^3)
      XXXX   15123.8427   XXXY  -25253.7148   XXXZ    5367.0692
      XXYY   -5266.1445   XXYZ    2529.0757   XXZZ   -9857.6982
      XYYY   19507.5343   XYYZ   -2644.8008   XYZZ    5746.1805
      XZZZ   -2722.2684   YYYY   -1841.6841   YYYZ   -2205.4982
      YYZZ    7107.8287   YZZZ    -323.5774   ZZZZ    2749.8696
 -----------------------------------------------------------------

--------------------------------------------------
-  Entering drvman on Sun Oct 13 22:46:47 2024  -
--------------------------------------------------

 Calculating analytic gradient of the SCF energy
 Gradient of SCF Energy
            1           2           3           4           5           6
    1  -0.0000590   0.0000735  -0.0000792  -0.0000376   0.0001145   0.0000109
    2   0.0001325  -0.0002940  -0.0000386   0.0001360  -0.0001825   0.0001693
    3   0.0000060   0.0000474  -0.0000375   0.0002560  -0.0001304  -0.0001800
            7           8           9          10          11          12
    1  -0.0003207   0.0000678   0.0002178   0.0000465  -0.0003362   0.0005203
    2   0.0002357   0.0004514  -0.0001983   0.0002929  -0.0002386  -0.0010593
    3   0.0000795   0.0002078   0.0004740  -0.0002898  -0.0000278  -0.0007445
           13          14          15          16          17          18
    1  -0.0000399  -0.0004246  -0.0000873   0.0000790   0.0000155  -0.0005765
    2  -0.0003214   0.0004302  -0.0001391  -0.0000572   0.0000037   0.0013121
    3  -0.0002788   0.0001464  -0.0000471   0.0000857  -0.0000569   0.0010091
           19          20          21          22          23          24
    1   0.0000976   0.0000515   0.0000190   0.0000336  -0.0000727  -0.0001295
    2  -0.0000408  -0.0000300  -0.0000473   0.0000407   0.0000257   0.0002000
    3  -0.0000105   0.0000026   0.0000428   0.0000153  -0.0000419  -0.0000015
           25          26          27          28          29          30
    1   0.0000803   0.0005052   0.0000462   0.0000174  -0.0000137   0.0000324
    2  -0.0000021  -0.0000078  -0.0001632   0.0000165   0.0000386   0.0000680
    3  -0.0000050   0.0002364   0.0000197   0.0000999   0.0000077   0.0000242
           31          32          33          34          35          36
    1   0.0000006  -0.0000389  -0.0000067   0.0000146  -0.0000212   0.0000088
```

```
    2  -0.0000011   0.0000437   0.0000119  -0.0000093   0.0000181  -0.0000128
    3   0.0000157  -0.0000155   0.0000073  -0.0000329  -0.0000134  -0.0000136
            37          38
    1   0.0003243  -0.0001333
    2  -0.0006387  -0.0001450
    3  -0.0004609  -0.0003956
 Max gradient component =       1.312E-03
 RMS gradient           =       2.718E-04
 Gradient time:  CPU 100.84 s  wall 183.39 s

--------------------------------------------------
-  Entering optman on Sun Oct 13 22:49:50 2024  -
--------------------------------------------------

 Geometry Optimization Parameters
   NAtoms,    NIC,     NZ,  NCons,   NDum,   NFix, NCnnct, MaxDiis
       38     272       0       0       0       0       0       0

 Cartesian Hessian Update
 Hessian updated using BFGS update


** GEOMETRY OPTIMIZATION IN DELOCALIZED INTERNAL COORDINATES **
   Searching for a Minimum

   Optimization Cycle:  10

                    Coordinates (Angstroms)
     ATOM              X               Y               Z
      1  N         2.8692177272    0.0735225767   -0.1406543057
      2  C         3.0411914968   -0.9207707525   -1.0594703750
      3  C         3.9794714658   -1.9059280542   -0.7888227192
      4  C         4.7305208967   -1.8754977392    0.3757972313
      5  C         4.5389977230   -0.8400501489    1.2870047140
      6  C         3.6044413489    0.1219013586    1.0001878970
      7  C         2.2522470330   -0.9044458075   -2.3295442840
      8  C         1.8905736772    1.1540593364   -0.3863655511
      9  N        -1.2036577296   -0.4938438455    0.9842889530
     10  C        -2.1863706393    0.0042514489    0.1833908929
     11  N        -1.8985446011    0.8668019088   -0.7537995147
     12  C        -0.6324536607    1.2834384564   -0.9352060911
     13  C         0.4523266189    0.7314004000   -0.1659655805
     14  C         0.0936825894   -0.1474339352    0.8068514645
     15  C        -3.5934691696   -0.4044441082    0.4345567212
     16  C        -4.3366065670    0.6661691084    1.2678456642
     17  C        -5.7905234750    0.2700487384    1.5004405152
     18  N        -0.4665152253    2.2205508585   -1.8599520051
     19  H         4.1246254039   -2.6945718818   -1.5160155404
     20  H         5.4666961922   -2.6473148443    0.5691084174
     21  H         5.1120155668   -0.7706377320    2.2023204972
     22  H         3.4161117954    0.9611626068    1.6574992674
     23  H         2.4580947659   -1.8070692180   -2.9016543879
     24  H         2.5341783720   -0.0503917758   -2.9538294079
     25  H         1.1767409179   -0.8590394164   -2.1460017893
     26  H         2.0467786866    1.4954292181   -1.4093946440
     27  H         2.1532860302    1.9877519416    0.2667721762
     28  H        -1.4541028412   -1.1468119155    1.7169084255
     29  H         0.8059760587   -0.6214789184    1.4716771530
     30  H        -4.0844608703   -0.5167412707   -0.5342928674
     31  H        -3.6261093490   -1.3708762459    0.9464687678
     32  H        -4.2845044808    1.6199070964    0.7355751185
     33  H        -3.8262363718    0.8020353939    2.2277652328
     34  H        -6.3234573270    0.1542774699    0.5537383185
     35  H        -6.3025814609    1.0392186938    2.0800372236
     36  H        -5.8661629376   -0.6703186544    2.0520265626
     37  H         0.4139754447    2.6554977284   -2.0779225823
     38  H        -1.2853931052    2.5402419243   -2.3613695680
   Point Group: c1    Number of degrees of freedom:   108
```

```
   Energy is   -763.969093256

 Hessian updated using BFGS update
  internal optimization (0)

108 Hessian modes will be used to form the next step
  Hessian Eigenvalues:
     0.000855    0.002740    0.003147    0.003747    0.007980    0.011539
     0.017230    0.018577    0.019205    0.019912    0.020576    0.022387
     0.022664    0.022921    0.024129    0.024315    0.025784    0.026762
     0.027416    0.028271    0.028950    0.030165    0.030835    0.036026
     0.038400    0.041441    0.043264    0.043802    0.044131    0.047604
     0.051986    0.054127    0.054848    0.056587    0.060191    0.077553
     0.083864    0.093442    0.121910    0.122338    0.126856    0.128119
     0.132009    0.132983    0.136615    0.140853    0.142859    0.143980
     0.147457    0.147530    0.149925    0.151661    0.152558    0.152985
     0.157829    0.161168    0.190779    0.193786    0.206300    0.216548
     0.225365    0.228719    0.238641    0.247776    0.248490    0.263612
     0.271784    0.275572    0.282744    0.295215    0.298800    0.300153
     0.300565    0.301088    0.301574    0.302514    0.304099    0.304150
     0.304868    0.305108    0.306256    0.308222    0.308346    0.312640
     0.320516    0.329262    0.332187    0.333405    0.334785    0.337130
     0.341491    0.344916    0.356592    0.365597    0.381214    0.389747
     0.397696    0.406648    0.417776    0.420363    0.421439    0.430736
     0.439325    0.447192    0.459867    0.501961    0.572537    0.729541

 Minimum search - taking simple RFO step
 Searching for Lamda that Minimizes Along All modes
 Value Taken    Lamda =  -0.00057759
 Calculated Step too Large.  Step scaled by  0.524451
 Step Taken.  Stepsize is  0.300000

                          Maximum     Tolerance    Cnvgd?
        Gradient          0.001942     0.000800      NO
        Displacement      0.103809     0.001400      NO
        Energy change    -0.000286     0.000228      NO


 New Cartesian Coordinates Obtained by Inverse Iteration

 Displacement from previous Coordinates is:  0.822603
 ----------------------------------------------------------------
             Standard Nuclear Orientation (Angstroms)
    I     Atom           X                Y                Z
 ----------------------------------------------------------------
    1      N       2.8640518275     0.0708310852    -0.1600041584
    2      C       3.0627730708    -0.9343474474    -1.0610829338
    3      C       4.0019589031    -1.9087776692    -0.7567136083
    4      C       4.7302796856    -1.8541421383     0.4213498905
    5      C       4.5129227440    -0.8067309859     1.3132206292
    6      C       3.5757683527     0.1423651321     0.9945712956
    7      C       2.3052272571    -0.9402304204    -2.3502395133
    8      C       1.8770226435     1.1335957242    -0.4394821128
    9      N      -1.2035452852    -0.5508285398     0.9260184885
   10      C      -2.1968180024     0.0034677857     0.1785993601
   11      N      -1.9182770939     0.9095097633    -0.7206680312
   12      C      -0.6506163941     1.3135510407    -0.9124086559
   13      C       0.4431734633     0.7091480990    -0.1975216348
   14      C       0.0954614322    -0.2149813778     0.7358825917
   15      C      -3.6048424175    -0.3958222110     0.4414333885
   16      C      -4.3346126184     0.6767476021     1.2829231875
   17      C      -5.7873036164     0.2862211557     1.5317805559
   18      N      -0.4881569420     2.2922106288    -1.7951551107
   19      H       4.1667264124    -2.7076934470    -1.4683784464
   20      H       5.4686580829    -2.6168150063     0.6408457928
   21      H       5.0678549273    -0.7188982102     2.2380626156
   22      H       3.3656182032     0.9885397712     1.6363698051
   23      H       2.4978305689    -1.8684989225    -2.8849661372
   24      H       2.6360556969    -0.1193649962    -2.9953659168
   25      H       1.2273389940    -0.8502385833    -2.2006556234
```

```
   26      H       2.0217318040     1.4377461585    -1.4763524841
   27      H       2.1347144879     1.9938630945     0.1813332966
   28      H      -1.4470958719    -1.2389725838     1.6281973385
   29      H       0.8147585978    -0.7343233548     1.3577512733
   30      H      -4.1053807373    -0.5020201678    -0.5236488601
   31      H      -3.6403754139    -1.3641055310     0.9495678357
   32      H      -4.2844431718     1.6302437846     0.7502305600
   33      H      -3.8129515897     0.8100615827     2.2370128879
   34      H      -6.3310452385     0.1709432699     0.5911315998
   35      H      -6.2902799027     1.0580172825     2.1158362643
   36      H      -5.8604708839    -0.6529903937     2.0857070999
   37      H       0.3967992724     2.7141455547    -2.0221071460
   38      H      -1.3105112480     2.6385734711    -2.2730753837
 ----------------------------------------------------------------
 Nuclear Repulsion Energy =        1296.95477513 hartrees
 There are       65 alpha and       65 beta electrons

--------------------------------------------------
-  Entering fldman on Sun Oct 13 22:49:50 2024  -
--------------------------------------------------

 Applying Cartesian multipole field
    Component          Value
    ---------          -----
     (2,0,0)        1.00000E-12
     (0,2,0)        2.00000E-11
     (0,0,2)       -3.00000E-11
 Nucleus-field energy     =    -0.0000000032 hartrees

--------------------------------------------------
-  Entering gesman on Sun Oct 13 22:49:50 2024  -
--------------------------------------------------

 Requested basis set is 6-311+G(d,p)
 There are 188 shells and 516 basis functions
 A cutoff of  1.0D-12 yielded  12516 shell pairs
 There are     99852 function pairs (    106406 Cartesian)
 Smallest overlap matrix eigenvalue = 1.43E-06
 Linear dependence detected in AO basis
 Tighter screening thresholds may be required for diffuse basis sets
 Use S2THRESH > 12 and THRESH = 14 in case of SCF convergence issues
 Number of orthogonalized atomic orbitals = 510
 Maximum deviation from orthogonality = 2.637E-11
 Guess MOs from SCF MO coefficient file
 Reading MOs from coefficient file
 Reading MOs from coefficient file

--------------------------------------------------
-  Entering scfman on Sun Oct 13 22:49:50 2024  -
--------------------------------------------------

 Long-range K will be added via erf
 Coulomb attenuation parameter = 0.2 bohr**(-1)
 A restricted hybrid HF-DFT SCF calculation will be
 performed using Pulay DIIS + Geometric Direct Minimization
 Exchange:     0.2220 Hartree-Fock + 1.0000 wB97X-D + LR-HF
 Correlation:  1.0000 wB97X-D
 Using Euler-Maclaurin-Lebedev (75,302) quadrature formula
 Dispersion:   Grimme D
 SCF converges when RMS gradient is below 1.0E-07
 Geometry optimization detected.  Setting ReadMinima to 0
 Setting SaveMinima to 0
 ---------------------------------------
  Cycle       Energy         DIIS Error
 ---------------------------------------
    1    -763.9685565148      8.05E-04
    2    -763.9686117649      9.51E-05
    3    -763.9692128572      6.41E-05
    4    -763.9693266427      3.05E-05
    5    -763.9693632941      6.57E-06
```

```
    6    -763.9693653070      2.71E-06
    7    -763.9693656344      8.13E-07
    8    -763.9693656819      3.85E-07
    9    -763.9693656891      1.16E-07
   10    -763.9693656903      5.36E-08 Convergence criterion met
 ---------------------------------------
 SCF time:  CPU 240.16 s  wall 418.40 s
 SCF   energy in the final basis set = -763.96936569
 Total energy in the final basis set = -763.96936569

--------------------------------------------------
-  Entering anlman on Sun Oct 13 22:56:49 2024  -
--------------------------------------------------



 --------------------------------------------------------------

                    Orbital Energies (a.u.)
 --------------------------------------------------------------

 Alpha MOs
 -- Occupied --
-14.8204 -14.7880 -14.7368 -14.7043 -10.6753 -10.6593 -10.6455 -10.6428
-10.6383 -10.6302 -10.6064 -10.5876 -10.5761 -10.5748 -10.5528 -10.5231
-10.4905 -10.4467  -1.4144  -1.4085  -1.3461  -1.2736  -1.2326  -1.1999
 -1.1921  -1.1409  -1.1004  -1.0869  -1.0539  -1.0490  -1.0368  -0.9980
 -0.9920  -0.9531  -0.9415  -0.9110  -0.9061  -0.8912  -0.8859  -0.8622
 -0.8565  -0.8477  -0.8327  -0.8250  -0.8082  -0.7996  -0.7895  -0.7865
 -0.7659  -0.7610  -0.7557  -0.7527  -0.7478  -0.7326  -0.7209  -0.7091
 -0.6792  -0.6657  -0.6579  -0.6394  -0.6294  -0.6249  -0.6144  -0.6115
 -0.6058
 -- Virtual --
 -0.2736  -0.2573  -0.2385  -0.2285  -0.1747  -0.1656  -0.1504  -0.1452
 -0.1348  -0.1184  -0.1177  -0.1138  -0.1113  -0.1044  -0.1013  -0.0966
 -0.0908  -0.0864  -0.0795  -0.0776  -0.0758  -0.0710  -0.0693  -0.0676
 -0.0619  -0.0592  -0.0551  -0.0545  -0.0507  -0.0458  -0.0403  -0.0313
 -0.0288  -0.0260  -0.0237  -0.0208  -0.0164  -0.0137  -0.0121  -0.0075
 -0.0051   0.0002   0.0025   0.0050   0.0058   0.0114   0.0140   0.0178
  0.0204   0.0242   0.0272   0.0324   0.0337   0.0362   0.0400   0.0416
  0.0432   0.0459   0.0536   0.0553   0.0560   0.0607   0.0619   0.0668
  0.0700   0.0731   0.0760   0.0783   0.0796   0.0890   0.0914   0.0955
  0.0979   0.1005   0.1021   0.1062   0.1099   0.1121   0.1181   0.1204
  0.1243   0.1267   0.1303   0.1355   0.1381   0.1432   0.1448   0.1503
  0.1547   0.1571   0.1623   0.1685   0.1691   0.1728   0.1771   0.1785
  0.1851   0.1897   0.1935   0.2002   0.2017   0.2115   0.2153   0.2245
  0.2306   0.2411   0.2587   0.2745   0.2783   0.2943   0.3056   0.3144
  0.3258   0.3328   0.3401   0.3442   0.3526   0.3573   0.3661   0.3710
  0.3788   0.3844   0.3884   0.3946   0.3957   0.4050   0.4100   0.4200
  0.4233   0.4384   0.4399   0.4494   0.4600   0.4641   0.4713   0.4759
  0.4829   0.4865   0.4906   0.5042   0.5113   0.5148   0.5185   0.5232
  0.5244   0.5338   0.5363   0.5411   0.5439   0.5529   0.5561   0.5604
  0.5651   0.5711   0.5743   0.5830   0.5876   0.5936   0.6014   0.6059
  0.6140   0.6210   0.6259   0.6301   0.6337   0.6524   0.6555   0.6645
  0.6658   0.6771   0.6832   0.6883   0.6914   0.6946   0.7007   0.7105
  0.7154   0.7366   0.7381   0.7419   0.7611   0.7654   0.7793   0.7852
  0.7974   0.8018   0.8053   0.8243   0.8290   0.8459   0.8525   0.8584
  0.8648   0.8696   0.8881   0.8928   0.9076   0.9163   0.9202   0.9444
  0.9576   0.9598   0.9661   0.9863   0.9906   1.0217   1.0354   1.0417
  1.0502   1.0557   1.0869   1.1223   1.1321   1.1550   1.1640   1.1699
  1.1973   1.2063   1.2245   1.2294   1.2593   1.2823   1.2934   1.2990
  1.3054   1.3162   1.3285   1.3357   1.3523   1.3569   1.3710   1.3751
  1.3790   1.3874   1.3967   1.4014   1.4099   1.4192   1.4298   1.4342
  1.4425   1.4459   1.4523   1.4595   1.4677   1.4818   1.4903   1.5001
  1.5047   1.5084   1.5188   1.5293   1.5377   1.5397   1.5477   1.5551
  1.5608   1.5627   1.5695   1.5777   1.5956   1.6016   1.6143   1.6153
  1.6186   1.6258   1.6380   1.6412   1.6591   1.6654   1.6843   1.6962
  1.7136   1.7177   1.7293   1.7440   1.7474   1.7548   1.7632   1.7705
  1.7804   1.7929   1.8021   1.8256   1.8384   1.8605   1.8699   1.8846
  1.8929   1.9100   1.9241   1.9471   1.9606   1.9764   1.9917   2.0056
  2.0116   2.0163   2.0290   2.0318   2.0426   2.0591   2.0750   2.0834
```

| | | | | | | | |
|---|---|---|---|---|---|---|---|
| 2.0972 | 2.1185 | 2.1292 | 2.1528 | 2.1673 | 2.1899 | 2.2136 | 2.2187 |
| 2.2390 | 2.2473 | 2.2544 | 2.2603 | 2.2634 | 2.2706 | 2.2885 | 2.2914 |
| 2.3105 | 2.3126 | 2.3292 | 2.3547 | 2.3674 | 2.3813 | 2.3937 | 2.4038 |
| 2.4162 | 2.4345 | 2.4424 | 2.4457 | 2.4498 | 2.4633 | 2.4764 | 2.4920 |
| 2.4994 | 2.5116 | 2.5159 | 2.5246 | 2.5331 | 2.5400 | 2.5513 | 2.5636 |
| 2.5653 | 2.5753 | 2.5885 | 2.5914 | 2.5929 | 2.5977 | 2.6032 | 2.6185 |
| 2.6247 | 2.6482 | 2.6610 | 2.6687 | 2.6801 | 2.6897 | 2.7069 | 2.7130 |
| 2.7172 | 2.7227 | 2.7261 | 2.7348 | 2.7512 | 2.7586 | 2.7776 | 2.7809 |
| 2.8021 | 2.8084 | 2.8131 | 2.8446 | 2.8611 | 2.8671 | 2.8771 | 2.8860 |
| 2.9005 | 2.9062 | 2.9424 | 2.9520 | 3.0498 | 3.0562 | 3.1088 | 3.1454 |
| 3.1596 | 3.2283 | 3.2560 | 3.2655 | 3.3029 | 3.3243 | 3.3701 | 3.4075 |
| 3.4246 | 3.4773 | 3.5303 | 3.5322 | 3.5615 | 3.5941 | 3.6176 | 3.6448 |
| 3.6829 | 3.6988 | 3.7242 | 3.7344 | 3.7382 | 3.7409 | 3.7516 | 3.7663 |
| 3.7666 | 3.8272 | 3.8635 | 3.8990 | 3.9372 | 3.9826 | 4.0535 | 4.0789 |
| 4.0926 | 4.1893 | 4.2907 | 4.3121 | 4.5939 | 4.6701 | 4.6889 | 4.7734 |
| 4.9296 | 5.0308 | 5.2014 | 23.5244 | 23.5883 | 23.7203 | 23.7324 | 23.7417 |
| 23.7774 | 23.8093 | 23.8309 | 23.8406 | 23.8913 | 23.9284 | 23.9609 | 23.9825 |
| 24.0283 | 35.4116 | 35.4655 | 35.5244 | 35.6263 | | | |

--------------------------------------------------------------

Ground-State Mulliken Net Atomic Charges

| Atom | Charge (a.u.) |
|---|---|
| 1 N | 0.364641 |
| 2 C | 0.147746 |
| 3 C | -0.026835 |
| 4 C | -0.201947 |
| 5 C | -0.308646 |
| 6 C | 0.047013 |
| 7 C | -0.815946 |
| 8 C | -0.156867 |
| 9 N | -0.110366 |
| 10 C | -0.378415 |
| 11 N | 0.014154 |
| 12 C | 0.236466 |
| 13 C | -0.067899 |
| 14 C | 0.070481 |
| 15 C | -0.317160 |
| 16 C | -0.109748 |
| 17 C | -0.611494 |
| 18 N | -0.476196 |
| 19 H | 0.222453 |
| 20 H | 0.237342 |
| 21 H | 0.233844 |
| 22 H | 0.222566 |
| 23 H | 0.230310 |
| 24 H | 0.253333 |
| 25 H | 0.227225 |
| 26 H | 0.184582 |
| 27 H | 0.255884 |
| 28 H | 0.377877 |
| 29 H | 0.210446 |
| 30 H | 0.244646 |
| 31 H | 0.186779 |
| 32 H | 0.200930 |
| 33 H | 0.172604 |
| 34 H | 0.175331 |
| 35 H | 0.197508 |
| 36 H | 0.169315 |
| 37 H | 0.316719 |
| 38 H | 0.381324 |

Sum of atomic charges = 2.000000

-----------------------------------------------------------------

Cartesian Multipole Moments

-----------------------------------------------------------------

Charge (ESU x 10^10)
9.6064

```
    Dipole Moment (Debye)
         X       9.8489      Y      -0.5851      Z      -0.2988
       Tot       9.8708
    Quadrupole Moments (Debye-Ang)
        XX      -0.8346     XY     -15.8110     YY     -73.6994
        XZ      -5.2434     YZ      -9.4686     ZZ     -74.6197
    Traceless Quadrupole Moments (Debye-Ang)
       QXX     146.6498    QYY     -71.9444    QZZ     -74.7054
       QXY     -47.4329    QXZ     -15.7303    QYZ     -28.4057
    Octopole Moments (Debye-Ang^2)
       XXX      60.4368    XXY     -91.1829    XYY      22.3912
       YYY       8.9097    XXZ      72.5185    XYZ      13.2206
       YYZ     -30.6193    XZZ       3.5683    YZZ       7.3780
       ZZZ     -12.1651
    Traceless Octopole Moments (Debye-Ang^2)
       XXX     128.9859    YYY     807.7016    ZZZ    -450.0833
       XXY   -1143.0576    XXZ     998.5754    XYY      76.6789
       XYZ     198.3086    XZZ    -205.6648    YYZ    -548.4922
       YZZ     335.3560
    Hexadecapole Moments (Debye-Ang^3)
      XXXX   -4287.4172   XXXY     -66.9852   XXYY    -904.6081
      XYYY     355.8451   YYYY    -709.9496   XXXZ     394.1870
      XXYZ      26.1551   XYYZ      91.4549   YYYZ     -18.0911
      XXZZ    -965.3938   XYZZ     110.5405   YYZZ    -190.3266
      XZZZ     316.3412   YZZZ      13.0038   ZZZZ    -826.2358
    Traceless Hexadecapole Moments (Debye-Ang^3)
      XXXX   14490.5778   XXXY  -25006.4685   XXXZ    5300.4001
      XXYY   -5382.0763   XXYZ    2430.3294   XXZZ   -9108.5015
      XYYY   19390.7198   XYYZ   -2426.9835   XYZZ    5615.7487
      XZZZ   -2873.4167   YYYY   -1603.4586   YYYZ   -2847.4464
      YYZZ    6985.5349   YZZZ     417.1170   ZZZZ    2122.9666
 -----------------------------------------------------------------

-------------------------------------------------
-  Entering drvman on Sun Oct 13 22:56:49 2024  -
-------------------------------------------------

 Calculating analytic gradient of the SCF energy
 Gradient of SCF Energy
            1           2           3           4           5           6
    1  -0.0000196   0.0001650  -0.0003032  -0.0000434   0.0001128   0.0001173
    2   0.0000621   0.0000602  -0.0000488   0.0002005  -0.0002134   0.0001072
    3   0.0001836   0.0000843  -0.0001430   0.0001944  -0.0001307  -0.0002409
            7           8           9          10          11          12
    1  -0.0004819   0.0001041   0.0001828   0.0003605  -0.0002709   0.0004379
    2  -0.0000235   0.0003208   0.0000522  -0.0001395   0.0001302  -0.0017438
    3   0.0001542  -0.0000435   0.0002322   0.0001184  -0.0002910   0.0000380
           13          14          15          16          17          18
    1  -0.0003414  -0.0004007  -0.0001474   0.0001173   0.0000212  -0.0004113
    2  -0.0003841   0.0002799  -0.0000574  -0.0001194   0.0000217   0.0020248
    3  -0.0005741   0.0003105  -0.0000377   0.0000160  -0.0000482   0.0010104
           19          20          21          22          23          24
    1   0.0000773   0.0000725   0.0000489   0.0000627   0.0001825   0.0000414
    2  -0.0000915  -0.0000210  -0.0000592   0.0000446   0.0000523   0.0001330
    3   0.0000126   0.0000245   0.0000400   0.0000696  -0.0001204  -0.0000368
           25          26          27          28          29          30
    1  -0.0000139   0.0004162  -0.0000684  -0.0000551  -0.0000972  -0.0000134
    2   0.0002415  -0.0000217  -0.0000302  -0.0000488   0.0000136   0.0000533
    3   0.0000609   0.0001843   0.0000287   0.0001444   0.0000769   0.0000455
           31          32          33          34          35          36
    1  -0.0000099  -0.0000559  -0.0000254   0.0000195  -0.0000237   0.0000138
    2  -0.0000137   0.0000542   0.0000276  -0.0000179   0.0000271  -0.0000121
    3   0.0000074  -0.0000248   0.0000113  -0.0000413  -0.0000087  -0.0000054
           37          38
    1   0.0005007  -0.0002719
    2  -0.0004937  -0.0003669
    3  -0.0005837  -0.0007179
 Max gradient component =       2.025E-03
 RMS gradient           =       3.344E-04
 Gradient time:  CPU 99.58 s  wall 181.47 s
```

```
---------------------------------------------------
-  Entering optman on Sun Oct 13 22:59:50 2024  -
---------------------------------------------------

 Geometry Optimization Parameters
   NAtoms,    NIC,     NZ,  NCons,   NDum,   NFix, NCnnct, MaxDiis
       38     272       0       0       0       0       0       0

 Cartesian Hessian Update
 Hessian updated using BFGS update


** GEOMETRY OPTIMIZATION IN DELOCALIZED INTERNAL COORDINATES **
   Searching for a Minimum

   Optimization Cycle:  11

                    Coordinates (Angstroms)
     ATOM                X               Y               Z
      1  N         2.8640518275    0.0708310852   -0.1600041584
      2  C         3.0627730708   -0.9343474474   -1.0610829338
      3  C         4.0019589031   -1.9087776692   -0.7567136083
      4  C         4.7302796856   -1.8541421383    0.4213498905
      5  C         4.5129227440   -0.8067309859    1.3132206292
      6  C         3.5757683527    0.1423651321    0.9945712956
      7  C         2.3052272571   -0.9402304204   -2.3502395133
      8  C         1.8770226435    1.1335957242   -0.4394821128
      9  N        -1.2035452852   -0.5508285398    0.9260184885
     10  C        -2.1968180024    0.0034677857    0.1785993601
     11  N        -1.9182770939    0.9095097633   -0.7206680312
     12  C        -0.6506163941    1.3135510407   -0.9124086559
     13  C         0.4431734633    0.7091480990   -0.1975216348
     14  C         0.0954614322   -0.2149813778    0.7358825917
     15  C        -3.6048424175   -0.3958222110    0.4414333885
     16  C        -4.3346126184    0.6767476021    1.2829231875
     17  C        -5.7873036164    0.2862211557    1.5317805559
     18  N        -0.4881569420    2.2922106288   -1.7951551107
     19  H         4.1667264124   -2.7076934470   -1.4683784464
     20  H         5.4686580829   -2.6168150063    0.6408457928
     21  H         5.0678549273   -0.7188982102    2.2380626156
     22  H         3.3656182032    0.9885397712    1.6363698051
     23  H         2.4978305689   -1.8684989225   -2.8849661372
     24  H         2.6360556969   -0.1193649962   -2.9953659168
     25  H         1.2273389940   -0.8502385833   -2.2006556234
     26  H         2.0217318040    1.4377461585   -1.4763524841
     27  H         2.1347144879    1.9938630945    0.1813332966
     28  H        -1.4470958719   -1.2389725838    1.6281973385
     29  H         0.8147585978   -0.7343233548    1.3577512733
     30  H        -4.1053807373   -0.5020201678   -0.5236488601
     31  H        -3.6403754139   -1.3641055310    0.9495678357
     32  H        -4.2844431718    1.6302437846    0.7502305600
     33  H        -3.8129515897    0.8100615827    2.2370128879
     34  H        -6.3310452385    0.1709432699    0.5911315998
     35  H        -6.2902799027    1.0580172825    2.1158362643
     36  H        -5.8604708839   -0.6529903937    2.0857070999
     37  H         0.3967992724    2.7141455547   -2.0221071460
     38  H        -1.3105112480    2.6385734711   -2.2730753837
   Point Group: c1    Number of degrees of freedom:   108


   Energy is   -763.969365690

 Hessian updated using BFGS update
  internal optimization (0)

108 Hessian modes will be used to form the next step
  Hessian Eigenvalues:
     0.000449    0.002742    0.003048    0.003743    0.007662    0.011593
     0.016125    0.018814    0.019220    0.019937    0.020474    0.022425
```

| | | | | | |
|---|---|---|---|---|---|
| 0.022709 | 0.023251 | 0.024038 | 0.024549 | 0.025781 | 0.026918 |
| 0.026987 | 0.028259 | 0.028917 | 0.030192 | 0.030936 | 0.036023 |
| 0.038391 | 0.041427 | 0.043224 | 0.043801 | 0.044062 | 0.047611 |
| 0.051530 | 0.053976 | 0.054500 | 0.058785 | 0.061289 | 0.077587 |
| 0.083900 | 0.093610 | 0.121997 | 0.122376 | 0.126856 | 0.128146 |
| 0.132015 | 0.133013 | 0.136609 | 0.140922 | 0.143377 | 0.144473 |
| 0.147149 | 0.147529 | 0.149942 | 0.151660 | 0.152569 | 0.152917 |
| 0.157973 | 0.161176 | 0.190836 | 0.193698 | 0.206326 | 0.216524 |
| 0.226110 | 0.229060 | 0.238431 | 0.247930 | 0.249161 | 0.263677 |
| 0.271773 | 0.275849 | 0.282605 | 0.294681 | 0.297878 | 0.300155 |
| 0.300553 | 0.301039 | 0.301510 | 0.302513 | 0.303732 | 0.304132 |
| 0.304493 | 0.304950 | 0.305978 | 0.308046 | 0.308694 | 0.312637 |
| 0.319195 | 0.329251 | 0.332267 | 0.333357 | 0.334888 | 0.337135 |
| 0.341145 | 0.344510 | 0.356177 | 0.366021 | 0.383929 | 0.389836 |
| 0.397744 | 0.406527 | 0.417735 | 0.420340 | 0.422229 | 0.436242 |
| 0.439806 | 0.448312 | 0.469380 | 0.510932 | 0.568458 | 0.720182 |

Minimum search - taking simple RFO step
Searching for Lamda that Minimizes Along All modes
Value Taken    Lamda =  -0.00074320
Calculated Step too Large.  Step scaled by  0.415942
Step Taken.  Stepsize is  0.300000

| | Maximum | Tolerance | Cnvgd? |
|---|---|---|---|
| Gradient | 0.002383 | 0.000800 | NO |
| Displacement | 0.110865 | 0.001400 | NO |
| Energy change | -0.000272 | 0.000228 | NO |

New Cartesian Coordinates Obtained by Inverse Iteration

Displacement from previous Coordinates is:  0.902405

Standard Nuclear Orientation (Angstroms)

| I | Atom | X | Y | Z |
|---|---|---|---|---|
| 1 | N | 2.8572098015 | 0.0670148658 | -0.1823842619 |
| 2 | C | 3.0862743941 | -0.9502547419 | -1.0622743796 |
| 3 | C | 4.0291860322 | -1.9092423557 | -0.7208417613 |
| 4 | C | 4.7319032229 | -1.8260730287 | 0.4709478958 |
| 5 | C | 4.4835018574 | -0.7665358829 | 1.3405049876 |
| 6 | C | 3.5423781784 | 0.1655353472 | 0.9864924032 |
| 7 | C | 2.3603810077 | -0.9861073824 | -2.3689768660 |
| 8 | C | 1.8619228768 | 1.1099351753 | -0.4981298917 |
| 9 | N | -1.2079846395 | -0.6085799364 | 0.8562493697 |
| 10 | C | -2.2089615370 | 0.0057734125 | 0.1703851356 |
| 11 | N | -1.9365322964 | 0.9562248405 | -0.6847472357 |
| 12 | C | -0.6669351556 | 1.3448365519 | -0.8884335302 |
| 13 | C | 0.4328371187 | 0.6853809739 | -0.2341270746 |
| 14 | C | 0.0930367134 | -0.2854215964 | 0.6527123085 |
| 15 | C | -3.6180251359 | -0.3828086420 | 0.4451162279 |
| 16 | C | -4.3308789546 | 0.6867788708 | 1.3036289641 |
| 17 | C | -5.7824120536 | 0.3026405187 | 1.5685244103 |
| 18 | N | -0.5039119976 | 2.3642470743 | -1.7246305736 |
| 19 | H | 4.2171212768 | -2.7185665054 | -1.4147974526 |
| 20 | H | 5.4738565202 | -2.5763281201 | 0.7191371093 |
| 21 | H | 5.0175913956 | -0.6568183241 | 2.2752234928 |
| 22 | H | 3.3069274531 | 1.0184851699 | 1.6103949108 |
| 23 | H | 2.5506616710 | -1.9347486112 | -2.8677691348 |
| 24 | H | 2.7263894391 | -0.1938684235 | -3.0309235831 |
| 25 | H | 1.2807438413 | -0.8703092196 | -2.2542023611 |
| 26 | H | 1.9951826831 | 1.3749492327 | -1.5477725172 |
| 27 | H | 2.1140092285 | 1.9961996286 | 0.0884646609 |
| 28 | H | -1.4469689930 | -1.3332209161 | 1.5222988076 |
| 29 | H | 0.8171141060 | -0.8530626915 | 1.2247120979 |
| 30 | H | -4.1301232104 | -0.4750850338 | -0.5158319904 |
| 31 | H | -3.6573425976 | -1.3565166595 | 0.9424342545 |
| 32 | H | -4.2815983620 | 1.6436809179 | 0.7772655436 |
| 33 | H | -3.7960735661 | 0.8096467756 | 2.2517065395 |
| 34 | H | -6.3387750151 | 0.1952147064 | 0.6342965573 |

```
  35      H      -6.2738557263     1.0739099821     2.1629814534
  36      H      -5.8538479208    -0.6390152837     2.1185488647
  37      H       0.3847266874     2.7772146530    -1.9547303476
  38      H      -1.3287283437     2.7448946577    -2.1714530332
 ----------------------------------------------------------------
 Nuclear Repulsion Energy =        1296.16001798 hartrees
 There are       65 alpha and       65 beta electrons

-------------------------------------------------
-  Entering fldman on Sun Oct 13 22:59:50 2024  -
-------------------------------------------------

 Applying Cartesian multipole field
    Component          Value
    ---------          -----
     (2,0,0)        1.00000E-12
     (0,2,0)        2.00000E-11
     (0,0,2)       -3.00000E-11
 Nucleus-field energy     =    -0.0000000026 hartrees

-------------------------------------------------
-  Entering gesman on Sun Oct 13 22:59:50 2024  -
-------------------------------------------------

 Requested basis set is 6-311+G(d,p)
 There are 188 shells and 516 basis functions
 A cutoff of  1.0D-12 yielded  12521 shell pairs
 There are     99958 function pairs (    106519 Cartesian)
 Smallest overlap matrix eigenvalue = 1.43E-06
 Linear dependence detected in AO basis
 Tighter screening thresholds may be required for diffuse basis sets
 Use S2THRESH > 12 and THRESH = 14 in case of SCF convergence issues
 Number of orthogonalized atomic orbitals = 510
 Maximum deviation from orthogonality = 1.645E-11
 Guess MOs from SCF MO coefficient file
 Reading MOs from coefficient file
 Reading MOs from coefficient file

-------------------------------------------------
-  Entering scfman on Sun Oct 13 22:59:51 2024  -
-------------------------------------------------

 Long-range K will be added via erf
 Coulomb attenuation parameter = 0.2 bohr**(-1)
 A restricted hybrid HF-DFT SCF calculation will be
 performed using Pulay DIIS + Geometric Direct Minimization
 Exchange:     0.2220 Hartree-Fock + 1.0000 wB97X-D + LR-HF
 Correlation:  1.0000 wB97X-D
 Using Euler-Maclaurin-Lebedev (75,302) quadrature formula
 Dispersion:   Grimme D
 SCF converges when RMS gradient is below 1.0E-07
 Geometry optimization detected.  Setting ReadMinima to 0
 Setting SaveMinima to 0
 ---------------------------------------
  Cycle       Energy         DIIS Error
 ---------------------------------------
    1    -763.9655361541      8.82E-04
    2    -763.9687697739      1.05E-04
    3    -763.9694775954      7.00E-05
    4    -763.9696142315      3.29E-05
    5    -763.9696564044      7.86E-06
    6    -763.9696592114      3.01E-06
    7    -763.9696596239      9.03E-07
    8    -763.9696596832      4.32E-07
    9    -763.9696596926      1.29E-07
   10    -763.9696596941      6.17E-08 Convergence criterion met
 ---------------------------------------
 SCF time:  CPU 233.67 s  wall 417.29 s
 SCF   energy in the final basis set = -763.96965969
 Total energy in the final basis set = -763.96965969
```

```
--------------------------------------------------
-  Entering anlman on Sun Oct 13 23:06:48 2024  -
--------------------------------------------------


 --------------------------------------------------------------

                    Orbital Energies (a.u.)
 --------------------------------------------------------------

 Alpha MOs
 -- Occupied --
-14.8207 -14.7878 -14.7366 -14.7040 -10.6750 -10.6592 -10.6452 -10.6433
-10.6384 -10.6304 -10.6065 -10.5880 -10.5764 -10.5751 -10.5527 -10.5230
-10.4896 -10.4462  -1.4145  -1.4087  -1.3458  -1.2734  -1.2328  -1.1999
 -1.1924  -1.1409  -1.1005  -1.0871  -1.0540  -1.0487  -1.0370  -0.9977
 -0.9921  -0.9530  -0.9413  -0.9107  -0.9057  -0.8907  -0.8865  -0.8619
 -0.8560  -0.8480  -0.8336  -0.8251  -0.8076  -0.8001  -0.7902  -0.7868
 -0.7658  -0.7608  -0.7553  -0.7526  -0.7474  -0.7328  -0.7209  -0.7085
 -0.6792  -0.6655  -0.6576  -0.6389  -0.6292  -0.6252  -0.6141  -0.6109
 -0.6056
 -- Virtual --
 -0.2740  -0.2568  -0.2379  -0.2285  -0.1749  -0.1656  -0.1506  -0.1454
 -0.1353  -0.1179  -0.1168  -0.1134  -0.1110  -0.1046  -0.1015  -0.0963
 -0.0911  -0.0869  -0.0801  -0.0778  -0.0757  -0.0712  -0.0697  -0.0677
 -0.0619  -0.0597  -0.0551  -0.0545  -0.0512  -0.0460  -0.0395  -0.0306
 -0.0292  -0.0267  -0.0229  -0.0205  -0.0169  -0.0134  -0.0120  -0.0075
 -0.0044  -0.0003   0.0022   0.0039   0.0065   0.0118   0.0140   0.0168
  0.0198   0.0248   0.0270   0.0313   0.0350   0.0372   0.0406   0.0409
  0.0436   0.0462   0.0531   0.0542   0.0569   0.0605   0.0622   0.0663
  0.0696   0.0723   0.0757   0.0777   0.0803   0.0879   0.0913   0.0957
  0.0984   0.0995   0.1016   0.1064   0.1109   0.1138   0.1175   0.1211
  0.1253   0.1276   0.1300   0.1352   0.1372   0.1427   0.1448   0.1510
  0.1537   0.1565   0.1625   0.1674   0.1684   0.1712   0.1773   0.1793
  0.1846   0.1892   0.1933   0.1976   0.2042   0.2104   0.2165   0.2252
  0.2311   0.2407   0.2593   0.2769   0.2815   0.2970   0.3050   0.3149
  0.3243   0.3341   0.3395   0.3435   0.3539   0.3583   0.3621   0.3708
  0.3760   0.3843   0.3892   0.3899   0.3973   0.4044   0.4073   0.4215
  0.4227   0.4373   0.4375   0.4480   0.4598   0.4643   0.4731   0.4783
  0.4822   0.4845   0.4917   0.5049   0.5112   0.5140   0.5176   0.5229
  0.5247   0.5334   0.5388   0.5425   0.5440   0.5520   0.5572   0.5602
  0.5637   0.5704   0.5746   0.5831   0.5867   0.5941   0.6012   0.6036
  0.6121   0.6222   0.6261   0.6279   0.6337   0.6518   0.6554   0.6636
  0.6681   0.6748   0.6816   0.6884   0.6902   0.6955   0.6984   0.7099
  0.7140   0.7371   0.7429   0.7449   0.7611   0.7660   0.7758   0.7828
  0.7995   0.8020   0.8058   0.8225   0.8301   0.8405   0.8516   0.8567
  0.8659   0.8676   0.8898   0.8943   0.9073   0.9144   0.9214   0.9498
  0.9563   0.9632   0.9679   0.9858   0.9929   1.0171   1.0388   1.0442
  1.0478   1.0600   1.0892   1.1178   1.1338   1.1546   1.1627   1.1742
  1.1965   1.2053   1.2262   1.2328   1.2558   1.2777   1.2898   1.2949
  1.3066   1.3157   1.3278   1.3343   1.3532   1.3581   1.3728   1.3738
  1.3803   1.3885   1.3982   1.3998   1.4076   1.4174   1.4296   1.4313
  1.4406   1.4425   1.4522   1.4584   1.4680   1.4791   1.4892   1.4999
  1.5057   1.5101   1.5216   1.5287   1.5370   1.5430   1.5465   1.5555
  1.5585   1.5636   1.5704   1.5775   1.5931   1.6014   1.6143   1.6184
  1.6200   1.6245   1.6375   1.6429   1.6613   1.6676   1.6813   1.6986
  1.7150   1.7183   1.7304   1.7465   1.7489   1.7542   1.7632   1.7711
  1.7803   1.7922   1.8016   1.8232   1.8380   1.8612   1.8679   1.8840
  1.8910   1.9092   1.9281   1.9476   1.9627   1.9750   1.9959   2.0087
  2.0132   2.0174   2.0305   2.0335   2.0456   2.0602   2.0786   2.0839
  2.0987   2.1196   2.1283   2.1521   2.1689   2.1883   2.2121   2.2186
  2.2401   2.2468   2.2551   2.2635   2.2650   2.2713   2.2885   2.2911
  2.3076   2.3120   2.3312   2.3525   2.3705   2.3816   2.3931   2.4048
  2.4155   2.4367   2.4406   2.4444   2.4510   2.4648   2.4749   2.4923
  2.5015   2.5103   2.5176   2.5235   2.5349   2.5390   2.5525   2.5582
  2.5671   2.5743   2.5873   2.5907   2.5930   2.5975   2.6005   2.6186
  2.6282   2.6471   2.6579   2.6677   2.6814   2.6925   2.7080   2.7139
  2.7162   2.7241   2.7287   2.7363   2.7544   2.7579   2.7770   2.7818
  2.8008   2.8097   2.8117   2.8418   2.8556   2.8652   2.8784   2.8882
```

```
  2.9012   2.9086   2.9445   2.9533   3.0512   3.0568   3.1058   3.1450
  3.1601   3.2284   3.2567   3.2662   3.3001   3.3249   3.3707   3.4075
  3.4251   3.4776   3.5308   3.5325   3.5630   3.5924   3.6195   3.6463
  3.6815   3.6979   3.7256   3.7324   3.7371   3.7411   3.7479   3.7660
  3.7675   3.8261   3.8632   3.8987   3.9371   3.9815   4.0542   4.0761
  4.0935   4.1910   4.2899   4.3145   4.5944   4.6703   4.6866   4.7824
  4.9293   5.0298   5.2038  23.5275  23.5886  23.7219  23.7315  23.7417
 23.7760  23.8094  23.8296  23.8404  23.8917  23.9289  23.9605  23.9836
 24.0278  35.4115  35.4657  35.5253  35.6277
--------------------------------------------------------------

         Ground-State Mulliken Net Atomic Charges

    Atom                 Charge (a.u.)
  ----------------------------------------
      1 N                     0.350291
      2 C                     0.148025
      3 C                    -0.036159
      4 C                    -0.197407
      5 C                    -0.305852
      6 C                     0.047483
      7 C                    -0.812853
      8 C                    -0.167505
      9 N                    -0.109386
     10 C                    -0.360706
     11 N                     0.003329
     12 C                     0.251311
     13 C                    -0.059352
     14 C                     0.051915
     15 C                    -0.306350
     16 C                    -0.117616
     17 C                    -0.610999
     18 N                    -0.474541
     19 H                     0.222808
     20 H                     0.237518
     21 H                     0.233988
     22 H                     0.223011
     23 H                     0.229286
     24 H                     0.257283
     25 H                     0.222444
     26 H                     0.196372
     27 H                     0.255540
     28 H                     0.374628
     29 H                     0.210730
     30 H                     0.246012
     31 H                     0.183889
     32 H                     0.201216
     33 H                     0.172700
     34 H                     0.174946
     35 H                     0.197346
     36 H                     0.169334
     37 H                     0.317610
     38 H                     0.379708
  ----------------------------------------
  Sum of atomic charges =     2.000000

-----------------------------------------------------------------
                    Cartesian Multipole Moments
-----------------------------------------------------------------
    Charge (ESU x 10^10)
                9.6064
    Dipole Moment (Debye)
         X       9.8642      Y      -0.6647      Z      -0.4578
       Tot       9.8972
    Quadrupole Moments (Debye-Ang)
        XX      -0.8616     XY     -15.8989     YY     -72.6254
        XZ      -5.3079     YZ      -9.4753     ZZ     -75.5691
    Traceless Quadrupole Moments (Debye-Ang)
       QXX     146.4713    QYY     -68.8201    QZZ     -77.6512
       QXY     -47.6967    QXZ     -15.9237    QYZ     -28.4260
```

```
   Octopole Moments (Debye-Ang^2)
      XXX      61.2035    XXY     -91.0268    XYY      21.1717
      YYY      11.0083    XXZ      72.2348    XYZ      12.9772
      YYZ     -30.7791    XZZ       5.5494    YZZ       6.2613
      ZZZ     -11.8138
   Traceless Octopole Moments (Debye-Ang^2)
      XXX     126.7312    YYY     828.9390    ZZZ    -443.9840
      XXY   -1144.1299    XXZ     994.5956    XYY      53.8012
      XYZ     194.6580    XZZ    -180.5324    YYZ    -550.6116
      YZZ     315.1909
   Hexadecapole Moments (Debye-Ang^3)
     XXXX   -4306.6951   XXXY     -58.8352   XXYY    -910.5040
     XYYY     360.9459   YYYY    -714.8497   XXXZ     398.8990
     XXYZ      25.3627   XYYZ      95.1608   YYYZ     -26.4761
     XXZZ    -959.4483   XYZZ     111.1724   YYZZ    -193.5205
     XZZZ     320.6554   YZZZ      19.3617   ZZZZ    -826.6974
   Traceless Hexadecapole Moments (Debye-Ang^3)
     XXXX   13918.5957   XXXY  -24775.4374   XXXZ    5222.2147
     XXYY   -5595.6610   XXYZ    2394.7817   XXZZ   -8322.9348
     XYYY   19301.5774   XYYZ   -2228.8468   XYZZ    5473.8600
     XZZZ   -2993.3679   YYYY   -1137.2341   YYYZ   -3584.8844
     YYZZ    6732.8950   YZZZ    1190.1027   ZZZZ    1590.0397
 -----------------------------------------------------------------

-------------------------------------------------
-  Entering drvman on Sun Oct 13 23:06:48 2024  -
-------------------------------------------------

 Calculating analytic gradient of the SCF energy
 Gradient of SCF Energy
           1           2           3           4           5           6
    1  -0.0000353  -0.0000374  -0.0003550  -0.0000141   0.0001201   0.0003353
    2   0.0000808   0.0003556  -0.0001237   0.0001524  -0.0002482   0.0000361
    3   0.0003350  -0.0000974  -0.0001860   0.0000922  -0.0001283  -0.0001204
           7           8           9          10          11          12
    1  -0.0005088   0.0001790   0.0000283   0.0005835  -0.0001756   0.0003690
    2  -0.0000489   0.0003265   0.0003040  -0.0004073   0.0003909  -0.0021926
    3   0.0002806  -0.0001584  -0.0000213   0.0005590  -0.0003896   0.0005215
          13          14          15          16          17          18
    1  -0.0005119  -0.0002951  -0.0001253   0.0001226   0.0000145  -0.0001547
    2  -0.0006387   0.0002904   0.0000109  -0.0001146   0.0000262   0.0023023
    3  -0.0006759   0.0004162  -0.0000238  -0.0000578  -0.0000349   0.0010027
          19          20          21          22          23          24
    1   0.0000391   0.0000615   0.0000725   0.0000624   0.0003074   0.0001388
    2  -0.0000999  -0.0000058  -0.0000546   0.0000311   0.0000441  -0.0000165
    3   0.0000243   0.0000306   0.0000249   0.0000980  -0.0001344  -0.0000329
          25          26          27          28          29          30
    1  -0.0000600   0.0001863  -0.0001468  -0.0000742  -0.0001392  -0.0000983
    2   0.0003048   0.0000543   0.0001247  -0.0000823  -0.0000993   0.0000379
    3  -0.0000769   0.0001254   0.0000484   0.0001023   0.0000553   0.0000491
          31          32          33          34          35          36
    1  -0.0000118  -0.0000517  -0.0000339   0.0000167  -0.0000209   0.0000138
    2  -0.0000236   0.0000350   0.0000321  -0.0000316   0.0000311  -0.0000073
    3  -0.0000235  -0.0000450  -0.0000021  -0.0000455  -0.0000133   0.0000066
          37          38
    1   0.0005477  -0.0003486
    2  -0.0002834  -0.0004930
    3  -0.0005584  -0.0009462
 Max gradient component =       2.302E-03
 RMS gradient           =       3.964E-04
 Gradient time:  CPU 107.06 s  wall 181.69 s

-------------------------------------------------
-  Entering optman on Sun Oct 13 23:09:50 2024  -
-------------------------------------------------

 Geometry Optimization Parameters
   NAtoms,     NIC,      NZ,  NCons,    NDum,    NFix, NCnnct, MaxDiis
       38     272        0        0        0        0        0        0
```

```
 Cartesian Hessian Update
 Hessian updated using BFGS update


** GEOMETRY OPTIMIZATION IN DELOCALIZED INTERNAL COORDINATES **
   Searching for a Minimum

   Optimization Cycle:  12

                    Coordinates (Angstroms)
     ATOM              X               Y               Z
      1  N        2.8572098015    0.0670148658   -0.1823842619
      2  C        3.0862743941   -0.9502547419   -1.0622743796
      3  C        4.0291860322   -1.9092423557   -0.7208417613
      4  C        4.7319032229   -1.8260730287    0.4709478958
      5  C        4.4835018574   -0.7665358829    1.3405049876
      6  C        3.5423781784    0.1655353472    0.9864924032
      7  C        2.3603810077   -0.9861073824   -2.3689768660
      8  C        1.8619228768    1.1099351753   -0.4981298917
      9  N       -1.2079846395   -0.6085799364    0.8562493697
     10  C       -2.2089615370    0.0057734125    0.1703851356
     11  N       -1.9365322964    0.9562248405   -0.6847472357
     12  C       -0.6669351556    1.3448365519   -0.8884335302
     13  C        0.4328371187    0.6853809739   -0.2341270746
     14  C        0.0930367134   -0.2854215964    0.6527123085
     15  C       -3.6180251359   -0.3828086420    0.4451162279
     16  C       -4.3308789546    0.6867788708    1.3036289641
     17  C       -5.7824120536    0.3026405187    1.5685244103
     18  N       -0.5039119976    2.3642470743   -1.7246305736
     19  H        4.2171212768   -2.7185665054   -1.4147974526
     20  H        5.4738565202   -2.5763281201    0.7191371093
     21  H        5.0175913956   -0.6568183241    2.2752234928
     22  H        3.3069274531    1.0184851699    1.6103949108
     23  H        2.5506616710   -1.9347486112   -2.8677691348
     24  H        2.7263894391   -0.1938684235   -3.0309235831
     25  H        1.2807438413   -0.8703092196   -2.2542023611
     26  H        1.9951826831    1.3749492327   -1.5477725172
     27  H        2.1140092285    1.9961996286    0.0884646609
     28  H       -1.4469689930   -1.3332209161    1.5222988076
     29  H        0.8171141060   -0.8530626915    1.2247120979
     30  H       -4.1301232104   -0.4750850338   -0.5158319904
     31  H       -3.6573425976   -1.3565166595    0.9424342545
     32  H       -4.2815983620    1.6436809179    0.7772655436
     33  H       -3.7960735661    0.8096467756    2.2517065395
     34  H       -6.3387750151    0.1952147064    0.6342965573
     35  H       -6.2738557263    1.0739099821    2.1629814534
     36  H       -5.8538479208   -0.6390152837    2.1185488647
     37  H        0.3847266874    2.7772146530   -1.9547303476
     38  H       -1.3287283437    2.7448946577   -2.1714530332
   Point Group: c1    Number of degrees of freedom:   108


   Energy is   -763.969659694

 Hessian updated using BFGS update
  internal optimization (0)

108 Hessian modes will be used to form the next step
  Hessian Eigenvalues:
     0.000292    0.002741    0.002946    0.003758    0.007229    0.011581
     0.015589    0.018803    0.019211    0.019995    0.020407    0.022674
     0.022716    0.023600    0.024061    0.024617    0.025788    0.026504
     0.027208    0.028252    0.028903    0.030241    0.031074    0.036021
     0.038377    0.041393    0.043153    0.043802    0.043931    0.047578
     0.050348    0.053556    0.054705    0.057871    0.062304    0.077574
     0.083891    0.094673    0.121990    0.122642    0.126856    0.128904
     0.132018    0.133057    0.136622    0.140893    0.143525    0.145226
     0.146605    0.147529    0.150631    0.151659    0.152574    0.153688
     0.158015    0.160965    0.190816    0.193640    0.206323    0.216481
     0.226115    0.229054    0.239062    0.248021    0.251552    0.267730
```

| | | | | | |
|---|---|---|---|---|---|
| 0.271790 | 0.276360 | 0.282642 | 0.293935 | 0.296939 | 0.300157 |
| 0.300514 | 0.300914 | 0.301442 | 0.302467 | 0.302873 | 0.304135 |
| 0.304375 | 0.304936 | 0.305806 | 0.308114 | 0.308878 | 0.312705 |
| 0.317434 | 0.329226 | 0.332277 | 0.333299 | 0.334882 | 0.337131 |
| 0.340879 | 0.344389 | 0.355719 | 0.366113 | 0.385003 | 0.390397 |
| 0.398191 | 0.406368 | 0.417742 | 0.420437 | 0.422170 | 0.437551 |
| 0.444236 | 0.448510 | 0.487611 | 0.516523 | 0.570930 | 0.701123 |

Minimum search - taking simple RFO step
Searching for Lamda that Minimizes Along All modes
Value Taken    Lamda =  -0.00089298
Calculated Step too Large.  Step scaled by  0.381303
Step Taken.  Stepsize is  0.300000

| | Maximum | Tolerance | Cnvgd? |
|---|---|---|---|
| Gradient | 0.002961 | 0.000800 | NO |
| Displacement | 0.116573 | 0.001400 | NO |
| Energy change | -0.000294 | 0.000228 | NO |

New Cartesian Coordinates Obtained by Inverse Iteration

Displacement from previous Coordinates is:  1.008213

Standard Nuclear Orientation (Angstroms)

| I | Atom | X | Y | Z |
|---|---|---|---|---|
| 1 | N | 2.8476661376 | 0.0613668131 | -0.2082245912 |
| 2 | C | 3.1136682626 | -0.9679036840 | -1.0630833983 |
| 3 | C | 4.0623120763 | -1.9058825212 | -0.6801820488 |
| 4 | C | 4.7337529999 | -1.7897414292 | 0.5266716184 |
| 5 | C | 4.4464839688 | -0.7190856567 | 1.3707230519 |
| 6 | C | 3.5003290130 | 0.1907821695 | 0.9764032043 |
| 7 | C | 2.4219554374 | -1.0407258871 | -2.3866350794 |
| 8 | C | 1.8441074595 | 1.0814429082 | -0.5643674320 |
| 9 | N | -1.2196111215 | -0.6661598635 | 0.7727790670 |
| 10 | C | -2.2240293657 | 0.0097585710 | 0.1549855043 |
| 11 | N | -1.9534759946 | 1.0043402636 | -0.6500475194 |
| 12 | C | -0.6817634169 | 1.3751390152 | -0.8666455562 |
| 13 | C | 0.4198847342 | 0.6589423034 | -0.2785885620 |
| 14 | C | 0.0837007386 | -0.3578299145 | 0.5556420010 |
| 15 | C | -3.6345056244 | -0.3647573637 | 0.4434231863 |
| 16 | C | -4.3221090883 | 0.6939150293 | 1.3343604867 |
| 17 | C | -5.7733910058 | 0.3201419132 | 1.6148376705 |
| 18 | N | -0.5133157927 | 2.4338545559 | -1.6520456111 |
| 19 | H | 4.2788223802 | -2.7251332462 | -1.3538864703 |
| 20 | H | 5.4805503353 | -2.5235984735 | 0.8071097735 |
| 21 | H | 4.9547649307 | -0.5843439465 | 2.3164621286 |
| 22 | H | 3.2343908559 | 1.0496010639 | 1.5797565925 |
| 23 | H | 2.6213046184 | -2.0050106131 | -2.8507479694 |
| 24 | H | 2.8120041330 | -0.2712477426 | -3.0616894216 |
| 25 | H | 1.3402270950 | -0.9151384845 | -2.3064580276 |
| 26 | H | 1.9674631564 | 1.3050153048 | -1.6254303047 |
| 27 | H | 2.0893360613 | 1.9930743226 | -0.0138666536 |
| 28 | H | -1.4576719333 | -1.4263043021 | 1.3983120271 |
| 29 | H | 0.8091879951 | -0.9737123134 | 1.0732852481 |
| 30 | H | -4.1621844530 | -0.4293755411 | -0.5118473419 |
| 31 | H | -3.6799206880 | -1.3493504578 | 0.9182763594 |
| 32 | H | -4.2700107355 | 1.6604916140 | 0.8265551979 |
| 33 | H | -3.7710195528 | 0.7914892128 | 2.2759137495 |
| 34 | H | -6.3456342340 | 0.2357622037 | 0.6878147315 |
| 35 | H | -6.2472321126 | 1.0853550825 | 2.2309876582 |
| 36 | H | -5.8465749389 | -0.6305915033 | 2.1488001134 |
| 37 | H | 0.3792597103 | 2.8397845275 | -1.8802300067 |
| 38 | H | -1.3387220415 | 2.8556360700 | -2.0591233756 |

Nuclear Repulsion Energy =        1295.35972158 hartrees
There are       65 alpha and       65 beta electrons

```
-  Entering fldman on Sun Oct 13 23:09:50 2024  -
-------------------------------------------------

 Applying Cartesian multipole field
    Component          Value
    ---------          -----
     (2,0,0)        1.00000E-12
     (0,2,0)        2.00000E-11
     (0,0,2)       -3.00000E-11
 Nucleus-field energy     =    -0.0000000021 hartrees

-------------------------------------------------
-  Entering gesman on Sun Oct 13 23:09:50 2024  -
-------------------------------------------------

 Requested basis set is 6-311+G(d,p)
 There are 188 shells and 516 basis functions
 A cutoff of  1.0D-12 yielded  12508 shell pairs
 There are     99760 function pairs (    106298 Cartesian)
 Smallest overlap matrix eigenvalue = 1.43E-06
 Linear dependence detected in AO basis
 Tighter screening thresholds may be required for diffuse basis sets
 Use S2THRESH > 12 and THRESH = 14 in case of SCF convergence issues
 Number of orthogonalized atomic orbitals = 510
 Maximum deviation from orthogonality = 2.267E-11
 Guess MOs from SCF MO coefficient file
 Reading MOs from coefficient file
 Reading MOs from coefficient file

-------------------------------------------------
-  Entering scfman on Sun Oct 13 23:09:50 2024  -
-------------------------------------------------

 Long-range K will be added via erf
 Coulomb attenuation parameter = 0.2 bohr**(-1)
 A restricted hybrid HF-DFT SCF calculation will be
 performed using Pulay DIIS + Geometric Direct Minimization
 Exchange:     0.2220 Hartree-Fock + 1.0000 wB97X-D + LR-HF
 Correlation:  1.0000 wB97X-D
 Using Euler-Maclaurin-Lebedev (75,302) quadrature formula
 Dispersion:   Grimme D
 SCF converges when RMS gradient is below 1.0E-07
 Geometry optimization detected.  Setting ReadMinima to 0
 Setting SaveMinima to 0
 ---------------------------------------
  Cycle       Energy         DIIS Error
 ---------------------------------------
    1    -763.9623680906      9.68E-04
    2    -763.9689293161      1.15E-04
    3    -763.9697663969      7.61E-05
    4    -763.9699292648      3.57E-05
    5    -763.9699781058      9.14E-06
    6    -763.9699817854      3.38E-06
    7    -763.9699823030      1.02E-06
    8    -763.9699823764      4.87E-07
    9    -763.9699823890      1.38E-07
   10    -763.9699823907      6.79E-08 Convergence criterion met
 ---------------------------------------
 SCF time:  CPU 233.28 s  wall 426.43 s
 SCF   energy in the final basis set = -763.96998239
 Total energy in the final basis set = -763.96998239

-------------------------------------------------
-  Entering anlman on Sun Oct 13 23:16:56 2024  -
-------------------------------------------------


 --------------------------------------------------------------

                    Orbital Energies (a.u.)
```

```
--------------------------------------------------------------

 Alpha MOs
 -- Occupied --
-14.8210 -14.7877 -14.7364 -14.7037 -10.6746 -10.6590 -10.6450 -10.6437
-10.6386 -10.6306 -10.6065 -10.5884 -10.5765 -10.5754 -10.5525 -10.5229
-10.4889 -10.4458  -1.4146  -1.4089  -1.3456  -1.2732  -1.2330  -1.2000
 -1.1926  -1.1410  -1.1005  -1.0871  -1.0542  -1.0484  -1.0372  -0.9975
 -0.9922  -0.9529  -0.9412  -0.9105  -0.9052  -0.8903  -0.8871  -0.8618
 -0.8554  -0.8482  -0.8345  -0.8251  -0.8070  -0.8008  -0.7910  -0.7869
 -0.7656  -0.7606  -0.7550  -0.7526  -0.7469  -0.7330  -0.7210  -0.7079
 -0.6791  -0.6653  -0.6573  -0.6385  -0.6292  -0.6254  -0.6138  -0.6104
 -0.6055
 -- Virtual --
 -0.2743  -0.2563  -0.2373  -0.2284  -0.1751  -0.1656  -0.1509  -0.1456
 -0.1358  -0.1178  -0.1158  -0.1132  -0.1104  -0.1047  -0.1013  -0.0959
 -0.0915  -0.0872  -0.0811  -0.0781  -0.0755  -0.0711  -0.0699  -0.0677
 -0.0623  -0.0597  -0.0556  -0.0543  -0.0514  -0.0459  -0.0386  -0.0307
 -0.0290  -0.0275  -0.0221  -0.0207  -0.0172  -0.0131  -0.0119  -0.0078
 -0.0036  -0.0010   0.0017   0.0034   0.0079   0.0118   0.0142   0.0157
  0.0183   0.0253   0.0273   0.0309   0.0359   0.0380   0.0398   0.0404
  0.0440   0.0473   0.0504   0.0547   0.0582   0.0604   0.0624   0.0659
  0.0691   0.0713   0.0748   0.0776   0.0822   0.0861   0.0905   0.0961
  0.0984   0.0993   0.1016   0.1070   0.1110   0.1155   0.1167   0.1218
  0.1258   0.1276   0.1304   0.1349   0.1362   0.1431   0.1450   0.1504
  0.1536   0.1564   0.1622   0.1666   0.1683   0.1699   0.1768   0.1803
  0.1846   0.1859   0.1933   0.1959   0.2058   0.2091   0.2162   0.2267
  0.2321   0.2404   0.2596   0.2790   0.2847   0.2969   0.3038   0.3151
  0.3217   0.3340   0.3402   0.3439   0.3521   0.3609   0.3616   0.3698
  0.3733   0.3844   0.3876   0.3895   0.3992   0.4031   0.4092   0.4228
  0.4231   0.4350   0.4365   0.4466   0.4600   0.4652   0.4748   0.4784
  0.4834   0.4845   0.4920   0.5069   0.5099   0.5152   0.5173   0.5227
  0.5257   0.5324   0.5422   0.5428   0.5451   0.5511   0.5579   0.5595
  0.5619   0.5700   0.5749   0.5839   0.5852   0.5936   0.5963   0.6044
  0.6108   0.6227   0.6265   0.6286   0.6341   0.6499   0.6563   0.6620
  0.6699   0.6723   0.6805   0.6856   0.6909   0.6957   0.6976   0.7084
  0.7131   0.7376   0.7459   0.7507   0.7605   0.7670   0.7726   0.7803
  0.8007   0.8023   0.8089   0.8220   0.8309   0.8364   0.8470   0.8567
  0.8649   0.8681   0.8913   0.8952   0.9077   0.9140   0.9215   0.9511
  0.9582   0.9677   0.9699   0.9868   0.9970   1.0115   1.0378   1.0409
  1.0522   1.0619   1.0919   1.1125   1.1356   1.1522   1.1618   1.1796
  1.1959   1.2033   1.2268   1.2348   1.2525   1.2711   1.2877   1.2941
  1.3076   1.3159   1.3273   1.3343   1.3541   1.3590   1.3719   1.3752
  1.3825   1.3896   1.3957   1.4007   1.4053   1.4154   1.4276   1.4298
  1.4404   1.4417   1.4521   1.4586   1.4688   1.4767   1.4881   1.4995
  1.5063   1.5120   1.5198   1.5307   1.5368   1.5439   1.5484   1.5550
  1.5580   1.5651   1.5716   1.5779   1.5898   1.6008   1.6134   1.6185
  1.6224   1.6243   1.6381   1.6448   1.6633   1.6702   1.6790   1.7022
  1.7155   1.7190   1.7315   1.7467   1.7526   1.7536   1.7630   1.7719
  1.7805   1.7904   1.8008   1.8222   1.8373   1.8620   1.8666   1.8822
  1.8922   1.9089   1.9321   1.9490   1.9661   1.9734   1.9992   2.0102
  2.0137   2.0199   2.0318   2.0362   2.0490   2.0626   2.0823   2.0836
  2.1015   2.1202   2.1275   2.1495   2.1700   2.1871   2.2113   2.2192
  2.2419   2.2469   2.2563   2.2650   2.2680   2.2712   2.2878   2.2906
  2.3053   2.3110   2.3347   2.3495   2.3744   2.3816   2.3924   2.4059
  2.4154   2.4373   2.4380   2.4459   2.4519   2.4650   2.4759   2.4920
  2.5023   2.5082   2.5187   2.5222   2.5349   2.5394   2.5524   2.5551
  2.5689   2.5738   2.5856   2.5893   2.5938   2.5979   2.6000   2.6182
  2.6313   2.6456   2.6553   2.6668   2.6822   2.6952   2.7078   2.7128
  2.7186   2.7224   2.7312   2.7390   2.7567   2.7636   2.7757   2.7855
  2.8015   2.8103   2.8121   2.8380   2.8493   2.8688   2.8788   2.8910
  2.8999   2.9117   2.9471   2.9554   3.0486   3.0603   3.1062   3.1450
  3.1628   3.2296   3.2578   3.2678   3.2975   3.3254   3.3710   3.4084
  3.4262   3.4784   3.5313   3.5327   3.5650   3.5914   3.6203   3.6473
  3.6798   3.6972   3.7266   3.7311   3.7364   3.7415   3.7449   3.7654
  3.7678   3.8245   3.8632   3.8985   3.9370   3.9801   4.0552   4.0741
  4.0949   4.1928   4.2887   4.3169   4.5952   4.6702   4.6847   4.7912
  4.9291   5.0292   5.2067  23.5309  23.5891  23.7238  23.7312  23.7418
 23.7748  23.8094  23.8283  23.8404  23.8924  23.9294  23.9606  23.9848
 24.0276  35.4120  35.4660  35.5266  35.6292
```

Ground-State Mulliken Net Atomic Charges

| Atom | Charge (a.u.) |
|---|---|
| 1 N | 0.339208 |
| 2 C | 0.157436 |
| 3 C | -0.050596 |
| 4 C | -0.197425 |
| 5 C | -0.289079 |
| 6 C | 0.020179 |
| 7 C | -0.809971 |
| 8 C | -0.179506 |
| 9 N | -0.109852 |
| 10 C | -0.344125 |
| 11 N | -0.004651 |
| 12 C | 0.267657 |
| 13 C | -0.043535 |
| 14 C | 0.039909 |
| 15 C | -0.298868 |
| 16 C | -0.124702 |
| 17 C | -0.609813 |
| 18 N | -0.475581 |
| 19 H | 0.222968 |
| 20 H | 0.237724 |
| 21 H | 0.234049 |
| 22 H | 0.223291 |
| 23 H | 0.228621 |
| 24 H | 0.260021 |
| 25 H | 0.217102 |
| 26 H | 0.210650 |
| 27 H | 0.253567 |
| 28 H | 0.372084 |
| 29 H | 0.210972 |
| 30 H | 0.247184 |
| 31 H | 0.181927 |
| 32 H | 0.201267 |
| 33 H | 0.172586 |
| 34 H | 0.174671 |
| 35 H | 0.197160 |
| 36 H | 0.169355 |
| 37 H | 0.319715 |
| 38 H | 0.378401 |

Sum of atomic charges = 2.000000

Cartesian Multipole Moments

Charge (ESU x 10^10)
9.6064

Dipole Moment (Debye)

| | | | | | |
|---|---|---|---|---|---|
| X | 9.8565 | Y | -0.7319 | Z | -0.6265 |
| Tot | 9.9035 | | | | |

Quadrupole Moments (Debye-Ang)

| | | | | | |
|---|---|---|---|---|---|
| XX | -0.9072 | XY | -15.9602 | YY | -71.4851 |
| XZ | -5.4428 | YZ | -9.3001 | ZZ | -76.5841 |

Traceless Quadrupole Moments (Debye-Ang)

| | | | | | |
|---|---|---|---|---|---|
| QXX | 146.2547 | QYY | -65.4789 | QZZ | -80.7758 |
| QXY | -47.8806 | QXZ | -16.3284 | QYZ | -27.9004 |

Octopole Moments (Debye-Ang^2)

| | | | | | |
|---|---|---|---|---|---|
| XXX | 61.8297 | XXY | -90.9655 | XYY | 19.7399 |
| YYY | 13.5551 | XXZ | 72.0599 | XYZ | 12.6521 |
| YYZ | -30.8012 | XZZ | 7.7352 | YZZ | 5.0831 |
| ZZZ | -10.6952 | | | | |

Traceless Octopole Moments (Debye-Ang^2)

| | | | | | |
|---|---|---|---|---|---|
| XXX | 123.7026 | YYY | 854.2723 | ZZZ | -435.4999 |
| XXY | -1147.5002 | XXZ | 989.2082 | XYY | 28.1842 |
| XYZ | 189.7816 | XZZ | -151.8868 | YYZ | -553.7083 |

```
       YZZ     293.2279
    Hexadecapole Moments (Debye-Ang^3)
      XXXX   -4324.0560   XXXY     -50.9107   XXYY    -916.7784
      XYYY     366.2927   YYYY    -716.7626   XXXZ     403.2457
      XXYZ      24.7217   XYYZ      98.7632   YYYZ     -36.3258
      XXZZ    -952.4597   XYZZ     111.4761   YYZZ    -198.7884
      XZZZ     326.0568   YZZZ      25.4529   ZZZZ    -829.3471
    Traceless Hexadecapole Moments (Debye-Ang^3)
      XXXX   13314.6187   XXXY  -24554.2385   XXXZ    5077.8384
      XXYY   -5896.0343   XXYZ    2394.8371   XXZZ   -7418.5844
      XYYY   19252.1166   XYYZ   -2050.8439   XYZZ    5302.1220
      XZZZ   -3026.9945   YYYY    -406.3936   YYYZ   -4417.0251
      YYZZ    6302.4279   YZZZ    2022.1880   ZZZZ    1116.1565
 -----------------------------------------------------------------

-------------------------------------------------
-  Entering drvman on Sun Oct 13 23:16:57 2024  -
-------------------------------------------------

 Calculating analytic gradient of the SCF energy
 Gradient of SCF Energy
           1           2           3           4           5           6
    1  -0.0000805  -0.0002470  -0.0003923   0.0000183   0.0000702   0.0005664
    2   0.0000546   0.0006039  -0.0001778   0.0001073  -0.0001787  -0.0001149
    3   0.0004583  -0.0002323  -0.0002498  -0.0000200  -0.0000653  -0.0000336
           7           8           9          10          11          12
    1  -0.0004707   0.0001770  -0.0002098   0.0008109   0.0000080   0.0002218
    2  -0.0000985   0.0004252   0.0006371  -0.0008144   0.0006116  -0.0024246
    3   0.0003291  -0.0002016  -0.0003137   0.0008959  -0.0004408   0.0008159
          13          14          15          16          17          18
    1  -0.0004543  -0.0001356  -0.0000830   0.0000371   0.0000104   0.0001763
    2  -0.0009279   0.0002066   0.0002196  -0.0001238   0.0000427   0.0022543
    3  -0.0007323   0.0005038   0.0001441  -0.0001656  -0.0000023   0.0008506
          19          20          21          22          23          24
    1  -0.0000339   0.0000301   0.0000739   0.0000628   0.0003967   0.0002084
    2  -0.0000840  -0.0000033  -0.0000325   0.0000039   0.0000300  -0.0000998
    3   0.0000082   0.0000104   0.0000028   0.0001336  -0.0001347   0.0000167
          25          26          27          28          29          30
    1  -0.0001715  -0.0000308  -0.0002413  -0.0000903  -0.0001127  -0.0001828
    2   0.0004806  -0.0000631   0.0002630  -0.0001264  -0.0001873   0.0000150
    3  -0.0000670  -0.0000750   0.0001116   0.0000675   0.0000193   0.0000258
          31          32          33          34          35          36
    1   0.0000205  -0.0000280  -0.0000556   0.0000121  -0.0000283   0.0000145
    2  -0.0000322  -0.0000039   0.0000449  -0.0000481   0.0000310   0.0000028
    3  -0.0000711  -0.0000702  -0.0000240  -0.0000488  -0.0000301   0.0000244
          37          38
    1   0.0005298  -0.0003969
    2   0.0000171  -0.0005102
    3  -0.0004167  -0.0010231
 Max gradient component =       2.425E-03
 RMS gradient           =       4.431E-04
 Gradient time:  CPU 101.77 s  wall 180.55 s

-------------------------------------------------
-  Entering optman on Sun Oct 13 23:19:57 2024  -
-------------------------------------------------


 Geometry Optimization Parameters
   NAtoms,    NIC,     NZ,  NCons,   NDum,   NFix, NCnnct, MaxDiis
       38     272       0       0       0       0       0       0

 Cartesian Hessian Update
 Hessian updated using BFGS update


** GEOMETRY OPTIMIZATION IN DELOCALIZED INTERNAL COORDINATES **
   Searching for a Minimum

   Optimization Cycle:  13
```

| ATOM | | Coordinates (Angstroms) X | Y | Z |
|---|---|---|---|---|
| 1 | N | 2.8476661376 | 0.0613668131 | -0.2082245912 |
| 2 | C | 3.1136682626 | -0.9679036840 | -1.0630833983 |
| 3 | C | 4.0623120763 | -1.9058825212 | -0.6801820488 |
| 4 | C | 4.7337529999 | -1.7897414292 | 0.5266716184 |
| 5 | C | 4.4464839688 | -0.7190856567 | 1.3707230519 |
| 6 | C | 3.5003290130 | 0.1907821695 | 0.9764032043 |
| 7 | C | 2.4219554374 | -1.0407258871 | -2.3866350794 |
| 8 | C | 1.8441074595 | 1.0814429082 | -0.5643674320 |
| 9 | N | -1.2196111215 | -0.6661598635 | 0.7727790670 |
| 10 | C | -2.2240293657 | 0.0097585710 | 0.1549855043 |
| 11 | N | -1.9534759946 | 1.0043402636 | -0.6500475194 |
| 12 | C | -0.6817634169 | 1.3751390152 | -0.8666455562 |
| 13 | C | 0.4198847342 | 0.6589423034 | -0.2785885620 |
| 14 | C | 0.0837007386 | -0.3578299145 | 0.5556420010 |
| 15 | C | -3.6345056244 | -0.3647573637 | 0.4434231863 |
| 16 | C | -4.3221090883 | 0.6939150293 | 1.3343604867 |
| 17 | C | -5.7733910058 | 0.3201419132 | 1.6148376705 |
| 18 | N | -0.5133157927 | 2.4338545559 | -1.6520456111 |
| 19 | H | 4.2788223802 | -2.7251332462 | -1.3538864703 |
| 20 | H | 5.4805503353 | -2.5235984735 | 0.8071097735 |
| 21 | H | 4.9547649307 | -0.5843439465 | 2.3164621286 |
| 22 | H | 3.2343908559 | 1.0496010639 | 1.5797565925 |
| 23 | H | 2.6213046184 | -2.0050106131 | -2.8507479694 |
| 24 | H | 2.8120041330 | -0.2712477426 | -3.0616894216 |
| 25 | H | 1.3402270950 | -0.9151384845 | -2.3064580276 |
| 26 | H | 1.9674631564 | 1.3050153048 | -1.6254303047 |
| 27 | H | 2.0893360613 | 1.9930743226 | -0.0138666536 |
| 28 | H | -1.4576719333 | -1.4263043021 | 1.3983120271 |
| 29 | H | 0.8091879951 | -0.9737123134 | 1.0732852481 |
| 30 | H | -4.1621844530 | -0.4293755411 | -0.5118473419 |
| 31 | H | -3.6799206880 | -1.3493504578 | 0.9182763594 |

***Abridged***Abridged***Abridged***

| | | | | | | | |
|---|---|---|---|---|---|---|---|
| 0.0464 | 0.0489 | 0.0537 | 0.0574 | 0.0583 | 0.0623 | 0.0636 | 0.0693 |
| 0.0701 | 0.0741 | 0.0750 | 0.0783 | 0.0849 | 0.0878 | 0.0909 | 0.0953 |
| 0.0991 | 0.1015 | 0.1092 | 0.1105 | 0.1123 | 0.1179 | 0.1207 | 0.1252 |
| 0.1264 | 0.1307 | 0.1350 | 0.1371 | 0.1404 | 0.1448 | 0.1491 | 0.1537 |
| 0.1560 | 0.1606 | 0.1666 | 0.1703 | 0.1731 | 0.1760 | 0.1766 | 0.1801 |
| 0.1835 | 0.1878 | 0.1994 | 0.2018 | 0.2094 | 0.2139 | 0.2267 | 0.2311 |
| 0.2381 | 0.2431 | 0.2689 | 0.2736 | 0.2807 | 0.2838 | 0.2912 | 0.2964 |
| 0.3026 | 0.3132 | 0.3281 | 0.3297 | 0.3330 | 0.3405 | 0.3472 | 0.3511 |
| 0.3551 | 0.3611 | 0.3631 | 0.3690 | 0.3725 | 0.3801 | 0.3854 | 0.3933 |
| 0.3980 | 0.4017 | 0.4087 | 0.4143 | 0.4211 | 0.4239 | 0.4252 | 0.4312 |
| 0.4414 | 0.4433 | 0.4475 | 0.4515 | 0.4559 | 0.4608 | 0.4636 | 0.4668 |
| 0.4730 | 0.4736 | 0.4814 | 0.4839 | 0.4879 | 0.4942 | 0.4962 | 0.5013 |
| 0.5046 | 0.5070 | 0.5118 | 0.5171 | 0.5233 | 0.5254 | 0.5270 | 0.5283 |
| 0.5355 | 0.5390 | 0.5422 | 0.5461 | 0.5472 | 0.5531 | 0.5563 | 0.5575 |
| 0.5619 | 0.5680 | 0.5734 | 0.5783 | 0.5826 | 0.5859 | 0.5968 | 0.5996 |
| 0.6031 | 0.6106 | 0.6131 | 0.6162 | 0.6218 | 0.6253 | 0.6328 | 0.6361 |
| 0.6413 | 0.6449 | 0.6481 | 0.6515 | 0.6639 | 0.6723 | 0.6770 | 0.6845 |
| 0.6966 | 0.7027 | 0.7091 | 0.7145 | 0.7183 | 0.7327 | 0.7385 | 0.7415 |
| 0.7520 | 0.7567 | 0.7593 | 0.7738 | 0.7868 | 0.7880 | 0.7955 | 0.8048 |
| 0.8127 | 0.8184 | 0.8222 | 0.8317 | 0.8372 | 0.8381 | 0.8498 | 0.8554 |
| 0.8612 | 0.8734 | 0.8769 | 0.8853 | 0.8879 | 0.9020 | 0.9078 | 0.9186 |
| 0.9229 | 0.9301 | 0.9356 | 0.9375 | 0.9435 | 0.9464 | 0.9625 | 0.9665 |
| 0.9781 | 0.9897 | 0.9984 | 1.0008 | 1.0062 | 1.0182 | 1.0210 | 1.0294 |
| 1.0390 | 1.0414 | 1.0425 | 1.0497 | 1.0526 | 1.0629 | 1.0637 | 1.0746 |
| 1.0812 | 1.0923 | 1.0936 | 1.0994 | 1.1052 | 1.1140 | 1.1225 | 1.1302 |
| 1.1330 | 1.1405 | 1.1464 | 1.1513 | 1.1555 | 1.1651 | 1.1695 | 1.1730 |
| 1.1815 | 1.1912 | 1.1920 | 1.1990 | 1.2066 | 1.2086 | 1.2143 | 1.2202 |
| 1.2306 | 1.2337 | 1.2405 | 1.2505 | 1.2546 | 1.2605 | 1.2697 | 1.2805 |
| 1.2833 | 1.2903 | 1.2946 | 1.3008 | 1.3120 | 1.3156 | 1.3177 | 1.3224 |
| 1.3281 | 1.3350 | 1.3426 | 1.3453 | 1.3487 | 1.3606 | 1.3630 | 1.3676 |
| 1.3737 | 1.3846 | 1.3885 | 1.4033 | 1.4107 | 1.4115 | 1.4183 | 1.4286 |
| 1.4369 | 1.4459 | 1.4481 | 1.4544 | 1.4700 | 1.4749 | 1.4784 | 1.4890 |
| 1.4991 | 1.5115 | 1.5178 | 1.5238 | 1.5322 | 1.5386 | 1.5458 | 1.5546 |
| 1.5570 | 1.5624 | 1.5676 | 1.5900 | 1.5987 | 1.6029 | 1.6288 | 1.6361 |

| | | | | | | | |
|---|---|---|---|---|---|---|---|
| 1.6424 | 1.6712 | 1.6773 | 1.6952 | 1.7265 | 1.7485 | 1.7548 | 1.7887 |
| 1.8019 | 1.8184 | 1.8363 | 1.8500 | 1.8583 | 1.8700 | 1.9128 | 1.9358 |
| 1.9646 | 1.9757 | 2.0130 | 2.0343 | 2.0986 | 2.1297 | 2.1802 | 2.2019 |
| 2.2629 | 2.3299 | 2.3523 | 2.3682 | 2.3795 | 2.4067 | 2.4481 | 2.4628 |
| 2.4653 | 2.4782 | 2.4848 | 2.4919 | 2.5060 | 2.5123 | 2.5204 | 2.5288 |
| 2.5431 | 2.5495 | 2.5536 | 2.5617 | 2.5712 | 2.5821 | 2.5860 | 2.5969 |
| 2.6049 | 2.6069 | 2.6164 | 2.6181 | 2.6252 | 2.6360 | 2.6467 | 2.6531 |
| 2.6654 | 2.6746 | 2.6816 | 2.6868 | 2.6936 | 2.6964 | 2.7051 | 2.7105 |
| 2.7152 | 2.7214 | 2.7273 | 2.7335 | 2.7372 | 2.7484 | 2.7589 | 2.7661 |
| 2.7773 | 2.7884 | 2.7989 | 2.8016 | 2.8080 | 2.8119 | 2.8228 | 2.8255 |
| 2.8334 | 2.8446 | 2.8605 | 2.8657 | 2.8703 | 2.8741 | 2.8809 | 2.8936 |
| 2.8951 | 2.8977 | 2.9102 | 2.9154 | 2.9170 | 2.9304 | 2.9449 | 2.9490 |
| 2.9561 | 2.9615 | 2.9686 | 2.9714 | 2.9895 | 2.9950 | 3.0019 | 3.0148 |
| 3.0207 | 3.0237 | 3.0341 | 3.0410 | 3.0497 | 3.0532 | 3.0574 | 3.0662 |
| 3.0713 | 3.0829 | 3.0940 | 3.0993 | 3.1057 | 3.1093 | 3.1147 | 3.1205 |
| 3.1305 | 3.1357 | 3.1438 | 3.1539 | 3.1581 | 3.1725 | 3.1805 | 3.1849 |
| 3.1923 | 3.1948 | 3.2013 | 3.2090 | 3.2103 | 3.2226 | 3.2263 | 3.2341 |
| 3.2411 | 3.2476 | 3.2518 | 3.2606 | 3.2623 | 3.2667 | 3.2731 | 3.2796 |
| 3.2883 | 3.2948 | 3.2975 | 3.3040 | 3.3097 | 3.3149 | 3.3236 | 3.3271 |
| 3.3303 | 3.3468 | 3.3519 | 3.3656 | 3.3809 | 3.3871 | 3.3910 | 3.3941 |
| 3.4079 | 3.4157 | 3.4290 | 3.4353 | 3.4445 | 3.4541 | 3.4569 | 3.4666 |
| 3.4716 | 3.4907 | 3.4942 | 3.4993 | 3.5120 | 3.5203 | 3.5297 | 3.5362 |
| 3.5448 | 3.5492 | 3.5597 | 3.5653 | 3.5766 | 3.5829 | 3.5888 | 3.5965 |
| 3.6101 | 3.6134 | 3.6223 | 3.6305 | 3.6420 | 3.6485 | 3.6490 | 3.6558 |
| 3.6652 | 3.6855 | 3.6898 | 3.7037 | 3.7078 | 3.7234 | 3.7299 | 3.7406 |
| 3.7462 | 3.7488 | 3.7627 | 3.7693 | 3.7796 | 3.7922 | 3.7976 | 3.8016 |
| 3.8033 | 3.8153 | 3.8258 | 3.8332 | 3.8422 | 3.8488 | 3.8583 | 3.8738 |
| 3.8873 | 3.8941 | 3.9111 | 3.9203 | 3.9242 | 3.9330 | 3.9433 | 3.9531 |
| 3.9645 | 3.9670 | 3.9679 | 3.9767 | 3.9858 | 4.0048 | 4.0069 | 4.0153 |
| 4.0261 | 4.0349 | 4.0450 | 4.0507 | 4.0620 | 4.0747 | 4.0905 | 4.0931 |
| 4.1067 | 4.1161 | 4.1241 | 4.1295 | 4.1452 | 4.1474 | 4.1597 | 4.1701 |
| 4.1766 | 4.1774 | 4.1869 | 4.1922 | 4.1995 | 4.2170 | 4.2272 | 4.2302 |
| 4.2364 | 4.2444 | 4.2574 | 4.2699 | 4.2946 | 4.3071 | 4.3118 | 4.3194 |
| 4.3220 | 4.3308 | 4.3359 | 4.3449 | 4.3664 | 4.3716 | 4.3792 | 4.3839 |
| 4.3903 | 4.3964 | 4.4064 | 4.4197 | 4.4272 | 4.4343 | 4.4458 | 4.4519 |
| 4.4571 | 4.4738 | 4.4830 | 4.4893 | 4.4945 | 4.5059 | 4.5210 | 4.5354 |
| 4.5448 | 4.5638 | 4.5739 | 4.5830 | 4.6007 | 4.6127 | 4.6317 | 4.6484 |
| 4.6568 | 4.6639 | 4.7009 | 4.7181 | 4.7329 | 4.7451 | 4.7561 | 4.7799 |
| 4.8089 | 4.8355 | 4.8474 | 4.8626 | 4.8946 | 4.9038 | 4.9110 | 4.9205 |
| 4.9421 | 4.9465 | 4.9650 | 4.9790 | 4.9897 | 5.0003 | 5.0310 | 5.0532 |
| 5.0621 | 5.0662 | 5.0893 | 5.1031 | 5.1166 | 5.1216 | 5.1696 | 5.1933 |
| 5.2243 | 5.2417 | 5.2487 | 5.2671 | 5.2774 | 5.3050 | 5.3480 | 5.3623 |
| 5.4040 | 5.4320 | 5.4478 | 5.4538 | 5.5201 | 5.5280 | 5.5413 | 5.5607 |
| 5.5871 | 5.5914 | 5.5978 | 5.6186 | 5.6512 | 5.6594 | 5.6713 | 5.6739 |
| 5.6949 | 5.7186 | 5.7389 | 5.7544 | 5.7839 | 5.8061 | 5.8361 | 5.8788 |
| 5.9372 | 5.9543 | 5.9853 | 6.0035 | 6.0798 | 6.0902 | 6.1279 | 6.1861 |
| 6.2577 | 6.2973 | 6.3401 | 6.5327 | 6.5754 | 6.8063 | 23.9735 | 24.1254 |
| 24.1462 | 24.2581 | 24.2757 | 24.2817 | 24.3144 | 24.3448 | 24.4245 | 24.6071 |
| 24.6164 | 24.6260 | 24.8051 | 24.9159 | 35.7598 | 35.7709 | 35.7852 | 35.9447 |

Ground-State Mulliken Net Atomic Charges

| Atom | | Charge (a.u.) |
|---|---|---|
| 1 | N | 0.325161 |
| 2 | C | 0.130118 |
| 3 | C | -0.235078 |
| 4 | C | -0.160342 |
| 5 | C | -0.269523 |
| 6 | C | 0.030301 |
| 7 | C | -0.364506 |
| 8 | C | -0.053443 |
| 9 | N | -0.167128 |
| 10 | C | -0.005362 |
| 11 | N | -0.315046 |
| 12 | C | 0.509491 |
| 13 | C | -0.062736 |
| 14 | C | 0.032847 |
| 15 | C | -0.233082 |

```
    16 C                     -0.020242
    17 C                     -0.469985
    18 N                     -0.382583
    19 H                      0.199455
    20 H                      0.215065
    21 H                      0.204807
    22 H                      0.208360
    23 H                      0.163128
    24 H                      0.187905
    25 H                      0.163705
    26 H                      0.184938
    27 H                      0.184463
    28 H                      0.279189
    29 H                      0.184797
    30 H                      0.176885
    31 H                      0.130183
    32 H                      0.149437
    33 H                      0.121162
    34 H                      0.132303
    35 H                      0.146243
    36 H                      0.126180
    37 H                      0.244172
    38 H                      0.308761
 ----------------------------------------
 Sum of atomic charges =     2.000000

-----------------------------------------------------------------
                   Cartesian Multipole Moments
-----------------------------------------------------------------
   Charge (ESU x 10^10)
                9.6064
   Dipole Moment (Debye)
        X       9.5841      Y      -0.6678      Z      -1.1034
      Tot       9.6704
   Quadrupole Moments (Debye-Ang)
       XX      -1.5085     XY     -16.4208     YY     -69.3169
       XZ      -5.5095     YZ      -8.8176     ZZ     -78.1278
   Traceless Quadrupole Moments (Debye-Ang)
      QXX     144.4278    QYY     -58.9975    QZZ     -85.4302
      QXY     -49.2623    QXZ     -16.5286    QYZ     -26.4529
   Octopole Moments (Debye-Ang^2)
      XXX      59.7685    XXY     -91.5902    XYY      16.8987
      YYY      19.2033    XXZ      73.4794    XYZ      10.7470
      YYZ     -31.2279    XZZ      12.6630    YZZ       4.1147
      ZZZ      -5.2756
   Traceless Octopole Moments (Debye-Ang^2)
      XXX      92.5554    YYY     902.4998    ZZZ    -411.9172
      XXY   -1169.0364    XXZ     991.2631    XYY     -14.5101
      XYZ     161.2054    XZZ     -78.0453    YYZ    -579.3459
      YZZ     266.5366
   Hexadecapole Moments (Debye-Ang^3)
     XXXX   -4339.2560   XXXY     -33.9582   XXYY    -925.1545
     XYYY     373.4460   YYYY    -715.3978   XXXZ     403.0067
     XXYZ      20.7539   XYYZ     107.5621   YYYZ     -53.5800
     XXZZ    -930.9989   XYZZ     108.0645   YYZZ    -217.6655
     XZZZ     336.2907   YZZZ      37.1418   ZZZZ    -865.4181
   Traceless Hexadecapole Moments (Debye-Ang^3)
     XXXX   11355.5782   XXXY  -23705.4656   XXXZ    4207.0243
     XXYY   -6539.9419   XXYZ    2116.5531   XXZZ   -4815.6364
     XYYY   19071.9746   XYYZ   -1408.8683   XYZZ    4633.4910
     XZZZ   -2798.1560   YYYY    1513.4419   YYYZ   -5813.7261
     YYZZ    5026.5000   YZZZ    3697.1730   ZZZZ    -210.8636
-----------------------------------------------------------------
Total job time:  538.34s(wall), 386.02s(cpu)
Mon Oct 14 03:46:14 2024
```

```
Wavefunction Version of Q-Chem

 Parts of Q-Chem use Armadillo 8.300.2 (Tropical Shenanigans).
 http://arma.sourceforge.net/

 Q-Chem begins on Sun Oct 13 17:53:15 2024


     Scratch files written to
C:/Users/hille/AppData/Local/Temp/WFB0CB2A5C99266D7A/Conformer.10//scratch///
Processing default memory
  ... MEM_TOTAL 4076 MB (default) [16 cores]
Processing $rem in C:/Program
Files/Wavefunction/Spartan24v110/P4e//../auxdir/config/preferences:
   (site specific preferences)
  ... THRESH             9
  ... SMALL_PROD_XCMAT   9
  ... BASIS_LIN_DEP_THRESH     5
  ... SCF_ALGORITHM     DIIS_GDM
  ... MAXSCF            250
  ... MAXDIIS          45
  ... THRESHDIIS         -1  (i.e. don't switch on delta-E)
  ... ECP_FIT          TRUE (Convert deprecated ECP files)
  ... GUI              GUI_SPARTAN
  ... TERSE_OUTPUT     TRUE !turn on spartan printing
  ... SCF_CONVERGENCE  7
  ... CCMAN2 FALSE  (qc4.3)
  ... SYMMETRY   FALSE  ! turn of symmetry for spartan16
  ... SYM_IGNORE TRUE   ! ..use FORCESYMMETRY to override
  ... GEOM_OPT_TOL_GRADIENT       700  ! loosen tolernaces for organic geometries
  ... GEOM_OPT_TOL_DISPLACEMENT  1400  ! was 1200 = .0012
  ... GEOM_OPT_TOL_ENERGY        2000  ! was  100 = .000 001
  ... GEN_SCFMAN  FALSE
Processing $rem in input file
  ... JOBTYPE    OPT
  ... TIDY_SYM   TRUE
  ... METHOD            WB97X-D
  ... xc_grid           75000302  (75,302)
  ... BASIS             6-311+G**
  ... THRESH             12  #diffuse default
  ... MAXSCF            350  #diffuse default
  ... GEOM_OPT_TOL_ENERGY       22850
  ... GEOM_OPT_TOL_GRADIENT       800
  ... VARTHRESH         2    (default DFT)
  ... INCDFT            TRUE (default DFT)
  ... GEOM_OPT_HESSIAN      READ (main opt)
  ... EXTERNAL_HESSIAN      1
  ... GUI                GUI_SPARTAN
  ... TERSE_OUTPUT       TRUE
NAlpha2: 130
NElect   130
Mult     1

Checking the input file for inconsistencies...  ...done.

--------------------------------------------------------------
User input:
--------------------------------------------------------------
$comment
Molecule9
$end
$molecule
 2 1
    7      2.010756028     0.2253372561    -1.6584236419
    6     2.8376646358     1.3147399033    -1.7545729689
    6     2.9676959376     1.9718709268    -2.9898435684
    6     2.2826771482      1.520188126    -4.1074258319
    6     1.4752201912    0.40470558168    -3.9930355295
    6     1.3606181102   -0.22842093524    -2.7643404032
```

```
    6     3.6325852787     1.8198979366   -0.58170320118
    6     1.8409179433   -0.52872694356   -0.37574013994
    7    -1.3668348233    -1.9602238523    0.99075534828
    6    -2.1599104645   -0.87956839146    0.98713926305
    7    -1.7182627537    0.33695941215     0.5891260807
    6   -0.44587960097    0.44892967913     0.1906315991
    6    0.41391987386   -0.63706660608    0.11804807222
    6  -0.096477458935     -1.862638852    0.56476797352
    6    -3.5763173377    -1.0111777193     1.4744142411
    6    -3.7500049739    -0.5456469859     2.9208390148
    6    -3.0602262495    -1.4532408038     3.9301244098
    7  -0.047254711577      1.732738789   -0.14008488773
    1     3.6154832021     2.8435357866    -3.0889853576
    1      2.388344628     2.0326624961    -5.0634042451
    1    0.93984057775   0.029102727689     -4.863177604
    1    0.74044196941    -1.1137672205    -2.6617482008
    1     4.2299688107     2.6981766028   -0.84843864497
    1     4.3241608874     1.0458995016   -0.23541095703
    1     2.9671883295     2.1146452292    0.23428729927
    1      2.448325286  -0.085921305398    0.41539557044
    1      2.255329061    -1.5242664905   -0.58186927015
    1    -1.7457113123    -2.8494114412     1.3239528853
    1    0.47791784826    -2.7843651688    0.59337258709
    1    -4.2186292918   -0.40550681307     0.8238017233
    1    -3.9112695105    -2.0493877758     1.3662485161
    1    -4.8216117582   -0.52372835091     3.1525675561
    1    -3.3766515029    0.47841367726      3.040894374
    1    -3.4069227743    -2.4866905842     3.8301128758
    1    -3.2819539498    -1.1188938933     4.9486895332
    1    -1.9733305943    -1.4394036872     3.8047968821
    1    0.62449495889      2.132484506    0.51206617054
    1   -0.87630163745     2.3377656825   -0.10382752343
$end
$rem
JOBTYPE    OPT
TIDY_SYM   TRUE
METHOD          WB97X-D
xc_grid         75000302  (75,302)
BASIS           6-311+G**
THRESH           12  #diffuse default
MAXSCF          350  #diffuse default
GEOM_OPT_TOL_ENERGY     22850
GEOM_OPT_TOL_GRADIENT     800
VARTHRESH        2    (default DFT)
INCDFT          TRUE (default DFT)
GEOM_OPT_HESSIAN      READ (main opt)
EXTERNAL_HESSIAN      1
GUI              GUI_SPARTAN
TERSE_OUTPUT     TRUE
$end
$opt
$end
--------------------------------------------------------------
 --------------------------------------------------------------
             Standard Nuclear Orientation (Angstroms)
    I     Atom           X                Y                Z
 --------------------------------------------------------------
    1      N      2.0107560280     0.2253372561    -1.6584236419
    2      C      2.8376646358     1.3147399033    -1.7545729689
    3      C      2.9676959376     1.9718709268    -2.9898435684
    4      C      2.2826771482     1.5201881260    -4.1074258319
    5      C      1.4752201912     0.4047055817    -3.9930355295
    6      C      1.3606181102    -0.2284209352    -2.7643404032
    7      C      3.6325852787     1.8198979366    -0.5817032012
    8      C      1.8409179433    -0.5287269436    -0.3757401399
    9      N     -1.3668348233    -1.9602238523     0.9907553483
   10      C     -2.1599104645    -0.8795683915     0.9871392631
   11      N     -1.7182627537     0.3369594122     0.5891260807
   12      C     -0.4458796010     0.4489296791     0.1906315991
   13      C      0.4139198739    -0.6370666061     0.1180480722
```

```
  14      C      -0.0964774589    -1.8626388520     0.5647679735
  15      C      -3.5763173377    -1.0111777193     1.4744142411
  16      C      -3.7500049739    -0.5456469859     2.9208390148
  17      C      -3.0602262495    -1.4532408038     3.9301244098
  18      N      -0.0472547116     1.7327387890    -0.1400848877
  19      H       3.6154832021     2.8435357866    -3.0889853576
  20      H       2.3883446280     2.0326624961    -5.0634042451
  21      H       0.9398405778     0.0291027277    -4.8631776040
  22      H       0.7404419694    -1.1137672205    -2.6617482008
  23      H       4.2299688107     2.6981766028    -0.8484386450
  24      H       4.3241608874     1.0458995016    -0.2354109570
  25      H       2.9671883295     2.1146452292     0.2342872993
  26      H       2.4483252860    -0.0859213054     0.4153955704
  27      H       2.2553290610    -1.5242664905    -0.5818692702
  28      H      -1.7457113123    -2.8494114412     1.3239528853
  29      H       0.4779178483    -2.7843651688     0.5933725871
  30      H      -4.2186292918    -0.4055068131     0.8238017233
  31      H      -3.9112695105    -2.0493877758     1.3662485161
  32      H      -4.8216117582    -0.5237283509     3.1525675561
  33      H      -3.3766515029     0.4784136773     3.0408943740
  34      H      -3.4069227743    -2.4866905842     3.8301128758
  35      H      -3.2819539498    -1.1188938933     4.9486895332
  36      H      -1.9733305943    -1.4394036872     3.8047968821
  37      H       0.6244949589     2.1324845060     0.5120661705
  38      H      -0.8763016375     2.3377656825    -0.1038275234
 ----------------------------------------------------------------
 Nuclear Repulsion Energy =        1308.21737265 hartrees
 There are       65 alpha and       65 beta electrons
 Requested basis set is 6-311+G(d,p)
 There are 188 shells and 516 basis functions

 Total QAlloc Memory Limit   4076 MB
 Mega-Array Size       188 MB
 MEM_STATIC part       192 MB

 ..                                                                          (5.2.P)

--------------------------------------------------
-  Entering fldman on Sun Oct 13 17:53:15 2024  -
--------------------------------------------------

 A cutoff of  1.0D-12 yielded  12661 shell pairs
 There are    101279 function pairs (    108025 Cartesian)
 Smallest overlap matrix eigenvalue = 2.05E-06
 Linear dependence detected in AO basis
 Tighter screening thresholds may be required for diffuse basis sets
 Use S2THRESH > 12 and THRESH = 14 in case of SCF convergence issues
 Number of orthogonalized atomic orbitals = 510
 Maximum deviation from orthogonality = 2.036E-11

 Scale SEOQF with 1.000000e-01/1.000000e-01/1.000000e-01

 Standard Electronic Orientation quadrupole field applied
 Nucleus-field energy     =    -0.0000000269 hartrees

--------------------------------------------------
-  Entering gesman on Sun Oct 13 17:53:15 2024  -
--------------------------------------------------

 Guess from superposition of atomic densities
 Warning:  Energy on first SCF cycle will be non-variational
 SAD guess density has 132.000000 electrons

--------------------------------------------------
-  Entering scfman on Sun Oct 13 17:53:15 2024  -
--------------------------------------------------

 Long-range K will be added via erf
 Coulomb attenuation parameter = 0.2 bohr**(-1)
 A restricted hybrid HF-DFT SCF calculation will be
```

```
 performed using Pulay DIIS + Geometric Direct Minimization
 Exchange:     0.2220 Hartree-Fock + 1.0000 wB97X-D + LR-HF
 Correlation:  1.0000 wB97X-D
 Using Euler-Maclaurin-Lebedev (75,302) quadrature formula
 Dispersion:   Grimme D
 SCF converges when RMS gradient is below 1.0E-07
 Exchange:     0.2220 Hartree-Fock + 1.0000 wB97X-D + LR-HF
 Correlation:  1.0000 wB97X-D
 Using Euler-Maclaurin-Lebedev (75,302) quadrature formula
 Dispersion:   Grimme D
 ---------------------------------------
  Cycle       Energy         DIIS Error
 ---------------------------------------
    1    -770.2911945620      2.95E-02
    2    -763.7499317595      2.38E-03
    3    -763.7617125153      2.54E-03
    4    -763.9429114501      3.65E-04
    5    -763.9460963271      1.88E-04
    6    -763.9470835798      3.66E-05
    7    -763.9471218321      1.42E-05
    8    -763.9471281562      3.76E-06
    9    -763.9471287423      1.84E-06
   10    -763.9471288807      6.13E-07
   11    -763.9471289011      2.12E-07
   12    -763.9471289048      9.03E-08 Convergence criterion met
 ---------------------------------------
 SCF time:  CPU 409.36 s  wall 684.32 s
 SCF   energy in the final basis set = -763.94712890
 Total energy in the final basis set = -763.94712890

-------------------------------------------------
-  Entering anlman on Sun Oct 13 18:04:40 2024  -
-------------------------------------------------



 --------------------------------------------------------------

                   Orbital Energies (a.u.)
 --------------------------------------------------------------


 Alpha MOs
 -- Occupied --
-14.8136 -14.8132 -14.7243 -14.7162 -10.6749 -10.6714 -10.6618 -10.6360
-10.6355 -10.6229 -10.6156 -10.5745 -10.5669 -10.5658 -10.5455 -10.5339
-10.4974 -10.4716  -1.4290  -1.3945  -1.3458  -1.2673  -1.2275  -1.1987
 -1.1758  -1.1454  -1.1027  -1.0746  -1.0654  -1.0513  -1.0237  -1.0105
 -0.9851  -0.9498  -0.9476  -0.9080  -0.8971  -0.8844  -0.8776  -0.8602
 -0.8483  -0.8406  -0.8361  -0.8168  -0.8070  -0.7917  -0.7838  -0.7794
 -0.7756  -0.7551  -0.7471  -0.7437  -0.7322  -0.7240  -0.7210  -0.7062
 -0.7034  -0.6693  -0.6551  -0.6484  -0.6377  -0.6312  -0.6280  -0.6228
 -0.6087
 -- Virtual --
 -0.2786  -0.2645  -0.2571  -0.2200  -0.1769  -0.1636  -0.1468  -0.1437
 -0.1348  -0.1263  -0.1171  -0.1131  -0.1115  -0.1044  -0.0989  -0.0936
 -0.0897  -0.0875  -0.0795  -0.0783  -0.0745  -0.0728  -0.0720  -0.0671
 -0.0655  -0.0589  -0.0578  -0.0529  -0.0501  -0.0446  -0.0407  -0.0356
 -0.0318  -0.0242  -0.0208  -0.0184  -0.0176  -0.0166  -0.0128  -0.0089
 -0.0050  -0.0043  -0.0018   0.0009   0.0072   0.0076   0.0144   0.0159
  0.0172   0.0182   0.0229   0.0267   0.0309   0.0341   0.0378   0.0387
  0.0424   0.0455   0.0472   0.0518   0.0529   0.0603   0.0639   0.0651
  0.0693   0.0713   0.0746   0.0768   0.0777   0.0801   0.0838   0.0889
  0.0946   0.0978   0.1016   0.1028   0.1086   0.1094   0.1145   0.1176
  0.1196   0.1264   0.1275   0.1320   0.1323   0.1358   0.1386   0.1455
  0.1521   0.1575   0.1611   0.1662   0.1680   0.1713   0.1718   0.1765
  0.1847   0.1885   0.1903   0.1950   0.2033   0.2055   0.2126   0.2284
  0.2350   0.2503   0.2538   0.2729   0.2826   0.2998   0.3029   0.3165
  0.3249   0.3293   0.3324   0.3456   0.3483   0.3549   0.3607   0.3663
  0.3684   0.3798   0.3813   0.3959   0.3999   0.4060   0.4101   0.4171
  0.4274   0.4331   0.4398   0.4445   0.4555   0.4648   0.4678   0.4785
  0.4804   0.4839   0.4936   0.4966   0.5007   0.5113   0.5138   0.5176
```

| | | | | | | | |
|---|---|---|---|---|---|---|---|
| 0.5201 | 0.5232 | 0.5297 | 0.5361 | 0.5431 | 0.5477 | 0.5546 | 0.5624 |
| 0.5628 | 0.5674 | 0.5685 | 0.5800 | 0.5821 | 0.5874 | 0.5931 | 0.5997 |
| 0.6078 | 0.6163 | 0.6200 | 0.6248 | 0.6268 | 0.6382 | 0.6437 | 0.6580 |
| 0.6635 | 0.6766 | 0.6829 | 0.6858 | 0.6936 | 0.7006 | 0.7067 | 0.7178 |
| 0.7221 | 0.7282 | 0.7391 | 0.7449 | 0.7481 | 0.7572 | 0.7774 | 0.7919 |
| 0.7968 | 0.8042 | 0.8090 | 0.8173 | 0.8291 | 0.8320 | 0.8444 | 0.8627 |
| 0.8670 | 0.8745 | 0.8823 | 0.8880 | 0.8987 | 0.9102 | 0.9204 | 0.9297 |
| 0.9402 | 0.9450 | 0.9728 | 0.9832 | 0.9926 | 1.0144 | 1.0268 | 1.0379 |
| 1.0503 | 1.0688 | 1.0847 | 1.1058 | 1.1252 | 1.1448 | 1.1629 | 1.1847 |
| 1.1990 | 1.2120 | 1.2397 | 1.2468 | 1.2666 | 1.2693 | 1.2920 | 1.3008 |
| 1.3093 | 1.3312 | 1.3357 | 1.3403 | 1.3521 | 1.3633 | 1.3697 | 1.3828 |
| 1.3890 | 1.3941 | 1.4050 | 1.4134 | 1.4260 | 1.4332 | 1.4337 | 1.4383 |
| 1.4432 | 1.4465 | 1.4511 | 1.4550 | 1.4581 | 1.4733 | 1.4819 | 1.4964 |
| 1.4980 | 1.5066 | 1.5152 | 1.5172 | 1.5258 | 1.5370 | 1.5451 | 1.5474 |
| 1.5628 | 1.5674 | 1.5719 | 1.5805 | 1.5854 | 1.5962 | 1.6034 | 1.6083 |
| 1.6165 | 1.6321 | 1.6375 | 1.6604 | 1.6661 | 1.6708 | 1.6771 | 1.6832 |
| 1.6916 | 1.7003 | 1.7048 | 1.7181 | 1.7267 | 1.7387 | 1.7635 | 1.7759 |
| 1.7794 | 1.7940 | 1.8022 | 1.8145 | 1.8257 | 1.8388 | 1.8549 | 1.8621 |
| 1.8793 | 1.8919 | 1.9056 | 1.9069 | 1.9333 | 1.9434 | 1.9644 | 1.9924 |
| 1.9940 | 2.0226 | 2.0249 | 2.0319 | 2.0487 | 2.0533 | 2.0861 | 2.0932 |
| 2.0990 | 2.1047 | 2.1243 | 2.1398 | 2.1513 | 2.1613 | 2.2017 | 2.2167 |
| 2.2260 | 2.2363 | 2.2420 | 2.2557 | 2.2659 | 2.2843 | 2.2929 | 2.3024 |
| 2.3081 | 2.3155 | 2.3497 | 2.3605 | 2.3662 | 2.3730 | 2.3836 | 2.3928 |
| 2.4127 | 2.4197 | 2.4321 | 2.4409 | 2.4480 | 2.4509 | 2.4819 | 2.4848 |
| 2.4980 | 2.5130 | 2.5164 | 2.5338 | 2.5364 | 2.5469 | 2.5510 | 2.5543 |
| 2.5607 | 2.5715 | 2.5845 | 2.5932 | 2.5970 | 2.6028 | 2.6124 | 2.6192 |
| 2.6285 | 2.6331 | 2.6459 | 2.6618 | 2.6684 | 2.6708 | 2.6804 | 2.6930 |
| 2.6986 | 2.7228 | 2.7258 | 2.7341 | 2.7367 | 2.7537 | 2.7679 | 2.7769 |
| 2.7882 | 2.7913 | 2.8061 | 2.8232 | 2.8327 | 2.8455 | 2.8748 | 2.8903 |
| 2.9061 | 2.9365 | 2.9395 | 2.9915 | 3.0423 | 3.0559 | 3.0796 | 3.1342 |
| 3.1381 | 3.1816 | 3.2413 | 3.2589 | 3.2751 | 3.3195 | 3.3286 | 3.3874 |
| 3.4072 | 3.4641 | 3.4907 | 3.5267 | 3.5421 | 3.5945 | 3.6219 | 3.6461 |
| 3.6584 | 3.6751 | 3.7014 | 3.7099 | 3.7312 | 3.7526 | 3.7643 | 3.7761 |
| 3.8161 | 3.8668 | 3.8903 | 3.9114 | 3.9494 | 4.0329 | 4.0487 | 4.0963 |
| 4.1142 | 4.2053 | 4.3010 | 4.3119 | 4.5621 | 4.6386 | 4.6552 | 4.7350 |
| 4.8691 | 5.0252 | 5.1433 | 23.5326 | 23.5543 | 23.6812 | 23.7094 | 23.7330 |
| 23.7901 | 23.7985 | 23.8291 | 23.8464 | 23.8759 | 23.9077 | 23.9192 | 23.9340 |
| 24.0316 | 35.4110 | 35.4372 | 35.4825 | 35.5750 | | | |

Ground-State Mulliken Net Atomic Charges

| Atom | Charge (a.u.) |
|---|---|
| 1 N | 0.481895 |
| 2 C | 0.113581 |
| 3 C | -0.032503 |
| 4 C | -0.136306 |
| 5 C | -0.198144 |
| 6 C | -0.142870 |
| 7 C | -0.724285 |
| 8 C | -0.239465 |
| 9 N | -0.099615 |
| 10 C | -0.291604 |
| 11 N | 0.055704 |
| 12 C | 0.107340 |
| 13 C | -0.116552 |
| 14 C | 0.067544 |
| 15 C | -0.363832 |
| 16 C | -0.240703 |
| 17 C | -0.521955 |
| 18 N | -0.499066 |
| 19 H | 0.221754 |
| 20 H | 0.232011 |
| 21 H | 0.227380 |
| 22 H | 0.220729 |
| 23 H | 0.233124 |
| 24 H | 0.245049 |
| 25 H | 0.176231 |
| 26 H | 0.266404 |

```
    27 H                     0.242374
    28 H                     0.404937
    29 H                     0.226909
    30 H                     0.251530
    31 H                     0.210312
    32 H                     0.223886
    33 H                     0.200910
    34 H                     0.178171
    35 H                     0.217472
    36 H                     0.134712
    37 H                     0.316274
    38 H                     0.350667
  ----------------------------------------
  Sum of atomic charges =     2.000000

 -----------------------------------------------------------------
                    Cartesian Multipole Moments
 -----------------------------------------------------------------
    Charge (ESU x 10^10)
                 9.6064
    Dipole Moment (Debye)
         X       4.3222      Y      -1.8249      Z      -4.3167
       Tot       6.3754
    Quadrupole Moments (Debye-Ang)
        XX     -40.5555     XY      27.8904     YY     -63.3281
        XZ     -38.5910     YZ     -19.8272     ZZ     -45.4767
    Traceless Quadrupole Moments (Debye-Ang)
       QXX      27.6938    QYY     -40.6239    QZZ      12.9301
       QXY      83.6713    QXZ    -115.7731    QYZ     -59.4815
    Octopole Moments (Debye-Ang^2)
       XXX     -18.5032    XXY      24.7114    XYY      11.4190
       YYY     -20.8352    XXZ      34.3607    XYZ     -19.9490
       YYZ      20.1834    XZZ      24.0226    YZZ      33.3738
       ZZZ     -70.0861
    Traceless Octopole Moments (Debye-Ang^2)
       XXX    -429.9935    YYY    -647.7784    ZZZ    -911.4130
       XXY     258.9217    XXZ     562.0368    XYY     120.4696
       XYZ    -299.2354    XZZ     309.5239    YYZ     349.3763
       YZZ     388.8567
    Hexadecapole Moments (Debye-Ang^3)
      XXXX   -2741.2582   XXXY    -273.9961   XXYY    -546.5161
      XYYY    -448.3704   YYYY    -874.7740   XXXZ     892.5764
      XXYZ     -35.1799   XYYZ     279.3917   YYYZ     398.6255
      XXZZ    -756.5193   XYZZ      13.2348   YYZZ    -569.4562
      XZZZ     744.3772   YZZZ     241.7646   ZZZZ   -2057.5907
    Traceless Hexadecapole Moments (Debye-Ang^3)
      XXXX   -8613.1384   XXXY    3141.3390   XXXZ    7484.9857
      XXYY    4885.5891   XXYZ  -12760.5732   XXZZ    3727.5493
      XYYY  -15167.9671   XYYZ     590.9515   XYZZ   12026.6281
      XZZZ   -8075.9372   YYYY    2548.4444   YYYZ   14655.6234
      YYZZ   -7434.0335   YZZZ   -1895.0503   ZZZZ    3706.4842
 -----------------------------------------------------------------

--------------------------------------------------
-  Entering drvman on Sun Oct 13 18:04:40 2024  -
--------------------------------------------------

 Calculating analytic gradient of the SCF energy
 Gradient of SCF Energy
           1           2           3           4           5           6
    1  -0.0038410  -0.0057443   0.0032552  -0.0058262   0.0074652   0.0016234
    2  -0.0091534  -0.0118278   0.0062395  -0.0077370   0.0122802  -0.0042250
    3   0.0032256   0.0085188  -0.0035925  -0.0009633  -0.0067838   0.0100856
           7           8           9          10          11          12
    1   0.0040382  -0.0045094  -0.0053457   0.0215262   0.0070303   0.0177542
    2   0.0048194   0.0070425  -0.0020674  -0.0391333   0.0424939  -0.0316022
    3  -0.0004381   0.0046076  -0.0003765   0.0062587  -0.0072233  -0.0099058
          13          14          15          16          17          18
    1  -0.0068955  -0.0190793   0.0007104   0.0001763   0.0000780   0.0141063
    2   0.0417391  -0.0133399  -0.0005591  -0.0022030   0.0001660  -0.0011631
```

```
    3  -0.0016611   0.0114846   0.0110505  -0.0123603  -0.0007390  -0.0088554
             19          20          21          22          23          24
    1   0.0022118   0.0003731  -0.0008169  -0.0018954   0.0040949   0.0003775
    2   0.0052345   0.0018334   0.0018900  -0.0018184   0.0043014  -0.0012890
    3  -0.0055351  -0.0036854  -0.0058504  -0.0038509   0.0020567  -0.0026631
             25          26          27          28          29          30
    1  -0.0026556  -0.0020562   0.0021519  -0.0034464  -0.0014849  -0.0047006
    2   0.0007415  -0.0025748  -0.0023580  -0.0048321  -0.0027381   0.0004406
    3  -0.0021680   0.0059486  -0.0050294   0.0012054   0.0020471   0.0001186
             31          32          33          34          35          36
    1  -0.0012743  -0.0023820   0.0023251   0.0000830  -0.0018554  -0.0019064
    2  -0.0000876  -0.0009072   0.0008865   0.0000675   0.0021376   0.0003921
    3  -0.0012535   0.0046133   0.0031537  -0.0015526   0.0022112  -0.0019059
             37          38
    1  -0.0065637  -0.0071015
    2   0.0037609   0.0031499
    3   0.0071473   0.0026600
 Max gradient component =       4.249E-02
 RMS gradient           =       9.380E-03
 Gradient time:  CPU 155.72 s  wall 263.29 s

-------------------------------------------------
-  Entering optman on Sun Oct 13 18:09:03 2024  -
-------------------------------------------------

 Geometry Optimization Parameters
   NAtoms,    NIC,     NZ,  NCons,   NDum,   NFix, NCnnct, MaxDiis
       38     272       0       0       0       0       0       0

 Cartesian Hessian read from HESS file


** GEOMETRY OPTIMIZATION IN DELOCALIZED INTERNAL COORDINATES **
   Searching for a Minimum

   Optimization Cycle:   1

                    Coordinates (Angstroms)
     ATOM                X               Y               Z
      1  N         2.0107560280    0.2253372561   -1.6584236419
      2  C         2.8376646358    1.3147399033   -1.7545729689
      3  C         2.9676959376    1.9718709268   -2.9898435684
      4  C         2.2826771482    1.5201881260   -4.1074258319
      5  C         1.4752201912    0.4047055817   -3.9930355295
      6  C         1.3606181102   -0.2284209352   -2.7643404032
      7  C         3.6325852787    1.8198979366   -0.5817032012
      8  C         1.8409179433   -0.5287269436   -0.3757401399
      9  N        -1.3668348233   -1.9602238523    0.9907553483
     10  C        -2.1599104645   -0.8795683915    0.9871392630
     11  N        -1.7182627537    0.3369594122    0.5891260807
     12  C        -0.4458796010    0.4489296791    0.1906315991
     13  C         0.4139198739   -0.6370666061    0.1180480722
     14  C        -0.0964774589   -1.8626388520    0.5647679735
     15  C        -3.5763173377   -1.0111777193    1.4744142411
     16  C        -3.7500049739   -0.5456469859    2.9208390148
     17  C        -3.0602262495   -1.4532408038    3.9301244098
     18  N        -0.0472547116    1.7327387890   -0.1400848877
     19  H         3.6154832021    2.8435357866   -3.0889853576
     20  H         2.3883446280    2.0326624961   -5.0634042451
     21  H         0.9398405777    0.0291027277   -4.8631776040
     22  H         0.7404419694   -1.1137672205   -2.6617482008
     23  H         4.2299688107    2.6981766028   -0.8484386450
     24  H         4.3241608874    1.0458995016   -0.2354109570
     25  H         2.9671883295    2.1146452292    0.2342872993
     26  H         2.4483252860   -0.0859213054    0.4153955704
     27  H         2.2553290610   -1.5242664905   -0.5818692702
     28  H        -1.7457113123   -2.8494114412    1.3239528853
     29  H         0.4779178483   -2.7843651688    0.5933725871
     30  H        -4.2186292918   -0.4055068131    0.8238017233
     31  H        -3.9112695105   -2.0493877758    1.3662485161
```

```
    32  H        -4.8216117582   -0.5237283509    3.1525675561
    33  H        -3.3766515029    0.4784136773    3.0408943740
    34  H        -3.4069227743   -2.4866905842    3.8301128758
    35  H        -3.2819539498   -1.1188938933    4.9486895332
    36  H        -1.9733305943   -1.4394036872    3.8047968821
    37  H         0.6244949589    2.1324845060    0.5120661705
    38  H        -0.8763016375    2.3377656825   -0.1038275234
  Point Group: c1    Number of degrees of freedom:   108


  Energy is   -763.947128905


 Attempting to generate delocalized internal coordinates

 Transforming Cartesian Hessian to Internal Coordinates
 Hessian Transformation does not Include Derivative of B-matrix
  internal optimization (0)

108 Hessian modes will be used to form the next step
  Hessian Eigenvalues:
     0.001845    0.002645    0.004020    0.004933    0.008208    0.011377
     0.013074    0.019044    0.019465    0.019912    0.021220    0.022023
     0.022602    0.023640    0.024043    0.024563    0.025256    0.026857
     0.027670    0.028199    0.029056    0.030836    0.035918    0.038418
     0.038983    0.040350    0.042912    0.043561    0.044672    0.046040
     0.048009    0.053835    0.055770    0.057608    0.066426    0.078776
     0.085511    0.094994    0.121690    0.122092    0.127095    0.130873
     0.131421    0.132801    0.135523    0.139165    0.143886    0.146686
     0.147677    0.148262    0.148875    0.150919    0.152419    0.152922
     0.153415    0.156628    0.175877    0.206077    0.209726    0.214017
     0.217127    0.228763    0.236227    0.239610    0.246988    0.252529
     0.263588    0.268998    0.274223    0.282165    0.298514    0.300090
     0.300605    0.301151    0.301664    0.302516    0.303965    0.304395
     0.304896    0.305165    0.306333    0.308480    0.311095    0.313780
     0.323855    0.330031    0.332570    0.334798    0.339098    0.340944
     0.346865    0.348334    0.353083    0.359887    0.363418    0.383608
     0.384550    0.394128    0.398256    0.404806    0.415525    0.422390
     0.432094    0.436707    0.452239    0.465687    0.634301    0.726590

 Minimum search - taking simple RFO step
 Searching for Lamda that Minimizes Along All modes
 Value Taken    Lamda =  -0.02375464
 Calculated Step too Large.  Step scaled by  0.696375 !!
 Step Taken.  Stepsize is  0.300000  0.013735

                             Maximum     Tolerance    Cnvgd?
        Gradient            0.025188      0.000800      NO
        Displacement        0.097369      0.001400      NO
        Energy change      *********      0.000228      NO


 New Cartesian Coordinates Obtained by Inverse Iteration

 Displacement from previous Coordinates is:  0.592170
 ----------------------------------------------------------------
             Standard Nuclear Orientation (Angstroms)
    I     Atom           X                Y                Z
 ----------------------------------------------------------------
    1      N       2.0103882108     0.2330258094    -1.6392663548
    2      C       2.8364412907     1.3141191031    -1.7402939466
    3      C       2.9453112889     1.9570483501    -2.9661501424
    4      C       2.2401044669     1.5012337383    -4.0689167184
    5      C       1.4258193156     0.3817114993    -3.9445994963
    6      C       1.3380006053    -0.2294971944    -2.7237653784
    7      C       3.6386058367     1.7927258668    -0.5673914142
    8      C       1.8518202100    -0.5206965856    -0.3648769526
    9      N      -1.3560759116    -1.9175323019     0.9651808569
   10      C      -2.1794019761    -0.8465208507     0.9620563690
   11      N      -1.7469242399     0.3399817831     0.5893203170
```

```
  12      C      -0.4675242087     0.4712507207     0.2117157427
  13      C       0.4219884782    -0.6285662840     0.1219915585
  14      C      -0.0778295417    -1.8280314197     0.5451437145
  15      C      -3.5837950846    -1.0074036269     1.4426438652
  16      C      -3.7479747150    -0.5566588553     2.9056114322
  17      C      -3.0244234765    -1.4574537735     3.9017327375
  18      N      -0.0704544181     1.7403949676    -0.1503126996
  19      H       3.5955679877     2.8195505663    -3.0606772676
  20      H       2.3321896753     2.0082953434    -5.0247837557
  21      H       0.8739922235    -0.0078314921    -4.7914160552
  22      H       0.7253109022    -1.1117077204    -2.5840930658
  23      H       4.2288095514     2.6642865779    -0.8491814395
  24      H       4.3348939589     1.0215165361    -0.2248463446
  25      H       3.0002532507     2.0892225852     0.2691815619
  26      H       2.4653803826    -0.0659752459     0.4102853737
  27      H       2.2642983186    -1.5160762190    -0.5511132013
  28      H      -1.7250630411    -2.8071641621     1.2941569257
  29      H       0.4947975690    -2.7490385585     0.5670672013
  30      H      -4.2288176882    -0.4059533510     0.7968200956
  31      H      -3.8986334438    -2.0509904348     1.3351867127
  32      H      -4.8175663847    -0.5547632097     3.1298422947
  33      H      -3.4009933544     0.4755628049     3.0147289756
  34      H      -3.3504179560    -2.4978652951     3.8043892447
  35      H      -3.2366159390    -1.1399687542     4.9242601089
  36      H      -1.9364954433    -1.4225008895     3.7726687380
  37      H       0.6890920087     2.1347854856     0.3936505635
  38      H      -0.8640587089     2.3774844865    -0.1059501568
 ----------------------------------------------------------------
 Nuclear Repulsion Energy =        1313.85943071 hartrees
 There are       65 alpha and       65 beta electrons

-------------------------------------------------
-  Entering fldman on Sun Oct 13 18:09:03 2024  -
-------------------------------------------------

 Applying Cartesian multipole field
    Component          Value
    ---------          -----
     (2,0,0)        1.00000E-11
     (0,2,0)        2.00000E-11
     (0,0,2)       -3.00000E-11
 Nucleus-field energy     =    -0.0000000258 hartrees

-------------------------------------------------
-  Entering gesman on Sun Oct 13 18:09:03 2024  -
-------------------------------------------------

 Requested basis set is 6-311+G(d,p)
 There are 188 shells and 516 basis functions
 A cutoff of  1.0D-12 yielded  12704 shell pairs
 There are    101530 function pairs (    108318 Cartesian)
 Smallest overlap matrix eigenvalue = 2.04E-06
 Linear dependence detected in AO basis
 Tighter screening thresholds may be required for diffuse basis sets
 Use S2THRESH > 12 and THRESH = 14 in case of SCF convergence issues
 Number of orthogonalized atomic orbitals = 510
 Maximum deviation from orthogonality = 2.181E-11
 Guess MOs from SCF MO coefficient file
 Reading MOs from coefficient file
 Reading MOs from coefficient file

-------------------------------------------------
-  Entering scfman on Sun Oct 13 18:09:03 2024  -
-------------------------------------------------

 Long-range K will be added via erf
 Coulomb attenuation parameter = 0.2 bohr**(-1)
 A restricted hybrid HF-DFT SCF calculation will be
 performed using Pulay DIIS + Geometric Direct Minimization
 Exchange:     0.2220 Hartree-Fock + 1.0000 wB97X-D + LR-HF
```

```
 Correlation:  1.0000 wB97X-D
 Using Euler-Maclaurin-Lebedev (75,302) quadrature formula
 Dispersion:   Grimme D
 SCF converges when RMS gradient is below 1.0E-07
 Geometry optimization detected.  Setting ReadMinima to 0
 Setting SaveMinima to 0
 ---------------------------------------
  Cycle       Energy         DIIS Error
 ---------------------------------------
    1    -764.0635208545      8.48E-04
    2    -763.9569661445      1.75E-04
    3    -763.9574114862      1.62E-04
    4    -763.9581366904      2.76E-05
    5    -763.9581620491      1.32E-05
    6    -763.9581695843      4.76E-06
    7    -763.9581708159      1.89E-06
    8    -763.9581710330      6.30E-07
    9    -763.9581710648      3.43E-07
   10    -763.9581710705      9.26E-08 Convergence criterion met
 ---------------------------------------
 SCF time:  CPU 366.06 s  wall 615.91 s
 SCF   energy in the final basis set = -763.95817107
 Total energy in the final basis set = -763.95817107

-------------------------------------------------
-  Entering anlman on Sun Oct 13 18:19:19 2024  -
-------------------------------------------------



 --------------------------------------------------------------

                   Orbital Energies (a.u.)
 --------------------------------------------------------------


 Alpha MOs
 -- Occupied --
-14.8128 -14.8037 -14.7232 -14.7197 -10.6775 -10.6697 -10.6543 -10.6359
-10.6345 -10.6218 -10.6119 -10.5789 -10.5678 -10.5664 -10.5480 -10.5314
-10.4975 -10.4716  -1.4256  -1.4001  -1.3444  -1.2757  -1.2303  -1.2015
 -1.1832  -1.1442  -1.1023  -1.0801  -1.0610  -1.0536  -1.0279  -1.0087
 -0.9887  -0.9507  -0.9485  -0.9101  -0.9000  -0.8870  -0.8801  -0.8628
 -0.8526  -0.8413  -0.8390  -0.8184  -0.8082  -0.7938  -0.7872  -0.7820
 -0.7790  -0.7556  -0.7497  -0.7461  -0.7342  -0.7249  -0.7222  -0.7122
 -0.7035  -0.6728  -0.6547  -0.6481  -0.6389  -0.6317  -0.6278  -0.6230
 -0.6144
 -- Virtual --
 -0.2744  -0.2666  -0.2520  -0.2197  -0.1757  -0.1630  -0.1465  -0.1435
 -0.1345  -0.1251  -0.1174  -0.1135  -0.1105  -0.1038  -0.0987  -0.0932
 -0.0900  -0.0878  -0.0803  -0.0777  -0.0746  -0.0737  -0.0721  -0.0675
 -0.0655  -0.0586  -0.0578  -0.0523  -0.0499  -0.0447  -0.0404  -0.0345
 -0.0309  -0.0240  -0.0210  -0.0179  -0.0178  -0.0162  -0.0121  -0.0092
 -0.0051  -0.0039  -0.0004   0.0009   0.0083   0.0091   0.0139   0.0151
  0.0176   0.0186   0.0252   0.0285   0.0297   0.0360   0.0372   0.0396
  0.0434   0.0456   0.0473   0.0524   0.0544   0.0612   0.0624   0.0650
  0.0708   0.0727   0.0753   0.0775   0.0786   0.0808   0.0859   0.0900
  0.0957   0.0970   0.1018   0.1035   0.1090   0.1102   0.1152   0.1178
  0.1215   0.1259   0.1264   0.1316   0.1337   0.1369   0.1415   0.1472
  0.1530   0.1589   0.1599   0.1644   0.1694   0.1695   0.1747   0.1792
  0.1861   0.1894   0.1911   0.1960   0.2015   0.2057   0.2146   0.2284
  0.2339   0.2507   0.2551   0.2687   0.2817   0.3001   0.3024   0.3177
  0.3260   0.3274   0.3358   0.3472   0.3500   0.3571   0.3615   0.3658
  0.3707   0.3819   0.3831   0.3971   0.4005   0.4055   0.4109   0.4186
  0.4282   0.4352   0.4420   0.4454   0.4556   0.4653   0.4690   0.4802
  0.4838   0.4856   0.4926   0.4983   0.5028   0.5123   0.5146   0.5205
  0.5223   0.5246   0.5328   0.5363   0.5429   0.5487   0.5562   0.5621
  0.5645   0.5671   0.5711   0.5790   0.5829   0.5887   0.5961   0.6015
  0.6056   0.6183   0.6211   0.6255   0.6295   0.6365   0.6434   0.6586
  0.6642   0.6762   0.6808   0.6846   0.6960   0.7015   0.7056   0.7173
  0.7278   0.7307   0.7387   0.7467   0.7492   0.7590   0.7780   0.7918
  0.8007   0.8065   0.8106   0.8190   0.8288   0.8346   0.8433   0.8636
```

| | | | | | | | |
|---|---|---|---|---|---|---|---|
| 0.8666 | 0.8733 | 0.8861 | 0.8888 | 0.8999 | 0.9184 | 0.9249 | 0.9356 |
| 0.9431 | 0.9468 | 0.9736 | 0.9798 | 0.9919 | 1.0159 | 1.0239 | 1.0425 |
| 1.0505 | 1.0669 | 1.0942 | 1.1068 | 1.1285 | 1.1447 | 1.1670 | 1.1865 |
| 1.1935 | 1.2028 | 1.2376 | 1.2506 | 1.2644 | 1.2667 | 1.2916 | 1.3029 |
| 1.3159 | 1.3337 | 1.3394 | 1.3425 | 1.3544 | 1.3652 | 1.3703 | 1.3836 |
| 1.3861 | 1.3949 | 1.4047 | 1.4123 | 1.4255 | 1.4308 | 1.4361 | 1.4408 |
| 1.4436 | 1.4506 | 1.4535 | 1.4558 | 1.4696 | 1.4779 | 1.4886 | 1.4946 |
| 1.5015 | 1.5121 | 1.5154 | 1.5184 | 1.5222 | 1.5416 | 1.5431 | 1.5568 |
| 1.5674 | 1.5707 | 1.5722 | 1.5826 | 1.5920 | 1.5980 | 1.6051 | 1.6095 |
| 1.6198 | 1.6348 | 1.6401 | 1.6658 | 1.6696 | 1.6757 | 1.6784 | 1.6878 |
| 1.6968 | 1.7003 | 1.7066 | 1.7237 | 1.7315 | 1.7465 | 1.7658 | 1.7796 |
| 1.7872 | 1.7931 | 1.8072 | 1.8230 | 1.8337 | 1.8535 | 1.8556 | 1.8650 |
| 1.8902 | 1.8953 | 1.9037 | 1.9114 | 1.9376 | 1.9449 | 1.9625 | 1.9921 |
| 1.9984 | 2.0179 | 2.0296 | 2.0345 | 2.0547 | 2.0595 | 2.0837 | 2.0929 |
| 2.1090 | 2.1160 | 2.1265 | 2.1418 | 2.1581 | 2.1651 | 2.2056 | 2.2244 |
| 2.2281 | 2.2394 | 2.2458 | 2.2609 | 2.2773 | 2.2891 | 2.2983 | 2.3083 |
| 2.3116 | 2.3169 | 2.3510 | 2.3629 | 2.3676 | 2.3770 | 2.3867 | 2.3949 |
| 2.4148 | 2.4210 | 2.4309 | 2.4409 | 2.4503 | 2.4597 | 2.4741 | 2.4949 |
| 2.4994 | 2.5162 | 2.5220 | 2.5350 | 2.5413 | 2.5483 | 2.5534 | 2.5589 |
| 2.5732 | 2.5787 | 2.5818 | 2.5957 | 2.5996 | 2.6046 | 2.6165 | 2.6188 |
| 2.6378 | 2.6466 | 2.6507 | 2.6666 | 2.6706 | 2.6746 | 2.6835 | 2.7005 |
| 2.7049 | 2.7269 | 2.7334 | 2.7398 | 2.7436 | 2.7617 | 2.7742 | 2.7827 |
| 2.7867 | 2.8020 | 2.8078 | 2.8270 | 2.8414 | 2.8522 | 2.8826 | 2.8951 |
| 2.9119 | 2.9341 | 2.9478 | 2.9966 | 3.0550 | 3.0658 | 3.1040 | 3.1376 |
| 3.1437 | 3.1884 | 3.2523 | 3.2556 | 3.2883 | 3.3366 | 3.3495 | 3.3908 |
| 3.4168 | 3.4725 | 3.4994 | 3.5416 | 3.5573 | 3.6049 | 3.6336 | 3.6464 |
| 3.6617 | 3.6846 | 3.7025 | 3.7126 | 3.7354 | 3.7548 | 3.7761 | 3.7791 |
| 3.8241 | 3.8744 | 3.9005 | 3.9138 | 3.9452 | 4.0305 | 4.0368 | 4.0955 |
| 4.1051 | 4.2028 | 4.3025 | 4.3192 | 4.5859 | 4.6481 | 4.6680 | 4.7408 |
| 4.8814 | 5.0409 | 5.1688 | 23.5374 | 23.5555 | 23.7140 | 23.7194 | 23.7396 |
| 23.7733 | 23.7999 | 23.8327 | 23.8535 | 23.8964 | 23.9101 | 23.9616 | 23.9731 |
| 24.0440 | 35.4212 | 35.4427 | 35.4968 | 35.5903 | | | |

---

Ground-State Mulliken Net Atomic Charges

| Atom | | Charge (a.u.) |
|---|---|---|
| 1 | N | 0.455836 |
| 2 | C | 0.135123 |
| 3 | C | -0.037839 |
| 4 | C | -0.137675 |
| 5 | C | -0.189538 |
| 6 | C | -0.145917 |
| 7 | C | -0.741204 |
| 8 | C | -0.246486 |
| 9 | N | -0.138383 |
| 10 | C | -0.268152 |
| 11 | N | 0.056656 |
| 12 | C | 0.119552 |
| 13 | C | -0.115562 |
| 14 | C | 0.099124 |
| 15 | C | -0.363872 |
| 16 | C | -0.242935 |
| 17 | C | -0.526062 |
| 18 | N | -0.487413 |
| 19 | H | 0.219952 |
| 20 | H | 0.233591 |
| 21 | H | 0.226763 |
| 22 | H | 0.219506 |
| 23 | H | 0.232028 |
| 24 | H | 0.249125 |
| 25 | H | 0.177211 |
| 26 | H | 0.263959 |
| 27 | H | 0.243839 |
| 28 | H | 0.400510 |
| 29 | H | 0.223325 |
| 30 | H | 0.250083 |
| 31 | H | 0.210938 |
| 32 | H | 0.224127 |

```
    33 H                     0.200579
    34 H                     0.179297
    35 H                     0.217866
    36 H                     0.136360
    37 H                     0.302294
    38 H                     0.363395
  ----------------------------------------
  Sum of atomic charges =     2.000000

 -----------------------------------------------------------------
                    Cartesian Multipole Moments
 -----------------------------------------------------------------
    Charge (ESU x 10^10)
                 9.6064
    Dipole Moment (Debye)
         X       4.5566      Y      -1.3301      Z      -4.5475
       Tot       6.5735
    Quadrupole Moments (Debye-Ang)
        XX     -40.4541     XY      27.3957     YY     -63.8689
        XZ     -37.6873     YZ     -19.5005     ZZ     -46.5613
    Traceless Quadrupole Moments (Debye-Ang)
       QXX      29.5219    QYY     -40.7224    QZZ      11.2005
       QXY      82.1870    QXZ    -113.0619    QYZ     -58.5015
    Octopole Moments (Debye-Ang^2)
       XXX     -14.1528    XXY      24.3480    XYY      12.7889
       YYY     -17.1888    XXZ      34.0381    XYZ     -19.2645
       YYZ      18.4493    XZZ      22.7648    YZZ      31.2316
       ZZZ     -66.9882
    Traceless Octopole Moments (Debye-Ang^2)
       XXX    -404.9006    YYY    -603.3489    ZZZ    -874.3162
       XXY     250.0478    XXZ     554.0740    XYY     127.6313
       XYZ    -288.9681    XZZ     277.2693    YYZ     320.2422
       YZZ     353.3010
    Hexadecapole Moments (Debye-Ang^3)
      XXXX   -2718.0932   XXXY    -270.4570   XXYY    -547.3583
      XYYY    -447.7443   YYYY    -865.3618   XXXZ     877.5923
      XXYZ     -31.3949   XYYZ     276.3716   YYYZ     393.5771
      XXZZ    -757.1658   XYZZ       9.3488   YYZZ    -561.7423
      XZZZ     741.3239   YZZZ     239.3500   ZZZZ   -2027.4770
    Traceless Hexadecapole Moments (Debye-Ang^3)
      XXXX   -7455.4144   XXXY    3500.3777   XXXZ    6859.2402
      XXYY    4453.1797   XXYZ  -12314.2012   XXZZ    3002.2347
      XYYY  -15114.7901   XYYZ     589.6985   XYZZ   11614.4125
      XZZZ   -7448.9387   YYYY    2747.4399   YYYZ   14272.4010
      YYZZ   -7200.6196   YZZZ   -1958.1998   ZZZZ    4198.3849
 -----------------------------------------------------------------


-------------------------------------------------
-  Entering drvman on Sun Oct 13 18:19:19 2024  -
-------------------------------------------------

 Calculating analytic gradient of the SCF energy
 Gradient of SCF Energy
            1           2           3           4           5           6
    1  -0.0014196  -0.0026633   0.0027216  -0.0023088   0.0005871   0.0026313
    2  -0.0024674  -0.0025744   0.0020354  -0.0035013  -0.0001946   0.0019747
    3   0.0009008  -0.0017815   0.0040222   0.0009054   0.0016782  -0.0008200
            7           8           9          10          11          12
    1   0.0016915  -0.0010078   0.0001045   0.0128917  -0.0075890   0.0026577
    2   0.0023212   0.0021498   0.0021366  -0.0056877   0.0078889  -0.0192572
    3  -0.0006919   0.0009116  -0.0018982  -0.0009393   0.0040138   0.0004475
           13          14          15          16          17          18
    1  -0.0038758  -0.0031703   0.0013859  -0.0010848  -0.0003665   0.0124380
    2   0.0047584   0.0026343   0.0016154  -0.0005586   0.0000951   0.0050857
    3   0.0012136   0.0014808   0.0042816  -0.0059968   0.0011749  -0.0110323
           19          20          21          22          23          24
    1   0.0004662  -0.0000159  -0.0001696  -0.0006198   0.0010414   0.0003686
    2   0.0015233   0.0004583   0.0009263  -0.0004218   0.0009537  -0.0005354
    3  -0.0020232  -0.0013645  -0.0016131  -0.0026112   0.0006721  -0.0008430
           25          26          27          28          29          30
```

```
    1  -0.0012296  -0.0014012   0.0009618  -0.0018754  -0.0005159  -0.0021131
    2   0.0003243  -0.0021768  -0.0002700  -0.0020747  -0.0008290  -0.0005986
    3  -0.0010325   0.0027040  -0.0027370   0.0005604   0.0012844   0.0000358
          31          32          33          34          35          36
    1  -0.0004490  -0.0005451   0.0012105   0.0001068  -0.0006014  -0.0003915
    2  -0.0001282  -0.0001762   0.0000803  -0.0000196   0.0006507   0.0000380
    3  -0.0006360   0.0018894   0.0016396  -0.0007806   0.0005497  -0.0007700
          37          38
    1  -0.0060472  -0.0018040
    2   0.0033177   0.0005030
    3   0.0063852   0.0008201
 Max gradient component =       1.926E-02
 RMS gradient           =       3.521E-03
 Gradient time:  CPU 161.89 s  wall 267.73 s

-------------------------------------------------
-  Entering optman on Sun Oct 13 18:23:47 2024  -
-------------------------------------------------

 Geometry Optimization Parameters
   NAtoms,    NIC,     NZ,  NCons,   NDum,   NFix, NCnnct, MaxDiis
       38     272       0       0       0       0       0       0

 Cartesian Hessian Update
 Hessian updated using BFGS update


** GEOMETRY OPTIMIZATION IN DELOCALIZED INTERNAL COORDINATES **
   Searching for a Minimum

   Optimization Cycle:   2

                    Coordinates (Angstroms)
     ATOM                X               Y               Z
      1  N         2.0103882108    0.2330258094   -1.6392663548
      2  C         2.8364412907    1.3141191031   -1.7402939466
      3  C         2.9453112889    1.9570483501   -2.9661501424
      4  C         2.2401044669    1.5012337383   -4.0689167184
      5  C         1.4258193156    0.3817114993   -3.9445994963
      6  C         1.3380006053   -0.2294971944   -2.7237653784
      7  C         3.6386058367    1.7927258668   -0.5673914142
      8  C         1.8518202100   -0.5206965856   -0.3648769526
      9  N        -1.3560759116   -1.9175323019    0.9651808569
     10  C        -2.1794019761   -0.8465208507    0.9620563690
     11  N        -1.7469242399    0.3399817831    0.5893203170
     12  C        -0.4675242087    0.4712507207    0.2117157427
     13  C         0.4219884782   -0.6285662840    0.1219915585
     14  C        -0.0778295417   -1.8280314197    0.5451437145
     15  C        -3.5837950846   -1.0074036269    1.4426438652
     16  C        -3.7479747150   -0.5566588553    2.9056114322
     17  C        -3.0244234765   -1.4574537735    3.9017327375
     18  N        -0.0704544181    1.7403949676   -0.1503126996
     19  H         3.5955679877    2.8195505663   -3.0606772676
     20  H         2.3321896753    2.0082953434   -5.0247837557
     21  H         0.8739922235   -0.0078314921   -4.7914160552
     22  H         0.7253109022   -1.1117077204   -2.5840930658
     23  H         4.2288095514    2.6642865779   -0.8491814395
     24  H         4.3348939589    1.0215165361   -0.2248463446
     25  H         3.0002532507    2.0892225852    0.2691815619
     26  H         2.4653803826   -0.0659752459    0.4102853737
     27  H         2.2642983186   -1.5160762190   -0.5511132013
     28  H        -1.7250630411   -2.8071641621    1.2941569257
     29  H         0.4947975690   -2.7490385585    0.5670672013
     30  H        -4.2288176882   -0.4059533510    0.7968200956
     31  H        -3.8986334438   -2.0509904348    1.3351867127
     32  H        -4.8175663847   -0.5547632097    3.1298422947
     33  H        -3.4009933544    0.4755628049    3.0147289756
     34  H        -3.3504179560   -2.4978652951    3.8043892447
     35  H        -3.2366159390   -1.1399687542    4.9242601089
     36  H        -1.9364954433   -1.4225008895    3.7726687380
```

```
     37  H          0.6890920087    2.1347854856    0.3936505635
     38  H         -0.8640587089    2.3774844865   -0.1059501568
   Point Group: c1    Number of degrees of freedom:   108


   Energy is   -763.958171071

 Hessian updated using BFGS update
  internal optimization (0)

108 Hessian modes will be used to form the next step
  Hessian Eigenvalues:
     0.001845    0.002645    0.004020    0.004928    0.008113    0.010741
     0.013077    0.019045    0.019470    0.019925    0.021215    0.022027
     0.022608    0.023643    0.024048    0.024581    0.025255    0.026857
     0.027752    0.028198    0.029036    0.030899    0.035930    0.038323
     0.039064    0.040410    0.042822    0.043529    0.044651    0.046032
     0.048014    0.053835    0.055750    0.055783    0.066447    0.078420
     0.085487    0.094995    0.121620    0.121808    0.127014    0.130262
     0.131030    0.132811    0.135011    0.138477    0.143873    0.146409
     0.146930    0.147684    0.148627    0.150393    0.152262    0.152823
     0.154525    0.156322    0.179088    0.205895    0.209746    0.214017
     0.219757    0.228937    0.233515    0.237595    0.248239    0.252808
     0.264419    0.269024    0.280483    0.286405    0.298382    0.300120
     0.300604    0.301139    0.301637    0.302604    0.303958    0.304398
     0.304902    0.305171    0.306315    0.308586    0.310897    0.313813
     0.324334    0.329692    0.332323    0.334341    0.339157    0.340963
     0.347442    0.350309    0.355974    0.361741    0.378008    0.383611
     0.392526    0.397341    0.403854    0.413916    0.419843    0.423963
     0.432756    0.440462    0.455282    0.513503    0.635671    0.725315

 Minimum search - taking simple RFO step
 Searching for Lamda that Minimizes Along All modes
 Value Taken    Lamda =  -0.00954345
 Calculated Step too Large.  Step scaled by  0.502903 !!
 Step Taken.  Stepsize is  0.300000  0.002923

                         Maximum     Tolerance    Cnvgd?
        Gradient         0.010519     0.000800      NO
        Displacement     0.104107     0.001400      NO
        Energy change   -0.011042     0.000228      NO


 New Cartesian Coordinates Obtained by Inverse Iteration

 Displacement from previous Coordinates is:  0.461520
 ----------------------------------------------------------------
             Standard Nuclear Orientation (Angstroms)
    I     Atom           X                Y                Z
 ----------------------------------------------------------------
    1      N       2.0080272270     0.2437211959    -1.6216267746
    2      C       2.8464021305     1.3112407166    -1.7339502085
    3      C       2.9468599440     1.9424356880    -2.9667446906
    4      C       2.2260927754     1.4925077819    -4.0599948224
    5      C       1.3978128837     0.3824001063    -3.9182945733
    6      C       1.3149958787    -0.2190790415    -2.6919035423
    7      C       3.6653665063     1.7755934396    -0.5687670321
    8      C       1.8511611685    -0.4992188577    -0.3447646792
    9      N      -1.3571478596    -1.9121273335     0.9571287749
   10      C      -2.1942213200    -0.8470685369     0.9524587381
   11      N      -1.7567872704     0.3382976902     0.5879511778
   12      C      -0.4784086391     0.4952978587     0.2279786004
   13      C       0.4180691644    -0.6078932898     0.1318339683
   14      C      -0.0758725026    -1.8141346960     0.5411231332
   15      C      -3.5947686187    -1.0203454885     1.4268806816
   16      C      -3.7532444816    -0.5721842850     2.8961512230
   17      C      -3.0089976582    -1.4624010772     3.8865896050
   18      N      -0.1054456643     1.7531753257    -0.1448350950
   19      H       3.6095682532     2.7930139222    -3.0639225995
   20      H       2.3155593644     1.9922799267    -5.0178324524
```

```
  21      H       0.8305083375    -0.0121021238    -4.7509394210
  22      H       0.6948083120    -1.0906639640    -2.5279325802
  23      H       4.2615537212     2.6366654821    -0.8642545767
  24      H       4.3558132066     0.9978823161    -0.2301646421
  25      H       3.0490481338     2.0837504573     0.2801181167
  26      H       2.4642128540    -0.0276700015     0.4195297233
  27      H       2.2647513203    -1.4962551874    -0.5095845530
  28      H      -1.7169296383    -2.8047792969     1.2803840155
  29      H       0.5036679227    -2.7304389106     0.5516434598
  30      H      -4.2380381945    -0.4178966703     0.7825525870
  31      H      -3.9014787726    -2.0658652264     1.3212294361
  32      H      -4.8219358352    -0.5831431745     3.1185452109
  33      H      -3.4234329008     0.4659669591     2.9944037977
  34      H      -3.3217899203    -2.5074190137     3.7962884251
  35      H      -3.2160081999    -1.1486004026     4.9103529996
  36      H      -1.9216602876    -1.4134506452     3.7536859448
  37      H       0.7369346178     2.1387409132     0.2594000102
  38      H      -0.8750459582     2.4097674436    -0.0607173861
 ----------------------------------------------------------------
 Nuclear Repulsion Energy =        1315.13429208 hartrees
 There are       65 alpha and       65 beta electrons

--------------------------------------------------
-  Entering fldman on Sun Oct 13 18:23:47 2024  -
--------------------------------------------------

 Applying Cartesian multipole field
    Component          Value
    ---------          -----
     (2,0,0)        1.00000E-11
     (0,2,0)        2.00000E-11
     (0,0,2)       -3.00000E-11
 Nucleus-field energy     =    -0.0000000252 hartrees

--------------------------------------------------
-  Entering gesman on Sun Oct 13 18:23:47 2024  -
--------------------------------------------------

 Requested basis set is 6-311+G(d,p)
 There are 188 shells and 516 basis functions
 A cutoff of  1.0D-12 yielded  12718 shell pairs
 There are    101596 function pairs (    108385 Cartesian)
 Smallest overlap matrix eigenvalue = 2.01E-06
 Linear dependence detected in AO basis
 Tighter screening thresholds may be required for diffuse basis sets
 Use S2THRESH > 12 and THRESH = 14 in case of SCF convergence issues
 Number of orthogonalized atomic orbitals = 510
 Maximum deviation from orthogonality = 2.080E-11
 Guess MOs from SCF MO coefficient file
 Reading MOs from coefficient file
 Reading MOs from coefficient file

--------------------------------------------------
-  Entering scfman on Sun Oct 13 18:23:47 2024  -
--------------------------------------------------

 Long-range K will be added via erf
 Coulomb attenuation parameter = 0.2 bohr**(-1)
 A restricted hybrid HF-DFT SCF calculation will be
 performed using Pulay DIIS + Geometric Direct Minimization
 Exchange:     0.2220 Hartree-Fock + 1.0000 wB97X-D + LR-HF
 Correlation:  1.0000 wB97X-D
 Using Euler-Maclaurin-Lebedev (75,302) quadrature formula
 Dispersion:   Grimme D
 SCF converges when RMS gradient is below 1.0E-07
 Geometry optimization detected.  Setting ReadMinima to 0
 Setting SaveMinima to 0
 ---------------------------------------
  Cycle       Energy         DIIS Error
 ---------------------------------------
```

```
    1    -764.0007607940      4.69E-04
    2    -763.9615287294      1.29E-04
    3    -763.9616940479      1.32E-04
    4    -763.9622022114      1.72E-05
    5    -763.9622149850      1.03E-05
    6    -763.9622188432      3.59E-06
    7    -763.9622195276      1.30E-06
    8    -763.9622196143      4.75E-07
    9    -763.9622196293      1.76E-07
   10    -763.9622196312      5.67E-08 Convergence criterion met
 ---------------------------------------
 SCF time:  CPU 362.95 s  wall 604.72 s
 SCF   energy in the final basis set = -763.96221963
 Total energy in the final basis set = -763.96221963

--------------------------------------------------
-  Entering anlman on Sun Oct 13 18:33:52 2024  -
--------------------------------------------------


 --------------------------------------------------------------

                   Orbital Energies (a.u.)
 --------------------------------------------------------------

 Alpha MOs
 -- Occupied --
-14.8136 -14.7992 -14.7284 -14.7156 -10.6775 -10.6681 -10.6516 -10.6376
-10.6336 -10.6229 -10.6100 -10.5809 -10.5698 -10.5681 -10.5500 -10.5303
-10.4973 -10.4713  -1.4222  -1.4028  -1.3456  -1.2764  -1.2305  -1.2017
 -1.1856  -1.1438  -1.1016  -1.0824  -1.0586  -1.0544  -1.0299  -1.0084
 -0.9900  -0.9513  -0.9472  -0.9118  -0.9029  -0.8887  -0.8813  -0.8636
 -0.8542  -0.8415  -0.8403  -0.8195  -0.8088  -0.7940  -0.7890  -0.7826
 -0.7787  -0.7570  -0.7510  -0.7476  -0.7379  -0.7266  -0.7216  -0.7152
 -0.7030  -0.6721  -0.6551  -0.6464  -0.6387  -0.6310  -0.6264  -0.6221
 -0.6153
 -- Virtual --
 -0.2702  -0.2679  -0.2502  -0.2216  -0.1752  -0.1625  -0.1459  -0.1431
 -0.1348  -0.1246  -0.1173  -0.1136  -0.1100  -0.1030  -0.0986  -0.0934
 -0.0902  -0.0879  -0.0808  -0.0776  -0.0762  -0.0737  -0.0720  -0.0679
 -0.0652  -0.0585  -0.0577  -0.0521  -0.0497  -0.0450  -0.0402  -0.0339
 -0.0299  -0.0253  -0.0212  -0.0183  -0.0176  -0.0157  -0.0121  -0.0094
 -0.0048  -0.0041  -0.0002   0.0006   0.0084   0.0091   0.0129   0.0149
  0.0180   0.0190   0.0259   0.0278   0.0299   0.0356   0.0371   0.0400
  0.0436   0.0453   0.0482   0.0520   0.0556   0.0612   0.0625   0.0643
  0.0710   0.0737   0.0749   0.0780   0.0793   0.0807   0.0866   0.0904
  0.0948   0.0967   0.1018   0.1039   0.1088   0.1095   0.1153   0.1173
  0.1228   0.1258   0.1263   0.1312   0.1347   0.1376   0.1428   0.1478
  0.1539   0.1590   0.1599   0.1609   0.1684   0.1703   0.1740   0.1817
  0.1861   0.1884   0.1921   0.1964   0.2012   0.2055   0.2162   0.2272
  0.2329   0.2486   0.2520   0.2694   0.2823   0.2978   0.3024   0.3184
  0.3222   0.3279   0.3361   0.3479   0.3526   0.3566   0.3603   0.3654
  0.3715   0.3821   0.3847   0.3963   0.4020   0.4059   0.4105   0.4190
  0.4283   0.4361   0.4402   0.4462   0.4567   0.4642   0.4697   0.4771
  0.4843   0.4882   0.4926   0.4991   0.5046   0.5121   0.5156   0.5211
  0.5237   0.5267   0.5328   0.5357   0.5434   0.5484   0.5559   0.5594
  0.5652   0.5664   0.5718   0.5759   0.5832   0.5891   0.5969   0.6016
  0.6032   0.6187   0.6220   0.6254   0.6309   0.6357   0.6428   0.6589
  0.6637   0.6750   0.6784   0.6834   0.6978   0.7015   0.7055   0.7131
  0.7315   0.7336   0.7385   0.7473   0.7508   0.7644   0.7786   0.7895
  0.8034   0.8064   0.8107   0.8221   0.8285   0.8352   0.8411   0.8624
  0.8663   0.8721   0.8865   0.8898   0.9024   0.9204   0.9320   0.9352
  0.9420   0.9494   0.9718   0.9757   0.9892   1.0121   1.0217   1.0441
  1.0488   1.0669   1.0987   1.1046   1.1318   1.1459   1.1692   1.1900
  1.1906   1.1983   1.2379   1.2488   1.2627   1.2668   1.2888   1.3042
  1.3183   1.3327   1.3364   1.3426   1.3530   1.3634   1.3713   1.3826
  1.3855   1.3950   1.4030   1.4104   1.4203   1.4282   1.4348   1.4409
  1.4424   1.4482   1.4510   1.4566   1.4683   1.4825   1.4893   1.4989
  1.5019   1.5093   1.5139   1.5185   1.5235   1.5417   1.5450   1.5593
  1.5695   1.5716   1.5724   1.5829   1.5953   1.6008   1.6058   1.6093
```

| | | | | | | | |
|---|---|---|---|---|---|---|---|
| 1.6211 | 1.6353 | 1.6432 | 1.6646 | 1.6712 | 1.6767 | 1.6781 | 1.6927 |
| 1.6973 | 1.7003 | 1.7162 | 1.7256 | 1.7321 | 1.7469 | 1.7653 | 1.7816 |
| 1.7875 | 1.7913 | 1.8090 | 1.8247 | 1.8301 | 1.8517 | 1.8627 | 1.8633 |
| 1.8878 | 1.8997 | 1.9107 | 1.9135 | 1.9388 | 1.9442 | 1.9508 | 1.9901 |
| 2.0000 | 2.0120 | 2.0306 | 2.0351 | 2.0560 | 2.0626 | 2.0830 | 2.0881 |
| 2.1121 | 2.1200 | 2.1288 | 2.1393 | 2.1600 | 2.1663 | 2.2038 | 2.2249 |
| 2.2284 | 2.2400 | 2.2470 | 2.2615 | 2.2805 | 2.2883 | 2.2966 | 2.3068 |
| 2.3120 | 2.3139 | 2.3493 | 2.3630 | 2.3690 | 2.3781 | 2.3861 | 2.3954 |
| 2.4168 | 2.4187 | 2.4269 | 2.4417 | 2.4504 | 2.4624 | 2.4704 | 2.4938 |
| 2.4983 | 2.5198 | 2.5227 | 2.5340 | 2.5418 | 2.5469 | 2.5540 | 2.5616 |
| 2.5770 | 2.5800 | 2.5848 | 2.5954 | 2.5971 | 2.6054 | 2.6141 | 2.6214 |
| 2.6408 | 2.6493 | 2.6565 | 2.6691 | 2.6717 | 2.6761 | 2.6864 | 2.7029 |
| 2.7077 | 2.7262 | 2.7294 | 2.7412 | 2.7455 | 2.7656 | 2.7740 | 2.7799 |
| 2.7905 | 2.8050 | 2.8094 | 2.8326 | 2.8437 | 2.8564 | 2.8853 | 2.8946 |
| 2.9175 | 2.9276 | 2.9507 | 2.9972 | 3.0599 | 3.0666 | 3.1295 | 3.1437 |
| 3.1444 | 3.1884 | 3.2504 | 3.2563 | 3.2916 | 3.3366 | 3.3538 | 3.3940 |
| 3.4172 | 3.4738 | 3.5043 | 3.5457 | 3.5613 | 3.6057 | 3.6324 | 3.6446 |
| 3.6654 | 3.6887 | 3.7026 | 3.7147 | 3.7389 | 3.7562 | 3.7750 | 3.7816 |
| 3.8287 | 3.8741 | 3.9032 | 3.9134 | 3.9396 | 4.0065 | 4.0333 | 4.0876 |
| 4.1039 | 4.2025 | 4.3002 | 4.3103 | 4.6000 | 4.6539 | 4.6788 | 4.7415 |
| 4.8915 | 5.0432 | 5.1826 | 23.5395 | 23.5612 | 23.7145 | 23.7233 | 23.7406 |
| 23.7696 | 23.7986 | 23.8308 | 23.8564 | 23.8954 | 23.9118 | 23.9624 | 23.9741 |
| 24.0416 | 35.4222 | 35.4448 | 35.5019 | 35.5992 | | | |

Ground-State Mulliken Net Atomic Charges

| Atom | Charge (a.u.) |
|---|---|
| 1 N | 0.436255 |
| 2 C | 0.140408 |
| 3 C | -0.037972 |
| 4 C | -0.138766 |
| 5 C | -0.186098 |
| 6 C | -0.142466 |
| 7 C | -0.751031 |
| 8 C | -0.254415 |
| 9 N | -0.146959 |
| 10 C | -0.261917 |
| 11 N | 0.044482 |
| 12 C | 0.150193 |
| 13 C | -0.128328 |
| 14 C | 0.107674 |
| 15 C | -0.363660 |
| 16 C | -0.244768 |
| 17 C | -0.528341 |
| 18 N | -0.469000 |
| 19 H | 0.219596 |
| 20 H | 0.234485 |
| 21 H | 0.227231 |
| 22 H | 0.220661 |
| 23 H | 0.232127 |
| 24 H | 0.250683 |
| 25 H | 0.179135 |
| 26 H | 0.263210 |
| 27 H | 0.244067 |
| 28 H | 0.398686 |
| 29 H | 0.221802 |
| 30 H | 0.249659 |
| 31 H | 0.211780 |
| 32 H | 0.224197 |
| 33 H | 0.200908 |
| 34 H | 0.179774 |
| 35 H | 0.217834 |
| 36 H | 0.137486 |
| 37 H | 0.286072 |
| 38 H | 0.375316 |

Sum of atomic charges = 2.000000

```
 -----------------------------------------------------------------
                   Cartesian Multipole Moments
 -----------------------------------------------------------------
    Charge (ESU x 10^10)
                 9.6064
    Dipole Moment (Debye)
         X       4.7307      Y      -1.0393      Z      -4.7074
       Tot       6.7542
    Quadrupole Moments (Debye-Ang)
        XX     -39.9677     XY      27.5889     YY     -63.8551
        XZ     -37.5679     YZ     -19.6983     ZZ     -47.1560
    Traceless Quadrupole Moments (Debye-Ang)
       QXX      31.0756    QYY     -40.5864    QZZ       9.5107
       QXY      82.7666    QXZ    -112.7037    QYZ     -59.0949
    Octopole Moments (Debye-Ang^2)
       XXX      -9.9669    XXY      24.6666    XYY      12.7813
       YYY     -16.0276    XXZ      33.1391    XYZ     -19.6043
       YYZ      17.9205    XZZ      22.9140    YZZ      30.6980
       ZZZ     -66.9966
    Traceless Octopole Moments (Debye-Ang^2)
       XXX    -381.0589    YYY    -594.4467    ZZZ    -861.5156
       XXY     251.9879    XXZ     544.8971    XYY     114.5341
       XYZ    -294.0639    XZZ     266.5248    YYZ     316.6185
       YZZ     342.4588
    Hexadecapole Moments (Debye-Ang^3)
      XXXX   -2710.0011   XXXY    -267.0154   XXYY    -548.0881
      XYYY    -452.1017   YYYY    -861.2393   XXXZ     867.2040
      XXYZ     -31.5227   XYYZ     273.8298   YYYZ     394.4157
      XXZZ    -756.9080   XYZZ       8.6998   YYZZ    -559.0927
      XZZZ     738.7447   YZZZ     237.7620   ZZZZ   -2010.3484
    Traceless Hexadecapole Moments (Debye-Ang^3)
      XXXX   -6988.2656   XXXY    3932.1605   XXXZ    6466.3915
      XXYY    4272.7139   XXYZ  -12308.2823   XXZZ    2715.5517
      XYYY  -15501.9011   XYYZ     555.4476   XYZZ   11569.7406
      XZZZ   -7021.8391   YYYY    2939.7765   YYYZ   14418.4632
      YYZZ   -7212.4903   YZZZ   -2110.1809   ZZZZ    4496.9387
 -----------------------------------------------------------------

--------------------------------------------------
-  Entering drvman on Sun Oct 13 18:33:52 2024  -
--------------------------------------------------

 Calculating analytic gradient of the SCF energy
 Gradient of SCF Energy
           1           2           3           4           5           6
    1  -0.0001421  -0.0014437   0.0008852  -0.0005175   0.0000944   0.0012439
    2  -0.0002669  -0.0006431   0.0004609  -0.0004884  -0.0004759   0.0002732
    3   0.0011011  -0.0004378   0.0015824  -0.0001480   0.0010334   0.0001011
           7           8           9          10          11          12
    1   0.0006322   0.0003252   0.0007473   0.0035148  -0.0011402   0.0000175
    2   0.0008069   0.0010395   0.0020502  -0.0036277   0.0031898  -0.0089797
    3  -0.0002912  -0.0012543  -0.0013441   0.0000217   0.0016026   0.0047533
          13          14          15          16          17          18
    1  -0.0046850  -0.0007839   0.0007283  -0.0009242  -0.0004215   0.0092147
    2   0.0038859  -0.0012923   0.0010258  -0.0003042   0.0000220   0.0031460
    3  -0.0006053   0.0012047   0.0018594  -0.0028332   0.0010660  -0.0111641
          19          20          21          22          23          24
    1   0.0000091  -0.0000663   0.0000066  -0.0001028   0.0000645   0.0002068
    2   0.0003319  -0.0000513   0.0004400  -0.0001144  -0.0000150  -0.0001706
    3  -0.0007200  -0.0002695  -0.0003890  -0.0015021   0.0001283  -0.0002126
          25          26          27          28          29          30
    1  -0.0005648  -0.0005468  -0.0000119  -0.0008840  -0.0001592  -0.0008041
    2   0.0000811  -0.0013458   0.0001337  -0.0004879  -0.0004215  -0.0006974
    3  -0.0006758   0.0010900  -0.0015460   0.0001216   0.0008250  -0.0000314
          31          32          33          34          35          36
    1  -0.0002023   0.0000250   0.0006648   0.0000948  -0.0001336   0.0000376
    2  -0.0000943   0.0002111  -0.0000712  -0.0000155   0.0001588  -0.0000530
    3  -0.0001802   0.0006686   0.0008443  -0.0003583   0.0000393  -0.0002347
          37          38
    1  -0.0049213  -0.0000577
```

```
    2   0.0030890  -0.0007299
    3   0.0057186   0.0004365
 Max gradient component =       1.116E-02
 RMS gradient           =       2.140E-03
 Gradient time:  CPU 158.42 s  wall 264.25 s

-------------------------------------------------
-  Entering optman on Sun Oct 13 18:38:17 2024  -
-------------------------------------------------

 Geometry Optimization Parameters
   NAtoms,    NIC,     NZ,  NCons,   NDum,   NFix, NCnnct, MaxDiis
       38     272       0       0       0       0       0       0

 Cartesian Hessian Update
 Hessian updated using BFGS update


** GEOMETRY OPTIMIZATION IN DELOCALIZED INTERNAL COORDINATES **
   Searching for a Minimum

   Optimization Cycle:   3

                    Coordinates (Angstroms)
     ATOM                X               Y               Z
      1  N         2.0080272270    0.2437211959   -1.6216267746
      2  C         2.8464021305    1.3112407166   -1.7339502085
      3  C         2.9468599440    1.9424356880   -2.9667446906
      4  C         2.2260927754    1.4925077819   -4.0599948224
      5  C         1.3978128837    0.3824001063   -3.9182945733
      6  C         1.3149958787   -0.2190790415   -2.6919035423
      7  C         3.6653665063    1.7755934396   -0.5687670321
      8  C         1.8511611685   -0.4992188577   -0.3447646792
      9  N        -1.3571478596   -1.9121273335    0.9571287749
     10  C        -2.1942213200   -0.8470685369    0.9524587381
     11  N        -1.7567872704    0.3382976902    0.5879511778
     12  C        -0.4784086391    0.4952978587    0.2279786004
     13  C         0.4180691644   -0.6078932898    0.1318339683
     14  C        -0.0758725026   -1.8141346960    0.5411231332
     15  C        -3.5947686187   -1.0203454885    1.4268806816
     16  C        -3.7532444816   -0.5721842850    2.8961512230
     17  C        -3.0089976582   -1.4624010772    3.8865896050
     18  N        -0.1054456643    1.7531753257   -0.1448350950
     19  H         3.6095682532    2.7930139222   -3.0639225995
     20  H         2.3155593644    1.9922799267   -5.0178324524
     21  H         0.8305083375   -0.0121021238   -4.7509394210
     22  H         0.6948083120   -1.0906639640   -2.5279325802
     23  H         4.2615537212    2.6366654821   -0.8642545767
     24  H         4.3558132066    0.9978823161   -0.2301646421
     25  H         3.0490481338    2.0837504573    0.2801181167
     26  H         2.4642128540   -0.0276700015    0.4195297233
     27  H         2.2647513203   -1.4962551874   -0.5095845530
     28  H        -1.7169296383   -2.8047792969    1.2803840155
     29  H         0.5036679227   -2.7304389106    0.5516434598
     30  H        -4.2380381945   -0.4178966703    0.7825525870
     31  H        -3.9014787726   -2.0658652264    1.3212294361
     32  H        -4.8219358352   -0.5831431745    3.1185452109
     33  H        -3.4234329008    0.4659669591    2.9944037977
     34  H        -3.3217899203   -2.5074190137    3.7962884251
     35  H        -3.2160081999   -1.1486004026    4.9103529996
     36  H        -1.9216602876   -1.4134506452    3.7536859448
     37  H         0.7369346178    2.1387409132    0.2594000102
     38  H        -0.8750459582    2.4097674436   -0.0607173861
   Point Group: c1    Number of degrees of freedom:   108


   Energy is   -763.962219631

 Hessian updated using BFGS update
  internal optimization (0)
```

```
108 Hessian modes will be used to form the next step
  Hessian Eigenvalues:
     0.001843    0.002646    0.004020    0.004775    0.007434    0.009852
     0.013136    0.019046    0.019480    0.019971    0.021182    0.022025
     0.022624    0.023692    0.024051    0.024575    0.025280    0.026852
     0.027982    0.028188    0.028865    0.030403    0.035819    0.037533
     0.038809    0.040629    0.042726    0.043557    0.044568    0.045957
     0.048014    0.053711    0.054075    0.055809    0.066556    0.079232
     0.085571    0.095066    0.121723    0.122433    0.127114    0.130981
     0.131766    0.132809    0.135914    0.139422    0.143715    0.145272
     0.146943    0.147684    0.148569    0.152004    0.152724    0.152799
     0.154357    0.157376    0.192279    0.206108    0.209612    0.214356
     0.219617    0.228802    0.237296    0.247342    0.249816    0.253509
     0.266952    0.271404    0.281845    0.286439    0.298893    0.300128
     0.300599    0.301207    0.301795    0.303066    0.304001    0.304821
     0.305012    0.305233    0.306351    0.308499    0.311644    0.313823
     0.323417    0.331861    0.332331    0.334748    0.339125    0.340970
     0.345266    0.351112    0.358686    0.361584    0.377850    0.384431
     0.391764    0.399918    0.404676    0.415208    0.416967    0.426646
     0.433418    0.441195    0.455670    0.516627    0.637728    0.730309

 Minimum search - taking simple RFO step
 Searching for Lamda that Minimizes Along All modes
 Value Taken    Lamda =  -0.00714235
 Calculated Step too Large.  Step scaled by  0.459185 !!
 Step Taken.  Stepsize is  0.300000  0.002743

                              Maximum     Tolerance    Cnvgd?
         Gradient            0.005017      0.000800      NO
         Displacement        0.112271      0.001400      NO
         Energy change      -0.004049      0.000228      NO


 New Cartesian Coordinates Obtained by Inverse Iteration

 Displacement from previous Coordinates is:  0.454835
 ----------------------------------------------------------------
             Standard Nuclear Orientation (Angstroms)
    I     Atom           X                Y                Z
 ----------------------------------------------------------------
    1      N       2.0062401795     0.2557040402    -1.6078544297
    2      C       2.8616966642     1.3076775566    -1.7333382491
    3      C       2.9605717591     1.9258473163    -2.9725730424
    4      C       2.2245262066     1.4787482915    -4.0564593198
    5      C       1.3782327684     0.3830990901    -3.8995792080
    6      C       1.2951661299    -0.2052069328    -2.6669153547
    7      C       3.6975818038     1.7627890339    -0.5763892732
    8      C       1.8476154950    -0.4737474740    -0.3226274795
    9      N      -1.3612830894    -1.9075523362     0.9478600582
   10      C      -2.2076002441    -0.8466394949     0.9471642567
   11      N      -1.7742497952     0.3378262787     0.5907063511
   12      C      -0.4961545957     0.5113207271     0.2370127949
   13      C       0.4122413629    -0.5899512524     0.1373500252
   14      C      -0.0786585717    -1.7974644775     0.5334707703
   15      C      -3.6056935537    -1.0330063036     1.4174275637
   16      C      -3.7617043974    -0.5898742880     2.8914484914
   17      C      -2.9949447511    -1.4676668914     3.8748444995
   18      N      -0.1458551540     1.7646270019    -0.1221831346
   19      H       3.6372834464     2.7636709816    -3.0778200289
   20      H       2.3155806583     1.9698255833    -5.0182332947
   21      H       0.7971690920    -0.0120630262    -4.7218597384
   22      H       0.6624395397    -1.0629252903    -2.4812319402
   23      H       4.3059134499     2.6111475977    -0.8833270932
   24      H       4.3769663686     0.9750115824    -0.2393995633
   25      H       3.0998379586     2.0866145401     0.2804365808
   26      H       2.4528278333     0.0183113859     0.4346954555
   27      H       2.2707733232    -1.4697599501    -0.4655545741
   28      H      -1.7121505010    -2.8049316789     1.2642085761
   29      H       0.5064691674    -2.7100095846     0.5342883415
```

```
   30      H      -4.2515516896   -0.4297418278    0.7770792053
   31      H      -3.9032282810   -2.0804840900    1.3095017213
   32      H      -4.8293366186   -0.6193350742    3.1158442326
   33      H      -3.4514490880    0.4544936047    2.9832992049
   34      H      -3.2901595860   -2.5180369685    3.7881488993
   35      H      -3.1998557804   -1.1588615123    4.9003824567
   36      H      -1.9093813754   -1.3994611345    3.7366606603
   37      H       0.7564560816    2.1316109283    0.1387990597
   38      H      -0.8923322159    2.4383940477    0.0047165187
 ----------------------------------------------------------------
 Nuclear Repulsion Energy =        1315.59543220 hartrees
 There are       65 alpha and       65 beta electrons

--------------------------------------------------
-  Entering fldman on Sun Oct 13 18:38:17 2024  -
--------------------------------------------------

 Applying Cartesian multipole field
    Component          Value
    ---------          -----
     (2,0,0)        1.00000E-11
     (0,2,0)        2.00000E-11
     (0,0,2)       -3.00000E-11
 Nucleus-field energy     =    -0.0000000247 hartrees

--------------------------------------------------
-  Entering gesman on Sun Oct 13 18:38:17 2024  -
--------------------------------------------------

 Requested basis set is 6-311+G(d,p)
 There are 188 shells and 516 basis functions
 A cutoff of  1.0D-12 yielded  12722 shell pairs
 There are    101634 function pairs (    108408 Cartesian)
 Smallest overlap matrix eigenvalue = 1.91E-06
 Linear dependence detected in AO basis
 Tighter screening thresholds may be required for diffuse basis sets
 Use S2THRESH > 12 and THRESH = 14 in case of SCF convergence issues
 Number of orthogonalized atomic orbitals = 510
 Maximum deviation from orthogonality = 2.686E-11
 Guess MOs from SCF MO coefficient file
 Reading MOs from coefficient file
 Reading MOs from coefficient file

--------------------------------------------------
-  Entering scfman on Sun Oct 13 18:38:17 2024  -
--------------------------------------------------

 Long-range K will be added via erf
 Coulomb attenuation parameter = 0.2 bohr**(-1)
 A restricted hybrid HF-DFT SCF calculation will be
 performed using Pulay DIIS + Geometric Direct Minimization
 Exchange:     0.2220 Hartree-Fock + 1.0000 wB97X-D + LR-HF
 Correlation:  1.0000 wB97X-D
 Using Euler-Maclaurin-Lebedev (75,302) quadrature formula
 Dispersion:   Grimme D
 SCF converges when RMS gradient is below 1.0E-07
 Geometry optimization detected.  Setting ReadMinima to 0
 Setting SaveMinima to 0
 ---------------------------------------
  Cycle       Energy         DIIS Error
 ---------------------------------------
    1    -763.9952850814      4.57E-04
    2    -763.9644557363      1.18E-04
    3    -763.9646095127      1.21E-04
    4    -763.9650411042      1.71E-05
    5    -763.9650541069      9.26E-06
    6    -763.9650572915      3.52E-06
    7    -763.9650578890      1.26E-06
    8    -763.9650579678      4.22E-07
    9    -763.9650579796      1.59E-07
```

```
   10    -763.9650579810      5.25E-08 Convergence criterion met
 ---------------------------------------
 SCF time:  CPU 369.44 s  wall 622.24 s
 SCF   energy in the final basis set = -763.96505798
 Total energy in the final basis set = -763.96505798

--------------------------------------------------
-  Entering anlman on Sun Oct 13 18:48:39 2024  -
--------------------------------------------------



 --------------------------------------------------------------

                   Orbital Energies (a.u.)
 --------------------------------------------------------------

 Alpha MOs
 -- Occupied --
-14.8149 -14.7950 -14.7332 -14.7123 -10.6777 -10.6658 -10.6491 -10.6393
-10.6337 -10.6243 -10.6082 -10.5828 -10.5717 -10.5699 -10.5514 -10.5287
-10.4965 -10.4706  -1.4198  -1.4044  -1.3473  -1.2772  -1.2312  -1.2019
 -1.1874  -1.1433  -1.1012  -1.0840  -1.0569  -1.0547  -1.0314  -1.0078
 -0.9910  -0.9528  -0.9461  -0.9134  -0.9054  -0.8893  -0.8822  -0.8645
 -0.8554  -0.8422  -0.8408  -0.8204  -0.8087  -0.7950  -0.7905  -0.7827
 -0.7777  -0.7589  -0.7512  -0.7484  -0.7413  -0.7282  -0.7209  -0.7168
 -0.7022  -0.6715  -0.6562  -0.6455  -0.6379  -0.6299  -0.6252  -0.6220
 -0.6151
 -- Virtual --
 -0.2702  -0.2652  -0.2479  -0.2232  -0.1749  -0.1621  -0.1458  -0.1429
 -0.1353  -0.1241  -0.1175  -0.1137  -0.1098  -0.1023  -0.0987  -0.0940
 -0.0904  -0.0877  -0.0812  -0.0781  -0.0775  -0.0739  -0.0719  -0.0682
 -0.0648  -0.0586  -0.0574  -0.0521  -0.0493  -0.0451  -0.0402  -0.0332
 -0.0292  -0.0278  -0.0210  -0.0188  -0.0174  -0.0155  -0.0125  -0.0092
 -0.0049  -0.0043  -0.0003   0.0003   0.0083   0.0090   0.0115   0.0149
  0.0183   0.0196   0.0266   0.0268   0.0299   0.0346   0.0372   0.0402
  0.0435   0.0452   0.0485   0.0516   0.0566   0.0613   0.0629   0.0637
  0.0707   0.0743   0.0747   0.0785   0.0797   0.0808   0.0867   0.0914
  0.0936   0.0966   0.1015   0.1044   0.1083   0.1094   0.1157   0.1170
  0.1240   0.1255   0.1272   0.1308   0.1354   0.1373   0.1429   0.1490
  0.1541   0.1555   0.1593   0.1617   0.1680   0.1707   0.1734   0.1832
  0.1855   0.1880   0.1929   0.1982   0.2025   0.2054   0.2183   0.2253
  0.2318   0.2420   0.2534   0.2705   0.2830   0.2969   0.3025   0.3183
  0.3190   0.3286   0.3362   0.3482   0.3534   0.3574   0.3596   0.3654
  0.3717   0.3829   0.3853   0.3954   0.4027   0.4059   0.4102   0.4196
  0.4282   0.4345   0.4397   0.4477   0.4575   0.4635   0.4691   0.4770
  0.4847   0.4886   0.4934   0.4998   0.5059   0.5116   0.5168   0.5220
  0.5244   0.5289   0.5325   0.5359   0.5434   0.5478   0.5549   0.5579
  0.5654   0.5665   0.5721   0.5728   0.5840   0.5908   0.5978   0.5992
  0.6039   0.6189   0.6234   0.6261   0.6321   0.6363   0.6440   0.6588
  0.6634   0.6738   0.6783   0.6839   0.6981   0.7029   0.7073   0.7113
  0.7313   0.7329   0.7451   0.7485   0.7516   0.7690   0.7802   0.7855
  0.8019   0.8060   0.8121   0.8208   0.8294   0.8334   0.8421   0.8622
  0.8658   0.8716   0.8872   0.8915   0.9043   0.9178   0.9318   0.9394
  0.9445   0.9523   0.9691   0.9741   0.9878   1.0108   1.0222   1.0451
  1.0473   1.0672   1.0949   1.1020   1.1330   1.1475   1.1702   1.1872
  1.1944   1.1955   1.2353   1.2462   1.2592   1.2673   1.2853   1.3052
  1.3195   1.3289   1.3352   1.3417   1.3511   1.3621   1.3720   1.3817
  1.3887   1.3958   1.4013   1.4085   1.4108   1.4273   1.4323   1.4395
  1.4417   1.4479   1.4502   1.4562   1.4704   1.4847   1.4912   1.5019
  1.5031   1.5089   1.5122   1.5200   1.5246   1.5407   1.5469   1.5602
  1.5702   1.5712   1.5726   1.5821   1.5954   1.6015   1.6058   1.6099
  1.6223   1.6355   1.6441   1.6642   1.6725   1.6762   1.6774   1.6924
  1.7005   1.7013   1.7245   1.7302   1.7325   1.7451   1.7617   1.7841
  1.7860   1.7901   1.8107   1.8241   1.8278   1.8513   1.8590   1.8670
  1.8865   1.8971   1.9106   1.9251   1.9342   1.9451   1.9513   1.9896
  2.0000   2.0114   2.0318   2.0380   2.0576   2.0654   2.0823   2.0888
  2.1123   2.1233   2.1309   2.1363   2.1620   2.1681   2.2031   2.2258
  2.2302   2.2405   2.2467   2.2613   2.2817   2.2853   2.2936   2.3046
  2.3113   2.3133   2.3461   2.3631   2.3688   2.3782   2.3852   2.3963
  2.4147   2.4186   2.4260   2.4424   2.4527   2.4638   2.4682   2.4917
```

```
 2.4999   2.5216   2.5231   2.5332   2.5425   2.5450   2.5552   2.5637
 2.5763   2.5806   2.5859   2.5946   2.5958   2.6052   2.6127   2.6221
 2.6418   2.6515   2.6590   2.6711   2.6757   2.6777   2.6918   2.7054
 2.7085   2.7239   2.7315   2.7445   2.7466   2.7675   2.7741   2.7783
 2.7920   2.8029   2.8143   2.8381   2.8436   2.8607   2.8864   2.8952
 2.9170   2.9259   2.9523   2.9974   3.0633   3.0670   3.1334   3.1443
 3.1683   3.2014   3.2501   3.2559   3.2906   3.3364   3.3595   3.3979
 3.4170   3.4745   3.5112   3.5479   3.5613   3.6037   3.6280   3.6448
 3.6653   3.6910   3.7034   3.7182   3.7421   3.7570   3.7697   3.7810
 3.8325   3.8721   3.8815   3.9114   3.9356   3.9880   4.0322   4.0834
 4.1044   4.2030   4.2986   4.3095   4.6041   4.6597   4.6879   4.7386
 4.9028   5.0424   5.1887  23.5384  23.5654  23.7168  23.7272  23.7400
23.7671  23.7996  23.8286  23.8585  23.8937  23.9132  23.9699  23.9777
24.0401  35.4211  35.4480  35.5082  35.6082
--------------------------------------------------------------

         Ground-State Mulliken Net Atomic Charges

    Atom                 Charge (a.u.)
 ----------------------------------------
     1 N                    0.419328
     2 C                    0.145322
     3 C                   -0.034475
     4 C                   -0.141385
     5 C                   -0.180966
     6 C                   -0.138289
     7 C                   -0.758768
     8 C                   -0.262204
     9 N                   -0.152917
    10 C                   -0.259955
    11 N                    0.034995
    12 C                    0.170115
    13 C                   -0.142156
    14 C                    0.115692
    15 C                   -0.362318
    16 C                   -0.247228
    17 C                   -0.528929
    18 N                   -0.454667
    19 H                    0.219963
    20 H                    0.235320
    21 H                    0.227818
    22 H                    0.222271
    23 H                    0.232575
    24 H                    0.251316
    25 H                    0.182575
    26 H                    0.263006
    27 H                    0.245334
    28 H                    0.397365
    29 H                    0.219805
    30 H                    0.249142
    31 H                    0.211760
    32 H                    0.224015
    33 H                    0.201169
    34 H                    0.179745
    35 H                    0.217519
    36 H                    0.138293
    37 H                    0.276525
    38 H                    0.383288
 ----------------------------------------
 Sum of atomic charges =     2.000000

-----------------------------------------------------------------
                   Cartesian Multipole Moments
-----------------------------------------------------------------
   Charge (ESU x 10^10)
                9.6064
   Dipole Moment (Debye)
        X       4.9102       Y      -0.7681       Z      -4.8992
      Tot       6.9787
   Quadrupole Moments (Debye-Ang)
```

```
       XX      -39.5129      XY       27.7527      YY      -63.9703
       XZ      -37.6324      YZ      -19.8322      ZZ      -47.5323
   Traceless Quadrupole Moments (Debye-Ang)
      QXX       32.4769     QYY      -40.8956     QZZ        8.4187
      QXY       83.2581     QXZ     -112.8973     QYZ      -59.4966
   Octopole Moments (Debye-Ang^2)
      XXX       -4.8227     XXY       25.4194     XYY       12.5367
      YYY      -15.1232     XXZ       31.4236     XYZ      -20.3934
      YYZ       17.6734     XZZ       23.9893     YZZ       30.5662
      ZZZ      -68.0736
   Traceless Octopole Moments (Debye-Ang^2)
      XXX     -357.6698     YYY     -594.6101     ZZZ     -850.3144
      XXY      258.7038     XXZ      528.2841     XYY       92.9404
      XYZ     -305.9003     XZZ      264.7294     YYZ      322.0303
      YZZ      335.9063
   Hexadecapole Moments (Debye-Ang^3)
     XXXX   -2711.5975    XXXY     -262.8692    XXYY     -549.4781
     XYYY    -457.7383    YYYY     -856.8986    XXXZ      859.2936
     XXYZ     -33.4118    XYYZ      272.5354    YYYZ      396.3980
     XXZZ    -756.5213    XYZZ        8.7049    YYZZ     -558.3200
     XZZZ     737.6009    YZZZ      236.7169    ZZZZ    -1998.2424
   Traceless Hexadecapole Moments (Debye-Ang^3)
     XXXX   -6792.4150    XXXY     4434.3519    XXXZ     6101.4788
     XXYY    4153.0710    XXYZ   -12493.0368    XXZZ     2639.3440
     XYYY  -16026.9024    XYYZ      574.7708    XYZZ    11592.5505
     XZZZ   -6676.2496    YYYY     3189.9510    YYYZ    14667.4146
     YYZZ   -7343.0220    YZZZ    -2174.3778    ZZZZ     4703.6780
 -----------------------------------------------------------------

--------------------------------------------------
-  Entering drvman on Sun Oct 13 18:48:39 2024  -
--------------------------------------------------

 Calculating analytic gradient of the SCF energy
 Gradient of SCF Energy
           1           2           3           4           5           6
    1   0.0002953  -0.0009327   0.0002117   0.0000574  -0.0003126   0.0005731
    2   0.0005472   0.0002292  -0.0000521   0.0002563  -0.0007124  -0.0000660
    3   0.0006909  -0.0002180   0.0007394  -0.0000064   0.0006767  -0.0002598
           7           8           9          10          11          12
    1   0.0001411   0.0003297   0.0005172   0.0004711   0.0005536  -0.0013917
    2   0.0001371   0.0003156   0.0018605  -0.0003191  -0.0002797  -0.0031886
    3  -0.0001725  -0.0010953  -0.0006369  -0.0001440   0.0010375   0.0052428
          13          14          15          16          17          18
    1  -0.0032078   0.0009807   0.0005186  -0.0004208  -0.0003510   0.0056758
    2   0.0003609   0.0005504   0.0005337  -0.0000577  -0.0000864  -0.0000696
    3  -0.0008609   0.0001547   0.0002580  -0.0007361   0.0005117  -0.0098622
          19          20          21          22          23          24
    1  -0.0000863  -0.0000252   0.0000305   0.0001485  -0.0001226   0.0000373
    2  -0.0000854  -0.0000838   0.0001218  -0.0000223  -0.0001219   0.0000218
    3  -0.0000420   0.0000070   0.0001019  -0.0006264  -0.0000198   0.0000292
          25          26          27          28          29          30
    1  -0.0001011   0.0000987  -0.0004636  -0.0003812  -0.0000254  -0.0003560
    2   0.0000030  -0.0010133   0.0001071  -0.0000046  -0.0001532  -0.0004967
    3  -0.0002371   0.0002650  -0.0009147  -0.0000088   0.0003715  -0.0000535
          31          32          33          34          35          36
    1   0.0000805   0.0001021   0.0003757   0.0000729   0.0000220   0.0000996
    2  -0.0000609   0.0003349  -0.0001138   0.0000028   0.0000067  -0.0000495
    3  -0.0000263   0.0000618   0.0003223  -0.0000987  -0.0000797   0.0000150
          37          38
    1  -0.0036090   0.0003938
    2   0.0026091  -0.0009608
    3   0.0050509   0.0005629
 Max gradient component =       9.862E-03
 RMS gradient           =       1.467E-03
 Gradient time:  CPU 159.28 s  wall 265.54 s

--------------------------------------------------
-  Entering optman on Sun Oct 13 18:53:05 2024  -
--------------------------------------------------
```

```
 Geometry Optimization Parameters
   NAtoms,    NIC,     NZ,  NCons,   NDum,   NFix, NCnnct, MaxDiis
       38     272       0       0       0       0       0       0

 Cartesian Hessian Update
 Hessian updated using BFGS update


** GEOMETRY OPTIMIZATION IN DELOCALIZED INTERNAL COORDINATES **
   Searching for a Minimum

   Optimization Cycle:   4

                    Coordinates (Angstroms)
     ATOM              X               Y               Z
      1  N         2.0062401795    0.2557040402   -1.6078544297
      2  C         2.8616966642    1.3076775566   -1.7333382491
      3  C         2.9605717591    1.9258473163   -2.9725730424
      4  C         2.2245262066    1.4787482915   -4.0564593198
      5  C         1.3782327684    0.3830990901   -3.8995792080
      6  C         1.2951661299   -0.2052069328   -2.6669153547
      7  C         3.6975818038    1.7627890339   -0.5763892732
      8  C         1.8476154950   -0.4737474740   -0.3226274795
      9  N        -1.3612830894   -1.9075523362    0.9478600582
     10  C        -2.2076002441   -0.8466394949    0.9471642567
     11  N        -1.7742497952    0.3378262787    0.5907063511
     12  C        -0.4961545957    0.5113207271    0.2370127949
     13  C         0.4122413629   -0.5899512524    0.1373500252
     14  C        -0.0786585717   -1.7974644775    0.5334707703
     15  C        -3.6056935537   -1.0330063036    1.4174275637
     16  C        -3.7617043974   -0.5898742880    2.8914484914
     17  C        -2.9949447511   -1.4676668914    3.8748444995
     18  N        -0.1458551540    1.7646270019   -0.1221831346
     19  H         3.6372834464    2.7636709816   -3.0778200289
     20  H         2.3155806583    1.9698255833   -5.0182332947
     21  H         0.7971690920   -0.0120630262   -4.7218597384
     22  H         0.6624395397   -1.0629252903   -2.4812319402
     23  H         4.3059134499    2.6111475977   -0.8833270932
     24  H         4.3769663686    0.9750115824   -0.2393995633
     25  H         3.0998379586    2.0866145401    0.2804365808
     26  H         2.4528278333    0.0183113859    0.4346954555
     27  H         2.2707733232   -1.4697599501   -0.4655545741
     28  H        -1.7121505010   -2.8049316789    1.2642085761
     29  H         0.5064691674   -2.7100095846    0.5342883415
     30  H        -4.2515516896   -0.4297418278    0.7770792053
     31  H        -3.9032282810   -2.0804840900    1.3095017213
     32  H        -4.8293366186   -0.6193350742    3.1158442326
     33  H        -3.4514490880    0.4544936047    2.9832992049
     34  H        -3.2901595860   -2.5180369685    3.7881488993
     35  H        -3.1998557804   -1.1588615123    4.9003824567
     36  H        -1.9093813754   -1.3994611345    3.7366606603
     37  H         0.7564560816    2.1316109283    0.1387990597
     38  H        -0.8923322159    2.4383940477    0.0047165187
   Point Group: c1    Number of degrees of freedom:   108


   Energy is   -763.965057981

 Hessian updated using BFGS update
  internal optimization (0)

108 Hessian modes will be used to form the next step
  Hessian Eigenvalues:
     0.001839    0.002645    0.004013    0.004434    0.007550    0.010299
     0.013156    0.019043    0.019491    0.019990    0.021193    0.022024
     0.022632    0.023800    0.024051    0.024581    0.025279    0.026831
     0.027743    0.028167    0.028564    0.029714    0.035466    0.036933
     0.038718    0.040561    0.042792    0.043581    0.044519    0.045859
     0.047968    0.052732    0.053923    0.055804    0.066582    0.079794
```

```
    0.085585    0.095112    0.121735    0.122602    0.127149    0.130995
    0.131927    0.132814    0.137137    0.140378    0.143128    0.144646
    0.146886    0.147687    0.148623    0.152009    0.152776    0.153087
    0.154809    0.159964    0.192203    0.206232    0.209751    0.214425
    0.220152    0.229085    0.237443    0.248855    0.250919    0.253645
    0.267522    0.271962    0.282711    0.286664    0.298955    0.300135
    0.300610    0.301207    0.301815    0.303068    0.304015    0.304850
    0.305020    0.305233    0.306388    0.308593    0.312040    0.313921
    0.324512    0.331885    0.332858    0.335432    0.339146    0.340966
    0.348111    0.351899    0.360802    0.362978    0.377790    0.384768
    0.393269    0.402901    0.405152    0.416626    0.418939    0.433009
    0.433215    0.448971    0.457091    0.526654    0.637623    0.731024

 Minimum search - taking simple RFO step
 Searching for Lamda that Minimizes Along All modes
 Value Taken    Lamda =  -0.00500576
 Calculated Step too Large.  Step scaled by  0.473486
 Step Taken.  Stepsize is  0.300000

                            Maximum     Tolerance    Cnvgd?
        Gradient           0.003163      0.000800      NO
        Displacement       0.106692      0.001400      NO
        Energy change     -0.002838      0.000228      NO


 New Cartesian Coordinates Obtained by Inverse Iteration

 Displacement from previous Coordinates is:  0.499189
 ----------------------------------------------------------------
             Standard Nuclear Orientation (Angstroms)
    I     Atom           X                Y                Z
 ----------------------------------------------------------------
    1      N       2.0027677837     0.2717763369    -1.5966004137
    2      C       2.8811626339     1.3032251673    -1.7384737492
    3      C       2.9878646028     1.9018175261    -2.9866459608
    4      C       2.2390732249     1.4555691951    -4.0620242864
    5      C       1.3689628647     0.3811021596    -3.8875317711
    6      C       1.2769565488    -0.1877000309    -2.6460621873
    7      C       3.7302773452     1.7537159516    -0.5889988333
    8      C       1.8382718777    -0.4386299501    -0.2996720835
    9      N      -1.3714107831    -1.9067041148     0.9305627788
   10      C      -2.2237493333    -0.8479967556     0.9439958368
   11      N      -1.7960256477     0.3412611652     0.6018379954
   12      C      -0.5177511132     0.5309230832     0.2503806068
   13      C       0.3998050655    -0.5681247696     0.1411503302
   14      C      -0.0885386349    -1.7828513122     0.5181041964
   15      C      -3.6193006464    -1.0479526996     1.4148403064
   16      C      -3.7728928059    -0.6132602213     2.8928689319
   17      C      -2.9795687533    -1.4766963276     3.8673199156
   18      N      -0.1816391531     1.7851757465    -0.0817588145
   19      H       3.6822293546     2.7232548081    -3.1040480981
   20      H       2.3377847256     1.9319386155    -5.0302462916
   21      H       0.7760080060    -0.0134180805    -4.7015196383
   22      H       0.6255322513    -1.0268403418    -2.4415449852
   23      H       4.3574665424     2.5830397569    -0.9094570595
   24      H       4.3916745770     0.9544658187    -0.2434670328
   25      H       3.1451675172     2.1033067272     0.2668167947
   26      H       2.4259083525     0.0802775708     0.4537055100
   27      H       2.2788330135    -1.4305328227    -0.4152827941
   28      H      -1.7150697330    -2.8101597458     1.2352465796
   29      H       0.5028099088    -2.6911145821     0.5063513754
   30      H      -4.2699143172    -0.4438143909     0.7805427235
   31      H      -3.9086322601    -2.0970052299     1.3021686927
   32      H      -4.8382498836    -0.6657239179     3.1234008885
   33      H      -3.4848491511     0.4374836366     2.9822933068
   34      H      -3.2534950209    -2.5328949898     3.7816704648
   35      H      -3.1822804809    -1.1747601033     4.8953172171
   36      H      -1.8968812833    -1.3851236103     3.7210084627
   37      H       0.7567558947     2.1285269961     0.0425600972
   38      H      -0.9050630894     2.4744437355     0.0811909882
```

```
 --------------------------------------------------------------------
 Nuclear Repulsion Energy =        1314.87639347 hartrees
 There are       65 alpha and       65 beta electrons

--------------------------------------------------
-  Entering fldman on Sun Oct 13 18:53:05 2024  -
--------------------------------------------------

 Applying Cartesian multipole field
    Component          Value
    ---------          -----
      (2,0,0)        1.00000E-11
      (0,2,0)        2.00000E-11
      (0,0,2)       -3.00000E-11
 Nucleus-field energy     =    -0.0000000244 hartrees

--------------------------------------------------
-  Entering gesman on Sun Oct 13 18:53:05 2024  -
--------------------------------------------------

 Requested basis set is 6-311+G(d,p)
 There are 188 shells and 516 basis functions
 A cutoff of  1.0D-12 yielded  12704 shell pairs
 There are    101591 function pairs (    108363 Cartesian)
 Smallest overlap matrix eigenvalue = 1.81E-06
 Linear dependence detected in AO basis
 Tighter screening thresholds may be required for diffuse basis sets
 Use S2THRESH > 12 and THRESH = 14 in case of SCF convergence issues
 Number of orthogonalized atomic orbitals = 510
 Maximum deviation from orthogonality = 5.066E-12
 Guess MOs from SCF MO coefficient file
 Reading MOs from coefficient file
 Reading MOs from coefficient file

--------------------------------------------------
-  Entering scfman on Sun Oct 13 18:53:05 2024  -
--------------------------------------------------

 Long-range K will be added via erf
 Coulomb attenuation parameter = 0.2 bohr**(-1)
 A restricted hybrid HF-DFT SCF calculation will be
 performed using Pulay DIIS + Geometric Direct Minimization
 Exchange:     0.2220 Hartree-Fock + 1.0000 wB97X-D + LR-HF
 Correlation:  1.0000 wB97X-D
 Using Euler-Maclaurin-Lebedev (75,302) quadrature formula
 Dispersion:   Grimme D
 SCF converges when RMS gradient is below 1.0E-07
 Geometry optimization detected.  Setting ReadMinima to 0
 Setting SaveMinima to 0
 ---------------------------------------
  Cycle       Energy         DIIS Error
 ---------------------------------------
    1    -763.9826317608      4.77E-04
    2    -763.9665666148      9.96E-05
    3    -763.9667678902      9.87E-05
    4    -763.9670602457      1.72E-05
    5    -763.9670740064      8.02E-06
    6    -763.9670763590      3.43E-06
    7    -763.9670768461      9.45E-07
    8    -763.9670768931      3.92E-07
    9    -763.9670769016      1.15E-07
   10    -763.9670769025      4.68E-08 Convergence criterion met
 ---------------------------------------
 SCF time:  CPU 358.94 s  wall 590.32 s
 SCF   energy in the final basis set = -763.96707690
 Total energy in the final basis set = -763.96707690

--------------------------------------------------
-  Entering anlman on Sun Oct 13 19:02:56 2024  -
--------------------------------------------------
```

```
 ---------------------------------------------------------------
                   Orbital Energies (a.u.)
 ---------------------------------------------------------------

 Alpha MOs
 -- Occupied --
-14.8160 -14.7922 -14.7370 -14.7101 -10.6780 -10.6644 -10.6478 -10.6405
-10.6339 -10.6254 -10.6072 -10.5840 -10.5730 -10.5711 -10.5519 -10.5276
-10.4956 -10.4699  -1.4177  -1.4053  -1.3485  -1.2772  -1.2314  -1.2018
 -1.1885  -1.1427  -1.1007  -1.0848  -1.0558  -1.0546  -1.0322  -1.0072
 -0.9917  -0.9538  -0.9451  -0.9151  -0.9069  -0.8895  -0.8826  -0.8653
 -0.8562  -0.8426  -0.8409  -0.8211  -0.8086  -0.7954  -0.7912  -0.7824
 -0.7767  -0.7603  -0.7512  -0.7486  -0.7434  -0.7294  -0.7207  -0.7173
 -0.7013  -0.6708  -0.6572  -0.6446  -0.6368  -0.6288  -0.6243  -0.6224
 -0.6142
 -- Virtual --
 -0.2713  -0.2621  -0.2464  -0.2246  -0.1748  -0.1619  -0.1458  -0.1427
 -0.1357  -0.1236  -0.1174  -0.1138  -0.1096  -0.1018  -0.0988  -0.0949
 -0.0903  -0.0875  -0.0812  -0.0791  -0.0778  -0.0740  -0.0719  -0.0684
 -0.0643  -0.0586  -0.0572  -0.0521  -0.0489  -0.0452  -0.0405  -0.0325
 -0.0309  -0.0286  -0.0206  -0.0194  -0.0172  -0.0154  -0.0129  -0.0090
 -0.0052  -0.0040  -0.0006   0.0001   0.0083   0.0087   0.0105   0.0149
  0.0183   0.0203   0.0257   0.0272   0.0293   0.0338   0.0375   0.0399
  0.0433   0.0455   0.0484   0.0514   0.0571   0.0614   0.0632   0.0637
  0.0700   0.0739   0.0753   0.0789   0.0797   0.0810   0.0863   0.0918
  0.0932   0.0966   0.1007   0.1048   0.1078   0.1095   0.1158   0.1168
  0.1242   0.1256   0.1280   0.1306   0.1353   0.1368   0.1417   0.1500
  0.1513   0.1544   0.1594   0.1629   0.1681   0.1710   0.1733   0.1828
  0.1845   0.1879   0.1930   0.1998   0.2038   0.2068   0.2192   0.2229
  0.2303   0.2381   0.2552   0.2710   0.2838   0.2969   0.3023   0.3158
  0.3189   0.3291   0.3364   0.3479   0.3523   0.3583   0.3588   0.3657
  0.3708   0.3839   0.3861   0.3948   0.4031   0.4061   0.4097   0.4205
  0.4281   0.4320   0.4416   0.4488   0.4570   0.4634   0.4702   0.4773
  0.4846   0.4884   0.4937   0.5012   0.5055   0.5121   0.5178   0.5225
  0.5244   0.5290   0.5322   0.5369   0.5430   0.5468   0.5545   0.5575
  0.5649   0.5676   0.5708   0.5727   0.5852   0.5929   0.5970   0.6002
  0.6051   0.6184   0.6247   0.6281   0.6340   0.6373   0.6459   0.6576
  0.6642   0.6744   0.6781   0.6854   0.6969   0.7058   0.7084   0.7111
  0.7266   0.7334   0.7492   0.7500   0.7553   0.7718   0.7806   0.7822
  0.7981   0.8063   0.8135   0.8164   0.8300   0.8346   0.8444   0.8628
  0.8643   0.8720   0.8868   0.8933   0.9051   0.9122   0.9304   0.9403
  0.9468   0.9577   0.9669   0.9729   0.9883   1.0093   1.0227   1.0454
  1.0480   1.0672   1.0867   1.1000   1.1319   1.1491   1.1716   1.1851
  1.1931   1.1997   1.2316   1.2429   1.2575   1.2674   1.2819   1.3060
  1.3191   1.3248   1.3345   1.3397   1.3485   1.3620   1.3720   1.3810
  1.3921   1.3973   1.4003   1.4014   1.4094   1.4224   1.4317   1.4397
  1.4427   1.4470   1.4505   1.4552   1.4721   1.4856   1.4923   1.5005
  1.5050   1.5089   1.5110   1.5213   1.5255   1.5392   1.5482   1.5611
  1.5695   1.5712   1.5728   1.5811   1.5943   1.5984   1.6051   1.6111
  1.6232   1.6347   1.6432   1.6638   1.6732   1.6751   1.6762   1.6918
  1.7003   1.7055   1.7242   1.7276   1.7360   1.7431   1.7602   1.7812
  1.7871   1.7901   1.8116   1.8215   1.8275   1.8521   1.8551   1.8676
  1.8851   1.8959   1.9102   1.9249   1.9346   1.9469   1.9596   1.9886
  2.0001   2.0117   2.0325   2.0411   2.0581   2.0691   2.0847   2.0914
  2.1110   2.1256   2.1325   2.1347   2.1639   2.1710   2.2031   2.2267
  2.2311   2.2401   2.2472   2.2612   2.2802   2.2833   2.2893   2.3029
  2.3091   2.3131   2.3412   2.3633   2.3685   2.3769   2.3852   2.3970
  2.4099   2.4162   2.4261   2.4428   2.4527   2.4607   2.4699   2.4917
  2.5002   2.5200   2.5259   2.5313   2.5439   2.5445   2.5560   2.5648
  2.5734   2.5797   2.5869   2.5932   2.5965   2.6030   2.6116   2.6194
  2.6413   2.6537   2.6616   2.6705   2.6757   2.6809   2.6970   2.7006
  2.7131   2.7260   2.7334   2.7457   2.7485   2.7676   2.7745   2.7786
  2.7916   2.8008   2.8179   2.8399   2.8462   2.8635   2.8845   2.8973
  2.9130   2.9281   2.9514   2.9966   3.0617   3.0717   3.1312   3.1432
  3.1765   3.2269   3.2504   3.2549   3.2884   3.3367   3.3620   3.4016
  3.4176   3.4731   3.5165   3.5486   3.5592   3.6026   3.6239   3.6451
  3.6648   3.6920   3.7047   3.7223   3.7441   3.7557   3.7644   3.7792
```

```
  3.8350    3.8411    3.8718    3.9105    3.9343    3.9806    4.0308    4.0812
  4.1051    4.2041    4.2976    4.3090    4.6051    4.6631    4.6923    4.7343
  4.9118    5.0411    5.1921   23.5354   23.5685   23.7169   23.7280   23.7394
 23.7648   23.8001   23.8270   23.8594   23.8921   23.9144   23.9690   23.9775
 24.0386   35.4197   35.4504   35.5125   35.6127
--------------------------------------------------------------

         Ground-State Mulliken Net Atomic Charges

     Atom                 Charge (a.u.)
  ----------------------------------------
      1 N                    0.409135
      2 C                    0.143677
      3 C                   -0.026870
      4 C                   -0.144787
      5 C                   -0.176124
      6 C                   -0.135105
      7 C                   -0.764100
      8 C                   -0.269880
      9 N                   -0.152251
     10 C                   -0.262941
     11 N                    0.027720
     12 C                    0.192845
     13 C                   -0.157569
     14 C                    0.120144
     15 C                   -0.359374
     16 C                   -0.251039
     17 C                   -0.527867
     18 N                   -0.450401
     19 H                    0.220477
     20 H                    0.235784
     21 H                    0.228219
     22 H                    0.223942
     23 H                    0.232871
     24 H                    0.250940
     25 H                    0.185940
     26 H                    0.261778
     27 H                    0.246805
     28 H                    0.396244
     29 H                    0.218501
     30 H                    0.249007
     31 H                    0.211000
     32 H                    0.223802
     33 H                    0.201707
     34 H                    0.179288
     35 H                    0.217161
     36 H                    0.138947
     37 H                    0.275406
     38 H                    0.386967
  ----------------------------------------
  Sum of atomic charges =     2.000000

-----------------------------------------------------------------
                   Cartesian Multipole Moments
-----------------------------------------------------------------
    Charge (ESU x 10^10)
                 9.6064
    Dipole Moment (Debye)
         X       5.0343      Y      -0.5571      Z      -5.0887
       Tot       7.1798
    Quadrupole Moments (Debye-Ang)
        XX     -39.0060     XY      28.0035     YY     -64.1038
        XZ     -37.9145     YZ     -19.8528     ZZ     -47.7167
    Traceless Quadrupole Moments (Debye-Ang)
       QXX      33.8085    QYY     -41.4849    QZZ       7.6764
       QXY      84.0104    QXZ    -113.7436    QYZ     -59.5583
    Octopole Moments (Debye-Ang^2)
       XXX       0.2754    XXY      26.0655    XYY      11.6801
       YYY     -14.8507    XXZ      28.9877    XYZ     -21.3167
       YYZ      17.9340    XZZ      26.0148    YZZ      30.5423
```

```
      ZZZ     -69.9932
   Traceless Octopole Moments (Debye-Ang^2)
      XXX    -337.6021    YYY    -598.5742    ZZZ    -842.2550
      XXY     265.7116    XXZ     504.0306    XYY      61.2904
      XYZ    -319.7508    XZZ     276.3117    YYZ     338.2245
      YZZ     332.8626
   Hexadecapole Moments (Debye-Ang^3)
     XXXX   -2719.9329   XXXY    -258.4521   XXYY    -552.1978
     XYYY    -465.9094   YYYY    -853.0547   XXXZ     852.8220
     XXYZ     -37.5895   XYYZ     273.4026   YYYZ     400.0453
     XXZZ    -754.2796   XYZZ       9.2750   YYZZ    -560.8513
     XZZZ     735.9050   YZZZ     235.6161   ZZZZ   -1989.9015
   Traceless Hexadecapole Moments (Debye-Ang^3)
     XXXX   -6893.9511   XXXY    5041.4207   XXXZ    5750.4823
     XXYY    4014.3056   XXYZ  -12908.7371   XXZZ    2879.6455
     XYYY  -16741.5914   XYYZ     775.3282   XYZZ   11700.1707
     XZZZ   -6525.8105   YYYY    3700.6751   YYYZ   15119.2465
     YYZZ   -7714.9807   YZZZ   -2210.5093   ZZZZ    4835.3352
 -----------------------------------------------------------------

--------------------------------------------------
-  Entering drvman on Sun Oct 13 19:02:56 2024  -
--------------------------------------------------

 Calculating analytic gradient of the SCF energy
 Gradient of SCF Energy
            1           2           3           4           5           6
    1   0.0004003  -0.0006667  -0.0001265   0.0003085  -0.0004037   0.0003234
    2   0.0005708   0.0006324  -0.0002613   0.0004962  -0.0006292  -0.0001372
    3   0.0003206  -0.0002341   0.0002769   0.0000792   0.0004413  -0.0002895
            7           8           9          10          11          12
    1  -0.0000859   0.0004730   0.0002506  -0.0015037   0.0010743  -0.0013177
    2  -0.0000136  -0.0000147   0.0009768   0.0002282  -0.0010124   0.0012131
    3  -0.0001299  -0.0009891   0.0000515  -0.0002245   0.0002105   0.0046126
           13          14          15          16          17          18
    1  -0.0013061   0.0011011   0.0002231  -0.0002408  -0.0002361   0.0025961
    2  -0.0001780  -0.0004046   0.0002403  -0.0000823  -0.0000935  -0.0011690
    3  -0.0009703   0.0001270  -0.0004040   0.0002544   0.0002429  -0.0082714
           19          20          21          22          23          24
    1  -0.0001139  -0.0000086   0.0000330   0.0001979  -0.0002083   0.0000016
    2  -0.0002034  -0.0000722  -0.0000149  -0.0000232  -0.0001709   0.0000775
    3   0.0002002   0.0001493   0.0002307  -0.0000493  -0.0000369   0.0001193
           25          26          27          28          29          30
    1   0.0001236   0.0003967  -0.0006555  -0.0000189   0.0000186  -0.0000543
    2  -0.0000147  -0.0008407   0.0000315   0.0002065   0.0000014  -0.0003469
    3   0.0000719  -0.0000591  -0.0005472  -0.0000497   0.0000904  -0.0000079
           31          32          33          34          35          36
    1   0.0002014   0.0001084   0.0002276   0.0000257   0.0000873   0.0001108
    2  -0.0000480   0.0003725  -0.0001150   0.0000298  -0.0000467  -0.0000278
    3   0.0000750  -0.0001744   0.0000381   0.0000160  -0.0001012   0.0001246
           37          38
    1  -0.0020519   0.0007156
    2   0.0017916  -0.0009480
    3   0.0039809   0.0008252
 Max gradient component =       8.271E-03
 RMS gradient           =       1.121E-03
 Gradient time:  CPU 165.00 s  wall 276.92 s

--------------------------------------------------
-  Entering optman on Sun Oct 13 19:07:32 2024  -
--------------------------------------------------

 Geometry Optimization Parameters
   NAtoms,    NIC,     NZ,  NCons,   NDum,   NFix, NCnnct, MaxDiis
      38     272       0       0       0       0       0       0

 Cartesian Hessian Update
 Hessian updated using BFGS update
```

```
** GEOMETRY OPTIMIZATION IN DELOCALIZED INTERNAL COORDINATES **
   Searching for a Minimum

   Optimization Cycle:   5

                    Coordinates (Angstroms)
     ATOM                X               Y               Z
      1  N         2.0027677837    0.2717763369   -1.5966004137
      2  C         2.8811626339    1.3032251673   -1.7384737492
      3  C         2.9878646028    1.9018175261   -2.9866459608
      4  C         2.2390732249    1.4555691951   -4.0620242864
      5  C         1.3689628647    0.3811021596   -3.8875317711
      6  C         1.2769565488   -0.1877000309   -2.6460621873
      7  C         3.7302773452    1.7537159516   -0.5889988333
      8  C         1.8382718777   -0.4386299501   -0.2996720835
      9  N        -1.3714107831   -1.9067041148    0.9305627788
     10  C        -2.2237493333   -0.8479967556    0.9439958368
     11  N        -1.7960256477    0.3412611652    0.6018379954
     12  C        -0.5177511132    0.5309230832    0.2503806068
     13  C         0.3998050655   -0.5681247696    0.1411503302
     14  C        -0.0885386349   -1.7828513122    0.5181041964
     15  C        -3.6193006464   -1.0479526996    1.4148403064
     16  C        -3.7728928059   -0.6132602213    2.8928689319
     17  C        -2.9795687533   -1.4766963276    3.8673199156
     18  N        -0.1816391531    1.7851757465   -0.0817588145
     19  H         3.6822293546    2.7232548081   -3.1040480981
     20  H         2.3377847256    1.9319386155   -5.0302462916
     21  H         0.7760080060   -0.0134180805   -4.7015196383
     22  H         0.6255322513   -1.0268403418   -2.4415449852
     23  H         4.3574665424    2.5830397569   -0.9094570595
     24  H         4.3916745770    0.9544658187   -0.2434670328
     25  H         3.1451675172    2.1033067272    0.2668167947
     26  H         2.4259083525    0.0802775708    0.4537055100
     27  H         2.2788330135   -1.4305328227   -0.4152827941
     28  H        -1.7150697330   -2.8101597458    1.2352465796
     29  H         0.5028099088   -2.6911145821    0.5063513754
     30  H        -4.2699143172   -0.4438143909    0.7805427235
     31  H        -3.9086322601   -2.0970052299    1.3021686927
     32  H        -4.8382498836   -0.6657239179    3.1234008885
     33  H        -3.4848491511    0.4374836366    2.9822933068
     34  H        -3.2534950209   -2.5328949898    3.7816704648
     35  H        -3.1822804809   -1.1747601033    4.8953172171
     36  H        -1.8968812833   -1.3851236103    3.7210084627
     37  H         0.7567558947    2.1285269961    0.0425600972
     38  H        -0.9050630894    2.4744437355    0.0811909882
   Point Group: c1    Number of degrees of freedom:   108


   Energy is   -763.967076902

 Hessian updated using BFGS update
  internal optimization (0)

108 Hessian modes will be used to form the next step
  Hessian Eigenvalues:
     0.001833    0.002646    0.003992    0.004267    0.007615    0.011532
     0.013188    0.019050    0.019496    0.020063    0.021179    0.022035
     0.022632    0.023914    0.024054    0.024571    0.025283    0.026706
     0.027167    0.028164    0.028413    0.029449    0.035092    0.036645
     0.038688    0.040525    0.042806    0.043602    0.044478    0.045785
     0.047938    0.051882    0.053917    0.055803    0.066636    0.079981
     0.085590    0.095103    0.121747    0.122657    0.127153    0.130995
     0.131930    0.132818    0.137130    0.140499    0.143169    0.144454
     0.146887    0.147688    0.148603    0.152044    0.152818    0.153102
     0.154803    0.160634    0.192472    0.206292    0.209876    0.214400
     0.220193    0.229132    0.237430    0.248852    0.251849    0.253651
     0.267753    0.272348    0.282898    0.286644    0.298988    0.300139
     0.300612    0.301210    0.301828    0.303073    0.304019    0.304855
     0.305028    0.305230    0.306402    0.308714    0.312122    0.313928
     0.325439    0.331882    0.332907    0.335738    0.339151    0.341000
```

```
    0.348353    0.352264    0.361348    0.365533    0.379064    0.384868
    0.393229    0.402381    0.405198    0.416843    0.419778    0.433109
    0.441639    0.450697    0.457465    0.524661    0.637026    0.730991

 Minimum search - taking simple RFO step
 Searching for Lamda that Minimizes Along All modes
 Value Taken    Lamda =  -0.00322553
 Calculated Step too Large.  Step scaled by  0.521024
 Step Taken.  Stepsize is  0.300000

                             Maximum     Tolerance    Cnvgd?
         Gradient           0.002346      0.000800      NO
         Displacement       0.094181      0.001400      NO
         Energy change     -0.002019      0.000228      NO


 New Cartesian Coordinates Obtained by Inverse Iteration

 Displacement from previous Coordinates is:  0.583374
 ----------------------------------------------------------------
             Standard Nuclear Orientation (Angstroms)
    I     Atom           X                Y                Z
 ----------------------------------------------------------------
    1      N       1.9968944592     0.2923185596    -1.5867030667
    2      C       2.9046524847     1.2963486511    -1.7470521681
    3      C       3.0274271625     1.8685446391    -3.0066957571
    4      C       2.2675448448     1.4230736301    -4.0746151793
    5      C       1.3674344561     0.3772145827    -3.8804724692
    6      C       1.2584181677    -0.1658797443    -2.6279409798
    7      C       3.7643522152     1.7442345516    -0.6042664058
    8      C       1.8223594256    -0.3930216368    -0.2752904633
    9      N      -1.3853609271    -1.9039759252     0.9064596196
   10      C      -2.2415726242    -0.8472684970     0.9439228193
   11      N      -1.8215668368     0.3477368994     0.6202893173
   12      C      -0.5447597590     0.5511090156     0.2659068702
   13      C       0.3811114112    -0.5427162594     0.1422363127
   14      C      -0.1034332835    -1.7645570054     0.4954243465
   15      C      -3.6339389311    -1.0623099639     1.4176735101
   16      C      -3.7833881674    -0.6415933234     2.9004296449
   17      C      -2.9599222181    -1.4915424140     3.8612412626
   18      N      -0.2187083676     1.8092581630    -0.0296705266
   19      H       3.7441465009     2.6684663345    -3.1379983178
   20      H       2.3799971494     1.8787330169    -5.0512016058
   21      H       0.7636164112    -0.0160112837    -4.6871662200
   22      H       0.5830233853    -0.9813851571    -2.4068224421
   23      H       4.4189261373     2.5456584652    -0.9407165049
   24      H       4.3983499745     0.9317695469    -0.2389442135
   25      H       3.1881015973     2.1317187065     0.2413551890
   26      H       2.3824327249     0.1609552336     0.4744838406
   27      H       2.2885467992    -1.3766371896    -0.3561707123
   28      H      -1.7227434168    -2.8145756226     1.1952919479
   29      H       0.4941668121    -2.6683559589     0.4691184264
   30      H      -4.2912054817    -0.4558013281     0.7925560609
   31      H      -3.9158226828    -2.1123232522     1.2962974736
   32      H      -4.8451151791    -0.7215090654     3.1397713145
   33      H      -3.5203837129     0.4153776451     2.9924185552
   34      H      -3.2090143042    -2.5536189240     3.7721553675
   35      H      -3.1597846763    -1.2009692037     4.8930953785
   36      H      -1.8813818218    -1.3726161902     3.7049876806
   37      H       0.7326015639     2.1356521082    -0.0289255394
   38      H      -0.9260012923     2.5084981958     0.1555376341
 ----------------------------------------------------------------
 Nuclear Repulsion Energy =        1313.81742029 hartrees
 There are       65 alpha and      65 beta electrons

--------------------------------------------------
-  Entering fldman on Sun Oct 13 19:07:33 2024  -
--------------------------------------------------

 Applying Cartesian multipole field
```

```
   Component          Value
   ---------          -----
     (2,0,0)       1.00000E-11
     (0,2,0)       2.00000E-11
     (0,0,2)      -3.00000E-11
 Nucleus-field energy     =    -0.0000000243 hartrees

------------------------------------------------
-  Entering gesman on Sun Oct 13 19:07:33 2024  -
------------------------------------------------


 Requested basis set is 6-311+G(d,p)
 There are 188 shells and 516 basis functions
 A cutoff of  1.0D-12 yielded  12687 shell pairs
 There are    101480 function pairs (    108245 Cartesian)
 Smallest overlap matrix eigenvalue = 1.71E-06
 Linear dependence detected in AO basis
 Tighter screening thresholds may be required for diffuse basis sets
 Use S2THRESH > 12 and THRESH = 14 in case of SCF convergence issues
 Number of orthogonalized atomic orbitals = 510
 Maximum deviation from orthogonality = 1.517E-11
 Guess MOs from SCF MO coefficient file
 Reading MOs from coefficient file
 Reading MOs from coefficient file

------------------------------------------------
-  Entering scfman on Sun Oct 13 19:07:33 2024  -
------------------------------------------------


 Long-range K will be added via erf
 Coulomb attenuation parameter = 0.2 bohr**(-1)
 A restricted hybrid HF-DFT SCF calculation will be
 performed using Pulay DIIS + Geometric Direct Minimization
 Exchange:     0.2220 Hartree-Fock + 1.0000 wB97X-D + LR-HF
 Correlation:  1.0000 wB97X-D
 Using Euler-Maclaurin-Lebedev (75,302) quadrature formula
 Dispersion:   Grimme D
 SCF converges when RMS gradient is below 1.0E-07
 Geometry optimization detected.  Setting ReadMinima to 0
 Setting SaveMinima to 0
 ---------------------------------------
  Cycle       Energy         DIIS Error
 ---------------------------------------
    1    -763.9782532565      5.39E-04
    2    -763.9679829447      8.79E-05
    3    -763.9683028903      7.24E-05
    4    -763.9684646507      2.17E-05
    5    -763.9684848231      7.04E-06
    6    -763.9684868108      3.42E-06
    7    -763.9684872248      8.38E-07
    8    -763.9684872652      3.64E-07
    9    -763.9684872719      1.02E-07
   10    -763.9684872727      4.36E-08 Convergence criterion met
 ---------------------------------------
 SCF time:  CPU 364.31 s  wall 611.46 s
 SCF   energy in the final basis set = -763.96848727
 Total energy in the final basis set = -763.96848727

------------------------------------------------
-  Entering anlman on Sun Oct 13 19:17:44 2024  -
------------------------------------------------



 --------------------------------------------------------------

                   Orbital Energies (a.u.)
 --------------------------------------------------------------

 Alpha MOs
 -- Occupied --
```

```
-14.8169 -14.7901 -14.7396 -14.7084 -10.6780 -10.6631 -10.6467 -10.6413
-10.6342 -10.6262 -10.6063 -10.5847 -10.5739 -10.5720 -10.5519 -10.5266
-10.4948 -10.4693  -1.4165  -1.4056  -1.3495  -1.2773  -1.2316  -1.2018
 -1.1889  -1.1424  -1.1004  -1.0850  -1.0555  -1.0543  -1.0325  -1.0068
 -0.9920  -0.9549  -0.9444  -0.9166  -0.9080  -0.8894  -0.8828  -0.8663
 -0.8568  -0.8428  -0.8410  -0.8214  -0.8086  -0.7959  -0.7914  -0.7818
 -0.7763  -0.7608  -0.7513  -0.7484  -0.7445  -0.7300  -0.7206  -0.7173
 -0.7004  -0.6702  -0.6580  -0.6440  -0.6359  -0.6278  -0.6238  -0.6224
 -0.6133
 -- Virtual --
 -0.2719  -0.2598  -0.2449  -0.2256  -0.1748  -0.1619  -0.1459  -0.1426
 -0.1360  -0.1231  -0.1171  -0.1138  -0.1094  -0.1014  -0.0989  -0.0957
 -0.0902  -0.0873  -0.0813  -0.0795  -0.0779  -0.0739  -0.0719  -0.0685
 -0.0639  -0.0583  -0.0570  -0.0522  -0.0485  -0.0454  -0.0408  -0.0339
 -0.0317  -0.0280  -0.0205  -0.0199  -0.0169  -0.0156  -0.0130  -0.0087
 -0.0052  -0.0039  -0.0007   0.0001   0.0082   0.0084   0.0099   0.0151
  0.0182   0.0211   0.0249   0.0273   0.0291   0.0336   0.0379   0.0395
  0.0430   0.0461   0.0480   0.0515   0.0573   0.0613   0.0634   0.0647
  0.0694   0.0736   0.0760   0.0792   0.0800   0.0811   0.0862   0.0911
  0.0939   0.0968   0.1000   0.1053   0.1077   0.1096   0.1159   0.1169
  0.1240   0.1258   0.1274   0.1309   0.1339   0.1371   0.1412   0.1480
  0.1516   0.1545   0.1596   0.1640   0.1682   0.1714   0.1738   0.1813
  0.1844   0.1882   0.1935   0.2007   0.2044   0.2096   0.2191   0.2221
  0.2292   0.2366   0.2570   0.2718   0.2847   0.2974   0.3016   0.3138
  0.3187   0.3293   0.3367   0.3468   0.3516   0.3582   0.3587   0.3662
  0.3698   0.3848   0.3873   0.3945   0.4027   0.4068   0.4094   0.4221
  0.4285   0.4302   0.4439   0.4496   0.4563   0.4631   0.4732   0.4779
  0.4836   0.4887   0.4945   0.5031   0.5048   0.5124   0.5186   0.5217
  0.5247   0.5283   0.5317   0.5362   0.5427   0.5472   0.5559   0.5579
  0.5643   0.5680   0.5712   0.5733   0.5866   0.5946   0.5957   0.6049
  0.6068   0.6178   0.6252   0.6302   0.6381   0.6397   0.6484   0.6567
  0.6663   0.6749   0.6776   0.6876   0.6958   0.7073   0.7092   0.7138
  0.7215   0.7332   0.7491   0.7507   0.7617   0.7711   0.7794   0.7840
  0.7928   0.8069   0.8096   0.8189   0.8285   0.8382   0.8465   0.8602
  0.8650   0.8737   0.8858   0.8953   0.9025   0.9080   0.9297   0.9391
  0.9481   0.9602   0.9698   0.9729   0.9919   1.0075   1.0237   1.0457
  1.0519   1.0662   1.0778   1.0995   1.1290   1.1490   1.1736   1.1841
  1.1914   1.2047   1.2264   1.2383   1.2588   1.2673   1.2792   1.3069
  1.3181   1.3205   1.3338   1.3368   1.3454   1.3630   1.3722   1.3799
  1.3935   1.3938   1.3985   1.4042   1.4105   1.4159   1.4324   1.4404
  1.4445   1.4463   1.4513   1.4543   1.4736   1.4857   1.4939   1.4982
  1.5042   1.5078   1.5142   1.5229   1.5271   1.5387   1.5493   1.5615
  1.5683   1.5709   1.5737   1.5809   1.5886   1.5972   1.6046   1.6119
  1.6246   1.6331   1.6422   1.6633   1.6719   1.6752   1.6767   1.6915
  1.6997   1.7084   1.7204   1.7268   1.7357   1.7427   1.7607   1.7763
  1.7878   1.7931   1.8124   1.8181   1.8274   1.8503   1.8579   1.8666
  1.8850   1.8964   1.9108   1.9279   1.9371   1.9493   1.9695   1.9887
  2.0004   2.0118   2.0337   2.0422   2.0585   2.0735   2.0861   2.0976
  2.1098   2.1265   2.1333   2.1361   2.1667   2.1758   2.2056   2.2276
  2.2322   2.2393   2.2484   2.2617   2.2767   2.2836   2.2849   2.3011
  2.3046   2.3131   2.3371   2.3633   2.3685   2.3737   2.3867   2.3970
  2.4053   2.4142   2.4257   2.4433   2.4487   2.4615   2.4707   2.4937
  2.5005   2.5185   2.5258   2.5304   2.5442   2.5470   2.5578   2.5648
  2.5705   2.5795   2.5878   2.5923   2.5972   2.6024   2.6093   2.6182
  2.6412   2.6559   2.6650   2.6693   2.6753   2.6816   2.6962   2.7021
  2.7160   2.7302   2.7371   2.7447   2.7540   2.7681   2.7753   2.7827
  2.7911   2.7987   2.8207   2.8400   2.8487   2.8670   2.8848   2.8991
  2.9102   2.9336   2.9502   2.9978   3.0597   3.0790   3.1309   3.1420
  3.1791   3.2468   3.2529   3.2572   3.2862   3.3370   3.3663   3.4046
  3.4209   3.4738   3.5224   3.5490   3.5560   3.6022   3.6206   3.6459
  3.6646   3.6922   3.7064   3.7265   3.7451   3.7526   3.7616   3.7758
  3.8056   3.8377   3.8705   3.9102   3.9352   3.9787   4.0296   4.0806
  4.1067   4.2053   4.2969   4.3108   4.6059   4.6660   4.6949   4.7306
  4.9198   5.0395   5.1959  23.5315  23.5710  23.7182  23.7290  23.7398
 23.7659  23.8011  23.8259  23.8598  23.8901  23.9155  23.9675  23.9814
 24.0368  35.4183  35.4529  35.5177  35.6179
 --------------------------------------------------------------

          Ground-State Mulliken Net Atomic Charges
```

```
    Atom                 Charge (a.u.)
 ----------------------------------------
      1 N                     0.401243
      2 C                     0.137257
      3 C                    -0.016574
      4 C                    -0.148577
      5 C                    -0.171796
      6 C                    -0.133806
      7 C                    -0.768081
      8 C                    -0.276369
      9 N                    -0.149495
     10 C                    -0.268832
     11 N                     0.021782
     12 C                     0.219453
     13 C                    -0.173193
     14 C                     0.125412
     15 C                    -0.356109
     16 C                    -0.255589
     17 C                    -0.525580
     18 N                    -0.454721
     19 H                     0.220896
     20 H                     0.235970
     21 H                     0.228449
     22 H                     0.225735
     23 H                     0.233016
     24 H                     0.249266
     25 H                     0.189360
     26 H                     0.259491
     27 H                     0.248844
     28 H                     0.395410
     29 H                     0.217329
     30 H                     0.249138
     31 H                     0.210056
     32 H                     0.223596
     33 H                     0.202542
     34 H                     0.178647
     35 H                     0.216846
     36 H                     0.139560
     37 H                     0.281398
     38 H                     0.388024
 ----------------------------------------
 Sum of atomic charges =     2.000000

-----------------------------------------------------------------
                  Cartesian Multipole Moments
-----------------------------------------------------------------
   Charge (ESU x 10^10)
                 9.6064
   Dipole Moment (Debye)
        X       5.1025      Y      -0.3777      Z      -5.2708
      Tot       7.3457
   Quadrupole Moments (Debye-Ang)
       XX     -38.4213     XY      28.2523     YY     -64.3695
       XZ     -38.3148     YZ     -19.7557     ZZ     -47.7671
   Traceless Quadrupole Moments (Debye-Ang)
      QXX      35.2940    QYY     -42.5505    QZZ       7.2566
      QXY      84.7570    QXZ    -114.9444    QYZ     -59.2670
   Octopole Moments (Debye-Ang^2)
      XXX       5.5824    XXY      26.7007    XYY      10.2234
      YYY     -14.9737    XXZ      25.8396    XYZ     -22.1933
      YYZ      18.6331    XZZ      28.7893    YZZ      30.3451
      ZZZ     -72.3353
   Traceless Octopole Moments (Debye-Ang^2)
      XXX    -317.6206    YYY    -603.2540    ZZZ    -834.2663
      XXY     274.2934    XXZ     471.1819    XYY      19.5661
      XYZ    -332.8996    XZZ     298.0545    YYZ     363.0844
      YZZ     328.9606
   Hexadecapole Moments (Debye-Ang^3)
     XXXX   -2731.7011   XXXY    -253.3352   XXYY    -555.8965
     XYYY    -475.9813   YYYY    -848.4322   XXXZ     846.7094
```

```
      XXYZ     -43.4702   XYYZ     276.5985   YYYZ     405.2334
      XXZZ    -750.0382   XYZZ      10.2977   YYZZ    -565.9679
      XZZZ     733.0402   YZZZ     234.4320   ZZZZ   -1984.1355
    Traceless Hexadecapole Moments (Debye-Ang^3)
      XXXX   -7214.0575   XXXY    5755.6475   XXXZ    5368.8244
      XXYY    3825.6289   XXYZ  -13500.8145   XXZZ    3388.4286
      XYYY  -17622.1908   XYYZ    1197.6184   XYZZ   11866.5433
      XZZZ   -6566.4427   YYYY    4468.6488   YYYZ   15740.1602
      YYZZ   -8294.2776   YZZZ   -2239.3457   ZZZZ    4905.8490
 -----------------------------------------------------------------

--------------------------------------------------
-  Entering drvman on Sun Oct 13 19:17:44 2024  -
--------------------------------------------------

 Calculating analytic gradient of the SCF energy
 Gradient of SCF Energy
           1           2           3           4           5           6
    1   0.0003806  -0.0003444  -0.0003555   0.0003591  -0.0002215   0.0000347
    2   0.0003850   0.0006772  -0.0002776   0.0005446  -0.0001715  -0.0004556
    3   0.0000677   0.0000977  -0.0002761  -0.0001150  -0.0000478   0.0001293
           7           8           9          10          11          12
    1  -0.0001492   0.0003415   0.0001471  -0.0015782   0.0010155  -0.0008902
    2   0.0000179  -0.0003065   0.0003791   0.0013504  -0.0017978   0.0031295
    3  -0.0000506  -0.0006207   0.0004032  -0.0002663  -0.0001903   0.0030887
          13          14          15          16          17          18
    1  -0.0002839   0.0010119   0.0000569  -0.0000270  -0.0001256   0.0001503
    2  -0.0013409   0.0005958   0.0000522  -0.0000823  -0.0000840  -0.0022137
    3  -0.0004009  -0.0002755  -0.0007127   0.0007060   0.0000413  -0.0058262
          19          20          21          22          23          24
    1  -0.0001009   0.0000044   0.0000299   0.0001747  -0.0002087  -0.0000024
    2  -0.0001969  -0.0000573  -0.0000704  -0.0000293  -0.0001377   0.0000913
    3   0.0002591   0.0002107   0.0002198   0.0002699  -0.0000286   0.0001148
          25          26          27          28          29          30
    1   0.0001810   0.0005303  -0.0006704   0.0001517   0.0000342   0.0000562
    2  -0.0000408  -0.0006285  -0.0000456   0.0002734   0.0000934  -0.0001927
    3   0.0002417  -0.0001928  -0.0003389  -0.0000502  -0.0000810   0.0000223
          31          32          33          34          35          36
    1   0.0002328   0.0000797   0.0001044   0.0000050   0.0000901   0.0000823
    2  -0.0000428   0.0003267  -0.0000828   0.0000360  -0.0000445  -0.0000117
    3   0.0000858  -0.0002889  -0.0001218   0.0000889  -0.0000964   0.0001541
          37          38
    1  -0.0012188   0.0009227
    2   0.0010487  -0.0006906
    3   0.0028360   0.0009438
 Max gradient component =       5.826E-03
 RMS gradient           =       8.896E-04
 Gradient time:  CPU 158.63 s  wall 264.54 s

--------------------------------------------------
-  Entering optman on Sun Oct 13 19:22:09 2024  -
--------------------------------------------------

 Geometry Optimization Parameters
   NAtoms,    NIC,     NZ,  NCons,   NDum,   NFix, NCnnct, MaxDiis
       38     272       0       0       0       0       0       0

 Cartesian Hessian Update
 Hessian updated using BFGS update


** GEOMETRY OPTIMIZATION IN DELOCALIZED INTERNAL COORDINATES **
   Searching for a Minimum

   Optimization Cycle:   6

                      Coordinates (Angstroms)
     ATOM                X               Y                Z
      1  N         1.9968944592     0.2923185596    -1.5867030667
      2  C         2.9046524847     1.2963486511    -1.7470521681
```

```
     3  C          3.0274271625    1.8685446391   -3.0066957571
     4  C          2.2675448448    1.4230736301   -4.0746151793
     5  C          1.3674344561    0.3772145827   -3.8804724692
     6  C          1.2584181677   -0.1658797443   -2.6279409798
     7  C          3.7643522152    1.7442345516   -0.6042664058
     8  C          1.8223594256   -0.3930216368   -0.2752904633
     9  N         -1.3853609271   -1.9039759252    0.9064596196
    10  C         -2.2415726242   -0.8472684970    0.9439228193
    11  N         -1.8215668368    0.3477368994    0.6202893173
    12  C         -0.5447597590    0.5511090156    0.2659068702
    13  C          0.3811114112   -0.5427162594    0.1422363127
    14  C         -0.1034332835   -1.7645570054    0.4954243465
    15  C         -3.6339389311   -1.0623099639    1.4176735101
    16  C         -3.7833881674   -0.6415933234    2.9004296449
    17  C         -2.9599222181   -1.4915424140    3.8612412626
    18  N         -0.2187083676    1.8092581630   -0.0296705266
    19  H          3.7441465009    2.6684663345   -3.1379983178
    20  H          2.3799971494    1.8787330169   -5.0512016058
    21  H          0.7636164112   -0.0160112837   -4.6871662200
    22  H          0.5830233853   -0.9813851571   -2.4068224421
    23  H          4.4189261373    2.5456584652   -0.9407165049
    24  H          4.3983499745    0.9317695469   -0.2389442135
    25  H          3.1881015973    2.1317187065    0.2413551890
    26  H          2.3824327249    0.1609552336    0.4744838406
    27  H          2.2885467992   -1.3766371896   -0.3561707123
    28  H         -1.7227434168   -2.8145756226    1.1952919479
    29  H          0.4941668121   -2.6683559589    0.4691184264
    30  H         -4.2912054817   -0.4558013281    0.7925560609
    31  H         -3.9158226828   -2.1123232522    1.2962974736
    32  H         -4.8451151791   -0.7215090654    3.1397713145
    33  H         -3.5203837129    0.4153776451    2.9924185552
    34  H         -3.2090143042   -2.5536189240    3.7721553675
    35  H         -3.1597846763   -1.2009692037    4.8930953785
    36  H         -1.8813818218   -1.3726161902    3.7049876806
    37  H          0.7326015639    2.1356521082   -0.0289255394
    38  H         -0.9260012923    2.5084981958    0.1555376341
   Point Group: c1    Number of degrees of freedom:   108


   Energy is   -763.968487273

 Hessian updated using BFGS update
  internal optimization (0)

108 Hessian modes will be used to form the next step
  Hessian Eigenvalues:
     0.001813    0.002646    0.003848    0.004119    0.007341    0.012112
     0.013265    0.019051    0.019496    0.020177    0.021147    0.022082
     0.022623    0.023937    0.024071    0.024612    0.025315    0.026387
     0.027030    0.028186    0.028535    0.029360    0.034839    0.036532
     0.038672    0.040500    0.042801    0.043604    0.044442    0.045723
     0.047918    0.051288    0.053912    0.055805    0.066658    0.080057
     0.085591    0.095126    0.121749    0.122651    0.127154    0.131024
     0.131956    0.132825    0.137073    0.140781    0.143593    0.144631
     0.146863    0.147688    0.148587    0.152087    0.152832    0.153220
     0.155080    0.160642    0.192342    0.206292    0.209924    0.214419
     0.220883    0.229247    0.237395    0.248980    0.251585    0.253966
     0.267755    0.272348    0.282879    0.286623    0.298979    0.300140
     0.300613    0.301209    0.301826    0.303070    0.304020    0.304853
     0.305023    0.305233    0.306402    0.308756    0.312096    0.313943
     0.325602    0.332124    0.333006    0.335833    0.339157    0.341009
     0.350148    0.352387    0.361378    0.365980    0.379006    0.385292
     0.393693    0.401705    0.405688    0.416862    0.420256    0.433761
     0.442048    0.451980    0.462564    0.521881    0.636492    0.731221

 Minimum search - taking simple RFO step
 Searching for Lamda that Minimizes Along All modes
 Value Taken    Lamda =  -0.00207163
 Calculated Step too Large.  Step scaled by  0.558728
 Step Taken.  Stepsize is  0.300000
```

```
                              Maximum     Tolerance    Cnvgd?
          Gradient           0.002334      0.000800      NO
          Displacement       0.099098      0.001400      NO
          Energy change     -0.001410      0.000228      NO


 New Cartesian Coordinates Obtained by Inverse Iteration

 Displacement from previous Coordinates is:  0.665134
 ----------------------------------------------------------------
             Standard Nuclear Orientation (Angstroms)
    I     Atom           X                Y                Z
 ----------------------------------------------------------------
    1      N       1.9891634681     0.3180959822    -1.5779957254
    2      C       2.9321386250     1.2869502512    -1.7570164980
    3      C       3.0772461942     1.8285645530    -3.0274133383
    4      C       2.3056812535     1.3858288456    -4.0882853731
    5      C       1.3699924943     0.3756638109    -3.8756073163
    6      C       1.2389062240    -0.1366304499    -2.6124464425
    7      C       3.8018537141     1.7301701437    -0.6200758459
    8      C       1.8005493873    -0.3375789208    -0.2510761634
    9      N      -1.4015910777    -1.9001274082     0.8801949164
   10      C      -2.2618506431    -0.8468234415     0.9454256207
   11      N      -1.8513907416     0.3565478551     0.6414581855
   12      C      -0.5771400440     0.5737854341     0.2803984618
   13      C       0.3570186509    -0.5126262094     0.1413137490
   14      C      -0.1207157073    -1.7441426048     0.4717811912
   15      C      -3.6498117462    -1.0779977765     1.4260145587
   16      C      -3.7922884200    -0.6745054034     2.9138611201
   17      C      -2.9372503674    -1.5126888374     3.8571150929
   18      N      -0.2578524584     1.8368086024     0.0155438929
   19      H       3.8203047718     2.6016147761    -3.1731429814
   20      H       2.4359315135     1.8170392900    -5.0739175987
   21      H       0.7554341254    -0.0135873818    -4.6761692673
   22      H       0.5352633081    -0.9236744766    -2.3774455194
   23      H       4.4942992011     2.4914919803    -0.9738468532
   24      H       4.3960262085     0.9020967783    -0.2243563393
   25      H       3.2344110258     2.1689421900     0.2062292731
   26      H       2.3243825983     0.2563320334     0.4947877964
   27      H       2.2973864322    -1.3086861864    -0.2913800684
   28      H      -1.7326133845    -2.8180645938     1.1524138738
   29      H       0.4847540913    -2.6421988017     0.4324375787
   30      H      -4.3158170581    -0.4689722735     0.8125311758
   31      H      -3.9246779944    -2.1286052202     1.2943091678
   32      H      -4.8489046225    -0.7829425537     3.1648120284
   33      H      -3.5538863735     0.3876684656     3.0121836882
   34      H      -3.1609594098    -2.5798269164     3.7608297352
   35      H      -3.1323232909    -1.2366527686     4.8939325631
   36      H      -1.8640301935    -1.3654071040     3.6892074252
   37      H       0.6897138233     2.1530357809    -0.0957891479
   38      H      -0.9573535776     2.5411025560     0.2091833837
 ----------------------------------------------------------------
 Nuclear Repulsion Energy =        1312.38999375 hartrees
 There are       65 alpha and       65 beta electrons

--------------------------------------------------
-  Entering fldman on Sun Oct 13 19:22:09 2024  -
--------------------------------------------------

 Applying Cartesian multipole field
    Component           Value
    ---------           -----
     (2,0,0)       1.00000E-11
     (0,2,0)       2.00000E-11
     (0,0,2)      -3.00000E-11
 Nucleus-field energy     =    -0.0000000242 hartrees

--------------------------------------------------
-  Entering gesman on Sun Oct 13 19:22:09 2024  -
```

```
--------------------------------------------------

 Requested basis set is 6-311+G(d,p)
 There are 188 shells and 516 basis functions
 A cutoff of  1.0D-12 yielded  12689 shell pairs
 There are    101391 function pairs (    108149 Cartesian)
 Smallest overlap matrix eigenvalue = 1.65E-06
 Linear dependence detected in AO basis
 Tighter screening thresholds may be required for diffuse basis sets
 Use S2THRESH > 12 and THRESH = 14 in case of SCF convergence issues
 Number of orthogonalized atomic orbitals = 510
 Maximum deviation from orthogonality = 1.879E-11
 Guess MOs from SCF MO coefficient file
 Reading MOs from coefficient file
 Reading MOs from coefficient file

--------------------------------------------------
-  Entering scfman on Sun Oct 13 19:22:09 2024  -
--------------------------------------------------

 Long-range K will be added via erf
 Coulomb attenuation parameter = 0.2 bohr**(-1)
 A restricted hybrid HF-DFT SCF calculation will be
 performed using Pulay DIIS + Geometric Direct Minimization
 Exchange:     0.2220 Hartree-Fock + 1.0000 wB97X-D + LR-HF
 Correlation:  1.0000 wB97X-D
 Using Euler-Maclaurin-Lebedev (75,302) quadrature formula
 Dispersion:   Grimme D
 SCF converges when RMS gradient is below 1.0E-07
 Geometry optimization detected.  Setting ReadMinima to 0
 Setting SaveMinima to 0
 ---------------------------------------
  Cycle       Energy         DIIS Error
 ---------------------------------------
    1    -763.9685112871      5.80E-04
    2    -763.9689111380      8.26E-05
    3    -763.9693397920      4.88E-05
    4    -763.9694153697      2.38E-05
    5    -763.9694381464      6.37E-06
    6    -763.9694399156      2.92E-06
    7    -763.9694402027      7.81E-07
    8    -763.9694402381      3.17E-07
    9    -763.9694402426      9.25E-08 Convergence criterion met
 ---------------------------------------
 SCF time:  CPU 323.98 s  wall 542.29 s
 SCF   energy in the final basis set = -763.96944024
 Total energy in the final basis set = -763.96944024

--------------------------------------------------
-  Entering anlman on Sun Oct 13 19:31:12 2024  -
--------------------------------------------------



 --------------------------------------------------------------

                   Orbital Energies (a.u.)
 --------------------------------------------------------------

 Alpha MOs
 -- Occupied --
-14.8175 -14.7892 -14.7412 -14.7077 -10.6780 -10.6625 -10.6465 -10.6415
-10.6344 -10.6266 -10.6059 -10.5851 -10.5743 -10.5723 -10.5513 -10.5260
-10.4942 -10.4690  -1.4157  -1.4055  -1.3497  -1.2771  -1.2315  -1.2017
 -1.1890  -1.1422  -1.1002  -1.0849  -1.0554  -1.0541  -1.0322  -1.0065
 -0.9920  -0.9555  -0.9440  -0.9176  -0.9086  -0.8893  -0.8827  -0.8671
 -0.8573  -0.8427  -0.8409  -0.8215  -0.8085  -0.7962  -0.7912  -0.7810
 -0.7762  -0.7606  -0.7513  -0.7481  -0.7449  -0.7303  -0.7206  -0.7171
 -0.6998  -0.6697  -0.6585  -0.6436  -0.6353  -0.6272  -0.6236  -0.6223
 -0.6128
 -- Virtual --
```

| | | | | | | | |
|---|---|---|---|---|---|---|---|
| -0.2721 | -0.2586 | -0.2443 | -0.2261 | -0.1749 | -0.1620 | -0.1460 | -0.1427 |
| -0.1363 | -0.1225 | -0.1164 | -0.1138 | -0.1092 | -0.1010 | -0.0990 | -0.0963 |
| -0.0902 | -0.0872 | -0.0817 | -0.0788 | -0.0777 | -0.0737 | -0.0716 | -0.0686 |
| -0.0637 | -0.0578 | -0.0568 | -0.0522 | -0.0482 | -0.0458 | -0.0412 | -0.0362 |
| -0.0311 | -0.0275 | -0.0218 | -0.0198 | -0.0163 | -0.0158 | -0.0128 | -0.0082 |
| -0.0047 | -0.0040 | -0.0006 | 0.0001 | 0.0080 | 0.0082 | 0.0096 | 0.0153 |
| 0.0181 | 0.0213 | 0.0245 | 0.0266 | 0.0299 | 0.0339 | 0.0382 | 0.0389 |
| 0.0425 | 0.0465 | 0.0480 | 0.0519 | 0.0571 | 0.0612 | 0.0635 | 0.0651 |
| 0.0695 | 0.0734 | 0.0770 | 0.0793 | 0.0801 | 0.0809 | 0.0868 | 0.0904 |
| 0.0947 | 0.0974 | 0.0995 | 0.1055 | 0.1078 | 0.1096 | 0.1157 | 0.1167 |
| 0.1231 | 0.1253 | 0.1269 | 0.1308 | 0.1328 | 0.1370 | 0.1430 | 0.1459 |
| 0.1520 | 0.1546 | 0.1596 | 0.1645 | 0.1681 | 0.1718 | 0.1742 | 0.1811 |
| 0.1848 | 0.1887 | 0.1942 | 0.2009 | 0.2047 | 0.2115 | 0.2172 | 0.2230 |
| 0.2290 | 0.2366 | 0.2574 | 0.2733 | 0.2854 | 0.2978 | 0.2999 | 0.3124 |
| 0.3181 | 0.3291 | 0.3371 | 0.3452 | 0.3512 | 0.3570 | 0.3588 | 0.3672 |
| 0.3686 | 0.3853 | 0.3883 | 0.3941 | 0.4012 | 0.4086 | 0.4093 | 0.4240 |
| 0.4280 | 0.4303 | 0.4456 | 0.4498 | 0.4561 | 0.4622 | 0.4759 | 0.4793 |
| 0.4818 | 0.4893 | 0.4960 | 0.5033 | 0.5060 | 0.5120 | 0.5190 | 0.5204 |
| 0.5253 | 0.5281 | 0.5313 | 0.5349 | 0.5421 | 0.5484 | 0.5570 | 0.5588 |
| 0.5635 | 0.5682 | 0.5734 | 0.5737 | 0.5880 | 0.5948 | 0.5954 | 0.6064 |
| 0.6110 | 0.6174 | 0.6258 | 0.6306 | 0.6398 | 0.6454 | 0.6510 | 0.6564 |
| 0.6694 | 0.6746 | 0.6770 | 0.6890 | 0.6954 | 0.7061 | 0.7086 | 0.7182 |
| 0.7185 | 0.7319 | 0.7476 | 0.7505 | 0.7667 | 0.7677 | 0.7792 | 0.7864 |
| 0.7890 | 0.8046 | 0.8087 | 0.8207 | 0.8308 | 0.8406 | 0.8488 | 0.8589 |
| 0.8654 | 0.8754 | 0.8845 | 0.8939 | 0.9004 | 0.9089 | 0.9290 | 0.9379 |
| 0.9475 | 0.9615 | 0.9726 | 0.9765 | 0.9998 | 1.0052 | 1.0247 | 1.0460 |
| 1.0539 | 1.0626 | 1.0725 | 1.1011 | 1.1259 | 1.1467 | 1.1749 | 1.1836 |
| 1.1910 | 1.2047 | 1.2215 | 1.2328 | 1.2613 | 1.2686 | 1.2782 | 1.3073 |
| 1.3150 | 1.3190 | 1.3329 | 1.3342 | 1.3416 | 1.3640 | 1.3724 | 1.3768 |
| 1.3904 | 1.3938 | 1.3985 | 1.4062 | 1.4092 | 1.4140 | 1.4324 | 1.4407 |
| 1.4444 | 1.4482 | 1.4520 | 1.4540 | 1.4739 | 1.4839 | 1.4956 | 1.4960 |
| 1.5021 | 1.5063 | 1.5207 | 1.5241 | 1.5296 | 1.5396 | 1.5506 | 1.5596 |
| 1.5675 | 1.5700 | 1.5742 | 1.5811 | 1.5830 | 1.5970 | 1.6042 | 1.6121 |
| 1.6262 | 1.6316 | 1.6413 | 1.6621 | 1.6703 | 1.6747 | 1.6785 | 1.6903 |
| 1.6978 | 1.7062 | 1.7202 | 1.7275 | 1.7363 | 1.7421 | 1.7598 | 1.7740 |
| 1.7890 | 1.7950 | 1.8127 | 1.8148 | 1.8263 | 1.8488 | 1.8628 | 1.8650 |
| 1.8855 | 1.8978 | 1.9112 | 1.9346 | 1.9392 | 1.9516 | 1.9768 | 1.9897 |
| 2.0027 | 2.0119 | 2.0354 | 2.0406 | 2.0596 | 2.0763 | 2.0847 | 2.1005 |
| 2.1108 | 2.1253 | 2.1333 | 2.1404 | 2.1693 | 2.1810 | 2.2102 | 2.2283 |
| 2.2335 | 2.2385 | 2.2495 | 2.2622 | 2.2723 | 2.2808 | 2.2847 | 2.2953 |
| 2.2996 | 2.3131 | 2.3356 | 2.3624 | 2.3673 | 2.3702 | 2.3884 | 2.3956 |
| 2.4036 | 2.4127 | 2.4240 | 2.4422 | 2.4461 | 2.4621 | 2.4700 | 2.4939 |
| 2.5003 | 2.5176 | 2.5235 | 2.5311 | 2.5447 | 2.5480 | 2.5606 | 2.5646 |
| 2.5688 | 2.5794 | 2.5891 | 2.5918 | 2.5970 | 2.6048 | 2.6073 | 2.6181 |
| 2.6407 | 2.6569 | 2.6671 | 2.6696 | 2.6745 | 2.6792 | 2.6934 | 2.7057 |
| 2.7160 | 2.7314 | 2.7412 | 2.7452 | 2.7569 | 2.7714 | 2.7760 | 2.7880 |
| 2.7925 | 2.7977 | 2.8223 | 2.8398 | 2.8482 | 2.8701 | 2.8878 | 2.9004 |
| 2.9098 | 2.9406 | 2.9488 | 2.9998 | 3.0589 | 3.0872 | 3.1306 | 3.1408 |
| 3.1799 | 3.2482 | 3.2532 | 3.2700 | 3.2856 | 3.3375 | 3.3687 | 3.4061 |
| 3.4265 | 3.4742 | 3.5259 | 3.5484 | 3.5534 | 3.6019 | 3.6179 | 3.6468 |
| 3.6647 | 3.6918 | 3.7078 | 3.7302 | 3.7431 | 3.7480 | 3.7597 | 3.7698 |
| 3.7874 | 3.8391 | 3.8699 | 3.9096 | 3.9350 | 3.9780 | 4.0286 | 4.0805 |
| 4.1085 | 4.2062 | 4.2966 | 4.3122 | 4.6070 | 4.6670 | 4.6953 | 4.7273 |
| 4.9240 | 5.0386 | 5.1988 | 23.5277 | 23.5728 | 23.7183 | 23.7286 | 23.7402 |
| 23.7675 | 23.8014 | 23.8256 | 23.8596 | 23.8894 | 23.9162 | 23.9674 | 23.9799 |
| 24.0367 | 35.4175 | 35.4543 | 35.5208 | 35.6199 | | | |

---

Ground-State Mulliken Net Atomic Charges

| Atom | Charge (a.u.) |
|---|---|
| 1 N | 0.391651 |
| 2 C | 0.128747 |
| 3 C | -0.006637 |
| 4 C | -0.151390 |
| 5 C | -0.167267 |
| 6 C | -0.133130 |
| 7 C | -0.769936 |
| 8 C | -0.281608 |

```
     9  N                         -0.143865
    10  C                         -0.278246
    11  N                          0.017288
    12  C                          0.249321
    13  C                         -0.188240
    14  C                          0.130551
    15  C                         -0.352980
    16  C                         -0.260900
    17  C                         -0.522686
    18  N                         -0.466800
    19  H                          0.221214
    20  H                          0.235998
    21  H                          0.228441
    22  H                          0.227574
    23  H                          0.232960
    24  H                          0.245905
    25  H                          0.193957
    26  H                          0.256672
    27  H                          0.251260
    28  H                          0.394787
    29  H                          0.216758
    30  H                          0.249523
    31  H                          0.209177
    32  H                          0.223533
    33  H                          0.203654
    34  H                          0.177913
    35  H                          0.216667
    36  H                          0.140064
    37  H                          0.292467
    38  H                          0.387602
 ----------------------------------------
 Sum of atomic charges =      2.000000

 -------------------------------------------------------------------
                   Cartesian Multipole Moments
 -------------------------------------------------------------------
    Charge (ESU x 10^10)
                9.6064
    Dipole Moment (Debye)
         X       5.1112      Y      -0.2422      Z      -5.4340
       Tot       7.4640
    Quadrupole Moments (Debye-Ang)
        XX     -37.7028     XY      28.4897     YY     -64.7310
        XZ     -38.8055     YZ     -19.7023     ZZ     -47.7584
    Traceless Quadrupole Moments (Debye-Ang)
       QXX      37.0837    QYY     -44.0007    QZZ       6.9170
       QXY      85.4690    QXZ    -116.4166    QYZ     -59.1068
    Octopole Moments (Debye-Ang^2)
       XXX      11.1488    XXY      27.1618    XYY       8.2166
       YYY     -15.5953    XXZ      22.3158    XYZ     -22.8261
       YYZ      19.6195    XZZ      31.7731    YZZ      29.7343
       ZZZ     -74.3265
    Traceless Octopole Moments (Debye-Ang^2)
       XXX    -293.0146    YYY    -605.6364    ZZZ    -823.3767
       XXY     283.5241    XXZ     431.9112    XYY     -30.1660
       XYZ    -342.3914    XZZ     323.1806    YYZ     391.4655
       YZZ     322.1123
    Hexadecapole Moments (Debye-Ang^3)
      XXXX   -2744.7490   XXXY    -247.5764   XXYY    -560.8810
      XYYY    -488.0213   YYYY    -843.9197   XXXZ     839.3405
      XXYZ     -50.0908   XYYZ     281.6901   YYYZ     411.9136
      XXZZ    -744.0029   XYZZ      11.2659   YYZZ    -572.9315
      XZZZ     729.6617   YZZZ     234.8605   ZZZZ   -1981.2018
    Traceless Hexadecapole Moments (Debye-Ang^3)
      XXXX   -7661.1927   XXXY    6599.4100   XXXZ    4849.6003
      XXYY    3541.4641   XXYZ  -14196.8756   XXZZ    4119.7286
      XYYY  -18647.3057   XYYZ    1817.0712   XYZZ   12047.8957
      XZZZ   -6666.6715   YYYY    5454.8210   YYYZ   16438.9034
      YYZZ   -8996.2850   YZZZ   -2242.0278   ZZZZ    4876.5564
 -------------------------------------------------------------------
```

```
-----------------------------------------------
-  Entering drvman on Sun Oct 13 19:31:12 2024  -
-----------------------------------------------

 Calculating analytic gradient of the SCF energy
 Gradient of SCF Energy
           1           2           3           4           5           6
    1   0.0003124  -0.0002527  -0.0002119   0.0002474  -0.0003154   0.0001191
    2   0.0002574   0.0006405  -0.0001952   0.0002207  -0.0002587  -0.0001667
    3  -0.0000749  -0.0002834   0.0000279   0.0001262   0.0001187  -0.0001950
           7           8           9          10          11          12
    1  -0.0002005   0.0002813   0.0000269  -0.0015808   0.0005933  -0.0003952
    2   0.0001313  -0.0004474  -0.0002415   0.0009674  -0.0013532   0.0033989
    3   0.0000072  -0.0003253   0.0005863  -0.0002183  -0.0005604   0.0020489
          13          14          15          16          17          18
    1   0.0006211   0.0004547  -0.0000574   0.0000148  -0.0000343  -0.0005306
    2  -0.0007394  -0.0000460  -0.0000749  -0.0000860  -0.0000546  -0.0016256
    3  -0.0002001  -0.0001829  -0.0005384   0.0006690  -0.0000642  -0.0038837
          19          20          21          22          23          24
    1  -0.0000863   0.0000181   0.0000286   0.0001388  -0.0001305   0.0000532
    2  -0.0001345  -0.0000143  -0.0000782  -0.0000456  -0.0000628   0.0000622
    3   0.0002174   0.0001205   0.0001743   0.0002539   0.0000094   0.0000408
          25          26          27          28          29          30
    1   0.0001745   0.0004259  -0.0005826   0.0002149   0.0000446   0.0000843
    2  -0.0000400  -0.0004694  -0.0001014   0.0002170   0.0001214  -0.0000693
    3   0.0001967  -0.0001633  -0.0002476  -0.0000331  -0.0001312   0.0000396
          31          32          33          34          35          36
    1   0.0001976   0.0000326   0.0000395  -0.0000007   0.0000624   0.0000463
    2  -0.0000269   0.0002354  -0.0000451   0.0000393  -0.0000133  -0.0000015
    3   0.0000663  -0.0002299  -0.0001615   0.0000981  -0.0000606   0.0001180
          37          38
    1  -0.0007219   0.0008686
    2   0.0005228  -0.0004227
    3   0.0016747   0.0009597
 Max gradient component =       3.884E-03
 RMS gradient           =       6.680E-04
 Gradient time:  CPU 164.50 s  wall 272.00 s

-----------------------------------------------
-  Entering optman on Sun Oct 13 19:35:44 2024  -
-----------------------------------------------

 Geometry Optimization Parameters
   NAtoms,    NIC,     NZ,  NCons,   NDum,   NFix, NCnnct, MaxDiis
       38     272       0       0       0       0       0       0

 Cartesian Hessian Update
 Hessian updated using BFGS update


** GEOMETRY OPTIMIZATION IN DELOCALIZED INTERNAL COORDINATES **
   Searching for a Minimum

   Optimization Cycle:   7

                      Coordinates (Angstroms)
     ATOM              X              Y              Z
      1  N        1.9891634681    0.3180959822   -1.5779957254
      2  C        2.9321386250    1.2869502512   -1.7570164980
      3  C        3.0772461942    1.8285645530   -3.0274133383
      4  C        2.3056812535    1.3858288456   -4.0882853731
      5  C        1.3699924943    0.3756638109   -3.8756073163
      6  C        1.2389062240   -0.1366304499   -2.6124464425
      7  C        3.8018537141    1.7301701437   -0.6200758459
      8  C        1.8005493873   -0.3375789208   -0.2510761634
      9  N       -1.4015910777   -1.9001274082    0.8801949164
     10  C       -2.2618506431   -0.8468234415    0.9454256207
     11  N       -1.8513907416    0.3565478551    0.6414581855
     12  C       -0.5771400440    0.5737854341    0.2803984618
```

```
    13  C         0.3570186509   -0.5126262094    0.1413137490
    14  C        -0.1207157073   -1.7441426048    0.4717811912
    15  C        -3.6498117462   -1.0779977765    1.4260145587
    16  C        -3.7922884200   -0.6745054034    2.9138611201
    17  C        -2.9372503674   -1.5126888374    3.8571150929
    18  N        -0.2578524584    1.8368086024    0.0155438929
    19  H         3.8203047718    2.6016147761   -3.1731429814
    20  H         2.4359315135    1.8170392900   -5.0739175987
    21  H         0.7554341254   -0.0135873818   -4.6761692673
    22  H         0.5352633081   -0.9236744766   -2.3774455194
    23  H         4.4942992011    2.4914919803   -0.9738468532
    24  H         4.3960262085    0.9020967783   -0.2243563393
    25  H         3.2344110258    2.1689421900    0.2062292731
    26  H         2.3243825983    0.2563320334    0.4947877964
    27  H         2.2973864322   -1.3086861864   -0.2913800684
    28  H        -1.7326133845   -2.8180645938    1.1524138738
    29  H         0.4847540913   -2.6421988017    0.4324375787
    30  H        -4.3158170581   -0.4689722735    0.8125311758
    31  H        -3.9246779944   -2.1286052202    1.2943091678
    32  H        -4.8489046225   -0.7829425537    3.1648120284
    33  H        -3.5538863735    0.3876684656    3.0121836882
    34  H        -3.1609594098   -2.5798269164    3.7608297352
    35  H        -3.1323232909   -1.2366527686    4.8939325631
    36  H        -1.8640301935   -1.3654071040    3.6892074252
    37  H         0.6897138233    2.1530357809   -0.0957891479
    38  H        -0.9573535776    2.5411025560    0.2091833837
  Point Group: c1    Number of degrees of freedom:   108


  Energy is   -763.969440243

 Hessian updated using BFGS update
  internal optimization (0)

108 Hessian modes will be used to form the next step
  Hessian Eigenvalues:
     0.001782    0.002646    0.003492    0.004098    0.007023    0.012619
     0.013612    0.019080    0.019510    0.020340    0.021139    0.022142
     0.022614    0.023938    0.024084    0.024621    0.025352    0.025945
     0.026976    0.028201    0.028684    0.029341    0.034661    0.036457
     0.038660    0.040480    0.042795    0.043608    0.044403    0.045660
     0.047890    0.050742    0.053909    0.055804    0.066680    0.080019
     0.085591    0.095127    0.121753    0.122639    0.127153    0.131024
     0.131978    0.132827    0.136952    0.140755    0.143911    0.144666
     0.146833    0.147690    0.148589    0.152138    0.152898    0.153307
     0.155314    0.160456    0.192148    0.206291    0.209953    0.214436
     0.220596    0.229216    0.237529    0.248943    0.251302    0.253945
     0.267748    0.272368    0.282820    0.286785    0.298968    0.300141
     0.300613    0.301209    0.301823    0.303076    0.304020    0.304857
     0.305020    0.305238    0.306405    0.308739    0.312056    0.313951
     0.325445    0.332143    0.333010    0.335746    0.339166    0.341024
     0.350100    0.352434    0.361495    0.365131    0.379159    0.385404
     0.393709    0.401972    0.406175    0.416866    0.420276    0.434001
     0.442398    0.450231    0.460558    0.519633    0.636427    0.731810

 Minimum search - taking simple RFO step
 Searching for Lamda that Minimizes Along All modes
 Value Taken    Lamda =  -0.00129173
 Calculated Step too Large.  Step scaled by  0.603009
 Step Taken.  Stepsize is  0.300000

                             Maximum     Tolerance    Cnvgd?
         Gradient           0.001681      0.000800      NO
         Displacement       0.113795      0.001400      NO
         Energy change     -0.000953      0.000228      NO


 New Cartesian Coordinates Obtained by Inverse Iteration

 Displacement from previous Coordinates is:  0.729278
```

```
 --------------------------------------------------------------
              Standard Nuclear Orientation (Angstroms)
    I     Atom           X                Y                Z
 --------------------------------------------------------------
    1      N       1.9800518839     0.3476721314    -1.5705205756
    2      C       2.9626836115     1.2738442803    -1.7665674065
    3      C       3.1339820982     1.7824141090    -3.0480847379
    4      C       2.3504427953     1.3479325466    -4.1034882636
    5      C       1.3751305469     0.3803309914    -3.8731681880
    6      C       1.2174390332    -0.0995373770    -2.5993206558
    7      C       3.8415103243     1.7091190630    -0.6339351163
    8      C       1.7741753612    -0.2747973790    -0.2283596493
    9      N      -1.4163395184    -1.8929443369     0.8556537407
   10      C      -2.2816159341    -0.8453243696     0.9494973870
   11      N      -1.8836840932     0.3656283290     0.6625160205
   12      C      -0.6145750798     0.5958473365     0.2893541143
   13      C       0.3296021509    -0.4802095717     0.1373637409
   14      C      -0.1371605435    -1.7198439389     0.4493608074
   15      C      -3.6639706693    -1.0941782093     1.4384484030
   16      C      -3.7974461084    -0.7096254966     2.9316479098
   17      C      -2.9134531460    -1.5393844959     3.8554387733
   18      N      -0.3029090128     1.8630252183     0.0490216687
   19      H       3.9070442200     2.5231339037    -3.2061898267
   20      H       2.5011997174     1.7526218269    -5.0973715679
   21      H       0.7487615444    -0.0013885997    -4.6683735093
   22      H       0.4815508726    -0.8524725090    -2.3526520863
   23      H       4.5793649178     2.4179018021    -1.0048073988
   24      H       4.3837198157     0.8655917291    -0.1987015825
   25      H       3.2855978406     2.2095171564     0.1649359844
   26      H       2.2550277580     0.3623668351     0.5113325984
   27      H       2.3049613475    -1.2286842185    -0.2228303233
   28      H      -1.7399681550    -2.8177840805     1.1130344826
   29      H       0.4773791125    -2.6112296292     0.4001705443
   30      H      -4.3401791439    -0.4833699497     0.8378607203
   31      H      -3.9314618151    -2.1452965375     1.2958713070
   32      H      -4.8483158747    -0.8445157219     3.1939988828
   33      H      -3.5809193464     0.3562904072     3.0390629983
   34      H      -3.1142242702    -2.6101754094     3.7488703052
   35      H      -3.1027286912    -1.2798972450     4.8976127318
   36      H      -1.8459403861    -1.3658742698     3.6766260995
   37      H       0.6292101197     2.1759350087    -0.1591385602
   38      H      -1.0039432831     2.5673606703     0.2358302280
 --------------------------------------------------------------
 Nuclear Repulsion Energy =        1311.01746038 hartrees
 There are       65 alpha and       65 beta electrons

------------------------------------------------
-  Entering fldman on Sun Oct 13 19:35:44 2024  -
------------------------------------------------


 Applying Cartesian multipole field
    Component          Value
    ---------          -----
      (2,0,0)        1.00000E-11
      (0,2,0)        2.00000E-11
      (0,0,2)       -3.00000E-11
 Nucleus-field energy     =    -0.0000000243 hartrees

------------------------------------------------
-  Entering gesman on Sun Oct 13 19:35:44 2024  -
------------------------------------------------


 Requested basis set is 6-311+G(d,p)
 There are 188 shells and 516 basis functions
 A cutoff of  1.0D-12 yielded  12666 shell pairs
 There are    101183 function pairs (    107926 Cartesian)
 Smallest overlap matrix eigenvalue = 1.63E-06
 Linear dependence detected in AO basis
 Tighter screening thresholds may be required for diffuse basis sets
 Use S2THRESH > 12 and THRESH = 14 in case of SCF convergence issues
```

```
 Number of orthogonalized atomic orbitals = 510
 Maximum deviation from orthogonality = 1.322E-11
 Guess MOs from SCF MO coefficient file
 Reading MOs from coefficient file
 Reading MOs from coefficient file

--------------------------------------------------
-  Entering scfman on Sun Oct 13 19:35:44 2024  -
--------------------------------------------------

 Long-range K will be added via erf
 Coulomb attenuation parameter = 0.2 bohr**(-1)
 A restricted hybrid HF-DFT SCF calculation will be
 performed using Pulay DIIS + Geometric Direct Minimization
 Exchange:     0.2220 Hartree-Fock + 1.0000 wB97X-D + LR-HF
 Correlation:  1.0000 wB97X-D
 Using Euler-Maclaurin-Lebedev (75,302) quadrature formula
 Dispersion:   Grimme D
 SCF converges when RMS gradient is below 1.0E-07
 Geometry optimization detected.  Setting ReadMinima to 0
 Setting SaveMinima to 0
 ---------------------------------------
  Cycle       Energy         DIIS Error
 ---------------------------------------
    1    -763.9652661768      6.16E-04
    2    -763.9694646359      8.54E-05
    3    -763.9699665439      4.55E-05
    4    -763.9700353022      2.25E-05
    5    -763.9700558452      6.20E-06
    6    -763.9700575460      2.10E-06
    7    -763.9700577147      8.09E-07
    8    -763.9700577477      2.84E-07
    9    -763.9700577513      7.71E-08 Convergence criterion met
 ---------------------------------------
 SCF time:  CPU 323.67 s  wall 535.04 s
 SCF   energy in the final basis set = -763.97005775
 Total energy in the final basis set = -763.97005775

--------------------------------------------------
-  Entering anlman on Sun Oct 13 19:44:39 2024  -
--------------------------------------------------


 --------------------------------------------------------------

                   Orbital Energies (a.u.)
 --------------------------------------------------------------

 Alpha MOs
 -- Occupied --
-14.8178 -14.7887 -14.7421 -14.7072 -10.6778 -10.6621 -10.6465 -10.6415
-10.6345 -10.6269 -10.6055 -10.5851 -10.5745 -10.5724 -10.5504 -10.5256
-10.4937 -10.4688  -1.4154  -1.4054  -1.3500  -1.2773  -1.2314  -1.2017
 -1.1887  -1.1422  -1.1002  -1.0844  -1.0555  -1.0541  -1.0317  -1.0064
 -0.9918  -0.9562  -0.9437  -0.9182  -0.9091  -0.8891  -0.8825  -0.8676
 -0.8578  -0.8426  -0.8410  -0.8215  -0.8085  -0.7966  -0.7907  -0.7800
 -0.7764  -0.7601  -0.7511  -0.7479  -0.7448  -0.7303  -0.7205  -0.7169
 -0.6994  -0.6695  -0.6587  -0.6435  -0.6350  -0.6268  -0.6233  -0.6221
 -0.6127
 -- Virtual --
 -0.2720  -0.2578  -0.2439  -0.2265  -0.1753  -0.1621  -0.1464  -0.1428
 -0.1365  -0.1220  -0.1157  -0.1137  -0.1091  -0.1007  -0.0990  -0.0966
 -0.0902  -0.0872  -0.0821  -0.0779  -0.0773  -0.0738  -0.0711  -0.0686
 -0.0636  -0.0572  -0.0566  -0.0525  -0.0481  -0.0460  -0.0415  -0.0377
 -0.0306  -0.0275  -0.0232  -0.0201  -0.0161  -0.0154  -0.0127  -0.0074
 -0.0044  -0.0038  -0.0003   0.0004   0.0077   0.0082   0.0095   0.0157
  0.0181   0.0205   0.0249   0.0262   0.0313   0.0347   0.0378   0.0384
  0.0420   0.0466   0.0487   0.0526   0.0567   0.0614   0.0637   0.0648
  0.0705   0.0731   0.0782   0.0793   0.0801   0.0808   0.0882   0.0899
  0.0957   0.0981   0.0993   0.1053   0.1082   0.1094   0.1155   0.1162
```

| | | | | | | | |
|---|---|---|---|---|---|---|---|
| 0.1213 | 0.1246 | 0.1270 | 0.1299 | 0.1328 | 0.1371 | 0.1442 | 0.1459 |
| 0.1521 | 0.1545 | 0.1594 | 0.1648 | 0.1678 | 0.1721 | 0.1739 | 0.1822 |
| 0.1861 | 0.1894 | 0.1951 | 0.2005 | 0.2051 | 0.2124 | 0.2158 | 0.2228 |
| 0.2307 | 0.2385 | 0.2570 | 0.2749 | 0.2858 | 0.2974 | 0.2982 | 0.3112 |
| 0.3174 | 0.3282 | 0.3367 | 0.3437 | 0.3510 | 0.3557 | 0.3592 | 0.3673 |
| 0.3694 | 0.3857 | 0.3890 | 0.3934 | 0.3992 | 0.4086 | 0.4125 | 0.4264 |
| 0.4268 | 0.4323 | 0.4457 | 0.4502 | 0.4557 | 0.4612 | 0.4769 | 0.4797 |
| 0.4828 | 0.4901 | 0.4977 | 0.5028 | 0.5079 | 0.5107 | 0.5188 | 0.5193 |
| 0.5256 | 0.5275 | 0.5321 | 0.5350 | 0.5415 | 0.5496 | 0.5572 | 0.5592 |
| 0.5632 | 0.5689 | 0.5740 | 0.5759 | 0.5897 | 0.5947 | 0.5956 | 0.6065 |
| 0.6126 | 0.6173 | 0.6269 | 0.6302 | 0.6409 | 0.6497 | 0.6545 | 0.6572 |
| 0.6707 | 0.6751 | 0.6766 | 0.6887 | 0.6953 | 0.7027 | 0.7101 | 0.7173 |
| 0.7215 | 0.7297 | 0.7448 | 0.7499 | 0.7638 | 0.7697 | 0.7787 | 0.7836 |
| 0.7910 | 0.8016 | 0.8105 | 0.8199 | 0.8332 | 0.8399 | 0.8538 | 0.8605 |
| 0.8656 | 0.8754 | 0.8802 | 0.8938 | 0.9042 | 0.9108 | 0.9293 | 0.9363 |
| 0.9481 | 0.9629 | 0.9719 | 0.9834 | 1.0035 | 1.0099 | 1.0257 | 1.0392 |
| 1.0468 | 1.0709 | 1.0721 | 1.1052 | 1.1242 | 1.1418 | 1.1738 | 1.1830 |
| 1.1916 | 1.1951 | 1.2212 | 1.2292 | 1.2631 | 1.2715 | 1.2812 | 1.3071 |
| 1.3113 | 1.3206 | 1.3300 | 1.3321 | 1.3393 | 1.3648 | 1.3720 | 1.3734 |
| 1.3896 | 1.3940 | 1.3983 | 1.4026 | 1.4091 | 1.4157 | 1.4326 | 1.4406 |
| 1.4436 | 1.4491 | 1.4536 | 1.4545 | 1.4731 | 1.4817 | 1.4941 | 1.4980 |
| 1.5001 | 1.5063 | 1.5243 | 1.5277 | 1.5329 | 1.5414 | 1.5526 | 1.5548 |
| 1.5669 | 1.5703 | 1.5734 | 1.5769 | 1.5853 | 1.5971 | 1.6042 | 1.6124 |
| 1.6283 | 1.6311 | 1.6405 | 1.6605 | 1.6692 | 1.6736 | 1.6801 | 1.6875 |
| 1.6964 | 1.7044 | 1.7223 | 1.7281 | 1.7375 | 1.7423 | 1.7580 | 1.7756 |
| 1.7909 | 1.7962 | 1.8112 | 1.8150 | 1.8251 | 1.8483 | 1.8638 | 1.8668 |
| 1.8859 | 1.8999 | 1.9117 | 1.9369 | 1.9462 | 1.9554 | 1.9807 | 1.9884 |
| 2.0088 | 2.0149 | 2.0344 | 2.0399 | 2.0625 | 2.0774 | 2.0801 | 2.0993 |
| 2.1122 | 2.1241 | 2.1325 | 2.1444 | 2.1713 | 2.1856 | 2.2179 | 2.2285 |
| 2.2360 | 2.2377 | 2.2507 | 2.2619 | 2.2680 | 2.2765 | 2.2848 | 2.2891 |
| 2.2949 | 2.3134 | 2.3369 | 2.3596 | 2.3658 | 2.3670 | 2.3901 | 2.3962 |
| 2.4057 | 2.4126 | 2.4223 | 2.4400 | 2.4465 | 2.4617 | 2.4687 | 2.4932 |
| 2.5004 | 2.5163 | 2.5233 | 2.5320 | 2.5451 | 2.5474 | 2.5617 | 2.5668 |
| 2.5680 | 2.5795 | 2.5895 | 2.5923 | 2.5969 | 2.6032 | 2.6117 | 2.6186 |
| 2.6401 | 2.6558 | 2.6667 | 2.6733 | 2.6740 | 2.6770 | 2.6921 | 2.7078 |
| 2.7147 | 2.7319 | 2.7423 | 2.7475 | 2.7569 | 2.7750 | 2.7795 | 2.7900 |
| 2.7927 | 2.8007 | 2.8234 | 2.8386 | 2.8470 | 2.8730 | 2.8907 | 2.9005 |
| 2.9147 | 2.9448 | 2.9514 | 3.0028 | 3.0584 | 3.0958 | 3.1309 | 3.1399 |
| 3.1809 | 3.2474 | 3.2533 | 3.2784 | 3.2865 | 3.3359 | 3.3712 | 3.4062 |
| 3.4319 | 3.4763 | 3.5285 | 3.5471 | 3.5520 | 3.6012 | 3.6153 | 3.6472 |
| 3.6654 | 3.6912 | 3.7086 | 3.7331 | 3.7345 | 3.7467 | 3.7578 | 3.7680 |
| 3.7820 | 3.8399 | 3.8693 | 3.9097 | 3.9351 | 3.9787 | 4.0281 | 4.0807 |
| 4.1104 | 4.2069 | 4.2960 | 4.3147 | 4.6079 | 4.6676 | 4.6954 | 4.7254 |
| 4.9262 | 5.0376 | 5.2018 | 23.5250 | 23.5746 | 23.7189 | 23.7288 | 23.7414 |
| 23.7709 | 23.8019 | 23.8256 | 23.8595 | 23.8880 | 23.9167 | 23.9658 | 23.9822 |
| 24.0354 | 35.4170 | 35.4554 | 35.5239 | 35.6226 | | | |

Ground-State Mulliken Net Atomic Charges

| Atom | Charge (a.u.) |
|---|---|
| 1 N | 0.378965 |
| 2 C | 0.120478 |
| 3 C | 0.000950 |
| 4 C | -0.152483 |
| 5 C | -0.163252 |
| 6 C | -0.131613 |
| 7 C | -0.769708 |
| 8 C | -0.285638 |
| 9 N | -0.139349 |
| 10 C | -0.288710 |
| 11 N | 0.013770 |
| 12 C | 0.276661 |
| 13 C | -0.201772 |
| 14 C | 0.137725 |
| 15 C | -0.349955 |
| 16 C | -0.265649 |
| 17 C | -0.520048 |
| 18 N | -0.481797 |

```
    19 H                      0.221403
    20 H                      0.235884
    21 H                      0.228390
    22 H                      0.229810
    23 H                      0.232751
    24 H                      0.241110
    25 H                      0.200135
    26 H                      0.253659
    27 H                      0.253988
    28 H                      0.394344
    29 H                      0.216433
    30 H                      0.249931
    31 H                      0.208605
    32 H                      0.223498
    33 H                      0.204747
    34 H                      0.177277
    35 H                      0.216600
    36 H                      0.140406
    37 H                      0.306360
    38 H                      0.386095
  ----------------------------------------
  Sum of atomic charges =     2.000000

 -----------------------------------------------------------------
                    Cartesian Multipole Moments
 -----------------------------------------------------------------
    Charge (ESU x 10^10)
                 9.6064
    Dipole Moment (Debye)
         X       5.0808      Y      -0.1222      Z      -5.5820
       Tot       7.5491
    Quadrupole Moments (Debye-Ang)
        XX     -36.8958     XY      28.6209     YY     -65.2379
        XZ     -39.3013     YZ     -19.7574     ZZ     -47.6976
    Traceless Quadrupole Moments (Debye-Ang)
       QXX      39.1439    QYY     -45.8824    QZZ       6.7385
       QXY      85.8628    QXZ    -117.9038    QYZ     -59.2721
    Octopole Moments (Debye-Ang^2)
       XXX      17.1815    XXY      27.5355    XYY       5.7259
       YYY     -16.5427    XXZ      18.5316    XYZ     -23.1425
       YYZ      20.7865    XZZ      34.8260    YZZ      28.8296
       ZZZ     -75.8564
    Traceless Octopole Moments (Debye-Ang^2)
       XXX    -261.8779    YYY    -606.5424    ZZZ    -809.0020
       XXY     293.5654    XXZ     387.5893    XYY     -87.3111
       XYZ    -347.1382    XZZ     349.1891    YYZ     421.4127
       YZZ     312.9770
    Hexadecapole Moments (Debye-Ang^3)
      XXXX   -2757.0983   XXXY    -241.4835   XXYY    -567.1309
      XYYY    -501.3544   YYYY    -839.2163   XXXZ     831.0036
      XXYZ     -56.4423   XYYZ     288.5333   YYYZ     419.9142
      XXZZ    -736.6607   XYZZ      12.4479   YYZZ    -580.9186
      XZZZ     725.6485   YZZZ     236.3575   ZZZZ   -1980.9005
    Traceless Hexadecapole Moments (Debye-Ang^3)
      XXXX   -8134.9510   XXXY    7511.7806   XXXZ    4222.0315
      XXYY    3133.6848   XXYZ  -14918.5169   XXZZ    5001.2662
      XYYY  -19774.6638   XYYZ    2618.2154   XYZZ   12262.8832
      XZZZ   -6840.2470   YYYY    6616.4891   YYYZ   17114.7533
      YYZZ   -9750.1739   YZZZ   -2196.2364   ZZZZ    4748.9077
 -----------------------------------------------------------------

--------------------------------------------------
-  Entering drvman on Sun Oct 13 19:44:39 2024  -
--------------------------------------------------

 Calculating analytic gradient of the SCF energy
 Gradient of SCF Energy
           1           2           3           4           5           6
    1   0.0000442  -0.0000392  -0.0003191   0.0002873  -0.0000088  -0.0000671
    2   0.0003091   0.0002914  -0.0001223   0.0002417   0.0001573  -0.0004829
```

```
    3   0.0000733   0.0001866  -0.0004928  -0.0001510  -0.0003040   0.0004250
           7           8           9          10          11          12
    1  -0.0002200   0.0001262   0.0000568  -0.0008056   0.0002262  -0.0001403
    2   0.0002408  -0.0005257  -0.0003591   0.0013080  -0.0013303   0.0029750
    3   0.0001633  -0.0000934   0.0004824  -0.0000802  -0.0004453   0.0007114
          13          14          15          16          17          18
    1   0.0007116   0.0002435  -0.0000913   0.0001047   0.0000027  -0.0010524
    2  -0.0010877   0.0006154  -0.0000967   0.0000014  -0.0000323  -0.0018197
    3   0.0001269  -0.0003534  -0.0004188   0.0005038  -0.0000908  -0.0017045
          19          20          21          22          23          24
    1  -0.0000625   0.0000122   0.0000143   0.0001047  -0.0000719   0.0000978
    2  -0.0000537  -0.0000155  -0.0000719  -0.0000482  -0.0000899   0.0000590
    3   0.0001332   0.0001232   0.0000777   0.0002300  -0.0000215  -0.0000521
          25          26          27          28          29          30
    1   0.0001163   0.0002280  -0.0004417   0.0001937   0.0000352   0.0000594
    2   0.0000185  -0.0003683  -0.0000750   0.0001409   0.0001331  -0.0000035
    3   0.0001346  -0.0001228  -0.0001418  -0.0000329  -0.0001257   0.0000296
          31          32          33          34          35          36
    1   0.0001291   0.0000139  -0.0000111   0.0000038   0.0000386   0.0000180
    2  -0.0000131   0.0001189  -0.0000233   0.0000243  -0.0000026   0.0000191
    3   0.0000212  -0.0001782  -0.0001490   0.0000876  -0.0000414   0.0000624
          37          38
    1  -0.0003225   0.0007855
    2   0.0001501  -0.0001823
    3   0.0007147   0.0007130
 Max gradient component =       2.975E-03
 RMS gradient           =       5.000E-04
 Gradient time:  CPU 161.94 s  wall 268.10 s

-------------------------------------------------
-  Entering optman on Sun Oct 13 19:49:07 2024  -
-------------------------------------------------

 Geometry Optimization Parameters
   NAtoms,    NIC,     NZ,  NCons,   NDum,   NFix, NCnnct, MaxDiis
       38     272       0       0       0       0       0       0

 Cartesian Hessian Update
 Hessian updated using BFGS update


** GEOMETRY OPTIMIZATION IN DELOCALIZED INTERNAL COORDINATES **
   Searching for a Minimum

   Optimization Cycle:   8

                     Coordinates (Angstroms)
     ATOM                X               Y               Z
      1  N         1.9800518839    0.3476721314   -1.5705205756
      2  C         2.9626836115    1.2738442803   -1.7665674065
      3  C         3.1339820982    1.7824141090   -3.0480847379
      4  C         2.3504427953    1.3479325466   -4.1034882636
      5  C         1.3751305469    0.3803309914   -3.8731681880
      6  C         1.2174390332   -0.0995373770   -2.5993206558
      7  C         3.8415103243    1.7091190630   -0.6339351163
      8  C         1.7741753612   -0.2747973790   -0.2283596493
      9  N        -1.4163395184   -1.8929443369    0.8556537407
     10  C        -2.2816159341   -0.8453243696    0.9494973870
     11  N        -1.8836840932    0.3656283290    0.6625160205
     12  C        -0.6145750798    0.5958473365    0.2893541143
     13  C         0.3296021509   -0.4802095717    0.1373637409
     14  C        -0.1371605435   -1.7198439389    0.4493608074
     15  C        -3.6639706693   -1.0941782093    1.4384484030
     16  C        -3.7974461084   -0.7096254966    2.9316479098
     17  C        -2.9134531460   -1.5393844959    3.8554387733
     18  N        -0.3029090128    1.8630252183    0.0490216687
     19  H         3.9070442200    2.5231339037   -3.2061898267
     20  H         2.5011997174    1.7526218269   -5.0973715679
     21  H         0.7487615444   -0.0013885997   -4.6683735093
     22  H         0.4815508726   -0.8524725090   -2.3526520863
```

```
    23  H         4.5793649178    2.4179018021   -1.0048073988
    24  H         4.3837198157    0.8655917291   -0.1987015825
    25  H         3.2855978406    2.2095171564    0.1649359844
    26  H         2.2550277580    0.3623668351    0.5113325984
    27  H         2.3049613475   -1.2286842185   -0.2228303233
    28  H        -1.7399681550   -2.8177840805    1.1130344826
    29  H         0.4773791125   -2.6112296292    0.4001705443
    30  H        -4.3401791439   -0.4833699497    0.8378607203
    31  H        -3.9314618151   -2.1452965375    1.2958713070
    32  H        -4.8483158747   -0.8445157219    3.1939988828
    33  H        -3.5809193464    0.3562904072    3.0390629983
    34  H        -3.1142242702   -2.6101754094    3.7488703052
    35  H        -3.1027286912   -1.2798972450    4.8976127318
    36  H        -1.8459403861   -1.3658742698    3.6766260995
    37  H         0.6292101197    2.1759350087   -0.1591385602
    38  H        -1.0039432831    2.5673606703    0.2358302280
   Point Group: c1    Number of degrees of freedom:   108


   Energy is   -763.970057751

 Hessian updated using BFGS update
  internal optimization (0)

108 Hessian modes will be used to form the next step
  Hessian Eigenvalues:
     0.001804    0.002640    0.003161    0.004110    0.006780    0.012904
     0.014085    0.019081    0.019521    0.020460    0.021146    0.022150
     0.022604    0.023931    0.024087    0.024644    0.025363    0.025831
     0.026962    0.028202    0.028932    0.029332    0.034744    0.036428
     0.038642    0.040446    0.042787    0.043610    0.044349    0.045622
     0.047666    0.050184    0.053908    0.055823    0.066627    0.079943
     0.085591    0.095127    0.121752    0.122639    0.127151    0.131031
     0.132021    0.132826    0.136940    0.140752    0.143965    0.145150
     0.146818    0.147689    0.148647    0.152276    0.152901    0.153500
     0.155891    0.160506    0.192396    0.206286    0.209854    0.214443
     0.220203    0.229163    0.237531    0.248781    0.251655    0.253947
     0.267741    0.272340    0.283082    0.286905    0.298950    0.300147
     0.300613    0.301209    0.301827    0.303083    0.304023    0.304863
     0.305015    0.305239    0.306412    0.308678    0.312095    0.313988
     0.325145    0.332433    0.333087    0.335638    0.339163    0.341113
     0.350631    0.352378    0.362244    0.363911    0.378649    0.385871
     0.394048    0.401746    0.406408    0.416891    0.420483    0.434112
     0.440051    0.448918    0.459976    0.518176    0.637711    0.732168

 Minimum search - taking simple RFO step
 Searching for Lamda that Minimizes Along All modes
 Value Taken    Lamda =  -0.00066285
 Calculated Step too Large.  Step scaled by  0.741626
 Step Taken.  Stepsize is  0.300000

                             Maximum     Tolerance    Cnvgd?
         Gradient           0.001720      0.000800      NO
         Displacement       0.121208      0.001400      NO
         Energy change     -0.000618      0.000228      NO


 New Cartesian Coordinates Obtained by Inverse Iteration

 Displacement from previous Coordinates is:  0.808504
 ----------------------------------------------------------------
             Standard Nuclear Orientation (Angstroms)
    I     Atom           X                Y                Z
 ----------------------------------------------------------------
    1      N       1.9707573776     0.3813300482    -1.5654125205
    2      C       2.9973092688     1.2561247355    -1.7757021061
    3      C       3.1986427284     1.7299372831    -3.0651204671
    4      C       2.3997001695     1.3105006477    -4.1154389723
    5      C       1.3792486139     0.3943956733    -3.8713958337
    6      C       1.1932768518    -0.0507997118    -2.5895586705
```

```
    7      C        3.8860386663     1.6760723846    -0.6455614190
    8      C        1.7437650954    -0.2033338939    -0.2078858410
    9      N       -1.4283937378    -1.8845489625     0.8351017989
   10      C       -2.3021413823    -0.8462743769     0.9530884598
   11      N       -1.9203605256     0.3737033390     0.6783982003
   12      C       -0.6577345718     0.6197712389     0.2916366447
   13      C        0.2994365710    -0.4426360415     0.1315936669
   14      C       -0.1511014619    -1.6921686294     0.4318013178
   15      C       -3.6768494539    -1.1146246562     1.4545358359
   16      C       -3.7986082769    -0.7470764219     2.9524828535
   17      C       -2.8884653773    -1.5702485608     3.8566287604
   18      N       -0.3577076344     1.8920597072     0.0626863186
   19      H        4.0056508682     2.4310099343    -3.2342637638
   20      H        2.5730791983     1.6875595343    -5.1167149017
   21      H        0.7390918840     0.0270582004    -4.6623751985
   22      H        0.4209044122    -0.7625578885    -2.3333912972
   23      H        4.6712221195     2.3253948918    -1.0282379240
   24      H        4.3674746357     0.8182331909    -0.1691703252
   25      H        3.3465757443     2.2396089845     0.1223869694
   26      H        2.1765789947     0.4789199519     0.5220574075
   27      H        2.3095876913    -1.1354674187    -0.1521187871
   28      H       -1.7419016274    -2.8158604513     1.0819694112
   29      H        0.4756704636    -2.5747698996     0.3777162922
   30      H       -4.3656776253    -0.5046307729     0.8674610511
   31      H       -3.9356457896    -2.1667604096     1.3033979837
   32      H       -4.8432857840    -0.9042201303     3.2273235678
   33      H       -3.5996779490     0.3213247327     3.0688545859
   34      H       -3.0712870139    -2.6433522105     3.7407564837
   35      H       -3.0692410691    -1.3251374951     4.9038257953
   36      H       -1.8266928269    -1.3755701065     3.6658257790
   37      H        0.5508958107     2.2073890748    -0.2293061585
   38      H       -1.0701350582     2.5896444845     0.2321250027
 ----------------------------------------------------------------
 Nuclear Repulsion Energy =        1309.49053871 hartrees
 There are       65 alpha and       65 beta electrons

------------------------------------------------
-  Entering fldman on Sun Oct 13 19:49:07 2024  -
------------------------------------------------

 Applying Cartesian multipole field
    Component          Value
    ---------          -----
      (2,0,0)        1.00000E-11
      (0,2,0)        2.00000E-11
      (0,0,2)       -3.00000E-11
 Nucleus-field energy     =    -0.0000000244 hartrees

------------------------------------------------
-  Entering gesman on Sun Oct 13 19:49:07 2024  -
------------------------------------------------

 Requested basis set is 6-311+G(d,p)
 There are 188 shells and 516 basis functions
 A cutoff of  1.0D-12 yielded  12631 shell pairs
 There are    100871 function pairs (    107578 Cartesian)
 Smallest overlap matrix eigenvalue = 1.63E-06
 Linear dependence detected in AO basis
 Tighter screening thresholds may be required for diffuse basis sets
 Use S2THRESH > 12 and THRESH = 14 in case of SCF convergence issues
 Number of orthogonalized atomic orbitals = 510
 Maximum deviation from orthogonality = 1.816E-11
 Guess MOs from SCF MO coefficient file
 Reading MOs from coefficient file
 Reading MOs from coefficient file

------------------------------------------------
-  Entering scfman on Sun Oct 13 19:49:08 2024  -
------------------------------------------------
```

```
 Long-range K will be added via erf
 Coulomb attenuation parameter = 0.2 bohr**(-1)
 A restricted hybrid HF-DFT SCF calculation will be
 performed using Pulay DIIS + Geometric Direct Minimization
 Exchange:     0.2220 Hartree-Fock + 1.0000 wB97X-D + LR-HF
 Correlation:  1.0000 wB97X-D
 Using Euler-Maclaurin-Lebedev (75,302) quadrature formula
 Dispersion:   Grimme D
 SCF converges when RMS gradient is below 1.0E-07
 Geometry optimization detected.  Setting ReadMinima to 0
 Setting SaveMinima to 0
 ---------------------------------------
  Cycle       Energy         DIIS Error
 ---------------------------------------
    1    -763.9567787101      6.66E-04
    2    -763.9697035236      9.28E-05
    3    -763.9702758859      5.62E-05
    4    -763.9703724645      2.57E-05
    5    -763.9703981078      8.07E-06
    6    -763.9704005262      3.30E-06
    7    -763.9704009134      8.36E-07
    8    -763.9704009528      3.46E-07
    9    -763.9704009579      8.81E-08 Convergence criterion met
 ---------------------------------------
 SCF time:  CPU 330.78 s  wall 553.06 s
 SCF   energy in the final basis set = -763.97040096
 Total energy in the final basis set = -763.97040096

-------------------------------------------------
-  Entering anlman on Sun Oct 13 19:58:21 2024  -
-------------------------------------------------



 --------------------------------------------------------------

                   Orbital Energies (a.u.)
 --------------------------------------------------------------


 Alpha MOs
 -- Occupied --
-14.8178 -14.7889 -14.7423 -14.7073 -10.6777 -10.6623 -10.6470 -10.6409
-10.6347 -10.6268 -10.6054 -10.5850 -10.5743 -10.5720 -10.5492 -10.5256
-10.4936 -10.4688  -1.4153  -1.4051  -1.3499  -1.2773  -1.2312  -1.2017
 -1.1884  -1.1422  -1.1002  -1.0838  -1.0556  -1.0541  -1.0309  -1.0063
 -0.9915  -0.9564  -0.9436  -0.9183  -0.9095  -0.8889  -0.8821  -0.8677
 -0.8583  -0.8423  -0.8409  -0.8213  -0.8084  -0.7969  -0.7901  -0.7788
 -0.7766  -0.7595  -0.7509  -0.7476  -0.7446  -0.7301  -0.7204  -0.7166
 -0.6992  -0.6692  -0.6587  -0.6434  -0.6350  -0.6266  -0.6230  -0.6222
 -0.6130
 -- Virtual --
 -0.2716  -0.2578  -0.2441  -0.2264  -0.1757  -0.1623  -0.1469  -0.1428
 -0.1366  -0.1216  -0.1148  -0.1134  -0.1092  -0.1007  -0.0987  -0.0967
 -0.0902  -0.0872  -0.0822  -0.0771  -0.0763  -0.0739  -0.0705  -0.0685
 -0.0636  -0.0568  -0.0561  -0.0527  -0.0485  -0.0458  -0.0416  -0.0383
 -0.0304  -0.0281  -0.0243  -0.0208  -0.0163  -0.0149  -0.0127  -0.0064
 -0.0050  -0.0024  -0.0002   0.0008   0.0074   0.0080   0.0098   0.0162
  0.0183   0.0194   0.0246   0.0267   0.0330   0.0360   0.0369   0.0377
  0.0419   0.0467   0.0495   0.0534   0.0561   0.0616   0.0638   0.0642
  0.0717   0.0727   0.0772   0.0798   0.0801   0.0820   0.0892   0.0900
  0.0965   0.0987   0.0998   0.1047   0.1086   0.1093   0.1149   0.1154
  0.1193   0.1236   0.1274   0.1291   0.1334   0.1378   0.1430   0.1486
  0.1517   0.1545   0.1591   0.1651   0.1674   0.1720   0.1737   0.1834
  0.1868   0.1904   0.1961   0.1994   0.2057   0.2125   0.2149   0.2220
  0.2326   0.2415   0.2557   0.2760   0.2853   0.2953   0.2976   0.3099
  0.3172   0.3269   0.3353   0.3428   0.3507   0.3548   0.3600   0.3657
  0.3712   0.3859   0.3890   0.3918   0.3983   0.4078   0.4166   0.4261
  0.4292   0.4346   0.4451   0.4506   0.4543   0.4604   0.4770   0.4776
  0.4867   0.4904   0.4987   0.5028   0.5088   0.5105   0.5174   0.5185
  0.5248   0.5267   0.5338   0.5363   0.5422   0.5504   0.5566   0.5578
  0.5645   0.5695   0.5749   0.5774   0.5913   0.5942   0.5953   0.6056
```

| | | | | | | | |
|---|---|---|---|---|---|---|---|
| 0.6119 | 0.6156 | 0.6273 | 0.6296 | 0.6409 | 0.6513 | 0.6570 | 0.6593 |
| 0.6685 | 0.6752 | 0.6790 | 0.6863 | 0.6945 | 0.7019 | 0.7123 | 0.7166 |
| 0.7212 | 0.7276 | 0.7423 | 0.7481 | 0.7607 | 0.7685 | 0.7798 | 0.7825 |
| 0.7935 | 0.8003 | 0.8105 | 0.8195 | 0.8328 | 0.8382 | 0.8550 | 0.8625 |
| 0.8663 | 0.8717 | 0.8789 | 0.8954 | 0.9080 | 0.9128 | 0.9292 | 0.9351 |
| 0.9529 | 0.9642 | 0.9699 | 0.9894 | 1.0008 | 1.0050 | 1.0266 | 1.0338 |
| 1.0482 | 1.0722 | 1.0809 | 1.1119 | 1.1247 | 1.1356 | 1.1688 | 1.1818 |
| 1.1914 | 1.1930 | 1.2199 | 1.2303 | 1.2637 | 1.2737 | 1.2891 | 1.3066 |
| 1.3094 | 1.3231 | 1.3248 | 1.3321 | 1.3402 | 1.3639 | 1.3690 | 1.3739 |
| 1.3902 | 1.3942 | 1.3975 | 1.3995 | 1.4089 | 1.4171 | 1.4330 | 1.4393 |
| 1.4427 | 1.4492 | 1.4551 | 1.4560 | 1.4714 | 1.4802 | 1.4927 | 1.4975 |
| 1.5003 | 1.5078 | 1.5245 | 1.5293 | 1.5338 | 1.5431 | 1.5509 | 1.5555 |
| 1.5664 | 1.5693 | 1.5717 | 1.5772 | 1.5872 | 1.5978 | 1.6053 | 1.6134 |
| 1.6302 | 1.6311 | 1.6396 | 1.6589 | 1.6683 | 1.6722 | 1.6806 | 1.6862 |
| 1.6953 | 1.7046 | 1.7238 | 1.7287 | 1.7388 | 1.7442 | 1.7569 | 1.7780 |
| 1.7925 | 1.7967 | 1.8103 | 1.8167 | 1.8236 | 1.8489 | 1.8631 | 1.8694 |
| 1.8857 | 1.9028 | 1.9118 | 1.9359 | 1.9517 | 1.9592 | 1.9834 | 1.9872 |
| 2.0101 | 2.0227 | 2.0277 | 2.0437 | 2.0665 | 2.0704 | 2.0777 | 2.0966 |
| 2.1117 | 2.1235 | 2.1308 | 2.1451 | 2.1717 | 2.1886 | 2.2242 | 2.2279 |
| 2.2367 | 2.2415 | 2.2525 | 2.2573 | 2.2662 | 2.2710 | 2.2845 | 2.2883 |
| 2.2903 | 2.3141 | 2.3393 | 2.3577 | 2.3641 | 2.3657 | 2.3911 | 2.3981 |
| 2.4089 | 2.4147 | 2.4209 | 2.4381 | 2.4463 | 2.4604 | 2.4668 | 2.4913 |
| 2.5006 | 2.5149 | 2.5245 | 2.5322 | 2.5451 | 2.5462 | 2.5609 | 2.5656 |
| 2.5726 | 2.5795 | 2.5895 | 2.5923 | 2.5972 | 2.5996 | 2.6157 | 2.6204 |
| 2.6387 | 2.6533 | 2.6664 | 2.6710 | 2.6757 | 2.6790 | 2.6914 | 2.7068 |
| 2.7137 | 2.7318 | 2.7431 | 2.7495 | 2.7563 | 2.7731 | 2.7825 | 2.7891 |
| 2.7917 | 2.8019 | 2.8246 | 2.8363 | 2.8466 | 2.8759 | 2.8917 | 2.8980 |
| 2.9197 | 2.9442 | 2.9604 | 3.0047 | 3.0595 | 3.1014 | 3.1299 | 3.1385 |
| 3.1799 | 3.2466 | 3.2536 | 3.2802 | 3.2886 | 3.3333 | 3.3706 | 3.4071 |
| 3.4347 | 3.4778 | 3.5279 | 3.5463 | 3.5508 | 3.6002 | 3.6117 | 3.6468 |
| 3.6664 | 3.6910 | 3.7087 | 3.7289 | 3.7345 | 3.7462 | 3.7562 | 3.7695 |
| 3.7806 | 3.8397 | 3.8695 | 3.9093 | 3.9339 | 3.9787 | 4.0279 | 4.0812 |
| 4.1119 | 4.2071 | 4.2959 | 4.3157 | 4.6074 | 4.6669 | 4.6951 | 4.7231 |
| 4.9256 | 5.0376 | 5.2024 | 23.5231 | 23.5758 | 23.7183 | 23.7280 | 23.7423 |
| 23.7731 | 23.8015 | 23.8267 | 23.8590 | 23.8884 | 23.9168 | 23.9674 | 23.9789 |
| 24.0362 | 35.4173 | 35.4556 | 35.5243 | 35.6222 | | | |

Ground-State Mulliken Net Atomic Charges

| Atom | Charge (a.u.) |
|---|---|
| 1 N | 0.359987 |
| 2 C | 0.119747 |
| 3 C | 0.003425 |
| 4 C | -0.152842 |
| 5 C | -0.156891 |
| 6 C | -0.129552 |
| 7 C | -0.765745 |
| 8 C | -0.290034 |
| 9 N | -0.135679 |
| 10 C | -0.297901 |
| 11 N | 0.012202 |
| 12 C | 0.294343 |
| 13 C | -0.214363 |
| 14 C | 0.144857 |
| 15 C | -0.346490 |
| 16 C | -0.269991 |
| 17 C | -0.518181 |
| 18 N | -0.492854 |
| 19 H | 0.221562 |
| 20 H | 0.235748 |
| 21 H | 0.228153 |
| 22 H | 0.232135 |
| 23 H | 0.232439 |
| 24 H | 0.235670 |
| 25 H | 0.207103 |
| 26 H | 0.251531 |
| 27 H | 0.256532 |
| 28 H | 0.394260 |

```
    29 H                     0.216647
    30 H                     0.250309
    31 H                     0.208393
    32 H                     0.223605
    33 H                     0.205713
    34 H                     0.176900
    35 H                     0.216640
    36 H                     0.140418
    37 H                     0.319565
    38 H                     0.382641
  ----------------------------------------
  Sum of atomic charges =     2.000000

 -----------------------------------------------------------------
                    Cartesian Multipole Moments
 -----------------------------------------------------------------
    Charge (ESU x 10^10)
                 9.6064
    Dipole Moment (Debye)
         X       5.0105      Y      -0.0313      Z      -5.7037
       Tot       7.5920
    Quadrupole Moments (Debye-Ang)
        XX     -35.9422     XY      28.6341     YY     -65.8640
        XZ     -39.7967     YZ     -19.9885     ZZ     -47.6204
    Traceless Quadrupole Moments (Debye-Ang)
       QXX      41.6000    QYY     -48.1654    QZZ       6.5654
       QXY      85.9022    QXZ    -119.3901    QYZ     -59.9654
    Octopole Moments (Debye-Ang^2)
       XXX      23.9466    XXY      27.6710    XYY       2.6734
       YYY     -17.9765    XXZ      14.6369    XYZ     -23.0010
       YYZ      22.1900    XZZ      37.6126    YZZ      27.5426
       ZZZ     -76.4323
    Traceless Octopole Moments (Debye-Ang^2)
       XXX    -218.8943    YYY    -604.7813    ZZZ    -790.0350
       XXY     303.3540    XXZ     338.3693    XYY    -152.5973
       XYZ    -345.0153    XZZ     371.4916    YYZ     451.6657
       YZZ     301.4273
    Hexadecapole Moments (Debye-Ang^3)
      XXXX   -2767.8968   XXXY    -235.0100   XXYY    -575.1007
      XYYY    -515.7423   YYYY    -834.5516   XXXZ     820.1129
      XXYZ     -61.8720   XYYZ     296.7488   YYYZ     429.1147
      XXZZ    -728.3315   XYZZ      13.3664   YYZZ    -589.5894
      XZZZ     721.9060   YZZZ     240.2147   ZZZZ   -1983.4924
    Traceless Hexadecapole Moments (Debye-Ang^3)
      XXXX   -8557.4091   XXXY    8506.3122   XXXZ    3367.3035
      XXYY    2557.0389   XXYZ  -15605.2029   XXZZ    6000.3702
      XYYY  -20970.5765   XYYZ    3577.1095   XYZZ   12464.2643
      XZZZ   -6944.4130   YYYY    7955.9726   YYYZ   17731.1238
      YYZZ  -10513.0115   YZZZ   -2125.9209   ZZZZ    4512.6413
 -----------------------------------------------------------------

-------------------------------------------------
-  Entering drvman on Sun Oct 13 19:58:21 2024  -
-------------------------------------------------

 Calculating analytic gradient of the SCF energy
 Gradient of SCF Energy
            1           2           3           4           5           6
    1  -0.0000416  -0.0000765   0.0000465   0.0000370  -0.0002901   0.0002088
    2   0.0003207   0.0001975  -0.0000296  -0.0000759  -0.0003141  -0.0000137
    3  -0.0000691  -0.0005272   0.0003195   0.0003112   0.0001896  -0.0003933
            7           8           9          10          11          12
    1   0.0000075  -0.0001217  -0.0000730  -0.0005242  -0.0000022   0.0000872
    2   0.0000507  -0.0004542  -0.0004307   0.0002118  -0.0002807   0.0016593
    3   0.0000135   0.0001983   0.0003490  -0.0000965  -0.0003540   0.0001556
           13          14          15          16          17          18
    1   0.0006611  -0.0001084  -0.0000673   0.0000259   0.0000318  -0.0000927
    2   0.0000247  -0.0002256  -0.0001249   0.0000422   0.0000104  -0.0008397
    3  -0.0000861  -0.0001327  -0.0000204   0.0001807  -0.0000787  -0.0002405
           19          20          21          22          23          24
```

```
    1  -0.0000511   0.0000005   0.0000175   0.0000970  -0.0000275   0.0000461
    2  -0.0000014   0.0000369  -0.0000486   0.0000133   0.0000520   0.0000391
    3   0.0000853  -0.0000160   0.0000537   0.0000766  -0.0000161  -0.0000480
           25          26          27          28          29          30
    1  -0.0000060  -0.0000258  -0.0001314   0.0001115   0.0000347   0.0000433
    2   0.0001687  -0.0001826  -0.0000519   0.0000503   0.0000733   0.0000436
    3   0.0000923  -0.0000306  -0.0000703  -0.0000188  -0.0000605   0.0000062
           31          32          33          34          35          36
    1   0.0000493  -0.0000071  -0.0000157   0.0000023   0.0000104  -0.0000109
    2   0.0000112   0.0000242   0.0000028   0.0000113   0.0000127   0.0000207
    3  -0.0000015  -0.0000390  -0.0000701   0.0000421  -0.0000033  -0.0000009
           37          38
    1  -0.0002388   0.0003936
    2  -0.0000383   0.0000347
    3  -0.0000675   0.0003676
 Max gradient component =       1.659E-03
 RMS gradient           =       2.451E-04
 Gradient time:  CPU 156.56 s  wall 258.88 s

--------------------------------------------------
-  Entering optman on Sun Oct 13 20:02:40 2024  -
--------------------------------------------------

 Geometry Optimization Parameters
   NAtoms,    NIC,     NZ,  NCons,   NDum,   NFix, NCnnct, MaxDiis
       38     272       0       0       0       0       0       0

 Cartesian Hessian Update
 Hessian updated using BFGS update


** GEOMETRY OPTIMIZATION IN DELOCALIZED INTERNAL COORDINATES **
   Searching for a Minimum

   Optimization Cycle:   9

                    Coordinates (Angstroms)
     ATOM              X               Y               Z
      1  N         1.9707573776    0.3813300482   -1.5654125205
      2  C         2.9973092688    1.2561247355   -1.7757021061
      3  C         3.1986427284    1.7299372831   -3.0651204671
      4  C         2.3997001695    1.3105006477   -4.1154389723
      5  C         1.3792486139    0.3943956733   -3.8713958337
      6  C         1.1932768518   -0.0507997118   -2.5895586705
      7  C         3.8860386663    1.6760723846   -0.6455614190
      8  C         1.7437650954   -0.2033338939   -0.2078858410
      9  N        -1.4283937378   -1.8845489625    0.8351017989
     10  C        -2.3021413823   -0.8462743769    0.9530884598
     11  N        -1.9203605256    0.3737033390    0.6783982003
     12  C        -0.6577345718    0.6197712389    0.2916366447
     13  C         0.2994365710   -0.4426360415    0.1315936669
     14  C        -0.1511014619   -1.6921686294    0.4318013178
     15  C        -3.6768494539   -1.1146246562    1.4545358359
     16  C        -3.7986082769   -0.7470764219    2.9524828535
     17  C        -2.8884653773   -1.5702485608    3.8566287604
     18  N        -0.3577076344    1.8920597072    0.0626863186
     19  H         4.0056508682    2.4310099343   -3.2342637638
     20  H         2.5730791983    1.6875595343   -5.1167149017
     21  H         0.7390918840    0.0270582004   -4.6623751985
     22  H         0.4209044122   -0.7625578885   -2.3333912972
     23  H         4.6712221195    2.3253948918   -1.0282379240
     24  H         4.3674746357    0.8182331909   -0.1691703252
     25  H         3.3465757443    2.2396089845    0.1223869694
     26  H         2.1765789947    0.4789199519    0.5220574075
     27  H         2.3095876913   -1.1354674187   -0.1521187871
     28  H        -1.7419016274   -2.8158604513    1.0819694112
     29  H         0.4756704636   -2.5747698996    0.3777162922
     30  H        -4.3656776253   -0.5046307729    0.8674610511
     31  H        -3.9356457896   -2.1667604096    1.3033979837
     32  H        -4.8432857840   -0.9042201303    3.2273235678
```

```
    33  H        -3.5996779490    0.3213247327    3.0688545859
    34  H        -3.0712870139   -2.6433522105    3.7407564837
    35  H        -3.0692410691   -1.3251374951    4.9038257953
    36  H        -1.8266928269   -1.3755701065    3.6658257790
    37  H         0.5508958107    2.2073890748   -0.2293061585
    38  H        -1.0701350582    2.5896444845    0.2321250027
   Point Group: c1    Number of degrees of freedom:   108


   Energy is   -763.970400958

 Hessian updated using BFGS update
  internal optimization (0)

108 Hessian modes will be used to form the next step
  Hessian Eigenvalues:
     0.001862    0.002632    0.002931    0.004111    0.006580    0.012910
     0.014715    0.019101    0.019537    0.020559    0.021088    0.022118
     0.022612    0.023919    0.024091    0.024641    0.025360    0.025656
     0.026954    0.028202    0.029100    0.029247    0.035007    0.036412
     0.038615    0.040342    0.042776    0.043608    0.044276    0.045613
     0.047157    0.049887    0.053961    0.055822    0.066421    0.079898
     0.085591    0.095128    0.121750    0.122639    0.127148    0.130979
     0.132092    0.132820    0.136869    0.140859    0.143846    0.145013
     0.146802    0.147689    0.148644    0.152275    0.152892    0.153589
     0.157850    0.160572    0.192395    0.206282    0.209714    0.214488
     0.219311    0.229047    0.237530    0.249330    0.251646    0.254588
     0.267738    0.272291    0.283424    0.286941    0.298946    0.300147
     0.300613    0.301210    0.301827    0.303116    0.304022    0.304867
     0.305036    0.305246    0.306458    0.308487    0.312074    0.313988
     0.324764    0.332491    0.333351    0.335829    0.339149    0.341113
     0.351392    0.352885    0.362345    0.363748    0.378164    0.386697
     0.394613    0.402330    0.407145    0.416883    0.420826    0.435571
     0.438969    0.450772    0.460309    0.517996    0.637578    0.730494

 Minimum search - taking simple RFO step
 Searching for Lamda that Minimizes Along All modes
 Value Taken    Lamda =  -0.00020654
 Step Taken.  Stepsize is  0.233154

                         Maximum     Tolerance    Cnvgd?
        Gradient        0.001732      0.000800      NO
        Displacement    0.094652      0.001400      NO
        Energy change  -0.000343      0.000228      NO


 New Cartesian Coordinates Obtained by Inverse Iteration

 Displacement from previous Coordinates is:  0.734113
 ----------------------------------------------------------------
             Standard Nuclear Orientation (Angstroms)
    I     Atom           X                Y                Z
 ----------------------------------------------------------------
    1      N       1.9621021390     0.4132194222    -1.5622345743
    2      C       3.0284675914     1.2372368257    -1.7816908431
    3      C       3.2567697690     1.6815546395    -3.0783145728
    4      C       2.4433030927     1.2853878276    -4.1263211276
    5      C       1.3799444581     0.4231068436    -3.8712488483
    6      C       1.1678520372     0.0044028570    -2.5830104007
    7      C       3.9264413257     1.6363222214    -0.6521150069
    8      C       1.7175049388    -0.1406747237    -0.1943782714
    9      N      -1.4307514455    -1.8751263732     0.8298353908
   10      C      -2.3160209245    -0.8482681276     0.9590430265
   11      N      -1.9535077748     0.3770080366     0.6841544853
   12      C      -0.6982478203     0.6375571868     0.2836461740
   13      C       0.2749642274    -0.4096972315     0.1267309415
   14      C      -0.1560815455    -1.6650729196     0.4279760898
   15      C      -3.6837389846    -1.1353311234     1.4693487783
   16      C      -3.7976894584    -0.7792059752     2.9704091860
   17      C      -2.8708656438    -1.5976521390     3.8619152352
```

```
   18       N      -0.4153435072     1.9136591329     0.0503670677
   19       H       4.0965592173     2.3415462696    -3.2539390577
   20       H       2.6375220114     1.6383359917    -5.1323274739
   21       H       0.7248187319     0.0755577775    -4.6591407293
   22       H       0.3610633279    -0.6660454856    -2.3217691758
   23       H       4.7486365590     2.2343829235    -1.0401735533
   24       H       4.3556115973     0.7670660414    -0.1474002354
   25       H       3.4055738542     2.2462664669     0.0929797783
   26       H       2.1120845796     0.5752186762     0.5256770028
   27       H       2.3093420015    -1.0533997178    -0.0993254347
   28       H      -1.7322065190    -2.8109374154     1.0751655609
   29       H       0.4836400929    -2.5385543295     0.3769445357
   30       H      -4.3841739235    -0.5295706237     0.8916745058
   31       H      -3.9323666043    -2.1892524622     1.3132251938
   32       H      -4.8380473922    -0.9512875901     3.2526632690
   33       H      -3.6108243778     0.2906564743     3.0931531446
   34       H      -3.0415611628    -2.6720306328     3.7392973030
   35       H      -3.0466804385    -1.3623454151     4.9121912866
   36       H      -1.8129844754    -1.3887352283     3.6644384151
   37       H       0.4731197488     2.2349808399    -0.2939570161
   38       H      -1.1442293028     2.5997210597     0.1965099503
 ----------------------------------------------------------------
 Nuclear Repulsion Energy =        1308.27181548 hartrees
 There are       65 alpha and       65 beta electrons

--------------------------------------------------
-  Entering fldman on Sun Oct 13 20:02:40 2024  -
--------------------------------------------------

 Applying Cartesian multipole field
    Component          Value
    ---------          -----
     (2,0,0)        1.00000E-11
     (0,2,0)        2.00000E-11
     (0,0,2)       -3.00000E-11
 Nucleus-field energy     =    -0.0000000245 hartrees

--------------------------------------------------
-  Entering gesman on Sun Oct 13 20:02:40 2024  -
--------------------------------------------------

 Requested basis set is 6-311+G(d,p)
 There are 188 shells and 516 basis functions
 A cutoff of  1.0D-12 yielded  12610 shell pairs
 There are    100704 function pairs (    107394 Cartesian)
 Smallest overlap matrix eigenvalue = 1.61E-06
 Linear dependence detected in AO basis
 Tighter screening thresholds may be required for diffuse basis sets
 Use S2THRESH > 12 and THRESH = 14 in case of SCF convergence issues
 Number of orthogonalized atomic orbitals = 510
 Maximum deviation from orthogonality = 1.110E-11
 Guess MOs from SCF MO coefficient file
 Reading MOs from coefficient file
 Reading MOs from coefficient file

--------------------------------------------------
-  Entering scfman on Sun Oct 13 20:02:40 2024  -
--------------------------------------------------

 Long-range K will be added via erf
 Coulomb attenuation parameter = 0.2 bohr**(-1)
 A restricted hybrid HF-DFT SCF calculation will be
 performed using Pulay DIIS + Geometric Direct Minimization
 Exchange:     0.2220 Hartree-Fock + 1.0000 wB97X-D + LR-HF
 Correlation:  1.0000 wB97X-D
 Using Euler-Maclaurin-Lebedev (75,302) quadrature formula
 Dispersion:   Grimme D
 SCF converges when RMS gradient is below 1.0E-07
 Geometry optimization detected.  Setting ReadMinima to 0
 Setting SaveMinima to 0
```

```
 ---------------------------------------
  Cycle       Energy         DIIS Error
 ---------------------------------------
    1    -763.9594303127      6.16E-04
    2    -763.9699506269      8.42E-05
    3    -763.9704118929      5.61E-05
    4    -763.9705078141      2.37E-05
    5    -763.9705304849      7.83E-06
    6    -763.9705327308      3.13E-06
    7    -763.9705330757      7.46E-07
    8    -763.9705331050      3.13E-07
    9    -763.9705331089      7.45E-08 Convergence criterion met
 ---------------------------------------
 SCF time:  CPU 329.42 s  wall 547.31 s
 SCF   energy in the final basis set = -763.97053311
 Total energy in the final basis set = -763.97053311

-------------------------------------------------
-  Entering anlman on Sun Oct 13 20:11:47 2024  -
-------------------------------------------------


 --------------------------------------------------------------

                    Orbital Energies (a.u.)
 --------------------------------------------------------------


 Alpha MOs
 -- Occupied --
-14.8179 -14.7891 -14.7422 -14.7075 -10.6777 -10.6623 -10.6472 -10.6405
-10.6348 -10.6269 -10.6054 -10.5846 -10.5742 -10.5718 -10.5483 -10.5256
-10.4936 -10.4689  -1.4156  -1.4049  -1.3498  -1.2775  -1.2311  -1.2017
 -1.1878  -1.1424  -1.1004  -1.0831  -1.0559  -1.0543  -1.0302  -1.0063
 -0.9912  -0.9566  -0.9436  -0.9180  -0.9097  -0.8887  -0.8818  -0.8675
 -0.8589  -0.8422  -0.8409  -0.8212  -0.8083  -0.7974  -0.7894  -0.7778
 -0.7767  -0.7590  -0.7506  -0.7475  -0.7444  -0.7297  -0.7204  -0.7164
 -0.6992  -0.6693  -0.6585  -0.6436  -0.6353  -0.6267  -0.6228  -0.6218
 -0.6133
 -- Virtual --
 -0.2710  -0.2579  -0.2443  -0.2265  -0.1761  -0.1623  -0.1476  -0.1429
 -0.1365  -0.1212  -0.1144  -0.1126  -0.1093  -0.1007  -0.0985  -0.0967
 -0.0901  -0.0870  -0.0821  -0.0766  -0.0759  -0.0738  -0.0702  -0.0682
 -0.0636  -0.0567  -0.0558  -0.0530  -0.0490  -0.0454  -0.0417  -0.0383
 -0.0303  -0.0291  -0.0245  -0.0215  -0.0165  -0.0147  -0.0126  -0.0059
 -0.0051  -0.0013  -0.0004   0.0011   0.0072   0.0079   0.0105   0.0168
  0.0178   0.0194   0.0241   0.0275   0.0343   0.0358   0.0368   0.0383
  0.0421   0.0465   0.0502   0.0539   0.0561   0.0614   0.0636   0.0643
  0.0716   0.0735   0.0761   0.0794   0.0809   0.0835   0.0893   0.0909
  0.0960   0.1000   0.1009   0.1039   0.1088   0.1093   0.1137   0.1156
  0.1181   0.1228   0.1278   0.1288   0.1340   0.1389   0.1425   0.1497
  0.1517   0.1548   0.1586   0.1658   0.1669   0.1716   0.1738   0.1832
  0.1867   0.1913   0.1973   0.1987   0.2061   0.2125   0.2147   0.2222
  0.2349   0.2434   0.2539   0.2762   0.2851   0.2939   0.2974   0.3090
  0.3177   0.3260   0.3333   0.3423   0.3505   0.3548   0.3609   0.3645
  0.3722   0.3858   0.3885   0.3911   0.3979   0.4069   0.4185   0.4264
  0.4310   0.4366   0.4443   0.4509   0.4530   0.4604   0.4760   0.4768
  0.4892   0.4904   0.4986   0.5033   0.5092   0.5113   0.5166   0.5184
  0.5229   0.5270   0.5347   0.5369   0.5430   0.5505   0.5558   0.5566
  0.5657   0.5705   0.5757   0.5776   0.5922   0.5934   0.5954   0.6047
  0.6106   0.6140   0.6268   0.6300   0.6410   0.6514   0.6576   0.6612
  0.6672   0.6743   0.6807   0.6841   0.6947   0.7024   0.7137   0.7165
  0.7194   0.7276   0.7412   0.7458   0.7595   0.7671   0.7769   0.7849
  0.7967   0.7998   0.8102   0.8188   0.8323   0.8350   0.8535   0.8619
  0.8649   0.8711   0.8803   0.8955   0.9084   0.9141   0.9278   0.9352
  0.9561   0.9636   0.9682   0.9891   0.9945   1.0025   1.0271   1.0389
  1.0506   1.0730   1.0862   1.1155   1.1260   1.1336   1.1670   1.1818
  1.1930   1.1945   1.2182   1.2331   1.2630   1.2759   1.2966   1.3066
  1.3105   1.3210   1.3251   1.3346   1.3414   1.3623   1.3685   1.3747
  1.3907   1.3942   1.3961   1.3984   1.4080   1.4181   1.4329   1.4375
  1.4421   1.4491   1.4554   1.4577   1.4700   1.4795   1.4919   1.4960
```

| | | | | | | | |
|---|---|---|---|---|---|---|---|
| 1.5016 | 1.5092 | 1.5242 | 1.5257 | 1.5317 | 1.5449 | 1.5503 | 1.5576 |
| 1.5658 | 1.5671 | 1.5725 | 1.5783 | 1.5878 | 1.5983 | 1.6072 | 1.6148 |
| 1.6307 | 1.6320 | 1.6391 | 1.6582 | 1.6680 | 1.6715 | 1.6804 | 1.6862 |
| 1.6951 | 1.7071 | 1.7251 | 1.7290 | 1.7392 | 1.7468 | 1.7565 | 1.7794 |
| 1.7932 | 1.7969 | 1.8099 | 1.8187 | 1.8231 | 1.8490 | 1.8634 | 1.8710 |
| 1.8851 | 1.9058 | 1.9123 | 1.9345 | 1.9519 | 1.9610 | 1.9845 | 1.9900 |
| 2.0101 | 2.0180 | 2.0317 | 2.0471 | 2.0586 | 2.0698 | 2.0771 | 2.0958 |
| 2.1109 | 2.1235 | 2.1293 | 2.1433 | 2.1715 | 2.1907 | 2.2259 | 2.2266 |
| 2.2361 | 2.2463 | 2.2521 | 2.2568 | 2.2628 | 2.2707 | 2.2850 | 2.2878 |
| 2.2892 | 2.3150 | 2.3410 | 2.3583 | 2.3633 | 2.3663 | 2.3917 | 2.3993 |
| 2.4111 | 2.4186 | 2.4203 | 2.4377 | 2.4456 | 2.4595 | 2.4660 | 2.4902 |
| 2.5016 | 2.5142 | 2.5251 | 2.5319 | 2.5449 | 2.5460 | 2.5594 | 2.5652 |
| 2.5755 | 2.5793 | 2.5886 | 2.5906 | 2.5965 | 2.5988 | 2.6156 | 2.6245 |
| 2.6375 | 2.6503 | 2.6651 | 2.6696 | 2.6758 | 2.6828 | 2.6911 | 2.7048 |
| 2.7133 | 2.7314 | 2.7426 | 2.7514 | 2.7557 | 2.7672 | 2.7812 | 2.7871 |
| 2.7919 | 2.8055 | 2.8271 | 2.8349 | 2.8460 | 2.8780 | 2.8902 | 2.8961 |
| 2.9185 | 2.9452 | 2.9688 | 3.0063 | 3.0602 | 3.1007 | 3.1296 | 3.1379 |
| 3.1794 | 3.2455 | 3.2528 | 3.2803 | 3.2908 | 3.3285 | 3.3699 | 3.4066 |
| 3.4343 | 3.4797 | 3.5270 | 3.5471 | 3.5495 | 3.6004 | 3.6082 | 3.6454 |
| 3.6673 | 3.6911 | 3.7086 | 3.7286 | 3.7348 | 3.7461 | 3.7557 | 3.7699 |
| 3.7822 | 3.8391 | 3.8692 | 3.9098 | 3.9338 | 3.9790 | 4.0280 | 4.0815 |
| 4.1125 | 4.2071 | 4.2955 | 4.3171 | 4.6060 | 4.6666 | 4.6957 | 4.7226 |
| 4.9248 | 5.0374 | 5.2023 | 23.5225 | 23.5766 | 23.7185 | 23.7284 | 23.7432 |
| 23.7747 | 23.8017 | 23.8279 | 23.8590 | 23.8871 | 23.9168 | 23.9650 | 23.9803 |
| 24.0346 | 35.4173 | 35.4560 | 35.5243 | 35.6226 | | | |

Ground-State Mulliken Net Atomic Charges

| Atom | Charge (a.u.) |
|---|---|
| 1 N | 0.342033 |
| 2 C | 0.124792 |
| 3 C | 0.001669 |
| 4 C | -0.155786 |
| 5 C | -0.151859 |
| 6 C | -0.126477 |
| 7 C | -0.758875 |
| 8 C | -0.291213 |
| 9 N | -0.136722 |
| 10 C | -0.299576 |
| 11 N | 0.011174 |
| 12 C | 0.300311 |
| 13 C | -0.227797 |
| 14 C | 0.150782 |
| 15 C | -0.342912 |
| 16 C | -0.272006 |
| 17 C | -0.517881 |
| 18 N | -0.495460 |
| 19 H | 0.221622 |
| 20 H | 0.235533 |
| 21 H | 0.228074 |
| 22 H | 0.234746 |
| 23 H | 0.232177 |
| 24 H | 0.230995 |
| 25 H | 0.212829 |
| 26 H | 0.251425 |
| 27 H | 0.257794 |
| 28 H | 0.394552 |
| 29 H | 0.216596 |
| 30 H | 0.250368 |
| 31 H | 0.208878 |
| 32 H | 0.223672 |
| 33 H | 0.206233 |
| 34 H | 0.176798 |
| 35 H | 0.216775 |
| 36 H | 0.140242 |
| 37 H | 0.327347 |
| 38 H | 0.379144 |

```
 Sum of atomic charges =     2.000000

 -----------------------------------------------------------------
                    Cartesian Multipole Moments
 -----------------------------------------------------------------
    Charge (ESU x 10^10)
                 9.6064
    Dipole Moment (Debye)
         X       4.9527      Y       0.0455      Z      -5.7746
       Tot       7.6077
    Quadrupole Moments (Debye-Ang)
        XX     -35.0476     XY      28.4646     YY     -66.5586
        XZ     -40.1809     YZ     -20.4405     ZZ     -47.4667
    Traceless Quadrupole Moments (Debye-Ang)
       QXX      43.9302    QYY     -50.6029    QZZ       6.6727
       QXY      85.3938    QXZ    -120.5428    QYZ     -61.3215
    Octopole Moments (Debye-Ang^2)
       XXX      30.5058    XXY      27.6427    XYY      -0.2147
       YYY     -19.6986    XXZ      11.2462    XYZ     -22.5041
       YYZ      23.6015    XZZ      39.7642    YZZ      26.5248
       ZZZ     -76.4551
    Traceless Octopole Moments (Debye-Ang^2)
       XXX    -172.9107    YYY    -605.6999    ZZZ    -772.3598
       XXY     311.2342    XXZ     293.5158    XYY    -213.3858
       XYZ    -337.5614    XZZ     386.2966    YYZ     478.8441
       YZZ     294.4658
    Hexadecapole Moments (Debye-Ang^3)
      XXXX   -2774.6942   XXXY    -230.0764   XXYY    -583.4746
      XYYY    -528.6558   YYYY    -831.1363   XXXZ     809.2179
      XXYZ     -64.6855   XYYZ     304.6340   YYYZ     439.1481
      XXZZ    -720.6727   XYZZ      14.7688   YYZZ    -596.6660
      XZZZ     718.1917   YZZZ     245.0025   ZZZZ   -1987.8295
    Traceless Hexadecapole Moments (Debye-Ang^3)
      XXXX   -8804.7298   XXXY    9320.3316   XXXZ    2525.9160
      XXYY    1901.0789   XXYZ  -16083.9186   XXZZ    6903.6509
      XYYY  -22030.5076   XYYZ    4505.9119   XYZZ   12710.1760
      XZZZ   -7031.8279   YYYY    9188.0299   YYYZ   18234.7342
      YYZZ  -11089.1088   YZZZ   -2150.8156   ZZZZ    4185.4579
 -----------------------------------------------------------------


-------------------------------------------------
-  Entering drvman on Sun Oct 13 20:11:47 2024  -
-------------------------------------------------


 Calculating analytic gradient of the SCF energy
 Gradient of SCF Energy
           1           2           3           4           5           6
    1  -0.0001070  -0.0000597  -0.0001648   0.0001314   0.0003503  -0.0001570
    2   0.0001551  -0.0000972  -0.0000665   0.0001992   0.0002134  -0.0003797
    3   0.0000763   0.0005526  -0.0006165  -0.0002543  -0.0004940   0.0006650
           7           8           9          10          11          12
    1   0.0001312  -0.0001495  -0.0000152   0.0003492  -0.0003103  -0.0001624
    2   0.0000206  -0.0002865   0.0000099   0.0004849  -0.0004099  -0.0000535
    3  -0.0000076   0.0002617  -0.0000176   0.0000904   0.0000793  -0.0003786
          13          14          15          16          17          18
    1   0.0000990  -0.0000269  -0.0000209   0.0000686  -0.0000049   0.0004205
    2  -0.0003920   0.0004426  -0.0000083   0.0000923   0.0000177  -0.0002210
    3  -0.0000188  -0.0001276   0.0000045  -0.0000217  -0.0000030   0.0009881
          19          20          21          22          23          24
    1  -0.0000157  -0.0000443   0.0000050   0.0000289  -0.0000990   0.0000322
    2   0.0000483   0.0000095  -0.0000485   0.0000386   0.0000623   0.0000001
    3  -0.0000100   0.0000732  -0.0000572   0.0000157  -0.0000225   0.0000031
          25          26          27          28          29          30
    1  -0.0001043  -0.0002175   0.0000885   0.0000043   0.0000088  -0.0000267
    2   0.0001640  -0.0000989  -0.0000151  -0.0000136   0.0000231   0.0000325
    3   0.0000503   0.0000087  -0.0000528  -0.0000207  -0.0000020  -0.0000198
          31          32          33          34          35          36
    1  -0.0000161   0.0000058  -0.0000169   0.0000075   0.0000061  -0.0000043
    2   0.0000226  -0.0000309   0.0000023   0.0000107   0.0000101   0.0000193
    3  -0.0000322  -0.0000097  -0.0000195   0.0000070   0.0000017  -0.0000344
```

```
          37          38
    1  -0.0000654   0.0000517
    2  -0.0000273   0.0000701
    3  -0.0005757  -0.0000813
 Max gradient component =       9.881E-04
 RMS gradient           =       2.097E-04
 Gradient time:  CPU 157.03 s  wall 261.37 s

-------------------------------------------------
-  Entering optman on Sun Oct 13 20:16:09 2024  -
-------------------------------------------------

 Geometry Optimization Parameters
   NAtoms,    NIC,     NZ,  NCons,   NDum,   NFix, NCnnct, MaxDiis
       38     272       0       0       0       0       0       0

 Cartesian Hessian Update
 Hessian updated using BFGS update


** GEOMETRY OPTIMIZATION IN DELOCALIZED INTERNAL COORDINATES **
   Searching for a Minimum

   Optimization Cycle:  10

                       Coordinates (Angstroms)
     ATOM                X               Y               Z
      1  N         1.9621021390    0.4132194222   -1.5622345743
      2  C         3.0284675914    1.2372368257   -1.7816908431
      3  C         3.2567697690    1.6815546395   -3.0783145728
      4  C         2.4433030927    1.2853878276   -4.1263211276
      5  C         1.3799444581    0.4231068436   -3.8712488483
      6  C         1.1678520372    0.0044028570   -2.5830104007
      7  C         3.9264413257    1.6363222214   -0.6521150069
      8  C         1.7175049388   -0.1406747237   -0.1943782714
      9  N        -1.4307514455   -1.8751263732    0.8298353908
     10  C        -2.3160209245   -0.8482681276    0.9590430265
     11  N        -1.9535077748    0.3770080366    0.6841544853
     12  C        -0.6982478203    0.6375571868    0.2836461740
     13  C         0.2749642274   -0.4096972315    0.1267309415
     14  C        -0.1560815455   -1.6650729196    0.4279760898
     15  C        -3.6837389846   -1.1353311234    1.4693487783
     16  C        -3.7976894584   -0.7792059752    2.9704091860
     17  C        -2.8708656438   -1.5976521390    3.8619152352
     18  N        -0.4153435072    1.9136591329    0.0503670677
     19  H         4.0965592173    2.3415462696   -3.2539390577
     20  H         2.6375220114    1.6383359917   -5.1323274739
     21  H         0.7248187319    0.0755577775   -4.6591407293
     22  H         0.3610633279   -0.6660454856   -2.3217691758
     23  H         4.7486365590    2.2343829235   -1.0401735533
     24  H         4.3556115973    0.7670660414   -0.1474002354
     25  H         3.4055738542    2.2462664669    0.0929797783
     26  H         2.1120845796    0.5752186762    0.5256770028
     27  H         2.3093420015   -1.0533997178   -0.0993254347
     28  H        -1.7322065190   -2.8109374154    1.0751655609
     29  H         0.4836400929   -2.5385543295    0.3769445357
     30  H        -4.3841739235   -0.5295706237    0.8916745058
     31  H        -3.9323666043   -2.1892524622    1.3132251938
     32  H        -4.8380473922   -0.9512875901    3.2526632690
     33  H        -3.6108243778    0.2906564743    3.0931531446
     34  H        -3.0415611628   -2.6720306328    3.7392973030
     35  H        -3.0466804385   -1.3623454151    4.9121912866
     36  H        -1.8129844754   -1.3887352283    3.6644384151
     37  H         0.4731197488    2.2349808399   -0.2939570161
     38  H        -1.1442293028    2.5997210597    0.1965099503
   Point Group: c1    Number of degrees of freedom:   108


   Energy is   -763.970533109
```

```
Hessian updated using BFGS update
 internal optimization (0)

108 Hessian modes will be used to form the next step
 Hessian Eigenvalues:
    0.001644    0.002458    0.002672    0.004109    0.006402    0.012985
    0.015457    0.019039    0.019617    0.020330    0.020732    0.022089
    0.022679    0.023911    0.024091    0.024645    0.025197    0.025856
    0.026976    0.028208    0.028726    0.029280    0.035204    0.036581
    0.038539    0.039884    0.042747    0.043600    0.044214    0.045870
    0.046331    0.050266    0.053919    0.055818    0.065428    0.079878
    0.085595    0.095142    0.121753    0.122641    0.127143    0.130865
    0.132215    0.132815    0.136918    0.140916    0.143620    0.144955
    0.146753    0.147692    0.148615    0.152340    0.152928    0.153926
    0.159775    0.163468    0.192794    0.206298    0.209521    0.214637
    0.217872    0.228914    0.237846    0.249377    0.252800    0.254933
    0.267841    0.272001    0.282939    0.287841    0.298970    0.300168
    0.300612    0.301217    0.301844    0.303194    0.304021    0.304868
    0.305050    0.305277    0.306409    0.307946    0.312324    0.314339
    0.324851    0.332602    0.333755    0.335897    0.339115    0.341257
    0.350483    0.353447    0.363338    0.370266    0.380035    0.388366
    0.395224    0.402914    0.411933    0.417299    0.421576    0.435271
    0.447231    0.451137    0.459957    0.517610    0.625526    0.728983

 Minimum search - taking simple RFO step
 Searching for Lamda that Minimizes Along All modes
 Value Taken    Lamda =  -0.00010582
 Step Taken.  Stepsize is  0.162414

                          Maximum     Tolerance    Cnvgd?
        Gradient          0.001493     0.000800      NO
        Displacement      0.063046     0.001400      NO
        Energy change    -0.000132     0.000228     YES


 New Cartesian Coordinates Obtained by Inverse Iteration

 Displacement from previous Coordinates is:  0.569261
 ----------------------------------------------------------------
            Standard Nuclear Orientation (Angstroms)
    I     Atom           X                Y                Z
 ----------------------------------------------------------------
    1      N       1.9560449810     0.4381770535    -1.5598313292
    2      C       3.0516384745     1.2212126913    -1.7863201062
    3      C       3.2967262112     1.6458137493    -3.0851181644
    4      C       2.4681529757     1.2706671866    -4.1294616948
    5      C       1.3721820150     0.4523880480    -3.8674686733
    6      C       1.1461483821     0.0517255531    -2.5769624174
    7      C       3.9591534459     1.5993066706    -0.6577250733
    8      C       1.7002635044    -0.0927331837    -0.1847771858
    9      N      -1.4267053838    -1.8688921485     0.8324243873
   10      C      -2.3249124685    -0.8532071158     0.9593146092
   11      N      -1.9787729291     0.3766063526     0.6782872704
   12      C      -0.7282766720     0.6509542060     0.2728358632
   13      C       0.2595616563    -0.3830736119     0.1258235756
   14      C      -0.1540227911    -1.6437377131     0.4329466877
   15      C      -3.6867280419    -1.1553586173     1.4768109591
   16      C      -3.7966889427    -0.8027490992     2.9790085219
   17      C      -2.8602106641    -1.6158581963     3.8653780688
   18      N      -0.4622339194     1.9294916741     0.0269935063
   19      H       4.1605576951     2.2722458300    -3.2665818858
   20      H       2.6757431422     1.6065978617    -5.1388265642
   21      H       0.7037903016     0.1244150660    -4.6524583567
   22      H       0.3152467510    -0.5868093813    -2.3112603963
   23      H       4.8127389152     2.1491307393    -1.0493598065
   24      H       4.3419829267     0.7214804208    -0.1311167351
   25      H       3.4592597575     2.2477094429     0.0689595637
   26      H       2.0696109155     0.6455868815     0.5265333309
   27      H       2.3082478256    -0.9914856897    -0.0629915298
   28      H      -1.7163004652    -2.8073935185     1.0820907674
```

```
  29      H      0.4978988946   -2.5085791118    0.3888651340
  30      H     -4.3965225464   -0.5563677766    0.9036921255
  31      H     -3.9251925771   -2.2116934931    1.3207915903
  32      H     -4.8344509375   -0.9830160793    3.2657529458
  33      H     -3.6172210476    0.2682506732    3.1032667206
  34      H     -3.0238375518   -2.6912149538    3.7416212038
  35      H     -3.0335030231   -1.3834465142    4.9167340819
  36      H     -1.8046122016   -1.3991120300    3.6641383075
  37      H      0.4196099040    2.2579518781   -0.3287027577
  38      H     -1.2043665121    2.6050162555    0.1566934557
 ----------------------------------------------------------------
 Nuclear Repulsion Energy =        1307.55808097 hartrees
 There are       65 alpha and       65 beta electrons

--------------------------------------------------
-  Entering fldman on Sun Oct 13 20:16:09 2024  -
--------------------------------------------------

 Applying Cartesian multipole field
    Component          Value
    ---------          -----
     (2,0,0)        1.00000E-11
     (0,2,0)        2.00000E-11
     (0,0,2)       -3.00000E-11
 Nucleus-field energy     =    -0.0000000245 hartrees

--------------------------------------------------
-  Entering gesman on Sun Oct 13 20:16:09 2024  -
--------------------------------------------------

 Requested basis set is 6-311+G(d,p)
 There are 188 shells and 516 basis functions
 A cutoff of  1.0D-12 yielded  12602 shell pairs
 There are    100644 function pairs (    107332 Cartesian)
 Smallest overlap matrix eigenvalue = 1.56E-06
 Linear dependence detected in AO basis
 Tighter screening thresholds may be required for diffuse basis sets
 Use S2THRESH > 12 and THRESH = 14 in case of SCF convergence issues
 Number of orthogonalized atomic orbitals = 510
 Maximum deviation from orthogonality = 1.300E-11
 Guess MOs from SCF MO coefficient file
 Reading MOs from coefficient file
 Reading MOs from coefficient file

--------------------------------------------------
-  Entering scfman on Sun Oct 13 20:16:09 2024  -
--------------------------------------------------

 Long-range K will be added via erf
 Coulomb attenuation parameter = 0.2 bohr**(-1)
 A restricted hybrid HF-DFT SCF calculation will be
 performed using Pulay DIIS + Geometric Direct Minimization
 Exchange:     0.2220 Hartree-Fock + 1.0000 wB97X-D + LR-HF
 Correlation:  1.0000 wB97X-D
 Using Euler-Maclaurin-Lebedev (75,302) quadrature formula
 Dispersion:   Grimme D
 SCF converges when RMS gradient is below 1.0E-07
 Geometry optimization detected.  Setting ReadMinima to 0
 Setting SaveMinima to 0
 ---------------------------------------
  Cycle       Energy         DIIS Error
 ---------------------------------------
    1    -763.9609753234      4.90E-04
    2    -763.9702633123      6.38E-05
    3    -763.9705460492      4.09E-05
    4    -763.9705964200      1.95E-05
    5    -763.9706111431      6.10E-06
    6    -763.9706125183      2.22E-06
    7    -763.9706126956      5.45E-07
    8    -763.9706127116      2.27E-07
```

```
    9    -763.9706127136      4.94E-08 Convergence criterion met
 ---------------------------------------
 SCF time:  CPU 322.47 s  wall 533.50 s
 SCF   energy in the final basis set = -763.97061271
 Total energy in the final basis set = -763.97061271

--------------------------------------------------
-  Entering anlman on Sun Oct 13 20:25:03 2024  -
--------------------------------------------------



 --------------------------------------------------------------

                    Orbital Energies (a.u.)
 --------------------------------------------------------------

 Alpha MOs
 -- Occupied --
-14.8177 -14.7895 -14.7421 -14.7078 -10.6776 -10.6627 -10.6476 -10.6398
-10.6350 -10.6267 -10.6055 -10.5843 -10.5738 -10.5714 -10.5475 -10.5258
-10.4938 -10.4691  -1.4156  -1.4047  -1.3496  -1.2774  -1.2309  -1.2017
 -1.1877  -1.1425  -1.1004  -1.0828  -1.0561  -1.0544  -1.0296  -1.0064
 -0.9910  -0.9566  -0.9436  -0.9178  -0.9099  -0.8887  -0.8815  -0.8672
 -0.8592  -0.8422  -0.8407  -0.8211  -0.8082  -0.7976  -0.7891  -0.7770
 -0.7767  -0.7589  -0.7505  -0.7473  -0.7442  -0.7294  -0.7204  -0.7162
 -0.6995  -0.6692  -0.6582  -0.6437  -0.6355  -0.6269  -0.6228  -0.6217
 -0.6134
 -- Virtual --
 -0.2705  -0.2580  -0.2447  -0.2261  -0.1765  -0.1624  -0.1481  -0.1429
 -0.1365  -0.1210  -0.1142  -0.1119  -0.1094  -0.1008  -0.0983  -0.0965
 -0.0900  -0.0867  -0.0819  -0.0764  -0.0758  -0.0736  -0.0700  -0.0679
 -0.0636  -0.0566  -0.0556  -0.0530  -0.0493  -0.0450  -0.0416  -0.0383
 -0.0302  -0.0298  -0.0244  -0.0222  -0.0168  -0.0147  -0.0126  -0.0061
 -0.0044  -0.0011   0.0001   0.0012   0.0071   0.0079   0.0109   0.0168
  0.0175   0.0199   0.0238   0.0282   0.0347   0.0354   0.0370   0.0395
  0.0424   0.0463   0.0504   0.0537   0.0566   0.0610   0.0635   0.0645
  0.0713   0.0743   0.0757   0.0789   0.0816   0.0846   0.0895   0.0914
  0.0949   0.1009   0.1019   0.1031   0.1091   0.1094   0.1131   0.1159
  0.1175   0.1223   0.1278   0.1290   0.1343   0.1396   0.1424   0.1498
  0.1519   0.1555   0.1584   0.1662   0.1664   0.1716   0.1741   0.1821
  0.1868   0.1916   0.1980   0.1990   0.2063   0.2123   0.2151   0.2229
  0.2368   0.2434   0.2519   0.2761   0.2850   0.2929   0.2977   0.3084
  0.3182   0.3256   0.3324   0.3417   0.3506   0.3553   0.3613   0.3640
  0.3721   0.3856   0.3885   0.3911   0.3977   0.4064   0.4189   0.4260
  0.4314   0.4377   0.4438   0.4507   0.4525   0.4613   0.4752   0.4759
  0.4896   0.4902   0.4978   0.5041   0.5099   0.5120   0.5165   0.5186
  0.5215   0.5281   0.5352   0.5376   0.5432   0.5501   0.5556   0.5557
  0.5660   0.5715   0.5754   0.5784   0.5903   0.5938   0.5950   0.6045
  0.6097   0.6134   0.6260   0.6305   0.6410   0.6504   0.6576   0.6619
  0.6672   0.6742   0.6817   0.6839   0.6948   0.7028   0.7135   0.7167
  0.7186   0.7282   0.7393   0.7454   0.7600   0.7663   0.7753   0.7871
  0.7977   0.8005   0.8099   0.8175   0.8314   0.8330   0.8509   0.8605
  0.8644   0.8715   0.8820   0.8947   0.9072   0.9151   0.9277   0.9362
  0.9556   0.9636   0.9682   0.9871   0.9967   1.0028   1.0273   1.0401
  1.0516   1.0732   1.0878   1.1154   1.1281   1.1338   1.1667   1.1819
  1.1940   1.1957   1.2178   1.2351   1.2621   1.2779   1.2988   1.3098
  1.3115   1.3187   1.3270   1.3374   1.3438   1.3614   1.3683   1.3752
  1.3907   1.3937   1.3949   1.3985   1.4070   1.4192   1.4323   1.4366
  1.4418   1.4491   1.4548   1.4595   1.4688   1.4792   1.4913   1.4956
  1.5027   1.5096   1.5214   1.5242   1.5308   1.5453   1.5507   1.5583
  1.5643   1.5678   1.5728   1.5782   1.5876   1.5984   1.6087   1.6154
  1.6305   1.6323   1.6389   1.6580   1.6680   1.6710   1.6801   1.6861
  1.6953   1.7104   1.7254   1.7294   1.7400   1.7489   1.7565   1.7799
  1.7933   1.7974   1.8100   1.8195   1.8231   1.8494   1.8639   1.8717
  1.8848   1.9080   1.9129   1.9336   1.9507   1.9615   1.9847   1.9935
  2.0098   2.0145   2.0330   2.0482   2.0522   2.0711   2.0768   2.0962
  2.1105   2.1233   2.1284   2.1418   2.1710   2.1921   2.2257   2.2268
  2.2356   2.2469   2.2508   2.2583   2.2610   2.2718   2.2854   2.2889
  2.2896   2.3160   2.3418   2.3588   2.3634   2.3682   2.3918   2.3995
  2.4124   2.4197   2.4208   2.4376   2.4441   2.4587   2.4658   2.4895
```

```
 2.5021   2.5140   2.5245   2.5316   2.5446   2.5461   2.5580   2.5651
 2.5760   2.5793   2.5863   2.5908   2.5959   2.5990   2.6144   2.6265
 2.6369   2.6483   2.6636   2.6700   2.6755   2.6842   2.6907   2.7039
 2.7128   2.7306   2.7423   2.7521   2.7559   2.7648   2.7804   2.7861
 2.7930   2.8084   2.8297   2.8345   2.8457   2.8786   2.8886   2.8950
 2.9150   2.9465   2.9733   3.0071   3.0615   3.0969   3.1295   3.1381
 3.1788   3.2456   3.2524   3.2789   3.2932   3.3269   3.3685   3.4079
 3.4334   3.4803   3.5258   3.5482   3.5493   3.6014   3.6057   3.6447
 3.6676   3.6914   3.7083   3.7291   3.7343   3.7461   3.7554   3.7694
 3.7841   3.8382   3.8694   3.9092   3.9332   3.9784   4.0282   4.0817
 4.1128   4.2068   4.2959   4.3166   4.6045   4.6658   4.6964   4.7217
 4.9236   5.0382   5.2018  23.5220  23.5771  23.7180  23.7281  23.7436
23.7753  23.8013  23.8296  23.8587  23.8882  23.9165  23.9674  23.9777
24.0360  35.4182  35.4561  35.5232  35.6214
--------------------------------------------------------------

         Ground-State Mulliken Net Atomic Charges

     Atom                 Charge (a.u.)
  ----------------------------------------
      1 N                     0.329439
      2 C                     0.137404
      3 C                    -0.001104
      4 C                    -0.159214
      5 C                    -0.148082
      6 C                    -0.125472
      7 C                    -0.756019
      8 C                    -0.293211
      9 N                    -0.138295
     10 C                    -0.295717
     11 N                     0.009692
     12 C                     0.302581
     13 C                    -0.240864
     14 C                     0.153636
     15 C                    -0.339626
     16 C                    -0.273099
     17 C                    -0.518620
     18 N                    -0.495256
     19 H                     0.221609
     20 H                     0.235432
     21 H                     0.227881
     22 H                     0.236550
     23 H                     0.232183
     24 H                     0.226777
     25 H                     0.217701
     26 H                     0.252486
     27 H                     0.257858
     28 H                     0.395102
     29 H                     0.216427
     30 H                     0.250242
     31 H                     0.209540
     32 H                     0.223843
     33 H                     0.206379
     34 H                     0.176895
     35 H                     0.216924
     36 H                     0.139962
     37 H                     0.330311
     38 H                     0.377724
  ----------------------------------------
  Sum of atomic charges =     2.000000

-----------------------------------------------------------------
                    Cartesian Multipole Moments
-----------------------------------------------------------------
    Charge (ESU x 10^10)
                 9.6064
    Dipole Moment (Debye)
         X       4.9170      Y       0.0997      Z      -5.7718
       Tot       7.5829
    Quadrupole Moments (Debye-Ang)
```

```
        XX     -34.3724     XY      28.2739     YY     -67.0668
        XZ     -40.3659     YZ     -20.8294     ZZ     -47.4061
   Traceless Quadrupole Moments (Debye-Ang)
       QXX      45.7281    QYY     -52.3550    QZZ       6.6269
       QXY      84.8218    QXZ    -121.0976    QYZ     -62.4881
   Octopole Moments (Debye-Ang^2)
       XXX      35.6884    XXY      27.3546    XYY      -2.3805
       YYY     -21.1269    XXZ       8.9954    XYZ     -21.8397
       YYZ      24.9788    XZZ      40.8016    YZZ      25.7882
       ZZZ     -75.6866
   Traceless Octopole Moments (Debye-Ang^2)
       XXX    -131.6596    YYY    -605.0468    ZZZ    -759.8876
       XXY     314.2711    XXZ     260.0682    XYY    -258.0359
       XYZ    -327.5957    XZZ     389.6955    YYZ     499.8194
       YZZ     290.7757
   Hexadecapole Moments (Debye-Ang^3)
      XXXX   -2775.8786   XXXY    -226.8155   XXYY    -590.4821
      XYYY    -537.9155   YYYY    -828.8120   XXXZ     798.3230
      XXYZ     -65.3091   XYYZ     309.9761   YYYZ     447.5288
      XXZZ    -715.3905   XYZZ      15.3291   YYZZ    -600.6123
      XZZZ     715.9212   YZZZ     249.8575   ZZZZ   -1990.6271
   Traceless Hexadecapole Moments (Debye-Ang^3)
      XXXX   -8784.2327   XXXY    9907.4552   XXXZ    1734.0012
      XXYY    1299.3811   XXYZ  -16325.7494   XXZZ    7484.8516
      XYYY  -22758.0417   XYYZ    5184.1826   XYZZ   12850.5865
      XZZZ   -6918.1838   YYYY   10091.7269   YYYZ   18585.6355
      YYZZ  -11391.1079   YZZZ   -2259.8861   ZZZZ    3906.2563
 -----------------------------------------------------------------

-------------------------------------------------
-  Entering drvman on Sun Oct 13 20:25:03 2024  -
-------------------------------------------------

 Calculating analytic gradient of the SCF energy
 Gradient of SCF Energy
           1           2           3           4           5           6
    1  -0.0001660   0.0000473   0.0001119  -0.0000576  -0.0001671   0.0002146
    2   0.0001163  -0.0000587   0.0000339  -0.0000812  -0.0002188   0.0001157
    3   0.0000784  -0.0003333   0.0003839   0.0001714   0.0002125  -0.0003772
           7           8           9          10          11          12
    1   0.0000512   0.0001948   0.0000134   0.0001291   0.0001669  -0.0002972
    2   0.0003091  -0.0000796   0.0001474  -0.0006334   0.0005851  -0.0011723
    3  -0.0000599   0.0003051  -0.0000962   0.0000254   0.0000919  -0.0003575
          13          14          15          16          17          18
    1  -0.0003506  -0.0001464   0.0000112  -0.0000401  -0.0000015   0.0008922
    2   0.0004400  -0.0002872   0.0000141   0.0000199   0.0000271   0.0007254
    3  -0.0001461   0.0000497   0.0001932  -0.0001362   0.0000255   0.0011662
          19          20          21          22          23          24
    1  -0.0000274  -0.0000077   0.0000217  -0.0000346   0.0000233   0.0000468
    2   0.0000418   0.0000226  -0.0000249   0.0000330  -0.0000308  -0.0000614
    3   0.0000020  -0.0000421  -0.0000041  -0.0000863   0.0000056  -0.0000257
          25          26          27          28          29          30
    1  -0.0002142  -0.0004271   0.0001389  -0.0000482  -0.0000039   0.0000060
    2   0.0000695  -0.0001192   0.0000271  -0.0000363  -0.0000486   0.0000170
    3  -0.0000182   0.0001009  -0.0001316   0.0000039   0.0000424  -0.0000245
          31          32          33          34          35          36
    1  -0.0000412   0.0000037   0.0000030  -0.0000005   0.0000059   0.0000031
    2   0.0000126  -0.0000187   0.0000132   0.0000122   0.0000183   0.0000062
    3  -0.0000057   0.0000530   0.0000185  -0.0000128   0.0000114  -0.0000350
          37          38
    1   0.0002675  -0.0003212
    2  -0.0000241   0.0000877
    3  -0.0007599  -0.0002885
 Max gradient component =       1.172E-03
 RMS gradient           =       2.601E-04
 Gradient time:  CPU 147.64 s  wall 243.60 s

-------------------------------------------------
-  Entering optman on Sun Oct 13 20:29:06 2024  -
-------------------------------------------------
```

```
 Geometry Optimization Parameters
   NAtoms,    NIC,     NZ,  NCons,   NDum,   NFix, NCnnct, MaxDiis
       38     272       0       0       0       0       0       0

 Cartesian Hessian Update
 Hessian updated using BFGS update


** GEOMETRY OPTIMIZATION IN DELOCALIZED INTERNAL COORDINATES **
   Searching for a Minimum

   Optimization Cycle:  11

                    Coordinates (Angstroms)
     ATOM              X               Y               Z
      1  N         1.9560449810    0.4381770535   -1.5598313292
      2  C         3.0516384745    1.2212126913   -1.7863201062
      3  C         3.2967262112    1.6458137493   -3.0851181644
      4  C         2.4681529757    1.2706671866   -4.1294616948
      5  C         1.3721820150    0.4523880480   -3.8674686733
      6  C         1.1461483821    0.0517255531   -2.5769624174
      7  C         3.9591534459    1.5993066706   -0.6577250733
      8  C         1.7002635044   -0.0927331837   -0.1847771858
      9  N        -1.4267053838   -1.8688921485    0.8324243873
     10  C        -2.3249124685   -0.8532071158    0.9593146092
     11  N        -1.9787729291    0.3766063526    0.6782872704
     12  C        -0.7282766720    0.6509542060    0.2728358632
     13  C         0.2595616563   -0.3830736119    0.1258235756
     14  C        -0.1540227911   -1.6437377131    0.4329466877
     15  C        -3.6867280419   -1.1553586173    1.4768109591
     16  C        -3.7966889427   -0.8027490992    2.9790085219
     17  C        -2.8602106641   -1.6158581963    3.8653780688
     18  N        -0.4622339194    1.9294916741    0.0269935063
     19  H         4.1605576951    2.2722458300   -3.2665818858
     20  H         2.6757431422    1.6065978617   -5.1388265642
     21  H         0.7037903016    0.1244150660   -4.6524583567
     22  H         0.3152467510   -0.5868093813   -2.3112603963
     23  H         4.8127389152    2.1491307393   -1.0493598065
     24  H         4.3419829267    0.7214804208   -0.1311167351
     25  H         3.4592597575    2.2477094429    0.0689595637
     26  H         2.0696109155    0.6455868815    0.5265333309
     27  H         2.3082478256   -0.9914856897   -0.0629915298
     28  H        -1.7163004652   -2.8073935185    1.0820907674
     29  H         0.4978988946   -2.5085791118    0.3888651340
     30  H        -4.3965225464   -0.5563677766    0.9036921255
     31  H        -3.9251925771   -2.2116934931    1.3207915903
     32  H        -4.8344509375   -0.9830160793    3.2657529458
     33  H        -3.6172210476    0.2682506732    3.1032667206
     34  H        -3.0238375518   -2.6912149538    3.7416212038
     35  H        -3.0335030231   -1.3834465142    4.9167340819
     36  H        -1.8046122016   -1.3991120300    3.6641383075
     37  H         0.4196099040    2.2579518781   -0.3287027577
     38  H        -1.2043665121    2.6050162555    0.1566934557
   Point Group: c1    Number of degrees of freedom:   108


   Energy is   -763.970612714

 Hessian updated using BFGS update
  internal optimization (0)

108 Hessian modes will be used to form the next step
  Hessian Eigenvalues:
     0.001227    0.002246    0.002653    0.004101    0.006208    0.012956
     0.013448    0.018700    0.019553    0.019681    0.020777    0.022148
     0.022606    0.023984    0.024091    0.024642    0.024838    0.025912
     0.026978    0.027013    0.028223    0.029282    0.034069    0.036504
     0.038632    0.039838    0.042729    0.043577    0.044204    0.045409
     0.046049    0.050635    0.054187    0.055819    0.062182    0.079899
```

| | | | | | |
|---|---|---|---|---|---|
| 0.085594 | 0.095619 | 0.121759 | 0.122691 | 0.127139 | 0.130701 |
| 0.132283 | 0.132811 | 0.137060 | 0.141214 | 0.143594 | 0.145217 |
| 0.146682 | 0.147691 | 0.148653 | 0.152057 | 0.152938 | 0.153399 |
| 0.160186 | 0.170367 | 0.192645 | 0.206294 | 0.209373 | 0.214465 |
| 0.215392 | 0.228859 | 0.237611 | 0.247692 | 0.250633 | 0.255287 |
| 0.267744 | 0.271514 | 0.278377 | 0.286941 | 0.298963 | 0.300160 |
| 0.300616 | 0.301208 | 0.301828 | 0.303170 | 0.304016 | 0.304899 |
| 0.305039 | 0.305268 | 0.306367 | 0.307431 | 0.312379 | 0.313812 |
| 0.324894 | 0.332649 | 0.334205 | 0.336391 | 0.339112 | 0.341378 |
| 0.351051 | 0.357529 | 0.365033 | 0.371421 | 0.379866 | 0.390411 |
| 0.399665 | 0.404665 | 0.412574 | 0.418395 | 0.421555 | 0.438021 |
| 0.444305 | 0.453197 | 0.511980 | 0.527583 | 0.597479 | 0.736116 |

Minimum search - taking simple RFO step
Searching for Lamda that Minimizes Along All modes
Value Taken    Lamda =  -0.00017180
Step Taken.  Stepsize is  0.249231

| | Maximum | Tolerance | Cnvgd? |
|---|---|---|---|
| Gradient | 0.001710 | 0.000800 | NO |
| Displacement | 0.093414 | 0.001400 | NO |
| Energy change | -0.000080 | 0.000228 | YES |

New Cartesian Coordinates Obtained by Inverse Iteration

Displacement from previous Coordinates is:  0.937972

Standard Nuclear Orientation (Angstroms)

| I | Atom | X | Y | Z |
|---|---|---|---|---|
| 1 | N | 1.9463295493 | 0.4790703683 | -1.5551942850 |
| 2 | C | 3.0859225783 | 1.1924518364 | -1.7947554183 |
| 3 | C | 3.3535813212 | 1.5841403289 | -3.0993613373 |
| 4 | C | 2.4995245395 | 1.2484718586 | -4.1366811398 |
| 5 | C | 1.3546225524 | 0.5057202413 | -3.8601348186 |
| 6 | C | 1.1095481461 | 0.1323737434 | -2.5645253811 |
| 7 | C | 4.0133346672 | 1.5304897067 | -0.6705383748 |
| 8 | C | 1.6767295183 | -0.0129688423 | -0.1684787320 |
| 9 | N | -1.4112879095 | -1.8544081557 | 0.8465170351 |
| 10 | C | -2.3342190278 | -0.8597575233 | 0.9602552646 |
| 11 | N | -2.0182086743 | 0.3748396811 | 0.6644271248 |
| 12 | C | -0.7739752684 | 0.6742162150 | 0.2570982616 |
| 13 | C | 0.2409126102 | -0.3354737126 | 0.1316570782 |
| 14 | C | -0.1421861970 | -1.6025876984 | 0.4524145097 |
| 15 | C | -3.6877601439 | -1.1896492934 | 1.4821604928 |
| 16 | C | -3.7990953233 | -0.8430335859 | 2.9858782468 |
| 17 | C | -2.8497042113 | -1.6447709322 | 3.8689984715 |
| 18 | N | -0.5410657081 | 1.9547709211 | -0.0145106237 |
| 19 | H | 4.2551338811 | 2.1516596435 | -3.2911628623 |
| 20 | H | 2.7250072485 | 1.5561955812 | -5.1511830448 |
| 21 | H | 0.6633533024 | 0.2123651055 | -4.6390104190 |
| 22 | H | 0.2429764290 | -0.4530761024 | -2.2906579707 |
| 23 | H | 4.9094567004 | 2.0019646461 | -1.0692748483 |
| 24 | H | 4.3236195800 | 0.6427311988 | -0.1141535396 |
| 25 | H | 3.5573105860 | 2.2361239828 | 0.0311866256 |
| 26 | H | 2.0143930028 | 0.7588032380 | 0.5240424567 |
| 27 | H | 2.3065652722 | -0.8899046924 | -0.0061256078 |
| 28 | H | -1.6792473405 | -2.7966816246 | 1.1063746694 |
| 29 | H | 0.5308859117 | -2.4517323211 | 0.4227143301 |
| 30 | H | -4.4118933500 | -0.6042619731 | 0.9132290956 |
| 31 | H | -3.9056029349 | -2.2504172093 | 1.3253995449 |
| 32 | H | -4.8335564778 | -1.0391287354 | 3.2741864566 |
| 33 | H | -3.6351579603 | 0.2301521364 | 3.1132628587 |
| 34 | H | -2.9966789367 | -2.7220677786 | 3.7412512835 |
| 35 | H | -3.0267168566 | -1.4189044941 | 4.9211815784 |
| 36 | H | -1.7975299433 | -1.4108094456 | 3.6691594737 |
| 37 | H | 0.3382450100 | 2.3016803541 | -0.3594418752 |
| 38 | H | -1.3035661427 | 2.6114133331 | 0.0937954199 |

```
 Nuclear Repulsion Energy =        1306.53027798 hartrees
 There are       65 alpha and       65 beta electrons

 --------------------------------------------------
 -  Entering fldman on Sun Oct 13 20:29:06 2024  -
 --------------------------------------------------

 Applying Cartesian multipole field
    Component          Value
    ---------          -----
     (2,0,0)        1.00000E-11
     (0,2,0)        2.00000E-11
     (0,0,2)       -3.00000E-11
 Nucleus-field energy     =    -0.0000000245 hartrees

 --------------------------------------------------
 -  Entering gesman on Sun Oct 13 20:29:06 2024  -
 --------------------------------------------------

 Requested basis set is 6-311+G(d,p)
 There are 188 shells and 516 basis functions
 A cutoff of  1.0D-12 yielded  12583 shell pairs
 There are    100506 function pairs (    107163 Cartesian)
 Smallest overlap matrix eigenvalue = 1.37E-06
 Linear dependence detected in AO basis
 Tighter screening thresholds may be required for diffuse basis sets
 Use S2THRESH > 12 and THRESH = 14 in case of SCF convergence issues
 Number of orthogonalized atomic orbitals = 510
 Maximum deviation from orthogonality = 2.718E-11
 Guess MOs from SCF MO coefficient file
 Reading MOs from coefficient file
 Reading MOs from coefficient file

 --------------------------------------------------
 -  Entering scfman on Sun Oct 13 20:29:07 2024  -
 --------------------------------------------------

 Long-range K will be added via erf
 Coulomb attenuation parameter = 0.2 bohr**(-1)
 A restricted hybrid HF-DFT SCF calculation will be
 performed using Pulay DIIS + Geometric Direct Minimization
 Exchange:     0.2220 Hartree-Fock + 1.0000 wB97X-D + LR-HF
 Correlation:  1.0000 wB97X-D
 Using Euler-Maclaurin-Lebedev (75,302) quadrature formula
 Dispersion:   Grimme D
 SCF converges when RMS gradient is below 1.0E-07
 Geometry optimization detected.  Setting ReadMinima to 0
 Setting SaveMinima to 0
 ---------------------------------------
  Cycle       Energy         DIIS Error
 ---------------------------------------
    1    -763.9429479363      8.27E-04
    2    -763.9697401339      1.09E-04
    3    -763.9705248196      7.34E-05
    4    -763.9706848701      3.41E-05
    5    -763.9707295837      1.05E-05
    6    -763.9707339246      3.39E-06
    7    -763.9707343553      9.59E-07
    8    -763.9707344037      3.60E-07
    9    -763.9707344091      8.96E-08 Convergence criterion met
 ---------------------------------------
 SCF time:  CPU 317.05 s  wall 526.74 s
 SCF   energy in the final basis set = -763.97073441
 Total energy in the final basis set = -763.97073441

 --------------------------------------------------
 -  Entering anlman on Sun Oct 13 20:37:54 2024  -
 --------------------------------------------------
```

---

Orbital Energies (a.u.)

---

```
 Alpha MOs
 -- Occupied --
-14.8174 -14.7897 -14.7421 -14.7080 -10.6778 -10.6628 -10.6480 -10.6390
-10.6353 -10.6264 -10.6057 -10.5837 -10.5733 -10.5708 -10.5465 -10.5259
-10.4941 -10.4695  -1.4158  -1.4044  -1.3496  -1.2775  -1.2307  -1.2016
 -1.1872  -1.1426  -1.1004  -1.0821  -1.0566  -1.0546  -1.0286  -1.0066
 -0.9906  -0.9567  -0.9437  -0.9176  -0.9099  -0.8888  -0.8811  -0.8669
 -0.8598  -0.8421  -0.8404  -0.8208  -0.8081  -0.7982  -0.7884  -0.7766
 -0.7756  -0.7589  -0.7504  -0.7470  -0.7442  -0.7288  -0.7205  -0.7158
 -0.6999  -0.6693  -0.6575  -0.6439  -0.6360  -0.6272  -0.6230  -0.6211
 -0.6135
 -- Virtual --
 -0.2695  -0.2581  -0.2449  -0.2258  -0.1771  -0.1623  -0.1489  -0.1429
 -0.1362  -0.1207  -0.1141  -0.1110  -0.1092  -0.1013  -0.0980  -0.0959
 -0.0897  -0.0862  -0.0816  -0.0772  -0.0747  -0.0732  -0.0698  -0.0674
 -0.0636  -0.0563  -0.0556  -0.0529  -0.0497  -0.0443  -0.0412  -0.0385
 -0.0308  -0.0295  -0.0242  -0.0234  -0.0174  -0.0147  -0.0128  -0.0062
 -0.0035  -0.0017   0.0010   0.0017   0.0073   0.0079   0.0113   0.0150
  0.0189   0.0207   0.0237   0.0293   0.0337   0.0358   0.0378   0.0411
  0.0433   0.0462   0.0507   0.0529   0.0575   0.0602   0.0635   0.0652
  0.0708   0.0744   0.0767   0.0784   0.0831   0.0860   0.0893   0.0916
  0.0934   0.1011   0.1017   0.1040   0.1089   0.1103   0.1138   0.1164
  0.1173   0.1220   0.1275   0.1299   0.1346   0.1406   0.1422   0.1499
  0.1524   0.1566   0.1575   0.1655   0.1666   0.1720   0.1746   0.1802
  0.1878   0.1914   0.1985   0.2006   0.2069   0.2125   0.2167   0.2248
  0.2394   0.2433   0.2497   0.2753   0.2856   0.2923   0.2993   0.3077
  0.3177   0.3259   0.3317   0.3413   0.3513   0.3563   0.3608   0.3654
  0.3714   0.3854   0.3887   0.3919   0.3971   0.4056   0.4186   0.4250
  0.4303   0.4376   0.4442   0.4499   0.4527   0.4633   0.4732   0.4758
  0.4879   0.4905   0.4972   0.5049   0.5100   0.5131   0.5160   0.5196
  0.5210   0.5308   0.5364   0.5386   0.5426   0.5490   0.5543   0.5566
  0.5661   0.5728   0.5743   0.5803   0.5855   0.5936   0.5949   0.6047
  0.6090   0.6138   0.6248   0.6316   0.6420   0.6493   0.6574   0.6619
  0.6686   0.6750   0.6824   0.6852   0.6940   0.7030   0.7111   0.7176
  0.7185   0.7287   0.7356   0.7466   0.7623   0.7656   0.7746   0.7906
  0.7952   0.8028   0.8113   0.8158   0.8274   0.8329   0.8472   0.8598
  0.8638   0.8699   0.8856   0.8930   0.9034   0.9178   0.9287   0.9372
  0.9544   0.9631   0.9720   0.9882   0.9951   1.0068   1.0274   1.0409
  1.0521   1.0726   1.0899   1.1142   1.1301   1.1349   1.1660   1.1812
  1.1930   1.1975   1.2173   1.2374   1.2607   1.2814   1.2970   1.3119
  1.3148   1.3195   1.3308   1.3415   1.3488   1.3614   1.3678   1.3766
  1.3902   1.3922   1.3942   1.4002   1.4061   1.4204   1.4306   1.4356
  1.4414   1.4491   1.4532   1.4624   1.4669   1.4792   1.4904   1.4976
  1.5046   1.5103   1.5163   1.5239   1.5297   1.5442   1.5511   1.5590
  1.5639   1.5694   1.5730   1.5766   1.5879   1.5983   1.6109   1.6152
  1.6297   1.6318   1.6380   1.6582   1.6687   1.6709   1.6796   1.6846
  1.6961   1.7153   1.7263   1.7304   1.7417   1.7524   1.7566   1.7801
  1.7926   1.7993   1.8094   1.8200   1.8245   1.8489   1.8653   1.8722
  1.8846   1.9103   1.9159   1.9328   1.9483   1.9621   1.9853   1.9982
  2.0092   2.0103   2.0320   2.0438   2.0512   2.0723   2.0773   2.0966
  2.1106   2.1221   2.1277   2.1407   2.1704   2.1940   2.2263   2.2266
  2.2348   2.2447   2.2499   2.2580   2.2608   2.2733   2.2864   2.2901
  2.2916   2.3175   2.3427   2.3590   2.3632   2.3734   2.3924   2.3993
  2.4145   2.4186   2.4227   2.4376   2.4420   2.4568   2.4655   2.4892
  2.5034   2.5142   2.5219   2.5314   2.5436   2.5463   2.5557   2.5647
  2.5734   2.5792   2.5839   2.5915   2.5950   2.5992   2.6128   2.6273
  2.6371   2.6470   2.6598   2.6722   2.6747   2.6840   2.6905   2.7041
  2.7119   2.7295   2.7409   2.7494   2.7586   2.7658   2.7796   2.7848
  2.7982   2.8119   2.8337   2.8357   2.8453   2.8791   2.8849   2.8931
  2.9088   2.9480   2.9756   3.0091   3.0628   3.0887   3.1298   3.1395
  3.1789   3.2454   3.2520   3.2778   3.2968   3.3244   3.3671   3.4093
  3.4309   3.4812   3.5254   3.5473   3.5526   3.6007   3.6039   3.6436
  3.6679   3.6915   3.7076   3.7297   3.7336   3.7461   3.7556   3.7683
  3.7867   3.8369   3.8694   3.9091   3.9333   3.9778   4.0283   4.0819
  4.1136   4.2065   4.2965   4.3162   4.6030   4.6653   4.6976   4.7214
```

```
 4.9231   5.0395   5.2013  23.5212  23.5785  23.7182  23.7288  23.7446
23.7760  23.8013  23.8323  23.8583  23.8882  23.9159  23.9675  23.9769
24.0359  35.4196  35.4571  35.5229  35.6206
--------------------------------------------------------------

         Ground-State Mulliken Net Atomic Charges

     Atom                 Charge (a.u.)
  ----------------------------------------
      1 N                    0.316149
      2 C                    0.160958
      3 C                   -0.006674
      4 C                   -0.167298
      5 C                   -0.145930
      6 C                   -0.125277
      7 C                   -0.750441
      8 C                   -0.294532
      9 N                   -0.143805
     10 C                   -0.284334
     11 N                    0.006214
     12 C                    0.305414
     13 C                   -0.261479
     14 C                    0.155773
     15 C                   -0.334039
     16 C                   -0.274409
     17 C                   -0.520346
     18 N                   -0.494335
     19 H                    0.221500
     20 H                    0.235169
     21 H                    0.227683
     22 H                    0.239122
     23 H                    0.232288
     24 H                    0.220328
     25 H                    0.224500
     26 H                    0.254324
     27 H                    0.256606
     28 H                    0.396329
     29 H                    0.215676
     30 H                    0.249797
     31 H                    0.210956
     32 H                    0.224090
     33 H                    0.206451
     34 H                    0.176893
     35 H                    0.217245
     36 H                    0.139695
     37 H                    0.332634
     38 H                    0.377104
  ----------------------------------------
  Sum of atomic charges =     2.000000

-----------------------------------------------------------------
                   Cartesian Multipole Moments
-----------------------------------------------------------------
   Charge (ESU x 10^10)
                9.6064
   Dipole Moment (Debye)
        X       4.8904      Y       0.2198      Z      -5.7071
      Tot       7.5190
   Quadrupole Moments (Debye-Ang)
       XX     -33.3625     XY      27.7994     YY     -67.8909
       XZ     -40.5610     YZ     -21.4131     ZZ     -47.3091
   Traceless Quadrupole Moments (Debye-Ang)
      QXX      48.4751    QYY     -55.1102    QZZ       6.6351
      QXY      83.3981    QXZ    -121.6830    QYZ     -64.2392
   Octopole Moments (Debye-Ang^2)
      XXX      44.0037    XXY      26.3352    XYY      -5.8749
      YYY     -23.1508    XXZ       5.4924    XYZ     -20.4204
      YYZ      27.5522    XZZ      42.2942    YZZ      24.6043
      ZZZ     -74.7273
   Traceless Octopole Moments (Debye-Ang^2)
```

```
       XXX     -63.7506    YYY    -597.3604    ZZZ    -745.7652
       XXY     311.6613    XXZ     207.4339    XYY    -329.3928
       XYZ    -306.3056    XZZ     393.1434    YYZ     538.3313
       YZZ     285.6991
    Hexadecapole Moments (Debye-Ang^3)
      XXXX   -2776.4747   XXXY    -223.3145   XXYY    -603.3728
      XYYY    -551.4433   YYYY    -824.2381   XXXZ     780.4833
      XXYZ     -64.1950   XYYZ     318.8359   YYYZ     462.1537
      XXZZ    -707.6780   XYZZ      16.0028   YYZZ    -605.9265
      XZZZ     711.9684   YZZZ     257.6258   ZZZZ   -1994.3511
    Traceless Hexadecapole Moments (Debye-Ang^3)
      XXXX   -8513.7157   XXXY   10695.9512   XXXZ     442.8008
      XXYY     174.7428   XXYZ  -16564.8552   XXZZ    8338.9729
      XYYY  -23757.5705   XYYZ    6308.4583   XYZZ   13061.6193
      XZZZ   -6751.2591   YYYY   11612.1963   YYYZ   19052.9982
      YYZZ  -11786.9391   YZZZ   -2488.1429   ZZZZ    3447.9662
 -----------------------------------------------------------------

 --------------------------------------------------
-  Entering drvman on Sun Oct 13 20:37:54 2024  -
 --------------------------------------------------

 Calculating analytic gradient of the SCF energy
 Gradient of SCF Energy
           1           2           3           4           5           6
    1  -0.0004342   0.0000111   0.0000684  -0.0000109  -0.0001232   0.0002171
    2   0.0001540  -0.0000444   0.0001274  -0.0001369   0.0000227   0.0000403
    3   0.0001895  -0.0001353   0.0003435  -0.0000788   0.0003225  -0.0003176
           7           8           9          10          11          12
    1  -0.0000358   0.0004886  -0.0001215   0.0003629   0.0001961  -0.0003073
    2   0.0001881   0.0000539   0.0004981  -0.0010567   0.0008037  -0.0022448
    3   0.0000897   0.0003030  -0.0002241   0.0000235   0.0002971  -0.0002596
          13          14          15          16          17          18
    1  -0.0007662   0.0000278   0.0000788  -0.0000694  -0.0000330   0.0011230
    2   0.0006021  -0.0003234   0.0001406  -0.0000612  -0.0000151   0.0015780
    3  -0.0002435   0.0001192   0.0002086  -0.0002003   0.0000756   0.0009376
          19          20          21          22          23          24
    1   0.0000098   0.0000499   0.0000307  -0.0001001   0.0000347  -0.0000347
    2  -0.0000168  -0.0000203   0.0000194   0.0000271  -0.0000647  -0.0000603
    3  -0.0000680  -0.0000574  -0.0000405  -0.0001461   0.0000391  -0.0000231
          25          26          27          28          29          30
    1  -0.0002610  -0.0003649   0.0002137  -0.0001204  -0.0000289  -0.0000199
    2  -0.0000075  -0.0000835   0.0000900  -0.0000823  -0.0001292  -0.0000293
    3  -0.0001652   0.0001597  -0.0001881   0.0000302   0.0000734  -0.0000232
          31          32          33          34          35          36
    1  -0.0000525   0.0000075   0.0000281   0.0000086   0.0000079   0.0000218
    2  -0.0000124  -0.0000016   0.0000247   0.0000246   0.0000306   0.0000053
    3   0.0000213   0.0000791   0.0000475  -0.0000368   0.0000189  -0.0000169
          37          38
    1   0.0005003  -0.0006031
    2  -0.0000669   0.0000267
    3  -0.0006457  -0.0005088
 Max gradient component =       2.245E-03
 RMS gradient           =       3.778E-04
 Gradient time:  CPU 149.31 s  wall 242.76 s

 --------------------------------------------------
-  Entering optman on Sun Oct 13 20:41:56 2024  -
 --------------------------------------------------

 Geometry Optimization Parameters
   NAtoms,    NIC,     NZ,  NCons,   NDum,   NFix, NCnnct, MaxDiis
       38     272       0       0       0       0       0       0

 Cartesian Hessian Update
 Hessian updated using BFGS update


** GEOMETRY OPTIMIZATION IN DELOCALIZED INTERNAL COORDINATES **
   Searching for a Minimum
```

Optimization Cycle: 12

Coordinates (Angstroms)

| ATOM | | X | Y | Z |
|---|---|---|---|---|
| 1 | N | 1.9463295493 | 0.4790703683 | -1.5551942850 |
| 2 | C | 3.0859225783 | 1.1924518364 | -1.7947554183 |
| 3 | C | 3.3535813212 | 1.5841403289 | -3.0993613373 |
| 4 | C | 2.4995245395 | 1.2484718586 | -4.1366811398 |
| 5 | C | 1.3546225524 | 0.5057202413 | -3.8601348186 |
| 6 | C | 1.1095481461 | 0.1323737434 | -2.5645253811 |
| 7 | C | 4.0133346672 | 1.5304897067 | -0.6705383748 |
| 8 | C | 1.6767295183 | -0.0129688423 | -0.1684787320 |
| 9 | N | -1.4112879095 | -1.8544081557 | 0.8465170351 |
| 10 | C | -2.3342190278 | -0.8597575233 | 0.9602552646 |
| 11 | N | -2.0182086743 | 0.3748396811 | 0.6644271248 |
| 12 | C | -0.7739752684 | 0.6742162150 | 0.2570982616 |
| 13 | C | 0.2409126102 | -0.3354737126 | 0.1316570782 |
| 14 | C | -0.1421861970 | -1.6025876984 | 0.4524145097 |
| 15 | C | -3.6877601439 | -1.1896492934 | 1.4821604928 |
| 16 | C | -3.7990953233 | -0.8430335859 | 2.9858782468 |
| 17 | C | -2.8497042113 | -1.6447709322 | 3.8689984715 |
| 18 | N | -0.5410657081 | 1.9547709211 | -0.0145106237 |
| 19 | H | 4.2551338811 | 2.1516596435 | -3.2911628623 |
| 20 | H | 2.7250072485 | 1.5561955812 | -5.1511830448 |
| 21 | H | 0.6633533024 | 0.2123651055 | -4.6390104190 |
| 22 | H | 0.2429764290 | -0.4530761024 | -2.2906579707 |
| 23 | H | 4.9094567004 | 2.0019646461 | -1.0692748483 |
| 24 | H | 4.3236195800 | 0.6427311988 | -0.1141535396 |
| 25 | H | 3.5573105860 | 2.2361239828 | 0.0311866256 |
| 26 | H | 2.0143930028 | 0.7588032380 | 0.5240424567 |
| 27 | H | 2.3065652722 | -0.8899046924 | -0.0061256078 |
| 28 | H | -1.6792473405 | -2.7966816246 | 1.1063746694 |
| 29 | H | 0.5308859117 | -2.4517323211 | 0.4227143301 |
| 30 | H | -4.4118933500 | -0.6042619731 | 0.9132290956 |
| 31 | H | -3.9056029349 | -2.2504172093 | 1.3253995449 |
| 32 | H | -4.8335564778 | -1.0391287354 | 3.2741864566 |
| 33 | H | -3.6351579603 | 0.2301521364 | 3.1132628587 |
| 34 | H | -2.9966789367 | -2.7220677786 | 3.7412512835 |
| 35 | H | -3.0267168566 | -1.4189044941 | 4.9211815784 |
| 36 | H | -1.7975299433 | -1.4108094456 | 3.6691594737 |
| 37 | H | 0.3382450100 | 2.3016803541 | -0.3594418752 |
| 38 | H | -1.3035661427 | 2.6114133331 | 0.0937954199 |

Point Group: c1 Number of degrees of freedom: 108

Energy is -763.970734409

Hessian updated using BFGS update
internal optimization (0)

108 Hessian modes will be used to form the next step
Hessian Eigenvalues:

| | | | | | |
|---|---|---|---|---|---|
| 0.001267 | 0.002277 | 0.002651 | 0.004093 | 0.006091 | 0.010410 |
| 0.013507 | 0.018714 | 0.019432 | 0.019663 | 0.020818 | 0.022153 |
| 0.022538 | 0.024024 | 0.024190 | 0.024476 | 0.024649 | 0.025938 |
| 0.026275 | 0.027010 | 0.028325 | 0.029295 | 0.033314 | 0.036471 |
| 0.038712 | 0.040702 | 0.042796 | 0.043576 | 0.044340 | 0.044970 |
| 0.046153 | 0.050519 | 0.054172 | 0.055819 | 0.059428 | 0.079904 |
| 0.085605 | 0.095388 | 0.121760 | 0.122670 | 0.127137 | 0.130660 |
| 0.132302 | 0.132813 | 0.137517 | 0.141355 | 0.143840 | 0.145430 |
| 0.146536 | 0.147689 | 0.148734 | 0.151588 | 0.152929 | 0.153097 |
| 0.159178 | 0.170234 | 0.192494 | 0.206306 | 0.209154 | 0.213112 |
| 0.215105 | 0.228802 | 0.236528 | 0.241758 | 0.250611 | 0.254885 |
| 0.267584 | 0.271245 | 0.276626 | 0.286824 | 0.298989 | 0.300159 |
| 0.300619 | 0.301205 | 0.301825 | 0.303176 | 0.304015 | 0.304919 |
| 0.305033 | 0.305272 | 0.306588 | 0.307253 | 0.312433 | 0.313651 |
| 0.324903 | 0.332655 | 0.334214 | 0.336418 | 0.339150 | 0.341365 |
| 0.351208 | 0.357296 | 0.364504 | 0.371573 | 0.380636 | 0.390434 |
| 0.399492 | 0.404649 | 0.412111 | 0.418774 | 0.421316 | 0.438479 |

```
    0.441746    0.453064    0.502845    0.524826    0.574378    0.756229

 Minimum search - taking simple RFO step
 Searching for Lamda that Minimizes Along All modes
 Value Taken    Lamda =  -0.00015219
 Step Taken.  Stepsize is  0.173328

                          Maximum     Tolerance    Cnvgd?
        Gradient         0.002435      0.000800      NO
        Displacement     0.056925      0.001400      NO
        Energy change   -0.000122      0.000228     YES


 New Cartesian Coordinates Obtained by Inverse Iteration

 Displacement from previous Coordinates is:  0.659992
 ----------------------------------------------------------------
             Standard Nuclear Orientation (Angstroms)
    I     Atom           X                Y                Z
 ----------------------------------------------------------------
    1      N       1.9434755420     0.5031136355    -1.5501640606
    2      C       3.1080512287     1.1697151590    -1.8036247819
    3      C       3.3804974433     1.5423611478    -3.1122688967
    4      C       2.5038925646     1.2352298493    -4.1397191776
    5      C       1.3332541356     0.5395464482    -3.8494170045
    6      C       1.0853427727     0.1829177054    -2.5499391727
    7      C       4.0567618750     1.4758034517    -0.6883927220
    8      C       1.6684367603     0.0437610399    -0.1535963991
    9      N      -1.3915594872    -1.8401043490     0.8647484011
   10      C      -2.3354127946    -0.8627933871     0.9585794593
   11      N      -2.0413508403     0.3739723319     0.6500600098
   12      C      -0.7992479525     0.6943536672     0.2511597136
   13      C       0.2363709297    -0.2965600531     0.1455941753
   14      C      -0.1249277505    -1.5674532328     0.4777425145
   15      C      -3.6852180390    -1.2150936430     1.4751690607
   16      C      -3.8074344657    -0.8727335809     2.9793463138
   17      C      -2.8522570109    -1.6632490816     3.8664233804
   18      N      -0.5944649283     1.9743176718    -0.0404810771
   19      H       4.3026722684     2.0716505654    -3.3151347797
   20      H       2.7327364950     1.5273365033    -5.1581477404
   21      H       0.6242811598     0.2696832643    -4.6207044874
   22      H       0.2006937537    -0.3697478837    -2.2660698403
   23      H       4.9742094348     1.8953343110    -1.0968605073
   24      H       4.3238952141     0.5824368222    -0.1189961491
   25      H       3.6403747216     2.2138179968     0.0047315817
   26      H       1.9924918934     0.8388637866     0.5197549357
   27      H       2.3086283039    -0.8196661442     0.0374283634
   28      H      -1.6426935681    -2.7843444351     1.1340224347
   29      H       0.5643279150    -2.4038351557     0.4609231602
   30      H      -4.4171702318    -0.6412524417     0.9047901324
   31      H      -3.8847156728    -2.2792767524     1.3168229344
   32      H      -4.8406671442    -1.0826564898     3.2622608693
   33      H      -3.6582826624     0.2023114156     3.1094033999
   34      H      -2.9835315822    -2.7422421629     3.7356404079
   35      H      -3.0393299069    -1.4422979514     4.9179176413
   36      H      -1.8021368267    -1.4141985855     3.6744978294
   37      H       0.2898766521     2.3445168486    -0.3452613596
   38      H      -1.3698701990     2.6164617082     0.0617614374
 ----------------------------------------------------------------
 Nuclear Repulsion Energy =        1306.00338579 hartrees
 There are       65 alpha and       65 beta electrons

--------------------------------------------------
-  Entering fldman on Sun Oct 13 20:41:56 2024  -
--------------------------------------------------

 Applying Cartesian multipole field
    Component           Value
    ---------           -----
     (2,0,0)       1.00000E-11
```

```
     (0,2,0)        2.00000E-11
     (0,0,2)       -3.00000E-11
 Nucleus-field energy     =    -0.0000000244 hartrees

--------------------------------------------------
-  Entering gesman on Sun Oct 13 20:41:56 2024  -
--------------------------------------------------

 Requested basis set is 6-311+G(d,p)
 There are 188 shells and 516 basis functions
 A cutoff of  1.0D-12 yielded  12578 shell pairs
 There are    100487 function pairs (    107142 Cartesian)
 Smallest overlap matrix eigenvalue = 1.18E-06
 Linear dependence detected in AO basis
 Tighter screening thresholds may be required for diffuse basis sets
 Use S2THRESH > 12 and THRESH = 14 in case of SCF convergence issues
 Number of orthogonalized atomic orbitals = 510
 Maximum deviation from orthogonality = 2.813E-11
 Guess MOs from SCF MO coefficient file
 Reading MOs from coefficient file
 Reading MOs from coefficient file

--------------------------------------------------
-  Entering scfman on Sun Oct 13 20:41:57 2024  -
--------------------------------------------------

 Long-range K will be added via erf
 Coulomb attenuation parameter = 0.2 bohr**(-1)
 A restricted hybrid HF-DFT SCF calculation will be
 performed using Pulay DIIS + Geometric Direct Minimization
 Exchange:     0.2220 Hartree-Fock + 1.0000 wB97X-D + LR-HF
 Correlation:  1.0000 wB97X-D
 Using Euler-Maclaurin-Lebedev (75,302) quadrature formula
 Dispersion:   Grimme D
 SCF converges when RMS gradient is below 1.0E-07
 Geometry optimization detected.  Setting ReadMinima to 0
 Setting SaveMinima to 0
 ---------------------------------------
  Cycle       Energy         DIIS Error
 ---------------------------------------
    1    -763.9532254049      5.88E-04
    2    -763.9703578932      7.62E-05
    3    -763.9707382148      4.85E-05
    4    -763.9708068317      2.48E-05
    5    -763.9708296154      6.98E-06
    6    -763.9708316517      2.24E-06
    7    -763.9708318445      6.53E-07
    8    -763.9708318696      2.44E-07
    9    -763.9708318723      6.93E-08 Convergence criterion met
 ---------------------------------------
 SCF time:  CPU 306.63 s  wall 504.47 s
 SCF   energy in the final basis set = -763.97083187
 Total energy in the final basis set = -763.97083187

--------------------------------------------------
-  Entering anlman on Sun Oct 13 20:50:21 2024  -
--------------------------------------------------


 --------------------------------------------------------------

                   Orbital Energies (a.u.)
 --------------------------------------------------------------

 Alpha MOs
 -- Occupied --
-14.8169 -14.7897 -14.7421 -14.7081 -10.6779 -10.6629 -10.6482 -10.6382
-10.6355 -10.6260 -10.6058 -10.5831 -10.5728 -10.5702 -10.5459 -10.5260
-10.4944 -10.4698  -1.4159  -1.4040  -1.3499  -1.2775  -1.2306  -1.2014
 -1.1868  -1.1426  -1.1003  -1.0816  -1.0568  -1.0548  -1.0280  -1.0068
```

```
-0.9902  -0.9568  -0.9438  -0.9179  -0.9098  -0.8889  -0.8807  -0.8667
-0.8598  -0.8421  -0.8401  -0.8205  -0.8080  -0.7985  -0.7881  -0.7766
-0.7745  -0.7590  -0.7502  -0.7466  -0.7443  -0.7284  -0.7206  -0.7155
-0.7002  -0.6694  -0.6569  -0.6440  -0.6363  -0.6275  -0.6232  -0.6207
-0.6134
-- Virtual --
-0.2687  -0.2581  -0.2450  -0.2253  -0.1775  -0.1623  -0.1493  -0.1429
-0.1359  -0.1206  -0.1141  -0.1104  -0.1089  -0.1017  -0.0976  -0.0954
-0.0894  -0.0857  -0.0815  -0.0779  -0.0739  -0.0728  -0.0697  -0.0671
-0.0636  -0.0561  -0.0557  -0.0526  -0.0500  -0.0437  -0.0408  -0.0388
-0.0312  -0.0289  -0.0246  -0.0238  -0.0177  -0.0146  -0.0131  -0.0060
-0.0037  -0.0016   0.0008   0.0026   0.0075   0.0081   0.0112   0.0139
 0.0198   0.0210   0.0245   0.0299   0.0334   0.0361   0.0382   0.0414
 0.0441   0.0466   0.0509   0.0525   0.0581   0.0600   0.0635   0.0658
 0.0705   0.0743   0.0771   0.0786   0.0842   0.0867   0.0880   0.0914
 0.0929   0.1002   0.1013   0.1051   0.1086   0.1115   0.1151   0.1163
 0.1181   0.1220   0.1270   0.1305   0.1346   0.1409   0.1420   0.1504
 0.1525   0.1564   0.1576   0.1645   0.1668   0.1725   0.1751   0.1794
 0.1888   0.1915   0.1991   0.2012   0.2075   0.2136   0.2180   0.2260
 0.2393   0.2420   0.2510   0.2742   0.2860   0.2926   0.3009   0.3073
 0.3166   0.3266   0.3321   0.3416   0.3521   0.3567   0.3606   0.3675
 0.3704   0.3858   0.3883   0.3929   0.3971   0.4053   0.4180   0.4243
 0.4286   0.4364   0.4448   0.4500   0.4527   0.4643   0.4716   0.4767
 0.4862   0.4904   0.4973   0.5044   0.5095   0.5131   0.5161   0.5204
 0.5217   0.5330   0.5372   0.5393   0.5417   0.5485   0.5533   0.5588
 0.5661   0.5735   0.5738   0.5804   0.5823   0.5928   0.5953   0.6050
 0.6084   0.6156   0.6241   0.6321   0.6426   0.6487   0.6571   0.6614
 0.6700   0.6761   0.6828   0.6857   0.6929   0.7030   0.7092   0.7164
 0.7194   0.7292   0.7344   0.7481   0.7635   0.7653   0.7773   0.7921
 0.7928   0.8011   0.8134   0.8149   0.8259   0.8338   0.8462   0.8602
 0.8628   0.8683   0.8882   0.8923   0.9005   0.9186   0.9294   0.9366
 0.9545   0.9636   0.9780   0.9919   0.9928   1.0095   1.0274   1.0417
 1.0523   1.0701   1.0917   1.1124   1.1314   1.1363   1.1649   1.1797
 1.1905   1.1988   1.2162   1.2379   1.2607   1.2835   1.2957   1.3117
 1.3145   1.3225   1.3333   1.3434   1.3529   1.3626   1.3674   1.3773
 1.3901   1.3925   1.3949   1.4018   1.4061   1.4207   1.4294   1.4354
 1.4410   1.4492   1.4520   1.4639   1.4658   1.4800   1.4902   1.5003
 1.5055   1.5111   1.5148   1.5237   1.5290   1.5430   1.5502   1.5598
 1.5641   1.5701   1.5730   1.5739   1.5891   1.5981   1.6127   1.6137
 1.6288   1.6309   1.6368   1.6588   1.6679   1.6730   1.6789   1.6838
 1.6969   1.7160   1.7282   1.7311   1.7438   1.7550   1.7575   1.7797
 1.7920   1.8009   1.8091   1.8202   1.8250   1.8489   1.8666   1.8719
 1.8848   1.9112   1.9186   1.9325   1.9475   1.9627   1.9853   1.9980
 2.0076   2.0094   2.0293   2.0450   2.0515   2.0738   2.0768   2.0972
 2.1110   2.1207   2.1273   2.1405   2.1701   2.1950   2.2252   2.2280
 2.2343   2.2413   2.2500   2.2565   2.2618   2.2736   2.2879   2.2912
 2.2940   2.3185   2.3434   2.3589   2.3628   2.3766   2.3928   2.3985
 2.4154   2.4171   2.4227   2.4379   2.4411   2.4551   2.4643   2.4891
 2.5039   2.5144   2.5199   2.5318   2.5423   2.5467   2.5546   2.5642
 2.5714   2.5780   2.5836   2.5918   2.5947   2.5992   2.6119   2.6268
 2.6379   2.6476   2.6558   2.6732   2.6741   2.6825   2.6906   2.7043
 2.7128   2.7286   2.7402   2.7490   2.7605   2.7681   2.7788   2.7849
 2.8041   2.8139   2.8333   2.8393   2.8464   2.8778   2.8809   2.8925
 2.9040   2.9482   2.9744   3.0099   3.0634   3.0841   3.1293   3.1411
 3.1780   3.2453   3.2523   3.2778   3.2997   3.3245   3.3662   3.4111
 3.4290   3.4811   3.5262   3.5469   3.5560   3.5983   3.6048   3.6430
 3.6679   3.6914   3.7071   3.7308   3.7332   3.7461   3.7560   3.7678
 3.7882   3.8362   3.8698   3.9087   3.9339   3.9779   4.0281   4.0818
 4.1141   4.2062   4.2973   4.3151   4.6023   4.6653   4.6987   4.7219
 4.9238   5.0411   5.2009  23.5203  23.5800  23.7184  23.7292  23.7457
23.7762  23.8015  23.8342  23.8580  23.8888  23.9155  23.9688  23.9763
24.0368  35.4210  35.4580  35.5233  35.6207
--------------------------------------------------------------

          Ground-State Mulliken Net Atomic Charges

     Atom                 Charge (a.u.)
  ----------------------------------------
      1 N                     0.312022
      2 C                     0.178136
```

```
     3  C                         -0.011236
     4  C                         -0.171571
     5  C                         -0.146707
     6  C                         -0.126595
     7  C                         -0.746500
     8  C                         -0.293110
     9  N                         -0.149295
    10  C                         -0.271948
    11  N                          0.003106
    12  C                          0.304784
    13  C                         -0.274503
    14  C                          0.155767
    15  C                         -0.330699
    16  C                         -0.274959
    17  C                         -0.522033
    18  N                         -0.492207
    19  H                          0.221406
    20  H                          0.234935
    21  H                          0.227409
    22  H                          0.240351
    23  H                          0.232411
    24  H                          0.216217
    25  H                          0.227903
    26  H                          0.253894
    27  H                          0.254711
    28  H                          0.397368
    29  H                          0.214810
    30  H                          0.249298
    31  H                          0.212199
    32  H                          0.224311
    33  H                          0.206343
    34  H                          0.176868
    35  H                          0.217538
    36  H                          0.139637
    37  H                          0.331876
    38  H                          0.378061
 ----------------------------------------
 Sum of atomic charges =      2.000000

-----------------------------------------------------------------
                    Cartesian Multipole Moments
-----------------------------------------------------------------
   Charge (ESU x 10^10)
                9.6064
   Dipole Moment (Debye)
         X       4.8925      Y       0.3358      Z      -5.5844
       Tot       7.4320
   Quadrupole Moments (Debye-Ang)
        XX     -32.7776     XY      27.3674     YY     -68.3506
        XZ     -40.5396     YZ     -21.6870     ZZ     -47.3676
   Traceless Quadrupole Moments (Debye-Ang)
      QXX      50.1630    QYY     -56.5560    QZZ       6.3930
      QXY      82.1023    QXZ    -121.6188    QYZ     -65.0610
   Octopole Moments (Debye-Ang^2)
      XXX      49.1302    XXY      25.1359    XYY      -8.0861
      YYY     -23.8187    XXZ       3.5983    XYZ     -19.3063
      YYZ      29.5742    XZZ      42.6798    YZZ      23.7497
      ZZZ     -73.9907
   Traceless Octopole Moments (Debye-Ang^2)
      XXX     -16.5615    YYY    -582.8830    ZZZ    -742.4962
      XXY     301.8381    XXZ     176.4289    XYY    -372.4636
      XYZ    -289.5944    XZZ     389.0251    YYZ     566.0673
      YZZ     281.0449
   Hexadecapole Moments (Debye-Ang^3)
     XXXX   -2776.6876   XXXY    -221.9538   XXYY    -612.9095
     XYYY    -558.6565   YYYY    -819.8094   XXXZ     768.4141
     XXYZ     -62.0065   XYYZ     323.8833   YYYZ     471.7065
     XXZZ    -704.9771   XYZZ      16.0774   YYZZ    -607.2194
     XZZZ     711.2602   YZZZ     262.2589   ZZZZ   -1995.5714
   Traceless Hexadecapole Moments (Debye-Ang^3)
```

```
     XXXX   -8021.0412   XXXY   11098.8327   XXXZ    -476.6064
     XXYY    -664.6508   XXYZ  -16586.1805   XXZZ    8685.6920
     XYYY  -24254.9518   XYYZ    6954.3804   XYZZ   13156.1190
     XZZZ   -6477.7739   YYYY   12533.9359   YYYZ   19302.6793
     YYZZ  -11869.2851   YZZZ   -2716.4988   ZZZZ    3183.5931
 -----------------------------------------------------------------

--------------------------------------------------
-  Entering drvman on Sun Oct 13 20:50:21 2024  -
--------------------------------------------------

 Calculating analytic gradient of the SCF energy
 Gradient of SCF Energy
            1           2           3           4           5           6
    1  -0.0004665  -0.0000584   0.0001746  -0.0001504  -0.0003431   0.0003682
    2   0.0001512   0.0000341   0.0001728  -0.0002115  -0.0000754   0.0001884
    3   0.0001445  -0.0003919   0.0006281   0.0001190   0.0005908  -0.0007560
            7           8           9          10          11          12
    1  -0.0001408   0.0007088  -0.0001289   0.0002198   0.0003615   0.0000362
    2  -0.0000305   0.0000044   0.0004519  -0.0010458   0.0010157  -0.0015639
    3   0.0000425   0.0002115  -0.0002172   0.0000471   0.0001556  -0.0000838
           13          14          15          16          17          18
    1  -0.0008797   0.0001144   0.0000695  -0.0000765  -0.0000109   0.0006701
    2   0.0006159  -0.0003494   0.0001450  -0.0001067  -0.0000168   0.0008340
    3  -0.0002931   0.0001271   0.0001539  -0.0001580   0.0000717   0.0003061
           19          20          21          22          23          24
    1   0.0000368   0.0000903   0.0000409  -0.0001213   0.0001001  -0.0000720
    2  -0.0000282  -0.0000627   0.0000609  -0.0000729  -0.0000540   0.0000040
    3  -0.0000551  -0.0001008  -0.0000049  -0.0000964   0.0000570   0.0000401
           25          26          27          28          29          30
    1  -0.0001506  -0.0001520   0.0001291  -0.0001121  -0.0000405  -0.0000212
    2   0.0000038   0.0000444   0.0000640  -0.0000547  -0.0001327  -0.0000669
    3  -0.0001420   0.0000863  -0.0001440   0.0000305   0.0000607   0.0000029
           31          32          33          34          35          36
    1  -0.0000375   0.0000078   0.0000413   0.0000127   0.0000049   0.0000299
    2  -0.0000304   0.0000192   0.0000195   0.0000203   0.0000267  -0.0000013
    3   0.0000455   0.0000647   0.0000500  -0.0000317   0.0000146   0.0000060
           37          38
    1   0.0001872  -0.0004414
    2   0.0000616  -0.0000338
    3  -0.0001064  -0.0004747
 Max gradient component =       1.564E-03
 RMS gradient           =       3.142E-04
 Gradient time:  CPU 146.94 s  wall 241.65 s

--------------------------------------------------
-  Entering optman on Sun Oct 13 20:54:23 2024  -
--------------------------------------------------

 Geometry Optimization Parameters
   NAtoms,    NIC,     NZ,  NCons,   NDum,   NFix, NCnnct, MaxDiis
       38     272       0       0       0       0       0       0

 Cartesian Hessian Update
 Hessian updated using BFGS update


** GEOMETRY OPTIMIZATION IN DELOCALIZED INTERNAL COORDINATES **
   Searching for a Minimum

   Optimization Cycle:  13

                    Coordinates (Angstroms)
     ATOM              X               Y               Z
      1  N         1.9434755420    0.5031136355   -1.5501640606
      2  C         3.1080512287    1.1697151590   -1.8036247819
      3  C         3.3804974433    1.5423611478   -3.1122688967
      4  C         2.5038925646    1.2352298493   -4.1397191776
      5  C         1.3332541356    0.5395464482   -3.8494170045
      6  C         1.0853427727    0.1829177054   -2.5499391727
```

| | | | | |
|---|---|---|---|---|
| 7 | C | 4.0567618750 | 1.4758034517 | -0.6883927220 |
| 8 | C | 1.6684367603 | 0.0437610399 | -0.1535963991 |
| 9 | N | -1.3915594872 | -1.8401043490 | 0.8647484011 |
| 10 | C | -2.3354127946 | -0.8627933871 | 0.9585794593 |
| 11 | N | -2.0413508403 | 0.3739723319 | 0.6500600098 |
| 12 | C | -0.7992479525 | 0.6943536672 | 0.2511597136 |
| 13 | C | 0.2363709297 | -0.2965600531 | 0.1455941753 |
| 14 | C | -0.1249277505 | -1.5674532328 | 0.4777425145 |
| 15 | C | -3.6852180390 | -1.2150936430 | 1.4751690607 |
| 16 | C | -3.8074344657 | -0.8727335809 | 2.9793463138 |
| 17 | C | -2.8522570109 | -1.6632490816 | 3.8664233804 |
| 18 | N | -0.5944649283 | 1.9743176718 | -0.0404810771 |
| 19 | H | 4.3026722684 | 2.0716505654 | -3.3151347797 |
| 20 | H | 2.7327364950 | 1.5273365033 | -5.1581477404 |
| 21 | H | 0.6242811598 | 0.2696832643 | -4.6207044874 |
| 22 | H | 0.2006937537 | -0.3697478837 | -2.2660698403 |
| 23 | H | 4.9742094348 | 1.8953343110 | -1.0968605073 |
| 24 | H | 4.3238952141 | 0.5824368222 | -0.1189961491 |
| 25 | H | 3.6403747216 | 2.2138179968 | 0.0047315817 |
| 26 | H | 1.9924918934 | 0.8388637866 | 0.5197549357 |
| 27 | H | 2.3086283039 | -0.8196661442 | 0.0374283634 |
| 28 | H | -1.6426935681 | -2.7843444351 | 1.1340224347 |
| 29 | H | 0.5643279150 | -2.4038351557 | 0.4609231602 |
| 30 | H | -4.4171702318 | -0.6412524417 | 0.9047901324 |
| 31 | H | -3.8847156728 | -2.2792767524 | 1.3168229344 |
| 32 | H | -4.8406671442 | -1.0826564898 | 3.2622608693 |
| 33 | H | -3.6582826624 | 0.2023114156 | 3.1094033999 |
| 34 | H | -2.9835315822 | -2.7422421629 | 3.7356404079 |
| 35 | H | -3.0393299069 | -1.4422979514 | 4.9179176413 |
| 36 | H | -1.8021368267 | -1.4141985855 | 3.6744978294 |
| 37 | H | 0.2898766521 | 2.3445168486 | -0.3452613596 |

***Abridged***Abridged***Abridged***

| | | | | | | | |
|---|---|---|---|---|---|---|---|
| 0.0436 | 0.0467 | 0.0529 | 0.0551 | 0.0597 | 0.0601 | 0.0645 | 0.0684 |
| 0.0707 | 0.0718 | 0.0760 | 0.0797 | 0.0848 | 0.0864 | 0.0884 | 0.0922 |
| 0.0957 | 0.1008 | 0.1016 | 0.1086 | 0.1115 | 0.1148 | 0.1174 | 0.1198 |
| 0.1203 | 0.1226 | 0.1292 | 0.1307 | 0.1385 | 0.1408 | 0.1445 | 0.1507 |
| 0.1537 | 0.1567 | 0.1597 | 0.1681 | 0.1701 | 0.1746 | 0.1794 | 0.1810 |
| 0.1904 | 0.1935 | 0.1996 | 0.2015 | 0.2058 | 0.2118 | 0.2201 | 0.2243 |
| 0.2291 | 0.2398 | 0.2399 | 0.2618 | 0.2643 | 0.2695 | 0.2732 | 0.2941 |
| 0.2963 | 0.2989 | 0.3128 | 0.3179 | 0.3288 | 0.3382 | 0.3434 | 0.3452 |
| 0.3537 | 0.3558 | 0.3602 | 0.3677 | 0.3722 | 0.3770 | 0.3807 | 0.3842 |
| 0.3900 | 0.3985 | 0.4065 | 0.4103 | 0.4172 | 0.4260 | 0.4268 | 0.4284 |
| 0.4331 | 0.4371 | 0.4446 | 0.4465 | 0.4499 | 0.4554 | 0.4573 | 0.4665 |
| 0.4678 | 0.4693 | 0.4735 | 0.4776 | 0.4828 | 0.4851 | 0.4916 | 0.4940 |
| 0.4981 | 0.5021 | 0.5061 | 0.5092 | 0.5156 | 0.5180 | 0.5200 | 0.5260 |
| 0.5310 | 0.5364 | 0.5407 | 0.5422 | 0.5456 | 0.5482 | 0.5498 | 0.5555 |
| 0.5588 | 0.5640 | 0.5717 | 0.5729 | 0.5766 | 0.5823 | 0.5855 | 0.5909 |
| 0.5982 | 0.6074 | 0.6078 | 0.6141 | 0.6212 | 0.6315 | 0.6363 | 0.6385 |
| 0.6418 | 0.6453 | 0.6526 | 0.6554 | 0.6695 | 0.6757 | 0.6830 | 0.6878 |
| 0.6940 | 0.7008 | 0.7066 | 0.7157 | 0.7221 | 0.7243 | 0.7342 | 0.7385 |
| 0.7473 | 0.7528 | 0.7602 | 0.7685 | 0.7706 | 0.7786 | 0.7879 | 0.7984 |
| 0.8068 | 0.8187 | 0.8215 | 0.8290 | 0.8318 | 0.8373 | 0.8425 | 0.8454 |
| 0.8606 | 0.8657 | 0.8698 | 0.8728 | 0.8741 | 0.8894 | 0.9074 | 0.9106 |
| 0.9191 | 0.9215 | 0.9273 | 0.9359 | 0.9431 | 0.9547 | 0.9612 | 0.9717 |
| 0.9724 | 0.9840 | 0.9904 | 0.9981 | 1.0089 | 1.0131 | 1.0192 | 1.0217 |
| 1.0280 | 1.0357 | 1.0459 | 1.0491 | 1.0591 | 1.0655 | 1.0714 | 1.0784 |
| 1.0884 | 1.0916 | 1.0960 | 1.1008 | 1.1047 | 1.1101 | 1.1236 | 1.1265 |
| 1.1282 | 1.1374 | 1.1397 | 1.1541 | 1.1572 | 1.1613 | 1.1644 | 1.1738 |
| 1.1762 | 1.1827 | 1.1940 | 1.1981 | 1.2024 | 1.2096 | 1.2154 | 1.2185 |
| 1.2214 | 1.2287 | 1.2306 | 1.2376 | 1.2451 | 1.2491 | 1.2652 | 1.2666 |
| 1.2701 | 1.2744 | 1.2841 | 1.2858 | 1.2999 | 1.3037 | 1.3106 | 1.3185 |
| 1.3243 | 1.3302 | 1.3357 | 1.3434 | 1.3462 | 1.3506 | 1.3544 | 1.3635 |
| 1.3709 | 1.3752 | 1.3870 | 1.3966 | 1.4007 | 1.4093 | 1.4124 | 1.4225 |
| 1.4289 | 1.4333 | 1.4354 | 1.4496 | 1.4582 | 1.4652 | 1.4797 | 1.4889 |
| 1.4932 | 1.4943 | 1.4997 | 1.5118 | 1.5126 | 1.5299 | 1.5477 | 1.5505 |
| 1.5543 | 1.5645 | 1.5716 | 1.5846 | 1.5948 | 1.6051 | 1.6241 | 1.6292 |
| 1.6450 | 1.6592 | 1.6797 | 1.6993 | 1.7041 | 1.7244 | 1.7636 | 1.7750 |
| 1.7837 | 1.8011 | 1.8118 | 1.8265 | 1.8419 | 1.8753 | 1.8802 | 1.9181 |

| | | | | | | | |
|---|---|---|---|---|---|---|---|
| 1.9486 | 1.9559 | 1.9937 | 2.0039 | 2.0459 | 2.1053 | 2.1432 | 2.1750 |
| 2.2035 | 2.2584 | 2.3256 | 2.3630 | 2.3773 | 2.3928 | 2.4021 | 2.4431 |
| 2.4463 | 2.4546 | 2.4590 | 2.4713 | 2.4786 | 2.4900 | 2.5097 | 2.5166 |
| 2.5328 | 2.5343 | 2.5448 | 2.5514 | 2.5609 | 2.5744 | 2.5823 | 2.5854 |
| 2.5967 | 2.6077 | 2.6160 | 2.6172 | 2.6316 | 2.6390 | 2.6429 | 2.6546 |
| 2.6616 | 2.6673 | 2.6796 | 2.6858 | 2.6870 | 2.6960 | 2.7030 | 2.7074 |
| 2.7187 | 2.7219 | 2.7269 | 2.7332 | 2.7442 | 2.7465 | 2.7522 | 2.7601 |
| 2.7700 | 2.7813 | 2.7902 | 2.7991 | 2.8017 | 2.8150 | 2.8227 | 2.8297 |
| 2.8389 | 2.8506 | 2.8582 | 2.8626 | 2.8676 | 2.8719 | 2.8818 | 2.8860 |
| 2.8926 | 2.8967 | 2.9148 | 2.9195 | 2.9232 | 2.9348 | 2.9388 | 2.9448 |
| 2.9532 | 2.9592 | 2.9713 | 2.9790 | 2.9852 | 2.9948 | 2.9982 | 3.0084 |
| 3.0121 | 3.0140 | 3.0209 | 3.0258 | 3.0384 | 3.0509 | 3.0547 | 3.0584 |
| 3.0607 | 3.0642 | 3.0686 | 3.0779 | 3.0911 | 3.1014 | 3.1034 | 3.1149 |
| 3.1236 | 3.1312 | 3.1404 | 3.1447 | 3.1546 | 3.1597 | 3.1632 | 3.1781 |
| 3.1853 | 3.1909 | 3.1951 | 3.1977 | 3.2042 | 3.2116 | 3.2241 | 3.2262 |
| 3.2360 | 3.2443 | 3.2513 | 3.2569 | 3.2614 | 3.2643 | 3.2722 | 3.2804 |
| 3.2819 | 3.2874 | 3.2970 | 3.2987 | 3.3070 | 3.3220 | 3.3266 | 3.3336 |
| 3.3451 | 3.3476 | 3.3542 | 3.3596 | 3.3630 | 3.3750 | 3.3782 | 3.3926 |
| 3.3958 | 3.4087 | 3.4109 | 3.4243 | 3.4365 | 3.4466 | 3.4562 | 3.4653 |
| 3.4738 | 3.4815 | 3.4906 | 3.4988 | 3.5041 | 3.5128 | 3.5278 | 3.5363 |
| 3.5383 | 3.5460 | 3.5523 | 3.5540 | 3.5638 | 3.5783 | 3.5928 | 3.6031 |
| 3.6097 | 3.6153 | 3.6267 | 3.6386 | 3.6459 | 3.6494 | 3.6552 | 3.6579 |
| 3.6711 | 3.6735 | 3.6761 | 3.6795 | 3.6913 | 3.7032 | 3.7139 | 3.7282 |
| 3.7354 | 3.7377 | 3.7541 | 3.7592 | 3.7641 | 3.7789 | 3.7801 | 3.7912 |
| 3.7951 | 3.8145 | 3.8235 | 3.8331 | 3.8400 | 3.8507 | 3.8527 | 3.8653 |
| 3.8708 | 3.8792 | 3.8946 | 3.9066 | 3.9094 | 3.9210 | 3.9309 | 3.9353 |
| 3.9492 | 3.9557 | 3.9580 | 3.9628 | 3.9742 | 3.9873 | 3.9904 | 4.0030 |
| 4.0221 | 4.0264 | 4.0381 | 4.0488 | 4.0579 | 4.0690 | 4.0716 | 4.0896 |
| 4.0989 | 4.1093 | 4.1134 | 4.1217 | 4.1359 | 4.1399 | 4.1463 | 4.1586 |
| 4.1633 | 4.1700 | 4.1774 | 4.1832 | 4.1907 | 4.2009 | 4.2054 | 4.2113 |
| 4.2194 | 4.2291 | 4.2412 | 4.2563 | 4.2619 | 4.2866 | 4.2990 | 4.3050 |
| 4.3178 | 4.3219 | 4.3270 | 4.3356 | 4.3441 | 4.3463 | 4.3710 | 4.3726 |
| 4.3864 | 4.3923 | 4.3979 | 4.4125 | 4.4218 | 4.4253 | 4.4284 | 4.4473 |
| 4.4584 | 4.4647 | 4.4786 | 4.4942 | 4.5002 | 4.5059 | 4.5116 | 4.5169 |
| 4.5408 | 4.5492 | 4.5586 | 4.5666 | 4.5846 | 4.5894 | 4.6194 | 4.6217 |
| 4.6378 | 4.6408 | 4.6618 | 4.6938 | 4.7057 | 4.7163 | 4.7295 | 4.7541 |
| 4.7713 | 4.7897 | 4.8189 | 4.8309 | 4.8626 | 4.8837 | 4.9005 | 4.9105 |
| 4.9275 | 4.9424 | 4.9660 | 4.9755 | 4.9842 | 4.9864 | 5.0065 | 5.0385 |
| 5.0497 | 5.0734 | 5.0780 | 5.0909 | 5.1118 | 5.1147 | 5.1310 | 5.1887 |
| 5.2019 | 5.2198 | 5.2457 | 5.2510 | 5.2657 | 5.2848 | 5.3026 | 5.3511 |
| 5.3743 | 5.4007 | 5.4124 | 5.4462 | 5.4640 | 5.5217 | 5.5263 | 5.5470 |
| 5.5721 | 5.5972 | 5.6014 | 5.6199 | 5.6373 | 5.6531 | 5.6639 | 5.6736 |
| 5.6902 | 5.6989 | 5.7137 | 5.7337 | 5.7613 | 5.7672 | 5.7971 | 5.8286 |
| 5.8861 | 5.9003 | 5.9440 | 5.9932 | 6.0340 | 6.0665 | 6.0803 | 6.1318 |
| 6.1467 | 6.2412 | 6.2900 | 6.3332 | 6.5349 | 6.5908 | 6.7967 | 23.9664 |
| 24.1402 | 24.1598 | 24.2421 | 24.2824 | 24.2832 | 24.3046 | 24.3200 | 24.4316 |
| 24.5969 | 24.6154 | 24.6282 | 24.7993 | 24.9239 | 35.7562 | 35.7684 | 35.7886 |
| 35.9408 | | | | | | | |

---

Ground-State Mulliken Net Atomic Charges

| Atom | | Charge (a.u.) |
|---|---|---|
| 1 | N | 0.330205 |
| 2 | C | 0.195354 |
| 3 | C | -0.175777 |
| 4 | C | -0.147962 |
| 5 | C | -0.221555 |
| 6 | C | -0.096370 |
| 7 | C | -0.362470 |
| 8 | C | -0.153744 |
| 9 | N | -0.160598 |
| 10 | C | 0.080851 |
| 11 | N | -0.308987 |
| 12 | C | 0.528882 |
| 13 | C | -0.262393 |
| 14 | C | 0.157776 |
| 15 | C | -0.270936 |
| 16 | C | -0.088538 |

```
   17 C                    -0.441459
   18 N                    -0.398590
   19 H                     0.197958
   20 H                     0.213528
   21 H                     0.202428
   22 H                     0.227563
   23 H                     0.172644
   24 H                     0.166200
   25 H                     0.161362
   26 H                     0.198490
   27 H                     0.176265
   28 H                     0.297088
   29 H                     0.195222
   30 H                     0.174857
   31 H                     0.152997
   32 H                     0.150064
   33 H                     0.143181
   34 H                     0.134886
   35 H                     0.164040
   36 H                     0.105677
   37 H                     0.249390
   38 H                     0.312471
 ----------------------------------------
 Sum of atomic charges =     2.000000

-----------------------------------------------------------------
                    Cartesian Multipole Moments
-----------------------------------------------------------------
   Charge (ESU x 10^10)
                 9.6064
   Dipole Moment (Debye)
         X       4.8541      Y       0.5450      Z      -5.4989
       Tot       7.3551
   Quadrupole Moments (Debye-Ang)
        XX     -32.5941     XY      26.9718     YY     -68.6388
        XZ     -40.6426     YZ     -21.5323     ZZ     -47.2958
   Traceless Quadrupole Moments (Debye-Ang)
       QXX      50.7464    QYY     -57.3876    QZZ       6.6413
       QXY      80.9155    QXZ    -121.9278    QYZ     -64.5968
   Octopole Moments (Debye-Ang^2)
       XXX      49.9031    XXY      24.5600    XYY      -8.5592
       YYY     -22.2896    XXZ       3.0378    XYZ     -19.2132
       YYZ      30.4287    XZZ      42.8542    YZZ      23.7500
       ZZZ     -76.0880
   Traceless Octopole Moments (Debye-Ang^2)
       XXX      -9.2371    YYY    -568.5271    ZZZ    -757.7268
       XXY     290.3384    XXZ     173.4317    XYY    -380.9819
       XYZ    -288.1974    XZZ     390.2190    YYZ     584.2950
       YZZ     278.1888
   Hexadecapole Moments (Debye-Ang^3)
      XXXX   -2790.6577   XXXY    -224.7794   XXYY    -620.9866
      XYYY    -564.3725   YYYY    -822.7790   XXXZ     769.3530
      XXYZ     -60.3614   XYYZ     326.1143   YYYZ     476.6698
      XXZZ    -708.4609   XYZZ      15.7847   YYZZ    -606.7548
      XZZZ     713.6870   YZZZ     261.0154   ZZZZ   -1998.3440
   Traceless Hexadecapole Moments (Debye-Ang^3)
      XXXX   -7567.2633   XXXY   11199.6859   XXXZ    -629.8767
      XXYY   -1096.7631   XXYZ  -16497.5720   XXZZ    8664.0263
      XYYY  -24457.5863   XYYZ    7104.6910   XYZZ   13257.9003
      XZZZ   -6474.8143   YYYY   12797.3714   YYYZ   19571.4521
      YYZZ  -11700.6083   YZZZ   -3073.8800   ZZZZ    3036.5820
-----------------------------------------------------------------
Total job time:  637.65s(wall), 456.45s(cpu)
Mon Oct 14 03:37:12 2024
```

```
Wavefunction Version of Q-Chem

 Parts of Q-Chem use Armadillo 8.300.2 (Tropical Shenanigans).
 http://arma.sourceforge.net/

 Q-Chem begins on Sun Oct 13 12:03:21 2024


     Scratch files written to
C:/Users/hille/AppData/Local/Temp/WFB0CB2A5C99266D7A/Conformer.2//scratch///
Processing default memory
  ... MEM_TOTAL 4076 MB (default) [16 cores]
Processing $rem in C:/Program
Files/Wavefunction/Spartan24v110/P4e//../auxdir/config/preferences:
   (site specific preferences)
  ... THRESH             9
  ... SMALL_PROD_XCMAT   9
  ... BASIS_LIN_DEP_THRESH     5
  ... SCF_ALGORITHM     DIIS_GDM
  ... MAXSCF            250
  ... MAXDIIS          45
  ... THRESHDIIS         -1  (i.e. don't switch on delta-E)
  ... ECP_FIT          TRUE (Convert deprecated ECP files)
  ... GUI              GUI_SPARTAN
  ... TERSE_OUTPUT     TRUE !turn on spartan printing
  ... SCF_CONVERGENCE  7
  ... CCMAN2 FALSE  (qc4.3)
  ... SYMMETRY   FALSE  ! turn of symmetry for spartan16
  ... SYM_IGNORE TRUE   ! ..use FORCESYMMETRY to override
  ... GEOM_OPT_TOL_GRADIENT       700  ! loosen tolernaces for organic geometries
  ... GEOM_OPT_TOL_DISPLACEMENT  1400  ! was 1200 = .0012
  ... GEOM_OPT_TOL_ENERGY        2000  ! was  100 = .000 001
  ... GEN_SCFMAN  FALSE
Processing $rem in input file
  ... JOBTYPE    OPT
  ... TIDY_SYM   TRUE
  ... METHOD            WB97X-D
  ... xc_grid           75000302  (75,302)
  ... BASIS             6-311+G**
  ... THRESH             12  #diffuse default
  ... MAXSCF            350  #diffuse default
  ... GEOM_OPT_TOL_ENERGY       22850
  ... GEOM_OPT_TOL_GRADIENT       800
  ... VARTHRESH         2    (default DFT)
  ... INCDFT            TRUE (default DFT)
  ... GEOM_OPT_HESSIAN      READ (main opt)
  ... EXTERNAL_HESSIAN      1
  ... GUI                GUI_SPARTAN
  ... TERSE_OUTPUT       TRUE
NAlpha2: 130
NElect   130
Mult     1

Checking the input file for inconsistencies...  ...done.

--------------------------------------------------------------
User input:
--------------------------------------------------------------
$comment
Molecule1
$end
$molecule
 2 1
    7     2.3911357821    0.50996154498   0.091913713209
    6     3.0955534041  -0.022906576004     1.1393178098
    6     3.9507836532    -1.1136201421    0.90794072073
    6     4.0994417624    -1.6383999273    -0.3668554596
    6      3.408462758    -1.0601149898    -1.4144989924
    6     2.5720464746   0.018015353548    -1.1658145763
```

```
    6      3.015313447    0.54759727258     2.5285902872
    6     1.4762075353     1.6823041692    0.25571299822
    7    -2.0012661836    0.41714081949     1.0129895084
    6    -2.5834607921    0.54453633144   -0.18779859575
    7    -1.9232976741     1.0413520304    -1.2596293721
    6   -0.65588676555     1.4339872671    -1.0920541998
    6   0.017176018928      1.309398092     0.1166285066
    6    -0.7191297183     0.7826657046     1.1820125932
    6    -4.0082193561   0.095823747374   -0.36009643026
    6    -4.1177911729    -1.2948054246   -0.98720702303
    6    -3.6186661731    -2.4059498148  -0.073690183201
    7  -0.047058898606     1.9522212462    -2.2243712645
    1     4.5247245137     -1.553710522     1.7244884009
    1     4.7703178791    -2.4791097504   -0.54399665362
    1     3.5331464733    -1.4393586093    -2.4276931767
    1      2.046564278    0.49922748726    -1.9863408833
    1     3.5847994436  -0.054751306659     3.2443249808
    1     1.9795472666    0.56704052699     2.8765114306
    1     3.4302873283     1.5598276083     2.5427279182
    1     1.7899863414     2.4281174513   -0.48447253296
    1     1.6435420062     2.1546429656     1.2267986332
    1    -2.5483955177   0.033497641754     1.7864235319
    1   -0.32359839665    0.63679218687     2.1815520796
    1    -4.5245008637    0.11863249824    0.60669649964
    1    -4.5190453904    0.82085764105    -1.0051303239
    1    -3.5677944316    -1.3316152639    -1.9351888612
    1    -5.1713635243    -1.4882016724     -1.221925499
    1    -2.5451728366    -2.3157671533    0.11802565674
    1    -3.7926266331    -3.3813314358   -0.53929882882
    1    -4.1441023484    -2.3906715237    0.88646464673
    1    0.19677299228     2.9401151812     -2.151023787
    1   -0.71443268133     1.8765593446    -3.0020332723
$end
$rem
JOBTYPE     OPT
TIDY_SYM    TRUE
METHOD           WB97X-D
xc_grid          75000302  (75,302)
BASIS            6-311+G**
THRESH            12  #diffuse default
MAXSCF           350  #diffuse default
GEOM_OPT_TOL_ENERGY     22850
GEOM_OPT_TOL_GRADIENT     800
VARTHRESH        2    (default DFT)
INCDFT           TRUE (default DFT)
GEOM_OPT_HESSIAN      READ (main opt)
EXTERNAL_HESSIAN      1
GUI               GUI_SPARTAN
TERSE_OUTPUT      TRUE
$end
$opt
$end
--------------------------------------------------------------
 ---------------------------------------------------------------
             Standard Nuclear Orientation (Angstroms)
    I     Atom           X                Y                Z
 ---------------------------------------------------------------
    1      N       2.3911357821     0.5099615450     0.0919137132
    2      C       3.0955534041    -0.0229065760     1.1393178098
    3      C       3.9507836532    -1.1136201421     0.9079407207
    4      C       4.0994417624    -1.6383999273    -0.3668554596
    5      C       3.4084627580    -1.0601149898    -1.4144989924
    6      C       2.5720464746     0.0180153535    -1.1658145763
    7      C       3.0153134470     0.5475972726     2.5285902872
    8      C       1.4762075353     1.6823041692     0.2557129982
    9      N      -2.0012661836     0.4171408195     1.0129895084
   10      C      -2.5834607921     0.5445363314    -0.1877985957
   11      N      -1.9232976741     1.0413520304    -1.2596293721
   12      C      -0.6558867656     1.4339872671    -1.0920541998
   13      C       0.0171760189     1.3093980920     0.1166285066
```

```
   14      C      -0.7191297183     0.7826657046     1.1820125932
   15      C      -4.0082193561     0.0958237474    -0.3600964303
   16      C      -4.1177911729    -1.2948054246    -0.9872070230
   17      C      -3.6186661731    -2.4059498148    -0.0736901832
   18      N      -0.0470588986     1.9522212462    -2.2243712645
   19      H       4.5247245137    -1.5537105220     1.7244884009
   20      H       4.7703178791    -2.4791097504    -0.5439966536
   21      H       3.5331464733    -1.4393586093    -2.4276931767
   22      H       2.0465642780     0.4992274873    -1.9863408833
   23      H       3.5847994436    -0.0547513067     3.2443249808
   24      H       1.9795472666     0.5670405270     2.8765114306
   25      H       3.4302873283     1.5598276083     2.5427279182
   26      H       1.7899863414     2.4281174513    -0.4844725330
   27      H       1.6435420062     2.1546429656     1.2267986332
   28      H      -2.5483955177     0.0334976418     1.7864235319
   29      H      -0.3235983967     0.6367921869     2.1815520796
   30      H      -4.5245008637     0.1186324982     0.6066964996
   31      H      -4.5190453904     0.8208576411    -1.0051303239
   32      H      -3.5677944316    -1.3316152639    -1.9351888612
   33      H      -5.1713635243    -1.4882016724    -1.2219254990
   34      H      -2.5451728366    -2.3157671533     0.1180256567
   35      H      -3.7926266331    -3.3813314358    -0.5392988288
   36      H      -4.1441023484    -2.3906715237     0.8864646467
   37      H       0.1967729923     2.9401151812    -2.1510237870
   38      H      -0.7144326813     1.8765593446    -3.0020332723
 ----------------------------------------------------------------
 Nuclear Repulsion Energy =        1309.71375464 hartrees
 There are       65 alpha and       65 beta electrons
 Requested basis set is 6-311+G(d,p)
 There are 188 shells and 516 basis functions

 Total QAlloc Memory Limit   4076 MB
 Mega-Array Size       188 MB
 MEM_STATIC part       192 MB

 ..                                                                        (5.2.P)

--------------------------------------------------
-  Entering fldman on Sun Oct 13 12:03:21 2024  -
--------------------------------------------------

 A cutoff of  1.0D-12 yielded  12862 shell pairs
 There are    102084 function pairs (    108777 Cartesian)
 Smallest overlap matrix eigenvalue = 2.14E-06
 Linear dependence detected in AO basis
 Tighter screening thresholds may be required for diffuse basis sets
 Use S2THRESH > 12 and THRESH = 14 in case of SCF convergence issues
 Number of orthogonalized atomic orbitals = 510
 Maximum deviation from orthogonality = 1.178E-11

 Scale SEOQF with 1.000000e-01/1.000000e-01/1.000000e-01

 Standard Electronic Orientation quadrupole field applied
 Nucleus-field energy     =     0.0000000307 hartrees

--------------------------------------------------
-  Entering gesman on Sun Oct 13 12:03:21 2024  -
--------------------------------------------------

 Guess from superposition of atomic densities
 Warning:  Energy on first SCF cycle will be non-variational
 SAD guess density has 132.000000 electrons

--------------------------------------------------
-  Entering scfman on Sun Oct 13 12:03:21 2024  -
--------------------------------------------------

 Long-range K will be added via erf
 Coulomb attenuation parameter = 0.2 bohr**(-1)
 A restricted hybrid HF-DFT SCF calculation will be
```

```
 performed using Pulay DIIS + Geometric Direct Minimization
 Exchange:     0.2220 Hartree-Fock + 1.0000 wB97X-D + LR-HF
 Correlation:  1.0000 wB97X-D
 Using Euler-Maclaurin-Lebedev (75,302) quadrature formula
 Dispersion:   Grimme D
 SCF converges when RMS gradient is below 1.0E-07
 Exchange:     0.2220 Hartree-Fock + 1.0000 wB97X-D + LR-HF
 Correlation:  1.0000 wB97X-D
 Using Euler-Maclaurin-Lebedev (75,302) quadrature formula
 Dispersion:   Grimme D
 ---------------------------------------
  Cycle       Energy         DIIS Error
 ---------------------------------------
    1    -770.2965594320      2.95E-02
    2    -763.7512266392      2.39E-03
    3    -763.7586703317      2.57E-03
    4    -763.9444491693      3.72E-04
    5    -763.9478346097      1.87E-04
    6    -763.9488312957      3.37E-05
    7    -763.9488652697      1.32E-05
    8    -763.9488707539      3.22E-06
    9    -763.9488712746      1.52E-06
   10    -763.9488713736      5.68E-07
   11    -763.9488713922      2.18E-07
   12    -763.9488713960      8.95E-08 Convergence criterion met
 ---------------------------------------
 SCF time:  CPU 340.39 s  wall 682.47 s
 SCF   energy in the final basis set = -763.94887140
 Total energy in the final basis set = -763.94887140

-------------------------------------------------
-  Entering anlman on Sun Oct 13 12:14:44 2024  -
-------------------------------------------------



 --------------------------------------------------------------

                   Orbital Energies (a.u.)
 --------------------------------------------------------------


 Alpha MOs
 -- Occupied --
-14.8147 -14.8145 -14.7243 -14.7168 -10.6766 -10.6719 -10.6640 -10.6381
-10.6344 -10.6210 -10.6146 -10.5766 -10.5684 -10.5674 -10.5492 -10.5352
-10.5001 -10.4760  -1.4303  -1.3961  -1.3461  -1.2680  -1.2278  -1.1995
 -1.1780  -1.1469  -1.1047  -1.0770  -1.0667  -1.0524  -1.0244  -1.0128
 -0.9850  -0.9545  -0.9463  -0.9084  -0.9018  -0.8888  -0.8777  -0.8630
 -0.8472  -0.8412  -0.8331  -0.8173  -0.8077  -0.7931  -0.7852  -0.7812
 -0.7721  -0.7561  -0.7487  -0.7449  -0.7364  -0.7292  -0.7224  -0.7078
 -0.7066  -0.6711  -0.6579  -0.6511  -0.6407  -0.6331  -0.6308  -0.6238
 -0.6119
 -- Virtual --
 -0.2795  -0.2691  -0.2570  -0.2226  -0.1779  -0.1634  -0.1466  -0.1448
 -0.1342  -0.1258  -0.1204  -0.1150  -0.1086  -0.1059  -0.1008  -0.0921
 -0.0879  -0.0833  -0.0822  -0.0793  -0.0780  -0.0721  -0.0708  -0.0654
 -0.0626  -0.0604  -0.0587  -0.0530  -0.0491  -0.0468  -0.0443  -0.0374
 -0.0330  -0.0286  -0.0278  -0.0239  -0.0196  -0.0157  -0.0141  -0.0111
 -0.0075  -0.0063  -0.0005   0.0011   0.0051   0.0106   0.0116   0.0151
  0.0197   0.0207   0.0262   0.0278   0.0293   0.0331   0.0356   0.0391
  0.0416   0.0448   0.0471   0.0519   0.0535   0.0552   0.0578   0.0626
  0.0661   0.0709   0.0723   0.0743   0.0758   0.0768   0.0806   0.0842
  0.0915   0.0962   0.0992   0.0996   0.1075   0.1078   0.1119   0.1129
  0.1173   0.1193   0.1264   0.1286   0.1327   0.1362   0.1393   0.1437
  0.1456   0.1541   0.1587   0.1595   0.1639   0.1673   0.1695   0.1732
  0.1740   0.1745   0.1851   0.1942   0.1968   0.2079   0.2165   0.2241
  0.2312   0.2348   0.2529   0.2630   0.2796   0.2895   0.2949   0.3121
  0.3190   0.3271   0.3368   0.3391   0.3456   0.3510   0.3615   0.3643
  0.3705   0.3760   0.3818   0.3850   0.3958   0.4015   0.4182   0.4211
  0.4252   0.4327   0.4422   0.4493   0.4580   0.4624   0.4664   0.4712
  0.4821   0.4862   0.4962   0.5010   0.5074   0.5089   0.5112   0.5152
```

| | | | | | | | |
|---|---|---|---|---|---|---|---|
| 0.5187 | 0.5247 | 0.5259 | 0.5310 | 0.5392 | 0.5468 | 0.5514 | 0.5558 |
| 0.5586 | 0.5624 | 0.5679 | 0.5719 | 0.5785 | 0.5811 | 0.5940 | 0.5976 |
| 0.6021 | 0.6094 | 0.6189 | 0.6269 | 0.6331 | 0.6448 | 0.6573 | 0.6617 |
| 0.6691 | 0.6719 | 0.6750 | 0.6832 | 0.6860 | 0.6971 | 0.7013 | 0.7057 |
| 0.7146 | 0.7184 | 0.7357 | 0.7399 | 0.7547 | 0.7588 | 0.7661 | 0.7719 |
| 0.7866 | 0.7990 | 0.8039 | 0.8075 | 0.8252 | 0.8414 | 0.8452 | 0.8504 |
| 0.8579 | 0.8677 | 0.8709 | 0.8810 | 0.8931 | 0.8996 | 0.9163 | 0.9296 |
| 0.9319 | 0.9534 | 0.9603 | 0.9807 | 0.9932 | 1.0004 | 1.0176 | 1.0303 |
| 1.0550 | 1.0653 | 1.0835 | 1.0984 | 1.1107 | 1.1698 | 1.1748 | 1.1979 |
| 1.2065 | 1.2161 | 1.2254 | 1.2597 | 1.2643 | 1.2697 | 1.2916 | 1.3040 |
| 1.3090 | 1.3263 | 1.3324 | 1.3363 | 1.3476 | 1.3650 | 1.3767 | 1.3785 |
| 1.3815 | 1.3971 | 1.3985 | 1.4057 | 1.4105 | 1.4223 | 1.4279 | 1.4318 |
| 1.4383 | 1.4388 | 1.4478 | 1.4555 | 1.4671 | 1.4688 | 1.4697 | 1.4799 |
| 1.4891 | 1.4944 | 1.5122 | 1.5144 | 1.5250 | 1.5411 | 1.5476 | 1.5554 |
| 1.5564 | 1.5674 | 1.5750 | 1.5789 | 1.5836 | 1.5908 | 1.5956 | 1.6082 |
| 1.6243 | 1.6300 | 1.6438 | 1.6544 | 1.6602 | 1.6649 | 1.6804 | 1.6819 |
| 1.6884 | 1.6972 | 1.7090 | 1.7203 | 1.7258 | 1.7473 | 1.7507 | 1.7745 |
| 1.7777 | 1.7873 | 1.7970 | 1.8116 | 1.8307 | 1.8375 | 1.8470 | 1.8651 |
| 1.8845 | 1.8912 | 1.9007 | 1.9106 | 1.9302 | 1.9507 | 1.9673 | 1.9861 |
| 1.9946 | 2.0063 | 2.0105 | 2.0315 | 2.0552 | 2.0626 | 2.0716 | 2.0918 |
| 2.1004 | 2.1155 | 2.1181 | 2.1355 | 2.1537 | 2.1679 | 2.2063 | 2.2140 |
| 2.2284 | 2.2332 | 2.2383 | 2.2631 | 2.2659 | 2.2763 | 2.2913 | 2.2996 |
| 2.3018 | 2.3143 | 2.3395 | 2.3573 | 2.3623 | 2.3656 | 2.3881 | 2.3904 |
| 2.4040 | 2.4113 | 2.4162 | 2.4384 | 2.4435 | 2.4687 | 2.4755 | 2.4918 |
| 2.5010 | 2.5132 | 2.5231 | 2.5334 | 2.5395 | 2.5421 | 2.5435 | 2.5518 |
| 2.5587 | 2.5710 | 2.5793 | 2.5853 | 2.5893 | 2.5992 | 2.6045 | 2.6124 |
| 2.6192 | 2.6332 | 2.6461 | 2.6643 | 2.6720 | 2.6782 | 2.6877 | 2.6985 |
| 2.7074 | 2.7113 | 2.7193 | 2.7267 | 2.7333 | 2.7459 | 2.7667 | 2.7762 |
| 2.7844 | 2.7956 | 2.8056 | 2.8134 | 2.8367 | 2.8524 | 2.8609 | 2.8856 |
| 2.8948 | 2.9309 | 2.9403 | 2.9802 | 3.0252 | 3.0517 | 3.0749 | 3.1124 |
| 3.1461 | 3.1601 | 3.2400 | 3.2559 | 3.2861 | 3.3116 | 3.3339 | 3.3760 |
| 3.4007 | 3.4677 | 3.4908 | 3.5356 | 3.5530 | 3.5887 | 3.6106 | 3.6497 |
| 3.6554 | 3.6728 | 3.6989 | 3.7089 | 3.7279 | 3.7456 | 3.7548 | 3.7639 |
| 3.8187 | 3.8653 | 3.8946 | 3.9055 | 3.9429 | 4.0312 | 4.0356 | 4.1015 |
| 4.1139 | 4.2027 | 4.3004 | 4.3131 | 4.5500 | 4.6394 | 4.6779 | 4.7067 |
| 4.8654 | 5.0267 | 5.1446 | 23.5213 | 23.5601 | 23.6809 | 23.7174 | 23.7362 |
| 23.7922 | 23.7958 | 23.8247 | 23.8440 | 23.8677 | 23.9044 | 23.9265 | 23.9318 |
| 24.0290 | 35.4139 | 35.4387 | 35.4825 | 35.5752 | | | |

Ground-State Mulliken Net Atomic Charges

| Atom | | Charge (a.u.) |
|---|---|---|
| 1 | N | 0.410510 |
| 2 | C | 0.141309 |
| 3 | C | -0.044224 |
| 4 | C | -0.140824 |
| 5 | C | -0.184392 |
| 6 | C | -0.180707 |
| 7 | C | -0.793085 |
| 8 | C | -0.262468 |
| 9 | N | -0.105637 |
| 10 | C | -0.236491 |
| 11 | N | 0.042347 |
| 12 | C | 0.064035 |
| 13 | C | 0.031801 |
| 14 | C | 0.085419 |
| 15 | C | -0.332266 |
| 16 | C | -0.251225 |
| 17 | C | -0.524029 |
| 18 | N | -0.464361 |
| 19 | H | 0.222316 |
| 20 | H | 0.232201 |
| 21 | H | 0.228280 |
| 22 | H | 0.252245 |
| 23 | H | 0.232265 |
| 24 | H | 0.164103 |
| 25 | H | 0.250488 |
| 26 | H | 0.238836 |

```
    27 H                     0.244023
    28 H                     0.394798
    29 H                     0.209109
    30 H                     0.210241
    31 H                     0.255399
    32 H                     0.202412
    33 H                     0.225142
    34 H                     0.128394
    35 H                     0.216307
    36 H                     0.179320
    37 H                     0.324741
    38 H                     0.333668
  ----------------------------------------
  Sum of atomic charges =     2.000000

 -----------------------------------------------------------------
                    Cartesian Multipole Moments
 -----------------------------------------------------------------
    Charge (ESU x 10^10)
                 9.6064
    Dipole Moment (Debye)
         X       4.4810      Y       2.9958      Z       2.0762
       Tot       5.7762
    Quadrupole Moments (Debye-Ang)
        XX     -12.0861     XY      -5.3826     YY     -76.3582
        XZ       3.0834     YZ      -2.1870     ZZ     -69.8572
    Traceless Quadrupole Moments (Debye-Ang)
       QXX     122.0433    QYY     -70.7731    QZZ     -51.2701
       QXY     -16.1477    QXZ       9.2501    QYZ      -6.5610
    Octopole Moments (Debye-Ang^2)
       XXX      -2.4348    XXY     -98.8117    XYY      12.3305
       YYY     -14.3953    XXZ      34.0252    XYZ       7.8589
       YYZ     -20.4011    XZZ      15.6973    YZZ       7.1935
       ZZZ      33.8178
    Traceless Octopole Moments (Debye-Ang^2)
       XXX    -266.8600    YYY     738.1919    ZZZ      80.2891
       XXY   -1164.1349    XXZ     368.0528    XYY     108.1790
       XYZ     117.8837    XZZ     158.6811    YYZ    -448.3420
       YZZ     425.9430
    Hexadecapole Moments (Debye-Ang^3)
      XXXX   -3625.5644   XXXY    -251.4635   XXYY    -782.5602
      XYYY     -26.0066   YYYY    -931.9876   XXXZ    -114.9207
      XXYZ      67.3768   XYYZ     -67.8165   YYYZ     -27.9173
      XXZZ    -781.7863   XYZZ     -38.5351   YYZZ    -277.2265
      XZZZ    -201.5549   YZZZ     -15.9577   ZZZZ    -837.8672
    Traceless Hexadecapole Moments (Debye-Ang^3)
      XXXX    4700.6331   XXXY  -12183.4337   XXXZ    5226.4669
      XXYY   -1679.2358   XXYZ    6707.6756   XXZZ   -3021.3973
      XYYY   11489.5428   XYYZ   -1356.3468   XYZZ     693.8909
      XZZZ   -3870.1202   YYYY    -306.1007   YYYZ   -4031.9952
      YYZZ    1985.3364   YZZZ   -2675.6804   ZZZZ    1036.0609
 -----------------------------------------------------------------

--------------------------------------------------
-  Entering drvman on Sun Oct 13 12:14:44 2024  -
--------------------------------------------------

 Calculating analytic gradient of the SCF energy
 Gradient of SCF Energy
           1           2           3           4           5           6
    1  -0.0094929  -0.0111609   0.0047432  -0.0046830   0.0090713  -0.0054410
    2   0.0037751   0.0119885  -0.0051770   0.0031418  -0.0095793   0.0106433
    3  -0.0016386  -0.0065295   0.0027323  -0.0066821   0.0070425   0.0058134
           7           8           9          10          11          12
    1   0.0052144  -0.0050910  -0.0040853   0.0113137   0.0130802   0.0145563
    2  -0.0010803  -0.0036881  -0.0023013  -0.0034344   0.0170366  -0.0099053
    3   0.0037296  -0.0045699  -0.0007403   0.0434607  -0.0371938   0.0319239
          13          14          15          16          17          18
    1  -0.0008117  -0.0212924   0.0004243  -0.0002948   0.0003171   0.0183681
    2   0.0132922  -0.0096686  -0.0112469   0.0118709   0.0008587  -0.0025736
```

```
   3  -0.0377850   0.0096726  -0.0006927   0.0034480  -0.0000828   0.0003164
          19          20          21          22          23          24
   1   0.0055326   0.0027503   0.0029298   0.0022096   0.0014533  -0.0009216
   2  -0.0058234  -0.0030641  -0.0045171  -0.0021507  -0.0009092  -0.0028548
   3   0.0011197  -0.0011649  -0.0030125  -0.0053813   0.0060391  -0.0022514
          25          26          27          28          29          30
   1   0.0017260   0.0036860  -0.0022934  -0.0038746  -0.0022374  -0.0011650
   2  -0.0006096   0.0047025   0.0048926  -0.0026911  -0.0013664   0.0010825
   3  -0.0018694  -0.0017424   0.0018923   0.0037150   0.0018187   0.0000203
          31          32          33          34          35          36
   1  -0.0045224   0.0024240  -0.0026090  -0.0018884  -0.0014070   0.0000933
   2   0.0001375  -0.0031572  -0.0046024   0.0017904  -0.0018755   0.0015532
   3  -0.0014732  -0.0007452   0.0000191  -0.0005570  -0.0025947   0.0001097
          37          38
   1  -0.0090042  -0.0076171
   2   0.0051356   0.0003752
   3  -0.0020308  -0.0041357
 Max gradient component =       4.346E-02
 RMS gradient           =       9.256E-03
 Gradient time:  CPU 134.86 s  wall 308.56 s

--------------------------------------------------
-  Entering optman on Sun Oct 13 12:19:52 2024  -
--------------------------------------------------

 Geometry Optimization Parameters
   NAtoms,    NIC,     NZ,  NCons,   NDum,   NFix, NCnnct, MaxDiis
       38     272       0       0       0       0       0       0

 Cartesian Hessian read from HESS file


** GEOMETRY OPTIMIZATION IN DELOCALIZED INTERNAL COORDINATES **
   Searching for a Minimum

   Optimization Cycle:   1

                    Coordinates (Angstroms)
     ATOM                X               Y                Z
      1  N         2.3911357821    0.5099615450    0.0919137132
      2  C         3.0955534041   -0.0229065760    1.1393178098
      3  C         3.9507836532   -1.1136201421    0.9079407207
      4  C         4.0994417624   -1.6383999273   -0.3668554596
      5  C         3.4084627580   -1.0601149898   -1.4144989924
      6  C         2.5720464746    0.0180153535   -1.1658145763
      7  C         3.0153134470    0.5475972726    2.5285902872
      8  C         1.4762075353    1.6823041692    0.2557129982
      9  N        -2.0012661836    0.4171408195    1.0129895084
     10  C        -2.5834607921    0.5445363314   -0.1877985958
     11  N        -1.9232976741    1.0413520304   -1.2596293721
     12  C        -0.6558867655    1.4339872671   -1.0920541998
     13  C         0.0171760189    1.3093980920    0.1166285066
     14  C        -0.7191297183    0.7826657046    1.1820125932
     15  C        -4.0082193561    0.0958237474   -0.3600964303
     16  C        -4.1177911729   -1.2948054246   -0.9872070230
     17  C        -3.6186661731   -2.4059498148   -0.0736901832
     18  N        -0.0470588986    1.9522212462   -2.2243712645
     19  H         4.5247245137   -1.5537105220    1.7244884009
     20  H         4.7703178791   -2.4791097504   -0.5439966536
     21  H         3.5331464733   -1.4393586093   -2.4276931767
     22  H         2.0465642780    0.4992274873   -1.9863408833
     23  H         3.5847994436   -0.0547513067    3.2443249808
     24  H         1.9795472666    0.5670405270    2.8765114306
     25  H         3.4302873283    1.5598276083    2.5427279182
     26  H         1.7899863414    2.4281174513   -0.4844725330
     27  H         1.6435420062    2.1546429656    1.2267986332
     28  H        -2.5483955177    0.0334976418    1.7864235319
     29  H        -0.3235983966    0.6367921869    2.1815520796
     30  H        -4.5245008637    0.1186324982    0.6066964996
     31  H        -4.5190453904    0.8208576411   -1.0051303239
```

```
    32  H         -3.5677944316   -1.3316152639   -1.9351888612
    33  H         -5.1713635243   -1.4882016724   -1.2219254990
    34  H         -2.5451728366   -2.3157671533    0.1180256567
    35  H         -3.7926266331   -3.3813314358   -0.5392988288
    36  H         -4.1441023484   -2.3906715237    0.8864646467
    37  H          0.1967729923    2.9401151812   -2.1510237870
    38  H         -0.7144326813    1.8765593446   -3.0020332723
  Point Group: c1    Number of degrees of freedom:   108


  Energy is   -763.948871396


 Attempting to generate delocalized internal coordinates

 Transforming Cartesian Hessian to Internal Coordinates
 Hessian Transformation does not Include Derivative of B-matrix
  internal optimization (0)

108 Hessian modes will be used to form the next step
  Hessian Eigenvalues:
     0.001689    0.002579    0.003936    0.006043    0.009691    0.012366
     0.015050    0.018849    0.019627    0.019977    0.021543    0.022209
     0.022608    0.022754    0.024497    0.024671    0.026083    0.026851
     0.027351    0.028195    0.029947    0.031073    0.036120    0.037578
     0.038769    0.042064    0.043244    0.044037    0.045718    0.047473
     0.053383    0.055523    0.057472    0.058339    0.062589    0.078771
     0.085436    0.091654    0.121725    0.121791    0.127103    0.130632
     0.131546    0.132837    0.137709    0.139159    0.143297    0.146737
     0.147678    0.148734    0.149325    0.150868    0.152122    0.153079
     0.153391    0.153879    0.177484    0.206394    0.209177    0.216249
     0.221192    0.230264    0.236702    0.242443    0.246950    0.262501
     0.266141    0.268705    0.283020    0.292287    0.300075    0.300346
     0.300623    0.301168    0.301657    0.302513    0.304045    0.304360
     0.305133    0.305224    0.305889    0.308470    0.311364    0.314804
     0.320921    0.329266    0.331899    0.334202    0.339502    0.343188
     0.346894    0.348775    0.351651    0.363055    0.372771    0.382702
     0.394359    0.394996    0.403163    0.409863    0.416212    0.416650
     0.428863    0.436509    0.452848    0.492760    0.571753    0.744825

 Minimum search - taking simple RFO step
 Searching for Lamda that Minimizes Along All modes
 Value Taken    Lamda =  -0.02217692
 Calculated Step too Large.  Step scaled by  0.741366 !!
 Step Taken.  Stepsize is  0.300000  0.021612

                             Maximum     Tolerance    Cnvgd?
        Gradient            0.024464      0.000800      NO
        Displacement        0.092734      0.001400      NO
        Energy change      *********      0.000228      NO


 New Cartesian Coordinates Obtained by Inverse Iteration

 Displacement from previous Coordinates is:  0.470687
 -----------------------------------------------------------------
             Standard Nuclear Orientation (Angstroms)
    I     Atom           X                Y                Z
 -----------------------------------------------------------------
    1      N       2.3945318406     0.4944130104     0.1050319275
    2      C       3.0985755545    -0.0336002462     1.1487353774
    3      C       3.9431525556    -1.1069948881     0.9055744326
    4      C       4.0807966806    -1.6235757212    -0.3743155380
    5      C       3.3737792236    -1.0478616743    -1.4239772230
    6      C       2.5493441673     0.0100870464    -1.1556077769
    7      C       2.9986618251     0.5500191784     2.5263885917
    8      C       1.4803102754     1.6617767169     0.2743849100
    9      N      -1.9919253214     0.4321180657     0.9851354984
   10      C      -2.5994211260     0.5527135298    -0.2165253635
   11      N      -1.9572521177     1.0415013611    -1.2538202127
```

```
  12      C      -0.6867371304     1.4389444906    -1.0960397009
  13      C       0.0188413328     1.2964597375     0.1295360720
  14      C      -0.7026793453     0.7864059855     1.1671698366
  15      C      -4.0152889182     0.1047351158    -0.3627285439
  16      C      -4.1129312139    -1.3009453260    -0.9836029214
  17      C      -3.5802608474    -2.4030038015    -0.0730077175
  18      N      -0.0633305885     1.9279164948    -2.2230479370
  19      H       4.5109590850    -1.5345602716     1.7239670104
  20      H       4.7489852295    -2.4595477088    -0.5573877244
  21      H       3.4759795681    -1.4119534520    -2.4388806964
  22      H       1.9958031384     0.5064113036    -1.9431785468
  23      H       3.5745200364    -0.0527926868     3.2274657104
  24      H       1.9687592230     0.5748755037     2.8904804910
  25      H       3.4062107072     1.5652681606     2.5515031876
  26      H       1.7781708817     2.4022861265    -0.4675824373
  27      H       1.6604577390     2.1222507756     1.2432836647
  28      H      -2.5326532442     0.0544694573     1.7594424777
  29      H      -0.3091558757     0.6359429977     2.1654008126
  30      H      -4.5157185027     0.1192118969     0.6114362126
  31      H      -4.5290765379     0.8273585836    -1.0014358752
  32      H      -3.5848301407    -1.3175006444    -1.9418447966
  33      H      -5.1669042388    -1.4883836525    -1.2021126793
  34      H      -2.5025871671    -2.3052635865     0.1034234303
  35      H      -3.7440128537    -3.3823785165    -0.5258541625
  36      H      -4.0904620804    -2.3980686753     0.8954542470
  37      H       0.3291605819     2.8620588735    -2.1570213646
  38      H      -0.7017723959     1.8992064398    -3.0158426726
 ----------------------------------------------------------------
 Nuclear Repulsion Energy =        1313.60797859 hartrees
 There are       65 alpha and       65 beta electrons

-------------------------------------------------
-  Entering fldman on Sun Oct 13 12:19:52 2024  -
-------------------------------------------------

 Applying Cartesian multipole field
    Component          Value
    ---------          -----
     (2,0,0)        1.00000E-11
     (0,2,0)        2.00000E-11
     (0,0,2)       -3.00000E-11
 Nucleus-field energy     =     0.0000000304 hartrees

-------------------------------------------------
-  Entering gesman on Sun Oct 13 12:19:52 2024  -
-------------------------------------------------

 Requested basis set is 6-311+G(d,p)
 There are 188 shells and 516 basis functions
 A cutoff of  1.0D-12 yielded  12892 shell pairs
 There are    102219 function pairs (    108926 Cartesian)
 Smallest overlap matrix eigenvalue = 2.05E-06
 Linear dependence detected in AO basis
 Tighter screening thresholds may be required for diffuse basis sets
 Use S2THRESH > 12 and THRESH = 14 in case of SCF convergence issues
 Number of orthogonalized atomic orbitals = 510
 Maximum deviation from orthogonality = 1.939E-11
 Guess MOs from SCF MO coefficient file
 Reading MOs from coefficient file
 Reading MOs from coefficient file

-------------------------------------------------
-  Entering scfman on Sun Oct 13 12:19:53 2024  -
-------------------------------------------------

 Long-range K will be added via erf
 Coulomb attenuation parameter = 0.2 bohr**(-1)
 A restricted hybrid HF-DFT SCF calculation will be
 performed using Pulay DIIS + Geometric Direct Minimization
 Exchange:     0.2220 Hartree-Fock + 1.0000 wB97X-D + LR-HF
```

```
 Correlation:  1.0000 wB97X-D
 Using Euler-Maclaurin-Lebedev (75,302) quadrature formula
 Dispersion:   Grimme D
 SCF converges when RMS gradient is below 1.0E-07
 Geometry optimization detected.  Setting ReadMinima to 0
 Setting SaveMinima to 0
 ---------------------------------------
  Cycle       Energy         DIIS Error
 ---------------------------------------
    1    -764.0672649573      8.64E-04
    2    -763.9581578683      1.69E-04
    3    -763.9586296320      1.50E-04
    4    -763.9592454102      2.52E-05
    5    -763.9592674678      1.23E-05
    6    -763.9592739035      4.48E-06
    7    -763.9592749965      1.81E-06
    8    -763.9592751901      5.64E-07
    9    -763.9592752193      3.01E-07
   10    -763.9592752238      9.30E-08 Convergence criterion met
 ---------------------------------------
 SCF time:  CPU 243.80 s  wall 566.24 s
 SCF   energy in the final basis set = -763.95927522
 Total energy in the final basis set = -763.95927522

-------------------------------------------------
-  Entering anlman on Sun Oct 13 12:29:19 2024  -
-------------------------------------------------



 --------------------------------------------------------------

                   Orbital Energies (a.u.)
 --------------------------------------------------------------

 Alpha MOs
 -- Occupied --
-14.8129 -14.8044 -14.7241 -14.7194 -10.6786 -10.6709 -10.6561 -10.6370
-10.6335 -10.6194 -10.6111 -10.5800 -10.5675 -10.5675 -10.5493 -10.5323
-10.5001 -10.4760  -1.4264  -1.3995  -1.3451  -1.2768  -1.2301  -1.2013
 -1.1839  -1.1454  -1.1040  -1.0807  -1.0621  -1.0547  -1.0274  -1.0108
 -0.9880  -0.9537  -0.9478  -0.9110  -0.9038  -0.8920  -0.8800  -0.8645
 -0.8482  -0.8425  -0.8366  -0.8175  -0.8109  -0.7940  -0.7883  -0.7860
 -0.7747  -0.7557  -0.7491  -0.7456  -0.7386  -0.7275  -0.7239  -0.7131
 -0.7063  -0.6751  -0.6569  -0.6500  -0.6420  -0.6336  -0.6300  -0.6223
 -0.6173
 -- Virtual --
 -0.2750  -0.2681  -0.2540  -0.2210  -0.1757  -0.1639  -0.1456  -0.1444
 -0.1341  -0.1261  -0.1201  -0.1143  -0.1081  -0.1058  -0.0999  -0.0922
 -0.0884  -0.0836  -0.0804  -0.0786  -0.0775  -0.0719  -0.0703  -0.0649
 -0.0626  -0.0598  -0.0584  -0.0519  -0.0489  -0.0461  -0.0431  -0.0363
 -0.0318  -0.0292  -0.0279  -0.0229  -0.0202  -0.0150  -0.0132  -0.0104
 -0.0068  -0.0052  -0.0001   0.0023   0.0054   0.0105   0.0124   0.0157
  0.0205   0.0223   0.0272   0.0290   0.0292   0.0329   0.0376   0.0399
  0.0426   0.0437   0.0482   0.0532   0.0541   0.0569   0.0607   0.0637
  0.0659   0.0691   0.0719   0.0747   0.0764   0.0776   0.0814   0.0849
  0.0928   0.0965   0.0997   0.1013   0.1073   0.1110   0.1122   0.1146
  0.1184   0.1236   0.1280   0.1292   0.1330   0.1367   0.1418   0.1450
  0.1469   0.1555   0.1576   0.1600   0.1663   0.1683   0.1721   0.1738
  0.1756   0.1777   0.1842   0.1940   0.1964   0.2055   0.2205   0.2239
  0.2315   0.2338   0.2519   0.2638   0.2802   0.2910   0.2944   0.3118
  0.3224   0.3293   0.3343   0.3421   0.3492   0.3502   0.3632   0.3670
  0.3707   0.3772   0.3833   0.3858   0.3971   0.4038   0.4223   0.4233
  0.4266   0.4338   0.4413   0.4493   0.4617   0.4658   0.4666   0.4738
  0.4870   0.4913   0.4974   0.5013   0.5073   0.5108   0.5147   0.5179
  0.5223   0.5260   0.5316   0.5322   0.5394   0.5461   0.5524   0.5531
  0.5586   0.5630   0.5687   0.5749   0.5814   0.5832   0.5967   0.6007
  0.6036   0.6120   0.6162   0.6291   0.6336   0.6473   0.6579   0.6604
  0.6707   0.6741   0.6777   0.6851   0.6900   0.6920   0.7022   0.7084
  0.7132   0.7205   0.7369   0.7462   0.7559   0.7608   0.7664   0.7732
  0.7875   0.8002   0.8038   0.8101   0.8253   0.8447   0.8481   0.8514
```

| | | | | | | | |
|---|---|---|---|---|---|---|---|
| 0.8590 | 0.8738 | 0.8751 | 0.8885 | 0.8941 | 0.8988 | 0.9179 | 0.9300 |
| 0.9334 | 0.9554 | 0.9659 | 0.9766 | 0.9942 | 1.0035 | 1.0185 | 1.0371 |
| 1.0549 | 1.0674 | 1.0852 | 1.1018 | 1.1080 | 1.1734 | 1.1747 | 1.1950 |
| 1.2028 | 1.2105 | 1.2251 | 1.2557 | 1.2606 | 1.2682 | 1.2992 | 1.3111 |
| 1.3169 | 1.3264 | 1.3389 | 1.3447 | 1.3464 | 1.3663 | 1.3739 | 1.3771 |
| 1.3830 | 1.3949 | 1.3989 | 1.4043 | 1.4131 | 1.4225 | 1.4311 | 1.4336 |
| 1.4376 | 1.4422 | 1.4570 | 1.4588 | 1.4657 | 1.4713 | 1.4778 | 1.4837 |
| 1.4908 | 1.4945 | 1.5148 | 1.5180 | 1.5231 | 1.5432 | 1.5499 | 1.5571 |
| 1.5639 | 1.5678 | 1.5737 | 1.5804 | 1.5902 | 1.5965 | 1.6023 | 1.6133 |
| 1.6253 | 1.6318 | 1.6468 | 1.6590 | 1.6642 | 1.6711 | 1.6831 | 1.6864 |
| 1.6914 | 1.7015 | 1.7088 | 1.7325 | 1.7336 | 1.7486 | 1.7555 | 1.7781 |
| 1.7825 | 1.7903 | 1.8059 | 1.8172 | 1.8373 | 1.8459 | 1.8592 | 1.8749 |
| 1.8886 | 1.8993 | 1.9032 | 1.9105 | 1.9345 | 1.9544 | 1.9657 | 1.9846 |
| 1.9971 | 2.0063 | 2.0109 | 2.0368 | 2.0602 | 2.0639 | 2.0767 | 2.0982 |
| 2.1115 | 2.1146 | 2.1222 | 2.1403 | 2.1594 | 2.1747 | 2.2091 | 2.2221 |
| 2.2282 | 2.2397 | 2.2434 | 2.2664 | 2.2761 | 2.2851 | 2.2988 | 2.3004 |
| 2.3103 | 2.3142 | 2.3465 | 2.3579 | 2.3687 | 2.3722 | 2.3874 | 2.3936 |
| 2.4069 | 2.4139 | 2.4183 | 2.4418 | 2.4472 | 2.4658 | 2.4765 | 2.4941 |
| 2.4985 | 2.5157 | 2.5268 | 2.5375 | 2.5406 | 2.5469 | 2.5514 | 2.5552 |
| 2.5579 | 2.5725 | 2.5858 | 2.5890 | 2.5916 | 2.5983 | 2.6050 | 2.6220 |
| 2.6243 | 2.6364 | 2.6536 | 2.6713 | 2.6765 | 2.6866 | 2.6907 | 2.6943 |
| 2.7114 | 2.7141 | 2.7293 | 2.7356 | 2.7459 | 2.7527 | 2.7741 | 2.7798 |
| 2.7884 | 2.7996 | 2.8114 | 2.8146 | 2.8446 | 2.8549 | 2.8719 | 2.8916 |
| 2.9023 | 2.9352 | 2.9502 | 2.9861 | 3.0359 | 3.0632 | 3.0943 | 3.1230 |
| 3.1458 | 3.1694 | 3.2491 | 3.2532 | 3.2991 | 3.3284 | 3.3560 | 3.3778 |
| 3.4181 | 3.4767 | 3.5003 | 3.5450 | 3.5648 | 3.5974 | 3.6330 | 3.6421 |
| 3.6648 | 3.6824 | 3.7002 | 3.7110 | 3.7324 | 3.7486 | 3.7656 | 3.7673 |
| 3.8270 | 3.8722 | 3.8979 | 3.9104 | 3.9388 | 4.0128 | 4.0326 | 4.1038 |
| 4.1091 | 4.2002 | 4.3032 | 4.3210 | 4.5721 | 4.6490 | 4.6894 | 4.7110 |
| 4.8795 | 5.0427 | 5.1713 | 23.5254 | 23.5612 | 23.7170 | 23.7231 | 23.7368 |
| 23.7774 | 23.7975 | 23.8307 | 23.8530 | 23.8904 | 23.9072 | 23.9707 | 23.9765 |
| 24.0418 | 35.4242 | 35.4453 | 35.4969 | 35.5921 | | | |

Ground-State Mulliken Net Atomic Charges

| Atom | Charge (a.u.) |
|---|---|
| 1 N | 0.401122 |
| 2 C | 0.170575 |
| 3 C | -0.045920 |
| 4 C | -0.149830 |
| 5 C | -0.172185 |
| 6 C | -0.183828 |
| 7 C | -0.810404 |
| 8 C | -0.268835 |
| 9 N | -0.145659 |
| 10 C | -0.212912 |
| 11 N | 0.042326 |
| 12 C | 0.087983 |
| 13 C | 0.026947 |
| 14 C | 0.113878 |
| 15 C | -0.333794 |
| 16 C | -0.246617 |
| 17 C | -0.528844 |
| 18 N | -0.476739 |
| 19 H | 0.219996 |
| 20 H | 0.233332 |
| 21 H | 0.226729 |
| 22 H | 0.247116 |
| 23 H | 0.230278 |
| 24 H | 0.169990 |
| 25 H | 0.252080 |
| 26 H | 0.230448 |
| 27 H | 0.244498 |
| 28 H | 0.389494 |
| 29 H | 0.204203 |
| 30 H | 0.210941 |
| 31 H | 0.254311 |
| 32 H | 0.201551 |

```
    33 H                    0.224955
    34 H                    0.129419
    35 H                    0.216338
    36 H                    0.180653
    37 H                    0.323051
    38 H                    0.343356
  ----------------------------------------
  Sum of atomic charges =     2.000000

 -----------------------------------------------------------------
                    Cartesian Multipole Moments
 -----------------------------------------------------------------
    Charge (ESU x 10^10)
                9.6064
    Dipole Moment (Debye)
         X       4.8363      Y       3.1010      Z       1.6703
       Tot       5.9830
    Quadrupole Moments (Debye-Ang)
        XX     -12.8277     XY      -4.8297     YY     -76.4969
        XZ       3.1964     YZ      -2.7626     ZZ     -69.1998
    Traceless Quadrupole Moments (Debye-Ang)
       QXX     120.0413    QYY     -70.9664    QZZ     -49.0749
       QXY     -14.4890    QXZ       9.5893    QYZ      -8.2877
    Octopole Moments (Debye-Ang^2)
       XXX      -1.0744    XXY     -96.3525    XYY      13.5963
       YYY     -16.0326    XXZ      33.2495    XYZ       7.1875
       YYZ     -20.6089    XZZ      18.1379    YZZ       9.6809
       ZZZ      30.5829
    Traceless Octopole Moments (Debye-Ang^2)
       XXX    -292.0540    YYY     683.8496    ZZZ      69.7319
       XXY   -1137.1753    XXZ     369.0719    XYY     111.9645
       XYZ     107.8119    XZZ     180.0894    YYZ    -438.8038
       YZZ     453.3258
    Hexadecapole Moments (Debye-Ang^3)
      XXXX   -3613.6981   XXXY    -245.8993   XXYY    -780.3887
      XYYY     -17.6149   YYYY    -931.0759   XXXZ    -117.7238
      XXYZ      64.0647   XYYZ     -73.8918   YYYZ     -28.8265
      XXZZ    -780.3054   XYZZ     -33.7135   YYZZ    -271.4351
      XZZZ    -206.8597   YZZZ     -22.8889   ZZZZ    -826.7421
    Traceless Hexadecapole Moments (Debye-Ang^3)
      XXXX    4935.0248   XXXY  -12444.1809   XXXZ    5570.3910
      XXYY   -1688.7570   XXYZ    6528.2175   XXZZ   -3246.2678
      XYYY   11525.6864   XYYZ   -1781.5093   XYZZ     918.4946
      XZZZ   -3788.8817   YYYY    -623.9684   YYYZ   -3622.4979
      YYZZ    2312.7254   YZZZ   -2905.7195   ZZZZ     933.5424
 -----------------------------------------------------------------

-------------------------------------------------
-  Entering drvman on Sun Oct 13 12:29:19 2024  -
-------------------------------------------------

 Calculating analytic gradient of the SCF energy
 Gradient of SCF Energy
           1           2           3           4           5           6
    1  -0.0031791   0.0001306  -0.0007830  -0.0023357  -0.0014424   0.0023098
    2  -0.0005894  -0.0007153   0.0022115   0.0017658   0.0017859  -0.0006823
    3  -0.0004926  -0.0043113   0.0051844  -0.0021867   0.0005705   0.0018664
           7           8           9          10          11          12
    1   0.0025369  -0.0015488   0.0013749   0.0105179  -0.0084745  -0.0020679
    2  -0.0009531  -0.0009691   0.0005684   0.0028882   0.0013399  -0.0046747
    3   0.0010829  -0.0009572  -0.0029003   0.0066473  -0.0062666   0.0175299
          13          14          15          16          17          18
    1  -0.0027011  -0.0008528   0.0015244  -0.0010145  -0.0002558   0.0165116
    2   0.0002060   0.0002126  -0.0032420   0.0053334  -0.0010751  -0.0016712
    3  -0.0020027  -0.0035996  -0.0017290   0.0008311  -0.0003068  -0.0050872
          19          20          21          22          23          24
    1   0.0016111   0.0007871   0.0007543   0.0019467   0.0000060  -0.0005505
    2  -0.0016316  -0.0008724  -0.0011820  -0.0013421  -0.0001019  -0.0007130
    3  -0.0001204  -0.0005615  -0.0004047  -0.0018033   0.0012541  -0.0003882
          25          26          27          28          29          30
```

```
    1   0.0004687   0.0000515  -0.0005744  -0.0018733  -0.0008980  -0.0004843
    2   0.0000311   0.0008951   0.0020483  -0.0011756  -0.0002961   0.0005404
    3  -0.0004770  -0.0003442   0.0001296   0.0012823   0.0005552   0.0000846
            31          32          33          34          35          36
    1  -0.0020542   0.0010636  -0.0004950  -0.0003080  -0.0004260   0.0000576
    2  -0.0000362  -0.0014956  -0.0016249   0.0005823  -0.0003244   0.0006828
    3   0.0001853   0.0000337  -0.0000537  -0.0000426  -0.0006560   0.0001642
            37          38
    1  -0.0074507  -0.0018828
    2   0.0039125   0.0003638
    3  -0.0023437  -0.0003660
 Max gradient component =       1.753E-02
 RMS gradient           =       3.277E-03
 Gradient time:  CPU 158.95 s  wall 307.75 s

-------------------------------------------------
-  Entering optman on Sun Oct 13 12:34:27 2024  -
-------------------------------------------------

 Geometry Optimization Parameters
   NAtoms,    NIC,     NZ,  NCons,   NDum,   NFix, NCnnct, MaxDiis
       38     272       0       0       0       0       0       0

 Cartesian Hessian Update
 Hessian updated using BFGS update


** GEOMETRY OPTIMIZATION IN DELOCALIZED INTERNAL COORDINATES **
   Searching for a Minimum

   Optimization Cycle:   2

                        Coordinates (Angstroms)
     ATOM                X               Y               Z
      1  N         2.3945318406    0.4944130104    0.1050319275
      2  C         3.0985755545   -0.0336002462    1.1487353774
      3  C         3.9431525556   -1.1069948881    0.9055744326
      4  C         4.0807966806   -1.6235757212   -0.3743155380
      5  C         3.3737792236   -1.0478616743   -1.4239772230
      6  C         2.5493441673    0.0100870464   -1.1556077769
      7  C         2.9986618251    0.5500191784    2.5263885917
      8  C         1.4803102754    1.6617767169    0.2743849100
      9  N        -1.9919253214    0.4321180657    0.9851354984
     10  C        -2.5994211260    0.5527135298   -0.2165253635
     11  N        -1.9572521177    1.0415013611   -1.2538202127
     12  C        -0.6867371304    1.4389444906   -1.0960397009
     13  C         0.0188413328    1.2964597375    0.1295360720
     14  C        -0.7026793453    0.7864059855    1.1671698366
     15  C        -4.0152889182    0.1047351158   -0.3627285439
     16  C        -4.1129312139   -1.3009453260   -0.9836029214
     17  C        -3.5802608474   -2.4030038015   -0.0730077175
     18  N        -0.0633305885    1.9279164948   -2.2230479370
     19  H         4.5109590850   -1.5345602716    1.7239670104
     20  H         4.7489852295   -2.4595477088   -0.5573877244
     21  H         3.4759795681   -1.4119534520   -2.4388806964
     22  H         1.9958031384    0.5064113036   -1.9431785468
     23  H         3.5745200364   -0.0527926868    3.2274657104
     24  H         1.9687592230    0.5748755037    2.8904804910
     25  H         3.4062107072    1.5652681606    2.5515031876
     26  H         1.7781708817    2.4022861265   -0.4675824373
     27  H         1.6604577390    2.1222507756    1.2432836647
     28  H        -2.5326532442    0.0544694573    1.7594424777
     29  H        -0.3091558757    0.6359429977    2.1654008126
     30  H        -4.5157185027    0.1192118969    0.6114362126
     31  H        -4.5290765379    0.8273585836   -1.0014358752
     32  H        -3.5848301407   -1.3175006444   -1.9418447966
     33  H        -5.1669042388   -1.4883836525   -1.2021126793
     34  H        -2.5025871671   -2.3052635865    0.1034234303
     35  H        -3.7440128537   -3.3823785165   -0.5258541625
     36  H        -4.0904620804   -2.3980686753    0.8954542470
```

```
    37  H          0.3291605819    2.8620588735   -2.1570213646
    38  H         -0.7017723959    1.8992064398   -3.0158426726
  Point Group: c1    Number of degrees of freedom:   108


  Energy is   -763.959275224

 Hessian updated using BFGS update
  internal optimization (0)

108 Hessian modes will be used to form the next step
  Hessian Eigenvalues:
     0.001689    0.002579    0.003936    0.006044    0.009615    0.011952
     0.014833    0.018808    0.019625    0.019976    0.021560    0.022197
     0.022620    0.022760    0.024491    0.024667    0.026061    0.026845
     0.027301    0.028194    0.029939    0.031048    0.036067    0.037511
     0.038784    0.042019    0.043236    0.044033    0.045684    0.047514
     0.053323    0.055463    0.056291    0.057694    0.062639    0.078420
     0.085391    0.091656    0.121574    0.121741    0.127047    0.130226
     0.131111    0.132828    0.136130    0.138792    0.143302    0.146619
     0.147111    0.147686    0.148997    0.150501    0.152212    0.152994
     0.153492    0.156117    0.181408    0.206150    0.209012    0.216272
     0.224521    0.230318    0.234513    0.240105    0.247284    0.262330
     0.268210    0.274523    0.284404    0.292916    0.300158    0.300338
     0.300623    0.301158    0.301597    0.302562    0.304033    0.304400
     0.305189    0.305241    0.306364    0.308381    0.311614    0.315758
     0.320885    0.329144    0.332351    0.334350    0.339521    0.343191
     0.347399    0.351092    0.361264    0.370783    0.380508    0.390193
     0.392381    0.402802    0.404706    0.413881    0.416360    0.419441
     0.434658    0.442600    0.477627    0.510085    0.575435    0.743901

 Minimum search - taking simple RFO step
 Searching for Lamda that Minimizes Along All modes
 Value Taken    Lamda =  -0.00738310
 Calculated Step too Large.  Step scaled by  0.581494 !!
 Step Taken.  Stepsize is  0.300000  0.014721

                           Maximum     Tolerance    Cnvgd?
         Gradient          0.009120     0.000800      NO
         Displacement      0.098527     0.001400      NO
         Energy change    -0.010404     0.000228      NO


 New Cartesian Coordinates Obtained by Inverse Iteration

 Displacement from previous Coordinates is:  0.397641
 ----------------------------------------------------------------
             Standard Nuclear Orientation (Angstroms)
    I     Atom           X                Y                Z
 ----------------------------------------------------------------
    1      N       2.3878237788     0.4892829910     0.1093534912
    2      C       3.0947739723    -0.0336288454     1.1510973637
    3      C       3.9401623139    -1.1054777385     0.8981078252
    4      C       4.0736436615    -1.6262626742    -0.3787056227
    5      C       3.3510787852    -1.0563518864    -1.4236794671
    6      C       2.5232046963    -0.0008154436    -1.1505864932
    7      C       2.9912695994     0.5548043216     2.5246941496
    8      C       1.4774369359     1.6552762312     0.2807490983
    9      N      -1.9996547314     0.4396749262     0.9852538250
   10      C      -2.6133441785     0.5591793430    -0.2178189607
   11      N      -1.9622430956     1.0535182111    -1.2466491754
   12      C      -0.6956971338     1.4595136442    -1.1043332049
   13      C       0.0142218700     1.2970085528     0.1244659339
   14      C      -0.7062416353     0.7879643856     1.1651467004
   15      C      -4.0229482634     0.1051305393    -0.3625566146
   16      C      -4.1022261472    -1.3071011644    -0.9828007346
   17      C      -3.5475602864    -2.4001111054    -0.0745669485
   18      N      -0.0875198687     1.9333340452    -2.2264416571
   19      H       4.5075246369    -1.5229854040     1.7197604245
   20      H       4.7420336278    -2.4593070784    -0.5622822885
```

```
   21      H       3.4378551136   -1.4187679256   -2.4392847200
   22      H       1.9480643277    0.4933757722   -1.9230043752
   23      H       3.5760107158   -0.0449368183    3.2188286851
   24      H       1.9650523015    0.5764779710    2.8978081269
   25      H       3.3921755720    1.5724728150    2.5510715457
   26      H       1.7804920139    2.3977294891   -0.4546662603
   27      H       1.6555835501    2.1022358016    1.2543531433
   28      H      -2.5349650700    0.0642570409    1.7609375731
   29      H      -0.3049703392    0.6341064718    2.1594682508
   30      H      -4.5232355972    0.1129003885    0.6110056593
   31      H      -4.5336508308    0.8241438553   -1.0052480868
   32      H      -3.5831658039   -1.3068681586   -1.9452291798
   33      H      -5.1550228875   -1.4998123386   -1.1963490718
   34      H      -2.4699795657   -2.2898435254    0.0976031282
   35      H      -3.6967291520   -3.3820728440   -0.5247358854
   36      H      -4.0537795411   -2.4071105943    0.8958516053
   37      H       0.4579852857    2.7836335406   -2.1704154121
   38      H      -0.7234586307    1.9654332075   -3.0162023707
 ----------------------------------------------------------------
 Nuclear Repulsion Energy =        1314.49982603 hartrees
 There are       65 alpha and       65 beta electrons

--------------------------------------------------
-  Entering fldman on Sun Oct 13 12:34:27 2024  -
--------------------------------------------------

 Applying Cartesian multipole field
    Component          Value
    ---------          -----
     (2,0,0)        1.00000E-11
     (0,2,0)        2.00000E-11
     (0,0,2)       -3.00000E-11
 Nucleus-field energy     =     0.0000000302 hartrees

--------------------------------------------------
-  Entering gesman on Sun Oct 13 12:34:27 2024  -
--------------------------------------------------

 Requested basis set is 6-311+G(d,p)
 There are 188 shells and 516 basis functions
 A cutoff of  1.0D-12 yielded  12904 shell pairs
 There are    102300 function pairs (    109019 Cartesian)
 Smallest overlap matrix eigenvalue = 2.08E-06
 Linear dependence detected in AO basis
 Tighter screening thresholds may be required for diffuse basis sets
 Use S2THRESH > 12 and THRESH = 14 in case of SCF convergence issues
 Number of orthogonalized atomic orbitals = 510
 Maximum deviation from orthogonality = 1.065E-11
 Guess MOs from SCF MO coefficient file
 Reading MOs from coefficient file
 Reading MOs from coefficient file

--------------------------------------------------
-  Entering scfman on Sun Oct 13 12:34:27 2024  -
--------------------------------------------------

 Long-range K will be added via erf
 Coulomb attenuation parameter = 0.2 bohr**(-1)
 A restricted hybrid HF-DFT SCF calculation will be
 performed using Pulay DIIS + Geometric Direct Minimization
 Exchange:     0.2220 Hartree-Fock + 1.0000 wB97X-D + LR-HF
 Correlation:  1.0000 wB97X-D
 Using Euler-Maclaurin-Lebedev (75,302) quadrature formula
 Dispersion:   Grimme D
 SCF converges when RMS gradient is below 1.0E-07
 Geometry optimization detected.  Setting ReadMinima to 0
 Setting SaveMinima to 0
 ---------------------------------------
  Cycle       Energy         DIIS Error
 ---------------------------------------
```

```
    1    -763.9819892716      4.13E-04
    2    -763.9622229078      1.02E-04
    3    -763.9624004669      9.52E-05
    4    -763.9626602947      1.51E-05
    5    -763.9626707870      6.53E-06
    6    -763.9626727048      3.17E-06
    7    -763.9626731984      9.97E-07
    8    -763.9626732515      4.65E-07
    9    -763.9626732634      1.35E-07
   10    -763.9626732647      6.59E-08 Convergence criterion met
 ---------------------------------------
 SCF time:  CPU 298.39 s  wall 624.04 s
 SCF   energy in the final basis set = -763.96267326
 Total energy in the final basis set = -763.96267326

-------------------------------------------------
-  Entering anlman on Sun Oct 13 12:44:51 2024  -
-------------------------------------------------



 --------------------------------------------------------------

                    Orbital Energies (a.u.)
 --------------------------------------------------------------

 Alpha MOs
 -- Occupied --
-14.8130 -14.8000 -14.7291 -14.7153 -10.6786 -10.6693 -10.6537 -10.6377
-10.6333 -10.6207 -10.6095 -10.5810 -10.5691 -10.5682 -10.5497 -10.5311
-10.4999 -10.4759  -1.4226  -1.4011  -1.3466  -1.2772  -1.2299  -1.2007
 -1.1853  -1.1449  -1.1031  -1.0819  -1.0600  -1.0554  -1.0287  -1.0106
 -0.9886  -0.9529  -0.9475  -0.9131  -0.9054  -0.8945  -0.8811  -0.8653
 -0.8482  -0.8430  -0.8378  -0.8178  -0.8123  -0.7944  -0.7888  -0.7869
 -0.7754  -0.7557  -0.7499  -0.7461  -0.7426  -0.7265  -0.7251  -0.7152
 -0.7058  -0.6740  -0.6567  -0.6481  -0.6418  -0.6332  -0.6282  -0.6199
 -0.6179
 -- Virtual --
 -0.2715  -0.2674  -0.2524  -0.2222  -0.1747  -0.1642  -0.1450  -0.1438
 -0.1345  -0.1263  -0.1199  -0.1139  -0.1076  -0.1058  -0.0994  -0.0924
 -0.0888  -0.0834  -0.0801  -0.0783  -0.0772  -0.0721  -0.0707  -0.0647
 -0.0631  -0.0598  -0.0584  -0.0513  -0.0490  -0.0457  -0.0425  -0.0357
 -0.0322  -0.0293  -0.0285  -0.0224  -0.0204  -0.0148  -0.0131  -0.0099
 -0.0062  -0.0048  -0.0003   0.0022   0.0057   0.0102   0.0131   0.0157
  0.0207   0.0225   0.0272   0.0289   0.0298   0.0334   0.0365   0.0400
  0.0427   0.0435   0.0490   0.0537   0.0542   0.0561   0.0624   0.0639
  0.0654   0.0689   0.0711   0.0752   0.0764   0.0781   0.0811   0.0856
  0.0929   0.0965   0.1001   0.1024   0.1065   0.1115   0.1129   0.1154
  0.1189   0.1242   0.1278   0.1298   0.1331   0.1365   0.1430   0.1445
  0.1484   0.1561   0.1573   0.1603   0.1672   0.1688   0.1736   0.1741
  0.1762   0.1793   0.1845   0.1950   0.1962   0.2062   0.2212   0.2245
  0.2324   0.2335   0.2480   0.2648   0.2808   0.2899   0.2943   0.3115
  0.3223   0.3289   0.3332   0.3425   0.3486   0.3492   0.3633   0.3681
  0.3694   0.3779   0.3824   0.3868   0.3981   0.4055   0.4241   0.4256
  0.4298   0.4341   0.4397   0.4481   0.4622   0.4654   0.4691   0.4762
  0.4884   0.4933   0.4980   0.5020   0.5091   0.5107   0.5173   0.5183
  0.5241   0.5271   0.5319   0.5333   0.5384   0.5454   0.5493   0.5526
  0.5587   0.5631   0.5690   0.5752   0.5816   0.5847   0.5977   0.6011
  0.6035   0.6120   0.6164   0.6288   0.6342   0.6478   0.6572   0.6611
  0.6709   0.6746   0.6781   0.6833   0.6873   0.6933   0.7028   0.7096
  0.7124   0.7203   0.7351   0.7483   0.7577   0.7619   0.7660   0.7739
  0.7880   0.8017   0.8072   0.8108   0.8257   0.8458   0.8490   0.8525
  0.8611   0.8738   0.8800   0.8909   0.8953   0.8989   0.9196   0.9306
  0.9341   0.9553   0.9665   0.9740   0.9925   1.0022   1.0194   1.0411
  1.0535   1.0682   1.0839   1.1011   1.1061   1.1746   1.1776   1.1919
  1.2039   1.2080   1.2290   1.2495   1.2588   1.2674   1.2993   1.3119
  1.3172   1.3220   1.3387   1.3446   1.3474   1.3662   1.3747   1.3756
  1.3850   1.3933   1.3988   1.4047   1.4183   1.4200   1.4312   1.4335
  1.4365   1.4421   1.4593   1.4624   1.4638   1.4718   1.4788   1.4857
  1.4913   1.4935   1.5152   1.5197   1.5226   1.5429   1.5501   1.5557
  1.5644   1.5682   1.5745   1.5811   1.5908   1.5952   1.6084   1.6157
```

| | | | | | | | |
|---|---|---|---|---|---|---|---|
| 1.6232 | 1.6315 | 1.6474 | 1.6592 | 1.6649 | 1.6741 | 1.6827 | 1.6872 |
| 1.6980 | 1.7033 | 1.7102 | 1.7330 | 1.7383 | 1.7482 | 1.7578 | 1.7779 |
| 1.7803 | 1.7886 | 1.8088 | 1.8183 | 1.8359 | 1.8461 | 1.8622 | 1.8823 |
| 1.8888 | 1.9001 | 1.9016 | 1.9176 | 1.9351 | 1.9470 | 1.9610 | 1.9810 |
| 1.9995 | 2.0002 | 2.0102 | 2.0388 | 2.0565 | 2.0639 | 2.0776 | 2.0977 |
| 2.1111 | 2.1151 | 2.1245 | 2.1414 | 2.1611 | 2.1767 | 2.2073 | 2.2222 |
| 2.2275 | 2.2410 | 2.2442 | 2.2619 | 2.2778 | 2.2865 | 2.2979 | 2.3013 |
| 2.3051 | 2.3140 | 2.3470 | 2.3578 | 2.3703 | 2.3732 | 2.3873 | 2.3937 |
| 2.4099 | 2.4142 | 2.4184 | 2.4412 | 2.4471 | 2.4597 | 2.4749 | 2.4913 |
| 2.5019 | 2.5170 | 2.5265 | 2.5382 | 2.5392 | 2.5451 | 2.5520 | 2.5563 |
| 2.5593 | 2.5730 | 2.5874 | 2.5921 | 2.5927 | 2.5947 | 2.6054 | 2.6221 |
| 2.6277 | 2.6377 | 2.6570 | 2.6756 | 2.6764 | 2.6844 | 2.6922 | 2.6983 |
| 2.7091 | 2.7159 | 2.7304 | 2.7360 | 2.7484 | 2.7571 | 2.7732 | 2.7852 |
| 2.7881 | 2.8012 | 2.8101 | 2.8199 | 2.8477 | 2.8553 | 2.8736 | 2.8918 |
| 2.9042 | 2.9355 | 2.9531 | 2.9873 | 3.0406 | 3.0632 | 3.1056 | 3.1438 |
| 3.1481 | 3.1710 | 3.2472 | 3.2543 | 3.3011 | 3.3297 | 3.3613 | 3.3820 |
| 3.4195 | 3.4765 | 3.5050 | 3.5473 | 3.5662 | 3.5977 | 3.6380 | 3.6401 |
| 3.6683 | 3.6867 | 3.7002 | 3.7130 | 3.7356 | 3.7501 | 3.7684 | 3.7692 |
| 3.8309 | 3.8671 | 3.8914 | 3.9122 | 3.9356 | 3.9976 | 4.0314 | 4.0997 |
| 4.1065 | 4.1996 | 4.3011 | 4.3096 | 4.5857 | 4.6548 | 4.6995 | 4.7132 |
| 4.8910 | 5.0456 | 5.1854 | 23.5266 | 23.5685 | 23.7167 | 23.7241 | 23.7384 |
| 23.7746 | 23.7956 | 23.8299 | 23.8565 | 23.8898 | 23.9088 | 23.9677 | 23.9739 |
| 24.0394 | 35.4261 | 35.4479 | 35.5029 | 35.6010 | | | |

Ground-State Mulliken Net Atomic Charges

| Atom | Charge (a.u.) |
|---|---|
| 1 N | 0.404253 |
| 2 C | 0.171432 |
| 3 C | -0.043163 |
| 4 C | -0.155042 |
| 5 C | -0.171710 |
| 6 C | -0.181426 |
| 7 C | -0.814642 |
| 8 C | -0.277491 |
| 9 N | -0.154533 |
| 10 C | -0.212462 |
| 11 N | 0.031352 |
| 12 C | 0.135929 |
| 13 C | 0.014661 |
| 14 C | 0.116439 |
| 15 C | -0.334408 |
| 16 C | -0.243585 |
| 17 C | -0.533184 |
| 18 N | -0.481596 |
| 19 H | 0.219058 |
| 20 H | 0.233750 |
| 21 H | 0.226692 |
| 22 H | 0.240854 |
| 23 H | 0.229757 |
| 24 H | 0.173298 |
| 25 H | 0.252578 |
| 26 H | 0.225967 |
| 27 H | 0.244107 |
| 28 H | 0.387475 |
| 29 H | 0.202051 |
| 30 H | 0.211921 |
| 31 H | 0.253890 |
| 32 H | 0.201690 |
| 33 H | 0.224942 |
| 34 H | 0.130098 |
| 35 H | 0.216136 |
| 36 H | 0.181314 |
| 37 H | 0.320171 |
| 38 H | 0.353427 |

Sum of atomic charges = 2.000000

```
 -----------------------------------------------------------------
                    Cartesian Multipole Moments
 -----------------------------------------------------------------
    Charge (ESU x 10^10)
                 9.6064
    Dipole Moment (Debye)
         X       5.0623      Y       3.1366      Z       1.4690
       Tot       6.1338
    Quadrupole Moments (Debye-Ang)
        XX     -13.0291     XY      -4.4390     YY     -76.6795
        XZ       2.9699     YZ      -3.2155     ZZ     -68.6501
    Traceless Quadrupole Moments (Debye-Ang)
       QXX     119.2715    QYY     -71.6799    QZZ     -47.5915
       QXY     -13.3171    QXZ       8.9096    QYZ      -9.6466
    Octopole Moments (Debye-Ang^2)
       XXX      -0.3293    XXY     -95.3810    XYY      15.3258
       YYY     -18.5799    XXZ      33.2309    XYZ       6.7360
       YYZ     -21.3185    XZZ      19.2838    YZZ      11.5880
       ZZZ      28.9432
    Traceless Octopole Moments (Debye-Ang^2)
       XXX    -313.4624    YYY     642.6578    ZZZ      66.4478
       XXY   -1123.5970    XXZ     375.8969    XYY     127.0464
       XYZ     101.0398    XZZ     186.4160    YYZ    -442.3446
       YZZ     480.9391
    Hexadecapole Moments (Debye-Ang^3)
      XXXX   -3599.3284   XXXY    -242.3341   XXYY    -777.1250
      XYYY     -10.8617   YYYY    -943.6879   XXXZ    -117.9040
      XXYZ      62.0934   XYYZ     -77.5111   YYYZ     -28.1057
      XXZZ    -776.8471   XYZZ     -30.0151   YYZZ    -267.9508
      XZZZ    -208.2157   YZZZ     -28.7812   ZZZZ    -820.7285
    Traceless Hexadecapole Moments (Debye-Ang^3)
      XXXX    4799.2453   XXXY  -12700.5896   XXXZ    5783.4659
      XXYY   -1489.9334   XXYZ    6429.9902   XXZZ   -3309.3118
      XYYY   11604.0133   XYYZ   -2084.2027   XYZZ    1096.5762
      XZZZ   -3699.2631   YYYY   -1166.8116   YYYZ   -3220.5422
      YYZZ    2656.7451   YZZZ   -3209.4480   ZZZZ     652.5668
 -----------------------------------------------------------------

--------------------------------------------------
-  Entering drvman on Sun Oct 13 12:44:51 2024  -
--------------------------------------------------

 Calculating analytic gradient of the SCF energy
 Gradient of SCF Energy
           1           2           3           4           5           6
    1  -0.0014593   0.0006273  -0.0004304   0.0000839  -0.0004780   0.0004416
    2  -0.0005555  -0.0007384   0.0009575  -0.0004812   0.0007002   0.0004617
    3   0.0000236  -0.0008471   0.0015497  -0.0008150   0.0004152  -0.0000956
           7           8           9          10          11          12
    1   0.0007853   0.0008532   0.0011487   0.0010943  -0.0005466  -0.0041900
    2  -0.0004662  -0.0001511   0.0005765  -0.0004426   0.0012019   0.0020227
    3   0.0002951  -0.0003844  -0.0020539   0.0034607  -0.0019713   0.0071164
          13          14          15          16          17          18
    1  -0.0024897   0.0001257   0.0006564  -0.0007892  -0.0003269   0.0120434
    2  -0.0010668  -0.0008767  -0.0007379   0.0019707  -0.0008691  -0.0038522
    3  -0.0030744   0.0015513  -0.0006746   0.0002172  -0.0001878  -0.0031153
          19          20          21          22          23          24
    1   0.0003158  -0.0000588   0.0001569   0.0009229  -0.0002398  -0.0001696
    2  -0.0002199   0.0000417  -0.0001568  -0.0002387   0.0000475   0.0000217
    3  -0.0002793  -0.0001366   0.0001608  -0.0003666  -0.0001778   0.0001043
          25          26          27          28          29          30
    1   0.0000210  -0.0008742  -0.0003267  -0.0006017  -0.0003241  -0.0002518
    2   0.0000797  -0.0006250   0.0007160  -0.0002995  -0.0001132   0.0000828
    3  -0.0000679  -0.0003203  -0.0002276  -0.0001056   0.0003535   0.0000220
          31          32          33          34          35          36
    1  -0.0006353   0.0004255   0.0001532   0.0000888  -0.0000666   0.0000477
    2  -0.0000117  -0.0005851  -0.0003408   0.0000461   0.0001102   0.0002312
    3   0.0005187   0.0001588  -0.0002041   0.0000700  -0.0000296   0.0000998
          37          38
    1  -0.0056492  -0.0000835
```

```
    2   0.0032241   0.0003359
    3  -0.0021748   0.0011928
 Max gradient component =       1.204E-02
 RMS gradient           =       1.776E-03
 Gradient time:  CPU 141.31 s  wall 300.99 s

-------------------------------------------------
-  Entering optman on Sun Oct 13 12:49:52 2024  -
-------------------------------------------------

 Geometry Optimization Parameters
   NAtoms,    NIC,     NZ,  NCons,   NDum,   NFix, NCnnct, MaxDiis
       38     272       0       0       0       0       0       0

 Cartesian Hessian Update
 Hessian updated using BFGS update


** GEOMETRY OPTIMIZATION IN DELOCALIZED INTERNAL COORDINATES **
   Searching for a Minimum

   Optimization Cycle:   3

                       Coordinates (Angstroms)
     ATOM                X               Y                Z
      1  N         2.3878237788    0.4892829910    0.1093534912
      2  C         3.0947739723   -0.0336288454    1.1510973637
      3  C         3.9401623139   -1.1054777385    0.8981078252
      4  C         4.0736436615   -1.6262626742   -0.3787056227
      5  C         3.3510787852   -1.0563518864   -1.4236794671
      6  C         2.5232046963   -0.0008154436   -1.1505864932
      7  C         2.9912695994    0.5548043216    2.5246941496
      8  C         1.4774369359    1.6552762312    0.2807490983
      9  N        -1.9996547314    0.4396749262    0.9852538250
     10  C        -2.6133441785    0.5591793430   -0.2178189607
     11  N        -1.9622430956    1.0535182111   -1.2466491754
     12  C        -0.6956971338    1.4595136442   -1.1043332049
     13  C         0.0142218700    1.2970085528    0.1244659339
     14  C        -0.7062416353    0.7879643856    1.1651467004
     15  C        -4.0229482634    0.1051305393   -0.3625566146
     16  C        -4.1022261472   -1.3071011644   -0.9828007346
     17  C        -3.5475602864   -2.4001111054   -0.0745669485
     18  N        -0.0875198687    1.9333340452   -2.2264416571
     19  H         4.5075246369   -1.5229854040    1.7197604245
     20  H         4.7420336278   -2.4593070784   -0.5622822885
     21  H         3.4378551136   -1.4187679256   -2.4392847200
     22  H         1.9480643277    0.4933757722   -1.9230043752
     23  H         3.5760107158   -0.0449368183    3.2188286851
     24  H         1.9650523015    0.5764779710    2.8978081269
     25  H         3.3921755720    1.5724728150    2.5510715457
     26  H         1.7804920139    2.3977294891   -0.4546662603
     27  H         1.6555835501    2.1022358016    1.2543531433
     28  H        -2.5349650700    0.0642570409    1.7609375731
     29  H        -0.3049703392    0.6341064718    2.1594682508
     30  H        -4.5232355972    0.1129003885    0.6110056593
     31  H        -4.5336508308    0.8241438553   -1.0052480868
     32  H        -3.5831658039   -1.3068681586   -1.9452291798
     33  H        -5.1550228875   -1.4998123386   -1.1963490718
     34  H        -2.4699795657   -2.2898435254    0.0976031282
     35  H        -3.6967291520   -3.3820728440   -0.5247358854
     36  H        -4.0537795411   -2.4071105943    0.8958516053
     37  H         0.4579852857    2.7836335406   -2.1704154121
     38  H        -0.7234586307    1.9654332075   -3.0162023707
   Point Group: c1    Number of degrees of freedom:   108


   Energy is   -763.962673265

 Hessian updated using BFGS update
  internal optimization (0)
```

```
108 Hessian modes will be used to form the next step
  Hessian Eigenvalues:
     0.001689    0.002577    0.003927    0.006048    0.008728    0.011179
     0.014366    0.018693    0.019628    0.019973    0.021553    0.022122
     0.022648    0.022762    0.024488    0.024656    0.025911    0.026793
     0.027090    0.028191    0.029842    0.030704    0.035326    0.036802
     0.038819    0.042383    0.043232    0.044025    0.045649    0.047457
     0.053084    0.054709    0.055609    0.058040    0.062771    0.079158
     0.085485    0.091879    0.121713    0.122334    0.127113    0.130843
     0.131950    0.132827    0.138503    0.139514    0.143245    0.146067
     0.146918    0.147684    0.149001    0.152103    0.152946    0.153269
     0.153652    0.157186    0.194172    0.206291    0.209510    0.216184
     0.224590    0.230304    0.238043    0.246423    0.251352    0.265701
     0.270445    0.274691    0.284502    0.293451    0.300161    0.300582
     0.300863    0.301304    0.301873    0.303013    0.304099    0.304942
     0.305188    0.305285    0.306391    0.309028    0.311772    0.316716
     0.321219    0.332295    0.332832    0.334455    0.339518    0.343127
     0.347178    0.353375    0.360086    0.369803    0.381214    0.390096
     0.390927    0.401148    0.407400    0.412061    0.416621    0.419677
     0.439566    0.445321    0.478788    0.509971    0.581607    0.743008

 Minimum search - taking simple RFO step
 Searching for Lamda that Minimizes Along All modes
 Value Taken    Lamda =  -0.00473371
  dLimit reduced  1.260000000000000E-002  0.300000000000000
  1.817726489358823E-002  3.389132027180773E-003  7.999999999999999E-004
           0
 Calculated Step part too Large.  Step scaled by  0.207952
 Step Taken.  Stepsize is  0.112052  0.018177

                           Maximum     Tolerance    Cnvgd?
         Gradient         0.003389      0.000800      NO
         Displacement     0.038984      0.001400      NO
         Energy change   -0.003398      0.000228      NO


 New Cartesian Coordinates Obtained by Inverse Iteration

 Displacement from previous Coordinates is:  0.149184
 ----------------------------------------------------------------
             Standard Nuclear Orientation (Angstroms)
    I     Atom           X                Y                Z
 ----------------------------------------------------------------
    1      N       2.3854681526     0.4869668099     0.1112584442
    2      C       3.0951968000    -0.0333266337     1.1518729227
    3      C       3.9403565266    -1.1050448885     0.8969544032
    4      C       4.0697667104    -1.6279204842    -0.3790612874
    5      C       3.3412097109    -1.0622258114    -1.4226470922
    6      C       2.5132984535    -0.0072725030    -1.1477461424
    7      C       2.9935568607     0.5570719473     2.5246076634
    8      C       1.4757949457     1.6530282669     0.2838196726
    9      N      -2.0034531807     0.4428942562     0.9835218248
   10      C      -2.6176238963     0.5626146755    -0.2203477830
   11      N      -1.9660715378     1.0574214412    -1.2463479965
   12      C      -0.6989055258     1.4630148293    -1.1063729991
   13      C       0.0131640218     1.2966779455     0.1229783272
   14      C      -0.7088102117     0.7891420201     1.1617639712
   15      C      -4.0253976490     0.1050497633    -0.3654728885
   16      C      -4.0983209385    -1.3096270116    -0.9834690157
   17      C      -3.5339832010    -2.3976267508    -0.0756109480
   18      N      -0.1003680825     1.9412754244    -2.2253028538
   19      H       4.5096356366    -1.5198972206     1.7184325903
   20      H       4.7384827966    -2.4605485571    -0.5629604035
   21      H       3.4223026655    -1.4268917806    -2.4378102319
   22      H       1.9304020026     0.4838984421    -1.9164008460
   23      H       3.5817996217    -0.0404074318     3.2178322931
   24      H       1.9683538102     0.5765860291     2.9003686322
   25      H       3.3921303806     1.5756292590     2.5491936418
   26      H       1.7834253574     2.3984920433    -0.4468339652
```

```
  27      H       1.6525978217     2.0944618633     1.2599842349
  28      H      -2.5373811840     0.0681870649     1.7600297052
  29      H      -0.3063886648     0.6343733082     2.1554680338
  30      H      -4.5258087957     0.1124675048     0.6078498527
  31      H      -4.5365821981     0.8210431965    -1.0109445305
  32      H      -3.5832727357    -1.3056438334    -1.9479214705
  33      H      -5.1509663500    -1.5071258818    -1.1930846844
  34      H      -2.4565153999    -2.2811301758     0.0927908183
  35      H      -3.6784513770    -3.3812225771    -0.5237385856
  36      H      -4.0370549383    -2.4071744421     0.8963798279
  37      H       0.4944211742     2.7567671233    -2.1696282848
  38      H      -0.7360075825     1.9960227693    -3.0134048507
 ----------------------------------------------------------------
 Nuclear Repulsion Energy =        1314.81620884 hartrees
 There are       65 alpha and       65 beta electrons

--------------------------------------------------
-  Entering fldman on Sun Oct 13 12:49:52 2024  -
--------------------------------------------------

 Applying Cartesian multipole field
    Component          Value
    ---------          -----
     (2,0,0)        1.00000E-11
     (0,2,0)        2.00000E-11
     (0,0,2)       -3.00000E-11
 Nucleus-field energy     =     0.0000000302 hartrees

--------------------------------------------------
-  Entering gesman on Sun Oct 13 12:49:52 2024  -
--------------------------------------------------

 Requested basis set is 6-311+G(d,p)
 There are 188 shells and 516 basis functions
 A cutoff of  1.0D-12 yielded  12908 shell pairs
 There are    102338 function pairs (    109062 Cartesian)
 Smallest overlap matrix eigenvalue = 2.08E-06
 Linear dependence detected in AO basis
 Tighter screening thresholds may be required for diffuse basis sets
 Use S2THRESH > 12 and THRESH = 14 in case of SCF convergence issues
 Number of orthogonalized atomic orbitals = 510
 Maximum deviation from orthogonality = 1.306E-11
 Guess MOs from SCF MO coefficient file
 Reading MOs from coefficient file
 Reading MOs from coefficient file

--------------------------------------------------
-  Entering scfman on Sun Oct 13 12:49:53 2024  -
--------------------------------------------------

 Long-range K will be added via erf
 Coulomb attenuation parameter = 0.2 bohr**(-1)
 A restricted hybrid HF-DFT SCF calculation will be
 performed using Pulay DIIS + Geometric Direct Minimization
 Exchange:     0.2220 Hartree-Fock + 1.0000 wB97X-D + LR-HF
 Correlation:  1.0000 wB97X-D
 Using Euler-Maclaurin-Lebedev (75,302) quadrature formula
 Dispersion:   Grimme D
 SCF converges when RMS gradient is below 1.0E-07
 Geometry optimization detected.  Setting ReadMinima to 0
 Setting SaveMinima to 0
 ---------------------------------------
  Cycle       Energy         DIIS Error
 ---------------------------------------
    1    -763.9702367504      1.50E-04
    2    -763.9635316720      3.57E-05
    3    -763.9635544456      3.39E-05
    4    -763.9635878311      5.78E-06
    5    -763.9635894776      2.46E-06
    6    -763.9635897497      1.16E-06
```

```
    7    -763.9635898204      3.85E-07
    8    -763.9635898291      1.64E-07
    9    -763.9635898308      5.20E-08 Convergence criterion met
 ---------------------------------------
 SCF time:  CPU 336.42 s  wall 682.23 s
 SCF   energy in the final basis set = -763.96358983
 Total energy in the final basis set = -763.96358983

--------------------------------------------------
-  Entering anlman on Sun Oct 13 13:01:15 2024  -
--------------------------------------------------



 --------------------------------------------------------------

                   Orbital Energies (a.u.)
 --------------------------------------------------------------


 Alpha MOs
 -- Occupied --
-14.8132 -14.7984 -14.7308 -14.7140 -10.6787 -10.6684 -10.6528 -10.6380
-10.6336 -10.6213 -10.6089 -10.5814 -10.5697 -10.5685 -10.5499 -10.5305
-10.4997 -10.4757  -1.4217  -1.4016  -1.3472  -1.2775  -1.2301  -1.2007
 -1.1858  -1.1447  -1.1029  -1.0823  -1.0595  -1.0555  -1.0292  -1.0104
 -0.9888  -0.9531  -0.9474  -0.9139  -0.9061  -0.8949  -0.8815  -0.8655
 -0.8484  -0.8431  -0.8382  -0.8179  -0.8126  -0.7946  -0.7889  -0.7868
 -0.7756  -0.7557  -0.7502  -0.7463  -0.7437  -0.7263  -0.7254  -0.7156
 -0.7055  -0.6738  -0.6569  -0.6478  -0.6414  -0.6330  -0.6277  -0.6195
 -0.6178
 -- Virtual --
 -0.2708  -0.2667  -0.2515  -0.2227  -0.1744  -0.1644  -0.1449  -0.1437
 -0.1347  -0.1263  -0.1199  -0.1139  -0.1075  -0.1058  -0.0992  -0.0924
 -0.0889  -0.0834  -0.0800  -0.0783  -0.0771  -0.0723  -0.0709  -0.0646
 -0.0633  -0.0598  -0.0584  -0.0512  -0.0491  -0.0457  -0.0424  -0.0356
 -0.0328  -0.0293  -0.0288  -0.0224  -0.0204  -0.0148  -0.0131  -0.0098
 -0.0061  -0.0047  -0.0004   0.0022   0.0059   0.0100   0.0132   0.0158
  0.0206   0.0225   0.0270   0.0287   0.0298   0.0335   0.0360   0.0401
  0.0428   0.0436   0.0492   0.0534   0.0543   0.0559   0.0628   0.0640
  0.0654   0.0691   0.0709   0.0754   0.0764   0.0783   0.0812   0.0858
  0.0929   0.0967   0.1002   0.1030   0.1063   0.1115   0.1133   0.1157
  0.1189   0.1244   0.1275   0.1304   0.1334   0.1363   0.1431   0.1444
  0.1488   0.1562   0.1572   0.1606   0.1675   0.1690   0.1738   0.1744
  0.1764   0.1798   0.1851   0.1951   0.1962   0.2067   0.2217   0.2250
  0.2319   0.2343   0.2463   0.2650   0.2806   0.2893   0.2942   0.3116
  0.3223   0.3288   0.3330   0.3423   0.3488   0.3490   0.3634   0.3675
  0.3697   0.3780   0.3822   0.3870   0.3984   0.4061   0.4239   0.4263
  0.4305   0.4347   0.4391   0.4477   0.4621   0.4651   0.4691   0.4773
  0.4886   0.4933   0.4983   0.5021   0.5091   0.5112   0.5178   0.5196
  0.5251   0.5274   0.5319   0.5335   0.5389   0.5450   0.5495   0.5527
  0.5587   0.5633   0.5690   0.5754   0.5816   0.5855   0.5982   0.6007
  0.6038   0.6119   0.6168   0.6287   0.6344   0.6483   0.6569   0.6613
  0.6706   0.6746   0.6782   0.6838   0.6871   0.6935   0.7032   0.7094
  0.7123   0.7198   0.7341   0.7491   0.7575   0.7623   0.7661   0.7743
  0.7878   0.8020   0.8078   0.8110   0.8260   0.8460   0.8491   0.8530
  0.8617   0.8735   0.8816   0.8916   0.8968   0.9000   0.9205   0.9312
  0.9341   0.9558   0.9661   0.9746   0.9916   1.0020   1.0199   1.0418
  1.0529   1.0678   1.0828   1.0995   1.1061   1.1751   1.1792   1.1913
  1.2040   1.2071   1.2289   1.2471   1.2583   1.2669   1.2991   1.3120
  1.3174   1.3211   1.3382   1.3442   1.3476   1.3660   1.3745   1.3759
  1.3857   1.3931   1.3987   1.4048   1.4179   1.4212   1.4317   1.4335
  1.4368   1.4423   1.4600   1.4629   1.4638   1.4718   1.4790   1.4862
  1.4913   1.4939   1.5155   1.5200   1.5229   1.5424   1.5497   1.5555
  1.5647   1.5681   1.5744   1.5812   1.5904   1.5950   1.6100   1.6164
  1.6227   1.6315   1.6474   1.6601   1.6650   1.6744   1.6815   1.6871
  1.6982   1.7038   1.7131   1.7317   1.7397   1.7486   1.7579   1.7777
  1.7796   1.7880   1.8094   1.8180   1.8351   1.8460   1.8621   1.8842
  1.8887   1.8976   1.9012   1.9227   1.9355   1.9455   1.9609   1.9803
  1.9977   2.0003   2.0103   2.0398   2.0562   2.0643   2.0779   2.0964
  2.1120   2.1149   2.1251   2.1415   2.1621   2.1776   2.2074   2.2222
  2.2277   2.2415   2.2438   2.2605   2.2780   2.2862   2.2973   2.3014
```

| | | | | | | | |
|---|---|---|---|---|---|---|---|
| 2.3030 | 2.3137 | 2.3466 | 2.3581 | 2.3703 | 2.3729 | 2.3876 | 2.3939 |
| 2.4110 | 2.4141 | 2.4184 | 2.4407 | 2.4479 | 2.4585 | 2.4742 | 2.4896 |
| 2.5035 | 2.5175 | 2.5262 | 2.5369 | 2.5397 | 2.5446 | 2.5517 | 2.5562 |
| 2.5599 | 2.5731 | 2.5866 | 2.5923 | 2.5933 | 2.5942 | 2.6063 | 2.6224 |
| 2.6288 | 2.6381 | 2.6592 | 2.6752 | 2.6778 | 2.6845 | 2.6931 | 2.7002 |
| 2.7085 | 2.7180 | 2.7307 | 2.7361 | 2.7496 | 2.7585 | 2.7728 | 2.7869 |
| 2.7879 | 2.8016 | 2.8102 | 2.8219 | 2.8483 | 2.8557 | 2.8732 | 2.8920 |
| 2.9042 | 2.9354 | 2.9541 | 2.9875 | 3.0427 | 3.0632 | 3.1077 | 3.1448 |
| 3.1564 | 3.1756 | 3.2471 | 3.2548 | 3.3011 | 3.3299 | 3.3631 | 3.3842 |
| 3.4198 | 3.4772 | 3.5076 | 3.5481 | 3.5658 | 3.5971 | 3.6379 | 3.6403 |
| 3.6679 | 3.6876 | 3.7003 | 3.7144 | 3.7367 | 3.7502 | 3.7686 | 3.7695 |
| 3.8321 | 3.8621 | 3.8859 | 3.9115 | 3.9355 | 3.9931 | 4.0313 | 4.0985 |
| 4.1065 | 4.1998 | 4.3006 | 4.3090 | 4.5883 | 4.6570 | 4.7031 | 4.7130 |
| 4.8955 | 5.0459 | 5.1874 | 23.5264 | 23.5704 | 23.7181 | 23.7248 | 23.7384 |
| 23.7736 | 23.7961 | 23.8293 | 23.8571 | 23.8895 | 23.9092 | 23.9695 | 23.9757 |
| 24.0391 | 35.4262 | 35.4493 | 35.5054 | 35.6049 | | | |

Ground-State Mulliken Net Atomic Charges

| Atom | Charge (a.u.) |
|---|---|
| 1 N | 0.404496 |
| 2 C | 0.172380 |
| 3 C | -0.041605 |
| 4 C | -0.156304 |
| 5 C | -0.171945 |
| 6 C | -0.180925 |
| 7 C | -0.815554 |
| 8 C | -0.280561 |
| 9 N | -0.156546 |
| 10 C | -0.215081 |
| 11 N | 0.029235 |
| 12 C | 0.149384 |
| 13 C | 0.012528 |
| 14 C | 0.116540 |
| 15 C | -0.334637 |
| 16 C | -0.243741 |
| 17 C | -0.533982 |
| 18 N | -0.481490 |
| 19 H | 0.219023 |
| 20 H | 0.233916 |
| 21 H | 0.226757 |
| 22 H | 0.237707 |
| 23 H | 0.229827 |
| 24 H | 0.174407 |
| 25 H | 0.252556 |
| 26 H | 0.225519 |
| 27 H | 0.244051 |
| 28 H | 0.387006 |
| 29 H | 0.201261 |
| 30 H | 0.211921 |
| 31 H | 0.253537 |
| 32 H | 0.201758 |
| 33 H | 0.224939 |
| 34 H | 0.130199 |
| 35 H | 0.216024 |
| 36 H | 0.181339 |
| 37 H | 0.318813 |
| 38 H | 0.357250 |

Sum of atomic charges = 2.000000

Cartesian Multipole Moments

Charge (ESU x 10^10)
9.6064
Dipole Moment (Debye)
X 5.1541 Y 3.1452 Z 1.4060

```
       Tot        6.1995
    Quadrupole Moments (Debye-Ang)
        XX     -13.1106      XY      -4.3761      YY     -76.7365
        XZ       2.9737      YZ      -3.3219      ZZ     -68.5149
    Traceless Quadrupole Moments (Debye-Ang)
       QXX     119.0303     QYY     -71.8475     QZZ     -47.1828
       QXY     -13.1282     QXZ       8.9210     QYZ      -9.9658
    Octopole Moments (Debye-Ang^2)
       XXX       0.5584     XXY     -95.0029     XYY      15.8642
       YYY     -19.3672     XXZ      33.4376     XYZ       6.7281
       YYZ     -21.5214     XZZ      19.6695     YZZ      12.1032
       ZZZ      28.5939
    Traceless Octopole Moments (Debye-Ang^2)
       XXX    -316.4532     YYY     629.8937     ZZZ      64.3173
       XXY   -1118.2427     XXZ     380.0343     XYY     129.6867
       XYZ     100.9220     XZZ     186.7665     YYZ    -444.3516
       YZZ     488.3490
    Hexadecapole Moments (Debye-Ang^3)
      XXXX   -3595.5877    XXXY    -240.3253    XXYY    -776.0550
      XYYY      -8.5985    YYYY    -948.5324    XXXZ    -117.9771
      XXYZ      61.3502    XYYZ     -78.6890    YYYZ     -27.7816
      XXZZ    -775.2288    XYZZ     -29.1379    YYZZ    -266.8592
      XZZZ    -209.5894    YZZZ     -30.6042    ZZZZ    -818.9067
    Traceless Hexadecapole Moments (Debye-Ang^3)
      XXXX    4687.9122    XXXY  -12721.3812    XXXZ    5893.9047
      XXYY   -1408.9384    XXYZ    6388.2438    XXZZ   -3278.9738
      XYYY   11609.9371    XYYZ   -2168.5113    XYZZ    1111.4441
      XZZZ   -3725.3934    YYYY   -1359.5303    YYYZ   -3077.6542
      YYZZ    2768.4687    YZZZ   -3310.5896    ZZZZ     510.5052
 -----------------------------------------------------------------

--------------------------------------------------
-  Entering drvman on Sun Oct 13 13:01:15 2024  -
--------------------------------------------------

 Calculating analytic gradient of the SCF energy
 Gradient of SCF Energy
           1           2           3           4           5           6
    1  -0.0010470   0.0005789  -0.0003702   0.0001408  -0.0003717   0.0004357
    2  -0.0004736  -0.0007437   0.0007721  -0.0004130   0.0005412   0.0003211
    3   0.0000385  -0.0004297   0.0010559  -0.0005659   0.0002916  -0.0002934
           7           8           9          10          11          12
    1   0.0004814   0.0007860   0.0008429   0.0004623  -0.0000438  -0.0042057
    2  -0.0003299  -0.0000931   0.0005340  -0.0003193   0.0008809   0.0023731
    3   0.0001762  -0.0002714  -0.0019333   0.0021415  -0.0007277   0.0053385
          13          14          15          16          17          18
    1  -0.0021364   0.0006858   0.0005880  -0.0006441  -0.0002972   0.0102457
    2  -0.0013839  -0.0004326  -0.0003357   0.0011853  -0.0006330  -0.0042982
    3  -0.0018335   0.0008720  -0.0004636   0.0000762  -0.0000951  -0.0023140
          19          20          21          22          23          24
    1   0.0001317  -0.0000849   0.0000670   0.0005543  -0.0001965  -0.0000998
    2  -0.0000260   0.0000670  -0.0000138   0.0000309   0.0000279   0.0000691
    3  -0.0002120  -0.0000747   0.0001710  -0.0001936  -0.0002037   0.0001122
          25          26          27          28          29          30
    1  -0.0000084  -0.0007872  -0.0001980  -0.0004251  -0.0001957  -0.0001016
    2   0.0000536  -0.0007323   0.0005193  -0.0002001  -0.0001190   0.0000258
    3  -0.0000342  -0.0003239  -0.0002316  -0.0001796   0.0002603   0.0000329
          31          32          33          34          35          36
    1  -0.0004398   0.0003183   0.0001659   0.0000905  -0.0000286   0.0000465
    2   0.0000204  -0.0003877  -0.0001513  -0.0000164   0.0001148   0.0001475
    3   0.0004432   0.0001561  -0.0002213   0.0000611   0.0000210   0.0000679
          37          38
    1  -0.0049651   0.0000250
    2   0.0031142   0.0003042
    3  -0.0018863   0.0011722
 Max gradient component =       1.025E-02
 RMS gradient           =       1.497E-03
 Gradient time:  CPU 217.86 s  wall 412.18 s

--------------------------------------------------
```

```
-  Entering optman on Sun Oct 13 13:08:07 2024  -
-------------------------------------------------

 Geometry Optimization Parameters
   NAtoms,    NIC,     NZ,  NCons,   NDum,   NFix, NCnnct, MaxDiis
       38     272       0       0       0       0       0       0

 Cartesian Hessian Update
 Hessian updated using BFGS update


** GEOMETRY OPTIMIZATION IN DELOCALIZED INTERNAL COORDINATES **
   Searching for a Minimum

   Optimization Cycle:   4

                    Coordinates (Angstroms)
     ATOM              X               Y               Z
      1  N         2.3854681526    0.4869668099    0.1112584442
      2  C         3.0951968000   -0.0333266337    1.1518729227
      3  C         3.9403565266   -1.1050448885    0.8969544032
      4  C         4.0697667104   -1.6279204842   -0.3790612874
      5  C         3.3412097109   -1.0622258114   -1.4226470922
      6  C         2.5132984535   -0.0072725030   -1.1477461424
      7  C         2.9935568607    0.5570719473    2.5246076634
      8  C         1.4757949457    1.6530282669    0.2838196726
      9  N        -2.0034531807    0.4428942562    0.9835218248
     10  C        -2.6176238963    0.5626146755   -0.2203477830
     11  N        -1.9660715378    1.0574214412   -1.2463479965
     12  C        -0.6989055258    1.4630148293   -1.1063729991
     13  C         0.0131640218    1.2966779455    0.1229783272
     14  C        -0.7088102117    0.7891420201    1.1617639712
     15  C        -4.0253976490    0.1050497633   -0.3654728885
     16  C        -4.0983209385   -1.3096270116   -0.9834690157
     17  C        -3.5339832010   -2.3976267508   -0.0756109480
     18  N        -0.1003680825    1.9412754244   -2.2253028538
     19  H         4.5096356366   -1.5198972206    1.7184325903
     20  H         4.7384827966   -2.4605485571   -0.5629604035
     21  H         3.4223026655   -1.4268917806   -2.4378102319
     22  H         1.9304020026    0.4838984421   -1.9164008460
     23  H         3.5817996217   -0.0404074318    3.2178322931
     24  H         1.9683538102    0.5765860291    2.9003686322
     25  H         3.3921303806    1.5756292590    2.5491936418
     26  H         1.7834253574    2.3984920433   -0.4468339652
     27  H         1.6525978217    2.0944618633    1.2599842349
     28  H        -2.5373811840    0.0681870649    1.7600297052
     29  H        -0.3063886648    0.6343733082    2.1554680338
     30  H        -4.5258087957    0.1124675048    0.6078498527
     31  H        -4.5365821981    0.8210431965   -1.0109445305
     32  H        -3.5832727357   -1.3056438334   -1.9479214705
     33  H        -5.1509663500   -1.5071258818   -1.1930846844
     34  H        -2.4565153999   -2.2811301758    0.0927908183
     35  H        -3.6784513770   -3.3812225771   -0.5237385856
     36  H        -4.0370549383   -2.4071744421    0.8963798279
     37  H         0.4944211742    2.7567671233   -2.1696282848
     38  H        -0.7360075825    1.9960227693   -3.0134048507
   Point Group: c1    Number of degrees of freedom:   108


   Energy is   -763.963589831

 Hessian updated using BFGS update
  internal optimization (0)

108 Hessian modes will be used to form the next step
  Hessian Eigenvalues:
     0.001689    0.002574    0.003914    0.006055    0.007801    0.010907
     0.014297    0.018800    0.019630    0.019977    0.021551    0.022136
     0.022652    0.022758    0.024524    0.024685    0.025889    0.026820
     0.027072    0.028188    0.029607    0.030354    0.034477    0.036603
```

```
    0.038816    0.042543    0.043241    0.044019    0.045624    0.047350
    0.052907    0.054439    0.055583    0.058059    0.062771    0.079678
    0.085562    0.091933    0.121727    0.122340    0.127134    0.130855
    0.132047    0.132863    0.138599    0.141146    0.143088    0.145032
    0.147072    0.147685    0.149638    0.152292    0.152934    0.153595
    0.153737    0.157798    0.194259    0.206431    0.209533    0.216180
    0.226567    0.230328    0.238652    0.246740    0.251446    0.266879
    0.270946    0.275093    0.285671    0.293567    0.300172    0.300600
    0.300826    0.301295    0.301883    0.303040    0.304104    0.304930
    0.305191    0.305284    0.306412    0.309006    0.312119    0.317286
    0.321567    0.332339    0.333405    0.334449    0.339701    0.343168
    0.348308    0.353452    0.362025    0.370104    0.381235    0.390627
    0.397652    0.404095    0.410447    0.414059    0.416740    0.420133
    0.441690    0.447068    0.480052    0.515410    0.582041    0.742251

Minimum search - taking simple RFO step
Searching for Lamda that Minimizes Along All modes
Value Taken    Lamda =  -0.00422260
Calculated Step too Large.  Step scaled by  0.537676
Step Taken.  Stepsize is  0.300000

                            Maximum     Tolerance    Cnvgd?
        Gradient           0.003108      0.000800      NO
        Displacement       0.101230      0.001400      NO
        Energy change     -0.000917      0.000228      NO


New Cartesian Coordinates Obtained by Inverse Iteration

Displacement from previous Coordinates is:  0.412347
----------------------------------------------------------------
            Standard Nuclear Orientation (Angstroms)
   I     Atom           X                Y                Z
----------------------------------------------------------------
   1      N       2.3787229952     0.4802045978     0.1166957868
   2      C       3.0984962598    -0.0308514634     1.1540190394
   3      C       3.9413280970    -1.1046393889     0.8971192168
   4      C       4.0564447022    -1.6376517226    -0.3752650452
   5      C       3.3117646480    -1.0848328369    -1.4152447568
   6      C       2.4861179832    -0.0289683222    -1.1379163335
   7      C       3.0068356964     0.5690064930     2.5231391479
   8      C       1.4710396590     1.6480448283     0.2910748242
   9      N      -2.0122306305     0.4459309229     0.9784902707
  10      C      -2.6276534605     0.5694866287    -0.2273632248
  11      N      -1.9779325972     1.0710356910    -1.2481131143
  12      C      -0.7077866992     1.4767805411    -1.1132986305
  13      C       0.0096655215     1.2969320215     0.1165501465
  14      C      -0.7157622247     0.7884123112     1.1524258361
  15      C      -4.0319674332     0.1027975032    -0.3730776014
  16      C      -4.0893021098    -1.3160734653    -0.9863108966
  17      C      -3.4988985198    -2.3926757761    -0.0820967822
  18      N      -0.1347091511     1.9800445999    -2.2202619593
  19      H       4.5182525709    -1.5121724141     1.7168152912
  20      H       4.7245064796    -2.4707261631    -0.5592727539
  21      H       3.3772896340    -1.4600207505    -2.4276973462
  22      H       1.8860243726     0.4509628913    -1.9003705970
  23      H       3.6047362262    -0.0209688621     3.2147145596
  24      H       1.9848613323     0.5847973517     2.9077473445
  25      H       3.4003483026     1.5895936678     2.5384579380
  26      H       1.7920017151     2.4029015034    -0.4251523539
  27      H       1.6418564103     2.0749957532     1.2744512741
  28      H      -2.5424819130     0.0702414249     1.7562478807
  29      H      -0.3098405768     0.6312846380     2.1442869403
  30      H      -4.5327299164     0.1087600931     0.5998524412
  31      H      -4.5464033870     0.8114051663    -1.0236942372
  32      H      -3.5855404993    -1.3037633425    -1.9564791175
  33      H      -5.1415023017    -1.5278612065    -1.1839372889
  34      H      -2.4216900670    -2.2598298914     0.0745795856
  35      H      -3.6327706124    -3.3797142835    -0.5260982188
  36      H      -3.9917988372    -2.4086309761     0.8950023773
```

```
   37      H       0.5769354261     2.6931445418    -2.1658629560
   38      H      -0.7662270951     2.0926176948    -3.0041566872
 ----------------------------------------------------------------
 Nuclear Repulsion Energy =        1315.07241752 hartrees
 There are       65 alpha and       65 beta electrons

--------------------------------------------------
-  Entering fldman on Sun Oct 13 13:08:07 2024  -
--------------------------------------------------

 Applying Cartesian multipole field
    Component          Value
    ---------          -----
     (2,0,0)        1.00000E-11
     (0,2,0)        2.00000E-11
     (0,0,2)       -3.00000E-11
 Nucleus-field energy     =     0.0000000303 hartrees

--------------------------------------------------
-  Entering gesman on Sun Oct 13 13:08:07 2024  -
--------------------------------------------------

 Requested basis set is 6-311+G(d,p)
 There are 188 shells and 516 basis functions
 A cutoff of  1.0D-12 yielded  12919 shell pairs
 There are    102424 function pairs (    109160 Cartesian)
 Smallest overlap matrix eigenvalue = 2.11E-06
 Linear dependence detected in AO basis
 Tighter screening thresholds may be required for diffuse basis sets
 Use S2THRESH > 12 and THRESH = 14 in case of SCF convergence issues
 Number of orthogonalized atomic orbitals = 510
 Maximum deviation from orthogonality = 2.382E-11
 Guess MOs from SCF MO coefficient file
 Reading MOs from coefficient file
 Reading MOs from coefficient file

--------------------------------------------------
-  Entering scfman on Sun Oct 13 13:08:08 2024  -
--------------------------------------------------

 Long-range K will be added via erf
 Coulomb attenuation parameter = 0.2 bohr**(-1)
 A restricted hybrid HF-DFT SCF calculation will be
 performed using Pulay DIIS + Geometric Direct Minimization
 Exchange:     0.2220 Hartree-Fock + 1.0000 wB97X-D + LR-HF
 Correlation:  1.0000 wB97X-D
 Using Euler-Maclaurin-Lebedev (75,302) quadrature formula
 Dispersion:   Grimme D
 SCF converges when RMS gradient is below 1.0E-07
 Geometry optimization detected.  Setting ReadMinima to 0
 Setting SaveMinima to 0
 ---------------------------------------
  Cycle       Energy         DIIS Error
 ---------------------------------------
    1    -763.9666589406      4.11E-04
    2    -763.9651091523      7.65E-05
    3    -763.9653124785      6.01E-05
    4    -763.9654202102      1.77E-05
    5    -763.9654333080      4.65E-06
    6    -763.9654345742      2.52E-06
    7    -763.9654348802      8.30E-07
    8    -763.9654349257      3.54E-07
    9    -763.9654349329      1.14E-07
   10    -763.9654349339      5.26E-08 Convergence criterion met
 ---------------------------------------
 SCF time:  CPU 404.14 s  wall 782.34 s
 SCF   energy in the final basis set = -763.96543493
 Total energy in the final basis set = -763.96543493

--------------------------------------------------
```

```
-  Entering anlman on Sun Oct 13 13:21:10 2024  -
--------------------------------------------------



 ---------------------------------------------------------------

                    Orbital Energies (a.u.)
 ---------------------------------------------------------------

 Alpha MOs
 -- Occupied --
-14.8139 -14.7951 -14.7348 -14.7114 -10.6788 -10.6662 -10.6511 -10.6388
-10.6343 -10.6230 -10.6078 -10.5823 -10.5711 -10.5692 -10.5502 -10.5291
-10.4989 -10.4755  -1.4191  -1.4026  -1.3484  -1.2775  -1.2302  -1.2003
 -1.1869  -1.1440  -1.1023  -1.0832  -1.0583  -1.0554  -1.0300  -1.0098
 -0.9893  -0.9535  -0.9471  -0.9157  -0.9077  -0.8950  -0.8828  -0.8659
 -0.8490  -0.8433  -0.8387  -0.8184  -0.8131  -0.7954  -0.7894  -0.7858
 -0.7757  -0.7562  -0.7508  -0.7468  -0.7457  -0.7270  -0.7251  -0.7162
 -0.7048  -0.6729  -0.6575  -0.6471  -0.6403  -0.6323  -0.6265  -0.6200
 -0.6158
 -- Virtual --
 -0.2706  -0.2642  -0.2492  -0.2237  -0.1741  -0.1646  -0.1448  -0.1436
 -0.1354  -0.1262  -0.1199  -0.1139  -0.1072  -0.1059  -0.0988  -0.0925
 -0.0892  -0.0833  -0.0802  -0.0786  -0.0766  -0.0726  -0.0713  -0.0647
 -0.0638  -0.0601  -0.0584  -0.0508  -0.0494  -0.0460  -0.0422  -0.0363
 -0.0338  -0.0301  -0.0290  -0.0223  -0.0200  -0.0146  -0.0133  -0.0092
 -0.0059  -0.0044  -0.0010   0.0020   0.0063   0.0097   0.0130   0.0159
  0.0205   0.0223   0.0263   0.0280   0.0299   0.0337   0.0347   0.0404
  0.0430   0.0437   0.0495   0.0521   0.0547   0.0555   0.0626   0.0643
  0.0666   0.0700   0.0709   0.0756   0.0768   0.0791   0.0814   0.0863
  0.0929   0.0973   0.1001   0.1046   0.1055   0.1114   0.1142   0.1163
  0.1189   0.1246   0.1264   0.1320   0.1338   0.1358   0.1431   0.1437
  0.1493   0.1557   0.1576   0.1614   0.1676   0.1690   0.1739   0.1754
  0.1773   0.1809   0.1867   0.1942   0.1972   0.2076   0.2219   0.2261
  0.2303   0.2362   0.2427   0.2652   0.2796   0.2877   0.2941   0.3119
  0.3219   0.3284   0.3328   0.3410   0.3486   0.3498   0.3632   0.3660
  0.3701   0.3781   0.3820   0.3875   0.3992   0.4075   0.4223   0.4278
  0.4304   0.4371   0.4382   0.4469   0.4613   0.4642   0.4678   0.4785
  0.4886   0.4924   0.4993   0.5022   0.5075   0.5123   0.5173   0.5224
  0.5265   0.5287   0.5324   0.5341   0.5439   0.5481   0.5507   0.5538
  0.5586   0.5638   0.5688   0.5765   0.5817   0.5878   0.5989   0.6005
  0.6043   0.6119   0.6185   0.6284   0.6344   0.6494   0.6563   0.6615
  0.6688   0.6738   0.6781   0.6851   0.6863   0.6931   0.7034   0.7085
  0.7134   0.7182   0.7315   0.7509   0.7567   0.7620   0.7672   0.7754
  0.7878   0.8021   0.8073   0.8141   0.8272   0.8458   0.8491   0.8548
  0.8629   0.8725   0.8850   0.8927   0.9003   0.9035   0.9227   0.9329
  0.9349   0.9571   0.9643   0.9761   0.9892   1.0014   1.0210   1.0430
  1.0505   1.0664   1.0799   1.0946   1.1069   1.1758   1.1842   1.1903
  1.2034   1.2054   1.2266   1.2405   1.2572   1.2661   1.2986   1.3113
  1.3180   1.3193   1.3363   1.3425   1.3477   1.3653   1.3742   1.3763
  1.3869   1.3931   1.3984   1.4053   1.4145   1.4253   1.4325   1.4330
  1.4388   1.4427   1.4571   1.4609   1.4663   1.4720   1.4791   1.4862
  1.4892   1.4961   1.5155   1.5210   1.5241   1.5403   1.5479   1.5551
  1.5655   1.5684   1.5739   1.5813   1.5873   1.5946   1.6125   1.6178
  1.6219   1.6313   1.6465   1.6619   1.6649   1.6734   1.6779   1.6865
  1.6962   1.7030   1.7223   1.7271   1.7429   1.7496   1.7578   1.7749
  1.7792   1.7869   1.8096   1.8174   1.8320   1.8453   1.8597   1.8856
  1.8886   1.8934   1.9016   1.9282   1.9438   1.9449   1.9624   1.9781
  1.9927   2.0017   2.0112   2.0425   2.0551   2.0652   2.0788   2.0921
  2.1131   2.1155   2.1264   2.1418   2.1647   2.1795   2.2082   2.2221
  2.2279   2.2417   2.2430   2.2578   2.2778   2.2845   2.2953   2.2971
  2.3014   2.3126   2.3436   2.3587   2.3697   2.3709   2.3880   2.3945
  2.4118   2.4131   2.4200   2.4399   2.4502   2.4572   2.4677   2.4865
  2.5068   2.5189   2.5255   2.5326   2.5413   2.5432   2.5508   2.5553
  2.5610   2.5732   2.5821   2.5918   2.5935   2.5953   2.6071   2.6223
  2.6320   2.6384   2.6642   2.6735   2.6801   2.6844   2.6910   2.7021
  2.7107   2.7265   2.7298   2.7386   2.7518   2.7617   2.7716   2.7870
  2.7899   2.8016   2.8106   2.8271   2.8503   2.8558   2.8724   2.8923
  2.9012   2.9335   2.9571   2.9883   3.0469   3.0632   3.1093   3.1435
  3.1623   3.2031   3.2471   3.2560   3.3005   3.3301   3.3649   3.3911
```

```
  3.4196   3.4769   3.5125   3.5503   3.5643   3.5954   3.6377   3.6398
  3.6667   3.6887   3.7006   3.7184   3.7390   3.7500   3.7686   3.7703
  3.8329   3.8356   3.8767   3.9097   3.9365   3.9856   4.0302   4.0943
  4.1071   4.2006   4.2991   4.3075   4.5927   4.6613   4.7091   4.7117
  4.9059   5.0456   5.1901  23.5255  23.5746  23.7183  23.7260  23.7378
 23.7705  23.7972  23.8275  23.8579  23.8882  23.9099  23.9691  23.9759
 24.0381  35.4260  35.4526  35.5108  35.6116
--------------------------------------------------------------

        Ground-State Mulliken Net Atomic Charges

     Atom                 Charge (a.u.)
  ----------------------------------------
      1 N                    0.406854
      2 C                    0.171664
      3 C                   -0.036319
      4 C                   -0.157587
      5 C                   -0.175036
      6 C                   -0.178920
      7 C                   -0.814677
      8 C                   -0.287858
      9 N                   -0.156870
     10 C                   -0.225269
     11 N                    0.023794
     12 C                    0.183344
     13 C                    0.005431
     14 C                    0.113133
     15 C                   -0.334498
     16 C                   -0.245277
     17 C                   -0.535478
     18 N                   -0.479381
     19 H                    0.219173
     20 H                    0.234265
     21 H                    0.226960
     22 H                    0.227165
     23 H                    0.230003
     24 H                    0.176856
     25 H                    0.252346
     26 H                    0.225610
     27 H                    0.243921
     28 H                    0.385845
     29 H                    0.199717
     30 H                    0.212073
     31 H                    0.252750
     32 H                    0.202120
     33 H                    0.224904
     34 H                    0.130261
     35 H                    0.215808
     36 H                    0.181155
     37 H                    0.315073
     38 H                    0.366945
  ----------------------------------------
  Sum of atomic charges =     2.000000

-----------------------------------------------------------------
                   Cartesian Multipole Moments
-----------------------------------------------------------------
    Charge (ESU x 10^10)
                 9.6064
    Dipole Moment (Debye)
         X       5.3761      Y       3.1424      Z       1.2643
       Tot       6.3542
    Quadrupole Moments (Debye-Ang)
        XX     -13.3328     XY      -4.2728     YY     -76.8111
        XZ       3.0650     YZ      -3.5691     ZZ     -68.2411
    Traceless Quadrupole Moments (Debye-Ang)
       QXX     118.3867    QYY     -72.0484    QZZ     -46.3383
       QXY     -12.8183    QXZ       9.1950    QYZ     -10.7072
    Octopole Moments (Debye-Ang^2)
       XXX       2.9966    XXY     -94.1710    XYY      17.2246
```

```
       YYY     -21.3118    XXZ      34.4202    XYZ       6.9490
       YYZ     -22.2588    XZZ      20.5786    YZZ      13.3778
       ZZZ      27.8276
    Traceless Octopole Moments (Debye-Ang^2)
       XXX    -322.2492    YYY     599.2681    ZZZ      57.5125
       XXY   -1106.2506    XXZ     396.3366    XYY     135.9697
       XYZ     104.2354    XZZ     186.2795    YYZ    -453.8491
       YZZ     506.9826
    Hexadecapole Moments (Debye-Ang^3)
      XXXX   -3586.5259   XXXY    -234.2157   XXYY    -773.4912
      XYYY      -4.1095   YYYY    -964.5258   XXXZ    -118.2250
      XXYZ      59.6921   XYYZ     -81.4961   YYYZ     -27.0156
      XXZZ    -771.5099   XYZZ     -27.0934   YYZZ    -264.0672
      XZZZ    -214.9036   YZZZ     -34.9066   ZZZZ    -814.2924
    Traceless Hexadecapole Moments (Debye-Ang^3)
      XXXX    4400.8833   XXXY  -12648.8108   XXXZ    6244.4870
      XXYY   -1162.8463   XXYZ    6287.5193   XXZZ   -3238.0370
      XYYY   11512.3378   XYYZ   -2337.7161   XYZZ    1136.4730
      XZZZ   -3906.7709   YYYY   -1938.9572   YYYZ   -2777.0769
      YYZZ    3101.8035   YZZZ   -3510.4424   ZZZZ     136.2335
 -----------------------------------------------------------------

--------------------------------------------------
-  Entering drvman on Sun Oct 13 13:21:10 2024  -
--------------------------------------------------

 Calculating analytic gradient of the SCF energy
 Gradient of SCF Energy
           1           2           3           4           5           6
    1  -0.0002735   0.0003077  -0.0002051   0.0001429  -0.0002588   0.0004553
    2  -0.0003720  -0.0006341   0.0003730  -0.0001940   0.0003140   0.0001039
    3  -0.0000014   0.0001211   0.0002432  -0.0000729   0.0000570  -0.0004200
           7           8           9          10          11          12
    1   0.0000484   0.0005870   0.0001750  -0.0007485   0.0006237  -0.0029606
    2  -0.0000708   0.0000629   0.0002550  -0.0008373   0.0005294   0.0030287
    3  -0.0000305  -0.0000053  -0.0009195   0.0005561   0.0001245   0.0010876
          13          14          15          16          17          18
    1  -0.0005233   0.0004815   0.0001617  -0.0002522  -0.0001987   0.0059320
    2  -0.0009495  -0.0003854   0.0002515   0.0001366  -0.0002677  -0.0045053
    3  -0.0005809   0.0011690  -0.0000831  -0.0000906   0.0000438  -0.0011970
          19          20          21          22          23          24
    1  -0.0000714  -0.0000846  -0.0000150  -0.0000353  -0.0001401  -0.0000651
    2   0.0001357   0.0000968   0.0001061   0.0003463   0.0000099   0.0000998
    3  -0.0001012   0.0000038   0.0001024   0.0000508  -0.0002001   0.0000714
          25          26          27          28          29          30
    1  -0.0000176  -0.0005254   0.0000667  -0.0000622  -0.0000042   0.0000729
    2   0.0000190  -0.0006392   0.0001912  -0.0000152  -0.0000936  -0.0000565
    3   0.0000378  -0.0003345  -0.0001545  -0.0001586   0.0000787   0.0000549
          31          32          33          34          35          36
    1  -0.0001895   0.0001741   0.0001255   0.0000771   0.0000200   0.0000227
    2   0.0000307  -0.0000965   0.0001162  -0.0000205   0.0000717   0.0000400
    3   0.0002949   0.0001332  -0.0001888   0.0000397   0.0000739   0.0000184
          37          38
    1  -0.0029861   0.0001431
    2   0.0024476   0.0003716
    3  -0.0008626   0.0010396
 Max gradient component =       5.932E-03
 RMS gradient           =       9.510E-04
 Gradient time:  CPU 192.70 s  wall 395.27 s

--------------------------------------------------
-  Entering optman on Sun Oct 13 13:27:46 2024  -
--------------------------------------------------

 Geometry Optimization Parameters
   NAtoms,    NIC,     NZ,  NCons,   NDum,   NFix, NCnnct, MaxDiis
       38     272       0       0       0       0       0       0

 Cartesian Hessian Update
 Hessian updated using BFGS update
```

```
** GEOMETRY OPTIMIZATION IN DELOCALIZED INTERNAL COORDINATES **
   Searching for a Minimum

   Optimization Cycle:   5

                    Coordinates (Angstroms)
     ATOM                X               Y               Z
      1  N         2.3787229952    0.4802045978    0.1166957868
      2  C         3.0984962598   -0.0308514634    1.1540190394
      3  C         3.9413280970   -1.1046393889    0.8971192168
      4  C         4.0564447022   -1.6376517226   -0.3752650452
      5  C         3.3117646480   -1.0848328369   -1.4152447568
      6  C         2.4861179832   -0.0289683222   -1.1379163335
      7  C         3.0068356964    0.5690064930    2.5231391479
      8  C         1.4710396590    1.6480448283    0.2910748242
      9  N        -2.0122306305    0.4459309229    0.9784902707
     10  C        -2.6276534605    0.5694866287   -0.2273632248
     11  N        -1.9779325972    1.0710356910   -1.2481131143
     12  C        -0.7077866992    1.4767805411   -1.1132986305
     13  C         0.0096655215    1.2969320215    0.1165501465
     14  C        -0.7157622247    0.7884123112    1.1524258361
     15  C        -4.0319674332    0.1027975032   -0.3730776014
     16  C        -4.0893021098   -1.3160734653   -0.9863108966
     17  C        -3.4988985198   -2.3926757761   -0.0820967822
     18  N        -0.1347091511    1.9800445999   -2.2202619593
     19  H         4.5182525709   -1.5121724141    1.7168152912
     20  H         4.7245064796   -2.4707261631   -0.5592727539
     21  H         3.3772896340   -1.4600207505   -2.4276973462
     22  H         1.8860243726    0.4509628913   -1.9003705970
     23  H         3.6047362262   -0.0209688621    3.2147145596
     24  H         1.9848613323    0.5847973517    2.9077473445
     25  H         3.4003483026    1.5895936678    2.5384579380
     26  H         1.7920017151    2.4029015034   -0.4251523539
     27  H         1.6418564103    2.0749957532    1.2744512741
     28  H        -2.5424819130    0.0702414249    1.7562478807
     29  H        -0.3098405768    0.6312846380    2.1442869403
     30  H        -4.5327299164    0.1087600931    0.5998524412
     31  H        -4.5464033870    0.8114051663   -1.0236942372
     32  H        -3.5855404993   -1.3037633425   -1.9564791175
     33  H        -5.1415023017   -1.5278612065   -1.1839372889
     34  H        -2.4216900670   -2.2598298914    0.0745795856
     35  H        -3.6327706124   -3.3797142835   -0.5260982188
     36  H        -3.9917988372   -2.4086309761    0.8950023773
     37  H         0.5769354261    2.6931445418   -2.1658629560
     38  H        -0.7662270951    2.0926176948   -3.0041566872
   Point Group: c1    Number of degrees of freedom:   108


   Energy is   -763.965434934

 Hessian updated using BFGS update
  internal optimization (0)

108 Hessian modes will be used to form the next step
  Hessian Eigenvalues:
     0.001688    0.002572    0.003914    0.006053    0.007928    0.011258
     0.014301    0.018737    0.019627    0.019974    0.021559    0.022157
     0.022625    0.022760    0.024529    0.024703    0.025833    0.026809
     0.026988    0.028184    0.029296    0.030217    0.033915    0.036538
     0.038815    0.042622    0.043254    0.044014    0.045586    0.047264
     0.052587    0.054171    0.055570    0.058047    0.062770    0.079814
     0.085581    0.091957    0.121727    0.122345    0.127134    0.130848
     0.132045    0.132863    0.138568    0.141214    0.143093    0.144663
     0.147080    0.147686    0.149758    0.152327    0.152995    0.153675
     0.153736    0.157997    0.193797    0.206490    0.209572    0.216226
     0.226700    0.230416    0.238632    0.246734    0.251588    0.267083
     0.271314    0.275141    0.285901    0.293701    0.300173    0.300602
     0.300803    0.301289    0.301880    0.303027    0.304104    0.304918
```

```
    0.305193    0.305279    0.306406    0.308978    0.312191    0.317344
    0.321556    0.332324    0.333414    0.334452    0.339723    0.343169
    0.348302    0.353418    0.362413    0.371934    0.381231    0.390666
    0.397651    0.403545    0.411415    0.415675    0.417873    0.422147
    0.444130    0.447059    0.480226    0.515265    0.582629    0.741732

 Minimum search - taking simple RFO step
 Searching for Lamda that Minimizes Along All modes
 Value Taken    Lamda =  -0.00209558
 Calculated Step too Large.  Step scaled by  0.683655
 Step Taken.  Stepsize is  0.300000

                             Maximum     Tolerance    Cnvgd?
         Gradient           0.001825      0.000800      NO
         Displacement       0.098240      0.001400      NO
         Energy change     -0.001845      0.000228      NO


 New Cartesian Coordinates Obtained by Inverse Iteration

 Displacement from previous Coordinates is:  0.468499
 ----------------------------------------------------------------
             Standard Nuclear Orientation (Angstroms)
    I     Atom           X                Y                Z
 ----------------------------------------------------------------
    1      N       2.3708339446     0.4702193203     0.1251894708
    2      C       3.1082001549    -0.0250793941     1.1573287942
    3      C       3.9492894413    -1.1015116787     0.9001508955
    4      C       4.0447713528    -1.6517530656    -0.3658956912
    5      C       3.2784957865    -1.1173311335    -1.4001363928
    6      C       2.4542802220    -0.0599610548    -1.1222706108
    7      C       3.0329981561     0.5892941276     2.5209797629
    8      C       1.4642147983     1.6394930005     0.3000148113
    9      N      -2.0208677774     0.4430685360     0.9701427916
   10      C      -2.6382367904     0.5785621173    -0.2355297353
   11      N      -1.9918715641     1.0900773961    -1.2494048195
   12      C      -0.7182958508     1.4936072662    -1.1163895693
   13      C       0.0046220316     1.2938706797     0.1101986147
   14      C      -0.7228737071     0.7795886918     1.1401107140
   15      C      -4.0391196914     0.1024413221    -0.3838641524
   16      C      -4.0840640961    -1.3195748645    -0.9921371145
   17      C      -3.4640755469    -2.3858613303    -0.0959853364
   18      N      -0.1712583071     2.0339744175    -2.2073138373
   19      H       4.5392695537    -1.4972115625     1.7164453648
   20      H       4.7122593171    -2.4853471104    -0.5494097381
   21      H       3.3251937762    -1.5080072309    -2.4078591883
   22      H       1.8362239984     0.4032422666    -1.8803441143
   23      H       3.6500054374     0.0144969549     3.2087809380
   24      H       2.0170213382     0.5955615907     2.9217157702
   25      H       3.4137299530     1.6147037232     2.5192301572
   26      H       1.7997867712     2.4048403619    -0.3992996696
   27      H       1.6247688806     2.0517130553     1.2911697888
   28      H      -2.5480338056     0.0617947872     1.7467692375
   29      H      -0.3136233459     0.6153234521     2.1295388581
   30      H      -4.5419491607     0.1081457885     0.5879037070
   31      H      -4.5561198323     0.8041749088    -1.0396283310
   32      H      -3.5962840180    -1.3002176076    -1.9702485509
   33      H      -5.1362083230    -1.5467183740    -1.1727561448
   34      H      -2.3861911230    -2.2392276975     0.0416061438
   35      H      -3.5926926698    -3.3757103204    -0.5354258234
   36      H      -3.9399128163    -2.4066398912     0.8894918928
   37      H       0.6339429698     2.6375045862    -2.1673553695
   38      H      -0.7982294580     2.1944539655    -2.9855135239
 ----------------------------------------------------------------
 Nuclear Repulsion Energy =        1315.16447070 hartrees
 There are       65 alpha and       65 beta electrons

 --------------------------------------------------
 -  Entering fldman on Sun Oct 13 13:27:46 2024  -
 --------------------------------------------------
```

```
 Applying Cartesian multipole field
    Component          Value
    ---------          -----
     (2,0,0)        1.00000E-11
     (0,2,0)        2.00000E-11
     (0,0,2)       -3.00000E-11
 Nucleus-field energy     =     0.0000000306 hartrees

--------------------------------------------------
-  Entering gesman on Sun Oct 13 13:27:46 2024  -
--------------------------------------------------

 Requested basis set is 6-311+G(d,p)
 There are 188 shells and 516 basis functions
 A cutoff of  1.0D-12 yielded  12924 shell pairs
 There are    102426 function pairs (    109160 Cartesian)
 Smallest overlap matrix eigenvalue = 2.12E-06
 Linear dependence detected in AO basis
 Tighter screening thresholds may be required for diffuse basis sets
 Use S2THRESH > 12 and THRESH = 14 in case of SCF convergence issues
 Number of orthogonalized atomic orbitals = 510
 Maximum deviation from orthogonality = 2.500E-11
 Guess MOs from SCF MO coefficient file
 Reading MOs from coefficient file
 Reading MOs from coefficient file

--------------------------------------------------
-  Entering scfman on Sun Oct 13 13:27:46 2024  -
--------------------------------------------------

 Long-range K will be added via erf
 Coulomb attenuation parameter = 0.2 bohr**(-1)
 A restricted hybrid HF-DFT SCF calculation will be
 performed using Pulay DIIS + Geometric Direct Minimization
 Exchange:     0.2220 Hartree-Fock + 1.0000 wB97X-D + LR-HF
 Correlation:  1.0000 wB97X-D
 Using Euler-Maclaurin-Lebedev (75,302) quadrature formula
 Dispersion:   Grimme D
 SCF converges when RMS gradient is below 1.0E-07
 Geometry optimization detected.  Setting ReadMinima to 0
 Setting SaveMinima to 0
 ---------------------------------------
  Cycle       Energy         DIIS Error
 ---------------------------------------
    1    -763.9654731474      4.71E-04
    2    -763.9661268146      7.10E-05
    3    -763.9663975293      4.24E-05
    4    -763.9664531963      2.06E-05
    5    -763.9664690277      4.31E-06
    6    -763.9664701951      2.04E-06
    7    -763.9664703895      7.77E-07
    8    -763.9664704275      2.89E-07
    9    -763.9664704326      1.03E-07
   10    -763.9664704336      4.46E-08 Convergence criterion met
 ---------------------------------------
 SCF time:  CPU 325.92 s  wall 618.34 s
 SCF   energy in the final basis set = -763.96647043
 Total energy in the final basis set = -763.96647043

--------------------------------------------------
-  Entering anlman on Sun Oct 13 13:38:04 2024  -
--------------------------------------------------


 --------------------------------------------------------------

                    Orbital Energies (a.u.)
 --------------------------------------------------------------
```

```
 Alpha MOs
 -- Occupied --
-14.8145 -14.7923 -14.7377 -14.7092 -10.6787 -10.6641 -10.6496 -10.6396
-10.6348 -10.6245 -10.6067 -10.5829 -10.5723 -10.5699 -10.5503 -10.5278
-10.4983 -10.4754  -1.4175  -1.4033  -1.3495  -1.2775  -1.2305  -1.2001
 -1.1876  -1.1434  -1.1019  -1.0837  -1.0575  -1.0554  -1.0306  -1.0093
 -0.9898  -0.9544  -0.9468  -0.9174  -0.9089  -0.8948  -0.8838  -0.8660
 -0.8498  -0.8436  -0.8390  -0.8189  -0.8133  -0.7962  -0.7897  -0.7843
 -0.7754  -0.7575  -0.7508  -0.7473  -0.7464  -0.7278  -0.7247  -0.7165
 -0.7042  -0.6723  -0.6581  -0.6467  -0.6393  -0.6317  -0.6258  -0.6207
 -0.6137
 -- Virtual --
 -0.2709  -0.2616  -0.2469  -0.2246  -0.1739  -0.1648  -0.1449  -0.1436
 -0.1359  -0.1262  -0.1200  -0.1138  -0.1069  -0.1058  -0.0986  -0.0926
 -0.0894  -0.0833  -0.0805  -0.0789  -0.0760  -0.0728  -0.0716  -0.0652
 -0.0640  -0.0605  -0.0585  -0.0508  -0.0495  -0.0467  -0.0418  -0.0377
 -0.0337  -0.0311  -0.0289  -0.0224  -0.0197  -0.0145  -0.0133  -0.0084
 -0.0060  -0.0041  -0.0015   0.0017   0.0068   0.0096   0.0127   0.0163
  0.0200   0.0221   0.0260   0.0271   0.0300   0.0334   0.0344   0.0405
  0.0434   0.0437   0.0496   0.0508   0.0548   0.0557   0.0617   0.0648
  0.0676   0.0707   0.0722   0.0757   0.0776   0.0806   0.0823   0.0868
  0.0933   0.0980   0.1000   0.1044   0.1066   0.1112   0.1147   0.1169
  0.1190   0.1241   0.1255   0.1331   0.1343   0.1356   0.1426   0.1428
  0.1493   0.1554   0.1582   0.1626   0.1670   0.1687   0.1738   0.1755
  0.1792   0.1819   0.1878   0.1927   0.1990   0.2078   0.2204   0.2241
  0.2321   0.2377   0.2419   0.2653   0.2781   0.2870   0.2942   0.3124
  0.3220   0.3279   0.3331   0.3389   0.3489   0.3516   0.3633   0.3656
  0.3696   0.3781   0.3825   0.3884   0.4001   0.4091   0.4204   0.4288
  0.4294   0.4360   0.4414   0.4468   0.4599   0.4624   0.4664   0.4785
  0.4882   0.4913   0.4996   0.5022   0.5070   0.5128   0.5164   0.5233
  0.5262   0.5301   0.5332   0.5343   0.5431   0.5508   0.5537   0.5581
  0.5620   0.5671   0.5690   0.5783   0.5827   0.5907   0.5970   0.6045
  0.6054   0.6131   0.6208   0.6287   0.6345   0.6507   0.6559   0.6611
  0.6664   0.6722   0.6780   0.6830   0.6880   0.6930   0.7022   0.7089
  0.7132   0.7205   0.7289   0.7520   0.7558   0.7613   0.7689   0.7764
  0.7870   0.8011   0.8065   0.8198   0.8286   0.8449   0.8490   0.8570
  0.8634   0.8716   0.8873   0.8940   0.9031   0.9084   0.9250   0.9324
  0.9393   0.9575   0.9643   0.9777   0.9875   1.0003   1.0220   1.0435
  1.0486   1.0641   1.0771   1.0898   1.1084   1.1759   1.1872   1.1909
  1.2001   1.2053   1.2178   1.2348   1.2566   1.2670   1.2983   1.3105
  1.3176   1.3197   1.3338   1.3402   1.3479   1.3641   1.3741   1.3752
  1.3872   1.3929   1.3980   1.4060   1.4120   1.4278   1.4315   1.4333
  1.4415   1.4434   1.4467   1.4626   1.4681   1.4734   1.4792   1.4851
  1.4894   1.4994   1.5154   1.5237   1.5263   1.5385   1.5459   1.5553
  1.5655   1.5698   1.5737   1.5805   1.5845   1.5946   1.6131   1.6186
  1.6225   1.6310   1.6438   1.6621   1.6650   1.6701   1.6770   1.6857
  1.6952   1.7016   1.7173   1.7310   1.7446   1.7490   1.7588   1.7702
  1.7824   1.7873   1.8097   1.8191   1.8287   1.8438   1.8590   1.8844
  1.8899   1.8956   1.9047   1.9308   1.9482   1.9511   1.9681   1.9774
  1.9914   2.0030   2.0135   2.0455   2.0557   2.0657   2.0790   2.0901
  2.1138   2.1178   2.1282   2.1418   2.1674   2.1812   2.2112   2.2226
  2.2279   2.2407   2.2447   2.2566   2.2773   2.2824   2.2906   2.2932
  2.3015   2.3107   2.3402   2.3591   2.3659   2.3702   2.3880   2.3956
  2.4102   2.4113   2.4244   2.4399   2.4505   2.4566   2.4610   2.4860
  2.5084   2.5196   2.5251   2.5297   2.5424   2.5432   2.5511   2.5546
  2.5619   2.5735   2.5785   2.5907   2.5940   2.5971   2.6062   2.6218
  2.6352   2.6396   2.6683   2.6721   2.6799   2.6851   2.6873   2.7032
  2.7162   2.7284   2.7315   2.7479   2.7522   2.7646   2.7715   2.7866
  2.7912   2.8018   2.8121   2.8314   2.8525   2.8569   2.8743   2.8926
  2.8958   2.9329   2.9603   2.9895   3.0517   3.0637   3.1126   3.1426
  3.1624   3.2309   3.2470   3.2586   3.2994   3.3307   3.3677   3.4005
  3.4185   3.4779   3.5174   3.5532   3.5632   3.5939   3.6370   3.6396
  3.6652   3.6889   3.7006   3.7234   3.7409   3.7497   3.7685   3.7714
  3.7983   3.8360   3.8735   3.9089   3.9387   3.9832   4.0293   4.0910
  4.1082   4.2019   4.2976   4.3078   4.5949   4.6651   4.7090   4.7165
  4.9156   5.0444   5.1930  23.5246  23.5773  23.7199  23.7282  23.7374
 23.7710  23.7991  23.8257  23.8579  23.8864  23.9103  23.9686  23.9797
 24.0367  35.4253  35.4562  35.5169  35.6184
 --------------------------------------------------------------
```

Ground-State Mulliken Net Atomic Charges

| Atom | Charge (a.u.) |
|---|---|
| 1 N | 0.411490 |
| 2 C | 0.162940 |
| 3 C | -0.027847 |
| 4 C | -0.156751 |
| 5 C | -0.180804 |
| 6 C | -0.175931 |
| 7 C | -0.808165 |
| 8 C | -0.297008 |
| 9 N | -0.153306 |
| 10 C | -0.237070 |
| 11 N | 0.017357 |
| 12 C | 0.218698 |
| 13 C | -0.000087 |
| 14 C | 0.105103 |
| 15 C | -0.333983 |
| 16 C | -0.248191 |
| 17 C | -0.536454 |
| 18 N | -0.478627 |
| 19 H | 0.219374 |
| 20 H | 0.234527 |
| 21 H | 0.227145 |
| 22 H | 0.214854 |
| 23 H | 0.230119 |
| 24 H | 0.179939 |
| 25 H | 0.251183 |
| 26 H | 0.228054 |
| 27 H | 0.243195 |
| 28 H | 0.384454 |
| 29 H | 0.198521 |
| 30 H | 0.212551 |
| 31 H | 0.251751 |
| 32 H | 0.202772 |
| 33 H | 0.225012 |
| 34 H | 0.130432 |
| 35 H | 0.215766 |
| 36 H | 0.180490 |
| 37 H | 0.313523 |
| 38 H | 0.374972 |

Sum of atomic charges = 2.000000

Cartesian Multipole Moments

Charge (ESU x 10^10)

9.6064

Dipole Moment (Debye)

| | | | | | |
|---|---|---|---|---|---|
| X | 5.5540 | Y | 3.0998 | Z | 1.1507 |
| Tot | 6.4637 | | | | |

Quadrupole Moments (Debye-Ang)

| | | | | | |
|---|---|---|---|---|---|
| XX | -13.4323 | XY | -4.2724 | YY | -76.8509 |
| XZ | 3.3751 | YZ | -3.7203 | ZZ | -68.1234 |

Traceless Quadrupole Moments (Debye-Ang)

| | | | | | |
|---|---|---|---|---|---|
| QXX | 118.1096 | QYY | -72.1460 | QZZ | -45.9636 |
| QXY | -12.8172 | QXZ | 10.1253 | QYZ | -11.1608 |

Octopole Moments (Debye-Ang^2)

| | | | | | |
|---|---|---|---|---|---|
| XXX | 5.9885 | XXY | -93.5511 | XYY | 18.2348 |
| YYY | -22.9911 | XXZ | 35.9180 | XYZ | 7.5343 |
| YYZ | -23.2163 | XZZ | 21.2627 | YZZ | 14.4122 |
| ZZZ | 27.1392 | | | | |

Traceless Octopole Moments (Debye-Ang^2)

| | | | | | |
|---|---|---|---|---|---|
| XXX | -319.5467 | YYY | 574.3041 | ZZZ | 48.5193 |
| XXY | -1096.8768 | XXZ | 419.2475 | XYY | 137.0644 |
| XYZ | 113.0148 | XZZ | 182.4823 | YYZ | -467.7668 |
| YZZ | 522.5727 | | | | |

Hexadecapole Moments (Debye-Ang^3)

```
      XXXX   -3578.0349   XXXY    -226.8384   XXYY    -772.0531
      XYYY      -0.0307   YYYY    -981.1824   XXXZ    -118.1708
      XXYZ      58.3932   XYYZ     -84.7555   YYYZ     -27.0995
      XXZZ    -768.7630   XYZZ     -24.4842   YYZZ    -261.1243
      XZZZ    -223.2051   YZZZ     -38.7934   ZZZZ    -809.1520
    Traceless Hexadecapole Moments (Debye-Ang^3)
      XXXX    4252.6690   XXXY  -12507.1353   XXXZ    6767.9794
      XXYY    -984.1613   XXYZ    6231.8778   XXZZ   -3268.5077
      XYYY   11307.6744   XYYZ   -2507.3534   XYZZ    1199.4609
      XZZZ   -4260.6260   YYYY   -2482.0236   YYYZ   -2543.6645
      YYZZ    3466.1849   YZZZ   -3688.2133   ZZZZ    -197.6771
 -----------------------------------------------------------------

 ------------------------------------------------
 -  Entering drvman on Sun Oct 13 13:38:04 2024  -
 ------------------------------------------------

 Calculating analytic gradient of the SCF energy
 Gradient of SCF Energy
            1           2           3           4           5           6
    1   0.0001340  -0.0000570   0.0000214   0.0002179   0.0001355  -0.0000737
    2  -0.0000195  -0.0000677  -0.0001330  -0.0001976  -0.0001627   0.0003550
    3  -0.0000330   0.0004140  -0.0003959   0.0001044   0.0000187  -0.0003348
            7           8           9          10          11          12
    1  -0.0002876   0.0001827  -0.0000661  -0.0004895   0.0004936  -0.0010736
    2  -0.0000120  -0.0000254   0.0000294  -0.0001214  -0.0002583   0.0027265
    3  -0.0000279   0.0001289  -0.0002915  -0.0008114   0.0009309  -0.0013848
           13          14          15          16          17          18
    1  -0.0000756   0.0005850   0.0001383  -0.0001074  -0.0001363   0.0017364
    2  -0.0005899   0.0001532   0.0003238  -0.0003939  -0.0000136  -0.0039261
    3   0.0010071  -0.0001795   0.0000345  -0.0000953   0.0001301   0.0001363
           19          20          21          22          23          24
    1  -0.0001286  -0.0000787  -0.0000111  -0.0000828  -0.0000477  -0.0000419
    2   0.0001104   0.0001218   0.0001122   0.0002936   0.0000083   0.0001006
    3  -0.0000304   0.0000354   0.0000296   0.0001509  -0.0001241   0.0000047
           25          26          27          28          29          30
    1  -0.0000012  -0.0001393   0.0001322   0.0001089   0.0000911   0.0001562
    2  -0.0000174  -0.0004507  -0.0000033   0.0000945  -0.0000414  -0.0000895
    3   0.0000432  -0.0001589  -0.0001078  -0.0001193  -0.0000218   0.0000331
           31          32          33          34          35          36
    1  -0.0000220   0.0000567   0.0000660   0.0000582   0.0000647   0.0000200
    2   0.0000313   0.0000866   0.0001643  -0.0000031   0.0000357   0.0000055
    3   0.0001367   0.0000935  -0.0001483   0.0000292   0.0000756  -0.0000227
           37          38
    1  -0.0016270   0.0001484
    2   0.0010587   0.0007152
    3  -0.0001762   0.0009268
 Max gradient component =       3.926E-03
 RMS gradient           =       5.923E-04
 Gradient time:  CPU 146.81 s  wall 298.03 s

 ------------------------------------------------
 -  Entering optman on Sun Oct 13 13:43:02 2024  -
 ------------------------------------------------

 Geometry Optimization Parameters
   NAtoms,    NIC,     NZ,  NCons,   NDum,   NFix, NCnnct, MaxDiis
       38     272       0       0       0       0       0       0

 Cartesian Hessian Update
 Hessian updated using BFGS update


** GEOMETRY OPTIMIZATION IN DELOCALIZED INTERNAL COORDINATES **
   Searching for a Minimum

   Optimization Cycle:   6

                        Coordinates (Angstroms)
     ATOM                X               Y               Z
```

| | | | | |
|---|---|---|---|---|
| 1 | N | 2.3708339446 | 0.4702193203 | 0.1251894708 |
| 2 | C | 3.1082001549 | -0.0250793941 | 1.1573287942 |
| 3 | C | 3.9492894413 | -1.1015116787 | 0.9001508955 |
| 4 | C | 4.0447713528 | -1.6517530656 | -0.3658956912 |
| 5 | C | 3.2784957865 | -1.1173311335 | -1.4001363928 |
| 6 | C | 2.4542802220 | -0.0599610548 | -1.1222706108 |
| 7 | C | 3.0329981561 | 0.5892941276 | 2.5209797629 |
| 8 | C | 1.4642147983 | 1.6394930005 | 0.3000148113 |
| 9 | N | -2.0208677774 | 0.4430685360 | 0.9701427916 |
| 10 | C | -2.6382367904 | 0.5785621173 | -0.2355297353 |
| 11 | N | -1.9918715641 | 1.0900773961 | -1.2494048195 |
| 12 | C | -0.7182958508 | 1.4936072662 | -1.1163895693 |
| 13 | C | 0.0046220316 | 1.2938706797 | 0.1101986147 |
| 14 | C | -0.7228737071 | 0.7795886918 | 1.1401107140 |
| 15 | C | -4.0391196914 | 0.1024413221 | -0.3838641524 |
| 16 | C | -4.0840640961 | -1.3195748645 | -0.9921371145 |
| 17 | C | -3.4640755469 | -2.3858613303 | -0.0959853364 |
| 18 | N | -0.1712583071 | 2.0339744175 | -2.2073138373 |
| 19 | H | 4.5392695537 | -1.4972115625 | 1.7164453648 |
| 20 | H | 4.7122593171 | -2.4853471104 | -0.5494097381 |
| 21 | H | 3.3251937762 | -1.5080072309 | -2.4078591883 |
| 22 | H | 1.8362239984 | 0.4032422666 | -1.8803441143 |
| 23 | H | 3.6500054374 | 0.0144969549 | 3.2087809380 |
| 24 | H | 2.0170213382 | 0.5955615907 | 2.9217157702 |
| 25 | H | 3.4137299530 | 1.6147037232 | 2.5192301572 |
| 26 | H | 1.7997867712 | 2.4048403619 | -0.3992996696 |
| 27 | H | 1.6247688806 | 2.0517130553 | 1.2911697888 |
| 28 | H | -2.5480338056 | 0.0617947872 | 1.7467692375 |
| 29 | H | -0.3136233459 | 0.6153234521 | 2.1295388581 |
| 30 | H | -4.5419491607 | 0.1081457885 | 0.5879037070 |
| 31 | H | -4.5561198323 | 0.8041749088 | -1.0396283310 |
| 32 | H | -3.5962840180 | -1.3002176076 | -1.9702485509 |
| 33 | H | -5.1362083230 | -1.5467183740 | -1.1727561448 |
| 34 | H | -2.3861911230 | -2.2392276975 | 0.0416061438 |
| 35 | H | -3.5926926698 | -3.3757103204 | -0.5354258234 |
| 36 | H | -3.9399128163 | -2.4066398912 | 0.8894918928 |
| 37 | H | 0.6339429698 | 2.6375045862 | -2.1673553695 |
| 38 | H | -0.7982294580 | 2.1944539655 | -2.9855135239 |

Point Group: c1 Number of degrees of freedom: 108

Energy is -763.966470434

Hessian updated using BFGS update
internal optimization (0)

108 Hessian modes will be used to form the next step
Hessian Eigenvalues:

| | | | | | |
|---|---|---|---|---|---|
| 0.001689 | 0.002568 | 0.003899 | 0.006045 | 0.007600 | 0.011884 |
| 0.014335 | 0.018692 | 0.019624 | 0.019986 | 0.021608 | 0.022232 |
| 0.022559 | 0.022760 | 0.024506 | 0.024721 | 0.025799 | 0.026827 |
| 0.026933 | 0.028184 | 0.029026 | 0.030162 | 0.033515 | 0.036504 |
| 0.038815 | 0.042657 | 0.043260 | 0.044011 | 0.045549 | 0.047165 |
| 0.052244 | 0.053994 | 0.055563 | 0.058036 | 0.062842 | 0.079867 |
| 0.085585 | 0.091997 | 0.121734 | 0.122345 | 0.127134 | 0.130842 |
| 0.132069 | 0.132866 | 0.138562 | 0.141471 | 0.143102 | 0.144619 |
| 0.147177 | 0.147687 | 0.149740 | 0.152386 | 0.153000 | 0.153704 |
| 0.154073 | 0.157951 | 0.193788 | 0.206503 | 0.209589 | 0.216221 |
| 0.227434 | 0.230463 | 0.238623 | 0.246747 | 0.251775 | 0.267137 |
| 0.271197 | 0.275210 | 0.285903 | 0.293680 | 0.300173 | 0.300603 |
| 0.300781 | 0.301288 | 0.301874 | 0.303021 | 0.304104 | 0.304906 |
| 0.305193 | 0.305279 | 0.306400 | 0.308959 | 0.312179 | 0.317338 |
| 0.321629 | 0.332389 | 0.333317 | 0.334462 | 0.339742 | 0.343200 |
| 0.348488 | 0.353823 | 0.362859 | 0.371936 | 0.381310 | 0.390655 |
| 0.397838 | 0.404158 | 0.411425 | 0.415844 | 0.418899 | 0.431866 |
| 0.443988 | 0.450287 | 0.480246 | 0.514971 | 0.582499 | 0.742528 |

Minimum search - taking simple RFO step
Searching for Lamda that Minimizes Along All modes
Value Taken Lamda = -0.00063209

```
 Step Taken.  Stepsize is  0.270991

                          Maximum     Tolerance    Cnvgd?
        Gradient          0.001503     0.000800      NO
        Displacement      0.090220     0.001400      NO
        Energy change    -0.001035     0.000228      NO


 New Cartesian Coordinates Obtained by Inverse Iteration

 Displacement from previous Coordinates is:  0.558998
 ----------------------------------------------------------------
             Standard Nuclear Orientation (Angstroms)
    I     Atom           X                Y                Z
 ----------------------------------------------------------------
    1      N       2.3619892487     0.4556993508     0.1387911972
    2      C       3.1289828816    -0.0135940410     1.1613558428
    3      C       3.9702200054    -1.0897448675     0.9050176282
    4      C       4.0363078118    -1.6651408070    -0.3515943669
    5      C       3.2379511231    -1.1594251671    -1.3762779821
    6      C       2.4143585325    -0.1029307927    -1.0976988952
    7      C       3.0795044200     0.6233926516     2.5158274251
    8      C       1.4553667428     1.6262443565     0.3131619928
    9      N      -2.0312419304     0.4293897852     0.9604798314
   10      C      -2.6497435033     0.5845646245    -0.2439183945
   11      N      -2.0052287254     1.1122201259    -1.2500697125
   12      C      -0.7279881662     1.5111512477    -1.1146984387
   13      C      -0.0024746642     1.2852281806     0.1070907191
   14      C      -0.7319325667     0.7583536463     1.1306057937
   15      C      -4.0492416756     0.1048705148    -0.3979895647
   16      C      -4.0881578285    -1.3184563517    -1.0035203570
   17      C      -3.4390765459    -2.3791776326    -0.1216052352
   18      N      -0.1960863878     2.0953784867    -2.1858812082
   19      H       4.5829791561    -1.4656754409     1.7138870255
   20      H       4.7048292123    -2.4981615064    -0.5352414268
   21      H       3.2594540200    -1.5723015341    -2.3759623979
   22      H       1.7713978534     0.3363645178    -1.8491080731
   23      H       3.7277579691     0.0741173398     3.1959006453
   24      H       2.0742367643     0.6124099510     2.9435959130
   25      H       3.4363568494     1.6568155685     2.4870744391
   26      H       1.8041513346     2.4018523161    -0.3692412901
   27      H       1.6020319853     2.0245328956     1.3119967288
   28      H      -2.5572844459     0.0377786435     1.7325777689
   29      H      -0.3198253189     0.5813454724     2.1168338383
   30      H      -4.5564927300     0.1118361062     0.5715270416
   31      H      -4.5649142663     0.8029993601    -1.0584984528
   32      H      -3.6208977461    -1.2934568063    -1.9914823376
   33      H      -5.1412004459    -1.5577951909    -1.1625265250
   34      H      -2.3590809386    -2.2262453407    -0.0113507805
   35      H      -3.5727187089    -3.3704179076    -0.5565826640
   36      H      -3.8901701608    -2.4022704516     0.8755406035
   37      H       0.6703780141     2.6068623801    -2.1630736244
   38      H      -0.8144971691     2.2813863163    -2.9649427070
 ----------------------------------------------------------------
 Nuclear Repulsion Energy =        1314.72036253 hartrees
 There are       65 alpha and       65 beta electrons

--------------------------------------------------
-  Entering fldman on Sun Oct 13 13:43:02 2024  -
--------------------------------------------------

 Applying Cartesian multipole field
    Component           Value
    ---------           -----
     (2,0,0)        1.00000E-11
     (0,2,0)        2.00000E-11
     (0,0,2)       -3.00000E-11
 Nucleus-field energy     =     0.0000000310 hartrees

--------------------------------------------------
```

```
-  Entering gesman on Sun Oct 13 13:43:03 2024  -
--------------------------------------------------

 Requested basis set is 6-311+G(d,p)
 There are 188 shells and 516 basis functions
 A cutoff of  1.0D-12 yielded  12924 shell pairs
 There are    102466 function pairs (    109206 Cartesian)
 Smallest overlap matrix eigenvalue = 2.10E-06
 Linear dependence detected in AO basis
 Tighter screening thresholds may be required for diffuse basis sets
 Use S2THRESH > 12 and THRESH = 14 in case of SCF convergence issues
 Number of orthogonalized atomic orbitals = 510
 Maximum deviation from orthogonality = 9.441E-12
 Guess MOs from SCF MO coefficient file
 Reading MOs from coefficient file
 Reading MOs from coefficient file

--------------------------------------------------
-  Entering scfman on Sun Oct 13 13:43:03 2024  -
--------------------------------------------------

 Long-range K will be added via erf
 Coulomb attenuation parameter = 0.2 bohr**(-1)
 A restricted hybrid HF-DFT SCF calculation will be
 performed using Pulay DIIS + Geometric Direct Minimization
 Exchange:     0.2220 Hartree-Fock + 1.0000 wB97X-D + LR-HF
 Correlation:  1.0000 wB97X-D
 Using Euler-Maclaurin-Lebedev (75,302) quadrature formula
 Dispersion:   Grimme D
 SCF converges when RMS gradient is below 1.0E-07
 Geometry optimization detected.  Setting ReadMinima to 0
 Setting SaveMinima to 0
 ----------------------------------------
  Cycle       Energy         DIIS Error
 ----------------------------------------
    1    -763.9563283022      5.43E-04
    2    -763.9664308537      7.12E-05
    3    -763.9667839568      3.47E-05
    4    -763.9668243260      2.19E-05
    5    -763.9668415715      5.87E-06
    6    -763.9668430817      2.06E-06
    7    -763.9668432564      8.41E-07
    8    -763.9668432931      2.53E-07
    9    -763.9668432971      7.69E-08 Convergence criterion met
 ----------------------------------------
 SCF time:  CPU 315.28 s  wall 596.92 s
 SCF   energy in the final basis set = -763.96684330
 Total energy in the final basis set = -763.96684330

--------------------------------------------------
-  Entering anlman on Sun Oct 13 13:53:00 2024  -
--------------------------------------------------



 --------------------------------------------------------------


                   Orbital Energies (a.u.)
 --------------------------------------------------------------


 Alpha MOs
 -- Occupied --
-14.8149 -14.7908 -14.7392 -14.7082 -10.6787 -10.6631 -10.6489 -10.6398
-10.6349 -10.6253 -10.6061 -10.5833 -10.5728 -10.5702 -10.5500 -10.5270
-10.4981 -10.4758  -1.4161  -1.4036  -1.3494  -1.2768  -1.2305  -1.1998
 -1.1880  -1.1429  -1.1014  -1.0839  -1.0571  -1.0552  -1.0307  -1.0090
 -0.9900  -0.9546  -0.9466  -0.9183  -0.9096  -0.8944  -0.8843  -0.8660
 -0.8503  -0.8436  -0.8390  -0.8192  -0.8130  -0.7970  -0.7898  -0.7832
 -0.7748  -0.7581  -0.7505  -0.7474  -0.7464  -0.7283  -0.7245  -0.7165
 -0.7040  -0.6717  -0.6583  -0.6466  -0.6388  -0.6315  -0.6254  -0.6212
 -0.6121
```

-- Virtual --

| | | | | | | | |
|---|---|---|---|---|---|---|---|
| -0.2712 | -0.2602 | -0.2456 | -0.2249 | -0.1738 | -0.1647 | -0.1453 | -0.1437 |
| -0.1357 | -0.1263 | -0.1201 | -0.1134 | -0.1068 | -0.1057 | -0.0985 | -0.0925 |
| -0.0893 | -0.0834 | -0.0812 | -0.0790 | -0.0755 | -0.0727 | -0.0718 | -0.0655 |
| -0.0642 | -0.0612 | -0.0584 | -0.0514 | -0.0491 | -0.0472 | -0.0413 | -0.0382 |
| -0.0336 | -0.0316 | -0.0290 | -0.0228 | -0.0200 | -0.0145 | -0.0130 | -0.0072 |
| -0.0062 | -0.0041 | -0.0020 | 0.0012 | 0.0074 | 0.0094 | 0.0122 | 0.0170 |
| 0.0193 | 0.0220 | 0.0260 | 0.0265 | 0.0304 | 0.0328 | 0.0349 | 0.0404 |
| 0.0431 | 0.0443 | 0.0494 | 0.0500 | 0.0546 | 0.0561 | 0.0610 | 0.0653 |
| 0.0677 | 0.0711 | 0.0740 | 0.0761 | 0.0787 | 0.0814 | 0.0841 | 0.0870 |
| 0.0946 | 0.0984 | 0.1004 | 0.1034 | 0.1084 | 0.1112 | 0.1141 | 0.1170 |
| 0.1192 | 0.1228 | 0.1250 | 0.1313 | 0.1344 | 0.1377 | 0.1414 | 0.1421 |
| 0.1490 | 0.1546 | 0.1588 | 0.1637 | 0.1657 | 0.1687 | 0.1736 | 0.1753 |
| 0.1807 | 0.1833 | 0.1879 | 0.1915 | 0.2001 | 0.2053 | 0.2173 | 0.2231 |
| 0.2328 | 0.2388 | 0.2425 | 0.2652 | 0.2770 | 0.2870 | 0.2944 | 0.3130 |
| 0.3221 | 0.3279 | 0.3338 | 0.3372 | 0.3497 | 0.3528 | 0.3628 | 0.3662 |
| 0.3683 | 0.3783 | 0.3831 | 0.3896 | 0.4011 | 0.4101 | 0.4197 | 0.4281 |
| 0.4291 | 0.4350 | 0.4434 | 0.4486 | 0.4579 | 0.4604 | 0.4656 | 0.4779 |
| 0.4875 | 0.4900 | 0.4993 | 0.5019 | 0.5079 | 0.5130 | 0.5156 | 0.5234 |
| 0.5263 | 0.5306 | 0.5334 | 0.5356 | 0.5419 | 0.5507 | 0.5562 | 0.5579 |
| 0.5626 | 0.5697 | 0.5704 | 0.5786 | 0.5852 | 0.5917 | 0.5960 | 0.6045 |
| 0.6089 | 0.6162 | 0.6245 | 0.6318 | 0.6344 | 0.6523 | 0.6551 | 0.6598 |
| 0.6639 | 0.6709 | 0.6779 | 0.6806 | 0.6893 | 0.6935 | 0.7006 | 0.7066 |
| 0.7123 | 0.7224 | 0.7332 | 0.7507 | 0.7556 | 0.7614 | 0.7708 | 0.7775 |
| 0.7866 | 0.7997 | 0.8075 | 0.8244 | 0.8312 | 0.8441 | 0.8487 | 0.8593 |
| 0.8623 | 0.8709 | 0.8885 | 0.8955 | 0.9046 | 0.9129 | 0.9284 | 0.9310 |
| 0.9443 | 0.9559 | 0.9678 | 0.9785 | 0.9870 | 0.9994 | 1.0221 | 1.0432 |
| 1.0490 | 1.0612 | 1.0748 | 1.0844 | 1.1103 | 1.1739 | 1.1849 | 1.1862 |
| 1.1972 | 1.2044 | 1.2093 | 1.2321 | 1.2566 | 1.2698 | 1.2982 | 1.3097 |
| 1.3143 | 1.3219 | 1.3314 | 1.3390 | 1.3474 | 1.3620 | 1.3710 | 1.3737 |
| 1.3870 | 1.3923 | 1.3977 | 1.4065 | 1.4114 | 1.4274 | 1.4297 | 1.4333 |
| 1.4379 | 1.4432 | 1.4461 | 1.4646 | 1.4683 | 1.4737 | 1.4796 | 1.4863 |
| 1.4905 | 1.5013 | 1.5161 | 1.5235 | 1.5316 | 1.5376 | 1.5446 | 1.5560 |
| 1.5644 | 1.5698 | 1.5739 | 1.5777 | 1.5858 | 1.5946 | 1.6123 | 1.6191 |
| 1.6237 | 1.6309 | 1.6395 | 1.6594 | 1.6639 | 1.6693 | 1.6767 | 1.6848 |
| 1.6943 | 1.7003 | 1.7119 | 1.7326 | 1.7429 | 1.7474 | 1.7601 | 1.7660 |
| 1.7848 | 1.7895 | 1.8091 | 1.8212 | 1.8273 | 1.8422 | 1.8616 | 1.8833 |
| 1.8901 | 1.8992 | 1.9086 | 1.9339 | 1.9506 | 1.9573 | 1.9728 | 1.9793 |
| 1.9918 | 2.0038 | 2.0171 | 2.0477 | 2.0583 | 2.0642 | 2.0743 | 2.0919 |
| 2.1147 | 2.1196 | 2.1291 | 2.1409 | 2.1693 | 2.1826 | 2.2150 | 2.2236 |
| 2.2273 | 2.2402 | 2.2469 | 2.2547 | 2.2763 | 2.2804 | 2.2837 | 2.2907 |
| 2.3014 | 2.3084 | 2.3374 | 2.3589 | 2.3601 | 2.3709 | 2.3871 | 2.3961 |
| 2.4075 | 2.4090 | 2.4278 | 2.4396 | 2.4470 | 2.4541 | 2.4607 | 2.4855 |
| 2.5053 | 2.5187 | 2.5242 | 2.5295 | 2.5431 | 2.5449 | 2.5509 | 2.5555 |
| 2.5618 | 2.5734 | 2.5768 | 2.5898 | 2.5950 | 2.5979 | 2.6048 | 2.6206 |
| 2.6356 | 2.6402 | 2.6686 | 2.6726 | 2.6777 | 2.6844 | 2.6878 | 2.7039 |
| 2.7197 | 2.7290 | 2.7326 | 2.7514 | 2.7568 | 2.7645 | 2.7715 | 2.7871 |
| 2.7910 | 2.8034 | 2.8136 | 2.8328 | 2.8514 | 2.8599 | 2.8778 | 2.8897 |
| 2.8940 | 2.9324 | 2.9636 | 2.9909 | 3.0542 | 3.0661 | 3.1155 | 3.1424 |
| 3.1616 | 3.2408 | 3.2473 | 3.2637 | 3.2987 | 3.3328 | 3.3686 | 3.4082 |
| 3.4183 | 3.4772 | 3.5184 | 3.5550 | 3.5625 | 3.5929 | 3.6351 | 3.6402 |
| 3.6639 | 3.6873 | 3.7002 | 3.7292 | 3.7413 | 3.7491 | 3.7657 | 3.7723 |
| 3.7734 | 3.8368 | 3.8724 | 3.9082 | 3.9400 | 3.9824 | 4.0269 | 4.0882 |
| 4.1090 | 4.2033 | 4.2965 | 4.3072 | 4.5953 | 4.6664 | 4.7079 | 4.7186 |
| 4.9204 | 5.0436 | 5.1954 | 23.5236 | 23.5775 | 23.7198 | 23.7282 | 23.7364 |
| 23.7716 | 23.7998 | 23.8247 | 23.8566 | 23.8860 | 23.9101 | 23.9692 | 23.9781 |
| 24.0372 | 35.4247 | 35.4579 | 35.5195 | 35.6202 | | | |

Ground-State Mulliken Net Atomic Charges

| Atom | Charge (a.u.) |
|---|---|
| 1 N | 0.417172 |
| 2 C | 0.147072 |
| 3 C | -0.017558 |
| 4 C | -0.154761 |
| 5 C | -0.186307 |
| 6 C | -0.174767 |
| 7 C | -0.795643 |

```
    8 C                   -0.306204
    9 N                   -0.146787
   10 C                   -0.250438
   11 N                    0.011783
   12 C                    0.254290
   13 C                   -0.003707
   14 C                    0.091803
   15 C                   -0.333665
   16 C                   -0.251675
   17 C                   -0.536870
   18 N                   -0.481496
   19 H                    0.219544
   20 H                    0.234686
   21 H                    0.227110
   22 H                    0.204627
   23 H                    0.230044
   24 H                    0.183961
   25 H                    0.248906
   26 H                    0.229995
   27 H                    0.242020
   28 H                    0.382947
   29 H                    0.199005
   30 H                    0.213869
   31 H                    0.251147
   32 H                    0.203959
   33 H                    0.225234
   34 H                    0.131009
   35 H                    0.216043
   36 H                    0.179182
   37 H                    0.314773
   38 H                    0.379697
 ----------------------------------------
 Sum of atomic charges =     2.000000

-----------------------------------------------------------------
                   Cartesian Multipole Moments
-----------------------------------------------------------------
   Charge (ESU x 10^10)
                9.6064
   Dipole Moment (Debye)
        X       5.6478      Y       3.0025      Z       1.1038
      Tot       6.4908
   Quadrupole Moments (Debye-Ang)
       XX     -13.1993     XY      -4.1699     YY     -76.9225
       XZ       3.9146     YZ      -3.6591     ZZ     -68.2307
   Traceless Quadrupole Moments (Debye-Ang)
      QXX     118.7546    QYY     -72.4151    QZZ     -46.3395
      QXY     -12.5098    QXZ      11.7437    QYZ     -10.9774
   Octopole Moments (Debye-Ang^2)
      XXX       9.0780    XXY     -93.0332    XYY      19.0200
      YYY     -24.1198    XXZ      38.2577    XYZ       8.3202
      YYZ     -24.2361    XZZ      21.5068    YZZ      14.9728
      ZZZ      26.5479
   Traceless Octopole Moments (Debye-Ang^2)
      XXX    -310.2734    YYY     557.8249    ZZZ      33.0927
      XXY   -1088.9570    XXZ     452.1577    XYY     136.4851
      XYZ     124.8030    XZZ     173.7882    YYZ    -485.2505
      YZZ     531.1321
   Hexadecapole Moments (Debye-Ang^3)
     XXXX   -3569.7052   XXXY    -219.5017   XXYY    -773.6705
     XYYY       3.2532   YYYY    -996.1149   XXXZ    -116.4990
     XXYZ      57.8417   XYYZ     -89.7212   YYYZ     -28.4852
     XXZZ    -768.5918   XYZZ     -21.1153   YYZZ    -258.7983
     XZZZ    -235.1545   YZZZ     -42.3177   ZZZZ    -803.3566
   Traceless Hexadecapole Moments (Debye-Ang^3)
     XXXX    4516.3460   XXXY  -12366.3127   XXXZ    7629.4658
     XXYY   -1041.0247   XXYZ    6263.0326   XXZZ   -3475.3212
     XYYY   11022.9590   XYYZ   -2800.1079   XYZZ    1343.3537
     XZZZ   -4829.3578   YYYY   -2761.2135   YYYZ   -2421.9899
     YYZZ    3802.2382   YZZZ   -3841.0428   ZZZZ    -326.9170
```

```
 ---------------------------------------------------------------------

 ---------------------------------------------------
 -  Entering drvman on Sun Oct 13 13:53:00 2024  -
 ---------------------------------------------------

 Calculating analytic gradient of the SCF energy
 Gradient of SCF Energy
           1           2           3           4           5           6
    1   0.0003749   0.0002066  -0.0001534  -0.0001015  -0.0002861   0.0003472
    2   0.0001013  -0.0003317   0.0001591   0.0002038   0.0002367  -0.0004304
    3  -0.0001459   0.0000728   0.0000105   0.0001273  -0.0002332  -0.0001261
           7           8           9          10          11          12
    1  -0.0003307  -0.0000196  -0.0001853  -0.0007768   0.0002313   0.0002228
    2  -0.0000930   0.0000440  -0.0001501  -0.0002081  -0.0003801   0.0011769
    3   0.0000088   0.0000601   0.0004788  -0.0006211   0.0004088  -0.0019071
          13          14          15          16          17          18
    1   0.0006292  -0.0000959  -0.0000632   0.0000895  -0.0000857  -0.0000806
    2   0.0002724  -0.0001564   0.0001655  -0.0003889   0.0001004  -0.0015214
    3   0.0006553   0.0003565   0.0000781   0.0000240   0.0001379  -0.0000358
          19          20          21          22          23          24
    1  -0.0001375   0.0000038  -0.0000067  -0.0001177   0.0000153  -0.0001164
    2   0.0000718   0.0000207   0.0001208   0.0001272  -0.0000083   0.0000385
    3   0.0000104   0.0000112  -0.0000016   0.0001405  -0.0000135  -0.0000307
          25          26          27          28          29          30
    1   0.0000053   0.0002882  -0.0000413   0.0001537   0.0001755   0.0000669
    2  -0.0000082  -0.0001662  -0.0000461   0.0001410   0.0000299  -0.0000545
    3   0.0000041   0.0001356  -0.0000661  -0.0000235  -0.0000235  -0.0000051
          31          32          33          34          35          36
    1   0.0000392  -0.0000289   0.0000158   0.0000317   0.0000792   0.0000282
    2   0.0000147   0.0001288   0.0001182  -0.0000495  -0.0000167   0.0000199
    3  -0.0000154   0.0000080  -0.0000389   0.0000041   0.0000556  -0.0000388
          37          38
    1  -0.0003886   0.0000118
    2  -0.0000534   0.0007713
    3  -0.0000247   0.0005628
 Max gradient component =       1.907E-03
 RMS gradient           =       3.364E-04
 Gradient time:  CPU 183.30 s  wall 331.22 s

 ---------------------------------------------------
 -  Entering optman on Sun Oct 13 13:58:31 2024  -
 ---------------------------------------------------

 Geometry Optimization Parameters
   NAtoms,    NIC,     NZ,  NCons,   NDum,   NFix, NCnnct, MaxDiis
       38     272       0       0       0       0       0       0

 Cartesian Hessian Update
 Hessian updated using BFGS update


** GEOMETRY OPTIMIZATION IN DELOCALIZED INTERNAL COORDINATES **
   Searching for a Minimum

   Optimization Cycle:   7

                        Coordinates (Angstroms)
     ATOM              X               Y               Z
      1  N         2.3619892487    0.4556993508    0.1387911972
      2  C         3.1289828816   -0.0135940410    1.1613558428
      3  C         3.9702200054   -1.0897448675    0.9050176282
      4  C         4.0363078118   -1.6651408070   -0.3515943669
      5  C         3.2379511231   -1.1594251671   -1.3762779821
      6  C         2.4143585325   -0.1029307927   -1.0976988952
      7  C         3.0795044200    0.6233926516    2.5158274251
      8  C         1.4553667428    1.6262443565    0.3131619928
      9  N        -2.0312419304    0.4293897852    0.9604798314
     10  C        -2.6497435033    0.5845646245   -0.2439183945
     11  N        -2.0052287254    1.1122201259   -1.2500697125
```

```
    12  C         -0.7279881662    1.5111512477   -1.1146984387
    13  C         -0.0024746642    1.2852281806    0.1070907191
    14  C         -0.7319325667    0.7583536463    1.1306057937
    15  C         -4.0492416756    0.1048705148   -0.3979895647
    16  C         -4.0881578285   -1.3184563517   -1.0035203570
    17  C         -3.4390765459   -2.3791776326   -0.1216052352
    18  N         -0.1960863878    2.0953784867   -2.1858812082
    19  H          4.5829791561   -1.4656754409    1.7138870255
    20  H          4.7048292123   -2.4981615064   -0.5352414268
    21  H          3.2594540200   -1.5723015341   -2.3759623979
    22  H          1.7713978534    0.3363645178   -1.8491080731
    23  H          3.7277579691    0.0741173398    3.1959006453
    24  H          2.0742367643    0.6124099510    2.9435959130
    25  H          3.4363568494    1.6568155685    2.4870744391
    26  H          1.8041513346    2.4018523161   -0.3692412901
    27  H          1.6020319853    2.0245328956    1.3119967288
    28  H         -2.5572844459    0.0377786435    1.7325777689
    29  H         -0.3198253189    0.5813454724    2.1168338383
    30  H         -4.5564927300    0.1118361062    0.5715270416
    31  H         -4.5649142663    0.8029993601   -1.0584984528
    32  H         -3.6208977461   -1.2934568063   -1.9914823376
    33  H         -5.1412004459   -1.5577951909   -1.1625265250
    34  H         -2.3590809386   -2.2262453407   -0.0113507805
    35  H         -3.5727187089   -3.3704179076   -0.5565826640
    36  H         -3.8901701608   -2.4022704516    0.8755406035
    37  H          0.6703780141    2.6068623801   -2.1630736244
    38  H         -0.8144971691    2.2813863163   -2.9649427070
   Point Group: c1    Number of degrees of freedom:   108


   Energy is   -763.966843297

 Hessian updated using BFGS update
  internal optimization (0)

108 Hessian modes will be used to form the next step
  Hessian Eigenvalues:
     0.001689    0.002570    0.003799    0.005915    0.006939    0.012361
     0.014424    0.018640    0.019646    0.019985    0.021648    0.022392
     0.022480    0.022761    0.024454    0.024786    0.025760    0.026794
     0.026946    0.028176    0.028622    0.030119    0.032975    0.036483
     0.038813    0.042595    0.043260    0.043996    0.045427    0.046928
     0.051786    0.053857    0.055557    0.058039    0.062980    0.079853
     0.085583    0.092018    0.121733    0.122346    0.127138    0.130832
     0.132079    0.132866    0.138550    0.141535    0.143238    0.144578
     0.147269    0.147684    0.149702    0.152300    0.153017    0.153627
     0.155080    0.157918    0.193615    0.206512    0.209584    0.216245
     0.227284    0.230396    0.238881    0.246903    0.252015    0.267127
     0.270965    0.274867    0.285984    0.294111    0.300173    0.300591
     0.300746    0.301284    0.301863    0.303037    0.304105    0.304897
     0.305205    0.305284    0.306520    0.308976    0.312160    0.317604
     0.321771    0.332434    0.333200    0.334478    0.339737    0.343200
     0.348601    0.354437    0.362856    0.372492    0.381336    0.390686
     0.398203    0.404036    0.413939    0.415797    0.419362    0.425654
     0.442737    0.449467    0.481911    0.513664    0.585052    0.743464

 Minimum search - taking simple RFO step
 Searching for Lamda that Minimizes Along All modes
 Value Taken    Lamda =  -0.00013687
 Step Taken.  Stepsize is  0.108508

                             Maximum     Tolerance    Cnvgd?
         Gradient           0.002103      0.000800      NO
         Displacement       0.032173      0.001400      NO
         Energy change     -0.000373      0.000228      NO


 New Cartesian Coordinates Obtained by Inverse Iteration

 Displacement from previous Coordinates is:  0.387907
```

```
 ----------------------------------------------------------------
             Standard Nuclear Orientation (Angstroms)
    I     Atom           X                Y                Z
 ----------------------------------------------------------------
    1      N       2.3568267421     0.4464111689     0.1490775954
    2      C       3.1470617739    -0.0018026039     1.1635784041
    3      C       3.9901580148    -1.0781712101     0.9077895535
    4      C       4.0358008252    -1.6743370817    -0.3397342513
    5      C       3.2146422529    -1.1889486543    -1.3559718244
    6      C       2.3896559363    -0.1316511002    -1.0791082463
    7      C       3.1199155011     0.6550352888     2.5091877671
    8      C       1.4488612353     1.6163580661     0.3222165327
    9      N      -2.0367712749     0.4137726343     0.9551149795
   10      C      -2.6560520817     0.5859241261    -0.2464627579
   11      N      -2.0128570142     1.1251714715    -1.2463249056
   12      C      -0.7344657436     1.5187822632    -1.1062011128
   13      C      -0.0081573306     1.2760103430     0.1109885776
   14      C      -0.7370570106     0.7382006691     1.1279241655
   15      C      -4.0559988975     0.1084115297    -0.4051279755
   16      C      -4.0966017818    -1.3122117918    -1.0165185089
   17      C      -3.4385248374    -2.3767508005    -0.1458074146
   18      N      -0.2048049707     2.1228013719    -2.1668842335
   19      H       4.6208695269    -1.4373331786     1.7106726972
   20      H       4.7052700098    -2.5065769937    -0.5229001300
   21      H       3.2191504121    -1.6179966165    -2.3492048998
   22      H       1.7314684919     0.2912393137    -1.8267368448
   23      H       3.7898113990     0.1238439761     3.1825870373
   24      H       2.1241340811     0.6360875313     2.9588649937
   25      H       3.4617978625     1.6925206229     2.4582526799
   26      H       1.7992651685     2.3947134136    -0.3566776367
   27      H       1.5904297754     2.0116861790     1.3229449159
   28      H      -2.5628448473     0.0121772051     1.7221761879
   29      H      -0.3243477500     0.5489012098     2.1116887937
   30      H      -4.5650412766     0.1127611457     0.5635362877
   31      H      -4.5696427062     0.8095843171    -1.0639152788
   32      H      -3.6373659422    -1.2822559540    -2.0080855605
   33      H      -5.1503871446    -1.5534792154    -1.1677946052
   34      H      -2.3578496564    -2.2232132174    -0.0440972153
   35      H      -3.5755062271    -3.3656733418    -0.5849767489
   36      H      -3.8810105471    -2.4060185027     0.8552020242
   37      H       0.6759452357     2.6096150104    -2.1468534780
   38      H      -0.8157772040     2.3064114053    -2.9524195639
 ----------------------------------------------------------------
 Nuclear Repulsion Energy =         1314.35835367 hartrees
 There are       65 alpha and       65 beta electrons

------------------------------------------------
-  Entering fldman on Sun Oct 13 13:58:31 2024  -
------------------------------------------------

 Applying Cartesian multipole field
    Component          Value
    ---------          -----
     (2,0,0)        1.00000E-11
     (0,2,0)        2.00000E-11
     (0,0,2)       -3.00000E-11
 Nucleus-field energy     =     0.0000000314 hartrees

------------------------------------------------
-  Entering gesman on Sun Oct 13 13:58:31 2024  -
------------------------------------------------

 Requested basis set is 6-311+G(d,p)
 There are 188 shells and 516 basis functions
 A cutoff of  1.0D-12 yielded  12922 shell pairs
 There are    102418 function pairs (    109148 Cartesian)
 Smallest overlap matrix eigenvalue = 2.09E-06
 Linear dependence detected in AO basis
 Tighter screening thresholds may be required for diffuse basis sets
 Use S2THRESH > 12 and THRESH = 14 in case of SCF convergence issues
```

```
 Number of orthogonalized atomic orbitals = 510
 Maximum deviation from orthogonality = 2.084E-11
 Guess MOs from SCF MO coefficient file
 Reading MOs from coefficient file
 Reading MOs from coefficient file

--------------------------------------------------
-  Entering scfman on Sun Oct 13 13:58:31 2024  -
--------------------------------------------------

 Long-range K will be added via erf
 Coulomb attenuation parameter = 0.2 bohr**(-1)
 A restricted hybrid HF-DFT SCF calculation will be
 performed using Pulay DIIS + Geometric Direct Minimization
 Exchange:     0.2220 Hartree-Fock + 1.0000 wB97X-D + LR-HF
 Correlation:  1.0000 wB97X-D
 Using Euler-Maclaurin-Lebedev (75,302) quadrature formula
 Dispersion:   Grimme D
 SCF converges when RMS gradient is below 1.0E-07
 Geometry optimization detected.  Setting ReadMinima to 0
 Setting SaveMinima to 0
 ---------------------------------------
  Cycle       Energy         DIIS Error
 ---------------------------------------
    1    -763.9650113236      3.52E-04
    2    -763.9667812949      4.29E-05
    3    -763.9669261768      1.97E-05
    4    -763.9669413700      1.13E-05
    5    -763.9669464275      4.34E-06
    6    -763.9669471322      1.22E-06
    7    -763.9669471906      4.26E-07
    8    -763.9669471998      1.80E-07
    9    -763.9669472011      3.62E-08 Convergence criterion met
 ---------------------------------------
 SCF time:  CPU 344.77 s  wall 601.78 s
 SCF   energy in the final basis set = -763.96694720
 Total energy in the final basis set = -763.96694720

--------------------------------------------------
-  Entering anlman on Sun Oct 13 14:08:33 2024  -
--------------------------------------------------


 --------------------------------------------------------------

                   Orbital Energies (a.u.)
 --------------------------------------------------------------

 Alpha MOs
 -- Occupied --
-14.8151 -14.7904 -14.7393 -14.7080 -10.6785 -10.6627 -10.6485 -10.6400
-10.6350 -10.6255 -10.6057 -10.5833 -10.5730 -10.5703 -10.5497 -10.5268
-10.4980 -10.4760  -1.4161  -1.4035  -1.3492  -1.2768  -1.2305  -1.1999
 -1.1877  -1.1428  -1.1014  -1.0836  -1.0571  -1.0552  -1.0305  -1.0090
 -0.9899  -0.9548  -0.9465  -0.9183  -0.9096  -0.8942  -0.8843  -0.8659
 -0.8503  -0.8435  -0.8392  -0.8193  -0.8128  -0.7972  -0.7894  -0.7829
 -0.7746  -0.7581  -0.7502  -0.7474  -0.7463  -0.7282  -0.7246  -0.7164
 -0.7040  -0.6717  -0.6583  -0.6466  -0.6389  -0.6314  -0.6254  -0.6211
 -0.6116
 -- Virtual --
 -0.2713  -0.2597  -0.2452  -0.2252  -0.1738  -0.1646  -0.1456  -0.1438
 -0.1352  -0.1265  -0.1202  -0.1131  -0.1067  -0.1056  -0.0985  -0.0925
 -0.0893  -0.0834  -0.0814  -0.0789  -0.0754  -0.0726  -0.0718  -0.0655
 -0.0644  -0.0616  -0.0583  -0.0516  -0.0489  -0.0471  -0.0410  -0.0379
 -0.0336  -0.0316  -0.0291  -0.0232  -0.0204  -0.0146  -0.0127  -0.0066
 -0.0063  -0.0042  -0.0022   0.0009   0.0079   0.0093   0.0120   0.0174
  0.0189   0.0218   0.0259   0.0265   0.0306   0.0328   0.0353   0.0401
  0.0429   0.0445   0.0494   0.0501   0.0544   0.0564   0.0607   0.0657
  0.0675   0.0712   0.0748   0.0765   0.0793   0.0816   0.0852   0.0871
  0.0955   0.0986   0.1004   0.1031   0.1091   0.1116   0.1134   0.1167
```

| | | | | | | | |
|---|---|---|---|---|---|---|---|
| 0.1190 | 0.1223 | 0.1247 | 0.1305 | 0.1342 | 0.1388 | 0.1406 | 0.1422 |
| 0.1488 | 0.1537 | 0.1591 | 0.1642 | 0.1647 | 0.1690 | 0.1733 | 0.1756 |
| 0.1811 | 0.1839 | 0.1878 | 0.1916 | 0.2005 | 0.2035 | 0.2167 | 0.2230 |
| 0.2327 | 0.2392 | 0.2430 | 0.2649 | 0.2769 | 0.2870 | 0.2946 | 0.3130 |
| 0.3220 | 0.3280 | 0.3343 | 0.3370 | 0.3499 | 0.3533 | 0.3627 | 0.3659 |
| 0.3689 | 0.3783 | 0.3832 | 0.3902 | 0.4014 | 0.4097 | 0.4205 | 0.4275 |
| 0.4288 | 0.4350 | 0.4435 | 0.4500 | 0.4565 | 0.4598 | 0.4655 | 0.4774 |
| 0.4874 | 0.4894 | 0.4992 | 0.5016 | 0.5085 | 0.5129 | 0.5153 | 0.5229 |
| 0.5268 | 0.5308 | 0.5330 | 0.5372 | 0.5414 | 0.5509 | 0.5564 | 0.5580 |
| 0.5629 | 0.5696 | 0.5706 | 0.5786 | 0.5861 | 0.5909 | 0.5962 | 0.6049 |
| 0.6098 | 0.6168 | 0.6258 | 0.6338 | 0.6346 | 0.6530 | 0.6548 | 0.6595 |
| 0.6631 | 0.6709 | 0.6780 | 0.6805 | 0.6899 | 0.6943 | 0.6996 | 0.7050 |
| 0.7133 | 0.7214 | 0.7365 | 0.7497 | 0.7553 | 0.7611 | 0.7719 | 0.7778 |
| 0.7866 | 0.7991 | 0.8083 | 0.8254 | 0.8328 | 0.8443 | 0.8480 | 0.8602 |
| 0.8612 | 0.8707 | 0.8879 | 0.8963 | 0.9053 | 0.9140 | 0.9290 | 0.9299 |
| 0.9469 | 0.9558 | 0.9695 | 0.9786 | 0.9865 | 0.9996 | 1.0225 | 1.0431 |
| 1.0506 | 1.0594 | 1.0743 | 1.0821 | 1.1106 | 1.1705 | 1.1790 | 1.1842 |
| 1.1972 | 1.2035 | 1.2088 | 1.2314 | 1.2566 | 1.2718 | 1.2985 | 1.3094 |
| 1.3126 | 1.3228 | 1.3303 | 1.3385 | 1.3468 | 1.3608 | 1.3699 | 1.3735 |
| 1.3871 | 1.3921 | 1.3981 | 1.4069 | 1.4116 | 1.4263 | 1.4293 | 1.4333 |
| 1.4368 | 1.4447 | 1.4465 | 1.4650 | 1.4687 | 1.4734 | 1.4802 | 1.4871 |
| 1.4910 | 1.5020 | 1.5170 | 1.5233 | 1.5326 | 1.5374 | 1.5448 | 1.5567 |
| 1.5637 | 1.5689 | 1.5741 | 1.5772 | 1.5867 | 1.5949 | 1.6115 | 1.6192 |
| 1.6246 | 1.6312 | 1.6378 | 1.6581 | 1.6634 | 1.6694 | 1.6765 | 1.6843 |
| 1.6943 | 1.7002 | 1.7115 | 1.7325 | 1.7420 | 1.7470 | 1.7600 | 1.7643 |
| 1.7853 | 1.7908 | 1.8088 | 1.8219 | 1.8283 | 1.8413 | 1.8632 | 1.8824 |
| 1.8901 | 1.9012 | 1.9098 | 1.9347 | 1.9506 | 1.9613 | 1.9736 | 1.9812 |
| 1.9924 | 2.0040 | 2.0186 | 2.0481 | 2.0595 | 2.0636 | 2.0718 | 2.0933 |
| 2.1155 | 2.1204 | 2.1294 | 2.1402 | 2.1700 | 2.1833 | 2.2164 | 2.2244 |
| 2.2269 | 2.2400 | 2.2484 | 2.2535 | 2.2759 | 2.2786 | 2.2816 | 2.2897 |
| 2.3012 | 2.3075 | 2.3367 | 2.3582 | 2.3587 | 2.3713 | 2.3870 | 2.3963 |
| 2.4062 | 2.4084 | 2.4290 | 2.4393 | 2.4457 | 2.4538 | 2.4613 | 2.4848 |
| 2.5027 | 2.5188 | 2.5236 | 2.5298 | 2.5435 | 2.5458 | 2.5504 | 2.5567 |
| 2.5615 | 2.5725 | 2.5773 | 2.5896 | 2.5957 | 2.5981 | 2.6047 | 2.6202 |
| 2.6349 | 2.6404 | 2.6682 | 2.6725 | 2.6770 | 2.6827 | 2.6890 | 2.7046 |
| 2.7193 | 2.7304 | 2.7332 | 2.7518 | 2.7572 | 2.7652 | 2.7713 | 2.7872 |
| 2.7898 | 2.8052 | 2.8143 | 2.8330 | 2.8497 | 2.8615 | 2.8793 | 2.8888 |
| 2.8941 | 2.9327 | 2.9652 | 2.9913 | 3.0548 | 3.0670 | 3.1185 | 3.1425 |
| 3.1619 | 3.2415 | 3.2471 | 3.2664 | 3.2976 | 3.3330 | 3.3700 | 3.4108 |
| 3.4170 | 3.4777 | 3.5189 | 3.5557 | 3.5622 | 3.5930 | 3.6339 | 3.6410 |
| 3.6633 | 3.6865 | 3.7003 | 3.7313 | 3.7408 | 3.7490 | 3.7597 | 3.7712 |
| 3.7732 | 3.8372 | 3.8720 | 3.9087 | 3.9406 | 3.9828 | 4.0262 | 4.0871 |
| 4.1092 | 4.2038 | 4.2961 | 4.3086 | 4.5952 | 4.6667 | 4.7075 | 4.7198 |
| 4.9218 | 5.0429 | 5.1968 | 23.5232 | 23.5769 | 23.7203 | 23.7288 | 23.7363 |
| 23.7733 | 23.8004 | 23.8240 | 23.8561 | 23.8848 | 23.9099 | 23.9674 | 23.9811 |
| 24.0358 | 35.4240 | 35.4584 | 35.5205 | 35.6214 | | | |

---

Ground-State Mulliken Net Atomic Charges

| Atom | Charge (a.u.) |
|---|---|
| 1 N | 0.420610 |
| 2 C | 0.136830 |
| 3 C | -0.013114 |
| 4 C | -0.153122 |
| 5 C | -0.189706 |
| 6 C | -0.174145 |
| 7 C | -0.788894 |
| 8 C | -0.309671 |
| 9 N | -0.145058 |
| 10 C | -0.255805 |
| 11 N | 0.010045 |
| 12 C | 0.268083 |
| 13 C | -0.006070 |
| 14 C | 0.087667 |
| 15 C | -0.333730 |
| 16 C | -0.253357 |
| 17 C | -0.536907 |
| 18 N | -0.482894 |

```
    19 H                       0.219639
    20 H                       0.234640
    21 H                       0.227110
    22 H                       0.203085
    23 H                       0.229947
    24 H                       0.186578
    25 H                       0.247529
    26 H                       0.228952
    27 H                       0.242206
    28 H                       0.382318
    29 H                       0.200049
    30 H                       0.214578
    31 H                       0.250891
    32 H                       0.204331
    33 H                       0.225393
    34 H                       0.131085
    35 H                       0.216221
    36 H                       0.178772
    37 H                       0.315759
    38 H                       0.380155
  ----------------------------------------
  Sum of atomic charges =     2.000000

 -----------------------------------------------------------------
                    Cartesian Multipole Moments
 -----------------------------------------------------------------
    Charge (ESU x 10^10)
                 9.6064
    Dipole Moment (Debye)
         X       5.6723      Y       2.9311      Z       1.1193
       Tot       6.4822
    Quadrupole Moments (Debye-Ang)
        XX     -12.8642     XY      -4.0156     YY     -76.9378
        XZ       4.3809     YZ      -3.4721     ZZ     -68.4725
    Traceless Quadrupole Moments (Debye-Ang)
       QXX     119.6818    QYY     -72.5389    QZZ     -47.1430
       QXY     -12.0468    QXZ      13.1427    QYZ     -10.4163
    Octopole Moments (Debye-Ang^2)
       XXX      11.3307    XXY     -92.8243    XYY      19.5892
       YYY     -24.2336    XXZ      39.9682    XYZ       8.9088
       YYZ     -24.7217    XZZ      21.2439    YZZ      14.9138
       ZZZ      26.1481
    Traceless Octopole Moments (Debye-Ang^2)
       XXX    -299.5140    YYY     555.7927    ZZZ      19.6698
       XXY   -1085.9327    XXZ     475.3396    XYY     137.3462
       XYZ     133.6323    XZZ     162.1678    YYZ    -495.0094
       YZZ     530.1399
    Hexadecapole Moments (Debye-Ang^3)
      XXXX   -3568.8897   XXXY    -216.3614   XXYY    -776.4849
      XYYY       3.9745   YYYY   -1001.8539   XXXZ    -114.3844
      XXYZ      58.0589   XYYZ     -93.7493   YYYZ     -30.4364
      XXZZ    -770.6017   XYZZ     -18.5192   YYZZ    -258.1517
      XZZZ    -243.6708   YZZZ     -44.5708   ZZZZ    -798.7449
    Traceless Hexadecapole Moments (Debye-Ang^3)
      XXXX    4884.7615   XXXY  -12327.1733   XXXZ    8320.8396
      XXYY   -1183.8072   XXYZ    6341.8469   XXZZ   -3700.9543
      XYYY   10808.0993   XYYZ   -3066.6073   XYZZ    1519.0741
      XZZZ   -5254.2323   YYYY   -2730.2038   YYYZ   -2458.8342
      YYZZ    3914.0110   YZZZ   -3883.0126   ZZZZ    -213.0568
 -----------------------------------------------------------------

----------------------------------------------------
-  Entering drvman on Sun Oct 13 14:08:33 2024  -
----------------------------------------------------

 Calculating analytic gradient of the SCF energy
 Gradient of SCF Energy
            1           2           3           4           5           6
    1   0.0001466  -0.0002677   0.0001235   0.0001879   0.0003650  -0.0003621
    2   0.0001933   0.0003297  -0.0003956  -0.0001562  -0.0004163   0.0004630
```

```
   3  -0.0001110   0.0002370  -0.0004417   0.0000391   0.0000718   0.0000397
           7           8           9          10          11          12
   1  -0.0001696  -0.0000128   0.0000432  -0.0000121   0.0000379   0.0000466
   2  -0.0000001  -0.0000390  -0.0000570   0.0003156  -0.0003247  -0.0000852
   3   0.0000454   0.0000618   0.0002311  -0.0006322   0.0005480  -0.0007855
          13          14          15          16          17          18
   1   0.0000612   0.0000988   0.0000077   0.0000516   0.0000365  -0.0006400
   2   0.0000670   0.0001704   0.0000452  -0.0002206   0.0000816  -0.0004350
   3   0.0008036  -0.0005007   0.0000189  -0.0000132   0.0000582   0.0000390
          19          20          21          22          23          24
   1  -0.0000338  -0.0000194   0.0000326  -0.0000342  -0.0000124  -0.0001350
   2  -0.0000215   0.0000577   0.0000248   0.0000227  -0.0000313  -0.0000277
   3   0.0000194   0.0000104  -0.0000606   0.0000389   0.0000017  -0.0000379
          25          26          27          28          29          30
   1  -0.0000089   0.0002171  -0.0001238   0.0000699   0.0001424   0.0000258
   2  -0.0000224  -0.0000140  -0.0000384   0.0000903   0.0000750   0.0000008
   3   0.0000268   0.0000181  -0.0000421   0.0000443  -0.0000190  -0.0000114
          31          32          33          34          35          36
   1   0.0000497  -0.0000326   0.0000002   0.0000091   0.0000279   0.0000024
   2  -0.0000176   0.0000627   0.0000335  -0.0000209  -0.0000043   0.0000088
   3  -0.0000624   0.0000084   0.0000210   0.0000196   0.0000442   0.0000036
          37          38
   1   0.0000819  -0.0000011
   2  -0.0001277   0.0004134
   3   0.0000950   0.0001729
 Max gradient component =       8.036E-04
 RMS gradient           =       2.100E-04
 Gradient time:  CPU 167.84 s  wall 369.07 s

-------------------------------------------------
-  Entering optman on Sun Oct 13 14:14:42 2024  -
-------------------------------------------------

 Geometry Optimization Parameters
   NAtoms,    NIC,     NZ,  NCons,   NDum,   NFix, NCnnct, MaxDiis
       38     272       0       0       0       0       0       0

 Cartesian Hessian Update
 Hessian updated using BFGS update


** GEOMETRY OPTIMIZATION IN DELOCALIZED INTERNAL COORDINATES **
   Searching for a Minimum

   Optimization Cycle:   8

                     Coordinates (Angstroms)
     ATOM                X               Y               Z
      1  N         2.3568267421    0.4464111689    0.1490775954
      2  C         3.1470617739   -0.0018026039    1.1635784041
      3  C         3.9901580148   -1.0781712101    0.9077895535
      4  C         4.0358008252   -1.6743370817   -0.3397342513
      5  C         3.2146422529   -1.1889486543   -1.3559718244
      6  C         2.3896559363   -0.1316511002   -1.0791082463
      7  C         3.1199155011    0.6550352888    2.5091877671
      8  C         1.4488612353    1.6163580661    0.3222165327
      9  N        -2.0367712749    0.4137726343    0.9551149795
     10  C        -2.6560520817    0.5859241261   -0.2464627579
     11  N        -2.0128570142    1.1251714715   -1.2463249056
     12  C        -0.7344657436    1.5187822632   -1.1062011128
     13  C        -0.0081573306    1.2760103430    0.1109885776
     14  C        -0.7370570106    0.7382006691    1.1279241655
     15  C        -4.0559988975    0.1084115297   -0.4051279755
     16  C        -4.0966017818   -1.3122117918   -1.0165185089
     17  C        -3.4385248374   -2.3767508005   -0.1458074146
     18  N        -0.2048049707    2.1228013719   -2.1668842335
     19  H         4.6208695269   -1.4373331786    1.7106726972
     20  H         4.7052700098   -2.5065769937   -0.5229001300
     21  H         3.2191504121   -1.6179966165   -2.3492048998
     22  H         1.7314684919    0.2912393137   -1.8267368448
```

```
    23  H         3.7898113990    0.1238439761    3.1825870373
    24  H         2.1241340811    0.6360875313    2.9588649937
    25  H         3.4617978625    1.6925206229    2.4582526799
    26  H         1.7992651685    2.3947134136   -0.3566776367
    27  H         1.5904297754    2.0116861790    1.3229449159
    28  H        -2.5628448473    0.0121772051    1.7221761879
    29  H        -0.3243477500    0.5489012098    2.1116887937
    30  H        -4.5650412766    0.1127611457    0.5635362877
    31  H        -4.5696427062    0.8095843171   -1.0639152788
    32  H        -3.6373659422   -1.2822559540   -2.0080855605
    33  H        -5.1503871446   -1.5534792154   -1.1677946052
    34  H        -2.3578496564   -2.2232132174   -0.0440972153
    35  H        -3.5755062271   -3.3656733418   -0.5849767489
    36  H        -3.8810105471   -2.4060185027    0.8552020242
    37  H         0.6759452357    2.6096150104   -2.1468534780
    38  H        -0.8157772040    2.3064114053   -2.9524195639
  Point Group: c1    Number of degrees of freedom:   108


  Energy is   -763.966947201

 Hessian updated using BFGS update
  internal optimization (0)

108 Hessian modes will be used to form the next step
  Hessian Eigenvalues:
     0.001666    0.002544    0.003301    0.004724    0.006391    0.012446
     0.014488    0.018336    0.019185    0.020003    0.020693    0.021833
     0.022566    0.022771    0.023688    0.024677    0.025902    0.026079
     0.027059    0.028124    0.028245    0.030057    0.031223    0.036348
     0.038794    0.041613    0.043254    0.043829    0.044742    0.046360
     0.051784    0.054430    0.055588    0.057973    0.062185    0.079768
     0.085600    0.091970    0.121736    0.122345    0.127131    0.130810
     0.132370    0.132882    0.139325    0.141493    0.143052    0.144741
     0.145800    0.147674    0.149187    0.150267    0.152635    0.153085
     0.156639    0.165129    0.195254    0.206571    0.209586    0.216465
     0.227591    0.230150    0.238612    0.246803    0.251084    0.261085
     0.268320    0.273595    0.285941    0.293267    0.300172    0.300595
     0.300810    0.301292    0.301885    0.303052    0.304095    0.304880
     0.305229    0.305283    0.306509    0.308601    0.312658    0.316883
     0.322933    0.332827    0.333696    0.334568    0.339821    0.343203
     0.348818    0.359207    0.363519    0.381195    0.384341    0.389003
     0.391401    0.402456    0.410315    0.415479    0.419314    0.422227
     0.446274    0.475232    0.478703    0.513271    0.581295    0.740329

 Minimum search - taking simple RFO step
 Searching for Lamda that Minimizes Along All modes
 Value Taken    Lamda =  -0.00018430
 Step Taken.  Stepsize is  0.179067

                            Maximum     Tolerance    Cnvgd?
        Gradient           0.002042      0.000800      NO
        Displacement       0.054014      0.001400      NO
        Energy change     -0.000104      0.000228     YES


 New Cartesian Coordinates Obtained by Inverse Iteration

 Displacement from previous Coordinates is:  0.814683
 ----------------------------------------------------------------
            Standard Nuclear Orientation (Angstroms)
    I     Atom           X                Y                Z
 ----------------------------------------------------------------
    1      N       2.3493281553     0.4298149209     0.1715587685
    2      C       3.1859228927     0.0248141965     1.1672425993
    3      C       4.0290894643    -1.0512468583     0.9151273064
    4      C       4.0300997661    -1.6890356430    -0.3127655487
    5      C       3.1658658498    -1.2450757491    -1.3121101117
    6      C       2.3427563030    -0.1872997674    -1.0379758560
    7      C       3.2071074902     0.7266077348     2.4901238363
```

```
    8      C       1.4385998725     1.5986878589     0.3419471234
    9      N      -2.0447412423     0.3784605187     0.9499274396
   10      C      -2.6657988447     0.5827151837    -0.2453705112
   11      N      -2.0243177905     1.1459241777    -1.2341964988
   12      C      -0.7438999775     1.5317058713    -1.0859088785
   13      C      -0.0173066525     1.2588442052     0.1230954824
   14      C      -0.7445603827     0.6968582166     1.1291513446
   15      C      -4.0681320364     0.1134696441    -0.4118545722
   16      C      -4.1161753885    -1.2950903158    -1.0489810447
   17      C      -3.4540951290    -2.3782407909    -0.2043883749
   18      N      -0.2139772241     2.1606647298    -2.1316348672
   19      H       4.6957910485    -1.3773371981     1.7029329520
   20      H       4.6998710967    -2.5216819070    -0.4946208394
   21      H       3.1366351491    -1.7072532295    -2.2898448050
   22      H       1.6551443410     0.2054057721    -1.7754908185
   23      H       3.9198647447     0.2334562588     3.1483364489
   24      H       2.2335631961     0.6989348238     2.9864768911
   25      H       3.5206905617     1.7692735939     2.3897558633
   26      H       1.7904287180     2.3800966854    -0.3329588240
   27      H       1.5727943812     1.9914200943     1.3447471780
   28      H      -2.5704996120    -0.0438425260     1.7062249146
   29      H      -0.3301010196     0.4822340147     2.1071072668
   30      H      -4.5766954158     0.1030103537     0.5571499821
   31      H      -4.5795883073     0.8286578176    -1.0570737562
   32      H      -3.6640022650    -1.2491224788    -2.0432196035
   33      H      -5.1714313024    -1.5325881590    -1.1963775435
   34      H      -2.3725910170    -2.2283090855    -0.1069765100
   35      H      -3.5966948516    -3.3576482423    -0.6627008398
   36      H      -3.8897111126    -2.4273568678     0.7990784700
   37      H       0.6845361455     2.6137432316    -2.1178187546
   38      H      -0.8137696053     2.3363289144    -2.9277153088
 ----------------------------------------------------------------
 Nuclear Repulsion Energy =        1313.51332954 hartrees
 There are       65 alpha and       65 beta electrons

--------------------------------------------------
-  Entering fldman on Sun Oct 13 14:14:43 2024  -
--------------------------------------------------

 Applying Cartesian multipole field
    Component          Value
    ---------          -----
     (2,0,0)        1.00000E-11
     (0,2,0)        2.00000E-11
     (0,0,2)       -3.00000E-11
 Nucleus-field energy     =     0.0000000323 hartrees

--------------------------------------------------
-  Entering gesman on Sun Oct 13 14:14:43 2024  -
--------------------------------------------------

 Requested basis set is 6-311+G(d,p)
 There are 188 shells and 516 basis functions
 A cutoff of  1.0D-12 yielded  12897 shell pairs
 There are    102333 function pairs (    109055 Cartesian)
 Smallest overlap matrix eigenvalue = 2.03E-06
 Linear dependence detected in AO basis
 Tighter screening thresholds may be required for diffuse basis sets
 Use S2THRESH > 12 and THRESH = 14 in case of SCF convergence issues
 Number of orthogonalized atomic orbitals = 510
 Maximum deviation from orthogonality = 1.689E-11
 Guess MOs from SCF MO coefficient file
 Reading MOs from coefficient file
 Reading MOs from coefficient file

--------------------------------------------------
-  Entering scfman on Sun Oct 13 14:14:43 2024  -
--------------------------------------------------

 Long-range K will be added via erf
```

```
 Coulomb attenuation parameter = 0.2 bohr**(-1)
 A restricted hybrid HF-DFT SCF calculation will be
 performed using Pulay DIIS + Geometric Direct Minimization
 Exchange:     0.2220 Hartree-Fock + 1.0000 wB97X-D + LR-HF
 Correlation:  1.0000 wB97X-D
 Using Euler-Maclaurin-Lebedev (75,302) quadrature formula
 Dispersion:   Grimme D
 SCF converges when RMS gradient is below 1.0E-07
 Geometry optimization detected.  Setting ReadMinima to 0
 Setting SaveMinima to 0
 ---------------------------------------
  Cycle       Energy         DIIS Error
 ---------------------------------------
    1    -763.9527800321      7.00E-04
    2    -763.9664060640      8.64E-05
    3    -763.9669797089      4.38E-05
    4    -763.9670450709      2.52E-05
    5    -763.9670678844      9.08E-06
    6    -763.9670710254      2.38E-06
    7    -763.9670712621      7.97E-07
    8    -763.9670712970      3.42E-07
    9    -763.9670713022      7.47E-08 Convergence criterion met
 ---------------------------------------
 SCF time:  CPU 344.14 s  wall 708.17 s
 SCF   energy in the final basis set = -763.96707130
 Total energy in the final basis set = -763.96707130

-------------------------------------------------
-  Entering anlman on Sun Oct 13 14:26:31 2024  -
-------------------------------------------------



 --------------------------------------------------------------

                   Orbital Energies (a.u.)
 --------------------------------------------------------------

 Alpha MOs
 -- Occupied --
-14.8151 -14.7902 -14.7393 -14.7078 -10.6781 -10.6624 -10.6485 -10.6398
-10.6350 -10.6256 -10.6054 -10.5834 -10.5730 -10.5701 -10.5489 -10.5266
-10.4979 -10.4761  -1.4158  -1.4034  -1.3489  -1.2763  -1.2304  -1.1998
 -1.1876  -1.1427  -1.1013  -1.0834  -1.0571  -1.0550  -1.0303  -1.0089
 -0.9899  -0.9549  -0.9463  -0.9182  -0.9097  -0.8939  -0.8843  -0.8658
 -0.8503  -0.8432  -0.8393  -0.8194  -0.8123  -0.7979  -0.7892  -0.7825
 -0.7741  -0.7579  -0.7498  -0.7474  -0.7459  -0.7282  -0.7245  -0.7160
 -0.7039  -0.6714  -0.6581  -0.6466  -0.6388  -0.6312  -0.6254  -0.6214
 -0.6109
 -- Virtual --
 -0.2713  -0.2595  -0.2449  -0.2252  -0.1739  -0.1644  -0.1464  -0.1439
 -0.1342  -0.1268  -0.1204  -0.1124  -0.1067  -0.1055  -0.0987  -0.0923
 -0.0891  -0.0834  -0.0819  -0.0787  -0.0752  -0.0722  -0.0717  -0.0655
 -0.0650  -0.0621  -0.0579  -0.0519  -0.0486  -0.0464  -0.0404  -0.0374
 -0.0336  -0.0314  -0.0294  -0.0243  -0.0210  -0.0146  -0.0121  -0.0067
 -0.0058  -0.0041  -0.0024   0.0002   0.0085   0.0091   0.0117   0.0180
  0.0183   0.0215   0.0256   0.0266   0.0308   0.0332   0.0363   0.0389
  0.0428   0.0448   0.0491   0.0505   0.0538   0.0567   0.0605   0.0663
  0.0674   0.0711   0.0758   0.0773   0.0804   0.0818   0.0863   0.0876
  0.0969   0.0992   0.1003   0.1031   0.1097   0.1123   0.1128   0.1156
  0.1183   0.1216   0.1243   0.1297   0.1339   0.1388   0.1402   0.1429
  0.1475   0.1517   0.1594   0.1637   0.1650   0.1699   0.1728   0.1769
  0.1814   0.1847   0.1871   0.1926   0.2004   0.2014   0.2167   0.2230
  0.2325   0.2398   0.2435   0.2644   0.2768   0.2867   0.2953   0.3132
  0.3215   0.3284   0.3349   0.3367   0.3504   0.3538   0.3623   0.3640
  0.3712   0.3786   0.3832   0.3911   0.4013   0.4078   0.4226   0.4259
  0.4294   0.4354   0.4438   0.4501   0.4556   0.4594   0.4658   0.4759
  0.4878   0.4886   0.4993   0.5018   0.5088   0.5125   0.5155   0.5211
  0.5278   0.5309   0.5328   0.5396   0.5413   0.5514   0.5557   0.5579
  0.5631   0.5684   0.5712   0.5789   0.5869   0.5895   0.5969   0.6057
  0.6109   0.6174   0.6273   0.6321   0.6385   0.6537   0.6545   0.6584
```

| | | | | | | | |
|---|---|---|---|---|---|---|---|
| 0.6629 | 0.6712 | 0.6783 | 0.6801 | 0.6899 | 0.6965 | 0.6983 | 0.7048 |
| 0.7148 | 0.7195 | 0.7409 | 0.7484 | 0.7537 | 0.7605 | 0.7740 | 0.7788 |
| 0.7871 | 0.7988 | 0.8098 | 0.8255 | 0.8356 | 0.8445 | 0.8461 | 0.8584 |
| 0.8607 | 0.8708 | 0.8870 | 0.8966 | 0.9078 | 0.9156 | 0.9276 | 0.9294 |
| 0.9512 | 0.9566 | 0.9728 | 0.9785 | 0.9859 | 1.0002 | 1.0233 | 1.0425 |
| 1.0525 | 1.0565 | 1.0732 | 1.0778 | 1.1110 | 1.1589 | 1.1758 | 1.1822 |
| 1.1959 | 1.2031 | 1.2102 | 1.2312 | 1.2568 | 1.2756 | 1.2986 | 1.3069 |
| 1.3112 | 1.3225 | 1.3295 | 1.3389 | 1.3462 | 1.3589 | 1.3691 | 1.3736 |
| 1.3876 | 1.3920 | 1.3992 | 1.4074 | 1.4131 | 1.4251 | 1.4291 | 1.4338 |
| 1.4359 | 1.4462 | 1.4492 | 1.4652 | 1.4697 | 1.4723 | 1.4807 | 1.4880 |
| 1.4922 | 1.5018 | 1.5182 | 1.5226 | 1.5320 | 1.5373 | 1.5454 | 1.5575 |
| 1.5626 | 1.5670 | 1.5742 | 1.5778 | 1.5874 | 1.5959 | 1.6101 | 1.6196 |
| 1.6259 | 1.6319 | 1.6354 | 1.6565 | 1.6628 | 1.6698 | 1.6761 | 1.6835 |
| 1.6956 | 1.6999 | 1.7124 | 1.7317 | 1.7411 | 1.7461 | 1.7589 | 1.7629 |
| 1.7864 | 1.7928 | 1.8073 | 1.8225 | 1.8308 | 1.8407 | 1.8654 | 1.8806 |
| 1.8899 | 1.9048 | 1.9109 | 1.9353 | 1.9508 | 1.9656 | 1.9748 | 1.9854 |
| 1.9941 | 2.0040 | 2.0217 | 2.0473 | 2.0594 | 2.0631 | 2.0692 | 2.0952 |
| 2.1167 | 2.1211 | 2.1290 | 2.1393 | 2.1704 | 2.1843 | 2.2179 | 2.2246 |
| 2.2269 | 2.2404 | 2.2490 | 2.2533 | 2.2745 | 2.2764 | 2.2797 | 2.2892 |
| 2.3003 | 2.3076 | 2.3362 | 2.3566 | 2.3587 | 2.3712 | 2.3869 | 2.3965 |
| 2.4049 | 2.4083 | 2.4305 | 2.4385 | 2.4428 | 2.4539 | 2.4619 | 2.4836 |
| 2.4981 | 2.5192 | 2.5233 | 2.5304 | 2.5446 | 2.5468 | 2.5499 | 2.5586 |
| 2.5615 | 2.5713 | 2.5785 | 2.5897 | 2.5970 | 2.5985 | 2.6048 | 2.6195 |
| 2.6327 | 2.6398 | 2.6666 | 2.6719 | 2.6763 | 2.6782 | 2.6901 | 2.7054 |
| 2.7173 | 2.7327 | 2.7351 | 2.7515 | 2.7558 | 2.7673 | 2.7699 | 2.7877 |
| 2.7887 | 2.8074 | 2.8142 | 2.8328 | 2.8469 | 2.8646 | 2.8811 | 2.8882 |
| 2.8945 | 2.9326 | 2.9674 | 2.9920 | 3.0557 | 3.0689 | 3.1221 | 3.1426 |
| 3.1622 | 3.2415 | 3.2471 | 3.2699 | 3.2975 | 3.3353 | 3.3702 | 3.4144 |
| 3.4172 | 3.4773 | 3.5187 | 3.5559 | 3.5622 | 3.5939 | 3.6313 | 3.6430 |
| 3.6626 | 3.6859 | 3.7004 | 3.7325 | 3.7391 | 3.7470 | 3.7535 | 3.7710 |
| 3.7740 | 3.8375 | 3.8722 | 3.9084 | 3.9404 | 3.9834 | 4.0255 | 4.0853 |
| 4.1097 | 4.2042 | 4.2961 | 4.3098 | 4.5953 | 4.6664 | 4.7071 | 4.7204 |
| 4.9235 | 5.0430 | 5.1980 | 23.5225 | 23.5756 | 23.7199 | 23.7285 | 23.7362 |
| 23.7754 | 23.8007 | 23.8241 | 23.8556 | 23.8854 | 23.9097 | 23.9694 | 23.9799 |
| 24.0374 | 35.4235 | 35.4584 | 35.5209 | 35.6213 | | | |

Ground-State Mulliken Net Atomic Charges

| Atom | | Charge (a.u.) |
|---|---|---|
| 1 | N | 0.423511 |
| 2 | C | 0.125807 |
| 3 | C | -0.007737 |
| 4 | C | -0.150486 |
| 5 | C | -0.192946 |
| 6 | C | -0.174294 |
| 7 | C | -0.778366 |
| 8 | C | -0.317983 |
| 9 | N | -0.141834 |
| 10 | C | -0.264187 |
| 11 | N | 0.007419 |
| 12 | C | 0.287763 |
| 13 | C | -0.014288 |
| 14 | C | 0.085261 |
| 15 | C | -0.334745 |
| 16 | C | -0.254586 |
| 17 | C | -0.537269 |
| 18 | N | -0.482893 |
| 19 | H | 0.219838 |
| 20 | H | 0.234627 |
| 21 | H | 0.226857 |
| 22 | H | 0.202282 |
| 23 | H | 0.229792 |
| 24 | H | 0.191414 |
| 25 | H | 0.244905 |
| 26 | H | 0.226880 |
| 27 | H | 0.243175 |
| 28 | H | 0.381378 |
| 29 | H | 0.202811 |

```
    30 H                    0.215228
    31 H                    0.250801
    32 H                    0.204478
    33 H                    0.225493
    34 H                    0.130853
    35 H                    0.216374
    36 H                    0.178567
    37 H                    0.316802
    38 H                    0.379298
  ----------------------------------------
  Sum of atomic charges =     2.000000

 -----------------------------------------------------------------
                    Cartesian Multipole Moments
 -----------------------------------------------------------------
    Charge (ESU x 10^10)
                 9.6064
    Dipole Moment (Debye)
         X       5.7072      Y       2.7924      Z       1.1875
       Tot       6.4637
    Quadrupole Moments (Debye-Ang)
        XX     -12.1111     XY      -3.5454     YY     -76.9689
        XZ       5.3129     YZ      -3.0492     ZZ     -69.0225
    Traceless Quadrupole Moments (Debye-Ang)
       QXX     121.7691    QYY     -72.8043    QZZ     -48.9649
       QXY     -10.6361    QXZ      15.9386    QYZ      -9.1476
    Octopole Moments (Debye-Ang^2)
       XXX      15.6027    XXY     -91.7896    XYY      20.6636
       YYY     -24.3460    XXZ      43.4098    XYZ       9.9978
       YYZ     -25.5790    XZZ      20.4388    YZZ      14.7001
       ZZZ      25.1674
    Traceless Octopole Moments (Debye-Ang^2)
       XXX    -276.3054    YYY     547.7296    ZZZ      -9.4729
       XXY   -1072.5382    XXZ     522.1528    XYY     139.8394
       XYZ     149.9675    XZZ     136.4660    YYZ    -512.6799
       YZZ     524.8086
    Hexadecapole Moments (Debye-Ang^3)
      XXXX   -3569.8817   XXXY    -210.8646   XXYY    -783.3269
      XYYY       1.2219   YYYY   -1012.0655   XXXZ    -109.2611
      XXYZ      58.3150   XYYZ    -101.6379   YYYZ     -35.3448
      XXZZ    -775.3717   XYZZ     -13.7519   YYZZ    -257.1836
      XZZZ    -261.8722   YZZZ     -50.1355   ZZZZ    -789.9783
    Traceless Hexadecapole Moments (Debye-Ang^3)
      XXXX    5701.4439   XXXY  -12088.0244   XXXZ    9802.2901
      XXYY   -1543.0518   XXYZ    6528.5198   XXZZ   -4158.3921
      XYYY   10181.0597   XYYZ   -3580.4146   XYZZ    1906.9647
      XZZZ   -6221.8755   YYYY   -2568.2449   YYYZ   -2494.8598
      YYZZ    4111.2967   YZZZ   -4033.6601   ZZZZ      47.0954
 -----------------------------------------------------------------

--------------------------------------------------
-  Entering drvman on Sun Oct 13 14:26:31 2024  -
--------------------------------------------------

 Calculating analytic gradient of the SCF energy
 Gradient of SCF Energy
           1           2           3           4           5           6
    1  -0.0000478   0.0001200  -0.0001783  -0.0002591  -0.0003827   0.0004969
    2   0.0000365  -0.0003096   0.0002931   0.0002886   0.0002728  -0.0003714
    3  -0.0000074  -0.0003508   0.0004785  -0.0001472  -0.0000753   0.0001212
           7           8           9          10          11          12
    1   0.0001425   0.0000273  -0.0000240   0.0001748   0.0003319   0.0000613
    2  -0.0000442  -0.0000390   0.0000557  -0.0002146   0.0002883  -0.0006873
    3   0.0000401  -0.0000561  -0.0000297   0.0005766  -0.0005653   0.0006264
          13          14          15          16          17          18
    1  -0.0001440  -0.0002121  -0.0000626   0.0000716   0.0001317  -0.0004872
    2   0.0002418  -0.0000816  -0.0000871   0.0002043  -0.0000078   0.0011192
    3  -0.0006433   0.0001519   0.0000055  -0.0000415  -0.0000722   0.0002906
          19          20          21          22          23          24
    1   0.0000495   0.0000838   0.0000210   0.0002012  -0.0000140  -0.0001558
```

```
    2  -0.0000539  -0.0000653  -0.0000073  -0.0001618  -0.0000236  -0.0001316
    3   0.0000087  -0.0000527  -0.0000104  -0.0001574   0.0000808  -0.0000460
           25          26          27          28          29          30
    1   0.0000474   0.0000629  -0.0002355  -0.0000701   0.0000687  -0.0000713
    2  -0.0000201   0.0000658   0.0000709  -0.0000019   0.0000688   0.0000696
    3   0.0000598  -0.0000858  -0.0000601   0.0000844   0.0000490  -0.0000086
           31          32          33          34          35          36
    1   0.0000122   0.0000218  -0.0000133  -0.0000395  -0.0000524  -0.0000279
    2  -0.0000543  -0.0001124  -0.0001140   0.0000538  -0.0000021  -0.0000159
    3  -0.0000588   0.0000084   0.0000984   0.0000360   0.0000162   0.0000780
           37          38
    1   0.0003752  -0.0000241
    2  -0.0002842  -0.0002379
    3   0.0000204  -0.0003624
 Max gradient component =       1.119E-03
 RMS gradient           =       2.329E-04
 Gradient time:  CPU 176.61 s  wall 374.72 s

-------------------------------------------------
-  Entering optman on Sun Oct 13 14:32:46 2024  -
-------------------------------------------------

 Geometry Optimization Parameters
   NAtoms,    NIC,     NZ,  NCons,   NDum,   NFix, NCnnct, MaxDiis
       38     272       0       0       0       0       0       0

 Cartesian Hessian Update
 Hessian updated using BFGS update


** GEOMETRY OPTIMIZATION IN DELOCALIZED INTERNAL COORDINATES **
   Searching for a Minimum

   Optimization Cycle:   9

                    Coordinates (Angstroms)
     ATOM                X               Y               Z
      1  N         2.3493281553    0.4298149209    0.1715587685
      2  C         3.1859228927    0.0248141965    1.1672425993
      3  C         4.0290894643   -1.0512468583    0.9151273064
      4  C         4.0300997661   -1.6890356430   -0.3127655487
      5  C         3.1658658498   -1.2450757491   -1.3121101117
      6  C         2.3427563030   -0.1872997674   -1.0379758560
      7  C         3.2071074902    0.7266077348    2.4901238363
      8  C         1.4385998725    1.5986878589    0.3419471234
      9  N        -2.0447412423    0.3784605187    0.9499274396
     10  C        -2.6657988447    0.5827151837   -0.2453705112
     11  N        -2.0243177905    1.1459241777   -1.2341964988
     12  C        -0.7438999775    1.5317058713   -1.0859088785
     13  C        -0.0173066525    1.2588442052    0.1230954824
     14  C        -0.7445603827    0.6968582166    1.1291513446
     15  C        -4.0681320364    0.1134696441   -0.4118545722
     16  C        -4.1161753885   -1.2950903158   -1.0489810447
     17  C        -3.4540951290   -2.3782407909   -0.2043883749
     18  N        -0.2139772241    2.1606647298   -2.1316348672
     19  H         4.6957910485   -1.3773371981    1.7029329520
     20  H         4.6998710967   -2.5216819070   -0.4946208394
     21  H         3.1366351491   -1.7072532295   -2.2898448050
     22  H         1.6551443410    0.2054057721   -1.7754908185
     23  H         3.9198647447    0.2334562588    3.1483364489
     24  H         2.2335631961    0.6989348238    2.9864768911
     25  H         3.5206905617    1.7692735939    2.3897558633
     26  H         1.7904287180    2.3800966854   -0.3329588240
     27  H         1.5727943812    1.9914200943    1.3447471780
     28  H        -2.5704996120   -0.0438425260    1.7062249146
     29  H        -0.3301010196    0.4822340147    2.1071072668
     30  H        -4.5766954158    0.1030103537    0.5571499821
     31  H        -4.5795883073    0.8286578176   -1.0570737562
     32  H        -3.6640022650   -1.2491224788   -2.0432196035
     33  H        -5.1714313024   -1.5325881590   -1.1963775435
```

```
    34  H        -2.3725910170   -2.2283090855   -0.1069765100
    35  H        -3.5966948516   -3.3576482423   -0.6627008398
    36  H        -3.8897111126   -2.4273568678    0.7990784700
    37  H         0.6845361455    2.6137432316   -2.1178187546
    38  H        -0.8137696053    2.3363289144   -2.9277153088
  Point Group: c1    Number of degrees of freedom:   108


  Energy is   -763.967071302

 Hessian updated using BFGS update
  internal optimization (0)

108 Hessian modes will be used to form the next step
  Hessian Eigenvalues:
     0.001594    0.002511    0.002844    0.004256    0.006301    0.012620
     0.014362    0.016055    0.018886    0.020004    0.020080    0.021774
     0.022553    0.022776    0.023594    0.024646    0.025891    0.026009
     0.027019    0.028211    0.028817    0.030029    0.030889    0.036306
     0.038837    0.041597    0.043259    0.043859    0.044598    0.046446
     0.052572    0.055498    0.056671    0.059144    0.061618    0.080163
     0.085665    0.092188    0.121769    0.122345    0.127129    0.130762
     0.132386    0.132897    0.138947    0.141446    0.142758    0.144416
     0.144758    0.147681    0.148509    0.150236    0.152611    0.153105
     0.157989    0.167786    0.195168    0.206561    0.209650    0.216445
     0.228746    0.229596    0.237051    0.246290    0.250266    0.258702
     0.268199    0.273512    0.286477    0.292641    0.300181    0.300622
     0.300861    0.301295    0.301899    0.303064    0.304101    0.304932
     0.305229    0.305279    0.306487    0.308445    0.312673    0.316416
     0.323159    0.333092    0.333678    0.334984    0.339942    0.343198
     0.349918    0.359220    0.369291    0.380901    0.384100    0.389722
     0.395780    0.403060    0.410334    0.416148    0.420804    0.438808
     0.448802    0.471347    0.479506    0.519858    0.579220    0.741566

 Minimum search - taking simple RFO step
 Searching for Lamda that Minimizes Along All modes
 Value Taken    Lamda =  -0.00011923
 Step Taken.  Stepsize is  0.157302

                            Maximum     Tolerance    Cnvgd?
        Gradient            0.001388      0.000800      NO
        Displacement        0.045754      0.001400      NO
        Energy change      -0.000124      0.000228     YES


 New Cartesian Coordinates Obtained by Inverse Iteration

 Displacement from previous Coordinates is:  0.825446
 ----------------------------------------------------------------
             Standard Nuclear Orientation (Angstroms)
    I     Atom           X                Y                Z
 ----------------------------------------------------------------
    1      N       2.3442480235     0.4162253384     0.1952167821
    2      C       3.2245421891     0.0534505935     1.1695227713
    3      C       4.0681803504    -1.0242892199     0.9200460844
    4      C       4.0285381126    -1.7036732812    -0.2843261729
    5      C       3.1240612158    -1.2977746023    -1.2640558537
    6      C       2.3000713495    -0.2383828922    -0.9936017907
    7      C       3.2919254497     0.8007259851     2.4655447577
    8      C       1.4296661876     1.5824006014     0.3638689882
    9      N      -2.0508465511     0.3449256512     0.9491327485
   10      C      -2.6744453560     0.5771736168    -0.2396362971
   11      N      -2.0342749252     1.1575355007    -1.2185872341
   12      C      -0.7512076134     1.5344002386    -1.0650335702
   13      C      -0.0247552003     1.2408478279     0.1389740123
   14      C      -0.7502569539     0.6585045816     1.1340734937
   15      C      -4.0797297475     0.1181437672    -0.4110320787
   16      C      -4.1387324718    -1.2728533360    -1.0842815683
   17      C      -3.4778340938    -2.3810415751    -0.2716983933
   18      N      -0.2188783651     2.1757552695    -2.1020941389
```

```
    19       H        4.7677357267    -1.3178661794     1.6920705890
    20       H        4.6977830014    -2.5369732522    -0.4639289160
    21       H        3.0629643429    -1.7912542176    -2.2249724862
    22       H        1.5834507352     0.1252158386    -1.7181776227
    23       H        4.0489825660     0.3482504210     3.1030459010
    24       H        2.3446055320     0.7658296802     3.0103420721
    25       H        3.5722415588     1.8467745624     2.3153365909
    26       H        1.7810394338     2.3667730004    -0.3077092384
    27       H        1.5583955164     1.9727630557     1.3683776203
    28       H       -2.5759370344    -0.0949287175     1.6958436453
    29       H       -0.3346348069     0.4216339799     2.1064897757
    30       H       -4.5831146124     0.0845284630     0.5601733236
    31       H       -4.5914955735     0.8527317648    -1.0338444707
    32       H       -3.6909712239    -1.2037703102    -2.0792355807
    33       H       -5.1957886853    -1.5013138214    -1.2328736032
    34       H       -2.3952472722    -2.2381420236    -0.1749662049
    35       H       -3.6264827620    -3.3472907193    -0.7552789818
    36       H       -3.9091747507    -2.4549725393     0.7320928078
    37       H        0.6877425115     2.6122324817    -2.0911258480
    38       H       -0.8123658036     2.3477044676    -2.9036919134
 ----------------------------------------------------------------
 Nuclear Repulsion Energy =        1312.66893569 hartrees
 There are       65 alpha and       65 beta electrons

-------------------------------------------------
-  Entering fldman on Sun Oct 13 14:32:46 2024  -
-------------------------------------------------

 Applying Cartesian multipole field
    Component           Value
    ---------           -----
     (2,0,0)        1.00000E-11
     (0,2,0)        2.00000E-11
     (0,0,2)       -3.00000E-11
 Nucleus-field energy     =     0.0000000331 hartrees

-------------------------------------------------
-  Entering gesman on Sun Oct 13 14:32:46 2024  -
-------------------------------------------------

 Requested basis set is 6-311+G(d,p)
 There are 188 shells and 516 basis functions
 A cutoff of  1.0D-12 yielded  12870 shell pairs
 There are    102139 function pairs (    108855 Cartesian)
 Smallest overlap matrix eigenvalue = 1.99E-06
 Linear dependence detected in AO basis
 Tighter screening thresholds may be required for diffuse basis sets
 Use S2THRESH > 12 and THRESH = 14 in case of SCF convergence issues
 Number of orthogonalized atomic orbitals = 510
 Maximum deviation from orthogonality = 1.571E-11
 Guess MOs from SCF MO coefficient file
 Reading MOs from coefficient file
 Reading MOs from coefficient file

-------------------------------------------------
-  Entering scfman on Sun Oct 13 14:32:46 2024  -
-------------------------------------------------

 Long-range K will be added via erf
 Coulomb attenuation parameter = 0.2 bohr**(-1)
 A restricted hybrid HF-DFT SCF calculation will be
 performed using Pulay DIIS + Geometric Direct Minimization
 Exchange:     0.2220 Hartree-Fock + 1.0000 wB97X-D + LR-HF
 Correlation:  1.0000 wB97X-D
 Using Euler-Maclaurin-Lebedev (75,302) quadrature formula
 Dispersion:   Grimme D
 SCF converges when RMS gradient is below 1.0E-07
 Geometry optimization detected.  Setting ReadMinima to 0
 Setting SaveMinima to 0
 ---------------------------------------
```

```
  Cycle       Energy         DIIS Error
 ---------------------------------------
    1    -763.9518257843      6.69E-04
    2    -763.9665373124      8.48E-05
    3    -763.9670686070      4.61E-05
    4    -763.9671390287      2.44E-05
    5    -763.9671609657      9.28E-06
    6    -763.9671641997      2.41E-06
    7    -763.9671644430      7.45E-07
    8    -763.9671644753      3.50E-07
    9    -763.9671644808      7.82E-08 Convergence criterion met
 ---------------------------------------
 SCF time:  CPU 343.36 s  wall 697.94 s
 SCF   energy in the final basis set = -763.96716448
 Total energy in the final basis set = -763.96716448

-------------------------------------------------
-  Entering anlman on Sun Oct 13 14:44:24 2024  -
-------------------------------------------------



 --------------------------------------------------------------

                   Orbital Energies (a.u.)
 --------------------------------------------------------------

 Alpha MOs
 -- Occupied --
-14.8152 -14.7901 -14.7392 -14.7078 -10.6781 -10.6622 -10.6485 -10.6398
-10.6350 -10.6257 -10.6053 -10.5832 -10.5730 -10.5701 -10.5481 -10.5265
-10.4978 -10.4759  -1.4158  -1.4034  -1.3487  -1.2763  -1.2304  -1.1998
 -1.1872  -1.1426  -1.1013  -1.0829  -1.0572  -1.0549  -1.0299  -1.0087
 -0.9897  -0.9551  -0.9461  -0.9182  -0.9096  -0.8937  -0.8840  -0.8656
 -0.8504  -0.8431  -0.8394  -0.8194  -0.8119  -0.7983  -0.7887  -0.7822
 -0.7737  -0.7578  -0.7494  -0.7474  -0.7455  -0.7280  -0.7243  -0.7158
 -0.7037  -0.6715  -0.6579  -0.6465  -0.6387  -0.6311  -0.6255  -0.6212
 -0.6106
 -- Virtual --
 -0.2711  -0.2594  -0.2447  -0.2254  -0.1740  -0.1642  -0.1471  -0.1440
 -0.1333  -0.1269  -0.1204  -0.1119  -0.1066  -0.1054  -0.0989  -0.0920
 -0.0890  -0.0835  -0.0825  -0.0783  -0.0750  -0.0719  -0.0712  -0.0658
 -0.0656  -0.0624  -0.0574  -0.0518  -0.0486  -0.0453  -0.0399  -0.0370
 -0.0334  -0.0314  -0.0298  -0.0252  -0.0212  -0.0147  -0.0114  -0.0070
 -0.0056  -0.0037  -0.0021  -0.0004   0.0090   0.0090   0.0113   0.0171
  0.0193   0.0210   0.0254   0.0267   0.0313   0.0337   0.0370   0.0378
  0.0430   0.0450   0.0488   0.0510   0.0536   0.0570   0.0604   0.0663
  0.0676   0.0709   0.0764   0.0778   0.0811   0.0825   0.0863   0.0885
  0.0976   0.0992   0.1005   0.1036   0.1090   0.1122   0.1133   0.1152
  0.1177   0.1214   0.1240   0.1296   0.1336   0.1369   0.1411   0.1436
  0.1458   0.1505   0.1593   0.1635   0.1660   0.1702   0.1723   0.1783
  0.1815   0.1848   0.1866   0.1936   0.1997   0.2011   0.2171   0.2233
  0.2325   0.2403   0.2440   0.2643   0.2772   0.2864   0.2960   0.3132
  0.3210   0.3284   0.3352   0.3368   0.3505   0.3538   0.3619   0.3628
  0.3734   0.3795   0.3832   0.3919   0.4006   0.4062   0.4235   0.4250
  0.4298   0.4360   0.4441   0.4495   0.4558   0.4591   0.4668   0.4748
  0.4876   0.4887   0.4992   0.5030   0.5090   0.5122   0.5159   0.5196
  0.5277   0.5306   0.5330   0.5407   0.5422   0.5517   0.5547   0.5567
  0.5638   0.5675   0.5718   0.5792   0.5872   0.5887   0.5975   0.6063
  0.6108   0.6189   0.6273   0.6311   0.6409   0.6537   0.6545   0.6585
  0.6628   0.6713   0.6783   0.6806   0.6889   0.6965   0.6987   0.7081
  0.7155   0.7188   0.7427   0.7467   0.7527   0.7602   0.7747   0.7793
  0.7873   0.7989   0.8109   0.8246   0.8371   0.8424   0.8456   0.8559
  0.8605   0.8722   0.8869   0.8951   0.9123   0.9172   0.9259   0.9280
  0.9527   0.9581   0.9750   0.9776   0.9868   1.0005   1.0242   1.0416
  1.0523   1.0552   1.0725   1.0766   1.1119   1.1535   1.1751   1.1807
  1.1937   1.2034   1.2124   1.2319   1.2568   1.2792   1.2974   1.3043
  1.3113   1.3207   1.3293   1.3388   1.3465   1.3581   1.3688   1.3744
  1.3880   1.3923   1.4000   1.4074   1.4149   1.4242   1.4289   1.4338
  1.4355   1.4468   1.4528   1.4651   1.4698   1.4724   1.4811   1.4884
  1.4940   1.5011   1.5189   1.5223   1.5303   1.5375   1.5444   1.5565
```

| | | | | | | | |
|---|---|---|---|---|---|---|---|
| 1.5626 | 1.5660 | 1.5742 | 1.5789 | 1.5875 | 1.5970 | 1.6099 | 1.6200 |
| 1.6267 | 1.6321 | 1.6348 | 1.6557 | 1.6634 | 1.6698 | 1.6757 | 1.6826 |
| 1.6974 | 1.6993 | 1.7143 | 1.7309 | 1.7404 | 1.7466 | 1.7566 | 1.7628 |
| 1.7871 | 1.7941 | 1.8061 | 1.8227 | 1.8326 | 1.8402 | 1.8663 | 1.8790 |
| 1.8897 | 1.9075 | 1.9114 | 1.9353 | 1.9505 | 1.9674 | 1.9768 | 1.9887 |
| 1.9946 | 2.0040 | 2.0240 | 2.0468 | 2.0581 | 2.0631 | 2.0697 | 2.0958 |
| 2.1174 | 2.1208 | 2.1289 | 2.1389 | 2.1708 | 2.1849 | 2.2183 | 2.2241 |
| 2.2273 | 2.2411 | 2.2482 | 2.2547 | 2.2720 | 2.2770 | 2.2795 | 2.2892 |
| 2.2989 | 2.3084 | 2.3362 | 2.3564 | 2.3590 | 2.3706 | 2.3870 | 2.3968 |
| 2.4041 | 2.4094 | 2.4316 | 2.4376 | 2.4418 | 2.4545 | 2.4619 | 2.4833 |
| 2.4960 | 2.5189 | 2.5234 | 2.5307 | 2.5448 | 2.5464 | 2.5503 | 2.5594 |
| 2.5625 | 2.5709 | 2.5796 | 2.5900 | 2.5966 | 2.6006 | 2.6054 | 2.6187 |
| 2.6311 | 2.6389 | 2.6651 | 2.6704 | 2.6744 | 2.6757 | 2.6895 | 2.7057 |
| 2.7153 | 2.7329 | 2.7370 | 2.7495 | 2.7557 | 2.7673 | 2.7696 | 2.7870 |
| 2.7893 | 2.8083 | 2.8133 | 2.8321 | 2.8451 | 2.8680 | 2.8820 | 2.8889 |
| 2.8936 | 2.9316 | 2.9677 | 2.9928 | 3.0560 | 3.0702 | 3.1254 | 3.1428 |
| 3.1629 | 3.2420 | 3.2463 | 3.2717 | 3.2970 | 3.3351 | 3.3701 | 3.4149 |
| 3.4176 | 3.4776 | 3.5190 | 3.5553 | 3.5619 | 3.5947 | 3.6281 | 3.6445 |
| 3.6626 | 3.6861 | 3.7005 | 3.7322 | 3.7376 | 3.7444 | 3.7536 | 3.7713 |
| 3.7747 | 3.8379 | 3.8720 | 3.9092 | 3.9400 | 3.9834 | 4.0260 | 4.0838 |
| 4.1105 | 4.2045 | 4.2960 | 4.3112 | 4.5953 | 4.6662 | 4.7069 | 4.7206 |
| 4.9244 | 5.0428 | 5.1989 | 23.5222 | 23.5743 | 23.7193 | 23.7292 | 23.7366 |
| 23.7761 | 23.8010 | 23.8236 | 23.8557 | 23.8843 | 23.9099 | 23.9678 | 23.9813 |
| 24.0362 | 35.4227 | 35.4581 | 35.5213 | 35.6215 | | | |

Ground-State Mulliken Net Atomic Charges

| Atom | Charge (a.u.) |
|---|---|
| 1 N | 0.425880 |
| 2 C | 0.115127 |
| 3 C | -0.004572 |
| 4 C | -0.147251 |
| 5 C | -0.195937 |
| 6 C | -0.172348 |
| 7 C | -0.769513 |
| 8 C | -0.326671 |
| 9 N | -0.140289 |
| 10 C | -0.272030 |
| 11 N | 0.007378 |
| 12 C | 0.299337 |
| 13 C | -0.021728 |
| 14 C | 0.088095 |
| 15 C | -0.336824 |
| 16 C | -0.255145 |
| 17 C | -0.536966 |
| 18 N | -0.481580 |
| 19 H | 0.219972 |
| 20 H | 0.234522 |
| 21 H | 0.226750 |
| 22 H | 0.203369 |
| 23 H | 0.229694 |
| 24 H | 0.196698 |
| 25 H | 0.242193 |
| 26 H | 0.225597 |
| 27 H | 0.244089 |
| 28 H | 0.380670 |
| 29 H | 0.205395 |
| 30 H | 0.214708 |
| 31 H | 0.250940 |
| 32 H | 0.204278 |
| 33 H | 0.225464 |
| 34 H | 0.130517 |
| 35 H | 0.216341 |
| 36 H | 0.178545 |
| 37 H | 0.317170 |
| 38 H | 0.378128 |

Sum of atomic charges = 2.000000

```
-------------------------------------------------------------------
                   Cartesian Multipole Moments
-------------------------------------------------------------------
    Charge (ESU x 10^10)
                9.6064
    Dipole Moment (Debye)
         X       5.7294      Y       2.6872      Z       1.2817
       Tot       6.4568
    Quadrupole Moments (Debye-Ang)
        XX     -11.2901     XY      -3.0894     YY     -76.9350
        XZ       6.2211     YZ      -2.6165     ZZ     -69.6551
    Traceless Quadrupole Moments (Debye-Ang)
       QXX     124.0098    QYY     -72.9247    QZZ     -51.0851
       QXY      -9.2681    QXZ      18.6632    QYZ      -7.8494
    Octopole Moments (Debye-Ang^2)
       XXX      20.0203    XXY     -90.3847    XYY      21.6956
       YYY     -24.0653    XXZ      46.4572    XYZ      11.0879
       YYZ     -26.2786    XZZ      19.1381    YZZ      14.6163
       ZZZ      24.2212
    Traceless Octopole Moments (Debye-Ang^2)
       XXX    -247.3823    YYY     537.5241    ZZZ     -36.2809
       XXY   -1056.2696    XXZ     563.6591    XYY     142.8722
       XYZ     166.3191    XZZ     104.5101    YYZ    -527.3782
       YZZ     518.7455
    Hexadecapole Moments (Debye-Ang^3)
      XXXX   -3573.5240   XXXY    -207.1910   XXYY    -790.2602
      XYYY      -4.1705   YYYY   -1017.9688   XXXZ    -104.4851
      XXYZ      58.6365   XYYZ    -108.7365   YYYZ     -41.5008
      XXZZ    -781.6510   XYZZ      -9.1113   YYZZ    -256.0492
      XZZZ    -280.0292   YZZZ     -56.7295   ZZZZ    -782.0730
    Traceless Hexadecapole Moments (Debye-Ang^3)
      XXXX    6603.7689   XXXY  -11833.7841   XXXZ   11225.3478
      XXYY   -1920.0763   XXYZ    6739.7977   XXZZ   -4683.6926
      XYYY    9483.3769   XYYZ   -4018.5668   XYZZ    2350.4072
      XZZZ   -7206.7810   YYYY   -2367.0657   YYYZ   -2608.6912
      YYZZ    4287.1420   YZZZ   -4131.1065   ZZZZ     396.5505
-------------------------------------------------------------------

--------------------------------------------------
-  Entering drvman on Sun Oct 13 14:44:24 2024  -
--------------------------------------------------

 Calculating analytic gradient of the SCF energy
 Gradient of SCF Energy
            1           2           3           4           5           6
    1  -0.0001948  -0.0003065   0.0001035   0.0001192   0.0002780  -0.0003177
    2   0.0001121   0.0003293  -0.0002764  -0.0002088  -0.0002242   0.0005063
    3   0.0001784  -0.0000655  -0.0000929  -0.0000910   0.0001670   0.0001191
            7           8           9          10          11          12
    1   0.0001755   0.0000686  -0.0000246   0.0006094  -0.0004223  -0.0000027
    2  -0.0001116  -0.0000748   0.0002282   0.0000789   0.0001407  -0.0011097
    3   0.0001076  -0.0002379  -0.0002768   0.0003959  -0.0003855   0.0013020
           13          14          15          16          17          18
    1  -0.0004374   0.0001708   0.0000382  -0.0000243   0.0000648  -0.0001992
    2  -0.0000705   0.0001137  -0.0000899   0.0002680  -0.0000651   0.0018854
    3  -0.0004239  -0.0000567  -0.0001296   0.0000277  -0.0000738   0.0003320
           19          20          21          22          23          24
    1   0.0000868   0.0000106   0.0000239   0.0002278   0.0000090  -0.0001219
    2  -0.0000972   0.0000130  -0.0000649  -0.0001139   0.0000142  -0.0000951
    3   0.0000035  -0.0000333  -0.0000164  -0.0002154   0.0000007  -0.0000118
           25          26          27          28          29          30
    1   0.0000644  -0.0000769  -0.0001812  -0.0001239   0.0000342  -0.0000636
    2  -0.0000168  -0.0000185   0.0001739  -0.0000622  -0.0000038   0.0000515
    3   0.0000758  -0.0000787  -0.0001330   0.0000291   0.0000586  -0.0000095
           31          32          33          34          35          36
    1   0.0000127   0.0000418   0.0000095  -0.0000189  -0.0000439  -0.0000164
    2  -0.0000473  -0.0001628  -0.0001391   0.0000366   0.0000060  -0.0000202
    3  -0.0000024   0.0000152   0.0000556   0.0000393   0.0000106   0.0000723
           37          38
```

```
    1   0.0003757   0.0000517
    2  -0.0002247  -0.0006604
    3  -0.0000840  -0.0005724
 Max gradient component =       1.885E-03
 RMS gradient           =       3.049E-04
 Gradient time:  CPU 168.27 s  wall 314.86 s

-------------------------------------------------
-  Entering optman on Sun Oct 13 14:49:39 2024  -
-------------------------------------------------

 Geometry Optimization Parameters
   NAtoms,    NIC,     NZ,  NCons,   NDum,   NFix, NCnnct, MaxDiis
       38     272       0       0       0       0       0       0

 Cartesian Hessian Update
 Hessian updated using BFGS update


** GEOMETRY OPTIMIZATION IN DELOCALIZED INTERNAL COORDINATES **
   Searching for a Minimum

   Optimization Cycle:  10

                     Coordinates (Angstroms)
     ATOM                X               Y               Z
      1  N         2.3442480235    0.4162253384    0.1952167821
      2  C         3.2245421891    0.0534505935    1.1695227713
      3  C         4.0681803504   -1.0242892199    0.9200460844
      4  C         4.0285381126   -1.7036732812   -0.2843261729
      5  C         3.1240612158   -1.2977746023   -1.2640558537
      6  C         2.3000713495   -0.2383828922   -0.9936017907
      7  C         3.2919254497    0.8007259851    2.4655447577
      8  C         1.4296661876    1.5824006014    0.3638689882
      9  N        -2.0508465511    0.3449256512    0.9491327485
     10  C        -2.6744453560    0.5771736168   -0.2396362971
     11  N        -2.0342749252    1.1575355007   -1.2185872341
     12  C        -0.7512076134    1.5344002386   -1.0650335702
     13  C        -0.0247552003    1.2408478279    0.1389740123
     14  C        -0.7502569539    0.6585045816    1.1340734937
     15  C        -4.0797297475    0.1181437672   -0.4110320787
     16  C        -4.1387324718   -1.2728533360   -1.0842815683
     17  C        -3.4778340938   -2.3810415751   -0.2716983933
     18  N        -0.2188783651    2.1757552695   -2.1020941389
     19  H         4.7677357267   -1.3178661794    1.6920705890
     20  H         4.6977830014   -2.5369732522   -0.4639289160
     21  H         3.0629643429   -1.7912542176   -2.2249724862
     22  H         1.5834507352    0.1252158386   -1.7181776227
     23  H         4.0489825660    0.3482504210    3.1030459010
     24  H         2.3446055320    0.7658296802    3.0103420721
     25  H         3.5722415588    1.8467745624    2.3153365909
     26  H         1.7810394338    2.3667730004   -0.3077092384
     27  H         1.5583955164    1.9727630557    1.3683776203
     28  H        -2.5759370344   -0.0949287175    1.6958436453
     29  H        -0.3346348069    0.4216339799    2.1064897757
     30  H        -4.5831146124    0.0845284630    0.5601733236
     31  H        -4.5914955735    0.8527317648   -1.0338444707
     32  H        -3.6909712239   -1.2037703102   -2.0792355807
     33  H        -5.1957886853   -1.5013138214   -1.2328736032
     34  H        -2.3952472722   -2.2381420236   -0.1749662049
     35  H        -3.6264827620   -3.3472907193   -0.7552789818
     36  H        -3.9091747507   -2.4549725393    0.7320928078
     37  H         0.6877425115    2.6122324817   -2.0911258480
     38  H        -0.8123658036    2.3477044676   -2.9036919134
   Point Group: c1    Number of degrees of freedom:   108


   Energy is   -763.967164481

 Hessian updated using BFGS update
```

internal optimization (0)

108 Hessian modes will be used to form the next step
Hessian Eigenvalues:

| | | | | | |
|---|---|---|---|---|---|
| 0.001183 | 0.001935 | 0.002612 | 0.004041 | 0.006239 | 0.012152 |
| 0.013344 | 0.015116 | 0.018904 | 0.019933 | 0.020007 | 0.021762 |
| 0.022495 | 0.022758 | 0.023589 | 0.024635 | 0.025877 | 0.025948 |
| 0.026989 | 0.028195 | 0.029261 | 0.029820 | 0.030487 | 0.036254 |
| 0.038822 | 0.041848 | 0.043299 | 0.043859 | 0.044487 | 0.046628 |
| 0.053119 | 0.055091 | 0.055653 | 0.058872 | 0.061211 | 0.080041 |
| 0.085592 | 0.092087 | 0.121704 | 0.122343 | 0.127132 | 0.130355 |
| 0.132390 | 0.132898 | 0.138943 | 0.141329 | 0.142950 | 0.143591 |
| 0.146224 | 0.147677 | 0.147807 | 0.149904 | 0.152617 | 0.153100 |
| 0.157324 | 0.175841 | 0.195115 | 0.206758 | 0.209700 | 0.216891 |
| 0.225173 | 0.230128 | 0.236529 | 0.247689 | 0.251968 | 0.258576 |
| 0.268645 | 0.273824 | 0.285684 | 0.291182 | 0.300168 | 0.300611 |
| 0.300925 | 0.301301 | 0.301897 | 0.303134 | 0.304042 | 0.304939 |
| 0.305253 | 0.305288 | 0.306594 | 0.308391 | 0.312913 | 0.316189 |
| 0.323800 | 0.333058 | 0.333766 | 0.335632 | 0.340440 | 0.343396 |
| 0.349944 | 0.359899 | 0.368842 | 0.380516 | 0.388274 | 0.391998 |
| 0.395752 | 0.403836 | 0.415872 | 0.417883 | 0.425652 | 0.437458 |
| 0.447113 | 0.475664 | 0.487537 | 0.528668 | 0.577257 | 0.753287 |

Minimum search - taking simple RFO step
Searching for Lamda that Minimizes Along All modes
Value Taken Lamda = -0.00021428
Calculated Step too Large. Step scaled by 0.965835
Step Taken. Stepsize is 0.300000

| | Maximum | Tolerance | Cnvgd? |
|---|---|---|---|
| Gradient | 0.001018 | 0.000800 | NO |
| Displacement | 0.089695 | 0.001400 | NO |
| Energy change | -0.000093 | 0.000228 | YES |

New Cartesian Coordinates Obtained by Inverse Iteration

Displacement from previous Coordinates is: 1.613642

Standard Nuclear Orientation (Angstroms)

| I | Atom | X | Y | Z |
|---|---|---|---|---|
| 1 | N | 2.3367228653 | 0.3863463316 | 0.2440910554 |
| 2 | C | 3.2982503562 | 0.1079074058 | 1.1685638008 |
| 3 | C | 4.1481168023 | -0.9659357091 | 0.9243729411 |
| 4 | C | 4.0359510833 | -1.7230129951 | -0.2278905279 |
| 5 | C | 3.0542986116 | -1.3969975657 | -1.1623630599 |
| 6 | C | 2.2247424883 | -0.3409337914 | -0.8968613260 |
| 7 | C | 3.4445330009 | 0.9423182930 | 2.4032476769 |
| 8 | C | 1.4124541076 | 1.5458824943 | 0.4107847192 |
| 9 | N | -2.0643669701 | 0.2817405531 | 0.9533206460 |
| 10 | C | -2.6918464610 | 0.5632708991 | -0.2227527705 |
| 11 | N | -2.0507176435 | 1.1699878273 | -1.1848194674 |
| 12 | C | -0.7627186842 | 1.5278450885 | -1.0248511933 |
| 13 | C | -0.0386654816 | 1.2012520683 | 0.1720177015 |
| 14 | C | -0.7631000186 | 0.5859855780 | 1.1476867660 |
| 15 | C | -4.1038685069 | 0.1276936831 | -0.4013752712 |
| 16 | C | -4.1868402738 | -1.2216038824 | -1.1516924832 |
| 17 | C | -3.5238559857 | -2.3791454226 | -0.4127954284 |
| 18 | N | -0.2240167153 | 2.1811463445 | -2.0505662034 |
| 19 | H | 4.9099039595 | -1.1942234567 | 1.6584753703 |
| 20 | H | 4.7092164049 | -2.5538546924 | -0.4035330629 |
| 21 | H | 2.9367272123 | -1.9506945316 | -2.0843938211 |
| 22 | H | 1.4499857975 | -0.0371930324 | -1.5878687807 |
| 23 | H | 4.2759301685 | 0.5653704492 | 2.9955656526 |
| 24 | H | 2.5520975873 | 0.9030513422 | 3.0344130006 |
| 25 | H | 3.6582047249 | 1.9869599760 | 2.1623339145 |
| 26 | H | 1.7636866113 | 2.3382161638 | -0.2516714524 |
| 27 | H | 1.5290180511 | 1.9283024713 | 1.4200663620 |
| 28 | H | -2.5889393449 | -0.1855418494 | 1.6834817498 |

```
   29      H      -0.3462138233     0.3130011619     2.1102281745
   30      H      -4.5949490329     0.0432065620     0.5730164379
   31      H      -4.6162578124     0.9021222436    -0.9736270910
   32      H      -3.7523241216    -1.0997929487    -2.1476003666
   33      H      -5.2479093466    -1.4318493319    -1.2981970412
   34      H      -2.4389124808    -2.2492153516    -0.3228123669
   35      H      -3.6860177829    -3.3154093975    -0.9481594615
   36      H      -3.9419058770    -2.5065764523     0.5911987194
   37      H       0.6949041574     2.5911330106    -2.0437174320
   38      H      -0.8113176271     2.3592404634    -2.8553160808
 ----------------------------------------------------------------
 Nuclear Repulsion Energy =        1311.24529974 hartrees
 There are       65 alpha and       65 beta electrons

--------------------------------------------------
-  Entering fldman on Sun Oct 13 14:49:39 2024  -
--------------------------------------------------

 Applying Cartesian multipole field
    Component          Value
    ---------          -----
     (2,0,0)        1.00000E-11
     (0,2,0)        2.00000E-11
     (0,0,2)       -3.00000E-11
 Nucleus-field energy     =     0.0000000347 hartrees

--------------------------------------------------
-  Entering gesman on Sun Oct 13 14:49:39 2024  -
--------------------------------------------------

 Requested basis set is 6-311+G(d,p)
 There are 188 shells and 516 basis functions
 A cutoff of  1.0D-12 yielded  12818 shell pairs
 There are    101877 function pairs (    108594 Cartesian)
 Smallest overlap matrix eigenvalue = 1.89E-06
 Linear dependence detected in AO basis
 Tighter screening thresholds may be required for diffuse basis sets
 Use S2THRESH > 12 and THRESH = 14 in case of SCF convergence issues
 Number of orthogonalized atomic orbitals = 510
 Maximum deviation from orthogonality = 8.419E-12
 Guess MOs from SCF MO coefficient file
 Reading MOs from coefficient file
 Reading MOs from coefficient file

--------------------------------------------------
-  Entering scfman on Sun Oct 13 14:49:40 2024  -
--------------------------------------------------

 Long-range K will be added via erf
 Coulomb attenuation parameter = 0.2 bohr**(-1)
 A restricted hybrid HF-DFT SCF calculation will be
 performed using Pulay DIIS + Geometric Direct Minimization
 Exchange:     0.2220 Hartree-Fock + 1.0000 wB97X-D + LR-HF
 Correlation:  1.0000 wB97X-D
 Using Euler-Maclaurin-Lebedev (75,302) quadrature formula
 Dispersion:   Grimme D
 SCF converges when RMS gradient is below 1.0E-07
 Geometry optimization detected.  Setting ReadMinima to 0
 Setting SaveMinima to 0
 ---------------------------------------
  Cycle       Energy         DIIS Error
 ---------------------------------------
    1    -763.9083487238      1.29E-03
    2    -763.9648075190      1.79E-04
    3    -763.9668068356      1.17E-04
    4    -763.9672273335      4.72E-05
    5    -763.9673097764      2.06E-05
    6    -763.9673257720      4.91E-06
    7    -763.9673268199      1.59E-06
    8    -763.9673269566      7.27E-07
```

```
    9    -763.9673269815      1.83E-07
   10    -763.9673269834      7.74E-08 Convergence criterion met
 ---------------------------------------
 SCF time:  CPU 341.89 s  wall 626.24 s
 SCF   energy in the final basis set = -763.96732698
 Total energy in the final basis set = -763.96732698

--------------------------------------------------
-  Entering anlman on Sun Oct 13 15:00:06 2024  -
--------------------------------------------------



 --------------------------------------------------------------

                    Orbital Energies (a.u.)
 --------------------------------------------------------------

 Alpha MOs
 -- Occupied --
-14.8152 -14.7901 -14.7391 -14.7077 -10.6780 -10.6621 -10.6485 -10.6393
-10.6350 -10.6255 -10.6054 -10.5830 -10.5729 -10.5697 -10.5468 -10.5265
-10.4974 -10.4756  -1.4158  -1.4032  -1.3486  -1.2762  -1.2303  -1.1998
 -1.1867  -1.1425  -1.1012  -1.0822  -1.0573  -1.0547  -1.0293  -1.0086
 -0.9894  -0.9553  -0.9458  -0.9181  -0.9096  -0.8935  -0.8835  -0.8650
 -0.8506  -0.8430  -0.8392  -0.8193  -0.8111  -0.7992  -0.7881  -0.7815
 -0.7731  -0.7579  -0.7488  -0.7475  -0.7449  -0.7277  -0.7240  -0.7153
 -0.7032  -0.6714  -0.6573  -0.6465  -0.6383  -0.6307  -0.6256  -0.6212
 -0.6102
 -- Virtual --
 -0.2706  -0.2593  -0.2447  -0.2255  -0.1743  -0.1638  -0.1485  -0.1442
 -0.1322  -0.1264  -0.1205  -0.1110  -0.1065  -0.1051  -0.0991  -0.0914
 -0.0890  -0.0840  -0.0834  -0.0776  -0.0747  -0.0717  -0.0699  -0.0668
 -0.0656  -0.0625  -0.0565  -0.0516  -0.0485  -0.0433  -0.0395  -0.0366
 -0.0332  -0.0326  -0.0300  -0.0253  -0.0211  -0.0150  -0.0106  -0.0075
 -0.0058  -0.0031  -0.0014  -0.0004   0.0085   0.0094   0.0107   0.0163
  0.0192   0.0212   0.0251   0.0280   0.0326   0.0339   0.0351   0.0393
  0.0435   0.0451   0.0486   0.0513   0.0535   0.0583   0.0605   0.0655
  0.0682   0.0706   0.0774   0.0781   0.0815   0.0850   0.0858   0.0897
  0.0979   0.0985   0.1000   0.1043   0.1076   0.1120   0.1127   0.1164
  0.1178   0.1218   0.1246   0.1292   0.1317   0.1356   0.1418   0.1424
  0.1464   0.1512   0.1594   0.1638   0.1675   0.1702   0.1724   0.1785
  0.1836   0.1845   0.1868   0.1942   0.1982   0.2042   0.2177   0.2241
  0.2325   0.2412   0.2452   0.2657   0.2780   0.2867   0.2979   0.3135
  0.3206   0.3278   0.3349   0.3377   0.3508   0.3533   0.3596   0.3629
  0.3763   0.3822   0.3845   0.3936   0.3976   0.4051   0.4203   0.4252
  0.4301   0.4365   0.4438   0.4531   0.4563   0.4576   0.4688   0.4735
  0.4863   0.4895   0.4995   0.5043   0.5106   0.5128   0.5175   0.5178
  0.5264   0.5295   0.5347   0.5412   0.5437   0.5508   0.5538   0.5542
  0.5652   0.5673   0.5727   0.5790   0.5873   0.5882   0.5980   0.6060
  0.6091   0.6240   0.6258   0.6333   0.6439   0.6522   0.6551   0.6599
  0.6621   0.6726   0.6780   0.6806   0.6887   0.6952   0.7006   0.7120
  0.7138   0.7244   0.7425   0.7444   0.7522   0.7598   0.7747   0.7794
  0.7875   0.7993   0.8124   0.8217   0.8321   0.8425   0.8468   0.8540
  0.8608   0.8745   0.8886   0.8929   0.9187   0.9223   0.9258   0.9270
  0.9516   0.9608   0.9694   0.9841   0.9905   1.0001   1.0250   1.0385
  1.0504   1.0542   1.0704   1.0806   1.1168   1.1494   1.1737   1.1794
  1.1894   1.2044   1.2167   1.2367   1.2569   1.2844   1.2878   1.3044
  1.3124   1.3177   1.3297   1.3377   1.3477   1.3572   1.3680   1.3759
  1.3890   1.3913   1.4025   1.4059   1.4188   1.4225   1.4279   1.4334
  1.4360   1.4481   1.4589   1.4663   1.4688   1.4738   1.4830   1.4887
  1.4974   1.5001   1.5198   1.5210   1.5273   1.5377   1.5410   1.5535
  1.5636   1.5662   1.5739   1.5811   1.5873   1.5983   1.6107   1.6197
  1.6278   1.6315   1.6362   1.6542   1.6651   1.6713   1.6749   1.6816
  1.6966   1.7014   1.7190   1.7297   1.7391   1.7484   1.7539   1.7639
  1.7880   1.7951   1.8043   1.8234   1.8361   1.8411   1.8664   1.8767
  1.8892   1.9111   1.9117   1.9350   1.9477   1.9698   1.9841   1.9933
  1.9957   2.0044   2.0284   2.0476   2.0553   2.0637   2.0728   2.0956
  2.1174   2.1192   2.1285   2.1380   2.1718   2.1851   2.2191   2.2249
  2.2279   2.2433   2.2478   2.2564   2.2682   2.2783   2.2805   2.2894
  2.2962   2.3104   2.3369   2.3581   2.3600   2.3683   2.3866   2.3974
```

```
  2.4033    2.4126    2.4336    2.4373    2.4416    2.4568    2.4611    2.4845
  2.4944    2.5168    2.5244    2.5305    2.5427    2.5477    2.5511    2.5599
  2.5644    2.5729    2.5815    2.5904    2.5955    2.6062    2.6071    2.6148
  2.6311    2.6386    2.6610    2.6626    2.6739    2.6750    2.6877    2.7046
  2.7124    2.7329    2.7389    2.7446    2.7593    2.7632    2.7727    2.7870
  2.7915    2.8091    2.8113    2.8301    2.8447    2.8763    2.8837    2.8902
  2.8920    2.9289    2.9657    2.9955    3.0577    3.0726    3.1335    3.1426
  3.1652    3.2429    3.2455    3.2735    3.2981    3.3350    3.3693    3.4150
  3.4205    3.4787    3.5209    3.5530    3.5611    3.5961    3.6213    3.6474
  3.6632    3.6859    3.7014    3.7314    3.7348    3.7431    3.7549    3.7707
  3.7767    3.8389    3.8721    3.9098    3.9394    3.9840    4.0267    4.0820
  4.1117    4.2048    4.2966    4.3128    4.5952    4.6662    4.7061    4.7205
  4.9255    5.0432    5.2000   23.5217   23.5733   23.7179   23.7297   23.7383
 23.7772   23.8009   23.8241   23.8561   23.8843   23.9105   23.9690   23.9820
 24.0367   35.4218   35.4571   35.5218   35.6220
 --------------------------------------------------------------

          Ground-State Mulliken Net Atomic Charges

     Atom                 Charge (a.u.)
  ----------------------------------------
      1 N                     0.424925
      2 C                     0.098784
      3 C                     0.000114
      4 C                    -0.142876
      5 C                    -0.200530
      6 C                    -0.163540
      7 C                    -0.749718
      8 C                    -0.347739
      9 N                    -0.138404
     10 C                    -0.284709
     11 N                     0.009495
     12 C                     0.314530
     13 C                    -0.038150
     14 C                     0.096959
     15 C                    -0.341353
     16 C                    -0.258561
     17 C                    -0.534210
     18 N                    -0.478234
     19 H                     0.220197
     20 H                     0.234358
     21 H                     0.226535
     22 H                     0.206759
     23 H                     0.229382
     24 H                     0.205544
     25 H                     0.237829
     26 H                     0.224900
     27 H                     0.245647
     28 H                     0.380323
     29 H                     0.209232
     30 H                     0.212922
     31 H                     0.251829
     32 H                     0.204396
     33 H                     0.225419
     34 H                     0.129784
     35 H                     0.216250
     36 H                     0.178335
     37 H                     0.316830
     38 H                     0.376745
  ----------------------------------------
  Sum of atomic charges =     2.000000

 -----------------------------------------------------------------
                    Cartesian Multipole Moments
 -----------------------------------------------------------------
    Charge (ESU x 10^10)
                9.6064
    Dipole Moment (Debye)
         X       5.7531      Y       2.5043      Z       1.4867
       Tot       6.4483
```

```
   Quadrupole Moments (Debye-Ang)
       XX      -9.3787     XY      -2.2224     YY     -76.8816
       XZ       7.7643     YZ      -1.8351     ZZ     -71.0178
   Traceless Quadrupole Moments (Debye-Ang)
      QXX     129.1420    QYY     -73.3668    QZZ     -55.7752
      QXY      -6.6673    QXZ      23.2929    QYZ      -5.5054
   Octopole Moments (Debye-Ang^2)
      XXX      29.5948    XXY     -86.7650    XYY      23.3732
      YYY     -23.0925    XXZ      51.2656    XYZ      13.1129
      YYZ     -27.4014    XZZ      15.7945    YZZ      14.6828
      ZZZ      22.1588
   Traceless Octopole Moments (Debye-Ang^2)
      XXX    -174.9411    YYY     510.1850    ZZZ     -81.8242
      XXY   -1015.9507    XXZ     630.9149    XYY     144.3109
      XYZ     196.6936    XZZ      30.6302    YYZ    -549.0907
      YZZ     505.7657
   Hexadecapole Moments (Debye-Ang^3)
     XXXX   -3574.7663   XXXY    -199.7311   XXYY    -804.7313
     XYYY     -16.0412   YYYY   -1023.4582   XXXZ     -97.4473
     XXYZ      59.8420   XYYZ    -120.7208   YYYZ     -53.8323
     XXZZ    -796.3722   XYZZ      -0.0713   YYZZ    -252.5899
     XZZZ    -315.1707   YZZZ     -70.3275   ZZZZ    -766.6064
   Traceless Hexadecapole Moments (Debye-Ang^3)
     XXXX    8827.8611   XXXY  -11258.8037   XXXZ   13768.2828
     XXYY   -2863.6981   XXYZ    7243.2609   XXZZ   -5964.1630
     XYYY    8028.6332   XYYZ   -4675.6026   XYZZ    3230.1705
     XZZZ   -9092.6801   YYYY   -1842.9282   YYYZ   -2772.8277
     YYZZ    4706.6263   YZZZ   -4470.4333   ZZZZ    1257.5367
 -----------------------------------------------------------------

--------------------------------------------------
-  Entering drvman on Sun Oct 13 15:00:06 2024  -
--------------------------------------------------

 Calculating analytic gradient of the SCF energy
 Gradient of SCF Energy
           1           2           3           4           5           6
    1  -0.0000938  -0.0001967   0.0002399   0.0000788   0.0002007  -0.0004721
    2   0.0001020   0.0000389  -0.0001184  -0.0003737   0.0001335   0.0003176
    3   0.0004832  -0.0000099  -0.0001672   0.0000641   0.0000558   0.0001320
           7           8           9          10          11          12
    1   0.0003347   0.0000051   0.0001241   0.0006250  -0.0003398  -0.0000904
    2   0.0000366  -0.0000568   0.0004199   0.0000816   0.0003688  -0.0011480
    3   0.0001642  -0.0005440  -0.0003072   0.0004426  -0.0005281   0.0016140
          13          14          15          16          17          18
    1  -0.0007543   0.0003478   0.0001546  -0.0002813   0.0000391  -0.0002218
    2  -0.0004052   0.0001083  -0.0000506   0.0002421  -0.0001406   0.0021476
    3  -0.0003715  -0.0001361  -0.0002518   0.0003353  -0.0001361   0.0004975
          19          20          21          22          23          24
    1   0.0000200  -0.0000444  -0.0000224   0.0002489  -0.0000498  -0.0000728
    2  -0.0000842   0.0000029  -0.0000484   0.0000441  -0.0000599  -0.0000807
    3  -0.0000205  -0.0000080   0.0000087  -0.0001425  -0.0000274   0.0000891
          25          26          27          28          29          30
    1   0.0000246  -0.0002074  -0.0000449  -0.0001320  -0.0000044  -0.0000379
    2  -0.0000612  -0.0001104   0.0002938  -0.0001325  -0.0001177  -0.0000140
    3  -0.0000134  -0.0001266  -0.0001833  -0.0000848   0.0000738  -0.0000242
          31          32          33          34          35          36
    1   0.0000213   0.0000465   0.0000327   0.0000107  -0.0000009   0.0000054
    2  -0.0000442  -0.0001439  -0.0000817  -0.0000307  -0.0000008  -0.0000030
    3   0.0000591  -0.0000361  -0.0000515   0.0000418   0.0000176   0.0000443
          37          38
    1   0.0003586   0.0001483
    2  -0.0000347  -0.0009962
    3  -0.0002335  -0.0007195
 Max gradient component =       2.148E-03
 RMS gradient           =       3.650E-04
 Gradient time:  CPU 160.52 s  wall 294.63 s

--------------------------------------------------
-  Entering optman on Sun Oct 13 15:05:01 2024  -
```

```
-------------------------------------------------

 Geometry Optimization Parameters
   NAtoms,    NIC,     NZ,  NCons,   NDum,   NFix, NCnnct, MaxDiis
       38     272       0       0       0       0       0       0

 Cartesian Hessian Update
 Hessian updated using BFGS update


** GEOMETRY OPTIMIZATION IN DELOCALIZED INTERNAL COORDINATES **
   Searching for a Minimum

   Optimization Cycle:  11

                    Coordinates (Angstroms)
     ATOM                X               Y               Z
      1  N         2.3367228653    0.3863463316    0.2440910554
      2  C         3.2982503562    0.1079074058    1.1685638008
      3  C         4.1481168023   -0.9659357091    0.9243729411
      4  C         4.0359510833   -1.7230129951   -0.2278905279
      5  C         3.0542986116   -1.3969975657   -1.1623630599
      6  C         2.2247424883   -0.3409337914   -0.8968613260
      7  C         3.4445330009    0.9423182930    2.4032476769
      8  C         1.4124541076    1.5458824943    0.4107847192
      9  N        -2.0643669701    0.2817405531    0.9533206460
     10  C        -2.6918464610    0.5632708991   -0.2227527705
     11  N        -2.0507176435    1.1699878273   -1.1848194674
     12  C        -0.7627186842    1.5278450885   -1.0248511933
     13  C        -0.0386654816    1.2012520683    0.1720177015
     14  C        -0.7631000186    0.5859855780    1.1476867660
     15  C        -4.1038685069    0.1276936831   -0.4013752712
     16  C        -4.1868402738   -1.2216038824   -1.1516924832
     17  C        -3.5238559857   -2.3791454226   -0.4127954284
     18  N        -0.2240167153    2.1811463445   -2.0505662034
     19  H         4.9099039595   -1.1942234567    1.6584753703
     20  H         4.7092164049   -2.5538546924   -0.4035330629
     21  H         2.9367272123   -1.9506945316   -2.0843938211
     22  H         1.4499857975   -0.0371930324   -1.5878687807
     23  H         4.2759301685    0.5653704492    2.9955656526
     24  H         2.5520975873    0.9030513422    3.0344130006
     25  H         3.6582047249    1.9869599760    2.1623339145
     26  H         1.7636866113    2.3382161638   -0.2516714524
     27  H         1.5290180511    1.9283024713    1.4200663620
     28  H        -2.5889393449   -0.1855418494    1.6834817498
     29  H        -0.3462138233    0.3130011619    2.1102281745
     30  H        -4.5949490329    0.0432065620    0.5730164379
     31  H        -4.6162578124    0.9021222436   -0.9736270910
     32  H        -3.7523241216   -1.0997929487   -2.1476003666
     33  H        -5.2479093466   -1.4318493319   -1.2981970412
     34  H        -2.4389124808   -2.2492153516   -0.3228123669
     35  H        -3.6860177829   -3.3154093975   -0.9481594615
     36  H        -3.9419058770   -2.5065764523    0.5911987194
     37  H         0.6949041574    2.5911330106   -2.0437174320
     38  H        -0.8113176271    2.3592404634   -2.8553160808
   Point Group: c1    Number of degrees of freedom:   108


   Energy is   -763.967326983

 Hessian updated using BFGS update
  internal optimization (0)

108 Hessian modes will be used to form the next step
  Hessian Eigenvalues:
     0.000756    0.001842    0.002597    0.003942    0.006160    0.011237
     0.013988    0.015209    0.019089    0.019871    0.020010    0.021771
     0.022460    0.022769    0.023582    0.024615    0.025809    0.025927
     0.026995    0.028128    0.029378    0.029703    0.030912    0.036290
     0.038834    0.042074    0.043318    0.043854    0.044362    0.046641
```

```
    0.051974    0.053209    0.055554    0.058492    0.061315    0.079805
    0.085541    0.092006    0.121660    0.122403    0.127133    0.130111
    0.132423    0.132903    0.139959    0.141847    0.143400    0.143582
    0.146964    0.147680    0.148671    0.149788    0.152627    0.153115
    0.157647    0.175262    0.195576    0.206734    0.210004    0.216397
    0.223020    0.230929    0.236899    0.248083    0.252735    0.258717
    0.268907    0.273928    0.285175    0.291205    0.300166    0.300608
    0.300925    0.301311    0.301896    0.303129    0.304093    0.304939
    0.305271    0.305289    0.306759    0.308418    0.312859    0.316212
    0.323708    0.333050    0.333647    0.335695    0.341091    0.343518
    0.350915    0.360829    0.368563    0.380443    0.388205    0.392175
    0.396083    0.403799    0.415922    0.417805    0.421134    0.436738
    0.446404    0.471545    0.486908    0.530361    0.576363    0.764937

Minimum search - taking simple RFO step
Searching for Lamda that Minimizes Along All modes
Value Taken    Lamda =  -0.00028438
Calculated Step too Large.  Step scaled by  0.729600
Step Taken.  Stepsize is  0.300000

                          Maximum     Tolerance    Cnvgd?
        Gradient          0.001338     0.000800      NO
        Displacement      0.081932     0.001400      NO
        Energy change    -0.000163     0.000228     YES


New Cartesian Coordinates Obtained by Inverse Iteration

Displacement from previous Coordinates is:  1.557416
-----------------------------------------------------------------
            Standard Nuclear Orientation (Angstroms)
   I     Atom           X                Y                Z
-----------------------------------------------------------------
   1      N       2.3318375818     0.3531476868     0.2931852330
   2      C       3.3668296699     0.1591797349     1.1586968738
   3      C       4.2317166129    -0.9027431497     0.9156850438
   4      C       4.0636943381    -1.7297889981    -0.1800887206
   5      C       3.0096491228    -1.4866728947    -1.0592654118
   6      C       2.1655839094    -0.4411488722    -0.7950379128
   7      C       3.5737144459     1.0720712717     2.3270323433
   8      C       1.3940011528     1.5021414072     0.4642832717
   9      N      -2.0849327173     0.2282969154     0.9659506257
  10      C      -2.7118721399     0.5481610710    -0.2007686643
  11      N      -2.0645887160     1.1682209995    -1.1496699890
  12      C      -0.7709691897     1.5042752881    -0.9855065871
  13      C      -0.0534046769     1.1558237958     0.2096428043
  14      C      -0.7825479937     0.5218244939     1.1699043807
  15      C      -4.1306417956     0.1380532299    -0.3867435831
  16      C      -4.2351045250    -1.1625900199    -1.2157709740
  17      C      -3.5733085875    -2.3657642726    -0.5526619715
  18      N      -0.2197464200     2.1541942215    -2.0067519788
  19      H       5.0503710660    -1.0645348804     1.6047789241
  20      H       4.7486509930    -2.5510543692    -0.3550247915
  21      H       2.8466430551    -2.0961464983    -1.9381854130
  22      H       1.3351754689    -0.2010367350    -1.4444580549
  23      H       4.4714718010     0.7716684587     2.8635954730
  24      H       2.7417153606     1.0279842274     3.0361117076
  25      H       3.7097797039     2.1095453311     2.0106628068
  26      H       1.7440792007     2.3074272271    -0.1833437170
  27      H       1.4970131213     1.8702512094     1.4805799734
  28      H      -2.6125300718    -0.2545295875     1.6836228369
  29      H      -0.3683664809     0.2244964704     2.1265498149
  30      H      -4.6109859098    -0.0001325860     0.5868142130
  31      H      -4.6417743878     0.9507000349    -0.9050900025
  32      H      -3.8101828218    -0.9850479663    -2.2074615323
  33      H      -5.2995173713    -1.3547426883    -1.3624864911
  34      H      -2.4867139854    -2.2471329313    -0.4651670740
  35      H      -3.7458087598    -3.2680045640    -1.1405485782
  36      H      -3.9827987166    -2.5491211281     0.4460871929
  37      H       0.7079403551     2.5439039032    -1.9993940468
```

```
  38      H      -0.8040716926     2.3488251634    -2.8097580249
 ----------------------------------------------------------------
 Nuclear Repulsion Energy =        1309.76653057 hartrees
 There are       65 alpha and       65 beta electrons

--------------------------------------------------
-  Entering fldman on Sun Oct 13 15:05:01 2024  -
--------------------------------------------------

 Applying Cartesian multipole field
    Component          Value
    ---------          -----
     (2,0,0)        1.00000E-11
     (0,2,0)        2.00000E-11
     (0,0,2)       -3.00000E-11
 Nucleus-field energy     =     0.0000000362 hartrees

--------------------------------------------------
-  Entering gesman on Sun Oct 13 15:05:01 2024  -
--------------------------------------------------

 Requested basis set is 6-311+G(d,p)
 There are 188 shells and 516 basis functions
 A cutoff of  1.0D-12 yielded  12788 shell pairs
 There are    101685 function pairs (    108387 Cartesian)
 Smallest overlap matrix eigenvalue = 1.84E-06
 Linear dependence detected in AO basis
 Tighter screening thresholds may be required for diffuse basis sets
 Use S2THRESH > 12 and THRESH = 14 in case of SCF convergence issues
 Number of orthogonalized atomic orbitals = 510
 Maximum deviation from orthogonality = 1.660E-11
 Guess MOs from SCF MO coefficient file
 Reading MOs from coefficient file
 Reading MOs from coefficient file

--------------------------------------------------
-  Entering scfman on Sun Oct 13 15:05:01 2024  -
--------------------------------------------------

 Long-range K will be added via erf
 Coulomb attenuation parameter = 0.2 bohr**(-1)
 A restricted hybrid HF-DFT SCF calculation will be
 performed using Pulay DIIS + Geometric Direct Minimization
 Exchange:     0.2220 Hartree-Fock + 1.0000 wB97X-D + LR-HF
 Correlation:  1.0000 wB97X-D
 Using Euler-Maclaurin-Lebedev (75,302) quadrature formula
 Dispersion:   Grimme D
 SCF converges when RMS gradient is below 1.0E-07
 Geometry optimization detected.  Setting ReadMinima to 0
 Setting SaveMinima to 0
 ---------------------------------------
  Cycle       Energy         DIIS Error
 ---------------------------------------
    1    -763.9085220325      1.22E-03
    2    -763.9652302457      1.72E-04
    3    -763.9670138413      1.17E-04
    4    -763.9674275227      4.63E-05
    5    -763.9675071038      1.92E-05
    6    -763.9675214049      4.59E-06
    7    -763.9675223185      1.52E-06
    8    -763.9675224388      6.85E-07
    9    -763.9675224604      1.73E-07
   10    -763.9675224621      7.10E-08 Convergence criterion met
 ---------------------------------------
 SCF time:  CPU 341.20 s  wall 621.99 s
 SCF   energy in the final basis set = -763.96752246
 Total energy in the final basis set = -763.96752246

--------------------------------------------------
-  Entering anlman on Sun Oct 13 15:15:23 2024  -
```

```
--------------------------------------------------------


 --------------------------------------------------------------

                    Orbital Energies (a.u.)
 --------------------------------------------------------------

 Alpha MOs
 -- Occupied --
-14.8151 -14.7902 -14.7390 -14.7076 -10.6780 -10.6622 -10.6486 -10.6387
-10.6351 -10.6253 -10.6055 -10.5825 -10.5727 -10.5693 -10.5458 -10.5265
-10.4969 -10.4750  -1.4157  -1.4028  -1.3485  -1.2762  -1.2301  -1.1997
 -1.1861  -1.1425  -1.1012  -1.0815  -1.0574  -1.0544  -1.0286  -1.0084
 -0.9891  -0.9555  -0.9453  -0.9180  -0.9095  -0.8933  -0.8829  -0.8646
 -0.8510  -0.8429  -0.8388  -0.8192  -0.8104  -0.8000  -0.7875  -0.7808
 -0.7723  -0.7580  -0.7485  -0.7474  -0.7444  -0.7274  -0.7236  -0.7149
 -0.7026  -0.6713  -0.6566  -0.6464  -0.6376  -0.6304  -0.6256  -0.6209
 -0.6101
 -- Virtual --
 -0.2697  -0.2595  -0.2448  -0.2254  -0.1746  -0.1635  -0.1497  -0.1443
 -0.1321  -0.1250  -0.1204  -0.1104  -0.1063  -0.1045  -0.0989  -0.0908
 -0.0891  -0.0851  -0.0836  -0.0770  -0.0745  -0.0713  -0.0691  -0.0671
 -0.0650  -0.0623  -0.0559  -0.0519  -0.0477  -0.0422  -0.0400  -0.0366
 -0.0336  -0.0322  -0.0294  -0.0244  -0.0207  -0.0154  -0.0101  -0.0081
 -0.0063  -0.0029  -0.0014   0.0009   0.0075   0.0092   0.0105   0.0162
  0.0178   0.0219   0.0257   0.0302   0.0324   0.0342   0.0347   0.0403
  0.0438   0.0447   0.0492   0.0520   0.0533   0.0607   0.0608   0.0642
  0.0682   0.0709   0.0777   0.0786   0.0816   0.0848   0.0875   0.0912
  0.0966   0.0982   0.0998   0.1030   0.1081   0.1108   0.1121   0.1172
  0.1187   0.1228   0.1249   0.1273   0.1322   0.1368   0.1408   0.1435
  0.1475   0.1539   0.1598   0.1635   0.1683   0.1705   0.1724   0.1787
  0.1830   0.1868   0.1875   0.1943   0.1972   0.2081   0.2175   0.2253
  0.2323   0.2420   0.2454   0.2685   0.2796   0.2878   0.2990   0.3135
  0.3206   0.3247   0.3360   0.3396   0.3502   0.3522   0.3572   0.3637
  0.3777   0.3829   0.3872   0.3942   0.3960   0.4062   0.4183   0.4219
  0.4314   0.4374   0.4404   0.4527   0.4575   0.4597   0.4703   0.4747
  0.4842   0.4892   0.5010   0.5030   0.5099   0.5141   0.5175   0.5200
  0.5242   0.5305   0.5363   0.5396   0.5450   0.5484   0.5524   0.5548
  0.5658   0.5681   0.5730   0.5785   0.5869   0.5889   0.5983   0.6033
  0.6091   0.6241   0.6291   0.6356   0.6458   0.6505   0.6564   0.6591
  0.6646   0.6749   0.6759   0.6804   0.6906   0.6963   0.7022   0.7105
  0.7146   0.7270   0.7397   0.7473   0.7520   0.7604   0.7737   0.7801
  0.7867   0.7986   0.8096   0.8193   0.8281   0.8452   0.8470   0.8540
  0.8625   0.8763   0.8878   0.8952   0.9155   0.9206   0.9260   0.9370
  0.9498   0.9616   0.9650   0.9900   0.9948   0.9997   1.0236   1.0356
  1.0498   1.0560   1.0721   1.0860   1.1223   1.1476   1.1703   1.1796
  1.1871   1.2058   1.2191   1.2414   1.2597   1.2773   1.2889   1.3048
  1.3134   1.3165   1.3301   1.3326   1.3461   1.3597   1.3666   1.3764
  1.3890   1.3902   1.4026   1.4061   1.4207   1.4216   1.4271   1.4337
  1.4365   1.4489   1.4599   1.4683   1.4696   1.4728   1.4851   1.4891
  1.4966   1.5031   1.5187   1.5205   1.5228   1.5378   1.5414   1.5522
  1.5647   1.5679   1.5732   1.5836   1.5862   1.5982   1.6127   1.6159
  1.6296   1.6318   1.6384   1.6540   1.6652   1.6736   1.6738   1.6817
  1.6936   1.7043   1.7235   1.7290   1.7390   1.7484   1.7564   1.7645
  1.7874   1.7952   1.8035   1.8245   1.8363   1.8447   1.8656   1.8752
  1.8898   1.9110   1.9120   1.9348   1.9438   1.9708   1.9906   1.9912
  2.0012   2.0055   2.0312   2.0492   2.0543   2.0642   2.0766   2.0955
  2.1152   2.1178   2.1282   2.1372   2.1727   2.1850   2.2208   2.2263
  2.2286   2.2453   2.2489   2.2567   2.2652   2.2792   2.2813   2.2898
  2.2931   2.3121   2.3380   2.3599   2.3616   2.3661   2.3866   2.3981
  2.4034   2.4153   2.4335   2.4403   2.4425   2.4590   2.4598   2.4856
  2.4951   2.5153   2.5249   2.5285   2.5407   2.5489   2.5501   2.5603
  2.5646   2.5754   2.5844   2.5900   2.5942   2.6081   2.6108   2.6114
  2.6329   2.6434   2.6520   2.6605   2.6730   2.6757   2.6854   2.7016
  2.7096   2.7311   2.7368   2.7442   2.7578   2.7644   2.7753   2.7881
  2.7919   2.8071   2.8130   2.8285   2.8458   2.8819   2.8874   2.8886
  2.8929   2.9249   2.9628   2.9987   3.0595   3.0744   3.1407   3.1428
  3.1678   3.2437   3.2452   3.2732   3.2997   3.3330   3.3680   3.4145
  3.4209   3.4800   3.5232   3.5506   3.5600   3.5960   3.6146   3.6489
```

```
  3.6641   3.6858   3.7031   3.7307   3.7342   3.7434   3.7553   3.7687
  3.7799   3.8400   3.8722   3.9104   3.9390   3.9843   4.0273   4.0819
  4.1121   4.2045   4.2975   4.3139   4.5952   4.6661   4.7052   4.7194
  4.9254   5.0432   5.2002  23.5215  23.5734  23.7164  23.7302  23.7405
 23.7768  23.8003  23.8252  23.8568  23.8844  23.9116  23.9693  23.9808
 24.0366  35.4213  35.4560  35.5217  35.6219
--------------------------------------------------------------

         Ground-State Mulliken Net Atomic Charges

     Atom                 Charge (a.u.)
  ----------------------------------------
      1 N                    0.417076
      2 C                    0.094520
      3 C                    0.000489
      4 C                   -0.142164
      5 C                   -0.202239
      6 C                   -0.149316
      7 C                   -0.733390
      8 C                   -0.367927
      9 N                   -0.136479
     10 C                   -0.293358
     11 N                    0.013324
     12 C                    0.318083
     13 C                   -0.052106
     14 C                    0.103504
     15 C                   -0.344977
     16 C                   -0.262938
     17 C                   -0.530293
     18 N                   -0.474724
     19 H                    0.220375
     20 H                    0.234129
     21 H                    0.226401
     22 H                    0.211910
     23 H                    0.229135
     24 H                    0.213572
     25 H                    0.232922
     26 H                    0.226331
     27 H                    0.246385
     28 H                    0.380856
     29 H                    0.211349
     30 H                    0.210209
     31 H                    0.253229
     32 H                    0.204754
     33 H                    0.225299
     34 H                    0.128997
     35 H                    0.215973
     36 H                    0.178508
     37 H                    0.315909
     38 H                    0.376672
  ----------------------------------------
  Sum of atomic charges =     2.000000

-----------------------------------------------------------------
                    Cartesian Multipole Moments
-----------------------------------------------------------------
    Charge (ESU x 10^10)
                 9.6064
    Dipole Moment (Debye)
         X       5.7614      Y       2.3553      Z       1.7057
       Tot       6.4537
    Quadrupole Moments (Debye-Ang)
        XX      -7.0653     XY      -1.4697     YY     -76.9818
        XZ       8.8189     YZ      -1.0807     ZZ     -72.3868
    Traceless Quadrupole Moments (Debye-Ang)
       QXX     135.2379    QYY     -74.5114    QZZ     -60.7265
       QXY      -4.4091    QXZ      26.4568    QYZ      -3.2422
    Octopole Moments (Debye-Ang^2)
       XXX      40.2623    XXY     -82.5450    XYY      24.6790
       YYY     -21.9162    XXZ      54.2092    XYZ      15.0077
```

```
       YYZ      -27.9552    XZZ       11.6160    YZZ       14.9194
       ZZZ       19.9063
    Traceless Octopole Moments (Debye-Ang^2)
       XXX      -85.0815    YYY      477.1330    ZZZ     -116.8479
       XXY     -969.5495    XXZ      674.6568    XYY      140.5135
       XYZ      225.1152    XZZ      -55.4320    YYZ     -557.8089
       YZZ      492.4165
    Hexadecapole Moments (Debye-Ang^3)
      XXXX   -3571.6974   XXXY    -193.3611   XXYY    -820.3435
      XYYY     -27.8111   YYYY   -1020.3352   XXXZ     -93.9141
      XXYZ      62.5981   XYYZ    -129.9909   YYYZ     -65.2244
      XXZZ    -815.1604   XYZZ       8.7493   YYZZ    -248.5931
      XZZZ    -346.9955   YZZZ     -83.8875   ZZZZ    -752.0846
    Traceless Hexadecapole Moments (Debye-Ang^3)
      XXXX   11609.0858   XXXY  -10743.8837   XXXZ   15829.5405
      XXYY   -4025.9008   XXYZ    7857.1951   XXZZ   -7583.1850
      XYYY    6638.8599   XYYZ   -5085.5391   XYZZ    4105.0238
      XZZZ  -10744.0014   YYYY   -1111.5371   YYYZ   -2995.3767
      YYZZ    5137.4379   YZZZ   -4861.8184   ZZZZ    2445.7471
 -----------------------------------------------------------------

--------------------------------------------------
-  Entering drvman on Sun Oct 13 15:15:23 2024  -
--------------------------------------------------

 Calculating analytic gradient of the SCF energy
 Gradient of SCF Energy
           1           2           3           4           5           6
    1  -0.0001069  -0.0000897   0.0001904   0.0003802   0.0002244  -0.0007643
    2  -0.0000745   0.0002480  -0.0003972  -0.0002642  -0.0000525   0.0005414
    3   0.0008554   0.0000388  -0.0003547   0.0001968  -0.0000384   0.0000593
           7           8           9          10          11          12
    1   0.0001953   0.0000202   0.0001119   0.0005011  -0.0006041  -0.0003111
    2  -0.0002083  -0.0000119   0.0004521   0.0002031   0.0001618  -0.0009356
    3   0.0002467  -0.0006930  -0.0003150   0.0001717  -0.0002323   0.0018479
          13          14          15          16          17          18
    1  -0.0007069   0.0004863   0.0001517  -0.0004532   0.0001046   0.0002304
    2  -0.0004494   0.0000356   0.0000370   0.0000997  -0.0000817   0.0019719
    3  -0.0001550  -0.0000802  -0.0002789   0.0004243  -0.0001515  -0.0000292
          19          20          21          22          23          24
    1  -0.0000001  -0.0001128   0.0000102   0.0002706  -0.0000583  -0.0000007
    2  -0.0000061   0.0000102  -0.0000430   0.0001263  -0.0000494   0.0000250
    3  -0.0000756   0.0000422  -0.0000271  -0.0001005  -0.0000665   0.0002412
          25          26          27          28          29          30
    1   0.0001205  -0.0002592  -0.0000270  -0.0001026   0.0000298   0.0000244
    2  -0.0000186  -0.0002108   0.0003908  -0.0001832  -0.0001934  -0.0000755
    3  -0.0000513  -0.0001529  -0.0003694  -0.0001820   0.0000931   0.0000279
          31          32          33          34          35          36
    1   0.0000342   0.0000323   0.0000447   0.0000267   0.0000332   0.0000024
    2  -0.0000380  -0.0001029   0.0000152  -0.0000960  -0.0000021  -0.0000141
    3   0.0001241  -0.0001380  -0.0000982  -0.0000027   0.0000264   0.0000668
          37          38
    1   0.0002757   0.0000957
    2   0.0001549  -0.0009646
    3  -0.0002430  -0.0006277
 Max gradient component =       1.972E-03
 RMS gradient           =       3.770E-04
 Gradient time:  CPU 157.66 s  wall 286.75 s

--------------------------------------------------
-  Entering optman on Sun Oct 13 15:20:10 2024  -
--------------------------------------------------


 Geometry Optimization Parameters
   NAtoms,    NIC,     NZ,  NCons,   NDum,   NFix, NCnnct, MaxDiis
       38     272       0       0       0       0       0       0

 Cartesian Hessian Update
 Hessian updated using BFGS update
```

```
** GEOMETRY OPTIMIZATION IN DELOCALIZED INTERNAL COORDINATES **
   Searching for a Minimum

   Optimization Cycle:  12

                    Coordinates (Angstroms)
     ATOM                X               Y               Z
      1  N         2.3318375818    0.3531476868    0.2931852330
      2  C         3.3668296699    0.1591797349    1.1586968738
      3  C         4.2317166129   -0.9027431497    0.9156850438
      4  C         4.0636943381   -1.7297889981   -0.1800887206
      5  C         3.0096491228   -1.4866728947   -1.0592654118
      6  C         2.1655839094   -0.4411488722   -0.7950379128
      7  C         3.5737144459    1.0720712717    2.3270323433
      8  C         1.3940011528    1.5021414072    0.4642832717
      9  N        -2.0849327173    0.2282969154    0.9659506257
     10  C        -2.7118721399    0.5481610710   -0.2007686643
     11  N        -2.0645887160    1.1682209995   -1.1496699890
     12  C        -0.7709691897    1.5042752881   -0.9855065871
     13  C        -0.0534046769    1.1558237958    0.2096428043
     14  C        -0.7825479937    0.5218244939    1.1699043807
     15  C        -4.1306417956    0.1380532299   -0.3867435831
     16  C        -4.2351045250   -1.1625900199   -1.2157709740
     17  C        -3.5733085875   -2.3657642726   -0.5526619715
     18  N        -0.2197464200    2.1541942215   -2.0067519788
     19  H         5.0503710660   -1.0645348804    1.6047789241
     20  H         4.7486509930   -2.5510543692   -0.3550247915
     21  H         2.8466430551   -2.0961464983   -1.9381854130
     22  H         1.3351754689   -0.2010367350   -1.4444580549
     23  H         4.4714718010    0.7716684587    2.8635954730
     24  H         2.7417153606    1.0279842274    3.0361117076
     25  H         3.7097797039    2.1095453311    2.0106628068
     26  H         1.7440792007    2.3074272271   -0.1833437170
     27  H         1.4970131213    1.8702512094    1.4805799734
     28  H        -2.6125300718   -0.2545295875    1.6836228369
     29  H        -0.3683664809    0.2244964704    2.1265498149
     30  H        -4.6109859098   -0.0001325860    0.5868142130
     31  H        -4.6417743878    0.9507000349   -0.9050900025
     32  H        -3.8101828218   -0.9850479663   -2.2074615323
     33  H        -5.2995173713   -1.3547426883   -1.3624864911
     34  H        -2.4867139854   -2.2471329313   -0.4651670740
     35  H        -3.7458087598   -3.2680045640   -1.1405485782
     36  H        -3.9827987166   -2.5491211281    0.4460871929
     37  H         0.7079403551    2.5439039032   -1.9993940468
     38  H        -0.8040716926    2.3488251634   -2.8097580249
   Point Group: c1    Number of degrees of freedom:   108


   Energy is   -763.967522462

 Hessian updated using BFGS update
  internal optimization (0)

108 Hessian modes will be used to form the next step
  Hessian Eigenvalues:
     0.000099    0.001873    0.002599    0.003826    0.005796    0.010965
     0.015113    0.017765    0.019720    0.019850    0.021532    0.022040
     0.022672    0.023253    0.023711    0.024646    0.025526    0.025926
     0.026761    0.027423    0.028569    0.029704    0.032079    0.036537
     0.039136    0.042043    0.043172    0.043750    0.043994    0.046236
     0.046713    0.053447    0.055773    0.057807    0.065540    0.079879
     0.085663    0.092020    0.121581    0.122832    0.127182    0.130811
     0.132518    0.132917    0.139854    0.141802    0.143331    0.144081
     0.146655    0.147695    0.149635    0.152573    0.152986    0.153222
     0.159083    0.175059    0.196075    0.206736    0.210265    0.215985
     0.222299    0.231078    0.236916    0.248265    0.252276    0.263128
     0.268913    0.273971    0.284728    0.289625    0.300167    0.300692
     0.301112    0.301375    0.301900    0.303141    0.304091    0.304945
     0.305271    0.305399    0.306763    0.309116    0.312774    0.316439
```

```
     0.323579    0.333067    0.333548    0.335646    0.341048    0.343780
     0.351330    0.362346    0.368895    0.380761    0.388623    0.392493
     0.396130    0.404019    0.415452    0.418576    0.419345    0.441054
     0.446699    0.468145    0.487737    0.530693    0.600499    0.761390
**WARNING** Magnitude of eigenvalue   1 too small.  Replaced by     0.000100

 Minimum search - taking simple RFO step
 Searching for Lamda that Minimizes Along All modes
 Value Taken    Lamda =  -0.00067320
 Calculated Step too Large.  Step scaled by  0.365635
 Step Taken.  Stepsize is  0.300000

                             Maximum     Tolerance    Cnvgd?
         Gradient           0.001356      0.000800      NO
         Displacement       0.087315      0.001400      NO
         Energy change     -0.000195      0.000228     YES


 New Cartesian Coordinates Obtained by Inverse Iteration

 Displacement from previous Coordinates is:  1.505623
 ----------------------------------------------------------------
             Standard Nuclear Orientation (Angstroms)
    I     Atom           X                 Y                 Z
 ----------------------------------------------------------------
    1      N       2.3296833793     0.3187202830     0.3419807326
    2      C       3.4286790330     0.2072264642     1.1413859807
    3      C       4.3121227406    -0.8382220346     0.8962517134
    4      C       4.1001290001    -1.7297329265    -0.1396450978
    5      C       2.9822383713    -1.5683596156    -0.9563270423
    6      C       2.1204158497    -0.5374446768    -0.6900563393
    7      C       3.6822546181     1.1935282678     2.2382362758
    8      C       1.3756459718     1.4539304176     0.5235415504
    9      N      -2.1085964379     0.1791897769     0.9827948668
   10      C      -2.7311638773     0.5313561724    -0.1772498487
   11      N      -2.0741543012     1.1586287689    -1.1143258987
   12      C      -0.7754242818     1.4717786195    -0.9457573121
   13      C      -0.0675774091     1.1065821804     0.2510205495
   14      C      -0.8052343236     0.4613632702     1.1974830164
   15      C      -4.1560842466     0.1478132607    -0.3726918935
   16      C      -4.2786564930    -1.1004186722    -1.2761600099
   17      C      -3.6250275204    -2.3447006438    -0.6851908846
   18      N      -0.2097252330     2.1127758275    -1.9640131689
   19      H       5.1803953063    -0.9348821098     1.5349056148
   20      H       4.7997893476    -2.5379706305    -0.3166935780
   21      H       2.7824566691    -2.2299024822    -1.7887012561
   22      H       1.2397329879    -0.3612213607    -1.2910397196
   23      H       4.6364542621     0.9691016292     2.7106925047
   24      H       2.9153305714     1.1474481787     3.0173598110
   25      H       3.7336683536     2.2172556696     1.8584781646
   26      H       1.7229649585     2.2761118786    -0.1045551032
   27      H       1.4651313689     1.8020290660     1.5485223375
   28      H      -2.6418820618    -0.3123994516     1.6901308475
   29      H      -0.3968708172     0.1462615379     2.1510454150
   30      H      -4.6303610479    -0.0416473457     0.5950617556
   31      H      -4.6624918695     0.9946843350    -0.8390282450
   32      H      -3.8563288990    -0.8691128635    -2.2577113837
   33      H      -5.3456090252    -1.2731576569    -1.4282015349
   34      H      -2.5374504037    -2.2380292228    -0.5925970251
   35      H      -3.8041715122    -3.2101944929    -1.3240507332
   36      H      -4.0341479015    -2.5821111253     0.3020368297
   37      H       0.7251049300     2.4847634998    -1.9535744554
   38      H      -0.7912400574     2.3289582076    -2.7633574362
 ----------------------------------------------------------------
 Nuclear Repulsion Energy =        1308.40112744 hartrees
 There are       65 alpha and       65 beta electrons

 ------------------------------------------------
 -  Entering fldman on Sun Oct 13 15:20:10 2024  -
 ------------------------------------------------
```

```
 Applying Cartesian multipole field
    Component          Value
    ---------          -----
     (2,0,0)        1.00000E-11
     (0,2,0)        2.00000E-11
     (0,0,2)       -3.00000E-11
 Nucleus-field energy     =     0.0000000376 hartrees

--------------------------------------------------
-  Entering gesman on Sun Oct 13 15:20:10 2024  -
--------------------------------------------------

 Requested basis set is 6-311+G(d,p)
 There are 188 shells and 516 basis functions
 A cutoff of  1.0D-12 yielded  12756 shell pairs
 There are    101388 function pairs (    108055 Cartesian)
 Smallest overlap matrix eigenvalue = 1.62E-06
 Linear dependence detected in AO basis
 Tighter screening thresholds may be required for diffuse basis sets
 Use S2THRESH > 12 and THRESH = 14 in case of SCF convergence issues
 Number of orthogonalized atomic orbitals = 510
 Maximum deviation from orthogonality = 1.589E-11
 Guess MOs from SCF MO coefficient file
 Reading MOs from coefficient file
 Reading MOs from coefficient file

--------------------------------------------------
-  Entering scfman on Sun Oct 13 15:20:10 2024  -
--------------------------------------------------

 Long-range K will be added via erf
 Coulomb attenuation parameter = 0.2 bohr**(-1)
 A restricted hybrid HF-DFT SCF calculation will be
 performed using Pulay DIIS + Geometric Direct Minimization
 Exchange:     0.2220 Hartree-Fock + 1.0000 wB97X-D + LR-HF
 Correlation:  1.0000 wB97X-D
 Using Euler-Maclaurin-Lebedev (75,302) quadrature formula
 Dispersion:   Grimme D
 SCF converges when RMS gradient is below 1.0E-07
 Geometry optimization detected.  Setting ReadMinima to 0
 Setting SaveMinima to 0
 ---------------------------------------
  Cycle       Energy         DIIS Error
 ---------------------------------------
    1    -763.9110794661      1.17E-03
    2    -763.9656561959      1.65E-04
    3    -763.9672742036      1.17E-04
    4    -763.9676792031      4.57E-05
    5    -763.9677572883      1.77E-05
    6    -763.9677698648      4.27E-06
    7    -763.9677706547      1.47E-06
    8    -763.9677707635      6.60E-07
    9    -763.9677707830      1.65E-07
   10    -763.9677707844      6.68E-08 Convergence criterion met
 ---------------------------------------
 SCF time:  CPU 335.61 s  wall 618.97 s
 SCF   energy in the final basis set = -763.96777078
 Total energy in the final basis set = -763.96777078

--------------------------------------------------
-  Entering anlman on Sun Oct 13 15:30:29 2024  -
--------------------------------------------------


 --------------------------------------------------------------

                   Orbital Energies (a.u.)
 --------------------------------------------------------------
```

```
 Alpha MOs
 -- Occupied --
-14.8151 -14.7903 -14.7390 -14.7075 -10.6781 -10.6624 -10.6487 -10.6380
-10.6353 -10.6251 -10.6058 -10.5821 -10.5723 -10.5688 -10.5449 -10.5266
-10.4964 -10.4744  -1.4157  -1.4025  -1.3486  -1.2763  -1.2299  -1.1997
 -1.1856  -1.1425  -1.1011  -1.0809  -1.0574  -1.0542  -1.0279  -1.0083
 -0.9888  -0.9557  -0.9449  -0.9180  -0.9095  -0.8931  -0.8821  -0.8643
 -0.8517  -0.8427  -0.8385  -0.8191  -0.8097  -0.8006  -0.7869  -0.7803
 -0.7714  -0.7582  -0.7485  -0.7472  -0.7441  -0.7271  -0.7231  -0.7145
 -0.7021  -0.6710  -0.6560  -0.6463  -0.6369  -0.6301  -0.6256  -0.6205
 -0.6102
 -- Virtual --
 -0.2687  -0.2598  -0.2450  -0.2252  -0.1749  -0.1633  -0.1507  -0.1443
 -0.1325  -0.1235  -0.1203  -0.1100  -0.1060  -0.1038  -0.0986  -0.0903
 -0.0893  -0.0858  -0.0837  -0.0766  -0.0745  -0.0709  -0.0687  -0.0668
 -0.0641  -0.0615  -0.0558  -0.0528  -0.0464  -0.0427  -0.0403  -0.0367
 -0.0322  -0.0319  -0.0286  -0.0237  -0.0199  -0.0157  -0.0101  -0.0083
 -0.0072  -0.0030  -0.0014   0.0019   0.0067   0.0088   0.0105   0.0163
  0.0173   0.0218   0.0268   0.0309   0.0312   0.0354   0.0364   0.0404
  0.0435   0.0443   0.0494   0.0530   0.0542   0.0608   0.0622   0.0649
  0.0679   0.0718   0.0772   0.0787   0.0817   0.0836   0.0893   0.0933
  0.0956   0.0984   0.1000   0.1028   0.1071   0.1095   0.1119   0.1183
  0.1191   0.1233   0.1241   0.1286   0.1322   0.1374   0.1431   0.1449
  0.1490   0.1562   0.1598   0.1635   0.1692   0.1715   0.1723   0.1795
  0.1826   0.1863   0.1878   0.1934   0.1988   0.2121   0.2168   0.2267
  0.2335   0.2422   0.2446   0.2715   0.2818   0.2896   0.2990   0.3132
  0.3185   0.3240   0.3378   0.3418   0.3492   0.3517   0.3555   0.3643
  0.3783   0.3828   0.3885   0.3931   0.3968   0.4069   0.4187   0.4208
  0.4311   0.4334   0.4387   0.4519   0.4587   0.4611   0.4729   0.4774
  0.4818   0.4894   0.5000   0.5029   0.5078   0.5130   0.5180   0.5214
  0.5231   0.5324   0.5364   0.5391   0.5449   0.5470   0.5506   0.5564
  0.5657   0.5694   0.5725   0.5784   0.5865   0.5897   0.5982   0.6018
  0.6097   0.6227   0.6307   0.6353   0.6467   0.6515   0.6566   0.6587
  0.6681   0.6727   0.6776   0.6817   0.6913   0.6978   0.7043   0.7101
  0.7141   0.7257   0.7395   0.7486   0.7506   0.7654   0.7729   0.7815
  0.7860   0.7974   0.8036   0.8189   0.8267   0.8432   0.8491   0.8542
  0.8656   0.8774   0.8857   0.8982   0.9074   0.9182   0.9284   0.9414
  0.9499   0.9601   0.9665   0.9914   0.9986   1.0036   1.0222   1.0346
  1.0508   1.0580   1.0757   1.0897   1.1255   1.1451   1.1652   1.1806
  1.1872   1.2089   1.2196   1.2407   1.2678   1.2705   1.2916   1.3037
  1.3139   1.3177   1.3251   1.3318   1.3454   1.3634   1.3658   1.3770
  1.3873   1.3904   1.4002   1.4081   1.4193   1.4220   1.4268   1.4346
  1.4370   1.4489   1.4591   1.4687   1.4698   1.4725   1.4864   1.4894
  1.4954   1.5048   1.5155   1.5188   1.5224   1.5385   1.5441   1.5532
  1.5659   1.5694   1.5728   1.5845   1.5873   1.5976   1.6099   1.6147
  1.6309   1.6340   1.6406   1.6550   1.6644   1.6723   1.6766   1.6826
  1.6904   1.7080   1.7269   1.7287   1.7408   1.7485   1.7606   1.7645
  1.7861   1.7951   1.8031   1.8259   1.8351   1.8481   1.8641   1.8751
  1.8911   1.9101   1.9113   1.9347   1.9407   1.9711   1.9878   1.9923
  2.0044   2.0108   2.0314   2.0469   2.0561   2.0650   2.0804   2.0968
  2.1119   2.1177   2.1279   2.1372   2.1734   2.1861   2.2222   2.2270
  2.2301   2.2460   2.2505   2.2577   2.2625   2.2805   2.2815   2.2886
  2.2920   2.3140   2.3397   2.3599   2.3630   2.3679   2.3874   2.3989
  2.4037   2.4175   2.4335   2.4407   2.4472   2.4568   2.4623   2.4861
  2.4944   2.5157   2.5231   2.5267   2.5406   2.5473   2.5494   2.5611
  2.5645   2.5766   2.5864   2.5907   2.5935   2.6057   2.6137   2.6141
  2.6332   2.6424   2.6510   2.6601   2.6712   2.6774   2.6838   2.6980
  2.7061   2.7285   2.7353   2.7444   2.7552   2.7669   2.7776   2.7903
  2.7906   2.8051   2.8164   2.8289   2.8469   2.8815   2.8846   2.8938
  2.8966   2.9206   2.9617   3.0018   3.0611   3.0763   3.1418   3.1465
  3.1694   3.2443   3.2469   3.2721   3.3016   3.3299   3.3668   3.4139
  3.4197   3.4808   3.5255   3.5493   3.5593   3.5951   3.6086   3.6493
  3.6654   3.6860   3.7053   3.7302   3.7349   3.7440   3.7547   3.7666
  3.7836   3.8408   3.8725   3.9106   3.9387   3.9850   4.0279   4.0827
  4.1120   4.2042   4.2985   4.3145   4.5954   4.6661   4.7044   4.7186
  4.9253   5.0434   5.2001  23.5215  23.5746  23.7149  23.7306  23.7431
 23.7761  23.7998  23.8270  23.8577  23.8851  23.9130  23.9700  23.9790
 24.0367  35.4215  35.4554  35.5218  35.6223
 --------------------------------------------------------------
```

```
       Ground-State Mulliken Net Atomic Charges

   Atom                 Charge (a.u.)
 ----------------------------------------
    1 N                     0.402113
    2 C                     0.110700
    3 C                    -0.004023
    4 C                    -0.146684
    5 C                    -0.198413
    6 C                    -0.136107
    7 C                    -0.725181
    8 C                    -0.378604
    9 N                    -0.134273
   10 C                    -0.296825
   11 N                     0.015553
   12 C                     0.312297
   13 C                    -0.072686
   14 C                     0.107087
   15 C                    -0.346090
   16 C                    -0.264700
   17 C                    -0.527475
   18 N                    -0.468709
   19 H                     0.220558
   20 H                     0.233931
   21 H                     0.226200
   22 H                     0.218803
   23 H                     0.229033
   24 H                     0.220591
   25 H                     0.227567
   26 H                     0.228433
   27 H                     0.246419
   28 H                     0.381810
   29 H                     0.212088
   30 H                     0.207415
   31 H                     0.254687
   32 H                     0.205012
   33 H                     0.224970
   34 H                     0.128393
   35 H                     0.215515
   36 H                     0.179130
   37 H                     0.314300
   38 H                     0.377169
 ----------------------------------------
 Sum of atomic charges =     2.000000

-----------------------------------------------------------------
                   Cartesian Multipole Moments
-----------------------------------------------------------------
   Charge (ESU x 10^10)
                 9.6064
   Dipole Moment (Debye)
        X       5.7607      Y       2.2219      Z       1.9222
      Tot       6.4666
   Quadrupole Moments (Debye-Ang)
       XX      -4.6772     XY      -0.7543     YY     -77.1052
       XZ       9.4698     YZ      -0.4354     ZZ     -73.7474
   Traceless Quadrupole Moments (Debye-Ang)
      QXX     141.4981    QYY     -75.7857    QZZ     -65.7124
      QXY      -2.2629    QXZ      28.4094    QYZ      -1.3062
   Octopole Moments (Debye-Ang^2)
      XXX      50.8688    XXY     -77.8436    XYY      25.9023
      YYY     -20.6302    XXZ      55.3866    XYZ      16.7276
      YYZ     -28.1206    XZZ       6.8292    YZZ      15.1843
      ZZZ      17.3988
   Traceless Octopole Moments (Debye-Ang^2)
      XXX      10.6287    YYY     440.1521    ZZZ    -141.0016
      XXY    -917.7854    XXZ     696.8044    XYY     137.7337
      XYZ     250.9139    XZZ    -148.3623    YYZ    -555.8028
      YZZ     477.6333
   Hexadecapole Moments (Debye-Ang^3)
```

```
      XXXX   -3565.4209   XXXY    -186.2021   XXYY    -835.4259
      XYYY     -39.7859   YYYY   -1013.1037   XXXZ     -94.2950
      XXYZ      66.3278   XYYZ    -136.9033   YYYZ     -75.0594
      XXZZ    -836.5500   XYZZ      16.6874   YYZZ    -244.7216
      XZZZ    -375.4402   YZZZ     -96.3863   ZZZZ    -737.7774
    Traceless Hexadecapole Moments (Debye-Ang^3)
      XXXX   14649.2472   XXXY  -10132.6920   XXXZ   17397.7557
      XXYY   -5209.0916   XXYZ    8535.3888   XXZZ   -9440.1556
      XYYY    5241.0046   XYYZ   -5275.2671   XYZZ    4891.6874
      XZZZ  -12122.4886   YYYY    -330.5524   YYYZ   -3168.3109
      YYZZ    5539.6440   YZZZ   -5367.0779   ZZZZ    3900.5116
 -----------------------------------------------------------------

--------------------------------------------------
-  Entering drvman on Sun Oct 13 15:30:29 2024  -
--------------------------------------------------

 Calculating analytic gradient of the SCF energy
 Gradient of SCF Energy
           1           2           3           4           5           6
    1  -0.0001229  -0.0001905   0.0001748   0.0005272   0.0001208  -0.0007146
    2  -0.0004207   0.0002517  -0.0004999  -0.0000812  -0.0000961   0.0006250
    3   0.0012842   0.0003154  -0.0005599   0.0003373  -0.0002670  -0.0001910
           7           8           9          10          11          12
    1   0.0004975   0.0000871   0.0001266   0.0002248  -0.0005898  -0.0003446
    2  -0.0003146   0.0000330   0.0003520   0.0002372   0.0000507  -0.0004452
    3   0.0002144  -0.0007603  -0.0002545  -0.0001175   0.0000392   0.0015985
          13          14          15          16          17          18
    1  -0.0007425   0.0005654   0.0000407  -0.0003744   0.0001599   0.0003748
    2  -0.0003747  -0.0000647   0.0000271  -0.0000703   0.0000406   0.0012462
    3   0.0001767  -0.0000555  -0.0001111   0.0002155  -0.0000239  -0.0003501
          19          20          21          22          23          24
    1  -0.0000619  -0.0001455   0.0000002   0.0003261  -0.0000733   0.0000518
    2   0.0000030   0.0000225  -0.0000035   0.0001452  -0.0000332   0.0001069
    3  -0.0000499   0.0000876   0.0000162  -0.0000513  -0.0000395   0.0002648
          25          26          27          28          29          30
    1   0.0001577  -0.0002729  -0.0001745  -0.0000412   0.0000722   0.0000540
    2  -0.0000243  -0.0002665   0.0005187  -0.0001908  -0.0002345  -0.0000917
    3  -0.0001020  -0.0002024  -0.0005888  -0.0002475   0.0000880   0.0000868
          31          32          33          34          35          36
    1   0.0000029   0.0000068   0.0000495   0.0000505   0.0000295  -0.0000128
    2   0.0000031  -0.0000748   0.0001136  -0.0000972   0.0000322   0.0000007
    3   0.0001198  -0.0001872  -0.0001051  -0.0000556   0.0000283   0.0000556
          37          38
    1   0.0000973   0.0000633
    2   0.0003244  -0.0007502
    3  -0.0002153  -0.0003927
 Max gradient component =       1.599E-03
 RMS gradient           =       3.503E-04
 Gradient time:  CPU 155.22 s  wall 283.31 s

--------------------------------------------------
-  Entering optman on Sun Oct 13 15:35:13 2024  -
--------------------------------------------------

 Geometry Optimization Parameters
   NAtoms,    NIC,     NZ,  NCons,   NDum,   NFix, NCnnct, MaxDiis
       38     272       0       0       0       0       0       0

 Cartesian Hessian Update
 Hessian updated using BFGS update


** GEOMETRY OPTIMIZATION IN DELOCALIZED INTERNAL COORDINATES **
   Searching for a Minimum

   Optimization Cycle:  13

                        Coordinates (Angstroms)
     ATOM                X               Y               Z
```

| | | | | |
|---|---|---|---|---|
| 1 | N | 2.3296833793 | 0.3187202830 | 0.3419807326 |
| 2 | C | 3.4286790330 | 0.2072264642 | 1.1413859807 |
| 3 | C | 4.3121227406 | -0.8382220346 | 0.8962517134 |
| 4 | C | 4.1001290001 | -1.7297329265 | -0.1396450978 |
| 5 | C | 2.9822383713 | -1.5683596156 | -0.9563270423 |
| 6 | C | 2.1204158497 | -0.5374446768 | -0.6900563393 |
| 7 | C | 3.6822546181 | 1.1935282678 | 2.2382362758 |
| 8 | C | 1.3756459718 | 1.4539304176 | 0.5235415504 |
| 9 | N | -2.1085964379 | 0.1791897769 | 0.9827948668 |
| 10 | C | -2.7311638773 | 0.5313561724 | -0.1772498487 |
| 11 | N | -2.0741543012 | 1.1586287689 | -1.1143258987 |
| 12 | C | -0.7754242818 | 1.4717786195 | -0.9457573121 |
| 13 | C | -0.0675774091 | 1.1065821804 | 0.2510205495 |
| 14 | C | -0.8052343236 | 0.4613632702 | 1.1974830164 |
| 15 | C | -4.1560842466 | 0.1478132607 | -0.3726918935 |
| 16 | C | -4.2786564930 | -1.1004186722 | -1.2761600099 |
| 17 | C | -3.6250275204 | -2.3447006438 | -0.6851908846 |
| 18 | N | -0.2097252330 | 2.1127758275 | -1.9640131689 |
| 19 | H | 5.1803953063 | -0.9348821098 | 1.5349056148 |
| 20 | H | 4.7997893476 | -2.5379706305 | -0.3166935780 |
| 21 | H | 2.7824566691 | -2.2299024822 | -1.7887012561 |
| 22 | H | 1.2397329879 | -0.3612213607 | -1.2910397196 |
| 23 | H | 4.6364542621 | 0.9691016292 | 2.7106925047 |
| 24 | H | 2.9153305714 | 1.1474481787 | 3.0173598110 |
| 25 | H | 3.7336683536 | 2.2172556696 | 1.8584781646 |
| 26 | H | 1.7229649585 | 2.2761118786 | -0.1045551032 |
| 27 | H | 1.4651313689 | 1.8020290660 | 1.5485223375 |
| 28 | H | -2.6418820618 | -0.3123994516 | 1.6901308475 |
| 29 | H | -0.3968708172 | 0.1462615379 | 2.1510454150 |
| 30 | H | -4.6303610479 | -0.0416473457 | 0.5950617556 |
| 31 | H | -4.6624918695 | 0.9946843350 | -0.8390282450 |
| 32 | H | -3.8563288990 | -0.8691128635 | -2.2577113837 |

***Abridged***Abridged***Abridged***

| | | | | | | | |
|---|---|---|---|---|---|---|---|
| 0.0437 | 0.0477 | 0.0547 | 0.0562 | 0.0581 | 0.0611 | 0.0613 | 0.0643 |
| 0.0729 | 0.0763 | 0.0775 | 0.0802 | 0.0823 | 0.0855 | 0.0860 | 0.0916 |
| 0.0962 | 0.1017 | 0.1043 | 0.1058 | 0.1081 | 0.1129 | 0.1152 | 0.1188 |
| 0.1203 | 0.1237 | 0.1270 | 0.1315 | 0.1364 | 0.1412 | 0.1437 | 0.1474 |
| 0.1534 | 0.1592 | 0.1623 | 0.1680 | 0.1697 | 0.1728 | 0.1735 | 0.1813 |
| 0.1851 | 0.1906 | 0.1929 | 0.1956 | 0.2075 | 0.2108 | 0.2192 | 0.2232 |
| 0.2350 | 0.2384 | 0.2460 | 0.2557 | 0.2596 | 0.2695 | 0.2747 | 0.2925 |
| 0.2983 | 0.3018 | 0.3137 | 0.3185 | 0.3311 | 0.3383 | 0.3419 | 0.3474 |
| 0.3525 | 0.3541 | 0.3601 | 0.3679 | 0.3739 | 0.3804 | 0.3850 | 0.3908 |
| 0.3928 | 0.4013 | 0.4050 | 0.4082 | 0.4111 | 0.4208 | 0.4247 | 0.4301 |
| 0.4342 | 0.4358 | 0.4411 | 0.4462 | 0.4483 | 0.4519 | 0.4609 | 0.4686 |
| 0.4707 | 0.4735 | 0.4770 | 0.4780 | 0.4848 | 0.4868 | 0.4926 | 0.4950 |
| 0.4984 | 0.4991 | 0.5070 | 0.5118 | 0.5138 | 0.5228 | 0.5254 | 0.5280 |
| 0.5299 | 0.5337 | 0.5384 | 0.5417 | 0.5445 | 0.5489 | 0.5503 | 0.5560 |
| 0.5596 | 0.5651 | 0.5699 | 0.5734 | 0.5808 | 0.5839 | 0.5851 | 0.5905 |
| 0.5985 | 0.6043 | 0.6095 | 0.6184 | 0.6246 | 0.6310 | 0.6318 | 0.6372 |
| 0.6438 | 0.6473 | 0.6512 | 0.6569 | 0.6638 | 0.6669 | 0.6787 | 0.6820 |
| 0.6942 | 0.6985 | 0.7043 | 0.7132 | 0.7197 | 0.7292 | 0.7356 | 0.7397 |
| 0.7441 | 0.7517 | 0.7611 | 0.7651 | 0.7714 | 0.7788 | 0.7951 | 0.7992 |
| 0.8094 | 0.8156 | 0.8197 | 0.8256 | 0.8343 | 0.8413 | 0.8480 | 0.8512 |
| 0.8606 | 0.8656 | 0.8697 | 0.8737 | 0.8828 | 0.8879 | 0.9080 | 0.9110 |
| 0.9167 | 0.9250 | 0.9271 | 0.9305 | 0.9408 | 0.9486 | 0.9595 | 0.9634 |
| 0.9767 | 0.9867 | 0.9958 | 1.0002 | 1.0042 | 1.0083 | 1.0125 | 1.0214 |
| 1.0281 | 1.0344 | 1.0392 | 1.0581 | 1.0598 | 1.0686 | 1.0758 | 1.0812 |
| 1.0843 | 1.0858 | 1.0910 | 1.0946 | 1.1067 | 1.1084 | 1.1172 | 1.1222 |
| 1.1315 | 1.1422 | 1.1434 | 1.1520 | 1.1556 | 1.1582 | 1.1669 | 1.1695 |
| 1.1787 | 1.1838 | 1.1882 | 1.1967 | 1.2066 | 1.2106 | 1.2139 | 1.2155 |
| 1.2211 | 1.2306 | 1.2321 | 1.2334 | 1.2513 | 1.2520 | 1.2638 | 1.2642 |
| 1.2704 | 1.2765 | 1.2899 | 1.2922 | 1.3029 | 1.3060 | 1.3091 | 1.3124 |
| 1.3231 | 1.3313 | 1.3388 | 1.3457 | 1.3468 | 1.3511 | 1.3565 | 1.3597 |
| 1.3690 | 1.3786 | 1.3852 | 1.3955 | 1.4000 | 1.4025 | 1.4102 | 1.4170 |
| 1.4269 | 1.4338 | 1.4384 | 1.4508 | 1.4608 | 1.4709 | 1.4760 | 1.4872 |
| 1.4929 | 1.4999 | 1.5008 | 1.5091 | 1.5215 | 1.5314 | 1.5448 | 1.5468 |
| 1.5590 | 1.5692 | 1.5811 | 1.5817 | 1.5923 | 1.6066 | 1.6135 | 1.6315 |
| 1.6476 | 1.6572 | 1.6837 | 1.7001 | 1.7056 | 1.7234 | 1.7607 | 1.7762 |

| | | | | | | | |
|---|---|---|---|---|---|---|---|
| 1.7838 | 1.8074 | 1.8144 | 1.8273 | 1.8480 | 1.8740 | 1.8817 | 1.9179 |
| 1.9489 | 1.9603 | 1.9887 | 2.0095 | 2.0459 | 2.1081 | 2.1433 | 2.1736 |
| 2.1984 | 2.2572 | 2.3271 | 2.3505 | 2.3775 | 2.3937 | 2.4011 | 2.4360 |
| 2.4421 | 2.4542 | 2.4655 | 2.4745 | 2.4767 | 2.4877 | 2.5150 | 2.5184 |
| 2.5259 | 2.5321 | 2.5449 | 2.5536 | 2.5602 | 2.5711 | 2.5794 | 2.5835 |
| 2.6023 | 2.6065 | 2.6164 | 2.6182 | 2.6289 | 2.6396 | 2.6438 | 2.6518 |
| 2.6595 | 2.6651 | 2.6789 | 2.6877 | 2.6953 | 2.7016 | 2.7083 | 2.7095 |
| 2.7198 | 2.7239 | 2.7310 | 2.7370 | 2.7414 | 2.7498 | 2.7565 | 2.7604 |
| 2.7709 | 2.7822 | 2.7877 | 2.7993 | 2.8057 | 2.8110 | 2.8261 | 2.8312 |
| 2.8397 | 2.8504 | 2.8536 | 2.8611 | 2.8622 | 2.8754 | 2.8881 | 2.8909 |
| 2.8940 | 2.9016 | 2.9092 | 2.9143 | 2.9222 | 2.9242 | 2.9374 | 2.9455 |
| 2.9499 | 2.9600 | 2.9743 | 2.9779 | 2.9838 | 2.9932 | 3.0026 | 3.0041 |
| 3.0092 | 3.0114 | 3.0146 | 3.0342 | 3.0380 | 3.0419 | 3.0554 | 3.0610 |
| 3.0627 | 3.0680 | 3.0738 | 3.0747 | 3.0880 | 3.0982 | 3.1172 | 3.1209 |
| 3.1214 | 3.1337 | 3.1398 | 3.1448 | 3.1528 | 3.1628 | 3.1681 | 3.1752 |
| 3.1850 | 3.1905 | 3.1945 | 3.1996 | 3.2106 | 3.2111 | 3.2162 | 3.2244 |
| 3.2368 | 3.2442 | 3.2532 | 3.2588 | 3.2626 | 3.2679 | 3.2725 | 3.2749 |
| 3.2791 | 3.2853 | 3.2945 | 3.2994 | 3.3085 | 3.3237 | 3.3242 | 3.3338 |
| 3.3373 | 3.3437 | 3.3521 | 3.3610 | 3.3649 | 3.3778 | 3.3830 | 3.3897 |
| 3.4047 | 3.4095 | 3.4143 | 3.4211 | 3.4346 | 3.4544 | 3.4579 | 3.4600 |
| 3.4656 | 3.4797 | 3.4879 | 3.4988 | 3.5062 | 3.5137 | 3.5252 | 3.5363 |
| 3.5368 | 3.5411 | 3.5437 | 3.5607 | 3.5641 | 3.5755 | 3.5820 | 3.6034 |
| 3.6094 | 3.6174 | 3.6263 | 3.6334 | 3.6442 | 3.6464 | 3.6520 | 3.6549 |
| 3.6702 | 3.6733 | 3.6754 | 3.6800 | 3.6925 | 3.6974 | 3.7079 | 3.7239 |
| 3.7357 | 3.7367 | 3.7531 | 3.7614 | 3.7715 | 3.7806 | 3.7816 | 3.7915 |
| 3.7960 | 3.8083 | 3.8232 | 3.8385 | 3.8451 | 3.8482 | 3.8586 | 3.8640 |
| 3.8683 | 3.8789 | 3.8883 | 3.9097 | 3.9145 | 3.9219 | 3.9364 | 3.9392 |
| 3.9501 | 3.9540 | 3.9554 | 3.9650 | 3.9761 | 3.9805 | 3.9938 | 4.0024 |
| 4.0114 | 4.0323 | 4.0380 | 4.0533 | 4.0630 | 4.0668 | 4.0744 | 4.0883 |
| 4.0919 | 4.1045 | 4.1202 | 4.1282 | 4.1331 | 4.1391 | 4.1465 | 4.1547 |
| 4.1636 | 4.1664 | 4.1711 | 4.1785 | 4.1857 | 4.2035 | 4.2082 | 4.2089 |
| 4.2215 | 4.2301 | 4.2383 | 4.2536 | 4.2617 | 4.2866 | 4.2990 | 4.3024 |
| 4.3198 | 4.3228 | 4.3239 | 4.3398 | 4.3500 | 4.3515 | 4.3682 | 4.3745 |
| 4.3862 | 4.3927 | 4.3975 | 4.4108 | 4.4231 | 4.4289 | 4.4315 | 4.4384 |
| 4.4586 | 4.4653 | 4.4739 | 4.4778 | 4.4982 | 4.5039 | 4.5151 | 4.5189 |
| 4.5362 | 4.5434 | 4.5649 | 4.5753 | 4.5886 | 4.5919 | 4.6077 | 4.6233 |
| 4.6274 | 4.6367 | 4.6640 | 4.6976 | 4.7111 | 4.7161 | 4.7391 | 4.7485 |
| 4.7766 | 4.7893 | 4.8123 | 4.8364 | 4.8617 | 4.8805 | 4.9026 | 4.9102 |
| 4.9277 | 4.9443 | 4.9627 | 4.9696 | 4.9855 | 4.9907 | 5.0052 | 5.0255 |
| 5.0530 | 5.0796 | 5.0825 | 5.0935 | 5.1112 | 5.1182 | 5.1350 | 5.1951 |
| 5.1992 | 5.2160 | 5.2418 | 5.2572 | 5.2614 | 5.2803 | 5.3030 | 5.3553 |
| 5.3730 | 5.3973 | 5.4165 | 5.4430 | 5.4667 | 5.5197 | 5.5264 | 5.5430 |
| 5.5699 | 5.5975 | 5.6008 | 5.6183 | 5.6359 | 5.6507 | 5.6640 | 5.6676 |
| 5.6883 | 5.7043 | 5.7138 | 5.7322 | 5.7593 | 5.7680 | 5.7954 | 5.8288 |
| 5.8909 | 5.8989 | 5.9417 | 5.9938 | 6.0362 | 6.0687 | 6.0888 | 6.1329 |
| 6.1495 | 6.2429 | 6.2901 | 6.3311 | 6.5322 | 6.5938 | 6.7991 | 23.9664 |
| 24.1419 | 24.1611 | 24.2409 | 24.2817 | 24.2847 | 24.3027 | 24.3196 | 24.4330 |
| 24.5983 | 24.6174 | 24.6294 | 24.8014 | 24.9260 | 35.7577 | 35.7686 | 35.7898 |
| 35.9412 | | | | | | | |

---

Ground-State Mulliken Net Atomic Charges

| Atom | Charge (a.u.) |
|---|---|
| 1 N | 0.324233 |
| 2 C | 0.203551 |
| 3 C | -0.183263 |
| 4 C | -0.144677 |
| 5 C | -0.217066 |
| 6 C | -0.106125 |
| 7 C | -0.359764 |
| 8 C | -0.140133 |
| 9 N | -0.149354 |
| 10 C | 0.053557 |
| 11 N | -0.297860 |
| 12 C | 0.521555 |
| 13 C | -0.237458 |
| 14 C | 0.140744 |
| 15 C | -0.262942 |

```
    16 C                     -0.101495
    17 C                     -0.431956
    18 N                     -0.397621
    19 H                      0.198138
    20 H                      0.213443
    21 H                      0.202401
    22 H                      0.231305
    23 H                      0.172830
    24 H                      0.164445
    25 H                      0.162537
    26 H                      0.198139
    27 H                      0.176443
    28 H                      0.290754
    29 H                      0.195391
    30 H                      0.147275
    31 H                      0.181387
    32 H                      0.143319
    33 H                      0.151466
    34 H                      0.095758
    35 H                      0.160314
    36 H                      0.136567
    37 H                      0.252048
    38 H                      0.312113
 ----------------------------------------
 Sum of atomic charges =     2.000000

-----------------------------------------------------------------
                    Cartesian Multipole Moments
-----------------------------------------------------------------
   Charge (ESU x 10^10)
                9.6064
   Dipole Moment (Debye)
         X       5.5265      Y       1.5020      Z       2.8777
       Tot       6.4093
   Quadrupole Moments (Debye-Ang)
        XX       7.1959     XY       3.6712     YY     -80.9434
        XZ       3.8415     YZ       4.1888     ZZ     -79.3523
   Traceless Quadrupole Moments (Debye-Ang)
       QXX     174.6875    QYY     -89.7305    QZZ     -84.9571
       QXY      11.0137    QXZ      11.5246    QYZ      12.5664
   Octopole Moments (Debye-Ang^2)
       XXX      82.4370    XXY     -36.0328    XYY      27.4744
       YYY     -20.7072    XXZ      14.0188    XYZ      23.6594
       YYZ     -20.3351    XZZ     -21.6255    YZZ       9.6295
       ZZZ      13.4974
   Traceless Octopole Moments (Debye-Ang^2)
       XXX     441.9823    YYY     113.3866    ZZZ     137.8314
       XXY    -399.1600    XXZ     188.7389    XYY     147.2581
       XYZ     354.8904    XZZ    -589.2404    YYZ    -326.5703
       YZZ     285.7734
   Hexadecapole Moments (Debye-Ang^3)
      XXXX   -3442.5297   XXXY    -117.0840   XXYY    -887.8988
      XYYY    -104.0973   YYYY    -908.6875   XXXZ    -221.8182
      XXYZ     101.0971   XYYZ    -128.5490   YYYZ    -101.6912
      XXZZ    -936.9383   XYZZ      15.1237   YYZZ    -245.3628
      XZZZ    -403.9706   YZZZ    -145.7853   ZZZZ    -681.6478
   Traceless Hexadecapole Moments (Debye-Ang^3)
      XXXX   30038.0117   XXXY   -3021.2284   XXXZ   10654.2939
      XXYY  -11109.4298   XXYZ   12799.1073   XXZZ  -18928.5819
      XYYY   -1657.6228   XYYZ   -2182.5831   XYZZ    4678.8512
      XZZZ   -8471.7107   YYYY    5803.8463   YYYZ   -4125.8423
      YYZZ    5305.5835   YZZZ   -8673.2651   ZZZZ   13622.9984
-----------------------------------------------------------------
Total job time:  640.28s(wall), 460.55s(cpu)
Mon Oct 14 03:37:14 2024
```

```
Wavefunction Version of Q-Chem

 Parts of Q-Chem use Armadillo 8.300.2 (Tropical Shenanigans).
 http://arma.sourceforge.net/

 Q-Chem begins on Sun Oct 13 18:15:39 2024


     Scratch files written to
C:/Users/hille/AppData/Local/Temp/WFB0CB2A5C99266D7A/Conformer.11//scratch///
Processing default memory
  ... MEM_TOTAL 4076 MB (default) [16 cores]
Processing $rem in C:/Program
Files/Wavefunction/Spartan24v110/P4e//../auxdir/config/preferences:
   (site specific preferences)
  ... THRESH              9
  ... SMALL_PROD_XCMAT   9
  ... BASIS_LIN_DEP_THRESH     5
  ... SCF_ALGORITHM     DIIS_GDM
  ... MAXSCF            250
  ... MAXDIIS          45
  ... THRESHDIIS         -1  (i.e. don't switch on delta-E)
  ... ECP_FIT          TRUE (Convert deprecated ECP files)
  ... GUI              GUI_SPARTAN
  ... TERSE_OUTPUT     TRUE !turn on spartan printing
  ... SCF_CONVERGENCE  7
  ... CCMAN2 FALSE  (qc4.3)
  ... SYMMETRY   FALSE  ! turn of symmetry for spartan16
  ... SYM_IGNORE TRUE   ! ..use FORCESYMMETRY to override
  ... GEOM_OPT_TOL_GRADIENT       700  ! loosen tolernaces for organic geometries
  ... GEOM_OPT_TOL_DISPLACEMENT  1400  ! was 1200 = .0012
  ... GEOM_OPT_TOL_ENERGY        2000  ! was  100 = .000 001
  ... GEN_SCFMAN  FALSE
Processing $rem in input file
  ... JOBTYPE    OPT
  ... TIDY_SYM   TRUE
  ... METHOD            WB97X-D
  ... xc_grid           75000302  (75,302)
  ... BASIS             6-311+G**
  ... THRESH             12  #diffuse default
  ... MAXSCF            350  #diffuse default
  ... GEOM_OPT_TOL_ENERGY       22850
  ... GEOM_OPT_TOL_GRADIENT       800
  ... VARTHRESH         2    (default DFT)
  ... INCDFT            TRUE (default DFT)
  ... GEOM_OPT_HESSIAN      READ (main opt)
  ... EXTERNAL_HESSIAN      1
  ... GUI                GUI_SPARTAN
  ... TERSE_OUTPUT       TRUE
NAlpha2: 130
NElect   130
Mult     1

Checking the input file for inconsistencies...  ...done.

--------------------------------------------------------------
User input:
--------------------------------------------------------------
$comment
Molecule10
$end
$molecule
 2 1
    7     2.2457708795    0.12352269454    -1.5799866909
    6     3.0892548848     1.1987715257    -1.6904415517
    6     3.2521720833     1.8187544328    -2.9409231525
    6     2.5827942576     1.3445338308    -4.0586465233
    6     1.7580682521    0.24346554115    -3.9288488937
    6      1.611027358   -0.35291060576    -2.6853099322
```

```
    6     3.8682030474     1.7265453769   -0.51681377192
    6     2.0407961517   -0.59147092677   -0.28017022701
    7    -1.2122077549    -1.9411410434    1.0624407701
    6    -1.9903920332   -0.85100574072    1.0103820666
    7    -1.5243150148    0.34829976457   0.58956166387
    6   -0.24294569859    0.43201365756     0.213283458
    6    0.60312724598   -0.66696131803   0.18818467201
    6   0.067503130999    -1.8725508605   0.65938021487
    6    -3.4157191861   -0.95154683702     1.477226574
    6    -3.5143888913   -0.73582643442    2.9860323549
    6    -4.9528615263   -0.83298237068    3.4682415772
    7    0.17986276443     1.7005800957   -0.14517114516
    1     3.9136497344     2.6786485874   -3.0517688448
    1     2.7139698935     1.8281890443   -5.0264073383
    1     1.2345524301   -0.14965648696   -4.7984708664
    1    0.97692055203    -1.2267608464   -2.5700901954
    1     4.4826626613     2.5889178761  -0.79635201338
    1      4.542237081    0.95364676734   -0.1352711469
    1      3.191261434     2.0531410594   0.27725938399
    1     2.6387801954   -0.13438163442   0.51001974021
    1     2.4454229349    -1.5978373925  -0.44996020021
    1    -1.6106038328    -2.8148946678    1.4133412854
    1    0.62864863907    -2.8004718783   0.72573903124
    1    -4.0199376666   -0.20553927727   0.94746896183
    1    -3.8133551525    -1.9366620798    1.2062862304
    1    -3.1106249356    0.24849223908    3.2531851694
    1    -2.9067255097    -1.4808269709    3.5142069838
    1    -5.5800004564   -0.07603659416    2.9865193671
    1    -5.0013766925    -0.6757280367    4.5502888963
    1    -5.3741833752    -1.8192783382    3.2490679345
    1    0.84420208345     2.1095531494   0.50885304965
    1   -0.64124996823      2.317394698   -0.14233689159
$end
$rem
JOBTYPE    OPT
TIDY_SYM   TRUE
METHOD          WB97X-D
xc_grid         75000302  (75,302)
BASIS           6-311+G**
THRESH           12  #diffuse default
MAXSCF          350  #diffuse default
GEOM_OPT_TOL_ENERGY     22850
GEOM_OPT_TOL_GRADIENT     800
VARTHRESH       2    (default DFT)
INCDFT          TRUE (default DFT)
GEOM_OPT_HESSIAN      READ (main opt)
EXTERNAL_HESSIAN      1
GUI              GUI_SPARTAN
TERSE_OUTPUT     TRUE
$end
$opt
$end
--------------------------------------------------------------
 --------------------------------------------------------------
             Standard Nuclear Orientation (Angstroms)
    I     Atom           X                Y                Z
 --------------------------------------------------------------
    1      N       2.2457708795     0.1235226945    -1.5799866909
    2      C       3.0892548848     1.1987715257    -1.6904415517
    3      C       3.2521720833     1.8187544328    -2.9409231525
    4      C       2.5827942576     1.3445338308    -4.0586465233
    5      C       1.7580682521     0.2434655411    -3.9288488937
    6      C       1.6110273580    -0.3529106058    -2.6853099322
    7      C       3.8682030474     1.7265453769    -0.5168137719
    8      C       2.0407961517    -0.5914709268    -0.2801702270
    9      N      -1.2122077549    -1.9411410434     1.0624407701
   10      C      -1.9903920332    -0.8510057407     1.0103820666
   11      N      -1.5243150148     0.3482997646     0.5895616639
   12      C      -0.2429456986     0.4320136576     0.2132834580
   13      C       0.6031272460    -0.6669613180     0.1881846720
```

```
   14      C       0.0675031310    -1.8725508605     0.6593802149
   15      C      -3.4157191861    -0.9515468370     1.4772265740
   16      C      -3.5143888913    -0.7358264344     2.9860323549
   17      C      -4.9528615263    -0.8329823707     3.4682415772
   18      N       0.1798627644     1.7005800957    -0.1451711452
   19      H       3.9136497344     2.6786485874    -3.0517688448
   20      H       2.7139698935     1.8281890443    -5.0264073383
   21      H       1.2345524301    -0.1496564870    -4.7984708664
   22      H       0.9769205520    -1.2267608464    -2.5700901954
   23      H       4.4826626613     2.5889178761    -0.7963520134
   24      H       4.5422370810     0.9536467673    -0.1352711469
   25      H       3.1912614340     2.0531410594     0.2772593840
   26      H       2.6387801954    -0.1343816344     0.5100197402
   27      H       2.4454229349    -1.5978373925    -0.4499602002
   28      H      -1.6106038328    -2.8148946678     1.4133412854
   29      H       0.6286486391    -2.8004718783     0.7257390312
   30      H      -4.0199376666    -0.2055392773     0.9474689618
   31      H      -3.8133551525    -1.9366620798     1.2062862304
   32      H      -3.1106249356     0.2484922391     3.2531851694
   33      H      -2.9067255097    -1.4808269709     3.5142069838
   34      H      -5.5800004564    -0.0760365942     2.9865193671
   35      H      -5.0013766925    -0.6757280367     4.5502888963
   36      H      -5.3741833752    -1.8192783382     3.2490679345
   37      H       0.8442020835     2.1095531494     0.5088530496
   38      H      -0.6412499682     2.3173946980    -0.1423368916
 ----------------------------------------------------------------
 Nuclear Repulsion Energy =        1296.03843610 hartrees
 There are       65 alpha and       65 beta electrons
 Requested basis set is 6-311+G(d,p)
 There are 188 shells and 516 basis functions

 Total QAlloc Memory Limit   4076 MB
 Mega-Array Size       188 MB
 MEM_STATIC part       192 MB

 ..                                                                          (5.2.P)

------------------------------------------------
-  Entering fldman on Sun Oct 13 18:15:39 2024  -
------------------------------------------------

 A cutoff of  1.0D-12 yielded  12441 shell pairs
 There are     99813 function pairs (    106446 Cartesian)
 Smallest overlap matrix eigenvalue = 1.31E-06
 Linear dependence detected in AO basis
 Tighter screening thresholds may be required for diffuse basis sets
 Use S2THRESH > 12 and THRESH = 14 in case of SCF convergence issues
 Number of orthogonalized atomic orbitals = 510
 Maximum deviation from orthogonality = 3.186E-11

 Scale SEOQF with 1.000000e-02/1.000000e-01/1.000000e-01

 Standard Electronic Orientation quadrupole field applied
 Nucleus-field energy     =    -0.0000000475 hartrees

------------------------------------------------
-  Entering gesman on Sun Oct 13 18:15:39 2024  -
------------------------------------------------

 Guess from superposition of atomic densities
 Warning:  Energy on first SCF cycle will be non-variational
 SAD guess density has 132.000000 electrons

------------------------------------------------
-  Entering scfman on Sun Oct 13 18:15:39 2024  -
------------------------------------------------

 Long-range K will be added via erf
 Coulomb attenuation parameter = 0.2 bohr**(-1)
 A restricted hybrid HF-DFT SCF calculation will be
```

```
 performed using Pulay DIIS + Geometric Direct Minimization
 Exchange:     0.2220 Hartree-Fock + 1.0000 wB97X-D + LR-HF
 Correlation:  1.0000 wB97X-D
 Using Euler-Maclaurin-Lebedev (75,302) quadrature formula
 Dispersion:   Grimme D
 SCF converges when RMS gradient is below 1.0E-07
 Exchange:     0.2220 Hartree-Fock + 1.0000 wB97X-D + LR-HF
 Correlation:  1.0000 wB97X-D
 Using Euler-Maclaurin-Lebedev (75,302) quadrature formula
 Dispersion:   Grimme D
 ---------------------------------------
  Cycle       Energy         DIIS Error
 ---------------------------------------
    1    -770.2952233737      2.95E-02
    2    -763.7481294907      2.39E-03
    3    -763.7570087470      2.57E-03
    4    -763.9426856405      3.54E-04
    5    -763.9456362023      1.90E-04
    6    -763.9466443902      3.53E-05
    7    -763.9466805338      1.46E-05
    8    -763.9466872245      3.63E-06
    9    -763.9466877808      1.89E-06
   10    -763.9466879284      5.87E-07
   11    -763.9466879479      2.26E-07
   12    -763.9466879521      8.55E-08 Convergence criterion met
 ---------------------------------------
 SCF time:  CPU 397.67 s  wall 666.67 s
 SCF   energy in the final basis set = -763.94668795
 Total energy in the final basis set = -763.94668795

-------------------------------------------------
-  Entering anlman on Sun Oct 13 18:26:46 2024  -
-------------------------------------------------



 --------------------------------------------------------------

                   Orbital Energies (a.u.)
 --------------------------------------------------------------

 Alpha MOs
 -- Occupied --
-14.8134 -14.8134 -14.7237 -14.7161 -10.6743 -10.6711 -10.6621 -10.6358
-10.6354 -10.6228 -10.6152 -10.5743 -10.5668 -10.5656 -10.5453 -10.5327
-10.4987 -10.4538  -1.4292  -1.3943  -1.3456  -1.2670  -1.2274  -1.1986
 -1.1757  -1.1452  -1.1019  -1.0744  -1.0651  -1.0500  -1.0236  -1.0053
 -0.9839  -0.9480  -0.9444  -0.9034  -0.8974  -0.8879  -0.8781  -0.8588
 -0.8479  -0.8392  -0.8335  -0.8158  -0.8075  -0.7909  -0.7830  -0.7765
 -0.7716  -0.7696  -0.7470  -0.7433  -0.7291  -0.7214  -0.7190  -0.7045
 -0.6841  -0.6691  -0.6512  -0.6500  -0.6360  -0.6262  -0.6210  -0.6160
 -0.6085
 -- Virtual --
 -0.2786  -0.2643  -0.2563  -0.2198  -0.1773  -0.1638  -0.1470  -0.1438
 -0.1347  -0.1254  -0.1171  -0.1127  -0.1109  -0.1061  -0.0985  -0.0926
 -0.0901  -0.0872  -0.0791  -0.0774  -0.0746  -0.0729  -0.0716  -0.0670
 -0.0622  -0.0592  -0.0566  -0.0514  -0.0460  -0.0439  -0.0381  -0.0323
 -0.0306  -0.0257  -0.0212  -0.0170  -0.0165  -0.0129  -0.0101  -0.0082
 -0.0057  -0.0020   0.0008   0.0041   0.0076   0.0110   0.0129   0.0155
  0.0179   0.0217   0.0229   0.0260   0.0317   0.0334   0.0349   0.0385
  0.0431   0.0447   0.0496   0.0522   0.0551   0.0608   0.0650   0.0675
  0.0707   0.0718   0.0763   0.0778   0.0786   0.0851   0.0876   0.0942
  0.0976   0.0994   0.1039   0.1049   0.1061   0.1133   0.1176   0.1199
  0.1218   0.1249   0.1296   0.1324   0.1384   0.1408   0.1436   0.1481
  0.1513   0.1518   0.1591   0.1609   0.1657   0.1701   0.1723   0.1791
  0.1835   0.1852   0.1864   0.1949   0.1992   0.2033   0.2198   0.2277
  0.2347   0.2431   0.2550   0.2726   0.2832   0.2924   0.2996   0.3148
  0.3262   0.3277   0.3320   0.3450   0.3492   0.3547   0.3607   0.3671
  0.3695   0.3753   0.3818   0.3945   0.4013   0.4074   0.4147   0.4206
  0.4296   0.4326   0.4419   0.4472   0.4593   0.4638   0.4686   0.4759
  0.4813   0.4873   0.4925   0.4951   0.5038   0.5117   0.5128   0.5210
```

| | | | | | | | |
|---|---|---|---|---|---|---|---|
| 0.5243 | 0.5274 | 0.5304 | 0.5381 | 0.5455 | 0.5507 | 0.5574 | 0.5587 |
| 0.5655 | 0.5673 | 0.5714 | 0.5792 | 0.5842 | 0.5856 | 0.5924 | 0.6008 |
| 0.6069 | 0.6162 | 0.6217 | 0.6300 | 0.6306 | 0.6427 | 0.6527 | 0.6568 |
| 0.6717 | 0.6730 | 0.6787 | 0.6851 | 0.6923 | 0.7022 | 0.7113 | 0.7125 |
| 0.7170 | 0.7332 | 0.7357 | 0.7421 | 0.7489 | 0.7564 | 0.7754 | 0.7966 |
| 0.7981 | 0.8096 | 0.8131 | 0.8191 | 0.8306 | 0.8373 | 0.8441 | 0.8649 |
| 0.8691 | 0.8752 | 0.8810 | 0.8845 | 0.8921 | 0.9063 | 0.9077 | 0.9277 |
| 0.9344 | 0.9445 | 0.9651 | 0.9814 | 0.9896 | 1.0024 | 1.0224 | 1.0269 |
| 1.0540 | 1.0809 | 1.1043 | 1.1144 | 1.1319 | 1.1447 | 1.1605 | 1.1873 |
| 1.2012 | 1.2112 | 1.2397 | 1.2441 | 1.2671 | 1.2755 | 1.2951 | 1.3010 |
| 1.3066 | 1.3175 | 1.3341 | 1.3387 | 1.3470 | 1.3613 | 1.3651 | 1.3766 |
| 1.3862 | 1.3931 | 1.4001 | 1.4047 | 1.4140 | 1.4224 | 1.4313 | 1.4374 |
| 1.4409 | 1.4515 | 1.4535 | 1.4589 | 1.4662 | 1.4794 | 1.4854 | 1.4897 |
| 1.4995 | 1.5035 | 1.5112 | 1.5141 | 1.5199 | 1.5363 | 1.5453 | 1.5474 |
| 1.5533 | 1.5683 | 1.5731 | 1.5813 | 1.5921 | 1.5977 | 1.6114 | 1.6136 |
| 1.6256 | 1.6283 | 1.6356 | 1.6494 | 1.6664 | 1.6701 | 1.6786 | 1.6899 |
| 1.6975 | 1.7155 | 1.7224 | 1.7277 | 1.7412 | 1.7437 | 1.7649 | 1.7675 |
| 1.7807 | 1.7952 | 1.8046 | 1.8276 | 1.8400 | 1.8470 | 1.8550 | 1.8733 |
| 1.8758 | 1.8969 | 1.9066 | 1.9194 | 1.9376 | 1.9544 | 1.9636 | 1.9969 |
| 2.0071 | 2.0210 | 2.0239 | 2.0345 | 2.0494 | 2.0529 | 2.0796 | 2.0879 |
| 2.0984 | 2.1020 | 2.1142 | 2.1279 | 2.1453 | 2.1845 | 2.1883 | 2.2199 |
| 2.2309 | 2.2415 | 2.2465 | 2.2576 | 2.2683 | 2.2908 | 2.2942 | 2.3032 |
| 2.3127 | 2.3175 | 2.3488 | 2.3602 | 2.3693 | 2.3775 | 2.3856 | 2.4043 |
| 2.4155 | 2.4202 | 2.4268 | 2.4450 | 2.4468 | 2.4740 | 2.4782 | 2.4853 |
| 2.4952 | 2.5012 | 2.5141 | 2.5243 | 2.5277 | 2.5410 | 2.5440 | 2.5544 |
| 2.5613 | 2.5710 | 2.5858 | 2.5922 | 2.6031 | 2.6060 | 2.6109 | 2.6232 |
| 2.6332 | 2.6371 | 2.6507 | 2.6533 | 2.6737 | 2.6764 | 2.6819 | 2.6949 |
| 2.7073 | 2.7133 | 2.7241 | 2.7290 | 2.7351 | 2.7518 | 2.7638 | 2.7678 |
| 2.7794 | 2.7944 | 2.8078 | 2.8239 | 2.8356 | 2.8477 | 2.8675 | 2.8885 |
| 2.9053 | 2.9369 | 2.9378 | 2.9545 | 3.0421 | 3.0576 | 3.0792 | 3.1264 |
| 3.1348 | 3.1812 | 3.2412 | 3.2645 | 3.2760 | 3.3193 | 3.3270 | 3.3866 |
| 3.4060 | 3.4620 | 3.4894 | 3.5289 | 3.5431 | 3.5947 | 3.6221 | 3.6462 |
| 3.6586 | 3.6706 | 3.7005 | 3.7303 | 3.7312 | 3.7524 | 3.7647 | 3.7752 |
| 3.8067 | 3.8669 | 3.8909 | 3.9120 | 3.9548 | 4.0453 | 4.0545 | 4.0964 |
| 4.1146 | 4.1924 | 4.3006 | 4.3086 | 4.5616 | 4.6384 | 4.6565 | 4.7356 |
| 4.8731 | 5.0253 | 5.1421 | 23.5336 | 23.5600 | 23.6875 | 23.7094 | 23.7331 |
| 23.7894 | 23.8084 | 23.8212 | 23.8298 | 23.8761 | 23.9156 | 23.9191 | 23.9341 |
| 24.0317 | 35.4112 | 35.4398 | 35.4816 | 35.5744 | | | |

Ground-State Mulliken Net Atomic Charges

| Atom | Charge (a.u.) |
|---|---|
| 1 N | 0.485197 |
| 2 C | 0.122022 |
| 3 C | -0.031018 |
| 4 C | -0.136145 |
| 5 C | -0.198888 |
| 6 C | -0.134640 |
| 7 C | -0.726260 |
| 8 C | -0.264642 |
| 9 N | -0.048428 |
| 10 C | -0.451555 |
| 11 N | 0.077067 |
| 12 C | 0.118686 |
| 13 C | -0.061411 |
| 14 C | 0.080449 |
| 15 C | -0.370624 |
| 16 C | -0.089563 |
| 17 C | -0.607853 |
| 18 N | -0.492580 |
| 19 H | 0.221754 |
| 20 H | 0.231953 |
| 21 H | 0.227279 |
| 22 H | 0.220170 |
| 23 H | 0.233070 |
| 24 H | 0.244963 |
| 25 H | 0.176089 |
| 26 H | 0.269133 |

```
    27 H                       0.243920
    28 H                       0.404875
    29 H                       0.226041
    30 H                       0.242828
    31 H                       0.211113
    32 H                       0.198798
    33 H                       0.170106
    34 H                       0.177900
    35 H                       0.199081
    36 H                       0.169228
    37 H                       0.312368
    38 H                       0.349517
  ----------------------------------------
  Sum of atomic charges =      2.000000

 -----------------------------------------------------------------
                    Cartesian Multipole Moments
 -----------------------------------------------------------------
    Charge (ESU x 10^10)
                9.6064
    Dipole Moment (Debye)
        X       6.0398      Y      -2.3577      Z      -3.8673
      Tot       7.5494
    Quadrupole Moments (Debye-Ang)
        XX     -33.4532     XY      26.4707     YY     -64.6347
        XZ     -40.8381     YZ     -18.3669     ZZ     -47.5682
    Traceless Quadrupole Moments (Debye-Ang)
       QXX      45.2966    QYY     -48.2481    QZZ       2.9516
       QXY      79.4120    QXZ    -122.5144    QYZ     -55.1008
    Octopole Moments (Debye-Ang^2)
       XXX     -20.1035    XXY      35.4681    XYY       6.9869
       YYY     -19.8924    XXZ      27.7004    XYZ     -20.6312
       YYZ      24.9682    XZZ      28.3985    YZZ      27.1190
       ZZZ     -76.7017
    Traceless Octopole Moments (Debye-Ang^2)
       XXX    -439.0901    YYY    -682.6388    ZZZ    -934.2274
       XXY     403.9381    XXZ     487.6049    XYY      58.9577
       XYZ    -309.4676    XZZ     380.1323    YYZ     446.6224
       YZZ     278.7007
    Hexadecapole Moments (Debye-Ang^3)
      XXXX   -3389.4820   XXXY    -224.3355   XXYY    -662.8105
      XYYY    -446.0463   YYYY    -770.3858   XXXZ     941.4944
      XXYZ     -75.4586   XYYZ     344.9013   YYYZ     315.4261
      XXZZ    -788.0560   XYZZ       9.4760   YYZZ    -535.8974
      XZZZ     855.7381   YZZZ     198.1125   ZZZZ   -1950.3065
    Traceless Hexadecapole Moments (Debye-Ang^3)
      XXXX  -11017.5591   XXXY    6185.5334   XXXZ    2460.8884
      XXYY    2295.4220   XXYZ  -14492.6637   XXZZ    8722.1370
      XYYY  -17094.1021   XYYZ    4082.6310   XYZZ   10908.5687
      XZZZ   -6543.5195   YYYY    5574.6063   YYYZ   13411.2023
      YYZZ   -7870.0284   YZZZ    1081.4614   ZZZZ    -852.1087
 -----------------------------------------------------------------

--------------------------------------------------
-  Entering drvman on Sun Oct 13 18:26:46 2024  -
--------------------------------------------------

 Calculating analytic gradient of the SCF energy
 Gradient of SCF Energy
           1           2           3           4           5           6
    1  -0.0039724  -0.0060932   0.0034434  -0.0059461   0.0077750   0.0013506
    2  -0.0090484  -0.0114772   0.0060676  -0.0076535   0.0119498  -0.0039384
    3   0.0034014   0.0087190  -0.0036822  -0.0008615  -0.0070077   0.0102403
           7           8           9          10          11          12
    1   0.0041192  -0.0044724  -0.0053315   0.0221561   0.0075661   0.0167611
    2   0.0047172   0.0072445  -0.0017496  -0.0418133   0.0421215  -0.0321404
    3  -0.0004741   0.0042576  -0.0010388   0.0105117  -0.0076061  -0.0087331
          13          14          15          16          17          18
    1  -0.0062256  -0.0193543  -0.0055219  -0.0019032   0.0039032   0.0143154
    2   0.0415370  -0.0127043   0.0014694  -0.0025376   0.0007990  -0.0014164
```

```
   3  -0.0028160   0.0110541   0.0106440  -0.0110533  -0.0011001  -0.0086362
          19          20          21          22          23          24
   1   0.0023838   0.0004789  -0.0006858  -0.0018375   0.0041256   0.0004034
   2   0.0050552   0.0017104   0.0017470  -0.0019169   0.0042980  -0.0013705
   3  -0.0056245  -0.0037157  -0.0059182  -0.0038488   0.0020055  -0.0026123
          25          26          27          28          29          30
   1  -0.0025894  -0.0022005   0.0021816  -0.0037098  -0.0015321  -0.0048004
   2   0.0007256  -0.0023375  -0.0025160  -0.0044000  -0.0026266   0.0010339
   3  -0.0022362   0.0059776  -0.0049050   0.0018391   0.0021489  -0.0009554
          31          32          33          34          35          36
   1  -0.0001028  -0.0007018  -0.0005999  -0.0003543   0.0002423   0.0004975
   2   0.0002006   0.0015368  -0.0001221   0.0011081   0.0003754  -0.0012922
   3  -0.0012507   0.0025315   0.0005984  -0.0010674   0.0026584  -0.0007606
          37          38
   1  -0.0067045  -0.0070636
   2   0.0040363   0.0033279
   3   0.0069179   0.0023987
 Max gradient component =       4.212E-02
 RMS gradient           =       9.478E-03
 Gradient time:  CPU 161.59 s  wall 269.28 s

-------------------------------------------------
-  Entering optman on Sun Oct 13 18:31:16 2024  -
-------------------------------------------------

 Geometry Optimization Parameters
   NAtoms,    NIC,     NZ,  NCons,   NDum,   NFix, NCnnct, MaxDiis
       38     272       0       0       0       0       0       0

 Cartesian Hessian read from HESS file


** GEOMETRY OPTIMIZATION IN DELOCALIZED INTERNAL COORDINATES **
   Searching for a Minimum

   Optimization Cycle:   1

                    Coordinates (Angstroms)
     ATOM              X                Y                Z
      1  N         2.2457708795     0.1235226945    -1.5799866909
      2  C         3.0892548848     1.1987715257    -1.6904415517
      3  C         3.2521720833     1.8187544328    -2.9409231525
      4  C         2.5827942576     1.3445338308    -4.0586465233
      5  C         1.7580682521     0.2434655412    -3.9288488937
      6  C         1.6110273580    -0.3529106058    -2.6853099322
      7  C         3.8682030474     1.7265453769    -0.5168137719
      8  C         2.0407961517    -0.5914709268    -0.2801702270
      9  N        -1.2122077549    -1.9411410434     1.0624407701
     10  C        -1.9903920332    -0.8510057407     1.0103820666
     11  N        -1.5243150148     0.3482997646     0.5895616639
     12  C        -0.2429456986     0.4320136576     0.2132834580
     13  C         0.6031272460    -0.6669613180     0.1881846720
     14  C         0.0675031310    -1.8725508605     0.6593802149
     15  C        -3.4157191861    -0.9515468370     1.4772265740
     16  C        -3.5143888913    -0.7358264344     2.9860323549
     17  C        -4.9528615263    -0.8329823707     3.4682415772
     18  N         0.1798627644     1.7005800957    -0.1451711452
     19  H         3.9136497344     2.6786485874    -3.0517688448
     20  H         2.7139698935     1.8281890443    -5.0264073383
     21  H         1.2345524301    -0.1496564870    -4.7984708664
     22  H         0.9769205520    -1.2267608464    -2.5700901954
     23  H         4.4826626613     2.5889178761    -0.7963520134
     24  H         4.5422370810     0.9536467673    -0.1352711469
     25  H         3.1912614340     2.0531410594     0.2772593840
     26  H         2.6387801954    -0.1343816344     0.5100197402
     27  H         2.4454229349    -1.5978373925    -0.4499602002
     28  H        -1.6106038328    -2.8148946678     1.4133412854
     29  H         0.6286486391    -2.8004718783     0.7257390312
     30  H        -4.0199376666    -0.2055392773     0.9474689618
     31  H        -3.8133551525    -1.9366620798     1.2062862304
```

```
    32  H        -3.1106249356    0.2484922391    3.2531851694
    33  H        -2.9067255097   -1.4808269709    3.5142069838
    34  H        -5.5800004564   -0.0760365942    2.9865193671
    35  H        -5.0013766925   -0.6757280367    4.5502888963
    36  H        -5.3741833752   -1.8192783382    3.2490679345
    37  H         0.8442020834    2.1095531494    0.5088530497
    38  H        -0.6412499682    2.3173946980   -0.1423368916
  Point Group: c1    Number of degrees of freedom:   108


  Energy is   -763.946687952


 Attempting to generate delocalized internal coordinates

 Transforming Cartesian Hessian to Internal Coordinates
 Hessian Transformation does not Include Derivative of B-matrix
  internal optimization (0)

108 Hessian modes will be used to form the next step
  Hessian Eigenvalues:
     0.001807    0.002732    0.003599    0.004198    0.007214    0.011189
     0.012467    0.018896    0.019203    0.019904    0.021078    0.022029
     0.022550    0.023346    0.024036    0.024291    0.025214    0.026868
     0.027706    0.028206    0.028982    0.030632    0.035823    0.037320
     0.038360    0.040836    0.043075    0.043687    0.043845    0.046724
     0.048856    0.053708    0.055767    0.057613    0.067704    0.077191
     0.083898    0.095013    0.122087    0.122100    0.126857    0.128045
     0.131380    0.132789    0.135506    0.139163    0.143862    0.146665
     0.147505    0.148251    0.148875    0.150911    0.151656    0.152473
     0.153224    0.156233    0.175921    0.191517    0.206377    0.213436
     0.216604    0.228233    0.238524    0.239134    0.246570    0.255286
     0.265386    0.271685    0.275170    0.282370    0.295184    0.298528
     0.300135    0.300593    0.301100    0.302446    0.302519    0.304115
     0.304825    0.304902    0.304972    0.306448    0.309385    0.311141
     0.319097    0.329987    0.330678    0.332754    0.335189    0.339326
     0.340959    0.347781    0.348571    0.362204    0.363615    0.383816
     0.385095    0.394141    0.401212    0.405358    0.415444    0.422411
     0.433253    0.436604    0.451422    0.466964    0.646626    0.724327

 Minimum search - taking simple RFO step
 Searching for Lamda that Minimizes Along All modes
 Value Taken    Lamda =  -0.02340141
 Calculated Step too Large.  Step scaled by  0.698186 !!
 Step Taken.  Stepsize is  0.300000  0.012338

                             Maximum     Tolerance    Cnvgd?
         Gradient           0.027314      0.000800      NO
         Displacement       0.110954      0.001400      NO
         Energy change     *********      0.000228      NO


 New Cartesian Coordinates Obtained by Inverse Iteration

 Displacement from previous Coordinates is:  0.540971
 ----------------------------------------------------------------
             Standard Nuclear Orientation (Angstroms)
    I     Atom           X                Y                Z
 ----------------------------------------------------------------
    1      N       2.2487038768     0.1327463258    -1.5667866716
    2      C       3.0912286076     1.1997273251    -1.6809009461
    3      C       3.2332354810     1.8065795545    -2.9216303747
    4      C       2.5441169256     1.3292275818    -4.0254284965
    5      C       1.7121811443     0.2244559513    -3.8866293495
    6      C       1.5916697554    -0.3510180129    -2.6513918203
    7      C       3.8760481522     1.7005877659    -0.5055929121
    8      C       2.0549072119    -0.5834161969    -0.2757507471
    9      N      -1.1971799107    -1.8983486318     1.0304092288
   10      C      -2.0053221715    -0.8163288888     0.9814423531
   11      N      -1.5487542692     0.3533672582     0.5853774168
```

```
  12      C      -0.2602380693     0.4561082667     0.2288062637
  13      C       0.6146414601    -0.6580912534     0.1857794381
  14      C       0.0896062756    -1.8383657165     0.6318531345
  15      C      -3.4139762776    -0.9483252049     1.4548531467
  16      C      -3.5089486992    -0.7381518491     2.9774272500
  17      C      -4.9490084311    -0.8556613781     3.4631789050
  18      N       0.1616057741     1.7091130962    -0.1595571723
  19      H       3.8969770117     2.6574747413    -3.0267658012
  20      H       2.6622056807     1.8080131173    -4.9929479002
  21      H       1.1721601610    -0.1815491205    -4.7332986481
  22      H       0.9643438935    -1.2208371932    -2.4996245733
  23      H       4.4832362972     2.5563671599    -0.7993113855
  24      H       4.5550441640     0.9305105161    -0.1276313113
  25      H       3.2251360217     2.0284426724     0.3093116263
  26      H       2.6597849835    -0.1159025258     0.4985966507
  27      H       2.4569522390    -1.5890334551    -0.4264810203
  28      H      -1.5849534913    -2.7740509756     1.3749727452
  29      H       0.6481338363    -2.7665144326     0.6889500775
  30      H      -4.0231660544    -0.2061022767     0.9344266369
  31      H      -3.8013428426    -1.9383323622     1.1888816117
  32      H      -3.1121396172     0.2495886876     3.2339456311
  33      H      -2.8859655425    -1.4763080923     3.4968656282
  34      H      -5.5867750425    -0.1070468988     2.9860681699
  35      H      -4.9966958297    -0.6999673785     4.5425039095
  36      H      -5.3616228299    -1.8446032826     3.2461910808
  37      H       0.9175064201     2.1074076078     0.3865878245
  38      H      -0.6233362944     2.3582374980    -0.1466995986
 ----------------------------------------------------------------
 Nuclear Repulsion Energy =        1300.92603757 hartrees
 There are       65 alpha and       65 beta electrons

--------------------------------------------------
-  Entering fldman on Sun Oct 13 18:31:16 2024  -
--------------------------------------------------

 Applying Cartesian multipole field
    Component           Value
    ---------           -----
     (2,0,0)        1.00000E-12
     (0,2,0)        2.00000E-11
     (0,0,2)       -3.00000E-11
 Nucleus-field energy     =    -0.0000000467 hartrees

--------------------------------------------------
-  Entering gesman on Sun Oct 13 18:31:16 2024  -
--------------------------------------------------

 Requested basis set is 6-311+G(d,p)
 There are 188 shells and 516 basis functions
 A cutoff of  1.0D-12 yielded  12477 shell pairs
 There are    100034 function pairs (    106697 Cartesian)
 Smallest overlap matrix eigenvalue = 1.33E-06
 Linear dependence detected in AO basis
 Tighter screening thresholds may be required for diffuse basis sets
 Use S2THRESH > 12 and THRESH = 14 in case of SCF convergence issues
 Number of orthogonalized atomic orbitals = 510
 Maximum deviation from orthogonality = 4.433E-11
 Guess MOs from SCF MO coefficient file
 Reading MOs from coefficient file
 Reading MOs from coefficient file

--------------------------------------------------
-  Entering scfman on Sun Oct 13 18:31:16 2024  -
--------------------------------------------------

 Long-range K will be added via erf
 Coulomb attenuation parameter = 0.2 bohr**(-1)
 A restricted hybrid HF-DFT SCF calculation will be
 performed using Pulay DIIS + Geometric Direct Minimization
 Exchange:     0.2220 Hartree-Fock + 1.0000 wB97X-D + LR-HF
```

```
 Correlation:  1.0000 wB97X-D
 Using Euler-Maclaurin-Lebedev (75,302) quadrature formula
 Dispersion:   Grimme D
 SCF converges when RMS gradient is below 1.0E-07
 Geometry optimization detected.  Setting ReadMinima to 0
 Setting SaveMinima to 0
 ---------------------------------------
  Cycle       Energy         DIIS Error
 ---------------------------------------
    1    -764.0623605942      8.40E-04
    2    -763.9563024614      1.82E-04
    3    -763.9567195263      1.73E-04
    4    -763.9575464703      2.71E-05
    5    -763.9575713110      1.32E-05
    6    -763.9575787577      4.79E-06
    7    -763.9575800249      1.88E-06
    8    -763.9575802469      6.52E-07
    9    -763.9575802787      3.54E-07
   10    -763.9575802849      8.92E-08 Convergence criterion met
 ---------------------------------------
 SCF time:  CPU 354.95 s  wall 593.19 s
 SCF   energy in the final basis set = -763.95758028
 Total energy in the final basis set = -763.95758028

-------------------------------------------------
-  Entering anlman on Sun Oct 13 18:41:09 2024  -
-------------------------------------------------



 --------------------------------------------------------------

                    Orbital Energies (a.u.)
 --------------------------------------------------------------


 Alpha MOs
 -- Occupied --
-14.8127 -14.8038 -14.7233 -14.7194 -10.6774 -10.6695 -10.6546 -10.6358
-10.6344 -10.6217 -10.6116 -10.5788 -10.5677 -10.5663 -10.5479 -10.5301
-10.4989 -10.4527  -1.4256  -1.4000  -1.3444  -1.2755  -1.2302  -1.2015
 -1.1831  -1.1436  -1.1013  -1.0800  -1.0607  -1.0522  -1.0278  -1.0032
 -0.9879  -0.9504  -0.9441  -0.9047  -0.9003  -0.8900  -0.8809  -0.8619
 -0.8524  -0.8398  -0.8366  -0.8172  -0.8087  -0.7934  -0.7865  -0.7816
 -0.7727  -0.7679  -0.7504  -0.7459  -0.7319  -0.7245  -0.7188  -0.7104
 -0.6840  -0.6713  -0.6521  -0.6499  -0.6358  -0.6264  -0.6202  -0.6168
 -0.6135
 -- Virtual --
 -0.2747  -0.2666  -0.2512  -0.2196  -0.1760  -0.1634  -0.1467  -0.1437
 -0.1345  -0.1244  -0.1174  -0.1130  -0.1100  -0.1051  -0.0984  -0.0924
 -0.0905  -0.0874  -0.0801  -0.0766  -0.0745  -0.0740  -0.0721  -0.0673
 -0.0622  -0.0592  -0.0568  -0.0511  -0.0462  -0.0441  -0.0364  -0.0318
 -0.0284  -0.0246  -0.0205  -0.0177  -0.0170  -0.0139  -0.0106  -0.0073
 -0.0040  -0.0016   0.0014   0.0049   0.0065   0.0124   0.0139   0.0151
  0.0186   0.0229   0.0243   0.0271   0.0324   0.0330   0.0353   0.0394
  0.0420   0.0451   0.0509   0.0533   0.0560   0.0597   0.0641   0.0698
  0.0716   0.0728   0.0763   0.0775   0.0787   0.0860   0.0891   0.0953
  0.0976   0.0998   0.1040   0.1051   0.1073   0.1134   0.1181   0.1194
  0.1214   0.1253   0.1301   0.1334   0.1390   0.1410   0.1448   0.1494
  0.1512   0.1534   0.1617   0.1633   0.1669   0.1695   0.1744   0.1786
  0.1830   0.1840   0.1916   0.1948   0.1986   0.2025   0.2197   0.2268
  0.2366   0.2451   0.2539   0.2688   0.2815   0.2915   0.2995   0.3163
  0.3264   0.3318   0.3334   0.3474   0.3491   0.3548   0.3615   0.3661
  0.3719   0.3779   0.3834   0.3964   0.3999   0.4066   0.4136   0.4230
  0.4300   0.4352   0.4418   0.4494   0.4585   0.4640   0.4716   0.4787
  0.4830   0.4909   0.4943   0.4967   0.5033   0.5120   0.5147   0.5228
  0.5262   0.5294   0.5322   0.5384   0.5466   0.5543   0.5579   0.5607
  0.5654   0.5669   0.5702   0.5823   0.5832   0.5862   0.5946   0.5995
  0.6052   0.6191   0.6244   0.6274   0.6301   0.6429   0.6563   0.6588
  0.6720   0.6735   0.6784   0.6839   0.6902   0.7015   0.7076   0.7124
  0.7188   0.7335   0.7390   0.7454   0.7514   0.7569   0.7756   0.7969
  0.8004   0.8120   0.8161   0.8222   0.8281   0.8402   0.8419   0.8662
```

| | | | | | | | |
|---|---|---|---|---|---|---|---|
| 0.8698 | 0.8735 | 0.8830 | 0.8854 | 0.8978 | 0.9044 | 0.9168 | 0.9313 |
| 0.9378 | 0.9455 | 0.9628 | 0.9813 | 0.9896 | 1.0015 | 1.0247 | 1.0259 |
| 1.0527 | 1.0928 | 1.1044 | 1.1159 | 1.1333 | 1.1447 | 1.1619 | 1.1903 |
| 1.1978 | 1.2007 | 1.2370 | 1.2486 | 1.2655 | 1.2730 | 1.2953 | 1.3032 |
| 1.3103 | 1.3213 | 1.3380 | 1.3402 | 1.3471 | 1.3614 | 1.3675 | 1.3784 |
| 1.3836 | 1.3950 | 1.4000 | 1.4040 | 1.4134 | 1.4226 | 1.4299 | 1.4389 |
| 1.4425 | 1.4539 | 1.4606 | 1.4676 | 1.4700 | 1.4830 | 1.4861 | 1.4955 |
| 1.5018 | 1.5033 | 1.5099 | 1.5133 | 1.5175 | 1.5357 | 1.5460 | 1.5541 |
| 1.5588 | 1.5729 | 1.5742 | 1.5825 | 1.5942 | 1.6034 | 1.6132 | 1.6187 |
| 1.6260 | 1.6300 | 1.6374 | 1.6493 | 1.6666 | 1.6744 | 1.6881 | 1.6928 |
| 1.7003 | 1.7219 | 1.7276 | 1.7345 | 1.7440 | 1.7486 | 1.7657 | 1.7699 |
| 1.7871 | 1.7931 | 1.8146 | 1.8326 | 1.8499 | 1.8531 | 1.8620 | 1.8739 |
| 1.8883 | 1.8991 | 1.9079 | 1.9217 | 1.9413 | 1.9576 | 1.9633 | 1.9957 |
| 2.0070 | 2.0162 | 2.0273 | 2.0406 | 2.0492 | 2.0588 | 2.0832 | 2.0888 |
| 2.0939 | 2.1098 | 2.1223 | 2.1352 | 2.1467 | 2.1855 | 2.1932 | 2.2302 |
| 2.2378 | 2.2464 | 2.2498 | 2.2620 | 2.2792 | 2.2931 | 2.2980 | 2.3103 |
| 2.3170 | 2.3192 | 2.3516 | 2.3642 | 2.3732 | 2.3808 | 2.3897 | 2.4080 |
| 2.4195 | 2.4238 | 2.4280 | 2.4441 | 2.4545 | 2.4718 | 2.4775 | 2.4892 |
| 2.4933 | 2.5078 | 2.5193 | 2.5261 | 2.5331 | 2.5441 | 2.5466 | 2.5533 |
| 2.5669 | 2.5778 | 2.5881 | 2.5934 | 2.6011 | 2.6086 | 2.6120 | 2.6244 |
| 2.6444 | 2.6475 | 2.6520 | 2.6599 | 2.6770 | 2.6827 | 2.6859 | 2.6978 |
| 2.7144 | 2.7188 | 2.7285 | 2.7357 | 2.7406 | 2.7611 | 2.7651 | 2.7781 |
| 2.7823 | 2.8013 | 2.8092 | 2.8328 | 2.8382 | 2.8520 | 2.8748 | 2.8940 |
| 2.9115 | 2.9324 | 2.9446 | 2.9579 | 3.0548 | 3.0660 | 3.1043 | 3.1309 |
| 3.1396 | 3.1853 | 3.2527 | 3.2612 | 3.2893 | 3.3361 | 3.3470 | 3.3887 |
| 3.4164 | 3.4702 | 3.4972 | 3.5451 | 3.5573 | 3.6050 | 3.6336 | 3.6465 |
| 3.6619 | 3.6748 | 3.7094 | 3.7332 | 3.7354 | 3.7572 | 3.7762 | 3.7787 |
| 3.8154 | 3.8744 | 3.8998 | 3.9137 | 3.9492 | 4.0341 | 4.0492 | 4.0953 |
| 4.1053 | 4.1852 | 4.3022 | 4.3128 | 4.5856 | 4.6488 | 4.6687 | 4.7412 |
| 4.8849 | 5.0410 | 5.1676 | 23.5387 | 23.5604 | 23.7173 | 23.7231 | 23.7398 |
| 23.7729 | 23.8033 | 23.8302 | 23.8339 | 23.8966 | 23.9203 | 23.9613 | 23.9733 |
| 24.0442 | 35.4214 | 35.4452 | 35.4958 | 35.5887 | | | |

### Ground-State Mulliken Net Atomic Charges

| Atom | Charge (a.u.) |
|---|---|
| 1 N | 0.458529 |
| 2 C | 0.144587 |
| 3 C | -0.036364 |
| 4 C | -0.138504 |
| 5 C | -0.190278 |
| 6 C | -0.136818 |
| 7 C | -0.743052 |
| 8 C | -0.270145 |
| 9 N | -0.084541 |
| 10 C | -0.433842 |
| 11 N | 0.079721 |
| 12 C | 0.122541 |
| 13 C | -0.057688 |
| 14 C | 0.115086 |
| 15 C | -0.377715 |
| 16 C | -0.081527 |
| 17 C | -0.610024 |
| 18 N | -0.480932 |
| 19 H | 0.219948 |
| 20 H | 0.233560 |
| 21 H | 0.226696 |
| 22 H | 0.218811 |
| 23 H | 0.231982 |
| 24 H | 0.249122 |
| 25 H | 0.176990 |
| 26 H | 0.266651 |
| 27 H | 0.245378 |
| 28 H | 0.400074 |
| 29 H | 0.222660 |
| 30 H | 0.241076 |
| 31 H | 0.211220 |
| 32 H | 0.197504 |

```
    33 H                    0.171094
    34 H                    0.177829
    35 H                    0.198944
    36 H                    0.170236
    37 H                    0.298535
    38 H                    0.362658
 ----------------------------------------
 Sum of atomic charges =     2.000000

 -----------------------------------------------------------------
                    Cartesian Multipole Moments
 -----------------------------------------------------------------
    Charge (ESU x 10^10)
                 9.6064
    Dipole Moment (Debye)
         X       6.3214      Y      -1.8533      Z      -4.1550
       Tot       7.7884
    Quadrupole Moments (Debye-Ang)
        XX     -33.1282     XY      26.1410     YY     -65.1865
        XZ     -40.1924     YZ     -17.9598     ZZ     -48.5805
    Traceless Quadrupole Moments (Debye-Ang)
       QXX      47.5104    QYY     -48.6642    QZZ       1.1538
       QXY      78.4229    QXZ    -120.5772    QYZ     -53.8795
    Octopole Moments (Debye-Ang^2)
       XXX     -16.3174    XXY      34.6333    XYY       8.5082
       YYY     -16.3845    XXZ      28.7416    XYZ     -20.1392
       YYZ      22.6374    XZZ      26.4861    YZZ      25.9456
       ZZZ     -74.1134
    Traceless Octopole Moments (Debye-Ang^2)
       XXX    -412.8540    YYY    -643.5174    ZZZ    -907.0915
       XXY     386.9162    XXZ     499.3278    XYY      71.5927
       XYZ    -302.0886    XZZ     341.2613    YYZ     407.7638
       YZZ     256.6013
    Hexadecapole Moments (Debye-Ang^3)
      XXXX   -3363.0989   XXXY    -221.4613   XXYY    -663.5551
      XYYY    -447.4042   YYYY    -763.0177   XXXZ     926.7304
      XXYZ     -72.6168   XYYZ     343.4102   YYYZ     313.8620
      XXZZ    -787.1901   XYZZ       5.2128   YYZZ    -531.9930
      XZZZ     854.5751   YZZZ     199.0613   ZZZZ   -1931.8366
    Traceless Hexadecapole Moments (Debye-Ang^3)
      XXXX  -10090.2847   XXXY    6610.9331   XXXZ    1694.4846
      XXYY    1842.5764   XXYZ  -14228.4460   XXZZ    8247.7083
      XYYY  -17113.0640   XYYZ    4187.3386   XYZZ   10502.1309
      XZZZ   -5881.8232   YYYY    5943.1929   YYYZ   13144.4770
      YYZZ   -7785.7693   YZZZ    1083.9691   ZZZZ    -461.9390
 -----------------------------------------------------------------


--------------------------------------------------
-  Entering drvman on Sun Oct 13 18:41:09 2024  -
--------------------------------------------------

 Calculating analytic gradient of the SCF energy
 Gradient of SCF Energy
           1           2           3           4           5           6
    1  -0.0014398  -0.0026536   0.0026735  -0.0023564   0.0005494   0.0026438
    2  -0.0024465  -0.0025693   0.0020780  -0.0033885  -0.0002110   0.0019584
    3   0.0009232  -0.0018048   0.0040529   0.0009742   0.0017362  -0.0008738
           7           8           9          10          11          12
    1   0.0017286  -0.0009911  -0.0000762   0.0133097  -0.0073369   0.0018694
    2   0.0022509   0.0021614   0.0023289  -0.0075525   0.0081853  -0.0192070
    3  -0.0007130   0.0007238  -0.0022141   0.0001956   0.0039708   0.0011479
          13          14          15          16          17          18
    1  -0.0038336  -0.0029185  -0.0013768  -0.0004869   0.0010057   0.0128136
    2   0.0046794   0.0028431   0.0027339  -0.0010115   0.0003010   0.0047576
    3   0.0010643   0.0011175   0.0038862  -0.0048969   0.0003581  -0.0110551
          19          20          21          22          23          24
    1   0.0005136   0.0000183  -0.0001210  -0.0005559   0.0010365   0.0003572
    2   0.0014492   0.0004164   0.0008858  -0.0004852   0.0009415  -0.0005704
    3  -0.0020331  -0.0013672  -0.0016264  -0.0026111   0.0006599  -0.0008050
          25          26          27          28          29          30
```

```
    1  -0.0012166  -0.0014813   0.0010094  -0.0019579  -0.0005423  -0.0018317
    2   0.0003226  -0.0020312  -0.0003520  -0.0018594  -0.0007722  -0.0002540
    3  -0.0010608   0.0027156  -0.0026689   0.0010040   0.0013359  -0.0002846
           31          32          33          34          35          36
    1  -0.0001012  -0.0003167  -0.0001962  -0.0000636   0.0000915   0.0001448
    2  -0.0001001   0.0004703  -0.0000588   0.0002963   0.0000953  -0.0003815
    3  -0.0006691   0.0012572   0.0006756  -0.0004329   0.0006526  -0.0003142
           37          38
    1  -0.0061202  -0.0017908
    2   0.0035729   0.0005231
    3   0.0062066   0.0007728
 Max gradient component =       1.921E-02
 RMS gradient           =       3.542E-03
 Gradient time:  CPU 163.09 s  wall 270.71 s

--------------------------------------------------
-  Entering optman on Sun Oct 13 18:45:40 2024  -
--------------------------------------------------

 Geometry Optimization Parameters
   NAtoms,    NIC,     NZ,  NCons,   NDum,   NFix, NCnnct, MaxDiis
       38     272       0       0       0       0       0       0

 Cartesian Hessian Update
 Hessian updated using BFGS update


** GEOMETRY OPTIMIZATION IN DELOCALIZED INTERNAL COORDINATES **
   Searching for a Minimum

   Optimization Cycle:   2

                    Coordinates (Angstroms)
     ATOM              X               Y               Z
      1  N         2.2487038768    0.1327463258   -1.5667866716
      2  C         3.0912286076    1.1997273251   -1.6809009461
      3  C         3.2332354810    1.8065795545   -2.9216303747
      4  C         2.5441169256    1.3292275818   -4.0254284965
      5  C         1.7121811443    0.2244559513   -3.8866293495
      6  C         1.5916697554   -0.3510180129   -2.6513918203
      7  C         3.8760481522    1.7005877659   -0.5055929121
      8  C         2.0549072119   -0.5834161969   -0.2757507471
      9  N        -1.1971799107   -1.8983486318    1.0304092288
     10  C        -2.0053221715   -0.8163288888    0.9814423531
     11  N        -1.5487542692    0.3533672582    0.5853774168
     12  C        -0.2602380693    0.4561082667    0.2288062637
     13  C         0.6146414601   -0.6580912534    0.1857794381
     14  C         0.0896062756   -1.8383657165    0.6318531345
     15  C        -3.4139762776   -0.9483252049    1.4548531467
     16  C        -3.5089486992   -0.7381518491    2.9774272500
     17  C        -4.9490084311   -0.8556613781    3.4631789050
     18  N         0.1616057741    1.7091130962   -0.1595571723
     19  H         3.8969770117    2.6574747413   -3.0267658012
     20  H         2.6622056807    1.8080131173   -4.9929479002
     21  H         1.1721601610   -0.1815491205   -4.7332986481
     22  H         0.9643438935   -1.2208371932   -2.4996245733
     23  H         4.4832362972    2.5563671599   -0.7993113855
     24  H         4.5550441640    0.9305105161   -0.1276313113
     25  H         3.2251360217    2.0284426724    0.3093116263
     26  H         2.6597849835   -0.1159025258    0.4985966507
     27  H         2.4569522390   -1.5890334551   -0.4264810203
     28  H        -1.5849534913   -2.7740509756    1.3749727452
     29  H         0.6481338363   -2.7665144326    0.6889500775
     30  H        -4.0231660544   -0.2061022767    0.9344266369
     31  H        -3.8013428426   -1.9383323622    1.1888816117
     32  H        -3.1121396172    0.2495886876    3.2339456311
     33  H        -2.8859655425   -1.4763080923    3.4968656282
     34  H        -5.5867750425   -0.1070468988    2.9860681699
     35  H        -4.9966958297   -0.6999673785    4.5425039095
     36  H        -5.3616228299   -1.8446032826    3.2461910808
```

```
    37  H          0.9175064201    2.1074076078    0.3865878245
    38  H         -0.6233362944    2.3582374980   -0.1466995986
  Point Group: c1    Number of degrees of freedom:   108


  Energy is   -763.957580285

 Hessian updated using BFGS update
  internal optimization (0)

108 Hessian modes will be used to form the next step
  Hessian Eigenvalues:
     0.001807    0.002732    0.003599    0.004200    0.007156    0.010505
     0.012444    0.018921    0.019207    0.019922    0.021073    0.022034
     0.022564    0.023362    0.024042    0.024280    0.025220    0.026867
     0.027810    0.028205    0.028965    0.030742    0.035820    0.037184
     0.038339    0.040878    0.043037    0.043647    0.043843    0.046754
     0.048849    0.053710    0.055729    0.055777    0.067753    0.077053
     0.083911    0.095016    0.121696    0.122099    0.126858    0.127717
     0.130668    0.132794    0.134955    0.138424    0.143824    0.146387
     0.146866    0.147519    0.148632    0.150388    0.151659    0.152289
     0.153902    0.156258    0.179210    0.191511    0.206285    0.213546
     0.218208    0.229700    0.235044    0.238726    0.247727    0.255508
     0.266642    0.271734    0.280359    0.286334    0.294900    0.298416
     0.300145    0.300604    0.301100    0.302443    0.302612    0.304116
     0.304822    0.304906    0.304970    0.306420    0.309685    0.310891
     0.319217    0.329680    0.330693    0.332760    0.334679    0.339372
     0.340978    0.348253    0.355273    0.361812    0.378913    0.383812
     0.392493    0.400182    0.404226    0.413684    0.420005    0.423731
     0.433517    0.441059    0.454528    0.514311    0.647736    0.723309

 Minimum search - taking simple RFO step
 Searching for Lamda that Minimizes Along All modes
 Value Taken    Lamda =  -0.00942905
 Calculated Step too Large.  Step scaled by  0.502918 !!
 Step Taken.  Stepsize is  0.300000  0.002763

                         Maximum     Tolerance    Cnvgd?
        Gradient         0.011306     0.000800      NO
        Displacement     0.104457     0.001400      NO
        Energy change   -0.010892     0.000228      NO


 New Cartesian Coordinates Obtained by Inverse Iteration

 Displacement from previous Coordinates is:  0.445477
 ----------------------------------------------------------------
             Standard Nuclear Orientation (Angstroms)
    I     Atom           X                Y                Z
 ----------------------------------------------------------------
    1      N       2.2481147142     0.1449339516    -1.5520350403
    2      C       3.1028408483     1.1980266035    -1.6767579593
    3      C       3.2359229916     1.7934180168    -2.9241920640
    4      C       2.5308574880     1.3225983411    -4.0188834221
    5      C       1.6847898161     0.2278742956    -3.8632719092
    6      C       1.5698087897    -0.3381304948    -2.6227652524
    7      C       3.9048602469     1.6839125683    -0.5086087164
    8      C       2.0566811149    -0.5612239041    -0.2590836012
    9      N      -1.1948116148    -1.8939215342     1.0202486355
   10      C      -2.0167707156    -0.8171002482     0.9721769573
   11      N      -1.5561050000     0.3511912232     0.5831238198
   12      C      -0.2684831049     0.4801693352     0.2425748698
   13      C       0.6134944459    -0.6374575910     0.1924831933
   14      C       0.0945057617    -1.8252934363     0.6241802649
   15      C      -3.4197045306    -0.9629846479     1.4441707781
   16      C      -3.5126004005    -0.7479707907     2.9714688143
   17      C      -4.9526590464    -0.8682825012     3.4591017101
   18      N       0.1283413082     1.7220448021    -0.1566739613
   19      H       3.9119657207     2.6321916352    -3.0314271274
   20      H       2.6459844941     1.7943137987    -4.9881428860
```

```
  21      H       1.1287041197    -0.1823360610    -4.6958651702
  22      H       0.9347321612    -1.1966201878    -2.4471953320
  23      H       4.5176066509     2.5291990035    -0.8154852169
  24      H       4.5785851483     0.9074218372    -0.1353070846
  25      H       3.2762576563     2.0227582752     0.3192906014
  26      H       2.6617065292    -0.0776351882     0.5040375336
  27      H       2.4595304360    -1.5677558681    -0.3892253008
  28      H      -1.5731393959    -2.7740890218     1.3564488027
  29      H       0.6599281739    -2.7494441938     0.6676934506
  30      H      -4.0281430254    -0.2212372477     0.9250342344
  31      H      -3.8027877212    -1.9552954919     1.1831629330
  32      H      -3.1161888389     0.2416573891     3.2178933326
  33      H      -2.8842283900    -1.4825445690     3.4887618179
  34      H      -5.5934716028    -0.1233795905     2.9815329135
  35      H      -4.9991382304    -0.7085430414     4.5370773932
  36      H      -5.3639703590    -1.8584139441     3.2477258446
  37      H       0.9700446438     2.1066785298     0.2497067396
  38      H      -0.6330612830     2.3912699476    -0.1029745960
 ----------------------------------------------------------------
 Nuclear Repulsion Energy =        1301.78603095 hartrees
 There are       65 alpha and       65 beta electrons

--------------------------------------------------
-  Entering fldman on Sun Oct 13 18:45:40 2024  -
--------------------------------------------------

 Applying Cartesian multipole field
    Component          Value
    ---------          -----
     (2,0,0)        1.00000E-12
     (0,2,0)        2.00000E-11
     (0,0,2)       -3.00000E-11
 Nucleus-field energy     =    -0.0000000463 hartrees

--------------------------------------------------
-  Entering gesman on Sun Oct 13 18:45:40 2024  -
--------------------------------------------------

 Requested basis set is 6-311+G(d,p)
 There are 188 shells and 516 basis functions
 A cutoff of  1.0D-12 yielded  12482 shell pairs
 There are    100056 function pairs (    106706 Cartesian)
 Smallest overlap matrix eigenvalue = 1.35E-06
 Linear dependence detected in AO basis
 Tighter screening thresholds may be required for diffuse basis sets
 Use S2THRESH > 12 and THRESH = 14 in case of SCF convergence issues
 Number of orthogonalized atomic orbitals = 510
 Maximum deviation from orthogonality = 1.851E-11
 Guess MOs from SCF MO coefficient file
 Reading MOs from coefficient file
 Reading MOs from coefficient file

--------------------------------------------------
-  Entering scfman on Sun Oct 13 18:45:40 2024  -
--------------------------------------------------

 Long-range K will be added via erf
 Coulomb attenuation parameter = 0.2 bohr**(-1)
 A restricted hybrid HF-DFT SCF calculation will be
 performed using Pulay DIIS + Geometric Direct Minimization
 Exchange:     0.2220 Hartree-Fock + 1.0000 wB97X-D + LR-HF
 Correlation:  1.0000 wB97X-D
 Using Euler-Maclaurin-Lebedev (75,302) quadrature formula
 Dispersion:   Grimme D
 SCF converges when RMS gradient is below 1.0E-07
 Geometry optimization detected.  Setting ReadMinima to 0
 Setting SaveMinima to 0
 ---------------------------------------
  Cycle       Energy         DIIS Error
 ---------------------------------------
```

```
    1    -764.0017976657      4.66E-04
    2    -763.9609104467      1.28E-04
    3    -763.9610802333      1.30E-04
    4    -763.9615704575      1.74E-05
    5    -763.9615833615      1.02E-05
    6    -763.9615871780      3.61E-06
    7    -763.9615878666      1.31E-06
    8    -763.9615879543      4.75E-07
    9    -763.9615879690      1.78E-07
   10    -763.9615879710      5.62E-08 Convergence criterion met
 ---------------------------------------
 SCF time:  CPU 352.77 s  wall 586.69 s
 SCF   energy in the final basis set = -763.96158797
 Total energy in the final basis set = -763.96158797

--------------------------------------------------
-  Entering anlman on Sun Oct 13 18:55:27 2024  -
--------------------------------------------------



 --------------------------------------------------------------

                   Orbital Energies (a.u.)
 --------------------------------------------------------------

 Alpha MOs
 -- Occupied --
-14.8136 -14.7992 -14.7286 -14.7155 -10.6775 -10.6680 -10.6519 -10.6376
-10.6335 -10.6228 -10.6097 -10.5808 -10.5698 -10.5681 -10.5499 -10.5289
-10.4986 -10.4520  -1.4221  -1.4027  -1.3457  -1.2763  -1.2305  -1.2016
 -1.1856  -1.1430  -1.1005  -1.0824  -1.0584  -1.0530  -1.0299  -1.0026
 -0.9893  -0.9513  -0.9428  -0.9060  -0.9028  -0.8918  -0.8821  -0.8631
 -0.8540  -0.8407  -0.8372  -0.8182  -0.8094  -0.7939  -0.7884  -0.7821
 -0.7726  -0.7667  -0.7530  -0.7476  -0.7367  -0.7263  -0.7186  -0.7125
 -0.6836  -0.6702  -0.6534  -0.6472  -0.6351  -0.6249  -0.6196  -0.6172
 -0.6133
 -- Virtual --
 -0.2705  -0.2679  -0.2496  -0.2215  -0.1754  -0.1629  -0.1460  -0.1434
 -0.1348  -0.1241  -0.1173  -0.1131  -0.1096  -0.1042  -0.0982  -0.0929
 -0.0905  -0.0876  -0.0806  -0.0769  -0.0751  -0.0739  -0.0724  -0.0677
 -0.0618  -0.0589  -0.0571  -0.0510  -0.0462  -0.0443  -0.0359  -0.0313
 -0.0277  -0.0249  -0.0202  -0.0178  -0.0172  -0.0149  -0.0115  -0.0069
 -0.0038  -0.0014   0.0018   0.0050   0.0059   0.0122   0.0132   0.0151
  0.0190   0.0231   0.0245   0.0275   0.0319   0.0325   0.0361   0.0397
  0.0417   0.0453   0.0515   0.0540   0.0562   0.0596   0.0640   0.0700
  0.0716   0.0725   0.0755   0.0778   0.0789   0.0863   0.0905   0.0953
  0.0976   0.1004   0.1036   0.1049   0.1072   0.1134   0.1176   0.1190
  0.1209   0.1255   0.1295   0.1345   0.1389   0.1417   0.1455   0.1498
  0.1515   0.1544   0.1617   0.1650   0.1672   0.1701   0.1726   0.1762
  0.1830   0.1843   0.1923   0.1943   0.1996   0.2027   0.2184   0.2265
  0.2351   0.2446   0.2517   0.2687   0.2815   0.2906   0.2986   0.3166
  0.3241   0.3331   0.3349   0.3461   0.3499   0.3546   0.3618   0.3644
  0.3726   0.3789   0.3845   0.3973   0.3985   0.4064   0.4123   0.4250
  0.4299   0.4364   0.4401   0.4502   0.4592   0.4644   0.4724   0.4770
  0.4841   0.4915   0.4940   0.4992   0.5039   0.5102   0.5170   0.5222
  0.5277   0.5297   0.5318   0.5382   0.5462   0.5536   0.5574   0.5609
  0.5633   0.5662   0.5700   0.5823   0.5842   0.5869   0.5934   0.5989
  0.6055   0.6190   0.6252   0.6262   0.6302   0.6430   0.6563   0.6593
  0.6705   0.6733   0.6788   0.6820   0.6892   0.6997   0.7042   0.7106
  0.7263   0.7335   0.7394   0.7451   0.7553   0.7619   0.7764   0.7926
  0.8030   0.8118   0.8164   0.8242   0.8248   0.8363   0.8441   0.8675
  0.8684   0.8738   0.8815   0.8860   0.9017   0.9044   0.9239   0.9339
  0.9374   0.9436   0.9606   0.9786   0.9875   1.0003   1.0210   1.0274
  1.0508   1.0986   1.1028   1.1175   1.1338   1.1461   1.1629   1.1902
  1.1955   1.1988   1.2371   1.2471   1.2623   1.2732   1.2925   1.3038
  1.3123   1.3240   1.3359   1.3400   1.3432   1.3611   1.3679   1.3790
  1.3813   1.3950   1.3987   1.4028   1.4124   1.4203   1.4249   1.4361
  1.4426   1.4534   1.4552   1.4647   1.4718   1.4847   1.4905   1.4947
  1.5028   1.5046   1.5079   1.5117   1.5179   1.5371   1.5470   1.5528
  1.5635   1.5738   1.5747   1.5835   1.5950   1.6058   1.6134   1.6214
```

| | | | | | | | |
|---|---|---|---|---|---|---|---|
| 1.6264 | 1.6312 | 1.6403 | 1.6479 | 1.6653 | 1.6748 | 1.6904 | 1.6949 |
| 1.7105 | 1.7244 | 1.7286 | 1.7373 | 1.7445 | 1.7502 | 1.7660 | 1.7690 |
| 1.7867 | 1.7904 | 1.8174 | 1.8290 | 1.8476 | 1.8526 | 1.8664 | 1.8742 |
| 1.8959 | 1.9021 | 1.9066 | 1.9240 | 1.9409 | 1.9528 | 1.9609 | 1.9928 |
| 2.0055 | 2.0109 | 2.0280 | 2.0419 | 2.0478 | 2.0619 | 2.0839 | 2.0865 |
| 2.0921 | 2.1120 | 2.1263 | 2.1368 | 2.1454 | 2.1823 | 2.1956 | 2.2331 |
| 2.2391 | 2.2467 | 2.2505 | 2.2625 | 2.2823 | 2.2903 | 2.2963 | 2.3100 |
| 2.3127 | 2.3194 | 2.3512 | 2.3670 | 2.3735 | 2.3810 | 2.3892 | 2.4103 |
| 2.4174 | 2.4243 | 2.4293 | 2.4439 | 2.4561 | 2.4671 | 2.4773 | 2.4882 |
| 2.4922 | 2.5087 | 2.5209 | 2.5264 | 2.5346 | 2.5446 | 2.5479 | 2.5513 |
| 2.5694 | 2.5811 | 2.5884 | 2.5929 | 2.5979 | 2.6081 | 2.6105 | 2.6278 |
| 2.6468 | 2.6485 | 2.6555 | 2.6659 | 2.6761 | 2.6862 | 2.6883 | 2.6991 |
| 2.7121 | 2.7224 | 2.7287 | 2.7309 | 2.7430 | 2.7612 | 2.7693 | 2.7772 |
| 2.7877 | 2.8041 | 2.8083 | 2.8355 | 2.8422 | 2.8537 | 2.8783 | 2.8936 |
| 2.9175 | 2.9275 | 2.9431 | 2.9582 | 3.0597 | 3.0664 | 3.1284 | 3.1359 |
| 3.1443 | 3.1833 | 3.2527 | 3.2600 | 3.2927 | 3.3361 | 3.3508 | 3.3921 |
| 3.4167 | 3.4716 | 3.5016 | 3.5494 | 3.5611 | 3.6057 | 3.6323 | 3.6445 |
| 3.6656 | 3.6766 | 3.7138 | 3.7349 | 3.7374 | 3.7604 | 3.7745 | 3.7816 |
| 3.8207 | 3.8741 | 3.9016 | 3.9133 | 3.9417 | 4.0072 | 4.0486 | 4.0877 |
| 4.1043 | 4.1826 | 4.2993 | 4.3037 | 4.5996 | 4.6543 | 4.6796 | 4.7419 |
| 4.8945 | 5.0432 | 5.1813 | 23.5407 | 23.5660 | 23.7180 | 23.7268 | 23.7410 |
| 23.7694 | 23.8011 | 23.8302 | 23.8357 | 23.8955 | 23.9223 | 23.9619 | 23.9742 |
| 24.0418 | 35.4223 | 35.4473 | 35.5010 | 35.5972 | | | |

Ground-State Mulliken Net Atomic Charges

| Atom | Charge (a.u.) |
|---|---|
| 1 N | 0.438321 |
| 2 C | 0.149603 |
| 3 C | -0.036698 |
| 4 C | -0.139739 |
| 5 C | -0.186748 |
| 6 C | -0.133436 |
| 7 C | -0.752212 |
| 8 C | -0.278322 |
| 9 N | -0.091859 |
| 10 C | -0.430329 |
| 11 N | 0.068082 |
| 12 C | 0.151437 |
| 13 C | -0.069368 |
| 14 C | 0.125103 |
| 15 C | -0.381476 |
| 16 C | -0.078317 |
| 17 C | -0.610387 |
| 18 N | -0.463631 |
| 19 H | 0.219605 |
| 20 H | 0.234470 |
| 21 H | 0.227182 |
| 22 H | 0.219985 |
| 23 H | 0.232074 |
| 24 H | 0.250785 |
| 25 H | 0.179060 |
| 26 H | 0.265923 |
| 27 H | 0.245721 |
| 28 H | 0.398104 |
| 29 H | 0.221263 |
| 30 H | 0.240478 |
| 31 H | 0.211559 |
| 32 H | 0.197081 |
| 33 H | 0.171762 |
| 34 H | 0.177718 |
| 35 H | 0.198760 |
| 36 H | 0.170719 |
| 37 H | 0.282995 |
| 38 H | 0.374729 |
| Sum of atomic charges = | 2.000000 |

```
 ----------------------------------------------------------------
                 Cartesian Multipole Moments
 ----------------------------------------------------------------
    Charge (ESU x 10^10)
                 9.6064
    Dipole Moment (Debye)
         X       6.5248      Y      -1.5609      Z      -4.3441
       Tot       7.9925
    Quadrupole Moments (Debye-Ang)
        XX     -32.5005     XY      26.4157     YY     -65.1868
        XZ     -40.1960     YZ     -18.1240     ZZ     -49.1366
    Traceless Quadrupole Moments (Debye-Ang)
       QXX      49.3224    QYY     -48.7365    QZZ      -0.5859
       QXY      79.2472    QXZ    -120.5880    QYZ     -54.3719
    Octopole Moments (Debye-Ang^2)
       XXX     -11.9036    XXY      34.8906    XYY       8.5354
       YYY     -15.3169    XXZ      28.3104    XYZ     -20.6020
       YYZ      21.8564    XZZ      26.2484    YZZ      25.8225
       ZZZ     -74.3351
    Traceless Octopole Moments (Debye-Ang^2)
       XXX    -384.4766    YYY    -638.3196    ZZZ    -897.5125
       XXY     387.1707    XXZ     497.1611    XYY      59.3907
       XYZ    -309.0302    XZZ     325.0859    YYZ     400.3514
       YZZ     251.1489
    Hexadecapole Moments (Debye-Ang^3)
      XXXX   -3352.6276   XXXY    -218.3596   XXYY    -664.9735
      XYYY    -452.8759   YYYY    -759.4062   XXXZ     916.3171
      XXYZ     -73.1783   XYYZ     341.9240   YYYZ     316.2681
      XXZZ    -786.5245   XYZZ       4.2213   YYZZ    -531.4155
      XZZZ     852.9202   YZZZ     199.4162   ZZZZ   -1920.4102
    Traceless Hexadecapole Moments (Debye-Ang^3)
      XXXX   -9639.0370   XXXY    7087.8794   XXXZ    1211.0363
      XXYY    1581.7828   XXYZ  -14315.0695   XXZZ    8057.2543
      XYYY  -17536.3287   XYYZ    4234.6024   XYZZ   10448.4493
      XZZZ   -5445.6387   YYYY    6299.4784   YYYZ   13314.1074
      YYZZ   -7881.2612   YZZZ    1000.9621   ZZZZ    -175.9931
 ----------------------------------------------------------------

------------------------------------------------
-  Entering drvman on Sun Oct 13 18:55:27 2024  -
------------------------------------------------

 Calculating analytic gradient of the SCF energy
 Gradient of SCF Energy
           1           2           3           4           5           6
    1  -0.0001389  -0.0014275   0.0008646  -0.0005163   0.0000697   0.0012257
    2  -0.0002372  -0.0006092   0.0004764  -0.0004585  -0.0004892   0.0003021
    3   0.0010955  -0.0004543   0.0016055  -0.0001317   0.0010838   0.0000546
           7           8           9          10          11          12
    1   0.0006323   0.0003755   0.0004988   0.0036967  -0.0009864  -0.0004706
    2   0.0007687   0.0009797   0.0022460  -0.0046002   0.0032332  -0.0087697
    3  -0.0002949  -0.0013563  -0.0015710   0.0003964   0.0016002   0.0051489
          13          14          15          16          17          18
    1  -0.0046159  -0.0006109  -0.0003660  -0.0001041   0.0001959   0.0095212
    2   0.0037584  -0.0011175   0.0014823  -0.0004805   0.0001098   0.0027698
    3  -0.0007873   0.0011077   0.0016083  -0.0022410   0.0004842  -0.0111944
          19          20          21          22          23          24
    1   0.0000186  -0.0000595   0.0000246  -0.0000652   0.0000559   0.0001939
    2   0.0003013  -0.0000623   0.0004279  -0.0001498  -0.0000266  -0.0001806
    3  -0.0007084  -0.0002623  -0.0003848  -0.0014848   0.0001281  -0.0001867
          25          26          27          28          29          30
    1  -0.0005336  -0.0005943   0.0000226  -0.0008806  -0.0001782  -0.0004475
    2   0.0000801  -0.0012917   0.0000967  -0.0003781  -0.0003981  -0.0004030
    3  -0.0006942   0.0010868  -0.0015101   0.0004175   0.0008545  -0.0001000
          31          32          33          34          35          36
    1  -0.0002179  -0.0001495  -0.0000416   0.0000066   0.0000382   0.0000384
    2  -0.0000932   0.0001213  -0.0000024   0.0000388   0.0000320  -0.0000592
    3  -0.0002676   0.0006599   0.0004587  -0.0001485   0.0000539  -0.0001223
          37          38
    1  -0.0050236  -0.0000507
```

```
    2   0.0033226  -0.0007400
    3   0.0055837   0.0004724
 Max gradient component =       1.119E-02
 RMS gradient           =       2.155E-03
 Gradient time:  CPU 156.67 s  wall 260.45 s

-------------------------------------------------
-  Entering optman on Sun Oct 13 18:59:47 2024  -
-------------------------------------------------

 Geometry Optimization Parameters
   NAtoms,    NIC,     NZ,  NCons,   NDum,   NFix, NCnnct, MaxDiis
       38     272       0       0       0       0       0       0

 Cartesian Hessian Update
 Hessian updated using BFGS update


** GEOMETRY OPTIMIZATION IN DELOCALIZED INTERNAL COORDINATES **
   Searching for a Minimum

   Optimization Cycle:   3

                       Coordinates (Angstroms)
     ATOM                X               Y               Z
      1  N         2.2481147142    0.1449339516   -1.5520350403
      2  C         3.1028408483    1.1980266035   -1.6767579593
      3  C         3.2359229916    1.7934180168   -2.9241920640
      4  C         2.5308574880    1.3225983411   -4.0188834221
      5  C         1.6847898161    0.2278742956   -3.8632719092
      6  C         1.5698087897   -0.3381304948   -2.6227652524
      7  C         3.9048602469    1.6839125683   -0.5086087164
      8  C         2.0566811149   -0.5612239041   -0.2590836012
      9  N        -1.1948116148   -1.8939215342    1.0202486355
     10  C        -2.0167707156   -0.8171002482    0.9721769573
     11  N        -1.5561050000    0.3511912232    0.5831238198
     12  C        -0.2684831049    0.4801693352    0.2425748698
     13  C         0.6134944459   -0.6374575910    0.1924831933
     14  C         0.0945057617   -1.8252934363    0.6241802649
     15  C        -3.4197045306   -0.9629846479    1.4441707781
     16  C        -3.5126004005   -0.7479707907    2.9714688143
     17  C        -4.9526590464   -0.8682825012    3.4591017101
     18  N         0.1283413082    1.7220448021   -0.1566739613
     19  H         3.9119657207    2.6321916352   -3.0314271274
     20  H         2.6459844941    1.7943137987   -4.9881428860
     21  H         1.1287041197   -0.1823360610   -4.6958651702
     22  H         0.9347321612   -1.1966201878   -2.4471953320
     23  H         4.5176066509    2.5291990035   -0.8154852169
     24  H         4.5785851483    0.9074218372   -0.1353070846
     25  H         3.2762576563    2.0227582752    0.3192906014
     26  H         2.6617065292   -0.0776351882    0.5040375336
     27  H         2.4595304360   -1.5677558681   -0.3892253008
     28  H        -1.5731393959   -2.7740890218    1.3564488027
     29  H         0.6599281739   -2.7494441938    0.6676934506
     30  H        -4.0281430254   -0.2212372477    0.9250342344
     31  H        -3.8027877212   -1.9552954919    1.1831629330
     32  H        -3.1161888389    0.2416573891    3.2178933326
     33  H        -2.8842283900   -1.4825445690    3.4887618179
     34  H        -5.5934716028   -0.1233795905    2.9815329135
     35  H        -4.9991382304   -0.7085430414    4.5370773932
     36  H        -5.3639703590   -1.8584139441    3.2477258446
     37  H         0.9700446438    2.1066785298    0.2497067396
     38  H        -0.6330612830    2.3912699476   -0.1029745960
   Point Group: c1    Number of degrees of freedom:   108


   Energy is   -763.961587971

 Hessian updated using BFGS update
  internal optimization (0)
```

```
108 Hessian modes will be used to form the next step
  Hessian Eigenvalues:
     0.001806    0.002732    0.003588    0.004169    0.006652    0.009182
     0.012523    0.018933    0.019211    0.019968    0.021050    0.022034
     0.022590    0.023427    0.024026    0.024235    0.025257    0.026862
     0.028062    0.028196    0.028823    0.030524    0.035327    0.036319
     0.038295    0.040929    0.042889    0.043583    0.043838    0.046812
     0.049013    0.053597    0.053949    0.055806    0.067972    0.077281
     0.083918    0.095104    0.122099    0.122395    0.126857    0.128358
     0.131615    0.132777    0.135943    0.139453    0.143626    0.145261
     0.146892    0.147513    0.148573    0.151658    0.152122    0.152481
     0.153896    0.157363    0.191355    0.192411    0.206465    0.213910
     0.218090    0.230511    0.238684    0.247302    0.248779    0.255403
     0.271639    0.272389    0.282044    0.286376    0.296123    0.299047
     0.300138    0.300603    0.301235    0.302437    0.303288    0.304125
     0.304872    0.304905    0.305498    0.306397    0.309633    0.311773
     0.318797    0.330093    0.332364    0.333023    0.334787    0.339371
     0.340990    0.347269    0.358030    0.361696    0.378627    0.384359
     0.391816    0.402460    0.404882    0.415502    0.417039    0.426997
     0.433906    0.441385    0.455415    0.517298    0.649973    0.727871

 Minimum search - taking simple RFO step
 Searching for Lamda that Minimizes Along All modes
 Value Taken    Lamda =  -0.00715206
 Calculated Step too Large.  Step scaled by  0.458149 !!
 Step Taken.  Stepsize is  0.300000  0.002810

                              Maximum     Tolerance    Cnvgd?
         Gradient            0.005044      0.000800      NO
         Displacement        0.118074      0.001400      NO
         Energy change      -0.004008      0.000228      NO


 New Cartesian Coordinates Obtained by Inverse Iteration

 Displacement from previous Coordinates is:  0.439297
 ----------------------------------------------------------------
             Standard Nuclear Orientation (Angstroms)
    I     Atom           X                Y                Z
 ----------------------------------------------------------------
    1      N       2.2479147276     0.1575471438    -1.5406169822
    2      C       3.1192100515     1.1949381258    -1.6780289041
    3      C       3.2493542961     1.7776825015    -2.9314611239
    4      C       2.5282243451     1.3102320675    -4.0167825085
    5      C       1.6646354587     0.2301748617    -3.8464630616
    6      C       1.5507676979    -0.3230388625    -2.6001609135
    7      C       3.9388241911     1.6710888085    -0.5179376627
    8      C       2.0557569021    -0.5354610462    -0.2397963552
    9      N      -1.1956365209    -1.8892889447     1.0104018634
   10      C      -2.0265195413    -0.8153634168     0.9683377890
   11      N      -1.5700539271     0.3518480363     0.5865514996
   12      C      -0.2826740938     0.4969640211     0.2505913256
   13      C       0.6107675879    -0.6193486900     0.1962378714
   14      C       0.0946470678    -1.8089944974     0.6142636314
   15      C      -3.4265567405    -0.9743715857     1.4376443685
   16      C      -3.5180699466    -0.7571654117     2.9676312636
   17      C      -4.9569015917    -0.8847115513     3.4571066921
   18      N       0.0908761582     1.7347423953    -0.1354643498
   19      H       3.9389413658     2.6036319936    -3.0463466152
   20      H       2.6439137790     1.7735713942    -4.9895981158
   21      H       1.0940996840    -0.1802056306    -4.6686229398
   22      H       0.9034712466    -1.1672718079    -2.4033235314
   23      H       4.5628244715     2.5038755394    -0.8357579942
   24      H       4.6023513280     0.8849438035    -0.1472371677
   25      H       3.3293332664     2.0245812216     0.3186444749
   26      H       2.6542202193    -0.0320129781     0.5153817120
   27      H       2.4673315347    -1.5405679283    -0.3488655288
   28      H      -1.5655823620    -2.7752653948     1.3369978568
   29      H       0.6653357082    -2.7301215817     0.6459900183
```

```
   30      H      -4.0384095277    -0.2344355390     0.9203476482
   31      H      -3.8024866312    -1.9692563751     1.1782636253
   32      H      -3.1254841402     0.2351965949     3.2074152014
   33      H      -2.8833141372    -1.4874822367     3.4824083949
   34      H      -5.6027899592    -0.1443026490     2.9798261256
   35      H      -5.0026010331    -0.7230151154     4.5346935035
   36      H      -5.3634819955    -1.8773334357     3.2492527148
   37      H       0.9952011889     2.0957186145     0.1266854991
   38      H      -0.6474401281     2.4222775554    -0.0382093252
 ----------------------------------------------------------------
 Nuclear Repulsion Energy =        1301.97228676 hartrees
 There are       65 alpha and       65 beta electrons

--------------------------------------------------
-  Entering fldman on Sun Oct 13 18:59:47 2024  -
--------------------------------------------------

 Applying Cartesian multipole field
    Component          Value
    ---------          -----
     (2,0,0)        1.00000E-12
     (0,2,0)        2.00000E-11
     (0,0,2)       -3.00000E-11
 Nucleus-field energy     =    -0.0000000461 hartrees

--------------------------------------------------
-  Entering gesman on Sun Oct 13 18:59:47 2024  -
--------------------------------------------------

 Requested basis set is 6-311+G(d,p)
 There are 188 shells and 516 basis functions
 A cutoff of  1.0D-12 yielded  12486 shell pairs
 There are    100125 function pairs (    106773 Cartesian)
 Smallest overlap matrix eigenvalue = 1.32E-06
 Linear dependence detected in AO basis
 Tighter screening thresholds may be required for diffuse basis sets
 Use S2THRESH > 12 and THRESH = 14 in case of SCF convergence issues
 Number of orthogonalized atomic orbitals = 510
 Maximum deviation from orthogonality = 1.500E-11
 Guess MOs from SCF MO coefficient file
 Reading MOs from coefficient file
 Reading MOs from coefficient file

--------------------------------------------------
-  Entering scfman on Sun Oct 13 18:59:48 2024  -
--------------------------------------------------

 Long-range K will be added via erf
 Coulomb attenuation parameter = 0.2 bohr**(-1)
 A restricted hybrid HF-DFT SCF calculation will be
 performed using Pulay DIIS + Geometric Direct Minimization
 Exchange:     0.2220 Hartree-Fock + 1.0000 wB97X-D + LR-HF
 Correlation:  1.0000 wB97X-D
 Using Euler-Maclaurin-Lebedev (75,302) quadrature formula
 Dispersion:   Grimme D
 SCF converges when RMS gradient is below 1.0E-07
 Geometry optimization detected.  Setting ReadMinima to 0
 Setting SaveMinima to 0
 ---------------------------------------
  Cycle       Energy         DIIS Error
 ---------------------------------------
    1    -763.9968138670      4.53E-04
    2    -763.9638255790      1.17E-04
    3    -763.9639778202      1.19E-04
    4    -763.9643990251      1.72E-05
    5    -763.9644120488      9.17E-06
    6    -763.9644151913      3.52E-06
    7    -763.9644157924      1.26E-06
    8    -763.9644158711      4.22E-07
    9    -763.9644158827      1.61E-07
```

```
   10    -763.9644158842       5.19E-08 Convergence criterion met
 ---------------------------------------
 SCF time:  CPU 357.41 s  wall 599.27 s
 SCF   energy in the final basis set = -763.96441588
 Total energy in the final basis set = -763.96441588

--------------------------------------------------
-  Entering anlman on Sun Oct 13 19:09:47 2024  -
--------------------------------------------------



 --------------------------------------------------------------

                   Orbital Energies (a.u.)
 --------------------------------------------------------------

 Alpha MOs
 -- Occupied --
-14.8149 -14.7950 -14.7335 -14.7122 -10.6778 -10.6659 -10.6494 -10.6393
-10.6336 -10.6242 -10.6079 -10.5827 -10.5716 -10.5699 -10.5514 -10.5273
-10.4977 -10.4511  -1.4197  -1.4044  -1.3475  -1.2772  -1.2312  -1.2019
 -1.1874  -1.1425  -1.1000  -1.0840  -1.0568  -1.0532  -1.0314  -1.0018
 -0.9904  -0.9528  -0.9419  -0.9084  -0.9042  -0.8923  -0.8832  -0.8642
 -0.8553  -0.8416  -0.8376  -0.8190  -0.8093  -0.7949  -0.7900  -0.7820
 -0.7715  -0.7654  -0.7555  -0.7488  -0.7409  -0.7281  -0.7191  -0.7126
 -0.6828  -0.6694  -0.6548  -0.6460  -0.6339  -0.6237  -0.6199  -0.6167
 -0.6123
 -- Virtual --
 -0.2702  -0.2656  -0.2473  -0.2232  -0.1750  -0.1626  -0.1458  -0.1432
 -0.1353  -0.1238  -0.1175  -0.1133  -0.1094  -0.1033  -0.0983  -0.0938
 -0.0903  -0.0877  -0.0811  -0.0781  -0.0754  -0.0742  -0.0724  -0.0679
 -0.0614  -0.0585  -0.0573  -0.0510  -0.0462  -0.0444  -0.0360  -0.0311
 -0.0278  -0.0253  -0.0200  -0.0181  -0.0172  -0.0156  -0.0122  -0.0067
 -0.0044  -0.0013   0.0019   0.0046   0.0062   0.0121   0.0122   0.0152
  0.0192   0.0229   0.0244   0.0278   0.0311   0.0327   0.0364   0.0399
  0.0416   0.0451   0.0514   0.0547   0.0557   0.0599   0.0637   0.0697
  0.0709   0.0721   0.0758   0.0781   0.0792   0.0866   0.0919   0.0949
  0.0978   0.1008   0.1030   0.1043   0.1070   0.1134   0.1171   0.1189
  0.1209   0.1255   0.1289   0.1354   0.1382   0.1422   0.1461   0.1498
  0.1517   0.1554   0.1601   0.1656   0.1665   0.1688   0.1723   0.1746
  0.1848   0.1858   0.1929   0.1951   0.2005   0.2040   0.2182   0.2261
  0.2319   0.2424   0.2517   0.2690   0.2818   0.2901   0.2993   0.3151
  0.3228   0.3333   0.3369   0.3454   0.3509   0.3551   0.3613   0.3634
  0.3718   0.3798   0.3856   0.3972   0.3979   0.4061   0.4116   0.4277
  0.4299   0.4355   0.4390   0.4512   0.4602   0.4652   0.4725   0.4757
  0.4847   0.4912   0.4948   0.5008   0.5055   0.5082   0.5194   0.5225
  0.5277   0.5290   0.5314   0.5390   0.5452   0.5538   0.5571   0.5602
  0.5617   0.5667   0.5698   0.5822   0.5857   0.5871   0.5925   0.5997
  0.6056   0.6188   0.6257   0.6276   0.6304   0.6443   0.6568   0.6593
  0.6688   0.6737   0.6795   0.6821   0.6909   0.6996   0.7036   0.7107
  0.7316   0.7346   0.7385   0.7470   0.7571   0.7675   0.7781   0.7869
  0.8028   0.8112   0.8136   0.8200   0.8260   0.8349   0.8478   0.8674
  0.8683   0.8740   0.8814   0.8869   0.9033   0.9048   0.9224   0.9345
  0.9409   0.9467   0.9587   0.9771   0.9860   1.0015   1.0199   1.0290
  1.0492   1.0948   1.1004   1.1189   1.1343   1.1489   1.1629   1.1862
  1.1951   1.2005   1.2347   1.2449   1.2583   1.2739   1.2888   1.3039
  1.3137   1.3246   1.3343   1.3379   1.3409   1.3612   1.3679   1.3793
  1.3816   1.3955   1.3988   1.4013   1.4098   1.4122   1.4249   1.4302
  1.4443   1.4528   1.4549   1.4619   1.4733   1.4861   1.4927   1.4965
  1.5029   1.5047   1.5080   1.5135   1.5185   1.5388   1.5471   1.5515
  1.5660   1.5728   1.5742   1.5839   1.5951   1.6068   1.6130   1.6226
  1.6253   1.6319   1.6416   1.6475   1.6634   1.6747   1.6887   1.6977
  1.7207   1.7260   1.7285   1.7388   1.7446   1.7534   1.7625   1.7694
  1.7853   1.7884   1.8184   1.8269   1.8445   1.8527   1.8661   1.8751
  1.8954   1.9014   1.9136   1.9288   1.9326   1.9540   1.9648   1.9920
  2.0055   2.0108   2.0294   2.0439   2.0485   2.0662   2.0818   2.0865
  2.0944   2.1115   2.1302   2.1356   2.1461   2.1803   2.1979   2.2359
  2.2398   2.2463   2.2513   2.2621   2.2834   2.2860   2.2941   2.3063
  2.3119   2.3188   2.3495   2.3679   2.3736   2.3797   2.3888   2.4136
  2.4147   2.4215   2.4342   2.4442   2.4573   2.4643   2.4777   2.4850
```

| | | | | | | | |
|---|---|---|---|---|---|---|---|
| 2.4943 | 2.5096 | 2.5207 | 2.5247 | 2.5381 | 2.5445 | 2.5487 | 2.5513 |
| 2.5707 | 2.5796 | 2.5890 | 2.5919 | 2.5962 | 2.6055 | 2.6094 | 2.6289 |
| 2.6466 | 2.6533 | 2.6594 | 2.6698 | 2.6761 | 2.6878 | 2.6927 | 2.7013 |
| 2.7119 | 2.7250 | 2.7271 | 2.7340 | 2.7463 | 2.7598 | 2.7727 | 2.7775 |
| 2.7894 | 2.8022 | 2.8119 | 2.8359 | 2.8447 | 2.8563 | 2.8826 | 2.8926 |
| 2.9176 | 2.9265 | 2.9418 | 2.9566 | 3.0631 | 3.0665 | 3.1298 | 3.1394 |
| 3.1670 | 3.1971 | 3.2526 | 3.2598 | 3.2915 | 3.3359 | 3.3566 | 3.3965 |
| 3.4164 | 3.4722 | 3.5085 | 3.5515 | 3.5612 | 3.6037 | 3.6278 | 3.6446 |
| 3.6654 | 3.6782 | 3.7170 | 3.7362 | 3.7394 | 3.7628 | 3.7687 | 3.7809 |
| 3.8246 | 3.8718 | 3.8798 | 3.9113 | 3.9361 | 3.9882 | 4.0490 | 4.0837 |
| 4.1049 | 4.1819 | 4.2980 | 4.3023 | 4.6037 | 4.6601 | 4.6889 | 4.7391 |
| 4.9056 | 5.0425 | 5.1874 | 23.5395 | 23.5704 | 23.7193 | 23.7308 | 23.7415 |
| 23.7673 | 23.8017 | 23.8283 | 23.8384 | 23.8938 | 23.9236 | 23.9697 | 23.9773 |
| 24.0401 | 35.4212 | 35.4505 | 35.5073 | 35.6062 | | | |

Ground-State Mulliken Net Atomic Charges

| Atom | Charge (a.u.) |
|---|---|
| 1 N | 0.420583 |
| 2 C | 0.153345 |
| 3 C | -0.033152 |
| 4 C | -0.142197 |
| 5 C | -0.181528 |
| 6 C | -0.129650 |
| 7 C | -0.759240 |
| 8 C | -0.285781 |
| 9 N | -0.098001 |
| 10 C | -0.426706 |
| 11 N | 0.058087 |
| 12 C | 0.170272 |
| 13 C | -0.083005 |
| 14 C | 0.134864 |
| 15 C | -0.382217 |
| 16 C | -0.077643 |
| 17 C | -0.610631 |
| 18 N | -0.450609 |
| 19 H | 0.219971 |
| 20 H | 0.235322 |
| 21 H | 0.227785 |
| 22 H | 0.221626 |
| 23 H | 0.232506 |
| 24 H | 0.251621 |
| 25 H | 0.182523 |
| 26 H | 0.265730 |
| 27 H | 0.247019 |
| 28 H | 0.396709 |
| 29 H | 0.219319 |
| 30 H | 0.239736 |
| 31 H | 0.210961 |
| 32 H | 0.196672 |
| 33 H | 0.172168 |
| 34 H | 0.177443 |
| 35 H | 0.198431 |
| 36 H | 0.170900 |
| 37 H | 0.274080 |
| 38 H | 0.382685 |

Sum of atomic charges = 2.000000

Cartesian Multipole Moments

Charge (ESU x 10^10)
9.6064
Dipole Moment (Debye)
X 6.7312 Y -1.2879 Z -4.5601
Tot 8.2318
Quadrupole Moments (Debye-Ang)

```
        XX     -31.9559     XY      26.6224     YY     -65.3184
        XZ     -40.3282     YZ     -18.2255     ZZ     -49.4899
    Traceless Quadrupole Moments (Debye-Ang)
       QXX      50.8965    QYY     -49.1910    QZZ      -1.7054
       QXY      79.8671    QXZ    -120.9845    QYZ     -54.6765
    Octopole Moments (Debye-Ang^2)
       XXX      -6.2648    XXY      35.5993    XYY       8.2751
       YYY     -14.4485    XXZ      26.6758    XYZ     -21.4348
       YYZ      21.3968    XZZ      27.0664    YZZ      25.8885
       ZZZ     -75.5134
    Traceless Octopole Moments (Debye-Ang^2)
       XXX    -355.6618    YYY    -640.0819    ZZZ    -885.7335
       XXY     392.8721    XXZ     482.4591    XYY      36.8963
       XYZ    -321.5224    XZZ     318.7654    YYZ     403.2744
       YZZ     247.2098
    Hexadecapole Moments (Debye-Ang^3)
      XXXX   -3352.2939   XXXY    -214.9735   XXYY    -667.3930
      XYYY    -459.2928   YYYY    -755.2719   XXXZ     909.1762
      XXYZ     -75.1533   XYYZ     341.5766   YYYZ     319.2632
      XXZZ    -786.6315   XYZZ       3.7642   YYZZ    -532.0661
      XZZZ     853.1550   YZZZ     199.7637   ZZZZ   -1912.5988
    Traceless Hexadecapole Moments (Debye-Ang^3)
      XXXX   -9353.3142   XXXY    7600.3792   XXXZ     787.6518
      XXYY    1362.4375   XXYZ  -14537.7447   XXZZ    7990.8767
      XYYY  -18053.1509   XYYZ    4306.9260   XYZZ   10452.7717
      XZZZ   -5094.5778   YYYY    6691.1251   YYYZ   13582.6928
      YYZZ   -8053.5626   YZZZ     955.0519   ZZZZ      62.6858
 -----------------------------------------------------------------

 ------------------------------------------------
 -  Entering drvman on Sun Oct 13 19:09:47 2024  -
 ------------------------------------------------

 Calculating analytic gradient of the SCF energy
 Gradient of SCF Energy
           1           2           3           4           5           6
    1   0.0003232  -0.0008903   0.0001799   0.0000785  -0.0003422   0.0005566
    2   0.0005606   0.0002756  -0.0000534   0.0002883  -0.0007218  -0.0000560
    3   0.0006555  -0.0002479   0.0007512  -0.0000088   0.0007115  -0.0002823
           7           8           9          10          11          12
    1   0.0001472   0.0003481   0.0003783   0.0005790   0.0006016  -0.0016592
    2   0.0001364   0.0002961   0.0019301  -0.0006919  -0.0003578  -0.0028454
    3  -0.0001786  -0.0011327  -0.0008429   0.0000225   0.0010233   0.0054044
          13          14          15          16          17          18
    1  -0.0031347   0.0011218   0.0001636   0.0001387  -0.0000769   0.0057846
    2   0.0002327   0.0005694   0.0005915  -0.0000984  -0.0000007  -0.0004745
    3  -0.0009758   0.0001351   0.0000840  -0.0004991   0.0002052  -0.0098059
          19          20          21          22          23          24
    1  -0.0000945  -0.0000200   0.0000347   0.0001668  -0.0001380   0.0000312
    2  -0.0001009  -0.0000918   0.0001199  -0.0000328  -0.0001351   0.0000295
    3  -0.0000194   0.0000231   0.0001213  -0.0006023  -0.0000205   0.0000461
          25          26          27          28          29          30
    1  -0.0000986   0.0000516  -0.0004445  -0.0003322  -0.0000355  -0.0000219
    2   0.0000063  -0.0010391   0.0000934   0.0000698  -0.0001437  -0.0002130
    3  -0.0002355   0.0002823  -0.0008925   0.0001783   0.0003703  -0.0000440
          31          32          33          34          35          36
    1  -0.0001317  -0.0000547   0.0000050   0.0000194   0.0000404  -0.0000140
    2  -0.0000103  -0.0000301   0.0000333  -0.0000344   0.0000179   0.0000456
    3  -0.0001217   0.0002626   0.0001934  -0.0000168  -0.0000867  -0.0000122
          37          38
    1  -0.0036606   0.0003991
    2   0.0028035  -0.0009690
    3   0.0049536   0.0006018
 Max gradient component =       9.806E-03
 RMS gradient           =       1.469E-03
 Gradient time:  CPU 160.06 s  wall 271.11 s

 ------------------------------------------------
 -  Entering optman on Sun Oct 13 19:14:18 2024  -
 ------------------------------------------------
```

```
 Geometry Optimization Parameters
   NAtoms,    NIC,     NZ,  NCons,   NDum,   NFix, NCnnct, MaxDiis
       38     272       0       0       0       0       0       0

 Cartesian Hessian Update
 Hessian updated using BFGS update


** GEOMETRY OPTIMIZATION IN DELOCALIZED INTERNAL COORDINATES **
   Searching for a Minimum

   Optimization Cycle:   4

                    Coordinates (Angstroms)
     ATOM                X               Y               Z
      1  N         2.2479147276    0.1575471438   -1.5406169822
      2  C         3.1192100515    1.1949381258   -1.6780289041
      3  C         3.2493542961    1.7776825015   -2.9314611239
      4  C         2.5282243451    1.3102320675   -4.0167825085
      5  C         1.6646354587    0.2301748617   -3.8464630616
      6  C         1.5507676979   -0.3230388625   -2.6001609135
      7  C         3.9388241911    1.6710888085   -0.5179376627
      8  C         2.0557569021   -0.5354610462   -0.2397963552
      9  N        -1.1956365209   -1.8892889447    1.0104018634
     10  C        -2.0265195413   -0.8153634168    0.9683377890
     11  N        -1.5700539271    0.3518480363    0.5865514996
     12  C        -0.2826740938    0.4969640211    0.2505913256
     13  C         0.6107675879   -0.6193486900    0.1962378714
     14  C         0.0946470678   -1.8089944974    0.6142636314
     15  C        -3.4265567405   -0.9743715857    1.4376443685
     16  C        -3.5180699466   -0.7571654117    2.9676312636
     17  C        -4.9569015917   -0.8847115513    3.4571066921
     18  N         0.0908761582    1.7347423953   -0.1354643498
     19  H         3.9389413658    2.6036319936   -3.0463466152
     20  H         2.6439137790    1.7735713942   -4.9895981158
     21  H         1.0940996840   -0.1802056306   -4.6686229398
     22  H         0.9034712466   -1.1672718079   -2.4033235314
     23  H         4.5628244715    2.5038755394   -0.8357579942
     24  H         4.6023513280    0.8849438035   -0.1472371677
     25  H         3.3293332664    2.0245812216    0.3186444749
     26  H         2.6542202193   -0.0320129781    0.5153817120
     27  H         2.4673315347   -1.5405679283   -0.3488655288
     28  H        -1.5655823620   -2.7752653948    1.3369978568
     29  H         0.6653357082   -2.7301215817    0.6459900183
     30  H        -4.0384095277   -0.2344355390    0.9203476482
     31  H        -3.8024866312   -1.9692563751    1.1782636253
     32  H        -3.1254841402    0.2351965949    3.2074152014
     33  H        -2.8833141372   -1.4874822367    3.4824083949
     34  H        -5.6027899592   -0.1443026490    2.9798261256
     35  H        -5.0026010331   -0.7230151154    4.5346935035
     36  H        -5.3634819955   -1.8773334357    3.2492527148
     37  H         0.9952011889    2.0957186145    0.1266854991
     38  H        -0.6474401281    2.4222775554   -0.0382093252
   Point Group: c1    Number of degrees of freedom:   108


   Energy is   -763.964415884

 Hessian updated using BFGS update
  internal optimization (0)

108 Hessian modes will be used to form the next step
  Hessian Eigenvalues:
     0.001798    0.002729    0.003541    0.004055    0.006466    0.009857
     0.012548    0.018941    0.019222    0.019983    0.021063    0.022037
     0.022596    0.023466    0.024074    0.024271    0.025259    0.026844
     0.027868    0.028178    0.028575    0.029824    0.034445    0.036194
     0.038299    0.040795    0.042865    0.043602    0.043835    0.046763
     0.048902    0.052622    0.053809    0.055802    0.068033    0.077490
```

```
  0.083922   0.095137   0.122098   0.122569   0.126858   0.128363
  0.131781   0.132787   0.137136   0.140460   0.143031   0.144664
  0.146838   0.147522   0.148640   0.151658   0.152148   0.152637
  0.154557   0.159775   0.191553   0.192391   0.206499   0.213948
  0.218640   0.230621   0.239021   0.248321   0.249448   0.255780
  0.271697   0.273461   0.282943   0.286582   0.296522   0.299251
  0.300157   0.300616   0.301249   0.302462   0.303287   0.304128
  0.304880   0.304909   0.305528   0.306464   0.309710   0.312326
  0.319397   0.330522   0.332856   0.332998   0.335437   0.339370
  0.340990   0.349087   0.361459   0.363517   0.378508   0.384844
  0.393525   0.403884   0.407213   0.416584   0.418946   0.432811
  0.433923   0.449581   0.457494   0.527131   0.650239   0.728293

Minimum search - taking simple RFO step
Searching for Lamda that Minimizes Along All modes
Value Taken    Lamda =  -0.00496422
Calculated Step too Large.  Step scaled by  0.476070
Step Taken.  Stepsize is  0.300000

                          Maximum     Tolerance    Cnvgd?
       Gradient           0.003336     0.000800      NO
       Displacement       0.114612     0.001400      NO
       Energy change     -0.002828     0.000228      NO


New Cartesian Coordinates Obtained by Inverse Iteration

Displacement from previous Coordinates is:  0.486869
----------------------------------------------------------------
          Standard Nuclear Orientation (Angstroms)
   I     Atom           X                 Y                 Z
----------------------------------------------------------------
   1      N       2.2462124762      0.1743358711     -1.5314025883
   2      C       3.1404034293      1.1904229789     -1.6849672001
   3      C       3.2767283141      1.7538289104     -2.9465812140
   4      C       2.5413928122      1.2880108595     -4.0229516970
   5      C       1.6543576633      0.2296809257     -3.8354452325
   6      C       1.5331671159     -0.3042028416     -2.5810726452
   7      C       3.9748942358      1.6609346334     -0.5328149993
   8      C       2.0492916442     -0.4992481902     -0.2192446123
   9      N      -1.2027043603     -1.8881425700      0.9922511971
  10      C      -2.0396243848     -0.8154320137      0.9649139716
  11      N      -1.5886331871      0.3570082131      0.5973994049
  12      C      -0.3008340668      0.5179895556      0.2627244386
  13      C       0.6015146118     -0.5966645767      0.1986582867
  14      C       0.0877999100     -1.7941154205      0.5972899225
  15      C      -3.4368863656     -0.9874036027      1.4360816187
  16      C      -3.5250963617     -0.7712676863      2.9675798297
  17      C      -4.9615764742     -0.9068325961      3.4616487458
  18      N       0.0581573967      1.7575655656     -0.0966476750
  19      H       3.9837572858      2.5629646468     -3.0733914380
  20      H       2.6633419684      1.7368779627     -5.0016145263
  21      H       1.0709251344     -0.1794353611     -4.6490845150
  22      H       0.8674350246     -1.1293078098     -2.3658811039
  23      H       4.6179652302      2.4737465031     -0.8638788000
  24      H       4.6200810164      0.8632735046     -0.1545191649
  25      H       3.3795964083      2.0402438486      0.3030816955
  26      H       2.6310786279      0.0316014805      0.5304096538
  27      H       2.4778389230     -1.4997166592     -0.3010848516
  28      H      -1.5659826720     -2.7811450545      1.3049012032
  29      H       0.6647440871     -2.7115297484      0.6154284611
  30      H      -4.0544569560     -0.2498802735      0.9223816468
  31      H      -3.8059228260     -1.9846009857      1.1768033326
  32      H      -3.1368247819      0.2235247738      3.2036652385
  33      H      -2.8835898429     -1.4978978410      3.4788077729
  34      H      -5.6132274689     -0.1702421482      2.9864001847
  35      H      -5.0050497934     -0.7449787601      4.5392852603
  36      H      -5.3632724519     -1.9017646363      3.2557360269
  37      H       1.0002068998      2.0903883154      0.0281679904
  38      H      -0.6572082218      2.4614102267      0.0369663813
```

```
 --------------------------------------------------------------
 Nuclear Repulsion Energy =        1301.02928194 hartrees
 There are       65 alpha and       65 beta electrons

--------------------------------------------------
-  Entering fldman on Sun Oct 13 19:14:18 2024  -
--------------------------------------------------

 Applying Cartesian multipole field
    Component          Value
    ---------          -----
     (2,0,0)        1.00000E-12
     (0,2,0)        2.00000E-11
     (0,0,2)       -3.00000E-11
 Nucleus-field energy     =    -0.0000000461 hartrees

--------------------------------------------------
-  Entering gesman on Sun Oct 13 19:14:18 2024  -
--------------------------------------------------

 Requested basis set is 6-311+G(d,p)
 There are 188 shells and 516 basis functions
 A cutoff of  1.0D-12 yielded  12472 shell pairs
 There are    100040 function pairs (    106682 Cartesian)
 Smallest overlap matrix eigenvalue = 1.29E-06
 Linear dependence detected in AO basis
 Tighter screening thresholds may be required for diffuse basis sets
 Use S2THRESH > 12 and THRESH = 14 in case of SCF convergence issues
 Number of orthogonalized atomic orbitals = 510
 Maximum deviation from orthogonality = 1.455E-11
 Guess MOs from SCF MO coefficient file
 Reading MOs from coefficient file
 Reading MOs from coefficient file

--------------------------------------------------
-  Entering scfman on Sun Oct 13 19:14:19 2024  -
--------------------------------------------------

 Long-range K will be added via erf
 Coulomb attenuation parameter = 0.2 bohr**(-1)
 A restricted hybrid HF-DFT SCF calculation will be
 performed using Pulay DIIS + Geometric Direct Minimization
 Exchange:     0.2220 Hartree-Fock + 1.0000 wB97X-D + LR-HF
 Correlation:  1.0000 wB97X-D
 Using Euler-Maclaurin-Lebedev (75,302) quadrature formula
 Dispersion:   Grimme D
 SCF converges when RMS gradient is below 1.0E-07
 Geometry optimization detected.  Setting ReadMinima to 0
 Setting SaveMinima to 0
 ---------------------------------------
  Cycle       Energy         DIIS Error
 ---------------------------------------
    1    -763.9857205694      4.72E-04
    2    -763.9659225037      9.79E-05
    3    -763.9661215505      9.62E-05
    4    -763.9664002629      1.74E-05
    5    -763.9664140710      7.94E-06
    6    -763.9664164135      3.40E-06
    7    -763.9664168929      9.58E-07
    8    -763.9664169405      3.85E-07
    9    -763.9664169488      1.19E-07
   10    -763.9664169498      4.65E-08 Convergence criterion met
 ---------------------------------------
 SCF time:  CPU 349.44 s  wall 584.09 s
 SCF   energy in the final basis set = -763.96641695
 Total energy in the final basis set = -763.96641695

--------------------------------------------------
-  Entering anlman on Sun Oct 13 19:24:03 2024  -
--------------------------------------------------
```

```
 --------------------------------------------------------------

                   Orbital Energies (a.u.)
 --------------------------------------------------------------

 Alpha MOs
 -- Occupied --
-14.8160 -14.7922 -14.7374 -14.7100 -10.6781 -10.6644 -10.6481 -10.6405
-10.6338 -10.6254 -10.6069 -10.5840 -10.5730 -10.5711 -10.5519 -10.5261
-10.4967 -10.4503  -1.4176  -1.4052  -1.3487  -1.2772  -1.2314  -1.2017
 -1.1885  -1.1419  -1.0995  -1.0848  -1.0558  -1.0531  -1.0322  -1.0011
 -0.9910  -0.9538  -0.9411  -0.9109  -0.9047  -0.8922  -0.8839  -0.8652
 -0.8561  -0.8422  -0.8376  -0.8195  -0.8092  -0.7954  -0.7909  -0.7816
 -0.7702  -0.7644  -0.7570  -0.7495  -0.7437  -0.7292  -0.7197  -0.7120
 -0.6820  -0.6685  -0.6558  -0.6449  -0.6328  -0.6233  -0.6198  -0.6158
 -0.6111
 -- Virtual --
 -0.2713  -0.2625  -0.2459  -0.2245  -0.1748  -0.1625  -0.1456  -0.1430
 -0.1358  -0.1234  -0.1175  -0.1133  -0.1091  -0.1029  -0.0984  -0.0949
 -0.0900  -0.0878  -0.0812  -0.0790  -0.0756  -0.0743  -0.0722  -0.0681
 -0.0612  -0.0580  -0.0572  -0.0510  -0.0461  -0.0445  -0.0370  -0.0311
 -0.0284  -0.0255  -0.0201  -0.0182  -0.0170  -0.0158  -0.0128  -0.0066
 -0.0048  -0.0015   0.0015   0.0045   0.0068   0.0116   0.0123   0.0151
  0.0189   0.0222   0.0245   0.0280   0.0305   0.0330   0.0359   0.0402
  0.0419   0.0452   0.0510   0.0551   0.0555   0.0605   0.0640   0.0686
  0.0701   0.0721   0.0763   0.0783   0.0793   0.0870   0.0927   0.0946
  0.0975   0.1008   0.1025   0.1040   0.1068   0.1133   0.1166   0.1191
  0.1209   0.1255   0.1285   0.1358   0.1375   0.1415   0.1468   0.1485
  0.1519   0.1560   0.1589   0.1654   0.1656   0.1690   0.1721   0.1736
  0.1853   0.1877   0.1933   0.1957   0.2014   0.2064   0.2176   0.2251
  0.2287   0.2402   0.2523   0.2696   0.2825   0.2899   0.3004   0.3127
  0.3220   0.3335   0.3379   0.3453   0.3511   0.3550   0.3608   0.3630
  0.3700   0.3801   0.3865   0.3966   0.3988   0.4053   0.4118   0.4286
  0.4320   0.4337   0.4402   0.4518   0.4614   0.4663   0.4721   0.4758
  0.4848   0.4898   0.4957   0.5016   0.5056   0.5084   0.5196   0.5229
  0.5270   0.5285   0.5308   0.5402   0.5433   0.5544   0.5572   0.5595
  0.5611   0.5676   0.5695   0.5822   0.5865   0.5874   0.5933   0.6018
  0.6050   0.6194   0.6285   0.6292   0.6306   0.6465   0.6571   0.6589
  0.6697   0.6743   0.6800   0.6827   0.6918   0.6995   0.7048   0.7112
  0.7292   0.7359   0.7399   0.7504   0.7602   0.7726   0.7777   0.7822
  0.7977   0.8088   0.8136   0.8190   0.8247   0.8369   0.8517   0.8649
  0.8697   0.8743   0.8820   0.8873   0.9022   0.9052   0.9177   0.9325
  0.9452   0.9477   0.9605   0.9759   0.9865   1.0019   1.0200   1.0302
  1.0486   1.0859   1.0988   1.1196   1.1352   1.1514   1.1613   1.1845
  1.1935   1.2042   1.2318   1.2418   1.2566   1.2741   1.2849   1.3045
  1.3145   1.3225   1.3347   1.3350   1.3386   1.3609   1.3677   1.3780
  1.3846   1.3954   1.3975   1.4007   1.4029   1.4125   1.4216   1.4282
  1.4467   1.4526   1.4545   1.4603   1.4742   1.4871   1.4925   1.4975
  1.5021   1.5060   1.5075   1.5143   1.5188   1.5396   1.5471   1.5509
  1.5668   1.5714   1.5737   1.5844   1.5933   1.6072   1.6123   1.6206
  1.6237   1.6328   1.6408   1.6471   1.6602   1.6754   1.6880   1.7006
  1.7223   1.7255   1.7277   1.7372   1.7450   1.7554   1.7602   1.7700
  1.7828   1.7873   1.8179   1.8253   1.8443   1.8518   1.8643   1.8758
  1.8947   1.8998   1.9173   1.9263   1.9337   1.9582   1.9689   1.9907
  2.0067   2.0118   2.0310   2.0458   2.0481   2.0710   2.0835   2.0865
  2.0969   2.1108   2.1329   2.1348   2.1480   2.1802   2.1997   2.2363
  2.2399   2.2472   2.2518   2.2620   2.2810   2.2840   2.2901   2.3034
  2.3119   2.3153   2.3464   2.3684   2.3741   2.3775   2.3889   2.4096
  2.4153   2.4188   2.4374   2.4439   2.4522   2.4663   2.4769   2.4841
  2.4956   2.5103   2.5201   2.5225   2.5406   2.5445   2.5498   2.5525
  2.5706   2.5772   2.5891   2.5909   2.5960   2.6028   2.6064   2.6270
  2.6463   2.6563   2.6621   2.6701   2.6770   2.6888   2.6966   2.7047
  2.7121   2.7221   2.7303   2.7372   2.7499   2.7577   2.7737   2.7800
  2.7893   2.7992   2.8145   2.8354   2.8460   2.8585   2.8856   2.8924
  2.9142   2.9275   2.9407   2.9523   3.0614   3.0710   3.1281   3.1385
  3.1728   3.2245   3.2524   3.2598   3.2890   3.3360   3.3595   3.4010
  3.4168   3.4709   3.5139   3.5521   3.5589   3.6025   3.6236   3.6450
  3.6648   3.6795   3.7188   3.7369   3.7411   3.7621   3.7644   3.7791
```

```
  3.8270   3.8385   3.8717   3.9104   3.9336   3.9805   4.0492   4.0814
  4.1057   4.1824   4.2973   4.3013   4.6047   4.6631   4.6933   4.7350
  4.9144   5.0411   5.1905  23.5365  23.5734  23.7193  23.7309  23.7416
 23.7652  23.8024  23.8268  23.8399  23.8921  23.9245  23.9687  23.9769
 24.0385  35.4198  35.4531  35.5116  35.6105
--------------------------------------------------------------

          Ground-State Mulliken Net Atomic Charges

     Atom                 Charge (a.u.)
  ----------------------------------------
      1 N                    0.410671
      2 C                    0.148558
      3 C                   -0.025264
      4 C                   -0.145561
      5 C                   -0.176589
      6 C                   -0.127563
      7 C                   -0.762872
      8 C                   -0.293670
      9 N                   -0.097913
     10 C                   -0.422037
     11 N                    0.048374
     12 C                    0.192886
     13 C                   -0.099791
     14 C                    0.140834
     15 C                   -0.382078
     16 C                   -0.078863
     17 C                   -0.610021
     18 N                   -0.447527
     19 H                    0.220494
     20 H                    0.235797
     21 H                    0.228190
     22 H                    0.223350
     23 H                    0.232776
     24 H                    0.251248
     25 H                    0.185986
     26 H                    0.264486
     27 H                    0.248582
     28 H                    0.395645
     29 H                    0.218006
     30 H                    0.239306
     31 H                    0.209524
     32 H                    0.196363
     33 H                    0.172419
     34 H                    0.177116
     35 H                    0.198191
     36 H                    0.170959
     37 H                    0.273733
     38 H                    0.386257
  ----------------------------------------
  Sum of atomic charges =     2.000000

-----------------------------------------------------------------
                   Cartesian Multipole Moments
-----------------------------------------------------------------
    Charge (ESU x 10^10)
                 9.6064
    Dipole Moment (Debye)
         X       6.8778      Y      -1.0749      Z      -4.7675
       Tot       8.4374
    Quadrupole Moments (Debye-Ang)
        XX     -31.3634     XY      26.8942     YY     -65.4747
        XZ     -40.6696     YZ     -18.2202     ZZ     -49.6629
    Traceless Quadrupole Moments (Debye-Ang)
       QXX      52.4109    QYY     -49.9232    QZZ      -2.4877
       QXY      80.6826    QXZ    -122.0088    QYZ     -54.6607
    Octopole Moments (Debye-Ang^2)
       XXX      -0.4823    XXY      36.2184    XYY       7.3533
       YYY     -14.1542    XXZ      24.1514    XYZ     -22.3083
       YYZ      21.5077    XZZ      28.8136    YZZ      25.9490
```

```
      ZZZ     -77.3157
   Traceless Octopole Moments (Debye-Ang^2)
      XXX    -328.3954    YYY    -644.4319    ZZZ    -874.8267
      XXY     399.2369    XXZ     457.2411    XYY       3.2456
      XYZ    -334.6244    XZZ     325.1499    YYZ     417.5856
      YZZ     245.1949
   Hexadecapole Moments (Debye-Ang^3)
     XXXX   -3359.6331   XXXY    -211.7281   XXYY    -671.8685
     XYYY    -468.5360   YYYY    -751.2622   XXXZ     902.8766
     XXYZ     -79.3226   XYYZ     343.6677   YYYZ     323.5057
     XXZZ    -784.4844   XYZZ       3.6016   YYZZ    -535.6967
     XZZZ     853.0671   YZZZ     200.0179   ZZZZ   -1908.1151
   Traceless Hexadecapole Moments (Debye-Ang^3)
     XXXX   -9350.7271   XXXY    8218.3619   XXXZ     319.5354
     XXYY    1066.6815   XXYZ  -14991.6225   XXZZ    8284.0456
     XYYY  -18746.4654   XYYZ    4590.9347   XYZZ   10528.1036
     XZZZ   -4910.4701   YYYY    7383.9447   YYYZ   13979.8628
     YYZZ   -8450.6262   YZZZ    1011.7596   ZZZZ     166.5806
 -----------------------------------------------------------------

-------------------------------------------------
-  Entering drvman on Sun Oct 13 19:24:03 2024  -
-------------------------------------------------

 Calculating analytic gradient of the SCF energy
 Gradient of SCF Energy
           1           2           3           4           5           6
    1   0.0004042  -0.0006255  -0.0001484   0.0003247  -0.0004230   0.0003051
    2   0.0006187   0.0006560  -0.0002572   0.0005081  -0.0006164  -0.0001432
    3   0.0003144  -0.0002708   0.0002805   0.0000710   0.0004521  -0.0002818
           7           8           9          10          11          12
    1  -0.0000838   0.0004988   0.0001717  -0.0014920   0.0010676  -0.0013708
    2   0.0000112  -0.0000841   0.0009778   0.0000866  -0.0010913   0.0014991
    3  -0.0001528  -0.0009708  -0.0000531  -0.0002607   0.0001751   0.0046351
          13          14          15          16          17          18
    1  -0.0012705   0.0011294   0.0002694   0.0000931  -0.0001581   0.0026503
    2  -0.0002524  -0.0004042   0.0001525  -0.0000294  -0.0000197  -0.0014943
    3  -0.0010178   0.0001849  -0.0003767   0.0002369   0.0001517  -0.0082189
          19          20          21          22          23          24
    1  -0.0001208   0.0000007   0.0000307   0.0002023  -0.0002193   0.0000011
    2  -0.0002060  -0.0000756  -0.0000131  -0.0000246  -0.0001724   0.0000844
    3   0.0002117   0.0001563   0.0002451  -0.0000324  -0.0000308   0.0001347
          25          26          27          28          29          30
    1   0.0001250   0.0003724  -0.0006448   0.0000432   0.0000148   0.0002313
    2  -0.0000182  -0.0008395   0.0000264   0.0002409   0.0000017  -0.0001172
    3   0.0000855  -0.0000220  -0.0005499   0.0000659   0.0000746   0.0000145
          31          32          33          34          35          36
    1  -0.0000730  -0.0000029   0.0000345   0.0000292  -0.0000027  -0.0000139
    2   0.0000127  -0.0000677   0.0000427  -0.0000445   0.0000201   0.0000826
    3  -0.0000305   0.0000292   0.0000591   0.0000303  -0.0001220   0.0000265
          37          38
    1  -0.0020573   0.0007072
    2   0.0018921  -0.0009431
    3   0.0038747   0.0008810
 Max gradient component =       8.219E-03
 RMS gradient           =       1.124E-03
 Gradient time:  CPU 160.55 s  wall 269.37 s

-------------------------------------------------
-  Entering optman on Sun Oct 13 19:28:32 2024  -
-------------------------------------------------

 Geometry Optimization Parameters
   NAtoms,    NIC,     NZ,  NCons,   NDum,   NFix, NCnnct, MaxDiis
       38     272       0       0       0       0       0       0

 Cartesian Hessian Update
 Hessian updated using BFGS update
```

```
** GEOMETRY OPTIMIZATION IN DELOCALIZED INTERNAL COORDINATES **
   Searching for a Minimum

   Optimization Cycle:   5

                      Coordinates (Angstroms)
     ATOM                X               Y               Z
      1  N         2.2462124762    0.1743358711   -1.5314025883
      2  C         3.1404034293    1.1904229789   -1.6849672001
      3  C         3.2767283141    1.7538289104   -2.9465812140
      4  C         2.5413928122    1.2880108595   -4.0229516970
      5  C         1.6543576633    0.2296809257   -3.8354452325
      6  C         1.5331671159   -0.3042028416   -2.5810726452
      7  C         3.9748942358    1.6609346334   -0.5328149993
      8  C         2.0492916442   -0.4992481902   -0.2192446123
      9  N        -1.2027043603   -1.8881425700    0.9922511971
     10  C        -2.0396243848   -0.8154320137    0.9649139716
     11  N        -1.5886331871    0.3570082131    0.5973994049
     12  C        -0.3008340668    0.5179895556    0.2627244386
     13  C         0.6015146118   -0.5966645767    0.1986582867
     14  C         0.0877999100   -1.7941154205    0.5972899225
     15  C        -3.4368863656   -0.9874036027    1.4360816187
     16  C        -3.5250963617   -0.7712676863    2.9675798297
     17  C        -4.9615764742   -0.9068325961    3.4616487458
     18  N         0.0581573967    1.7575655656   -0.0966476750
     19  H         3.9837572858    2.5629646468   -3.0733914380
     20  H         2.6633419684    1.7368779627   -5.0016145263
     21  H         1.0709251344   -0.1794353611   -4.6490845150
     22  H         0.8674350246   -1.1293078098   -2.3658811039
     23  H         4.6179652302    2.4737465031   -0.8638788000
     24  H         4.6200810164    0.8632735046   -0.1545191649
     25  H         3.3795964083    2.0402438486    0.3030816955
     26  H         2.6310786279    0.0316014805    0.5304096538
     27  H         2.4778389230   -1.4997166592   -0.3010848516
     28  H        -1.5659826720   -2.7811450545    1.3049012032
     29  H         0.6647440871   -2.7115297484    0.6154284611
     30  H        -4.0544569560   -0.2498802735    0.9223816468
     31  H        -3.8059228260   -1.9846009857    1.1768033326
     32  H        -3.1368247819    0.2235247738    3.2036652385
     33  H        -2.8835898429   -1.4978978410    3.4788077729
     34  H        -5.6132274689   -0.1702421482    2.9864001847
     35  H        -5.0050497934   -0.7449787601    4.5392852603
     36  H        -5.3632724519   -1.9017646363    3.2557360269
     37  H         1.0002068998    2.0903883154    0.0281679904
     38  H        -0.6572082218    2.4614102267    0.0369663813
   Point Group: c1    Number of degrees of freedom:   108


   Energy is   -763.966416950

 Hessian updated using BFGS update
  internal optimization (0)

108 Hessian modes will be used to form the next step
  Hessian Eigenvalues:
     0.001792    0.002728    0.003529    0.003982    0.006368    0.011184
     0.012650    0.018972    0.019223    0.020056    0.021057    0.022047
     0.022600    0.023523    0.024115    0.024273    0.025263    0.026770
     0.027303    0.028178    0.028415    0.029477    0.033897    0.036155
     0.038296    0.040707    0.042844    0.043595    0.043833    0.046720
     0.048788    0.051818    0.053813    0.055802    0.068106    0.077583
     0.083921    0.095126    0.122097    0.122643    0.126858    0.128368
     0.131788    0.132792    0.137112    0.140559    0.143099    0.144465
     0.146838    0.147526    0.148618    0.151658    0.152239    0.152644
     0.154558    0.160442    0.191629    0.192607    0.206511    0.213946
     0.218662    0.230742    0.238963    0.248318    0.250221    0.255820
     0.271702    0.273934    0.283115    0.286563    0.296676    0.299351
     0.300160    0.300618    0.301247    0.302463    0.303296    0.304129
     0.304881    0.304909    0.305544    0.306490    0.309819    0.312465
     0.319927    0.330840    0.332908    0.332975    0.335747    0.339371
```

```
    0.341032    0.349144    0.361655    0.366442    0.380100    0.384863
    0.393480    0.403243    0.407474    0.416793    0.419751    0.433914
    0.440697    0.451336    0.457997    0.525022    0.649811    0.728290

 Minimum search - taking simple RFO step
 Searching for Lamda that Minimizes Along All modes
 Value Taken    Lamda =  -0.00313490
 Calculated Step too Large.  Step scaled by  0.528492
 Step Taken.  Stepsize is  0.300000

                             Maximum     Tolerance    Cnvgd?
         Gradient           0.002520      0.000800      NO
         Displacement       0.096691      0.001400      NO
         Energy change     -0.002001      0.000228      NO


 New Cartesian Coordinates Obtained by Inverse Iteration

 Displacement from previous Coordinates is:  0.573535
 ----------------------------------------------------------------
             Standard Nuclear Orientation (Angstroms)
    I     Atom           X                Y                Z
 ----------------------------------------------------------------
    1      N       2.2425600189     0.1953540495    -1.5242584234
    2      C       3.1662301528     1.1828319208    -1.6958128209
    3      C       3.3169966512     1.7198801170    -2.9678407378
    4      C       2.5690036362     1.2557697210    -4.0362205790
    5      C       1.6522750530     0.2268170708    -3.8297194074
    6      C       1.5156060153    -0.2812505066    -2.5652851825
    7      C       4.0129374986     1.6495679927    -0.5508767834
    8      C       2.0365062322    -0.4520570467    -0.1981218964
    9      N      -1.2134774584    -1.8850916698     0.9655296591
   10      C      -2.0540726439    -0.8130096637     0.9643191746
   11      N      -1.6105183848     0.3659667207     0.6163450848
   12      C      -0.3240421945     0.5403489301     0.2777099964
   13      C       0.5862393457    -0.5700092721     0.1975019842
   14      C       0.0760619414    -1.7755625992     0.5709259817
   15      C      -3.4481618589    -0.9996484600     1.4388685027
   16      C      -3.5312911056    -0.7889518901     2.9717511233
   17      C      -4.9646398017    -0.9359819507     3.4713519729
   18      N       0.0247209482     1.7853675261    -0.0431429839
   19      H       4.0462060422     2.5068870137    -3.1080496301
   20      H       2.7031895407     1.6841172493    -5.0223762014
   21      H       1.0569099233    -0.1803284252    -4.6357665228
   22      H       0.8260577757    -1.0819187882    -2.3339860963
   23      H       4.6844795283     2.4323137978    -0.8976809087
   24      H       4.6291797420     0.8387496223    -0.1530103008
   25      H       3.4284596302     2.0679136420     0.2741302318
   26      H       2.5915900529     0.1145937678     0.5459285884
   27      H       2.4903252240    -1.4436017056    -0.2446464737
   28      H      -1.5712901394    -2.7862911482     1.2592288377
   29      H       0.6590757124    -2.6892527907     0.5725782743
   30      H      -4.0735175504    -0.2642742821     0.9313613905
   31      H      -3.8100920560    -1.9986402131     1.1771079998
   32      H      -3.1487424636     0.2080223998     3.2077219165
   33      H      -2.8820482724    -1.5125766179     3.4771826379
   34      H      -5.6235035691    -0.2026194994     3.0010574311
   35      H      -5.0047317965    -0.7774150375     4.5496345045
   36      H      -5.3603560990    -1.9331222583     3.2645994340
   37      H       0.9806556867     2.0977762788    -0.0413616175
   38      H      -0.6747809574     2.4993260047     0.1133218397
 ----------------------------------------------------------------
 Nuclear Repulsion Energy =        1299.72227886 hartrees
 There are       65 alpha and       65 beta electrons

--------------------------------------------------
-  Entering fldman on Sun Oct 13 19:28:32 2024  -
--------------------------------------------------

 Applying Cartesian multipole field
```

```
   Component          Value
   ---------          -----
     (2,0,0)        1.00000E-12
     (0,2,0)        2.00000E-11
     (0,0,2)       -3.00000E-11
 Nucleus-field energy     =    -0.0000000463 hartrees

--------------------------------------------------
-  Entering gesman on Sun Oct 13 19:28:32 2024  -
--------------------------------------------------

 Requested basis set is 6-311+G(d,p)
 There are 188 shells and 516 basis functions
 A cutoff of  1.0D-12 yielded  12451 shell pairs
 There are     99883 function pairs (    106517 Cartesian)
 Smallest overlap matrix eigenvalue = 1.23E-06
 Linear dependence detected in AO basis
 Tighter screening thresholds may be required for diffuse basis sets
 Use S2THRESH > 12 and THRESH = 14 in case of SCF convergence issues
 Number of orthogonalized atomic orbitals = 510
 Maximum deviation from orthogonality = 1.496E-11
 Guess MOs from SCF MO coefficient file
 Reading MOs from coefficient file
 Reading MOs from coefficient file

--------------------------------------------------
-  Entering scfman on Sun Oct 13 19:28:33 2024  -
--------------------------------------------------

 Long-range K will be added via erf
 Coulomb attenuation parameter = 0.2 bohr**(-1)
 A restricted hybrid HF-DFT SCF calculation will be
 performed using Pulay DIIS + Geometric Direct Minimization
 Exchange:     0.2220 Hartree-Fock + 1.0000 wB97X-D + LR-HF
 Correlation:  1.0000 wB97X-D
 Using Euler-Maclaurin-Lebedev (75,302) quadrature formula
 Dispersion:   Grimme D
 SCF converges when RMS gradient is below 1.0E-07
 Geometry optimization detected.  Setting ReadMinima to 0
 Setting SaveMinima to 0
 ---------------------------------------
  Cycle       Energy         DIIS Error
 ---------------------------------------
    1    -763.9829510626      5.40E-04
    2    -763.9673028687      8.75E-05
    3    -763.9676189520      7.21E-05
    4    -763.9677803238      2.18E-05
    5    -763.9678005938      7.28E-06
    6    -763.9678027424      3.33E-06
    7    -763.9678031409      8.74E-07
    8    -763.9678031829      3.63E-07
    9    -763.9678031897      1.11E-07
   10    -763.9678031905      4.54E-08 Convergence criterion met
 ---------------------------------------
 SCF time:  CPU 349.92 s  wall 581.79 s
 SCF   energy in the final basis set = -763.96780319
 Total energy in the final basis set = -763.96780319

--------------------------------------------------
-  Entering anlman on Sun Oct 13 19:38:14 2024  -
--------------------------------------------------



 --------------------------------------------------------------

                   Orbital Energies (a.u.)
 --------------------------------------------------------------

 Alpha MOs
 -- Occupied --
```

```
-14.8169 -14.7902 -14.7400 -14.7084 -10.6781 -10.6632 -10.6471 -10.6412
-10.6341 -10.6262 -10.6060 -10.5847 -10.5739 -10.5720 -10.5518 -10.5251
-10.4958 -10.4497  -1.4164  -1.4055  -1.3497  -1.2774  -1.2315  -1.2017
 -1.1889  -1.1415  -1.0992  -1.0850  -1.0555  -1.0529  -1.0324  -1.0006
 -0.9913  -0.9549  -0.9406  -0.9129  -0.9052  -0.8920  -0.8842  -0.8663
 -0.8567  -0.8425  -0.8376  -0.8198  -0.8092  -0.7960  -0.7911  -0.7811
 -0.7693  -0.7636  -0.7576  -0.7498  -0.7452  -0.7299  -0.7199  -0.7115
 -0.6813  -0.6679  -0.6565  -0.6442  -0.6319  -0.6232  -0.6191  -0.6151
 -0.6102
 -- Virtual --
 -0.2719  -0.2601  -0.2445  -0.2255  -0.1748  -0.1625  -0.1456  -0.1429
 -0.1363  -0.1229  -0.1172  -0.1134  -0.1086  -0.1025  -0.0986  -0.0958
 -0.0897  -0.0878  -0.0813  -0.0793  -0.0757  -0.0743  -0.0720  -0.0681
 -0.0614  -0.0576  -0.0566  -0.0509  -0.0459  -0.0445  -0.0387  -0.0314
 -0.0288  -0.0254  -0.0208  -0.0182  -0.0168  -0.0157  -0.0129  -0.0066
 -0.0048  -0.0018   0.0011   0.0048   0.0075   0.0115   0.0129   0.0148
  0.0184   0.0213   0.0246   0.0284   0.0301   0.0332   0.0356   0.0405
  0.0422   0.0456   0.0507   0.0548   0.0561   0.0610   0.0647   0.0674
  0.0695   0.0724   0.0766   0.0789   0.0795   0.0877   0.0921   0.0948
  0.0970   0.1007   0.1023   0.1039   0.1067   0.1135   0.1163   0.1191
  0.1211   0.1258   0.1279   0.1347   0.1377   0.1400   0.1451   0.1486
  0.1525   0.1571   0.1597   0.1653   0.1656   0.1694   0.1723   0.1726
  0.1855   0.1890   0.1940   0.1964   0.2025   0.2100   0.2176   0.2230
  0.2273   0.2387   0.2526   0.2709   0.2836   0.2901   0.3014   0.3098
  0.3210   0.3340   0.3385   0.3456   0.3515   0.3550   0.3604   0.3626
  0.3684   0.3800   0.3873   0.3962   0.3999   0.4046   0.4124   0.4286
  0.4323   0.4351   0.4424   0.4523   0.4622   0.4677   0.4713   0.4776
  0.4854   0.4882   0.4969   0.5021   0.5064   0.5092   0.5186   0.5216
  0.5266   0.5281   0.5305   0.5412   0.5419   0.5556   0.5580   0.5593
  0.5612   0.5688   0.5695   0.5825   0.5857   0.5891   0.5953   0.6026
  0.6074   0.6201   0.6299   0.6310   0.6354   0.6493   0.6566   0.6598
  0.6719   0.6752   0.6808   0.6834   0.6918   0.6993   0.7074   0.7120
  0.7239   0.7357   0.7424   0.7517   0.7667   0.7731   0.7765   0.7823
  0.7902   0.8089   0.8150   0.8183   0.8218   0.8407   0.8544   0.8603
  0.8717   0.8754   0.8826   0.8868   0.8985   0.9052   0.9148   0.9301
  0.9437   0.9505   0.9653   0.9764   0.9896   1.0012   1.0217   1.0319
  1.0502   1.0753   1.0987   1.1186   1.1361   1.1523   1.1588   1.1843
  1.1924   1.2090   1.2274   1.2377   1.2583   1.2740   1.2813   1.3060
  1.3145   1.3201   1.3312   1.3356   1.3365   1.3608   1.3667   1.3772
  1.3873   1.3928   1.3950   1.4007   1.4044   1.4128   1.4160   1.4297
  1.4497   1.4503   1.4538   1.4603   1.4754   1.4887   1.4919   1.4967
  1.5013   1.5069   1.5094   1.5152   1.5195   1.5402   1.5476   1.5521
  1.5653   1.5703   1.5738   1.5846   1.5901   1.6075   1.6120   1.6162
  1.6248   1.6340   1.6396   1.6459   1.6580   1.6766   1.6879   1.7024
  1.7184   1.7251   1.7287   1.7364   1.7446   1.7544   1.7610   1.7697
  1.7819   1.7875   1.8151   1.8243   1.8474   1.8519   1.8632   1.8761
  1.8959   1.8987   1.9206   1.9284   1.9389   1.9600   1.9767   1.9893
  2.0070   2.0138   2.0327   2.0468   2.0495   2.0733   2.0841   2.0888
  2.0994   2.1122   2.1341   2.1370   2.1507   2.1845   2.2013   2.2361
  2.2404   2.2489   2.2525   2.2626   2.2770   2.2837   2.2859   2.3010
  2.3082   2.3126   2.3440   2.3692   2.3732   2.3761   2.3898   2.4050
  2.4148   2.4177   2.4378   2.4430   2.4479   2.4689   2.4768   2.4846
  2.4967   2.5104   2.5189   2.5213   2.5417   2.5459   2.5511   2.5549
  2.5690   2.5772   2.5883   2.5911   2.5964   2.6007   2.6036   2.6257
  2.6461   2.6592   2.6646   2.6696   2.6776   2.6890   2.7004   2.7093
  2.7131   2.7208   2.7332   2.7396   2.7549   2.7581   2.7767   2.7836
  2.7894   2.7962   2.8150   2.8350   2.8473   2.8615   2.8880   2.8944
  2.9107   2.9309   2.9413   2.9497   3.0595   3.0781   3.1283   3.1378
  3.1750   3.2469   3.2520   3.2631   3.2866   3.3362   3.3643   3.4044
  3.4204   3.4715   3.5203   3.5522   3.5558   3.6022   3.6203   3.6458
  3.6646   3.6805   3.7200   3.7375   3.7423   3.7565   3.7652   3.7757
  3.8032   3.8288   3.8704   3.9100   3.9338   3.9784   4.0499   4.0808
  4.1076   4.1830   4.2968   4.3030   4.6055   4.6658   4.6960   4.7319
  4.9223   5.0396   5.1943  23.5324  23.5759  23.7204  23.7314  23.7428
 23.7667  23.8038  23.8258  23.8410  23.8901  23.9252  23.9674  23.9807
 24.0368  35.4184  35.4558  35.5169  35.6158
 --------------------------------------------------------------

          Ground-State Mulliken Net Atomic Charges
```

```
   Atom                 Charge (a.u.)
 ----------------------------------------
    1 N                    0.402860
    2 C                    0.139210
    3 C                   -0.014448
    4 C                   -0.149555
    5 C                   -0.171873
    6 C                   -0.127612
    7 C                   -0.764920
    8 C                   -0.300248
    9 N                   -0.096189
   10 C                   -0.414928
   11 N                    0.038133
   12 C                    0.219055
   13 C                   -0.118332
   14 C                    0.147475
   15 C                   -0.380755
   16 C                   -0.081788
   17 C                   -0.608954
   18 N                   -0.452618
   19 H                    0.220902
   20 H                    0.235982
   21 H                    0.228394
   22 H                    0.225132
   23 H                    0.232890
   24 H                    0.249332
   25 H                    0.189429
   26 H                    0.261997
   27 H                    0.250672
   28 H                    0.394713
   29 H                    0.216818
   30 H                    0.239119
   31 H                    0.207629
   32 H                    0.196320
   33 H                    0.172737
   34 H                    0.176808
   35 H                    0.197972
   36 H                    0.170952
   37 H                    0.280482
   38 H                    0.387205
 ----------------------------------------
 Sum of atomic charges =     2.000000

-----------------------------------------------------------------
                  Cartesian Multipole Moments
-----------------------------------------------------------------
   Charge (ESU x 10^10)
                 9.6064
   Dipole Moment (Debye)
        X       6.9676      Y      -0.8920      Z      -4.9692
      Tot       8.6044
   Quadrupole Moments (Debye-Ang)
       XX     -30.7070     XY      27.1513     YY     -65.7601
       XZ     -41.1235     YZ     -18.0563     ZZ     -49.7146
   Traceless Quadrupole Moments (Debye-Ang)
      QXX      54.0606    QYY     -51.0986    QZZ      -2.9621
      QXY      81.4539    QXZ    -123.3706    QYZ     -54.1690
   Octopole Moments (Debye-Ang^2)
      XXX       5.6407    XXY      36.7759    XYY       5.7716
      YYY     -14.1864    XXZ      20.7650    XYZ     -23.0600
      YYZ      22.0588    XZZ      31.3482    YZZ      25.8326
      ZZZ     -79.5066
   Traceless Octopole Moments (Debye-Ang^2)
      XXX    -300.2349    YYY    -648.5954    ZZZ    -862.4538
      XXY     406.3730    XXZ     421.5232    XYY     -41.7069
      XYZ    -345.9006    XZZ     341.9418    YYZ     440.9306
      YZZ     242.2224
   Hexadecapole Moments (Debye-Ang^3)
     XXXX   -3370.9138   XXXY    -208.0594   XXYY    -678.0061
     XYYY    -479.8549   YYYY    -745.9730   XXXZ     897.0210
```

```
      XXYZ     -85.1586   XYYZ     348.6021   YYYZ     328.9092
      XXZZ    -780.1559   XYZZ       3.5140   YYZZ    -542.1183
      XZZZ     852.5190   YZZZ     200.0725   ZZZZ   -1907.0635
    Traceless Hexadecapole Moments (Debye-Ang^3)
      XXXX   -9549.7231   XXXY    8951.7770   XXXZ    -229.1878
      XXYY     663.4267   XXYZ  -15597.9154   XXZZ    8886.2964
      XYYY  -19586.7518   XYYZ    5131.0864   XYZZ   10634.9748
      XZZZ   -4901.8986   YYYY    8400.9994   YYYZ   14566.6881
      YYZZ   -9064.4261   YZZZ    1031.2273   ZZZZ     178.1297
 -----------------------------------------------------------------

--------------------------------------------------
-  Entering drvman on Sun Oct 13 19:38:14 2024  -
--------------------------------------------------

 Calculating analytic gradient of the SCF energy
 Gradient of SCF Energy
           1           2           3           4           5           6
    1   0.0003731  -0.0002967  -0.0003718   0.0003862  -0.0002385   0.0000137
    2   0.0003824   0.0006786  -0.0002821   0.0005355  -0.0001597  -0.0004572
    3   0.0000785   0.0000593  -0.0002773  -0.0001414  -0.0000401   0.0001684
           7           8           9          10          11          12
    1  -0.0002089   0.0003617   0.0001362  -0.0015774   0.0009554  -0.0008078
    2   0.0000104  -0.0003409   0.0003228   0.0014477  -0.0018898   0.0033810
    3  -0.0000219  -0.0005668   0.0003595  -0.0002842  -0.0002162   0.0029051
          13          14          15          16          17          18
    1  -0.0002795   0.0010121   0.0002621   0.0001424  -0.0001764   0.0000988
    2  -0.0013727   0.0005512  -0.0001332   0.0000406  -0.0000398  -0.0024994
    3  -0.0003825  -0.0002428  -0.0006439   0.0005883   0.0000232  -0.0056817
          19          20          21          22          23          24
    1  -0.0001090   0.0000122   0.0000279   0.0001796  -0.0002196   0.0000083
    2  -0.0001986  -0.0000574  -0.0000691  -0.0000226  -0.0001431   0.0001007
    3   0.0002732   0.0002158   0.0002350   0.0002702  -0.0000242   0.0001167
          25          26          27          28          29          30
    1   0.0002111   0.0005374  -0.0006602   0.0002188   0.0000314   0.0002873
    2  -0.0000278  -0.0006041  -0.0000301   0.0002862   0.0000956  -0.0000262
    3   0.0002362  -0.0001679  -0.0003523  -0.0000057  -0.0001073   0.0000361
          31          32          33          34          35          36
    1  -0.0000491   0.0000318   0.0000402   0.0000247   0.0000009  -0.0000283
    2   0.0000186  -0.0000671   0.0000435  -0.0000390   0.0000270   0.0000878
    3  -0.0000100  -0.0000941  -0.0000212   0.0000469  -0.0001219   0.0000511
          37          38
    1  -0.0012514   0.0009216
    2   0.0011276  -0.0006770
    3   0.0027488   0.0009907
 Max gradient component =       5.682E-03
 RMS gradient           =       8.894E-04
 Gradient time:  CPU 159.86 s  wall 268.28 s

--------------------------------------------------
-  Entering optman on Sun Oct 13 19:42:43 2024  -
--------------------------------------------------

 Geometry Optimization Parameters
   NAtoms,    NIC,     NZ,  NCons,   NDum,   NFix, NCnnct, MaxDiis
       38     272       0       0       0       0       0       0

 Cartesian Hessian Update
 Hessian updated using BFGS update


** GEOMETRY OPTIMIZATION IN DELOCALIZED INTERNAL COORDINATES **
   Searching for a Minimum

   Optimization Cycle:   6

                    Coordinates (Angstroms)
     ATOM                X               Y               Z
      1  N         2.2425600189    0.1953540495   -1.5242584234
      2  C         3.1662301528    1.1828319208   -1.6958128209
```

```
     3  C         3.3169966512    1.7198801170   -2.9678407378
     4  C         2.5690036362    1.2557697210   -4.0362205790
     5  C         1.6522750530    0.2268170708   -3.8297194074
     6  C         1.5156060153   -0.2812505066   -2.5652851825
     7  C         4.0129374986    1.6495679927   -0.5508767834
     8  C         2.0365062322   -0.4520570467   -0.1981218964
     9  N        -1.2134774584   -1.8850916698    0.9655296591
    10  C        -2.0540726439   -0.8130096637    0.9643191746
    11  N        -1.6105183848    0.3659667207    0.6163450848
    12  C        -0.3240421945    0.5403489301    0.2777099964
    13  C         0.5862393457   -0.5700092721    0.1975019842
    14  C         0.0760619414   -1.7755625992    0.5709259817
    15  C        -3.4481618589   -0.9996484600    1.4388685027
    16  C        -3.5312911056   -0.7889518901    2.9717511233
    17  C        -4.9646398017   -0.9359819507    3.4713519729
    18  N         0.0247209482    1.7853675261   -0.0431429839
    19  H         4.0462060422    2.5068870137   -3.1080496301
    20  H         2.7031895407    1.6841172493   -5.0223762014
    21  H         1.0569099233   -0.1803284252   -4.6357665228
    22  H         0.8260577757   -1.0819187882   -2.3339860963
    23  H         4.6844795283    2.4323137978   -0.8976809087
    24  H         4.6291797420    0.8387496223   -0.1530103008
    25  H         3.4284596302    2.0679136420    0.2741302318
    26  H         2.5915900529    0.1145937678    0.5459285884
    27  H         2.4903252240   -1.4436017056   -0.2446464737
    28  H        -1.5712901394   -2.7862911482    1.2592288377
    29  H         0.6590757124   -2.6892527907    0.5725782743
    30  H        -4.0735175504   -0.2642742821    0.9313613905
    31  H        -3.8100920560   -1.9986402131    1.1771079998
    32  H        -3.1487424636    0.2080223998    3.2077219165
    33  H        -2.8820482724   -1.5125766179    3.4771826379
    34  H        -5.6235035691   -0.2026194994    3.0010574311
    35  H        -5.0047317965   -0.7774150375    4.5496345045
    36  H        -5.3603560990   -1.9331222583    3.2645994340
    37  H         0.9806556867    2.0977762788   -0.0413616175
    38  H        -0.6747809574    2.4993260047    0.1133218397
   Point Group: c1    Number of degrees of freedom:   108


   Energy is   -763.967803191

 Hessian updated using BFGS update
  internal optimization (0)

108 Hessian modes will be used to form the next step
  Hessian Eigenvalues:
     0.001780     0.002727     0.003473     0.003893     0.005995     0.011433
     0.012906     0.018973     0.019226     0.020151     0.021040     0.022103
     0.022590     0.023523     0.024129     0.024359     0.025281     0.026547
     0.027093     0.028197     0.028520     0.029364     0.033674     0.036142
     0.038294     0.040638     0.042823     0.043590     0.043832     0.046677
     0.048647     0.051294     0.053809     0.055801     0.068114     0.077610
     0.083920     0.095159     0.122098     0.122640     0.126858     0.128368
     0.131827     0.132798     0.137067     0.140855     0.143565     0.144618
     0.146818     0.147529     0.148606     0.151659     0.152283     0.152743
     0.154891     0.160432     0.191565     0.192543     0.206511     0.213938
     0.219138     0.231167     0.238897     0.248593     0.250011     0.256056
     0.271699     0.273948     0.283151     0.286544     0.296651     0.299326
     0.300160     0.300621     0.301251     0.302463     0.303288     0.304129
     0.304880     0.304909     0.305534     0.306492     0.309820     0.312403
     0.320028     0.330832     0.332937     0.333309     0.335886     0.339371
     0.341039     0.351073     0.361729     0.366975     0.380121     0.385397
     0.393961     0.402673     0.407935     0.416824     0.420251     0.434564
     0.441371     0.452352     0.463589     0.522263     0.649078     0.728483

 Minimum search - taking simple RFO step
 Searching for Lamda that Minimizes Along All modes
 Value Taken    Lamda =  -0.00196481
 Calculated Step too Large.  Step scaled by  0.572135
 Step Taken.  Stepsize is  0.300000
```

```
                            Maximum     Tolerance    Cnvgd?
         Gradient          0.002347      0.000800      NO
         Displacement      0.101990      0.001400      NO
         Energy change    -0.001386      0.000228      NO


 New Cartesian Coordinates Obtained by Inverse Iteration

 Displacement from previous Coordinates is:  0.650304
 ----------------------------------------------------------------
             Standard Nuclear Orientation (Angstroms)
    I     Atom           X                Y                Z
 ----------------------------------------------------------------
    1      N       2.2372476370     0.2216033461    -1.5188169661
    2      C       3.1963439433     1.1725359615    -1.7078219905
    3      C       3.3680723812     1.6794576797    -2.9891940709
    4      C       2.6068398223     1.2196809666    -4.0502885865
    5      C       1.6546909295     0.2274025563    -3.8266750793
    6      C       1.4971754837    -0.2500923919    -2.5529664569
    7      C       4.0546866301     1.6329276762    -0.5690421830
    8      C       2.0180132026    -0.3960773174    -0.1782343880
    9      N      -1.2269984601    -1.8813203023     0.9356334744
   10      C      -2.0712221719    -0.8112346511     0.9640560804
   11      N      -1.6363093424     0.3771554805     0.6377509064
   12      C      -0.3520047003     0.5646866229     0.2920622332
   13      C       0.5657446891    -0.5394633082     0.1938577098
   14      C       0.0616893398    -1.7555282724     0.5430623994
   15      C      -3.4611592211    -1.0132503598     1.4456415483
   16      C      -3.5369221394    -0.8095168640     2.9795384736
   17      C      -4.9657071737    -0.9709644937     3.4873554961
   18      N      -0.0090170924     1.8158511471     0.0038552422
   19      H       4.1236002493     2.4387651025    -3.1429207136
   20      H       2.7575288753     1.6240479337    -5.0443577150
   21      H       1.0475583711    -0.1745417990    -4.6266247023
   22      H       0.7792576439    -1.0212587006    -2.3087569338
   23      H       4.7642494673     2.3740997803    -0.9316240907
   24      H       4.6297652160     0.8070190168    -0.1419259056
   25      H       3.4809559291     2.1019100738     0.2361153883
   26      H       2.5380145592     0.2104725622     0.5601087600
   27      H       2.5017423331    -1.3745543664    -0.1844277848
   28      H      -1.5796065564    -2.7906012008     1.2099337508
   29      H       0.6521433742    -2.6643377159     0.5299765046
   30      H      -4.0966903176    -0.2812209555     0.9456298686
   31      H      -3.8153840929    -2.0142838483     1.1814683596
   32      H      -3.1616099286     0.1899093127     3.2167648329
   33      H      -2.8782365174    -1.5295186838     3.4777595424
   34      H      -5.6337279586    -0.2412192685     3.0242750300
   35      H      -5.0006588832    -0.8175491462     4.5666400350
   36      H      -5.3540844914    -1.9706090862     3.2785926951
   37      H       0.9444207518     2.1144420588    -0.1060082512
   38      H      -0.7004017812     2.5351754542     0.1696074868
 ----------------------------------------------------------------
 Nuclear Repulsion Energy =        1298.03464551 hartrees
 There are       65 alpha and       65 beta electrons

--------------------------------------------------
-  Entering fldman on Sun Oct 13 19:42:43 2024  -
--------------------------------------------------

 Applying Cartesian multipole field
    Component          Value
    ---------          -----
     (2,0,0)       1.00000E-12
     (0,2,0)       2.00000E-11
     (0,0,2)      -3.00000E-11
 Nucleus-field energy     =    -0.0000000466 hartrees

--------------------------------------------------
-  Entering gesman on Sun Oct 13 19:42:43 2024  -
```

```
--------------------------------------------------------

 Requested basis set is 6-311+G(d,p)
 There are 188 shells and 516 basis functions
 A cutoff of  1.0D-12 yielded  12452 shell pairs
 There are     99807 function pairs (    106434 Cartesian)
 Smallest overlap matrix eigenvalue = 1.19E-06
 Linear dependence detected in AO basis
 Tighter screening thresholds may be required for diffuse basis sets
 Use S2THRESH > 12 and THRESH = 14 in case of SCF convergence issues
 Number of orthogonalized atomic orbitals = 510
 Maximum deviation from orthogonality = 4.249E-11
 Guess MOs from SCF MO coefficient file
 Reading MOs from coefficient file
 Reading MOs from coefficient file

--------------------------------------------------------
-  Entering scfman on Sun Oct 13 19:42:43 2024  -
--------------------------------------------------------

 Long-range K will be added via erf
 Coulomb attenuation parameter = 0.2 bohr**(-1)
 A restricted hybrid HF-DFT SCF calculation will be
 performed using Pulay DIIS + Geometric Direct Minimization
 Exchange:     0.2220 Hartree-Fock + 1.0000 wB97X-D + LR-HF
 Correlation:  1.0000 wB97X-D
 Using Euler-Maclaurin-Lebedev (75,302) quadrature formula
 Dispersion:   Grimme D
 SCF converges when RMS gradient is below 1.0E-07
 Geometry optimization detected.  Setting ReadMinima to 0
 Setting SaveMinima to 0
 ---------------------------------------
  Cycle       Energy         DIIS Error
 ---------------------------------------
    1    -763.9736605181      5.78E-04
    2    -763.9682079932      8.15E-05
    3    -763.9686274102      4.85E-05
    4    -763.9687022007      2.35E-05
    5    -763.9687245818      6.58E-06
    6    -763.9687264415      2.91E-06
    7    -763.9687267270      7.69E-07
    8    -763.9687267616      3.18E-07
    9    -763.9687267661      9.56E-08 Convergence criterion met
 ---------------------------------------
 SCF time:  CPU 322.16 s  wall 539.64 s
 SCF   energy in the final basis set = -763.96872677
 Total energy in the final basis set = -763.96872677

--------------------------------------------------------
-  Entering anlman on Sun Oct 13 19:51:43 2024  -
--------------------------------------------------------



 --------------------------------------------------------------

                   Orbital Energies (a.u.)
 --------------------------------------------------------------

 Alpha MOs
 -- Occupied --
-14.8174 -14.7894 -14.7415 -14.7077 -10.6781 -10.6626 -10.6469 -10.6414
-10.6343 -10.6266 -10.6056 -10.5851 -10.5743 -10.5722 -10.5512 -10.5246
-10.4951 -10.4492  -1.4156  -1.4055  -1.3499  -1.2772  -1.2314  -1.2016
 -1.1889  -1.1413  -1.0990  -1.0848  -1.0553  -1.0527  -1.0321  -1.0003
 -0.9913  -0.9554  -0.9402  -0.9139  -0.9056  -0.8917  -0.8842  -0.8671
 -0.8572  -0.8426  -0.8375  -0.8197  -0.8091  -0.7963  -0.7910  -0.7804
 -0.7689  -0.7632  -0.7574  -0.7498  -0.7456  -0.7301  -0.7198  -0.7111
 -0.6808  -0.6672  -0.6568  -0.6438  -0.6313  -0.6233  -0.6186  -0.6145
 -0.6097
 -- Virtual --
```

| | | | | | | | |
|---|---|---|---|---|---|---|---|
| -0.2721 | -0.2589 | -0.2439 | -0.2260 | -0.1749 | -0.1626 | -0.1458 | -0.1429 |
| -0.1366 | -0.1224 | -0.1166 | -0.1134 | -0.1082 | -0.1022 | -0.0988 | -0.0964 |
| -0.0895 | -0.0879 | -0.0816 | -0.0787 | -0.0757 | -0.0743 | -0.0715 | -0.0679 |
| -0.0616 | -0.0575 | -0.0556 | -0.0509 | -0.0457 | -0.0445 | -0.0408 | -0.0317 |
| -0.0288 | -0.0255 | -0.0220 | -0.0181 | -0.0166 | -0.0155 | -0.0126 | -0.0067 |
| -0.0043 | -0.0020 | 0.0005 | 0.0050 | 0.0082 | 0.0119 | 0.0134 | 0.0143 |
| 0.0177 | 0.0206 | 0.0248 | 0.0287 | 0.0300 | 0.0328 | 0.0360 | 0.0404 |
| 0.0428 | 0.0462 | 0.0508 | 0.0545 | 0.0566 | 0.0614 | 0.0656 | 0.0667 |
| 0.0695 | 0.0725 | 0.0768 | 0.0792 | 0.0796 | 0.0885 | 0.0905 | 0.0950 |
| 0.0971 | 0.1006 | 0.1024 | 0.1038 | 0.1068 | 0.1136 | 0.1158 | 0.1188 |
| 0.1211 | 0.1261 | 0.1273 | 0.1328 | 0.1386 | 0.1389 | 0.1430 | 0.1493 |
| 0.1529 | 0.1584 | 0.1612 | 0.1652 | 0.1657 | 0.1680 | 0.1721 | 0.1736 |
| 0.1856 | 0.1901 | 0.1943 | 0.1970 | 0.2034 | 0.2129 | 0.2175 | 0.2215 |
| 0.2266 | 0.2378 | 0.2524 | 0.2725 | 0.2847 | 0.2904 | 0.3015 | 0.3068 |
| 0.3197 | 0.3345 | 0.3389 | 0.3452 | 0.3525 | 0.3548 | 0.3601 | 0.3618 |
| 0.3677 | 0.3796 | 0.3875 | 0.3963 | 0.4001 | 0.4057 | 0.4128 | 0.4283 |
| 0.4325 | 0.4366 | 0.4447 | 0.4525 | 0.4619 | 0.4692 | 0.4704 | 0.4804 |
| 0.4847 | 0.4878 | 0.4986 | 0.5021 | 0.5079 | 0.5089 | 0.5171 | 0.5204 |
| 0.5259 | 0.5285 | 0.5303 | 0.5415 | 0.5419 | 0.5560 | 0.5591 | 0.5598 |
| 0.5612 | 0.5694 | 0.5701 | 0.5825 | 0.5848 | 0.5911 | 0.5967 | 0.6027 |
| 0.6102 | 0.6206 | 0.6301 | 0.6323 | 0.6429 | 0.6516 | 0.6559 | 0.6610 |
| 0.6742 | 0.6760 | 0.6816 | 0.6846 | 0.6915 | 0.6994 | 0.7086 | 0.7111 |
| 0.7199 | 0.7360 | 0.7432 | 0.7507 | 0.7683 | 0.7739 | 0.7796 | 0.7822 |
| 0.7863 | 0.8093 | 0.8133 | 0.8161 | 0.8232 | 0.8456 | 0.8558 | 0.8566 |
| 0.8724 | 0.8780 | 0.8814 | 0.8854 | 0.8937 | 0.9054 | 0.9189 | 0.9267 |
| 0.9412 | 0.9543 | 0.9703 | 0.9768 | 0.9942 | 1.0013 | 1.0247 | 1.0352 |
| 1.0513 | 1.0632 | 1.1008 | 1.1161 | 1.1374 | 1.1516 | 1.1561 | 1.1827 |
| 1.1927 | 1.2108 | 1.2223 | 1.2330 | 1.2614 | 1.2737 | 1.2798 | 1.3073 |
| 1.3141 | 1.3192 | 1.3275 | 1.3334 | 1.3379 | 1.3583 | 1.3653 | 1.3766 |
| 1.3867 | 1.3919 | 1.3941 | 1.4011 | 1.4053 | 1.4098 | 1.4158 | 1.4311 |
| 1.4470 | 1.4514 | 1.4537 | 1.4614 | 1.4753 | 1.4885 | 1.4924 | 1.4962 |
| 1.5003 | 1.5068 | 1.5096 | 1.5188 | 1.5227 | 1.5407 | 1.5481 | 1.5543 |
| 1.5615 | 1.5693 | 1.5737 | 1.5809 | 1.5910 | 1.6062 | 1.6127 | 1.6131 |
| 1.6261 | 1.6348 | 1.6391 | 1.6446 | 1.6563 | 1.6774 | 1.6870 | 1.7006 |
| 1.7140 | 1.7255 | 1.7315 | 1.7370 | 1.7434 | 1.7535 | 1.7597 | 1.7707 |
| 1.7822 | 1.7885 | 1.8114 | 1.8234 | 1.8477 | 1.8549 | 1.8628 | 1.8764 |
| 1.8973 | 1.8984 | 1.9218 | 1.9358 | 1.9440 | 1.9600 | 1.9844 | 1.9888 |
| 2.0060 | 2.0169 | 2.0344 | 2.0447 | 2.0531 | 2.0737 | 2.0804 | 2.0914 |
| 2.0982 | 2.1149 | 2.1357 | 2.1406 | 2.1532 | 2.1920 | 2.2029 | 2.2357 |
| 2.2408 | 2.2501 | 2.2528 | 2.2633 | 2.2728 | 2.2812 | 2.2850 | 2.2959 |
| 2.3004 | 2.3131 | 2.3433 | 2.3682 | 2.3699 | 2.3768 | 2.3908 | 2.4019 |
| 2.4133 | 2.4171 | 2.4333 | 2.4427 | 2.4466 | 2.4689 | 2.4768 | 2.4851 |
| 2.4970 | 2.5097 | 2.5170 | 2.5218 | 2.5418 | 2.5479 | 2.5521 | 2.5578 |
| 2.5673 | 2.5777 | 2.5881 | 2.5916 | 2.5957 | 2.6018 | 2.6023 | 2.6249 |
| 2.6456 | 2.6592 | 2.6670 | 2.6694 | 2.6770 | 2.6882 | 2.7026 | 2.7121 |
| 2.7136 | 2.7194 | 2.7345 | 2.7410 | 2.7564 | 2.7612 | 2.7805 | 2.7876 |
| 2.7913 | 2.7950 | 2.8137 | 2.8343 | 2.8481 | 2.8638 | 2.8904 | 2.8970 |
| 2.9090 | 2.9334 | 2.9415 | 2.9515 | 3.0587 | 3.0861 | 3.1283 | 3.1372 |
| 3.1758 | 3.2512 | 3.2535 | 3.2719 | 3.2856 | 3.3364 | 3.3672 | 3.4059 |
| 3.4264 | 3.4718 | 3.5241 | 3.5507 | 3.5541 | 3.6019 | 3.6177 | 3.6467 |
| 3.6648 | 3.6813 | 3.7203 | 3.7377 | 3.7421 | 3.7479 | 3.7656 | 3.7698 |
| 3.7860 | 3.8294 | 3.8698 | 3.9094 | 3.9329 | 3.9772 | 4.0503 | 4.0807 |
| 4.1095 | 4.1838 | 4.2965 | 4.3047 | 4.6066 | 4.6664 | 4.6962 | 4.7287 |
| 4.9261 | 5.0386 | 5.1969 | 23.5285 | 23.5775 | 23.7204 | 23.7309 | 23.7431 |
| 23.7680 | 23.8047 | 23.8255 | 23.8412 | 23.8893 | 23.9257 | 23.9673 | 23.9792 |
| 24.0367 | 35.4176 | 35.4573 | 35.5199 | 35.6176 | | | |

Ground-State Mulliken Net Atomic Charges

| Atom | Charge (a.u.) |
|---|---|
| 1 N | 0.393025 |
| 2 C | 0.128957 |
| 3 C | -0.003813 |
| 4 C | -0.152789 |
| 5 C | -0.166718 |
| 6 C | -0.129114 |
| 7 C | -0.765965 |
| 8 C | -0.305557 |

```
     9  N                     -0.091731
    10  C                     -0.407327
    11  N                      0.028844
    12  C                      0.247579
    13  C                     -0.136601
    14  C                      0.153384
    15  C                     -0.379737
    16  C                     -0.085694
    17  C                     -0.607291
    18  N                     -0.465395
    19  H                      0.221234
    20  H                      0.236007
    21  H                      0.228351
    22  H                      0.226960
    23  H                      0.232862
    24  H                      0.245854
    25  H                      0.193953
    26  H                      0.258862
    27  H                      0.253129
    28  H                      0.393891
    29  H                      0.216249
    30  H                      0.239290
    31  H                      0.205567
    32  H                      0.196555
    33  H                      0.172886
    34  H                      0.176605
    35  H                      0.197867
    36  H                      0.170858
    37  H                      0.292222
    38  H                      0.386742
  ----------------------------------------
  Sum of atomic charges =      2.000000

 -----------------------------------------------------------------
                    Cartesian Multipole Moments
 -----------------------------------------------------------------
    Charge (ESU x 10^10)
                 9.6064
    Dipole Moment (Debye)
         X       6.9981      Y      -0.7558      Z      -5.1541
       Tot       8.7241
    Quadrupole Moments (Debye-Ang)
        XX     -29.9103     XY      27.3969     YY     -66.1455
        XZ     -41.6718     YZ     -17.9382     ZZ     -49.7058
    Traceless Quadrupole Moments (Debye-Ang)
       QXX      56.0307    QYY     -52.6750    QZZ      -3.3558
       QXY      82.1906    QXZ    -125.0154    QYZ     -53.8145
    Octopole Moments (Debye-Ang^2)
       XXX      12.1210    XXY      37.1248    XYY       3.6510
       YYY     -14.7082    XXZ      16.9751    XYZ     -23.5737
       YYZ      22.8804    XZZ      34.0508    YZZ      25.3525
       ZZZ     -81.2998
    Traceless Octopole Moments (Debye-Ang^2)
       XXX    -266.5897    YYY    -650.5445    ZZZ    -846.4986
       XXY     413.5644    XXZ     378.9593    XYY     -94.7035
       XYZ    -353.6055    XZZ     361.2932    YYZ     467.5394
       YZZ     236.9801
    Hexadecapole Moments (Debye-Ang^3)
      XXXX   -3383.0133   XXXY    -204.2377   XXYY    -685.8788
      XYYY    -493.3165   YYYY    -740.3565   XXXZ     889.7575
      XXYZ     -91.5997   XYYZ     355.6188   YYYZ     335.4735
      XXZZ    -773.8678   XYZZ       3.1159   YYZZ    -550.3086
      XZZZ     851.9264   YZZZ     201.5285   ZZZZ   -1909.4904
    Traceless Hexadecapole Moments (Debye-Ang^3)
      XXXX   -9844.7425   XXXY    9804.7640   XXXZ    -954.0831
      XXYY     113.3710   XXYZ  -16291.0740   XXZZ    9731.3715
      XYYY  -20548.5076   XYYZ    5880.4330   XYZZ   10743.7436
      XZZZ   -4926.3498   YYYY    9674.7861   YYYZ   15205.3976
      YYZZ   -9788.1571   YZZZ    1085.6764   ZZZZ      56.7856
 -----------------------------------------------------------------
```

```
-----------------------------------------------
-  Entering drvman on Sun Oct 13 19:51:43 2024  -
-----------------------------------------------

 Calculating analytic gradient of the SCF energy
 Gradient of SCF Energy
           1           2           3           4           5           6
    1   0.0002742  -0.0002148  -0.0002161   0.0002531  -0.0003328   0.0001203
    2   0.0002627   0.0006037  -0.0001746   0.0001845  -0.0002272  -0.0001724
    3  -0.0000736  -0.0003187   0.0000276   0.0001283   0.0001219  -0.0001795
           7           8           9          10          11          12
    1  -0.0002301   0.0003064   0.0000990  -0.0016255   0.0004818  -0.0002413
    2   0.0001308  -0.0004894  -0.0003445   0.0010664  -0.0013849   0.0034078
    3   0.0000545  -0.0002447   0.0005809  -0.0002778  -0.0005983   0.0019267
          13          14          15          16          17          18
    1   0.0006147   0.0003663   0.0001535   0.0000539  -0.0001066  -0.0005083
    2  -0.0007029  -0.0000799  -0.0002247   0.0000154  -0.0000190  -0.0016604
    3  -0.0001959  -0.0001369  -0.0004325   0.0005051   0.0000046  -0.0037701
          19          20          21          22          23          24
    1  -0.0000847   0.0000161   0.0000278   0.0001338  -0.0001148   0.0000569
    2  -0.0001243  -0.0000052  -0.0000801  -0.0000472  -0.0000718   0.0000718
    3   0.0002197   0.0001140   0.0001768   0.0002748  -0.0000014   0.0000228
          25          26          27          28          29          30
    1   0.0001489   0.0004386  -0.0005566   0.0002601   0.0000454   0.0002362
    2  -0.0000538  -0.0004292  -0.0000787   0.0002054   0.0001229   0.0000317
    3   0.0002039  -0.0001695  -0.0002362  -0.0000247  -0.0001485   0.0000412
          31          32          33          34          35          36
    1  -0.0000061   0.0000349   0.0000232   0.0000236  -0.0000190  -0.0000182
    2   0.0000279  -0.0000407   0.0000341  -0.0000147   0.0000303   0.0000673
    3   0.0000006  -0.0001211  -0.0000469   0.0000283  -0.0000703   0.0000442
          37          38
    1  -0.0007271   0.0008335
    2   0.0005651  -0.0004021
    3   0.0015813   0.0009894
 Max gradient component =       3.770E-03
 RMS gradient           =       6.565E-04
 Gradient time:  CPU 155.52 s  wall 262.97 s

-----------------------------------------------
-  Entering optman on Sun Oct 13 19:56:06 2024  -
-----------------------------------------------

 Geometry Optimization Parameters
   NAtoms,    NIC,     NZ,  NCons,   NDum,   NFix, NCnnct, MaxDiis
       38     272       0       0       0       0       0       0

 Cartesian Hessian Update
 Hessian updated using BFGS update


** GEOMETRY OPTIMIZATION IN DELOCALIZED INTERNAL COORDINATES **
   Searching for a Minimum

   Optimization Cycle:   7

                     Coordinates (Angstroms)
     ATOM                X               Y               Z
      1  N         2.2372476370    0.2216033461   -1.5188169661
      2  C         3.1963439433    1.1725359615   -1.7078219905
      3  C         3.3680723812    1.6794576797   -2.9891940709
      4  C         2.6068398223    1.2196809666   -4.0502885865
      5  C         1.6546909295    0.2274025563   -3.8266750793
      6  C         1.4971754837   -0.2500923919   -2.5529664569
      7  C         4.0546866301    1.6329276762   -0.5690421830
      8  C         2.0180132026   -0.3960773174   -0.1782343880
      9  N        -1.2269984601   -1.8813203023    0.9356334744
     10  C        -2.0712221719   -0.8112346511    0.9640560804
     11  N        -1.6363093424    0.3771554805    0.6377509064
     12  C        -0.3520047003    0.5646866229    0.2920622332
```

```
    13  C         0.5657446891   -0.5394633082    0.1938577098
    14  C         0.0616893398   -1.7555282724    0.5430623994
    15  C        -3.4611592211   -1.0132503598    1.4456415483
    16  C        -3.5369221394   -0.8095168640    2.9795384736
    17  C        -4.9657071737   -0.9709644937    3.4873554961
    18  N        -0.0090170924    1.8158511471    0.0038552422
    19  H         4.1236002493    2.4387651025   -3.1429207136
    20  H         2.7575288753    1.6240479337   -5.0443577150
    21  H         1.0475583711   -0.1745417990   -4.6266247023
    22  H         0.7792576439   -1.0212587006   -2.3087569338
    23  H         4.7642494673    2.3740997803   -0.9316240907
    24  H         4.6297652160    0.8070190168   -0.1419259056
    25  H         3.4809559291    2.1019100738    0.2361153883
    26  H         2.5380145592    0.2104725622    0.5601087600
    27  H         2.5017423331   -1.3745543664   -0.1844277848
    28  H        -1.5796065564   -2.7906012008    1.2099337508
    29  H         0.6521433742   -2.6643377159    0.5299765046
    30  H        -4.0966903176   -0.2812209555    0.9456298686
    31  H        -3.8153840929   -2.0142838483    1.1814683596
    32  H        -3.1616099286    0.1899093127    3.2167648329
    33  H        -2.8782365174   -1.5295186838    3.4777595424
    34  H        -5.6337279586   -0.2412192685    3.0242750300
    35  H        -5.0006588832   -0.8175491462    4.5666400350
    36  H        -5.3540844914   -1.9706090862    3.2785926951
    37  H         0.9444207518    2.1144420588   -0.1060082512
    38  H        -0.7004017812    2.5351754542    0.1696074868
   Point Group: c1    Number of degrees of freedom:   108


   Energy is   -763.968726766

 Hessian updated using BFGS update
  internal optimization (0)

108 Hessian modes will be used to form the next step
  Hessian Eigenvalues:
     0.001741    0.002724    0.003322    0.003796    0.005552    0.011558
     0.013492    0.019046    0.019226    0.020316    0.021066    0.022171
     0.022580    0.023524    0.024120    0.024372    0.025308    0.026149
     0.027000    0.028211    0.028671    0.029303    0.033575    0.036135
     0.038293    0.040584    0.042801    0.043583    0.043831    0.046622
     0.048405    0.050862    0.053812    0.055800    0.068107    0.077603
     0.083919    0.095165    0.122100    0.122632    0.126858    0.128369
     0.131842    0.132798    0.136918    0.140827    0.143870    0.144615
     0.146788    0.147531    0.148598    0.151660    0.152374    0.152793
     0.155253    0.160231    0.191430    0.192432    0.206511    0.214027
     0.218962    0.231091    0.238876    0.248627    0.249898    0.255990
     0.271696    0.273947    0.283089    0.286720    0.296622    0.299293
     0.300159    0.300620    0.301251    0.302460    0.303304    0.304129
     0.304881    0.304909    0.305545    0.306490    0.309788    0.312327
     0.319910    0.330888    0.332955    0.333286    0.335785    0.339379
     0.341055    0.351031    0.361898    0.366153    0.380411    0.385462
     0.394016    0.402612    0.408682    0.416830    0.420277    0.434833
     0.441848    0.450811    0.461494    0.519628    0.648661    0.728892

 Minimum search - taking simple RFO step
 Searching for Lamda that Minimizes Along All modes
 Value Taken    Lamda =  -0.00121596
 Calculated Step too Large.  Step scaled by  0.614441
 Step Taken.  Stepsize is  0.300000

                            Maximum     Tolerance    Cnvgd?
         Gradient           0.001657     0.000800      NO
         Displacement       0.115692     0.001400      NO
         Energy change     -0.000924     0.000228      NO


 New Cartesian Coordinates Obtained by Inverse Iteration

 Displacement from previous Coordinates is:  0.720905
```

```
 --------------------------------------------------------------------
             Standard Nuclear Orientation (Angstroms)
    I     Atom           X                Y                Z
 --------------------------------------------------------------------
    1      N       2.2307087010     0.2514832193    -1.5150126803
    2      C       3.2300450957     1.1579291588    -1.7186328201
    3      C       3.4274304889     1.6327152311    -3.0093836403
    4      C       2.6531995122     1.1838571302    -4.0656091127
    5      C       1.6610190213     0.2356475527    -3.8268445902
    6      C       1.4772164365    -0.2101246980    -2.5441677641
    7      C       4.0986963231     1.6078764377    -0.5838832090
    8      C       1.9941805156    -0.3337814187    -0.1609941466
    9      N      -1.2403372090    -1.8738112426     0.9070717495
   10      C      -2.0888328464    -0.8078917694     0.9660753245
   11      N      -1.6653086846     0.3887259185     0.6582196138
   12      C      -0.3858946284     0.5879740316     0.2994670566
   13      C       0.5408836435    -0.5072679272     0.1858359726
   14      C       0.0469087928    -1.7319339627     0.5155685377
   15      C      -3.4734670131    -1.0268422454     1.4561880716
   16      C      -3.5403229871    -0.8311502538     2.9911971192
   17      C      -4.9635057188    -1.0111733667     3.5083273305
   18      N      -0.0490457771     1.8439730992     0.0377109809
   19      H       4.2133235422     2.3584490955    -3.1736305545
   20      H       2.8240136243     1.5624078882    -5.0664975155
   21      H       1.0412301152    -0.1567461820    -4.6220024044
   22      H       0.7265059180    -0.9458111808    -2.2903485884
   23      H       4.8547496229     2.2936334926    -0.9613651322
   24      H       4.6187658809     0.7671298792    -0.1174244288
   25      H       3.5392184374     2.1391065439     0.1923498105
   26      H       2.4726509329     0.3149022003     0.5701563871
   27      H       2.5103025703    -1.2949167347    -0.1225834148
   28      H      -1.5869003623    -2.7903613727     1.1644558661
   29      H       0.6456258969    -2.6350916654     0.4911239513
   30      H      -4.1208460396    -0.2992504649     0.9646608701
   31      H      -3.8192439533    -2.0301872456     1.1895888918
   32      H      -3.1745453301     0.1713107361     3.2304487504
   33      H      -2.8701092319    -1.5462089494     3.4810618060
   34      H      -5.6429257058    -0.2862624882     3.0542253260
   35      H      -4.9922743337    -0.8643484716     4.5887321322
   36      H      -5.3423517207    -2.0138958761     3.2966212802
   37      H       0.8903314834     2.1361811218    -0.1680352320
   38      H      -0.7410950131     2.5637547794     0.1973284052
 --------------------------------------------------------------------
 Nuclear Repulsion Energy =        1296.42453157 hartrees
 There are       65 alpha and       65 beta electrons

------------------------------------------------
-  Entering fldman on Sun Oct 13 19:56:06 2024  -
------------------------------------------------

 Applying Cartesian multipole field
    Component          Value
    ---------          -----
      (2,0,0)        1.00000E-12
      (0,2,0)        2.00000E-11
      (0,0,2)       -3.00000E-11
 Nucleus-field energy     =    -0.0000000471 hartrees

------------------------------------------------
-  Entering gesman on Sun Oct 13 19:56:06 2024  -
------------------------------------------------

 Requested basis set is 6-311+G(d,p)
 There are 188 shells and 516 basis functions
 A cutoff of  1.0D-12 yielded  12433 shell pairs
 There are     99632 function pairs (    106244 Cartesian)
 Smallest overlap matrix eigenvalue = 1.14E-06
 Linear dependence detected in AO basis
 Tighter screening thresholds may be required for diffuse basis sets
 Use S2THRESH > 12 and THRESH = 14 in case of SCF convergence issues
```

```
 Number of orthogonalized atomic orbitals = 510
 Maximum deviation from orthogonality = 4.081E-11
 Guess MOs from SCF MO coefficient file
 Reading MOs from coefficient file
 Reading MOs from coefficient file

--------------------------------------------------
-  Entering scfman on Sun Oct 13 19:56:06 2024  -
--------------------------------------------------

 Long-range K will be added via erf
 Coulomb attenuation parameter = 0.2 bohr**(-1)
 A restricted hybrid HF-DFT SCF calculation will be
 performed using Pulay DIIS + Geometric Direct Minimization
 Exchange:     0.2220 Hartree-Fock + 1.0000 wB97X-D + LR-HF
 Correlation:  1.0000 wB97X-D
 Using Euler-Maclaurin-Lebedev (75,302) quadrature formula
 Dispersion:   Grimme D
 SCF converges when RMS gradient is below 1.0E-07
 Geometry optimization detected.  Setting ReadMinima to 0
 Setting SaveMinima to 0
 ---------------------------------------
  Cycle       Energy         DIIS Error
 ---------------------------------------
    1    -763.9699992464      6.15E-04
    2    -763.9687195564      8.55E-05
    3    -763.9692162950      4.76E-05
    4    -763.9692900695      2.26E-05
    5    -763.9693109088      6.56E-06
    6    -763.9693127348      2.28E-06
    7    -763.9693129289      8.09E-07
    8    -763.9693129623      3.02E-07
    9    -763.9693129663      7.31E-08 Convergence criterion met
 ---------------------------------------
 SCF time:  CPU 320.72 s  wall 528.71 s
 SCF   energy in the final basis set = -763.96931297
 Total energy in the final basis set = -763.96931297

--------------------------------------------------
-  Entering anlman on Sun Oct 13 20:04:55 2024  -
--------------------------------------------------



 --------------------------------------------------------------


                   Orbital Energies (a.u.)
 --------------------------------------------------------------


 Alpha MOs
 -- Occupied --
-14.8177 -14.7889 -14.7424 -14.7072 -10.6779 -10.6623 -10.6468 -10.6414
-10.6344 -10.6268 -10.6052 -10.5851 -10.5745 -10.5723 -10.5503 -10.5243
-10.4945 -10.4488  -1.4153  -1.4053  -1.3502  -1.2774  -1.2314  -1.2016
 -1.1886  -1.1413  -1.0990  -1.0843  -1.0554  -1.0527  -1.0316  -1.0000
 -0.9911  -0.9561  -0.9400  -0.9146  -0.9060  -0.8915  -0.8839  -0.8676
 -0.8577  -0.8425  -0.8376  -0.8197  -0.8090  -0.7967  -0.7904  -0.7795
 -0.7689  -0.7629  -0.7569  -0.7497  -0.7456  -0.7300  -0.7195  -0.7109
 -0.6804  -0.6668  -0.6570  -0.6439  -0.6310  -0.6229  -0.6182  -0.6142
 -0.6095
 -- Virtual --
 -0.2719  -0.2581  -0.2436  -0.2264  -0.1752  -0.1628  -0.1460  -0.1430
 -0.1369  -0.1220  -0.1158  -0.1133  -0.1078  -0.1019  -0.0990  -0.0967
 -0.0894  -0.0881  -0.0818  -0.0775  -0.0756  -0.0743  -0.0708  -0.0677
 -0.0619  -0.0575  -0.0546  -0.0511  -0.0454  -0.0444  -0.0424  -0.0320
 -0.0287  -0.0261  -0.0231  -0.0180  -0.0164  -0.0156  -0.0116  -0.0068
 -0.0032  -0.0020  -0.0000   0.0053   0.0089   0.0120   0.0135   0.0142
  0.0173   0.0202   0.0252   0.0290   0.0300   0.0323   0.0368   0.0395
  0.0440   0.0472   0.0512   0.0542   0.0570   0.0618   0.0661   0.0668
  0.0699   0.0727   0.0771   0.0794   0.0799   0.0884   0.0896   0.0951
  0.0977   0.1004   0.1027   0.1038   0.1069   0.1133   0.1155   0.1183
```

| | | | | | | | |
|---|---|---|---|---|---|---|---|
| 0.1212 | 0.1256 | 0.1271 | 0.1311 | 0.1384 | 0.1397 | 0.1416 | 0.1501 |
| 0.1532 | 0.1597 | 0.1619 | 0.1648 | 0.1659 | 0.1667 | 0.1725 | 0.1752 |
| 0.1858 | 0.1915 | 0.1949 | 0.1980 | 0.2037 | 0.2144 | 0.2170 | 0.2227 |
| 0.2265 | 0.2377 | 0.2528 | 0.2742 | 0.2856 | 0.2910 | 0.2992 | 0.3052 |
| 0.3179 | 0.3345 | 0.3391 | 0.3436 | 0.3542 | 0.3548 | 0.3599 | 0.3613 |
| 0.3680 | 0.3793 | 0.3874 | 0.3958 | 0.3992 | 0.4091 | 0.4130 | 0.4279 |
| 0.4341 | 0.4369 | 0.4458 | 0.4532 | 0.4606 | 0.4696 | 0.4706 | 0.4815 |
| 0.4843 | 0.4893 | 0.5008 | 0.5021 | 0.5075 | 0.5097 | 0.5163 | 0.5189 |
| 0.5248 | 0.5299 | 0.5310 | 0.5410 | 0.5431 | 0.5559 | 0.5593 | 0.5606 |
| 0.5624 | 0.5697 | 0.5707 | 0.5822 | 0.5849 | 0.5931 | 0.5972 | 0.6037 |
| 0.6104 | 0.6209 | 0.6297 | 0.6338 | 0.6484 | 0.6523 | 0.6563 | 0.6619 |
| 0.6749 | 0.6764 | 0.6822 | 0.6869 | 0.6922 | 0.7002 | 0.7057 | 0.7112 |
| 0.7184 | 0.7346 | 0.7437 | 0.7497 | 0.7643 | 0.7756 | 0.7795 | 0.7831 |
| 0.7899 | 0.8082 | 0.8112 | 0.8139 | 0.8265 | 0.8489 | 0.8551 | 0.8568 |
| 0.8726 | 0.8780 | 0.8789 | 0.8813 | 0.8937 | 0.9060 | 0.9233 | 0.9289 |
| 0.9397 | 0.9571 | 0.9748 | 0.9772 | 0.9935 | 1.0073 | 1.0274 | 1.0374 |
| 1.0417 | 1.0643 | 1.1057 | 1.1131 | 1.1393 | 1.1490 | 1.1533 | 1.1801 |
| 1.1925 | 1.2036 | 1.2217 | 1.2311 | 1.2644 | 1.2740 | 1.2829 | 1.3071 |
| 1.3145 | 1.3199 | 1.3246 | 1.3316 | 1.3389 | 1.3537 | 1.3654 | 1.3759 |
| 1.3858 | 1.3928 | 1.3936 | 1.4014 | 1.4018 | 1.4095 | 1.4168 | 1.4321 |
| 1.4448 | 1.4499 | 1.4553 | 1.4628 | 1.4735 | 1.4874 | 1.4926 | 1.4976 |
| 1.4999 | 1.5076 | 1.5093 | 1.5203 | 1.5307 | 1.5415 | 1.5474 | 1.5551 |
| 1.5598 | 1.5686 | 1.5733 | 1.5760 | 1.5944 | 1.6045 | 1.6110 | 1.6148 |
| 1.6276 | 1.6351 | 1.6403 | 1.6433 | 1.6551 | 1.6759 | 1.6859 | 1.6990 |
| 1.7117 | 1.7258 | 1.7341 | 1.7385 | 1.7433 | 1.7528 | 1.7585 | 1.7735 |
| 1.7837 | 1.7902 | 1.8091 | 1.8226 | 1.8476 | 1.8572 | 1.8638 | 1.8768 |
| 1.8962 | 1.9011 | 1.9224 | 1.9453 | 1.9480 | 1.9613 | 1.9845 | 1.9946 |
| 2.0049 | 2.0224 | 2.0357 | 2.0417 | 2.0583 | 2.0726 | 2.0755 | 2.0898 |
| 2.0980 | 2.1147 | 2.1361 | 2.1450 | 2.1555 | 2.1982 | 2.2082 | 2.2355 |
| 2.2419 | 2.2504 | 2.2532 | 2.2636 | 2.2692 | 2.2771 | 2.2847 | 2.2905 |
| 2.2945 | 2.3136 | 2.3446 | 2.3645 | 2.3675 | 2.3778 | 2.3923 | 2.4034 |
| 2.4130 | 2.4164 | 2.4299 | 2.4425 | 2.4465 | 2.4672 | 2.4772 | 2.4856 |
| 2.4971 | 2.5082 | 2.5163 | 2.5234 | 2.5420 | 2.5493 | 2.5532 | 2.5618 |
| 2.5657 | 2.5774 | 2.5882 | 2.5919 | 2.5949 | 2.6005 | 2.6068 | 2.6251 |
| 2.6442 | 2.6575 | 2.6686 | 2.6710 | 2.6765 | 2.6872 | 2.7032 | 2.7129 |
| 2.7152 | 2.7180 | 2.7340 | 2.7431 | 2.7567 | 2.7636 | 2.7848 | 2.7883 |
| 2.7925 | 2.7992 | 2.8108 | 2.8336 | 2.8486 | 2.8660 | 2.8924 | 2.8989 |
| 2.9109 | 2.9337 | 2.9432 | 2.9579 | 3.0583 | 3.0946 | 3.1287 | 3.1369 |
| 3.1768 | 3.2501 | 3.2549 | 3.2797 | 3.2859 | 3.3348 | 3.3700 | 3.4059 |
| 3.4322 | 3.4739 | 3.5272 | 3.5487 | 3.5537 | 3.6011 | 3.6153 | 3.6469 |
| 3.6655 | 3.6820 | 3.7205 | 3.7334 | 3.7380 | 3.7446 | 3.7659 | 3.7681 |
| 3.7813 | 3.8296 | 3.8693 | 3.9095 | 3.9328 | 3.9775 | 4.0509 | 4.0808 |
| 4.1113 | 4.1844 | 4.2959 | 4.3077 | 4.6075 | 4.6670 | 4.6962 | 4.7272 |
| 4.9281 | 5.0377 | 5.1999 | 23.5256 | 23.5792 | 23.7207 | 23.7312 | 23.7444 |
| 23.7712 | 23.8056 | 23.8255 | 23.8414 | 23.8879 | 23.9260 | 23.9658 | 23.9816 |
| 24.0354 | 35.4170 | 35.4585 | 35.5231 | 35.6205 | | | |

Ground-State Mulliken Net Atomic Charges

| Atom | Charge (a.u.) |
|---|---|
| 1 N | 0.379840 |
| 2 C | 0.119617 |
| 3 C | 0.004967 |
| 4 C | -0.154215 |
| 5 C | -0.161958 |
| 6 C | -0.130165 |
| 7 C | -0.765731 |
| 8 C | -0.309456 |
| 9 N | -0.088692 |
| 10 C | -0.399326 |
| 11 N | 0.021096 |
| 12 C | 0.273172 |
| 13 C | -0.154400 |
| 14 C | 0.160428 |
| 15 C | -0.377996 |
| 16 C | -0.090224 |
| 17 C | -0.605561 |
| 18 N | -0.480648 |

```
   19 H                     0.221432
   20 H                     0.235883
   21 H                     0.228260
   22 H                     0.229264
   23 H                     0.232696
   24 H                     0.240830
   25 H                     0.200368
   26 H                     0.255614
   27 H                     0.255696
   28 H                     0.393025
   29 H                     0.215935
   30 H                     0.239581
   31 H                     0.203819
   32 H                     0.197028
   33 H                     0.172994
   34 H                     0.176507
   35 H                     0.197804
   36 H                     0.170711
   37 H                     0.306580
   38 H                     0.385226
 ----------------------------------------
 Sum of atomic charges =     2.000000

 -----------------------------------------------------------------
                    Cartesian Multipole Moments
 -----------------------------------------------------------------
    Charge (ESU x 10^10)
                 9.6064
    Dipole Moment (Debye)
         X       6.9881      Y      -0.6326      Z      -5.3267
       Tot       8.8095
    Quadrupole Moments (Debye-Ang)
        XX     -29.0189     XY      27.5376     YY     -66.6918
        XZ     -42.2352     YZ     -17.9283     ZZ     -49.6404
    Traceless Quadrupole Moments (Debye-Ang)
       QXX      58.2943    QYY     -54.7242    QZZ      -3.5701
       QXY      82.6127    QXZ    -126.7057    QYZ     -53.7850
    Octopole Moments (Debye-Ang^2)
       XXX      19.2029    XXY      37.2988    XYY       1.0294
       YYY     -15.5304    XXZ      12.9216    XYZ     -23.7814
       YYZ      23.8777    XZZ      36.8643    YZZ      24.6201
       ZZZ     -82.5944
    Traceless Octopole Moments (Debye-Ang^2)
       XXX    -225.8264    YYY    -650.4525    ZZZ    -826.7601
       XXY     420.3167    XXZ     331.2087    XYY    -155.8485
       XYZ    -356.7213    XZZ     381.6749    YYZ     495.5514
       YZZ     230.1358
    Hexadecapole Moments (Debye-Ang^3)
      XXXX   -3393.6858   XXXY    -200.2166   XXYY    -695.3993
      XYYY    -508.2248   YYYY    -733.9061   XXXZ     881.4184
      XXYZ     -97.6494   XYYZ     364.6173   YYYZ     343.2401
      XXZZ    -766.0333   XYZZ       2.8134   YYZZ    -559.5237
      XZZZ     851.0419   YZZZ     203.9855   ZZZZ   -1915.1538
    Traceless Hexadecapole Moments (Debye-Ang^3)
      XXXX  -10138.2802   XXXY   10730.5156   XXXZ   -1819.5565
      XXYY    -611.6869   XXYZ  -16982.0454   XXZZ   10749.9671
      XYYY  -21610.3384   XYYZ    6828.6544   XYZZ   10879.8228
      XZZZ   -5009.0979   YYYY   11172.5509   YYYZ   15853.6289
      YYZZ  -10560.8640   YZZZ    1128.4165   ZZZZ    -189.1031
 -----------------------------------------------------------------

--------------------------------------------------
-  Entering drvman on Sun Oct 13 20:04:55 2024  -
--------------------------------------------------

 Calculating analytic gradient of the SCF energy
 Gradient of SCF Energy
            1           2           3           4           5           6
    1   0.0000236  -0.0000091  -0.0003267   0.0002983   0.0000021  -0.0000899
    2   0.0003259   0.0002399  -0.0001106   0.0002126   0.0001483  -0.0004664
```

```
    3   0.0000544   0.0002031  -0.0005231  -0.0001344  -0.0003206   0.0004435
            7           8           9          10          11          12
    1  -0.0002254   0.0001091   0.0001540  -0.0008232   0.0001372   0.0000189
    2   0.0002136  -0.0005337  -0.0004127   0.0014747  -0.0013761   0.0029620
    3   0.0001423  -0.0000207   0.0004917  -0.0001290  -0.0004435   0.0005238
           13          14          15          16          17          18
    1   0.0006498   0.0001807   0.0001016   0.0000821  -0.0000835  -0.0011019
    2  -0.0010556   0.0005502  -0.0002020   0.0000469  -0.0000220  -0.0018317
    3   0.0001637  -0.0003385  -0.0003881   0.0003975  -0.0000364  -0.0015117
           19          20          21          22          23          24
    1  -0.0000661  -0.0000091   0.0000171   0.0001037  -0.0000564   0.0000715
    2  -0.0000426  -0.0000037  -0.0000753  -0.0000405  -0.0000886   0.0000698
    3   0.0001348   0.0001319   0.0000801   0.0002503  -0.0000270  -0.0000396
           25          26          27          28          29          30
    1   0.0001223   0.0002207  -0.0004082   0.0002220   0.0000393   0.0001474
    2   0.0000504  -0.0003300  -0.0000471   0.0001264   0.0001358   0.0000429
    3   0.0001394  -0.0001360  -0.0001247  -0.0000534  -0.0001345   0.0000384
           31          32          33          34          35          36
    1  -0.0000026   0.0000336   0.0000087   0.0000144  -0.0000076  -0.0000224
    2   0.0000206  -0.0000261   0.0000227  -0.0000057   0.0000270   0.0000524
    3  -0.0000074  -0.0001204  -0.0000475   0.0000169  -0.0000552   0.0000375
           37          38
    1  -0.0002906   0.0007647
    2   0.0001280  -0.0001799
    3   0.0006436   0.0006990
 Max gradient component =       2.962E-03
 RMS gradient           =       4.934E-04
 Gradient time:  CPU 160.67 s  wall 271.34 s

--------------------------------------------------
-  Entering optman on Sun Oct 13 20:09:26 2024  -
--------------------------------------------------

 Geometry Optimization Parameters
   NAtoms,    NIC,     NZ,  NCons,   NDum,   NFix, NCnnct, MaxDiis
       38     272       0       0       0       0       0       0

 Cartesian Hessian Update
 Hessian updated using BFGS update


** GEOMETRY OPTIMIZATION IN DELOCALIZED INTERNAL COORDINATES **
   Searching for a Minimum

   Optimization Cycle:   8

                     Coordinates (Angstroms)
     ATOM              X               Y               Z
      1  N         2.2307087010    0.2514832193   -1.5150126803
      2  C         3.2300450957    1.1579291588   -1.7186328201
      3  C         3.4274304889    1.6327152311   -3.0093836403
      4  C         2.6531995122    1.1838571302   -4.0656091127
      5  C         1.6610190213    0.2356475527   -3.8268445902
      6  C         1.4772164365   -0.2101246980   -2.5441677641
      7  C         4.0986963231    1.6078764377   -0.5838832090
      8  C         1.9941805156   -0.3337814187   -0.1609941466
      9  N        -1.2403372090   -1.8738112426    0.9070717495
     10  C        -2.0888328464   -0.8078917694    0.9660753245
     11  N        -1.6653086846    0.3887259185    0.6582196138
     12  C        -0.3858946284    0.5879740316    0.2994670566
     13  C         0.5408836435   -0.5072679272    0.1858359726
     14  C         0.0469087928   -1.7319339627    0.5155685377
     15  C        -3.4734670131   -1.0268422454    1.4561880716
     16  C        -3.5403229871   -0.8311502538    2.9911971192
     17  C        -4.9635057188   -1.0111733667    3.5083273305
     18  N        -0.0490457771    1.8439730992    0.0377109809
     19  H         4.2133235422    2.3584490955   -3.1736305545
     20  H         2.8240136243    1.5624078882   -5.0664975155
     21  H         1.0412301152   -0.1567461820   -4.6220024044
     22  H         0.7265059180   -0.9458111808   -2.2903485884
```

```
    23  H         4.8547496229    2.2936334926   -0.9613651322
    24  H         4.6187658809    0.7671298792   -0.1174244288
    25  H         3.5392184374    2.1391065439    0.1923498105
    26  H         2.4726509329    0.3149022003    0.5701563871
    27  H         2.5103025703   -1.2949167347   -0.1225834148
    28  H        -1.5869003623   -2.7903613727    1.1644558661
    29  H         0.6456258969   -2.6350916654    0.4911239513
    30  H        -4.1208460396   -0.2992504649    0.9646608701
    31  H        -3.8192439533   -2.0301872456    1.1895888918
    32  H        -3.1745453301    0.1713107361    3.2304487504
    33  H        -2.8701092319   -1.5462089494    3.4810618060
    34  H        -5.6429257058   -0.2862624882    3.0542253260
    35  H        -4.9922743337   -0.8643484716    4.5887321322
    36  H        -5.3423517207   -2.0138958761    3.2966212802
    37  H         0.8903314834    2.1361811218   -0.1680352320
    38  H        -0.7410950131    2.5637547794    0.1973284052
   Point Group: c1    Number of degrees of freedom:   108


   Energy is   -763.969312966

 Hessian updated using BFGS update
  internal optimization (0)

108 Hessian modes will be used to form the next step
  Hessian Eigenvalues:
     0.001846    0.002696    0.003019    0.003774    0.005279    0.011691
     0.013961    0.019050    0.019240    0.020414    0.021110    0.022180
     0.022570    0.023528    0.024125    0.024380    0.025309    0.026109
     0.026985    0.028210    0.028941    0.029242    0.033832    0.036143
     0.038290    0.040472    0.042779    0.043574    0.043830    0.046561
     0.047822    0.050557    0.053812    0.055814    0.068019    0.077576
     0.083918    0.095166    0.122102    0.122623    0.126858    0.128375
     0.131916    0.132799    0.136950    0.140796    0.143940    0.145180
     0.146763    0.147534    0.148690    0.151659    0.152424    0.153009
     0.155878    0.160264    0.191352    0.192627    0.206520    0.214037
     0.218753    0.230665    0.238837    0.248457    0.250228    0.256166
     0.271714    0.273950    0.283405    0.286845    0.296600    0.299246
     0.300160    0.300618    0.301256    0.302456    0.303310    0.304130
     0.304882    0.304915    0.305544    0.306489    0.309725    0.312327
     0.319881    0.330935    0.332953    0.333485    0.335673    0.339376
     0.341154    0.351857    0.362594    0.364783    0.379513    0.385941
     0.394293    0.402361    0.408950    0.416860    0.420534    0.434952
     0.438854    0.449757    0.461285    0.517951    0.650708    0.730093

 Minimum search - taking simple RFO step
 Searching for Lamda that Minimizes Along All modes
 Value Taken    Lamda =  -0.00059649
 Calculated Step too Large.  Step scaled by  0.789337
 Step Taken.  Stepsize is  0.300000

                           Maximum     Tolerance    Cnvgd?
        Gradient           0.002168      0.000800      NO
        Displacement       0.122836      0.001400      NO
        Energy change     -0.000586      0.000228      NO


 New Cartesian Coordinates Obtained by Inverse Iteration

 Displacement from previous Coordinates is:  0.827039
 ----------------------------------------------------------------
             Standard Nuclear Orientation (Angstroms)
    I     Atom           X                Y                Z
 ----------------------------------------------------------------
    1      N       2.2232927873     0.2868115872    -1.5140624904
    2      C       3.2687224393     1.1381340859    -1.7280209403
    3      C       3.4997504506     1.5767227569    -3.0249249069
    4      C       2.7100759441     1.1468798887    -4.0780475689
    5      C       1.6691533463     0.2554480932    -3.8292408778
    6      C       1.4543382460    -0.1552381469    -2.5404445859
```

```
    7      C        4.1471556776     1.5688964087    -0.5938751170
    8      C        1.9642415222    -0.2619335230    -0.1471090541
    9      N       -1.2532096832    -1.8650027193     0.8805366806
   10      C       -2.1095453273    -0.8073479283     0.9665093924
   11      N       -1.7012767489     0.3993178182     0.6728506016
   12      C       -0.4281570639     0.6135804428     0.2989003173
   13      C        0.5109482983    -0.4690168379     0.1741600280
   14      C        0.0324443007    -1.7042211624     0.4910268294
   15      C       -3.4865635622    -1.0454836207     1.4703523474
   16      C       -3.5407025259    -0.8546599975     3.0059461268
   17      C       -4.9560410520    -1.0547690704     3.5368174897
   18      N       -0.1020092787     1.8754337177     0.0496237016
   19      H        4.3222786808     2.2586935368    -3.1974359758
   20      H        2.9064350456     1.4965227170    -5.0850071137
   21      H        1.0350653181    -0.1190757034    -4.6217536267
   22      H        0.6645515609    -0.8461907861    -2.2805295182
   23      H        4.9459017815     2.2006190842    -0.9779115237
   24      H        4.6098234666     0.7144577823    -0.0933339930
   25      H        3.6044260621     2.1549474385     0.1547166367
   26      H        2.3966010407     0.4295568061     0.5743529009
   27      H        2.5133978422    -1.2017086411    -0.0603439653
   28      H       -1.5906585930    -2.7884680268     1.1256934369
   29      H        0.6428066516    -2.5994384263     0.4601563847
   30      H       -4.1492485034    -0.3258497790     0.9872384615
   31      H       -3.8217238894    -2.0527286324     1.2047896607
   32      H       -3.1857737770     0.1517781491     3.2449795674
   33      H       -2.8564933743    -1.5624057338     3.4870669654
   34      H       -5.6490956046    -0.3363199609     3.0930135107
   35      H       -4.9755883944    -0.9134301664     4.6182233039
   36      H       -5.3243793942    -2.0612393949     3.3240372181
   37      H        0.8142018406     2.1684429566    -0.2418768410
   38      H       -0.8051455306     2.5882849877     0.1929265370
 ----------------------------------------------------------------
 Nuclear Repulsion Energy =        1294.60330131 hartrees
 There are       65 alpha and       65 beta electrons

--------------------------------------------------
-  Entering fldman on Sun Oct 13 20:09:26 2024  -
--------------------------------------------------

 Applying Cartesian multipole field
    Component          Value
    ---------          -----
     (2,0,0)        1.00000E-12
     (0,2,0)        2.00000E-11
     (0,0,2)       -3.00000E-11
 Nucleus-field energy     =    -0.0000000477 hartrees

--------------------------------------------------
-  Entering gesman on Sun Oct 13 20:09:26 2024  -
--------------------------------------------------

 Requested basis set is 6-311+G(d,p)
 There are 188 shells and 516 basis functions
 A cutoff of  1.0D-12 yielded  12402 shell pairs
 There are     99349 function pairs (    105927 Cartesian)
 Smallest overlap matrix eigenvalue = 1.10E-06
 Linear dependence detected in AO basis
 Tighter screening thresholds may be required for diffuse basis sets
 Use S2THRESH > 12 and THRESH = 14 in case of SCF convergence issues
 Number of orthogonalized atomic orbitals = 510
 Maximum deviation from orthogonality = 2.007E-11
 Guess MOs from SCF MO coefficient file
 Reading MOs from coefficient file
 Reading MOs from coefficient file

--------------------------------------------------
-  Entering scfman on Sun Oct 13 20:09:27 2024  -
--------------------------------------------------
```

```
 Long-range K will be added via erf
 Coulomb attenuation parameter = 0.2 bohr**(-1)
 A restricted hybrid HF-DFT SCF calculation will be
 performed using Pulay DIIS + Geometric Direct Minimization
 Exchange:     0.2220 Hartree-Fock + 1.0000 wB97X-D + LR-HF
 Correlation:  1.0000 wB97X-D
 Using Euler-Maclaurin-Lebedev (75,302) quadrature formula
 Dispersion:   Grimme D
 SCF converges when RMS gradient is below 1.0E-07
 Geometry optimization detected.  Setting ReadMinima to 0
 Setting SaveMinima to 0
 ---------------------------------------
  Cycle       Energy         DIIS Error
 ---------------------------------------
    1    -763.9598398702      6.92E-04
    2    -763.9688899542      9.58E-05
    3    -763.9694927568      6.06E-05
    4    -763.9696048704      2.64E-05
    5    -763.9696327778      8.15E-06
    6    -763.9696353139      3.48E-06
    7    -763.9696357430      8.87E-07
    8    -763.9696357865      3.64E-07
    9    -763.9696357922      9.28E-08 Convergence criterion met
 ---------------------------------------
 SCF time:  CPU 316.00 s  wall 531.58 s
 SCF   energy in the final basis set = -763.96963579
 Total energy in the final basis set = -763.96963579

--------------------------------------------------
-  Entering anlman on Sun Oct 13 20:18:18 2024  -
--------------------------------------------------



 --------------------------------------------------------------

                   Orbital Energies (a.u.)
 --------------------------------------------------------------


 Alpha MOs
 -- Occupied --
-14.8177 -14.7892 -14.7424 -14.7074 -10.6777 -10.6625 -10.6473 -10.6408
-10.6346 -10.6267 -10.6052 -10.5849 -10.5742 -10.5720 -10.5491 -10.5243
-10.4943 -10.4487  -1.4152  -1.4049  -1.3500  -1.2773  -1.2311  -1.2016
 -1.1883  -1.1414  -1.0989  -1.0836  -1.0556  -1.0527  -1.0307  -0.9999
 -0.9908  -0.9562  -0.9398  -0.9145  -0.9063  -0.8913  -0.8834  -0.8676
 -0.8582  -0.8422  -0.8375  -0.8194  -0.8088  -0.7971  -0.7898  -0.7784
 -0.7691  -0.7627  -0.7563  -0.7492  -0.7452  -0.7298  -0.7190  -0.7108
 -0.6803  -0.6662  -0.6570  -0.6440  -0.6309  -0.6225  -0.6181  -0.6141
 -0.6096
 -- Virtual --
 -0.2715  -0.2581  -0.2439  -0.2263  -0.1757  -0.1630  -0.1466  -0.1430
 -0.1371  -0.1216  -0.1148  -0.1131  -0.1077  -0.1015  -0.0991  -0.0968
 -0.0895  -0.0881  -0.0817  -0.0763  -0.0752  -0.0744  -0.0700  -0.0671
 -0.0621  -0.0576  -0.0537  -0.0515  -0.0452  -0.0442  -0.0433  -0.0323
 -0.0284  -0.0279  -0.0237  -0.0181  -0.0164  -0.0156  -0.0103  -0.0070
 -0.0022  -0.0017  -0.0004   0.0057   0.0094   0.0115   0.0127   0.0150
  0.0175   0.0199   0.0258   0.0291   0.0302   0.0318   0.0367   0.0397
  0.0455   0.0485   0.0517   0.0540   0.0571   0.0623   0.0658   0.0680
  0.0706   0.0725   0.0772   0.0789   0.0803   0.0873   0.0909   0.0952
  0.0983   0.1004   0.1029   0.1037   0.1069   0.1122   0.1153   0.1177
  0.1217   0.1243   0.1276   0.1304   0.1381   0.1397   0.1417   0.1508
  0.1537   0.1602   0.1612   0.1638   0.1663   0.1674   0.1729   0.1763
  0.1864   0.1926   0.1953   0.1979   0.2034   0.2156   0.2167   0.2229
  0.2283   0.2381   0.2538   0.2747   0.2855   0.2917   0.2960   0.3040
  0.3167   0.3341   0.3389   0.3424   0.3542   0.3557   0.3603   0.3607
  0.3684   0.3790   0.3870   0.3946   0.3982   0.4111   0.4158   0.4274
  0.4351   0.4374   0.4455   0.4545   0.4591   0.4688   0.4714   0.4791
  0.4859   0.4907   0.5002   0.5026   0.5084   0.5109   0.5155   0.5182
  0.5232   0.5296   0.5342   0.5400   0.5459   0.5558   0.5588   0.5601
  0.5653   0.5695   0.5718   0.5819   0.5848   0.5946   0.5972   0.6036
```

| | | | | | | | |
|---|---|---|---|---|---|---|---|
| 0.6086 | 0.6207 | 0.6290 | 0.6339 | 0.6492 | 0.6519 | 0.6576 | 0.6618 |
| 0.6751 | 0.6770 | 0.6828 | 0.6878 | 0.6928 | 0.7005 | 0.7036 | 0.7120 |
| 0.7182 | 0.7310 | 0.7450 | 0.7492 | 0.7598 | 0.7707 | 0.7813 | 0.7872 |
| 0.7938 | 0.8045 | 0.8102 | 0.8142 | 0.8284 | 0.8496 | 0.8525 | 0.8582 |
| 0.8689 | 0.8747 | 0.8760 | 0.8838 | 0.8958 | 0.9068 | 0.9193 | 0.9377 |
| 0.9408 | 0.9601 | 0.9730 | 0.9810 | 0.9907 | 1.0049 | 1.0228 | 1.0294 |
| 1.0487 | 1.0750 | 1.1099 | 1.1134 | 1.1420 | 1.1449 | 1.1519 | 1.1777 |
| 1.1921 | 1.1989 | 1.2204 | 1.2337 | 1.2662 | 1.2745 | 1.2902 | 1.3057 |
| 1.3145 | 1.3199 | 1.3246 | 1.3317 | 1.3403 | 1.3499 | 1.3660 | 1.3748 |
| 1.3862 | 1.3930 | 1.3935 | 1.3987 | 1.4019 | 1.4086 | 1.4173 | 1.4329 |
| 1.4422 | 1.4494 | 1.4568 | 1.4640 | 1.4703 | 1.4866 | 1.4919 | 1.4975 |
| 1.5012 | 1.5081 | 1.5096 | 1.5209 | 1.5337 | 1.5424 | 1.5458 | 1.5544 |
| 1.5625 | 1.5685 | 1.5694 | 1.5751 | 1.5971 | 1.6048 | 1.6101 | 1.6167 |
| 1.6290 | 1.6351 | 1.6412 | 1.6431 | 1.6544 | 1.6731 | 1.6865 | 1.6985 |
| 1.7101 | 1.7263 | 1.7354 | 1.7410 | 1.7448 | 1.7524 | 1.7578 | 1.7753 |
| 1.7851 | 1.7916 | 1.8082 | 1.8216 | 1.8482 | 1.8585 | 1.8654 | 1.8771 |
| 1.8942 | 1.9049 | 1.9219 | 1.9479 | 1.9536 | 1.9644 | 1.9828 | 1.9988 |
| 2.0048 | 2.0265 | 2.0340 | 2.0421 | 2.0635 | 2.0658 | 2.0730 | 2.0827 |
| 2.1005 | 2.1129 | 2.1338 | 2.1479 | 2.1569 | 2.1988 | 2.2175 | 2.2348 |
| 2.2439 | 2.2501 | 2.2533 | 2.2620 | 2.2670 | 2.2715 | 2.2857 | 2.2889 |
| 2.2904 | 2.3142 | 2.3473 | 2.3618 | 2.3654 | 2.3786 | 2.3927 | 2.4070 |
| 2.4148 | 2.4162 | 2.4274 | 2.4419 | 2.4460 | 2.4647 | 2.4765 | 2.4862 |
| 2.4971 | 2.5058 | 2.5159 | 2.5261 | 2.5426 | 2.5499 | 2.5539 | 2.5618 |
| 2.5681 | 2.5771 | 2.5884 | 2.5909 | 2.5943 | 2.5982 | 2.6120 | 2.6269 |
| 2.6414 | 2.6538 | 2.6675 | 2.6718 | 2.6787 | 2.6856 | 2.7019 | 2.7103 |
| 2.7164 | 2.7178 | 2.7322 | 2.7456 | 2.7572 | 2.7644 | 2.7801 | 2.7881 |
| 2.7946 | 2.8016 | 2.8080 | 2.8332 | 2.8487 | 2.8680 | 2.8927 | 2.8976 |
| 2.9148 | 2.9303 | 2.9466 | 2.9663 | 3.0594 | 3.0999 | 3.1272 | 3.1365 |
| 3.1756 | 3.2494 | 3.2550 | 3.2811 | 3.2880 | 3.3324 | 3.3694 | 3.4067 |
| 3.4352 | 3.4752 | 3.5266 | 3.5478 | 3.5525 | 3.6004 | 3.6115 | 3.6466 |
| 3.6666 | 3.6827 | 3.7203 | 3.7266 | 3.7378 | 3.7446 | 3.7651 | 3.7695 |
| 3.7803 | 3.8289 | 3.8694 | 3.9091 | 3.9314 | 3.9773 | 4.0511 | 4.0814 |
| 4.1125 | 4.1849 | 4.2957 | 4.3096 | 4.6069 | 4.6663 | 4.6959 | 4.7252 |
| 4.9273 | 5.0378 | 5.2004 | 23.5235 | 23.5802 | 23.7200 | 23.7306 | 23.7450 |
| 23.7725 | 23.8058 | 23.8266 | 23.8410 | 23.8884 | 23.9260 | 23.9674 | 23.9783 |
| 24.0363 | 35.4173 | 35.4587 | 35.5234 | 35.6200 | | | |

Ground-State Mulliken Net Atomic Charges

| Atom | Charge (a.u.) |
|---|---|
| 1 N | 0.359521 |
| 2 C | 0.116938 |
| 3 C | 0.009041 |
| 4 C | -0.155591 |
| 5 C | -0.154701 |
| 6 C | -0.130836 |
| 7 C | -0.760155 |
| 8 C | -0.312990 |
| 9 N | -0.085360 |
| 10 C | -0.391866 |
| 11 N | 0.017194 |
| 12 C | 0.289763 |
| 13 C | -0.171258 |
| 14 C | 0.166252 |
| 15 C | -0.376678 |
| 16 C | -0.094659 |
| 17 C | -0.603785 |
| 18 N | -0.492115 |
| 19 H | 0.221604 |
| 20 H | 0.235728 |
| 21 H | 0.227990 |
| 22 H | 0.231862 |
| 23 H | 0.232301 |
| 24 H | 0.235982 |
| 25 H | 0.206451 |
| 26 H | 0.253063 |
| 27 H | 0.257895 |
| 28 H | 0.392348 |

```
    29 H                     0.216151
    30 H                     0.240026
    31 H                     0.202371
    32 H                     0.197598
    33 H                     0.172963
    34 H                     0.176549
    35 H                     0.197816
    36 H                     0.170535
    37 H                     0.320389
    38 H                     0.381665
  ----------------------------------------
  Sum of atomic charges =     2.000000

 -----------------------------------------------------------------
                   Cartesian Multipole Moments
 -----------------------------------------------------------------
    Charge (ESU x 10^10)
                 9.6064
    Dipole Moment (Debye)
         X       6.9341      Y      -0.5390      Z      -5.4826
       Tot       8.8561
    Quadrupole Moments (Debye-Ang)
        XX     -27.9248     XY      27.5500     YY     -67.3982
        XZ     -42.8532     YZ     -18.0916     ZZ     -49.5323
    Traceless Quadrupole Moments (Debye-Ang)
       QXX      61.0809    QYY     -57.3393    QZZ      -3.7416
       QXY      82.6500    QXZ    -128.5596    QYZ     -54.2749
    Octopole Moments (Debye-Ang^2)
       XXX      27.5179    XXY      37.0891    XYY      -2.3106
       YYY     -16.9067    XXZ       8.5535    XYZ     -23.5338
       YYZ      25.1928    XZZ      39.6190    YZZ      23.4483
       ZZZ     -82.9181
    Traceless Octopole Moments (Debye-Ang^2)
       XXX    -170.6682    YYY    -646.2765    ZZZ    -801.2245
       XXY     425.4442    XXZ     275.8175    XYY    -229.1379
       XYZ    -353.0069    XZZ     399.8061    YYZ     525.4070
       YZZ     220.8323
    Hexadecapole Moments (Debye-Ang^3)
      XXXX   -3401.6833   XXXY    -196.0943   XXYY    -707.2346
      XYYY    -524.5395   YYYY    -726.5300   XXXZ     870.0195
      XXYZ    -102.7359   XYYZ     375.5889   YYYZ     352.1766
      XXZZ    -756.1966   XYZZ       2.1773   YYZZ    -570.0028
      XZZZ     850.0639   YZZZ     208.8434   ZZZZ   -1924.1467
    Traceless Hexadecapole Moments (Debye-Ang^3)
      XXXX  -10389.4953   XXXY   11740.6422   XXXZ   -2953.2098
      XXYY   -1584.0889   XXYZ  -17648.8840   XXZZ   11973.5842
      XYYY  -22746.1053   XYYZ    8001.7522   XYZZ   11005.4631
      XZZZ   -5048.5425   YYYY   12980.3630   YYYZ   16393.7128
      YYZZ  -11396.2741   YZZZ    1255.1712   ZZZZ    -577.3101
 -----------------------------------------------------------------

-------------------------------------------------
-  Entering drvman on Sun Oct 13 20:18:18 2024  -
-------------------------------------------------

 Calculating analytic gradient of the SCF energy
 Gradient of SCF Energy
           1           2           3           4           5           6
    1   0.0000033  -0.0001726   0.0000948   0.0000033  -0.0002862   0.0002020
    2   0.0002284   0.0002711  -0.0000541  -0.0000624  -0.0003463   0.0000135
    3  -0.0001334  -0.0005217   0.0003395   0.0003499   0.0001805  -0.0003995
           7           8           9          10          11          12
    1   0.0001619  -0.0002484  -0.0000119  -0.0005013  -0.0000956   0.0002378
    2  -0.0001016  -0.0003349  -0.0003912   0.0001394  -0.0001716   0.0014315
    3  -0.0000536   0.0002511   0.0003703  -0.0001601  -0.0003394   0.0000699
          13          14          15          16          17          18
    1   0.0006254  -0.0001882   0.0000157  -0.0000406   0.0000054   0.0000549
    2   0.0000648  -0.0002729  -0.0001171   0.0000043   0.0000050  -0.0006862
    3  -0.0000972  -0.0000960  -0.0000017   0.0000980  -0.0000032  -0.0000539
          19          20          21          22          23          24
```

```
    1  -0.0000411  -0.0000262   0.0000208   0.0000932  -0.0000807   0.0000251
    2   0.0000011   0.0000532  -0.0000589   0.0000236   0.0001440   0.0000320
    3   0.0000729  -0.0000185   0.0000431   0.0000689  -0.0000041  -0.0000198
           25          26          27          28          29          30
    1  -0.0000253  -0.0000252  -0.0000732   0.0001010   0.0000397   0.0000522
    2   0.0002088  -0.0001594  -0.0000643   0.0000235   0.0000602   0.0000386
    3   0.0000974   0.0000007  -0.0000564  -0.0000417  -0.0000430   0.0000149
           31          32          33          34          35          36
    1   0.0000222   0.0000176  -0.0000042   0.0000029  -0.0000196  -0.0000054
    2   0.0000160  -0.0000007   0.0000121   0.0000178   0.0000243   0.0000238
    3   0.0000005  -0.0000541  -0.0000254  -0.0000029  -0.0000041   0.0000072
           37          38
    1  -0.0002614   0.0003277
    2  -0.0000752   0.0000597
    3  -0.0001587   0.0003238
 Max gradient component =       1.431E-03
 RMS gradient           =       2.253E-04
 Gradient time:  CPU 155.69 s  wall 256.23 s

--------------------------------------------------
-  Entering optman on Sun Oct 13 20:22:34 2024  -
--------------------------------------------------

 Geometry Optimization Parameters
   NAtoms,    NIC,     NZ,  NCons,   NDum,   NFix, NCnnct, MaxDiis
       38     272       0       0       0       0       0       0

 Cartesian Hessian Update
 Hessian updated using BFGS update


** GEOMETRY OPTIMIZATION IN DELOCALIZED INTERNAL COORDINATES **
   Searching for a Minimum

   Optimization Cycle:   9

                    Coordinates (Angstroms)
     ATOM              X                Y                Z
      1  N         2.2232927873     0.2868115872    -1.5140624904
      2  C         3.2687224393     1.1381340859    -1.7280209403
      3  C         3.4997504506     1.5767227569    -3.0249249069
      4  C         2.7100759441     1.1468798887    -4.0780475689
      5  C         1.6691533463     0.2554480932    -3.8292408778
      6  C         1.4543382460    -0.1552381469    -2.5404445859
      7  C         4.1471556776     1.5688964087    -0.5938751170
      8  C         1.9642415222    -0.2619335230    -0.1471090541
      9  N        -1.2532096832    -1.8650027193     0.8805366806
     10  C        -2.1095453273    -0.8073479283     0.9665093924
     11  N        -1.7012767489     0.3993178182     0.6728506016
     12  C        -0.4281570639     0.6135804428     0.2989003173
     13  C         0.5109482983    -0.4690168379     0.1741600280
     14  C         0.0324443007    -1.7042211624     0.4910268294
     15  C        -3.4865635622    -1.0454836207     1.4703523474
     16  C        -3.5407025259    -0.8546599975     3.0059461268
     17  C        -4.9560410520    -1.0547690704     3.5368174897
     18  N        -0.1020092787     1.8754337177     0.0496237016
     19  H         4.3222786808     2.2586935368    -3.1974359758
     20  H         2.9064350456     1.4965227170    -5.0850071137
     21  H         1.0350653181    -0.1190757034    -4.6217536267
     22  H         0.6645515609    -0.8461907861    -2.2805295182
     23  H         4.9459017815     2.2006190842    -0.9779115237
     24  H         4.6098234666     0.7144577823    -0.0933339930
     25  H         3.6044260621     2.1549474385     0.1547166367
     26  H         2.3966010407     0.4295568061     0.5743529009
     27  H         2.5133978422    -1.2017086411    -0.0603439653
     28  H        -1.5906585930    -2.7884680268     1.1256934369
     29  H         0.6428066516    -2.5994384263     0.4601563847
     30  H        -4.1492485034    -0.3258497790     0.9872384615
     31  H        -3.8217238894    -2.0527286324     1.2047896607
     32  H        -3.1857737770     0.1517781491     3.2449795674
```

```
    33  H         -2.8564933743   -1.5624057338    3.4870669654
    34  H         -5.6490956046   -0.3363199609    3.0930135107
    35  H         -4.9755883944   -0.9134301664    4.6182233039
    36  H         -5.3243793942   -2.0612393949    3.3240372181
    37  H          0.8142018406    2.1684429566   -0.2418768410
    38  H         -0.8051455306    2.5882849877    0.1929265370
   Point Group: c1    Number of degrees of freedom:   108


   Energy is   -763.969635792

 Hessian updated using BFGS update
  internal optimization (0)

108 Hessian modes will be used to form the next step
  Hessian Eigenvalues:
     0.001766    0.002665    0.002858    0.003771    0.005075    0.011648
     0.014805    0.019091    0.019239    0.020480    0.021046    0.022129
     0.022601    0.023520    0.024110    0.024370    0.025338    0.025914
     0.026973    0.028212    0.029065    0.029174    0.034112    0.036163
     0.038281    0.040206    0.042687    0.043545    0.043830    0.046499
     0.047167    0.050624    0.053961    0.055812    0.067836    0.077548
     0.083916    0.095162    0.122101    0.122619    0.126858    0.128373
     0.131998    0.132802    0.136875    0.140910    0.143749    0.145019
     0.146737    0.147534    0.148678    0.151654    0.152421    0.153007
     0.158062    0.160366    0.191400    0.192624    0.206520    0.214078
     0.218120    0.230256    0.238843    0.249033    0.250308    0.256675
     0.271712    0.273948    0.283871    0.286909    0.296591    0.299289
     0.300160    0.300617    0.301256    0.302450    0.303356    0.304131
     0.304884    0.304915    0.305597    0.306535    0.309506    0.312285
     0.319768    0.330837    0.332982    0.333572    0.335847    0.339373
     0.341148    0.353550    0.363168    0.363918    0.378539    0.386863
     0.394986    0.402649    0.409977    0.416860    0.421152    0.435905
     0.438406    0.452166    0.463641    0.517643    0.648832    0.727405

 Minimum search - taking simple RFO step
 Searching for Lamda that Minimizes Along All modes
 Value Taken    Lamda =  -0.00020141
 Step Taken.  Stepsize is  0.241722

                             Maximum     Tolerance    Cnvgd?
        Gradient            0.002167      0.000800      NO
        Displacement        0.099667      0.001400      NO
        Energy change      -0.000323      0.000228      NO


 New Cartesian Coordinates Obtained by Inverse Iteration

 Displacement from previous Coordinates is:  0.766588
 ----------------------------------------------------------------
             Standard Nuclear Orientation (Angstroms)
    I     Atom           X                Y                Z
 ----------------------------------------------------------------
    1      N       2.2165525719     0.3188696997    -1.5133896023
    2      C       3.3031756273     1.1162542812    -1.7323211420
    3      C       3.5624376732     1.5269594970    -3.0341713786
    4      C       2.7595090828     1.1233806230    -4.0874624968
    5      C       1.6744090125     0.2887597950    -3.8320067807
    6      C       1.4317421634    -0.0965113620    -2.5388251404
    7      C       4.1888082558     1.5240462342    -0.5961517544
    8      C       1.9374399491    -0.1999242508    -0.1383793260
    9      N      -1.2585317919    -1.8560963137     0.8678369849
   10      C      -2.1257930344    -0.8089105166     0.9672755503
   11      N      -1.7359709197     0.4033840769     0.6739769339
   12      C      -0.4699578938     0.6317115384     0.2857911287
   13      C       0.4849769515    -0.4364562231     0.1629237332
   14      C       0.0249259725    -1.6780409463     0.4789902324
   15      C      -3.4951515554    -1.0649231844     1.4832627721
   16      C      -3.5376626362    -0.8739212211     3.0190733256
   17      C      -4.9458066194    -1.0904262465     3.5625308036
```

```
   18       N      -0.1599740815     1.8976494996     0.0328999687
   19       H       4.4180757410     2.1663296536    -3.2095324568
   20       H       2.9786327512     1.4490241387    -5.0975507354
   21       H       1.0257634628    -0.0630626024    -4.6233509008
   22       H       0.6067497155    -0.7443642720    -2.2774597382
   23       H       5.0324546260     2.0919813431    -0.9833766308
   24       H       4.5883923129     0.6585122168    -0.0615175129
   25       H       3.6680494310     2.1643452369     0.1229589615
   26       H       2.3309006576     0.5247122299     0.5733538727
   27       H       2.5111632369    -1.1203728664    -0.0123219438
   28       H      -1.5845577894    -2.7843306089     1.1108444044
   29       H       0.6476600183    -2.5647756785     0.4497100666
   30       H      -4.1722639978    -0.3549440273     1.0057846589
   31       H      -3.8193608628    -2.0768091985     1.2215757452
   32       H      -3.1925287300     0.1366443534     3.2550457588
   33       H      -2.8410413222    -1.5735851733     3.4942499578
   34       H      -5.6507717689    -0.3789987853     3.1262195469
   35       H      -4.9567806905    -0.9509115457     4.6442854808
   36       H      -5.3049652223    -2.1006118573     3.3515715412
   37       H       0.7382326464     2.1964386184    -0.3066686850
   38       H      -0.8789329434     2.5989738441     0.1543247968
 ----------------------------------------------------------------
 Nuclear Repulsion Energy =        1293.32525787 hartrees
 There are       65 alpha and       65 beta electrons

------------------------------------------------
-  Entering fldman on Sun Oct 13 20:22:34 2024  -
------------------------------------------------

 Applying Cartesian multipole field
    Component          Value
    ---------          -----
     (2,0,0)        1.00000E-12
     (0,2,0)        2.00000E-11
     (0,0,2)       -3.00000E-11
 Nucleus-field energy     =    -0.0000000482 hartrees

------------------------------------------------
-  Entering gesman on Sun Oct 13 20:22:34 2024  -
------------------------------------------------

 Requested basis set is 6-311+G(d,p)
 There are 188 shells and 516 basis functions
 A cutoff of  1.0D-12 yielded  12390 shell pairs
 There are     99188 function pairs (    105738 Cartesian)
 Smallest overlap matrix eigenvalue = 1.05E-06
 Linear dependence detected in AO basis
 Tighter screening thresholds may be required for diffuse basis sets
 Use S2THRESH > 12 and THRESH = 14 in case of SCF convergence issues
 Number of orthogonalized atomic orbitals = 510
 Maximum deviation from orthogonality = 1.194E-11
 Guess MOs from SCF MO coefficient file
 Reading MOs from coefficient file
 Reading MOs from coefficient file

------------------------------------------------
-  Entering scfman on Sun Oct 13 20:22:35 2024  -
------------------------------------------------

 Long-range K will be added via erf
 Coulomb attenuation parameter = 0.2 bohr**(-1)
 A restricted hybrid HF-DFT SCF calculation will be
 performed using Pulay DIIS + Geometric Direct Minimization
 Exchange:     0.2220 Hartree-Fock + 1.0000 wB97X-D + LR-HF
 Correlation:  1.0000 wB97X-D
 Using Euler-Maclaurin-Lebedev (75,302) quadrature formula
 Dispersion:   Grimme D
 SCF converges when RMS gradient is below 1.0E-07
 Geometry optimization detected.  Setting ReadMinima to 0
 Setting SaveMinima to 0
```

```
 ---------------------------------------
  Cycle       Energy         DIIS Error
 ---------------------------------------
    1    -763.9582341308      6.36E-04
    2    -763.9691394732      8.80E-05
    3    -763.9696308124      6.06E-05
    4    -763.9697420995      2.44E-05
    5    -763.9697661957      8.43E-06
    6    -763.9697687862      3.17E-06
    7    -763.9697691435      8.21E-07
    8    -763.9697691770      3.25E-07
    9    -763.9697691814      8.00E-08 Convergence criterion met
 ---------------------------------------
 SCF time:  CPU 311.78 s  wall 523.16 s
 SCF   energy in the final basis set = -763.96976918
 Total energy in the final basis set = -763.96976918

--------------------------------------------------
-  Entering anlman on Sun Oct 13 20:31:18 2024  -
--------------------------------------------------



 ---------------------------------------------------------------

                    Orbital Energies (a.u.)
 ---------------------------------------------------------------


 Alpha MOs
 -- Occupied --
-14.8178 -14.7894 -14.7424 -14.7075 -10.6777 -10.6625 -10.6476 -10.6404
-10.6347 -10.6267 -10.6052 -10.5845 -10.5741 -10.5717 -10.5481 -10.5243
-10.4942 -10.4486  -1.4155  -1.4047  -1.3499  -1.2775  -1.2310  -1.2016
 -1.1877  -1.1415  -1.0991  -1.0829  -1.0559  -1.0528  -1.0299  -0.9999
 -0.9905  -0.9564  -0.9397  -0.9143  -0.9065  -0.8912  -0.8829  -0.8673
 -0.8587  -0.8420  -0.8376  -0.8193  -0.8086  -0.7975  -0.7890  -0.7774
 -0.7691  -0.7627  -0.7560  -0.7489  -0.7448  -0.7294  -0.7187  -0.7109
 -0.6803  -0.6660  -0.6569  -0.6444  -0.6309  -0.6218  -0.6182  -0.6141
 -0.6098
 -- Virtual --
 -0.2709  -0.2581  -0.2441  -0.2263  -0.1761  -0.1631  -0.1472  -0.1429
 -0.1371  -0.1213  -0.1141  -0.1125  -0.1078  -0.1013  -0.0991  -0.0968
 -0.0894  -0.0880  -0.0813  -0.0760  -0.0748  -0.0742  -0.0694  -0.0666
 -0.0623  -0.0576  -0.0532  -0.0520  -0.0454  -0.0440  -0.0432  -0.0326
 -0.0297  -0.0279  -0.0238  -0.0184  -0.0165  -0.0156  -0.0094  -0.0070
 -0.0024  -0.0004  -0.0002   0.0059   0.0098   0.0111   0.0120   0.0156
  0.0179   0.0199   0.0264   0.0290   0.0304   0.0318   0.0357   0.0410
  0.0467   0.0494   0.0523   0.0541   0.0569   0.0626   0.0657   0.0690
  0.0709   0.0724   0.0764   0.0789   0.0810   0.0871   0.0922   0.0951
  0.0986   0.0999   0.1032   0.1039   0.1069   0.1113   0.1154   0.1174
  0.1224   0.1234   0.1282   0.1309   0.1378   0.1390   0.1423   0.1509
  0.1545   0.1592   0.1609   0.1640   0.1660   0.1680   0.1729   0.1771
  0.1873   0.1937   0.1950   0.1965   0.2050   0.2145   0.2190   0.2226
  0.2304   0.2392   0.2533   0.2748   0.2854   0.2924   0.2942   0.3030
  0.3168   0.3329   0.3379   0.3416   0.3547   0.3560   0.3603   0.3613
  0.3687   0.3790   0.3872   0.3935   0.3976   0.4104   0.4194   0.4274
  0.4341   0.4386   0.4447   0.4559   0.4591   0.4679   0.4714   0.4774
  0.4864   0.4915   0.4987   0.5034   0.5093   0.5117   0.5154   0.5192
  0.5216   0.5296   0.5344   0.5399   0.5474   0.5559   0.5565   0.5609
  0.5667   0.5693   0.5734   0.5818   0.5854   0.5948   0.5972   0.6011
  0.6079   0.6203   0.6276   0.6332   0.6470   0.6527   0.6581   0.6616
  0.6750   0.6775   0.6833   0.6875   0.6929   0.7013   0.7050   0.7123
  0.7192   0.7290   0.7435   0.7494   0.7578   0.7676   0.7787   0.7911
  0.7975   0.8024   0.8093   0.8142   0.8286   0.8467   0.8517   0.8578
  0.8637   0.8751   0.8761   0.8861   0.8969   0.9074   0.9161   0.9379
  0.9431   0.9631   0.9688   0.9806   0.9906   0.9969   1.0232   1.0311
  1.0531   1.0820   1.1098   1.1183   1.1417   1.1431   1.1532   1.1781
  1.1930   1.1984   1.2191   1.2369   1.2657   1.2767   1.2983   1.3052
  1.3148   1.3176   1.3272   1.3342   1.3411   1.3492   1.3664   1.3742
  1.3873   1.3931   1.3934   1.3964   1.4018   1.4069   1.4178   1.4331
  1.4395   1.4512   1.4572   1.4635   1.4676   1.4857   1.4910   1.4973
```

| | | | | | | | |
|---|---|---|---|---|---|---|---|
| 1.5020 | 1.5086 | 1.5104 | 1.5194 | 1.5304 | 1.5431 | 1.5467 | 1.5558 |
| 1.5632 | 1.5660 | 1.5690 | 1.5755 | 1.5977 | 1.6063 | 1.6105 | 1.6181 |
| 1.6301 | 1.6351 | 1.6403 | 1.6454 | 1.6550 | 1.6711 | 1.6869 | 1.6998 |
| 1.7106 | 1.7267 | 1.7355 | 1.7428 | 1.7468 | 1.7535 | 1.7574 | 1.7762 |
| 1.7858 | 1.7930 | 1.8081 | 1.8209 | 1.8485 | 1.8604 | 1.8662 | 1.8772 |
| 1.8923 | 1.9082 | 1.9216 | 1.9460 | 1.9574 | 1.9662 | 1.9823 | 2.0008 |
| 2.0056 | 2.0224 | 2.0372 | 2.0445 | 2.0560 | 2.0671 | 2.0721 | 2.0788 |
| 2.1031 | 2.1113 | 2.1311 | 2.1487 | 2.1578 | 2.1987 | 2.2208 | 2.2339 |
| 2.2465 | 2.2495 | 2.2533 | 2.2610 | 2.2629 | 2.2721 | 2.2869 | 2.2882 |
| 2.2908 | 2.3149 | 2.3505 | 2.3602 | 2.3666 | 2.3790 | 2.3927 | 2.4098 |
| 2.4145 | 2.4192 | 2.4272 | 2.4418 | 2.4458 | 2.4636 | 2.4766 | 2.4865 |
| 2.4977 | 2.5035 | 2.5162 | 2.5281 | 2.5432 | 2.5502 | 2.5538 | 2.5596 |
| 2.5710 | 2.5772 | 2.5875 | 2.5885 | 2.5935 | 2.5981 | 2.6148 | 2.6298 |
| 2.6380 | 2.6509 | 2.6657 | 2.6706 | 2.6819 | 2.6839 | 2.7003 | 2.7082 |
| 2.7164 | 2.7195 | 2.7307 | 2.7464 | 2.7577 | 2.7652 | 2.7702 | 2.7878 |
| 2.7928 | 2.8060 | 2.8111 | 2.8332 | 2.8489 | 2.8690 | 2.8920 | 2.8952 |
| 2.9145 | 2.9281 | 2.9504 | 2.9752 | 3.0602 | 3.0990 | 3.1258 | 3.1366 |
| 3.1752 | 3.2480 | 3.2545 | 3.2811 | 3.2904 | 3.3280 | 3.3687 | 3.4061 |
| 3.4348 | 3.4771 | 3.5259 | 3.5485 | 3.5514 | 3.6007 | 3.6084 | 3.6454 |
| 3.6674 | 3.6829 | 3.7203 | 3.7259 | 3.7376 | 3.7449 | 3.7645 | 3.7698 |
| 3.7823 | 3.8285 | 3.8691 | 3.9098 | 3.9314 | 3.9776 | 4.0513 | 4.0816 |
| 4.1128 | 4.1848 | 4.2952 | 4.3116 | 4.6051 | 4.6660 | 4.6968 | 4.7245 |
| 4.9262 | 5.0376 | 5.2003 | 23.5229 | 23.5810 | 23.7199 | 23.7313 | 23.7460 |
| 23.7737 | 23.8059 | 23.8279 | 23.8409 | 23.8871 | 23.9260 | 23.9652 | 23.9797 |
| 24.0347 | 35.4175 | 35.4591 | 35.5235 | 35.6205 | | | |

Ground-State Mulliken Net Atomic Charges

| Atom | | Charge (a.u.) |
|---|---|---|
| 1 | N | 0.340939 |
| 2 | C | 0.123097 |
| 3 | C | 0.008364 |
| 4 | C | -0.158177 |
| 5 | C | -0.149490 |
| 6 | C | -0.129550 |
| 7 | C | -0.755132 |
| 8 | C | -0.316902 |
| 9 | N | -0.085573 |
| 10 | C | -0.387554 |
| 11 | N | 0.017853 |
| 12 | C | 0.295381 |
| 13 | C | -0.185370 |
| 14 | C | 0.171908 |
| 15 | C | -0.375323 |
| 16 | C | -0.097439 |
| 17 | C | -0.602950 |
| 18 | N | -0.494473 |
| 19 | H | 0.221648 |
| 20 | H | 0.235502 |
| 21 | H | 0.227879 |
| 22 | H | 0.234683 |
| 23 | H | 0.232133 |
| 24 | H | 0.230396 |
| 25 | H | 0.213709 |
| 26 | H | 0.253220 |
| 27 | H | 0.258832 |
| 28 | H | 0.392058 |
| 29 | H | 0.216042 |
| 30 | H | 0.240264 |
| 31 | H | 0.201995 |
| 32 | H | 0.197965 |
| 33 | H | 0.172984 |
| 34 | H | 0.176657 |
| 35 | H | 0.197797 |
| 36 | H | 0.170450 |
| 37 | H | 0.327791 |
| 38 | H | 0.378383 |

```
 Sum of atomic charges =     2.000000

 -----------------------------------------------------------------
                    Cartesian Multipole Moments
 -----------------------------------------------------------------
    Charge (ESU x 10^10)
                 9.6064
    Dipole Moment (Debye)
         X       6.8829      Y      -0.4573      Z      -5.5732
       Tot       8.8681
    Quadrupole Moments (Debye-Ang)
        XX     -26.9491     XY      27.3862     YY     -68.1303
        XZ     -43.2894     YZ     -18.4535     ZZ     -49.4016
    Traceless Quadrupole Moments (Debye-Ang)
       QXX      63.6337    QYY     -59.9099    QZZ      -3.7238
       QXY      82.1587    QXZ    -129.8681    QYZ     -55.3605
    Octopole Moments (Debye-Ang^2)
       XXX      35.4468    XXY      36.6523    XYY      -5.4671
       YYY     -18.4752    XXZ       4.9520    XYZ     -22.9643
       YYZ      26.5553    XZZ      41.7103    YZZ      22.5815
       ZZZ     -82.4326
    Traceless Octopole Moments (Debye-Ang^2)
       XXX    -113.5075    YYY    -643.9555    ZZZ    -778.1611
       XXY     427.5085    XXZ     227.0556    XYY    -297.0767
       XYZ    -344.4645    XZZ     410.5843    YYZ     551.1056
       YZZ     216.4470
    Hexadecapole Moments (Debye-Ang^3)
      XXXX   -3402.7797   XXXY    -193.0317   XXYY    -718.7004
      XYYY    -538.5395   YYYY    -720.3169   XXXZ     858.0205
      XXYZ    -104.8511   XYYZ     385.3810   YYYZ     361.5136
      XXZZ    -747.4552   XYZZ       2.1373   YYZZ    -577.9955
      XZZZ     848.4770   YZZZ     214.1884   ZZZZ   -1932.6253
    Traceless Hexadecapole Moments (Debye-Ang^3)
      XXXX  -10383.9083   XXXY   12556.1982   XXXZ   -4042.3840
      XXYY   -2606.3918   XXYZ  -18069.3057   XXZZ   12990.3002
      XYYY  -23722.1278   XYYZ    9086.8320   XYZZ   11165.9297
      XZZZ   -5044.4481   YYYY   14601.6593   YYYZ   16779.1169
      YYZZ  -11995.2674   YZZZ    1290.1888   ZZZZ    -995.0327
 -----------------------------------------------------------------

--------------------------------------------------
-  Entering drvman on Sun Oct 13 20:31:18 2024  -
--------------------------------------------------

 Calculating analytic gradient of the SCF energy
 Gradient of SCF Energy
            1           2           3           4           5           6
    1  -0.0001580   0.0000771  -0.0001862   0.0001461   0.0003352  -0.0001358
    2   0.0001471  -0.0002218  -0.0000238   0.0001837   0.0001875  -0.0003605
    3   0.0001282   0.0004941  -0.0005548  -0.0002465  -0.0004437   0.0006317
            7           8           9          10          11          12
    1   0.0000004  -0.0000562   0.0000003   0.0003533  -0.0003002  -0.0002576
    2   0.0003707  -0.0002570   0.0000524   0.0004326  -0.0003407  -0.0001596
    3  -0.0000114   0.0002751   0.0000591  -0.0000594   0.0001220  -0.0004268
           13          14          15          16          17          18
    1   0.0000295   0.0000198   0.0000172   0.0000001   0.0000025   0.0005427
    2  -0.0003701   0.0003676   0.0000098   0.0000423   0.0000005  -0.0000944
    3   0.0000013  -0.0001930  -0.0000090   0.0000048  -0.0000207   0.0011298
           19          20          21          22          23          24
    1  -0.0000364  -0.0000371   0.0000008   0.0000165  -0.0000190   0.0000871
    2   0.0000740  -0.0000018  -0.0000456   0.0000318  -0.0000547  -0.0000423
    3  -0.0000174   0.0000704  -0.0000502  -0.0000031  -0.0000325  -0.0000262
           25          26          27          28          29          30
    1  -0.0001226  -0.0003551   0.0000842  -0.0000088   0.0000139  -0.0000296
    2   0.0000954  -0.0001471   0.0000041  -0.0000234   0.0000178   0.0000112
    3   0.0000422   0.0000124  -0.0000671  -0.0000344   0.0000124   0.0000013
           31          32          33          34          35          36
    1   0.0000083   0.0000111  -0.0000013  -0.0000040   0.0000034  -0.0000085
    2   0.0000048  -0.0000006   0.0000025   0.0000192   0.0000181   0.0000164
    3  -0.0000047  -0.0000175  -0.0000058  -0.0000014  -0.0000091  -0.0000008
```

```
           37           38
    1  -0.0000303  -0.0000030
    2  -0.0000164   0.0000703
    3  -0.0006045  -0.0001447
 Max gradient component =       1.130E-03
 RMS gradient           =       2.161E-04
 Gradient time:  CPU 153.52 s  wall 254.58 s

-------------------------------------------------
-  Entering optman on Sun Oct 13 20:35:33 2024  -
-------------------------------------------------

 Geometry Optimization Parameters
   NAtoms,    NIC,     NZ,  NCons,   NDum,   NFix, NCnnct, MaxDiis
       38     272       0       0       0       0       0       0

 Cartesian Hessian Update
 Hessian updated using BFGS update


** GEOMETRY OPTIMIZATION IN DELOCALIZED INTERNAL COORDINATES **
   Searching for a Minimum

   Optimization Cycle:  10

                    Coordinates (Angstroms)
     ATOM                X               Y               Z
      1  N         2.2165525719    0.3188696997   -1.5133896023
      2  C         3.3031756273    1.1162542812   -1.7323211420
      3  C         3.5624376732    1.5269594970   -3.0341713786
      4  C         2.7595090828    1.1233806230   -4.0874624968
      5  C         1.6744090125    0.2887597950   -3.8320067807
      6  C         1.4317421634   -0.0965113620   -2.5388251404
      7  C         4.1888082558    1.5240462342   -0.5961517544
      8  C         1.9374399491   -0.1999242508   -0.1383793260
      9  N        -1.2585317919   -1.8560963137    0.8678369849
     10  C        -2.1257930344   -0.8089105166    0.9672755503
     11  N        -1.7359709197    0.4033840769    0.6739769339
     12  C        -0.4699578938    0.6317115384    0.2857911287
     13  C         0.4849769515   -0.4364562231    0.1629237332
     14  C         0.0249259725   -1.6780409463    0.4789902324
     15  C        -3.4951515554   -1.0649231844    1.4832627721
     16  C        -3.5376626362   -0.8739212211    3.0190733256
     17  C        -4.9458066194   -1.0904262465    3.5625308036
     18  N        -0.1599740815    1.8976494996    0.0328999687
     19  H         4.4180757410    2.1663296536   -3.2095324568
     20  H         2.9786327512    1.4490241387   -5.0975507354
     21  H         1.0257634628   -0.0630626024   -4.6233509008
     22  H         0.6067497155   -0.7443642720   -2.2774597382
     23  H         5.0324546260    2.0919813431   -0.9833766308
     24  H         4.5883923129    0.6585122168   -0.0615175129
     25  H         3.6680494310    2.1643452369    0.1229589615
     26  H         2.3309006576    0.5247122299    0.5733538727
     27  H         2.5111632369   -1.1203728664   -0.0123219438
     28  H        -1.5845577894   -2.7843306089    1.1108444044
     29  H         0.6476600183   -2.5647756785    0.4497100666
     30  H        -4.1722639978   -0.3549440273    1.0057846589
     31  H        -3.8193608628   -2.0768091985    1.2215757452
     32  H        -3.1925287300    0.1366443534    3.2550457588
     33  H        -2.8410413222   -1.5735851733    3.4942499578
     34  H        -5.6507717689   -0.3789987853    3.1262195469
     35  H        -4.9567806905   -0.9509115457    4.6442854808
     36  H        -5.3049652223   -2.1006118573    3.3515715412
     37  H         0.7382326464    2.1964386184   -0.3066686850
     38  H        -0.8789329434    2.5989738441    0.1543247968
   Point Group: c1    Number of degrees of freedom:   108


   Energy is   -763.969769181
```

```
 Hessian updated using BFGS update
  internal optimization (0)

108 Hessian modes will be used to form the next step
  Hessian Eigenvalues:
     0.001536    0.002346    0.002760    0.003764    0.004923    0.011734
     0.015075    0.019047    0.019295    0.020177    0.020644    0.022132
     0.022612    0.023504    0.024105    0.024388    0.025237    0.026115
     0.027003    0.028213    0.028629    0.029386    0.034217    0.036173
     0.038183    0.039663    0.042477    0.043495    0.043834    0.046498
     0.046913    0.050916    0.054165    0.055810    0.067273    0.077527
     0.083924    0.095317    0.122100    0.122641    0.126858    0.128383
     0.132152    0.132802    0.136845    0.140979    0.143614    0.145125
     0.146683    0.147534    0.148639    0.151647    0.152471    0.153291
     0.159498    0.164393    0.191414    0.192914    0.206518    0.214389
     0.217025    0.230024    0.238886    0.248995    0.250891    0.257755
     0.271710    0.273864    0.282990    0.288030    0.296584    0.299291
     0.300162    0.300621    0.301260    0.302441    0.303479    0.304133
     0.304884    0.304920    0.305667    0.306495    0.309056    0.312550
     0.320604    0.330626    0.332891    0.333933    0.335973    0.339385
     0.341262    0.353908    0.363353    0.370765    0.380875    0.388416
     0.395790    0.403015    0.414558    0.417465    0.423211    0.435697
     0.445897    0.453631    0.463663    0.517267    0.628111    0.723615

 Minimum search - taking simple RFO step
 Searching for Lamda that Minimizes Along All modes
 Value Taken    Lamda =  -0.00012406
 Step Taken.  Stepsize is  0.184486

                          Maximum     Tolerance    Cnvgd?
        Gradient          0.001873     0.000800      NO
        Displacement      0.073145     0.001400      NO
        Energy change    -0.000133     0.000228     YES


 New Cartesian Coordinates Obtained by Inverse Iteration

 Displacement from previous Coordinates is:  0.682108
 ----------------------------------------------------------------
            Standard Nuclear Orientation (Angstroms)
    I     Atom           X                 Y                 Z
 ----------------------------------------------------------------
    1      N       2.2101318216     0.3485877449    -1.5129105716
    2      C       3.3313028453     1.0956877513    -1.7356933880
    3      C       3.6138982239     1.4815580142    -3.0389784865
    4      C       2.7954402374     1.1050548582    -4.0908111859
    5      C       1.6710115311     0.3255526807    -3.8313904489
    6      C       1.4088743326    -0.0381130740    -2.5367478880
    7      C       4.2259531701     1.4767677316    -0.5979426513
    8      C       1.9160928810    -0.1447604834    -0.1314002355
    9      N      -1.2577541404    -1.8476166002     0.8654976105
   10      C      -2.1385790313    -0.8121526375     0.9655077478
   11      N      -1.7665637108     0.4052549667     0.6655906432
   12      C      -0.5058634638     0.6484985539     0.2701103481
   13      C       0.4652764816    -0.4050118130     0.1571505664
   14      C       0.0238077473    -1.6526742873     0.4789197568
   15      C      -3.5011158242    -1.0843413588     1.4914531125
   16      C      -3.5348505341    -0.8904293858     3.0270392944
   17      C      -4.9365462788    -1.1210888819     3.5811661643
   18      N      -0.2147546022     1.9169854295     0.0022209642
   19      H       4.4988897442     2.0785543302    -3.2177151416
   20      H       3.0327612010     1.4097768001    -5.1035335369
   21      H       1.0082623352    -0.0013828984    -4.6215842220
   22      H       0.5548327324    -0.6463268080    -2.2734466617
   23      H       5.1039719306     1.9899471517    -0.9852595146
   24      H       4.5705941865     0.6031464223    -0.0391486401
   25      H       3.7301442073     2.1599839559     0.0990465533
   26      H       2.2827918515     0.6040539319     0.5703859471
   27      H       2.5066096225    -1.0499078366     0.0244038799
   28      H      -1.5709587770    -2.7792693853     1.1126425818
```

```
  29      H       0.6596959425    -2.5302730427     0.4560755874
  30      H      -4.1907810267    -0.3845169104     1.0170094395
  31      H      -3.8141155605    -2.1010456806     1.2346581258
  32      H      -3.1992683943     0.1243459438     3.2588513454
  33      H      -2.8273048427    -1.5814532313     3.4987848544
  34      H      -5.6521367320    -0.4177353992     3.1490929656
  35      H      -4.9409245565    -0.9802544914     4.6628209820
  36      H      -5.2864487986    -2.1353436418     3.3741324920
  37      H       0.6754791829     2.2232251117    -0.3528202073
  38      H      -0.9478559344     2.6067164690     0.1068218174
 ----------------------------------------------------------------
 Nuclear Repulsion Energy =        1292.46260096 hartrees
 There are       65 alpha and       65 beta electrons

--------------------------------------------------
-  Entering fldman on Sun Oct 13 20:35:33 2024  -
--------------------------------------------------

 Applying Cartesian multipole field
    Component          Value
    ---------          -----
     (2,0,0)        1.00000E-12
     (0,2,0)        2.00000E-11
     (0,0,2)       -3.00000E-11
 Nucleus-field energy     =    -0.0000000486 hartrees

--------------------------------------------------
-  Entering gesman on Sun Oct 13 20:35:33 2024  -
--------------------------------------------------

 Requested basis set is 6-311+G(d,p)
 There are 188 shells and 516 basis functions
 A cutoff of  1.0D-12 yielded  12375 shell pairs
 There are     99108 function pairs (    105663 Cartesian)
 Smallest overlap matrix eigenvalue = 1.00E-06
 Linear dependence detected in AO basis
 Tighter screening thresholds may be required for diffuse basis sets
 Use S2THRESH > 12 and THRESH = 14 in case of SCF convergence issues
 Number of orthogonalized atomic orbitals = 510
 Maximum deviation from orthogonality = 1.585E-11
 Guess MOs from SCF MO coefficient file
 Reading MOs from coefficient file
 Reading MOs from coefficient file

--------------------------------------------------
-  Entering scfman on Sun Oct 13 20:35:33 2024  -
--------------------------------------------------

 Long-range K will be added via erf
 Coulomb attenuation parameter = 0.2 bohr**(-1)
 A restricted hybrid HF-DFT SCF calculation will be
 performed using Pulay DIIS + Geometric Direct Minimization
 Exchange:     0.2220 Hartree-Fock + 1.0000 wB97X-D + LR-HF
 Correlation:  1.0000 wB97X-D
 Using Euler-Maclaurin-Lebedev (75,302) quadrature formula
 Dispersion:   Grimme D
 SCF converges when RMS gradient is below 1.0E-07
 Geometry optimization detected.  Setting ReadMinima to 0
 Setting SaveMinima to 0
 ---------------------------------------
  Cycle       Energy         DIIS Error
 ---------------------------------------
    1    -763.9574478028      5.84E-04
    2    -763.9693539131      7.70E-05
    3    -763.9697542746      5.18E-05
    4    -763.9698349828      2.34E-05
    5    -763.9698564891      7.36E-06
    6    -763.9698585256      2.66E-06
    7    -763.9698587820      6.80E-07
    8    -763.9698588063      2.72E-07
```

```
    9    -763.9698588093      6.24E-08 Convergence criterion met
 ---------------------------------------
 SCF time:  CPU 297.55 s  wall 485.41 s
 SCF   energy in the final basis set = -763.96985881
 Total energy in the final basis set = -763.96985881

-------------------------------------------------
-  Entering anlman on Sun Oct 13 20:43:38 2024  -
-------------------------------------------------



 --------------------------------------------------------------

                   Orbital Energies (a.u.)
 --------------------------------------------------------------

 Alpha MOs
 -- Occupied --
-14.8175 -14.7898 -14.7422 -14.7077 -10.6776 -10.6628 -10.6480 -10.6396
-10.6349 -10.6265 -10.6053 -10.5842 -10.5737 -10.5712 -10.5472 -10.5245
-10.4944 -10.4487  -1.4156  -1.4045  -1.3497  -1.2774  -1.2308  -1.2016
 -1.1875  -1.1416  -1.0991  -1.0825  -1.0561  -1.0528  -1.0293  -1.0000
 -0.9903  -0.9563  -0.9398  -0.9140  -0.9067  -0.8913  -0.8825  -0.8670
 -0.8592  -0.8417  -0.8376  -0.8190  -0.8085  -0.7978  -0.7886  -0.7766
 -0.7690  -0.7628  -0.7560  -0.7484  -0.7447  -0.7291  -0.7183  -0.7109
 -0.6804  -0.6657  -0.6566  -0.6445  -0.6311  -0.6215  -0.6183  -0.6143
 -0.6100
 -- Virtual --
 -0.2703  -0.2582  -0.2444  -0.2260  -0.1765  -0.1632  -0.1478  -0.1429
 -0.1371  -0.1211  -0.1138  -0.1118  -0.1079  -0.1013  -0.0990  -0.0965
 -0.0893  -0.0877  -0.0809  -0.0764  -0.0744  -0.0737  -0.0689  -0.0662
 -0.0625  -0.0576  -0.0529  -0.0520  -0.0457  -0.0438  -0.0428  -0.0331
 -0.0309  -0.0272  -0.0238  -0.0189  -0.0166  -0.0155  -0.0092  -0.0066
 -0.0025  -0.0000   0.0008   0.0059   0.0098   0.0109   0.0119   0.0158
  0.0182   0.0201   0.0266   0.0293   0.0307   0.0317   0.0348   0.0423
  0.0473   0.0501   0.0527   0.0544   0.0564   0.0627   0.0658   0.0699
  0.0710   0.0722   0.0756   0.0790   0.0819   0.0876   0.0933   0.0945
  0.0988   0.0992   0.1040   0.1041   0.1069   0.1111   0.1154   0.1171
  0.1222   0.1241   0.1289   0.1316   0.1373   0.1388   0.1426   0.1506
  0.1556   0.1585   0.1610   0.1638   0.1660   0.1684   0.1727   0.1775
  0.1882   0.1932   0.1946   0.1961   0.2072   0.2134   0.2209   0.2232
  0.2317   0.2410   0.2510   0.2743   0.2858   0.2925   0.2937   0.3025
  0.3174   0.3322   0.3375   0.3413   0.3554   0.3563   0.3600   0.3622
  0.3690   0.3788   0.3875   0.3928   0.3973   0.4097   0.4209   0.4272
  0.4330   0.4387   0.4441   0.4564   0.4606   0.4666   0.4720   0.4765
  0.4865   0.4911   0.4979   0.5037   0.5097   0.5124   0.5157   0.5202
  0.5216   0.5304   0.5335   0.5400   0.5475   0.5544   0.5560   0.5619
  0.5664   0.5701   0.5754   0.5818   0.5857   0.5939   0.5970   0.5991
  0.6076   0.6198   0.6262   0.6328   0.6458   0.6525   0.6585   0.6613
  0.6754   0.6785   0.6838   0.6867   0.6929   0.7032   0.7059   0.7119
  0.7207   0.7287   0.7409   0.7500   0.7577   0.7659   0.7761   0.7937
  0.7999   0.8025   0.8089   0.8131   0.8285   0.8423   0.8515   0.8558
  0.8629   0.8747   0.8768   0.8886   0.8963   0.9084   0.9139   0.9384
  0.9430   0.9639   0.9678   0.9791   0.9926   0.9963   1.0227   1.0325
  1.0552   1.0840   1.1092   1.1216   1.1400   1.1432   1.1550   1.1792
  1.1938   1.1987   1.2185   1.2392   1.2649   1.2787   1.2997   1.3092
  1.3158   1.3162   1.3290   1.3380   1.3435   1.3495   1.3664   1.3740
  1.3877   1.3929   1.3931   1.3950   1.4014   1.4058   1.4187   1.4333
  1.4376   1.4537   1.4573   1.4626   1.4663   1.4847   1.4900   1.4975
  1.5024   1.5088   1.5108   1.5168   1.5282   1.5434   1.5468   1.5561
  1.5626   1.5661   1.5699   1.5754   1.5978   1.6069   1.6114   1.6189
  1.6307   1.6350   1.6392   1.6467   1.6557   1.6709   1.6867   1.7004
  1.7127   1.7275   1.7356   1.7436   1.7487   1.7545   1.7572   1.7760
  1.7859   1.7943   1.8080   1.8204   1.8489   1.8626   1.8664   1.8775
  1.8908   1.9115   1.9214   1.9449   1.9579   1.9664   1.9831   2.0019
  2.0073   2.0195   2.0380   2.0453   2.0490   2.0675   2.0733   2.0779
  2.1046   2.1107   2.1286   2.1488   2.1585   2.1989   2.2220   2.2332
  2.2471   2.2488   2.2536   2.2599   2.2612   2.2743   2.2877   2.2897
  2.2917   2.3160   2.3520   2.3597   2.3698   2.3790   2.3925   2.4115
  2.4138   2.4207   2.4280   2.4413   2.4452   2.4629   2.4768   2.4863
```

```
  2.4985   2.5017   2.5163   2.5296   2.5433   2.5502   2.5532   2.5578
  2.5717   2.5768   2.5846   2.5883   2.5937   2.5988   2.6160   2.6308
  2.6366   2.6491   2.6633   2.6698   2.6820   2.6837   2.6997   2.7064
  2.7162   2.7202   2.7294   2.7475   2.7584   2.7638   2.7662   2.7873
  2.7932   2.8077   2.8173   2.8342   2.8491   2.8691   2.8903   2.8941
  2.9114   2.9276   2.9523   2.9817   3.0618   3.0945   3.1246   3.1374
  3.1749   3.2481   3.2538   3.2798   3.2933   3.3263   3.3673   3.4076
  3.4336   3.4778   3.5248   3.5495   3.5512   3.6018   3.6052   3.6446
  3.6678   3.6827   3.7198   3.7269   3.7374   3.7451   3.7640   3.7692
  3.7845   3.8281   3.8694   3.9093   3.9310   3.9776   4.0511   4.0819
  4.1131   4.1847   4.2955   4.3117   4.6034   4.6653   4.6976   4.7235
  4.9250   5.0386   5.1998  23.5224  23.5818  23.7196  23.7314  23.7462
 23.7744  23.8056  23.8298  23.8405  23.8882  23.9259  23.9675  23.9771
 24.0360  35.4184  35.4593  35.5225  35.6194
 --------------------------------------------------------------

          Ground-State Mulliken Net Atomic Charges

     Atom                 Charge (a.u.)
  ----------------------------------------
      1 N                     0.325749
      2 C                     0.137163
      3 C                     0.005629
      4 C                    -0.162422
      5 C                    -0.144914
      6 C                    -0.129389
      7 C                    -0.751899
      8 C                    -0.321058
      9 N                    -0.086132
     10 C                    -0.385905
     11 N                     0.019738
     12 C                     0.298652
     13 C                    -0.197701
     14 C                     0.176602
     15 C                    -0.375272
     16 C                    -0.098545
     17 C                    -0.602749
     18 N                    -0.494582
     19 H                     0.221612
     20 H                     0.235378
     21 H                     0.227679
     22 H                     0.237031
     23 H                     0.232085
     24 H                     0.225931
     25 H                     0.219255
     26 H                     0.254691
     27 H                     0.258551
     28 H                     0.392317
     29 H                     0.215727
     30 H                     0.240410
     31 H                     0.202289
     32 H                     0.198133
     33 H                     0.172899
     34 H                     0.176796
     35 H                     0.197801
     36 H                     0.170448
     37 H                     0.331047
     38 H                     0.376952
  ----------------------------------------
  Sum of atomic charges =     2.000000

 -----------------------------------------------------------------
                    Cartesian Multipole Moments
 -----------------------------------------------------------------
    Charge (ESU x 10^10)
                 9.6064
    Dipole Moment (Debye)
         X       6.8439      Y      -0.3883      Z      -5.5934
       Tot       8.8473
    Quadrupole Moments (Debye-Ang)
```

```
        XX     -26.0956     XY      27.1485     YY     -68.7782
        XZ     -43.5584     YZ     -18.8292     ZZ     -49.3102
    Traceless Quadrupole Moments (Debye-Ang)
       QXX      65.8973    QYY     -62.1507    QZZ      -3.7465
       QXY      81.4454    QXZ    -130.6753    QYZ     -56.4875
    Octopole Moments (Debye-Ang^2)
       XXX      42.5492    XXY      35.8299    XYY      -8.2275
       YYY     -19.9606    XXZ       2.1571    XYZ     -22.0932
       YYZ      28.0908    XZZ      43.0068    YZZ      21.7243
       ZZZ     -81.3815
    Traceless Octopole Moments (Debye-Ang^2)
       XXX     -57.7190    YYY    -637.7515    ZZZ    -760.5192
       XXY     424.6679    XXZ     185.7570    XYY    -355.3977
       XYZ    -331.3987    XZZ     413.1166    YYZ     574.7622
       YZZ     213.0836
    Hexadecapole Moments (Debye-Ang^3)
      XXXX   -3398.5521   XXXY    -191.3478   XXYY    -729.2936
      XYYY    -549.7620   YYYY    -714.7032   XXXZ     844.7584
      XXYZ    -104.6939   XYYZ     393.3512   YYYZ     370.5483
      XXZZ    -740.3878   XYZZ       1.6420   YYZZ    -583.6065
      XZZZ     847.1503   YZZZ     220.3080   ZZZZ   -1938.9816
    Traceless Hexadecapole Moments (Debye-Ang^3)
      XXXX  -10136.2651   XXXY   13184.5283   XXXZ   -5137.0649
      XXYY   -3614.7142   XXYZ  -18280.6756   XXZZ   13750.9792
      XYYY  -24448.9547   XYYZ   10022.9785   XYZZ   11264.4264
      XZZZ   -4885.9137   YYYY   16011.1437   YYYZ   17044.1215
      YYZZ  -12396.4296   YZZZ    1236.5540   ZZZZ   -1354.5497
 -----------------------------------------------------------------

--------------------------------------------------
-  Entering drvman on Sun Oct 13 20:43:38 2024  -
--------------------------------------------------

 Calculating analytic gradient of the SCF energy
 Gradient of SCF Energy
           1           2           3           4           5           6
    1  -0.0001501   0.0000169   0.0001224  -0.0000778  -0.0001549   0.0002381
    2   0.0001295  -0.0000304   0.0000360  -0.0000788  -0.0001760   0.0000807
    3   0.0000950  -0.0003263   0.0004086   0.0001088   0.0002505  -0.0003717
           7           8           9          10          11          12
    1   0.0000993   0.0001399  -0.0000726   0.0002270   0.0001907  -0.0003247
    2   0.0002702  -0.0000326   0.0001862  -0.0006867   0.0006438  -0.0013724
    3  -0.0000153   0.0003275  -0.0001056   0.0000493   0.0000925  -0.0003340
          13          14          15          16          17          18
    1  -0.0003894  -0.0000901  -0.0000194  -0.0000404   0.0000289   0.0009610
    2   0.0003963  -0.0002093   0.0000390   0.0000076   0.0000213   0.0007962
    3  -0.0001988   0.0000370   0.0001589  -0.0001185   0.0000033   0.0013336
          19          20          21          22          23          24
    1  -0.0000238   0.0000118   0.0000215  -0.0000337   0.0000004   0.0000464
    2   0.0000318   0.0000081  -0.0000223   0.0000703  -0.0000253  -0.0000370
    3  -0.0000194  -0.0000496  -0.0000029  -0.0001193   0.0000085  -0.0000550
          25          26          27          28          29          30
    1  -0.0002285  -0.0004684   0.0001561  -0.0000814  -0.0000025  -0.0000464
    2   0.0000833  -0.0001638   0.0000372  -0.0000478  -0.0000591  -0.0000126
    3  -0.0000648   0.0001621  -0.0001397   0.0000008   0.0000524  -0.0000141
          31          32          33          34          35          36
    1   0.0000001   0.0000017  -0.0000023  -0.0000023   0.0000063   0.0000024
    2  -0.0000061   0.0000099  -0.0000016   0.0000221   0.0000123   0.0000047
    3   0.0000105   0.0000192   0.0000143  -0.0000084   0.0000107  -0.0000060
          37          38
    1   0.0003087  -0.0003709
    2  -0.0000146   0.0000900
    3  -0.0008263  -0.0003676
 Max gradient component =       1.372E-03
 RMS gradient           =       2.857E-04
 Gradient time:  CPU 155.59 s  wall 255.33 s

--------------------------------------------------
-  Entering optman on Sun Oct 13 20:47:54 2024  -
--------------------------------------------------
```

```
 Geometry Optimization Parameters
   NAtoms,    NIC,     NZ,  NCons,   NDum,   NFix, NCnnct, MaxDiis
       38     272       0       0       0       0       0       0

 Cartesian Hessian Update
 Hessian updated using BFGS update


** GEOMETRY OPTIMIZATION IN DELOCALIZED INTERNAL COORDINATES **
   Searching for a Minimum

   Optimization Cycle:  11

                    Coordinates (Angstroms)
     ATOM              X              Y              Z
      1  N         2.2101318216    0.3485877449   -1.5129105716
      2  C         3.3313028453    1.0956877513   -1.7356933880
      3  C         3.6138982239    1.4815580142   -3.0389784865
      4  C         2.7954402374    1.1050548582   -4.0908111859
      5  C         1.6710115311    0.3255526807   -3.8313904489
      6  C         1.4088743326   -0.0381130740   -2.5367478880
      7  C         4.2259531701    1.4767677316   -0.5979426513
      8  C         1.9160928810   -0.1447604834   -0.1314002355
      9  N        -1.2577541404   -1.8476166002    0.8654976105
     10  C        -2.1385790313   -0.8121526375    0.9655077478
     11  N        -1.7665637108    0.4052549667    0.6655906432
     12  C        -0.5058634638    0.6484985539    0.2701103481
     13  C         0.4652764816   -0.4050118130    0.1571505664
     14  C         0.0238077473   -1.6526742873    0.4789197568
     15  C        -3.5011158242   -1.0843413588    1.4914531125
     16  C        -3.5348505341   -0.8904293858    3.0270392944
     17  C        -4.9365462788   -1.1210888819    3.5811661643
     18  N        -0.2147546022    1.9169854295    0.0022209642
     19  H         4.4988897442    2.0785543302   -3.2177151416
     20  H         3.0327612010    1.4097768001   -5.1035335369
     21  H         1.0082623352   -0.0013828984   -4.6215842220
     22  H         0.5548327324   -0.6463268080   -2.2734466617
     23  H         5.1039719306    1.9899471517   -0.9852595146
     24  H         4.5705941865    0.6031464223   -0.0391486401
     25  H         3.7301442073    2.1599839559    0.0990465533
     26  H         2.2827918515    0.6040539319    0.5703859471
     27  H         2.5066096225   -1.0499078366    0.0244038799
     28  H        -1.5709587770   -2.7792693853    1.1126425818
     29  H         0.6596959425   -2.5302730427    0.4560755874
     30  H        -4.1907810267   -0.3845169104    1.0170094395
     31  H        -3.8141155605   -2.1010456806    1.2346581258
     32  H        -3.1992683943    0.1243459438    3.2588513454
     33  H        -2.8273048427   -1.5814532313    3.4987848544
     34  H        -5.6521367320   -0.4177353992    3.1490929656
     35  H        -4.9409245565   -0.9802544914    4.6628209820
     36  H        -5.2864487986   -2.1353436418    3.3741324920
     37  H         0.6754791829    2.2232251117   -0.3528202073
     38  H        -0.9478559344    2.6067164690    0.1068218174
   Point Group: c1    Number of degrees of freedom:   108


   Energy is   -763.969858809

 Hessian updated using BFGS update
  internal optimization (0)

108 Hessian modes will be used to form the next step
  Hessian Eigenvalues:
     0.001204    0.002435    0.002749    0.003755    0.004793    0.012158
     0.013333    0.018585    0.019163    0.019478    0.020625    0.022141
     0.022571    0.023582    0.024140    0.024390    0.024864    0.026114
     0.026911    0.027106    0.028248    0.029316    0.032659    0.036093
     0.038263    0.040300    0.042510    0.043489    0.043831    0.046091
     0.046987    0.051069    0.054162    0.055808    0.064144    0.077534
```

| | | | | | |
|---|---|---|---|---|---|
| 0.083897 | 0.095565 | 0.122100 | 0.122680 | 0.126858 | 0.128334 |
| 0.132206 | 0.132802 | 0.136915 | 0.141133 | 0.143571 | 0.145378 |
| 0.146509 | 0.147533 | 0.148682 | 0.151621 | 0.152358 | 0.152489 |
| 0.159999 | 0.167771 | 0.191411 | 0.192734 | 0.206499 | 0.214203 |
| 0.215186 | 0.229785 | 0.238809 | 0.248378 | 0.250157 | 0.252789 |
| 0.271688 | 0.273600 | 0.277889 | 0.287335 | 0.296618 | 0.299295 |
| 0.300162 | 0.300621 | 0.301261 | 0.302441 | 0.303429 | 0.304134 |
| 0.304884 | 0.304920 | 0.305664 | 0.306480 | 0.308178 | 0.312491 |
| 0.320255 | 0.330471 | 0.332853 | 0.334141 | 0.336377 | 0.339384 |
| 0.341236 | 0.358185 | 0.366785 | 0.372013 | 0.380497 | 0.390237 |
| 0.399845 | 0.404534 | 0.413602 | 0.418336 | 0.422769 | 0.437761 |
| 0.444845 | 0.452519 | 0.499040 | 0.522140 | 0.589944 | 0.732001 |

Minimum search - taking simple RFO step
Searching for Lamda that Minimizes Along All modes
Value Taken    Lamda =  -0.00016830
Step Taken.  Stepsize is  0.226709

| | Maximum | Tolerance | Cnvgd? |
|---|---|---|---|
| Gradient | 0.001868 | 0.000800 | NO |
| Displacement | 0.083134 | 0.001400 | NO |
| Energy change | -0.000090 | 0.000228 | YES |

New Cartesian Coordinates Obtained by Inverse Iteration

Displacement from previous Coordinates is:  0.852945

Standard Nuclear Orientation (Angstroms)

| I | Atom | X | Y | Z |
|---|---|---|---|---|
| 1 | N | 2.2032886160 | 0.3823395390 | -1.5105107179 |
| 2 | C | 3.3629135995 | 1.0659546391 | -1.7401230729 |
| 3 | C | 3.6658460529 | 1.4253837269 | -3.0464248989 |
| 4 | C | 2.8262521160 | 1.0873252349 | -4.0946682442 |
| 5 | C | 1.6600293159 | 0.3748630984 | -3.8275366440 |
| 6 | C | 1.3802149183 | 0.0330490274 | -2.5301049219 |
| 7 | C | 4.2737652687 | 1.4076416890 | -0.6034571335 |
| 8 | C | 1.8944318654 | -0.0751422506 | -0.1201927796 |
| 9 | N | -1.2493417310 | -1.8316521249 | 0.8737979721 |
| 10 | C | -2.1509069124 | -0.8133037490 | 0.9624411693 |
| 11 | N | -1.8039490365 | 0.4076258350 | 0.6473799416 |
| 12 | C | -0.5480403902 | 0.6713535374 | 0.2495845788 |
| 13 | C | 0.4466105442 | -0.3615542506 | 0.1577784391 |
| 14 | C | 0.0298298159 | -1.6146084693 | 0.4919919582 |
| 15 | C | -3.5060006291 | -1.1069320419 | 1.4959243684 |
| 16 | C | -3.5331937208 | -0.9106735775 | 3.0314928939 |
| 17 | C | -4.9273848122 | -1.1623026351 | 3.5953097684 |
| 18 | N | -0.2852102606 | 1.9408090689 | -0.0447998971 |
| 19 | H | 4.5833625119 | 1.9695386509 | -3.2301518511 |
| 20 | H | 3.0793980995 | 1.3694586090 | -5.1100926466 |
| 21 | H | 0.9786048824 | 0.0808450472 | -4.6147965288 |
| 22 | H | 0.4958952604 | -0.5284844007 | -2.2629604659 |
| 23 | H | 5.1922391449 | 1.8421213208 | -0.9934046745 |
| 24 | H | 4.5452077931 | 0.5278717566 | -0.0151139923 |
| 25 | H | 3.8224980233 | 2.1460298759 | 0.0671485424 |
| 26 | H | 2.2349153747 | 0.7037218789 | 0.5631059755 |
| 27 | H | 2.5019831158 | -0.9615384944 | 0.0730783502 |
| 28 | H | -1.5442585715 | -2.7665101503 | 1.1313959633 |
| 29 | H | 0.6833882451 | -2.4793897198 | 0.4821885783 |
| 30 | H | -4.2100993327 | -0.4199954283 | 1.0240567078 |
| 31 | H | -3.8031617437 | -2.1294585650 | 1.2432226430 |
| 32 | H | -3.2123542199 | 0.1097368812 | 3.2596167812 |
| 33 | H | -2.8118809146 | -1.5894996374 | 3.5001781693 |
| 34 | H | -5.6567241960 | -0.4711569326 | 3.1666390772 |
| 35 | H | -4.9266478512 | -1.0193695801 | 4.6767175615 |
| 36 | H | -5.2625213090 | -2.1823979584 | 3.3925808646 |
| 37 | H | 0.6056748209 | 2.2629635335 | -0.3842932070 |
| 38 | H | -1.0346737533 | 2.6153370157 | 0.0430013723 |

```
 Nuclear Repulsion Energy =         1291.65872452 hartrees
 There are       65 alpha and       65 beta electrons

--------------------------------------------------
-  Entering fldman on Sun Oct 13 20:47:54 2024  -
--------------------------------------------------

 Applying Cartesian multipole field
    Component          Value
    ---------          -----
     (2,0,0)        1.00000E-12
     (0,2,0)        2.00000E-11
     (0,0,2)       -3.00000E-11
 Nucleus-field energy     =    -0.0000000489 hartrees

--------------------------------------------------
-  Entering gesman on Sun Oct 13 20:47:54 2024  -
--------------------------------------------------

 Requested basis set is 6-311+G(d,p)
 There are 188 shells and 516 basis functions
 A cutoff of  1.0D-12 yielded  12365 shell pairs
 There are     98980 function pairs (    105505 Cartesian)
 Smallest overlap matrix eigenvalue = 9.12E-07
 Linear dependence detected in AO basis
 Tighter screening thresholds may be required for diffuse basis sets
 Use S2THRESH > 12 and THRESH = 14 in case of SCF convergence issues
 Number of orthogonalized atomic orbitals = 510
 Maximum deviation from orthogonality = 3.283E-11
 Guess MOs from SCF MO coefficient file
 Reading MOs from coefficient file
 Reading MOs from coefficient file

--------------------------------------------------
-  Entering scfman on Sun Oct 13 20:47:54 2024  -
--------------------------------------------------

 Long-range K will be added via erf
 Coulomb attenuation parameter = 0.2 bohr**(-1)
 A restricted hybrid HF-DFT SCF calculation will be
 performed using Pulay DIIS + Geometric Direct Minimization
 Exchange:     0.2220 Hartree-Fock + 1.0000 wB97X-D + LR-HF
 Correlation:  1.0000 wB97X-D
 Using Euler-Maclaurin-Lebedev (75,302) quadrature formula
 Dispersion:   Grimme D
 SCF converges when RMS gradient is below 1.0E-07
 Geometry optimization detected.  Setting ReadMinima to 0
 Setting SaveMinima to 0
 ---------------------------------------
  Cycle       Energy         DIIS Error
 ---------------------------------------
    1    -763.9464658448      7.37E-04
    2    -763.9691721056      9.86E-05
    3    -763.9697949309      6.92E-05
    4    -763.9699371490      3.06E-05
    5    -763.9699736870      9.38E-06
    6    -763.9699771822      2.90E-06
    7    -763.9699775035      8.74E-07
    8    -763.9699775423      3.28E-07
    9    -763.9699775468      7.74E-08 Convergence criterion met
 ---------------------------------------
 SCF time:  CPU 298.09 s  wall 489.64 s
 SCF   energy in the final basis set = -763.96997755
 Total energy in the final basis set = -763.96997755

--------------------------------------------------
-  Entering anlman on Sun Oct 13 20:56:04 2024  -
--------------------------------------------------
```

```
 ---------------------------------------------------------------

                   Orbital Energies (a.u.)
 ---------------------------------------------------------------

 Alpha MOs
 -- Occupied --
-14.8172 -14.7899 -14.7422 -14.7080 -10.6777 -10.6630 -10.6483 -10.6388
-10.6351 -10.6263 -10.6054 -10.5836 -10.5732 -10.5707 -10.5464 -10.5246
-10.4947 -10.4489  -1.4158  -1.4043  -1.3496  -1.2775  -1.2306  -1.2014
 -1.1870  -1.1417  -1.0991  -1.0819  -1.0565  -1.0530  -1.0284  -1.0001
 -0.9899  -0.9564  -0.9398  -0.9138  -0.9068  -0.8914  -0.8820  -0.8668
 -0.8597  -0.8413  -0.8375  -0.8188  -0.8083  -0.7984  -0.7880  -0.7755
 -0.7688  -0.7630  -0.7561  -0.7479  -0.7446  -0.7286  -0.7180  -0.7111
 -0.6805  -0.6657  -0.6562  -0.6447  -0.6313  -0.6211  -0.6183  -0.6145
 -0.6101
 -- Virtual --
 -0.2694  -0.2583  -0.2446  -0.2258  -0.1770  -0.1632  -0.1485  -0.1429
 -0.1369  -0.1209  -0.1136  -0.1108  -0.1079  -0.1016  -0.0987  -0.0961
 -0.0891  -0.0873  -0.0805  -0.0773  -0.0737  -0.0732  -0.0684  -0.0659
 -0.0626  -0.0575  -0.0527  -0.0520  -0.0462  -0.0435  -0.0422  -0.0338
 -0.0315  -0.0262  -0.0238  -0.0198  -0.0168  -0.0154  -0.0093  -0.0059
 -0.0025   0.0003   0.0020   0.0054   0.0096   0.0108   0.0120   0.0158
  0.0180   0.0209   0.0261   0.0303   0.0311   0.0316   0.0340   0.0440
  0.0477   0.0507   0.0529   0.0549   0.0560   0.0627   0.0660   0.0702
  0.0716   0.0726   0.0748   0.0790   0.0829   0.0886   0.0920   0.0951
  0.0984   0.0992   0.1042   0.1053   0.1072   0.1114   0.1153   0.1168
  0.1216   0.1258   0.1297   0.1320   0.1361   0.1390   0.1425   0.1499
  0.1566   0.1583   0.1613   0.1632   0.1662   0.1685   0.1722   0.1779
  0.1887   0.1912   0.1953   0.1969   0.2103   0.2121   0.2222   0.2255
  0.2323   0.2433   0.2489   0.2735   0.2868   0.2925   0.2940   0.3032
  0.3174   0.3314   0.3377   0.3410   0.3561   0.3572   0.3601   0.3640
  0.3692   0.3785   0.3882   0.3924   0.3973   0.4090   0.4210   0.4264
  0.4321   0.4374   0.4446   0.4563   0.4619   0.4645   0.4729   0.4771
  0.4861   0.4898   0.4976   0.5036   0.5096   0.5140   0.5156   0.5197
  0.5230   0.5324   0.5328   0.5398   0.5470   0.5545   0.5559   0.5623
  0.5656   0.5704   0.5780   0.5818   0.5845   0.5922   0.5957   0.5995
  0.6070   0.6193   0.6252   0.6329   0.6450   0.6520   0.6589   0.6612
  0.6762   0.6802   0.6839   0.6867   0.6933   0.7050   0.7069   0.7115
  0.7224   0.7280   0.7383   0.7500   0.7582   0.7637   0.7746   0.7955
  0.7993   0.8057   0.8096   0.8113   0.8285   0.8364   0.8506   0.8551
  0.8627   0.8745   0.8764   0.8917   0.8939   0.9097   0.9125   0.9385
  0.9439   0.9677   0.9686   0.9797   0.9912   0.9989   1.0222   1.0334
  1.0564   1.0843   1.1073   1.1241   1.1376   1.1441   1.1559   1.1794
  1.1919   1.2004   1.2175   1.2405   1.2633   1.2820   1.2978   1.3116
  1.3154   1.3213   1.3309   1.3413   1.3460   1.3524   1.3662   1.3741
  1.3874   1.3919   1.3942   1.3944   1.4009   1.4058   1.4197   1.4331
  1.4358   1.4556   1.4568   1.4619   1.4654   1.4842   1.4887   1.4985
  1.5031   1.5091   1.5118   1.5144   1.5263   1.5426   1.5468   1.5540
  1.5636   1.5671   1.5713   1.5743   1.5985   1.6061   1.6126   1.6193
  1.6309   1.6347   1.6374   1.6475   1.6556   1.6723   1.6854   1.7002
  1.7179   1.7286   1.7356   1.7436   1.7511   1.7544   1.7591   1.7756
  1.7855   1.7965   1.8075   1.8201   1.8483   1.8659   1.8663   1.8778
  1.8896   1.9151   1.9219   1.9444   1.9569   1.9667   1.9838   2.0022
  2.0090   2.0182   2.0359   2.0416   2.0477   2.0676   2.0755   2.0786
  2.1054   2.1110   2.1260   2.1486   2.1596   2.1999   2.2226   2.2323
  2.2460   2.2477   2.2534   2.2583   2.2625   2.2760   2.2886   2.2910
  2.2933   2.3172   2.3532   2.3596   2.3752   2.3787   2.3925   2.4123
  2.4140   2.4206   2.4290   2.4411   2.4444   2.4608   2.4772   2.4859
  2.4989   2.5007   2.5166   2.5312   2.5415   2.5501   2.5522   2.5556
  2.5694   2.5756   2.5832   2.5881   2.5941   2.5991   2.6165   2.6280
  2.6385   2.6484   2.6592   2.6693   2.6802   2.6842   2.7006   2.7045
  2.7161   2.7207   2.7285   2.7474   2.7580   2.7632   2.7674   2.7868
  2.7953   2.8111   2.8235   2.8366   2.8496   2.8691   2.8870   2.8926
  2.9069   2.9279   2.9531   2.9868   3.0635   3.0875   3.1236   3.1380
  3.1754   3.2481   3.2534   3.2791   3.2965   3.3245   3.3660   3.4088
  3.4309   3.4783   3.5246   3.5483   3.5541   3.6004   3.6037   3.6439
  3.6680   3.6822   3.7193   3.7278   3.7373   3.7456   3.7633   3.7681
  3.7870   3.8278   3.8693   3.9093   3.9316   3.9775   4.0506   4.0819
  4.1136   4.1845   4.2959   4.3118   4.6022   4.6650   4.6989   4.7230
```

```
 4.9246   5.0397   5.1995  23.5219  23.5834  23.7195  23.7326  23.7469
23.7749  23.8052  23.8321  23.8404  23.8881  23.9256  23.9673  23.9764
24.0357  35.4197  35.4601  35.5223  35.6188
--------------------------------------------------------------

         Ground-State Mulliken Net Atomic Charges

    Atom                 Charge (a.u.)
 ----------------------------------------
     1 N                     0.313805
     2 C                     0.159041
     3 C                     0.000845
     4 C                    -0.168147
     5 C                    -0.143636
     6 C                    -0.129542
     7 C                    -0.749335
     8 C                    -0.326575
     9 N                    -0.088720
    10 C                    -0.387031
    11 N                     0.022805
    12 C                     0.301509
    13 C                    -0.209134
    14 C                     0.183238
    15 C                    -0.376193
    16 C                    -0.098425
    17 C                    -0.603112
    18 N                    -0.494402
    19 H                     0.221541
    20 H                     0.235154
    21 H                     0.227542
    22 H                     0.239526
    23 H                     0.232234
    24 H                     0.220141
    25 H                     0.226154
    26 H                     0.256332
    27 H                     0.257097
    28 H                     0.392960
    29 H                     0.214913
    30 H                     0.240382
    31 H                     0.203356
    32 H                     0.198126
    33 H                     0.172676
    34 H                     0.177004
    35 H                     0.197776
    36 H                     0.170500
    37 H                     0.332502
    38 H                     0.377094
 ----------------------------------------
 Sum of atomic charges =     2.000000

-----------------------------------------------------------------
                   Cartesian Multipole Moments
-----------------------------------------------------------------
   Charge (ESU x 10^10)
                 9.6064
   Dipole Moment (Debye)
        X       6.8185      Y      -0.2668      Z      -5.5413
      Tot       8.7903
   Quadrupole Moments (Debye-Ang)
       XX     -25.1636     XY      26.7098     YY     -69.5541
       XZ     -43.7051     YZ     -19.2845     ZZ     -49.2500
   Traceless Quadrupole Moments (Debye-Ang)
      QXX      68.4770    QYY     -64.6946    QZZ      -3.7824
      QXY      80.1295    QXZ    -131.1154    QYZ     -57.8536
   Octopole Moments (Debye-Ang^2)
      XXX      50.9220    XXY      34.2229    XYY     -11.4431
      YYY     -21.3202    XXZ      -0.7137    XYZ     -20.7262
      YYZ      30.2037    XZZ      44.1474    YZZ      20.7351
      ZZZ     -79.9696
   Traceless Octopole Moments (Debye-Ang^2)
```

```
       XXX      11.1936    YYY    -622.5431    ZZZ    -745.2274
       XXY     412.4295    XXZ     140.7327    XYY    -422.5252
       XYZ    -310.8936    XZZ     411.3316    YYZ     604.4947
       YZZ     210.1137
    Hexadecapole Moments (Debye-Ang^3)
      XXXX   -3390.5377   XXXY    -191.2631   XXYY    -743.2620
      XYYY    -561.3916   YYYY    -707.0307   XXXZ     827.8621
      XXYZ    -102.1913   XYYZ     402.2134   YYYZ     382.3995
      XXZZ    -734.0951   XYZZ       1.2008   YYZZ    -588.1090
      XZZZ     845.5426   YZZZ     227.5261   ZZZZ   -1944.7997
    Traceless Hexadecapole Moments (Debye-Ang^3)
      XXXX   -9455.6307   XXXY   13732.8027   XXXZ   -6477.2951
      XXYY   -4967.9649   XXYZ  -18338.2095   XXZZ   14423.5956
      XYYY  -25130.6950   XYYZ   11098.1359   XYZZ   11397.8923
      XZZZ   -4620.8408   YYYY   17658.2322   YYYZ   17327.5793
      YYZZ  -12690.2673   YZZZ    1010.6301   ZZZZ   -1733.3283
 -----------------------------------------------------------------

-------------------------------------------------
-  Entering drvman on Sun Oct 13 20:56:04 2024  -
-------------------------------------------------

 Calculating analytic gradient of the SCF energy
 Gradient of SCF Energy
           1           2           3           4           5           6
    1  -0.0003985  -0.0000416   0.0000557   0.0000150  -0.0000838   0.0001869
    2   0.0001958  -0.0000298   0.0001262  -0.0000749   0.0000510  -0.0000346
    3   0.0002190  -0.0000677   0.0002535  -0.0001517   0.0002605  -0.0001863
           7           8           9          10          11          12
    1  -0.0000405   0.0004047  -0.0001751   0.0003967   0.0002215  -0.0003632
    2   0.0000547  -0.0000190   0.0005109  -0.0008897   0.0007059  -0.0024977
    3   0.0000599   0.0003289  -0.0002984   0.0000668   0.0002847  -0.0001266
          13          14          15          16          17          18
    1  -0.0007463   0.0000285  -0.0000037  -0.0000296   0.0000249   0.0010697
    2   0.0006152  -0.0002056   0.0000951   0.0000018   0.0000296   0.0015841
    3  -0.0003018   0.0001009   0.0001827  -0.0001430   0.0000164   0.0008987
          19          20          21          22          23          24
    1   0.0000040   0.0000779   0.0000247  -0.0000717   0.0000319  -0.0000588
    2  -0.0000053  -0.0000388   0.0000349   0.0000141  -0.0000575  -0.0000332
    3  -0.0000651  -0.0000557  -0.0000356  -0.0001477   0.0000321   0.0000531
          25          26          27          28          29          30
    1  -0.0002095  -0.0003074   0.0002309  -0.0001279  -0.0000357  -0.0000693
    2   0.0000700  -0.0000351   0.0000903  -0.0000712  -0.0001173  -0.0000328
    3  -0.0001766   0.0001532  -0.0002050   0.0000463   0.0000565  -0.0000156
          31          32          33          34          35          36
    1   0.0000007  -0.0000105  -0.0000020   0.0000063   0.0000190   0.0000135
    2  -0.0000136   0.0000154  -0.0000014   0.0000154   0.0000063  -0.0000035
    3   0.0000107   0.0000399   0.0000257  -0.0000096   0.0000236   0.0000045
          37          38
    1   0.0005540  -0.0005912
    2  -0.0000859   0.0000303
    3  -0.0006395  -0.0004915
 Max gradient component =       2.498E-03
 RMS gradient           =       3.802E-04
 Gradient time:  CPU 143.05 s  wall 233.47 s

-------------------------------------------------
-  Entering optman on Sun Oct 13 20:59:57 2024  -
-------------------------------------------------

 Geometry Optimization Parameters
   NAtoms,    NIC,     NZ,  NCons,   NDum,   NFix, NCnnct, MaxDiis
       38     272       0       0       0       0       0       0

 Cartesian Hessian Update
 Hessian updated using BFGS update


** GEOMETRY OPTIMIZATION IN DELOCALIZED INTERNAL COORDINATES **
   Searching for a Minimum
```

```
   Optimization Cycle:  12

                    Coordinates (Angstroms)
     ATOM                X                Y                Z
      1  N         2.2032886160    0.3823395390   -1.5105107179
      2  C         3.3629135995    1.0659546391   -1.7401230729
      3  C         3.6658460529    1.4253837269   -3.0464248989
      4  C         2.8262521160    1.0873252349   -4.0946682442
      5  C         1.6600293159    0.3748630984   -3.8275366440
      6  C         1.3802149183    0.0330490274   -2.5301049219
      7  C         4.2737652687    1.4076416890   -0.6034571335
      8  C         1.8944318654   -0.0751422506   -0.1201927796
      9  N        -1.2493417310   -1.8316521249    0.8737979721
     10  C        -2.1509069124   -0.8133037490    0.9624411693
     11  N        -1.8039490365    0.4076258350    0.6473799416
     12  C        -0.5480403902    0.6713535374    0.2495845788
     13  C         0.4466105442   -0.3615542506    0.1577784391
     14  C         0.0298298159   -1.6146084693    0.4919919582
     15  C        -3.5060006291   -1.1069320419    1.4959243684
     16  C        -3.5331937208   -0.9106735775    3.0314928939
     17  C        -4.9273848122   -1.1623026351    3.5953097684
     18  N        -0.2852102606    1.9408090689   -0.0447998971
     19  H         4.5833625119    1.9695386509   -3.2301518511
     20  H         3.0793980995    1.3694586090   -5.1100926466
     21  H         0.9786048824    0.0808450472   -4.6147965288
     22  H         0.4958952604   -0.5284844007   -2.2629604659
     23  H         5.1922391449    1.8421213208   -0.9934046745
     24  H         4.5452077931    0.5278717566   -0.0151139923
     25  H         3.8224980233    2.1460298759    0.0671485424
     26  H         2.2349153747    0.7037218789    0.5631059755
     27  H         2.5019831158   -0.9615384944    0.0730783502
     28  H        -1.5442585715   -2.7665101503    1.1313959633
     29  H         0.6833882451   -2.4793897198    0.4821885783
     30  H        -4.2100993327   -0.4199954283    1.0240567078
     31  H        -3.8031617437   -2.1294585650    1.2432226430
     32  H        -3.2123542199    0.1097368812    3.2596167812
     33  H        -2.8118809146   -1.5894996374    3.5001781693
     34  H        -5.6567241960   -0.4711569326    3.1666390772
     35  H        -4.9266478512   -1.0193695801    4.6767175615
     36  H        -5.2625213090   -2.1823979584    3.3925808646
     37  H         0.6056748209    2.2629635335   -0.3842932070
     38  H        -1.0346737533    2.6153370157    0.0430013723
   Point Group: c1    Number of degrees of freedom:   108


   Energy is   -763.969977547

 Hessian updated using BFGS update
  internal optimization (0)

108 Hessian modes will be used to form the next step
  Hessian Eigenvalues:
     0.001544    0.002255    0.002744    0.003748    0.004607    0.009920
     0.012533    0.018231    0.019126    0.019404    0.020642    0.022147
     0.022534    0.023611    0.024139    0.024379    0.024457    0.026063
     0.026249    0.027067    0.028307    0.029302    0.031891    0.036071
     0.038329    0.041000    0.042879    0.043543    0.043830    0.045686
     0.047057    0.050629    0.054176    0.055803    0.060720    0.077539
     0.083870    0.095141    0.122097    0.122620    0.126859    0.128299
     0.132216    0.132815    0.137965    0.141327    0.143636    0.145582
     0.145990    0.147534    0.148779    0.150905    0.151701    0.152483
     0.158094    0.168034    0.191406    0.192585    0.206470    0.212773
     0.215271    0.229284    0.236111    0.240670    0.249927    0.252776
     0.271671    0.273225    0.276083    0.287444    0.296627    0.299328
     0.300163    0.300618    0.301261    0.302441    0.303426    0.304136
     0.304884    0.304926    0.305687    0.306602    0.307866    0.312509
     0.319918    0.330497    0.332888    0.334140    0.336392    0.339383
     0.341224    0.358037    0.365825    0.372153    0.381747    0.390235
     0.399405    0.404515    0.412206    0.418518    0.421920    0.437843
```

```
    0.440451    0.450612    0.482403    0.520777    0.574534    0.753442

 Minimum search - taking simple RFO step
 Searching for Lamda that Minimizes Along All modes
 Value Taken    Lamda =  -0.00017537
 Step Taken.  Stepsize is  0.165068

                          Maximum     Tolerance    Cnvgd?
        Gradient         0.002549      0.000800      NO
        Displacement     0.049092      0.001400      NO
        Energy change   -0.000119      0.000228     YES


 New Cartesian Coordinates Obtained by Inverse Iteration

 Displacement from previous Coordinates is:  0.661847
 ----------------------------------------------------------------
             Standard Nuclear Orientation (Angstroms)
    I     Atom           X                Y                Z
 ----------------------------------------------------------------
    1      N       2.2003770944     0.4055623983    -1.5067476375
    2      C       3.3848051763     1.0407846899    -1.7477221136
    3      C       3.6943196165     1.3788633426    -3.0577688125
    4      C       2.8323028428     1.0722102575    -4.0975344355
    5      C       1.6393158922     0.4106494270    -3.8188140785
    6      C       1.3556383435     0.0859711869    -2.5179742574
    7      C       4.3166547257     1.3471943613    -0.6183420749
    8      C       1.8868490360    -0.0211873942    -0.1080752143
    9      N      -1.2323567818    -1.8115258748     0.8973744617
   10      C      -2.1535509769    -0.8088053165     0.9638132878
   11      N      -1.8268934228     0.4128796492     0.6314980958
   12      C      -0.5725975924     0.6953199075     0.2399389444
   13      C       0.4417800331    -0.3206593743     0.1712885350
   14      C       0.0448439202    -1.5761061287     0.5214100827
   15      C      -3.5062661667    -1.1207851534     1.4926163980
   16      C      -3.5393193516    -0.9275367052     3.0286173578
   17      C      -4.9308453936    -1.1997430487     3.5895364320
   18      N      -0.3371453720     1.9624125365    -0.0801759039
   19      H       4.6336016511     1.8811258698    -3.2508265362
   20      H       3.0892974613     1.3384826977    -5.1163021845
   21      H       0.9403919139     0.1422789716    -4.5997602250
   22      H       0.4526521812    -0.4404807128    -2.2419624392
   23      H       5.2458353668     1.7526454908    -1.0144256813
   24      H       4.5614399647     0.4574033506    -0.0338121766
   25      H       3.8977525618     2.0989672462     0.0585170970
   26      H       2.2211449252     0.7770843300     0.5566464236
   27      H       2.5005807025    -0.8973867071     0.1104384416
   28      H      -1.5114181065    -2.7476530091     1.1675265460
   29      H       0.7135033105    -2.4292751523     0.5266045669
   30      H      -4.2179902981    -0.4426200663     1.0198316205
   31      H      -3.7889761999    -2.1469159334     1.2376965422
   32      H      -3.2334245448     0.0969830164     3.2590141420
   33      H      -2.8094290399    -1.5967177053     3.4979552107
   34      H      -5.6692616085    -0.5189293707     3.1599863399
   35      H      -4.9341633945    -1.0575666213     4.6710353621
   36      H      -5.2507725944    -2.2244395150     3.3854467503
   37      H       0.5594499560     2.3072519173    -0.3786546625
   38      H      -1.0981258308     2.6242631418     0.0021057954
 ----------------------------------------------------------------
 Nuclear Repulsion Energy =        1291.20777435 hartrees
 There are       65 alpha and       65 beta electrons

--------------------------------------------------
-  Entering fldman on Sun Oct 13 20:59:57 2024  -
--------------------------------------------------

 Applying Cartesian multipole field
    Component          Value
    ---------          -----
     (2,0,0)       1.00000E-12
```

```
     (0,2,0)         2.00000E-11
     (0,0,2)        -3.00000E-11
 Nucleus-field energy     =    -0.0000000491 hartrees

 -------------------------------------------------
-  Entering gesman on Sun Oct 13 20:59:57 2024  -
 -------------------------------------------------

 Requested basis set is 6-311+G(d,p)
 There are 188 shells and 516 basis functions
 A cutoff of  1.0D-12 yielded  12364 shell pairs
 There are     98956 function pairs (    105482 Cartesian)
 Smallest overlap matrix eigenvalue = 8.13E-07
 Linear dependence detected in AO basis
 Tighter screening thresholds may be required for diffuse basis sets
 Use S2THRESH > 12 and THRESH = 14 in case of SCF convergence issues
 Number of orthogonalized atomic orbitals = 510
 Maximum deviation from orthogonality = 5.055E-11
 Guess MOs from SCF MO coefficient file
 Reading MOs from coefficient file
 Reading MOs from coefficient file

 -------------------------------------------------
-  Entering scfman on Sun Oct 13 20:59:58 2024  -
 -------------------------------------------------

 Long-range K will be added via erf
 Coulomb attenuation parameter = 0.2 bohr**(-1)
 A restricted hybrid HF-DFT SCF calculation will be
 performed using Pulay DIIS + Geometric Direct Minimization
 Exchange:     0.2220 Hartree-Fock + 1.0000 wB97X-D + LR-HF
 Correlation:  1.0000 wB97X-D
 Using Euler-Maclaurin-Lebedev (75,302) quadrature formula
 Dispersion:   Grimme D
 SCF converges when RMS gradient is below 1.0E-07
 Geometry optimization detected.  Setting ReadMinima to 0
 Setting SaveMinima to 0
 ---------------------------------------
  Cycle       Energy         DIIS Error
 ---------------------------------------
    1    -763.9564464175      5.95E-04
    2    -763.9696028169      7.57E-05
    3    -763.9699934370      4.75E-05
    4    -763.9700592645      2.55E-05
    5    -763.9700835910      6.60E-06
    6    -763.9700855592      2.06E-06
    7    -763.9700857307      6.96E-07
    8    -763.9700857581      2.35E-07
    9    -763.9700857607      6.95E-08 Convergence criterion met
 ---------------------------------------
 SCF time:  CPU 303.50 s  wall 499.78 s
 SCF   energy in the final basis set = -763.97008576
 Total energy in the final basis set = -763.97008576

 -------------------------------------------------
-  Entering anlman on Sun Oct 13 21:08:17 2024  -
 -------------------------------------------------


 --------------------------------------------------------------

                   Orbital Energies (a.u.)
 --------------------------------------------------------------

 Alpha MOs
 -- Occupied --
-14.8168 -14.7899 -14.7423 -14.7080 -10.6779 -10.6630 -10.6484 -10.6381
-10.6353 -10.6260 -10.6056 -10.5830 -10.5727 -10.5701 -10.5458 -10.5247
-10.4950 -10.4491  -1.4159  -1.4040  -1.3501  -1.2776  -1.2305  -1.2013
 -1.1867  -1.1417  -1.0990  -1.0815  -1.0568  -1.0532  -1.0278  -1.0003
```

```
-0.9895  -0.9565  -0.9399  -0.9142  -0.9068  -0.8916  -0.8816  -0.8666
-0.8597  -0.8410  -0.8375  -0.8185  -0.8082  -0.7988  -0.7877  -0.7746
-0.7685  -0.7631  -0.7563  -0.7476  -0.7447  -0.7282  -0.7177  -0.7111
-0.6806  -0.6659  -0.6558  -0.6447  -0.6314  -0.6209  -0.6182  -0.6147
-0.6102
-- Virtual --
-0.2686  -0.2582  -0.2446  -0.2253  -0.1774  -0.1633  -0.1490  -0.1428
-0.1367  -0.1208  -0.1137  -0.1100  -0.1076  -0.1021  -0.0984  -0.0956
-0.0887  -0.0867  -0.0805  -0.0780  -0.0731  -0.0727  -0.0680  -0.0656
-0.0626  -0.0574  -0.0528  -0.0519  -0.0467  -0.0430  -0.0419  -0.0347
-0.0316  -0.0252  -0.0237  -0.0207  -0.0171  -0.0153  -0.0094  -0.0057
-0.0023   0.0005   0.0025   0.0050   0.0096   0.0112   0.0122   0.0160
 0.0178   0.0216   0.0256   0.0309   0.0312   0.0317   0.0342   0.0453
 0.0478   0.0512   0.0526   0.0553   0.0558   0.0628   0.0662   0.0699
 0.0716   0.0738   0.0748   0.0789   0.0835   0.0893   0.0907   0.0960
 0.0974   0.0989   0.1040   0.1063   0.1082   0.1124   0.1153   0.1166
 0.1217   0.1269   0.1302   0.1318   0.1352   0.1394   0.1423   0.1493
 0.1561   0.1594   0.1619   0.1634   0.1665   0.1688   0.1718   0.1783
 0.1891   0.1909   0.1949   0.1988   0.2112   0.2128   0.2222   0.2280
 0.2323   0.2416   0.2511   0.2730   0.2870   0.2932   0.2944   0.3038
 0.3169   0.3313   0.3394   0.3418   0.3563   0.3578   0.3608   0.3655
 0.3700   0.3782   0.3884   0.3923   0.3980   0.4091   0.4205   0.4260
 0.4303   0.4363   0.4452   0.4558   0.4616   0.4636   0.4733   0.4788
 0.4855   0.4895   0.4978   0.5032   0.5093   0.5148   0.5158   0.5198
 0.5237   0.5325   0.5346   0.5389   0.5465   0.5543   0.5578   0.5628
 0.5655   0.5702   0.5786   0.5824   0.5832   0.5915   0.5949   0.6004
 0.6072   0.6186   0.6265   0.6334   0.6447   0.6512   0.6588   0.6612
 0.6772   0.6813   0.6825   0.6877   0.6941   0.7051   0.7084   0.7106
 0.7217   0.7281   0.7388   0.7508   0.7587   0.7624   0.7763   0.7952
 0.7991   0.8055   0.8094   0.8153   0.8279   0.8334   0.8499   0.8549
 0.8631   0.8712   0.8759   0.8911   0.8951   0.9087   0.9136   0.9373
 0.9458   0.9664   0.9758   0.9811   0.9895   1.0027   1.0236   1.0360
 1.0560   1.0841   1.1036   1.1243   1.1388   1.1461   1.1557   1.1788
 1.1880   1.2041   1.2153   1.2407   1.2633   1.2830   1.2967   1.3117
 1.3159   1.3232   1.3314   1.3435   1.3476   1.3575   1.3659   1.3747
 1.3875   1.3915   1.3946   1.3953   1.4017   1.4069   1.4204   1.4322
 1.4356   1.4537   1.4584   1.4619   1.4663   1.4843   1.4884   1.4992
 1.5041   1.5092   1.5122   1.5134   1.5244   1.5422   1.5463   1.5523
 1.5648   1.5674   1.5727   1.5734   1.6005   1.6052   1.6130   1.6191
 1.6295   1.6344   1.6365   1.6476   1.6543   1.6752   1.6840   1.6993
 1.7227   1.7287   1.7358   1.7434   1.7514   1.7544   1.7617   1.7743
 1.7846   1.7979   1.8069   1.8203   1.8476   1.8657   1.8689   1.8781
 1.8895   1.9164   1.9231   1.9444   1.9563   1.9669   1.9842   2.0016
 2.0080   2.0177   2.0337   2.0408   2.0491   2.0691   2.0741   2.0810
 2.1049   2.1121   2.1240   2.1470   2.1599   2.2008   2.2220   2.2324
 2.2435   2.2471   2.2530   2.2576   2.2646   2.2761   2.2902   2.2918
 2.2953   2.3180   2.3530   2.3604   2.3771   2.3782   2.3931   2.4112
 2.4151   2.4200   2.4297   2.4412   2.4436   2.4582   2.4764   2.4853
 2.4973   2.5027   2.5168   2.5323   2.5382   2.5505   2.5523   2.5547
 2.5662   2.5747   2.5830   2.5883   2.5946   2.5992   2.6165   2.6253
 2.6403   2.6487   2.6568   2.6683   2.6784   2.6845   2.7005   2.7033
 2.7154   2.7214   2.7280   2.7467   2.7590   2.7654   2.7691   2.7871
 2.7981   2.8153   2.8275   2.8393   2.8503   2.8691   2.8805   2.8922
 2.9030   2.9288   2.9529   2.9856   3.0638   3.0827   3.1224   3.1408
 3.1752   3.2487   3.2537   3.2793   3.2992   3.3247   3.3651   3.4108
 3.4285   3.4780   3.5250   3.5480   3.5572   3.5980   3.6052   3.6427
 3.6685   3.6815   3.7191   3.7299   3.7374   3.7459   3.7631   3.7682
 3.7882   3.8277   3.8697   3.9088   3.9326   3.9782   4.0501   4.0818
 4.1138   4.1842   4.2965   4.3107   4.6018   4.6652   4.6999   4.7238
 4.9254   5.0415   5.1992  23.5210  23.5852  23.7198  23.7338  23.7477
23.7750  23.8049  23.8339  23.8405  23.8886  23.9254  23.9683  23.9758
24.0365  35.4211  35.4611  35.5228  35.6191
--------------------------------------------------------------

          Ground-State Mulliken Net Atomic Charges

     Atom                 Charge (a.u.)
  ----------------------------------------
      1 N                     0.309136
      2 C                     0.171140
```

```
     3  C                     -0.004058
     4  C                     -0.172595
     5  C                     -0.145579
     6  C                     -0.130143
     7  C                     -0.742262
     8  C                     -0.322990
     9  N                     -0.092748
    10  C                     -0.389800
    11  N                      0.025107
    12  C                      0.300450
    13  C                     -0.214058
    14  C                      0.188418
    15  C                     -0.377304
    16  C                     -0.096793
    17  C                     -0.603884
    18  N                     -0.492401
    19  H                      0.221382
    20  H                      0.234904
    21  H                      0.227325
    22  H                      0.241120
    23  H                      0.232233
    24  H                      0.217809
    25  H                      0.226659
    26  H                      0.255088
    27  H                      0.254944
    28  H                      0.393887
    29  H                      0.213904
    30  H                      0.240115
    31  H                      0.204995
    32  H                      0.197951
    33  H                      0.172421
    34  H                      0.177180
    35  H                      0.197783
    36  H                      0.170516
    37  H                      0.331662
    38  H                      0.378484
 ----------------------------------------
 Sum of atomic charges =      2.000000

-----------------------------------------------------------------
                    Cartesian Multipole Moments
-----------------------------------------------------------------
   Charge (ESU x 10^10)
                9.6064
   Dipole Moment (Debye)
        X       6.8231      Y      -0.1346      Z      -5.4168
      Tot       8.7129
   Quadrupole Moments (Debye-Ang)
       XX     -24.5477     XY      26.2110     YY     -70.1207
       XZ     -43.6697     YZ     -19.5823     ZZ     -49.2528
   Traceless Quadrupole Moments (Debye-Ang)
      QXX      70.2780    QYY     -66.4409    QZZ      -3.8371
      QXY      78.6330    QXZ    -131.0090    QYZ     -58.7470
   Octopole Moments (Debye-Ang^2)
      XXX      56.4971    XXY      32.3192    XYY     -13.6512
      YYY     -21.7262    XXZ      -2.4407    XYZ     -19.4533
      YYZ      32.1196    XZZ      44.4771    YZZ      19.7381
      ZZZ     -79.2687
   Traceless Octopole Moments (Debye-Ang^2)
      XXX      61.5497    YYY    -598.8728    ZZZ    -742.7223
      XXY     393.7949    XXZ     112.1595    XYY    -466.7373
      XYZ    -291.7994    XZZ     405.1877    YYZ     630.5627
      YZZ     205.0779
   Hexadecapole Moments (Debye-Ang^3)
     XXXX   -3384.9784   XXXY    -192.5125   XXYY    -754.4994
     XYYY    -568.2408   YYYY    -700.1100   XXXZ     814.3330
     XXYZ     -98.7894   XYYZ     407.9167   YYYZ     391.5689
     XXZZ    -732.1719   XYZZ       0.8269   YYZZ    -589.2588
     XZZZ     845.7197   YZZZ     232.9613   ZZZZ   -1947.1405
   Traceless Hexadecapole Moments (Debye-Ang^3)
```

```
     XXXX   -8631.0670   XXXY   13982.8743   XXXZ   -7553.6562
     XXYY   -6041.9330   XXYZ  -18244.5746   XXZZ   14673.0000
     XYYY  -25468.5942   XYYZ   11811.7132   XYZZ   11485.7199
     XZZZ   -4258.0571   YYYY   18779.7850   YYYZ   17499.6530
     YYZZ  -12737.8520   YZZZ     744.9216   ZZZZ   -1935.1480
 -----------------------------------------------------------------

--------------------------------------------------
-  Entering drvman on Sun Oct 13 21:08:18 2024  -
--------------------------------------------------

 Calculating analytic gradient of the SCF energy
 Gradient of SCF Energy
           1           2           3           4           5           6
    1  -0.0003113  -0.0003092   0.0001738  -0.0001664  -0.0001905   0.0002563
    2   0.0001077   0.0003023   0.0000209  -0.0001334  -0.0000142   0.0001974
    3   0.0000581  -0.0001506   0.0003782   0.0000519   0.0004022  -0.0005204
           7           8           9          10          11          12
    1   0.0000019   0.0004860  -0.0001569   0.0003691   0.0003483  -0.0000151
    2  -0.0007032  -0.0000634   0.0004369  -0.0008052   0.0007689  -0.0012031
    3   0.0001078   0.0001785  -0.0004484   0.0003318   0.0001443  -0.0001048
          13          14          15          16          17          18
    1  -0.0007325   0.0001024  -0.0000183   0.0000490  -0.0000082   0.0004117
    2   0.0004793  -0.0002330   0.0000766   0.0000134   0.0000200   0.0004626
    3  -0.0003025   0.0002166   0.0000556  -0.0001055   0.0000191   0.0002393
          19          20          21          22          23          24
    1   0.0000977   0.0000751   0.0000527  -0.0000978   0.0000016  -0.0001273
    2  -0.0000723  -0.0000433   0.0000464  -0.0000739   0.0002188   0.0001406
    3  -0.0000359  -0.0000710  -0.0000166  -0.0000864   0.0000525   0.0000474
          25          26          27          28          29          30
    1  -0.0001194   0.0001450   0.0001013  -0.0001029  -0.0000532  -0.0000699
    2   0.0001354   0.0001172   0.0000264  -0.0000325  -0.0001272  -0.0000486
    3  -0.0001117   0.0000638  -0.0000919   0.0000452   0.0000403  -0.0000038
          31          32          33          34          35          36
    1  -0.0000231  -0.0000185   0.0000019   0.0000104   0.0000295   0.0000149
    2  -0.0000126   0.0000152   0.0000009   0.0000002   0.0000057  -0.0000024
    3   0.0000057   0.0000260   0.0000191  -0.0000054   0.0000196   0.0000180
          37          38
    1   0.0000887  -0.0002966
    2   0.0000277  -0.0000525
    3  -0.0000461  -0.0004203
 Max gradient component =       1.203E-03
 RMS gradient           =       2.549E-04
 Gradient time:  CPU 154.64 s  wall 257.50 s

--------------------------------------------------
-  Entering optman on Sun Oct 13 21:12:35 2024  -
--------------------------------------------------

 Geometry Optimization Parameters
   NAtoms,     NIC,      NZ,  NCons,   NDum,   NFix, NCnnct, MaxDiis
       38     272       0       0       0       0       0       0

 Cartesian Hessian Update
 Hessian updated using BFGS update


** GEOMETRY OPTIMIZATION IN DELOCALIZED INTERNAL COORDINATES **
   Searching for a Minimum

   Optimization Cycle:  13

                    Coordinates (Angstroms)
     ATOM              X               Y               Z
      1  N         2.2003770944    0.4055623983   -1.5067476375
      2  C         3.3848051763    1.0407846899   -1.7477221136
      3  C         3.6943196165    1.3788633426   -3.0577688125
      4  C         2.8323028428    1.0722102575   -4.0975344355
      5  C         1.6393158922    0.4106494270   -3.8188140785
      6  C         1.3556383435    0.0859711869   -2.5179742574
```

| | | | | |
|---|---|---|---|---|
| 7 | C | 4.3166547257 | 1.3471943613 | -0.6183420749 |
| 8 | C | 1.8868490360 | -0.0211873942 | -0.1080752143 |
| 9 | N | -1.2323567818 | -1.8115258748 | 0.8973744617 |
| 10 | C | -2.1535509769 | -0.8088053165 | 0.9638132878 |
| 11 | N | -1.8268934228 | 0.4128796492 | 0.6314980958 |
| 12 | C | -0.5725975924 | 0.6953199075 | 0.2399389444 |
| 13 | C | 0.4417800331 | -0.3206593743 | 0.1712885350 |
| 14 | C | 0.0448439202 | -1.5761061287 | 0.5214100827 |
| 15 | C | -3.5062661667 | -1.1207851534 | 1.4926163980 |
| 16 | C | -3.5393193516 | -0.9275367052 | 3.0286173578 |
| 17 | C | -4.9308453936 | -1.1997430487 | 3.5895364320 |
| 18 | N | -0.3371453720 | 1.9624125365 | -0.0801759039 |
| 19 | H | 4.6336016511 | 1.8811258698 | -3.2508265362 |
| 20 | H | 3.0892974613 | 1.3384826977 | -5.1163021845 |
| 21 | H | 0.9403919139 | 0.1422789716 | -4.5997602250 |
| 22 | H | 0.4526521812 | -0.4404807128 | -2.2419624392 |
| 23 | H | 5.2458353668 | 1.7526454908 | -1.0144256813 |
| 24 | H | 4.5614399647 | 0.4574033506 | -0.0338121766 |
| 25 | H | 3.8977525618 | 2.0989672462 | 0.0585170970 |
| 26 | H | 2.2211449252 | 0.7770843300 | 0.5566464236 |
| 27 | H | 2.5005807025 | -0.8973867071 | 0.1104384416 |
| 28 | H | -1.5114181065 | -2.7476530091 | 1.1675265460 |
| 29 | H | 0.7135033105 | -2.4292751523 | 0.5266045669 |
| 30 | H | -4.2179902981 | -0.4426200663 | 1.0198316205 |
| 31 | H | -3.7889761999 | -2.1469159334 | 1.2376965422 |
| 32 | H | -3.2334245448 | 0.0969830164 | 3.2590141420 |
| 33 | H | -2.8094290399 | -1.5967177053 | 3.4979552107 |
| 34 | H | -5.6692616085 | -0.5189293707 | 3.1599863399 |
| 35 | H | -4.9341633945 | -1.0575666213 | 4.6710353621 |
| 36 | H | -5.2507725944 | -2.2244395150 | 3.3854467503 |
| 37 | H | 0.5594499560 | 2.3072519173 | -0.3786546625 |

***Abridged***Abridged***Abridged***

| | | | | | | | |
|---|---|---|---|---|---|---|---|
| 0.0470 | 0.0514 | 0.0545 | 0.0560 | 0.0581 | 0.0643 | 0.0680 | 0.0694 |
| 0.0728 | 0.0730 | 0.0768 | 0.0781 | 0.0834 | 0.0884 | 0.0925 | 0.0955 |
| 0.0983 | 0.1006 | 0.1060 | 0.1082 | 0.1123 | 0.1143 | 0.1162 | 0.1177 |
| 0.1228 | 0.1262 | 0.1318 | 0.1321 | 0.1352 | 0.1405 | 0.1447 | 0.1499 |
| 0.1594 | 0.1621 | 0.1647 | 0.1675 | 0.1699 | 0.1711 | 0.1749 | 0.1839 |
| 0.1887 | 0.1921 | 0.1946 | 0.1986 | 0.2078 | 0.2130 | 0.2201 | 0.2274 |
| 0.2304 | 0.2335 | 0.2399 | 0.2601 | 0.2653 | 0.2714 | 0.2771 | 0.2894 |
| 0.2962 | 0.3039 | 0.3195 | 0.3236 | 0.3323 | 0.3381 | 0.3427 | 0.3520 |
| 0.3536 | 0.3554 | 0.3612 | 0.3633 | 0.3702 | 0.3781 | 0.3829 | 0.3896 |
| 0.3906 | 0.4003 | 0.4054 | 0.4093 | 0.4103 | 0.4163 | 0.4247 | 0.4278 |
| 0.4303 | 0.4389 | 0.4433 | 0.4477 | 0.4488 | 0.4570 | 0.4609 | 0.4670 |
| 0.4679 | 0.4696 | 0.4736 | 0.4796 | 0.4867 | 0.4937 | 0.4954 | 0.4971 |
| 0.5028 | 0.5060 | 0.5093 | 0.5150 | 0.5199 | 0.5203 | 0.5227 | 0.5286 |
| 0.5314 | 0.5340 | 0.5385 | 0.5407 | 0.5444 | 0.5487 | 0.5556 | 0.5586 |
| 0.5629 | 0.5663 | 0.5724 | 0.5748 | 0.5769 | 0.5804 | 0.5855 | 0.5949 |
| 0.5967 | 0.6000 | 0.6146 | 0.6173 | 0.6220 | 0.6326 | 0.6346 | 0.6368 |
| 0.6412 | 0.6440 | 0.6544 | 0.6577 | 0.6669 | 0.6721 | 0.6821 | 0.6889 |
| 0.6919 | 0.6966 | 0.7038 | 0.7144 | 0.7189 | 0.7233 | 0.7383 | 0.7425 |
| 0.7490 | 0.7502 | 0.7586 | 0.7655 | 0.7696 | 0.7765 | 0.7837 | 0.7980 |
| 0.8053 | 0.8193 | 0.8227 | 0.8282 | 0.8324 | 0.8413 | 0.8442 | 0.8484 |
| 0.8508 | 0.8570 | 0.8684 | 0.8739 | 0.8779 | 0.8925 | 0.8965 | 0.9035 |
| 0.9120 | 0.9164 | 0.9269 | 0.9310 | 0.9393 | 0.9439 | 0.9601 | 0.9624 |
| 0.9767 | 0.9805 | 0.9917 | 1.0021 | 1.0082 | 1.0105 | 1.0168 | 1.0214 |
| 1.0259 | 1.0324 | 1.0404 | 1.0464 | 1.0487 | 1.0578 | 1.0712 | 1.0759 |
| 1.0834 | 1.0893 | 1.0955 | 1.0966 | 1.1028 | 1.1095 | 1.1148 | 1.1198 |
| 1.1254 | 1.1307 | 1.1396 | 1.1428 | 1.1571 | 1.1614 | 1.1682 | 1.1711 |
| 1.1743 | 1.1816 | 1.1868 | 1.1977 | 1.2038 | 1.2049 | 1.2070 | 1.2184 |
| 1.2219 | 1.2277 | 1.2323 | 1.2392 | 1.2444 | 1.2488 | 1.2617 | 1.2681 |
| 1.2772 | 1.2835 | 1.2935 | 1.2968 | 1.2989 | 1.3068 | 1.3199 | 1.3236 |
| 1.3283 | 1.3311 | 1.3356 | 1.3373 | 1.3474 | 1.3513 | 1.3580 | 1.3620 |
| 1.3716 | 1.3769 | 1.3876 | 1.3925 | 1.4042 | 1.4124 | 1.4154 | 1.4258 |
| 1.4312 | 1.4403 | 1.4481 | 1.4576 | 1.4596 | 1.4651 | 1.4723 | 1.4742 |
| 1.4896 | 1.4961 | 1.5045 | 1.5122 | 1.5207 | 1.5281 | 1.5393 | 1.5465 |
| 1.5627 | 1.5686 | 1.5731 | 1.5831 | 1.5889 | 1.6015 | 1.6090 | 1.6187 |
| 1.6326 | 1.6481 | 1.6659 | 1.6948 | 1.7040 | 1.7260 | 1.7590 | 1.7754 |
| 1.7855 | 1.8082 | 1.8097 | 1.8282 | 1.8362 | 1.8753 | 1.8803 | 1.9134 |

| | | | | | | | |
|---|---|---|---|---|---|---|---|
| 1.9443 | 1.9514 | 1.9934 | 1.9984 | 2.0484 | 2.1016 | 2.1499 | 2.1775 |
| 2.2001 | 2.2566 | 2.3287 | 2.3606 | 2.3724 | 2.3789 | 2.4013 | 2.4441 |
| 2.4581 | 2.4634 | 2.4734 | 2.4744 | 2.4983 | 2.5076 | 2.5124 | 2.5208 |
| 2.5259 | 2.5384 | 2.5447 | 2.5577 | 2.5716 | 2.5719 | 2.5850 | 2.5923 |
| 2.6003 | 2.6069 | 2.6154 | 2.6173 | 2.6210 | 2.6393 | 2.6428 | 2.6598 |
| 2.6642 | 2.6725 | 2.6783 | 2.6806 | 2.6904 | 2.6938 | 2.6973 | 2.7026 |
| 2.7118 | 2.7186 | 2.7261 | 2.7291 | 2.7386 | 2.7480 | 2.7574 | 2.7576 |
| 2.7685 | 2.7818 | 2.7880 | 2.7936 | 2.7985 | 2.8027 | 2.8103 | 2.8235 |
| 2.8349 | 2.8372 | 2.8571 | 2.8590 | 2.8631 | 2.8753 | 2.8799 | 2.8850 |
| 2.8920 | 2.8977 | 2.9135 | 2.9204 | 2.9231 | 2.9305 | 2.9370 | 2.9409 |
| 2.9481 | 2.9607 | 2.9703 | 2.9748 | 2.9864 | 2.9935 | 2.9981 | 3.0056 |
| 3.0071 | 3.0193 | 3.0223 | 3.0371 | 3.0402 | 3.0513 | 3.0576 | 3.0595 |
| 3.0630 | 3.0728 | 3.0781 | 3.0847 | 3.0931 | 3.0990 | 3.1080 | 3.1099 |
| 3.1205 | 3.1219 | 3.1314 | 3.1418 | 3.1507 | 3.1594 | 3.1733 | 3.1750 |
| 3.1801 | 3.1870 | 3.1948 | 3.1982 | 3.2102 | 3.2124 | 3.2208 | 3.2303 |
| 3.2350 | 3.2378 | 3.2434 | 3.2567 | 3.2604 | 3.2654 | 3.2717 | 3.2752 |
| 3.2818 | 3.2882 | 3.2929 | 3.2966 | 3.3019 | 3.3107 | 3.3211 | 3.3323 |
| 3.3354 | 3.3448 | 3.3519 | 3.3580 | 3.3613 | 3.3682 | 3.3781 | 3.3874 |
| 3.3992 | 3.4070 | 3.4132 | 3.4257 | 3.4336 | 3.4504 | 3.4547 | 3.4649 |
| 3.4734 | 3.4800 | 3.4895 | 3.4963 | 3.5061 | 3.5132 | 3.5175 | 3.5194 |
| 3.5329 | 3.5420 | 3.5524 | 3.5589 | 3.5607 | 3.5691 | 3.5756 | 3.5999 |
| 3.6143 | 3.6226 | 3.6253 | 3.6321 | 3.6444 | 3.6484 | 3.6549 | 3.6594 |
| 3.6617 | 3.6743 | 3.6780 | 3.6835 | 3.6991 | 3.7010 | 3.7143 | 3.7279 |
| 3.7380 | 3.7433 | 3.7615 | 3.7627 | 3.7770 | 3.7782 | 3.7870 | 3.7927 |
| 3.8028 | 3.8128 | 3.8175 | 3.8295 | 3.8366 | 3.8472 | 3.8500 | 3.8597 |
| 3.8656 | 3.8887 | 3.8973 | 3.8991 | 3.9153 | 3.9281 | 3.9330 | 3.9439 |
| 3.9519 | 3.9548 | 3.9607 | 3.9677 | 3.9799 | 3.9828 | 3.9900 | 4.0159 |
| 4.0219 | 4.0241 | 4.0434 | 4.0478 | 4.0559 | 4.0570 | 4.0686 | 4.0777 |
| 4.0863 | 4.0999 | 4.1150 | 4.1243 | 4.1281 | 4.1362 | 4.1450 | 4.1594 |
| 4.1653 | 4.1735 | 4.1840 | 4.1852 | 4.1929 | 4.1988 | 4.2050 | 4.2108 |
| 4.2263 | 4.2292 | 4.2410 | 4.2452 | 4.2617 | 4.2801 | 4.3026 | 4.3096 |
| 4.3165 | 4.3238 | 4.3256 | 4.3386 | 4.3444 | 4.3510 | 4.3672 | 4.3758 |
| 4.3899 | 4.3949 | 4.4018 | 4.4142 | 4.4240 | 4.4306 | 4.4352 | 4.4435 |
| 4.4477 | 4.4680 | 4.4693 | 4.4800 | 4.4898 | 4.5008 | 4.5098 | 4.5305 |
| 4.5430 | 4.5567 | 4.5635 | 4.5846 | 4.5918 | 4.6049 | 4.6233 | 4.6337 |
| 4.6525 | 4.6552 | 4.6703 | 4.6852 | 4.6969 | 4.7252 | 4.7364 | 4.7631 |
| 4.7763 | 4.7963 | 4.8166 | 4.8349 | 4.8619 | 4.8812 | 4.9014 | 4.9091 |
| 4.9282 | 4.9417 | 4.9675 | 4.9779 | 4.9840 | 4.9892 | 5.0070 | 5.0433 |
| 5.0513 | 5.0715 | 5.0774 | 5.0913 | 5.1071 | 5.1144 | 5.1306 | 5.1684 |
| 5.1949 | 5.2177 | 5.2436 | 5.2510 | 5.2701 | 5.2857 | 5.3015 | 5.3527 |
| 5.3740 | 5.4000 | 5.4263 | 5.4482 | 5.4642 | 5.5192 | 5.5271 | 5.5526 |
| 5.5841 | 5.5984 | 5.6000 | 5.6167 | 5.6377 | 5.6529 | 5.6620 | 5.6665 |
| 5.6699 | 5.6913 | 5.7133 | 5.7333 | 5.7615 | 5.7710 | 5.7972 | 5.8267 |
| 5.8855 | 5.8991 | 5.9400 | 5.9898 | 6.0314 | 6.0665 | 6.0713 | 6.1309 |
| 6.1454 | 6.2397 | 6.2880 | 6.3303 | 6.5348 | 6.5917 | 6.7926 | 23.9665 |
| 24.1284 | 24.1454 | 24.2749 | 24.2784 | 24.2840 | 24.3078 | 24.3190 | 24.4323 |
| 24.5953 | 24.6143 | 24.6276 | 24.7983 | 24.9248 | 35.7545 | 35.7683 | 35.7886 |
| 35.9387 | | | | | | | |

---

Ground-State Mulliken Net Atomic Charges

| Atom | | Charge (a.u.) |
|---|---|---|
| 1 | N | 0.327140 |
| 2 | C | 0.192186 |
| 3 | C | -0.173387 |
| 4 | C | -0.146459 |
| 5 | C | -0.219679 |
| 6 | C | -0.098203 |
| 7 | C | -0.361980 |
| 8 | C | -0.174759 |
| 9 | N | -0.117237 |
| 10 | C | -0.027963 |
| 11 | N | -0.290991 |
| 12 | C | 0.509845 |
| 13 | C | -0.220233 |
| 14 | C | 0.175989 |
| 15 | C | -0.242234 |
| 16 | C | -0.036348 |

```
    17 C                      -0.459638
    18 N                      -0.397047
    19 H                       0.197962
    20 H                       0.213529
    21 H                       0.202407
    22 H                       0.227802
    23 H                       0.172419
    24 H                       0.166987
    25 H                       0.160651
    26 H                       0.199546
    27 H                       0.176398
    28 H                       0.292433
    29 H                       0.194325
    30 H                       0.169022
    31 H                       0.147998
    32 H                       0.146286
    33 H                       0.122379
    34 H                       0.133793
    35 H                       0.146869
    36 H                       0.127260
    37 H                       0.250284
    38 H                       0.312645
 ----------------------------------------
 Sum of atomic charges =     2.000000

-----------------------------------------------------------------
                   Cartesian Multipole Moments
-----------------------------------------------------------------
   Charge (ESU x 10^10)
                9.6064
   Dipole Moment (Debye)
        X       6.7759      Y       0.0961      Z      -5.3088
      Tot       8.6084
   Quadrupole Moments (Debye-Ang)
        XX     -24.3366     XY      25.7537     YY     -70.5389
        XZ     -43.8114     YZ     -19.4257     ZZ     -49.1198
   Traceless Quadrupole Moments (Debye-Ang)
      QXX      70.9854    QYY     -67.6214    QZZ      -3.3640
      QXY      77.2612    QXZ    -131.4341    QYZ     -58.2772
   Octopole Moments (Debye-Ang^2)
      XXX      56.1005    XXY      31.1943    XYY     -13.8797
      YYY     -19.8549    XXZ      -2.3885    XYZ     -19.1390
      YYZ      32.8476    XZZ      44.5498    YZZ      19.2160
      ZZZ     -81.9322
   Traceless Octopole Moments (Debye-Ang^2)
      XXX      60.5725    YYY    -572.8225    ZZZ    -765.7258
      XXY     376.2485    XXZ     118.5919    XYY    -468.5077
      XYZ    -287.0855    XZZ     407.9352    YYZ     647.1338
      YZZ     196.5740
   Hexadecapole Moments (Debye-Ang^3)
     XXXX   -3394.8639   XXXY    -196.0210   XXYY    -764.3746
     XYYY    -573.5985   YYYY    -701.9501   XXXZ     808.4885
     XXYZ     -97.0849   XYYZ     410.1964   YYYZ     395.9734
     XXZZ    -734.9092   XYZZ       0.0509   YYZZ    -588.2641
     XZZZ     847.2823   YZZZ     232.8333   ZZZZ   -1947.4380
   Traceless Hexadecapole Moments (Debye-Ang^3)
     XXXX   -7961.5456   XXXY   14048.3822   XXXZ   -8077.2281
     XXYY   -6686.3322   XXYZ  -18157.2421   XXZZ   14647.8778
     XYYY  -25597.2520   XYYZ   12081.1144   XYZZ   11548.8698
     XZZZ   -4003.8864   YYYY   19234.1037   YYYZ   17687.2220
     YYZZ  -12547.7715   YZZZ     470.0201   ZZZZ   -2100.1063
-----------------------------------------------------------------
Total job time:  544.04s(wall), 389.28s(cpu)
Mon Oct 14 03:46:12 2024
```

```
Wavefunction Version of Q-Chem

 Parts of Q-Chem use Armadillo 8.300.2 (Tropical Shenanigans).
 http://arma.sourceforge.net/

 Q-Chem begins on Sun Oct 13 12:03:21 2024


     Scratch files written to
C:/Users/hille/AppData/Local/Temp/WFB0CB2A5C99266D7A/Conformer.3//scratch///
Processing default memory
  ... MEM_TOTAL 4076 MB (default) [16 cores]
Processing $rem in C:/Program
Files/Wavefunction/Spartan24v110/P4e//../auxdir/config/preferences:
   (site specific preferences)
  ... THRESH             9
  ... SMALL_PROD_XCMAT   9
  ... BASIS_LIN_DEP_THRESH     5
  ... SCF_ALGORITHM     DIIS_GDM
  ... MAXSCF            250
  ... MAXDIIS          45
  ... THRESHDIIS         -1  (i.e. don't switch on delta-E)
  ... ECP_FIT          TRUE (Convert deprecated ECP files)
  ... GUI              GUI_SPARTAN
  ... TERSE_OUTPUT     TRUE !turn on spartan printing
  ... SCF_CONVERGENCE  7
  ... CCMAN2 FALSE  (qc4.3)
  ... SYMMETRY   FALSE  ! turn of symmetry for spartan16
  ... SYM_IGNORE TRUE   ! ..use FORCESYMMETRY to override
  ... GEOM_OPT_TOL_GRADIENT       700  ! loosen tolernaces for organic geometries
  ... GEOM_OPT_TOL_DISPLACEMENT  1400  ! was 1200 = .0012
  ... GEOM_OPT_TOL_ENERGY        2000  ! was  100 = .000 001
  ... GEN_SCFMAN  FALSE
Processing $rem in input file
  ... JOBTYPE    OPT
  ... TIDY_SYM   TRUE
  ... METHOD            WB97X-D
  ... xc_grid           75000302  (75,302)
  ... BASIS             6-311+G**
  ... THRESH             12  #diffuse default
  ... MAXSCF            350  #diffuse default
  ... GEOM_OPT_TOL_ENERGY       22850
  ... GEOM_OPT_TOL_GRADIENT       800
  ... VARTHRESH         2    (default DFT)
  ... INCDFT            TRUE (default DFT)
  ... GEOM_OPT_HESSIAN      READ (main opt)
  ... EXTERNAL_HESSIAN      1
  ... GUI                GUI_SPARTAN
  ... TERSE_OUTPUT       TRUE
NAlpha2: 130
NElect   130
Mult     1

Checking the input file for inconsistencies...  ...done.

--------------------------------------------------------------
User input:
--------------------------------------------------------------
$comment
Molecule2
$end
$molecule
 2 1
    7      2.030245444    0.19561668493    -1.6408043884
    6     2.2544172626   -0.66999099434    -2.6789276147
    6     2.3532830319   -0.16409547572    -3.9861877353
    6     2.2445172767     1.1967991159    -4.2285080864
    6     2.0568429098     2.0546373447    -3.1615928974
    6     1.9654917192      1.535727074    -1.8785438164
```

```
    6      2.4394886032    -2.1467119578    -2.4620767118
    6      1.9328614765   -0.26474610499    -0.2206555456
    7      -1.680440305    -1.0232063253    0.57695450737
    6     -2.0288138878   0.084450743882     1.2468553802
    7     -1.1506403194     1.0838238569     1.4935257729
    6     0.10533009784     0.9401853834     1.0570459414
    6     0.52838939925   -0.16631500973    0.33160315745
    6    -0.42780494714    -1.1621203864    0.11048792514
    6     -3.4352698478    0.21010876868     1.7632800952
    6       -3.68273638   -0.62760198932     3.0187500832
    6     -2.9215028367   -0.12136015063     4.2363656127
    7     0.95603959612     1.9917731064     1.3600889127
    1      2.5345727297   -0.83218805841    -4.8291714011
    1      2.3302092264      1.586433328    -5.2429458256
    1      1.9970501995     3.1292587168    -3.3275671844
    1      1.8474562318     2.2032030625    -1.0291389755
    1      2.5293696053    -2.6846055646    -3.4118178445
    1      1.5806331463    -2.5678823162    -1.9336810476
    1      3.3534898127    -2.3298584515    -1.8893019869
    1      2.6503258138    0.33575191329    0.35139650919
    1      2.2895847082    -1.2941702841    -0.1359340687
    1     -2.3842799418     -1.749859101    0.43061801317
    1    -0.23382245807    -2.0826263466   -0.42973462749
    1     -3.6618272926     1.2644562858     1.9612261987
    1     -4.1208768055   -0.11575151209     0.9719746032
    1     -3.4187611309    -1.6763966355      2.837763657
    1      -4.754984575    -0.6034787755     3.2471809111
    1     -1.8395843159   -0.20807306898     4.0975552234
    1     -3.1920409468   -0.70916229842      5.119383683
    1     -3.1610874422    0.92689556562     4.4412852155
    1      1.7121476355     1.7448523188     1.9989191495
    1     0.40272750644     2.7162275375     1.8343292057
$end
$rem
JOBTYPE    OPT
TIDY_SYM   TRUE
METHOD          WB97X-D
xc_grid         75000302  (75,302)
BASIS           6-311+G**
THRESH           12  #diffuse default
MAXSCF          350  #diffuse default
GEOM_OPT_TOL_ENERGY     22850
GEOM_OPT_TOL_GRADIENT     800
VARTHRESH       2    (default DFT)
INCDFT          TRUE (default DFT)
GEOM_OPT_HESSIAN      READ (main opt)
EXTERNAL_HESSIAN      1
GUI              GUI_SPARTAN
TERSE_OUTPUT     TRUE
$end
$opt
$end
--------------------------------------------------------------
 --------------------------------------------------------------
             Standard Nuclear Orientation (Angstroms)
    I     Atom           X                Y                Z
 --------------------------------------------------------------
    1      N       2.0302454440     0.1956166849    -1.6408043884
    2      C       2.2544172626    -0.6699909943    -2.6789276147
    3      C       2.3532830319    -0.1640954757    -3.9861877353
    4      C       2.2445172767     1.1967991159    -4.2285080864
    5      C       2.0568429098     2.0546373447    -3.1615928974
    6      C       1.9654917192     1.5357270740    -1.8785438164
    7      C       2.4394886032    -2.1467119578    -2.4620767118
    8      C       1.9328614765    -0.2647461050    -0.2206555456
    9      N      -1.6804403050    -1.0232063253     0.5769545074
   10      C      -2.0288138878     0.0844507439     1.2468553802
   11      N      -1.1506403194     1.0838238569     1.4935257729
   12      C       0.1053300978     0.9401853834     1.0570459414
   13      C       0.5283893992    -0.1663150097     0.3316031575
```

```
  14      C      -0.4278049471    -1.1621203864     0.1104879251
  15      C      -3.4352698478     0.2101087687     1.7632800952
  16      C      -3.6827363800    -0.6276019893     3.0187500832
  17      C      -2.9215028367    -0.1213601506     4.2363656127
  18      N       0.9560395961     1.9917731064     1.3600889127
  19      H       2.5345727297    -0.8321880584    -4.8291714011
  20      H       2.3302092264     1.5864333280    -5.2429458256
  21      H       1.9970501995     3.1292587168    -3.3275671844
  22      H       1.8474562318     2.2032030625    -1.0291389755
  23      H       2.5293696053    -2.6846055646    -3.4118178445
  24      H       1.5806331463    -2.5678823162    -1.9336810476
  25      H       3.3534898127    -2.3298584515    -1.8893019869
  26      H       2.6503258138     0.3357519133     0.3513965092
  27      H       2.2895847082    -1.2941702841    -0.1359340687
  28      H      -2.3842799418    -1.7498591010     0.4306180132
  29      H      -0.2338224581    -2.0826263466    -0.4297346275
  30      H      -3.6618272926     1.2644562858     1.9612261987
  31      H      -4.1208768055    -0.1157515121     0.9719746032
  32      H      -3.4187611309    -1.6763966355     2.8377636570
  33      H      -4.7549845750    -0.6034787755     3.2471809111
  34      H      -1.8395843159    -0.2080730690     4.0975552234
  35      H      -3.1920409468    -0.7091622984     5.1193836830
  36      H      -3.1610874422     0.9268955656     4.4412852155
  37      H       1.7121476355     1.7448523188     1.9989191495
  38      H       0.4027275064     2.7162275375     1.8343292057
 ----------------------------------------------------------------
 Nuclear Repulsion Energy =        1302.12193566 hartrees
 There are       65 alpha and       65 beta electrons
 Requested basis set is 6-311+G(d,p)
 There are 188 shells and 516 basis functions

 Total QAlloc Memory Limit   4076 MB
 Mega-Array Size       188 MB
 MEM_STATIC part       192 MB

 ..                                                                       (5.2.P)

--------------------------------------------------
-  Entering fldman on Sun Oct 13 12:03:21 2024  -
--------------------------------------------------

 A cutoff of  1.0D-12 yielded  12589 shell pairs
 There are    100395 function pairs (    106995 Cartesian)
 Smallest overlap matrix eigenvalue = 2.14E-06
 Linear dependence detected in AO basis
 Tighter screening thresholds may be required for diffuse basis sets
 Use S2THRESH > 12 and THRESH = 14 in case of SCF convergence issues
 Number of orthogonalized atomic orbitals = 510
 Maximum deviation from orthogonality = 1.442E-11

 Scale SEOQF with 1.000000e-01/1.000000e-01/1.000000e-02

 Standard Electronic Orientation quadrupole field applied
 Nucleus-field energy     =     0.0000000290 hartrees

--------------------------------------------------
-  Entering gesman on Sun Oct 13 12:03:21 2024  -
--------------------------------------------------

 Guess from superposition of atomic densities
 Warning:  Energy on first SCF cycle will be non-variational
 SAD guess density has 132.000000 electrons

--------------------------------------------------
-  Entering scfman on Sun Oct 13 12:03:21 2024  -
--------------------------------------------------

 Long-range K will be added via erf
 Coulomb attenuation parameter = 0.2 bohr**(-1)
 A restricted hybrid HF-DFT SCF calculation will be
```

```
 performed using Pulay DIIS + Geometric Direct Minimization
 Exchange:     0.2220 Hartree-Fock + 1.0000 wB97X-D + LR-HF
 Correlation:  1.0000 wB97X-D
 Using Euler-Maclaurin-Lebedev (75,302) quadrature formula
 Dispersion:   Grimme D
 SCF converges when RMS gradient is below 1.0E-07
 Exchange:     0.2220 Hartree-Fock + 1.0000 wB97X-D + LR-HF
 Correlation:  1.0000 wB97X-D
 Using Euler-Maclaurin-Lebedev (75,302) quadrature formula
 Dispersion:   Grimme D
 ---------------------------------------
  Cycle       Energy         DIIS Error
 ---------------------------------------
    1    -770.2956793689      2.95E-02
    2    -763.7495415536      2.39E-03
    3    -763.7565143513      2.58E-03
    4    -763.9435642311      3.71E-04
    5    -763.9469199587      1.88E-04
    6    -763.9479290900      3.28E-05
    7    -763.9479611612      1.36E-05
    8    -763.9479669754      3.07E-06
    9    -763.9479674818      1.57E-06
   10    -763.9479675880      5.36E-07
   11    -763.9479676068      2.38E-07
   12    -763.9479676111      8.72E-08 Convergence criterion met
 ---------------------------------------
 SCF time:  CPU 333.39 s  wall 671.00 s
 SCF   energy in the final basis set = -763.94796761
 Total energy in the final basis set = -763.94796761

-------------------------------------------------
-  Entering anlman on Sun Oct 13 12:14:32 2024  -
-------------------------------------------------



 --------------------------------------------------------------

                   Orbital Energies (a.u.)
 --------------------------------------------------------------


 Alpha MOs
 -- Occupied --
-14.8149 -14.8143 -14.7237 -14.7168 -10.6760 -10.6716 -10.6647 -10.6377
-10.6343 -10.6205 -10.6144 -10.5761 -10.5680 -10.5668 -10.5490 -10.5337
-10.5011 -10.4637  -1.4306  -1.3957  -1.3461  -1.2677  -1.2277  -1.1993
 -1.1775  -1.1466  -1.1033  -1.0766  -1.0664  -1.0507  -1.0239  -1.0098
 -0.9844  -0.9516  -0.9483  -0.9080  -0.9012  -0.8833  -0.8776  -0.8660
 -0.8426  -0.8415  -0.8362  -0.8171  -0.8078  -0.7908  -0.7836  -0.7807
 -0.7720  -0.7562  -0.7482  -0.7445  -0.7386  -0.7248  -0.7216  -0.7075
 -0.6996  -0.6667  -0.6563  -0.6521  -0.6360  -0.6290  -0.6261  -0.6215
 -0.6114
 -- Virtual --
 -0.2798  -0.2685  -0.2570  -0.2221  -0.1786  -0.1640  -0.1458  -0.1447
 -0.1345  -0.1256  -0.1210  -0.1145  -0.1088  -0.1061  -0.0980  -0.0885
 -0.0874  -0.0843  -0.0820  -0.0802  -0.0767  -0.0722  -0.0698  -0.0649
 -0.0611  -0.0590  -0.0553  -0.0510  -0.0498  -0.0460  -0.0427  -0.0367
 -0.0288  -0.0279  -0.0237  -0.0228  -0.0219  -0.0176  -0.0136  -0.0102
 -0.0052  -0.0039  -0.0025   0.0014   0.0037   0.0112   0.0141   0.0162
  0.0193   0.0229   0.0270   0.0285   0.0319   0.0322   0.0375   0.0392
  0.0416   0.0419   0.0485   0.0531   0.0552   0.0596   0.0599   0.0640
  0.0687   0.0711   0.0749   0.0756   0.0791   0.0808   0.0830   0.0864
  0.0888   0.0915   0.0991   0.1016   0.1045   0.1077   0.1082   0.1147
  0.1197   0.1216   0.1248   0.1307   0.1334   0.1376   0.1419   0.1450
  0.1456   0.1537   0.1633   0.1643   0.1686   0.1708   0.1721   0.1767
  0.1811   0.1827   0.1880   0.1935   0.1978   0.2043   0.2062   0.2157
  0.2308   0.2381   0.2498   0.2635   0.2808   0.2906   0.3137   0.3150
  0.3259   0.3274   0.3325   0.3402   0.3479   0.3499   0.3629   0.3635
  0.3692   0.3721   0.3843   0.3895   0.3957   0.4000   0.4054   0.4183
  0.4269   0.4365   0.4407   0.4447   0.4529   0.4631   0.4692   0.4706
  0.4759   0.4907   0.4920   0.4985   0.5080   0.5116   0.5146   0.5185
```

| | | | | | | | |
|---|---|---|---|---|---|---|---|
| 0.5240 | 0.5270 | 0.5289 | 0.5357 | 0.5375 | 0.5431 | 0.5546 | 0.5574 |
| 0.5625 | 0.5641 | 0.5690 | 0.5701 | 0.5796 | 0.5852 | 0.5963 | 0.6043 |
| 0.6044 | 0.6227 | 0.6255 | 0.6272 | 0.6301 | 0.6404 | 0.6489 | 0.6613 |
| 0.6669 | 0.6718 | 0.6779 | 0.6817 | 0.6841 | 0.6983 | 0.7005 | 0.7033 |
| 0.7186 | 0.7215 | 0.7347 | 0.7414 | 0.7539 | 0.7633 | 0.7695 | 0.7785 |
| 0.7907 | 0.7967 | 0.8091 | 0.8150 | 0.8296 | 0.8335 | 0.8425 | 0.8500 |
| 0.8615 | 0.8666 | 0.8695 | 0.8820 | 0.8948 | 0.9053 | 0.9153 | 0.9208 |
| 0.9316 | 0.9533 | 0.9621 | 0.9776 | 0.9906 | 0.9976 | 1.0110 | 1.0219 |
| 1.0581 | 1.0777 | 1.0886 | 1.0963 | 1.1186 | 1.1637 | 1.1782 | 1.1962 |
| 1.2044 | 1.2154 | 1.2293 | 1.2621 | 1.2662 | 1.2759 | 1.2897 | 1.3042 |
| 1.3079 | 1.3162 | 1.3314 | 1.3365 | 1.3437 | 1.3691 | 1.3769 | 1.3804 |
| 1.3862 | 1.3975 | 1.3988 | 1.4059 | 1.4072 | 1.4132 | 1.4261 | 1.4355 |
| 1.4410 | 1.4446 | 1.4514 | 1.4553 | 1.4587 | 1.4708 | 1.4763 | 1.4818 |
| 1.4950 | 1.5004 | 1.5087 | 1.5243 | 1.5325 | 1.5382 | 1.5463 | 1.5520 |
| 1.5613 | 1.5673 | 1.5704 | 1.5843 | 1.5914 | 1.5971 | 1.5993 | 1.6138 |
| 1.6264 | 1.6302 | 1.6413 | 1.6501 | 1.6552 | 1.6638 | 1.6733 | 1.6821 |
| 1.6914 | 1.7010 | 1.7034 | 1.7191 | 1.7290 | 1.7456 | 1.7630 | 1.7721 |
| 1.7785 | 1.7880 | 1.7944 | 1.8185 | 1.8355 | 1.8362 | 1.8458 | 1.8659 |
| 1.8865 | 1.8886 | 1.9013 | 1.9199 | 1.9334 | 1.9595 | 1.9668 | 1.9913 |
| 1.9953 | 2.0064 | 2.0199 | 2.0242 | 2.0504 | 2.0553 | 2.0765 | 2.0909 |
| 2.0953 | 2.1091 | 2.1238 | 2.1389 | 2.1522 | 2.1787 | 2.2132 | 2.2174 |
| 2.2266 | 2.2331 | 2.2430 | 2.2664 | 2.2681 | 2.2756 | 2.2893 | 2.3002 |
| 2.3025 | 2.3133 | 2.3435 | 2.3617 | 2.3646 | 2.3733 | 2.3802 | 2.3964 |
| 2.3990 | 2.4131 | 2.4355 | 2.4430 | 2.4502 | 2.4659 | 2.4799 | 2.4869 |
| 2.5075 | 2.5083 | 2.5193 | 2.5252 | 2.5363 | 2.5455 | 2.5491 | 2.5542 |
| 2.5612 | 2.5759 | 2.5809 | 2.5859 | 2.5887 | 2.5981 | 2.6006 | 2.6100 |
| 2.6168 | 2.6321 | 2.6410 | 2.6526 | 2.6779 | 2.6814 | 2.6869 | 2.6944 |
| 2.7053 | 2.7146 | 2.7155 | 2.7237 | 2.7399 | 2.7642 | 2.7668 | 2.7754 |
| 2.7832 | 2.7922 | 2.8020 | 2.8168 | 2.8352 | 2.8556 | 2.8571 | 2.8855 |
| 2.9005 | 2.9233 | 2.9393 | 2.9839 | 3.0162 | 3.0522 | 3.0736 | 3.1135 |
| 3.1446 | 3.1626 | 3.2400 | 3.2629 | 3.2860 | 3.3104 | 3.3325 | 3.3812 |
| 3.4020 | 3.4629 | 3.4869 | 3.5359 | 3.5527 | 3.5891 | 3.6113 | 3.6496 |
| 3.6560 | 3.6844 | 3.7009 | 3.7088 | 3.7331 | 3.7541 | 3.7595 | 3.7694 |
| 3.8166 | 3.8656 | 3.8921 | 3.9063 | 3.9401 | 4.0311 | 4.0348 | 4.1016 |
| 4.1137 | 4.2056 | 4.3008 | 4.3116 | 4.5495 | 4.6405 | 4.6785 | 4.7070 |
| 4.8670 | 5.0270 | 5.1456 | 23.5224 | 23.5573 | 23.6805 | 23.7176 | 23.7362 |
| 23.7905 | 23.7964 | 23.8251 | 23.8448 | 23.8682 | 23.9143 | 23.9263 | 23.9322 |
| 24.0295 | 35.4143 | 35.4366 | 35.4829 | 35.5741 | | | |

Ground-State Mulliken Net Atomic Charges

| Atom | | Charge (a.u.) |
|---|---|---|
| 1 | N | 0.410163 |
| 2 | C | 0.150396 |
| 3 | C | -0.050553 |
| 4 | C | -0.141477 |
| 5 | C | -0.177171 |
| 6 | C | -0.181789 |
| 7 | C | -0.801221 |
| 8 | C | -0.269231 |
| 9 | N | -0.097399 |
| 10 | C | -0.212478 |
| 11 | N | 0.044707 |
| 12 | C | 0.047894 |
| 13 | C | 0.046858 |
| 14 | C | 0.086770 |
| 15 | C | -0.399235 |
| 16 | C | -0.224634 |
| 17 | C | -0.510075 |
| 18 | N | -0.470425 |
| 19 | H | 0.222172 |
| 20 | H | 0.232273 |
| 21 | H | 0.228524 |
| 22 | H | 0.253502 |
| 23 | H | 0.232327 |
| 24 | H | 0.165333 |
| 25 | H | 0.251083 |
| 26 | H | 0.238796 |

```
    27 H                     0.244298
    28 H                     0.401657
    29 H                     0.211130
    30 H                     0.249137
    31 H                     0.226763
    32 H                     0.167400
    33 H                     0.226418
    34 H                     0.138819
    35 H                     0.208504
    36 H                     0.189849
    37 H                     0.326529
    38 H                     0.334384
  ----------------------------------------
  Sum of atomic charges =     2.000000

 -----------------------------------------------------------------
                    Cartesian Multipole Moments
 -----------------------------------------------------------------
    Charge (ESU x 10^10)
                 9.6064
    Dipole Moment (Debye)
         X       3.7585      Y      -1.1724      Z      -5.3532
       Tot       6.6451
    Quadrupole Moments (Debye-Ang)
        XX     -45.8901     XY       9.1419     YY     -72.1257
        XZ     -41.2246     YZ       0.1246     ZZ     -35.6173
    Traceless Quadrupole Moments (Debye-Ang)
       QXX      15.9628    QYY     -62.7439    QZZ      46.7811
       QXY      27.4256    QXZ    -123.6739    QYZ       0.3739
    Octopole Moments (Debye-Ang^2)
       XXX     -89.8991    XXY     -32.6973    XYY      15.9472
       YYY     -27.0513    XXZ      23.3226    XYZ       6.0604
       YYZ     -16.7570    XZZ      66.5826    YZZ      20.9994
       ZZZ     -99.2476
    Traceless Octopole Moments (Debye-Ang^2)
       XXX   -1282.1633    YYY     -57.0269    ZZZ    -654.5761
       XXY    -374.2115    XXZ     627.8854    XYY     261.3161
       XYZ      90.9066    XZZ    1020.8472    YYZ      26.6907
       YZZ     431.2384
    Hexadecapole Moments (Debye-Ang^3)
      XXXX   -2577.0991   XXXY    -116.8990   XXYY    -556.8996
      XYYY     -70.6823   YYYY    -801.7574   XXXZ    1055.3111
      XXYZ      20.5497   XYYZ     352.4028   YYYZ      47.9627
      XXZZ    -674.5030   XYZZ      39.8470   YYZZ    -571.5128
      XZZZ     692.8228   YZZZ     -76.3424   ZZZZ   -2515.9925
    Traceless Hexadecapole Moments (Debye-Ang^3)
      XXXX  -13336.3701   XXXY   -5626.3487   XXXZ   16283.5124
      XXYY    -896.4248   XXYZ    2270.0295   XXZZ   14232.7948
      XYYY    -773.5983   XYYZ    5494.2435   XYZZ    6399.9470
      XZZZ  -21777.7560   YYYY    4024.6335   YYYZ    5373.0251
      YYZZ   -3128.2088   YZZZ   -7643.0546   ZZZZ  -11104.5861
 -----------------------------------------------------------------

--------------------------------------------------
-  Entering drvman on Sun Oct 13 12:14:32 2024  -
--------------------------------------------------

 Calculating analytic gradient of the SCF energy
 Gradient of SCF Energy
           1           2           3           4           5           6
    1  -0.0056091  -0.0020418   0.0008682  -0.0017524   0.0016917   0.0012471
    2   0.0001381   0.0031910  -0.0013361   0.0055327  -0.0043406  -0.0082122
    3   0.0087747   0.0172427  -0.0073746   0.0063357  -0.0140647   0.0103102
           7           8           9          10          11          12
    1   0.0034528  -0.0059783  -0.0043486   0.0045258   0.0219203   0.0032643
    2  -0.0030935   0.0048573   0.0014547  -0.0462474   0.0338889  -0.0271046
    3  -0.0046116   0.0010831   0.0004415  -0.0202410   0.0141999  -0.0228094
          13          14          15          16          17          18
    1   0.0083990  -0.0227604   0.0012846   0.0009600  -0.0008875   0.0138999
    2   0.0337171  -0.0086593  -0.0015556   0.0037165   0.0007316   0.0015815
```

```
    3   0.0186354   0.0011720   0.0109075  -0.0110148  -0.0021632  -0.0127127
           19          20          21          22          23          24
    1   0.0011742   0.0006010   0.0000262   0.0008625   0.0003730  -0.0022916
    2   0.0005008   0.0019361   0.0040673   0.0058418  -0.0056237   0.0026989
    3  -0.0080144  -0.0037498  -0.0046783  -0.0017616  -0.0027827  -0.0012399
           25          26          27          28          29          30
    1   0.0011489   0.0058265   0.0008125  -0.0049900  -0.0026106  -0.0054541
    2   0.0021181   0.0010939  -0.0029547  -0.0030006  -0.0017676   0.0031279
    3  -0.0010508   0.0020274   0.0048923  -0.0005889  -0.0000421   0.0010465
           31          32          33          34          35          36
    1  -0.0006088   0.0023887  -0.0023230  -0.0007121  -0.0012553  -0.0008424
    2   0.0003667   0.0005866   0.0004860  -0.0009976  -0.0017426   0.0013284
    3   0.0007138   0.0009734   0.0048152  -0.0014555   0.0017819  -0.0001920
           37          38
    1  -0.0044869  -0.0057743
    2   0.0003238   0.0033504
    3   0.0097180   0.0054769
 Max gradient component =       4.625E-02
 RMS gradient           =       9.424E-03
 Gradient time:  CPU 132.95 s  wall 306.80 s

-------------------------------------------------
-  Entering optman on Sun Oct 13 12:19:39 2024  -
-------------------------------------------------

 Geometry Optimization Parameters
   NAtoms,    NIC,     NZ,  NCons,   NDum,   NFix, NCnnct, MaxDiis
       38     272       0       0       0       0       0       0

 Cartesian Hessian read from HESS file


** GEOMETRY OPTIMIZATION IN DELOCALIZED INTERNAL COORDINATES **
   Searching for a Minimum

   Optimization Cycle:   1

                       Coordinates (Angstroms)
     ATOM                X               Y               Z
      1  N         2.0302454440    0.1956166849   -1.6408043884
      2  C         2.2544172626   -0.6699909943   -2.6789276147
      3  C         2.3532830319   -0.1640954757   -3.9861877353
      4  C         2.2445172767    1.1967991159   -4.2285080864
      5  C         2.0568429098    2.0546373447   -3.1615928974
      6  C         1.9654917192    1.5357270740   -1.8785438164
      7  C         2.4394886032   -2.1467119578   -2.4620767118
      8  C         1.9328614765   -0.2647461050   -0.2206555456
      9  N        -1.6804403050   -1.0232063253    0.5769545074
     10  C        -2.0288138878    0.0844507439    1.2468553802
     11  N        -1.1506403194    1.0838238569    1.4935257729
     12  C         0.1053300978    0.9401853834    1.0570459414
     13  C         0.5283893992   -0.1663150097    0.3316031575
     14  C        -0.4278049471   -1.1621203864    0.1104879251
     15  C        -3.4352698478    0.2101087687    1.7632800952
     16  C        -3.6827363800   -0.6276019893    3.0187500832
     17  C        -2.9215028367   -0.1213601506    4.2363656127
     18  N         0.9560395961    1.9917731064    1.3600889127
     19  H         2.5345727297   -0.8321880584   -4.8291714011
     20  H         2.3302092264    1.5864333280   -5.2429458256
     21  H         1.9970501995    3.1292587168   -3.3275671844
     22  H         1.8474562318    2.2032030625   -1.0291389755
     23  H         2.5293696053   -2.6846055646   -3.4118178445
     24  H         1.5806331463   -2.5678823162   -1.9336810476
     25  H         3.3534898127   -2.3298584515   -1.8893019869
     26  H         2.6503258138    0.3357519133    0.3513965092
     27  H         2.2895847082   -1.2941702841   -0.1359340687
     28  H        -2.3842799418   -1.7498591010    0.4306180132
     29  H        -0.2338224581   -2.0826263466   -0.4297346275
     30  H        -3.6618272926    1.2644562858    1.9612261987
     31  H        -4.1208768055   -0.1157515121    0.9719746032
```

```
    32  H        -3.4187611309   -1.6763966355    2.8377636570
    33  H        -4.7549845750   -0.6034787755    3.2471809111
    34  H        -1.8395843159   -0.2080730690    4.0975552234
    35  H        -3.1920409468   -0.7091622984    5.1193836830
    36  H        -3.1610874422    0.9268955656    4.4412852155
    37  H         1.7121476355    1.7448523188    1.9989191495
    38  H         0.4027275064    2.7162275375    1.8343292057
  Point Group: c1    Number of degrees of freedom:   108


  Energy is   -763.947967611


 Attempting to generate delocalized internal coordinates

 Transforming Cartesian Hessian to Internal Coordinates
 Hessian Transformation does not Include Derivative of B-matrix
  internal optimization (0)

108 Hessian modes will be used to form the next step
  Hessian Eigenvalues:
     0.001687    0.002667    0.004079    0.005873    0.009888    0.012552
     0.015355    0.018940    0.019418    0.019943    0.021430    0.021958
     0.022701    0.022816    0.024290    0.024471    0.025941    0.026583
     0.027230    0.028216    0.030039    0.031188    0.035952    0.037534
     0.038826    0.041322    0.043221    0.043813    0.045339    0.045736
     0.052414    0.053711    0.055515    0.058176    0.063650    0.078768
     0.085425    0.091748    0.121734    0.121782    0.127081    0.130514
     0.131571    0.132844    0.137739    0.139162    0.143249    0.146596
     0.147672    0.148759    0.149389    0.150869    0.151592    0.152496
     0.153080    0.153838    0.177442    0.206266    0.209170    0.215772
     0.220844    0.227124    0.237224    0.242054    0.246654    0.261290
     0.264058    0.268998    0.281704    0.289374    0.300155    0.300312
     0.300546    0.301163    0.301718    0.302508    0.303942    0.304374
     0.305119    0.305186    0.305325    0.308481    0.311283    0.312643
     0.318264    0.329250    0.331673    0.333786    0.339922    0.343192
     0.345877    0.348636    0.354371    0.362421    0.363109    0.382291
     0.391754    0.394413    0.401952    0.405522    0.415599    0.416636
     0.422620    0.435768    0.449995    0.473693    0.570909    0.759928

 Minimum search - taking simple RFO step
 Searching for Lamda that Minimizes Along All modes
 Value Taken    Lamda =  -0.02248313
 Calculated Step too Large.  Step scaled by  0.725025 !!
 Step Taken.  Stepsize is  0.300000  0.007678

                              Maximum     Tolerance    Cnvgd?
        Gradient             0.024118      0.000800      NO
        Displacement         0.103827      0.001400      NO
        Energy change       *********      0.000228      NO


 New Cartesian Coordinates Obtained by Inverse Iteration

 Displacement from previous Coordinates is:  0.437120
 ----------------------------------------------------------------
             Standard Nuclear Orientation (Angstroms)
    I     Atom           X                Y                Z
 ----------------------------------------------------------------
    1      N       2.0291132953     0.1845418137    -1.6531093845
    2      C       2.2551114754    -0.6792500200    -2.6859343209
    3      C       2.3532168326    -0.1672668655    -3.9720131331
    4      C       2.2389589272     1.1958124481    -4.2019352264
    5      C       2.0385889780     2.0558289721    -3.1284713684
    6      C       1.9473046771     1.5239287213    -1.8711235970
    7      C       2.4338991584    -2.1484185450    -2.4446104184
    8      C       1.9289579597    -0.2791398742    -0.2377010527
    9      N      -1.6631614245    -0.9885407745     0.5818667116
   10      C      -2.0378737838     0.1252158992     1.2512680273
   11      N      -1.1775477759     1.0886649009     1.4982944888
```

```
  12      C       0.0877257010     0.9462232190     1.0760980511
  13      C       0.5264497937    -0.1734941677     0.3209547629
  14      C      -0.4090477152    -1.1436438391     0.1105087573
  15      C      -3.4418620530     0.2191445366     1.7486028779
  16      C      -3.6762994985    -0.6355213380     3.0102563369
  17      C      -2.9111595920    -0.1342595390     4.2306577735
  18      N       0.9344499911     1.9980183691     1.3491029418
  19      H       2.5356147348    -0.8406966058    -4.8018183596
  20      H       2.3231673496     1.5890703783    -5.2105735208
  21      H       1.9681341270     3.1273321842    -3.2706021241
  22      H       1.8145251815     2.1560162101    -1.0017442293
  23      H       2.5284407681    -2.6724089398    -3.3950271196
  24      H       1.5830566671    -2.5860537419    -1.9170499379
  25      H       3.3440858520    -2.3420205752    -1.8688052694
  26      H       2.6308355508     0.3229801895     0.3390784947
  27      H       2.2881883625    -1.3036412145    -0.1679285762
  28      H      -2.3583684244    -1.7170618845     0.4358754969
  29      H      -0.2187635689    -2.0646414588    -0.4277183108
  30      H      -3.6647036172     1.2673426953     1.9551364920
  31      H      -4.1203096701    -0.1090114250     0.9523489380
  32      H      -3.4138975344    -1.6821501120     2.8137443687
  33      H      -4.7484729989    -0.6193142483     3.2208390625
  34      H      -1.8273354343    -0.2086424045     4.0954126738
  35      H      -3.1736550760    -0.7265569572     5.1091941061
  36      H      -3.1549629567     0.9096407102     4.4445316221
  37      H       1.7778085710     1.7790293541     1.8706573587
  38      H       0.4297871701     2.7329439288     1.8417366064
 ----------------------------------------------------------------
 Nuclear Repulsion Energy =        1305.79793010 hartrees
 There are       65 alpha and       65 beta electrons

--------------------------------------------------
-  Entering fldman on Sun Oct 13 12:19:39 2024  -
--------------------------------------------------

 Applying Cartesian multipole field
    Component          Value
    ---------          -----
     (2,0,0)        1.00000E-11
     (0,2,0)        2.00000E-11
     (0,0,2)       -3.00000E-12
 Nucleus-field energy     =     0.0000000290 hartrees

--------------------------------------------------
-  Entering gesman on Sun Oct 13 12:19:39 2024  -
--------------------------------------------------

 Requested basis set is 6-311+G(d,p)
 There are 188 shells and 516 basis functions
 A cutoff of  1.0D-12 yielded  12619 shell pairs
 There are    100510 function pairs (    107111 Cartesian)
 Smallest overlap matrix eigenvalue = 2.07E-06
 Linear dependence detected in AO basis
 Tighter screening thresholds may be required for diffuse basis sets
 Use S2THRESH > 12 and THRESH = 14 in case of SCF convergence issues
 Number of orthogonalized atomic orbitals = 510
 Maximum deviation from orthogonality = 1.053E-11
 Guess MOs from SCF MO coefficient file
 Reading MOs from coefficient file
 Reading MOs from coefficient file

--------------------------------------------------
-  Entering scfman on Sun Oct 13 12:19:40 2024  -
--------------------------------------------------

 Long-range K will be added via erf
 Coulomb attenuation parameter = 0.2 bohr**(-1)
 A restricted hybrid HF-DFT SCF calculation will be
 performed using Pulay DIIS + Geometric Direct Minimization
 Exchange:     0.2220 Hartree-Fock + 1.0000 wB97X-D + LR-HF
```

```
 Correlation:  1.0000 wB97X-D
 Using Euler-Maclaurin-Lebedev (75,302) quadrature formula
 Dispersion:   Grimme D
 SCF converges when RMS gradient is below 1.0E-07
 Geometry optimization detected.  Setting ReadMinima to 0
 Setting SaveMinima to 0
 ---------------------------------------
  Cycle       Energy         DIIS Error
 ---------------------------------------
    1    -764.0607122988      8.28E-04
    2    -763.9574064045      1.69E-04
    3    -763.9578209764      1.54E-04
    4    -763.9584675835      2.45E-05
    5    -763.9584883744      1.17E-05
    6    -763.9584943473      4.43E-06
    7    -763.9584954492      1.75E-06
    8    -763.9584956371      5.40E-07
    9    -763.9584956640      3.01E-07
   10    -763.9584956682      8.57E-08 Convergence criterion met
 ---------------------------------------
 SCF time:  CPU 236.23 s  wall 552.67 s
 SCF   energy in the final basis set = -763.95849567
 Total energy in the final basis set = -763.95849567

--------------------------------------------------
-  Entering anlman on Sun Oct 13 12:28:52 2024  -
--------------------------------------------------



 --------------------------------------------------------------

                   Orbital Energies (a.u.)
 --------------------------------------------------------------


 Alpha MOs
 -- Occupied --
-14.8125 -14.8046 -14.7242 -14.7193 -10.6784 -10.6705 -10.6569 -10.6366
-10.6335 -10.6189 -10.6109 -10.5794 -10.5670 -10.5669 -10.5490 -10.5311
-10.5014 -10.4635  -1.4264  -1.3990  -1.3452  -1.2764  -1.2299  -1.2010
 -1.1833  -1.1449  -1.1024  -1.0802  -1.0620  -1.0528  -1.0269  -1.0078
 -0.9875  -0.9510  -0.9488  -0.9113  -0.9036  -0.8863  -0.8797  -0.8668
 -0.8457  -0.8413  -0.8388  -0.8179  -0.8112  -0.7921  -0.7865  -0.7833
 -0.7740  -0.7555  -0.7486  -0.7451  -0.7408  -0.7259  -0.7226  -0.7127
 -0.6991  -0.6700  -0.6562  -0.6516  -0.6371  -0.6284  -0.6263  -0.6197
 -0.6167
 -- Virtual --
 -0.2759  -0.2675  -0.2536  -0.2205  -0.1764  -0.1644  -0.1453  -0.1441
 -0.1343  -0.1254  -0.1207  -0.1139  -0.1088  -0.1050  -0.0984  -0.0882
 -0.0879  -0.0833  -0.0809  -0.0797  -0.0763  -0.0718  -0.0698  -0.0647
 -0.0609  -0.0589  -0.0547  -0.0503  -0.0494  -0.0442  -0.0423  -0.0351
 -0.0282  -0.0274  -0.0237  -0.0225  -0.0205  -0.0172  -0.0123  -0.0092
 -0.0056  -0.0036  -0.0018   0.0029   0.0045   0.0118   0.0152   0.0169
  0.0209   0.0230   0.0268   0.0303   0.0319   0.0334   0.0378   0.0404
  0.0420   0.0425   0.0503   0.0532   0.0560   0.0588   0.0612   0.0640
  0.0696   0.0721   0.0746   0.0760   0.0794   0.0802   0.0839   0.0873
  0.0913   0.0931   0.0990   0.1017   0.1060   0.1076   0.1095   0.1166
  0.1184   0.1230   0.1275   0.1297   0.1342   0.1386   0.1442   0.1455
  0.1491   0.1563   0.1639   0.1656   0.1708   0.1716   0.1746   0.1771
  0.1813   0.1840   0.1887   0.1945   0.2007   0.2035   0.2072   0.2147
  0.2287   0.2368   0.2495   0.2622   0.2815   0.2919   0.3146   0.3177
  0.3267   0.3287   0.3348   0.3426   0.3502   0.3525   0.3631   0.3646
  0.3716   0.3742   0.3856   0.3912   0.3943   0.4008   0.4068   0.4198
  0.4280   0.4382   0.4416   0.4479   0.4589   0.4644   0.4690   0.4715
  0.4785   0.4907   0.4963   0.4997   0.5082   0.5149   0.5159   0.5214
  0.5267   0.5278   0.5318   0.5358   0.5420   0.5443   0.5524   0.5561
  0.5622   0.5648   0.5709   0.5723   0.5819   0.5894   0.5984   0.6036
  0.6061   0.6234   0.6267   0.6290   0.6302   0.6399   0.6489   0.6597
  0.6657   0.6732   0.6823   0.6839   0.6885   0.6997   0.7020   0.7052
  0.7172   0.7245   0.7369   0.7431   0.7569   0.7625   0.7710   0.7802
  0.7909   0.7991   0.8080   0.8133   0.8291   0.8368   0.8477   0.8516
```

| | | | | | | | |
|---|---|---|---|---|---|---|---|
| 0.8633 | 0.8705 | 0.8767 | 0.8830 | 0.8954 | 0.9091 | 0.9172 | 0.9216 |
| 0.9353 | 0.9531 | 0.9668 | 0.9750 | 0.9899 | 0.9999 | 1.0121 | 1.0254 |
| 1.0604 | 1.0709 | 1.0893 | 1.0972 | 1.1220 | 1.1639 | 1.1809 | 1.1951 |
| 1.1983 | 1.2104 | 1.2268 | 1.2595 | 1.2675 | 1.2727 | 1.2974 | 1.3070 |
| 1.3113 | 1.3213 | 1.3379 | 1.3428 | 1.3452 | 1.3640 | 1.3745 | 1.3805 |
| 1.3890 | 1.3948 | 1.3989 | 1.4034 | 1.4074 | 1.4143 | 1.4276 | 1.4387 |
| 1.4402 | 1.4504 | 1.4564 | 1.4630 | 1.4650 | 1.4754 | 1.4771 | 1.4838 |
| 1.4970 | 1.5023 | 1.5105 | 1.5265 | 1.5321 | 1.5409 | 1.5497 | 1.5542 |
| 1.5644 | 1.5692 | 1.5721 | 1.5851 | 1.5972 | 1.5995 | 1.6027 | 1.6187 |
| 1.6272 | 1.6331 | 1.6427 | 1.6556 | 1.6584 | 1.6662 | 1.6857 | 1.6871 |
| 1.6945 | 1.7027 | 1.7053 | 1.7301 | 1.7369 | 1.7449 | 1.7654 | 1.7770 |
| 1.7804 | 1.7901 | 1.8061 | 1.8192 | 1.8420 | 1.8447 | 1.8601 | 1.8744 |
| 1.8860 | 1.8954 | 1.9020 | 1.9268 | 1.9372 | 1.9587 | 1.9695 | 1.9864 |
| 1.9978 | 2.0083 | 2.0168 | 2.0252 | 2.0523 | 2.0623 | 2.0817 | 2.0956 |
| 2.1036 | 2.1138 | 2.1284 | 2.1427 | 2.1577 | 2.1828 | 2.2193 | 2.2224 |
| 2.2320 | 2.2347 | 2.2498 | 2.2691 | 2.2783 | 2.2829 | 2.2955 | 2.3009 |
| 2.3098 | 2.3145 | 2.3481 | 2.3677 | 2.3699 | 2.3775 | 2.3866 | 2.3955 |
| 2.4048 | 2.4148 | 2.4394 | 2.4430 | 2.4525 | 2.4643 | 2.4753 | 2.4878 |
| 2.5054 | 2.5172 | 2.5259 | 2.5303 | 2.5379 | 2.5474 | 2.5511 | 2.5563 |
| 2.5643 | 2.5786 | 2.5830 | 2.5894 | 2.5917 | 2.5976 | 2.6047 | 2.6198 |
| 2.6219 | 2.6405 | 2.6494 | 2.6571 | 2.6773 | 2.6833 | 2.6941 | 2.7010 |
| 2.7077 | 2.7157 | 2.7251 | 2.7390 | 2.7460 | 2.7664 | 2.7704 | 2.7825 |
| 2.7848 | 2.7939 | 2.8084 | 2.8168 | 2.8413 | 2.8574 | 2.8720 | 2.8896 |
| 2.9097 | 2.9282 | 2.9495 | 2.9791 | 3.0272 | 3.0635 | 3.0929 | 3.1252 |
| 3.1452 | 3.1715 | 3.2517 | 3.2554 | 3.2983 | 3.3274 | 3.3530 | 3.3813 |
| 3.4187 | 3.4719 | 3.4943 | 3.5463 | 3.5644 | 3.5976 | 3.6336 | 3.6430 |
| 3.6641 | 3.6951 | 3.7052 | 3.7151 | 3.7331 | 3.7629 | 3.7651 | 3.7727 |
| 3.8261 | 3.8721 | 3.8962 | 3.9106 | 3.9338 | 4.0113 | 4.0316 | 4.1031 |
| 4.1094 | 4.2020 | 4.3037 | 4.3139 | 4.5713 | 4.6507 | 4.6887 | 4.7108 |
| 4.8801 | 5.0427 | 5.1714 | 23.5265 | 23.5577 | 23.7145 | 23.7238 | 23.7365 |
| 23.7769 | 23.7963 | 23.8309 | 23.8524 | 23.8905 | 23.9180 | 23.9693 | 23.9739 |
| 24.0420 | 35.4244 | 35.4434 | 35.4961 | 35.5880 | | | |

Ground-State Mulliken Net Atomic Charges

| Atom | | Charge (a.u.) |
|---|---|---|
| 1 | N | 0.399879 |
| 2 | C | 0.175744 |
| 3 | C | -0.050109 |
| 4 | C | -0.149966 |
| 5 | C | -0.166039 |
| 6 | C | -0.184229 |
| 7 | C | -0.817537 |
| 8 | C | -0.273815 |
| 9 | N | -0.129547 |
| 10 | C | -0.195654 |
| 11 | N | 0.041338 |
| 12 | C | 0.077533 |
| 13 | C | 0.038587 |
| 14 | C | 0.113237 |
| 15 | C | -0.403351 |
| 16 | C | -0.220012 |
| 17 | C | -0.512952 |
| 18 | N | -0.480514 |
| 19 | H | 0.219905 |
| 20 | H | 0.233374 |
| 21 | H | 0.226980 |
| 22 | H | 0.248529 |
| 23 | H | 0.230232 |
| 24 | H | 0.170929 |
| 25 | H | 0.252609 |
| 26 | H | 0.230607 |
| 27 | H | 0.245006 |
| 28 | H | 0.395955 |
| 29 | H | 0.206266 |
| 30 | H | 0.247354 |
| 31 | H | 0.227876 |
| 32 | H | 0.168423 |

```
    33 H                    0.225871
    34 H                    0.140061
    35 H                    0.208701
    36 H                    0.190138
    37 H                    0.325114
    38 H                    0.343476
 ----------------------------------------
 Sum of atomic charges =     2.000000

 -----------------------------------------------------------------
                   Cartesian Multipole Moments
 -----------------------------------------------------------------
    Charge (ESU x 10^10)
                 9.6064
    Dipole Moment (Debye)
         X       4.1472      Y      -0.7585      Z      -5.3545
       Tot       6.8150
    Quadrupole Moments (Debye-Ang)
        XX     -45.9041     XY       9.1863     YY     -71.5905
        XZ     -40.7997     YZ       0.5776     ZZ     -36.3459
    Traceless Quadrupole Moments (Debye-Ang)
       QXX      16.1283    QYY     -60.9311    QZZ      44.8029
       QXY      27.5589    QXZ    -122.3991    QYZ       1.7329
    Octopole Moments (Debye-Ang^2)
       XXX     -86.4468    XXY     -31.3341    XYY      18.9525
       YYY     -24.8945    XXZ      23.0563    XYZ       7.4725
       YYZ     -15.4806    XZZ      65.5047    YZZ      19.9600
       ZZZ     -97.7702
    Traceless Octopole Moments (Debye-Ang^2)
       XXX   -1278.7958    YYY     -47.0001    ZZZ    -654.8027
       XXY    -361.2053    XXZ     616.4285    XYY     290.2569
       XYZ     112.0880    XZZ     988.5389    YYZ      38.3742
       YZZ     408.2054
    Hexadecapole Moments (Debye-Ang^3)
      XXXX   -2565.4398   XXXY    -108.8747   XXYY    -553.5632
      XYYY     -63.3551   YYYY    -794.2483   XXXZ    1054.2427
      XXYZ      21.4818   XYYZ     354.4460   YYYZ      49.7545
      XXZZ    -675.6531   XYZZ      40.5340   YYZZ    -568.7098
      XZZZ     696.2359   YZZZ     -77.5555   ZZZZ   -2512.7390
    Traceless Hexadecapole Moments (Debye-Ang^3)
      XXXX  -13066.6438   XXXY   -5505.5289   XXXZ   15973.8742
      XXYY    -861.3139   XXYZ    2342.0410   XXZZ   13927.9577
      XYYY    -725.9767   XYYZ    5642.9609   XYZZ    6231.5056
      XZZZ  -21616.8351   YYYY    3876.3355   YYYZ    5483.5917
      YYZZ   -3015.0215   YZZZ   -7825.6327   ZZZZ  -10912.9362
 -----------------------------------------------------------------

--------------------------------------------------
-  Entering drvman on Sun Oct 13 12:28:52 2024  -
--------------------------------------------------

 Calculating analytic gradient of the SCF energy
 Gradient of SCF Energy
           1           2           3           4           5           6
    1  -0.0030581  -0.0000763   0.0003320  -0.0008605  -0.0001764   0.0014907
    2   0.0003144   0.0043377  -0.0054698   0.0017285  -0.0011105  -0.0016779
    3   0.0015684   0.0006261   0.0009782   0.0032504   0.0017395  -0.0020125
           7           8           9          10          11          12
    1   0.0014978  -0.0017084   0.0010798   0.0093385  -0.0048393  -0.0056306
    2  -0.0007376   0.0010257   0.0033835  -0.0102046   0.0060908  -0.0159865
    3  -0.0023703   0.0005625   0.0003430  -0.0064256   0.0076268  -0.0065722
          13          14          15          16          17          18
    1  -0.0019365  -0.0005044   0.0024260  -0.0012667  -0.0003676   0.0131260
    2   0.0021645   0.0035481   0.0011696   0.0012416   0.0001878   0.0064848
    3   0.0023080   0.0007096   0.0052015  -0.0049492   0.0002041  -0.0099829
          19          20          21          22          23          24
    1   0.0004079   0.0001904  -0.0000283   0.0009215  -0.0001132  -0.0008608
    2   0.0005712   0.0008137   0.0007698   0.0022891  -0.0013171   0.0005553
    3  -0.0023235  -0.0010859  -0.0013734  -0.0018161  -0.0003916  -0.0001921
          25          26          27          28          29          30
```

```
    1   0.0004412   0.0006653   0.0007103  -0.0024281  -0.0009643  -0.0021869
    2   0.0005549   0.0002272  -0.0006733  -0.0011637  -0.0006239   0.0004437
    3  -0.0001619   0.0007650   0.0020056  -0.0001510   0.0002552  -0.0000954
           31          32          33          34          35          36
    1  -0.0005389   0.0014371  -0.0005282  -0.0000740  -0.0003584  -0.0002405
    2   0.0002228   0.0000712  -0.0000364  -0.0003145  -0.0003975   0.0003585
    3  -0.0000830   0.0007872   0.0018345  -0.0004217   0.0003963  -0.0002405
           37          38
    1  -0.0039280  -0.0013904
    2   0.0009367   0.0002221
    3   0.0079184   0.0015685
 Max gradient component =       1.599E-02
 RMS gradient           =       3.421E-03
 Gradient time:  CPU 158.11 s  wall 305.28 s

-------------------------------------------------
-  Entering optman on Sun Oct 13 12:33:58 2024  -
-------------------------------------------------

 Geometry Optimization Parameters
   NAtoms,    NIC,     NZ,  NCons,   NDum,   NFix, NCnnct, MaxDiis
       38     272       0       0       0       0       0       0

 Cartesian Hessian Update
 Hessian updated using BFGS update


** GEOMETRY OPTIMIZATION IN DELOCALIZED INTERNAL COORDINATES **
   Searching for a Minimum

   Optimization Cycle:   2

                     Coordinates (Angstroms)
     ATOM                X               Y               Z
      1  N         2.0291132953    0.1845418137   -1.6531093845
      2  C         2.2551114754   -0.6792500200   -2.6859343209
      3  C         2.3532168326   -0.1672668655   -3.9720131331
      4  C         2.2389589272    1.1958124481   -4.2019352264
      5  C         2.0385889780    2.0558289721   -3.1284713684
      6  C         1.9473046771    1.5239287213   -1.8711235970
      7  C         2.4338991584   -2.1484185450   -2.4446104184
      8  C         1.9289579597   -0.2791398742   -0.2377010527
      9  N        -1.6631614245   -0.9885407745    0.5818667116
     10  C        -2.0378737838    0.1252158992    1.2512680273
     11  N        -1.1775477759    1.0886649009    1.4982944888
     12  C         0.0877257010    0.9462232190    1.0760980511
     13  C         0.5264497937   -0.1734941677    0.3209547629
     14  C        -0.4090477152   -1.1436438391    0.1105087573
     15  C        -3.4418620530    0.2191445366    1.7486028779
     16  C        -3.6762994985   -0.6355213380    3.0102563369
     17  C        -2.9111595920   -0.1342595390    4.2306577735
     18  N         0.9344499911    1.9980183691    1.3491029418
     19  H         2.5356147348   -0.8406966058   -4.8018183596
     20  H         2.3231673496    1.5890703783   -5.2105735208
     21  H         1.9681341270    3.1273321842   -3.2706021241
     22  H         1.8145251815    2.1560162101   -1.0017442293
     23  H         2.5284407681   -2.6724089398   -3.3950271196
     24  H         1.5830566671   -2.5860537419   -1.9170499379
     25  H         3.3440858520   -2.3420205752   -1.8688052694
     26  H         2.6308355508    0.3229801895    0.3390784947
     27  H         2.2881883625   -1.3036412145   -0.1679285762
     28  H        -2.3583684244   -1.7170618845    0.4358754969
     29  H        -0.2187635689   -2.0646414588   -0.4277183108
     30  H        -3.6647036172    1.2673426953    1.9551364920
     31  H        -4.1203096701   -0.1090114250    0.9523489380
     32  H        -3.4138975344   -1.6821501120    2.8137443687
     33  H        -4.7484729989   -0.6193142483    3.2208390625
     34  H        -1.8273354343   -0.2086424045    4.0954126738
     35  H        -3.1736550760   -0.7265569572    5.1091941061
     36  H        -3.1549629567    0.9096407102    4.4445316221
```

```
    37  H         1.7778085710    1.7790293541    1.8706573587
    38  H         0.4297871701    2.7329439288    1.8417366064
  Point Group: c1    Number of degrees of freedom:   108


  Energy is   -763.958495668

 Hessian updated using BFGS update
  internal optimization (0)

108 Hessian modes will be used to form the next step
  Hessian Eigenvalues:
     0.001687    0.002667    0.004079    0.005874    0.009772    0.012126
     0.015176    0.018919    0.019418    0.019943    0.021418    0.021977
     0.022700    0.022862    0.024295    0.024466    0.025930    0.026572
     0.027207    0.028216    0.030041    0.031221    0.035799    0.037566
     0.038744    0.041310    0.043223    0.043810    0.045264    0.045747
     0.052406    0.053700    0.055471    0.056275    0.063733    0.078535
     0.085376    0.091749    0.121549    0.121749    0.127012    0.129844
     0.131350    0.132812    0.135934    0.138815    0.143231    0.146532
     0.147198    0.147678    0.148990    0.150500    0.151756    0.152406
     0.153109    0.156162    0.181479    0.206122    0.209118    0.215820
     0.224433    0.226964    0.234924    0.240574    0.247083    0.258260
     0.268659    0.272742    0.284425    0.290493    0.300161    0.300359
     0.300547    0.301199    0.301671    0.302547    0.303988    0.304346
     0.305135    0.305220    0.306103    0.308311    0.311582    0.313538
     0.318347    0.329217    0.332116    0.333814    0.340023    0.343200
     0.347866    0.351488    0.359838    0.362378    0.381080    0.385556
     0.392374    0.400563    0.402894    0.412582    0.415405    0.416352
     0.431402    0.442911    0.459733    0.501504    0.574098    0.759126

 Minimum search - taking simple RFO step
 Searching for Lamda that Minimizes Along All modes
 Value Taken    Lamda =  -0.00774292
 Calculated Step too Large.  Step scaled by  0.571908 !!
 Step Taken.  Stepsize is  0.300000  0.007066

                           Maximum     Tolerance    Cnvgd?
        Gradient           0.010435     0.000800      NO
        Displacement       0.108077     0.001400      NO
        Energy change     -0.010528     0.000228      NO


 New Cartesian Coordinates Obtained by Inverse Iteration

 Displacement from previous Coordinates is:  0.370252
 ----------------------------------------------------------------
             Standard Nuclear Orientation (Angstroms)
    I     Atom           X                Y                Z
 ----------------------------------------------------------------
    1      N       2.0263367859     0.1798471533    -1.6511247586
    2      C       2.2573875280    -0.6834911537    -2.6805996425
    3      C       2.3558032018    -0.1631664858    -3.9643040230
    4      C       2.2341208143     1.1971434244    -4.1967609282
    5      C       2.0183210194     2.0534138882    -3.1205380654
    6      C       1.9245619973     1.5180479415    -1.8637793141
    7      C       2.4377170934    -2.1495725314    -2.4316999266
    8      C       1.9279214756    -0.2841249762    -0.2389803829
    9      N      -1.6628144184    -0.9837419810     0.5860826925
   10      C      -2.0457660285     0.1361777954     1.2500876032
   11      N      -1.1752010141     1.0888226728     1.4967716183
   12      C       0.0951630339     0.9539328876     1.0955418648
   13      C       0.5279331381    -0.1674024082     0.3265758461
   14      C      -0.4064833456    -1.1403382702     0.1162330778
   15      C      -3.4491846761     0.2261629497     1.7366692026
   16      C      -3.6815513372    -0.6321217411     3.0025371991
   17      C      -2.9008066576    -0.1456741903     4.2188944242
   18      N       0.9211265281     2.0016379144     1.3663298214
   19      H       2.5429284403    -0.8423533362    -4.7860912114
   20      H       2.3183654689     1.5895138068    -5.2035302552
```

```
  21      H        1.9351250258     3.1239129562    -3.2531841167
  22      H        1.7728726734     2.1346513255    -0.9872610273
  23      H        2.5415132607    -2.6671308996    -3.3830196053
  24      H        1.5884613423    -2.5957490546    -1.9097222341
  25      H        3.3443175066    -2.3442117949    -1.8509163757
  26      H        2.6342330410     0.3121861415     0.3351911333
  27      H        2.2775028497    -1.3106016876    -0.1786479175
  28      H       -2.3520701839    -1.7140852385     0.4394629788
  29      H       -0.2103534203    -2.0586086953    -0.4240655427
  30      H       -3.6588538655     1.2744520498     1.9489785137
  31      H       -4.1271869108    -0.0981481932     0.9395339758
  32      H       -3.4390657751    -1.6812335561     2.7946280297
  33      H       -4.7527157018    -0.6030839344     3.2105966409
  34      H       -1.8183792131    -0.2300646147     4.0782796864
  35      H       -3.1625883779    -0.7380295714     5.0964641960
  36      H       -3.1304031858     0.8993050000     4.4407777491
  37      H        1.8416578581     1.8188448324     1.7447185257
  38      H        0.4500540293     2.7248815751     1.8998705477
 ----------------------------------------------------------------
 Nuclear Repulsion Energy =        1306.70055790 hartrees
 There are       65 alpha and       65 beta electrons

--------------------------------------------------
-  Entering fldman on Sun Oct 13 12:33:58 2024  -
--------------------------------------------------

 Applying Cartesian multipole field
    Component          Value
    ---------          -----
     (2,0,0)        1.00000E-11
     (0,2,0)        2.00000E-11
     (0,0,2)       -3.00000E-12
 Nucleus-field energy     =     0.0000000290 hartrees

--------------------------------------------------
-  Entering gesman on Sun Oct 13 12:33:58 2024  -
--------------------------------------------------

 Requested basis set is 6-311+G(d,p)
 There are 188 shells and 516 basis functions
 A cutoff of  1.0D-12 yielded  12632 shell pairs
 There are    100686 function pairs (    107316 Cartesian)
 Smallest overlap matrix eigenvalue = 2.10E-06
 Linear dependence detected in AO basis
 Tighter screening thresholds may be required for diffuse basis sets
 Use S2THRESH > 12 and THRESH = 14 in case of SCF convergence issues
 Number of orthogonalized atomic orbitals = 510
 Maximum deviation from orthogonality = 2.308E-11
 Guess MOs from SCF MO coefficient file
 Reading MOs from coefficient file
 Reading MOs from coefficient file

--------------------------------------------------
-  Entering scfman on Sun Oct 13 12:33:58 2024  -
--------------------------------------------------

 Long-range K will be added via erf
 Coulomb attenuation parameter = 0.2 bohr**(-1)
 A restricted hybrid HF-DFT SCF calculation will be
 performed using Pulay DIIS + Geometric Direct Minimization
 Exchange:     0.2220 Hartree-Fock + 1.0000 wB97X-D + LR-HF
 Correlation:  1.0000 wB97X-D
 Using Euler-Maclaurin-Lebedev (75,302) quadrature formula
 Dispersion:   Grimme D
 SCF converges when RMS gradient is below 1.0E-07
 Geometry optimization detected.  Setting ReadMinima to 0
 Setting SaveMinima to 0
 ---------------------------------------
  Cycle       Energy         DIIS Error
 ---------------------------------------
```

```
    1    -763.9810439275      4.02E-04
    2    -763.9615862244      1.01E-04
    3    -763.9617519049      9.52E-05
    4    -763.9620148027      1.45E-05
    5    -763.9620246974      6.74E-06
    6    -763.9620266905      3.12E-06
    7    -763.9620271897      9.84E-07
    8    -763.9620272420      4.58E-07
    9    -763.9620272539      1.30E-07
   10    -763.9620272551      6.57E-08 Convergence criterion met
 ---------------------------------------
 SCF time:  CPU 294.97 s  wall 612.11 s
 SCF   energy in the final basis set = -763.96202726
 Total energy in the final basis set = -763.96202726

-------------------------------------------------
-  Entering anlman on Sun Oct 13 12:44:10 2024  -
-------------------------------------------------



 --------------------------------------------------------------

                   Orbital Energies (a.u.)
 --------------------------------------------------------------

 Alpha MOs
 -- Occupied --
-14.8125 -14.8000 -14.7294 -14.7154 -10.6785 -10.6689 -10.6544 -10.6372
-10.6332 -10.6201 -10.6091 -10.5804 -10.5685 -10.5676 -10.5494 -10.5300
-10.5012 -10.4632  -1.4226  -1.4005  -1.3468  -1.2770  -1.2297  -1.2004
 -1.1847  -1.1442  -1.1015  -1.0814  -1.0599  -1.0535  -1.0282  -1.0073
 -0.9880  -0.9513  -0.9473  -0.9142  -0.9053  -0.8888  -0.8806  -0.8670
 -0.8474  -0.8415  -0.8383  -0.8185  -0.8129  -0.7924  -0.7876  -0.7828
 -0.7744  -0.7553  -0.7495  -0.7459  -0.7446  -0.7260  -0.7234  -0.7148
 -0.6982  -0.6685  -0.6557  -0.6502  -0.6368  -0.6273  -0.6256  -0.6180
 -0.6166
 -- Virtual --
 -0.2722  -0.2671  -0.2521  -0.2217  -0.1753  -0.1646  -0.1449  -0.1434
 -0.1344  -0.1253  -0.1207  -0.1135  -0.1086  -0.1045  -0.0988  -0.0885
 -0.0877  -0.0829  -0.0804  -0.0791  -0.0763  -0.0720  -0.0702  -0.0648
 -0.0611  -0.0592  -0.0544  -0.0504  -0.0490  -0.0428  -0.0422  -0.0343
 -0.0293  -0.0268  -0.0237  -0.0229  -0.0195  -0.0171  -0.0120  -0.0090
 -0.0070  -0.0035  -0.0010   0.0040   0.0045   0.0121   0.0150   0.0168
  0.0212   0.0231   0.0268   0.0311   0.0318   0.0343   0.0380   0.0405
  0.0425   0.0426   0.0500   0.0535   0.0558   0.0579   0.0621   0.0656
  0.0691   0.0718   0.0745   0.0764   0.0790   0.0804   0.0844   0.0873
  0.0920   0.0935   0.0992   0.1016   0.1057   0.1075   0.1095   0.1175
  0.1183   0.1238   0.1282   0.1294   0.1354   0.1389   0.1449   0.1455
  0.1506   0.1572   0.1649   0.1656   0.1721   0.1736   0.1754   0.1784
  0.1810   0.1852   0.1887   0.1951   0.2009   0.2053   0.2069   0.2155
  0.2276   0.2362   0.2461   0.2629   0.2815   0.2912   0.3145   0.3187
  0.3269   0.3279   0.3364   0.3417   0.3492   0.3532   0.3623   0.3645
  0.3732   0.3743   0.3852   0.3918   0.3945   0.4011   0.4078   0.4220
  0.4287   0.4385   0.4414   0.4468   0.4601   0.4635   0.4710   0.4754
  0.4796   0.4905   0.4961   0.5037   0.5075   0.5139   0.5167   0.5225
  0.5269   0.5283   0.5330   0.5354   0.5433   0.5441   0.5487   0.5568
  0.5617   0.5654   0.5706   0.5717   0.5837   0.5901   0.5996   0.6036
  0.6065   0.6235   0.6258   0.6291   0.6303   0.6395   0.6477   0.6591
  0.6664   0.6735   0.6833   0.6846   0.6912   0.6967   0.7030   0.7054
  0.7166   0.7265   0.7358   0.7457   0.7568   0.7608   0.7717   0.7805
  0.7905   0.8026   0.8066   0.8138   0.8301   0.8394   0.8498   0.8550
  0.8644   0.8709   0.8797   0.8861   0.8959   0.9123   0.9171   0.9226
  0.9383   0.9514   0.9677   0.9724   0.9878   0.9994   1.0120   1.0311
  1.0605   1.0659   1.0872   1.0965   1.1240   1.1648   1.1812   1.1922
  1.2003   1.2078   1.2289   1.2578   1.2660   1.2688   1.2973   1.3049
  1.3121   1.3213   1.3370   1.3425   1.3462   1.3622   1.3730   1.3824
  1.3905   1.3934   1.3992   1.4032   1.4119   1.4148   1.4230   1.4365
  1.4396   1.4525   1.4574   1.4642   1.4688   1.4746   1.4785   1.4837
  1.4963   1.5048   1.5110   1.5267   1.5331   1.5404   1.5516   1.5561
  1.5629   1.5688   1.5746   1.5840   1.5962   1.5998   1.6079   1.6184
```

| | | | | | | | |
|---|---|---|---|---|---|---|---|
| 1.6274 | 1.6354 | 1.6430 | 1.6566 | 1.6591 | 1.6658 | 1.6872 | 1.6914 |
| 1.6976 | 1.7024 | 1.7126 | 1.7337 | 1.7384 | 1.7442 | 1.7654 | 1.7762 |
| 1.7794 | 1.7886 | 1.8082 | 1.8191 | 1.8406 | 1.8450 | 1.8622 | 1.8805 |
| 1.8859 | 1.8961 | 1.9077 | 1.9286 | 1.9390 | 1.9452 | 1.9719 | 1.9784 |
| 1.9976 | 2.0047 | 2.0151 | 2.0254 | 2.0522 | 2.0640 | 2.0813 | 2.0932 |
| 2.1070 | 2.1143 | 2.1308 | 2.1431 | 2.1595 | 2.1846 | 2.2184 | 2.2233 |
| 2.2304 | 2.2374 | 2.2512 | 2.2642 | 2.2804 | 2.2841 | 2.2941 | 2.3009 |
| 2.3050 | 2.3145 | 2.3485 | 2.3685 | 2.3714 | 2.3778 | 2.3868 | 2.3959 |
| 2.4090 | 2.4157 | 2.4383 | 2.4427 | 2.4517 | 2.4628 | 2.4710 | 2.4854 |
| 2.5031 | 2.5187 | 2.5277 | 2.5308 | 2.5377 | 2.5490 | 2.5531 | 2.5558 |
| 2.5639 | 2.5795 | 2.5817 | 2.5907 | 2.5931 | 2.5960 | 2.6079 | 2.6208 |
| 2.6242 | 2.6425 | 2.6531 | 2.6624 | 2.6774 | 2.6837 | 2.6946 | 2.7010 |
| 2.7044 | 2.7176 | 2.7314 | 2.7401 | 2.7459 | 2.7639 | 2.7738 | 2.7821 |
| 2.7891 | 2.7943 | 2.8094 | 2.8197 | 2.8448 | 2.8569 | 2.8757 | 2.8892 |
| 2.9127 | 2.9298 | 2.9521 | 2.9764 | 3.0329 | 3.0636 | 3.1072 | 3.1443 |
| 3.1483 | 3.1728 | 3.2508 | 3.2546 | 3.3005 | 3.3287 | 3.3580 | 3.3842 |
| 3.4200 | 3.4727 | 3.4980 | 3.5496 | 3.5661 | 3.5980 | 3.6389 | 3.6406 |
| 3.6678 | 3.6981 | 3.7098 | 3.7177 | 3.7336 | 3.7642 | 3.7681 | 3.7746 |
| 3.8316 | 3.8668 | 3.8908 | 3.9127 | 3.9299 | 3.9947 | 4.0298 | 4.0991 |
| 4.1071 | 4.2020 | 4.2999 | 4.3033 | 4.5846 | 4.6560 | 4.6987 | 4.7130 |
| 4.8912 | 5.0458 | 5.1851 | 23.5278 | 23.5648 | 23.7155 | 23.7245 | 23.7379 |
| 23.7738 | 23.7942 | 23.8301 | 23.8556 | 23.8902 | 23.9197 | 23.9656 | 23.9730 |
| 24.0398 | 35.4264 | 35.4460 | 35.5018 | 35.5965 | | | |

Ground-State Mulliken Net Atomic Charges

| Atom | Charge (a.u.) |
|---|---|
| 1 N | 0.401239 |
| 2 C | 0.176808 |
| 3 C | -0.047716 |
| 4 C | -0.154314 |
| 5 C | -0.165368 |
| 6 C | -0.182570 |
| 7 C | -0.823283 |
| 8 C | -0.278611 |
| 9 N | -0.133569 |
| 10 C | -0.203053 |
| 11 N | 0.030086 |
| 12 C | 0.127327 |
| 13 C | 0.025523 |
| 14 C | 0.116051 |
| 15 C | -0.404459 |
| 16 C | -0.217562 |
| 17 C | -0.514456 |
| 18 N | -0.484456 |
| 19 H | 0.218951 |
| 20 H | 0.233788 |
| 21 H | 0.226917 |
| 22 H | 0.242367 |
| 23 H | 0.229675 |
| 24 H | 0.174208 |
| 25 H | 0.253291 |
| 26 H | 0.225841 |
| 27 H | 0.244460 |
| 28 H | 0.393115 |
| 29 H | 0.204297 |
| 30 H | 0.246570 |
| 31 H | 0.229244 |
| 32 H | 0.168974 |
| 33 H | 0.225659 |
| 34 H | 0.140749 |
| 35 H | 0.208747 |
| 36 H | 0.190387 |
| 37 H | 0.322427 |
| 38 H | 0.352712 |

Sum of atomic charges = 2.000000

```
 -----------------------------------------------------------------
                    Cartesian Multipole Moments
 -----------------------------------------------------------------
    Charge (ESU x 10^10)
                 9.6064
    Dipole Moment (Debye)
         X       4.3912      Y      -0.5392      Z      -5.3849
       Tot       6.9692
    Quadrupole Moments (Debye-Ang)
        XX     -45.6171     XY       9.5145     YY     -71.1532
        XZ     -40.6154     YZ       0.8939     ZZ     -36.8139
    Traceless Quadrupole Moments (Debye-Ang)
       QXX      16.7328    QYY     -59.8754    QZZ      43.1426
       QXY      28.5435    QXZ    -121.8462    QYZ       2.6818
    Octopole Moments (Debye-Ang^2)
       XXX     -84.8863    XXY     -30.7080    XYY      20.7856
       YYY     -24.1135    XXZ      22.9256    XYZ       8.4793
       YYZ     -14.5797    XZZ      64.8526    YZZ      20.2629
       ZZZ     -98.7238
    Traceless Octopole Moments (Debye-Ang^2)
       XXX   -1280.0615    YYY     -50.6751    ZZZ    -667.4550
       XXY    -356.9445    XXZ     615.0174    XYY     309.5283
       XYZ     127.1893    XZZ     970.5332    YYZ      52.4377
       YZZ     407.6195
    Hexadecapole Moments (Debye-Ang^3)
      XXXX   -2557.6095   XXXY    -104.6741   XXYY    -551.1883
      XYYY     -58.8528   YYYY    -791.8023   XXXZ    1051.4772
      XXYZ      23.7290   XYYZ     355.4798   YYYZ      52.8906
      XXZZ    -677.2298   XYZZ      40.0251   YYZZ    -565.3068
      XZZZ     692.5606   YZZZ     -78.9855   ZZZZ   -2506.0900
    Traceless Hexadecapole Moments (Debye-Ang^3)
      XXXX  -12793.0710   XXXY   -5433.1985   XXXZ   15926.8161
      XXYY    -788.7542   XXYZ    2512.2550   XXZZ   13581.8253
      XYYY    -621.9630   XYYZ    5832.6122   XYZZ    6055.1616
      XZZZ  -21759.4283   YYYY    3620.9652   YYYZ    5615.6301
      YYZZ   -2832.2110   YZZZ   -8127.8851   ZZZZ  -10749.6143
 -----------------------------------------------------------------

--------------------------------------------------
-  Entering drvman on Sun Oct 13 12:44:10 2024  -
--------------------------------------------------

 Calculating analytic gradient of the SCF energy
 Gradient of SCF Energy
           1           2           3           4           5           6
    1  -0.0016759   0.0001583   0.0000711  -0.0002184  -0.0000124   0.0007164
    2  -0.0000483   0.0010234  -0.0017086   0.0009644  -0.0006380  -0.0000787
    3   0.0004704  -0.0004932   0.0005798  -0.0001533   0.0005053   0.0002650
           7           8           9          10          11          12
    1   0.0003895   0.0006506   0.0008955   0.0003129   0.0009880  -0.0029564
    2  -0.0001994   0.0004183   0.0024542  -0.0050530   0.0019705  -0.0076005
    3  -0.0008864  -0.0003664   0.0003263  -0.0026315   0.0022857   0.0021874
          13          14          15          16          17          18
    1  -0.0025165  -0.0004732   0.0011935  -0.0012311  -0.0002050   0.0081085
    2   0.0033061  -0.0011891   0.0004188   0.0004776   0.0000464   0.0046259
    3   0.0012231  -0.0013092   0.0019235  -0.0017858   0.0005228  -0.0096200
          19          20          21          22          23          24
    1   0.0001553  -0.0000154   0.0000174   0.0006743  -0.0001641  -0.0001528
    2   0.0003451   0.0001483  -0.0000478   0.0005910   0.0000523  -0.0000464
    3  -0.0004064   0.0000405  -0.0003071  -0.0007157   0.0001711   0.0000859
          25          26          27          28          29          30
    1   0.0000918  -0.0009750   0.0001892  -0.0007633  -0.0003770  -0.0004130
    2   0.0000827   0.0003876   0.0000637   0.0000300  -0.0003961  -0.0002589
    3   0.0000243   0.0000585   0.0008979   0.0000772   0.0000856  -0.0002942
          31          32          33          34          35          36
    1  -0.0004697   0.0007844   0.0000612   0.0000640  -0.0000438   0.0000027
    2   0.0001659  -0.0000152  -0.0002468  -0.0000585   0.0000486   0.0000462
    3  -0.0000852   0.0004128   0.0004801  -0.0000004   0.0000014  -0.0001415
          37          38
    1  -0.0028593  -0.0000021
```

```
    2   0.0010911  -0.0011731
    3   0.0063590   0.0002128
 Max gradient component =       9.620E-03
 RMS gradient           =       1.879E-03
 Gradient time:  CPU 139.31 s  wall 296.25 s

-------------------------------------------------
-  Entering optman on Sun Oct 13 12:49:07 2024  -
-------------------------------------------------

 Geometry Optimization Parameters
   NAtoms,    NIC,     NZ,  NCons,   NDum,   NFix, NCnnct, MaxDiis
       38     272       0       0       0       0       0       0

 Cartesian Hessian Update
 Hessian updated using BFGS update


** GEOMETRY OPTIMIZATION IN DELOCALIZED INTERNAL COORDINATES **
   Searching for a Minimum

   Optimization Cycle:   3

                      Coordinates (Angstroms)
     ATOM                X               Y               Z
      1  N         2.0263367859    0.1798471533   -1.6511247586
      2  C         2.2573875280   -0.6834911537   -2.6805996425
      3  C         2.3558032018   -0.1631664858   -3.9643040230
      4  C         2.2341208143    1.1971434244   -4.1967609282
      5  C         2.0183210194    2.0534138882   -3.1205380654
      6  C         1.9245619973    1.5180479415   -1.8637793141
      7  C         2.4377170934   -2.1495725314   -2.4316999266
      8  C         1.9279214756   -0.2841249762   -0.2389803829
      9  N        -1.6628144184   -0.9837419810    0.5860826925
     10  C        -2.0457660285    0.1361777954    1.2500876032
     11  N        -1.1752010141    1.0888226728    1.4967716183
     12  C         0.0951630339    0.9539328876    1.0955418648
     13  C         0.5279331381   -0.1674024082    0.3265758461
     14  C        -0.4064833456   -1.1403382702    0.1162330778
     15  C        -3.4491846761    0.2261629497    1.7366692026
     16  C        -3.6815513372   -0.6321217411    3.0025371991
     17  C        -2.9008066576   -0.1456741903    4.2188944242
     18  N         0.9211265281    2.0016379144    1.3663298214
     19  H         2.5429284403   -0.8423533362   -4.7860912114
     20  H         2.3183654689    1.5895138068   -5.2035302552
     21  H         1.9351250258    3.1239129562   -3.2531841167
     22  H         1.7728726734    2.1346513255   -0.9872610273
     23  H         2.5415132607   -2.6671308996   -3.3830196053
     24  H         1.5884613423   -2.5957490546   -1.9097222341
     25  H         3.3443175066   -2.3442117949   -1.8509163757
     26  H         2.6342330410    0.3121861415    0.3351911333
     27  H         2.2775028497   -1.3106016876   -0.1786479175
     28  H        -2.3520701839   -1.7140852385    0.4394629788
     29  H        -0.2103534203   -2.0586086953   -0.4240655427
     30  H        -3.6588538655    1.2744520498    1.9489785137
     31  H        -4.1271869108   -0.0981481932    0.9395339758
     32  H        -3.4390657751   -1.6812335561    2.7946280297
     33  H        -4.7527157018   -0.6030839344    3.2105966409
     34  H        -1.8183792131   -0.2300646147    4.0782796864
     35  H        -3.1625883779   -0.7380295714    5.0964641960
     36  H        -3.1304031858    0.8993050000    4.4407777491
     37  H         1.8416578581    1.8188448324    1.7447185257
     38  H         0.4500540293    2.7248815751    1.8998705477
   Point Group: c1    Number of degrees of freedom:   108


   Energy is   -763.962027255

 Hessian updated using BFGS update
  internal optimization (0)
```

```
108 Hessian modes will be used to form the next step
  Hessian Eigenvalues:
     0.001687    0.002667    0.004072    0.005816    0.008854    0.011443
     0.014803    0.018836    0.019416    0.019945    0.021358    0.021997
     0.022707    0.022944    0.024311    0.024460    0.025852    0.026507
     0.027094    0.028215    0.030020    0.031052    0.034164    0.037131
     0.039034    0.041586    0.043222    0.043808    0.045592    0.046127
     0.052124    0.053763    0.054724    0.055587    0.064100    0.078809
     0.085510    0.091887    0.121749    0.122298    0.127109    0.130618
     0.131808    0.132832    0.138540    0.139518    0.143130    0.146199
     0.146725    0.147674    0.148987    0.151714    0.152369    0.153095
     0.153423    0.157108    0.193582    0.206041    0.209730    0.215770
     0.224451    0.226939    0.238379    0.246348    0.249583    0.264673
     0.268979    0.273234    0.284612    0.291015    0.300344    0.300484
     0.300850    0.301410    0.301897    0.303086    0.303993    0.304714
     0.305179    0.305233    0.306089    0.309118    0.311627    0.315250
     0.318592    0.332058    0.332914    0.333938    0.340079    0.343110
     0.348493    0.351574    0.358999    0.362619    0.381901    0.385488
     0.390566    0.399284    0.405576    0.409398    0.416101    0.418518
     0.432642    0.443352    0.463247    0.501600    0.578750    0.758116

 Minimum search - taking simple RFO step
 Searching for Lamda that Minimizes Along All modes
 Value Taken    Lamda =  -0.00499302
  dLimit reduced  1.260000000000000E-002  0.300000000000000
  1.501764249443977E-002  3.710934001214064E-003  7.999999999999999E-004
           0
 Calculated Step part too Large.  Step scaled by  0.251704
 Step Taken.  Stepsize is  0.138258  0.015018

                            Maximum     Tolerance    Cnvgd?
        Gradient            0.003711      0.000800      NO
        Displacement        0.053269      0.001400      NO
        Energy change      -0.003532      0.000228      NO


 New Cartesian Coordinates Obtained by Inverse Iteration

 Displacement from previous Coordinates is:  0.173102
 ----------------------------------------------------------------
             Standard Nuclear Orientation (Angstroms)
    I     Atom           X                Y                Z
 ----------------------------------------------------------------
    1      N       2.0249879143     0.1776024808    -1.6517336744
    2      C       2.2618541685    -0.6851978689    -2.6796100437
    3      C       2.3606597979    -0.1633462908    -3.9626907120
    4      C       2.2324099832     1.1959054517    -4.1954205456
    5      C       2.0062986884     2.0514897334    -3.1201097600
    6      C       1.9117115070     1.5147540948    -1.8642048562
    7      C       2.4463439840    -2.1502220264    -2.4287382974
    8      C       1.9265874572    -0.2868944471    -0.2401108642
    9      N      -1.6631341985    -0.9799418938     0.5902064018
   10      C      -2.0472254767     0.1426713842     1.2521479270
   11      N      -1.1762413948     1.0917204423     1.4974985414
   12      C       0.0955928892     0.9577644777     1.0991920716
   13      C       0.5284933165    -0.1644328868     0.3270401012
   14      C      -0.4063688004    -1.1355872536     0.1200569548
   15      C      -3.4511216238     0.2333645914     1.7348275956
   16      C      -3.6852019301    -0.6282686868     2.9994997058
   17      C      -2.8974125966    -0.1522636641     4.2149859973
   18      N       0.9147774953     1.9995314762     1.3804464589
   19      H       2.5524470191    -0.8431900502    -4.7825740679
   20      H       2.3173806919     1.5880306540    -5.2021125363
   21      H       1.9149405832     3.1212828222    -3.2517634657
   22      H       1.7488470122     2.1270172835    -0.9865332583
   23      H       2.5569922824    -2.6672118935    -3.3796369160
   24      H       1.5962066113    -2.5995337868    -1.9110577892
   25      H       3.3507078859    -2.3421253382    -1.8437084413
   26      H       2.6388434027     0.3038419110     0.3327532137
```

```
  27      H       2.2706766732    -1.3152281455    -0.1841061449
  28      H      -2.3504589969    -1.7115202499     0.4441166421
  29      H      -0.2092089850    -2.0536697984    -0.4201943207
  30      H      -3.6572800263     1.2816680431     1.9506954425
  31      H      -4.1281769816    -0.0879936431     0.9361567304
  32      H      -3.4528127269    -1.6783081367     2.7852735596
  33      H      -4.7558891782    -0.5916968503     3.2083693138
  34      H      -1.8160109967    -0.2432714182     4.0710784709
  35      H      -3.1603476590    -0.7464395918     5.0909054839
  36      H      -3.1191146991     0.8931962133     4.4424098938
  37      H       1.8607933099     1.8297325488     1.6933765243
  38      H       0.4584535975     2.7167703125     1.9332686636
 ----------------------------------------------------------------
 Nuclear Repulsion Energy =        1306.98524712 hartrees
 There are       65 alpha and       65 beta electrons

------------------------------------------------
-  Entering fldman on Sun Oct 13 12:49:07 2024  -
------------------------------------------------

 Applying Cartesian multipole field
    Component          Value
    ---------          -----
     (2,0,0)        1.00000E-11
     (0,2,0)        2.00000E-11
     (0,0,2)       -3.00000E-12
 Nucleus-field energy     =     0.0000000290 hartrees

------------------------------------------------
-  Entering gesman on Sun Oct 13 12:49:07 2024  -
------------------------------------------------

 Requested basis set is 6-311+G(d,p)
 There are 188 shells and 516 basis functions
 A cutoff of  1.0D-12 yielded  12634 shell pairs
 There are    100714 function pairs (    107347 Cartesian)
 Smallest overlap matrix eigenvalue = 2.11E-06
 Linear dependence detected in AO basis
 Tighter screening thresholds may be required for diffuse basis sets
 Use S2THRESH > 12 and THRESH = 14 in case of SCF convergence issues
 Number of orthogonalized atomic orbitals = 510
 Maximum deviation from orthogonality = 6.521E-12
 Guess MOs from SCF MO coefficient file
 Reading MOs from coefficient file
 Reading MOs from coefficient file

------------------------------------------------
-  Entering scfman on Sun Oct 13 12:49:07 2024  -
------------------------------------------------

 Long-range K will be added via erf
 Coulomb attenuation parameter = 0.2 bohr**(-1)
 A restricted hybrid HF-DFT SCF calculation will be
 performed using Pulay DIIS + Geometric Direct Minimization
 Exchange:     0.2220 Hartree-Fock + 1.0000 wB97X-D + LR-HF
 Correlation:  1.0000 wB97X-D
 Using Euler-Maclaurin-Lebedev (75,302) quadrature formula
 Dispersion:   Grimme D
 SCF converges when RMS gradient is below 1.0E-07
 Geometry optimization detected.  Setting ReadMinima to 0
 Setting SaveMinima to 0
 ---------------------------------------
  Cycle       Energy         DIIS Error
 ---------------------------------------
    1    -763.9700133927      1.85E-04
    2    -763.9630970140      4.22E-05
    3    -763.9631319307      3.94E-05
    4    -763.9631775660      7.20E-06
    5    -763.9631800673      2.99E-06
    6    -763.9631804740      1.39E-06
```

```
    7    -763.9631805760      4.61E-07
    8    -763.9631805887      1.99E-07
    9    -763.9631805911      6.14E-08 Convergence criterion met
 ---------------------------------------
 SCF time:  CPU 322.28 s  wall 660.21 s
 SCF   energy in the final basis set = -763.96318059
 Total energy in the final basis set = -763.96318059

-------------------------------------------------
-  Entering anlman on Sun Oct 13 13:00:07 2024  -
-------------------------------------------------



 --------------------------------------------------------------

                    Orbital Energies (a.u.)
 --------------------------------------------------------------


 Alpha MOs
 -- Occupied --
-14.8127 -14.7980 -14.7315 -14.7139 -10.6786 -10.6677 -10.6533 -10.6375
-10.6334 -10.6208 -10.6083 -10.5808 -10.5692 -10.5679 -10.5496 -10.5292
-10.5008 -10.4630  -1.4215  -1.4011  -1.3476  -1.2774  -1.2299  -1.2004
 -1.1853  -1.1439  -1.1012  -1.0818  -1.0592  -1.0536  -1.0287  -1.0070
 -0.9883  -0.9518  -0.9468  -0.9154  -0.9060  -0.8894  -0.8812  -0.8671
 -0.8481  -0.8419  -0.8379  -0.8188  -0.8134  -0.7928  -0.7881  -0.7824
 -0.7745  -0.7553  -0.7501  -0.7467  -0.7452  -0.7262  -0.7235  -0.7152
 -0.6977  -0.6682  -0.6559  -0.6499  -0.6365  -0.6266  -0.6252  -0.6184
 -0.6158
 -- Virtual --
 -0.2711  -0.2665  -0.2509  -0.2221  -0.1750  -0.1648  -0.1449  -0.1432
 -0.1346  -0.1252  -0.1208  -0.1135  -0.1086  -0.1044  -0.0989  -0.0887
 -0.0875  -0.0829  -0.0802  -0.0789  -0.0763  -0.0720  -0.0704  -0.0648
 -0.0613  -0.0594  -0.0544  -0.0503  -0.0490  -0.0426  -0.0422  -0.0342
 -0.0300  -0.0266  -0.0237  -0.0230  -0.0192  -0.0173  -0.0122  -0.0089
 -0.0073  -0.0036  -0.0006   0.0043   0.0046   0.0122   0.0148   0.0167
  0.0212   0.0229   0.0267   0.0313   0.0318   0.0346   0.0380   0.0405
  0.0423   0.0428   0.0499   0.0536   0.0553   0.0580   0.0622   0.0667
  0.0688   0.0716   0.0745   0.0766   0.0790   0.0807   0.0847   0.0871
  0.0924   0.0936   0.0994   0.1020   0.1055   0.1074   0.1097   0.1175
  0.1186   0.1241   0.1281   0.1298   0.1359   0.1389   0.1449   0.1455
  0.1510   0.1575   0.1646   0.1659   0.1728   0.1741   0.1758   0.1792
  0.1815   0.1856   0.1886   0.1954   0.2006   0.2061   0.2080   0.2158
  0.2276   0.2356   0.2447   0.2633   0.2811   0.2905   0.3142   0.3188
  0.3268   0.3281   0.3370   0.3415   0.3484   0.3535   0.3621   0.3644
  0.3738   0.3747   0.3850   0.3922   0.3947   0.4013   0.4079   0.4226
  0.4290   0.4386   0.4407   0.4459   0.4596   0.4629   0.4705   0.4776
  0.4800   0.4908   0.4959   0.5045   0.5091   0.5136   0.5174   0.5228
  0.5272   0.5287   0.5329   0.5364   0.5438   0.5443   0.5483   0.5568
  0.5618   0.5656   0.5705   0.5717   0.5844   0.5904   0.5998   0.6038
  0.6070   0.6229   0.6260   0.6287   0.6309   0.6395   0.6474   0.6591
  0.6663   0.6740   0.6844   0.6849   0.6913   0.6966   0.7033   0.7055
  0.7160   0.7262   0.7347   0.7462   0.7570   0.7603   0.7723   0.7804
  0.7904   0.8035   0.8061   0.8135   0.8306   0.8406   0.8499   0.8565
  0.8649   0.8707   0.8798   0.8890   0.8973   0.9138   0.9177   0.9233
  0.9393   0.9510   0.9674   0.9728   0.9871   0.9992   1.0118   1.0329
  1.0599   1.0645   1.0854   1.0960   1.1246   1.1658   1.1818   1.1915
  1.2010   1.2066   1.2284   1.2571   1.2635   1.2679   1.2971   1.3040
  1.3124   1.3215   1.3362   1.3421   1.3459   1.3620   1.3724   1.3826
  1.3907   1.3933   1.3993   1.4031   1.4139   1.4159   1.4214   1.4362
  1.4397   1.4531   1.4572   1.4643   1.4699   1.4753   1.4790   1.4838
  1.4959   1.5052   1.5114   1.5266   1.5333   1.5404   1.5516   1.5573
  1.5622   1.5687   1.5748   1.5838   1.5948   1.6000   1.6097   1.6182
  1.6276   1.6360   1.6436   1.6567   1.6602   1.6650   1.6871   1.6908
  1.6978   1.7024   1.7179   1.7332   1.7396   1.7441   1.7655   1.7757
  1.7791   1.7881   1.8079   1.8186   1.8399   1.8452   1.8605   1.8817
  1.8869   1.8958   1.9107   1.9293   1.9405   1.9410   1.9730   1.9775
  1.9971   2.0043   2.0153   2.0263   2.0538   2.0647   2.0807   2.0919
  2.1079   2.1137   2.1315   2.1433   2.1607   2.1858   2.2186   2.2237
  2.2303   2.2377   2.2515   2.2623   2.2804   2.2848   2.2926   2.3005
```

```
  2.3031   2.3141   2.3482   2.3685   2.3711   2.3774   2.3871   2.3965
  2.4098   2.4168   2.4371   2.4427   2.4518   2.4638   2.4700   2.4830
  2.5027   2.5190   2.5275   2.5304   2.5375   2.5499   2.5539   2.5558
  2.5636   2.5787   2.5817   2.5904   2.5929   2.5962   2.6097   2.6212
  2.6255   2.6431   2.6547   2.6642   2.6785   2.6847   2.6952   2.6999
  2.7050   2.7192   2.7334   2.7414   2.7462   2.7631   2.7752   2.7820
  2.7912   2.7941   2.8103   2.8212   2.8466   2.8567   2.8759   2.8894
  2.9133   2.9299   2.9532   2.9765   3.0356   3.0636   3.1102   3.1442
  3.1616   3.1767   3.2512   3.2550   3.3007   3.3289   3.3605   3.3859
  3.4204   3.4737   3.5009   3.5509   3.5657   3.5974   3.6386   3.6411
  3.6676   3.6988   3.7119   3.7185   3.7343   3.7646   3.7682   3.7751
  3.8335   3.8600   3.8851   3.9120   3.9304   3.9888   4.0295   4.0978
  4.1072   4.2025   4.2990   4.3027   4.5876   4.6586   4.7030   4.7128
  4.8967   5.0463   5.1874  23.5275  23.5670  23.7177  23.7251  23.7379
 23.7725  23.7948  23.8294  23.8567  23.8898  23.9203  23.9687  23.9746
 24.0395  35.4267  35.4477  35.5049  35.6012
 --------------------------------------------------------------

          Ground-State Mulliken Net Atomic Charges

     Atom                 Charge (a.u.)
  ----------------------------------------
      1 N                    0.401111
      2 C                    0.178260
      3 C                   -0.046171
      4 C                   -0.155606
      5 C                   -0.165237
      6 C                   -0.182564
      7 C                   -0.824913
      8 C                   -0.281305
      9 N                   -0.134718
     10 C                   -0.207641
     11 N                    0.027109
     12 C                    0.143832
     13 C                    0.022032
     14 C                    0.116733
     15 C                   -0.404827
     16 C                   -0.217000
     17 C                   -0.514531
     18 N                   -0.484327
     19 H                    0.218887
     20 H                    0.233992
     21 H                    0.226978
     22 H                    0.238374
     23 H                    0.229749
     24 H                    0.175591
     25 H                    0.253223
     26 H                    0.225298
     27 H                    0.244232
     28 H                    0.392150
     29 H                    0.203428
     30 H                    0.246142
     31 H                    0.229624
     32 H                    0.168887
     33 H                    0.225505
     34 H                    0.140721
     35 H                    0.208680
     36 H                    0.190433
     37 H                    0.320849
     38 H                    0.357017
  ----------------------------------------
  Sum of atomic charges =     2.000000

 -----------------------------------------------------------------
                    Cartesian Multipole Moments
 -----------------------------------------------------------------
    Charge (ESU x 10^10)
                 9.6064
    Dipole Moment (Debye)
         X       4.5072      Y      -0.4478      Z      -5.4219
```

```
        Tot       7.0649
    Quadrupole Moments (Debye-Ang)
         XX     -45.5534     XY       9.5331     YY     -71.0198
         XZ     -40.6278     YZ       1.0130     ZZ     -36.9233
    Traceless Quadrupole Moments (Debye-Ang)
        QXX      16.8363    QYY     -59.5630    QZZ      42.7267
        QXY      28.5992    QXZ    -121.8833    QYZ       3.0389
    Octopole Moments (Debye-Ang^2)
        XXX     -83.7740    XXY     -30.6960    XYY      21.4400
        YYY     -23.9450    XXZ      22.4658    XYZ       8.9029
        YYZ     -14.3240    XZZ      64.9073    YZZ      20.4093
        ZZZ     -99.4747
    Traceless Octopole Moments (Debye-Ang^2)
        XXX   -1279.7692    YYY     -51.0904    ZZZ    -670.1243
        XXY    -357.7444    XXZ     610.9850    XYY     313.8799
        XYZ     133.5439    XZZ     965.8893    YYZ      59.1393
        YZZ     408.8348
    Hexadecapole Moments (Debye-Ang^3)
       XXXX   -2556.7179   XXXY    -102.9785   XXYY    -550.6519
       XYYY     -56.0278   YYYY    -790.4388   XXXZ    1050.8682
       XXYZ      25.1144   XYYZ     356.0867   YYYZ      53.4275
       XXZZ    -677.8927   XYZZ      39.4534   YYZZ    -563.8333
       XZZZ     691.3117   YZZZ     -79.2336   ZZZZ   -2505.9465
    Traceless Hexadecapole Moments (Debye-Ang^3)
       XXXX  -12722.4830   XXXY   -5432.8608   XXXZ   15919.1629
       XXYY    -779.2324   XXYZ    2643.8879   XXZZ   13501.7153
       XYYY    -503.0409   XYYZ    5915.1009   XYZZ    5935.9018
       XZZZ  -21834.2639   YYYY    3506.3584   YYYZ    5630.4978
       YYZZ   -2727.1260   YZZZ   -8274.3857   ZZZZ  -10774.5893
 -----------------------------------------------------------------

------------------------------------------------
-  Entering drvman on Sun Oct 13 13:00:07 2024  -
------------------------------------------------

 Calculating analytic gradient of the SCF energy
 Gradient of SCF Energy
           1           2           3           4           5           6
    1  -0.0011900   0.0000776   0.0000383  -0.0001166  -0.0000215   0.0006153
    2  -0.0000309   0.0005430  -0.0011117   0.0006339  -0.0004185   0.0002272
    3   0.0002351  -0.0006788   0.0005379  -0.0002102   0.0004133   0.0001213
           7           8           9          10          11          12
    1   0.0001632   0.0006126   0.0007004  -0.0000714   0.0009623  -0.0025406
    2  -0.0001027   0.0002678   0.0021676  -0.0029430   0.0004215  -0.0056090
    3  -0.0005245  -0.0003421   0.0003956  -0.0014970   0.0012597   0.0030714
          13          14          15          16          17          18
    1  -0.0024492   0.0004169   0.0009036  -0.0008635  -0.0001524   0.0059522
    2   0.0019020  -0.0005043   0.0002958   0.0002348   0.0000664   0.0036431
    3   0.0003208  -0.0010194   0.0009666  -0.0008618   0.0003971  -0.0089939
          19          20          21          22          23          24
    1   0.0000966  -0.0000366   0.0000260   0.0004923  -0.0001336  -0.0000664
    2   0.0001990   0.0000556  -0.0001252   0.0002203   0.0001222  -0.0000724
    3  -0.0000733   0.0000824  -0.0000908  -0.0002502   0.0001434   0.0001157
          25          26          27          28          29          30
    1   0.0000453  -0.0010172   0.0001886  -0.0004758  -0.0002117  -0.0001536
    2   0.0000344   0.0004650   0.0001274   0.0001346  -0.0002736  -0.0001915
    3   0.0000196  -0.0000746   0.0005993   0.0000729  -0.0000110  -0.0002317
          31          32          33          34          35          36
    1  -0.0003494   0.0005929   0.0000845   0.0000382  -0.0000207   0.0000333
    2   0.0001423   0.0000042  -0.0002437  -0.0000202   0.0000820   0.0000133
    3  -0.0000236   0.0002635   0.0001697   0.0000513  -0.0000323  -0.0000956
          37          38
    1  -0.0022697   0.0001001
    2   0.0008283  -0.0011850
    3   0.0056915   0.0000827
 Max gradient component =       8.994E-03
 RMS gradient           =       1.509E-03
 Gradient time:  CPU 209.58 s  wall 401.78 s

------------------------------------------------
```

```
-  Entering optman on Sun Oct 13 13:06:49 2024  -
-------------------------------------------------

 Geometry Optimization Parameters
   NAtoms,    NIC,     NZ,  NCons,   NDum,   NFix, NCnnct, MaxDiis
       38     272       0       0       0       0       0       0

 Cartesian Hessian Update
 Hessian updated using BFGS update


** GEOMETRY OPTIMIZATION IN DELOCALIZED INTERNAL COORDINATES **
   Searching for a Minimum

   Optimization Cycle:   4

                     Coordinates (Angstroms)
     ATOM                X               Y               Z
      1  N         2.0249879143    0.1776024808   -1.6517336744
      2  C         2.2618541685   -0.6851978689   -2.6796100437
      3  C         2.3606597979   -0.1633462908   -3.9626907120
      4  C         2.2324099832    1.1959054517   -4.1954205456
      5  C         2.0062986884    2.0514897334   -3.1201097600
      6  C         1.9117115070    1.5147540948   -1.8642048562
      7  C         2.4463439840   -2.1502220264   -2.4287382974
      8  C         1.9265874572   -0.2868944471   -0.2401108642
      9  N        -1.6631341985   -0.9799418938    0.5902064018
     10  C        -2.0472254767    0.1426713842    1.2521479270
     11  N        -1.1762413948    1.0917204423    1.4974985414
     12  C         0.0955928892    0.9577644777    1.0991920716
     13  C         0.5284933165   -0.1644328868    0.3270401012
     14  C        -0.4063688004   -1.1355872536    0.1200569548
     15  C        -3.4511216238    0.2333645914    1.7348275956
     16  C        -3.6852019301   -0.6282686868    2.9994997058
     17  C        -2.8974125966   -0.1522636641    4.2149859973
     18  N         0.9147774953    1.9995314762    1.3804464589
     19  H         2.5524470191   -0.8431900502   -4.7825740679
     20  H         2.3173806919    1.5880306540   -5.2021125363
     21  H         1.9149405832    3.1212828222   -3.2517634657
     22  H         1.7488470122    2.1270172835   -0.9865332583
     23  H         2.5569922824   -2.6672118935   -3.3796369160
     24  H         1.5962066113   -2.5995337868   -1.9110577892
     25  H         3.3507078859   -2.3421253382   -1.8437084413
     26  H         2.6388434027    0.3038419110    0.3327532137
     27  H         2.2706766732   -1.3152281455   -0.1841061449
     28  H        -2.3504589969   -1.7115202499    0.4441166421
     29  H        -0.2092089850   -2.0536697984   -0.4201943207
     30  H        -3.6572800263    1.2816680431    1.9506954425
     31  H        -4.1281769816   -0.0879936431    0.9361567304
     32  H        -3.4528127269   -1.6783081367    2.7852735596
     33  H        -4.7558891782   -0.5916968503    3.2083693138
     34  H        -1.8160109967   -0.2432714182    4.0710784709
     35  H        -3.1603476590   -0.7464395918    5.0909054839
     36  H        -3.1191146991    0.8931962133    4.4424098938
     37  H         1.8607933099    1.8297325488    1.6933765243
     38  H         0.4584535975    2.7167703125    1.9332686636
   Point Group: c1    Number of degrees of freedom:   108


   Energy is   -763.963180591

 Hessian updated using BFGS update
  internal optimization (0)

108 Hessian modes will be used to form the next step
  Hessian Eigenvalues:
     0.001686    0.002667    0.004070    0.005781    0.007665    0.011497
     0.014716    0.018840    0.019430    0.019942    0.021410    0.022039
     0.022719    0.022958    0.024348    0.024490    0.025850    0.026612
     0.027116    0.028213    0.029922    0.030556    0.033090    0.036968
```

```
    0.039056    0.041595    0.043228    0.043820    0.045642    0.046221
    0.051867    0.053725    0.054472    0.055567    0.064097    0.079332
    0.085536    0.091924    0.121756    0.122323    0.127118    0.130613
    0.131910    0.132870    0.138638    0.141086    0.142996    0.145247
    0.146854    0.147678    0.149733    0.151847    0.152361    0.153140
    0.153689    0.157918    0.193644    0.206269    0.209730    0.215835
    0.225868    0.227076    0.239255    0.246343    0.249488    0.267136
    0.269302    0.273799    0.285842    0.291113    0.300350    0.300527
    0.300828    0.301459    0.301896    0.303133    0.303994    0.304717
    0.305181    0.305234    0.306066    0.309113    0.311822    0.316052
    0.318571    0.332116    0.333373    0.334114    0.340274    0.343168
    0.348757    0.352042    0.361874    0.362704    0.381856    0.385193
    0.395677    0.403786    0.407291    0.414808    0.416347    0.419675
    0.432810    0.446169    0.463856    0.511972    0.579593    0.757199

Minimum search - taking simple RFO step
Searching for Lamda that Minimizes Along All modes
Value Taken    Lamda =  -0.00428124
Calculated Step too Large.  Step scaled by  0.529609
Step Taken.  Stepsize is  0.300000

                           Maximum     Tolerance    Cnvgd?
        Gradient           0.002583      0.000800      NO
        Displacement       0.111669      0.001400      NO
        Energy change     -0.001153      0.000228      NO


New Cartesian Coordinates Obtained by Inverse Iteration

Displacement from previous Coordinates is:  0.426079
----------------------------------------------------------------
            Standard Nuclear Orientation (Angstroms)
   I     Atom           X                Y                Z
----------------------------------------------------------------
   1      N       2.0196994875     0.1717867896    -1.6553569787
   2      C       2.2765446983    -0.6893664834    -2.6788662859
   3      C       2.3758567795    -0.1666979850    -3.9620844431
   4      C       2.2267559021     1.1891157138    -4.1979514104
   5      C       1.9734099521     2.0430333105    -3.1264618265
   6      C       1.8778928671     1.5053838967    -1.8712714343
   7      C       2.4787841414    -2.1509127507    -2.4226582506
   8      C       1.9209472156    -0.2931514317    -0.2434537355
   9      N      -1.6689840304    -0.9669707884     0.5966505936
  10      C      -2.0509560046     0.1602178188     1.2585964457
  11      N      -1.1768525561     1.1022594884     1.5055821294
  12      C       0.0986999013     0.9684315477     1.1107280017
  13      C       0.5273202392    -0.1546507309     0.3287387683
  14      C      -0.4125101474    -1.1217988487     0.1251186585
  15      C      -3.4558844861     0.2543976078     1.7366251510
  16      C      -3.6971609479    -0.6184155867     2.9933732883
  17      C      -2.8926338165    -0.1721248787     4.2086351621
  18      N       0.9116405595     1.9916581061     1.4216933842
  19      H       2.5832726612    -0.8461398143    -4.7783142705
  20      H       2.3135004277     1.5803375417    -5.2047346503
  21      H       1.8605342841     3.1104799376    -3.2600355433
  22      H       1.6892292391     2.1132601231    -0.9956812263
  23      H       2.6094763272    -2.6671569744    -3.3714710141
  24      H       1.6275074953    -2.6110710931    -1.9164374342
  25      H       3.3776003616    -2.3306740080    -1.8255607905
  26      H       2.6494140583     0.2815749182     0.3264508650
  27      H       2.2489259912    -1.3268395660    -0.1954637647
  28      H      -2.3547388215    -1.6991207606     0.4506599103
  29      H      -0.2148613213    -2.0392800007    -0.4159150704
  30      H      -3.6535006908     1.3020766835     1.9631890287
  31      H      -4.1321901866    -0.0555409542     0.9333345884
  32      H      -3.4880633496    -1.6698657756     2.7631670272
  33      H      -4.7660251163    -0.5639578643     3.2075063834
  34      H      -1.8139864923    -0.2797127815     4.0558424523
  35      H      -3.1591769791    -0.7729696847     5.0788555232
  36      H      -3.0946298860     0.8738444323     4.4515758528
```

```
  37      H       1.8957539628     1.8507166817     1.5929335566
  38      H       0.4893882806     2.6978441641     2.0124613584
 ----------------------------------------------------------------
 Nuclear Repulsion Energy =        1306.77385220 hartrees
 There are       65 alpha and       65 beta electrons

--------------------------------------------------
-  Entering fldman on Sun Oct 13 13:06:49 2024  -
--------------------------------------------------

 Applying Cartesian multipole field
    Component          Value
    ---------          -----
     (2,0,0)        1.00000E-11
     (0,2,0)        2.00000E-11
     (0,0,2)       -3.00000E-12
 Nucleus-field energy     =     0.0000000289 hartrees

--------------------------------------------------
-  Entering gesman on Sun Oct 13 13:06:49 2024  -
--------------------------------------------------

 Requested basis set is 6-311+G(d,p)
 There are 188 shells and 516 basis functions
 A cutoff of  1.0D-12 yielded  12631 shell pairs
 There are    100703 function pairs (    107338 Cartesian)
 Smallest overlap matrix eigenvalue = 2.13E-06
 Linear dependence detected in AO basis
 Tighter screening thresholds may be required for diffuse basis sets
 Use S2THRESH > 12 and THRESH = 14 in case of SCF convergence issues
 Number of orthogonalized atomic orbitals = 510
 Maximum deviation from orthogonality = 1.306E-11
 Guess MOs from SCF MO coefficient file
 Reading MOs from coefficient file
 Reading MOs from coefficient file

--------------------------------------------------
-  Entering scfman on Sun Oct 13 13:06:50 2024  -
--------------------------------------------------

 Long-range K will be added via erf
 Coulomb attenuation parameter = 0.2 bohr**(-1)
 A restricted hybrid HF-DFT SCF calculation will be
 performed using Pulay DIIS + Geometric Direct Minimization
 Exchange:     0.2220 Hartree-Fock + 1.0000 wB97X-D + LR-HF
 Correlation:  1.0000 wB97X-D
 Using Euler-Maclaurin-Lebedev (75,302) quadrature formula
 Dispersion:   Grimme D
 SCF converges when RMS gradient is below 1.0E-07
 Geometry optimization detected.  Setting ReadMinima to 0
 Setting SaveMinima to 0
 ---------------------------------------
  Cycle       Energy         DIIS Error
 ---------------------------------------
    1    -763.9624669506      4.26E-04
    2    -763.9646894153      7.79E-05
    3    -763.9649028594      6.19E-05
    4    -763.9650186730      1.77E-05
    5    -763.9650318783      5.17E-06
    6    -763.9650332421      2.62E-06
    7    -763.9650335622      8.23E-07
    8    -763.9650336062      3.58E-07
    9    -763.9650336137      1.07E-07
   10    -763.9650336147      4.97E-08 Convergence criterion met
 ---------------------------------------
 SCF time:  CPU 391.88 s  wall 766.56 s
 SCF   energy in the final basis set = -763.96503361
 Total energy in the final basis set = -763.96503361

--------------------------------------------------
```

```
-  Entering anlman on Sun Oct 13 13:19:36 2024  -
------------------------------------------------



 --------------------------------------------------------------

                    Orbital Energies (a.u.)
 --------------------------------------------------------------

 Alpha MOs
 -- Occupied --
-14.8133 -14.7947 -14.7356 -14.7114 -10.6789 -10.6657 -10.6516 -10.6382
-10.6341 -10.6223 -10.6073 -10.5816 -10.5704 -10.5686 -10.5497 -10.5278
-10.4998 -10.4625  -1.4189  -1.4020  -1.3488  -1.2775  -1.2299  -1.1999
 -1.1863  -1.1431  -1.1005  -1.0826  -1.0581  -1.0535  -1.0294  -1.0061
 -0.9888  -0.9526  -0.9458  -0.9175  -0.9072  -0.8900  -0.8822  -0.8670
 -0.8494  -0.8427  -0.8365  -0.8191  -0.8139  -0.7935  -0.7887  -0.7817
 -0.7746  -0.7559  -0.7506  -0.7481  -0.7456  -0.7267  -0.7232  -0.7157
 -0.6964  -0.6671  -0.6564  -0.6490  -0.6355  -0.6254  -0.6242  -0.6192
 -0.6137
 -- Virtual --
 -0.2703  -0.2643  -0.2488  -0.2230  -0.1746  -0.1650  -0.1451  -0.1429
 -0.1350  -0.1250  -0.1209  -0.1134  -0.1085  -0.1044  -0.0992  -0.0891
 -0.0870  -0.0829  -0.0799  -0.0783  -0.0765  -0.0722  -0.0709  -0.0650
 -0.0615  -0.0597  -0.0544  -0.0503  -0.0493  -0.0429  -0.0415  -0.0346
 -0.0311  -0.0264  -0.0240  -0.0231  -0.0189  -0.0178  -0.0130  -0.0088
 -0.0073  -0.0038   0.0002   0.0044   0.0052   0.0124   0.0143   0.0164
  0.0210   0.0224   0.0262   0.0310   0.0322   0.0353   0.0380   0.0401
  0.0417   0.0433   0.0495   0.0537   0.0547   0.0587   0.0621   0.0679
  0.0696   0.0714   0.0747   0.0772   0.0789   0.0817   0.0853   0.0867
  0.0929   0.0937   0.0996   0.1025   0.1048   0.1073   0.1103   0.1170
  0.1193   0.1246   0.1276   0.1305   0.1370   0.1389   0.1446   0.1455
  0.1515   0.1580   0.1637   0.1661   0.1738   0.1746   0.1765   0.1799
  0.1832   0.1861   0.1879   0.1961   0.1987   0.2059   0.2122   0.2160
  0.2282   0.2314   0.2434   0.2639   0.2796   0.2889   0.3132   0.3189
  0.3258   0.3288   0.3376   0.3411   0.3466   0.3543   0.3613   0.3640
  0.3749   0.3762   0.3848   0.3929   0.3955   0.4019   0.4080   0.4229
  0.4301   0.4376   0.4393   0.4445   0.4576   0.4610   0.4693   0.4783
  0.4817   0.4910   0.4955   0.5036   0.5124   0.5127   0.5196   0.5227
  0.5281   0.5294   0.5325   0.5399   0.5456   0.5470   0.5496   0.5566
  0.5622   0.5661   0.5704   0.5726   0.5856   0.5907   0.5992   0.6047
  0.6087   0.6208   0.6267   0.6279   0.6316   0.6394   0.6480   0.6588
  0.6652   0.6748   0.6850   0.6881   0.6904   0.6967   0.7024   0.7057
  0.7138   0.7245   0.7324   0.7471   0.7565   0.7604   0.7736   0.7794
  0.7899   0.8042   0.8061   0.8134   0.8324   0.8429   0.8494   0.8596
  0.8651   0.8708   0.8796   0.8968   0.8997   0.9174   0.9191   0.9251
  0.9412   0.9505   0.9658   0.9737   0.9857   0.9984   1.0114   1.0369
  1.0565   1.0629   1.0816   1.0940   1.1255   1.1683   1.1829   1.1904
  1.2025   1.2044   1.2260   1.2547   1.2571   1.2670   1.2962   1.3024
  1.3123   1.3220   1.3344   1.3405   1.3445   1.3614   1.3714   1.3825
  1.3908   1.3928   1.4000   1.4023   1.4138   1.4169   1.4238   1.4361
  1.4398   1.4534   1.4539   1.4634   1.4710   1.4763   1.4797   1.4842
  1.4941   1.5055   1.5122   1.5256   1.5334   1.5403   1.5513   1.5569
  1.5637   1.5689   1.5750   1.5835   1.5891   1.6008   1.6118   1.6184
  1.6280   1.6362   1.6449   1.6556   1.6618   1.6634   1.6863   1.6880
  1.6962   1.7023   1.7267   1.7290   1.7433   1.7439   1.7666   1.7730
  1.7787   1.7872   1.8064   1.8161   1.8372   1.8457   1.8565   1.8807
  1.8897   1.8953   1.9153   1.9302   1.9369   1.9436   1.9721   1.9824
  1.9944   2.0066   2.0157   2.0285   2.0567   2.0648   2.0806   2.0899
  2.1084   2.1126   2.1324   2.1433   2.1631   2.1883   2.2192   2.2242
  2.2299   2.2372   2.2519   2.2592   2.2789   2.2854   2.2893   2.2971
  2.3012   2.3128   2.3464   2.3683   2.3694   2.3766   2.3873   2.3970
  2.4101   2.4197   2.4341   2.4418   2.4521   2.4657   2.4677   2.4776
  2.5020   2.5190   2.5260   2.5303   2.5371   2.5509   2.5530   2.5569
  2.5640   2.5755   2.5805   2.5888   2.5922   2.5974   2.6121   2.6221
  2.6271   2.6426   2.6561   2.6674   2.6795   2.6832   2.6932   2.7002
  2.7069   2.7240   2.7365   2.7434   2.7490   2.7614   2.7774   2.7819
  2.7927   2.7942   2.8124   2.8246   2.8496   2.8556   2.8752   2.8895
  2.9119   2.9275   2.9560   2.9769   3.0401   3.0638   3.1118   3.1428
  3.1689   3.2030   3.2519   3.2564   3.3003   3.3294   3.3626   3.3913
```

```
  3.4203   3.4736   3.5052   3.5533   3.5644   3.5956   3.6380   3.6410
  3.6665   3.7000   3.7156   3.7197   3.7357   3.7652   3.7680   3.7757
  3.8312   3.8367   3.8776   3.9105   3.9313   3.9814   4.0293   4.0940
  4.1077   4.2037   4.2968   4.3018   4.5914   4.6622   4.7086   4.7117
  4.9067   5.0463   5.1897  23.5267  23.5706  23.7184  23.7256  23.7374
 23.7692  23.7955  23.8277  23.8581  23.8886  23.9213  23.9684  23.9748
 24.0388  35.4265  35.4508  35.5099  35.6075
--------------------------------------------------------------

          Ground-State Mulliken Net Atomic Charges

     Atom                 Charge (a.u.)
  ----------------------------------------
      1 N                     0.403810
      2 C                     0.177433
      3 C                    -0.041261
      4 C                    -0.156460
      5 C                    -0.166845
      6 C                    -0.183020
      7 C                    -0.824264
      8 C                    -0.289337
      9 N                    -0.132628
     10 C                    -0.220747
     11 N                     0.021315
     12 C                     0.176970
     13 C                     0.014209
     14 C                     0.115375
     15 C                    -0.404300
     16 C                    -0.216760
     17 C                    -0.514264
     18 N                    -0.483187
     19 H                     0.219000
     20 H                     0.234307
     21 H                     0.227113
     22 H                     0.228038
     23 H                     0.229862
     24 H                     0.178833
     25 H                     0.252520
     26 H                     0.226101
     27 H                     0.243786
     28 H                     0.390077
     29 H                     0.202140
     30 H                     0.245287
     31 H                     0.230132
     32 H                     0.168508
     33 H                     0.225301
     34 H                     0.140510
     35 H                     0.208450
     36 H                     0.190437
     37 H                     0.317766
     38 H                     0.365791
  ----------------------------------------
  Sum of atomic charges =     2.000000

-----------------------------------------------------------------
                    Cartesian Multipole Moments
-----------------------------------------------------------------
    Charge (ESU x 10^10)
                 9.6064
    Dipole Moment (Debye)
         X       4.7135      Y      -0.2684      Z      -5.5222
       Tot       7.2653
    Quadrupole Moments (Debye-Ang)
        XX     -45.3661     XY       9.4550     YY     -70.8352
        XZ     -40.7441     YZ       1.2484     ZZ     -37.0125
    Traceless Quadrupole Moments (Debye-Ang)
       QXX      17.1156    QYY     -59.2920    QZZ      42.1764
       QXY      28.3650    QXZ    -122.2323    QYZ       3.7452
    Octopole Moments (Debye-Ang^2)
       XXX     -81.4650    XXY     -31.1720    XYY      22.6875
```

```
       YYY     -23.7575    XXZ      21.2307    XYZ       9.9939
       YYZ     -13.8122    XZZ      65.4092    YZZ      20.8607
       ZZZ    -101.4522
    Traceless Octopole Moments (Debye-Ang^2)
       XXX   -1281.6597    YYY     -49.7436    ZZZ    -675.4805
       XXY    -365.3733    XXZ     600.5618    XYY     320.4170
       XYZ     149.9080    XZZ     961.2427    YYZ      74.9187
       YZZ     415.1169
    Hexadecapole Moments (Debye-Ang^3)
      XXXX   -2557.7295   XXXY     -99.6349   XXYY    -550.4737
      XYYY     -49.0460   YYYY    -786.9387   XXXZ    1049.0834
      XXYZ      28.7744   XYYZ     358.4249   YYYZ      53.6306
      XXZZ    -679.4665   XYZZ      38.3589   YYZZ    -561.6566
      XZZZ     688.6163   YZZZ     -80.7546   ZZZZ   -2510.0379
    Traceless Hexadecapole Moments (Debye-Ang^3)
      XXXX  -12612.4177   XXXY   -5497.1723   XXXZ   15828.1461
      XXYY    -812.3611   XXYZ    2985.2341   XXZZ   13424.7789
      XYYY    -185.3454   XYYZ    6192.7467   XYZZ    5682.5177
      XZZZ  -22020.8928   YYYY    3346.5508   YYYZ    5522.9893
      YYZZ   -2534.1896   YZZZ   -8508.2234   ZZZZ  -10890.5892
 -----------------------------------------------------------------

--------------------------------------------------
-  Entering drvman on Sun Oct 13 13:19:36 2024  -
--------------------------------------------------

 Calculating analytic gradient of the SCF energy
 Gradient of SCF Energy
           1           2           3           4           5           6
    1  -0.0004262  -0.0001499   0.0000036   0.0000177  -0.0000622   0.0005008
    2   0.0000749  -0.0000396  -0.0002506   0.0001103  -0.0001056   0.0004076
    3  -0.0001218  -0.0006867   0.0003420  -0.0002118   0.0002813  -0.0000346
           7           8           9          10          11          12
    1  -0.0000604   0.0005814   0.0002350  -0.0012888   0.0010431  -0.0007791
    2   0.0000575  -0.0000884   0.0009447  -0.0006979  -0.0003975  -0.0016910
    3  -0.0000332  -0.0002087   0.0002346  -0.0005315   0.0002873   0.0039509
          13          14          15          16          17          18
    1  -0.0010039   0.0001821   0.0003592  -0.0004791  -0.0000575   0.0023502
    2   0.0006384  -0.0009568   0.0001288   0.0000266   0.0000637   0.0024006
    3  -0.0004636  -0.0008627   0.0000700   0.0001123   0.0001949  -0.0068866
          19          20          21          22          23          24
    1   0.0000141  -0.0000330   0.0000372   0.0002024  -0.0001083   0.0000334
    2   0.0000452  -0.0000429  -0.0001257  -0.0000371   0.0001684  -0.0000836
    3   0.0001890   0.0001256   0.0000794   0.0002722   0.0001295   0.0001138
          25          26          27          28          29          30
    1   0.0000125  -0.0008018   0.0002218  -0.0000326  -0.0000298   0.0001635
    2  -0.0000511   0.0003753   0.0001249   0.0001462  -0.0000634  -0.0001448
    3   0.0000113  -0.0002692   0.0001581   0.0000282  -0.0000528  -0.0001575
          31          32          33          34          35          36
    1  -0.0001947   0.0003447   0.0000698   0.0000290   0.0000043   0.0000362
    2   0.0000469   0.0000200  -0.0002347   0.0000176   0.0001007  -0.0000048
    3   0.0000683   0.0000859  -0.0001188   0.0000830  -0.0000521  -0.0000486
          37          38
    1  -0.0011919   0.0002571
    2   0.0002295  -0.0011123
    3   0.0038635   0.0000590
 Max gradient component =       6.887E-03
 RMS gradient           =       9.709E-04
 Gradient time:  CPU 195.08 s  wall 394.24 s

--------------------------------------------------
-  Entering optman on Sun Oct 13 13:26:11 2024  -
--------------------------------------------------

 Geometry Optimization Parameters
   NAtoms,     NIC,      NZ,  NCons,    NDum,    NFix, NCnnct, MaxDiis
       38     272        0        0        0        0        0        0

 Cartesian Hessian Update
 Hessian updated using BFGS update
```

```
** GEOMETRY OPTIMIZATION IN DELOCALIZED INTERNAL COORDINATES **
   Searching for a Minimum

   Optimization Cycle:   5

                    Coordinates (Angstroms)
     ATOM                X               Y                Z
      1  N         2.0196994875    0.1717867896   -1.6553569787
      2  C         2.2765446983   -0.6893664834   -2.6788662859
      3  C         2.3758567795   -0.1666979850   -3.9620844431
      4  C         2.2267559021    1.1891157138   -4.1979514104
      5  C         1.9734099521    2.0430333105   -3.1264618265
      6  C         1.8778928671    1.5053838967   -1.8712714343
      7  C         2.4787841414   -2.1509127507   -2.4226582506
      8  C         1.9209472156   -0.2931514317   -0.2434537355
      9  N        -1.6689840304   -0.9669707884    0.5966505936
     10  C        -2.0509560046    0.1602178188    1.2585964457
     11  N        -1.1768525561    1.1022594884    1.5055821294
     12  C         0.0986999013    0.9684315477    1.1107280017
     13  C         0.5273202392   -0.1546507309    0.3287387683
     14  C        -0.4125101474   -1.1217988487    0.1251186585
     15  C        -3.4558844861    0.2543976078    1.7366251510
     16  C        -3.6971609479   -0.6184155867    2.9933732883
     17  C        -2.8926338165   -0.1721248787    4.2086351621
     18  N         0.9116405595    1.9916581061    1.4216933842
     19  H         2.5832726612   -0.8461398143   -4.7783142705
     20  H         2.3135004277    1.5803375417   -5.2047346503
     21  H         1.8605342841    3.1104799376   -3.2600355433
     22  H         1.6892292391    2.1132601231   -0.9956812263
     23  H         2.6094763272   -2.6671569744   -3.3714710141
     24  H         1.6275074953   -2.6110710931   -1.9164374342
     25  H         3.3776003616   -2.3306740080   -1.8255607905
     26  H         2.6494140583    0.2815749182    0.3264508650
     27  H         2.2489259912   -1.3268395660   -0.1954637647
     28  H        -2.3547388215   -1.6991207606    0.4506599103
     29  H        -0.2148613213   -2.0392800007   -0.4159150704
     30  H        -3.6535006908    1.3020766835    1.9631890287
     31  H        -4.1321901866   -0.0555409542    0.9333345884
     32  H        -3.4880633496   -1.6698657756    2.7631670272
     33  H        -4.7660251163   -0.5639578643    3.2075063834
     34  H        -1.8139864923   -0.2797127815    4.0558424523
     35  H        -3.1591769791   -0.7729696847    5.0788555232
     36  H        -3.0946298860    0.8738444323    4.4515758528
     37  H         1.8957539628    1.8507166817    1.5929335566
     38  H         0.4893882806    2.6978441641    2.0124613584
   Point Group: c1    Number of degrees of freedom:   108


   Energy is   -763.965033615

 Hessian updated using BFGS update
  internal optimization (0)

108 Hessian modes will be used to form the next step
  Hessian Eigenvalues:
     0.001687    0.002668    0.004084    0.005850    0.007511    0.011921
     0.014867    0.018799    0.019458    0.019951    0.021474    0.022068
     0.022706    0.022913    0.024353    0.024503    0.025834    0.026637
     0.027030    0.028211    0.029514    0.030217    0.032484    0.036920
     0.039063    0.041598    0.043231    0.043830    0.045659    0.046267
     0.051609    0.053694    0.054180    0.055554    0.064120    0.079439
     0.085544    0.091994    0.121757    0.122343    0.127120    0.130614
     0.131906    0.132870    0.138610    0.141232    0.142921    0.144708
     0.146920    0.147679    0.149801    0.151922    0.152402    0.153203
     0.153687    0.158163    0.193270    0.206302    0.209682    0.215885
     0.225977    0.227238    0.239265    0.246338    0.249411    0.267523
     0.269471    0.273988    0.286109    0.291201    0.300350    0.300532
     0.300798    0.301442    0.301892    0.303112    0.303994    0.304714
```

```
    0.305181    0.305238    0.306051    0.309092    0.311887    0.316188
    0.318565    0.332095    0.333364    0.334197    0.340314    0.343213
    0.348717    0.352106    0.362163    0.364636    0.381905    0.385466
    0.395683    0.403348    0.407475    0.415742    0.416839    0.424089
    0.437532    0.446218    0.463748    0.511968    0.579673    0.756911

 Minimum search - taking simple RFO step
 Searching for Lamda that Minimizes Along All modes
 Value Taken    Lamda =  -0.00223306
 Calculated Step too Large.  Step scaled by  0.660197
 Step Taken.  Stepsize is  0.300000

                             Maximum     Tolerance    Cnvgd?
         Gradient           0.002207      0.000800      NO
         Displacement       0.110257      0.001400      NO
         Energy change     -0.001853      0.000228      NO


 New Cartesian Coordinates Obtained by Inverse Iteration

 Displacement from previous Coordinates is:  0.541767
 ----------------------------------------------------------------
             Standard Nuclear Orientation (Angstroms)
    I     Atom           X                Y                Z
 ----------------------------------------------------------------
    1      N       2.0102281688     0.1643181199    -1.6630968987
    2      C       2.2991036752    -0.6948575097    -2.6791905220
    3      C       2.4004243491    -0.1742070669    -3.9641429433
    4      C       2.2210054627     1.1759038004    -4.2082357766
    5      C       1.9298110527     2.0274171187    -3.1440428785
    6      C       1.8316574920     1.4916971132    -1.8880161632
    7      C       2.5270922950    -2.1511084153    -2.4147796046
    8      C       1.9105499572    -0.2971311799    -0.2496573440
    9      N      -1.6786109847    -0.9439358239     0.6016936335
   10      C      -2.0520973108     0.1837707603     1.2720179668
   11      N      -1.1733817328     1.1159151962     1.5228195628
   12      C       0.1035443996     0.9788177193     1.1255664804
   13      C       0.5224855267    -0.1404155109     0.3281235422
   14      C      -0.4247164827    -1.0986236351     0.1246078074
   15      C      -3.4578272356     0.2851606846     1.7464055811
   16      C      -3.7126613146    -0.6057873841     2.9876387476
   17      C      -2.8893336861    -0.2036861933     4.2054556214
   18      N       0.9187369313     1.9755610879     1.4745799153
   19      H       2.6323313730    -0.8526227117    -4.7746624338
   20      H       2.3108930283     1.5646771249    -5.2156342046
   21      H       1.7885899255     3.0907679895    -3.2836475465
   22      H       1.6106929330     2.0974983218    -1.0189620631
   23      H       2.6893063566    -2.6664397857    -3.3593482210
   24      H       1.6734323219    -2.6266389362    -1.9266076475
   25      H       3.4158488659    -2.3115822668    -1.7977771038
   26      H       2.6565538266     0.2603666208     0.3164093968
   27      H       2.2190517622    -1.3366942824    -0.2059061532
   28      H      -2.3665793832    -1.6729576018     0.4533194375
   29      H      -0.2309922947    -2.0138192399    -0.4220049689
   30      H      -3.6435781107     1.3311378559     1.9906071283
   31      H      -4.1341307663    -0.0036540726     0.9354826638
   32      H      -3.5340656733    -1.6576180109     2.7349972027
   33      H      -4.7785346597    -0.5274405875     3.2093752648
   34      H      -1.8146574615    -0.3335582694     4.0423038000
   35      H      -3.1636141160    -0.8154548986     5.0656170082
   36      H      -3.0646716790     0.8416253828     4.4710346139
   37      H       1.9197993856     1.8711924186     1.5115074356
   38      H       0.5283138025     2.6724060680     2.0961496632
 ----------------------------------------------------------------
 Nuclear Repulsion Energy =        1306.29202271 hartrees
 There are       65 alpha and       65 beta electrons

 --------------------------------------------------------
 -  Entering fldman on Sun Oct 13 13:26:11 2024  -
 --------------------------------------------------------
```

```
 Applying Cartesian multipole field
    Component           Value
    ---------           -----
      (2,0,0)        1.00000E-11
      (0,2,0)        2.00000E-11
      (0,0,2)       -3.00000E-12
 Nucleus-field energy     =     0.0000000288 hartrees

--------------------------------------------------
-  Entering gesman on Sun Oct 13 13:26:11 2024  -
--------------------------------------------------

 Requested basis set is 6-311+G(d,p)
 There are 188 shells and 516 basis functions
 A cutoff of  1.0D-12 yielded  12634 shell pairs
 There are    100752 function pairs (    107395 Cartesian)
 Smallest overlap matrix eigenvalue = 2.13E-06
 Linear dependence detected in AO basis
 Tighter screening thresholds may be required for diffuse basis sets
 Use S2THRESH > 12 and THRESH = 14 in case of SCF convergence issues
 Number of orthogonalized atomic orbitals = 510
 Maximum deviation from orthogonality = 1.090E-11
 Guess MOs from SCF MO coefficient file
 Reading MOs from coefficient file
 Reading MOs from coefficient file

--------------------------------------------------
-  Entering scfman on Sun Oct 13 13:26:11 2024  -
--------------------------------------------------

 Long-range K will be added via erf
 Coulomb attenuation parameter = 0.2 bohr**(-1)
 A restricted hybrid HF-DFT SCF calculation will be
 performed using Pulay DIIS + Geometric Direct Minimization
 Exchange:     0.2220 Hartree-Fock + 1.0000 wB97X-D + LR-HF
 Correlation:  1.0000 wB97X-D
 Using Euler-Maclaurin-Lebedev (75,302) quadrature formula
 Dispersion:   Grimme D
 SCF converges when RMS gradient is below 1.0E-07
 Geometry optimization detected.  Setting ReadMinima to 0
 Setting SaveMinima to 0
 ---------------------------------------
  Cycle       Energy         DIIS Error
 ---------------------------------------
    1    -763.9581474569      5.20E-04
    2    -763.9657038611      7.83E-05
    3    -763.9660224009      5.10E-05
    4    -763.9661034229      2.19E-05
    5    -763.9661218479      5.96E-06
    6    -763.9661234721      2.53E-06
    7    -763.9661237510      8.30E-07
    8    -763.9661237931      3.40E-07
    9    -763.9661238000      1.06E-07
   10    -763.9661238010      4.65E-08 Convergence criterion met
 ---------------------------------------
 SCF time:  CPU 328.69 s  wall 629.25 s
 SCF   energy in the final basis set = -763.96612380
 Total energy in the final basis set = -763.96612380

--------------------------------------------------
-  Entering anlman on Sun Oct 13 13:36:40 2024  -
--------------------------------------------------


 --------------------------------------------------------------

                   Orbital Energies (a.u.)
 --------------------------------------------------------------
```

```
 Alpha MOs
 -- Occupied --
-14.8139 -14.7920 -14.7385 -14.7094 -10.6789 -10.6637 -10.6501 -10.6389
-10.6345 -10.6237 -10.6062 -10.5822 -10.5715 -10.5692 -10.5497 -10.5265
-10.4989 -10.4620  -1.4173  -1.4027  -1.3499  -1.2775  -1.2301  -1.1997
 -1.1869  -1.1425  -1.1000  -1.0830  -1.0575  -1.0533  -1.0299  -1.0054
 -0.9892  -0.9536  -0.9452  -0.9192  -0.9082  -0.8904  -0.8830  -0.8666
 -0.8507  -0.8434  -0.8353  -0.8195  -0.8140  -0.7942  -0.7891  -0.7813
 -0.7745  -0.7571  -0.7500  -0.7488  -0.7456  -0.7273  -0.7227  -0.7159
 -0.6952  -0.6665  -0.6568  -0.6483  -0.6345  -0.6245  -0.6233  -0.6198
 -0.6120
 -- Virtual --
 -0.2705  -0.2618  -0.2467  -0.2238  -0.1744  -0.1650  -0.1456  -0.1427
 -0.1351  -0.1250  -0.1210  -0.1134  -0.1083  -0.1043  -0.0992  -0.0894
 -0.0864  -0.0831  -0.0800  -0.0777  -0.0767  -0.0722  -0.0711  -0.0650
 -0.0618  -0.0600  -0.0546  -0.0505  -0.0497  -0.0435  -0.0408  -0.0351
 -0.0316  -0.0264  -0.0246  -0.0230  -0.0190  -0.0184  -0.0133  -0.0085
 -0.0069  -0.0040   0.0010   0.0043   0.0058   0.0124   0.0139   0.0157
  0.0209   0.0219   0.0259   0.0303   0.0329   0.0360   0.0380   0.0397
  0.0415   0.0436   0.0491   0.0537   0.0547   0.0597   0.0620   0.0673
  0.0706   0.0732   0.0749   0.0778   0.0790   0.0826   0.0863   0.0871
  0.0930   0.0944   0.1002   0.1029   0.1045   0.1075   0.1112   0.1163
  0.1200   0.1245   0.1273   0.1308   0.1379   0.1395   0.1440   0.1455
  0.1517   0.1584   0.1625   0.1659   0.1744   0.1749   0.1777   0.1801
  0.1841   0.1857   0.1875   0.1961   0.1970   0.2050   0.2156   0.2171
  0.2259   0.2306   0.2444   0.2646   0.2780   0.2873   0.3126   0.3190
  0.3249   0.3295   0.3380   0.3409   0.3457   0.3552   0.3608   0.3637
  0.3757   0.3785   0.3849   0.3934   0.3971   0.4031   0.4082   0.4222
  0.4312   0.4340   0.4401   0.4439   0.4552   0.4585   0.4680   0.4774
  0.4826   0.4912   0.4953   0.5026   0.5117   0.5133   0.5201   0.5230
  0.5289   0.5301   0.5323   0.5395   0.5473   0.5498   0.5549   0.5589
  0.5626   0.5668   0.5700   0.5780   0.5867   0.5915   0.5977   0.6056
  0.6125   0.6185   0.6268   0.6287   0.6330   0.6397   0.6503   0.6581
  0.6636   0.6753   0.6857   0.6873   0.6921   0.6971   0.7024   0.7078
  0.7103   0.7227   0.7306   0.7477   0.7563   0.7613   0.7731   0.7790
  0.7900   0.8043   0.8071   0.8131   0.8343   0.8447   0.8488   0.8610
  0.8640   0.8724   0.8796   0.9014   0.9065   0.9203   0.9221   0.9273
  0.9428   0.9499   0.9647   0.9749   0.9852   0.9975   1.0100   1.0424
  1.0530   1.0624   1.0796   1.0913   1.1256   1.1708   1.1821   1.1890
  1.2025   1.2044   1.2203   1.2448   1.2557   1.2677   1.2952   1.3017
  1.3117   1.3237   1.3319   1.3386   1.3436   1.3596   1.3705   1.3816
  1.3893   1.3917   1.4007   1.4019   1.4121   1.4162   1.4278   1.4368
  1.4398   1.4462   1.4545   1.4622   1.4725   1.4783   1.4812   1.4854
  1.4927   1.5055   1.5138   1.5243   1.5338   1.5410   1.5512   1.5554
  1.5677   1.5692   1.5756   1.5815   1.5843   1.6018   1.6126   1.6199
  1.6286   1.6348   1.6461   1.6524   1.6618   1.6645   1.6835   1.6863
  1.6946   1.7022   1.7197   1.7343   1.7445   1.7456   1.7671   1.7699
  1.7790   1.7875   1.8057   1.8168   1.8350   1.8457   1.8585   1.8786
  1.8913   1.8971   1.9178   1.9307   1.9415   1.9473   1.9711   1.9886
  1.9932   2.0130   2.0164   2.0312   2.0576   2.0647   2.0859   2.0877
  2.1080   2.1128   2.1326   2.1431   2.1660   2.1911   2.2216   2.2253
  2.2295   2.2368   2.2526   2.2575   2.2764   2.2833   2.2895   2.2914
  2.3012   2.3106   2.3446   2.3649   2.3690   2.3761   2.3877   2.3974
  2.4097   2.4218   2.4324   2.4395   2.4504   2.4653   2.4672   2.4790
  2.5007   2.5183   2.5245   2.5325   2.5375   2.5511   2.5519   2.5570
  2.5658   2.5727   2.5781   2.5872   2.5915   2.5994   2.6138   2.6236
  2.6288   2.6429   2.6560   2.6709   2.6774   2.6825   2.6922   2.6995
  2.7094   2.7283   2.7411   2.7453   2.7536   2.7611   2.7793   2.7843
  2.7918   2.7947   2.8152   2.8285   2.8520   2.8569   2.8751   2.8896
  2.9080   2.9260   2.9597   2.9789   3.0458   3.0643   3.1148   3.1422
  3.1695   3.2303   3.2523   3.2599   3.2995   3.3304   3.3653   3.3997
  3.4194   3.4749   3.5098   3.5561   3.5634   3.5941   3.6368   3.6412
  3.6653   3.7007   3.7191   3.7209   3.7371   3.7660   3.7677   3.7763
  3.7962   3.8388   3.8746   3.9099   3.9340   3.9795   4.0295   4.0909
  4.1088   4.2050   4.2961   4.3018   4.5931   4.6656   4.7090   4.7159
  4.9158   5.0455   5.1927  23.5257  23.5730  23.7201  23.7275  23.7371
 23.7696  23.7969  23.8261  23.8592  23.8869  23.9223  23.9691  23.9778
 24.0377  35.4259  35.4543  35.5158  35.6141
 --------------------------------------------------------------
```

Ground-State Mulliken Net Atomic Charges

| Atom | | Charge (a.u.) |
|---|---|---|
| 1 | N | 0.409555 |
| 2 | C | 0.167257 |
| 3 | C | -0.031885 |
| 4 | C | -0.155595 |
| 5 | C | -0.171473 |
| 6 | C | -0.183183 |
| 7 | C | -0.816451 |
| 8 | C | -0.298028 |
| 9 | N | -0.127088 |
| 10 | C | -0.235254 |
| 11 | N | 0.014765 |
| 12 | C | 0.208577 |
| 13 | C | 0.009132 |
| 14 | C | 0.109135 |
| 15 | C | -0.402775 |
| 16 | C | -0.218441 |
| 17 | C | -0.512717 |
| 18 | N | -0.482500 |
| 19 | H | 0.219203 |
| 20 | H | 0.234559 |
| 21 | H | 0.227251 |
| 22 | H | 0.215725 |
| 23 | H | 0.229933 |
| 24 | H | 0.182514 |
| 25 | H | 0.250913 |
| 26 | H | 0.228207 |
| 27 | H | 0.243124 |
| 28 | H | 0.387522 |
| 29 | H | 0.201410 |
| 30 | H | 0.244473 |
| 31 | H | 0.230670 |
| 32 | H | 0.168138 |
| 33 | H | 0.225135 |
| 34 | H | 0.140232 |
| 35 | H | 0.208235 |
| 36 | H | 0.190419 |
| 37 | H | 0.316282 |
| 38 | H | 0.373023 |

Sum of atomic charges = 2.000000

Cartesian Multipole Moments

Charge (ESU x 10^10)

9.6064

Dipole Moment (Debye)

| | | | | | |
|---|---|---|---|---|---|
| X | 4.8664 | Y | -0.0973 | Z | -5.6443 |
| Tot | 7.4531 | | | | |

Quadrupole Moments (Debye-Ang)

| | | | | | |
|---|---|---|---|---|---|
| XX | -45.1525 | XY | 9.1344 | YY | -70.8577 |
| XZ | -40.9788 | YZ | 1.4711 | ZZ | -36.8617 |

Traceless Quadrupole Moments (Debye-Ang)

| | | | | | |
|---|---|---|---|---|---|
| QXX | 17.4144 | QYY | -59.7013 | QZZ | 42.2869 |
| QXY | 27.4032 | QXZ | -122.9364 | QYZ | 4.4133 |

Octopole Moments (Debye-Ang^2)

| | | | | | |
|---|---|---|---|---|---|
| XXX | -79.0863 | XXY | -32.1819 | XYY | 23.6074 |
| YYY | -23.7939 | XXZ | 19.5489 | XYZ | 11.3407 |
| YYZ | -13.3401 | XZZ | 66.4522 | YZZ | 21.4587 |
| ZZZ | -103.5813 | | | | |

Traceless Octopole Moments (Debye-Ang^2)

| | | | | | |
|---|---|---|---|---|---|
| XXX | -1285.0539 | YYY | -46.2546 | ZZZ | -677.3673 |
| XXY | -379.1777 | XXZ | 585.3511 | XYY | 321.1915 |
| XYZ | 170.1112 | XZZ | 963.8624 | YYZ | 92.0161 |
| YZZ | 425.4324 | | | | |

Hexadecapole Moments (Debye-Ang^3)

```
      XXXX   -2560.5108   XXXY      -96.4469   XXYY    -550.8031
      XYYY     -41.1123   YYYY     -781.8931   XXXZ    1046.7388
      XXYZ      33.7750   XYYZ      362.1231   YYYZ      52.9407
      XXZZ    -680.0560   XYZZ       37.5641   YYZZ    -560.8262
      XZZZ     685.2602   YZZZ      -83.5078   ZZZZ   -2520.0613
    Traceless Hexadecapole Moments (Debye-Ang^3)
      XXXX  -12642.8638   XXXY    -5627.1436   XXXZ   15672.0766
      XXYY    -898.4490   XXYZ     3490.6389   XXZZ   13541.3128
      XYYY     182.9840   XYYZ     6611.0955   XYZZ    5444.1596
      XZZZ  -22283.1720   YYYY     3305.7207   YYYZ    5391.5666
      YYZZ   -2407.2716   YZZZ    -8882.2055   ZZZZ  -11134.0412
 -----------------------------------------------------------------

--------------------------------------------------
-  Entering drvman on Sun Oct 13 13:36:40 2024  -
--------------------------------------------------

 Calculating analytic gradient of the SCF energy
 Gradient of SCF Energy
           1           2           3           4           5           6
    1   0.0001703  -0.0001844  -0.0000408   0.0000917  -0.0000158   0.0002372
    2   0.0000791  -0.0004381   0.0004452  -0.0000543   0.0000276   0.0003407
    3  -0.0000913  -0.0001585  -0.0000379  -0.0003056  -0.0001865   0.0003332
           7           8           9          10          11          12
    1  -0.0002778   0.0001346   0.0000368  -0.0005563   0.0002398   0.0006734
    2   0.0000381  -0.0001659   0.0001543   0.0010527  -0.0011719   0.0008166
    3   0.0002056  -0.0001885   0.0000370   0.0004459  -0.0005765   0.0032214
          13          14          15          16          17          18
    1  -0.0004585   0.0005580   0.0000746  -0.0000883   0.0000341  -0.0008034
    2  -0.0009332   0.0001686   0.0000535  -0.0000174  -0.0000237   0.0007605
    3  -0.0007605  -0.0001840  -0.0005270   0.0005437   0.0000671  -0.0042939
          19          20          21          22          23          24
    1  -0.0000448  -0.0000169   0.0000623   0.0000158  -0.0000518   0.0000436
    2  -0.0000280  -0.0000837  -0.0000853  -0.0003398   0.0001418  -0.0000516
    3   0.0002223   0.0001460   0.0001266   0.0003128   0.0000663   0.0001088
          25          26          27          28          29          30
    1  -0.0000089  -0.0003386   0.0001591   0.0001949   0.0000836   0.0002314
    2  -0.0000454   0.0002572   0.0001456   0.0001124   0.0000175  -0.0000666
    3  -0.0000145  -0.0002912  -0.0000847  -0.0000043  -0.0000618  -0.0000695
          31          32          33          34          35          36
    1  -0.0000977   0.0001388   0.0000474  -0.0000137   0.0000081   0.0000211
    2  -0.0000133   0.0000204  -0.0001430   0.0000523   0.0000968   0.0000020
    3   0.0000903  -0.0000284  -0.0002421   0.0000782  -0.0000350  -0.0000164
          37          38
    1  -0.0007271   0.0004682
    2  -0.0000731  -0.0010485
    3   0.0018619   0.0002907
 Max gradient component =       4.294E-03
 RMS gradient           =       6.260E-04
 Gradient time:  CPU 142.95 s  wall 285.56 s

--------------------------------------------------
-  Entering optman on Sun Oct 13 13:41:26 2024  -
--------------------------------------------------

 Geometry Optimization Parameters
   NAtoms,    NIC,     NZ,  NCons,   NDum,   NFix, NCnnct, MaxDiis
       38     272       0       0       0       0       0       0

 Cartesian Hessian Update
 Hessian updated using BFGS update


** GEOMETRY OPTIMIZATION IN DELOCALIZED INTERNAL COORDINATES **
   Searching for a Minimum

   Optimization Cycle:   6

                        Coordinates (Angstroms)
     ATOM                X               Y               Z
```

| | | | | |
|---|---|---|---|---|
| 1 | N | 2.0102281688 | 0.1643181199 | -1.6630968987 |
| 2 | C | 2.2991036752 | -0.6948575097 | -2.6791905220 |
| 3 | C | 2.4004243491 | -0.1742070669 | -3.9641429433 |
| 4 | C | 2.2210054627 | 1.1759038004 | -4.2082357766 |
| 5 | C | 1.9298110527 | 2.0274171187 | -3.1440428785 |
| 6 | C | 1.8316574920 | 1.4916971132 | -1.8880161632 |
| 7 | C | 2.5270922950 | -2.1511084153 | -2.4147796046 |
| 8 | C | 1.9105499572 | -0.2971311799 | -0.2496573440 |
| 9 | N | -1.6786109847 | -0.9439358239 | 0.6016936335 |
| 10 | C | -2.0520973108 | 0.1837707603 | 1.2720179668 |
| 11 | N | -1.1733817328 | 1.1159151962 | 1.5228195628 |
| 12 | C | 0.1035443996 | 0.9788177193 | 1.1255664804 |
| 13 | C | 0.5224855267 | -0.1404155109 | 0.3281235422 |
| 14 | C | -0.4247164827 | -1.0986236351 | 0.1246078074 |
| 15 | C | -3.4578272356 | 0.2851606846 | 1.7464055811 |
| 16 | C | -3.7126613146 | -0.6057873841 | 2.9876387476 |
| 17 | C | -2.8893336861 | -0.2036861933 | 4.2054556214 |
| 18 | N | 0.9187369313 | 1.9755610879 | 1.4745799153 |
| 19 | H | 2.6323313730 | -0.8526227117 | -4.7746624338 |
| 20 | H | 2.3108930283 | 1.5646771249 | -5.2156342046 |
| 21 | H | 1.7885899255 | 3.0907679895 | -3.2836475465 |
| 22 | H | 1.6106929330 | 2.0974983218 | -1.0189620631 |
| 23 | H | 2.6893063566 | -2.6664397857 | -3.3593482210 |
| 24 | H | 1.6734323219 | -2.6266389362 | -1.9266076475 |
| 25 | H | 3.4158488659 | -2.3115822668 | -1.7977771038 |
| 26 | H | 2.6565538266 | 0.2603666208 | 0.3164093968 |
| 27 | H | 2.2190517622 | -1.3366942824 | -0.2059061532 |
| 28 | H | -2.3665793832 | -1.6729576018 | 0.4533194375 |
| 29 | H | -0.2309922947 | -2.0138192399 | -0.4220049689 |
| 30 | H | -3.6435781107 | 1.3311378559 | 1.9906071283 |
| 31 | H | -4.1341307663 | -0.0036540726 | 0.9354826638 |
| 32 | H | -3.5340656733 | -1.6576180109 | 2.7349972027 |
| 33 | H | -4.7785346597 | -0.5274405875 | 3.2093752648 |
| 34 | H | -1.8146574615 | -0.3335582694 | 4.0423038000 |
| 35 | H | -3.1636141160 | -0.8154548986 | 5.0656170082 |
| 36 | H | -3.0646716790 | 0.8416253828 | 4.4710346139 |
| 37 | H | 1.9197993856 | 1.8711924186 | 1.5115074356 |
| 38 | H | 0.5283138025 | 2.6724060680 | 2.0961496632 |

Point Group: c1    Number of degrees of freedom: 108

Energy is -763.966123801

Hessian updated using BFGS update
internal optimization (0)

108 Hessian modes will be used to form the next step
Hessian Eigenvalues:

| | | | | | |
|---|---|---|---|---|---|
| 0.001687 | 0.002668 | 0.004091 | 0.005863 | 0.007240 | 0.012392 |
| 0.014942 | 0.018777 | 0.019528 | 0.019951 | 0.021598 | 0.022156 |
| 0.022688 | 0.022865 | 0.024387 | 0.024509 | 0.025843 | 0.026740 |
| 0.026953 | 0.028206 | 0.028957 | 0.030143 | 0.032203 | 0.036897 |
| 0.039066 | 0.041592 | 0.043231 | 0.043833 | 0.045654 | 0.046259 |
| 0.051354 | 0.053620 | 0.053989 | 0.055548 | 0.064141 | 0.079493 |
| 0.085545 | 0.092050 | 0.121758 | 0.122342 | 0.127120 | 0.130610 |
| 0.131927 | 0.132873 | 0.138597 | 0.141496 | 0.142923 | 0.144650 |
| 0.147061 | 0.147679 | 0.149808 | 0.152007 | 0.152402 | 0.153229 |
| 0.153943 | 0.158091 | 0.193259 | 0.206348 | 0.209665 | 0.215893 |
| 0.226358 | 0.227533 | 0.239241 | 0.246321 | 0.249598 | 0.267509 |
| 0.269470 | 0.274018 | 0.286131 | 0.291222 | 0.300352 | 0.300521 |
| 0.300791 | 0.301453 | 0.301886 | 0.303111 | 0.303994 | 0.304716 |
| 0.305180 | 0.305239 | 0.306044 | 0.309076 | 0.311889 | 0.316165 |
| 0.318558 | 0.332241 | 0.333361 | 0.334173 | 0.340420 | 0.343224 |
| 0.349005 | 0.352458 | 0.362558 | 0.364539 | 0.382056 | 0.385424 |
| 0.395881 | 0.403738 | 0.407485 | 0.415771 | 0.417752 | 0.429948 |
| 0.439961 | 0.448291 | 0.464131 | 0.512106 | 0.579586 | 0.757124 |

Minimum search - taking simple RFO step
Searching for Lamda that Minimizes Along All modes
Value Taken    Lamda =  -0.00073638

```
 Step Taken.  Stepsize is  0.284596

                             Maximum     Tolerance    Cnvgd?
         Gradient           0.001535      0.000800      NO
         Displacement       0.103123      0.001400      NO
         Energy change     -0.001090      0.000228      NO


 New Cartesian Coordinates Obtained by Inverse Iteration

 Displacement from previous Coordinates is:  0.694115
 ----------------------------------------------------------------
             Standard Nuclear Orientation (Angstroms)
    I     Atom           X                Y                Z
 ----------------------------------------------------------------
    1      N       1.9958827823     0.1540452136    -1.6743803726
    2      C       2.3306280572    -0.7008147914    -2.6800402092
    3      C       2.4339544358    -0.1853338281    -3.9666625635
    4      C       2.2118752963     1.1561197669    -4.2222416059
    5      C       1.8723723924     2.0039904347    -3.1691355170
    6      C       1.7722712945     1.4721586953    -1.9125230556
    7      C       2.5972437687    -2.1483960994    -2.4039139409
    8      C       1.8940164518    -0.3024206281    -0.2583422226
    9      N      -1.6970460722    -0.9105138347     0.6034559925
   10      C      -2.0549260303     0.2150154065     1.2889896500
   11      N      -1.1657247934     1.1345122126     1.5470366719
   12      C       0.1105990564     0.9894423989     1.1426944749
   13      C       0.5118150758    -0.1234722260     0.3252405877
   14      C      -0.4477627889    -1.0695575569     0.1169746019
   15      C      -3.4596828992     0.3271939060     1.7657568069
   16      C      -3.7321796390    -0.5896607042     2.9833764457
   17      C      -2.8846925660    -0.2478195832     4.2029973209
   18      N       0.9413997673     1.9531907507     1.5300574460
   19      H       2.7005014870    -0.8603468094    -4.7693664588
   20      H       2.3047346499     1.5416205391    -5.2308501794
   21      H       1.6948327371     3.0605660706    -3.3186446594
   22      H       1.5144689166     2.0773730457    -1.0532962512
   23      H       2.8065693016    -2.6606753103    -3.3409940664
   24      H       1.7411984775    -2.6477614938    -1.9434532476
   25      H       3.4691294568    -2.2804975996    -1.7571364787
   26      H       2.6576099574     0.2358650479     0.3041031037
   27      H       2.1779805924    -1.3488252476    -0.2159796390
   28      H      -2.3921037108    -1.6317987692     0.4511798430
   29      H      -0.2630053467    -1.9804981454    -0.4401837907
   30      H      -3.6282968870     1.3697146307     2.0360471490
   31      H      -4.1384739712     0.0705252313     0.9461740516
   32      H      -3.5925222933    -1.6394950697     2.7002501308
   33      H      -4.7926226898    -0.4816713279     3.2188219969
   34      H      -1.8161697249    -0.4056490115     4.0240656238
   35      H      -3.1683829951    -0.8753406547     5.0486798502
   36      H      -3.0251942162     0.7943332120     4.4999438529
   37      H       1.9426407876     1.8844806816     1.4549229544
   38      H       0.5770618818     2.6504014470     2.1663757039
 ----------------------------------------------------------------
 Nuclear Repulsion Energy =        1305.29102209 hartrees
 There are       65 alpha and       65 beta electrons

--------------------------------------------------
-  Entering fldman on Sun Oct 13 13:41:26 2024  -
--------------------------------------------------

 Applying Cartesian multipole field
    Component           Value
    ---------           -----
      (2,0,0)        1.00000E-11
      (0,2,0)        2.00000E-11
      (0,0,2)       -3.00000E-12
 Nucleus-field energy     =     0.0000000287 hartrees

--------------------------------------------------
```

```
-  Entering gesman on Sun Oct 13 13:41:26 2024  -
--------------------------------------------------

 Requested basis set is 6-311+G(d,p)
 There are 188 shells and 516 basis functions
 A cutoff of  1.0D-12 yielded  12634 shell pairs
 There are     100745 function pairs (     107389 Cartesian)
 Smallest overlap matrix eigenvalue = 2.10E-06
 Linear dependence detected in AO basis
 Tighter screening thresholds may be required for diffuse basis sets
 Use S2THRESH > 12 and THRESH = 14 in case of SCF convergence issues
 Number of orthogonalized atomic orbitals = 510
 Maximum deviation from orthogonality = 2.807E-11
 Guess MOs from SCF MO coefficient file
 Reading MOs from coefficient file
 Reading MOs from coefficient file

--------------------------------------------------
-  Entering scfman on Sun Oct 13 13:41:26 2024  -
--------------------------------------------------

 Long-range K will be added via erf
 Coulomb attenuation parameter = 0.2 bohr**(-1)
 A restricted hybrid HF-DFT SCF calculation will be
 performed using Pulay DIIS + Geometric Direct Minimization
 Exchange:     0.2220 Hartree-Fock + 1.0000 wB97X-D + LR-HF
 Correlation:  1.0000 wB97X-D
 Using Euler-Maclaurin-Lebedev (75,302) quadrature formula
 Dispersion:   Grimme D
 SCF converges when RMS gradient is below 1.0E-07
 Geometry optimization detected.  Setting ReadMinima to 0
 Setting SaveMinima to 0
 ---------------------------------------
  Cycle       Energy         DIIS Error
 ---------------------------------------
    1    -763.9432543933      6.32E-04
    2    -763.9660181316      8.62E-05
    3    -763.9664731609      5.15E-05
    4    -763.9665549452      2.60E-05
    5    -763.9665795232      7.66E-06
    6    -763.9665818644      2.45E-06
    7    -763.9665821180      8.72E-07
    8    -763.9665821573      3.46E-07
    9    -763.9665821635      1.08E-07
   10    -763.9665821641      4.96E-08 Convergence criterion met
 ---------------------------------------
 SCF time:  CPU 335.16 s  wall 641.08 s
 SCF   energy in the final basis set = -763.96658216
 Total energy in the final basis set = -763.96658216

--------------------------------------------------
-  Entering anlman on Sun Oct 13 13:52:07 2024  -
--------------------------------------------------


 --------------------------------------------------------------

                   Orbital Energies (a.u.)
 --------------------------------------------------------------

 Alpha MOs
 -- Occupied --
-14.8143 -14.7905 -14.7400 -14.7083 -10.6788 -10.6626 -10.6493 -10.6392
-10.6347 -10.6245 -10.6055 -10.5826 -10.5721 -10.5695 -10.5493 -10.5256
-10.4982 -10.4618  -1.4158  -1.4029  -1.3498  -1.2769  -1.2301  -1.1994
 -1.1872  -1.1420  -1.0996  -1.0831  -1.0570  -1.0530  -1.0299  -1.0048
 -0.9894  -0.9539  -0.9447  -0.9201  -0.9088  -0.8904  -0.8834  -0.8661
 -0.8515  -0.8436  -0.8344  -0.8195  -0.8135  -0.7949  -0.7891  -0.7810
 -0.7744  -0.7578  -0.7493  -0.7488  -0.7453  -0.7276  -0.7223  -0.7158
 -0.6942  -0.6659  -0.6569  -0.6478  -0.6337  -0.6241  -0.6229  -0.6203
```

```
-0.6106
-- Virtual --
-0.2708  -0.2601  -0.2455  -0.2242  -0.1743  -0.1649  -0.1462  -0.1426
-0.1347  -0.1252  -0.1210  -0.1133  -0.1081  -0.1043  -0.0990  -0.0896
-0.0859  -0.0833  -0.0805  -0.0770  -0.0769  -0.0722  -0.0710  -0.0647
-0.0624  -0.0602  -0.0549  -0.0512  -0.0498  -0.0440  -0.0402  -0.0352
-0.0320  -0.0265  -0.0251  -0.0234  -0.0196  -0.0184  -0.0131  -0.0082
-0.0066  -0.0041   0.0017   0.0041   0.0065   0.0119   0.0139   0.0148
 0.0201   0.0220   0.0260   0.0298   0.0332   0.0369   0.0374   0.0404
 0.0415   0.0434   0.0487   0.0538   0.0549   0.0607   0.0620   0.0668
 0.0708   0.0747   0.0759   0.0780   0.0794   0.0831   0.0861   0.0897
 0.0925   0.0956   0.1011   0.1025   0.1049   0.1081   0.1114   0.1159
 0.1202   0.1237   0.1275   0.1302   0.1377   0.1406   0.1432   0.1454
 0.1512   0.1584   0.1613   0.1653   0.1749   0.1749   0.1792   0.1799
 0.1826   0.1837   0.1886   0.1934   0.1973   0.2039   0.2145   0.2193
 0.2219   0.2341   0.2456   0.2650   0.2768   0.2863   0.3121   0.3184
 0.3251   0.3300   0.3381   0.3411   0.3459   0.3558   0.3602   0.3635
 0.3762   0.3797   0.3847   0.3935   0.3990   0.4042   0.4095   0.4229
 0.4311   0.4314   0.4409   0.4436   0.4523   0.4571   0.4668   0.4754
 0.4828   0.4912   0.4952   0.5014   0.5106   0.5132   0.5195   0.5240
 0.5287   0.5310   0.5323   0.5381   0.5490   0.5526   0.5546   0.5615
 0.5634   0.5676   0.5696   0.5825   0.5869   0.5947   0.5958   0.6063
 0.6158   0.6170   0.6265   0.6305   0.6368   0.6405   0.6528   0.6569
 0.6618   0.6758   0.6836   0.6867   0.6920   0.6968   0.7009   0.7050
 0.7164   0.7263   0.7288   0.7478   0.7569   0.7620   0.7702   0.7813
 0.7911   0.8045   0.8087   0.8132   0.8368   0.8456   0.8490   0.8582
 0.8644   0.8742   0.8804   0.9041   0.9149   0.9200   0.9252   0.9319
 0.9436   0.9483   0.9674   0.9760   0.9854   0.9949   1.0089   1.0457
 1.0534   1.0615   1.0790   1.0873   1.1233   1.1705   1.1765   1.1856
 1.2012   1.2062   1.2115   1.2360   1.2548   1.2706   1.2943   1.3015
 1.3108   1.3252   1.3285   1.3373   1.3443   1.3549   1.3697   1.3800
 1.3848   1.3910   1.4004   1.4031   1.4109   1.4165   1.4252   1.4362
 1.4400   1.4431   1.4548   1.4616   1.4731   1.4813   1.4829   1.4866
 1.4919   1.5045   1.5155   1.5232   1.5341   1.5422   1.5512   1.5548
 1.5681   1.5713   1.5764   1.5768   1.5852   1.6026   1.6134   1.6207
 1.6285   1.6329   1.6461   1.6487   1.6623   1.6658   1.6794   1.6846
 1.6930   1.7017   1.7155   1.7341   1.7432   1.7489   1.7636   1.7686
 1.7797   1.7885   1.8045   1.8207   1.8338   1.8442   1.8643   1.8776
 1.8914   1.9000   1.9183   1.9303   1.9455   1.9593   1.9711   1.9884
 1.9951   2.0171   2.0226   2.0332   2.0543   2.0661   2.0835   2.0939
 2.1064   2.1142   2.1320   2.1422   2.1680   2.1942   2.2242   2.2267
 2.2290   2.2369   2.2528   2.2553   2.2736   2.2809   2.2833   2.2911
 2.3013   2.3084   2.3429   2.3597   2.3691   2.3758   2.3874   2.3973
 2.4087   2.4220   2.4316   2.4355   2.4479   2.4624   2.4680   2.4843
 2.4961   2.5177   2.5237   2.5354   2.5390   2.5492   2.5529   2.5564
 2.5665   2.5712   2.5767   2.5855   2.5913   2.6017   2.6143   2.6237
 2.6294   2.6423   2.6558   2.6691   2.6749   2.6840   2.6929   2.6967
 2.7108   2.7302   2.7421   2.7520   2.7544   2.7604   2.7806   2.7871
 2.7916   2.7943   2.8179   2.8318   2.8515   2.8600   2.8761   2.8899
 2.9027   2.9255   2.9631   2.9812   3.0497   3.0660   3.1176   3.1423
 3.1688   3.2431   3.2522   3.2654   3.2987   3.3324   3.3662   3.4075
 3.4189   3.4745   3.5112   3.5581   3.5628   3.5931   3.6348   3.6422
 3.6639   3.7008   3.7214   3.7223   3.7381   3.7611   3.7667   3.7716
 3.7767   3.8401   3.8733   3.9091   3.9348   3.9791   4.0297   4.0880
 4.1096   4.2056   4.2950   4.3019   4.5938   4.6667   4.7077   4.7178
 4.9205   5.0446   5.1953  23.5244  23.5731  23.7199  23.7276  23.7362
23.7705  23.7969  23.8250  23.8595  23.8864  23.9229  23.9695  23.9767
24.0380  35.4250  35.4560  35.5188  35.6164
--------------------------------------------------------------

        Ground-State Mulliken Net Atomic Charges

    Atom                 Charge (a.u.)
 ----------------------------------------
     1 N                    0.416155
     2 C                    0.148350
     3 C                   -0.019281
     4 C                   -0.152529
     5 C                   -0.178263
     6 C                   -0.182855
```

```
     7 C                   -0.802122
     8 C                   -0.308930
     9 N                   -0.117298
    10 C                   -0.253632
    11 N                    0.009973
    12 C                    0.239088
    13 C                    0.009223
    14 C                    0.096461
    15 C                   -0.400836
    16 C                   -0.222781
    17 C                   -0.509775
    18 N                   -0.483702
    19 H                    0.219429
    20 H                    0.234724
    21 H                    0.227242
    22 H                    0.205298
    23 H                    0.229859
    24 H                    0.187025
    25 H                    0.248287
    26 H                    0.230367
    27 H                    0.242372
    28 H                    0.384692
    29 H                    0.202250
    30 H                    0.243941
    31 H                    0.231466
    32 H                    0.167811
    33 H                    0.225248
    34 H                    0.140127
    35 H                    0.208049
    36 H                    0.190495
    37 H                    0.316766
    38 H                    0.377306
  ----------------------------------------
  Sum of atomic charges =     2.000000

-----------------------------------------------------------------
                   Cartesian Multipole Moments
-----------------------------------------------------------------
    Charge (ESU x 10^10)
                 9.6064
    Dipole Moment (Debye)
         X       4.9211      Y       0.0419      Z      -5.7720
       Tot       7.5852
    Quadrupole Moments (Debye-Ang)
        XX     -44.7185     XY       8.5545     YY     -71.1033
        XZ     -41.2918     YZ       1.6584     ZZ     -36.6558
    Traceless Quadrupole Moments (Debye-Ang)
       QXX      18.3222    QYY     -60.8324    QZZ      42.5102
       QXY      25.6636    QXZ    -123.8753    QYZ       4.9751
    Octopole Moments (Debye-Ang^2)
       XXX     -76.6809    XXY     -33.8343    XYY      24.0679
       YYY     -24.0271    XXZ      17.8746    XYZ      13.0839
       YYZ     -12.7942    XZZ      67.6007    YZZ      21.8673
       ZZZ    -105.4270
    Traceless Octopole Moments (Debye-Ang^2)
       XXX   -1285.1018    YYY     -36.4596    ZZZ    -678.2856
       XXY    -399.5324    XXZ     569.1591    XYY     316.0550
       XYZ     196.2584    XZZ     969.0468    YYZ     109.1264
       YZZ     435.9919
    Hexadecapole Moments (Debye-Ang^3)
      XXXX   -2564.0896   XXXY     -93.0468   XXYY    -551.7996
      XYYY     -31.9297   YYYY    -775.2506   XXXZ    1041.9241
      XXYZ      40.8690   XYYZ     367.1538   YYYZ      51.3836
      XXZZ    -679.0313   XYZZ      36.5708   YYZZ    -561.8997
      XZZZ     681.8009   YZZZ     -88.0547   ZZZZ   -2535.7553
    Traceless Hexadecapole Moments (Debye-Ang^3)
      XXXX  -12831.5701   XXXY   -5791.6581   XXXZ   15312.4845
      XXYY   -1062.5729   XXYZ    4227.4598   XXZZ   13894.1430
      XYYY     625.6363   XYYZ    7187.9635   XYZZ    5166.0218
      XZZZ  -22500.4480   YYYY    3459.1698   YYYZ    5203.9097
```

```
      YYZZ   -2396.5968   YZZZ   -9431.3695   ZZZZ  -11497.5462
 -------------------------------------------------------------------

--------------------------------------------------
-  Entering drvman on Sun Oct 13 13:52:07 2024  -
--------------------------------------------------

 Calculating analytic gradient of the SCF energy
 Gradient of SCF Energy
            1           2           3           4           5           6
    1   0.0004657  -0.0000529  -0.0000594   0.0000830  -0.0001438   0.0000903
    2   0.0000716  -0.0000590   0.0000595  -0.0002434   0.0002074   0.0002637
    3  -0.0001820  -0.0004071   0.0001796   0.0001460   0.0003778  -0.0004581
            7           8           9          10          11          12
    1  -0.0003000   0.0000343  -0.0001709  -0.0009048  -0.0000295   0.0010543
    2   0.0000128  -0.0001612  -0.0006514   0.0010746  -0.0004951   0.0018042
    3   0.0001441  -0.0000072  -0.0001449   0.0005778  -0.0006129   0.0014491
           13          14          15          16          17          18
    1   0.0006309  -0.0001718  -0.0001375   0.0000090   0.0001008  -0.0010942
    2  -0.0007884  -0.0003236  -0.0000818  -0.0000324  -0.0000380   0.0001786
    3  -0.0003572  -0.0001176  -0.0005234   0.0004824  -0.0000290  -0.0012584
           19          20          21          22          23          24
    1  -0.0000835   0.0000196   0.0000573  -0.0000649   0.0000019  -0.0001055
    2  -0.0000540  -0.0000299  -0.0000535  -0.0001616   0.0000642  -0.0000015
    3   0.0001792   0.0000356   0.0001406   0.0001938  -0.0000004   0.0001216
           25          26          27          28          29          30
    1   0.0000067   0.0000463  -0.0000344   0.0002679   0.0002385   0.0001544
    2  -0.0000493   0.0000201   0.0001247   0.0000265  -0.0000012  -0.0000172
    3  -0.0000558  -0.0002759  -0.0000217  -0.0000191  -0.0001122   0.0000087
           31          32          33          34          35          36
    1   0.0000396  -0.0000246   0.0000136  -0.0000207   0.0000057  -0.0000189
    2  -0.0000719   0.0000301  -0.0000428   0.0000564   0.0000590   0.0000082
    3   0.0000764  -0.0000605  -0.0001537   0.0000670  -0.0000169  -0.0000012
           37          38
    1  -0.0003607   0.0004580
    2   0.0000379  -0.0007423
    3   0.0001692   0.0004663
 Max gradient component =       1.804E-03
 RMS gradient           =       3.900E-04
 Gradient time:  CPU 180.83 s  wall 331.13 s

--------------------------------------------------
-  Entering optman on Sun Oct 13 13:57:39 2024  -
--------------------------------------------------

 Geometry Optimization Parameters
   NAtoms,    NIC,     NZ,  NCons,   NDum,   NFix, NCnnct, MaxDiis
       38     272       0       0       0       0       0       0

 Cartesian Hessian Update
 Hessian updated using BFGS update


** GEOMETRY OPTIMIZATION IN DELOCALIZED INTERNAL COORDINATES **
   Searching for a Minimum

   Optimization Cycle:   7

                        Coordinates (Angstroms)
     ATOM                X               Y               Z
      1  N         1.9958827823    0.1540452136   -1.6743803726
      2  C         2.3306280572   -0.7008147914   -2.6800402092
      3  C         2.4339544358   -0.1853338281   -3.9666625635
      4  C         2.2118752963    1.1561197669   -4.2222416059
      5  C         1.8723723924    2.0039904347   -3.1691355170
      6  C         1.7722712945    1.4721586953   -1.9125230556
      7  C         2.5972437687   -2.1483960994   -2.4039139409
      8  C         1.8940164518   -0.3024206281   -0.2583422226
      9  N        -1.6970460722   -0.9105138347    0.6034559925
     10  C        -2.0549260303    0.2150154065    1.2889896500
```

| | | | | |
|---|---|---|---|---|
| 11 | N | -1.1657247934 | 1.1345122126 | 1.5470366719 |
| 12 | C | 0.1105990564 | 0.9894423989 | 1.1426944749 |
| 13 | C | 0.5118150758 | -0.1234722260 | 0.3252405877 |
| 14 | C | -0.4477627889 | -1.0695575569 | 0.1169746019 |
| 15 | C | -3.4596828992 | 0.3271939060 | 1.7657568069 |
| 16 | C | -3.7321796390 | -0.5896607042 | 2.9833764457 |
| 17 | C | -2.8846925660 | -0.2478195832 | 4.2029973209 |
| 18 | N | 0.9413997673 | 1.9531907507 | 1.5300574460 |
| 19 | H | 2.7005014870 | -0.8603468094 | -4.7693664588 |
| 20 | H | 2.3047346499 | 1.5416205391 | -5.2308501794 |
| 21 | H | 1.6948327371 | 3.0605660706 | -3.3186446594 |
| 22 | H | 1.5144689166 | 2.0773730457 | -1.0532962512 |
| 23 | H | 2.8065693016 | -2.6606753103 | -3.3409940664 |
| 24 | H | 1.7411984775 | -2.6477614938 | -1.9434532476 |
| 25 | H | 3.4691294568 | -2.2804975996 | -1.7571364787 |
| 26 | H | 2.6576099574 | 0.2358650479 | 0.3041031037 |
| 27 | H | 2.1779805924 | -1.3488252476 | -0.2159796390 |
| 28 | H | -2.3921037108 | -1.6317987692 | 0.4511798430 |
| 29 | H | -0.2630053467 | -1.9804981454 | -0.4401837907 |
| 30 | H | -3.6282968870 | 1.3697146307 | 2.0360471490 |
| 31 | H | -4.1384739712 | 0.0705252313 | 0.9461740516 |
| 32 | H | -3.5925222933 | -1.6394950697 | 2.7002501308 |
| 33 | H | -4.7926226898 | -0.4816713279 | 3.2188219969 |
| 34 | H | -1.8161697249 | -0.4056490115 | 4.0240656238 |
| 35 | H | -3.1683829951 | -0.8753406547 | 5.0486798502 |
| 36 | H | -3.0251942162 | 0.7943332120 | 4.4999438529 |
| 37 | H | 1.9426407876 | 1.8844806816 | 1.4549229544 |
| 38 | H | 0.5770618818 | 2.6504014470 | 2.1663757039 |

Point Group: c1    Number of degrees of freedom:   108

Energy is   -763.966582164

Hessian updated using BFGS update
internal optimization (0)

108 Hessian modes will be used to form the next step
Hessian Eigenvalues:

| | | | | | |
|---|---|---|---|---|---|
| 0.001682 | 0.002668 | 0.004101 | 0.005538 | 0.006107 | 0.012804 |
| 0.015104 | 0.018768 | 0.019619 | 0.019949 | 0.021707 | 0.022351 |
| 0.022662 | 0.022842 | 0.024437 | 0.024583 | 0.025825 | 0.026758 |
| 0.026985 | 0.028188 | 0.028383 | 0.030111 | 0.032035 | 0.036858 |
| 0.039061 | 0.041577 | 0.043236 | 0.043836 | 0.045641 | 0.046100 |
| 0.051030 | 0.053486 | 0.053889 | 0.055541 | 0.064266 | 0.079473 |
| 0.085545 | 0.092035 | 0.121759 | 0.122340 | 0.127120 | 0.130603 |
| 0.131934 | 0.132873 | 0.138580 | 0.141512 | 0.143169 | 0.144588 |
| 0.147094 | 0.147680 | 0.149735 | 0.151896 | 0.152430 | 0.153119 |
| 0.154898 | 0.157950 | 0.193084 | 0.206338 | 0.209642 | 0.215936 |
| 0.226365 | 0.227379 | 0.239410 | 0.246273 | 0.249994 | 0.267389 |
| 0.269478 | 0.273468 | 0.286199 | 0.291445 | 0.300359 | 0.300500 |
| 0.300774 | 0.301458 | 0.301877 | 0.303119 | 0.303999 | 0.304718 |
| 0.305179 | 0.305239 | 0.306152 | 0.309130 | 0.311893 | 0.316273 |
| 0.318728 | 0.332291 | 0.333332 | 0.334129 | 0.340420 | 0.343226 |
| 0.349349 | 0.352452 | 0.362764 | 0.364561 | 0.382036 | 0.385407 |
| 0.395862 | 0.403944 | 0.409774 | 0.415727 | 0.417754 | 0.425724 |
| 0.437687 | 0.447079 | 0.464324 | 0.509819 | 0.580911 | 0.757799 |

Minimum search - taking simple RFO step
Searching for Lamda that Minimizes Along All modes
Value Taken    Lamda =  -0.00019966
Step Taken.  Stepsize is  0.147909

| | Maximum | Tolerance | Cnvgd? |
|---|---|---|---|
| Gradient | 0.001843 | 0.000800 | NO |
| Displacement | 0.048018 | 0.001400 | NO |
| Energy change | -0.000458 | 0.000228 | NO |

New Cartesian Coordinates Obtained by Inverse Iteration

```
 Displacement from previous Coordinates is:  0.547294
 ----------------------------------------------------------------
            Standard Nuclear Orientation (Angstroms)
    I     Atom           X                Y                Z
 ----------------------------------------------------------------
    1      N       1.9822423354     0.1447834627    -1.6836652222
    2      C       2.3557048427    -0.7047021348    -2.6805762095
    3      C       2.4617285091    -0.1920825350    -3.9693949015
    4      C       2.2056561587     1.1409089187    -4.2360144159
    5      C       1.8294948593     1.9832630797    -3.1911930092
    6      C       1.7267937886     1.4550841996    -1.9321038299
    7      C       2.6575644242    -2.1434844329    -2.3946727478
    8      C       1.8772816347    -0.3081995969    -0.2664123829
    9      N      -1.7160930569    -0.8809528675     0.6016680025
   10      C      -2.0570082643     0.2394164118     1.3036732099
   11      N      -1.1577929411     1.1459162309     1.5682382214
   12      C       0.1143506014     0.9912857220     1.1537664785
   13      C       0.4988187855    -0.1150590813     0.3205856945
   14      C      -0.4716061163    -1.0472908686     0.1063524690
   15      C      -3.4591378472     0.3620767159     1.7866982841
   16      C      -3.7439659050    -0.5775467408     2.9832638700
   17      C      -2.8820705075    -0.2813689994     4.2047692471
   18      N       0.9600173370     1.9337918869     1.5579238994
   19      H       2.7579745561    -0.8632795741    -4.7650618674
   20      H       2.3009508892     1.5239877878    -5.2452211719
   21      H       1.6250581462     3.0340089802    -3.3485810370
   22      H       1.4427744003     2.0593209531    -1.0806512234
   23      H       2.9088144766    -2.6502455431    -3.3244235821
   24      H       1.8021538810    -2.6662984832    -1.9591296051
   25      H       3.5131377707    -2.2501513723    -1.7221066288
   26      H       2.6493001663     0.2196905650     0.2949151212
   27      H       2.1464409181    -1.3584896142    -0.2234949774
   28      H      -2.4203037607    -1.5924261080     0.4446434028
   29      H      -0.2993942832    -1.9537657943    -0.4620473254
   30      H      -3.6117533623     1.4006907125     2.0805109074
   31      H      -4.1427148353     0.1349347868     0.9621977184
   32      H      -3.6291805285    -1.6234444748     2.6755983848
   33      H      -4.8002319476    -0.4519774213     3.2287819174
   34      H      -1.8182324901    -0.4572918888     4.0144234189
   35      H      -3.1730322040    -0.9221857091     5.0379380634
   36      H      -2.9985892575     0.7564262803     4.5263405401
   37      H       1.9579607740     1.8741680847     1.4422598874
   38      H       0.6068880527     2.6404884616     2.1902013993
 ----------------------------------------------------------------
 Nuclear Repulsion Energy =        1304.62902168 hartrees
 There are       65 alpha and       65 beta electrons

--------------------------------------------------
-  Entering fldman on Sun Oct 13 13:57:39 2024  -
--------------------------------------------------

 Applying Cartesian multipole field
    Component          Value
    ---------          -----
     (2,0,0)        1.00000E-11
     (0,2,0)        2.00000E-11
     (0,0,2)       -3.00000E-12
 Nucleus-field energy     =     0.0000000286 hartrees

--------------------------------------------------
-  Entering gesman on Sun Oct 13 13:57:39 2024  -
--------------------------------------------------

 Requested basis set is 6-311+G(d,p)
 There are 188 shells and 516 basis functions
 A cutoff of  1.0D-12 yielded  12614 shell pairs
 There are    100671 function pairs (    107308 Cartesian)
 Smallest overlap matrix eigenvalue = 2.07E-06
 Linear dependence detected in AO basis
 Tighter screening thresholds may be required for diffuse basis sets
```

```
 Use S2THRESH > 12 and THRESH = 14 in case of SCF convergence issues
 Number of orthogonalized atomic orbitals = 510
 Maximum deviation from orthogonality = 3.015E-11
 Guess MOs from SCF MO coefficient file
 Reading MOs from coefficient file
 Reading MOs from coefficient file

-------------------------------------------------
-  Entering scfman on Sun Oct 13 13:57:39 2024  -
-------------------------------------------------

 Long-range K will be added via erf
 Coulomb attenuation parameter = 0.2 bohr**(-1)
 A restricted hybrid HF-DFT SCF calculation will be
 performed using Pulay DIIS + Geometric Direct Minimization
 Exchange:     0.2220 Hartree-Fock + 1.0000 wB97X-D + LR-HF
 Correlation:  1.0000 wB97X-D
 Using Euler-Maclaurin-Lebedev (75,302) quadrature formula
 Dispersion:   Grimme D
 SCF converges when RMS gradient is below 1.0E-07
 Geometry optimization detected.  Setting ReadMinima to 0
 Setting SaveMinima to 0
 ---------------------------------------
  Cycle       Energy         DIIS Error
 ---------------------------------------
    1    -763.9532796658      4.78E-04
    2    -763.9664246424      6.11E-05
    3    -763.9666798779      3.45E-05
    4    -763.9667165798      1.89E-05
    5    -763.9667294893      5.63E-06
    6    -763.9667307270      1.59E-06
    7    -763.9667308323      5.30E-07
    8    -763.9667308471      2.39E-07
    9    -763.9667308494      6.51E-08 Convergence criterion met
 ---------------------------------------
 SCF time:  CPU 346.00 s  wall 591.65 s
 SCF   energy in the final basis set = -763.96673085
 Total energy in the final basis set = -763.96673085

-------------------------------------------------
-  Entering anlman on Sun Oct 13 14:07:31 2024  -
-------------------------------------------------


 --------------------------------------------------------------

                   Orbital Energies (a.u.)
 --------------------------------------------------------------

 Alpha MOs
 -- Occupied --
-14.8145 -14.7899 -14.7402 -14.7080 -10.6786 -10.6621 -10.6489 -10.6393
-10.6348 -10.6249 -10.6051 -10.5825 -10.5723 -10.5696 -10.5488 -10.5252
-10.4979 -10.4617  -1.4158  -1.4029  -1.3496  -1.2769  -1.2301  -1.1994
 -1.1869  -1.1419  -1.0995  -1.0828  -1.0570  -1.0529  -1.0297  -1.0046
 -0.9894  -0.9542  -0.9445  -0.9201  -0.9088  -0.8903  -0.8834  -0.8658
 -0.8517  -0.8436  -0.8347  -0.8196  -0.8130  -0.7952  -0.7887  -0.7809
 -0.7742  -0.7579  -0.7493  -0.7483  -0.7450  -0.7275  -0.7221  -0.7156
 -0.6939  -0.6659  -0.6569  -0.6477  -0.6333  -0.6240  -0.6227  -0.6202
 -0.6101
 -- Virtual --
 -0.2708  -0.2595  -0.2450  -0.2245  -0.1743  -0.1648  -0.1467  -0.1427
 -0.1341  -0.1255  -0.1210  -0.1130  -0.1081  -0.1042  -0.0988  -0.0896
 -0.0856  -0.0834  -0.0809  -0.0769  -0.0765  -0.0722  -0.0709  -0.0644
 -0.0628  -0.0601  -0.0550  -0.0512  -0.0499  -0.0441  -0.0398  -0.0351
 -0.0321  -0.0267  -0.0255  -0.0236  -0.0199  -0.0183  -0.0128  -0.0081
 -0.0065  -0.0041   0.0020   0.0040   0.0070   0.0114   0.0136   0.0147
  0.0193   0.0223   0.0263   0.0296   0.0332   0.0369   0.0376   0.0408
  0.0417   0.0433   0.0487   0.0538   0.0550   0.0612   0.0622   0.0666
  0.0710   0.0750   0.0765   0.0781   0.0796   0.0835   0.0858   0.0911
```

| | | | | | | | |
|---|---|---|---|---|---|---|---|
| 0.0924 | 0.0971 | 0.1015 | 0.1024 | 0.1052 | 0.1086 | 0.1110 | 0.1164 |
| 0.1199 | 0.1231 | 0.1277 | 0.1297 | 0.1367 | 0.1414 | 0.1428 | 0.1453 |
| 0.1506 | 0.1580 | 0.1608 | 0.1649 | 0.1747 | 0.1751 | 0.1785 | 0.1800 |
| 0.1822 | 0.1837 | 0.1892 | 0.1920 | 0.1973 | 0.2039 | 0.2139 | 0.2195 |
| 0.2211 | 0.2358 | 0.2465 | 0.2647 | 0.2766 | 0.2861 | 0.3115 | 0.3178 |
| 0.3253 | 0.3303 | 0.3384 | 0.3413 | 0.3464 | 0.3558 | 0.3602 | 0.3634 |
| 0.3760 | 0.3799 | 0.3848 | 0.3936 | 0.3994 | 0.4044 | 0.4114 | 0.4245 |
| 0.4303 | 0.4316 | 0.4405 | 0.4434 | 0.4505 | 0.4568 | 0.4664 | 0.4737 |
| 0.4829 | 0.4911 | 0.4956 | 0.5006 | 0.5101 | 0.5133 | 0.5194 | 0.5245 |
| 0.5283 | 0.5311 | 0.5324 | 0.5371 | 0.5501 | 0.5538 | 0.5546 | 0.5620 |
| 0.5636 | 0.5679 | 0.5700 | 0.5831 | 0.5871 | 0.5946 | 0.5970 | 0.6065 |
| 0.6159 | 0.6172 | 0.6267 | 0.6312 | 0.6395 | 0.6419 | 0.6538 | 0.6563 |
| 0.6613 | 0.6763 | 0.6817 | 0.6865 | 0.6917 | 0.6973 | 0.7010 | 0.7037 |
| 0.7163 | 0.7267 | 0.7332 | 0.7473 | 0.7569 | 0.7615 | 0.7695 | 0.7821 |
| 0.7921 | 0.8047 | 0.8096 | 0.8139 | 0.8381 | 0.8460 | 0.8499 | 0.8547 |
| 0.8649 | 0.8745 | 0.8808 | 0.9064 | 0.9177 | 0.9189 | 0.9241 | 0.9354 |
| 0.9426 | 0.9493 | 0.9701 | 0.9768 | 0.9851 | 0.9935 | 1.0088 | 1.0452 |
| 1.0568 | 1.0611 | 1.0803 | 1.0838 | 1.1202 | 1.1638 | 1.1762 | 1.1832 |
| 1.1996 | 1.2057 | 1.2099 | 1.2330 | 1.2545 | 1.2735 | 1.2945 | 1.3010 |
| 1.3106 | 1.3234 | 1.3279 | 1.3367 | 1.3455 | 1.3526 | 1.3693 | 1.3792 |
| 1.3822 | 1.3908 | 1.4006 | 1.4038 | 1.4110 | 1.4171 | 1.4234 | 1.4354 |
| 1.4402 | 1.4437 | 1.4550 | 1.4617 | 1.4731 | 1.4822 | 1.4838 | 1.4874 |
| 1.4928 | 1.5041 | 1.5159 | 1.5238 | 1.5337 | 1.5431 | 1.5514 | 1.5551 |
| 1.5661 | 1.5725 | 1.5759 | 1.5774 | 1.5865 | 1.6029 | 1.6145 | 1.6205 |
| 1.6285 | 1.6326 | 1.6447 | 1.6487 | 1.6621 | 1.6665 | 1.6776 | 1.6836 |
| 1.6932 | 1.7019 | 1.7157 | 1.7332 | 1.7417 | 1.7516 | 1.7611 | 1.7680 |
| 1.7801 | 1.7893 | 1.8036 | 1.8227 | 1.8346 | 1.8426 | 1.8663 | 1.8782 |
| 1.8914 | 1.9016 | 1.9183 | 1.9303 | 1.9479 | 1.9658 | 1.9731 | 1.9874 |
| 1.9957 | 2.0182 | 2.0273 | 2.0333 | 2.0519 | 2.0671 | 2.0818 | 2.0971 |
| 2.1057 | 2.1155 | 2.1320 | 2.1412 | 2.1689 | 2.1958 | 2.2245 | 2.2281 |
| 2.2297 | 2.2375 | 2.2524 | 2.2544 | 2.2728 | 2.2783 | 2.2809 | 2.2917 |
| 2.3009 | 2.3080 | 2.3426 | 2.3576 | 2.3691 | 2.3758 | 2.3875 | 2.3972 |
| 2.4089 | 2.4216 | 2.4325 | 2.4337 | 2.4472 | 2.4600 | 2.4683 | 2.4880 |
| 2.4931 | 2.5181 | 2.5234 | 2.5361 | 2.5402 | 2.5487 | 2.5542 | 2.5564 |
| 2.5668 | 2.5707 | 2.5774 | 2.5847 | 2.5913 | 2.6028 | 2.6144 | 2.6233 |
| 2.6304 | 2.6418 | 2.6560 | 2.6670 | 2.6733 | 2.6846 | 2.6934 | 2.6956 |
| 2.7108 | 2.7308 | 2.7426 | 2.7534 | 2.7544 | 2.7603 | 2.7813 | 2.7859 |
| 2.7917 | 2.7960 | 2.8190 | 2.8326 | 2.8506 | 2.8626 | 2.8773 | 2.8898 |
| 2.9013 | 2.9260 | 2.9644 | 2.9829 | 3.0513 | 3.0669 | 3.1211 | 3.1428 |
| 3.1688 | 3.2460 | 3.2519 | 3.2686 | 3.2978 | 3.3326 | 3.3676 | 3.4110 |
| 3.4175 | 3.4755 | 3.5120 | 3.5589 | 3.5625 | 3.5930 | 3.6330 | 3.6430 |
| 3.6633 | 3.7009 | 3.7217 | 3.7234 | 3.7384 | 3.7515 | 3.7671 | 3.7698 |
| 3.7771 | 3.8409 | 3.8727 | 3.9095 | 3.9358 | 3.9797 | 4.0302 | 4.0866 |
| 4.1100 | 4.2059 | 4.2951 | 4.3030 | 4.5940 | 4.6674 | 4.7074 | 4.7192 |
| 4.9221 | 5.0438 | 5.1971 | 23.5239 | 23.5726 | 23.7206 | 23.7283 | 23.7361 |
| 23.7726 | 23.7973 | 23.8242 | 23.8597 | 23.8850 | 23.9232 | 23.9682 | 23.9794 |
| 24.0367 | 35.4242 | 35.4567 | 35.5203 | 35.6180 | | | |

Ground-State Mulliken Net Atomic Charges

| Atom | Charge (a.u.) |
|---|---|
| 1 N | 0.420680 |
| 2 C | 0.133511 |
| 3 C | -0.012149 |
| 4 C | -0.149504 |
| 5 C | -0.183435 |
| 6 C | -0.182071 |
| 7 C | -0.791514 |
| 8 C | -0.316440 |
| 9 N | -0.112434 |
| 10 C | -0.263360 |
| 11 N | 0.008956 |
| 12 C | 0.253471 |
| 13 C | 0.008607 |
| 14 C | 0.090766 |
| 15 C | -0.401475 |
| 16 C | -0.225272 |
| 17 C | -0.507721 |

```
   18 N                      -0.483651
   19 H                       0.219587
   20 H                       0.234688
   21 H                       0.227262
   22 H                       0.203136
   23 H                       0.229813
   24 H                       0.190860
   25 H                       0.245880
   26 H                       0.230178
   27 H                       0.242455
   28 H                       0.383272
   29 H                       0.203592
   30 H                       0.243676
   31 H                       0.232176
   32 H                       0.167403
   33 H                       0.225309
   34 H                       0.140111
   35 H                       0.207988
   36 H                       0.190559
   37 H                       0.317388
   38 H                       0.377701
 ----------------------------------------
 Sum of atomic charges =     2.000000

 -----------------------------------------------------------------
                    Cartesian Multipole Moments
 -----------------------------------------------------------------
    Charge (ESU x 10^10)
                 9.6064
    Dipole Moment (Debye)
         X       4.9015      Y       0.1047      Z      -5.8532
       Tot       7.6351
    Quadrupole Moments (Debye-Ang)
        XX     -44.2714     XY       7.9648     YY     -71.4823
        XZ     -41.5219     YZ       1.7354     ZZ     -36.4808
    Traceless Quadrupole Moments (Debye-Ang)
       QXX      19.4203    QYY     -62.2125    QZZ      42.7921
       QXY      23.8945    QXZ    -124.5658    QYZ       5.2061
    Octopole Moments (Debye-Ang^2)
       XXX     -74.7332    XXY     -35.2609    XYY      23.8946
       YYY     -24.2712    XXZ      16.7070    XYZ      14.2657
       YYZ     -12.3810    XZZ      68.6166    YZZ      22.0718
       ZZZ    -106.6280
    Traceless Octopole Moments (Debye-Ang^2)
       XXX   -1280.9992    YYY     -26.9246    ZZZ    -678.7018
       XXY    -416.5330    XXZ     557.5109    XYY     305.0847
       XYZ     213.9851    XZZ     975.9145    YYZ     121.1909
       YZZ     443.4576
    Hexadecapole Moments (Debye-Ang^3)
      XXXX   -2566.9428   XXXY     -90.6178   XXYY    -552.7991
      XYYY     -25.7215   YYYY    -769.2614   XXXZ    1038.6064
      XXYZ      46.8225   XYYZ     371.2721   YYYZ      50.3439
      XXZZ    -676.8179   XYZZ      36.2533   YYZZ    -564.4619
      XZZZ     679.6137   YZZZ     -93.2052   ZZZZ   -2550.4077
    Traceless Hexadecapole Moments (Debye-Ang^3)
      XXXX  -13111.5348   XXXY   -5911.0049   XXXZ   15026.5242
      XXYY   -1221.9848   XXYZ    4853.8647   XXZZ   14333.5196
      XYYY     903.1146   XYYZ    7641.1837   XYZZ    5007.8902
      XZZZ  -22667.7080   YYYY    3741.6426   YYYZ    5098.6229
      YYZZ   -2519.6578   YZZZ   -9952.4876   ZZZZ  -11813.8618
 -----------------------------------------------------------------

-------------------------------------------------
-  Entering drvman on Sun Oct 13 14:07:31 2024  -
-------------------------------------------------

 Calculating analytic gradient of the SCF energy
 Gradient of SCF Energy
            1            2            3            4            5            6
    1   0.0003594   -0.0000656   -0.0001143    0.0000846    0.0000637   -0.0000739
```

```
   2   0.0000268  -0.0003475   0.0006053  -0.0000433   0.0000453  -0.0001302
   3   0.0000770   0.0003889  -0.0003284  -0.0003076  -0.0004920   0.0005721
           7           8           9          10          11          12
   1  -0.0001943  -0.0000534   0.0000203   0.0002301  -0.0003213   0.0001840
   2  -0.0000779  -0.0000135  -0.0003600   0.0009595  -0.0006429   0.0011117
   3   0.0000545  -0.0000226  -0.0000746   0.0006064  -0.0005416   0.0001507
          13          14          15          16          17          18
   1   0.0000730   0.0002580  -0.0000803   0.0001336   0.0000130  -0.0009370
   2  -0.0010081   0.0004680   0.0000038  -0.0001818   0.0000647  -0.0003349
   3  -0.0002134   0.0001693  -0.0003737   0.0002366  -0.0000082   0.0001929
          19          20          21          22          23          24
   1  -0.0000563   0.0000280   0.0000343   0.0000069   0.0000214  -0.0001500
   2  -0.0000233  -0.0000327   0.0000404  -0.0000533   0.0000465   0.0000169
   3   0.0000275   0.0000761   0.0000455   0.0000092  -0.0000116   0.0000794
          25          26          27          28          29          30
   1  -0.0000078   0.0001396  -0.0001248   0.0001488   0.0002286   0.0000459
   2  -0.0000275  -0.0000196   0.0000789  -0.0000413   0.0000077   0.0000049
   3  -0.0000254  -0.0001959   0.0000636  -0.0000166  -0.0000553   0.0000064
          31          32          33          34          35          36
   1   0.0000572  -0.0000719   0.0000145  -0.0000188  -0.0000028  -0.0000153
   2  -0.0000674   0.0000281   0.0000436   0.0000394   0.0000330   0.0000089
   3   0.0000138  -0.0000273  -0.0000784   0.0000222  -0.0000028   0.0000027
          37          38
   1  -0.0001109   0.0002541
   2   0.0000325  -0.0002607
   3  -0.0002968   0.0002773
 Max gradient component =       1.112E-03
 RMS gradient           =       2.723E-04
 Gradient time:  CPU 170.17 s  wall 357.07 s

-------------------------------------------------
-  Entering optman on Sun Oct 13 14:13:28 2024  -
-------------------------------------------------

 Geometry Optimization Parameters
   NAtoms,    NIC,     NZ,  NCons,   NDum,   NFix, NCnnct, MaxDiis
       38     272       0       0       0       0       0       0

 Cartesian Hessian Update
 Hessian updated using BFGS update


** GEOMETRY OPTIMIZATION IN DELOCALIZED INTERNAL COORDINATES **
   Searching for a Minimum

   Optimization Cycle:   8

                       Coordinates (Angstroms)
     ATOM                X               Y               Z
      1  N         1.9822423354    0.1447834627   -1.6836652222
      2  C         2.3557048427   -0.7047021348   -2.6805762095
      3  C         2.4617285091   -0.1920825350   -3.9693949015
      4  C         2.2056561587    1.1409089187   -4.2360144159
      5  C         1.8294948593    1.9832630797   -3.1911930092
      6  C         1.7267937886    1.4550841996   -1.9321038299
      7  C         2.6575644242   -2.1434844329   -2.3946727478
      8  C         1.8772816347   -0.3081995969   -0.2664123829
      9  N        -1.7160930569   -0.8809528675    0.6016680025
     10  C        -2.0570082643    0.2394164118    1.3036732099
     11  N        -1.1577929411    1.1459162309    1.5682382214
     12  C         0.1143506014    0.9912857220    1.1537664785
     13  C         0.4988187855   -0.1150590813    0.3205856945
     14  C        -0.4716061163   -1.0472908686    0.1063524690
     15  C        -3.4591378472    0.3620767159    1.7866982841
     16  C        -3.7439659050   -0.5775467408    2.9832638700
     17  C        -2.8820705075   -0.2813689994    4.2047692471
     18  N         0.9600173370    1.9337918869    1.5579238994
     19  H         2.7579745561   -0.8632795741   -4.7650618674
     20  H         2.3009508892    1.5239877878   -5.2452211719
     21  H         1.6250581462    3.0340089802   -3.3485810370
```

```
    22  H          1.4427744003    2.0593209531   -1.0806512234
    23  H          2.9088144766   -2.6502455431   -3.3244235821
    24  H          1.8021538810   -2.6662984832   -1.9591296051
    25  H          3.5131377707   -2.2501513723   -1.7221066288
    26  H          2.6493001663    0.2196905650    0.2949151212
    27  H          2.1464409181   -1.3584896142   -0.2234949774
    28  H         -2.4203037607   -1.5924261080    0.4446434028
    29  H         -0.2993942832   -1.9537657943   -0.4620473254
    30  H         -3.6117533623    1.4006907125    2.0805109074
    31  H         -4.1427148353    0.1349347868    0.9621977184
    32  H         -3.6291805285   -1.6234444748    2.6755983848
    33  H         -4.8002319476   -0.4519774213    3.2287819174
    34  H         -1.8182324901   -0.4572918888    4.0144234189
    35  H         -3.1730322040   -0.9221857091    5.0379380634
    36  H         -2.9985892575    0.7564262803    4.5263405401
    37  H          1.9579607740    1.8741680847    1.4422598874
    38  H          0.6068880527    2.6404884616    2.1902013993
  Point Group: c1    Number of degrees of freedom:   108


  Energy is   -763.966730849

 Hessian updated using BFGS update
  internal optimization (0)

108 Hessian modes will be used to form the next step
  Hessian Eigenvalues:
     0.001701    0.002668    0.003368    0.004121    0.005957    0.013046
     0.015216    0.018367    0.019377    0.019928    0.020968    0.021869
     0.022703    0.022931    0.023716    0.024521    0.025861    0.026046
     0.027141    0.028160    0.028388    0.030138    0.031599    0.036626
     0.039135    0.041548    0.043213    0.043847    0.044819    0.045799
     0.050997    0.053633    0.054283    0.055565    0.064125    0.079380
     0.085545    0.091983    0.121762    0.122344    0.127122    0.130589
     0.132189    0.132884    0.139227    0.141528    0.142938    0.144503
     0.146077    0.147677    0.149182    0.150194    0.152469    0.152593
     0.156446    0.162940    0.194334    0.206402    0.209366    0.215974
     0.226770    0.227143    0.239514    0.246192    0.249196    0.264613
     0.268856    0.271839    0.286208    0.290685    0.300338    0.300476
     0.300771    0.301462    0.301861    0.303127    0.303985    0.304718
     0.305161    0.305252    0.306137    0.308724    0.312341    0.316244
     0.319493    0.332764    0.334013    0.334135    0.340652    0.343217
     0.350513    0.352898    0.361290    0.370751    0.382014    0.388999
     0.394962    0.401755    0.408225    0.414711    0.416116    0.420349
     0.442748    0.450706    0.468169    0.508497    0.580676    0.757359

 Minimum search - taking simple RFO step
 Searching for Lamda that Minimizes Along All modes
 Value Taken    Lamda =  -0.00021980
 Step Taken.  Stepsize is  0.203451

                           Maximum     Tolerance    Cnvgd?
         Gradient         0.001719      0.000800      NO
         Displacement     0.079784      0.001400      NO
         Energy change   -0.000149      0.000228     YES


 New Cartesian Coordinates Obtained by Inverse Iteration

 Displacement from previous Coordinates is:  0.914399
 ----------------------------------------------------------------
             Standard Nuclear Orientation (Angstroms)
    I     Atom           X                Y                Z
 ----------------------------------------------------------------
    1      N       1.9578752190     0.1275110436    -1.6989656253
    2      C       2.3957486139    -0.7103794503    -2.6799674334
    3      C       2.5089068870    -0.2037180321    -3.9693774418
    4      C       2.1964280455     1.1142177603    -4.2521326919
    5      C       1.7589339353     1.9468360446    -3.2235504466
    6      C       1.6515809360     1.4235183093    -1.9642211780
```

```
    7      C        2.7585098158    -2.1313141543    -2.3757014881
    8      C        1.8479757072    -0.3200427989    -0.2796676560
    9      N       -1.7531363218    -0.8295025923     0.5946181741
   10      C       -2.0635310766     0.2813439591     1.3243798014
   11      N       -1.1435797847     1.1649663845     1.6006135125
   12      C        0.1212680776     0.9927818994     1.1695005527
   13      C        0.4753251334    -0.1041292910     0.3128725118
   14      C       -0.5162801938    -1.0122789142     0.0862047799
   15      C       -3.4586222909     0.4218446719     1.8248461230
   16      C       -3.7613155370    -0.5569750924     2.9838774964
   17      C       -2.8744211980    -0.3403144962     4.2042432075
   18      N        0.9912423465     1.9078673969     1.5856572176
   19      H        2.8558230943    -0.8650097149    -4.7527751877
   20      H        2.2967295912     1.4942098239    -5.2623548916
   21      H        1.5113694538     2.9860040605    -3.3949396930
   22      H        1.3246113135     2.0227233140    -1.1248385625
   23      H        3.0603146075    -2.6312147725    -3.2941605466
   24      H        1.9150415671    -2.6916384239    -1.9633556836
   25      H        3.5967497236    -2.1936422622    -1.6764649919
   26      H        2.6307758155     0.1941440562     0.2800029785
   27      H        2.0968602769    -1.3753422565    -0.2348260226
   28      H       -2.4745389336    -1.5218202772     0.4290113446
   29      H       -0.3677477206    -1.9102536230    -0.5021389166
   30      H       -3.5811563393     1.4514741353     2.1611959348
   31      H       -4.1534812250     0.2478459309     0.9965839969
   32      H       -3.6855521359    -1.5927374711     2.6327456542
   33      H       -4.8094963480    -0.4070414720     3.2498592972
   34      H       -1.8196386899    -0.5434425401     3.9910107608
   35      H       -3.1755236705    -1.0049890522     5.0148430015
   36      H       -2.9514105572     0.6867493734     4.5693425612
   37      H        1.9837760253     1.8517459779     1.4287197226
   38      H        0.6535858373     2.6300025458     2.2093098278
 ----------------------------------------------------------------
 Nuclear Repulsion Energy =        1303.69034395 hartrees
 There are       65 alpha and       65 beta electrons

--------------------------------------------------
-  Entering fldman on Sun Oct 13 14:13:28 2024  -
--------------------------------------------------

 Applying Cartesian multipole field
    Component          Value
    ---------          -----
      (2,0,0)        1.00000E-11
      (0,2,0)        2.00000E-11
      (0,0,2)       -3.00000E-12
 Nucleus-field energy     =     0.0000000284 hartrees

--------------------------------------------------
-  Entering gesman on Sun Oct 13 14:13:28 2024  -
--------------------------------------------------

 Requested basis set is 6-311+G(d,p)
 There are 188 shells and 516 basis functions
 A cutoff of  1.0D-12 yielded  12596 shell pairs
 There are    100554 function pairs (    107186 Cartesian)
 Smallest overlap matrix eigenvalue = 1.99E-06
 Linear dependence detected in AO basis
 Tighter screening thresholds may be required for diffuse basis sets
 Use S2THRESH > 12 and THRESH = 14 in case of SCF convergence issues
 Number of orthogonalized atomic orbitals = 510
 Maximum deviation from orthogonality = 1.962E-11
 Guess MOs from SCF MO coefficient file
 Reading MOs from coefficient file
 Reading MOs from coefficient file

--------------------------------------------------
-  Entering scfman on Sun Oct 13 14:13:28 2024  -
--------------------------------------------------
```

```
 Long-range K will be added via erf
 Coulomb attenuation parameter = 0.2 bohr**(-1)
 A restricted hybrid HF-DFT SCF calculation will be
 performed using Pulay DIIS + Geometric Direct Minimization
 Exchange:     0.2220 Hartree-Fock + 1.0000 wB97X-D + LR-HF
 Correlation:  1.0000 wB97X-D
 Using Euler-Maclaurin-Lebedev (75,302) quadrature formula
 Dispersion:   Grimme D
 SCF converges when RMS gradient is below 1.0E-07
 Geometry optimization detected.  Setting ReadMinima to 0
 Setting SaveMinima to 0
 ---------------------------------------
  Cycle       Energy         DIIS Error
 ---------------------------------------
    1    -763.9313907920      7.84E-04
    2    -763.9660559270      1.01E-04
    3    -763.9667413477      5.97E-05
    4    -763.9668497720      3.04E-05
    5    -763.9668826548      9.99E-06
    6    -763.9668865342      2.59E-06
    7    -763.9668868279      9.04E-07
    8    -763.9668868704      4.21E-07
    9    -763.9668868777      1.10E-07
   10    -763.9668868783      4.98E-08 Convergence criterion met
 ---------------------------------------
 SCF time:  CPU 369.92 s  wall 762.56 s
 SCF   energy in the final basis set = -763.96688688
 Total energy in the final basis set = -763.96688688

-------------------------------------------------
-  Entering anlman on Sun Oct 13 14:26:11 2024  -
-------------------------------------------------


 --------------------------------------------------------------

                   Orbital Energies (a.u.)
 --------------------------------------------------------------

 Alpha MOs
 -- Occupied --
-14.8146 -14.7899 -14.7401 -14.7079 -10.6782 -10.6619 -10.6490 -10.6391
-10.6349 -10.6249 -10.6050 -10.5826 -10.5722 -10.5693 -10.5479 -10.5250
-10.4978 -10.4618  -1.4155  -1.4027  -1.3492  -1.2763  -1.2300  -1.1994
 -1.1868  -1.1418  -1.0994  -1.0824  -1.0569  -1.0528  -1.0293  -1.0044
 -0.9893  -0.9542  -0.9443  -0.9201  -0.9089  -0.8899  -0.8834  -0.8654
 -0.8515  -0.8433  -0.8354  -0.8194  -0.8123  -0.7959  -0.7884  -0.7805
 -0.7735  -0.7581  -0.7493  -0.7477  -0.7445  -0.7274  -0.7219  -0.7152
 -0.6936  -0.6657  -0.6565  -0.6476  -0.6329  -0.6241  -0.6228  -0.6204
 -0.6096
 -- Virtual --
 -0.2707  -0.2593  -0.2448  -0.2245  -0.1744  -0.1645  -0.1474  -0.1429
 -0.1330  -0.1258  -0.1210  -0.1125  -0.1079  -0.1042  -0.0985  -0.0896
 -0.0853  -0.0835  -0.0816  -0.0768  -0.0758  -0.0723  -0.0705  -0.0646
 -0.0629  -0.0595  -0.0550  -0.0510  -0.0500  -0.0440  -0.0394  -0.0352
 -0.0320  -0.0276  -0.0255  -0.0238  -0.0199  -0.0180  -0.0123  -0.0082
 -0.0065  -0.0040   0.0021   0.0039   0.0078   0.0107   0.0124   0.0148
  0.0183   0.0229   0.0265   0.0294   0.0333   0.0357   0.0384   0.0408
  0.0424   0.0433   0.0484   0.0542   0.0553   0.0618   0.0627   0.0665
  0.0714   0.0752   0.0768   0.0777   0.0798   0.0843   0.0858   0.0909
  0.0931   0.0997   0.1007   0.1034   0.1052   0.1081   0.1118   0.1170
  0.1200   0.1224   0.1281   0.1294   0.1344   0.1420   0.1425   0.1453
  0.1496   0.1572   0.1604   0.1648   0.1739   0.1752   0.1762   0.1808
  0.1823   0.1853   0.1898   0.1903   0.1969   0.2042   0.2133   0.2183
  0.2215   0.2374   0.2479   0.2645   0.2765   0.2864   0.3109   0.3168
  0.3258   0.3305   0.3388   0.3415   0.3475   0.3557   0.3603   0.3636
  0.3754   0.3790   0.3848   0.3937   0.3990   0.4042   0.4154   0.4252
  0.4298   0.4339   0.4382   0.4434   0.4498   0.4563   0.4660   0.4714
  0.4829   0.4900   0.4962   0.4996   0.5097   0.5125   0.5195   0.5248
  0.5284   0.5304   0.5323   0.5357   0.5514   0.5535   0.5575   0.5621
```

| | | | | | | | |
|---|---|---|---|---|---|---|---|
| 0.5639 | 0.5679 | 0.5708 | 0.5829 | 0.5871 | 0.5932 | 0.6000 | 0.6071 |
| 0.6155 | 0.6180 | 0.6273 | 0.6311 | 0.6413 | 0.6449 | 0.6539 | 0.6561 |
| 0.6617 | 0.6773 | 0.6792 | 0.6860 | 0.6907 | 0.6983 | 0.7012 | 0.7053 |
| 0.7148 | 0.7246 | 0.7395 | 0.7466 | 0.7555 | 0.7613 | 0.7698 | 0.7823 |
| 0.7928 | 0.8053 | 0.8106 | 0.8160 | 0.8388 | 0.8465 | 0.8501 | 0.8529 |
| 0.8658 | 0.8730 | 0.8821 | 0.9098 | 0.9172 | 0.9198 | 0.9219 | 0.9385 |
| 0.9407 | 0.9541 | 0.9747 | 0.9778 | 0.9813 | 0.9935 | 1.0096 | 1.0424 |
| 1.0563 | 1.0639 | 1.0771 | 1.0837 | 1.1163 | 1.1547 | 1.1773 | 1.1809 |
| 1.1964 | 1.2044 | 1.2120 | 1.2317 | 1.2542 | 1.2769 | 1.2945 | 1.2977 |
| 1.3106 | 1.3206 | 1.3291 | 1.3363 | 1.3474 | 1.3508 | 1.3689 | 1.3779 |
| 1.3800 | 1.3908 | 1.4019 | 1.4040 | 1.4114 | 1.4187 | 1.4227 | 1.4345 |
| 1.4402 | 1.4456 | 1.4554 | 1.4629 | 1.4729 | 1.4823 | 1.4846 | 1.4885 |
| 1.4950 | 1.5035 | 1.5151 | 1.5248 | 1.5329 | 1.5435 | 1.5496 | 1.5555 |
| 1.5630 | 1.5730 | 1.5759 | 1.5788 | 1.5886 | 1.6031 | 1.6159 | 1.6201 |
| 1.6286 | 1.6334 | 1.6430 | 1.6500 | 1.6611 | 1.6682 | 1.6755 | 1.6829 |
| 1.6948 | 1.7018 | 1.7174 | 1.7321 | 1.7395 | 1.7542 | 1.7587 | 1.7672 |
| 1.7804 | 1.7909 | 1.8006 | 1.8253 | 1.8366 | 1.8410 | 1.8663 | 1.8796 |
| 1.8911 | 1.9028 | 1.9187 | 1.9296 | 1.9520 | 1.9679 | 1.9802 | 1.9869 |
| 1.9966 | 2.0201 | 2.0313 | 2.0315 | 2.0503 | 2.0681 | 2.0811 | 2.0990 |
| 2.1043 | 2.1169 | 2.1323 | 2.1396 | 2.1691 | 2.1980 | 2.2236 | 2.2280 |
| 2.2318 | 2.2394 | 2.2497 | 2.2552 | 2.2719 | 2.2752 | 2.2802 | 2.2919 |
| 2.2998 | 2.3091 | 2.3435 | 2.3568 | 2.3682 | 2.3754 | 2.3878 | 2.3965 |
| 2.4100 | 2.4207 | 2.4315 | 2.4342 | 2.4463 | 2.4567 | 2.4685 | 2.4891 |
| 2.4923 | 2.5184 | 2.5236 | 2.5358 | 2.5430 | 2.5480 | 2.5562 | 2.5569 |
| 2.5670 | 2.5700 | 2.5801 | 2.5839 | 2.5914 | 2.6038 | 2.6144 | 2.6216 |
| 2.6307 | 2.6401 | 2.6564 | 2.6634 | 2.6701 | 2.6847 | 2.6912 | 2.6951 |
| 2.7103 | 2.7325 | 2.7436 | 2.7510 | 2.7547 | 2.7595 | 2.7805 | 2.7847 |
| 2.7916 | 2.7979 | 2.8197 | 2.8318 | 2.8502 | 2.8660 | 2.8789 | 2.8902 |
| 2.8999 | 2.9262 | 2.9645 | 2.9846 | 3.0527 | 3.0685 | 3.1254 | 3.1428 |
| 3.1679 | 3.2468 | 3.2524 | 3.2710 | 3.2978 | 3.3344 | 3.3676 | 3.4143 |
| 3.4179 | 3.4756 | 3.5117 | 3.5589 | 3.5624 | 3.5937 | 3.6292 | 3.6452 |
| 3.6626 | 3.7008 | 3.7220 | 3.7241 | 3.7378 | 3.7428 | 3.7668 | 3.7699 |
| 3.7774 | 3.8417 | 3.8728 | 3.9090 | 3.9354 | 3.9801 | 4.0309 | 4.0848 |
| 4.1105 | 4.2061 | 4.2954 | 4.3043 | 4.5940 | 4.6670 | 4.7068 | 4.7192 |
| 4.9231 | 5.0439 | 5.1983 | 23.5229 | 23.5712 | 23.7198 | 23.7275 | 23.7362 |
| 23.7744 | 23.7966 | 23.8244 | 23.8594 | 23.8857 | 23.9232 | 23.9701 | 23.9777 |
| 24.0383 | 35.4234 | 35.4563 | 35.5204 | 35.6176 | | | |

Ground-State Mulliken Net Atomic Charges

| Atom | Charge (a.u.) |
|---|---|
| 1 N | 0.423791 |
| 2 C | 0.120076 |
| 3 C | -0.005076 |
| 4 C | -0.145723 |
| 5 C | -0.188131 |
| 6 C | -0.181999 |
| 7 C | -0.776467 |
| 8 C | -0.329878 |
| 9 N | -0.104744 |
| 10 C | -0.279085 |
| 11 N | 0.010915 |
| 12 C | 0.269981 |
| 13 C | 0.003038 |
| 14 C | 0.087725 |
| 15 C | -0.405226 |
| 16 C | -0.228816 |
| 17 C | -0.504511 |
| 18 N | -0.482313 |
| 19 H | 0.219868 |
| 20 H | 0.234671 |
| 21 H | 0.227085 |
| 22 H | 0.203175 |
| 23 H | 0.229609 |
| 24 H | 0.194929 |
| 25 H | 0.243774 |
| 26 H | 0.228976 |
| 27 H | 0.243776 |

```
    28 H                     0.381736
    29 H                     0.206579
    30 H                     0.243420
    31 H                     0.233581
    32 H                     0.166352
    33 H                     0.225597
    34 H                     0.140144
    35 H                     0.207967
    36 H                     0.190760
    37 H                     0.317571
    38 H                     0.376873
 ----------------------------------------
 Sum of atomic charges =     2.000000

 -----------------------------------------------------------------
                    Cartesian Multipole Moments
 -----------------------------------------------------------------
    Charge (ESU x 10^10)
                 9.6064
    Dipole Moment (Debye)
         X       4.8218      Y       0.1743      Z      -5.9663
       Tot       7.6731
    Quadrupole Moments (Debye-Ang)
        XX     -43.3129     XY       6.9615     YY     -72.2514
        XZ     -41.8654     YZ       1.8369     ZZ     -36.3199
    Traceless Quadrupole Moments (Debye-Ang)
       QXX      21.9455    QYY     -64.8699    QZZ      42.9245
       QXY      20.8845    QXZ    -125.5963    QYZ       5.5106
    Octopole Moments (Debye-Ang^2)
       XXX     -71.2375    XXY     -37.5591    XYY      23.4017
       YYY     -24.8705    XXZ      14.8704    XYZ      16.0996
       YYZ     -11.5883    XZZ      69.9077    YZZ      22.1292
       ZZZ    -107.5536
    Traceless Octopole Moments (Debye-Ang^2)
       XXX   -1267.2099    YYY     -10.3537    ZZZ    -674.8610
       XXY    -442.4860    XXZ     535.8705    XYY     284.8100
       XYZ     241.4933    XZZ     982.3998    YYZ     138.9905
       YZZ     452.8397
    Hexadecapole Moments (Debye-Ang^3)
      XXXX   -2568.6573   XXXY     -86.4897   XXYY    -554.5409
      XYYY     -15.6019   YYYY    -760.3269   XXXZ    1029.9467
      XXYZ      56.5155   XYYZ     377.8143   YYYZ      48.4556
      XXZZ    -671.6664   XYZZ      35.8216   YYZZ    -568.8135
      XZZZ     677.0322   YZZZ    -101.4600   ZZZZ   -2572.7581
    Traceless Hexadecapole Moments (Debye-Ang^3)
      XXXX  -13597.2572   XXXY   -6099.2723   XXXZ   14328.7090
      XXYY   -1523.9566   XXYZ    5874.5631   XXZZ   15121.2138
      XYYY    1343.9514   XYYZ    8398.5998   XYZZ    4755.3209
      XZZZ  -22727.3088   YYYY    4270.9367   YYYZ    4909.1366
      YYZZ   -2746.9801   YZZZ  -10783.6997   ZZZZ  -12374.2337
 -----------------------------------------------------------------

------------------------------------------------
-  Entering drvman on Sun Oct 13 14:26:11 2024  -
------------------------------------------------

 Calculating analytic gradient of the SCF energy
 Gradient of SCF Energy
            1           2           3           4           5           6
    1   0.0001485  -0.0002616   0.0001489  -0.0001432  -0.0000991   0.0000742
    2  -0.0000198   0.0003594  -0.0005095   0.0000450   0.0000056  -0.0000113
    3   0.0000815  -0.0004149   0.0003113   0.0003645   0.0005910  -0.0007172
            7           8           9          10          11          12
    1   0.0003063  -0.0000336  -0.0000729  -0.0001228   0.0004781  -0.0004700
    2   0.0000014   0.0001654  -0.0001406  -0.0004293   0.0004658  -0.0000856
    3  -0.0001404   0.0000027   0.0000997  -0.0003135  -0.0000120  -0.0007326
           13          14          15          16          17          18
    1   0.0001886  -0.0002011   0.0000082   0.0000318  -0.0000907   0.0002614
    2   0.0003843  -0.0002061  -0.0000982  -0.0001992   0.0002406  -0.0006262
    3   0.0003142  -0.0000527   0.0001111  -0.0001303  -0.0000103   0.0014089
```

```
          19          20          21          22          23          24
    1   0.0000006   0.0000310   0.0000107   0.0001185  -0.0000730  -0.0002341
    2   0.0000337   0.0000670   0.0000342   0.0001458  -0.0001147   0.0000200
    3  -0.0000567  -0.0000884   0.0000058  -0.0001645  -0.0000522   0.0000145
          25          26          27          28          29          30
    1  -0.0000074   0.0001624  -0.0001814  -0.0000289   0.0001357  -0.0000994
    2  -0.0000262   0.0000219   0.0000749  -0.0000752  -0.0000365  -0.0000013
    3  -0.0000443  -0.0000544   0.0002709   0.0000180  -0.0000091   0.0000105
          31          32          33          34          35          36
    1   0.0000808  -0.0000340  -0.0000190  -0.0000109  -0.0000084  -0.0000209
    2  -0.0000464   0.0000253   0.0000588   0.0000162  -0.0000010   0.0000252
    3  -0.0000557   0.0000312   0.0000763  -0.0000167   0.0000280  -0.0000081
          37          38
    1   0.0001865  -0.0001597
    2   0.0000376   0.0003989
    3  -0.0006006  -0.0000653
 Max gradient component =       1.409E-03
 RMS gradient           =       2.549E-04
 Gradient time:  CPU 171.06 s  wall 364.46 s

-------------------------------------------------
-  Entering optman on Sun Oct 13 14:32:16 2024  -
-------------------------------------------------

 Geometry Optimization Parameters
   NAtoms,    NIC,     NZ,  NCons,   NDum,   NFix, NCnnct, MaxDiis
       38     272       0       0       0       0       0       0

 Cartesian Hessian Update
 Hessian updated using BFGS update


** GEOMETRY OPTIMIZATION IN DELOCALIZED INTERNAL COORDINATES **
   Searching for a Minimum

   Optimization Cycle:   9

                    Coordinates (Angstroms)
     ATOM              X               Y               Z
      1  N        1.9578752190    0.1275110436   -1.6989656253
      2  C        2.3957486139   -0.7103794503   -2.6799674334
      3  C        2.5089068870   -0.2037180321   -3.9693774418
      4  C        2.1964280455    1.1142177603   -4.2521326919
      5  C        1.7589339353    1.9468360446   -3.2235504466
      6  C        1.6515809360    1.4235183093   -1.9642211780
      7  C        2.7585098158   -2.1313141543   -2.3757014881
      8  C        1.8479757072   -0.3200427989   -0.2796676560
      9  N       -1.7531363218   -0.8295025923    0.5946181741
     10  C       -2.0635310766    0.2813439591    1.3243798014
     11  N       -1.1435797847    1.1649663845    1.6006135125
     12  C        0.1212680776    0.9927818994    1.1695005527
     13  C        0.4753251334   -0.1041292910    0.3128725118
     14  C       -0.5162801938   -1.0122789142    0.0862047799
     15  C       -3.4586222909    0.4218446719    1.8248461230
     16  C       -3.7613155370   -0.5569750924    2.9838774964
     17  C       -2.8744211980   -0.3403144962    4.2042432075
     18  N        0.9912423465    1.9078673969    1.5856572176
     19  H        2.8558230943   -0.8650097149   -4.7527751877
     20  H        2.2967295912    1.4942098239   -5.2623548916
     21  H        1.5113694538    2.9860040605   -3.3949396930
     22  H        1.3246113135    2.0227233140   -1.1248385625
     23  H        3.0603146075   -2.6312147725   -3.2941605466
     24  H        1.9150415671   -2.6916384239   -1.9633556836
     25  H        3.5967497236   -2.1936422622   -1.6764649919
     26  H        2.6307758155    0.1941440562    0.2800029785
     27  H        2.0968602769   -1.3753422565   -0.2348260226
     28  H       -2.4745389336   -1.5218202772    0.4290113446
     29  H       -0.3677477206   -1.9102536230   -0.5021389166
     30  H       -3.5811563393    1.4514741353    2.1611959348
     31  H       -4.1534812250    0.2478459309    0.9965839969
```

```
    32  H        -3.6855521359   -1.5927374711    2.6327456542
    33  H        -4.8094963480   -0.4070414720    3.2498592972
    34  H        -1.8196386899   -0.5434425401    3.9910107608
    35  H        -3.1755236705   -1.0049890522    5.0148430015
    36  H        -2.9514105572    0.6867493734    4.5693425612
    37  H         1.9837760253    1.8517459779    1.4287197226
    38  H         0.6535858373    2.6300025458    2.2093098278
   Point Group: c1    Number of degrees of freedom:   108


   Energy is   -763.966886878

 Hessian updated using BFGS update
  internal optimization (0)

108 Hessian modes will be used to form the next step
  Hessian Eigenvalues:
     0.001495    0.002189    0.002669    0.004117    0.006054    0.013130
     0.015224    0.016371    0.019035    0.019880    0.020255    0.021831
     0.022698    0.022916    0.023513    0.024515    0.025826    0.026087
     0.027091    0.028197    0.028710    0.030171    0.031556    0.036339
     0.039160    0.041537    0.043215    0.043901    0.044694    0.046231
     0.051170    0.053754    0.055444    0.057570    0.063722    0.079458
     0.085547    0.092093    0.121767    0.122358    0.127123    0.130622
     0.132250    0.132904    0.138956    0.141871    0.142569    0.144085
     0.145018    0.147674    0.148330    0.150022    0.152465    0.152577
     0.158420    0.166272    0.194251    0.206417    0.209131    0.216014
     0.226342    0.228613    0.239337    0.246094    0.248447    0.264372
     0.268772    0.272076    0.286639    0.289678    0.300325    0.300591
     0.300804    0.301459    0.301858    0.303152    0.303977    0.304731
     0.305151    0.305255    0.306117    0.308499    0.312331    0.316449
     0.319583    0.333057    0.333987    0.334866    0.340372    0.343227
     0.350492    0.355509    0.363286    0.372371    0.382267    0.388662
     0.398438    0.401794    0.408450    0.415863    0.419958    0.438286
     0.443662    0.452304    0.472681    0.516987    0.578738    0.765112

 Minimum search - taking simple RFO step
 Searching for Lamda that Minimizes Along All modes
 Value Taken    Lamda =  -0.00022049
 Step Taken.  Stepsize is  0.276428

                             Maximum     Tolerance    Cnvgd?
        Gradient            0.001622      0.000800      NO
        Displacement        0.109166      0.001400      NO
        Energy change      -0.000156      0.000228     YES


 New Cartesian Coordinates Obtained by Inverse Iteration

 Displacement from previous Coordinates is:  1.289315
 ----------------------------------------------------------------
             Standard Nuclear Orientation (Angstroms)
    I     Atom           X                Y                Z
 ----------------------------------------------------------------
    1      N       1.9265279010     0.1031211416    -1.7167817473
    2      C       2.4484064397    -0.7163896785    -2.6725133210
    3      C       2.5687389793    -0.2187939084    -3.9658710215
    4      C       2.1865262691     1.0739272700    -4.2764811083
    5      C       1.6719780399     1.8906880174    -3.2712729333
    6      C       1.5566272144     1.3767649229    -2.0074180523
    7      C       2.8871226915    -2.1088475791    -2.3388360247
    8      C       1.8058602603    -0.3375223279    -0.2958097436
    9      N      -1.8040285861    -0.7531110421     0.5890231673
   10      C      -2.0720119692     0.3439986458     1.3545461749
   11      N      -1.1257695746     1.1956437231     1.6399723103
   12      C       0.1272459892     1.0007805703     1.1826012468
   13      C       0.4414571660    -0.0883003172     0.3021141717
   14      C      -0.5784551414    -0.9608958003     0.0630747779
   15      C      -3.4544109094     0.5077361265     1.8839584515
   16      C      -3.7816636381    -0.5286630569     2.9837115056
```

```
  17      C      -2.8567134772    -0.4387333462     4.1918104398
  18      N       1.0251502260     1.8878184901     1.6002242287
  19      H       2.9785958532    -0.8673253711    -4.7294547882
  20      H       2.2925618931     1.4470648429    -5.2885203611
  21      H       1.3698066419     2.9116599631    -3.4637261562
  22      H       1.1725466310     1.9673468814    -1.1866299809
  23      H       3.2779894559    -2.5880659604    -3.2343561420
  24      H       2.0590437323    -2.7236678180    -1.9754523552
  25      H       3.6836656580    -2.1138836609    -1.5898874081
  26      H       2.6014719776     0.1567823563     0.2639247882
  27      H       2.0265511680    -1.3992212775    -0.2495214437
  28      H      -2.5485926184    -1.4183037470     0.4141486088
  29      H      -0.4638247064    -1.8478697295    -0.5492902586
  30      H      -3.5305804987     1.5182554937     2.2854675686
  31      H      -4.1661388462     0.4148149647     1.0566853299
  32      H      -3.7663198161    -1.5419439597     2.5650547764
  33      H      -4.8147047266    -0.3460704459     3.2858914565
  34      H      -1.8190187474    -0.6800699337     3.9376221357
  35      H      -3.1728897409    -1.1391157801     4.9656866223
  36      H      -2.8713441922     0.5648279644     4.6241508904
  37      H       2.0108308529     1.8297730938     1.4060565650
  38      H       0.7077621488     2.6257902721     2.2160976295
 ----------------------------------------------------------------
 Nuclear Repulsion Energy =        1302.77047741 hartrees
 There are       65 alpha and       65 beta electrons

--------------------------------------------------
-  Entering fldman on Sun Oct 13 14:32:16 2024  -
--------------------------------------------------

 Applying Cartesian multipole field
    Component          Value
    ---------          -----
     (2,0,0)        1.00000E-11
     (0,2,0)        2.00000E-11
     (0,0,2)       -3.00000E-12
 Nucleus-field energy     =     0.0000000283 hartrees

--------------------------------------------------
-  Entering gesman on Sun Oct 13 14:32:16 2024  -
--------------------------------------------------

 Requested basis set is 6-311+G(d,p)
 There are 188 shells and 516 basis functions
 A cutoff of  1.0D-12 yielded  12581 shell pairs
 There are    100497 function pairs (    107133 Cartesian)
 Smallest overlap matrix eigenvalue = 1.89E-06
 Linear dependence detected in AO basis
 Tighter screening thresholds may be required for diffuse basis sets
 Use S2THRESH > 12 and THRESH = 14 in case of SCF convergence issues
 Number of orthogonalized atomic orbitals = 510
 Maximum deviation from orthogonality = 1.174E-11
 Guess MOs from SCF MO coefficient file
 Reading MOs from coefficient file
 Reading MOs from coefficient file

--------------------------------------------------
-  Entering scfman on Sun Oct 13 14:32:16 2024  -
--------------------------------------------------

 Long-range K will be added via erf
 Coulomb attenuation parameter = 0.2 bohr**(-1)
 A restricted hybrid HF-DFT SCF calculation will be
 performed using Pulay DIIS + Geometric Direct Minimization
 Exchange:     0.2220 Hartree-Fock + 1.0000 wB97X-D + LR-HF
 Correlation:  1.0000 wB97X-D
 Using Euler-Maclaurin-Lebedev (75,302) quadrature formula
 Dispersion:   Grimme D
 SCF converges when RMS gradient is below 1.0E-07
 Geometry optimization detected.  Setting ReadMinima to 0
```

```
 Setting SaveMinima to 0
 ---------------------------------------
  Cycle       Energy         DIIS Error
 ---------------------------------------
    1    -763.8990781729      1.05E-03
    2    -763.9654255222      1.48E-04
    3    -763.9666802834      9.96E-05
    4    -763.9669821510      4.03E-05
    5    -763.9670407074      1.56E-05
    6    -763.9670497823      3.66E-06
    7    -763.9670503918      1.26E-06
    8    -763.9670504758      6.02E-07
    9    -763.9670504917      1.82E-07
   10    -763.9670504935      7.19E-08 Convergence criterion met
 ---------------------------------------
 SCF time:  CPU 374.95 s  wall 757.95 s
 SCF   energy in the final basis set = -763.96705049
 Total energy in the final basis set = -763.96705049

--------------------------------------------------
-  Entering anlman on Sun Oct 13 14:44:54 2024  -
--------------------------------------------------



 --------------------------------------------------------------

                   Orbital Energies (a.u.)
 --------------------------------------------------------------

 Alpha MOs
 -- Occupied --
-14.8147 -14.7897 -14.7399 -14.7077 -10.6780 -10.6616 -10.6490 -10.6389
-10.6349 -10.6250 -10.6049 -10.5823 -10.5722 -10.5692 -10.5469 -10.5247
-10.4979 -10.4624  -1.4154  -1.4025  -1.3489  -1.2761  -1.2299  -1.1994
 -1.1862  -1.1418  -1.0995  -1.0818  -1.0570  -1.0528  -1.0288  -1.0045
 -0.9890  -0.9544  -0.9441  -0.9201  -0.9088  -0.8897  -0.8831  -0.8647
 -0.8513  -0.8429  -0.8366  -0.8193  -0.8115  -0.7966  -0.7878  -0.7800
 -0.7725  -0.7584  -0.7493  -0.7472  -0.7439  -0.7271  -0.7219  -0.7148
 -0.6933  -0.6661  -0.6563  -0.6478  -0.6326  -0.6245  -0.6231  -0.6204
 -0.6094
 -- Virtual --
 -0.2703  -0.2592  -0.2446  -0.2248  -0.1745  -0.1643  -0.1485  -0.1432
 -0.1319  -0.1256  -0.1208  -0.1119  -0.1077  -0.1041  -0.0978  -0.0899
 -0.0852  -0.0835  -0.0826  -0.0764  -0.0750  -0.0725  -0.0699  -0.0656
 -0.0632  -0.0579  -0.0551  -0.0511  -0.0494  -0.0434  -0.0394  -0.0365
 -0.0313  -0.0284  -0.0253  -0.0241  -0.0194  -0.0173  -0.0114  -0.0088
 -0.0065  -0.0034   0.0019   0.0041   0.0081   0.0098   0.0109   0.0139
  0.0183   0.0234   0.0263   0.0292   0.0330   0.0349   0.0388   0.0402
  0.0429   0.0449   0.0482   0.0545   0.0565   0.0623   0.0635   0.0660
  0.0722   0.0748   0.0767   0.0769   0.0797   0.0854   0.0860   0.0899
  0.0938   0.0984   0.1031   0.1041   0.1059   0.1070   0.1139   0.1153
  0.1210   0.1232   0.1288   0.1302   0.1311   0.1410   0.1427   0.1461
  0.1489   0.1567   0.1607   0.1647   0.1714   0.1747   0.1760   0.1805
  0.1816   0.1877   0.1895   0.1918   0.1961   0.2054   0.2132   0.2187
  0.2226   0.2383   0.2500   0.2647   0.2769   0.2879   0.3101   0.3153
  0.3248   0.3309   0.3389   0.3418   0.3492   0.3559   0.3605   0.3639
  0.3743   0.3774   0.3852   0.3938   0.3985   0.4043   0.4206   0.4244
  0.4289   0.4345   0.4388   0.4447   0.4511   0.4552   0.4665   0.4706
  0.4833   0.4876   0.4968   0.4993   0.5091   0.5111   0.5181   0.5234
  0.5283   0.5293   0.5314   0.5351   0.5527   0.5542   0.5599   0.5617
  0.5638   0.5684   0.5722   0.5819   0.5863   0.5944   0.6031   0.6084
  0.6151   0.6191   0.6280   0.6311   0.6422   0.6474   0.6532   0.6579
  0.6658   0.6753   0.6785   0.6850   0.6905   0.6975   0.7033   0.7078
  0.7151   0.7199   0.7424   0.7459   0.7519   0.7633   0.7693   0.7806
  0.7923   0.8061   0.8117   0.8187   0.8370   0.8455   0.8487   0.8573
  0.8675   0.8710   0.8840   0.9098   0.9120   0.9223   0.9255   0.9354
  0.9433   0.9635   0.9714   0.9783   0.9820   0.9961   1.0118   1.0400
  1.0509   1.0677   1.0696   1.0898   1.1160   1.1470   1.1769   1.1808
  1.1917   1.2037   1.2154   1.2325   1.2544   1.2822   1.2891   1.2963
  1.3109   1.3187   1.3290   1.3346   1.3480   1.3518   1.3687   1.3758
```

| | | | | | | | |
|---|---|---|---|---|---|---|---|
| 1.3798 | 1.3912 | 1.4012 | 1.4035 | 1.4111 | 1.4205 | 1.4229 | 1.4329 |
| 1.4395 | 1.4470 | 1.4560 | 1.4652 | 1.4723 | 1.4814 | 1.4860 | 1.4904 |
| 1.4977 | 1.5036 | 1.5128 | 1.5258 | 1.5319 | 1.5414 | 1.5466 | 1.5559 |
| 1.5626 | 1.5725 | 1.5752 | 1.5809 | 1.5906 | 1.6029 | 1.6170 | 1.6202 |
| 1.6283 | 1.6355 | 1.6426 | 1.6523 | 1.6595 | 1.6680 | 1.6754 | 1.6826 |
| 1.6979 | 1.7018 | 1.7206 | 1.7312 | 1.7373 | 1.7529 | 1.7621 | 1.7673 |
| 1.7800 | 1.7915 | 1.7971 | 1.8278 | 1.8382 | 1.8400 | 1.8654 | 1.8800 |
| 1.8913 | 1.9022 | 1.9187 | 1.9292 | 1.9557 | 1.9701 | 1.9859 | 1.9883 |
| 1.9976 | 2.0229 | 2.0297 | 2.0340 | 2.0518 | 2.0696 | 2.0819 | 2.0989 |
| 2.1027 | 2.1173 | 2.1318 | 2.1374 | 2.1695 | 2.2004 | 2.2230 | 2.2275 |
| 2.2337 | 2.2423 | 2.2476 | 2.2566 | 2.2693 | 2.2759 | 2.2797 | 2.2922 |
| 2.2972 | 2.3113 | 2.3454 | 2.3565 | 2.3663 | 2.3748 | 2.3884 | 2.3952 |
| 2.4118 | 2.4199 | 2.4300 | 2.4374 | 2.4457 | 2.4556 | 2.4690 | 2.4892 |
| 2.4958 | 2.5186 | 2.5238 | 2.5339 | 2.5443 | 2.5479 | 2.5560 | 2.5599 |
| 2.5665 | 2.5710 | 2.5836 | 2.5852 | 2.5910 | 2.6042 | 2.6146 | 2.6189 |
| 2.6325 | 2.6387 | 2.6571 | 2.6576 | 2.6664 | 2.6842 | 2.6882 | 2.6951 |
| 2.7093 | 2.7338 | 2.7428 | 2.7478 | 2.7540 | 2.7592 | 2.7781 | 2.7854 |
| 2.7912 | 2.7998 | 2.8209 | 2.8292 | 2.8523 | 2.8701 | 2.8808 | 2.8899 |
| 2.8992 | 2.9247 | 2.9618 | 2.9873 | 3.0543 | 3.0697 | 3.1326 | 3.1435 |
| 3.1675 | 3.2463 | 3.2541 | 3.2725 | 3.2977 | 3.3333 | 3.3674 | 3.4147 |
| 3.4186 | 3.4771 | 3.5120 | 3.5579 | 3.5624 | 3.5948 | 3.6240 | 3.6472 |
| 3.6625 | 3.7004 | 3.7228 | 3.7251 | 3.7347 | 3.7403 | 3.7660 | 3.7708 |
| 3.7779 | 3.8433 | 3.8726 | 3.9098 | 3.9356 | 3.9795 | 4.0306 | 4.0832 |
| 4.1112 | 4.2070 | 4.2958 | 4.3065 | 4.5940 | 4.6672 | 4.7063 | 4.7198 |
| 4.9244 | 5.0434 | 5.1993 | 23.5224 | 23.5697 | 23.7191 | 23.7279 | 23.7373 |
| 23.7760 | 23.7957 | 23.8242 | 23.8589 | 23.8847 | 23.9228 | 23.9686 | 23.9784 |
| 24.0370 | 35.4222 | 35.4559 | 35.5211 | 35.6179 | | | |

Ground-State Mulliken Net Atomic Charges

| Atom | Charge (a.u.) |
|---|---|
| 1 N | 0.427275 |
| 2 C | 0.106332 |
| 3 C | -0.000251 |
| 4 C | -0.139482 |
| 5 C | -0.193754 |
| 6 C | -0.178276 |
| 7 C | -0.762027 |
| 8 C | -0.350291 |
| 9 N | -0.095408 |
| 10 C | -0.305205 |
| 11 N | 0.019462 |
| 12 C | 0.284144 |
| 13 C | -0.006003 |
| 14 C | 0.094811 |
| 15 C | -0.414124 |
| 16 C | -0.235361 |
| 17 C | -0.498373 |
| 18 N | -0.479402 |
| 19 H | 0.220113 |
| 20 H | 0.234500 |
| 21 H | 0.227019 |
| 22 H | 0.205977 |
| 23 H | 0.229544 |
| 24 H | 0.203450 |
| 25 H | 0.239371 |
| 26 H | 0.228389 |
| 27 H | 0.245396 |
| 28 H | 0.379890 |
| 29 H | 0.209494 |
| 30 H | 0.243157 |
| 31 H | 0.236416 |
| 32 H | 0.164538 |
| 33 H | 0.226136 |
| 34 H | 0.140221 |
| 35 H | 0.208122 |
| 36 H | 0.191234 |
| 37 H | 0.317147 |

```
    38 H                        0.375816
  --------------------------------------
  Sum of atomic charges =     2.000000

 -----------------------------------------------------------------
                    Cartesian Multipole Moments
 -----------------------------------------------------------------
    Charge (ESU x 10^10)
                 9.6064
    Dipole Moment (Debye)
         X       4.6957      Y       0.2713      Z      -6.0735
       Tot       7.6819
    Quadrupole Moments (Debye-Ang)
        XX     -41.9907     XY       5.6011     YY     -73.3984
        XZ     -42.2776     YZ       1.9382     ZZ     -36.1162
    Traceless Quadrupole Moments (Debye-Ang)
       QXX      25.5334    QYY     -68.6899    QZZ      43.1566
       QXY      16.8032    QXZ    -126.8328    QYZ       5.8146
    Octopole Moments (Debye-Ang^2)
       XXX     -66.2325    XXY     -40.2808    XYY      22.6511
       YYY     -26.1469    XXZ      12.5171    XYZ      18.2579
       YYZ      -9.8770    XZZ      71.1170    YZZ      22.1546
       ZZZ    -108.4427
    Traceless Octopole Moments (Debye-Ang^2)
       XXX   -1241.3074    YYY       6.2539    ZZZ    -674.4169
       XXY    -471.3924    XXZ     505.1637    XYY     257.1593
       XYZ     273.8692    XZZ     984.1480    YYZ     169.2532
       YZZ     465.1385
    Hexadecapole Moments (Debye-Ang^3)
      XXXX   -2568.8374   XXXY     -81.7601   XXYY    -556.5112
      XYYY      -2.2577   YYYY    -751.1975   XXXZ    1015.7256
      XXYZ      68.8876   XYYZ     386.3058   YYYZ      47.5657
      XXZZ    -663.8014   XYZZ      36.1482   YYZZ    -575.2890
      XZZZ     673.8924   YZZZ    -112.8542   ZZZZ   -2594.5843
    Traceless Hexadecapole Moments (Debye-Ang^3)
      XXXX  -14256.8280   XXXY   -6430.6808   XXXZ   13234.6186
      XXYY   -1868.9292   XXYZ    7166.3954   XXZZ   16125.7573
      XYYY    1917.0742   XYYZ    9423.2488   XYZZ    4513.6065
      XZZZ  -22657.8674   YYYY    5041.6576   YYYZ    4794.0016
      YYZZ   -3172.7283   YZZZ  -11960.3970   ZZZZ  -12953.0289
 -----------------------------------------------------------------

--------------------------------------------------
-  Entering drvman on Sun Oct 13 14:44:54 2024  -
--------------------------------------------------

 Calculating analytic gradient of the SCF energy
 Gradient of SCF Energy
           1           2           3           4           5           6
    1  -0.0002048   0.0001836  -0.0000598  -0.0000484   0.0001274  -0.0001101
    2  -0.0003256   0.0001962   0.0000159   0.0001102  -0.0000704  -0.0003401
    3   0.0001434   0.0003286  -0.0002156  -0.0001802  -0.0002746   0.0005350
           7           8           9          10          11          12
    1  -0.0001078  -0.0000421   0.0000086   0.0005096  -0.0000506  -0.0010661
    2  -0.0002559   0.0003803   0.0002914  -0.0008212   0.0005614  -0.0011416
    3  -0.0004941  -0.0001508   0.0003886  -0.0008013   0.0004729  -0.0014044
          13          14          15          16          17          18
    1  -0.0002337   0.0002371   0.0001354  -0.0001841   0.0000239   0.0010698
    2   0.0007397  -0.0000696  -0.0001176   0.0001599   0.0000109  -0.0010382
    3   0.0003809  -0.0001137   0.0001229  -0.0000263  -0.0001176   0.0021392
          19          20          21          22          23          24
    1   0.0000404   0.0000114  -0.0000375   0.0002780   0.0000807  -0.0000959
    2   0.0000770   0.0000532   0.0000624   0.0002300  -0.0000883   0.0000309
    3  -0.0001707  -0.0000210  -0.0001015  -0.0001753   0.0000341  -0.0000058
          25          26          27          28          29          30
    1   0.0001639  -0.0000056  -0.0001487  -0.0001810   0.0000182  -0.0001584
    2  -0.0000309   0.0000601   0.0002483  -0.0000481  -0.0000753  -0.0000319
    3  -0.0000571   0.0000276   0.0006229   0.0000310  -0.0000341   0.0001254
          31          32          33          34          35          36
    1   0.0000394   0.0000814  -0.0000340  -0.0000168  -0.0000053  -0.0000345
```

```
    2   0.0000362  -0.0000224   0.0000423   0.0000484   0.0000196   0.0000803
    3  -0.0000604   0.0000263   0.0000957  -0.0000249   0.0000585  -0.0000329
           37          38
    1   0.0002241  -0.0004074
    2   0.0001273   0.0008951
    3  -0.0005245  -0.0005460
 Max gradient component =       2.139E-03
 RMS gradient           =       4.022E-04
 Gradient time:  CPU 162.81 s  wall 303.71 s

-------------------------------------------------
-  Entering optman on Sun Oct 13 14:49:58 2024  -
-------------------------------------------------

 Geometry Optimization Parameters
   NAtoms,    NIC,     NZ,  NCons,   NDum,   NFix, NCnnct, MaxDiis
       38     272       0       0       0       0       0       0

 Cartesian Hessian Update
 Hessian updated using BFGS update


** GEOMETRY OPTIMIZATION IN DELOCALIZED INTERNAL COORDINATES **
   Searching for a Minimum

   Optimization Cycle:  10

                    Coordinates (Angstroms)
     ATOM                X               Y               Z
      1  N         1.9265279010    0.1031211416   -1.7167817473
      2  C         2.4484064397   -0.7163896785   -2.6725133210
      3  C         2.5687389793   -0.2187939084   -3.9658710215
      4  C         2.1865262691    1.0739272700   -4.2764811083
      5  C         1.6719780399    1.8906880174   -3.2712729333
      6  C         1.5566272144    1.3767649229   -2.0074180523
      7  C         2.8871226915   -2.1088475791   -2.3388360247
      8  C         1.8058602603   -0.3375223279   -0.2958097436
      9  N        -1.8040285861   -0.7531110421    0.5890231673
     10  C        -2.0720119692    0.3439986458    1.3545461749
     11  N        -1.1257695746    1.1956437231    1.6399723103
     12  C         0.1272459892    1.0007805703    1.1826012468
     13  C         0.4414571660   -0.0883003172    0.3021141717
     14  C        -0.5784551414   -0.9608958003    0.0630747779
     15  C        -3.4544109094    0.5077361265    1.8839584515
     16  C        -3.7816636381   -0.5286630569    2.9837115056
     17  C        -2.8567134772   -0.4387333462    4.1918104398
     18  N         1.0251502260    1.8878184901    1.6002242287
     19  H         2.9785958532   -0.8673253711   -4.7294547882
     20  H         2.2925618931    1.4470648429   -5.2885203611
     21  H         1.3698066419    2.9116599631   -3.4637261562
     22  H         1.1725466310    1.9673468814   -1.1866299809
     23  H         3.2779894559   -2.5880659604   -3.2343561420
     24  H         2.0590437323   -2.7236678180   -1.9754523552
     25  H         3.6836656580   -2.1138836609   -1.5898874081
     26  H         2.6014719776    0.1567823563    0.2639247882
     27  H         2.0265511680   -1.3992212775   -0.2495214437
     28  H        -2.5485926184   -1.4183037470    0.4141486088
     29  H        -0.4638247064   -1.8478697295   -0.5492902586
     30  H        -3.5305804987    1.5182554937    2.2854675686
     31  H        -4.1661388462    0.4148149647    1.0566853299
     32  H        -3.7663198161   -1.5419439597    2.5650547764
     33  H        -4.8147047266   -0.3460704459    3.2858914565
     34  H        -1.8190187474   -0.6800699337    3.9376221357
     35  H        -3.1728897409   -1.1391157801    4.9656866223
     36  H        -2.8713441922    0.5648279644    4.6241508904
     37  H         2.0108308529    1.8297730938    1.4060565650
     38  H         0.7077621488    2.6257902721    2.2160976295
   Point Group: c1    Number of degrees of freedom:   108
```

```
  Energy is   -763.967050494

 Hessian updated using BFGS update
  internal optimization (0)

108 Hessian modes will be used to form the next step
  Hessian Eigenvalues:
     0.001041    0.001984    0.002672    0.004123    0.006117    0.012967
     0.014576    0.015521    0.019411    0.019870    0.020164    0.021816
     0.022701    0.022949    0.023451    0.024513    0.025815    0.026124
     0.027054    0.028201    0.029300    0.030294    0.031010    0.036617
     0.039099    0.041522    0.043234    0.043860    0.044613    0.046170
     0.051960    0.053830    0.055511    0.057742    0.063494    0.079458
     0.085584    0.092615    0.121797    0.122366    0.127118    0.129764
     0.132254    0.132910    0.138793    0.141433    0.142945    0.143682
     0.146026    0.147675    0.147934    0.149910    0.152497    0.152614
     0.157701    0.169735    0.194188    0.206499    0.207575    0.216185
     0.225358    0.228582    0.238538    0.245947    0.248357    0.265739
     0.268932    0.272086    0.286038    0.288584    0.300317    0.300558
     0.300792    0.301472    0.301868    0.303174    0.303969    0.304724
     0.305150    0.305283    0.306076    0.308377    0.312365    0.315696
     0.320311    0.333040    0.334048    0.334851    0.340250    0.343283
     0.350417    0.355418    0.364073    0.375643    0.382197    0.390295
     0.398353    0.403344    0.415079    0.415842    0.423944    0.435655
     0.445069    0.455421    0.475567    0.515080    0.578074    0.778592

 Minimum search - taking simple RFO step
 Searching for Lamda that Minimizes Along All modes
 Value Taken    Lamda =  -0.00022880
 Calculated Step too Large.  Step scaled by  0.942453
 Step Taken.  Stepsize is  0.300000

                            Maximum     Tolerance    Cnvgd?
        Gradient           0.001559      0.000800      NO
        Displacement       0.112055      0.001400      NO
        Energy change     -0.000164      0.000228     YES


 New Cartesian Coordinates Obtained by Inverse Iteration

 Displacement from previous Coordinates is:  1.372005
 ----------------------------------------------------------------
            Standard Nuclear Orientation (Angstroms)
    I     Atom           X                 Y                 Z
 ----------------------------------------------------------------
    1      N       1.8918422240     0.0766967313    -1.7381044470
    2      C       2.4988422529    -0.7188021467    -2.6637496433
    3      C       2.6326314642    -0.2307510240    -3.9587701059
    4      C       2.1828124319     1.0322520204    -4.2995861247
    5      C       1.5871411921     1.8285052796    -3.3231874069
    6      C       1.4586882162     1.3229973920    -2.0574478212
    7      C       3.0135292146    -2.0753081099    -2.2937825380
    8      C       1.7591665578    -0.3609462948    -0.3165346583
    9      N      -1.8538712016    -0.6739749872     0.5938007431
   10      C      -2.0754357418     0.4091742442     1.3930793545
   11      N      -1.1011234451     1.2287274684     1.6784774869
   12      C       0.1364989709     1.0125283507     1.1887111989
   13      C       0.4068156770    -0.0736688042     0.2906051387
   14      C      -0.6426179211    -0.9094477497     0.0474563544
   15      C      -3.4419658688     0.5970407778     1.9554851942
   16      C      -3.8011022421    -0.4988754729     2.9841697601
   17      C      -2.8473340399    -0.5478645186     4.1722800367
   18      N       1.0605476802     1.8795035692     1.5909337492
   19      H       3.1076456427    -0.8620785223    -4.6985797138
   20      H       2.2988943321     1.3987000247    -5.3129774287
   21      H       1.2314982748     2.8271130134    -3.5394941003
   22      H       1.0110059186     1.8985427490    -1.2590520008
   23      H       3.4975151494    -2.5243762005    -3.1588723554
   24      H       2.2077815129    -2.7479934356    -1.9858860916
   25      H       3.7548518134    -2.0239320586    -1.4921428899
```

```
   26      H       2.5707529114     0.1060473679     0.2440163828
   27      H       1.9457711998    -1.4299772447    -0.2760596726
   28      H      -2.6217545599    -1.3110159495     0.4147844432
   29      H      -0.5644293936    -1.7863806385    -0.5849900701
   30      H      -3.4661892795     1.5799855389     2.4257171080
   31      H      -4.1680759905     0.5940396691     1.1352090812
   32      H      -3.8491879650    -1.4785424374     2.4937925391
   33      H      -4.8164804812    -0.2870735756     3.3245015760
   34      H      -1.8295435638    -0.8227382000     3.8744385366
   35      H      -3.1837623607    -1.2871733383     4.8999102248
   36      H      -2.7987401933     0.4197902104     4.6777382254
   37      H       2.0387020551     1.8143165627     1.3636633688
   38      H       0.7686795557     2.6249597394     2.2104465659
 ----------------------------------------------------------------
 Nuclear Repulsion Energy =        1301.94175620 hartrees
 There are       65 alpha and       65 beta electrons

--------------------------------------------------
-  Entering fldman on Sun Oct 13 14:49:58 2024  -
--------------------------------------------------

 Applying Cartesian multipole field
    Component          Value
    ---------          -----
     (2,0,0)        1.00000E-11
     (0,2,0)        2.00000E-11
     (0,0,2)       -3.00000E-12
 Nucleus-field energy     =     0.0000000281 hartrees

--------------------------------------------------
-  Entering gesman on Sun Oct 13 14:49:58 2024  -
--------------------------------------------------

 Requested basis set is 6-311+G(d,p)
 There are 188 shells and 516 basis functions
 A cutoff of  1.0D-12 yielded  12579 shell pairs
 There are    100507 function pairs (    107155 Cartesian)
 Smallest overlap matrix eigenvalue = 1.75E-06
 Linear dependence detected in AO basis
 Tighter screening thresholds may be required for diffuse basis sets
 Use S2THRESH > 12 and THRESH = 14 in case of SCF convergence issues
 Number of orthogonalized atomic orbitals = 510
 Maximum deviation from orthogonality = 1.110E-11
 Guess MOs from SCF MO coefficient file
 Reading MOs from coefficient file
 Reading MOs from coefficient file

--------------------------------------------------
-  Entering scfman on Sun Oct 13 14:49:58 2024  -
--------------------------------------------------

 Long-range K will be added via erf
 Coulomb attenuation parameter = 0.2 bohr**(-1)
 A restricted hybrid HF-DFT SCF calculation will be
 performed using Pulay DIIS + Geometric Direct Minimization
 Exchange:     0.2220 Hartree-Fock + 1.0000 wB97X-D + LR-HF
 Correlation:  1.0000 wB97X-D
 Using Euler-Maclaurin-Lebedev (75,302) quadrature formula
 Dispersion:   Grimme D
 SCF converges when RMS gradient is below 1.0E-07
 Geometry optimization detected.  Setting ReadMinima to 0
 Setting SaveMinima to 0
 ---------------------------------------
  Cycle       Energy         DIIS Error
 ---------------------------------------
    1    -763.8912138606      1.11E-03
    2    -763.9653502402      1.61E-04
    3    -763.9667226315      1.15E-04
    4    -763.9671260247      4.20E-05
    5    -763.9671896629      1.68E-05
```

```
    6    -763.9672002304      4.02E-06
    7    -763.9672009498      1.41E-06
    8    -763.9672010521      6.52E-07
    9    -763.9672010715      1.99E-07
   10    -763.9672010738      7.95E-08 Convergence criterion met
 ---------------------------------------
 SCF time:  CPU 337.91 s  wall 615.92 s
 SCF   energy in the final basis set = -763.96720107
 Total energy in the final basis set = -763.96720107

-------------------------------------------------
-  Entering anlman on Sun Oct 13 15:00:14 2024  -
-------------------------------------------------


 --------------------------------------------------------------

                    Orbital Energies (a.u.)
 --------------------------------------------------------------

 Alpha MOs
 -- Occupied --
-14.8147 -14.7896 -14.7397 -14.7076 -10.6779 -10.6616 -10.6491 -10.6383
-10.6349 -10.6248 -10.6049 -10.5821 -10.5720 -10.5688 -10.5459 -10.5247
-10.4982 -10.4631  -1.4154  -1.4023  -1.3488  -1.2759  -1.2298  -1.1994
 -1.1858  -1.1419  -1.0995  -1.0813  -1.0570  -1.0532  -1.0281  -1.0047
 -0.9887  -0.9546  -0.9439  -0.9203  -0.9088  -0.8896  -0.8827  -0.8640
 -0.8512  -0.8424  -0.8377  -0.8191  -0.8108  -0.7974  -0.7874  -0.7794
 -0.7715  -0.7590  -0.7495  -0.7468  -0.7435  -0.7269  -0.7219  -0.7144
 -0.6934  -0.6666  -0.6558  -0.6479  -0.6325  -0.6252  -0.6235  -0.6205
 -0.6093
 -- Virtual --
 -0.2697  -0.2593  -0.2447  -0.2248  -0.1747  -0.1640  -0.1495  -0.1434
 -0.1316  -0.1245  -0.1205  -0.1114  -0.1075  -0.1038  -0.0970  -0.0903
 -0.0852  -0.0844  -0.0829  -0.0757  -0.0743  -0.0727  -0.0690  -0.0661
 -0.0636  -0.0562  -0.0553  -0.0516  -0.0479  -0.0426  -0.0406  -0.0372
 -0.0305  -0.0286  -0.0250  -0.0242  -0.0188  -0.0167  -0.0106  -0.0096
 -0.0067  -0.0027   0.0016   0.0041   0.0066   0.0096   0.0101   0.0125
  0.0196   0.0230   0.0267   0.0292   0.0326   0.0354   0.0381   0.0397
  0.0431   0.0460   0.0493   0.0540   0.0585   0.0619   0.0644   0.0659
  0.0732   0.0740   0.0757   0.0771   0.0793   0.0863   0.0871   0.0891
  0.0938   0.0974   0.1025   0.1049   0.1063   0.1083   0.1131   0.1158
  0.1204   0.1258   0.1280   0.1293   0.1324   0.1401   0.1421   0.1483
  0.1491   0.1571   0.1622   0.1636   0.1689   0.1739   0.1771   0.1797
  0.1824   0.1867   0.1893   0.1952   0.1964   0.2061   0.2143   0.2202
  0.2240   0.2385   0.2506   0.2666   0.2778   0.2901   0.3098   0.3136
  0.3225   0.3322   0.3382   0.3427   0.3501   0.3562   0.3605   0.3646
  0.3730   0.3755   0.3863   0.3941   0.3990   0.4044   0.4203   0.4255
  0.4264   0.4333   0.4416   0.4484   0.4513   0.4562   0.4688   0.4723
  0.4831   0.4855   0.4973   0.4985   0.5065   0.5113   0.5168   0.5216
  0.5265   0.5272   0.5321   0.5356   0.5541   0.5559   0.5586   0.5626
  0.5645   0.5691   0.5733   0.5808   0.5850   0.5963   0.6048   0.6089
  0.6156   0.6198   0.6273   0.6320   0.6429   0.6478   0.6540   0.6592
  0.6712   0.6719   0.6786   0.6838   0.6912   0.6965   0.7035   0.7128
  0.7145   0.7181   0.7422   0.7450   0.7489   0.7660   0.7689   0.7775
  0.7910   0.8066   0.8136   0.8183   0.8338   0.8445   0.8494   0.8611
  0.8693   0.8710   0.8858   0.9037   0.9109   0.9222   0.9281   0.9361
  0.9462   0.9628   0.9700   0.9789   0.9899   0.9988   1.0149   1.0388
  1.0467   1.0648   1.0705   1.0974   1.1194   1.1400   1.1740   1.1824
  1.1887   1.2042   1.2177   1.2359   1.2569   1.2796   1.2867   1.2974
  1.3115   1.3196   1.3292   1.3313   1.3467   1.3538   1.3692   1.3730
  1.3808   1.3921   1.3979   1.4036   1.4098   1.4210   1.4255   1.4298
  1.4389   1.4474   1.4565   1.4672   1.4715   1.4806   1.4866   1.4934
  1.4990   1.5050   1.5099   1.5247   1.5297   1.5385   1.5471   1.5565
  1.5639   1.5717   1.5746   1.5827   1.5920   1.6025   1.6173   1.6199
  1.6282   1.6379   1.6425   1.6525   1.6619   1.6664   1.6773   1.6834
  1.6994   1.7035   1.7245   1.7313   1.7369   1.7512   1.7665   1.7691
  1.7788   1.7887   1.7972   1.8292   1.8376   1.8435   1.8642   1.8789
  1.8924   1.9002   1.9180   1.9298   1.9548   1.9744   1.9851   1.9933
  2.0004   2.0230   2.0312   2.0363   2.0536   2.0730   2.0824   2.0974
```

```
 2.1014   2.1172   2.1299   2.1356   2.1703   2.2021   2.2253   2.2275
 2.2343   2.2447   2.2480   2.2583   2.2674   2.2768   2.2792   2.2928
 2.2943   2.3139   2.3484   2.3567   2.3645   2.3740   2.3892   2.3937
 2.4128   2.4198   2.4286   2.4415   2.4458   2.4571   2.4695   2.4922
 2.4982   2.5187   2.5244   2.5319   2.5424   2.5493   2.5547   2.5615
 2.5669   2.5732   2.5845   2.5894   2.5910   2.6040   2.6134   2.6166
 2.6356   2.6408   2.6494   2.6574   2.6657   2.6829   2.6856   2.6950
 2.7078   2.7353   2.7393   2.7462   2.7526   2.7604   2.7766   2.7877
 2.7907   2.8012   2.8219   2.8278   2.8544   2.8726   2.8803   2.8905
 2.8994   2.9218   2.9587   2.9901   3.0565   3.0706   3.1400   3.1459
 3.1677   3.2459   3.2560   3.2722   3.2991   3.3321   3.3670   3.4152
 3.4195   3.4791   3.5131   3.5569   3.5624   3.5953   3.6185   3.6489
 3.6635   3.7001   3.7227   3.7251   3.7337   3.7401   3.7650   3.7708
 3.7794   3.8444   3.8729   3.9101   3.9355   3.9781   4.0305   4.0826
 4.1117   4.2076   4.2964   4.3076   4.5937   4.6673   4.7053   4.7198
 4.9251   5.0437   5.2000  23.5220  23.5696  23.7178  23.7277  23.7393
23.7765  23.7940  23.8252  23.8585  23.8853  23.9221  23.9701  23.9780
24.0377  35.4218  35.4553  35.5215  35.6177
--------------------------------------------------------------

         Ground-State Mulliken Net Atomic Charges

    Atom                 Charge (a.u.)
 ----------------------------------------
     1 N                  0.425115
     2 C                  0.102469
     3 C                  0.000641
     4 C                 -0.136383
     5 C                 -0.195933
     6 C                 -0.171178
     7 C                 -0.748357
     8 C                 -0.374006
     9 N                 -0.086646
    10 C                 -0.329475
    11 N                  0.030404
    12 C                  0.292680
    13 C                 -0.017389
    14 C                  0.107336
    15 C                 -0.425238
    16 C                 -0.243218
    17 C                 -0.490913
    18 N                 -0.478144
    19 H                  0.220281
    20 H                  0.234330
    21 H                  0.226837
    22 H                  0.210194
    23 H                  0.229484
    24 H                  0.212376
    25 H                  0.234276
    26 H                  0.229447
    27 H                  0.246494
    28 H                  0.377625
    29 H                  0.211142
    30 H                  0.243362
    31 H                  0.240091
    32 H                  0.162228
    33 H                  0.226619
    34 H                  0.140536
    35 H                  0.208194
    36 H                  0.191932
    37 H                  0.316786
    38 H                  0.375999
 ----------------------------------------
 Sum of atomic charges =     2.000000

-----------------------------------------------------------------
                    Cartesian Multipole Moments
-----------------------------------------------------------------
   Charge (ESU x 10^10)
                 9.6064
```

```
    Dipole Moment (Debye)
         X       4.5568      Y       0.3648      Z      -6.1402
       Tot       7.6551
    Quadrupole Moments (Debye-Ang)
        XX     -40.5005     XY       4.2982     YY     -74.6524
```
[truncated: 635,509 more chars]
